# Characterisation of the *Carpinus betulus* L. Phyllomicrobiome in Urban and Forest Areas

**DOI:** 10.3389/fmicb.2019.01110

**Published:** 2019-05-29

**Authors:** Valeria Imperato, Lukasz Kowalkowski, Miguel Portillo-Estrada, Stanislaw W. Gawronski, Jaco Vangronsveld, Sofie Thijs

**Affiliations:** ^1^Department of Biology, Centre for Environmental Sciences, Hasselt University, Diepenbeek, Belgium; ^2^Faculty of Horticulture, Biotechnology and Landscape Architecture, Warsaw University of Life Sciences, Warsaw, Poland; ^3^Department of Biology, Centre of Excellence PLECO, University of Antwerp, Wilrijk, Belgium; ^4^Department of Plant Physiology, Faculty of Biology and Biotechnology, Maria Skłodowska-Curie University, Lublin, Poland

**Keywords:** *Carpinus betulus*, phyllosphere microbiome, city, air pollution, volatile organic compounds, shotgun metagenome, particulate matter, forest

## Abstract

Urban green areas are highly valued by citizens for their contribution to the quality of life in cities. Plants play an important role in mitigating airborne pollutants and are assisted in this role by the metabolic capacities of the millions of microbial cells that colonize leaf surfaces (phyllosphere). Many factors influence phyllosphere microbial community composition and function, but to what extent does airborne pollution in cities impact the composition of microbial communities and their functional degradation genes? Here we describe the characterization of the phyllospheric bacterial communities of *Carpinus betulus* L. trees (hornbeam) across three locations: the city center of Warsaw (Poland), a forest in a UNESCO World Heritage Site (Białowieża), and a forest in one of the world’s oldest operational oil fields (Bóbrka). *C. betulus* contained higher particulate matter (PM) concentrations, with higher concentrations of palladium and radon in the PM, on leaves in Warsaw than in the forests. Volatile organic compound (VOC) analyses of sampled air revealed higher concentrations of butanone methyl propanal, butylbenzene, and cyclohexane in Bóbrka than Warsaw and Białowieża, while in Warsaw, xylene and toluene were higher. Shotgun microbiome sequencing uncovered a dominance of Gammaproteobacteria (71%), mainly *Pseudomonas* spp., Actinobacteria, Alpha- and Betaproteobacteria, and Firmicutes. Community composition and function differed significantly between the forests and Warsaw city center. Statistically more hydrocarbon degradation genes were found in Białowieża compared to Warsaw and Bóbrka, and *in vitro* tests of diesel degradation and plant growth promotion traits of culturable representatives revealed that Białowieża held the highest number of bacteria with plant beneficial properties and degradation genes. This study provides the first detailed insights into the microbiome of *C. betulus* and sets the stage for developing to a more integrated understanding of phyllosphere microbiota in cities, and their relationships with human health.

## Introduction

Trees play an important role in scavenging and degrading airborne pollutants, and the combined action of plants and phyllospheric microorganisms for plant bioremediation is known as phylloremediation (Weyens et al., 2015; Wei et al., 2017). In contrast to soilborne pollutant biodegraders, less is known about how microbiota on tree leaves aid in dissipating toxic urban-related volatile organic compounds (VOCs) and particulate matter (PM). There is evidence of higher uptake rates of airborne phenol by leaves inoculated with hydrocarbonoclastic bacteria compared to surface-sterilized leaves (Sandhu et al., 2007), setting the stage for strategic utilizing of phyllosphere microbiota to combat air pollution. To accomplish this, a good understanding of phyllosphere microbiota, their distribution, and their contribution to phylloremediation is needed (Vorholt, 2012).

The phyllosphere supports diverse phyllospheric communities influenced by both plant factors and environmental conditions (Wagner et al., 2016). It is estimated that aboveground microbial density reaches 10^6^–10^7^ cells/cm^2^ of photosynthetically active leaves, remarkable given the hostile phyllosphere environment (Vorholt, 2012) where microorganisms are exposed to large daily and seasonal fluctuations of temperature and humidity, are exposed to air pollutants, have limited access to nutrients, and are exposed to harmful ultraviolet radiation. In addition, far from being static guests, these microorganisms constantly interact with their host plant in symbiotic relationships.

Culture-independent studies have indicated the importance of host plant characteristics to phyllospheric microbial community structure. For example, Redford et al. (2010) characterized distinct phyllospheric communities across 56 tree species. The geographic location (Finkel et al., 2011; Rastogi et al., 2013) of the host plant influences phyllospheric communities within plant species, and air pollution from vehicle traffic has been shown to influence phyllospheric bacterial cultures (Brighigna et al., 2000) and the composition of bacterial phyllospheres of ivy plants (Smets et al., 2016). These studies did not endeavor to understand how the functional diversity of phyllosphere microbiota responds to differences in air pollution.

Among plant species that are common in urban areas, *Carpinus betulus* trees (hornbeam) are widespread in most cities of Western, Central, Eastern, and Southern Europe, including Southern England. To the best of our knowledge, bacterial communities associated with hornbeam leaves have not been investigated before. The aims of this work were to perform, for the first time: (i) a phylogenetic characterization of bacterial communities hosted by *C. betulus* leaves; (ii) an assessment of spatial variability of bacterial phyllosphere communities associated with *C. betulus* trees located in areas of Poland with different types and levels of pollution (Warsaw city center, Bóbrka oil field, and the Białowieża primeval forest); (iii) an evaluation of air pollution impact on the abundance of linear and aromatic hydrocarbon degradation genes; (iv) a quantitative assessment of the potential of phyllosphere isolates to tolerate and metabolize diesel range organics; and (v) an assessment of plant growth promotion traits of isolated phyllospheric bacteria.

## Materials and Methods

### Sampling of Hornbeam Leaves

*Carpinus betulus* L. leaves were collected in May and June 2016 from three locations in Poland with different anthropogenic pressures: Warsaw city center, a deciduous forest in the Bóbrka oil field, and the Białowieża natural forest. Established in 1854, Bóbrka has the world’s oldest oil wells still in production, each producing one barrel per day. Because of the oil drilling activities, there is a strong smell of oil in the air. Białowieża forest is one of the largest (105 km^2^) and last remaining primeval forests in Europe. The forest is a UNESCO World Heritage Site and an EU Nature 2000 Special Area of Conservation. Warsaw city center is characterized by high year-round levels of air pollution including vehicle exhaust–related organic volatiles (benzene, benzaldehyde) and PM.

The sampling dates for Białowieża and Warsaw were May 30, 2016, and June 1, 2016, for Bóbrka. At each location, three subsites were selected, and at each subsite, three trees were sampled (*n* = 12 per location; Table 1). Branches with two leaves were collected at a height of 1.5 to 2 m. The leaves were collected with metal scissors and tweezers rinsed with ethanol to prevent DNA contamination from external sources, and the leaves were immediately stored in sterile 50-ml tubes filled with phosphate buffer (NaH_2_PO_4_.2H_2_O 11.95 g L^-1^, Na_2_HPO_4_.7H_2_O 16.5 g L^-1^, and 100 μl of Tween 80). The tubes were transported to the lab on ice for processing.

**Table 1:** Location, coordinates, sampling site characteristics, and average concentration of particulate matter captured on the hornbeam leaves.

Location	ID	GPS coordinates (latitude longitude)	Hornbeam sampling area characteristics	Average PM_10_ (μg/cm^2^) ± SE	Average PM_2.5_ (μg/cm^2^) ± SE
Warsaw	Wa11;14	52.236880 21.001079	Park near Grzybowska str.	7.54 ± 0.25*	3.70 ± 0.42*
	Wa22;24	52.236997 21.003501	Slow-traffic intersection	9.33 ± 0.63*	4.71 ± 0.77*
	Wa33;34	52.253019 21.012416	Multimedialny park	6.03 ± 1.40*	3.59 ± 0.22*
Bóbrka	Bo13;14	49.616790 21.708230	Oil well near entrance	2.38 ± 0.27	2.84 ± 0.19
	Bo23;24	49.617820 21.705440	Oil well in deep forest	3.52 ± 0.68	3.08 ± 0.46
	Bo32;33	49.618590 21.703850	Oil pipes in deep forest	3.27 ± 0.16	3.43 ± 0.21
Białowieża	Bi31;32	52.814444 23.935028	North entrance	3.32 ± 0.59	2.52 ± 0.29
	Bi42;43	52.813127 23.932727	Deeper in forest	3.17 ± 0.17	2.30 ± 0.22
	Bi51;53	52.814010 23.928421	Walking path in forest	3.12 ± 0.83	2.51 ± 0.23

^∗^*Significant differences (*p* ≤ 0.05, *n* = 3 replicates of 250-cm^*2*^ leaf surface area) between sampling areas (Kruskal–Wallis H test)*.

For determination of PM on leaves, approximately 250 cm^2^ of leaf tissue was collected per sample. For metal concentration analyses, 2 g of leaves were collected, put directly into paper bags (to prevent loss by electrostatic repulsion that occurs in plastic bags), and transported to the lab.

### Analysis of Particulate Matter on Hornbeam Leaves

A beaker was filled with 250 ml of distilled water to which *C. betulus* leaves (250 cm^2^) were added. The leaves were stirred vigorously for 1 min manually and subsequently with a magnetic stir bar for 30 min as described previously (Dzierzanowski et al., 2011). Particles larger than 100 μm were filtered with a metal sieve (Haver and Boecker, Germany). Subsequently, PM fractions were separated using Type 91 Whatman ashless filters with 10-μm retention and Type 42 with 2.5-μm retention. Prior to use, the filters were dried overnight in an oven at 60°C, and their weight was determined to correct for air humidity. For filtration, a 47-mm glass filter funnel with stopper support was assembled on a glass bottle connected to a vacuum pump with a rubber tube (chemical-resistant Laboport vacuum systems SR 840, KNF Verder N.V., Aartselaar, Belgium) (Supplementary Figure S1). Filters were dried and post-weighed using a Mettler Toledo AG64 precision balance (Mettler-Toledo S.A., Zaventem, Belgium) to calculate the mass of PM in each fraction of every sample. Each location was analyzed in triplicates.

The total area of leaf samples was measured using Image Analysis System (Skye Instruments Ltd., United Kingdom) and Skye Leaf software, which allowed the amount of PM to be expressed as μg cm^-2^ leaf area. Although PM was washed off from both the adaxial and abaxial surfaces of the leaves, the amount of PM was expressed per one surface of leaf area (as measured by image analysis).

### Analysis of Air VOC Concentrations Using PTR-TOF-MS

Air was sampled three times at each location. New 0.6-L Tedlar sampling bags equipped with a Thermogreen LB-2 septum (model 30284-U, Supelco, Sigma-Aldrich, Belgium) were filled using a clean 100-ml glass syringe. The nine sampling bags (three locations × three replicates) were measured within 3 days with a PTR-TOF-MS (proton-transfer-reaction time-of-flight mass spectrometer). The drift tube of the instrument was operated at 600 V of electric potential and 2.3 mbar of pressure at 60°C, resulting in a field density ratio (E/N) of ≈130 Td. More information can be found in Portillo-Estrada et al. (2018). The transmission values of the PTR-TOF-MS were calibrated with a mixture of 11 pure compounds ranging from m^+^/z 33 to 137. The coefficients of reaction between each VOC and H_3_O^+^ were calculated via the gas calibration mixture for benzene, toluene, and xylene. For the other VOCs, approximations were made using values described previously (Cappellin et al., 2012). While Tedlar is an inert material that is suitable for preserving VOCs in air, the effect of bag storage on air samples was assessed by measuring Antwerp air once per day over 6 days. A conversion factor (usually <20% change) corresponding to 3 days of transport was applied to the concentrations measured.

### Isolation of Culturable Epiphytic Bacteria

Epiphytic bacteria were released from leaf surfaces by vigorously vortexing Falcon tubes containing two leaves in 30 ml of phosphate buffer (NaH_2_PO_4_.2H_2_O 11.95 g L^-1^, Na_2_HPO_4_.7H_2_O 16.5 g L^-1^, and 100 μl of Tween 80) for 5 min followed by shaking for 15 min at 240 rpm on an orbital shaker. Consequently, tubes were centrifuged (4,000 RCF × 15 min), and the cell pellets were resuspended in 3 ml of phosphate buffer. Bacterial suspensions were diluted to 10^-6^ in tubes filled with 10 mM MgSO_4_. Aliquots of each dilution were plated on three different solid media for bacterial growth: 869 rich medium (Mergeay et al., 1985), 1/10 869 medium, and 284 minimal medium (Schlegel et al., 1991). Plates were stored at 28°C in a microbiological incubator for a week.

### Genotypic Characterization of the Isolates

From each isolated strain, the total genomic DNA was extracted using a DNeasy Blood and Tissue Kit (Qiagen, Venlo, Netherlands). The 27F (5′ AGAGTTTGATCMTGGCTCAG 3′) and 1492R (5′ TACGGYTACCTTGTTACGACTT 3′) primers were used for amplification of the 16S rRNA gene. The PCR master mix consisted of: DNA template (±1–10 ng/μl), 1X High Fidelity PCR buffer (Invitrogen, Carlsbad, CA, United States), 0.2 mM dNTPs, 2 mM MgCl_2_, 0.2 μM each of the forward and reverse primers, and 1 U High Fidelity Platinum Taq DNA polymerase (Invitrogen, Carlsbad, CA, United States) per 50 μl. PCR conditions were denaturation at 94°C for 5 min, 30 cycles of 94°C for 1 min, 54°C for 45 s, and 72°C for 1.5 min, followed by a final extension of 10 min at 72°C. The PCR products for each strain were sent to Macrogen (Amsterdam, Netherlands) for Sanger sequencing. Sequence chromatograms were quality-checked using Geneious v4.8.5 (Biomatters ApS, Denmark), and sequences were compared with nucleotide sequences present in the Ribosomal Database (RDP Release 11).

### *In vitro* Plant Growth Promoting Activity

The bacterial collection isolated here from hornbeam leaves was screened *in vitro* for plant growth promoting (PGP) traits including 1-aminocyclopropane-1-carboxylate (ACC)-deaminase, siderophore production, acetoin, and indole-3-acetic acid (IAA) production. The bacterial production of ACC-deaminase was estimated by monitoring the amount of α-ketobutyrate generated by the enzymatic hydrolysis of ACC (Belimov et al., 2005). Siderophore release was evaluated via the Chrome Azurol S (CAS) assay (Schwyn and Neilands, 1987). The bacterial production of the phytohormone IAA was estimated by a colorimetric assay using Salkowski’s reagent (Patten and Glick, 2002). The production of the volatile PGP compound acetoin was assessed using the Voges–Proskauer assay (Romick and Fleming, 1998). The evaluation of results was based on observations of colorimetric reactions after 5 days of incubation at 30°C.

### *In vitro* Estimation of Hydrocarbon Degradation Capabilities

Isolated bacterial strains were tested in triplicate for their capability to use diesel as the sole carbon source using the 2,6-dichlorophenol indophenol (DCPIP) assay (Buèková et al., 2013). In brief, the isolated strains were pre-cultured in 5 ml of 869 rich medium at 30°C and 160 rpm on an orbital shaker until an O.D._660_ nm of 1.0. Cells were pelleted by centrifugation (4,000 RCF for 20 min), washed three times with 10 mM MgSO_4_, and incubated overnight at 30°C to allow the strains to use all remaining carbon source. Subsequently, to a 2-ml sterile microcentrifuge tube was added 750 μl of Bushnell and Haas medium (Bushnell and Haas, 1941) supplemented with 50-μl DCPIP stock solution at 100 μg ml^-l^. Subsequently, 80 μl of cell suspension and 5-μl filter sterilized diesel were added. Cells were cultivated in the dark to avoid photodegradation of the redox dye (30°C, 120 rpm) for 1 week. Assays were performed in triplicate, and the color of the reaction medium was compared with three controls. Two negative controls, one with no diesel and one with no bacteria, and a positive control with *Pseudomonas aeruginosa* WatG (Wongsa et al., 2004) were included. Isolates were scored as positive for microbial hydrocarbon degradation capability if the solution looked clear and negative if it remained blue.

### Leaf Epiphytic DNA Isolation

Epiphytic bacterial cells were released from leaf surfaces in a manner similar to that described for the isolation of culturable epiphytic bacteria by vortexing Falcon tubes with two leaves in 30 ml of phosphate buffer for 5 min, followed by a shaking for 15 min at 240 rpm on an orbital shaker. Tubes were centrifuged (4,000 RCF for 15 min), and cell pellets were used for total DNA extraction using a DNeasy Blood and Tissue Kit (Qiagen, Venlo, Netherlands) according to the manufacturer’s instructions. The concentration and quality of DNA were verified using a NanoDrop ND-1000 (Thermo Fisher Scientific, United States) and by gel electrophoresis. All DNA samples were stored at -80°C.

### Quantitative Real-Time PCR

Quantitative PCR was used to evaluate levels of: the alkane monooxygenase B gene (*alkB*) (Chen et al., 2003; Schulz et al., 2012), a gene that codes for a protein involved in the catabolism of medium-chain-length (C_5_–C_16_) alkanes; naphthalene 1,2-dioxygenase reductase (coded by *nahA*), a protein that catalyzes the *cis*-dihydroxylation of naphthalene and other aromatic hydrocarbons; and the gene coding for phenol hydroxylase alpha subunit (Phe) (Baldwin et al., 2003), an enzyme that catalyzes the monooxygenation of phenol and other aromatic hydrocarbons (Baldwin et al., 2003). Primers Eub338F and Eub518R (Supplementary Table S1) were used for quantifying the total number of heterotrophic bacteria (Fierer et al., 2005). Group-specific primers (Supplementary Table S1) (Pfeiffer et al., 2014) were used for quantifying the following bacterial groups: Actinobacteria, Alphaproteobacteria, Bacteroidetes, Betaproteobacteria, Firmicutes, and *Pseudomonas* spp. (Bergmark et al., 2012). Plasmid standards were prepared from pure genome-sequenced bacterial cultures, and optimal PCR annealing temperatures were experimentally determined. Triplicate 10-μl reactions containing 5-μl 2X QuantiNova SYBR Green PCR Master Mix (QIAGEN, Venlo, Netherlands), an exact concentration of each primer (see Supplementary Table S1), and 2 μl of template DNA (0.1–3 ng μl^-1^) were executed. For all reactions, 96-well BIORAD polypropylene PCR plates were used (MLP-9601, BIO-RAD, Venendaal, Netherlands). Samples were run with the following cycling conditions on a 7500 Real-Time PCR System (Thermo Fisher Scientific, Geel, Belgium): an initial denaturation at 95°C for 15 min, followed by 40 cycles of denaturation at 94°C for 15 s, annealing at T_annealing_ (Supplementary Table S1) for 30 s and, extension at 72°C for 30 s. After every cycle, a dissociation curve was generated according to the following conditions: 95°C for 15 s, 60°C for 60 s, 95°C for 15 s, and 60°C for 15 s.

The total bacterial abundance was expressed as log copies of the 16S rRNA gene per gram of hornbeam leaf, and the results were compared to a standard curve prepared from plasmid DNA. Amplifications for all were linear (*R*^2^ > 0.99) over six orders of dynamic range from 10^3^ to 10^8^ copies μl^-o^, and efficiencies reached between 89.2 and 102%. Melting curves were evaluated to confirm that the detected fluorescence originated from specific products and not from primer dimers or other amplification artifacts.

### Shotgun Metagenomic Sequencing

Bacterial gDNA samples were quantified using a Qubit dsDNA assay kit (Thermo Fisher Scientific, Merelbeke, Belgium) according to the manufacturer protocols. DNA samples (2–10 ng μl^-1^) were sent to Macrogen (Seoul, South Korea) for library preparation using the Illumina Nextera DNA XT kit, followed by sequencing on the HiSeq4000 platform (560 M reads, 556 Gb, 100 bp PE).

### Bioinformatic Data Analyses and Statistics

The raw shotgun Illumina reads were quality-filtered and analyzed using the assembly, annotation, and genome binning pipeline in Atlas v1.0.34 (White et al., 2017) using the master branch Supplementary Data S6^1^. The assembly was performed using MEGAHIT (Li et al., 2015) according to the default configurations in Atlas (config. file in Supplementary Data S4). The assembled reads were annotated using the DIAMOND (Buchfink et al., 2014) RefSeq protein database (downloaded 17/04/2018). The comparative gene catalog file generated from Atlas was used as input for the KEGG Automatic Annotation Server (KAAS), and the GHOSTZ program was used to find homologs for the protein sequences (Suzuki et al., 2015). In addition, MetAnnotate was used for functional annotation of a selection of aromatic ring–hydroxylating genes and dioxygenases with HMMs retrieved from the FunGene repository^2^. To predict taxonomy, CLARK was used (Ounit et al., 2015) in addition to Kaiju (Menzel et al., 2016) against the NCBI NR+euk database (208-02-23), and MetAnnotate using a Hidden Markov Model (HMMs) for the *rpo*B gene (Petrenko et al., 2015). Lastly, genomic binning was performed using the binner branch of Atlas with the algorithms MetaBAT (Kang et al., 2015), CONCOCT (Alneberg et al., 2014), and MaxBin (Wu et al., 2014). DASTool (Sieber et al., 2018) was used for bin refining, concluding with a round of dRep (Olm et al., 2017) to keep only the unique bins.

In addition to Atlas, the Functional Mapping and Analysis Pipeline (FMAP) Supplementary Data S5 was used to statistically interpret the metagenome data and show differentially abundant pathways and genes in KEGG maps and bar charts (Kim et al., 2016). The scripts used were FMAP mapping, quantification, table, and comparison using *p* < 0.05 and log fold change >1.5. The KEGG orthology file generated by FMAP was translated into higher-hierarchy pathways and modules using custom Python scripts, and these were fed into STAMP for further comparative statistical analyses (Parks et al., 2014).

### NCBI Accession Numbers

Raw shotgun sequencing data were submitted to the Short Read Archive of NCBI with project identifier PRJNA506726 and individual FASTQ sample IDs SRX5062447–SRX5062463. The partial 16S rRNA gene Sanger sequences of the culturable isolates were submitted to GenBank through NCBI with the accession numbers MK216816–MK216879.

## Results

### Sampling-Day Characteristics and Airborne Pollutant Concentrations

**Figure 1:**
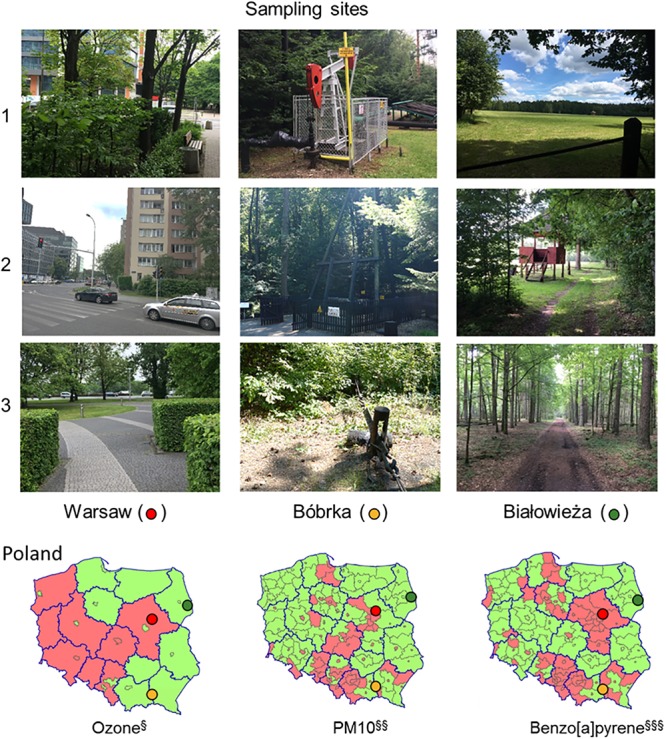
Photos of the sampling locations in Poland and annual ambient air quality assessment maps for 2008. The photos show the sampling locations of the hornbeam trees. The maps show the average ozone, PM_10_, and benzo[*a*]pyrene concentrations in two classes, A (green) and C (red), in accordance with the Regulation of the Ministry of the Environment of March 6, 2008, where for class A, concentrations do not exceed the limit value, and for class C, the concentrations exceed the limit value. Ambient air pollution maps are adapted from http://www.gios.gov.pl.

At each site, Warsaw, Bóbrka, and Białowieża, samples were collected at three locations, of which pictures are shown in Figure 1. Locations 1 and 3 in Warsaw were sampled in a park, and location 2 near a busy intersection. Location 1 in Bóbrka was close to the entrance of the forest while locations 2 and 3 were deeper in the forest, all close to active oil wells. Białowieża National Park was entered from the north side (location 1), and locations 2 and 3 were from deeper in the forest. Each time, healthy-looking hornbeam leaves were collected. On the day of sampling in Warsaw and Białowieża (May 30, 2016), the average temperature was 22°C, with a relative humidity of 41% and average wind speed of 11 km h^-1^. For the sampling day in Bóbrka (June 1, 2016), the average temperature was 23°C, with an average humidity of 56% and 12 km h^-1^ wind speed.

In addition to average year-round PM concentrations gleaned from ambient air quality maps, we also measured PM on hornbeam leaves at each location in order to reflect the local accumulated PM. Leaves from the Białowieża forest and Bóbrka harbored statistically lower (*p* < 0.05) concentrations of PM_10_ and PM_2.5_ compared to Warsaw city center (Table 1). The highest PM_2.5_ concentrations were measured at the slow-traffic intersection in Warsaw, on average 265% more than both forests. The busy intersection with often slow traffic (Figure 1) can explain part of these data.

**Figure 2:**
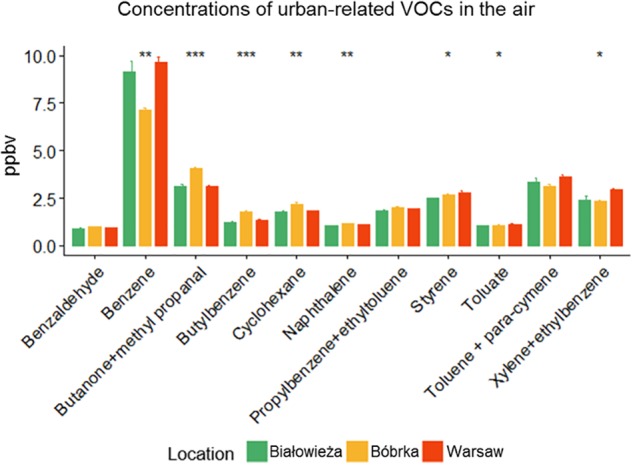
Concentrations of selected urban-related volatile organic compounds (VOCs) (in ppbV). The sampling date was May 30, 2016, for Białowieża and Warsaw, and June 1, 2016, for Bóbrka. Significant differences between sites were tested by a Kruskal–Wallis H test (^∗^*p* ≤ 0.05, ^∗∗^*p* ≤ 0.01, ^∗∗∗^*p* ≤ 0.001, *n* = 3).

Besides PM deposition, the concentrations of VOCs in the ambient air were determined using PTR-TOF of field-collected samples (Figure 2). Białowieża had the lowest concentration of urban traffic-related VOCs compared to Warsaw and Bóbrka, where the following volatiles were enriched: butatone-methyl propanal, cyclohexane, toluene, styrene, benzaldehyde, xylene, ethyltoluene, naphthalene, butylbenzene, and toluate. We detected a 10–20% higher level of benzene, xylene and ethylbenzene in Warsaw city in comparison to the forest.

### Trace Metals on Hornbeam Leaves

In addition to VOC analyses and bulk PM concentration measurements, the levels of trace metals in PM deposited on the leaves were determined for all sites (Table 2). Among the trace metals, platinum (Pt) was fourfold higher in Bóbrka than in Warsaw and Białowieża, rhodium (Rh) was highest in Warsaw at 10–26% more than in Bóbrka and Białowieża, and palladium (Pd) was 4–23% higher in Warsaw and Bóbrka compared to Białowieża. Among the macronutrients, magnesium (Mg) was found at the highest levels in Bóbrka, approximately 7% higher than on leaves at the other sites; phosphorous (P) was 8–9% higher in Białowieża than the other sites, while calcium (Ca) levels were highest in Warsaw, with up to 36% higher concentrations compared to Białowieża. For micronutrients, copper (Cu) was highest in Białowieża at 31% higher concentrations than in Warsaw; zinc (Zn) had the highest concentration in Bóbrka, at 5% and 9% more than in Białowieża and Warsaw, respectively (Table 2).

**Table 2:** Element concentration (average ± standard deviation, mg kg^-1^ DW) in the washwater of hornbeam leaves.

mg kg^-1^ DW	Białowieża	Bóbrka	Warsaw
**Macronutrients**			
Mg	1.79 ± 0.42	1.94 ± 0.59	1.79 ± 0.43
P	1.66 ± 0.23	1.41 ± 0.25^∗∗∗^	1.39 ± 0.19^∗∗∗^
Ca	9.73 ± 1.65	11.44 ± 1.67^∗∗∗^	13.26 ± 2.37^∗∗∗^
S	1.21 ± 0.14	1.39 ± 0.19^∗^	1.64 ± 0.69^∗∗∗^
K	3.70 ± 0.56	4.15 ± 0.32^∗∗∗^	4.81 ± 0.75^∗∗∗^
**Micronutrients**			
Fe	0.060 ± 0.0100	0.090 ± 0.0130^∗∗∗^	0.160 ± 0.0300^∗∗∗^
Mn	1.660 ± 0.5000	1.000 ± 0.7800^∗∗∗^	0.120 ± 0.1600^∗∗∗^
Cu	0.004 ± 0.0010	0.001 ± 0.0010^∗∗^	0.003 ± 0.0010^∗∗^
Zn	0.025 ± 0.0070	0.026 ± 0.0080	0.024 ± 0.0050
**Trace metals**			
Na	0.0150 ± 0.0030	0.0140 ± 0.0070^∗∗∗^	0.0420 ± 0.0170^∗∗^
Pb	0.0006 ± 0.0004	0.0007 ± 0.0004	0.0006 ± 0.0014
Pt	0.0010 ± 0.0001	0.0040 ± 0.0080^∗∗^	0.0010 ± 0.0010^∗^
Rh	0.0002 ± 0.0001	0.0002 ± 0.0001	0.0003 ± 0.0001^∗^
Pd	0.0002 ± 0.0001	0.0003 ± 0.0001	0.0003 ± 0.0001

*The statistical differences were tested with one-way ANOVA tests followed by Tukey–Kramer *post hoc* tests: ^∗^*p ≤* 0.05, ^∗∗^*p ≤* 0.01, and ^∗∗∗^*p* ≤ 0.001; *post hoc* results of the comparison with Białowieża*.

### Bacterial Community Composition

The concentration of DNA extracted from the leaves (Supplementary Table S2) ranged from 1.27 to 13.3 ng μl^-1^ with, on average, higher DNA concentrations in Warsaw (6.91 ± 3.7 ng μl^-1^; *t*-test *p* = 0.06) than the forest locations (3.9 ± 2.3 ng μl^-1^). Equimolar concentrations of DNA were used for shotgun metagenome sequencing, and this generated a total of 94,864,517,100 bp paired-end quality-filtered reads, with an average and standard deviation of 5,270,251 ± 518,665 reads per sample.

**Figure 3:**
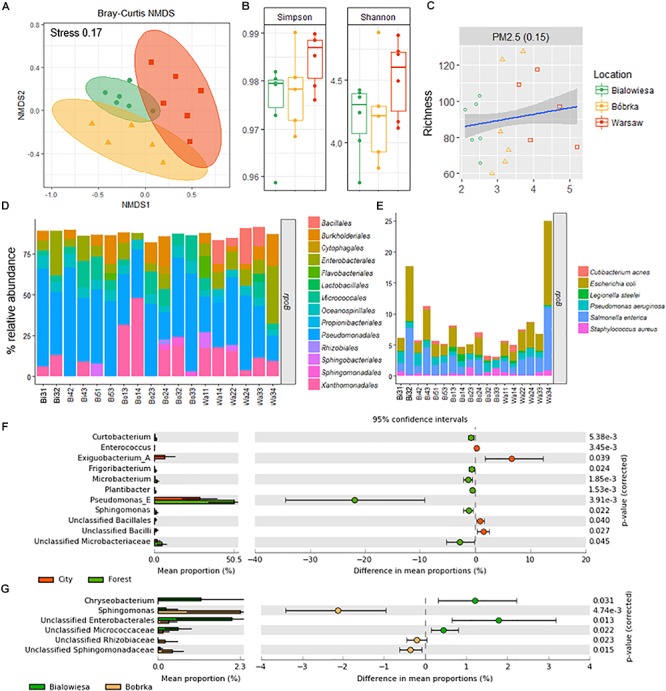
Diversity analyses and relative abundance bar charts of shotgun-derived operational taxonomic unit data. **(A)** NMDS plot based on the Bray–Curtis dissimilatory matrix, **(B)** alpha-diversity box plots, **(C)** correlation plot showing PM_2.5_ concentrations against species richness, **(D)** bar charts showing the bacterial community composition in % relative abundance relative to *rpoB*, **(E)** subplot of **(D)** showing human- and disease-related bacterial genera, **(F)** differentially abundant taxa in the city (Warsaw) vs. the forest (Białowieża and Bóbrka), and **(G)** differentially abundant taxa in the natural forest in Białowieża compared to the oil-polluted forest in Bóbrka (*p* < 0.05, Welch’s *t*-test using STAMP).

Different shotgun microbiome analysis methods were used to determine the hornbeam leaf microbiome structure: CLARK on the 16S rRNA gene (Ounit et al., 2015) (Supplementary Data S1), Kaiju using a protein-level classification against the NCBI NR+euk database (Menzel et al., 2016) (Supplementary Data S2), and MetAnnotate on the *rpo*B gene (Petrenko et al., 2015; Figure 3D, and Supplementary Data S3). All methods were in relative good accordance with each other in predicting the microbiome community structure in the higher-level phylogenetic groups and their relative abundances, though MetAnnotate gives the highest resolution of the three (Supplementary Figure S2). The most dominant group of bacteria colonizing hornbeam leaves were Proteobacteria (Supplementary Data S1–S3), in particular the class Gammaproteobacteria (71%), followed by Actinobacteria (9%), Betaproteobacteria (8%), Firmicutes (4%), Bacteroidetes (4%), and Alphaproteobacteria (3%). Within the Gammaproteobacteria, Pseudomonadales (39%), Xanthomonadales (13%), Enterobacteriales (10%), and Oceanospirillales (6%) made up the majority of this group (Figure 3D). Betaproteobacteria were represented by Burkholderiales (7%) and Sphingomonadales (1%), while Actinobacteria were represented by Micrococcales (7%). Firmicutes were represented by the Bacillales (4%), and Bacteroidetes by the Flavobacteriia (2%) and Sphingobacteria (1%).

The Kaiju krona charts also provide insights into the fungal communities because the NCBI NR+euk database was used (Supplementary Data S2). For all three locations, the fungi take in a small proportion compared to the bacteria, 1% for Warsaw and Białowieża and 2% of the total for Bóbrka (Supplementary Data S2). The most dominant group of fungi is the Ascomycota (57–68% of the fungal reads) for all three locations, in particular the Pezizomycotina. In Warsaw, this group of Pezizomycotina is dominated at 66% by Dothideomycetes, and 48% by Aureobasidia, but in Bóbrka and Białowieża, besides Dothideomycetes (43–57%), Sordariomycetes (22–36%), and Eurotiomycetes (14–15%) also play a part, with numerous sub-groups compared to Warsaw.

Based on beta-diversity analyses using the species operational taxonomic unit (OTU) table, the dissimilarity between the microbial communities according to location is significant (PERMANOVA: F = 2.51, *p* < 0.002) (Figure 3A). The taxonomic community composition based on the single-copy *rpo*B gene showed that the samples are indistinguishable at the order level (Figure 3D). A slightly increased Shannon and Simpson diversity was observed in Warsaw versus the forest samples (Figure 3B), though not statistically significant at *p* = 0.05, and a small positive correlation (*R* = 0.15, *p* = 0.01) between PM_2.5_ and increased richness is evident (Figure 3C). Despite the three sampling locations showing different characteristics and being located several hundreds of kilometers from each other (Figure 1), the phyllospheric bacterial communities were not distinguishable at the level of these taxonomic measurements.

At the species level, potentially human-associated bacteria of the Enterobacteriales (*Escherichia coli* and *Salmonella enterica*) made up as much as 25% of the microbial community at Warsaw location 3. Across all samples from all sites, no trend in the presence of human-associated strains was observed between urban and forest sites. In Białowieża and Bóbrka, a selection of human-associated species (*Cutibacterium acnes*, *E. coli, Legionella steelei, P. aeruginosa, S. enterica, Staphylococcus aureus*) made up 5% of the reads.

The strains that were statistically enriched in the urban environment include *Exiguobacterium* (Bacillales), unclassified Bacilli, and Enterococci (Enterobacteriales), while on the contrary, *Pseudomonas* sp. were 20–30% more abundant in the forest samples (White’s non-parametric *t*-test, *p* < 0.05) (Figure 3F). Between Białowieża and Bóbrka, *Sphingomonas*, unclassified Sphingomonadaceae, and Rhizobiaceae were enriched in Bóbrka, while *Chryseobacterium*, unclassified Enterobacteriales, and unclassified Micrococcaceae were enriched in Białowieża (White’s non-parametric *t*-test, *p* < 0.05) (Figure 3G).

**Figure 4:**
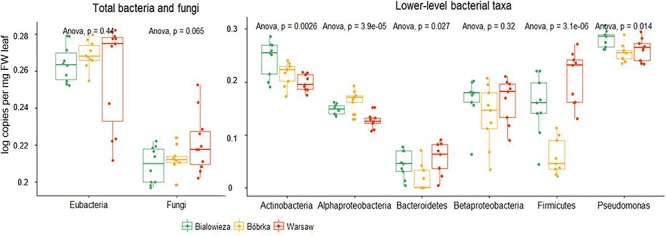
qPCR analyses of total bacteria and fungi in the hornbeam phyllosphere.

The absolute abundance of some selected bacterial groups was determined by quantitative polymerase chain reaction (qPCR) (Figure 4). There was no statistical difference in log copy number of Eubacteria on hornbeam leaves between the locations. On the whole, the highest heterogeneity in log copy number for all of the bacterial groups quantified was found in the Warsaw samples, which were taken near traffic intersections, while the inter-sample variability was lower in forests. Log copy number of fungi was statistically higher in Warsaw samples (ANOVA, *p* = 0.065) compared to forest sites.

The most dominant community members in terms of 16S rRNA gene copy numbers are *Pseudomonas* (Gammaproteobacteria), followed by Actinobacteria (Figure 4), which is in agreement with the shotgun metagenome sequencing data. We quantified the group pseudomonads, as qPCR primers with sufficient specificity for the entirety of Gammaproteobacteria could not be constructed, and found that *Pseudomonas* dominated Białowieża samples, in contrast to samples from Warsaw and Bóbrka. Other results in line with the shotgun sequencing data include the higher copy number of Firmicutes in Warsaw compared to Białowieża and Bóbrka (*p* < 0.001). The Actinobacteria gene copy number was higher in Białowieża (*p* < 0.001), and that of Alphaproteobacteria was higher in Bóbrka (*p* < 0.0001).

### Culturable Diversity of the Hornbeam Phyllosphere Microbiota

**Figure 5:**
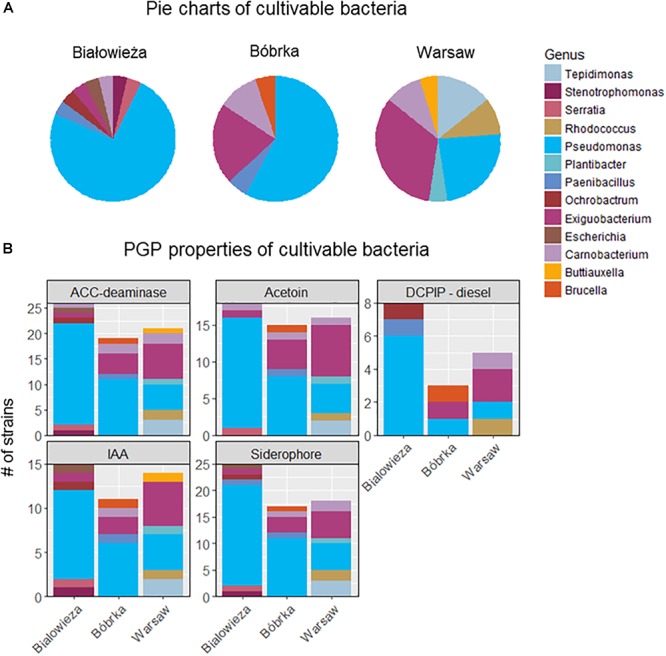
**(A)** Distribution of cultivable hornbeam leaf epiphytes from Białowieża, Bóbrka, Warsaw. **(B)** Their plant growth promoting properties.

Cultivable bacteria obtained from hornbeam leaves were dominated by Proteobacteria, especially Gammaproteobacteria, with the highest number of cultivated *Pseudomonas* spp. being from samples in Białowieża and Bóbrka, while in samples from Warsaw, the genus *Exiguobacterium* (Bacilli) was the most dominant isolate (Figure 5A). Overall, members of the dominant *Pseudomonadaceae* were most successfully cultured, followed by Alpha- and Betaproteobacteria, Actinobacteria (*Tepidomonas*), and Firmicutes (*Paenibacillus, Bacillus*). Interestingly, on the DNA level, *Exiguobacteria* were enriched in the city, corresponding with the isolation of more active culturable members of this genus in Warsaw compared to Bóbrka and Białowieża.

The isolated strains were tested for various PGP properties (Figure 5B). The strongest PGP properties were measured in strains from Białowieża, as reflected by results for ACC-deaminase, acetoin, indole acetic acid (IAA), and siderophore production. Most of these properties are attributed to strains of the genus *Pseudomonas* and, in lower numbers, to the genera *Exiguobacterium*, *Carnobacerium* (Bacilli), *Tepidimonas*, and *Rhodococcus*. The most diesel-degrading taxa were isolated from Białowieża, almost all belonging to *Pseudomonas*.

### Functional Analyses

The shotgun metagenome sequence data were analyzed in Atlas using the function genecatalog, to generate a table of functional diversity, and also, FMAP was used to statistically test hypotheses about the functional potential of hornbeam communities.

**Figure 6:**
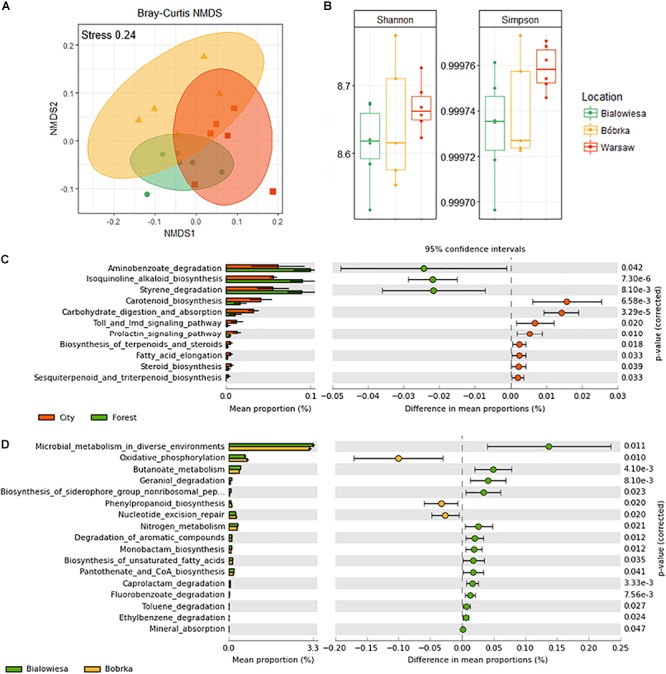
Diversity analyses of a shotgun-derived functional gene catalog and overview of differentially abundant metabolic pathways in communities from three locations. **(A)** Non-metric multidimensional scaling (NMDS) plot based on the Bray–Curtis distance matrix of shotgun predicted functional categories (KEGG orthologs), **(B)** Shannon and Simpson indices of the functional diversity profiles, **(C)** differentially abundant functional categories in the city vs. forest samples, and **(D)** differentially abundant functions in Białowieża vs. Bóbrka (*p* < 0.05, Welch’s *t*-test using STAMP).

An NMDS plot using the functional gene abundance table (Figure 6A) shows that the communities are functionally different based on the PERMANOVA test (PERMANOVA: F = 3.2, *p* < 0.003). There is a trend toward higher Simpson diversity in Warsaw versus the forest samples, though this trend is not significant at *p* < 0.05 (Figure 6B).

In order to better understand differences in metabolic potential between the microbial communities at different sites, KEGG orthological functional groups were filtered down to the 11 most abundant pathways (Welch’s *t*-test, *p* < 0.05; effect size >1.5) and sorted from highest to lowest effect size (Figure 6C). In the forest samples, there was an enrichment of genes involved in aminobenzoate degradation, alkaloid biosynthesis, and styrene degradation, while carotenoid biosynthesis, carbohydrate digestion, Toll signaling pathways, biosynthesis of terpenoids, and steroid biosynthesis, among others, were more prevalent in Warsaw samples (Figure 6C and Supplementary Figure S3). Comparing Białowieża with Bóbrka, more pathways were enriched in the natural forest of Białowieża compared to the oil-polluted site in Bóbrka (Welch’s *t*-test, *p* < 0.05; effect size >1.5); pathways of interest include general microbial metabolism, butanoate metabolism, and degradation pathways for geraniol, caprolactam, fluorobenzoate, toluene, and ethylbenzene, as well as pathways involved in nutrient provision, such as nitrogen metabolism, mineral absorption, and siderophore biosynthesis.

Following from this, a more detailed analysis of differentially abundant genes was carried out by using the FMAP comparison function to filter by log fold change of 1.5 and *p* < 0.05. There were 17 pathways differentially enriched between the forests and the city, and these pathways included 433 differentially abundant genes, of which 247 were more prevalent in the city (Supplementary Figure S4). For the ABC transporters, 60 genes were more enriched in the city (red, Supplementary Figure S5), and 56 were less prevalent (light blue, Supplementary Figure S5). Some upregulated genes in Warsaw city center include those involved in nickel transport (*NikA-E*), manganese, iron, zinc, and biotin, in addition to genes related to oligosaccharide transport (galacotse, raffinose, L-arabinose, xylobiose). Genes that were more prevalent in the forest include those related to glycine, proline, and taurine metabolism; sorbitol transport; amino acid transporters for arginine, glutamate, general L-amino acids, and urea; and peptide transporters. Regarding biofilm formation, two genes were more prevalent in the city, while 19 were more prevalent in the forest, including genes for flagellar biosynthesis units (*FleQ*) (Supplementary Figure S6). For starch and sucrose metabolism, 19 genes were more prevalent in the city, and 5 less prevalent, while for vancomycin antibiotic resistance, 7 genes were more prevalent in the city. Comparing Białowieża with Bóbrka, there were a total of 1,375 differentially abundant genes in 39 pathways (Supplementary Figure S7), of which 1,352 were more prevalent in Bóbrka, including genes for ribosome biosynthesis, RNA polymerase, cell cycle regulation, transcription factors, endocytosis, RNA transport, and DNA replication.

### Aliphatic Hydrocarbon and Aromatic Compound Degradation

One of our hypotheses was that hornbeam phyllosphere microbial communities in the city, and an oil-polluted forest, would harbor more urban and oil-related VOC-degrading genes than the natural forest. To test this, we used MetAnnotate, a software package that screens open reading frame (ORF) files with HMM profiles and assigns taxonomic identifications to the gene hits. A selection of genes involved in both aliphatic and aromatic compound degradation were chosen for analyses, including: benzoate 1,2-dioxygenase (*benA*), benzene 1,2-dioxygenase (*bphA1*), toluene dioxygenase (*bph*), dibenzofuran dioxygenase (*dbfA2*), naphthalene 1,2-dioxygenase (*npah*), alkane monooxygenase B (*AlkB*), xenobiotic reductase B (*xenB*), flavin oxidoreductase (*baiCD*), cytochrome P450 (*p450*), NAD(P)H-dependent oxidoreductase (*pnrB*), and xenobiotic reductase A (*xenA*).

**Figure 7:**
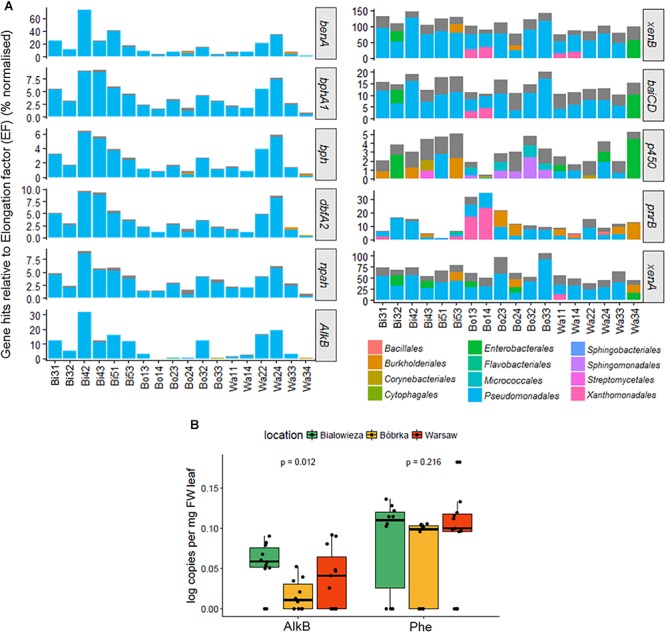
**(A)** Bar charts showing the percentage degradation gene hits relative to the transcription elongation factor (EF) gene using MetAnnotate. **(B)** PCR results of alkane monooxygenase B (*AlkB*) and phenol monooygenase (*Phe*) gene copies per mg FW leaf. The plots in **(A)** were filtered to show only the most dominant orders with a relative abundance of 10% or higher.

For the degradation genes *benA, bphA1, dbfA2, npah*, and *alkB* (Figure 7A), the highest relative number of aromatic compound–degrading gene hits was found in Białowieża, and in Warsaw, samples Wa22 and Wa24 from the vehicle intersection, compared to the other samples. qPCR analyses of the AlkB gene confirm these findings and show a higher AlkB log copy number in Białowieża compared to Bóbrka and Warsaw (Figure 7B). qPCR results were less conclusive for phenol hydroxylase because of the relatively high site-to-site variation (Figure 7B). For *xenB*, *baiCD*, *p450*, *pnrB*, and *xenA* (Figure 7A), a more diverse set of organisms carrying the degradation genes was detected, although the majority of hits were against *Pseudomonas* species. Interestingly, *pnrB* was represented by the Xanthomonadales in Bóbrka location 1 samples, and up to 30% of the bacterial community members in samples Bo13 and Bo14 contained *pnrB*, as normalized against the single-copy EF gene. Other genes, specifically *xenB*, *xenA*, *cytochrome p450*, and *baiCD* did not show significantly different patterns between the locations, and a majority of the strains in the communities harbor these genes, especially *xenB*, a gene that appears to be present in more than one gene copy number in most community members.

### Metagenome-Assembled Genomes

The relatively low microbial community diversity of the phyllosphere microbiomes examined here allowed for the assembly of 17 medium- to high-quality metagenome-assembled genomes (MAGs) (Supplementary Figure S8) using the ATLAS binning pipeline. MAGs were recovered with contamination at <30% and completeness at >90% for five *Pseudomonas* spp., seven *Stenotrophomonas* spp., two *Pantoea* spp., one *Exiguobacterium*, and two *Pedobacter* spp. (Bin report in Supplementary Data S4). Because of the extensiveness of individual genome analyses, annotation, and curation, more in-depth comparative analyses of these MAGs will be presented in a follow-up manuscript.

## Discussion

With this study, we aimed to explore the previously uncharacterized hornbeam phyllosphere microbiome and its relationship with urban and rural pollution. Hornbeam is an important ornamental tree that is widely used for landscaping in gardens and city parks, where it is often used as hedges. The hornbeam tree is common in forests in Western, Central, Eastern, and Southern Europe and is also native to Western Asia. Hornbeam keeps its leaves long into winter, and before shedding, it is quite resistant to pollution, thrives well in slightly acidic soils, likes both full sun and shade, and requires moderate soil fertility and moisture. These properties make this plant an ideal subject for airborne pollutant sequestration, particularly PM, VOCs, and inorganic air pollutants.

PM is a complex mixture that consists of solid particle material and bound inorganic and organic aromatic compounds, including mono- and polyaromatics, volatiles, and semivolatiles, which can cause harmful effects to humans when inhaled. From previous experiments in our laboratory, we learned that hornbeam plants show significant PM-capturing capacities (SG, pers. comm.). One of our questions here was: does the microbiome of hornbeam change when growing in an urban environment with urban-related VOCs as compared to trees that grow in a natural forest? To go a step further, we questioned what would happen with the phyllosphere microbiome if trees, in a forest, are chronically exposed to oil vapors from upwelling oil pits. We selected Warsaw, “the Beijing of Europe,” as a good example of a European city that is suffering year-round from high levels of airborne pollution. In January 2017, peaks as high as 437,000 ng m^-3^ PM_10_ were recorded, almost 10 times higher than the regulated EU maximum of 50,000 ng m^-3^. Concerning VOC pollution, Warsaw has severe problems with high concentrations of the carcinogen benzo[*a*]pyrene. Epidemiological studies have estimated that air pollution is associated with an increased risk of infant mortality (Scheers et al., 2011) and lowers average life expectancy by 1 year (WHO).

Our results provide the first detailed insights into the hornbeam phyllosphere microbiome, in addition to the relationship of a number of those microbiomes with airborne pollution in Poland. Bacterial community structures were found to cluster according to the forest location versus Warsaw according to NMDS and PERMANOVA test. Functionally, although the communities overlapped, we found more genes and pathways related to alkane/alkene and aromatic compound degradation in the natural forest phyllomicrobiome in contrast to Warsaw and Bóbrka. Deciduous trees naturally produce VOCs, like isoprenes, carbonyls, and aromatics (Isebrands et al., 1999), and these natural biogenic VOCs might explain the high abundance of genes involved in VOC degradation in the phyllosphere. This also indicates that natural forests are rich sources of metabolically diverse bacteria and that anthropogenic pressure, whether traffic or industry, may negatively influence bacterial diversity, or skew the abundance to a few dominant ones, and hence alter leaf microbial functionality. These stressors seem not to be driving forces for xenobiotic compound degradation functions on leaves, which is in sharp contrast to what is often observed for polluted soils. On the other hand, microbial communities from the city center of Warsaw were described by higher Simpson and Shannon diversity indices for both taxonomy and function. This raises questions about whether habitats prone to wind, solar radiations, rain as well as to interaction with human microbiomes are more susceptible to alteration of their microbial communities than more buffered habitats like dense canopy forests.

The airborne pollutants measured here, specifically PM, VOCs, and inorganic air pollutants, although a snapshot in time, are in general agreement with recently published data by the European Commission about PM and air quality in Poland (https://www.eea.europa.eu/themes/air/country-fact-sheets/poland). PM_10_ and PM_2.5_ analyses revealed a significantly higher concentration in Warsaw city center, in particular near the busy vehicle intersection, in comparison to Bóbrka and Białowieża (Table 1). PM_2.5_ sources are mainly transport, burning of fossil fuels, industries, and residences, as demonstrated in a study in China analyzing PM properties (Cao et al., 2012). PM_10_ is composed of dust, sand, and plant pollen and thus is a weaker indicator of traffic exposure (Chow et al., 2015). In Warsaw city, the annual exposure to both PM_10_ and PM_2.5_ regularly exceeds the EU target values for human health (Figure 1). Trace metals, including some rare earth elements, were found on leaves in all locations, with higher concentrations of rhodium, palladium, and calcium in Warsaw (Table 2). Palladium is commonly used in catalytic converters in gasoline engines and electric vehicles, which may explain the higher concentrations in the city. Lead was 21% higher in Bóbrka than in Białowieża and Warsaw, which may be due to historic oil processing methods or fuel enrichment additives (antiknock agents). The concentration of VOCs in air samples from Bóbrka and Warsaw confirmed higher concentrations of aromatic hydrocarbons such as toluene, styrene, xylene, and naphthalene compared to the natural forest at Białowieża (Figure 2). Some of these VOCs, particularly benzene, were at higher concentrations in the city center. A time-of-flight–based PTR-MS is a powerful analysis instrument for airborne pollutant VOC characterization, with a detection limit of ≈15 parts per trillion and with a mass resolution of 1/1,000 mass units at *m*^+^/*z* = 20 and 1/450 mass units at *m*^+^/*z* = 100 (x.0010 and x.0023 a.m.u., respectively). The high mass resolution is needed for peak discrimination when natural forest VOCs have a similar mass to the VOCs of study and the peaks partially overlap in the spectrum, e.g., norbornene (C_7_H_10_)H^+^ m^+^/z = 95.0861 and phenol (C_6_H_6_O)H^+^ m^+^/z = 95.0497, or benzaldehyde (C_7_H_6_O) H^+^ m^+^/z = 107.0491 and xylene+ethylbenzene (C_8_H_10_)H^+^ m^+^/z = 107.0860 (Portillo-Estrada et al., 2018).

Shotgun microbial community analyses revealed a significant location effect (Figure 3). Also, others have found a large component of environment influences such as geographic location on the composition of phyllosphere microbiomes (Koskella, 2013; Kembel et al., 2014). Of course, the so-called plant holobiont is shaped by a variety of complex factors (Sanchez-Canizares et al., 2017), and phyllospheric bacteria can be recruited physically from air, soil, and seeds (Bai et al., 2015; Sanchez-Canizares et al., 2017). Although we may have defined some tree- and location-specific taxa (Figure 3F), further investigations are needed to define with more certainty key bacterial strains in hornbeam microbial communities.

It has been postulated that keystone bacterial species may not be affected by environmental change because they play a critical role in plant fitness and are symbiotically adapted to host plant leaves (Agler et al., 2016). To better understand this, time course studies over the seasons are required, as when a new environment (such as a new leaf) is first colonized, there will be a rapid increase in bacterial diversity, followed by development of a more stable community over time, a community that will persist until a disturbance forces a shift (Sanchez-Canizares et al., 2017). From an evolutionary community ecology viewpoint, the diversity of microorganisms on leaves will also be partly explained by dispersal and diversification, and internal selection via biotic interactions such as competition or parasitism that regulate the fine structure of the microbial community (Vellend, 2010). Hornbeam is used as a food plant by caterpillars of a number of Lepidoptera, such as the hornbeam caterpillar, *Orgyia leucostigma*. Besides caterpillars, aphids are known to feed from the leaves and produce a sticky honeydew covering the leaves, and this may well influence food sources available for the leaf microbiota.

We noticed a slight trend between increasing PM_2.5_ and increased richness in the bacterial communities examined. Previous investigations highlighted that a high concentration of airborne pollutants may provide nutrients, such as SO_4_^2-^ and Ca^2+^, for growth, thus affecting the bacterial community structure resident on submicron particles (Michiels et al., 2002; Xu et al., 2017). It might be that the airborne PM in the Warsaw city center provides inorganic and organic nutrient sources for the phyllosphere microbiota of hornbeam, contributing to a wider richness of microbial species compared to a dense natural forest.

While we observed a Gammaproteobacteria-dominated phyllosphere community for hornbeam (Figure 3, 4), Kembel et al. (2014) found that the bacterial communities in the phyllospheres of 57 tree species were dominated by Alphaproteobacteria, followed by Gammaproteobacteria and Sphingobacteria. Their study described that host attributes such as plant taxonomic identity and phylogeny, growth and mortality rate, wood density, leaf mass per area, and leaf nitrogen and phosphorous concentrations correlated with bacterial community structure; thus, it is not unreasonable to obverse a different dominant microbial group in hornbeam microbiomes. For example, the *Arabidopsis thaliana* phyllosphere microbiome was dominated by Alphaproteobacteria, followed by Gamma- and Betaproteobacteria, Actinobacteria, and Bacteroidetes (Bodenhausen et al., 2013). Other studies have described phyllosphere microbial profiles of diverse plant species using amplicon sequencing approaches (Vorholt, 2012; Laforest-Lapointe et al., 2016; Muller et al., 2016), metaproteomics (Knief et al., 2011), and fluorescence *in situ* hybridization (Remus-Emsermann et al., 2014), highlighting the dominance of several generalist taxa belonging to the Proteobacteria, Bacteroidetes, and Actinobacteria (Delmotte et al., 2009; Knief et al., 2011), as well as the presence of specialist taxa, such as *Methylobacterium*, *Pseudomonas*, and *Sphingomonas*, adapted to particular phyllospheric environments (Knief et al., 2010). Here there was a higher abundance of *Exiguobacteria* and Bacilli (Firmicutes) on hornbeam leaves in the city compared to the forests. Firmicutes are Gram-positive bacteria, well known for being highly resistant to desiccation and extreme life conditions, which might be more extreme in the city phyllosphere than inside a forest. Firmicutes have also been detected in most diverse environments, including the phyllosphere of many agricultural and native plants (Williams and Marco, 2014).

Although one would expect more human-related taxa, such as pathogens, in the city center, this was not confirmed by our study. One exception is the samples taken at Warsaw location 3, where a relative high abundance of *E. coli* and *S. enterica* was found on the leaves; this may be related to human pedestrian traffic, as the location is a city park. Besides this, *Enterobacteriales* have been detected consistently in the phyllosphere of many species (Laforest-Lapointe et al., 2016). The question remains, however, whether some of these potential human pathogenic strains or commensals are in direct correlation with disease outbreaks, drinking water contamination, asthma, skin allergies like eczema, and acne (e.g., by *Propionibacterium* and *Cutibacterium acnes*) (Figure 3E).

With respect to potential aromatic pollutant degradation capacities of members of the hornbeam phyllosphere microbiome, our various analyses indicate a higher prevalence of catabolic genes in the natural forest compared to the city and oil-polluted forest (Figure 5–7). At first glance, it may appear surprising that a national nature reserve could harbor the highest number of bacteria able to tolerate and degrade aromatics; on the other hand, it may be justifiable considering that the forest of Białowieża is an undisturbed primeval forest, which is a reservoir not only for diverse flora and fauna, but for microbial diversity as well. Many degradation genes in the metagenomes sequenced here were correlated with *Pseudomonas* species (Figure 5, 7). Pseudomonads are well-known xenobiotic degraders, with high tolerance to toxic pollutants and the metabolic versatility to partially or completely biodegrade a range of aromatic hydrocarbons (Haigler et al., 1994; Caballero et al., 2005; Cao and Loh, 2008; Brown et al., 2012; Krell et al., 2012). The high abundance of Pseudomonadales (>60%) observed here might be explained by their competitive nature for acquisition of habitat and resources. The generalist nature of pseudomonads is reflected in their relatively large genome sizes (Moreno, 1998; Thijs et al., 2016). Besides *Pseudomonas* spp., aromatic compound degradation genes were also found in Xanthomonadales, and the DCPIP assay scored positive for strains of the hydrocarbonoclastic genera *Stenotrophomonas*, *Rhodococcus*, *Carnobacterium*, *Paenibacillus*, and *Brucella* (Figure 5; Arulazhagan et al., 2017). While none of the isolated strains were able to consume naphthalene or benzene for carbon and energy on their own under the conditions used, bioinformatic analyses showed that homologs of ring-hydroxylating dioxygenases were present in the communities; thus, more targeted selective enrichments might be needed to enhance the cultivability of aromatic hydrocarbon degraders.

As such, phyllosphere microbiome reconstitution experiments can be conducted under controlled conditions to study the VOC degradation capacity over time, as was done for *A. thaliana* (Bodenhausen et al., 2014). All strains cultivated in this study were also evaluated for plant growth promotion traits (ACC-deaminase, acetoin, IAA, and siderophore production), and strains isolated from the Białowieża national forest showed the highest percentages of PGP features. The Białowieża forest is the best-preserved forest ecosystem in Europe and the last lowland deciduous and mixed old-growth forest on the continent. Therefore, leaf-dwelling PGP microbial communities may be expected, and centuries of habitat preservation may have supported development of genetic and metabolic diversity, diversity that may be harnessed for detoxification of human environments.

## Conclusion

Although, in general, the phyllosphere can be considered a short-lived environment due to the fact that plants replace their foliage regularly, our study indicates that the phyllosphere communities of hornbeam are not random assemblies of microorganisms, but are instead represented by dominant phyla and distinct community structures in part explained by geographic location. The taxonomic insights provided by our investigations may provide a reliable description of the phyllosphere communities colonizing hornbeam in Poland, although this will have to be confirmed with multi-year studies over a wider geographical range. Screening of the hornbeam microbiome for aromatic compound degradation and plant growth promotion traits, functionally confirmed with several phyllosphere isolates, leads us to hypothesize that our study lays the foundation for a deeper understanding of urban phyllosphere microbiomes and their potential to be manipulated to aid in airborne pollutant degradation.

## Author Contributions

VI and ST wrote the manuscript. LK executed the main part of the experimental work, in which VI assisted with sampling. SG and JV experts in phyllo- and phytoremediation, critically reviewed the manuscript. MP-E performed the PTR-TOF-MS analyses at UAntwerp and revised the manuscript.

## Conflict of Interest Statement

The authors declare that the research was conducted in the absence of any commercial or financial relationships that could be construed as a potential conflict of interest.

## Footnotes

1 https://github.com/pnnl/atlas (accessed April 17, 2018).

2 http://fungene.cme.msu.edu/
