# Supplementary material for: Characterisation of the Carpinus betulus L. Phyllomicrobiome in Urban and Forest Areas
Source: Front Microbiol. 2019 May 29;10:1110. doi: 10.3389/fmicb.2019.01110 (PMC6549492; doi:10.3389/fmicb.2019.01110)
Supplement: Supplementary file 1 [file Table_1.DOCX]

**Supplementary material: Characterisation of the *Carpinus betulus* L. phyllomicrobiome in urban and forest areas**

**Figures**


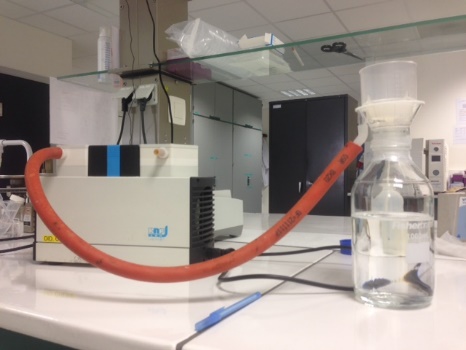


**Figure S1: Experimental set-up to filter PM from leaf wash.**

**
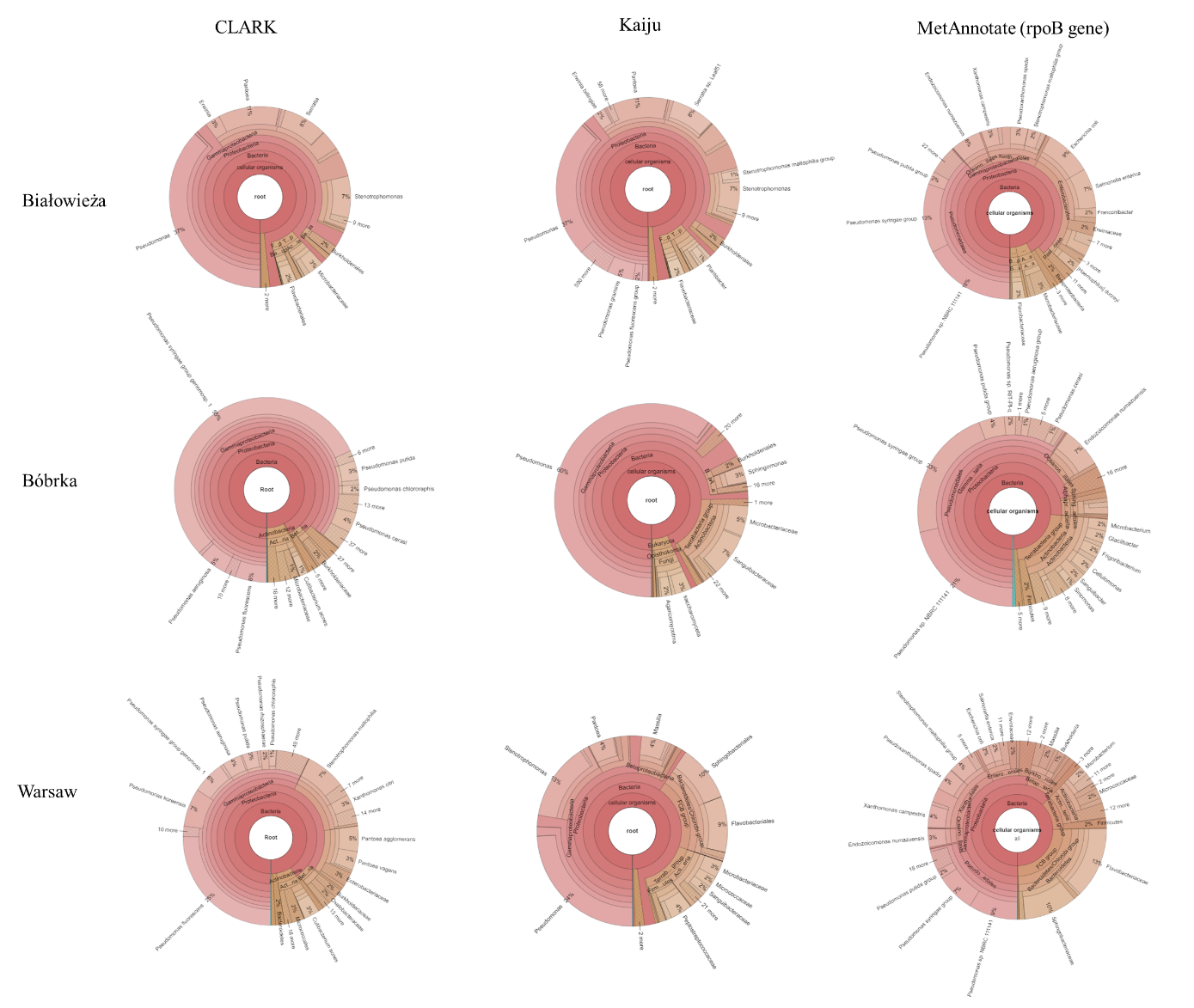
**

**Figure S2: Taxonomy Krona charts for Białowieża, Bóbrka and Warsaw using CLARK, Kaiju and MetAnnotate showing that the three methods largely agree on the hornbeam phyllosphere microbial community composition.** For Kaiju, the NCBI NR+euk, 2018-02-23 database was used. For interactive Krona charts, see Supplementary Data files 1 (CLARK), 2 (Kaiju) and 3 (MetAnnotate).


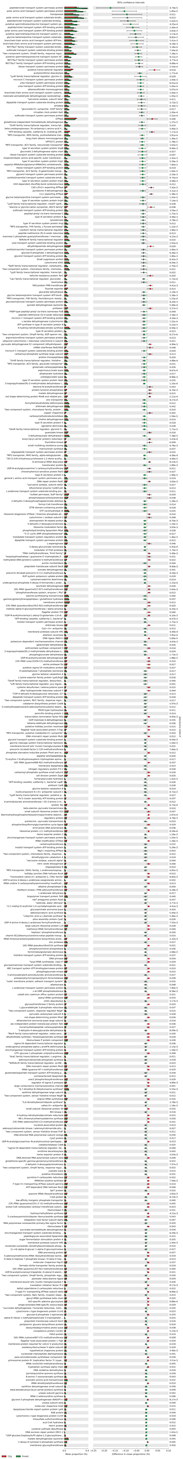


**Figure S3: Differentially abundant gene categories between the forests (Białowieża and Bóbrka) and the city (Warsaw).** This is a detailed version of Figure 6c. Differences calculated on the FMAP functional gene abundance table (*p* < 0.05, Welch’s t-test, in STAMP).


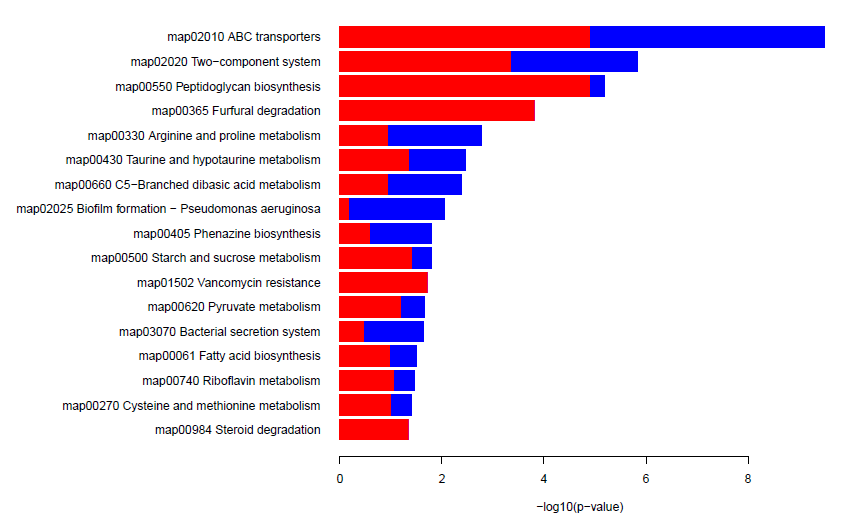


**Figure S4: Differentially abundant pathways in Warsaw versus the forest.** Red means genes within the pathway with a higher prevalence in Warsaw versus forest, and blue a lower prevalence in Warsaw versus forest. (FMAP comparison function, with *p* < 0.05 and logfoldchange > 1.5).

**
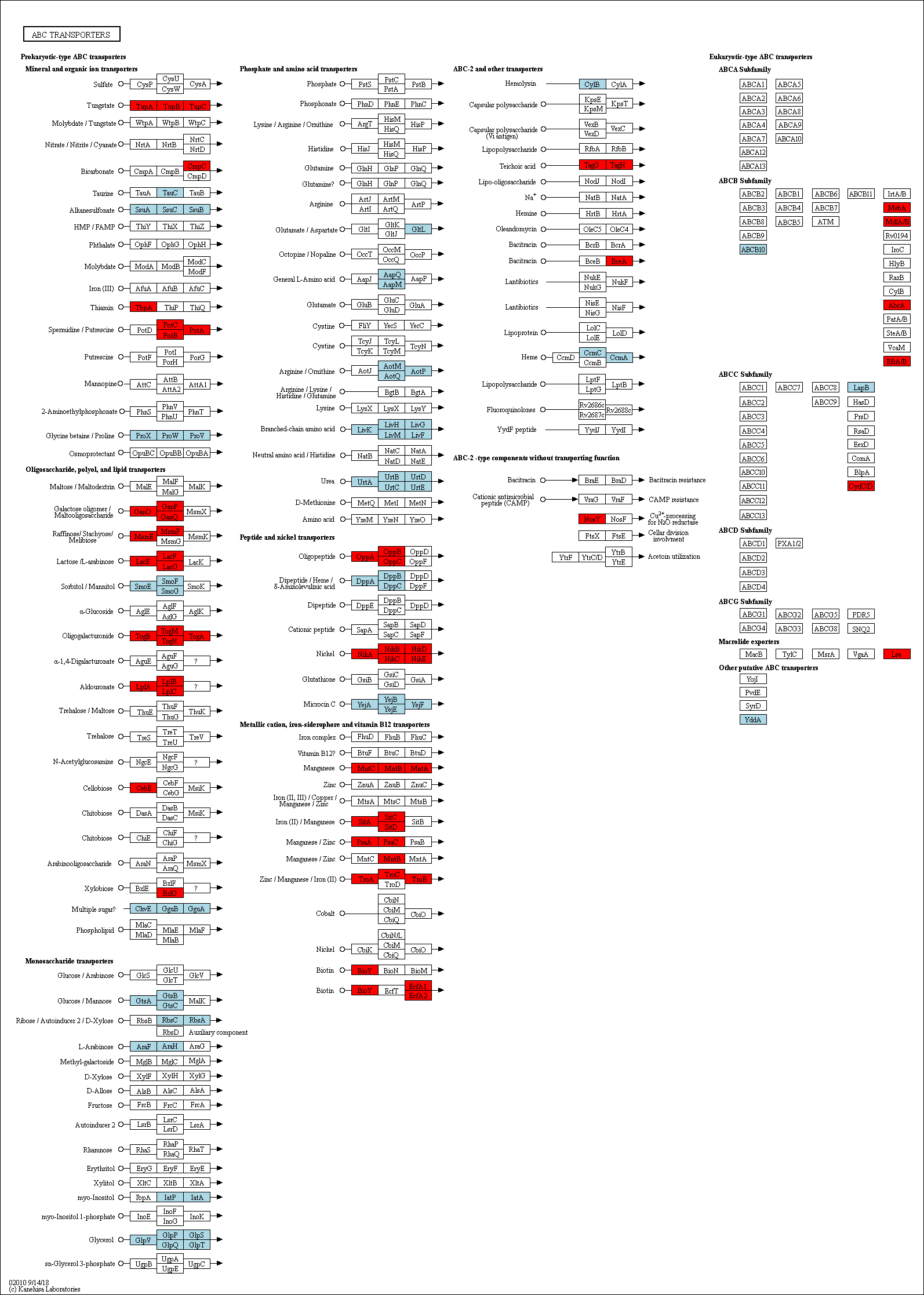
**

**Figure S5: Differentially abundant genes within the category ABC transporters in the city versus the forests.** Sixty genes were more enriched in the city (red) and 56 were more prevalent in the forest (light blue). (FMAP comparison function, with *p* < 0.05 and logfoldchange > 1.5).

**
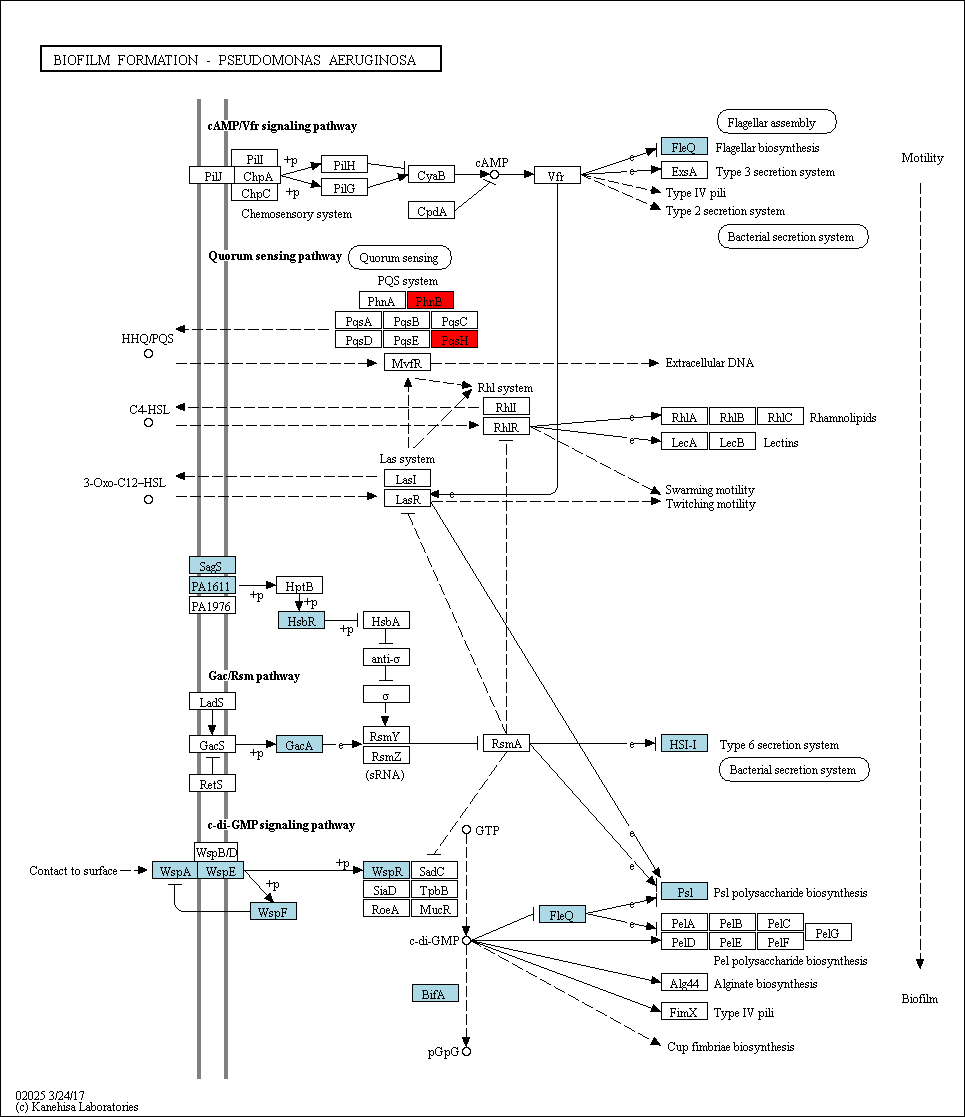
**

**Figure S6: Differentially abundant genes within the category biofilm formation in the city versus the forests.** Two genes were more prevalent in the city (red), while 19 were more prevalent in the forest, including genes for flagellar biosynthesis units (*FleQ*). (FMAP comparison function, with *p* < 0.05 and logfoldchange > 1.5).


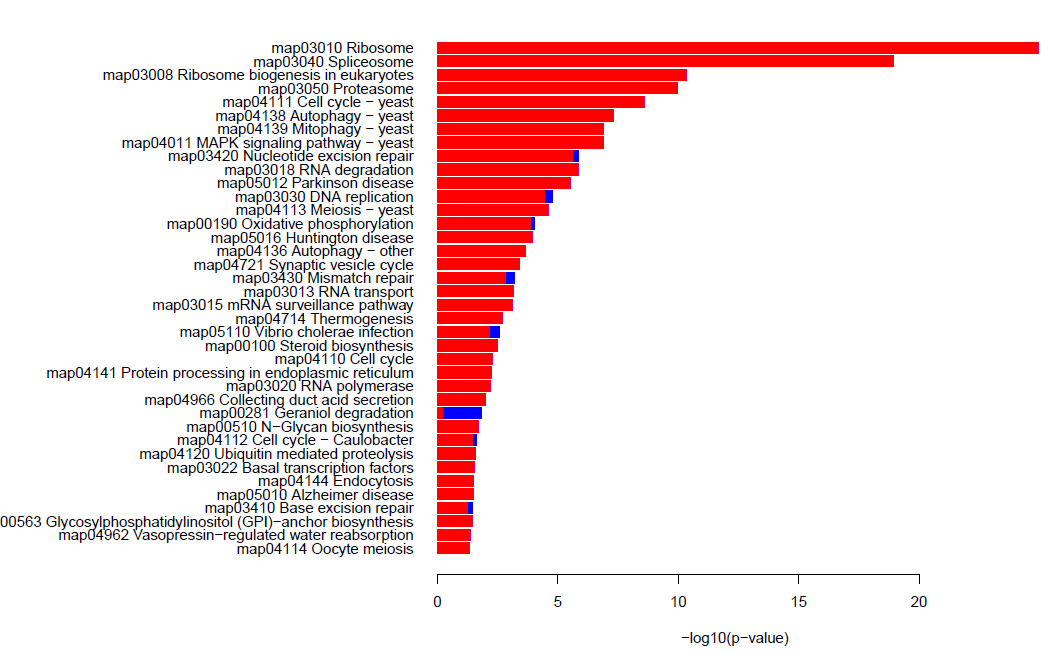


**Figure S7: Differentially abundant pathways in Bóbrka versus Białowieża.** Red means genes within the pathway with a higher prevalence in Bóbrka versus Białowieża, and blue a lower prevalence in Bóbrka versus Białowieża. (FMAP comparison function, with *p* < 0.05 and logfoldchange > 1.5).


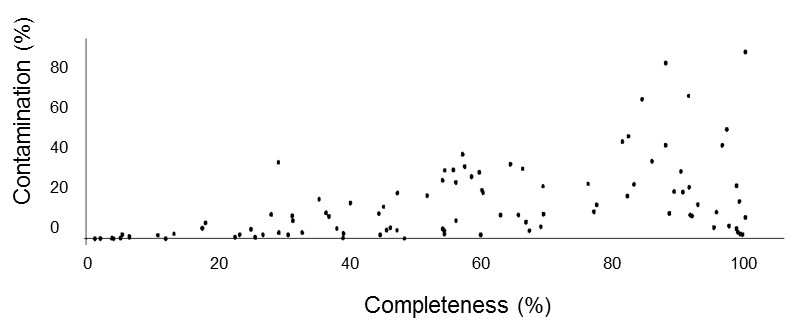


**Figure S8: Hornbeam phyllosphere-associated metagenome assembled genomes (MAGs).** Each dot represents a genome bin showing its degree of completeness (%) and level of contamination (%).

**Tables**

**Table S1**: qPCR primers used, sequence, annealing temperature, concentration and expected length of amplicon. In grey, primers targeting genes related to aliphatic and aromatic hydrocarbon degradation.

**Table S2: DNA-concentrations, raw reads and QC-filtered shotgun metagenomic reads**

| **Location** | **ID** | **Total DNA (ng/µl)** | **Total raw reads** | **QC filtered reads** | **Average QC filtered reads** |
| --- | --- | --- | --- | --- | --- |
| **Białowieża** | Bi32 | 8.95 | 5,291,979 | 5,080,331 | 5,473,305 |
|  | Bi41 | 5.25 | 6,090,690 | 5,753,410 |  |
|  | Bi42 | 2.95 | 6,029,292 | 5,718,881 |  |
|  | Bi43 | 7.1 | 5,088,144 | 4,757,372 |  |
|  | Bi51 | 2.11 | 6,394,221 | 5,983,844 |  |
|  | Bi53 | 6.25 | 5,904,793 | 5,545,992 |  |
| **Bóbrka** | Bo13 | 2.99 | 4,929,808 | 4,325,765 | 5,226,717 |
|  | Bo14 | 2.46 | 6,177,285 | 5,723,366 |  |
|  | Bo23 | 1.46 | 5,067,391 | 4,911,605 |  |
|  | Bo24 | 3.19 | 5,614,801 | 5,264,367 |  |
|  | Bo32 | 3.17 | 6,447,060 | 6,060,462 |  |
|  | Bo33 | 1.27 | 5,219,330 | 5,074,736 |  |
| **Warsaw** | Wa11 | 3.19 | 6,310,933 | 5,873,640 | 5,110,731 |
|  | Wa14 | 7.45 | 5,953,626 | 5,468,720 |  |
|  | Wa22 | 13.3 | 5,036,293 | 4,768,192 |  |
|  | Wa24 | 9.9 | 5,080,612 | 4,813,279 |  |
|  | Wa33 | 4.17 | 5,436,614 | 5,171,239 |  |
|  | Wa34 | 3.45 | 4,819,491 | 4,569,316 |  |
