# Supplementary material for: Characterisation of the Carpinus betulus L. Phyllomicrobiome in Urban and Forest Areas
Source: Front Microbiol. 2019 May 29;10:1110. doi: 10.3389/fmicb.2019.01110 (PMC6549492; doi:10.3389/fmicb.2019.01110)
Supplement: Supplementary file 2 [file Data_Sheet_1.ZIP › CLARK_epWa.html]

Javascript must be enabled to view this page.

members
magnitude
magnitudeUnassigned
count
unassigned
taxon
rank

epWa11
epWa14
epWa22
epWa24
epWa33
epWa34

284027892848269026982448
180143018657591447913226210522521092184859

2157
716975823311496349
705868546447
superkingdom

655359515844
phylum
683932792293488341
28890

class
19171312118
2564832224213260
224756

2191
23551548028
675342
order

2321
family
21465
1198451

121
genus
191
395331

171

358766 
358766 
358766 
 358766 
111
species


882104 
 882104 
2
1
species

475087
1555
genus
1111

 475088 

475088 
475088 
475088 

475088 
1555
species
1111

111111
family
101523215
88404

genus
111111
101523215
2192

101523215
 83984 

83984 
83984 
83984 
83984 
83984 
83984 
111111
species

family
321221
2194
116324313

11111
genus
230355
9212513

species
11111

54120 
54120 

54120 
54120 
54120 
 54120 
9212513

45989
243118
21111
genus

1
species
 86622 

86622 
1

11111
species
 83986 

83986 
83986 
83986 
83986 
83986 
143118

11
family
196137
341

341
2202
11
genus

11
species
341


2203 
2203 
 2203 

111111
order
570264
222121

570265
222121
family
111111

222121
570266
genus
111111

221


1175445 
1175445 
1175445 
 1175445 
111
species

111
species
221

1175444 


1175444 
1175444 
 1175444 

231426205375031
94695
1297865
order

159
143067
family
12

2222
159
12
genus

1
species
13
 301375 


301375 

species
11
146

2223 
2223 
 2223 

1177865
family
2206
230367205375031

21
196136
genus
11

 39669 


39669 

39669 
21
11
species

1
2175
1
genus


2176 
 2176 
1
species
1

1
genus
2225
1


2226 
 2226 
1
species
1

2220
14112
genus
1111

1111
species
 420950 

420950 
420950 

420950 
420950 
14112

957555
genus
228361205343831
2207

111111
species
149274146123022

2208 
2208 
2208 
2208 
2208 
2208 
 2208 

111
species
153

1434102 

1434102 
1434102 
 1434102 

species
1
 1434099 

1434099 
4


170861 
170861 
170861 
170861 
170861 
 170861 
34754
11111
species

11111
species
89313
 38027 

38027 
38027 
38027 
38027 

38027 

32112
 1434103 

1434103 
1434103 
1434103 

1434103 
1434103 
11111
species


2209 
2209 
2209 
2209 
2209 
2209 
 2209 
5672401323
species
111111


2210 
 2210 
1
1
species

species
1

2215 
 2215 
1

1
species


2214 
 2214 
1

33
 1434100 

1434100 

1434100 
species
11

class
689645
183925
150224405179158133

order
689645
150224405179158133
2158

2159
150224405179158133
689645
family

811062231017679
2172
434324
genus

 2173 

2173 
2173 
2173 
2173 
2173 
2173 
6195213997371
111111
species

1288134

294671 
294671 
294671 
294671 
294671 
294671 
 294671 
111111
species

111
species
513

83816 

83816 


83816 
 83816 

 230361 


230361 
1
species
1

species
1111
3311

224719 
224719 
224719 
224719 
 224719 

genus
3221
2160
30331

1822
 2162 


2162 
2162 
2162 
species
111

species
1
11
 59277 


59277 

111
 1911685 


1911685 

1911685 
1911685 
species
111


868131 
 868131 
1
species
1

112
genus
1192
2316

11
species
191


2317 
2317 
 2317 

11
species
11
 1789762 

1789762 

1789762 

111111
genus
145260
6869177758154

111111
species
6869177758154
 145261 

145261 
145261 
145261 
145261 
145261 
145261 

121253
class
85141819
183967

1235850
1321
order
1111

1577788
1321
family
1111

genus
1111
1291539
1321

 1291540 


1291540 
1291540 
1291540 
1291540 
1321
1111
species

12142
order
8511618
2301

83119
46659
1121
family

genus
1121
2302
83119

1
species
1
 2303 


2303 

83109

50339 
50339 


50339 
50339 
 50339 
species
1111

21
90142
family
11

11
genus
21
74968

species
11
 97393 


97393 


97393 
21

species
111
149


1054217 
1054217 
1054217 
 1054217 

445333
class
183939
1391658812916

2182
1391658812916
445333
order

196117
111
111
family

111
genus
111
196118

1
 644281 

644281 
species
1

1
species
1
 67760 


67760 


1301915 
 1301915 
1
1
species

1381648712916
2183
family
334333

2184
1381648412916
333333
genus

species
111111
457132235

42879 
42879 
42879 
42879 
42879 
42879 
 42879 

 39152 

39152 
39152 
39152 
39152 
39152 
39152 
2897225
species
111111

658445846
 2188 

2188 
2188 
2188 
2188 
2188 
2188 
111111
species

155862
3
genus
1

1
species
 155863 


155863 
3

class
635363
183968
21172414203

order
635363
2258
21172414203

family
635363
21172414203
2259

genus
524252
2263
13342112


187880 
 187880 
1
species
1

11
species
11
 122420 


122420 
122420 


1042877 
 1042877 
1
1
species

11
species
 195522 

195522 


195522 
11

species
11

2016361 


2016361 
 2016361 
11

11
species
11
 2008440 

2008440 


2008440 

11111
species
 311400 

311400 
311400 
311400 

311400 
311400 
92171

11
species

163003 


163003 
 163003 
11

 246969 


246969 
1
1
species

1
species
1


2264 
 2264 

species
1


54077 
 54077 
1

111111
genus
2260
814201291


2261 
2261 
2261 
2261 
2261 
2261 
 2261 
814201291
111111
species

1111
class
1112
183980

1111
order
1112
2231

1111
family
2232
1112

2233
112
genus
111

1
species
2


2234 
 2234 

1


58290 
 58290 
species
1

1
species


84156 
 84156 
1

54260
1
genus
1


54261 
 54261 
1
1
species

class
291825242921
183963
109375141151108

281325197347
1644060
order
7511899

281325197347
1644061
7511899
family

36164
63742
11111
genus

11111
species
 13769 

13769 

13769 
13769 
13769 
13769 
36164

11111
genus
614727
29287


29288 

29288 
29288 
29288 
29288 
 29288 
614727
species
11111

 1902251 


1902251 
1902251 
1902251 
1902251 
1902251 
414121
11111
species

121871
14334
12111
genus

1
 588898 


588898 
1
species

13334
 62320 

62320 

62320 
62320 
62320 
62320 
11111
species

genus
111111
314151
1269201


1333523 
1333523 
1333523 
1333523 
1333523 
1333523 
 1333523 
314151
species
111111

genus
111
353799
121

121
 353800 


353800 
353800 


353800 
species
111

1052171
387342
11111
genus

species
11111
1052171

387343 
387343 
387343 

387343 
387343 
 387343 

genus
12211
324104
88723

species
1111
 406552 

406552 

406552 
406552 

406552 
3114

1310
 69525 


69525 
69525 
69525 
species
111

203193
223294
genus
111111

223294
 229731 

229731 
229731 
229731 
229731 
229731 
229731 
species
111111

1
genus
2256
4

species
1
4


44930 
 44930 

order
945475
1644055
2491152214

6663911
1644056
333233
family

genus
111
122
60846


60847 
60847 


60847 
 60847 
122
species
111

293431
12
11
genus

11
species
 293091 


293091 
293091 
12

223212
genus
546369
2251

species
111
121

2246 

2246 
2246 
 2246 

species
111
 2252 


2252 
2252 


2252 
112


35746 
35746 
35746 
35746 
35746 
35746 
 35746 
433267
species
111111

512132
family
1963271
15351123

56688
41
genus
21

31

2247 


2247 
 2247 
species
11

1

29284 
 29284 
1
species

1209988
6132
genus
1111

species
1111
6132

1048396 

1048396 

1048396 
1048396 
 1048396 

1644057
434181
genus
111111

species
111111
434181

755307 
755307 
755307 
755307 
755307 
755307 
 755307 

1
1450140
genus
1

 1073996 

1073996 
1
1
species

species
111
311
 756883 

756883 


756883 
756883 

139912137
order
571515175647
2235

491213104045
1963268
867786
family

11111
genus
64146
146825

616

146826 

146826 


146826 
 146826 
111
species

11
species
 430914 


430914 


430914 
44

10122412
63743
genus
212211

411
 416273 

416273 

416273 
416273 
species
111

6111412
 2257 

2257 
2257 
2257 
2257 
2257 
2257 
111111
species


1679096 

1679096 
1679096 
1679096 
1679096 
 1679096 
923322
species
11111

203135
8232112
genus
111111

111111
species
8232112

57705 
57705 
57705 
57705 
57705 
57705 
 57705 

111111
genus
924131
1073987

species
111111
 1932360 

1932360 
1932360 
1932360 
1932360 
1932360 
1932360 
924131

genus
221231
2237
7312152

2112

1932004 
1932004 

1932004 

1932004 
 1932004 
1111
species

species
11
 1592728 


1592728 

1592728 
13

11
species
 2238 


2238 
2238 
12

species
111
5210
 51589 

51589 
51589 


51589 

8327162
2236
family
532551

2239
4314132
331231
genus

 751944 

751944 
751944 

751944 
751944 
751944 
112102
species
11111

species
1111

2242 
2242 

2242 
2242 
 2242 
2121


1407499 
1407499 
1407499 

1407499 
 1407499 
1112
1111
species

1656823
312
genus
111

species
111
 1604004 

1604004 


1604004 
1604004 
312

genus
1111
1980514
1111

species
1111

1873524 

1873524 
1873524 
1873524 
 1873524 
1111

genus
1
332246
1

 413810 


413810 
1
1
species

1
phylum
1
1801631

1
 1920749 


1920749 
species
1

651137
446143
phylum
22412

1111
genus
2322
1825023

2322

1846278 
1846278 
1846278 

1846278 
 1846278 
1111
species

31932
212141
11211
order

family
11211
338190
212141

1111
genus
1993653
11141

11141
 1898749 


1898749 
1898749 
1898749 
1898749 
1111
species

338191
21
genus
11

11
species
 1229909 

1229909 

1229909 
21

2
1643678
class
1

order
1
2
1033996

2
1033997
1
family

genus
1
497726
2

species
1
 1034015 


1034015 
2

phylum
325243
28889
293825458

class
325243
183924
293825458

order
111112
231512217
114380

family
111111
2307
231512216

genus
111111
54251
231512216

231512216
 54252 

54252 
54252 
54252 
54252 
54252 
54252 
111111
species

1
2272
family
1

genus
1
1
477695

 1200300 


1200300 
1
species
1

213121
order
2281
62311231

213121
family
62311231
118883

12914
1
genus
1

 312539 

312539 
1
species
1

111
69655
genus
111

111
species


111955 

111955 
111955 
 111955 
111

11211
genus
2284
5231022

species
11111
523922
 2285 

2285 
2285 
2285 
2285 
2285 

1
species
 43080 


43080 
1

2266
21
11
order

114378
1
1
family

genus
1
1
2268


2269 
 2269 
1
1
species

2
2267
1
family

1
genus
2276
2

2


2277 
 2277 
1
species

2
180071418647841447090226179422516132184510
277027312780263626342401
superkingdom

976
297091169613419697115471202
phylum
221185208181131111

212121
class
54151062
1937959

54151062
1936988
order
212121

532
89374
family
111

genus
111
532
1007

 1008 

1008 

1008 

1008 
532
species
111

111111
family
1937961
49121042

2349
49121042
genus
111111

species
111111
49121042
 2350 

2350 
2350 
2350 
2350 
2350 
2350 

867644
class
19613551168
1853228

1853229
19613551168
867644
order

family
867644
563835
19613551168

354354
511111
genus
111111

species
111111
 354356 

354356 
354356 
354356 
354356 
354356 
354356 
511111

528112
649460
111111
genus

111111
species
528112
 477680 

477680 
477680 
477680 
477680 
477680 
477680 

494622
379899
genus
22211

species
1111

1176587 
1176587 
1176587 

1176587 
 1176587 
14232

1111
species
 446683 

446683 
446683 
446683 
446683 
35232

genus
11111
1769012
2643112


1850526 
1850526 
1850526 
1850526 
1850526 
 1850526 
2643112
11111
species

genus
11
398041
312

11
species
312
 1492898 

1492898 


1492898 

genus
21221
79328
802963

1111
species

2029983 
2029983 
2029983 
2029983 
 2029983 
8243

species
1111

79329 

79329 
79329 

79329 
 79329 
72533

117747
1362229458393077928
16141616159
class

1362229458393077928
200666
order
16141616159

16141616159
family
1362229458393077928
84566

84567
4256135344211606
555553
genus

117951876510
 1727164 

1727164 
1727164 
1727164 
1727164 
1727164 
11111
species

species
11111
733751275
 430522 

430522 
430522 
430522 
430522 
430522 

64122632954
 363852 

363852 
363852 
363852 
363852 
363852 
363852 
species
111111

10925310366351

188932 
188932 
188932 
188932 
188932 
188932 
 188932 
111111
species

species
111111
6112402451
 984 

984 
984 
984 
984 
984 
984 

species
111111
2711371255

1986952 
1986952 
1986952 
1986952 
1986952 
1986952 
 1986952 

genus
555553
28453
858068529755913

9311891

743722 
743722 
743722 
743722 
743722 
 743722 
11111
species

111111
species
68322243311422

1538644 
1538644 
1538644 
1538644 
1538644 
1538644 
 1538644 

655363781237

1933220 
1933220 
1933220 
1933220 
1933220 
1933220 
 1933220 
111111
species

331313981
 1010 

1010 
1010 
1010 
1010 
1010 
species
11111

66964311224

2003121 
2003121 
2003121 
2003121 
2003121 
2003121 
 2003121 
species
111111

323321
genus
33288601222
423349

491461
 1550579 

1550579 

1550579 
1550579 
1550579 
species
1111

 1234841 

1234841 
1234841 
1234841 
1234841 

1234841 
126862542
11111
species


1300914 
1300914 
1300914 
1300914 
1300914 
 1300914 
15722121
species
11111

genus
111111
103215822
929509

 995 

995 
995 
995 
995 
995 
995 
103215822
species
111111

1649482
808691
genus
1111

species
1111
808691

151895 

151895 
151895 
151895 
 151895 

10037787291190
1100069
444444
order

563843
10037787291190
family
444444

29548
481714304163
111111
genus

species
111111
481714304163

29549 
29549 
29549 
29549 
29549 
29549 
 29549 

12845211518
 1779382 

1779382 
1779382 
1779382 
1779382 
1779382 
1779382 
111111
species

111111
species
2729101542
 2026787 

2026787 
2026787 
2026787 
2026787 
2026787 
2026787 

131010112067
146918
genus
111111

131010112067
 146919 

146919 
146919 
146919 
146919 
146919 
146919 
111111
species

383135302324
class
200643
657139122020415295

171549
543133119920014892
342731272122
order

511434
3
1
genus

1
species


511435 
 511435 
3

2005519
17654
family
11111

11111
genus
17654
397864

11111
species

397865 
397865 

397865 
397865 
397865 
 397865 
17654

family
221111
11471441
171550

genus
11
1611681
22

11
species
22
 1433126 

1433126 
1433126 

genus
111111
9271441
239759

species
111111
 214856 

214856 
214856 
214856 
214856 
214856 
214856 
9271441

15195218112
2005473
family
111111

genus
111111
1918540
15195218112

species
111111
 1796646 

1796646 
1796646 
1796646 
1796646 
1796646 
1796646 
15195218112

family
424343
10434046824219
171551

11111
genus
1784836
22132

11111
species

1562970 

1562970 
1562970 
1562970 
1562970 
 1562970 
22132

6930739773416
836
212121
genus


837 
837 
837 
837 
837 
837 
 837 
6830738773316
111111
species


393921 

393921 
 393921 
11
species
11

1
 28123 


28123 
1
species

111111
genus
307628
33335451

111111
species
33335451
 1642646 

1642646 
1642646 
1642646 
1642646 
1642646 
1642646 

40128392
2005525
434312
family

195950
10115292
222212
genus

species
111111
5104191

28112 
28112 
28112 
28112 
28112 
28112 
 28112 

51111

712710 
712710 
712710 
712710 

712710 
 712710 
11111
species

375288
30131
2121
genus

2621

2025876 

2025876 
2025876 
 2025876 
111
species


823 
823 
823 
 823 
411
species
111

426225476
1853231
111111
family

111111
genus
283168
426225476

111111
species
 28118 

28118 
28118 
28118 
28118 
28118 
28118 
426225476

1116728142916
171552
877566
family

1116728142916
838
877566
genus

11
species
21
 1177574 

1177574 

1177574 

111111
species

28131 
28131 
28131 
28131 
28131 
28131 
 28131 
444612957

species
11111
 52227 

52227 
52227 
52227 

52227 
52227 
1331122

42
 589436 


589436 


589436 
11
species

531211
 76123 

76123 
76123 
76123 
76123 
76123 
76123 
111111
species

1111
species
19331
 28129 

28129 
28129 
28129 


28129 

1548123
 28132 

28132 
28132 
28132 
28132 
28132 
28132 
111111
species

species
1111
 652716 

652716 
652716 

652716 
652716 
1411

1111
species
 839 

839 

839 
839 
839 
12218

2005523
29326
1111
family

1111
genus
346096
29326

1111
species

185300 
185300 
185300 
185300 
 185300 
29326

17963271474041
815
10711956
family

816
17963271474041
10711956
genus

217212

821 

821 
821 
821 
821 
 821 
11111
species

species
111
321
 28113 


28113 
28113 

28113 

1111
species
7122

818 
818 
818 
818 
 818 

1111
species
 376805 

376805 
376805 
376805 

376805 
102922


357276 

357276 
357276 

357276 
 357276 
1111
1111
species

9957138211831

817 
817 
817 
817 
817 
817 
 817 
species
111111

5114
 47678 

47678 

47678 
47678 

47678 
species
1111

111
species

246787 


246787 
246787 
 246787 
1755

1111
species

1796613 
1796613 
1796613 


1796613 
 1796613 
151522

921214
 28119 


28119 
28119 
28119 
28119 
species
1111

species
1111

28116 
28116 
28116 
28116 
 28116 
25121


290053 
290053 
290053 
 290053 
221
111
species

16411

1400053 
1400053 

1400053 
1400053 
1400053 
 1400053 
11111
species

2005520
10102
111
family

genus
111
10102
294702

species
111
10102
 1642647 

1642647 

1642647 
1642647 

order
444322
1970189
1146021443

family
1111
295022
558415

1193324
295022
genus
1111

1111
species

889453 
889453 
889453 
889453 
 889453 
295022

11111
family
1970190
33311

33311
1970191
11111
genus

33311

1307839 
1307839 
1307839 
1307839 

1307839 
 1307839 
11111
species

1573805
55611132
family
111111

55611132
 1717717 

1717717 
1717717 
1717717 
1717717 
1717717 
1717717 
species
111111

27151
1471398
family
1111

27151
1471399
1111
genus

 1168034 

1168034 
1168034 
1168034 

1168034 
27151
1111
species

14102969864926155591502
117743
11597106945641
class

order
11597106945641
14102969864926155591502
200644

39782
82118301059
563334
family

82118301059
34098
genus
563334

111111
species
 164514 

164514 
164514 
164514 
164514 
164514 
164514 
625320636

11
species
 624186 

624186 

624186 
84

 1316444 


1316444 

1316444 

1316444 
831
111
species

species
11111

1653831 
1653831 
1653831 

1653831 
1653831 
 1653831 
850611

211

367806 
367806 


367806 
 367806 
111
species

2
 39768 

39768 
1
species

11
species
 1186051 


1186051 


1186051 
31


298656 

298656 
 298656 
31
11
species

111111
genus
16715880281615
336809

111111
species
 336810 

336810 
336810 
336810 
336810 
336810 
336810 
16715880281615

8621
246874
1111
family

267986
8621
genus
1111

 253245 

253245 

253245 
253245 
253245 
8621
species
1111

1940138
1103
genus
111

1103
 1415657 

1415657 
1415657 
1415657 
species
111

1058899885136
family
13821941063626114569478
49546

genus
444311
8012311911
104267

species
11111
 669041 

669041 
669041 
669041 
669041 
669041 
231551

species
1111

1850252 
1850252 
1850252 
1850252 
 1850252 
21196

 584609 

584609 
584609 
584609 
584609 

584609 
2381181
species
11111

111
species
 107401 

107401 
107401 
107401 
1326

12912765
genus
172346730129
1016

24391
 28189 

28189 
28189 

28189 
28189 
1111
species

111111
species

1945658 
1945658 
1945658 
1945658 
1945658 
1945658 
 1945658 
59131221

 1945657 

1945657 

1945657 

1945657 
28101
111
species

1111
species

1316596 

1316596 
1316596 

1316596 
 1316596 
7213

11
species
12
 1705617 


1705617 


1705617 

1151121
 1019 

1019 
1019 
1019 
1019 
1019 
species
11111

82

209053 
209053 
 209053 
11
species

84

1316593 

1316593 
 1316593 
species
11

512412
 45243 

45243 
45243 
45243 
45243 
45243 
45243 
111111
species

11
species
 327575 

327575 

327575 
13

111
species
 1018 

1018 
1018 
1018 
431

 28188 

28188 
28188 
28188 
28188 
28188 
28188 
68112161
species
111111

37


1848904 
1848904 
 1848904 
11
species

3711
 1017 

1017 
1017 
1017 
1017 
species
1111

genus
11111
389486
52261

species
11111
52261
 1453352 

1453352 
1453352 
1453352 
1453352 
1453352 

genus
11111
1649495
111841

11111
species
111841

1936081 
1936081 

1936081 
1936081 
1936081 
 1936081 

232549922722249319303
28250
genus
111111

232549922722249319303

28251 
28251 
28251 
28251 
28251 
28251 
 28251 
111111
species

112040
171511
11111
genus

species
11111
171511

63186 
63186 
63186 
63186 
63186 
 63186 

1176327
177
11
genus

species
11
177

2094025 


2094025 
 2094025 

252306
39321
1111
genus


252307 

252307 

252307 
252307 
 252307 
39321
species
1111

genus
111
412
1247519

species
111
 1383885 

1383885 

1383885 
1383885 
412

424322
genus
1066171052
292691


1913577 

1913577 
1913577 

1913577 
 1913577 
15151
species
1111

5831112
 411153 

411153 
411153 
411153 
411153 
411153 
11111
species

11111
species
173341

2126553 
2126553 
2126553 
2126553 

2126553 
 2126553 

1623

1486245 

1486245 

1486245 
 1486245 
111
species

genus
33312
291183
78231313

111
species
331

983544 
983544 
983544 
 983544 

95712

2057808 
2057808 
2057808 
2057808 
2057808 
 2057808 
11111
species

species
1111
 1486034 

1486034 
1486034 
1486034 

1486034 
661551

genus
11
1415
178469

11
species

616991 

616991 
 616991 
1415

38172013155639865812
59732
genus
999994

11111
species
 558152 

558152 
558152 
558152 
558152 
558152 
2171321353052

species
11111

878220 
878220 
878220 
878220 
878220 
 878220 
2641551153172

860553471121621

253 
253 
253 
253 
253 
 253 
species
11111

42327814745561

1721091 
1721091 
1721091 
1721091 
1721091 
1721091 
 1721091 
species
111111


1324352 
1324352 
1324352 
1324352 
1324352 
 1324352 
173131772324
species
11111

6282144502

536441 
536441 
536441 
536441 
536441 
 536441 
11111
species

43318122538361

2015076 
2015076 
2015076 
2015076 
2015076 
2015076 
 2015076 
111111
species

species
111111
 1685010 

1685010 
1685010 
1685010 
1685010 
1685010 
1685010 
601484270945149

21878728311
 1265445 

1265445 
1265445 
1265445 
1265445 
1265445 
1265445 
111111
species

9378213061774945
237
15151515108
genus

 1492737 

1492737 
1492737 
1492737 
1492737 
1492737 
1492737 
291611711
111111
species

111111
species

2183896 
2183896 
2183896 
2183896 
2183896 
2183896 
 2183896 
3514541871

18673

312277 
312277 
312277 
312277 
 312277 
species
1111

1111
species
194561
 2201181 

2201181 
2201181 
2201181 
2201181 

111111
species
 986 

986 
986 
986 
986 
986 
986 
1293787623136

111111
species
20194381787
 996 

996 
996 
996 
996 
996 
996 

 96345 

96345 
96345 
96345 
96345 
96345 
96345 
261123456483
111111
species


1981981 
1981981 
1981981 
1981981 
 1981981 
6310154
species
1111

 1763534 

1763534 
1763534 
1763534 
1763534 
1763534 
21617102
11111
species

251314421
 1355330 

1355330 
1355330 
1355330 
1355330 
1355330 
1355330 
111111
species

111111
species
20327424

2162713 
2162713 
2162713 
2162713 
2162713 
2162713 
 2162713 

41486
 55197 

55197 
55197 
55197 
55197 
1111
species

species
1111

2175091 
2175091 
2175091 
2175091 
 2175091 
15131

111111
species
431771032
 1306519 

1306519 
1306519 
1306519 
1306519 
1306519 
1306519 

 2172098 

2172098 
2172098 
2172098 
2172098 
2172098 
171321
11111
species

2287
1209327
111
genus

species
111
2287

1803846 

1803846 
1803846 
 1803846 

genus
1221
22561
286104

11
species
 1936080 


1936080 
1936080 
35

22211
 754409 

754409 
754409 
754409 
754409 
1111
species

11111
genus
417127
457964

457964
 398743 

398743 
398743 
398743 
398743 

398743 
11111
species

species
1111

1150389 
1150389 
1150389 
1150389 
 1150389 
19336

1204360
16243
genus
1111


762954 
762954 
762954 
762954 
 762954 
16243
species
1111

6816591411
76831
434411
genus

species
111
1536

480520 

480520 
480520 
 480520 

species
111111
 1458492 

1458492 
1458492 
1458492 
1458492 
1458492 
1458492 
9914611

 1583100 

1583100 
1583100 
1583100 
1583100 
115121
species
1111

 76832 

76832 
76832 
76832 
76832 
332301
1111
species

 531844 

531844 
531844 
531844 
531844 
531844 
531844 
112417213332
111111
species

143222
813143
genus
11111

813143
 1729720 

1729720 
1729720 
1729720 
1729720 

1729720 
11111
species

574522
363408
4324
genus

 328515 

328515 
328515 

328515 
2328
species
111

1111
species
15112

1476901 
1476901 
1476901 
1476901 
 1476901 

1111
species
18144

2058134 
2058134 
2058134 
2058134 
 2058134 

species
11

319236 


319236 
 319236 
18

52959
6021212962
genus
555522

species
11111
767164

996801 
996801 
996801 
996801 
996801 
 996801 

1111
species

1529069 
1529069 
1529069 
1529069 
 1529069 
12414

species
11111
188611

313598 
313598 
313598 
313598 

313598 
 313598 

species
1111
5221
 1774273 

1774273 
1774273 
1774273 
1774273 

111111
species
1815721
 2058137 

2058137 
2058137 
2058137 
2058137 
2058137 
2058137 

252356
7461281
44421
genus

 1644130 

1644130 
1644130 
1644130 
1644130 
1644130 
452571
11111
species

 1178778 

1178778 
1178778 
1178778 
2212
111
species

 1836467 

1836467 
1836467 
1836467 
111
species
111


313603 
313603 
313603 
313603 
 313603 
6241
1111
species

111
genus
2155
261827

species
111

1736674 
1736674 
1736674 
 1736674 
2155

111
genus
323
216431

species
111
 313588 

313588 
313588 
313588 
323

genus
555531
308865
29955567151082326

species
1111

1756149 
1756149 
1756149 
1756149 
 1756149 
2271116022

11111
species
 238 

238 
238 
238 
238 
238 
32665931406

4713829

1756150 
1756150 
1756150 
1756150 
 1756150 
1111
species

11111
species
 172045 

172045 
172045 
172045 
172045 
172045 
1933164541


1117645 
1117645 
1117645 
1117645 
1117645 
1117645 
 1117645 
2202436390837256
species
111111

153265
2342
genus
111


101385 
101385 

101385 
 101385 
2342
species
111

1352
225842
221
genus

11
 1336795 

1336795 

1336795 
11
species

1242
 1336794 

1336794 

1336794 
1336794 
species
111

genus
11111
83612
182121

182121
 57029 

57029 
57029 
57029 
57029 
57029 
11111
species

63259045148
1013
111111
genus

63259045148

1014 
1014 
1014 
1014 
1014 
1014 
 1014 
species
111111

9015303128
104264
genus
333321

32339

59600 
59600 
59600 
59600 
 59600 
1111
species

11816121

979 
979 
979 
979 
979 
 979 
species
11111

species
111111
474111018

76594 
76594 
76594 
76594 
76594 
76594 
 76594 

genus
1111
111500
11431

1143
 111501 

111501 
111501 

111501 
species
111


516051 
 516051 
1
species
1

genus
1111
336276
111284


2058135 
2058135 
2058135 
2058135 
 2058135 
111284
species
1111

111111
genus
12677565452855067
34084


34085 
34085 
34085 
34085 
34085 
34085 
 34085 
12677565452855067
111111
species

genus
222211
272928811
358023

species
1111
 1850246 

1850246 
1850246 
1850246 
1850246 
164175

 1622118 

1622118 
1622118 
1622118 
1622118 
1622118 
1622118 
112511311
species
111111

genus
43312
4816522
326319

391

313590 

313590 


313590 
 313590 
111
species

species
1111
 983548 

983548 

983548 
983548 

983548 
10411

3033
 2173169 

2173169 

2173169 
2173169 
species
111

species
111
512

326320 


326320 
326320 
 326320 

71964
1518147
1111
genus

1111
species
71964

1790137 
1790137 
1790137 
1790137 
 1790137 

genus
1111
234212
393005

1111
species
234212

2069432 
2069432 
2069432 
2069432 
 2069432 

1853230
18211
111
family

18211
332102
111
genus

18211

191579 
191579 
191579 
 191579 
111
species

family
11
1755828
51

genus
11
1755829
51

51
 242600 

242600 


242600 
species
11

978262730212622377
768503
class
383238302728

768507
978262730212622377
order
383238302728

1210129109
family
3353774456524
89373

111111
genus
1664383
15631781

15631781
 2183547 

2183547 
2183547 
2183547 
2183547 
2183547 
2183547 
111111
species

8511112
978
genus
11111

 985 

985 
985 
985 

985 
985 
8511112
11111
species

111111
genus
120831
19211312


94254 
94254 
94254 
94254 
94254 
94254 
 94254 
19211312
species
111111

9112
105
genus
1111

species
1111
 106 

106 
106 
106 

106 
9112

5151
2173039
genus
111

5151

1784714 

1784714 


1784714 
 1784714 
species
111

111111
genus
611265
861914

species
111111

1834519 
1834519 
1834519 
1834519 
1834519 
1834519 
 1834519 
611265

genus
555554
107
1302625184713

391224

1211326 
1211326 
1211326 
1211326 
1211326 
 1211326 
species
11111


2057025 
2057025 
2057025 
2057025 
2057025 
2057025 
 2057025 
247101264
111111
species

2845665

1379870 
1379870 
1379870 
1379870 
1379870 
1379870 
 1379870 
species
111111


564064 
564064 
564064 
564064 
564064 
564064 
 564064 
14133752
111111
species


1178516 
1178516 
1178516 
1178516 
1178516 
1178516 
 1178516 
2515262
species
111111

20175
319458
111
genus

111
species
 316068 

316068 

316068 
316068 
20175

1853232
512199542142546342
family
131113121212

genus
323332
39924201411
1379908


1379910 
1379910 
1379910 
1379910 
1379910 
1379910 
 1379910 
537753
species
111111

species
1111
29944
 512763 

512763 

512763 
512763 
512763 

111111
species

1379909 
1379909 
1379909 
1379909 
1379909 
1379909 
 1379909 
568958

genus
212112
3319885
323449

28484
 400092 

400092 

400092 
400092 

400092 
species
1111


388950 
388950 
388950 

388950 
388950 
 388950 
51581
species
11111

genus
888888
89966
440189509114524326

111111
species
4393285819
 1446467 

1446467 
1446467 
1446467 
1446467 
1446467 
1446467 

111111
species
505564108553

1484118 
1484118 
1484118 
1484118 
1484118 
1484118 
 1484118 

111111
species
 1356852 

1356852 
1356852 
1356852 
1356852 
1356852 
1356852 
482162159628


1385664 
1385664 
1385664 
1385664 
1385664 
1385664 
 1385664 
29253465235
species
111111

681369136042
 1850093 

1850093 
1850093 
1850093 
1850093 
1850093 
1850093 
111111
species

 1484116 

1484116 
1484116 
1484116 
1484116 
1484116 
1484116 
1274513440101101
111111
species

 1411621 

1411621 
1411621 
1411621 
1411621 
1411621 
1411621 
581180124738
111111
species

171034102510

1385663 
1385663 
1385663 
1385663 
1385663 
1385663 
 1385663 
111111
species

563798
6717651254
655322
family

13245
246875
1111
genus

species
1111
 1727163 

1727163 
1727163 
1727163 
1727163 
13245

221219622
390846
genus
222111

111
species

1795355 
1795355 
1795355 
 1795355 
2084

2415622

390884 
390884 
390884 
390884 
390884 
390884 
 390884 
111111
species

232244
1014012
genus
11111

species
11111

232259 
232259 
232259 
232259 

232259 
 232259 
1014012

22223
68288
2111
genus


104 
104 
104 
 104 
1622
111
species

species
11
63

320787 


320787 
 320787 

201333
1853234
11111
family

201333
59740
11111
genus

11111
species

1085624 
1085624 
1085624 
1085624 

1085624 
 1085624 
201333

1501348
18521322
family
223212

273135
1852031
22221
genus

11111
species
1311221

247481 
247481 
247481 
247481 

247481 
 247481 

species
1111
5481
 249402 

249402 
249402 
249402 
249402 

genus
111
121
281119

111
species


281120 

281120 
281120 
 281120 
121

1152
1937968
1111
family

1937972
1152
genus
1111

 999 

999 
999 
999 
999 
1152
species
1111

family
323222
25220542
200667

812321
 1257021 

1257021 
1257021 
1257021 
1257021 
1257021 
1257021 
species
111111

genus
11111
11921
59739

11921
 1191459 

1191459 
1191459 
1191459 

1191459 
1191459 
11111
species

1692
869806
genus
111

1692
 1006 

1006 

1006 
1006 
species
111

361953505126
40117
576774
phylum

203693
361953505126
576774
class

361953505126
189778
576774
order

361953505126
189779
family
576774

28261
1
1
genus

species
1


28262 
 28262 
1

31750233926
1234
344444
genus


42253 
42253 
42253 
42253 
42253 
42253 
 42253 
413810813
species
111111

37319


1325564 
1325564 
1325564 
1325564 
1325564 
 1325564 
11111
species

2513473
 1715989 

1715989 
1715989 
1715989 
1715989 
1715989 
1715989 
111111
species

2226231

330214 
330214 
330214 
330214 
330214 
330214 
 330214 
111111
species

179
51132712
genus
22233

261385
131157
species group
11111


1660083 
1660083 
1660083 
1660083 
1660083 
 1660083 
131157
11111
species

species group
11111
48284
261386


178606 
178606 
178606 
178606 
178606 
 178606 
48284
11111
species

species group
11
41
655606

11
species
 180 


180 
180 
41

11111
phylum
1930617
41211

1962850
41211
11111
class

order
11111
41211
1962852

41211
1962854
11111
family

187144
41211
11111
genus

41211
 187145 

187145 
187145 
187145 

187145 
187145 
species
11111

95818
11312
phylum
1112

species
111
111

2056494 

2056494 
2056494 
 2056494 

131


1476577 

1476577 
 1476577 
species
11

phylum
534947282634
544448
145318531429430277292

2
 1911684 

1911684 
1
species

31969
145118531429429277292
524947272634
class

order
302829181722
2085
120314981281375239259

family
302829181722
2092
120314981281375239259

genus
212112
2129
868113

11111
species
34112
 134821 

134821 

134821 
134821 
134821 
134821 

1111
species

2130 
2130 
2130 


2130 
 2130 
5641

2093
119514921273374238256
282727171620
genus

species
11
16


29553 
29553 
 29553 

species
1
1


136241 
 136241 

13
 51365 

51365 
51365 
11
species

 65123 


65123 
1
species
1

species
111
122

2113 

2113 


2113 
 2113 


2123 


2123 
 2123 
22
11
species

111
species

2109 
2109 
2109 
 2109 
222

1111
species
 57372 

57372 

57372 
57372 

57372 
1531

111111
species
92614622

45362 
45362 
45362 
45362 
45362 
45362 
 45362 

species
111111

2107 
2107 
2107 
2107 
2107 
2107 
 2107 
150154119151640

species
111111

171632 
171632 
171632 
171632 
171632 
171632 
 171632 
33165021134

213441
 29501 

29501 

29501 
29501 
29501 
29501 
11111
species

181637182421

2096 
2096 
2096 
2096 
2096 
2096 
 2096 
species
111111

333223
species group
3132104106
656088

11111
species
 2105 

2105 
2105 
2105 
2105 

2105 
44231

species
111111
 2102 

2102 
2102 
2102 
2102 
2102 
2102 
17195144

 2095 

2095 
2095 
2095 

2095 
2095 
109361
species
11111

121
 86660 

86660 
86660 


86660 
species
111

species
1
1
 2094 

2094 

1
species
2


50052 
 50052 

111
species
 28227 

28227 
28227 
28227 
3161

111111
species
106165836811

2100 
2100 
2100 
2100 
2100 
2100 
 2100 

111
species
 29555 

29555 
29555 
29555 
214

11111
species

2098 
2098 
2098 

2098 
2098 
 2098 
21421

433

171284 
171284 
171284 
 171284 
species
111


2112 
2112 
2112 
 2112 
422
111
species

455508310326685

2099 
2099 
2099 
2099 
2099 
2099 
 2099 
species
111111


2115 
2115 
 2115 
11
11
species

species
111111

28903 
28903 
28903 
28903 
28903 
28903 
 28903 
5687139431111

species
111111
 171279 

171279 
171279 
171279 
171279 
171279 
171279 
221293159162844

species
111111
 92400 

92400 
92400 
92400 
92400 
92400 
92400 
2855331039

56822621421714
 2128 

2128 
2128 
2128 
2128 
2128 
2128 
111111
species

421952321

2104 
2104 
2104 
2104 
2104 
2104 
 2104 
111111
species

11

48003 


48003 
 48003 
11
species

3232

2097 
2097 
2097 


2097 
 2097 
1111
species

192273117192625
186328
161714557
order

2131
188269102142424
family
131410436

genus
131410436
2132
188269102142424

species
1111
1816102

2136 
2136 
2136 


2136 
 2136 


216935 
216935 
 216935 
33
species
11

131
 2144 

2144 
2144 


2144 
111
species


216933 
 216933 
1
species
1


47834 
47834 
47834 
 47834 
6122
111
species


216945 

216945 
 216945 
12
species
11

 1914410 


1914410 
1
1
species

11
 315358 


315358 
315358 
species
11


1898044 
1898044 


1898044 
 1898044 
111
species
111

111
species

2145 
2145 
2145 
 2145 
162


216944 
 216944 
1
species
1

11111
species
510522

362837 
362837 
362837 

362837 
362837 
 362837 

11
species
23
 2133 

2133 
2133 


2139 
 2139 
1
1
species

1
species
 216934 

216934 
1

1


2137 
 2137 
species
1

231
 216937 

216937 
216937 
216937 
111
species

species
111111
425821293
 216936 

216936 
216936 
216936 
216936 
216936 
216936 

1051515891315
 216946 

216946 
216946 
216946 
216946 
216946 
216946 
species
111111

334121
family
4415521
33925

1211
46238
genus
1211

111
species
111
 215578 


215578 
215578 

215578 


214888 
214888 
 214888 
11
species
11

213111
genus
3214511
46239

1
species
1


216427 
 216427 

 138853 

138853 
1
species
1

1
species
 219745 


219745 
1

 324078 


324078 
2
species
1

species
11111

2151 
2151 
2151 
2151 

2151 
 2151 
221151

644445
order
56823135128
186329

644445
family
56823135128
2146

genus
321322
2147
3933162

1111
species
12321

2148 
2148 
2148 
2148 
 2148 

1921

35623 


35623 
35623 
35623 
 35623 
species
1111

species
11111
17141

61635 
61635 

61635 
61635 
61635 
 61635 

genus
323123
33926
537328466

1111
species group
6511
85630

species
1111
 37692 

37692 
37692 
37692 


37692 
6511

species group
1
1
85632

1
 59748 


59748 
1
species

212112
species group
476827455
85620

22
 202462 

202462 

202462 
11
species

456825454

229545 
229545 
229545 
229545 
229545 
229545 
 229545 
species
111111

species
1
 100379 


100379 
1

1
1912503
1
genus

1
species
1


1541959 
 1541959 

206351314
65842
111111
phylum

206351314
204430
111111
class

111111
order
218872
206351314

111111
family
206351314
204431

832
206351314
111111
genus

species
111111
206351314

833 
833 
833 
833 
833 
833 
 833 

34373
200938
phylum
11111

34373
118001
class
11111

order
11111
34373
189769

11111
family
189770
34373

11111
genus
34373
393029

 936456 

936456 
936456 
936456 
936456 

936456 
34373
11111
species

7887109
phylum
1090
573125183775

573125183775
191410
7887109
class

7887109
order
191411
573125183775

family
7887109
191412
573125183775

genus
323133
256319
3812921645

254532
 1097 

1097 

1097 

1097 
1097 
species
1111

111111
species
684213
 274537 

274537 
274537 
274537 
274537 
274537 
274537 

11111
species
7411010
 274539 

274539 
274539 
274539 

274539 
274539 

genus
122231
1258611
1101

 281093 


281093 
281093 
281093 
281093 
281093 
117211
species
11111

species
1111


1868325 
1868325 
1868325 
1868325 
 1868325 
1412

11
species

1974213 


1974213 
 1974213 
12

100715
544332
genus
111111

 100716 

100716 
100716 
100716 
100716 
100716 
100716 
544332
species
111111

121122
genus
3331512
1099

species
111111
3231411
 1100 

1100 
1100 
1100 
1100 
1100 
1100 

111


34090 


34090 
34090 
 34090 
species
111

10104475
1091
111212
genus

1
 1092 


1092 
species
1

111111
species
10104374
 1096 

1096 
1096 
1096 
1096 
1096 
1096 


1094 
 1094 
1
species
1

phylum
333333
703634257147
142182

703634257147
219685
333333
class

order
333333
703634257147
219686

703634257147
219687
333333
family

1706036
301314162716
111111
genus

species
111111
301314162716
 861299 

861299 
861299 
861299 
861299 
861299 
861299 

genus
222222
173479
40232094431

species
111111
26191873419

173480 
173480 
173480 
173480 
173480 
173480 
 173480 

144221012
 1379270 

1379270 
1379270 
1379270 
1379270 
1379270 
1379270 
species
111111

383288618553379353
1117
676562507067
phylum

1890424
759312772113107
262825202523
order

3
1890429
1
family

155977
3
1
genus

1
species
3
 155978 


155978 

1111
family
1890436
3227

genus
1111
1152
3227

3227
 82654 


82654 
82654 

82654 
82654 
1111
species

1890426
476677557482
family
182018151815

1129
245867335747
141715121411
genus

1111
species

32049 
32049 


32049 
32049 
 32049 
2141

 166314 


166314 
2
species
1


195498 

195498 
 195498 
21
11
species

11113

32051 
32051 
32051 
32051 

32051 
 32051 
11111
species

species
1111
 84588 

84588 
84588 

84588 
84588 
62116


1350461 
1350461 
1350461 
 1350461 
122
species
111

11123

195253 
195253 
195253 

195253 
195253 
 195253 
species
11111

111
species
 316278 


316278 

316278 
316278 
211

21
 374981 


374981 


374981 
species
11

111
 316279 

316279 
316279 


316279 
species
111

species
11111

1916956 
1916956 
1916956 
1916956 

1916956 
 1916956 
11311

species
1111
29112


585423 
585423 
585423 
585423 
 585423 

1111
species
 110662 

110662 
110662 

110662 
110662 
1111


321332 


321332 
321332 
321332 
 321332 
1133
species
1111

 33070 


33070 
6
species
1

species
11111
 585425 

585425 
585425 
585425 
585425 
585425 
11276

species
11111
1136216
 321327 


321327 
321327 
321327 
321327 
321327 

11111
species

29410 
29410 
29410 

29410 
29410 
 29410 
31248

 1173263 


1173263 
1173263 
1173263 
211
111
species

111111
species

32046 
32046 
32046 
32046 
32046 
32046 
 32046 
23129109

1
species


64471 
 64471 
3

1111
species
 374982 

374982 
374982 
374982 


374982 
1251

111
species
1319
 1280380 

1280380 
1280380 
1280380 

 1827144 


1827144 
3
1
species

genus
222222
167375
1377181330

111111
species

59930 
59930 
59930 
59930 
59930 
59930 
 59930 
11461055

111111
species

1851505 
1851505 
1851505 
1851505 
1851505 
1851505 
 1851505 
2318825

111
genus
212
146785

species
111
 146786 

146786 


146786 
146786 
212

813433
13034
111111
genus

 292566 

292566 
292566 
292566 
292566 
292566 
292566 
813433
species
111111

12518128
1890428
111112
family

genus
111112
12518128
1142

species
111111
12518126

1148 
1148 
1148 
1148 
1148 
1148 
 1148 

2
 1147 


1147 
species
1

1082214308
1890438
444334
family

genus
333333
871914307
47251

111111
species
22101091
 1080068 

1080068 
1080068 
1080068 
1080068 
1080068 
1080068 

species
111111
348284
 111781 

111781 
111781 
111781 
111781 
111781 
111781 

species
111111
3112132
 1184 

1184 
1184 
1184 
1184 
1184 
1184 

2131
170610
1111
genus

species
1111
 1209493 

1209493 
1209493 
1209493 


1209493 
2131

family
321111
6118222
1213

genus
321111
1218
6118222

11
 1501269 

1501269 
1501269 
11
species


1219 
1219 
1219 
1219 
1219 
1219 
 1219 
3108222
111111
species

species
1
2

1501268 
 1501268 

1890505
212
111
order

212
1890528
family
111

212
54298
111
genus

212
 54299 


54299 
54299 
54299 
111
species

52604
5103231
122211
order

family
111
111
1890500

111
genus
44474
111

species
111
111


54308 

54308 

54308 
 54308 

59313
1890498
family
11211

102115
59313
11211
genus

11
species


102116 
102116 
 102116 
21

species
1111

1807358 
1807358 
1807358 

1807358 
 1807358 
5913

181416111815
subclass
190123129201144133
1301283

1191091110
order
1341009716013196
1150

family
432333
1892249
125528147

43988
125528147
genus
432333


43989 


43989 
 43989 
68
11
species

 41431 

41431 


41431 
41431 
424
111
species

111111
species
 65393 

65393 
65393 
65393 
65393 
65393 
65393 
114312

31


497965 


497965 
 497965 
11
species

1111711

395961 
395961 
395961 
395961 
395961 
 395961 
11111
species

1892254
10569791209377
323333
family

212222
genus
9566761178172
1158


118323 

118323 
118323 
118323 
118323 
 118323 
41119
11111
species

species
111111

482564 
482564 
482564 
482564 
482564 
482564 
 482564 
9166751168063

1155738
10333125
111111
genus

species
111111
10333125
 1155739 

1155739 
1155739 
1155739 
1155739 
1155739 
1155739 

122265188
1892252
family
333232

genus
111111
35823
41934123

 2153484 

2153484 
2153484 
2153484 
2153484 
2153484 
2153484 
41934123
111111
species

1205
32112
11111
genus

32112

1206 
1206 
1206 
1206 
1206 
 1206 
species
11111

44471
51245
11111
genus

51245
 1173027 

1173027 
1173027 
1173027 

1173027 
1173027 
11111
species

1892255
44732
family
11111

44732
241421
11111
genus

44732
 241425 


241425 
241425 
241425 
241425 
241425 
species
11111

family
1111
5332
1892251

1111
genus
5332
63132

1111
species

1173025 

1173025 

1173025 
1173025 
 1173025 
5332

order
756275
562332411337
1118

family
21342
1614411
1890464

102231
11
genus
11

species
11


1173026 

1173026 
 1173026 
11

genus
1121
669357
8126

species
11
11
 1615909 


1615909 

1615909 

816

1617448 


1617448 
1617448 
 1617448 
species
111

268175
81215
genus
11111

81215

2005460 
2005460 
2005460 

2005460 
2005460 
 2005460 
species
11111

41283041
1890450
family
212121

1453359
2111
1111
genus

1111
species

713887 

713887 

713887 
713887 
 713887 
2111

2127303
76023
genus
11111

species
11111
2127303

65093 
65093 
65093 
65093 
65093 
 65093 

31
1890452
family
11

11
genus
102234
31


379064 
379064 
 379064 
31
11
species

3392011525
1890449
family
221112

221112
genus
3392011525
1125

111111
species

1126 
1126 
1126 
1126 
1126 
1126 
 1126 
3152011524

241
 1967666 

1967666 
1967666 


1967666 
111
species

class
122222
307596
491826235

122222
order
491826235
307595

491826235
1890422
family
122222

33071
491826235
genus
122222

11111
species


1416614 
1416614 
1416614 
1416614 
1416614 
 1416614 
339134

111111
species
461517101
 33072 

33072 
33072 
33072 
33072 
33072 
33072 

 718217 

718217 
718217 
718217 
718217 
718217 
718217 
2330019864
species
111111

201816132224
order
1161
10750415289101

1111
family
1111
1892259

genus
1111
1111
159191


70799 

70799 

70799 
70799 
 70799 
1111
1111
species

11111
family
1892263
17364

11111
genus
1190
17364

species
11111
 1752063 

1752063 
1752063 
1752063 
1752063 
1752063 
17364

family
654268
1185
231718141416

11111
genus
373984
241052

11111
species
 373994 

373994 
373994 
373994 

373994 
373994 
241052

genus
543257
2113814914
1186

11111
species
83122
 32054 

32054 
32054 
32054 

32054 
32054 


1954171 
1954171 
 1954171 
21
11
species

11
species
 2005462 

2005462 


2005462 
13

123821
 2005469 

2005469 
2005469 
2005469 
2005469 
2005469 
2005469 
species
111111

species
1
1


1337936 
 1337936 

111111
species
564621

2005461 
2005461 
2005461 
2005461 
2005461 
2005461 
 2005461 

1111
species
6215
 99598 

99598 
99598 


99598 
99598 

1162
782518317077
1111991414
family

264688
41133
2122
genus

 264691 


264691 


264691 
264691 
3121
111
species

1112


1164 

1164 
1164 
1164 
 1164 
1111
species

genus
323233
1163
35353107

263122

1165 

1165 
1165 
1165 
1165 
 1165 
species
11111

421231
 1647413 

1647413 
1647413 
1647413 
1647413 
1647413 
1647413 
111111
species

species
11111
51154

46234 
46234 
46234 

46234 
46234 
 46234 

876699
genus
1177
431813274767

species
1111
 1618022 

1618022 
1618022 


1618022 
1618022 
5197

185131427

1306274 
1306274 

1306274 
1306274 
1306274 
 1306274 
species
11111

species
1111
4223
 317936 


317936 

317936 
317936 
317936 

 1751286 

1751286 
1751286 
1751286 
1751286 
1751286 
1751286 
211141
111111
species

111111
species
 272131 

272131 
272131 
272131 
272131 
272131 
272131 
621849

41624
 1869241 

1869241 
1869241 
1869241 

1869241 
1869241 
species
11111

species
11111
 103690 

103690 

103690 
103690 
103690 
103690 
21161

species
1111
2149

1261031 

1261031 

1261031 
1261031 
 1261031 

443226
 28072 

28072 
28072 
28072 
28072 
28072 
28072 
111111
species

11111
species
 1940762 

1940762 
1940762 
1940762 
1940762 

1940762 
41117

2221
phylum
74152
15432

141
641853
class
11

141
641854
order
11

641876
141
family
11

11
genus
141
423604

11
species
141
 423605 

423605 

423605 

class
111
322
447830

322
1783344
111
order

322
1783343
family
111

1408194
322
111
genus

322
 1408281 


1408281 
1408281 

1408281 
species
111

11
 167965 

167965 
167965 
species
11

232211
phylum
200930
1944621

class
232211
68337
1944621

232211
order
191393
1944621

1944621
191394
232211
family

13
2351
genus
11

species
11
13
 2352 


2352 
2352 

2242
117999
genus
1111

species
1111

118000 
118000 

118000 
118000 
 118000 
2242

111
genus
121
53572

111
species


197162 

197162 

197162 
 197162 
121

171
545865
genus
11

171
 477976 

477976 

477976 
11
species

15713568403273
32066
131412846
phylum

15713568403273
203490
class
131412846

15713568403273
203491
order
131412846

3271923
1129771
55522
family

1111
genus
34104
41912

species
1111

34105 
34105 

34105 

34105 
 34105 
41912

genus
11
168808
11

 187101 


187101 


187101 
11
species
11

111
genus
3461
32068

3461

826 
826 

826 
 826 
111
species

genus
334
2568
32067

202

712368 

712368 
 712368 
11
species

species
111

1785996 
1785996 
1785996 
 1785996 
222

 40542 

40542 
40542 
40542 
312
111
species

species
11
32
 712357 


712357 
712357 

family
897644
1256459383270
203492

103604928612
848
786533
genus

1111
species

861 
861 
861 
861 
 861 
11441

1111
species
45203


859 
859 
859 
859 
 859 

82
 849 

849 
849 
species
11

species
11111
 860 

860 
860 
860 
860 

860 
30211025

71
 850 

850 
850 
species
11

 851 

851 
851 
851 
851 
851 
851 
321915422
species
111111

111
species
 856 

856 
856 
856 
111

species
111111
14814115
 1583098 

1583098 
1583098 
1583098 
1583098 
1583098 
1583098 

genus
111111
22410102658
167639

22410102658

167642 
167642 
167642 
167642 
167642 
167642 
 167642 
species
111111

1297
307171261174400331
phylum
222222232322

222222232322
class
307171261174400331
188787

order
141414141414
118964
259130213120325299

183710
254129209118321296
family
131313131313

254129209118321296
1298
131313131313
genus


1182568 
1182568 
1182568 
1182568 
1182568 
1182568 
 1182568 
121465513
111111
species

10952127
 55148 

55148 
55148 
55148 
55148 
55148 
55148 
species
111111

111111
species
3373864220

1768108 
1768108 
1768108 
1768108 
1768108 
1768108 
 1768108 

75651425

68909 
68909 
68909 
68909 
68909 
68909 
 68909 
species
111111

species
111111
13569365
 309887 

309887 
309887 
309887 
309887 
309887 
309887 

111111
species

2080419 
2080419 
2080419 
2080419 
2080419 
2080419 
 2080419 
221015102117

species
111111
 1299 

1299 
1299 
1299 
1299 
1299 
1299 
402328134047

604649183510
 1182571 

1182571 
1182571 
1182571 
1182571 
1182571 
1182571 
species
111111

111111
species
1025111320
 502394 

502394 
502394 
502394 
502394 
502394 
502394 

species
111111
 2202254 

2202254 
2202254 
2202254 
2202254 
2202254 
2202254 
1348132152

111111
species

310783 
310783 
310783 
310783 
310783 
310783 
 310783 
1349101547

 1309411 

1309411 
1309411 
1309411 
1309411 
1309411 
1309411 
99191167
species
111111


432329 
432329 
432329 
432329 
432329 
432329 
 432329 
233741126
species
111111

514243
332247
111111
family

genus
111111
514243
332248

111111
species
514243

332249 
332249 
332249 
332249 
332249 
332249 
 332249 

888998
order
484148547532
68933

484148547532
188786
family
888998

73245512
186191
genus
111111

species
111111
73245512
 186192 

186192 
186192 
186192 
186192 
186192 
186192 

131175172
208447
111111
genus

111111
species
131175172

187137 
187137 
187137 
187137 
187137 
187137 
 187137 

genus
554654
262411404716
270

54


56957 
56957 
 56957 
species
11

species
11111
11112
 56956 


56956 
56956 
56956 
56956 
56956 

species
11111
 456163 

456163 
456163 

456163 
456163 
456163 
31213

11111
species
 1111069 

1111069 
1111069 
1111069 
1111069 
1111069 
21124

species
111111
1120631367
 274 

274 
274 
274 
274 
274 
274 

species
1111

37636 
37636 
37636 
37636 
 37636 
8133

11
species
21
 271 

271 


271 

236462
65551
genus
112122

1111
species
3111


52022 
52022 

52022 
52022 
 52022 

species
11111

277 

277 
277 
277 
277 
 277 
25451

phylum
111111
52112053
256845

111111
class
52112053
1313211

order
111111
52112053
278082

 2094242 

2094242 
2094242 
2094242 
2094242 
2094242 
2094242 
52112053
111111
species

121611120055098922714135363630831
1239
phylum
397445410402433267

3171271
1737404
541231
class

order
531121
3141131
1737405

1
family
2042895
21

genus
1
1505664
21

species
1
21
 1556 

1556 

1041131
1570339
431121
family

1111
genus
150022
6111

1111
species
6111

1260 
1260 

1260 

1260 
 1260 

1
genus
1
1161127

1
 1852373 


1852373 
1
species

genus
1
162289
1


1912856 
 1912856 
1
1
species

111
165779
genus
111

species
1


33034 
 33034 
1

species
11

1870984 
1870984 
 1870984 
11

111
genus
222
543311

species
111
222
 33033 

33033 
33033 


33033 

111
genus
314
1582879

111
species
314
 1852374 


1852374 

1852374 
1852374 

1211109149
class
909932
11212164479149

order
21121
26354
1843488

21121
family
909930
26354

26354
904
21121
genus

11111
species

905 
905 
905 

905 
905 
 905 
16334

 187327 

187327 


187327 
12
species
11

909929
577829315429
order
665673

577727315029
1843491
family
654663

73221
158846
genus
11111

 158847 

158847 
158847 
158847 
158847 
158847 
73221
11111
species

507425294929
970
543553
genus

4664810

69823 
69823 
69823 
69823 
69823 
69823 
 69823 
111111
species

 712538 

712538 
712538 

712538 
712538 
712538 
1371717
species
11111

111111
species
40641811172

971 
971 
971 
971 
971 
971 
 971 

456
 1884263 

1884263 


1884263 
1884263 
111
species

11121

713030 
713030 
713030 
713030 
713030 
 713030 
species
11111

111
family
124
1843490

365348
124
111
genus

124


365349 
365349 

365349 
 365349 
111
species

order
444355
1843489
533732163216

533732163216
31977
family
444355

111111
genus
181384118
909928

species
111111
181384118

1702287 
1702287 
1702287 
1702287 
1702287 
1702287 
 1702287 

111111
genus
19171311101
906

19171311101

907 
907 
907 
907 
907 
907 
 907 
species
111111

15510112
29465
111111
genus

 29466 

29466 
29466 
29466 
29466 
29466 
29466 
15510112
species
111111

39948
121105
genus
11122

54
 2161821 


2161821 
2161821 
11
species

11111
species
12151

39950 
39950 
39950 

39950 
39950 
 39950 

526524
14251912153
class
433442

526525
14251912153
order
433442

14251912153
128827
433442
family

1729679
1231
genus
1111

species
1111
1231

1702221 

1702221 
1702221 

1702221 
 1702221 

11111
species

1834207 
1834207 
1834207 
1834207 
1834207 
 1834207 
12311

1111
genus
21152
191303

1111
species
21152

1712675 
1712675 

1712675 
1712675 
 1712675 

1647
1012143122
genus
111121

species
1
1


1514105 
 1514105 

1012143112

1648 
1648 
1648 
1648 
1648 
1648 
 1648 
111111
species

111111
class
1676648
725276

725276
1676649
order
111111

111111
family
1676650
725276

1676651
725276
genus
111111

725276
 1555112 

1555112 
1555112 
1555112 
1555112 
1555112 
1555112 
species
111111

16
 243899 


243899 
1
species

2
1930845
1
genus

2
 1871025 


1871025 
1
species

294330312314331193
class
106161091244919522602035266430129
91061

186826
550223243261215716900428647
order
10611510610411785

7747362435421
186828
665665
family

genus
444442
7445252283917
2747

 208596 

208596 
208596 
208596 
208596 
208596 
3216010
species
11111

111111
species

1564681 
1564681 
1564681 
1564681 
1564681 
1564681 
 1564681 
1822571

species
111111

2751 
2751 
2751 
2751 
2751 
2751 
 2751 
572820111916

 147709 

147709 
147709 
147709 
147709 
147709 
13721323
11111
species

genus
22122
1470540
328153

 1903686 

1903686 
1903686 

1903686 
1903686 
1903686 
218142
11111
species

species
1111
1111
 708126 

708126 
708126 


708126 
708126 

191769
1171
genus
111

111
species
1171


1911586 
1911586 

1911586 
 1911586 

587648806226
81850
1010119104
family

485720463823
1243
genus
776762

11
species


255248 
255248 
 255248 
11

species
11111
743235

136609 
136609 
136609 
136609 
136609 
 136609 

species
111111
 1244 

1244 
1244 
1244 
1244 
1244 
1244 
1832512

species
11111
33113
 1252 

1252 
1252 
1252 
1252 
1252 

111111
species
13413122421

1245 
1245 
1245 
1245 
1245 
1245 
 1245 

 1246 


1246 
1246 
11
11
species

321024

1511761 
1511761 
1511761 
1511761 
1511761 
 1511761 
11111
species

 33964 

33964 


33964 
32
11
species

13

979982 
979982 
 979982 
species
11

42
46254
genus
11

species
11
42


1247 
1247 
 1247 

genus
324242
46255
10152634243

1111
species
2353
 1631871 

1631871 
1631871 
1631871 

1631871 

1


165096 
 165096 
species
1

species
111111
 137591 

137591 
137591 
137591 
137591 
137591 
137591 
7121531152

35


1249 

1249 
 1249 
11
species

species
1111

759620 

759620 
759620 

759620 
 759620 
1331

612
 1993866 


1993866 
1993866 
1993866 
111
species

family
553465
186827
36251094311

553465
genus
1375
36251094311

 128944 

128944 
128944 


128944 
115
111
species

species
111


119206 
119206 
119206 
 119206 
122

111111
species

51665 
51665 
51665 
51665 
51665 
51665 
 51665 
81152145

species
111111
653341

1377 
1377 
1377 
1377 
1377 
1377 
 1377 

1823122

1376 
1376 

1376 
1376 
1376 
 1376 
11111
species

36261
 87541 

87541 
87541 
87541 

87541 
87541 
11111
species

family
373735353827
416780386491604208
33958

1578
389733374433545202
323432333324
genus


1545702 

1545702 
 1545702 
21
11
species

282


1579 
1579 
1579 
 1579 
111
species

 33959 

33959 
33959 
33959 
33959 
33959 
33959 
141113289
111111
species

442162
 1610 

1610 
1610 
1610 
1610 
1610 
1610 
111111
species

species
11111
 1138822 

1138822 

1138822 
1138822 
1138822 
1138822 
324112

551561
 240427 

240427 
240427 
240427 
240427 
240427 
240427 
111111
species

 109790 

109790 
109790 


109790 
1193
111
species

species
111111

1613 
1613 
1613 
1613 
1613 
1613 
 1613 
8181362

1111
species


1622 
1622 

1622 
1622 
 1622 
45461

species
1
 392416 


392416 
1

species
1
1


1600 
 1600 

 1580 

1580 
1580 
1580 
1580 
1580 
1580 
21201123374
111111
species

11
species
 1847728 

1847728 


1847728 
11

species
11
11
 148814 


148814 

148814 

species
111111

1598 
1598 
1598 
1598 
1598 
1598 
 1598 
81741166


1603 
1603 
 1603 
32
11
species

 1601 

1601 
1601 

1601 
1601 
2312
species
1111

111
species
112


293371 


293371 
293371 
 293371 

111111
species
1313101
 375175 

375175 
375175 
375175 
375175 
375175 
375175 


1596 
1596 
1596 
1596 
1596 
1596 
 1596 
1423420
species
111111

111111
species

28038 
28038 
28038 
28038 
28038 
28038 
 28038 
113322432210

336
 1612 

1612 

1612 
1612 
111
species

1
species
2


637971 
 637971 

12121
 97478 

97478 
97478 
97478 
97478 
1111
species

1111
species
7468


1623 
1623 
1623 
1623 
 1623 

1
 1602 


1602 
1
species

10530321
 1587 

1587 
1587 
1587 
1587 
1587 
1587 
111111
species

1
species
2
 60520 


60520 

111111
species

1624 
1624 
1624 
1624 
1624 
1624 
 1624 
61651111435

111111
species
12820388927850

1599 
1599 
1599 
1599 
1599 
1599 
 1599 

 53444 

53444 

53444 
161
11
species

29


1581 


1581 
 1581 
species
11


47715 
47715 
47715 
47715 
47715 
47715 
 47715 
538411196
species
111111

1111
species
2472
 2099789 


2099789 
2099789 
2099789 
2099789 

11
 267818 

267818 


267818 
species
11

1
species
2


2108362 
 2108362 

111111
species

1590 
1590 
1590 
1590 
1590 
1590 
 1590 
26831016913023

1314144
 1604 

1604 
1604 
1604 

1604 
1604 
species
11111


1193095 
1193095 
 1193095 
172
species
11

33101
 152331 

152331 
152331 


152331 
152331 
1111
species

655183
8311234646
222221
species group


1597 
1597 
1597 
1597 
1597 
1597 
 1597 
421818356
species
111111

species
11111

1582 
1582 
1582 
1582 
1582 
 1582 
41041629

111111
species
17135353532
 1584 

1584 
1584 
1584 
1584 
1584 
1584 


83683 
83683 
83683 

83683 
83683 
 83683 
51821
species
11111

species
1111
 89059 

89059 
89059 

89059 

89059 
2311

11

1074467 


1074467 
 1074467 
11
species

111
species


1589 
1589 
1589 
 1589 
2106

31612264
 1007676 

1007676 
1007676 
1007676 
1007676 
1007676 
11111
species

27471258596
1253
533253
genus

species
111
847

114090 
114090 


114090 
 114090 

species
11111

1254 

1254 
1254 
1254 
1254 
 1254 
12661

2444

1255 
1255 


1255 
1255 
 1255 
species
1111

153995241
 51663 

51663 
51663 
51663 
51663 
51663 
species
11111

1111
species

187452 

187452 

187452 
187452 
 187452 
1111

81852
4492504228046116700128187
141613131616
family

genus
121222
1210110127
2737

918103


633807 
633807 
633807 
633807 
633807 
 633807 
11111
species

11111
species

519472 
519472 

519472 
519472 
519472 
 519472 
121224

genus
11111
33969
151103

11111
species
151103
 33970 

33970 
33970 
33970 

33970 
33970 

genus
111210101212
1350
4468483227744916689628163

3761581181246149
 1351 

1351 
1351 
1351 
1351 
1351 
1351 
111111
species

species
111111
24730721092834976811
 1352 

1352 
1352 
1352 
1352 
1352 
1352 

296114423249

53346 
53346 
53346 
53346 
53346 
53346 
 53346 
111111
species

species
111111
10101225914
 1354 

1354 
1354 
1354 
1354 
1354 
1354 

713433

2005703 
2005703 


2005703 
2005703 
 2005703 
1111
species

111111
species
5698913254324719
 2057791 

2057791 
2057791 
2057791 
2057791 
2057791 
2057791 

species
1111
15233

44008 
44008 


44008 
44008 
 44008 

111111
species

37734 
37734 
37734 
37734 
37734 
37734 
 37734 
1750332396872411376

species
111111
121127219955
 53345 

53345 
53345 
53345 
53345 
53345 
53345 

 1353 

1353 
1353 
1353 
1353 
1353 
1353 
6681395276894960
species
111111

111111
species
871192011382545991
 2060307 

2060307 
2060307 
2060307 
2060307 
2060307 
2060307 

11111
species
 417368 


417368 
417368 
417368 
417368 
417368 
462283

genus
111111
51668
116128314

species
111111
 51669 

51669 
51669 
51669 
51669 
51669 
51669 
116128314

4238865008711240194
1300
344038364128
family

1357
541087715538933
genus
144442

 1366 


1366 
1366 
1366 
1366 
1366 
51331
11111
species


1358 
1358 
1358 
1358 
1358 
1358 
 1358 
54906411536932
111111
species


1363 
1363 
1363 
1363 
 1363 
12113611
1111
species

1116
 1364 


1364 
1364 
1364 
1364 
1111
species

genus
333634323726
369778423716851161
1301

882241

1349 
1349 
1349 
1349 
1349 
1349 
 1349 
species
111111

 102684 

102684 
102684 
102684 
102684 
102684 
102684 
26131111
species
111111

species
111111

1302 
1302 
1302 
1302 
1302 
1302 
 1302 
19482171

11111
species
42294
 45634 

45634 

45634 
45634 
45634 
45634 

1111
species
4941
 1318 

1318 

1318 

1318 
1318 

 1348 


1348 
1348 
1348 
316
species
111

74884617238
 1308 

1308 
1308 
1308 
1308 
1308 
1308 
species
111111

111111
species
361114915520033

1314 
1314 
1314 
1314 
1314 
1314 
 1314 

species
1111
7345
 1156431 

1156431 
1156431 
1156431 


1156431 

 1305 

1305 
1305 
1305 
1305 
1305 
1305 
24371621202
111111
species

112
 1326 

1326 
1326 


1326 
111
species

species
111
161
 1825069 


1825069 
1825069 

1825069 

4
 1814128 


1814128 
1
species

species
11
51
 1902136 

1902136 
1902136 

 1303 

1303 
1303 
1303 
1303 
1303 
1303 
611201054
111111
species

481262
 150055 

150055 
150055 
150055 
150055 
150055 
150055 
111111
species

671232
7191731181
333331
species group

259203

76860 
76860 
76860 
76860 
76860 
 76860 
species
11111

11111
species
23433
 1328 

1328 
1328 
1328 
1328 
1328 

111111
species
31148121

1338 
1338 
1338 
1338 
1338 
1338 
 1338 

213
 1839799 


1839799 
1839799 
1839799 
species
111

111


1340 
1340 
1340 
 1340 
111
species

98547169899

1307 
1307 
1307 
1307 
1307 
1307 
 1307 
species
111111


1346 
1346 
1346 
1346 
 1346 
6133
species
1111


1811193 

1811193 
1811193 
 1811193 
561
species
111

species
11
 257758 

257758 
257758 
11

species group
222222
119603
84015185413

111111
species
41811263

1336 
1336 
1336 
1336 
1336 
1336 
 1336 

4224164810
 1334 

1334 
1334 
1334 
1334 
1334 
1334 
species
111111

11111
species
79312
 1156433 

1156433 
1156433 
1156433 

1156433 
1156433 

11111
species
 1310 

1310 

1310 
1310 
1310 
1310 
268162


1311 
1311 
1311 
1311 
1311 
1311 
 1311 
1188365912010
111111
species

 82348 


82348 

82348 
82348 
176
species
111

species
11111

1335 
1335 
1335 
1335 
1335 
 1335 
11111

1111
species

113107 
113107 


113107 
113107 
 113107 
13643

species
111111
14152311159

1304 
1304 
1304 
1304 
1304 
1304 
 1304 

 315405 

315405 
315405 
315405 
315405 
315405 
315405 
319811193
111111
species

111
species
414


1345 

1345 
1345 
 1345 

1
 400065 


400065 
species
1


59310 

59310 
59310 
 59310 
2181
species
111

112532471

28037 
28037 
28037 
28037 
28037 
28037 
 28037 
111111
species

11
species
23
 197614 

197614 
197614 


712633 
712633 
712633 
712633 
712633 
712633 
 712633 
156824
species
111111

631216312216738
 1313 

1313 
1313 
1313 
1313 
1313 
1313 
species
111111

species
111111
 1309 

1309 
1309 
1309 
1309 
1309 
1309 
3206843

 1759399 

1759399 
1759399 
1759399 
354
species
111

order
188215206210214108
5114106800459342238631836601482
1385

313733323419
family
10912408823990867206
90964

3561131115
69965
genus
13233

species
11111

69966 
69966 
69966 
69966 
69966 
 69966 
33821487

8814
 1855823 


1855823 

1855823 
1855823 
111
species

species
1111
109914
 1898474 


1898474 
1898474 
1898474 
1898474 

2005363
5612432
genus
111111

111111
species

1849491 
1849491 
1849491 
1849491 
1849491 
1849491 
 1849491 
5612432

10822344809932744204
1279
283229272918
genus

 61015 

61015 
61015 
61015 
61015 
61015 
415511
11111
species

 1284 


1284 
1284 
1284 
1284 
611010
species
1111

species
11111

985762 
985762 
985762 
985762 
985762 
 985762 
81211

738534311

1288 
1288 
1288 
1288 
1288 
1288 
 1288 
111111
species

species
111111
11151514
 45972 

45972 
45972 
45972 
45972 
45972 
45972 

159224
 170573 


170573 
170573 
170573 
170573 
species
1111

35


155085 

155085 
 155085 
11
species

 46127 

46127 
46127 


46127 
541
111
species

3151


1715860 
1715860 

1715860 
1715860 
 1715860 
1111
species

species
11111
7351133

643214 
643214 
643214 
643214 
643214 
 643214 

11111
species
 29379 

29379 
29379 
29379 
29379 
29379 
21516217

species
111111
 29382 

29382 
29382 
29382 
29382 
29382 
29382 
641331102

 29388 

29388 
29388 
29388 
29388 
29388 
29388 
4378482871002971
111111
species

1111
species
2244

70258 
70258 
70258 
70258 
 70258 

1111
species
 985002 

985002 
985002 
985002 

985002 
1253

1417218
 1281 

1281 
1281 
1281 
1281 
1281 
species
11111

species
111111
29471969381

29385 
29385 
29385 
29385 
29385 
29385 
 29385 

1111
species
216417

1294 
1294 
1294 
1294 
 1294 

species
111111
21366323532738447
 1280 

1280 
1280 
1280 
1280 
1280 
1280 

415112

70255 
70255 
70255 
70255 
70255 
 70255 
11111
species

species
111111
10987116
 246432 

246432 
246432 
246432 
246432 
246432 
246432 

species
111111
2611156188

1290 
1290 
1290 
1290 
1290 
1290 
 1290 

111111
species
7711895393
 1286 

1286 
1286 
1286 
1286 
1286 
1286 

species
111111

214473 
214473 
214473 
214473 
214473 
214473 
 214473 
32221263

3421731411

283734 
283734 
283734 
283734 
283734 
283734 
 283734 
111111
species


1282 
1282 
1282 
1282 
1282 
1282 
 1282 
20728974682745
111111
species

species
111111
 1283 

1283 
1283 
1283 
1283 
1283 
1283 
1532361464


28035 
28035 
28035 
28035 
28035 
28035 
 28035 
13442541163
species
111111

 29384 

29384 
29384 


29384 
291
species
111

species
11111

1296 
1296 
1296 

1296 
1296 
 1296 
113252

species
111111

1292 
1292 
1292 
1292 
1292 
1292 
 1292 
1086621

species
111111

1295 
1295 
1295 
1295 
1295 
1295 
 1295 
2285741

12235
45669
genus
11111

12235

407035 
407035 
407035 
407035 
407035 
 407035 
species
11111

186820
2256863407611024169
565343
family

2755
220613242
111111
genus

 2756 

2756 
2756 
2756 
2756 
2756 
2756 
220613242
species
111111

genus
454232
1637
2236663347481000167

species
111111
 1638 

1638 
1638 
1638 
1638 
1638 
1638 
15551838373

11
species
15

1640 
1640 
 1640 

8715
 1643 

1643 
1643 
1643 

1643 
1111
species

species
11


1642 
1642 
 1642 
21

species
111111
 1639 

1639 
1639 
1639 
1639 
1639 
1639 
199597314710958164

111111
family
114371101
186821

species
111111
114371101

85683 
85683 
85683 
85683 
85683 
85683 
 85683 

186824
2321413
family
112212

1111
genus
292635
2122


2071623 

2071623 
2071623 

2071623 
 2071623 
2122
1111
species

11111
genus
311211
1677050

311211


1471761 
1471761 
1471761 
1471761 
1471761 
 1471761 
species
11111

937438931112413513188
186822
family
403841424227

genus
343235363623
44249
906359927411283416156

species
111111
34229119551
 1870819 

1870819 
1870819 
1870819 
1870819 
1870819 
1870819 

species
111111
60482542679349

61624 
61624 
61624 
61624 
61624 
61624 
 61624 

species
111111
33411141
 1532905 

1532905 
1532905 
1532905 
1532905 
1532905 
1532905 

4514191

1462996 

1462996 
1462996 
1462996 
1462996 
 1462996 
11111
species

species
111111
17142854491
 1464 

1464 
1464 
1464 
1464 
1464 
1464 

11111
species

528191 
528191 
528191 
528191 
528191 
 528191 
28118504411881

species
1111

169760 

169760 
169760 
169760 
 169760 
981515

2617333

159743 
159743 
159743 
159743 
159743 
 159743 
11111
species

species
1111
32814


160799 
160799 
160799 
160799 
 160799 

species
111111
15414251713

1536772 
1536772 
1536772 
1536772 
1536772 
1536772 
 1536772 

111111
species

414771 
414771 
414771 
414771 
414771 
414771 
 414771 
22397274

species
111111

189425 
189425 
189425 
189425 
189425 
189425 
 189425 
911112281

species
11111
157691961
 481743 

481743 
481743 
481743 
481743 
481743 


162209 
162209 

162209 
162209 
162209 
 162209 
198341
11111
species

111111
species
182742232095516
 1406 

1406 
1406 
1406 
1406 
1406 
1406 

11111
species
5141619

1178515 
1178515 
1178515 
1178515 
1178515 
 1178515 

species
11111
268916
 324057 


324057 
324057 
324057 
324057 
324057 

3131415
 172713 

172713 
172713 
172713 
172713 
172713 
11111
species

111111
species
111251212
 2069255 

2069255 
2069255 
2069255 
2069255 
2069255 
2069255 

species
111111
15132254
 1763538 

1763538 
1763538 
1763538 
1763538 
1763538 
1763538 


59893 

59893 
59893 
59893 
 59893 
55116
1111
species

11111
species
 1695218 

1695218 
1695218 
1695218 
1695218 
1695218 
24192249

11111
species
 1566358 

1566358 
1566358 
1566358 
1566358 
1566358 
1935326

 365617 

365617 
365617 
365617 
365617 
365617 
365617 
86251512
111111
species

131221730
 1536770 

1536770 
1536770 
1536770 
1536770 
1536770 
species
11111

111111
species

1536769 
1536769 
1536769 
1536769 
1536769 
1536769 
 1536769 
947141720

 1616788 

1616788 
1616788 
1616788 
1616788 
1616788 
1616788 
126232833236
species
111111

111111
species

1536774 
1536774 
1536774 
1536774 
1536774 
1536774 
 1536774 
43918352

species
111111
 1870820 

1870820 
1870820 
1870820 
1870820 
1870820 
1870820 
39181024265


1536773 
1536773 
1536773 
1536773 
1536773 
1536773 
 1536773 
125834421
111111
species


44251 
44251 
44251 
44251 
44251 
44251 
 44251 
1651031223
111111
species


189426 
189426 
189426 
189426 
189426 
189426 
 189426 
3491434861
111111
species

species
111111
410623224
 1126833 

1126833 
1126833 
1126833 
1126833 
1126833 
1126833 

species
11111
 1536771 

1536771 
1536771 
1536771 
1536771 
1536771 
611720

715829
 1536775 

1536775 
1536775 
1536775 
1536775 
1536775 
species
11111

16314482
2044880
11111
species group

species
11111
 483937 

483937 

483937 
483937 
483937 
483937 
16314482

genus
111111
13157163010
76632

111111
species
13157163010
 377615 

377615 
377615 
377615 
377615 
377615 
377615 

genus
222221
59618263
55079

464148
 1450761 

1450761 
1450761 
1450761 
1450761 
1450761 
species
11111

1324183

1500254 
1500254 
1500254 
1500254 
1500254 
1500254 
 1500254 
species
111111

genus
333332
55080
135524794119

11111
species
 1393 

1393 
1393 
1393 
1393 
1393 
32182420

44615418
 54913 

54913 
54913 
54913 
54913 
54913 
54913 
species
111111


1465 
1465 
1465 
1465 
1465 
1465 
 1465 
6301040171
species
111111

family
7999929710041
25476334232257943173739
186817

182709
332453519
genus
11111

 182710 

182710 
182710 
182710 
182710 
182710 
332453519
11111
species

453441
genus
150247
129825144441

 294699 

294699 
294699 
294699 
294699 
294699 
1304538
11111
species

23
 1490057 

1490057 
1490057 
11
species

 33934 

33934 
33934 
33934 
33934 
33934 
33934 
7431754201
111111
species

1111
species
210145
 1490052 

1490052 
1490052 

1490052 
1490052 

species
1111


198467 
198467 
198467 
198467 
 198467 
1242311

genus
12122
23357430
45667

1111
species

402384 
402384 

402384 
402384 
 402384 
2416

 1570 


1570 
1570 
1570 
1570 
2957324
1111
species

553554
genus
14317291812
84406


1473 


1473 
1473 
1473 
 1473 
1211
1111
species

11111
species
32148
 1482 

1482 
1482 

1482 
1482 
1482 

811
 163877 


163877 
163877 
163877 
111
species

 403957 

403957 
403957 
403957 
403957 
403957 
11213
11111
species

8111

1911587 
1911587 


1911587 
1911587 
 1911587 
species
1111

species
111111
 2017483 

2017483 
2017483 
2017483 
2017483 
2017483 
2017483 
11942492

genus
22221
1329200
1251651


255247 
255247 
255247 
255247 
255247 
 255247 
112931
species
11111

1372


1221500 
1221500 
1221500 
1221500 
 1221500 
1111
species

432739222
1906945
111111
genus

 1426 

1426 
1426 
1426 
1426 
1426 
1426 
432739222
111111
species

1111
genus
4143
459532

 586416 


586416 
586416 
586416 
586416 
4143
1111
species

1111
genus
1434
200903

 2213194 

2213194 
2213194 

2213194 
2213194 
1434
1111
species

2084819
351195
genus
1111

1111
species
2084819


1230341 
1230341 
1230341 
1230341 
 1230341 

23845451215349582773669
1386
genus
486059596124

10251314
 2093834 

2093834 
2093834 
2093834 
2093834 
2093834 
11111
species

species
1111
 1127744 

1127744 

1127744 
1127744 
1127744 
11175

1331112
 86664 

86664 
86664 
86664 
86664 
86664 
species
11111

11111
species
 1479 

1479 
1479 
1479 
1479 
1479 
11573315

1111
species

1892404 
1892404 
1892404 
1892404 
 1892404 
112388

species
111
 1446792 


1446792 
1446792 
1446792 
114

11111
species
1214267

135735 
135735 
135735 
135735 
135735 
 135735 

111111
species
2952581

1467 
1467 
1467 
1467 
1467 
1467 
 1467 

species
11111

98228 
98228 
98228 
98228 
98228 
 98228 
19121

551

756828 
756828 

756828 
 756828 
111
species

species
11111

1664069 
1664069 
1664069 
1664069 
1664069 
 1664069 
111443410

 2009331 

2009331 
2009331 
2009331 
2009331 
2009331 
196328
11111
species

33141592731342

1398 
1398 
1398 
1398 
1398 
1398 
 1398 
111111
species

153221
 665099 


665099 
665099 
665099 
665099 
665099 
11111
species

111111
species
395102225
 1471 

1471 
1471 
1471 
1471 
1471 
1471 

511
 2053832 


2053832 

2053832 

2053832 
species
111

652177848
 1478 

1478 
1478 
1478 
1478 
1478 
species
11111


79880 
79880 
79880 
79880 
79880 
 79880 
4892616994
11111
species


563169 
563169 
563169 
563169 
 563169 
1234
species
1111

22052712

86665 
86665 
86665 
86665 
86665 
 86665 
11111
species


632773 
632773 
632773 
632773 
632773 
 632773 
3367458
11111
species

species
111111
710428223952
 1408 

1408 
1408 
1408 
1408 
1408 
1408 

889898
species group
653685
18394338219381100245

5745718590346837
1938374
233333
species subgroup

4031712362235132

492670 
492670 
492670 
492670 
492670 
492670 
 492670 
species
111111


659243 
659243 
659243 
659243 
659243 
 659243 
1441641
11111
species


1390 
1390 
1390 
1390 
1390 
1390 
 1390 
17126582651134
species
111111

species
111111
93305125562457174
 1423 

1423 
1423 
1423 
1423 
1423 
1423 


1452 
1452 
1452 
1452 
1452 
1452 
 1452 
125214128421
species
111111

111111
species
41613773428

1648923 
1648923 
1648923 
1648923 
1648923 
1648923 
 1648923 

species
1111

72361 

72361 

72361 
72361 
 72361 
11441

11111
species

119858 
119858 
119858 
119858 
119858 
 119858 
47116

species
111111

1402 
1402 
1402 
1402 
1402 
1402 
 1402 
1210630267894


1178537 

1178537 
1178537 
 1178537 
111
species
111

 658666 


658666 
658666 
658666 
658666 
61106
species
1111

5105
 264697 


264697 

264697 
264697 
111
species

6522174472

561879 
561879 
561879 
561879 
561879 
561879 
 561879 
species
111111

 1547283 


1547283 
1547283 
11
species
11

1111
species
 1827146 


1827146 
1827146 
1827146 
1827146 
131213

1111
species

1565991 
1565991 
1565991 

1565991 
 1565991 
14611


1839798 
1839798 
1839798 
 1839798 
3261
111
species

species
1

1837130 
 1837130 
1

15027819
 79885 

79885 
79885 
79885 
79885 
79885 
species
11111

42

2026248 


2026248 
 2026248 
11
species

 199441 


199441 
199441 
199441 
199441 
199441 
84952
11111
species

1111
species
1412
 79883 

79883 
79883 
79883 

79883 


33932 
33932 
33932 
33932 
 33932 
2911
species
1111

species
1
6


1574141 
 1574141 

51852312
1792192
species group
11111

11111
species
 293387 

293387 
293387 
293387 
293387 
293387 
51852312

species
11
11
 2049935 


2049935 

2049935 

species
111111
222691
 1774743 

1774743 
1774743 
1774743 
1774743 
1774743 
1774743 

1111
species


1941347 
1941347 
1941347 
1941347 
 1941347 
4122

species
1111
5131


1856406 
1856406 
1856406 
1856406 
 1856406 

 300825 

300825 
300825 
300825 
300825 
300825 
421853
species
11111

111
species
 1409 


1409 
1409 
1409 
211

species
111111
1720119535

1413 
1413 
1413 
1413 
1413 
1413 
 1413 


1570330 
1570330 
1570330 
1570330 
 1570330 
11132
1111
species

 1404 

1404 
1404 
1404 
1404 
1404 
1404 
174223611072358307295
species
111111

1673

1628753 
1628753 

1628753 
1628753 
 1628753 
species
1111

species group
565654
2771170375128862186
86661

27722171363

1405 
1405 
1405 
1405 
1405 
1405 
 1405 
111111
species

species
111111

1396 
1396 
1396 
1396 
1396 
1396 
 1396 
713858539015534

151052813863
 580165 

580165 
580165 
580165 
580165 
580165 
species
11111

species
111111

1392 
1392 
1392 
1392 
1392 
1392 
 1392 
47288903491867


155322 

155322 
 155322 
12
species
11


1428 
1428 
1428 
1428 
1428 
1428 
 1428 
11731915133818142
species
111111

11
species
 666686 


666686 


666686 
151

species
11111
2983212

2052936 
2052936 
2052936 
2052936 
2052936 
 2052936 

species
1111
642312
 412384 


412384 
412384 
412384 
412384 

species
11111
1434103712

859143 
859143 
859143 
859143 
859143 
 859143 

111
species
3212


35841 
35841 

35841 
 35841 

261125491

324767 
324767 
324767 
324767 
324767 
324767 
 324767 
species
111111

4846314
1055323
genus
111111

species
111111
4846314
 33936 

33936 
33936 
33936 
33936 
33936 
33936 

11111
genus
29331
4139165

11111
species
 1449 

1449 
1449 
1449 
1449 
1449 
4139165

355551
genus
400634
414273278392

1111
species


28031 
28031 
28031 
28031 
 28031 
34215

11111
species
 1421 

1421 
1421 
1421 
1421 
1421 
6292174022

 1145276 


1145276 
1145276 
1145276 
1145276 
4772810
1111
species

 2169540 

2169540 
2169540 
2169540 
2169540 
2169540 
2817231
species
11111

7374612
 2072025 

2072025 
2072025 
2072025 
2072025 
2072025 
2072025 
species
111111

1
genus
1
175304

1


1472767 
 1472767 
species
1

129337
481776134318938
13131312148
genus

species
11111
1123137

550542 
550542 
550542 
550542 
550542 
 550542 

species
111111
144434252

1233873 
1233873 
1233873 
1233873 
1233873 
1233873 
 1233873 

1505648
144224124692
species group
443442

111111
species
 33941 

33941 
33941 
33941 
33941 
33941 
33941 
8191162261

1892317

1462 
1462 
1462 
1462 
1462 
 1462 
species
11111

11111
species
313151

169283 
169283 

169283 
169283 
169283 
 169283 

 33938 

33938 
33938 
33938 
33938 
33938 
21443611
11111
species

112163782
 1422 

1422 
1422 
1422 
1422 
1422 
1422 
111111
species

species
11111
 471223 

471223 
471223 
471223 
471223 
471223 
41052522

45122
 691437 

691437 
691437 
691437 

691437 
691437 
11111
species

species
111111
620962355
 33940 

33940 
33940 
33940 
33940 
33940 
33940 

species
1111


129338 
129338 
129338 
129338 
 129338 
1192

 544556 

544556 

544556 
544556 
544556 
544556 
136413
species
11111


1921421 
1921421 
1921421 
1921421 
1921421 
1921421 
 1921421 
5213331412
species
111111

species
1111
1121

581103 
581103 
581103 

581103 
 581103 

186818
7613971798283689
family
19212021215

1569
1414840391534
444442
genus

111111
species

1571 
1571 
1571 
1571 
1571 
1571 
 1571 
1010021102423

species
11111
119122611
 1476 

1476 
1476 
1476 
1476 
1476 


1930764 
1930764 
1930764 
1930764 
1930764 
 1930764 
111362
11111
species

111111
species
21842281

1930546 
1930546 
1930546 
1930546 
1930546 
1930546 
 1930546 

41481710722
648800
22222
genus

 2048654 

2048654 
2048654 
2048654 
2048654 
2048654 
1248141
11111
species

 76853 

76853 
76853 
76853 
76853 
76853 
312499321
species
11111

genus
11111
4852516
648802

 241244 

241244 
241244 
241244 
241244 
241244 
4852516
species
11111

genus
1111
7271512
1649

7271512
 1750719 


1750719 
1750719 
1750719 
1750719 
species
1111

352320201
157226
genus
11111

species
11111
352320201


1508404 
1508404 
1508404 
1508404 
1508404 
 1508404 

12121112122
genus
54254963002454
1372

11111
species
33392231
 161360 

161360 
161360 
161360 
161360 
161360 

11111
species
 1215089 

1215089 
1215089 
1215089 
1215089 
1215089 
4581919

11111
species
 192421 

192421 
192421 
192421 
192421 
192421 
112986417

427234071

2058136 
2058136 
2058136 
2058136 
2058136 
 2058136 
species
11111

2317257

1526927 
1526927 
1526927 
1526927 
1526927 
 1526927 
11111
species

species
111111

2213202 
2213202 
2213202 
2213202 
2213202 
2213202 
 2213202 
295272

11111
species

1038856 
1038856 
1038856 
1038856 
1038856 
 1038856 
724202217

species
1111

414778 
414778 

414778 
414778 
 414778 
118610

11111
species
22062215
 1302659 

1302659 
1302659 
1302659 
1302659 
1302659 

species
111111
 1374 

1374 
1374 
1374 
1374 
1374 
1374 
824221172

61642918
 200991 

200991 
200991 
200991 
200991 
200991 
species
11111

11111
species
 1598147 

1598147 
1598147 
1598147 
1598147 
1598147 
41842816

545544
family
362416682911
186823

29330
1375911
genus
111111

111111
species
 405212 

405212 
405212 
405212 
405212 
405212 
405212 
1375911

1814751219
432330
222222
genus

111111
species
 1214604 

1214604 
1214604 
1214604 
1214604 
1214604 
1214604 
29434193

16531726

1903704 
1903704 
1903704 
1903704 
1903704 
1903704 
 1903704 
111111
species

534871
1129704
genus
212211


33943 
33943 
33943 
33943 
33943 
33943 
 33943 
132271
111111
species

111
species
426

2055160 

2055160 
2055160 
 2055160 

1378
28126
genus
12111

1
species
 29391 


29391 
5

23126

1785995 
1785995 
1785995 
1785995 
1785995 
 1785995 
species
11111

666666
genus
33986
1979548832937214094174669156

23185874357378661615327

132920 
132920 
132920 
132920 
132920 
132920 
 132920 
species
111111

111111
species
 1224749 

1224749 
1224749 
1224749 
1224749 
1224749 
1224749 
125545198271382694110

8758058180361554576848397
 332410 

332410 
332410 
332410 
332410 
332410 
332410 
species
111111

species
111111
12552917906465286059
 1849031 

1849031 
1849031 
1849031 
1849031 
1849031 
1849031 

18757199760813427110

1399115 
1399115 
1399115 
1399115 
1399115 
1399115 
 1399115 
111111
species

111111
species
45198577510872163
 360911 

360911 
360911 
360911 
360911 
360911 
360911 

819682718061
class
186801
1381272616891056852643

1284263216321014820621
186802
688265626651
order

222624231812
family
6701015937484309303
31979

genus
11111
18582
1981033

18582

2086584 
2086584 
2086584 
2086584 
2086584 
 2086584 
species
11111

49082
1311752
genus
31221

species
111
361
 1041504 


1041504 

1041504 
1041504 

911142
 1508644 


1508644 
1508644 
1508644 
1508644 
1508644 
species
11111

species
1
 49118 


49118 
1

1485
664992929452301298
genus
19202017149

21741
 1493 

1493 
1493 
1493 


1493 
species
1111

111111
species
 1492 

1492 
1492 
1492 
1492 
1492 
1492 
126152

2235

46867 
46867 
46867 
46867 
 46867 
1111
species


1216932 
 1216932 
2
1
species

1
species
2


84022 
 84022 


1548 
1548 

1548 
 1548 
122
111
species

2
 1488 


1488 
1
species

111111
species
384035526
 169679 

169679 
169679 
169679 
169679 
169679 
169679 

2121
 332101 

332101 

332101 
332101 
332101 
species
1111

species
1

1509 
 1509 
3

2436775363
 1502 

1502 
1502 
1502 
1502 
1502 
1502 
111111
species

11
species
21
 84023 


84023 
84023 

species
11111
49211

394958 
394958 

394958 
394958 
394958 
 394958 

species
1


29341 
 29341 
1

111111
species
18920591111526

1501 
1501 
1501 
1501 
1501 
1501 
 1501 

species
1111
15122


1497 
1497 
1497 
1497 
 1497 

species
111
436

1561 
1561 
1561 
 1561 

111
species


36745 
36745 
36745 
 36745 
652

1111
species
11111

1542 
1542 
1542 
1542 
 1542 

species
1111
11031
 1534 

1534 
1534 
1534 

1534 

25


1042156 
1042156 
 1042156 
species
11

 1494 

1494 
1494 
31
11
species

species
1


1519 
 1519 
1


1513 
1513 
1513 
1513 
1513 
1513 
 1513 
10102121
species
111111


238834 
238834 

238834 
 238834 
261
species
111


1491 
1491 
1491 
1491 
1491 
1491 
 1491 
328268747399226256
111111
species


217159 

217159 

217159 
 217159 
211
111
species

species
1111
3622911
 1520 

1520 
1520 
1520 
1520 

species
111


755731 
755731 


755731 
 755731 
522

111
genus
114627
111

1
 208226 


208226 
1
species

 461876 


461876 
461876 
11
11
species

3121

2082193 

2082193 
2082193 

2082193 
 2082193 
1111
species

genus
11111
21412
390805

11111
species
 1424294 

1424294 
1424294 

1424294 
1424294 
1424294 
21412

family
111111
2013743616
216572

459786
2013743616
genus
111111

2013743616
 351091 

351091 
351091 
351091 
351091 
351091 
351091 
111111
species

186807
45381871711
81175117
family

genus
11
2282742
21

21
 58138 


58138 


58138 
species
11

132124
genus
36853
383137

11111
species
 49338 


49338 
49338 
49338 
49338 
49338 
61112

 233055 


233055 
233055 


233055 
122
species
111

species
11
12
 36854 


36854 


36854 

 142877 

142877 


142877 
142877 
321
species
111

11221
genus
1562
91461

1111


1565 
1565 

1565 
1565 
 1565 
1111
species

species
11
93

59610 

59610 
 59610 


1833852 
 1833852 
5
species
1

471826
2
genus
1

2

471827 
 471827 
species
1

genus
1
8
278993

species
1
 863643 


863643 
8

51196
16111
genus
1111

species
1111
 51197 

51197 

51197 
51197 
51197 
16111

321222
genus
79206
14168433


1563 


1563 
1563 
1563 
 1563 
2121
1111
species

11158312

885581 
885581 
885581 
885581 
885581 
885581 
 885581 
species
111111

11
species
11

79209 
79209 
 79209 

genus
111
111
51514

111
species

51515 
51515 


51515 
 51515 
111

genus
111
2282740
121

species
111
121


102134 
102134 

102134 
 102134 

111
genus
56112
111

species
11
11


55583 


55583 
 55583 

species
1
1


1131462 
 1131462 

53783
539000
11111
family

53783
73918
11111
genus

53783
 73919 

73919 

73919 
73919 
73919 
73919 
11111
species

543314
111
111
family

1
86331
1
genus

species
1
1


114527 
 114527 

11
species
11


143393 


143393 
 143393 

family
1111
543347
54162

54162
178898
1111
genus


178899 
178899 

178899 

178899 
 178899 
54162
species
1111

family
111111
543349
392955

111111
genus
2733
392955

 2734 

2734 
2734 
2734 
2734 
2734 
2734 
392955
species
111111

family
11
68298
11

11
genus
129001
11

species
11
11
 86170 

86170 


86170 

111111
family
1921410
31984

1921410
2697
genus
111111

1921410

35701 
35701 
35701 
35701 
35701 
35701 
 35701 
species
111111

746863811825763
186803
family
15171491311

11111
species
38214

39491 
39491 
39491 
39491 
39491 
 39491 

555355
genus
1506553
22364142682212

34212
 1834196 

1834196 
1834196 
1834196 

1834196 
1834196 
11111
species

species
111111

208479 
208479 
208479 
208479 
208479 
208479 
 208479 
2282221

 84030 

84030 
84030 
84030 
84030 
84030 
84030 
23021336484
species
111111

species
111111
3151241
 1871021 

1871021 
1871021 
1871021 
1871021 
1871021 
1871021 

11111
species
1215474

66219 
66219 
66219 

66219 
66219 
 66219 

2039240
4731
genus
1111

species
1111
4731
 28446 

28446 
28446 
28446 
28446 

333222
genus
2324021131542
572511

1723417121438
 1796616 

1796616 
1796616 
1796616 
1796616 
1796616 
1796616 
111111
species


1322 
1322 
1322 
 1322 
212
111
species

species
111111
452114
 1912897 

1912897 
1912897 
1912897 
1912897 
1912897 
1912897 

116361
841
11111
genus

11111
species
 301301 

301301 
301301 
301301 

301301 
301301 
116361

111111
genus
1212131
698776

1212131
 29360 

29360 
29360 
29360 
29360 
29360 
29360 
111111
species

1211
genus
1411
830


43305 
43305 
 43305 
31
11
species

species
111
111
 185008 

185008 
185008 


185008 

species
111

712991 
712991 


712991 
 712991 
414

73
1663717
genus
11

 1679721 


1679721 


1679721 
73
species
11

4382079824
207244
111111
genus


649756 
649756 
649756 
649756 
649756 
649756 
 649756 
4382079824
species
111111

111111
genus
117202123
946234

111111
species
117202123

292800 
292800 
292800 
292800 
292800 
292800 
 292800 

family
241221
186804
411193964267

genus
11
1481960
12

12
 1731 

1731 
1731 
11
species

1849828
1
1
genus

1
species
1


1505 
 1505 

genus
1
186831
1

1


1511 
 1511 
species
1

44259
32
11
genus

32
 143361 


143361 
143361 
11
species

111111
genus
401153961247
1870884

401153961247

1496 
1496 
1496 
1496 
1496 
1496 
 1496 
species
111111

family
344332
186806
1718793128

11111
genus
33951
51111

species
11111
51111

33952 
33952 
33952 
33952 
33952 
 33952 

genus
233222
1218682118
1730

species
11111
299314

39488 
39488 
39488 
39488 
39488 
 39488 

species
1111
82112


39485 
39485 
39485 

39485 
 39485 

105476

1736 
1736 
1736 

1736 
1736 
 1736 
species
11111

genus
111111
1392389
66812101

species
111111
66812101
 1297617 

1297617 
1297617 
1297617 
1297617 
1297617 
1297617 

541000
5511133494942
family
912811119

1263
1316
genus
1111

species
11


1160721 
1160721 
 1160721 
16


1264 
1264 
 1264 
13
11
species

species
11111
 1572656 

1572656 
1572656 
1572656 

1572656 
1572656 
31111

555654
genus
1508657
347919322734

species
11111
11111

1521 

1521 
1521 
1521 
1521 
 1521 

species
111
3031
 84032 


84032 
84032 
84032 


1834198 
1834198 
1834198 
1834198 
1834198 
 1834198 
23172
species
11111

species
11
33
 288965 


288965 


288965 

169861611

1515 
1515 
1515 
1515 
1515 
1515 
 1515 
species
111111

species
111111
1234616520

1510 
1510 
1510 
1510 
1510 
1510 
 1510 

species
111

29343 


29343 

29343 
 29343 
312

genus
1111
1111
1637257


884684 

884684 
884684 
884684 
 884684 
1111
1111
species

1890281
121
111
genus

111
species


1677857 

1677857 

1677857 
 1677857 
121

genus
111
236752
354

354
 236753 


236753 

236753 
236753 
111
species

11188864
216851
genus
111111

111111
species
11188864
 853 

853 
853 
853 
853 
853 
853 

genus
11111
253238
65541

species
11111
65541

253239 
253239 
253239 

253239 
253239 
 253239 

111111
family
990719
340256176184304146

genus
111111
990721
340256176184304146

species
111111
340256176184304146

1805714 
1805714 
1805714 
1805714 
1805714 
1805714 
 1805714 

23013431
53433
124221
order

53434
229112
family
11111

genus
1111
22912
28186

species
1111
 28187 

28187 
28187 
28187 

28187 
22912

42417
1
1
genus

1
species
 42422 


42422 
1

972
112311
13111
family

11
genus
17
32636

 31909 


31909 
31909 
17
species
11

5311
2330
2111
genus

species
1111
3311


2331 
2331 
2331 
2331 
 2331 

1
species
 656519 


656519 
2

1211137129
order
955644382921
68295

337233
family
1459238
543371

1
291988
1
genus


291990 
 291990 
1
1
species

225223
genus
44000
1326228

111
species
 717609 

717609 

717609 
717609 
1011

 44001 

44001 

44001 


44001 
315
species
111

1
species
 52766 


52766 
1

species
11
11


413889 


413889 
 413889 

species
1111
 55205 


55205 
55205 
55205 
55205 
2112

1


31899 
 31899 
1
species

species
11
 52765 


52765 
52765 
11

1111
genus
1321
28895

1321

1517 
1517 
1517 

1517 
 1517 
species
1111

family
874475
774729352412
186814

28431734
44260
genus
111111

species
111111
28431734
 1525 

1525 
1525 
1525 
1525 
1525 
1525 

11
genus
140458
31

11
species
31


85874 


85874 
 85874 

genus
1
2
249529

2

911092 
 911092 
1
species

1754
3839211896
442333
genus

1
species

573062 
 573062 
1

species
1111
3111

496866 


496866 
496866 
496866 
 496866 

species
111


108150 
108150 
108150 
 108150 
125


46354 
46354 
46354 
46354 
46354 
46354 
 46354 
3334191264
species
111111

 399726 


399726 
3
species
1

1
species
1

583357 
 583357 

11
species
11


2325 


2325 
 2325 

1
species
2
 29323 


29323 

559
499228
genus
111

 499229 

499229 

499229 

499229 
559
111
species

1111
genus
42837
4122


42838 
42838 


42838 
42838 
 42838 
4122
1111
species

family
111111
443111
543372

genus
111111
252965
443111

443111

252966 
252966 
252966 
252966 
252966 
252966 
 252966 
111111
species

family
11
227387
31

227388
31
genus
11

3
 1794699 


1794699 
species
1


184064 
 184064 
1
1
species

order
1
8
485256

8
485255
family
1

8
375928
genus
1

species
1


375929 
 375929 
8

33415133
508458
phylum
223322

649775
33415133
223322
class

order
223322
649776
33415133

223322
family
649777
33415133

1111
genus
1113
508459

species
1111
 1197717 


1197717 
1197717 
1197717 
1197717 
1113

81461
2213102
11111
genus

11111
species

81462 

81462 
81462 
81462 
81462 
 81462 
2213102

11
49894
11
genus

 97477 

97477 


97477 
11
species
11

81466
211
111
genus

species
111
211


81468 
81468 
81468 
 81468 

998798
phylum
57723
76172243612690

332159
10981119
11111
class

11111
order
332160
10981119

family
11111
332161
10981119

11111
genus
332162
10981119

11111
species
10981119
 332163 

332163 
332163 
332163 

332163 
332163 

403414318030
204432
class
665665

order
665665
403414318030
204433

204434
403414318030
665665
family

392733
14195133422
genus
222222

111111
species

392734 
392734 
392734 
392734 
392734 
392734 
 392734 
91349277

species
111111
 870903 

870903 
870903 
870903 
870903 
870903 
870903 
5614715

169615347
940557
genus
222222

species
111111
7522154
 940614 

940614 
940614 
940614 
940614 
940614 
940614 

94413193
 940615 

940615 
940615 
940615 
940615 
940615 
940615 
species
111111

6125
33973
1111
genus

1111
species

33075 
33075 

33075 
33075 
 33075 
6125

111111
genus
453171
658061

111111
species
 658062 

658062 
658062 
658062 
658062 
658062 
658062 
453171

2339853250
1813735
class
111111

2339853250
2211325
111111
family

2339853250
2004797
genus
111111

111111
species
2339853250
 1855912 

1855912 
1855912 
1855912 
1855912 
1855912 
1855912 

class
11111
1562566
31131

458032
31131
11111
genus

11111
species

458033 
458033 
458033 

458033 
458033 
 458033 
31131

221
200940
phylum
111

111
class
67799
221

order
111
221
188710

221
188711
111
family

241192
2
1
genus

species
1
2


171695 
 171695 

genus
11
1740
21

11
species
21
 1741 


1741 


1741 

12121011138
phylum
200795
765741458686

class
232332
301297
212313132142

670486
533251
genus
121121


1839801 
1839801 
1839801 
1839801 
1839801 
1839801 
 1839801 
513231
species
111111

22


552810 


552810 
 552810 
11
species

162010111641
1202465
111211
order

1202464
162010111641
111211
family

genus
111211
162010111641
61434

species
1


1522671 
 1522671 
1

111111
species
162010101641

61435 
61435 
61435 
61435 
61435 
61435 
 61435 

137672013
1382928
111111
class

111111
order
1382929
137672013

1382930
137672013
family
111111

137672013
1988031
111111
genus

137672013

1806508 
1806508 
1806508 
1806508 
1806508 
1806508 
 1806508 
111111
species

3121241
292625
321121
class

292629
3121241
order
321121

family
321121
3121241
292628

2019482
12
genus
11

11
species
 1986204 

1986204 


1986204 
12

genus
111
233189
1112

111
species
1112
 167964 

167964 
167964 


167964 

species
11111

1889813 
1889813 
1889813 

1889813 
1889813 
 1889813 
11121

class
222121
189775
2842188

order
1111
12211
189776

12211
189777
1111
family

12211
499
1111
genus

12211

500 
500 
500 

500 
 500 
1111
species

111111
subclass
1621178
85000

111111
order
85001
1621178

1621178
255728
111111
suborder

1621178
85002
family
111111

1621178
2056
genus
111111

111111
species
 2057 

2057 
2057 
2057 
2057 
2057 
2057 
1621178

10118173013
32061
343442
class

343442
order
32064
10118173013

1508595
7873128
222221
suborder

1508635
7873128
family
222221

genus
222221
120961
7873128

species
111111
673138
 120962 

120962 
120962 
120962 
120962 
120962 
120962 

11429
 357808 

357808 
357808 
357808 
357808 
357808 
species
11111

suborder
121221
1508594
33114185

family
121221
33114185
1106

1107
33114185
121221
genus

113
 152260 


152260 

152260 
152260 
111
species


1108 
1108 
1108 
1108 
1108 
1108 
 1108 
32113155
species
111111

class
11111
111539
475962

111539
475963
11111
order

family
11111
475964
111539

11111
genus
233191
111539


133453 

133453 
133453 
133453 
133453 
 133453 
111539
species
11111

117251119549469
203691
252224141715
phylum

203692
117251119549469
252224141715
class

464858368044
136
order
19141581110

141518742
1643685
846332
family

332122
genus
138
41410222

2511
 40834 

40834 
40834 


40834 
40834 
species
1111

 44449 

44449 
44449 
44449 
44449 
44449 
17821
11111
species

1
 141 

141 
1
species

 47466 


47466 
47466 


47466 
221
species
111

64895
101852
51421
genus

species
11111
41332

29518 
29518 
29518 
29518 
29518 
 29518 

species
11
22
 29519 


29519 
29519 

12

664662 

664662 
 664662 
species
11

1

1674146 
 1674146 
1
species

11
species

139 

139 
 139 
21

species
1
2
 62088 

62088 

137
323340297642
11109588
family

genus
1
1616789
4

 1307761 


1307761 
4
species
1

146
7355413
212122
genus

species
1111
41212

46355 

46355 

46355 
46355 
 46355 

 154 

154 
154 
154 
154 
154 
154 
334521
species
111111

genus
222232
411201165
399320


1131703 

1131703 
1131703 
1131703 
1131703 
 1131703 
11124
11111
species


1131707 


1131707 
 1131707 
13
species
11

310191011

273376 
273376 
273376 
273376 
273376 
273376 
 273376 
111111
species

genus
774234
211911136624
157

18211

88058 
88058 
88058 

88058 
88058 
 88058 
species
11111

1


160 
 160 
species
1

11
species

221027 


221027 
 221027 
11

111111
species
 1539298 

1539298 
1539298 
1539298 
1539298 
1539298 
1539298 
53455610

11
 409322 

409322 
409322 
11
species

1
 215591 


215591 
species
1

species
1


53435 
 53435 
1

species
111111
9348912
 81028 

81028 
81028 
81028 
81028 
81028 
81028 


150829 
 150829 
3
species
1

11
species
12

158 
158 
 158 

order
244222
1643686
181120322

family
244222
143786
181120322

genus
244222
181120322
29521

species
111
121


84378 
84378 

84378 
 84378 

105721

159 
159 
159 
159 

159 
 159 
species
11111


84377 
84377 
 84377 
36
11
species


52584 
52584 
52584 
52584 
52584 
52584 
 52584 
825111
111111
species

family
445443
5319241151223
170

genus
111
338321
321

321

29510 

29510 


29510 
 29510 
111
species

171
5019239151222
344442
genus

7273


174 
174 
174 
174 
 174 
1111
species

species
111111
1191255
 28452 

28452 
28452 
28452 
28452 
28452 
28452 

11111
species
4123442
 173 

173 
173 
173 
173 
173 

3553322217

172 
172 
172 
172 
172 
172 
 172 
111111
species

488474481474485476
phylum
201174
121027130671510792019012752638030

1
species
4
 1848255 

1848255 

13912101311
class
84998
8039467110568

747464
order
16916244611
84999

7543261
84107
family
323231

genus
1111
1111
33870

1111
species

33871 
33871 
33871 

33871 
 33871 
1111

species
1111
41216
 1531429 

1531429 

1531429 
1531429 
1531429 

genus
111111
242191
102106

species
111111

74426 
74426 
74426 
74426 
74426 
74426 
 74426 
242191

424233
family
1643824
9412212010

1380
11
genus
11

species
11
11

1382 

1382 
 1382 

323233
genus
133925
8411212010

species
111111
 133926 

133926 
133926 
133926 
133926 
133926 
133926 
114366

635122

1805478 
1805478 
1805478 

1805478 
1805478 
 1805478 
11111
species

121822

712411 

712411 
712411 
712411 
712411 
 712411 
species
11111

655677
order
643030475957
1643822

1643826
643030475957
655677
family

genus
222222
14137131916
644652

7853127

1841863 
1841863 
1841863 
1841863 
1841863 
1841863 
 1841863 
111111
species


1335613 
1335613 
1335613 
1335613 
1335613 
1335613 
 1335613 
7521079
species
111111

genus
111111
84108
933869


84110 
84110 
84110 
84110 
84110 
84110 
 84110 
933869
species
111111

19333
447020
genus
1111

species
1111

446660 


446660 
446660 
446660 
 446660 
19333

221420233027
84111
genus
222222

species
111111
201319222625

84112 
84112 
84112 
84112 
84112 
84112 
 84112 

111111
species
 502558 

502558 
502558 
502558 
502558 
502558 
502558 
211142

79603
2
genus
1

1
species
2
 79604 


79604 

genus
1
1
84162

species
1
 84163 


84163 
1

221222
class
1612710131718
84995

84996
1612710131718
221222
order

1612710131718
84997
family
221222

221222
genus
1612710131718
42255

1502410111314

49319 
49319 
49319 
49319 
49319 
49319 
 49319 
species
111111

 42256 

42256 
42256 

42256 
42256 
42256 
113244
11111
species

1


1848754 
 1848754 
1
species

class
111111
84992
314222

314222
84993
order
111111

84994
314222
111111
family

53634
314222
genus
111111

314222
 53635 

53635 
53635 
53635 
53635 
53635 
53635 
111111
species

121
 1848755 


1848755 
1848755 

1848755 
111
species

120741130580509802009112733837889
1760
class
470460464460467461

111111
order
622452
14141653018975

14141653018975
83778
111111
family

genus
111111
14141653018975
33981

111111
species

131568 
131568 
131568 
131568 
131568 
131568 
 131568 
14141653018975

909090909090
order
7283274430612291109104108
85011

7283274430612291109104108
2062
family
909090909090

307144137141535165
2063
genus
444444

111111
species
9339453717845

2018025 
2018025 
2018025 
2018025 
2018025 
2018025 
 2018025 

624027559645
 1894 

1894 
1894 
1894 
1894 
1894 
1894 
111111
species

111111
species
533131219620

68173 
68173 
68173 
68173 
68173 
68173 
 68173 

 2066 

2066 
2066 
2066 
2066 
2066 
2066 
9934342816555
111111
species

genus
868686868686
6976260029242150103753943
1883

111111
species
572821157733
 2049881 

2049881 
2049881 
2049881 
2049881 
2049881 
2049881 

111111
species
9340221513425
 1849967 

1849967 
1849967 
1849967 
1849967 
1849967 
1849967 


1964449 
1964449 
1964449 
1964449 
1964449 
1964449 
 1964449 
712135159527
111111
species

species
111111
6516211310034

1837283 
1837283 
1837283 
1837283 
1837283 
1837283 
 1837283 

species
111111
13238524520076
 29303 

29303 
29303 
29303 
29303 
29303 
29303 

 47716 

47716 
47716 
47716 
47716 
47716 
47716 
601936279156
species
111111

species
111111

1971 
1971 
1971 
1971 
1971 
1971 
 1971 
672131267434


1437453 
1437453 
1437453 
1437453 
1437453 
1437453 
 1437453 
6721291911332
111111
species

592019148232
 1642299 

1642299 
1642299 
1642299 
1642299 
1642299 
1642299 
111111
species

111111
species
542415337523
 1827580 

1827580 
1827580 
1827580 
1827580 
1827580 
1827580 

111111
species

1961713 
1961713 
1961713 
1961713 
1961713 
1961713 
 1961713 
492022159039

species
111111

1616117 
1616117 
1616117 
1616117 
1616117 
1616117 
 1616117 
7418321111751

5832322610354
 362257 

362257 
362257 
362257 
362257 
362257 
362257 
species
111111

111111
species
 1783515 

1783515 
1783515 
1783515 
1783515 
1783515 
1783515 
7529333410961

111111
species group
1852274
26516812594515142

species
111111
26516812594515142
 1888 

1888 
1888 
1888 
1888 
1888 
1888 

629295
23110513371453108
333333
species subgroup


1911 
1911 
1911 
1911 
1911 
1911 
 1911 
4821381511229
species
111111

species
111111
 1908 

1908 
1908 
1908 
1908 
1908 
1908 
10239673321853

8145282312326

68179 
68179 
68179 
68179 
68179 
68179 
 68179 
species
111111

462122209272

206662 
206662 
206662 
206662 
206662 
206662 
 206662 
111111
species


1535768 
1535768 
1535768 
1535768 
1535768 
1535768 
 1535768 
691627129528
111111
species

5524333211826

2135430 
2135430 
2135430 
2135430 
2135430 
2135430 
 2135430 
species
111111

591846189243
 1661694 

1661694 
1661694 
1661694 
1661694 
1661694 
1661694 
111111
species

species
111111

565560 
565560 
565560 
565560 
565560 
565560 
 565560 
802024218835

111111
species
5514331810544
 862751 

862751 
862751 
862751 
862751 
862751 
862751 

9134312014757

1930 
1930 
1930 
1930 
1930 
1930 
 1930 
111111
species

111111
species

1751294 
1751294 
1751294 
1751294 
1751294 
1751294 
 1751294 
833526269121

species
111111
 1901 

1901 
1901 
1901 
1901 
1901 
1901 
53121166630

541921207422

1690221 
1690221 
1690221 
1690221 
1690221 
1690221 
 1690221 
111111
species

7426361110038

1914 
1914 
1914 
1914 
1914 
1914 
 1914 
111111
species


2202000 
2202000 
2202000 
2202000 
2202000 
2202000 
 2202000 
552821199524
111111
species

species
111111
831826267942

2059884 
2059884 
2059884 
2059884 
2059884 
2059884 
 2059884 

57191988120
 1038928 

1038928 
1038928 
1038928 
1038928 
1038928 
1038928 
111111
species

 1889 

1889 
1889 
1889 
1889 
1889 
1889 
13057565020460
111111
species

111111
species

68202 
68202 
68202 
68202 
68202 
68202 
 68202 
8512123169997

species
111111
 408015 

408015 
408015 
408015 
408015 
408015 
408015 
541631144220


68249 
68249 
68249 
68249 
68249 
68249 
 68249 
781318198123
111111
species

832326319871

1916 
1916 
1916 
1916 
1916 
1916 
 1916 
111111
species

species
111111

68192 
68192 
68192 
68192 
68192 
68192 
 68192 
582326248444


1915 
1915 
1915 
1915 
1915 
1915 
 1915 
8033263012275
111111
species

111111
species
964527309868

1355015 
1355015 
1355015 
1355015 
1355015 
1355015 
 1355015 

2141049169384156
 54571 

54571 
54571 
54571 
54571 
54571 
54571 
111111
species


2174846 
2174846 
2174846 
2174846 
2174846 
2174846 
 2174846 
512230139543
111111
species

species
111111
592534129128

1649184 
1649184 
1649184 
1649184 
1649184 
1649184 
 1649184 


1561022 
1561022 
1561022 
1561022 
1561022 
1561022 
 1561022 
10724322612860
species
111111


68214 
68214 
68214 
68214 
68214 
68214 
 68214 
571735147731
species
111111

species
111111
 47763 

47763 
47763 
47763 
47763 
47763 
47763 
222728780296171

111111
species

553510 
553510 
553510 
553510 
553510 
553510 
 553510 
9242301912541

111111
species
 1940 

1940 
1940 
1940 
1940 
1940 
1940 
6432211911436

6024314711743

33903 
33903 
33903 
33903 
33903 
33903 
 33903 
species
111111

111111
species
811427816027

1736046 
1736046 
1736046 
1736046 
1736046 
1736046 
 1736046 

111111
species
 75293 

75293 
75293 
75293 
75293 
75293 
75293 
402413146314

111111
species
672734148828

2094021 
2094021 
2094021 
2094021 
2094021 
2094021 
 2094021 

111111
species

1950 
1950 
1950 
1950 
1950 
1950 
 1950 
481319138519

111111
species
 146923 

146923 
146923 
146923 
146923 
146923 
146923 
5420392410927


92644 
92644 
92644 
92644 
92644 
92644 
 92644 
711432156518
111111
species

 379067 

379067 
379067 
379067 
379067 
379067 
379067 
5419275310732
species
111111

431717106844
 193462 

193462 
193462 
193462 
193462 
193462 
193462 
111111
species

244777370337129

68570 
68570 
68570 
68570 
68570 
68570 
 68570 
species
111111


1912 
1912 
1912 
1912 
1912 
1912 
 1912 
324140140133538190
species
111111

5316321610725

42684 
42684 
42684 
42684 
42684 
42684 
 42684 
111111
species

species
111111

1907 
1907 
1907 
1907 
1907 
1907 
 1907 
9139251010951

501925188923

285473 
285473 
285473 
285473 
285473 
285473 
 285473 
111111
species

111111
species
6223172110030
 465541 

465541 
465541 
465541 
465541 
465541 
465541 

species
111111
833226296729

1935 
1935 
1935 
1935 
1935 
1935 
 1935 

111111
species

1841249 
1841249 
1841249 
1841249 
1841249 
1841249 
 1841249 
8525333012687

species
111111
 1969 

1969 
1969 
1969 
1969 
1969 
1969 
177507750246108

 1262452 

1262452 
1262452 
1262452 
1262452 
1262452 
1262452 
66272789123
111111
species

species
111111
88201597725
 1109743 

1109743 
1109743 
1109743 
1109743 
1109743 
1109743 

 1984801 

1984801 
1984801 
1984801 
1984801 
1984801 
1984801 
7126182410838
111111
species

species
111111
775533129127
 1972846 

1972846 
1972846 
1972846 
1972846 
1972846 
1972846 

species
111111
 1885 

1885 
1885 
1885 
1885 
1885 
1885 
602727169257

species
111111
50182164838
 67267 

67267 
67267 
67267 
67267 
67267 
67267 

 1495638 

1495638 
1495638 
1495638 
1495638 
1495638 
1495638 
713527309725
species
111111

111111
species
7727252011127

234612 
234612 
234612 
234612 
234612 
234612 
 234612 

111111
species
 42239 

42239 
42239 
42239 
42239 
42239 
42239 
8013301512935

 1169025 

1169025 
1169025 
1169025 
1169025 
1169025 
1169025 
762131147424
111111
species

 68280 

68280 
68280 
68280 
68280 
68280 
68280 
572425167635
111111
species

species
111111
462022177843

38300 
38300 
38300 
38300 
38300 
38300 
 38300 

species
111111

1902 
1902 
1902 
1902 
1902 
1902 
 1902 
9218262512258


1265601 
1265601 
1265601 
1265601 
1265601 
1265601 
 1265601 
7120252811931
111111
species


1725411 
1725411 
1725411 
1725411 
1725411 
1725411 
 1725411 
8530292211750
111111
species

 444103 

444103 
444103 
444103 
444103 
444103 
444103 
8939351610527
species
111111

species
111111
 1812480 

1812480 
1812480 
1812480 
1812480 
1812480 
1812480 
563223448029

6136341710829
 164348 

164348 
164348 
164348 
164348 
164348 
164348 
111111
species


2184053 
2184053 
2184053 
2184053 
2184053 
2184053 
 2184053 
381317288422
species
111111

 1926 

1926 
1926 
1926 
1926 
1926 
1926 
572220129823
species
111111

282828282828
order
218386797669133931253
85010

2070
218386797669133931253
family
282828282828

7513213732511238477
1813
666666
genus

111111
species
12041524117850
 31958 

31958 
31958 
31958 
31958 
31958 
31958 

4313171711036

1911175 
1911175 
1911175 
1911175 
1911175 
1911175 
 1911175 
111111
species

species
111111
642036158231
 1896961 

1896961 
1896961 
1896961 
1896961 
1896961 
1896961 

 33910 

33910 
33910 
33910 
33910 
33910 
33910 
382165180129653270
111111
species

111111
species
805561309039
 1814 

1814 
1814 
1814 
1814 
1814 
1814 

111111
species
6227271912551
 208439 

208439 
208439 
208439 
208439 
208439 
208439 

111111
genus
2029
541125246619


860235 
860235 
860235 
860235 
860235 
860235 
 860235 
541125246619
species
111111

genus
111111
861631349262
43356

111111
species

43357 
43357 
43357 
43357 
43357 
43357 
 43357 
861631349262

genus
111111
674734
7924332114033

species
111111
7924332114033
 1653480 

1653480 
1653480 
1653480 
1653480 
1653480 
1653480 

1851
27131593915
111111
genus

111111
species
27131593915

1852 
1852 
1852 
1852 
1852 
1852 
 1852 

genus
222222
15671824432084
40566

species
111111
7628402312737
 42197 

42197 
42197 
42197 
42197 
42197 
42197 

111111
species
8043422119347
 40567 

40567 
40567 
40567 
40567 
40567 
40567 

2071
5851441812054
111111
genus


103731 
103731 
103731 
103731 
103731 
103731 
 103731 
5851441812054
111111
species

1835
9533313416142
111111
genus

9533313416142
 1836 

1836 
1836 
1836 
1836 
1836 
1836 
111111
species

genus
777777
1847
544208226149732273

111111
species

1096856 
1096856 
1096856 
1096856 
1096856 
1096856 
 1096856 
922933168839

species
111111
682222198733

1688404 
1688404 
1688404 
1688404 
1688404 
1688404 
 1688404 

species
111111
 1641402 

1641402 
1641402 
1641402 
1641402 
1641402 
1641402 
753444218838

species
111111
 445576 

445576 
445576 
445576 
445576 
445576 
445576 
8133311911446

species
111111

240495 
240495 
240495 
240495 
240495 
240495 
 240495 
10225372414130

111111
species
622533179632

1690815 
1690815 
1690815 
1690815 
1690815 
1690815 
 1690815 


1096868 
1096868 
1096868 
1096868 
1096868 
1096868 
 1096868 
6440263311855
species
111111

165301
9649363114772
genus
111111

species
111111
 1586287 

1586287 
1586287 
1586287 
1586287 
1586287 
1586287 
9649363114772

142577
25148114517
111111
genus

species
111111
 530584 

530584 
530584 
530584 
530584 
530584 
530584 
25148114517

212567265293105
65496
genus
555555


1612552 
1612552 
1612552 
1612552 
1612552 
1612552 
 1612552 
641318146749
species
111111


2072503 
2072503 
2072503 
2072503 
2072503 
2072503 
 2072503 
401316206819
species
111111

species
111111
 1612551 

1612551 
1612551 
1612551 
1612551 
1612551 
1612551 
4181095913

species
111111
421516124511
 1470176 

1470176 
1470176 
1470176 
1470176 
1470176 
1470176 


340345 
340345 
340345 
340345 
340345 
340345 
 340345 
25712105413
species
111111

483200141116737264
2037
order
151413141514

151413141514
family
483200141116737264
2049

11111
genus
23111
2050

23111

2051 
2051 

2051 
2051 
2051 
 2051 
11111
species

111111
genus
28263
11451192

species
111111
11451192
 28264 

28264 
28264 
28264 
28264 
28264 
28264 

221122
genus
7213127
1653174

 59505 

59505 
59505 


59505 
59505 
3163
species
1111

 2171980 

2171980 
2171980 
2171980 
2171980 
2171980 
2171980 
411364
111111
species

111111
genus
643124158369
1069494

111111
species
643124158369

1661 
1661 
1661 
1661 
1661 
1661 
 1661 

genus
1091010109
1654
39916011196622185

 1960083 

1960083 
1960083 
1960083 
1960083 
1960083 
1960083 
51121098313
111111
species


52773 
52773 
52773 
52773 
52773 
52773 
 52773 
10835183
species
111111

species
111111

2081702 
2081702 
2081702 
2081702 
2081702 
2081702 
 2081702 
2712138228

111111
species

2079536 
2079536 
2079536 
2079536 
2079536 
2079536 
 2079536 
591412115114

species
111111
 111015 

111015 
111015 
111015 
111015 
111015 
111015 
8836242019648

 1912795 

1912795 

1912795 
1912795 
1912795 
1224
species
1111

502210104319

1851395 
1851395 
1851395 
1851395 
1851395 
1851395 
 1851395 
species
111111

 1852377 

1852377 
1852377 
1852377 
1852377 
1852377 
1852377 
1196582
species
111111

species
111111
482213128324

712122 
712122 
712122 
712122 
712122 
712122 
 712122 

5425181411454

544580 
544580 
544580 
544580 
544580 
544580 
 544580 
species
111111

1643684
10448402212667
111111
order

111111
family
10448402212667
85031

53460
10448402212667
111111
genus

111111
species
10448402212667
 53461 

53461 
53461 
53461 
53461 
53461 
53461 

85007
10006732151173686128215707
order
154151152148153149

family
545353505350
1653
12646235575151455656

545353505350
genus
12646235575151455656
1716

8691
 702967 

702967 
702967 


702967 
702967 
species
1111

species
111111
391510135521

1230998 
1230998 
1230998 
1230998 
1230998 
1230998 
 1230998 


258224 
258224 
258224 

258224 
258224 
 258224 
32154
species
11111

 152794 

152794 
152794 
152794 
152794 
152794 
152794 
13952910
species
111111

species
111111
36101672614
 191493 

191493 
191493 
191493 
191493 
191493 
191493 

 161899 

161899 
161899 
161899 

161899 
142130
species
1111

1111
species
4119

161895 

161895 
161895 
161895 
 161895 

 1719 

1719 
1719 
1719 
1719 
1719 
1719 
13065675414676
111111
species

111111
species
 43771 

43771 
43771 
43771 
43771 
43771 
43771 
492033165744


1408191 
1408191 
1408191 
1408191 

1408191 
 1408191 
41221
species
11111

11322319

161879 
161879 
161879 
161879 
161879 
161879 
 161879 
111111
species

species
111111
32181573413

38305 
38305 
38305 
38305 
38305 
38305 
 38305 

11343405
 38301 

38301 
38301 
38301 
38301 
38301 
38301 
111111
species

111111
species
22171611449

1223514 
1223514 
1223514 
1223514 
1223514 
1223514 
 1223514 

433312

35757 
35757 
35757 
35757 
35757 
35757 
 35757 
species
111111

species
111111
 108486 

108486 
108486 
108486 
108486 
108486 
108486 
1021042825

species
111111
 2080740 

2080740 
2080740 
2080740 
2080740 
2080740 
2080740 
1511811307

2214612304

575200 
575200 
575200 
575200 
575200 
575200 
 575200 
species
111111

 28028 

28028 
28028 
28028 
28028 
28028 
28028 
5715233
111111
species

species
111111

1721 
1721 
1721 
1721 
1721 
1721 
 1721 
7851112


89154 
89154 
89154 
89154 
89154 
89154 
 89154 
261348911
species
111111


1737425 
1737425 
1737425 
1737425 
1737425 
1737425 
 1737425 
911811215
species
111111

111111
species
8231042
 42817 

42817 
42817 
42817 
42817 
42817 
42817 

111111
species
 1705 

1705 
1705 
1705 
1705 
1705 
1705 
19812102228

111111
species

156978 
156978 
156978 
156978 
156978 
156978 
 156978 
4215713386

111111
species
33229114619
 65058 

65058 
65058 
65058 
65058 
65058 
65058 

species
111111
231469195

161896 
161896 
161896 
161896 
161896 
161896 
 161896 

11
species
 571915 


571915 
571915 
21

834361
 1050174 

1050174 
1050174 
1050174 
1050174 
1050174 
1050174 
111111
species

111
species

35755 
35755 
35755 
 35755 
1219

species
111111
852133

160386 
160386 
160386 
160386 
160386 
160386 
 160386 

species
11111
51216
 92706 

92706 
92706 
92706 
92706 
92706 

17621194
 1072256 

1072256 
1072256 
1072256 
1072256 
1072256 
1072256 
111111
species

1510621510
 203263 

203263 
203263 
203263 
203263 
203263 
203263 
species
111111

6652135

1724 
1724 
1724 
1724 
1724 
1724 
 1724 
111111
species

133747611311359

1718 
1718 
1718 
1718 
1718 
1718 
 1718 
111111
species

11111
species
27632
 1231000 

1231000 
1231000 
1231000 

1231000 
1231000 


191610 
191610 
191610 
191610 
191610 
191610 
 191610 
7134163
species
111111


401472 
401472 
401472 
401472 
401472 
401472 
 401472 
197105146
species
111111

31125156819
 349751 

349751 
349751 
349751 
349751 
349751 
349751 
species
111111

species
111111
 1404244 

1404244 
1404244 
1404244 
1404244 
1404244 
1404244 
16829173

species
111111
 169292 

169292 
169292 
169292 
169292 
169292 
169292 
135252521

 1121358 

1121358 
1121358 
1121358 
1121358 
1121358 
1121358 
36151384121
111111
species

361419115730

1727 
1727 
1727 
1727 
1727 
1727 
 1727 
species
111111

31111144030

2079535 
2079535 
2079535 
2079535 
2079535 
2079535 
 2079535 
111111
species

8637122

1697 
1697 
1697 
1697 
1697 
1697 
 1697 
111111
species

111111
species
211312132212

146827 
146827 
146827 
146827 
146827 
146827 
 146827 

species
111111
13542315

136857 
136857 
136857 
136857 
136857 
136857 
 136857 

111111
species
23336512

187491 
187491 
187491 
187491 
187491 
187491 
 187491 

species
111111
 1717 

1717 
1717 
1717 
1717 
1717 
1717 
644532127123

111111
species
 1487956 

1487956 
1487956 
1487956 
1487956 
1487956 
1487956 
1534461

111111
species
391416145122
 225326 

225326 
225326 
225326 
225326 
225326 
225326 

 38289 

38289 
38289 
38289 
38289 
38289 
38289 
412918173215
111111
species

species
111111
211181

1652495 
1652495 
1652495 
1652495 
1652495 
1652495 
 1652495 

species
111111
441718303110
 43770 

43770 
43770 
43770 
43770 
43770 
43770 

111111
family
316606
2572183510

2572183510
286801
111111
genus

2572183510

286802 
286802 
286802 
286802 
286802 
286802 
 286802 
111111
species

family
777777
85026
619433214131495290

619433214131495290
2053
genus
777777


84595 
84595 
84595 
84595 
84595 
84595 
 84595 
462617136243
111111
species

111111
species
21917258365771

2054 
2054 
2054 
2054 
2054 
2054 
 2054 

species
111111
 1004901 

1004901 
1004901 
1004901 
1004901 
1004901 
1004901 
322115154423

species
111111
 1136941 

1136941 
1136941 
1136941 
1136941 
1136941 
1136941 
511919174338

species
111111
 2055 

2055 
2055 
2055 
2055 
2055 
2055 
161116543015977

species
111111

2059875 
2059875 
2059875 
2059875 
2059875 
2059875 
 2059875 
382621125913

72533087125
 337191 

337191 
337191 
337191 
337191 
337191 
337191 
species
111111

222222
family
85028
136697047161115

2060
136697047161115
222222
genus

835451298352

57704 
57704 
57704 
57704 
57704 
57704 
 57704 
species
111111

111111
species
531519187863
 2061 

2061 
2061 
2061 
2061 
2061 
2061 

family
323232323232
85025
280433711796112439381516

777777
genus
1817
561191230239819396

 135487 

135487 
135487 
135487 
135487 
135487 
135487 
7823303513057
species
111111

231515103611
 37330 

37330 
37330 
37330 
37330 
37330 
37330 
species
111111

 37329 

37329 
37329 
37329 
37329 
37329 
37329 
12938593213560
species
111111

species
111111
120244853208113
 37332 

37332 
37332 
37332 
37332 
37332 
37332 

species
111111
11856456315096

37326 
37326 
37326 
37326 
37326 
37326 
 37326 

461620218136
 455432 

455432 
455432 
455432 
455432 
455432 
455432 
species
111111

 2213200 

2213200 
2213200 
2213200 
2213200 
2213200 
2213200 
471913257923
111111
species

1827
22433180156688531191120
252525252525
genus

57632796514
 1807790 

1807790 
1807790 
1807790 
1807790 
1807790 
1807790 
111111
species

species
111111
 191292 

191292 
191292 
191292 
191292 
191292 
191292 
6017153010245

111111
species
522416195928
 1805827 

1805827 
1805827 
1805827 
1805827 
1805827 
1805827 

 1045808 

1045808 
1045808 
1045808 
1045808 
1045808 
1045808 
205425294310
species
111111


1653479 
1653479 
1653479 
1653479 
1653479 
1653479 
 1653479 
1944824316740782
species
111111

species
111111
 935199 

935199 
935199 
935199 
935199 
935199 
935199 
381515175224

382227447775351105
 1833 

1833 
1833 
1833 
1833 
1833 
1833 
species
111111

 38310 

38310 
38310 
38310 
38310 
38310 
38310 
40132395314
species
111111

species
111111
5633212810246

132919 
132919 
132919 
132919 
132919 
132919 
 132919 

451221154714

1570939 
1570939 
1570939 
1570939 
1570939 
1570939 
 1570939 
111111
species

111111
species
 43767 

43767 
43767 
43767 
43767 
43767 
43767 
106513823194112

species
111111
621519305625
 1500843 

1500843 
1500843 
1500843 
1500843 
1500843 
1500843 

species
111111
654822125811

1723645 
1723645 
1723645 
1723645 
1723645 
1723645 
 1723645 

 37919 

37919 
37919 
37919 
37919 
37919 
37919 
244838278264122
111111
species

species
111111
431012208322
 1829 

1829 
1829 
1829 
1829 
1829 
1829 

1906425714840373

1828 
1828 
1828 
1828 
1828 
1828 
 1828 
species
111111


334542 
334542 
334542 
334542 
334542 
334542 
 334542 
33492162419
species
111111


1653478 
1653478 
1653478 
1653478 
1653478 
1653478 
 1653478 
621519136959
species
111111

12126153320
 679318 

679318 
679318 
679318 
679318 
679318 
679318 
111111
species

 1830 

1830 
1830 
1830 
1830 
1830 
1830 
19564673925595
111111
species

species
111111
9846374513869
 103816 

103816 
103816 
103816 
103816 
103816 
103816 

species
111111
3999379618
 1727214 

1727214 
1727214 
1727214 
1727214 
1727214 
1727214 

 1033922 

1033922 
1033922 
1033922 
1033922 
1033922 
1033922 
423627126431
111111
species

441317146331
 1990687 

1990687 
1990687 
1990687 
1990687 
1990687 
1990687 
111111
species

111111
species
642222237341
 1564114 

1564114 
1564114 
1564114 
1564114 
1564114 
1564114 

555555
family
38834015878306163
85029

555555
genus
38834015878306163
37914

43272265517

2052657 
2052657 
2052657 
2052657 
2052657 
2052657 
 2052657 
111111
species

442725196915

139021 
139021 
139021 
139021 
139021 
139021 
 139021 
111111
species


546160 
546160 
546160 
546160 
546160 
546160 
 546160 
896933147242
species
111111


499555 
499555 
499555 
499555 
499555 
499555 
 499555 
29261384414
species
111111

111111
species
18319165316675

712270 
712270 
712270 
712270 
712270 
712270 
 712270 

11021764271229
1847725
111111
genus

11021764271229

1528099 
1528099 
1528099 
1528099 
1528099 
1528099 
 1528099 
species
111111

525051505251
family
1762
466022612237175664192928

1763
290715231484112737891759
312930293130
genus

111111
species

29311 
29311 
29311 
29311 
29311 
29311 
 29311 
3262157

342012293016
 1809 

1809 
1809 
1809 
1809 
1809 
1809 
species
111111

species
111111

1879023 
1879023 
1879023 
1879023 
1879023 
1879023 
 1879023 
391819145224

species group
111111
1915134475
2249310

111111
species

722731 
722731 
722731 
722731 
722731 
722731 
 722731 
1915134475

111111
species
35119131811
 1561223 

1561223 
1561223 
1561223 
1561223 
1561223 
1561223 


1920667 
1920667 
1920667 
1920667 
1920667 
1920667 
 1920667 
25273355430
species
111111

1558586

261524 
261524 
261524 
261524 
261524 
261524 
 261524 
111111
species

111111
species
 212767 

212767 
212767 
212767 
212767 
212767 
212767 
271413132814

111111
species

1545728 
1545728 
1545728 
1545728 
1545728 
1545728 
 1545728 
381313144510

12666316164201577689
120793
species group
666666

 222805 

222805 
222805 
222805 
222805 
222805 
222805 
34827018177248153
111111
species

236115114107357130

1767 
1767 
1767 
1767 
1767 
1767 
 1767 
111111
species


701042 
701042 
701042 
701042 
701042 
701042 
 701042 
3149113810
species
111111

 1764 

1764 
1764 
1764 
1764 
1764 
1764 
605222292187863355
111111
species

species
111111
2113863328

1138383 
1138383 
1138383 
1138383 
1138383 
1138383 
 1138383 

25712323813

339268 
339268 
339268 
339268 
339268 
339268 
 339268 
111111
species

2289112617

1570328 
1570328 
1570328 
1570328 
1570328 
1570328 
 1570328 
species
111111

77643
10235785544301399676
423243
species group

species
1111

78331 

78331 

78331 
78331 
 78331 
1222

11
species
41

33894 


33894 
 33894 

111111
species

1773 
1773 
1773 
1773 
1773 
1773 
 1773 
10045725454251368666

species
111111
 1765 

1765 
1765 
1765 
1765 
1765 
1765 
14675288

 189918 

189918 
189918 
189918 
189918 
189918 
189918 
431111145516
111111
species

651225245931

2051552 
2051552 
2051552 
2051552 
2051552 
2051552 
 2051552 
species
111111

342422277921

164757 
164757 
164757 
164757 
164757 
164757 
 164757 
111111
species

2481562555

1781 
1781 
1781 
1781 
1781 
1781 
 1781 
species
111111

401215204225
 1768 

1768 
1768 
1768 
1768 
1768 
1768 
species
111111

species
111111
 1682113 

1682113 
1682113 
1682113 
1682113 
1682113 
1682113 
232913114223

32910182616
 1273687 

1273687 
1273687 
1273687 
1273687 
1273687 
1273687 
111111
species


1168287 
1168287 
1168287 
1168287 
1168287 
1168287 
 1168287 
102517107
species
111111

512553
 1769 

1769 
1769 
1769 
1769 
1769 
1769 
111111
species

 482462 

482462 
482462 
482462 
482462 
482462 
482462 
29232337017
species
111111

111111
species
565038227740
 164756 

164756 
164756 
164756 
164756 
164756 
164756 

442120226828
1073531
genus
222222

species
111111
 1788 

1788 
1788 
1788 
1788 
1788 
1788 
20811102611

24139124217
 875328 

875328 
875328 
875328 
875328 
875328 
875328 
species
111111

12342526
697025
genus
111111


639313 
639313 
639313 
639313 
639313 
639313 
 639313 
12342526
111111
species

genus
131313131313
1866885
8903843643391261638

111524845100113

1804 
1804 
1804 
1804 
1804 
1804 
 1804 
111111
species

species
111111
32915202421
 758802 

758802 
758802 
758802 
758802 
758802 
758802 

species
111111
 1795 

1795 
1795 
1795 
1795 
1795 
1795 
241099207

111111
species
 1772 

1772 
1772 
1772 
1772 
1772 
1772 
354135137103543211

111111
species
581724426929
 134601 

134601 
134601 
134601 
134601 
134601 
134601 

species
111111
2426773125
 36814 

36814 
36814 
36814 
36814 
36814 
36814 

111111
species

1771 
1771 
1771 
1771 
1771 
1771 
 1771 
402419126022

452423199339

1800 
1800 
1800 
1800 
1800 
1800 
 1800 
111111
species

species
111111
491423118427
 110539 

110539 
110539 
110539 
110539 
110539 
110539 

species
111111

1810 
1810 
1810 
1810 
1810 
1810 
 1810 
431510209166

331818143015

1797 
1797 
1797 
1797 
1797 
1797 
 1797 
species
111111

111111
species
 1791 

1791 
1791 
1791 
1791 
1791 
1791 
392320196226

 1766 

1766 
1766 
1766 
1766 
1766 
1766 
381711185437
111111
species

8073303652661249497
670516
genus
555555

species
111111
1514334211
 1578165 

1578165 
1578165 
1578165 
1578165 
1578165 
1578165 

112107102
 1774 

1774 
1774 
1774 
1774 
1774 
1774 
species
111111

 36809 

36809 
36809 
36809 
36809 
36809 
36809 
7412823232461073438
111111
species

111111
species
 83262 

83262 
83262 
83262 
83262 
83262 
83262 
31282498614

94513832
 1520670 

1520670 
1520670 
1520670 
1520670 
1520670 
1520670 
species
111111

order
777777
85012
622302276181896360

family
111111
521421194121
2012

genus
111111
2019
521421194121


2020 
2020 
2020 
2020 
2020 
2020 
 2020 
521421194121
111111
species

2004
2091041064632596
222222
family

genus
111111
11773612020158
83681

111111
species
 1909395 

1909395 
1909395 
1909395 
1909395 
1909395 
1909395 
11773612020158

111111
genus
2000
9231452612438

9231452612438
 2001 

2001 
2001 
2001 
2001 
2001 
2001 
species
111111

family
444444
361184149116530243
83676

333333
genus
2013
342174136106505241


53437 
53437 
53437 
53437 
53437 
53437 
 53437 
7622231410633
111111
species

513120196362

280236 
280236 
280236 
280236 
280236 
280236 
 280236 
111111
species

species
111111
 2014 

2014 
2014 
2014 
2014 
2014 
2014 
2151219373336146

83677
19101310252
genus
111111

111111
species
19101310252

2021 
2021 
2021 
2021 
2021 
2021 
 2021 

species
111
212
 1650658 

1650658 


1650658 
1650658 

order
100981001009998
41255110321301867978819515788
85006

475146139121646194
85020
family
777777

4235113
36739
111111
genus

4235113
 1630135 

1630135 
1630135 
1630135 
1630135 
1630135 
1630135 
species
111111

555555
genus
43668
463137134113624190

species
111111

2017484 
2017484 
2017484 
2017484 
2017484 
2017484 
 2017484 
8424221912637

 2017485 

2017485 
2017485 
2017485 
2017485 
2017485 
2017485 
7031151810220
species
111111

 43669 

43669 
43669 
43669 
43669 
43669 
43669 
12630401912452
species
111111

species
111111
 1903186 

1903186 
1903186 
1903186 
1903186 
1903186 
1903186 
7121303113335

111111
species
11231272613946
 1331682 

1331682 
1331682 
1331682 
1331682 
1331682 
1331682 

111111
genus
472568
8723111

8723111
 472569 

472569 
472569 
472569 
472569 
472569 
472569 
111111
species

36019014044414192
145357
family
333333

genus
111111
57499
127965097444


1276 
1276 
1276 
1276 
1276 
1276 
 1276 
127965097444
species
111111

57495
12740461617691
111111
genus

12740461617691

1274 
1274 
1274 
1274 
1274 
1274 
 1274 
111111
species

genus
111111
745364
10654441916457

species
111111
10654441916457
 571913 

571913 
571913 
571913 
571913 
571913 
571913 

genus
111111
13513145
2038

111111
species
 2039 

2039 
2039 
2039 
2039 
2039 
2039 
13513145

111111
family
118312210113835
85019

1696
118312210113835
genus
111111

 1703 

1703 
1703 
1703 
1703 
1703 
1703 
118312210113835
species
111111

10544342591262264489
85016
444444
family

444444
genus
10544342591262264489
1707

species
111111
233944928485115
 1711 

1711 
1711 
1711 
1711 
1711 
1711 

111111
species

1708 
1708 
1708 
1708 
1708 
1708 
 1708 
3601569635898162

species
111111
215955031434112

11 
11 
11 
11 
11 
11 
 11 


2003551 
2003551 
2003551 
2003551 
2003551 
2003551 
 2003551 
246896432447100
species
111111

85021
487212190102707223
family
444444

7239401613338
265976
genus
111111

111111
species
 1758689 

1758689 
1758689 
1758689 
1758689 
1758689 
1758689 
7239401613338

20378512620570
53457
genus
111111

20378512620570

857417 
857417 
857417 
857417 
857417 
857417 
 857417 
species
111111

genus
111111
53357
8932323515853

8932323515853

53358 
53358 
53358 
53358 
53358 
53358 
 53358 
species
111111

12363672521162
267408
genus
111111

111111
species
12363672521162

1658671 
1658671 
1658671 
1658671 
1658671 
1658671 
 1658671 

85017
17747323361683265678
family
555555

222222
genus
592218109631094221
254250

species
111111
2931176534493120
 139208 

139208 
139208 
139208 
139208 
139208 
139208 

111111
species
2991014429601101

372663 
372663 
372663 
372663 
372663 
372663 
 372663 

157920
973439186861720373
genus
222222

111111
species
 1980001 

1980001 
1980001 
1980001 
1980001 
1980001 
1980001 
4912059547876201

111111
species
4822349139844172
 1710 

1710 
1710 
1710 
1710 
1710 
1710 

111111
genus
20975411945184
186188

111111
species

186189 
186189 
186189 
186189 
186189 
186189 
 186189 
20975411945184

313131313130
family
1268
961222711640248034221317

49101428235
1868332
111111
genus

species
111111

556325 
556325 
556325 
556325 
556325 
556325 
 556325 
49101428235

111111
genus
12640221005919
1742992

 43663 

43663 
43663 
43663 
43663 
43663 
43663 
12640221005919
species
111111

genus
222222
1456046308133
32207

 43675 

43675 
43675 
43675 
43675 
43675 
43675 
1184538286621
species
111111

species
111111
2715821512

2047 
2047 
2047 
2047 
2047 
2047 
 2047 

222222
genus
319479920613038
1742989

species
111111
19218541084513
 256701 

256701 
256701 
256701 
256701 
256701 
256701 

species
111111

1933880 
1933880 
1933880 
1933880 
1933880 
1933880 
 1933880 
1272945988525

genus
151515151515
1663
6961134575913741606635

species
111111
26968547310564

1704044 
1704044 
1704044 
1704044 
1704044 
1704044 
 1704044 

111111
species
 1652545 

1652545 
1652545 
1652545 
1652545 
1652545 
1652545 
21227621448934

111111
species
3966121610
 1618207 

1618207 
1618207 
1618207 
1618207 
1618207 
1618207 

1363253909286
 2079227 

2079227 
2079227 
2079227 
2079227 
2079227 
2079227 
111111
species

 1118963 

1118963 
1118963 
1118963 
1118963 
1118963 
1118963 
124463010610029
species
111111

 1806905 

1806905 
1806905 
1806905 
1806905 
1806905 
1806905 
1323453736937
species
111111

species
111111

656366 
656366 
656366 
656366 
656366 
656366 
 656366 
465268311517423877

15051498410733
 37928 

37928 
37928 
37928 
37928 
37928 
37928 
species
111111

species
111111

1771959 
1771959 
1771959 
1771959 
1771959 
1771959 
 1771959 
722226545718

111111
species
 1849032 

1849032 
1849032 
1849032 
1849032 
1849032 
1849032 
16464619316048

17648409811838

1357915 
1357915 
1357915 
1357915 
1357915 
1357915 
 1357915 
species
111111

 290399 

290399 
290399 
290399 
290399 
290399 
290399 
12944387611840
111111
species

111111
species
 1690248 

1690248 
1690248 
1690248 
1690248 
1690248 
1690248 
176497512911149


1494608 
1494608 
1494608 
1494608 
1494608 
1494608 
 1494608 
409140674912147
111111
species

species
111111
121313011910525
 2020486 

2020486 
2020486 
2020486 
2020486 
2020486 
2020486 

57493
600217233238634191
444444
genus

species
111111
17066656524658

72000 
72000 
72000 
72000 
72000 
72000 
 72000 

 71999 

71999 
71999 
71999 
71999 
71999 
71999 
10948393210743
species
111111

239669210518760

446860 
446860 
446860 
446860 
446860 
446860 
 446860 
111111
species

 1702043 

1702043 
1702043 
1702043 
1702043 
1702043 
1702043 
823737369430
species
111111

genus
333333
602163280365366122
1742993

111111
species
1403758729833

361575 
361575 
361575 
361575 
361575 
361575 
 361575 

111111
species
3198016721113950
 85085 

85085 
85085 
85085 
85085 
85085 
85085 

14346558212939

121292 
121292 
121292 
121292 
121292 
121292 
 121292 
111111
species

20463634724569
596707
111111
genus

20463634724569

37927 
37927 
37927 
37927 
37927 
37927 
 37927 
111111
species

58732111877261205
1269
111111
genus

111111
species
58732111877261205

1270 
1270 
1270 
1270 
1270 
1270 
 1270 

19561517
1645
genus
11111

11111
species
19561517

1646 
1646 
1646 
1646 
1646 
 1646 

145360
499719131121267171992285
family
111111

60919
499719131121267171992285
111111
genus

species
111111
499719131121267171992285

60920 
60920 
60920 
60920 
60920 
60920 
 60920 

393739393838
family
218984889903533095923610169
85023

33877
62114112883844374
222222
genus

111111
species
285645133382180

2080742 
2080742 
2080742 
2080742 
2080742 
2080742 
 2080742 

336777750462194

453304 
453304 
453304 
453304 
453304 
453304 
 453304 
111111
species

11543313471562077465771823
33882
131313131313
genus

111111
species
 1714373 

1714373 
1714373 
1714373 
1714373 
1714373 
1714373 
313608353406124

111111
species
 36805 

36805 
36805 
36805 
36805 
36805 
36805 
334889164368103

species
111111
294668346352131
 300019 

300019 
300019 
300019 
300019 
300019 
300019 

111111
species
369758150478149
 2033 

2033 
2033 
2033 
2033 
2033 
2033 

 367477 

367477 
367477 
367477 
367477 
367477 
367477 
56013011875656136
species
111111

species
111111
847178176951065132
 1938334 

1938334 
1938334 
1938334 
1938334 
1938334 
1938334 

244557139283103

84292 
84292 
84292 
84292 
84292 
84292 
 84292 
species
111111


1696072 
1696072 
1696072 
1696072 
1696072 
1696072 
 1696072 
32601054295468020102196
species
111111

328557934365112
 162426 

162426 
162426 
162426 
162426 
162426 
162426 
111111
species

species
111111

1795053 
1795053 
1795053 
1795053 
1795053 
1795053 
 1795053 
67211013768817141

 2103230 

2103230 
2103230 
2103230 
2103230 
2103230 
2103230 
64413415595904109
111111
species

species
111111
398779740712165

1906742 
1906742 
1906742 
1906742 
1906742 
1906742 
 1906742 

111111
species
32801052303173820069222
 1916917 

1916917 
1916917 
1916917 
1916917 
1916917 
1916917 

genus
111111
49811811873700360
110932

 1575 

1575 
1575 
1575 
1575 
1575 
1575 
49811811873700360
111111
species

11111
genus
444132
1434032

species
11111
 1159327 

1159327 

1159327 
1159327 
1159327 
1159327 
444132

286752711
1705353
genus
212222

111111
species
24641174
 1987356 

1987356 
1987356 
1987356 
1987356 
1987356 
1987356 

434107
 708131 

708131 

708131 
708131 
708131 
708131 
species
11111

241455949316147
518733
111111
genus

species
111111

412690 
412690 
412690 
412690 
412690 
412690 
 412690 
241455949316147

genus
555555
1573
204339451426338072415


33014 
33014 
33014 
33014 
33014 
33014 
 33014 
102320826412618151224
species
111111

species
111111
217465332420262
 31964 

31964 
31964 
31964 
31964 
31964 
31964 

111111
species
281415942474271

31963 
31963 
31963 
31963 
31963 
31963 
 31963 

276457434513271
 1874630 

1874630 
1874630 
1874630 
1874630 
1874630 
1874630 
species
111111

111111
species
246546429585387

28447 
28447 
28447 
28447 
28447 
28447 
 28447 

333333
genus
2034
513764357953245993650


69373 
69373 
69373 
69373 
69373 
69373 
 69373 
191625020222819141446
111111
species

111111
species
 1561023 

1561023 
1561023 
1561023 
1561023 
1561023 
1561023 
14141731791331078854

180722019817116071350
 1905847 

1905847 
1905847 
1905847 
1905847 
1905847 
1905847 
111111
species

788251810830
235888
genus
111111

111111
species
788251810830
 2079791 

2079791 
2079791 
2079791 
2079791 
2079791 
2079791 

76634
15627522723174
111111
genus

111111
species
15627522723174
 2079792 

2079792 
2079792 
2079792 
2079792 
2079792 
2079792 

447237
542798031424389
111111
genus


1795630 
1795630 
1795630 
1795630 
1795630 
1795630 
 1795630 
542798031424389
111111
species

111111
genus
142263519159101
337004

111111
species
142263519159101

279828 
279828 
279828 
279828 
279828 
279828 
 279828 

2208810028463250
33886
222222
genus

 33888 

33888 
33888 
33888 
33888 
33888 
33888 
171657822380188
111111
species

111111
species
49232268362

145458 
145458 
145458 
145458 
145458 
145458 
 145458 

111111
genus
227535034315187
1759331

 1619308 

1619308 
1619308 
1619308 
1619308 
1619308 
1619308 
227535034315187
species
111111

529883
922312
222211
genus


535712 
535712 
535712 
535712 
535712 
535712 
 535712 
411212
species
111111

5111

529884 
529884 
529884 
529884 
 529884 
1111
species

69578
40912512663652354
222222
genus

 1978566 

1978566 
1978566 
1978566 
1978566 
1978566 
1978566 
174595723307179
species
111111

 670052 

670052 
670052 
670052 
670052 
670052 
670052 
235666940345175
species
111111

111111
family
19652205
85018

19652205
1862
111111
genus

 1863 

1863 
1863 
1863 
1863 
1863 
1863 
19652205
111111
species

85022
13676392132667
family
111111

13676392132667
43673
111111
genus


43674 
43674 
43674 
43674 
43674 
43674 
 43674 
13676392132667
111111
species

family
222222
125316
3121279153544129

84756
15666402125973
genus
111111


84757 
84757 
84757 
84757 
84757 
84757 
 84757 
15666402125973
species
111111

111111
genus
15661513228556
947525

111111
species
15661513228556

2171623 
2171623 
2171623 
2171623 
2171623 
2171623 
 2171623 

548343
order
17142151412
2039638

family
548343
2162846
17142151412

genus
346142
622681
15141821411

species
111111
847267

1884914 
1884914 
1884914 
1884914 
1884914 
1884914 
 1884914 

species
11111
 1884913 

1884913 
1884913 
1884913 

1884913 
1884913 
21114

1
 573600 


573600 
species
1

11
 1884907 


1884907 
1884907 
11
species

species
1111
 1884905 

1884905 
1884905 
1884905 

1884905 
5866

 1884904 


1884904 

1884904 
21
species
11

2331
2039639
2221
genus

species
1111

1884916 

1884916 
1884916 

1884916 
 1884916 
1121

121
 1884915 

1884915 

1884915 
1884915 
111
species

order
111111
20121410327
622450

622451
20121410327
family
111111

1849
20121410327
genus
111111

species
111111

414996 
414996 
414996 
414996 
414996 
414996 
 414996 
20121410327

order
333333
1643682
362137311172655214

85030
362137311172655214
333333
family

161551306825079
88138
111111
genus

161551306825079
 477641 

477641 
477641 
477641 
477641 
477641 
477641 
111111
species

genus
111111
1860
10337855224355


1861 
1861 
1861 
1861 
1861 
1861 
 1861 
10337855224355
111111
species

111111
genus
38501
9845965216280

species
111111
9845965216280
 138336 

138336 
138336 
138336 
138336 
138336 
138336 

2268112080297127
85013
555555
order

74712
2268112080297127
family
555555

2268112080297127
1854
genus
555555

species
111111
502141215024

298653 
298653 
298653 
298653 
298653 
298653 
 298653 

631321168419

1859 
1859 
1859 
1859 
1859 
1859 
 1859 
111111
species


656024 
656024 
656024 
656024 
656024 
656024 
 656024 
32131573317
species
111111

392434257445

298654 
298654 
298654 
298654 
298654 
298654 
 298654 
111111
species


106370 
106370 
106370 
106370 
106370 
106370 
 106370 
42109115622
111111
species

111111
genus
147067
531714128724

111111
species

2006 
2006 
2006 
2006 
2006 
2006 
 2006 
531714128724

531395
1643683
order
111111

531395
85032
family
111111

genus
111111
531395
28048

531395

28049 
28049 
28049 
28049 
28049 
28049 
 28049 
species
111111

201917202021
order
85004
12275394723971809726

201917202021
family
12275394723971809726
31953

genus
111111
8121115
2701

8121115
 2702 

2702 
2702 
2702 
2702 
2702 
2702 
111111
species

1
genus
2
196081

 78259 


78259 
2
species
1

181715181818
genus
1678
12155374663921784693

 638619 

638619 
638619 
638619 
638619 
638619 
638619 
8431113
111111
species

147913189

1684 
1684 
1684 
1684 
1684 
1684 
 1684 
species
111111

1991672616
 1689 

1689 
1689 
1689 
1689 
1689 
1689 
species
111111

 1683 

1683 
1683 
1683 
1683 
1683 
1683 
29488149
species
111111

111111
species
 35760 

35760 
35760 
35760 
35760 
35760 
35760 
3681863735

111111
species

1680 
1680 
1680 
1680 
1680 
1680 
 1680 
2517996146

111111
species
 158787 

158787 
158787 
158787 
158787 
158787 
158787 
3244102315

 216816 

216816 
216816 
216816 
216816 
216816 
216816 
2601061107241899
111111
species

1991166642332130
 28025 

28025 
28025 
28025 
28025 
28025 
28025 
111111
species

 1691 

1691 

1691 
1691 
1691 
1691 
61152
species
11111

species
111111
9038312417649

1681 
1681 
1681 
1681 
1681 
1681 
 1681 

79177

28026 
28026 

28026 
28026 
28026 
 28026 
11111
species

11111
species
681133
 630129 

630129 
630129 

630129 
630129 
630129 

111111
species
111111

1686 
1686 
1686 
1686 
1686 
1686 
 1686 

411183173158562245
 1685 

1685 
1685 
1685 
1685 
1685 
1685 
111111
species

 1687 

1687 
1687 
1687 
1687 
1687 
1687 
35117131
111111
species

species
11111
633133

33905 
33905 

33905 
33905 
33905 
 33905 

species
111111
631516185420

1694 
1694 
1694 
1694 
1694 
1694 
 1694 

111111
genus
196082
41441426


78258 
78258 
78258 
78258 
78258 
78258 
 78258 
41441426
111111
species

order
111111
9738361816046
414714

111111
family
414877
9738361816046

414878
9738361816046
111111
genus

9738361816046
 304895 

304895 
304895 
304895 
304895 
304895 
304895 
species
111111

85014
57132764613
order
111111

85034
57132764613
111111
family

genus
111111
57132764613
283810

57132764613
 283811 

283811 
283811 
283811 
283811 
283811 
283811 
species
111111

5534710651626637507752598234
85009
order
222121222222

5453710623826328486240247773
31957
family
141313141414

1912217
208914634
111111
genus

111111
species

1750 
1750 
1750 
1750 
1750 
1750 
 1750 
208914634

203133
2116
1111
genus

species
1111
2116
 1871034 

1871034 


1871034 
1871034 
1871034 

555555
genus
72763
27610611161497124

111111
species
 1610493 

1610493 
1610493 
1610493 
1610493 
1610493 
1610493 
431527107218

111111
species
 1332264 

1332264 
1332264 
1332264 
1332264 
1332264 
1332264 
7036191611628

111111
species
3071738822
 2161816 

2161816 
2161816 
2161816 
2161816 
2161816 
2161816 

species
111111
 399497 

399497 
399497 
399497 
399497 
399497 
399497 
672023139428


1909732 
1909732 
1909732 
1909732 
1909732 
1909732 
 1909732 
6628251912728
species
111111

856863498196653295
1743
222222
genus

species
111111
395662307392750

671223 
671223 
671223 
671223 
671223 
671223 
 671223 

species
111111
461201191157626245

1744 
1744 
1744 
1744 
1744 
1744 
 1744 

genus
111111
1912215
2381068860275143

 1748 

1748 
1748 
1748 
1748 
1748 
1748 
2381068860275143
111111
species

genus
111111
29404
6228312811133

6228312811133

29405 
29405 
29405 
29405 
29405 
29405 
 29405 
species
111111

333333
genus
1912216
5308310512725591450224247168

species
111111
 33011 

33011 
33011 
33011 
33011 
33011 
33011 
2635174168

 1747 

1747 
1747 
1747 
1747 
1747 
1747 
5295310491025525448523427138
species
111111

species
111111
 33010 

33010 
33010 
33010 
33010 
33010 
33010 
10418249136622

8102783092151235461
85015
888888
family

654335269752
116071
111111
genus

111111
species

75385 
75385 
75385 
75385 
75385 
75385 
 75385 
654335269752

182639
13629432716341
genus
111111

13629432716341
 182640 

182640 
182640 
182640 
182640 
182640 
182640 
111111
species

333333
genus
1839
27610311388411173

111111
species
 196162 

196162 
196162 
196162 
196162 
196162 
196162 
11134443717765

10842442714968

450734 
450734 
450734 
450734 
450734 
450734 
 450734 
111111
species

111111
species
572725248540
 2045452 

2045452 
2045452 
2045452 
2045452 
2045452 
2045452 

222222
genus
213666644379102
2040

11234362318853
 2079793 

2079793 
2079793 
2079793 
2079793 
2079793 
2079793 
111111
species

10132302119149

2041 
2041 
2041 
2041 
2041 
2041 
 2041 
species
111111

12037523018593
2044
genus
111111

12037523018593

2045 
2045 
2045 
2045 
2045 
2045 
 2045 
111111
species

131313131313
order
12516556334971702857
85008

12516556334971702857
28056
family
131313131313

673534
514360310284570383
genus
333333


2108470 
2108470 
2108470 
2108470 
2108470 
2108470 
 2108470 
258209167157285224
species
111111

species
111111

2071627 
2071627 
2071627 
2071627 
2071627 
2071627 
 2071627 
199134110107232136

111111
species
571733205323
 2024580 

2024580 
2024580 
2024580 
2024580 
2024580 
2024580 

222222
genus
168694
642246137726

species
111111
29111183116
 168697 

168697 
168697 
168697 
168697 
168697 
168697 

111111
species
 168695 

168695 
168695 
168695 
168695 
168695 
168695 
35113554610

1873
12439533422592
genus
222222

species
111111

47850 
47850 
47850 
47850 
47850 
47850 
 47850 
7023251413032

111111
species

648999 
648999 
648999 
648999 
648999 
648999 
 648999 
541628209560

84593
632220127943
genus
111111

 1003110 

1003110 
1003110 
1003110 
1003110 
1003110 
1003110 
632220127943
111111
species

genus
555555
1865
486212204154751313

111111
species
8336463017049

1866 
1866 
1866 
1866 
1866 
1866 
 1866 


2033844 
2033844 
2033844 
2033844 
2033844 
2033844 
 2033844 
493121199046
species
111111

15968714926593

134676 
134676 
134676 
134676 
134676 
134676 
 134676 
species
111111

10256323914357
 196914 

196914 
196914 
196914 
196914 
196914 
196914 
species
111111

932134178368
 649831 

649831 
649831 
649831 
649831 
649831 
649831 
111111
species

1497346
382336136353
111111
class

382336136353
588673
order
111111

320583
382336136353
111111
family

191494
382336136353
genus
111111

 191495 

191495 
191495 
191495 
191495 
191495 
191495 
382336136353
species
111111

2511
1134404
111
phylum

111
class
795747
2511

2511
795748
order
111

11
1334117
11
family

11
genus
1134403
11

11


1134405 


1134405 
 1134405 
11
species

25
795749
family
1

genus
1
25
795750

 591197 

591197 
25
1
species

45412118249
204428
735463
phylum

class
735463
204429
45412118249

order
12
1963360
12

11
92714
family
11

11
71666
11
genus

11

71667 


71667 
 71667 
11
species

92713
1
family
1

1
genus
1
282132

1


389348 
 389348 
1
species

51291
44412118229
order
635443

family
635443
809
44412118229

810
44412118229
635443
genus

1152
 85991 

85991 

85991 
85991 
85991 
1111
species


1457153 
1457153 
 1457153 
12
species
11

1
species
1

83555 
 83555 

111
species
212

83554 


83554 
83554 
 83554 

2220610147

813 
813 
813 
813 
813 
813 
 813 
111111
species

11111
species
46841

83558 
83558 
83558 

83558 
83558 
 83558 

1111
species

83560 
83560 
83560 


83560 
 83560 
141551

203682
111128146114145115
phylum
141210111113

111111
class
24217348136
666505

order
111111
666506
24217348136

666507
24217348136
family
111111

24217348136
666508
111111
genus

 547188 

547188 
547188 
547188 
547188 
547188 
547188 
24217348136
species
111111

13119101012
class
203683
871077366132109

order
11
21
1127829

11
family
1127830
21

21
380738
genus
11

species
11
21

174633 


174633 
 174633 

851077366132108
112
order
12119101011

386418235051
126
765566
family

123
1211
1111
genus

1211


125 

125 
125 
125 
 125 
species
1111

genus
222222
23814184345
118

species
111111
14511113232

1636152 
1636152 
1636152 
1636152 
1636152 
1636152 
 1636152 

111111
species
 1632864 

1632864 
1632864 
1632864 
1632864 
1632864 
1632864 
93371113

genus
11111
1052223
1649490

1052223
 119 

119 
119 
119 

119 
119 
11111
species

genus
1
1
1676125

1

1331910 
 1331910 
species
1

1111
genus
1649480
1121

1111
species
1121
 120 

120 
120 


120 
120 

12122
265488
11111
genus

species
11111
12122

265606 
265606 
265606 
265606 
265606 
 265606 

genus
1111
1936111
2111

species
1111
2111

1891926 

1891926 
1891926 

1891926 
 1891926 

332323
family
1763524
193031193619

10527102512
1763521
111111
genus

species
111111
 1387353 

1387353 
1387353 
1387353 
1387353 
1387353 
1387353 
10527102512

genus
1111
2432
127

1111
species
 128 

128 
128 

128 

128 
2432

72146115
466152
genus
111111

species
111111
 466153 

466153 
466153 
466153 
466153 
466153 
466153 
72146115

family
222222
281324244638
1914233

222222
genus
113
281324244638

species
111111
 1630693 

1630693 
1630693 
1630693 
1630693 
1630693 
1630693 
46812234

24716122334

114 
114 
114 
114 
114 
114 
 114 
111111
species

phylum
121421
200783
4524111

class
121421
4524111
187857

order
12131
45231
32069

224027
44211
family
11111

44211
212790
genus
11111

species
11111
 436114 

436114 
436114 
436114 
436114 
436114 
44211

12
family
12
64898

genus
1
1
939

1
species
1
 940 


940 

genus
1
1
168657

species
1
1


380749 
 380749 

genus
1
75905
1

1
species
1
 136094 


136094 

order
111
1485951
1101

1101
558314
111
family

11
genus
171868
101

101


228745 
228745 
 228745 
species
11

genus
1
64159
1

1
species
1
 64160 


64160 

genus
1
262406
1

1
species

166501 
 166501 
1

phylum
1111
134625
1845

class
1111
1921781
1845

order
1111
1921782
1845

1845
1921783
1111
family

genus
1111
1921784
1845

1845

1307763 

1307763 
1307763 

1307763 
 1307763 
species
1111

5591
456828
11
phylum

5591
456826
11
genus

species
11
5591


456827 

456827 
 456827 

phylum
767879
11549514882120
74201

 1637999 

1637999 

1637999 
1637999 
1637999 
1637999 
7311110
species
11111

1955630
212
class
112

order
112
717963
212

family
112
717964
212

genus
112
511745
212

11


591154 

591154 
 591154 
11
species

species
11
21
 511746 


511746 


511746 

203494
39171015194
class
212222

48461
39171015194
212222
order

family
212222
39171015194
1647988

239934
39171015194
212222
genus

 239935 

239935 
239935 
239935 
239935 
239935 
239935 
2917911113
111111
species

101481

1679444 

1679444 
1679444 
1679444 
1679444 
 1679444 
species
11111

6930383152104
414999
class
444444

111111
order
415001
758441

family
111111
758441
415002

758441
442430
genus
111111

111111
species
758441

395922 
395922 
395922 
395922 
395922 
395922 
 395922 

415000
6225302748103
333333
order

family
333333
134623
6225302748103

1961799
1343121244
111111
genus

 1838286 

1838286 
1838286 
1838286 
1838286 
1838286 
1838286 
1343121244
species
111111

178440
405471742
genus
111111

species
111111

107709 
107709 
107709 
107709 
107709 
107709 
 107709 
405471742

9162381917
 794903 

794903 
794903 
794903 
794903 
794903 
794903 
species
111111

phylum
139613651438137413601330
163467416065291328643200580417670252112716
1224

580370
144842314
class
212122

144842314
580371
212122
order

144842314
580372
family
212122

genus
212122
144842314
377315

7474214
 1921087 

1921087 
1921087 
1921087 
1921087 
1921087 
1921087 
111111
species

71210

1921086 

1921086 

1921086 
1921086 
 1921086 
species
1111

class
380375383359366365
28211
149048998926384401919716617

order
111
351
1191478

family
111
351
1191479

351
162171
genus
111

species
111
351
 1124597 

1124597 
1124597 


1124597 

111111
order
10112264
255473

10112264
255474
111111
family

208215
10112264
111111
genus

10112264
 208216 

208216 
208216 
208216 
208216 
208216 
208216 
111111
species

862115
767891
11111
genus

 767892 

767892 
767892 
767892 

767892 
767892 
862115
species
11111

154152156150152150
order
8332453948804997114219179
356

genus
111111
10233633612
1734920

10233633612
 1235591 

1235591 
1235591 
1235591 
1235591 
1235591 
1235591 
species
111111

45401
261225187211368241
family
111111111111

5985569114939
59282
111111
genus

111111
species
5985569114939
 1079 

1079 
1079 
1079 
1079 
1079 
1079 

111111
genus
1082930
18133782

18133782

531813 
531813 
531813 
531813 
531813 
531813 
 531813 
111111
species

119044
1742185
111111
genus

111111
species
1742185
 1608628 

1608628 
1608628 
1608628 
1608628 
1608628 
1608628 

genus
333333
46913
7952494010496

species
111111
462013203967

1736675 
1736675 
1736675 
1736675 
1736675 
1736675 
 1736675 

111111
species
17282282613
 2083786 

2083786 
2083786 
2083786 
2083786 
2083786 
2083786 

16414123916

1643450 
1643450 
1643450 
1643450 
1643450 
1643450 
 1643450 
species
111111

1068
1114784
111111
genus

111111
species
 1069 

1069 
1069 
1069 
1069 
1069 
1069 
1114784

111111
genus
29407
331425273240

species
111111
 674703 

674703 
674703 
674703 
674703 
674703 
674703 
331425273240

genus
333333
81
605346374955


53399 
53399 
53399 
53399 
53399 
53399 
 53399 
24353332209
111111
species

species
111111
 717785 

717785 
717785 
717785 
717785 
717785 
717785 
164621629

species
111111

1427356 
1427356 
1427356 
1427356 
1427356 
1427356 
 1427356 
2014731317

141414141414
family
606327416440731739
69277

genus
111111
28100
1428121325


1867719 
1867719 
1867719 
1867719 
1867719 
1867719 
 1867719 
1428121325
111111
species

genus
888888
68287
434217217273522536

 593909 

593909 
593909 
593909 
593909 
593909 
593909 
492313405774
111111
species


381 
381 
381 
381 
381 
381 
 381 
461629463259
111111
species

species
111111
261123122350

71433 
71433 
71433 
71433 
71433 
71433 
 71433 

561241333976
 278153 

278153 
278153 
278153 
278153 
278153 
278153 
111111
species

111111
species

1670800 
1670800 
1670800 
1670800 
1670800 
1670800 
 1670800 
572816125345

 536018 

536018 
536018 
536018 
536018 
536018 
536018 
421218308346
species
111111

242321348922
 2066070 

2066070 
2066070 
2066070 
2066070 
2066070 
2066070 
species
111111


39645 
39645 
39645 
39645 
39645 
39645 
 39645 
134925666146164
species
111111

111111
genus
11798913
274591

species
111111

1620421 
1620421 
1620421 
1620421 
1620421 
1620421 
 1620421 
11798913

1442195
245876
genus
111111

1442195
 472175 

472175 
472175 
472175 
472175 
472175 
472175 
species
111111

29931716
449972
111111
genus

 266779 

266779 
266779 
266779 
266779 
266779 
266779 
29931716
111111
species

14488169142151144
31988
genus
222222

111111
species
9246526289105
 374606 

374606 
374606 
374606 
374606 
374606 
374606 


83263 
83263 
83263 
83263 
83263 
83263 
 83263 
5242117806239
species
111111

82115
320815661625186948963749
434245424444
family

genus
111111
652929146970
323620


879274 
879274 
879274 
879274 
879274 
879274 
 879274 
652929146970
111111
species

28105
108855256858816071167
genus
666666

 382 

382 
382 
382 
382 
382 
382 
6973223904521023865
species
111111

species
111111

380 
380 
380 
380 
380 
380 
 380 
196918176237173

111111
species

1842534 
1842534 
1842534 
1842534 
1842534 
1842534 
 1842534 
4532231813036

species
111111

194963 
194963 
194963 
194963 
194963 
194963 
 194963 
9375463113560

111111
species
 794846 

794846 
794846 
794846 
794846 
794846 
794846 
39281816329

species
111111
1841010194
 110321 

110321 
110321 
110321 
110321 
110321 
110321 

329453
34019
genus
224233


309868 
309868 
309868 
 309868 
211
111
species

11111
species
22311

34021 

34021 
34021 
34021 
34021 
 34021 

species
111
113

556287 
556287 
556287 
 556287 

species
111
 1273132 


1273132 


1273132 
1273132 
131

species
11
21


34020 


34020 
 34020 

252425242525
genus
1591761827100324711955
379

111111
species
2499102520
 1125847 

1125847 
1125847 
1125847 
1125847 
1125847 
1125847 

111111
species
5110582835
 2028343 

2028343 
2028343 
2028343 
2028343 
2028343 
2028343 

161431173443
 1301032 

1301032 
1301032 
1301032 
1301032 
1301032 
1301032 
species
111111

species
111111

1703964 
1703964 
1703964 
1703964 
1703964 
1703964 
 1703964 
912144419

species
111111
311415356348
 1914541 

1914541 
1914541 
1914541 
1914541 
1914541 
1914541 

species
111111

2020312 
2020312 
2020312 
2020312 
2020312 
2020312 
 2020312 
27715114319

111111
species
391527236976
 1571470 

1571470 
1571470 
1571470 
1571470 
1571470 
1571470 

488167181286658483
 384 

384 
384 
384 
384 
384 
384 
species
111111

211012233011
 1869170 

1869170 
1869170 
1869170 
1869170 
1869170 
1869170 
species
111111

 1703968 

1703968 
1703968 
1703968 
1703968 
1703968 
1703968 
13151834229
111111
species

111111
species
19128313227
 1703960 

1703960 
1703960 
1703960 
1703960 
1703960 
1703960 

species
111111
19138123629
 398 

398 
398 
398 
398 
398 
398 

545222
 1703967 

1703967 
1703967 

1703967 
1703967 
1703967 
species
11111

111111
species
352014174676

1981173 
1981173 
1981173 
1981173 
1981173 
1981173 
 1981173 

48712161

1703962 
1703962 
1703962 
1703962 
1703962 
1703962 
 1703962 
species
111111

123


1703961 

1703961 
1703961 
 1703961 
111
species

species
111111
28610273951
 2048897 

2048897 
2048897 
2048897 
2048897 
2048897 
2048897 

species
111111
30128104113
 2020313 

2020313 
2020313 
2020313 
2020313 
2020313 
2020313 

 1703969 

1703969 
1703969 
1703969 
1703969 
1703969 
1703969 
321233244461
species
111111

13456911

1703965 
1703965 
1703965 
1703965 
1703965 
1703965 
 1703965 
species
111111

37291697721

2020311 
2020311 
2020311 
2020311 
2020311 
2020311 
 2020311 
species
111111


396 
396 
396 
396 
396 
396 
 396 
383241236289612507
species
111111

111111
species

56730 
56730 
56730 
56730 
56730 
56730 
 56730 
3836253810677

 1703966 

1703966 

1703966 
24
species
11


29449 
29449 
29449 
29449 
29449 
29449 
 29449 
2128811492320281
111111
species

species
111111
 424182 

424182 
424182 
424182 
424182 
424182 
424182 
15311113622

111111
genus
7847354011046
1525371

111111
species

399 
399 
399 
399 
399 
399 
 399 
7847354011046

310146115167390310
357
genus
666666

species
111111

359 
359 
359 
359 
359 
359 
 359 
35812163546

111111
species
202813414928
 1842536 

1842536 
1842536 
1842536 
1842536 
1842536 
1842536 

 861208 

861208 
861208 
861208 
861208 
861208 
861208 
3119104621
species
111111

37734408

373 
373 
373 
373 
373 
373 
 373 
111111
species

1871027896220207
1183400
species group
222222

139785179177170

358 
358 
358 
358 
358 
358 
 358 
species
111111

 1176649 

1176649 
1176649 
1176649 
1176649 
1176649 
1176649 
482427174337
species
111111

222222
genus
73294253244198
106591


106592 
106592 
106592 
106592 
106592 
106592 
 106592 
53223336182135
species
111111

species
111111
2079176263

716925 
716925 
716925 
716925 
716925 
716925 
 716925 

191919191919
family
145873198176119391361
119045

333333
genus
2282523
486220328237678455

391722124312
 29429 

29429 
29429 
29429 
29429 
29429 
29429 
111111
species

species
111111
101326970117101

223967 
223967 
223967 
223967 
223967 
223967 
 223967 

346171237155518342
 408 

408 
408 
408 
408 
408 
408 
species
111111

222222
genus
73344729112137
186650

3331072932

2082949 
2082949 
2082949 
2082949 
2082949 
2082949 
 2082949 
species
111111

4031372283105

1882682 
1882682 
1882682 
1882682 
1882682 
1882682 
 1882682 
111111
species

genus
141414141414
407
8994776064951149769

111111
species
642040276846

2202826 
2202826 
2202826 
2202826 
2202826 
2202826 
 2202826 

795649228375

334852 
334852 
334852 
334852 
334852 
334852 
 334852 
species
111111


270351 
270351 
270351 
270351 
270351 
270351 
 270351 
514249819270
species
111111

471129196927
 2067957 

2067957 
2067957 
2067957 
2067957 
2067957 
2067957 
111111
species

111111
species

925818 
925818 
925818 
925818 
925818 
925818 
 925818 
333321255835

111111
species
 2202828 

2202828 
2202828 
2202828 
2202828 
2202828 
2202828 
5024284912047

 1826873 

1826873 
1826873 
1826873 
1826873 
1826873 
1826873 
7650683211926
species
111111

species
111111
 426117 

426117 
426117 
426117 
426117 
426117 
426117 
594440258739

species
111111
 2202825 

2202825 
2202825 
2202825 
2202825 
2202825 
2202825 
511028213815

species
111111
642035314647
 114616 

114616 
114616 
114616 
114616 
114616 
114616 


418223 
418223 
418223 
418223 
418223 
418223 
 418223 
5617464384108
111111
species

111111
species

1479019 
1479019 
1479019 
1479019 
1479019 
1479019 
 1479019 
8534525599100

species
111111
 31998 

31998 
31998 
31998 
31998 
31998 
31998 
916481318365

species
111111

2202827 
2202827 
2202827 
2202827 
2202827 
2202827 
 2202827 
9352403410369

222222
family
8484743810783
2036754

8484743810783
28209
222222
genus

367544187322

1702325 
1702325 
1702325 
1702325 
1702325 
1702325 
 1702325 
species
111111

48930203461
 444444 

444444 
444444 
444444 
444444 
444444 
444444 
111111
species

41294
1637103295588521541778
family
232323232323

genus
111111
402216256209444410
1073

species
111111

1076 
1076 
1076 
1076 
1076 
1076 
 1076 
402216256209444410

40136
513131438169
111111
genus

 40137 

40137 
40137 
40137 
40137 
40137 
40137 
513131438169
species
111111

211973275
1649510
genus
111111

211973275
 1333996 

1333996 
1333996 
1333996 
1333996 
1333996 
1333996 
species
111111

85413
1261218975267175
444444
genus

3023181010344
 2015316 

2015316 
2015316 
2015316 
2015316 
2015316 
2015316 
111111
species

species
111111
311621263756

1526658 
1526658 
1526658 
1526658 
1526658 
1526658 
 1526658 

111111
species

1792307 
1792307 
1792307 
1792307 
1792307 
1792307 
 1792307 
274819164730

species
111111
383431238045
 1842539 

1842539 
1842539 
1842539 
1842539 
1842539 
1842539 

911
261222253118
222222
genus

111111
species
 912 

912 
912 
912 
912 
912 
912 
19619171714

111111
species
7638144

913 
913 
913 
913 
913 
913 
 913 

141414141414
genus
374
101163355053013041101

111111
species
443528276853
 114615 

114615 
114615 
114615 
114615 
114615 
114615 

 1274631 

1274631 
1274631 
1274631 
1274631 
1274631 
1274631 
462433244150
species
111111

111111
species
908836477743
 2057741 

2057741 
2057741 
2057741 
2057741 
2057741 
2057741 

111111
species
291325309828
 335659 

335659 
335659 
335659 
335659 
335659 
335659 

372931298635

1404367 
1404367 
1404367 
1404367 
1404367 
1404367 
 1404367 
111111
species

111111
species
66172865132
 1223566 

1223566 
1223566 
1223566 
1223566 
1223566 
1223566 

 1355477 

1355477 
1355477 
1355477 
1355477 
1355477 
1355477 
228175148144318249
species
111111

111111
species
572825177141
 288000 

288000 
288000 
288000 
288000 
288000 
288000 

111111
species

44255 
44255 
44255 
44255 
44255 
44255 
 44255 
591921177133

111111
species

1404768 
1404768 
1404768 
1404768 
1404768 
1404768 
 1404768 
442528254177


931866 
931866 
931866 
931866 
931866 
931866 
 931866 
522212196078
species
111111


375 
375 
375 
375 
375 
375 
 375 
14511685100198239
111111
species

 115808 

115808 
115808 
115808 
115808 
115808 
115808 
462418335583
111111
species


376 
376 
376 
376 
376 
376 
 376 
681832126960
species
111111

616310335484659788
118882
family
161614151514

444444
genus
7245365375136
528

152917132953
 529 

529 
529 
529 
529 
529 
529 
species
111111

species
111111

271865 
271865 
271865 
271865 
271865 
271865 
 271865 
1341011236

276511665
 571256 

571256 
571256 
571256 
571256 
571256 
571256 
species
111111

1764181712
 419475 

419475 
419475 
419475 
419475 
419475 
419475 
species
111111

genus
121210111110
234
544265299431584652

8253935
 1149952 

1149952 
1149952 
1149952 
1149952 
1149952 
1149952 
species
111111

161
 236 

236 
236 


236 
species
111

111
species
 120577 


120577 
120577 


120577 
112

592439628878
 235 

235 
235 
235 
235 
235 
235 
species
111111

31272

120576 
120576 

120576 
120576 
120576 
 120576 
species
11111

 1844051 

1844051 
1844051 
1844051 
1844051 
1844051 
41344
11111
species

species
111111

981386 
981386 
981386 
981386 
981386 
981386 
 981386 
311411

6132131

1885919 
1885919 
1885919 
1885919 
1885919 
1885919 
 1885919 
species
111111

11111
species

444163 

444163 
444163 
444163 
444163 
 444163 
71119

111111
species
5948543410689
 29461 

29461 
29461 
29461 
29461 
29461 
29461 

359169184305320403

29459 
29459 
29459 
29459 
29459 
29459 
 29459 
111111
species

species
111

1891098 
1891098 

1891098 
 1891098 
121

3498133432

36855 
36855 
36855 
36855 
36855 
36855 
 36855 
111111
species

111111
genus
341311203927
1572860

species
111111
341311203927
 1482074 

1482074 
1482074 
1482074 
1482074 
1482074 
1482074 

444444
family
105514874161118
255475

genus
333333
7429406811270
293088

 686597 

686597 
686597 
686597 
686597 
686597 
686597 
19121211416
111111
species

species
111111
 293089 

293089 
293089 
293089 
293089 
293089 
293089 
32816334443


1486262 
1486262 
1486262 
1486262 
1486262 
1486262 
 1486262 
23912242721
species
111111

111111
genus
414371
3122864948

species
111111

1349819 
1349819 
1349819 
1349819 
1349819 
1349819 
 1349819 
3122864948

335928
148375155189120
family
333333

321015195553
152053
111111
genus

 921 

921 
921 
921 
921 
921 
921 
321015195553
species
111111

111111
genus
279
521922165322

111111
species
521922165322

280 
280 
280 
280 
280 
280 
 280 

64814208145
6
111111
genus

111111
species
64814208145
 7 

7 
7 
7 
7 
7 
7 

family
222222
119043
2484171628

1042121221
256616
genus
111111

1042121221
 256618 

256618 
256618 
256618 
256618 
256618 
256618 
species
111111

444432
1442547
111111
genus

1442547
 1922226 

1922226 
1922226 
1922226 
1922226 
1922226 
1922226 
111111
species

353728303656
31993
family
333333

genus
111111
171514121834
425

171514121834

426 
426 
426 
426 
426 
426 
 426 
species
111111

genus
222222
182214181822
133

species
111111
 655015 

655015 
655015 
655015 
655015 
655015 
655015 
10292610

111111
species

187303 
187303 
187303 
187303 
187303 
187303 
 187303 
8205161212

family
8810665
526195682211
772

773
526195682211
8810665
genus

11
species

1933912 


1933912 
 1933912 
11

species
1
1
 1933907 


1933907 

14161


1686310 
1686310 
1686310 
1686310 
1686310 
 1686310 
11111
species

1


1933910 
 1933910 
species
1

species
11111
 38323 

38323 
38323 
38323 

38323 
38323 
310341


85701 
85701 
85701 

85701 
85701 
 85701 
613411
species
11111

2130605997

803 
803 
803 
803 
803 
803 
 803 
species
111111

11
species
11
 515256 


515256 

515256 

species
111
151


1933906 
1933906 
1933906 
 1933906 

1541

388640 
388640 
388640 
 388640 
111
species


1933904 

1933904 
1933904 
 1933904 
491
111
species

species
1111

33045 
33045 
33045 
33045 
 33045 
1181

1
 774 


774 
species
1

14

56426 

56426 
 56426 
11
species

45404
362519214047
222222
family

111111
genus
2248121138
532

species
111111

533 
533 
533 
533 
533 
533 
 533 
2248121138

120652
1421119299
genus
111111

111111
species
 199596 

199596 
199596 
199596 
199596 
199596 
199596 
1421119299

1484898
18915212821
212222
genus

11111
species

1384459 

1384459 
1384459 
1384459 
1384459 
 1384459 
1076116

species
111111
 2170729 

2170729 
2170729 
2170729 
2170729 
2170729 
2170729 
89815275

204457
211913621602103924522256
676768666866
order

41297
18421168134880720551903
494849474947
family

1649486
391718133431
111111
genus

 1850238 

1850238 
1850238 
1850238 
1850238 
1850238 
1850238 
391718133431
111111
species

444444
genus
1638314277135137
165696

species
111111
27161471329
 205844 

205844 
205844 
205844 
205844 
205844 
205844 

111111
species
391826162943
 702113 

702113 
702113 
702113 
702113 
702113 
702113 

species
111111
862467447150
 158500 

158500 
158500 
158500 
158500 
158500 
158500 

 48935 

48935 
48935 
48935 
48935 
48935 
48935 
112535102215
111111
species

541
172921192520
genus
111111

 542 

542 
542 
542 
542 
542 
542 
172921192520
111111
species

3410981416
72173
genus
111111

111111
species

1634516 
1634516 
1634516 
1634516 
1634516 
1634516 
 1634516 
3410981416

989898
genus
165697
242164226121303301

species
111111
12101392321

117207 
117207 
117207 
117207 
117207 
117207 
 117207 

251022112323
 33052 

33052 
33052 
33052 
33052 
33052 
33052 
111111
species

111111
species

1515612 
1515612 
1515612 
1515612 
1515612 
1515612 
 1515612 
242011133159

111111
species
304116143848

1357916 
1357916 
1357916 
1357916 
1357916 
1357916 
 1357916 

111111
species
18931153739
 1866325 

1866325 
1866325 
1866325 
1866325 
1866325 
1866325 

111111
species
212438212826

267128 
267128 
267128 
267128 
267128 
267128 
 267128 


1913578 

1913578 

1913578 
 1913578 
237
species
111

species
111111
952167308166

33050 
33050 
33050 
33050 
33050 
33050 
 33050 


292913 
292913 
292913 
292913 
292913 
292913 
 292913 
15292583519
species
111111

826520533327974729
13687
141414141414
genus

species
111111
 1560345 

1560345 
1560345 
1560345 
1560345 
1560345 
1560345 
7359621711479


1609977 
1609977 
1609977 
1609977 
1609977 
1609977 
 1609977 
512433216492
species
111111

111111
species
15279823712198
 152682 

152682 
152682 
152682 
152682 
152682 
152682 


745310 
745310 
745310 
745310 
745310 
745310 
 745310 
232228174441
species
111111

111111
species
 1327635 

1327635 
1327635 
1327635 
1327635 
1327635 
1327635 
453231249740

111111
species

1961362 
1961362 
1961362 
1961362 
1961362 
1961362 
 1961362 
422931224747

111111
species

1030157 
1030157 
1030157 
1030157 
1030157 
1030157 
 1030157 
493228133357


93064 
93064 
93064 
93064 
93064 
93064 
 93064 
422527205315
111111
species

111111
species
476651227924

397260 
397260 
397260 
397260 
397260 
397260 
 397260 

111111
species
682716235543
 1390395 

1390395 
1390395 
1390395 
1390395 
1390395 
1390395 

161011122411

1921510 
1921510 
1921510 
1921510 
1921510 
1921510 
 1921510 
111111
species

species
111111
7125422610443
 1549858 

1549858 
1549858 
1549858 
1549858 
1549858 
1549858 

species
111111
221825124538

1938607 
1938607 
1938607 
1938607 
1938607 
1938607 
 1938607 


160791 
160791 
160791 
160791 
160791 
160791 
 160791 
12572666194101
species
111111

575060104026
150203
genus
111111

species
111111
575060104026

1842535 
1842535 
1842535 
1842535 
1842535 
1842535 
 1842535 

genus
444343
333216224366
1434046

species
1111
7724
 1922222 

1922222 
1922222 
1922222 

1922222 

111111
species
99451112

266812 
266812 
266812 
266812 
266812 
266812 
 266812 

1636101848
 2077182 

2077182 
2077182 
2077182 
2077182 
2077182 
2077182 
species
111111

species
111111
11347106

1806885 
1806885 
1806885 
1806885 
1806885 
1806885 
 1806885 

genus
141414141414
431263323210487577
165695

111111
species
10083664495150
 13690 

13690 
13690 
13690 
13690 
13690 
13690 

 1843368 

1843368 
1843368 
1843368 
1843368 
1843368 
1843368 
221229122322
111111
species

species
111111

627192 
627192 
627192 
627192 
627192 
627192 
 627192 
28232383512

121113102448

46429 
46429 
46429 
46429 
46429 
46429 
 46429 
species
111111

species
111111

120107 
120107 
120107 
120107 
120107 
120107 
 120107 
391412121740

23832164424

1315974 
1315974 
1315974 
1315974 
1315974 
1315974 
 1315974 
species
111111

 332056 

332056 
332056 
332056 
332056 
332056 
332056 
381217152815
111111
species

111111
species
 1673076 

1673076 
1673076 
1673076 
1673076 
1673076 
1673076 
312117204080

128812146
 76947 

76947 
76947 
76947 
76947 
76947 
76947 
species
111111

species
111111
1410116184

1332080 
1332080 
1332080 
1332080 
1332080 
1332080 
 1332080 

17141482629
 1855519 

1855519 
1855519 
1855519 
1855519 
1855519 
1855519 
111111
species


484429 
484429 
484429 
484429 
484429 
484429 
 484429 
392046175552
species
111111

111111
species

332055 
332055 
332055 
332055 
332055 
332055 
 332055 
481614214186

111111
species

407020 
407020 
407020 
407020 
407020 
407020 
 407020 
811219279

181919191919
family
277194254232397353
335929

genus
777777
82437973120146
361177

27417141520

1267766 
1267766 
1267766 
1267766 
1267766 
1267766 
 1267766 
species
111111

104128229
 692370 

692370 
692370 
692370 
692370 
692370 
692370 
111111
species


543877 
543877 
543877 
543877 
543877 
543877 
 543877 
125911366
species
111111

117912817

361183 
361183 
361183 
361183 
361183 
361183 
 361183 
111111
species

species
111111

476157 
476157 
476157 
476157 
476157 
476157 
 476157 
3242511


645517 
645517 
645517 
645517 
645517 
645517 
 645517 
91315201452
species
111111

species
111111
1081362031
 1982042 

1982042 
1982042 
1982042 
1982042 
1982042 
1982042 

genus
333333
522950335233
1111


1896196 
1896196 
1896196 
1896196 
1896196 
1896196 
 1896196 
23132481211
species
111111

species
111111
1661291517
 2023229 

2023229 
2023229 
2023229 
2023229 
2023229 
2023229 

 1112 

1112 
1112 
1112 
1112 
1112 
1112 
13101416255
111111
species

222222
genus
1295327
344122326440

1117462923
 1348774 

1348774 
1348774 
1348774 
1348774 
1348774 
1348774 
111111
species

species
111111
232418263517

450378 
450378 
450378 
450378 
450378 
450378 
 450378 

677777
genus
1041
1098110394161134

111111
species
 2011159 

2011159 
2011159 
2011159 
2011159 
2011159 
2011159 
674366

852213278
 192812 

192812 
192812 
192812 
192812 
192812 
192812 
111111
species

5637326

1648404 
1648404 
1648404 
1648404 
1648404 
1648404 
 1648404 
species
111111

141132


1922225 
1922225 
1922225 
1922225 
1922225 
 1922225 
11111
species

111111
species
10191223288

266951 
266951 
266951 
266951 
266951 
266951 
 266951 


39960 
39960 
39960 
39960 
39960 
39960 
 39960 
493641235168
111111
species


502682 
502682 
502682 
502682 
502682 
502682 
 502682 
31717141436
111111
species

order
676665666867
167712201040109823462282
204455

family
333443
69657
121019245669

genus
222222
85
878164321

111111
species
 81032 

81032 
81032 
81032 
81032 
81032 
81032 
4554308

 1906738 

1906738 
1906738 
1906738 
1906738 
1906738 
1906738 
423121313
111111
species

111111
genus
431171248
74317

431171248
 74318 

74318 
74318 
74318 
74318 
74318 
74318 
111111
species

11
2723
genus
11

11
 2724 


2724 
2724 
species
11

31989
166512101021107422902213
family
646362626464

genus
111111
16713112641
1609958

species
111111
 1609966 

1609966 
1609966 
1609966 
1609966 
1609966 
1609966 
16713112641

111111
species
 2033435 

2033435 
2033435 
2033435 
2033435 
2033435 
2033435 
91174247

222222
genus
3192873317
97050

2951232015
 89184 

89184 
89184 
89184 
89184 
89184 
89184 
111111
species

111111
species

292414 
292414 
292414 
292414 
292414 
292414 
 292414 
24164132

111111
genus
23616135236
436357

23616135236

2109625 
2109625 
2109625 
2109625 
2109625 
2109625 
 2109625 
111111
species

genus
111111
1097466
371822135246

371822135246
 1335048 

1335048 
1335048 
1335048 
1335048 
1335048 
1335048 
species
111111

111111
genus
301111226716
58842

301111226716
 2009329 

2009329 
2009329 
2009329 
2009329 
2009329 
2009329 
species
111111

285107
1020610447
111111
genus


1915078 
1915078 
1915078 
1915078 
1915078 
1915078 
 1915078 
1020610447
111111
species

6752102
2211641
genus
111111

111111
species
6752102
 245188 

245188 
245188 
245188 
245188 
245188 
245188 

111111
genus
227873
532719204037

532719204037

121719 
121719 
121719 
121719 
121719 
121719 
 121719 
111111
species

genus
11111
188905
1324832

1324832

290400 
290400 
290400 

290400 
290400 
 290400 
11111
species

species
11111
411105

1904441 
1904441 
1904441 

1904441 
1904441 
 1904441 

309512
3131292137
111111
genus

 215813 

215813 
215813 
215813 
215813 
215813 
215813 
3131292137
111111
species

genus
1111
2311
1284657

2311

1284658 


1284658 
1284658 
1284658 
 1284658 
1111
species

360528
222010113813
genus
111111

species
111111
 1250539 

1250539 
1250539 
1250539 
1250539 
1250539 
1250539 
222010113813

222222
genus
92944
7246524411465

102461013
 92947 

92947 
92947 
92947 
92947 
92947 
92947 
species
111111

 92945 

92945 
92945 
92945 
92945 
92945 
92945 
6244483810452
111111
species

genus
222222
231118145429
60136

111111
species
18389820

1917485 
1917485 
1917485 
1917485 
1917485 
1917485 
 1917485 

 1402135 

1402135 
1402135 
1402135 
1402135 
1402135 
1402135 
58105469
species
111111

genus
222222
354203
7837343711581

 311180 

311180 
311180 
311180 
311180 
311180 
311180 
471918125927
111111
species

species
111111
311816255654
 1792508 

1792508 
1792508 
1792508 
1792508 
1792508 
1792508 

444443
genus
318814386440
875170

51535710
 1411902 

1411902 
1411902 
1411902 
1411902 
1411902 
1411902 
species
111111

 1758178 

1758178 
1758178 
1758178 
1758178 
1758178 
1758178 
66214117
species
111111


1397108 
1397108 
1397108 
1397108 
1397108 
 1397108 
650224
species
11111

species
111111
14177174223

1208324 
1208324 
1208324 
1208324 
1208324 
1208324 
 1208324 

1579315
22
11
genus

species
11
22


1579316 


1579316 
 1579316 

genus
443444
256191176247335372
302485

623828445463

60890 
60890 
60890 
60890 
60890 
60890 
 60890 
species
111111

species
111111
271620523868
 1580596 

1580596 
1580596 
1580596 
1580596 
1580596 
1580596 


221822 
221822 
221822 
221822 
221822 
221822 
 221822 
165132128147222239
111111
species

254212
 1844006 

1844006 
1844006 

1844006 
1844006 
1844006 
11111
species

genus
333333
108765837153124
34008

111111
species

308754 
308754 
308754 
308754 
308754 
308754 
 308754 
352220135024

species
111111
817832212

1564506 
1564506 
1564506 
1564506 
1564506 
1564506 
 1564506 

653730218188
 35806 

35806 
35806 
35806 
35806 
35806 
35806 
111111
species

214153141160341259
1060
444444
genus

111111
species
 1061 

1061 
1061 
1061 
1061 
1061 
1061 
81311105013

27128141113
 1850250 

1850250 
1850250 
1850250 
1850250 
1850250 
1850250 
111111
species

111111
species
139108107120245209

1063 
1063 
1063 
1063 
1063 
1063 
 1063 

 2033869 

2033869 
2033869 
2033869 
2033869 
2033869 
2033869 
402015163524
111111
species

222221
genus
2433
1020119173

11111
species
242110

42443 
42443 
42443 
42443 
42443 
 42443 

species
111111
8169873
 2434 

2434 
2434 
2434 
2434 
2434 
2434 

genus
111111
12911195745
191028

12911195745
 133924 

133924 
133924 
133924 
133924 
133924 
133924 
species
111111

1810622128
159345
genus
111111

111111
species

159346 
159346 
159346 
159346 
159346 
159346 
 159346 
1810622128

genus
333333
392128425097
478070

179941615

187304 
187304 
187304 
187304 
187304 
187304 
 187304 
species
111111

1664151930

2021862 
2021862 
2021862 
2021862 
2021862 
2021862 
 2021862 
111111
species

6615231552

1674922 
1674922 
1674922 
1674922 
1674922 
1674922 
 1674922 
species
111111

299261
63725293
genus
111111

63725293
 299262 

299262 
299262 
299262 
299262 
299262 
299262 
111111
species

323323
genus
686121210
53945

species
111111
111111

1458307 
1458307 
1458307 
1458307 
1458307 
1458307 
 1458307 


1217908 
1217908 
1217908 
1217908 
1217908 
1217908 
 1217908 
2749118
species
111111

3121

53946 

53946 
53946 

53946 
 53946 
1111
species

367771
18710134573
genus
111111


42444 
42444 
42444 
42444 
42444 
42444 
 42444 
18710134573
species
111111

387336233171366582
265
999999
genus

 2065379 

2065379 
2065379 
2065379 
2065379 
2065379 
2065379 
35221476240
111111
species

111111
species
161141586389251

147645 
147645 
147645 
147645 
147645 
147645 
 147645 

263532173135

1945662 
1945662 
1945662 
1945662 
1945662 
1945662 
 1945662 
species
111111

 34003 

34003 
34003 
34003 
34003 
34003 
34003 
241115162520
111111
species

species
111111
 1499308 

1499308 
1499308 
1499308 
1499308 
1499308 
1499308 
353511132868


1529068 
1529068 
1529068 
1529068 
1529068 
1529068 
 1529068 
1763761049
species
111111

111111
species

266 
266 
266 
266 
266 
266 
 266 
304517205732


1077935 
1077935 
1077935 
1077935 
1077935 
1077935 
 1077935 
29101081122
species
111111

 34004 

34004 
34004 
34004 
34004 
34004 
34004 
303139215365
111111
species

111111
genus
1649279
423874


379347 
379347 
379347 
379347 
379347 
379347 
 379347 
423874
species
111111

222222
genus
74030
9317313415

111111
species
71626273

215743 
215743 
215743 
215743 
215743 
215743 
 215743 

species
111111
22115712

391613 
391613 
391613 
391613 
391613 
391613 
 391613 

451819283053
263377
111111
genus

species
111111
451819283053
 1229727 

1229727 
1229727 
1229727 
1229727 
1229727 
1229727 

21765715
204456
genus
111111


2169400 
2169400 
2169400 
2169400 
2169400 
2169400 
 2169400 
21765715
species
111111

1759396
23223
genus
11111

 1920883 

1920883 
1920883 

1920883 
1920883 
1920883 
23223
11111
species

111111
genus
1917145911
74032

111111
species
 74033 

74033 
74033 
74033 
74033 
74033 
74033 
1917145911

111
genus
322
119541

111
species
322
 441209 


441209 

441209 
441209 

order
332
54526
562

11
2045213
genus
11

species
11
 1925548 

1925548 
1925548 
11

222
family
452
1655514

198251
211
111
genus

species
1


1002672 
 1002672 
1

species
11
21

198252 
198252 
 198252 

 859653 

859653 
859653 
859653 
241
111
species

151515151515
order
204458
1173526569324907977

151515151515
family
76892
1173526569324907977

62818829269222254
41275
666666
genus

351920132931

74313 
74313 
74313 
74313 
74313 
74313 
 74313 
species
111111

621735133837

1532555 
1532555 
1532555 
1532555 
1532555 
1532555 
 1532555 
species
111111

species
111111
 1938605 

1938605 
1938605 
1938605 
1938605 
1938605 
1938605 
44322294731

2123974134332
 41276 

41276 
41276 
41276 
41276 
41276 
41276 
species
111111

111111
species
2494485132553
 1827469 

1827469 
1827469 
1827469 
1827469 
1827469 
1827469 

111111
species
26375684070
 588932 

588932 
588932 
588932 
588932 
588932 
588932 

222222
genus
20
765958289792

111111
species
237481938
 2201350 

2201350 
2201350 
2201350 
2201350 
2201350 
2201350 

111111
species
535254207854
 284016 

284016 
284016 
284016 
284016 
284016 
284016 

555555
genus
446251207203546557
75

109435661142166
 88688 

88688 
88688 
88688 
88688 
88688 
88688 
111111
species

species
111111
25431874752
 69666 

69666 
69666 
69666 
69666 
69666 
69666 

783219227857
 366602 

366602 
366602 
366602 
366602 
366602 
366602 
species
111111

111111
species
 69395 

69395 
69395 
69395 
69395 
69395 
69395 
464020185241

188939495227241
 155892 

155892 
155892 
155892 
155892 
155892 
155892 
species
111111

genus
111111
76890
15228232960

15228232960

78587 
78587 
78587 
78587 
78587 
78587 
 78587 
species
111111

 1759059 

1759059 
1759059 
1759059 
1759059 
1759059 
1759059 
86411314
111111
species

genus
111111
97312021
213485

species
111111

349221 
349221 
349221 
349221 
349221 
349221 
 349221 
97312021

232211
order
452311
1921002

family
11111
21121
44746

species
11111

86106 
86106 
86106 
86106 

86106 
 86106 
21121

1
1777752
1
family

1521255
1
1
genus

 91604 


91604 
1
species
1

family
11111
2100208
23111

genus
11111
1509243
23111

 1509244 

1509244 
1509244 
1509244 
1509244 
1509244 
23111
11111
species

genus
111111
332018153950
991903

 991904 

991904 
991904 
991904 
991904 
991904 
991904 
332018153950
species
111111

111111
genus
271716154934
1632780

111111
species
271716154934
 1868589 

1868589 
1868589 
1868589 
1868589 
1868589 
1868589 

1296100481188918881727
204441
424043404244
order

91671446357812341285
41295
201919202021
family

265330303943
1543704
111111
genus

species
111111

1612173 
1612173 
1612173 
1612173 
1612173 
1612173 
 1612173 
265330303943

genus
666666
191
546433229300698637

13079485017086

193 
193 
193 
193 
193 
193 
 193 
111111
species

species
111111
4644273511371
 2202148 

2202148 
2202148 
2202148 
2202148 
2202148 
2202148 

species
111111

664962 
664962 
664962 
664962 
664962 
664962 
 664962 
453035659456

 192 

192 
192 
192 
192 
192 
192 
1691075473146206
111111
species

species
111111
 682998 

682998 
682998 
682998 
682998 
682998 
682998 
544430255381

species
111111
 528244 

528244 
528244 
528244 
528244 
528244 
528244 
1021293552122137

1263978
11
genus
11

11
species
11


1263979 


1263979 
 1263979 

41041158
1182780
genus
111111

111111
species
41041158

1288970 
1288970 
1288970 
1288970 
1288970 
1288970 
 1288970 

1081
1015544399262
genus
222222

111111
species

1085 
1085 
1085 
1085 
1085 
1085 
 1085 
484022274512


34018 
34018 
34018 
34018 
34018 
34018 
 34018 
531522124750
111111
species

444444
genus
13134
120517587200293

111111
species
5328333975182
 55518 

55518 
55518 
55518 
55518 
55518 
55518 

111111
species

1663591 
1663591 
1663591 
1663591 
1663591 
1663591 
 1663591 
211313295441

 84159 

84159 
84159 
84159 
84159 
84159 
84159 
29218113756
111111
species

1781183414
 1639348 

1639348 
1639348 
1639348 
1639348 
1639348 
1639348 
species
111111

1111
1804663
genus
1111

1111
species
1111
 1549855 

1549855 


1549855 
1549855 
1549855 

111111
genus
171436
4330243210187

species
111111

171437 
171437 
171437 
171437 
171437 
171437 
 171437 
4330243210187

152511472362
168934
212222
genus


2048283 

2048283 
2048283 
2048283 
2048283 
 2048283 
341887
11111
species

111111
species
12257291555
 220697 

220697 
220697 
220697 
220697 
220697 
220697 

533937335262
1543705
genus
111111

111111
species
 28077 

28077 
28077 
28077 
28077 
28077 
28077 
533937335262

genus
111111
717981329
1612157


1084 
1084 
1084 
1084 
1084 
1084 
 1084 
717981329
species
111111

433
380290348311654442
222124202223
family

222222
genus
7547515115366
125216

402927306921

2018065 
2018065 
2018065 
2018065 
2018065 
2018065 
 2018065 
species
111111

 257708 

257708 
257708 
257708 
257708 
257708 
257708 
351824218445
111111
species

genus
658567
434
4555647649101


104102 
104102 
104102 
104102 
104102 
104102 
 104102 
613561
111111
species

species
111111
121314335

481146 
481146 
481146 
481146 
481146 
481146 
 481146 

species
11
22

65959 

65959 
 65959 

11111
subgenus
151157
6131128

species
11111
 435 

435 
435 
435 

435 
435 
6131128

232088
 446692 

446692 

446692 
446692 
446692 
446692 
species
11111

172048362426
 438 

438 
438 
438 
438 
438 
438 
species
111111

species
111
312


1633874 
1633874 

1633874 
 1633874 

species
1111
20171
 1076596 


1076596 
1076596 

1076596 
1076596 

1602345
23141
11111
genus

species
11111
 1510841 

1510841 
1510841 
1510841 

1510841 
1510841 
23141

111111
genus
7849527616240
364409

7849527616240

364410 
364410 
364410 
364410 
364410 
364410 
 364410 
species
111111

222222
genus
271812145352
522

111111
species
 62140 

62140 
62140 
62140 
62140 
62140 
62140 
1110682124

111111
species
168663228
 524 

524 
524 
524 
524 
524 
524 

553444305446
89583
111111
genus

species
111111
553444305446

33996 
33996 
33996 
33996 
33996 
33996 
 33996 

320496
1021312522
111111
genus

species
111111

320497 
320497 
320497 
320497 
320497 
320497 
 320497 
1021312522

321844126149
441
genus
222222

species
111111
92441523
 318683 

318683 
318683 
318683 
318683 
318683 
318683 

111111
species

442 
442 
442 
442 
442 
442 
 442 
23164084626

7364244
153497
genus
111111

species
111111
 153496 

153496 
153496 
153496 
153496 
153496 
153496 
7364244

genus
111111
91914
214372

214372

91915 
91915 
91915 
91915 
91915 
91915 
 91915 
species
111111

444444
genus
476057338259
1434011


1177712 
1177712 
1177712 
1177712 
1177712 
1177712 
 1177712 
6111491617
species
111111

species
111111
18231992928
 28448 

28448 
28448 
28448 
28448 
28448 
28448 

132811119
 33995 

33995 
33995 
33995 
33995 
33995 
33995 
111111
species

111111
species
 265960 

265960 
265960 
265960 
265960 
265960 
265960 
1024164265

242327161516
order
766
208280317574770

family
15121610107
10411593402215
775

2115980
11253
11111
genus

species
11111
11253

2115978 

2115978 
2115978 
2115978 
2115978 
 2115978 

10311592381712
33988
141215996
tribe

131114885
genus
9710167221511
780

73925118106
114277
999663
species group

111
species
212


783 
783 
783 
 783 

 42862 

42862 
42862 
14
species
11

111
species
131

1105106 
1105106 
1105106 
 1105106 

species
111

781 
781 
781 
 781 
112

111111
species
446132344
 33992 

33992 
33992 
33992 
33992 
33992 
33992 

species
11
11

33991 
33991 
 33991 


35791 
 35791 
1
species
1

21
 35790 


35790 

35790 
11
species

3


786 
 786 
1
species

149

33989 

33989 
33989 
 33989 
species
111

5113111
 47589 

47589 
47589 
47589 
47589 
47589 
47589 
species
111111

1
species
17

35792 
 35792 

1111
species
4211
 35794 


35794 

35794 
35794 
35794 


35788 
35788 
35788 
35788 
35788 
 35788 
25312
species
11111

1
 337479 


337479 
1
species

species group
212111
8512243
114292

species
111111
7510243

782 
782 
782 
782 
782 
782 
 782 

species
11
12

785 

785 
 785 

1129742
1643212
species group
212111

111111
species
542212

33990 
33990 
33990 
33990 
33990 
33990 
 33990 

 788 

788 

788 
111
species
11

69474
614251621
111111
genus

 784 

784 
784 
784 
784 
784 
784 
614251621
species
111111

32


1528098 
 1528098 
species
1

family
11
1328881
21

genus
11
1802983
21

 1802984 


1802984 
1802984 
21
11
species

942
104131223172555
9910659
family

37112
768
12112
genus

1
 770 


770 
1
species

3611
 769 

769 
769 
769 


769 
1111
species

species group
11
11
106179

11
species
 948 


948 

948 
11

443323
genus
943
49593513512

species
1
1
 391036 


391036 

49593413512
106178
species group
442323

species
11111
 779 

779 
779 

779 
779 
779 
103312


35795 
35795 
 35795 
11
species
11


945 
945 
945 
945 

945 
 945 
14742
species
11111

111111
species

944 
944 
944 
944 
944 
944 
 944 
375127648

526218732041
952
426234
tribe

426234
genus
953
526218732041

species
1
1


80849 
 80849 

11
species


66084 


66084 
 66084 
272

2
 246273 


246273 
1
species

226
 163164 

163164 

163164 
11
species

111
species
511

169402 


169402 
169402 
 169402 

111111
species
121910521834
 77038 

77038 
77038 
77038 
77038 
77038 
77038 

334326114

77551 
77551 
77551 
77551 
77551 
77551 
 77551 
species
111111

1
genus
3
33993

species
1
 950 


950 
3

class
123331
1553900
141732121312

213481
14152891012
order
111121

111121
family
213483
14152891012

genus
111121
14152891012
958

species
1
1


453816 
 453816 

111111
species
 959 

959 
959 
959 
959 
959 
959 
1415289912

1221
order
2433
2024979

2313
1652132
family
1111

genus
1111
2313
1652133

2313


97084 
97084 
97084 
97084 
 97084 
1111
species

family
11
263369
12

genus
11
146784
12

11
species
 960 


960 
960 
12

168616051473112214951135
68525
subphylum
899092818275

252732242117
class
38559135922312794
29547

322
235899
order
111

224467
322
family
111

322
191291
111
genus


244787 
244787 
244787 
 244787 
322
species
111

242630242116
order
38258935622312793
213849

family
171923171711
72294
2261131561227930

genus
13141511126
17993119906522
194

1
 497724 

497724 
species
1

1111
species
2221
 824 

824 
824 

824 

824 

11
species
17
 200 


200 

200 

123
 28898 


28898 
28898 
28898 
111
species

 75658 

75658 
75658 
75658 

75658 
2321
1111
species

52221238
 195 

195 
195 
195 
195 
195 
11111
species

21

827 


827 
 827 
11
species

11
species


76517 
76517 
 76517 
12

1111
species
3111
 1244531 


1244531 
1244531 
1244531 
1244531 

 1965231 

1965231 
1965231 
1965231 
1965231 
1965231 
11232
species
11111

11
species


1660064 

1660064 
 1660064 
71


198 
198 
198 
 198 
221
111
species

 522485 


522485 
1
species
1

111
species
 374106 

374106 
374106 


374106 
211

species
1
6


1500960 
 1500960 

5121


199 
199 
199 
199 
 199 
1111
species

71911
 201 

201 
201 
201 

201 
201 
species
11111

 197 

197 
197 
197 
197 
197 
197 
1394954454011
species
111111

11
species
54

488546 

488546 
 488546 

 1660074 


1660074 
1660074 
11
species
11

species
11
11


1660073 
1660073 
 1660073 


206 
 206 
2
species
1

957911
 196 

196 
196 
196 
196 
196 
196 
species
111111

1


1031542 
 1031542 
1
species

4317262385
28196
234222
genus

 28199 

28199 
28199 
28199 
28199 
28199 
28199 
133122
111111
species

13


944547 
944547 
 944547 
11
species

 1850254 


1850254 
5
1
species

species
111111
 28197 

28197 
28197 
28197 
28197 
28197 
28197 
4213152263

224433
genus
4311963
57665

species
11
31


1986224 


1986224 
 1986224 


44674 

44674 

44674 
44674 
 44674 
1111
1111
species

31222

65553 
65553 
65553 
65553 
65553 
 65553 
species
11111

 1581011 


1581011 
1
1
species

species
1
5
 66821 


66821 

 366522 


366522 

366522 
11
species
11

253


220622 

220622 
220622 
 220622 
species
111

269260
287568
111111
genus

287568
 269261 

269261 
269261 
269261 
269261 
269261 
269261 
111111
species

family
666634
154468193964255
72293

211111
genus
482211
202746

282211
 202747 

202747 
202747 
202747 
202747 
202747 
202747 
species
111111

species
1
 39766 

39766 
2

7
843
genus
1

7
 844 


844 
species
1

11
genus
101
286130

species
11
101
 148813 


148813 

148813 

150450191864154
209
445323
genus


213 
213 
213 
213 
 213 
24138
species
1111

111111
species

210 
210 
210 
210 
210 
210 
 210 
141435175723348

species
11

214 


214 
 214 
13

5
 32025 

32025 
species
1

 135569 


135569 
135569 
21
11
species

species
1
1
 1591088 


1591088 

species
11111
 138563 

138563 
138563 
138563 
138563 

138563 
3111013

11
genus
11
265570

11
 206403 


206403 


206403 
species
11

class
646360576158
28221
13011014111489913681041

order
111111
219692110
453227

family
111111
453228
219692110

111111
genus
453229
219692110

species
111111
 453230 

453230 
453230 
453230 
453230 
453230 
453230 
219692110

order
111111
1779134
391439

391439
1779135
family
111111

391439
1779136
genus
111111

species
111111
 1548548 

1548548 
1548548 
1548548 
1548548 
1548548 
1548548 
391439

order
11111
22191
213113

11111
family
117942
22191

11111
genus
84404
22191

22191
 84405 

84405 
84405 
84405 
84405 
84405 
11111
species

213115
309382568316256158
order
161615141616

213117
121224
family
111111

genus
111111
45662
121224

121224

45663 
45663 
45663 
45663 
45663 
45663 
 45663 
111111
species

family
131312111313
194924
297369557307219135

genus
111111
7914482171114
41707

species
111111
7914482171114

29546 
29546 
29546 
29546 
29546 
29546 
 29546 

genus
1099899
20420046627214492
872

11

1725232 


1725232 
 1725232 
11
species

species
111111
982228
 58180 

58180 
58180 
58180 
58180 
58180 
58180 

species
111111

631220 
631220 
631220 
631220 
631220 
631220 
 631220 
517715

species
11111

901 
901 
901 

901 
901 
 901 
11253

11111
species

880 
880 
880 

880 
880 
 880 
104144

 881 

881 
881 
881 
881 
881 
881 
437219216229
species
111111

 44742 

44742 
44742 
44742 
44742 
44742 
44742 
90984142232925
species
111111

8218256
 873 

873 
873 
873 
873 
873 
873 
111111
species

111111
species
15211869
 876 

876 
876 
876 
876 
876 
876 

species
111111
 184917 

184917 
184917 
184917 
184917 
184917 
184917 
221292103

genus
232233
2035811
14259186429

species
111111

1716143 
1716143 
1716143 
1716143 
1716143 
1716143 
 1716143 
11174114812

 182210 

182210 
182210 
182210 
182210 
182210 
182210 
37571514
species
111111

113
 57320 


57320 


57320 
57320 
111
species

213116
11111073519
222222
family

222222
genus
11111073519
898

5382617
 132132 

132132 
132132 
132132 
132132 
132132 
132132 
species
111111

111111
species
 899 

899 
899 
899 
899 
899 
899 
6825292

order
171616171615
69541
207138103113196174

665666
family
213421
615437506785

302413122115
890
222222
genus

species
111111
13851129
 1823759 

1823759 
1823759 
1823759 
1823759 
1823759 
1823759 


1603606 
1603606 
1603606 
1603606 
1603606 
1603606 
 1603606 
171681196
111111
species

313024384670
18
genus
443444

511033

1842532 
1842532 

1842532 
1842532 
1842532 
 1842532 
11111
species

 29542 

29542 
29542 
29542 
29542 
29542 
29542 
759162228
species
111111

 19 

19 
19 
19 
19 
19 
19 
9811322
species
111111

1016491937

29543 
29543 
29543 
29543 
29543 
29543 
 29543 
species
111111

213422
14684666312989
11101111109
family

genus
111111
392332
1332321


483547 
483547 
483547 
483547 
483547 
483547 
 483547 
1332321
species
111111

28231
13381646012788
109101098
genus

11111
species
1013228

28232 

28232 
28232 
28232 
28232 
 28232 


1340425 
1340425 
1340425 
1340425 
1340425 
1340425 
 1340425 
18886265
111111
species

2344101915

443143 
443143 
443143 
443143 
443143 
443143 
 443143 
species
111111

species
111111
1938361420

35554 
35554 
35554 
35554 
35554 
35554 
 35554 

111111
species
8574231

351604 
351604 
351604 
351604 
351604 
351604 
 351604 

1246181111
 225194 

225194 
225194 
225194 
225194 
225194 
225194 
111111
species

species
111111

345632 
345632 
345632 
345632 
345632 
345632 
 345632 
81103722

 443144 

443144 
443144 
443144 
443144 
443144 
443144 
32458176
species
111111

2111
 1203471 

1203471 
1203471 
1203471 
1203471 
1111
species

species
11111

313985 
313985 
313985 
313985 
313985 
 313985 
116728

1551504
555
genus
111

 673862 

673862 
673862 
673862 
555
111
species

order
132112
4142642
213462

213468
4161
2111
family

11
genus
16
60892

16
 60893 


60893 

60893 
11
species

3
43773
genus
1

3


316277 
 316277 
species
1

2357
11
genus
11

11


2358 


2358 
 2358 
11
species

11111
family
213465
410141

29526
410141
genus
11111

410141
 119484 

119484 
119484 
119484 

119484 
119484 
species
11111

865475
order
412419326563
213118

family
422123
1155489
213121

7451
427922
genus
1111

species
1111

427923 


427923 
427923 
427923 
 427923 
7451

1221
109168
genus
1111

species
1111

84980 
84980 
84980 


84980 
 84980 
1221

11111
genus
23337
893

23337
 1986146 

1986146 
1986146 
1986146 

1986146 
1986146 
11111
species

53318
1
genus
1

1
species
1

65555 
 65555 

213119
301914285754
family
443352

131313214446
896
222221
genus

11111
species
11396

181663 
181663 
181663 
181663 
181663 
 181663 

111111
species
121210123846
 897 

897 
897 
897 
897 
897 
897 

11111
genus
218207
135798

11111
species
 259354 

259354 
259354 

259354 
259354 
259354 
135798

1
genus
28222
1

1


28223 
 28223 
1
species

genus
1111
2295
4113

4113
 2296 

2296 
2296 
2296 

2296 
species
1111

709431409410822625
29
181818181818
order

80812
153126137102183189
suborder
333333

family
222222
12911810786145146
49

111111
genus
1141096967128124
39643

1141096967128124

56 
56 
56 
56 
56 
56 
 56 
species
111111

111111
genus
50
15938191722

species
111111
15938191722

52 
52 
52 
52 
52 
52 
 52 

family
111111
24830163843
1055686

1055688
24830163843
genus
111111

111111
species
 927083 

927083 
927083 
927083 
927083 
927083 
927083 
24830163843

suborder
111111
224462
582133464528

582133464528
224464
111111
family

genus
111111
162027
582133464528


80816 
80816 
80816 
80816 
80816 
80816 
 80816 
582133464528
species
111111

80811
498284239262594408
suborder
141414141414

143757229151121
1524215
family
333333

161492
143757229151121
333333
genus


447217 
447217 
447217 
447217 
447217 
447217 
 447217 
43101061315
species
111111

species
111111
26142032814
 404589 

404589 
404589 
404589 
404589 
404589 
404589 

7451422011092

161493 
161493 
161493 
161493 
161493 
161493 
 161493 
species
111111

1524213
10424154
111111
family

genus
111111
10424154
1524214

species
111111
10424154

1391653 
1391653 
1391653 
1391653 
1391653 
1391653 
 1391653 

11283575215490
39
444444
family

42
321614247015
genus
111111

species
111111
321614247015
 43 

43 
43 
43 
43 
43 
43 

44
411869257
genus
111111

species
111111

83453 
83453 
83453 
83453 
83453 
83453 
 83453 
411869257

14431873241
40
111111
genus

14431873241
 41 

41 
41 
41 
41 
41 
41 
111111
species

47
25619122727
genus
111111

111111
species
 48 

48 
48 
48 
48 
48 
48 
25619122727

family
666666
233122108177274193
31

391124312321
83461
genus
111111

111111
species
 184914 

184914 
184914 
184914 
184914 
184914 
184914 
391124312321

32
19411184146251172
555555
genus

6065307011871
 33 

33 
33 
33 
33 
33 
33 
species
111111

species
111111
 83455 

83455 
83455 
83455 
83455 
83455 
83455 
2587323731

species
111111

1297742 
1297742 
1297742 
1297742 
1297742 
1297742 
 1297742 
37144154237

species
111111
191835153117

34 
34 
34 
34 
34 
34 
 34 

5368142316
 35 

35 
35 
35 
35 
35 
35 
species
111111

28216
82356672014034152178126536173578
class
265261266264263261

111111
species
673027326190
 1904640 

1904640 
1904640 
1904640 
1904640 
1904640 
1904640 

order
161616161616
136762074791019972557
206389

family
333333
18488102138310326
75787

146937
67455887115161
111111
genus

species
111111
 146939 

146939 
146939 
146939 
146939 
146939 
146939 
67455887115161

111111
species
251413209918
 1898103 

1898103 
1898103 
1898103 
1898103 
1898103 
1898103 

111111
genus
551759
9229313196147

species
111111
 551760 

551760 
551760 
551760 
551760 
551760 
551760 
9229313196147

family
121212121212
2008794
116549961370716312128

12960
6012793073468591157
666666
genus


41977 
41977 
41977 
41977 
41977 
41977 
 41977 
135948578228184
111111
species

8728395279174
 748247 

748247 
748247 
748247 
748247 
748247 
748247 
species
111111

97364059169239
 418699 

418699 
418699 
418699 
418699 
418699 
418699 
111111
species

species
111111
7328434691160

198107 
198107 
198107 
198107 
198107 
198107 
 198107 

111111
species
124474850188206
 62928 

62928 
62928 
62928 
62928 
62928 
62928 

85465261104194

2067960 
2067960 
2067960 
2067960 
2067960 
2067960 
 2067960 
111111
species

genus
555555
33057
542214292341748913

111111
species
105275753149166
 59405 

59405 
59405 
59405 
59405 
59405 
59405 

species
111111

1134435 
1134435 
1134435 
1134435 
1134435 
1134435 
 1134435 
111265465171185


2005884 
2005884 
2005884 
2005884 
2005884 
2005884 
 2005884 
105405988114149
111111
species

111111
species
 96773 

96773 
96773 
96773 
96773 
96773 
96773 
123636366162184

 85643 

85643 
85643 
85643 
85643 
85643 
85643 
98585969152229
111111
species

22614202458
 2080469 

2080469 
2080469 
2080469 
2080469 
2080469 
2080469 
species
111111

111111
family
1833326556103
2008795

1833326556103
73029
111111
genus

111111
species

259537 
259537 
259537 
259537 
259537 
259537 
 259537 
1833326556103

genus
1111
1381133
5412

1111
species

669502 
669502 
669502 
669502 
 669502 
5412

11111
genus
1010111
1301080


1160784 
1160784 
1160784 

1160784 
1160784 
 1160784 
1010111
species
11111

species
1111
3155

543913 


543913 
543913 
543913 
 543913 

80840
78388650843822749364121353167086
order
195194197194195195

212743
448222204159536818
genus
111111

species
111111

946333 
946333 
946333 
946333 
946333 
946333 
 946333 
448222204159536818

family
242424242424
277733942710651162045118978191
75682

846
2310572076
111111
genus

species
111111
2310572076
 847 

847 
847 
847 
847 
847 
847 

genus
555555
263012511241157435316135
963

 80842 

80842 
80842 
80842 
80842 
80842 
80842 
298134169198363702
species
111111

 92645 

92645 
92645 
92645 
92645 
92645 
92645 
4282012232506301079
species
111111


341045 
341045 
341045 
341045 
341045 
341045 
 341045 
338181145163450785
species
111111

species
111111

2025949 
2025949 
2025949 
2025949 
2025949 
2025949 
 2025949 
16710186130205430

139963461883318833139
 964 

964 
964 
964 
964 
964 
964 
species
111111

193855751705592084001356669
149698
genus
777777

111111
species

1678028 
1678028 
1678028 
1678028 
1678028 
1678028 
 1678028 
111262342467216653202

species
111111
 2072590 

2072590 
2072590 
2072590 
2072590 
2072590 
2072590 
128955549470819604290

111111
species
155272661484023694695

2045208 
2045208 
2045208 
2045208 
2045208 
2045208 
 2045208 

 47229 

47229 
47229 
47229 
47229 
47229 
47229 
95391906335842482532127002
species
111111

 1593482 

1593482 
1593482 
1593482 
1593482 
1593482 
1593482 
123262346360618423871
species
111111

111111
species

1707785 
1707785 
1707785 
1707785 
1707785 
1707785 
 1707785 
2333712856110236727155

2328606846103231846454

1141883 
1141883 
1141883 
1141883 
1141883 
1141883 
 1141883 
111111
species

genus
222222
1727885125227404
303379

 204773 

204773 
204773 
204773 
204773 
204773 
204773 
123546495123231
species
111111


1809410 
1809410 
1809410 
1809410 
1809410 
1809410 
 1809410 
49242130104173
111111
species

666666
genus
29580
39303127915584258489410402

species
111111

55508 
55508 
55508 
55508 
55508 
55508 
 55508 
86489328557310411979

species
111111
97910584448129912692749
 1644131 

1644131 
1644131 
1644131 
1644131 
1644131 
1644131 

species
111111
69383239125149

375286 
375286 
375286 
375286 
375286 
375286 
 375286 

111111
species
9779385380120212612645

368607 
368607 
368607 
368607 
368607 
368607 
 368607 


1236179 
1236179 
1236179 
1236179 
1236179 
1236179 
 1236179 
121015131210
species
111111

species
111111
102910369398113211862870
 1938606 

1938606 
1938606 
1938606 
1938606 
1938606 
1938606 

202907
16331058707103225044505
333333
genus

species
111111
 279058 

279058 
279058 
279058 
279058 
279058 
279058 
5463572333347921561

5153592533388421499
 279113 

279113 
279113 
279113 
279113 
279113 
279113 
species
111111

 158899 

158899 
158899 
158899 
158899 
158899 
158899 
5723422213608701445
species
111111

622127084724318975219513
80864
family
424242424242

genus
111111
174951
243126136118275301

111111
species

94132 
94132 
94132 
94132 
94132 
94132 
 94132 
243126136118275301

genus
111111
352450
7119383283156

111111
species
 2109915 

2109915 
2109915 
2109915 
2109915 
2109915 
2109915 
7119383283156

361223103565
665874
genus
222222

species
111111

1678129 
1678129 
1678129 
1678129 
1678129 
1678129 
 1678129 
93103115

 1678128 

1678128 
1678128 
1678128 
1678128 
1678128 
1678128 
2791372460
111111
species

364316
102394658131218
111111
genus


364317 
364317 
364317 
364317 
364317 
364317 
 364317 
102394658131218
111111
species

genus
111111
23913223698
1436289

23913223698

1436290 
1436290 
1436290 
1436290 
1436290 
1436290 
 1436290 
species
111111

52972
17497111142247378
222222
genus

71414346123147
 296591 

296591 
296591 
296591 
296591 
296591 
296591 
111111
species

111111
species
103566896124231

216465 
216465 
216465 
216465 
216465 
216465 
 216465 

238749
104795365125149
genus
111111

species
111111
104795365125149

1546149 
1546149 
1546149 
1546149 
1546149 
1546149 
 1546149 

12916
133467463072016372277
genus
888888

84473337149187
 721785 

721785 
721785 
721785 
721785 
721785 
721785 
111111
species

111111
species
336177161152360554

80869 
80869 
80869 
80869 
80869 
80869 
 80869 

species
111111
 80867 

80867 
80867 
80867 
80867 
80867 
80867 
159868383186218

species
111111
103583937151120
 358220 

358220 
358220 
358220 
358220 
358220 
358220 

species
111111

1842533 
1842533 
1842533 
1842533 
1842533 
1842533 
 1842533 
139445555161183

 232721 

232721 
232721 
232721 
232721 
232721 
232721 
8328244498135
111111
species


553814 
553814 
553814 
553814 
553814 
553814 
 553814 
338191184252454702
111111
species

 1858609 

1858609 
1858609 
1858609 
1858609 
1858609 
1858609 
9243516078178
species
111111

2259995124278382
201096
111111
genus

species
111111
2259995124278382
 179636 

179636 
179636 
179636 
179636 
179636 
179636 

222222
genus
1649468
1937695124319339

111111
species
85333864208162
 2116657 

2116657 
2116657 
2116657 
2116657 
2116657 
2116657 

108435760111177
 2109913 

2109913 
2109913 
2109913 
2109913 
2109913 
2109913 
111111
species

333333
genus
269130148159382420
47420

111111
species

1763535 
1763535 
1763535 
1763535 
1763535 
1763535 
 1763535 
5725455598151

species
111111
11639644614798

795665 
795665 
795665 
795665 
795665 
795665 
 795665 

111111
species
 1842537 

1842537 
1842537 
1842537 
1842537 
1842537 
1842537 
96663958137171

444444
genus
63533221313608251047
80865

111111
species

742013 
742013 
742013 
742013 
742013 
742013 
 742013 
1738559786199231

111111
species

80866 
80866 
80866 
80866 
80866 
80866 
 80866 
16362590140196231

111111
species
1429832666221280

180282 
180282 
180282 
180282 
180282 
180282 
 180282 

species
111111
1578761868209305
 1920191 

1920191 
1920191 
1920191 
1920191 
1920191 
1920191 

283
401195234212648787
genus
333333

species
111111
551933267293

225992 
225992 
225992 
225992 
225992 
225992 
 225992 

species
111111
262145148129456584
 285 

285 
285 
285 
285 
285 
285 

111111
species

1082851 
1082851 
1082851 
1082851 
1082851 
1082851 
 1082851 
84315357120110

116519687120271
219181
222222
genus

10042857099250
 2109914 

2109914 
2109914 
2109914 
2109914 
2109914 
2109914 
111111
species

111111
species
 1658672 

1658672 
1658672 
1658672 
1658672 
1658672 
1658672 
16911172121

444444
genus
263139118150438414
28065

 1842727 

1842727 
1842727 
1842727 
1842727 
1842727 
1842727 
134815168219153
111111
species

111111
species

1484693 
1484693 
1484693 
1484693 
1484693 
1484693 
 1484693 
4616294093112

111111
species

192843 
192843 
192843 
192843 
192843 
192843 
 192843 
6424232789119

 81479 

81479 
81479 
81479 
81479 
81479 
81479 
191815153730
111111
species

195558670875018502001
34072
444444
genus

111111
species
487112161170432430

2126319 
2126319 
2126319 
2126319 
2126319 
2126319 
 2126319 

species
111111
1706280114211329

1795631 
1795631 
1795631 
1795631 
1795631 
1795631 
 1795631 

958296327333869841

34073 
34073 
34073 
34073 
34073 
34073 
 34073 
111111
species

111111
species
 436515 

436515 
436515 
436515 
436515 
436515 
436515 
340116140133338401

species
111111

1458426 
1458426 
1458426 
1458426 
1458426 
1458426 
 1458426 
292429224674

4821203446136

1458425 
1458425 
1458425 
1458425 
1458425 
1458425 
 1458425 
111111
species

1776662108181283
 413882 

413882 
413882 
413882 
413882 
413882 
413882 
111111
species

111111
species

1469502 
1469502 
1469502 
1469502 
1469502 
1469502 
 1469502 
321311182778

111111
genus
65047
172839391230284

species
111111
172839391230284
 1658665 

1658665 
1658665 
1658665 
1658665 
1658665 
1658665 

88
126626678166299
genus
111111

126626678166299

34029 
34029 
34029 
34029 
34029 
34029 
 34029 
111111
species

97575442201234
316612
genus
111111

species
111111
97575442201234

105560 
105560 
105560 
105560 
105560 
105560 
 105560 

120556760155242
93681
genus
111111

 76731 

76731 
76731 
76731 
76731 
76731 
76731 
120556760155242
species
111111

98555238158193
92793
111111
genus

111111
species
 1296669 

1296669 
1296669 
1296669 
1296669 
1296669 
1296669 
98555238158193

111111
genus
318147
11140373793133

11140373793133

1768242 
1768242 
1768242 
1768242 
1768242 
1768242 
 1768242 
111111
species

32012
8152394693188
111111
genus

8152394693188
 926 

926 
926 
926 
926 
926 
926 
species
111111

506
146528558806698031975324470
313031313130
family

111111
genus
103538577146134
1921582

species
111111
103538577146134

1851544 
1851544 
1851544 
1851544 
1851544 
1851544 
 1851544 

genus
111111
97578996144131
507

species
111111

511 
511 
511 
511 
511 
511 
 511 
97578996144131

111111
genus
112152
90243


90245 
90245 
90245 
90245 
90245 
90245 
 90245 
112152
111111
species

genus
11
22
1100891

22
 643674 

643674 

643674 
species
11

genus
222222
272022203010
290425


310575 
310575 
310575 
310575 
310575 
310575 
 310575 
2210136187
species
111111

 302406 

302406 
302406 
302406 
302406 
302406 
302406 
510914123
111111
species

171716171717
genus
517
118446909633778321562619739

111111
species
81433738137216

94624 
94624 
94624 
94624 
94624 
94624 
 94624 

111111
species
 519 

519 
519 
519 
519 
519 
519 
281121141179338307

93403921111162

1331258 
1331258 
1331258 
1331258 
1331258 
1331258 
 1331258 
species
111111

species
111111

463040 
463040 
463040 
463040 
463040 
463040 
 463040 
121575747184220

species
111111
194189124122278277

103855 
103855 
103855 
103855 
103855 
103855 
 103855 

species
111111
137745775161245
 123899 

123899 
123899 
123899 
123899 
123899 
123899 


1416806 
1416806 
1416806 
1416806 
1416806 
1416806 
 1416806 
70354890145161
111111
species

species
111111

463014 
463014 
463014 
463014 
463014 
463014 
 463014 
4530333992123

480332293282653888

35814 
35814 
35814 
35814 
35814 
35814 
 35814 
111111
species

111111
species
 521 

521 
521 
521 
521 
521 
521 
33111566910

216119125172333295
 463025 

463025 
463025 
463025 
463025 
463025 
463025 
111111
species

species
111111
 520 

520 
520 
520 
520 
520 
520 
95095519503463561219015815

5425222076134

463024 
463024 
463024 
463024 
463024 
463024 
 463024 
species
111111


1697043 
1697043 
1697043 
1697043 
1697043 
1697043 
 1697043 
392325157187
111111
species


1416803 
1416803 
1416803 
1416803 
1416803 
1416803 
 1416803 
138775560210234
111111
species

111111
species
345213232308566561
 518 

518 
518 
518 
518 
518 
518 

812124
 2163011 

2163011 
2163011 

2163011 
2163011 
2163011 
species
11111

genus
111
811
1472344


1472345 
1472345 
1472345 
 1472345 
811
111
species

genus
555555
241514401451168435774187
222

175133119118367362

217204 
217204 
217204 
217204 
217204 
217204 
 217204 
species
111111

 85698 

85698 
85698 
85698 
85698 
85698 
85698 
1610936993110223052689
111111
species

111111
species
236134142153350390

217203 
217203 
217203 
217203 
217203 
217203 
 217203 

species
111111

1758194 
1758194 
1758194 
1758194 
1758194 
1758194 
 1758194 
1257044101180263

111111
species
269167153210375483
 32002 

32002 
32002 
32002 
32002 
32002 
32002 

25139285536
305976
genus
111111

 1007105 

1007105 
1007105 
1007105 
1007105 
1007105 
1007105 
25139285536
111111
species

122405060147193
359336
genus
111111

species
111111
122405060147193

75697 
75697 
75697 
75697 
75697 
75697 
 75697 

111111
genus
16251142238
29574

111111
species
16251142238
 29575 

29575 
29575 
29575 
29575 
29575 
29575 

11
 1834205 


1834205 

1834205 
species
11

119060
281071361614033194134088551834
878788868688
family

genus
444444
48736
5132238723432792666510790

 305 

305 
305 
305 
305 
305 
305 
421919271943223955158943
species
111111

111111
species
240132125174349621
 190721 

190721 
190721 
190721 
190721 
190721 
190721 


329 
329 
329 
329 
329 
329 
 329 
409190150205495739
111111
species

species
111111
 105219 

105219 
105219 
105219 
105219 
105219 
105219 
264138125174306487

genus
111111
157932
2914849722265955581319

 29443 

29443 
29443 
29443 
29443 
29443 
29443 
2914849722265955581319
111111
species

genus
999999
17629721119117424383775
106589

377201245246522738

106590 
106590 
106590 
106590 
106590 
106590 
 106590 
species
111111


367825 
367825 
367825 
367825 
367825 
367825 
 367825 
172115107101198319
species
111111

200138131146334672
 119219 

119219 
119219 
119219 
119219 
119219 
119219 
111111
species

 248026 

248026 
248026 
248026 
248026 
248026 
248026 
1907095132258494
111111
species

111111
species

2036817 
2036817 
2036817 
2036817 
2036817 
2036817 
 2036817 
1378192152214296

111111
species

68895 
68895 
68895 
68895 
68895 
68895 
 68895 
16510511597212243

1539492111215366

1389192 
1389192 
1389192 
1389192 
1389192 
1389192 
 1389192 
111111
species


82541 
82541 
82541 
82541 
82541 
82541 
 82541 
1627110386207304
111111
species

20697139103278343

876364 
876364 
876364 
876364 
876364 
876364 
 876364 
species
111111

1822464
1695762780125523363248
121212121212
genus

species
111111
9747447176143
 134537 

134537 
134537 
134537 
134537 
134537 
134537 

111111
species

2026199 
2026199 
2026199 
2026199 
2026199 
2026199 
 2026199 
1146831112222172

96524549115100
 948107 

948107 
948107 
948107 
948107 
948107 
948107 
111111
species

111111
species
 252970 

252970 
252970 
252970 
252970 
252970 
252970 
120445454166201

102314278133160
 148447 

148447 
148447 
148447 
148447 
148447 
148447 
species
111111

 75105 

75105 
75105 
75105 
75105 
75105 
75105 
463203225404626923
111111
species

90475287117225

261302 
261302 
261302 
261302 
261302 
261302 
 261302 
species
111111

species
111111
88293267150198
 1926494 

1926494 
1926494 
1926494 
1926494 
1926494 
1926494 

85475749129132
 169430 

169430 
169430 
169430 
169430 
169430 
169430 
species
111111

 412963 

412963 
412963 
412963 
412963 
412963 
412963 
5822151445147
species
111111

 36873 

36873 
36873 
36873 
36873 
36873 
36873 
251143121159416565
species
111111

 311230 

311230 
311230 
311230 
311230 
311230 
311230 
1312962111141282
species
111111

genus
999999
93217
1527703859103923483541


93220 
93220 
93220 
93220 
93220 
93220 
 93220 
5032292793417631250
111111
species


93222 
93222 
93222 
93222 
93222 
93222 
 93222 
1928599136289402
species
111111

species
111111
 656178 

656178 
656178 
656178 
656178 
656178 
656178 
65404463135207

111111
species
 93218 

93218 
93218 
93218 
93218 
93218 
93218 
321128162211421711


93221 
93221 
93221 
93221 
93221 
93221 
 93221 
58565860129184
species
111111

111111
species
 656179 

656179 
656179 
656179 
656179 
656179 
656179 
70393932121166

species
111111

93219 
93219 
93219 
93219 
93219 
93219 
 93219 
96384647130131

82314471166233
 573737 

573737 
573737 
573737 
573737 
573737 
573737 
111111
species

 445709 

445709 
445709 
445709 
445709 
445709 
445709 
140578878194257
111111
species

431025282070
44013
genus
334224

111111
species

576611 
576611 
576611 
576611 
576611 
576611 
 576611 
33717241964

1111
species

576610 
576610 


576610 
576610 
 576610 
4214

6


1835254 
 1835254 
1
species

 556054 


556054 
556054 

556054 
141
111
species

species
1111
 1743172 

1743172 
1743172 
1743172 


1743172 
6111

494949494949
genus
1503479338185104662152029091
32008

111111
species
447192253356625914
 337 

337 
337 
337 
337 
337 
337 


758796 
758796 
758796 
758796 
758796 
758796 
 758796 
87605566144242
species
111111


1795043 
1795043 
1795043 
1795043 
1795043 
1795043 
 1795043 
57563538140134
111111
species

111111
species
411624346990
 1468409 

1468409 
1468409 
1468409 
1468409 
1468409 
1468409 

species
111111
74440139458011401153
 28095 

28095 
28095 
28095 
28095 
28095 
28095 

111111
species

1740163 
1740163 
1740163 
1740163 
1740163 
1740163 
 1740163 
12029304088113

84445062170230

2217913 
2217913 
2217913 
2217913 
2217913 
2217913 
 2217913 
111111
species

 758793 

758793 
758793 
758793 
758793 
758793 
758793 
108536974157177
species
111111

111111
species

1678678 
1678678 
1678678 
1678678 
1678678 
1678678 
 1678678 
80694280141269

111111
species
 640512 

640512 
640512 
640512 
640512 
640512 
640512 
7539204393119

6265343435404491933812575
87882
222222222222
species group

 95485 

95485 
95485 
95485 
95485 
95485 
95485 
1488562103234260
111111
species

species
111111
5416297393132

1637869 
1637869 
1637869 
1637869 
1637869 
1637869 
 1637869 

111111
species

101571 
101571 
101571 
101571 
101571 
101571 
 101571 
5673143873828471205


488731 
488731 
488731 
488731 
488731 
488731 
 488731 
56393460129172
species
111111

species
111111
18609691001127627783702
 95486 

95486 
95486 
95486 
95486 
95486 
95486 


488729 
488729 
488729 
488729 
488729 
488729 
 488729 
8154545697205
111111
species


1637862 
1637862 
1637862 
1637862 
1637862 
1637862 
 1637862 
70473577185126
111111
species

111111
species

1503054 
1503054 
1503054 
1503054 
1503054 
1503054 
 1503054 
110575491220316

69383448134154

488447 
488447 
488447 
488447 
488447 
488447 
 488447 
species
111111

6335424993189
 1503055 

1503055 
1503055 
1503055 
1503055 
1503055 
1503055 
species
111111

48332657107143

488732 
488732 
488732 
488732 
488732 
488732 
 488732 
111111
species


152480 
152480 
152480 
152480 
152480 
152480 
 152480 
313119122154406378
111111
species

111111
species
6530505191192
 152500 

152500 
152500 
152500 
152500 
152500 
152500 

species
111111

1637853 
1637853 
1637853 
1637853 
1637853 
1637853 
 1637853 
7313295984165


60552 
60552 
60552 
60552 
60552 
60552 
 60552 
6113183184458831205
species
111111

97466368863713741737
 292 

292 
292 
292 
292 
292 
292 
111111
species

111111
species
 482957 

482957 
482957 
482957 
482957 
482957 
482957 
17710195195265397

species
111111
83264365142184

265293 
265293 
265293 
265293 
265293 
265293 
 265293 

species
111111
 60550 

60550 
60550 
60550 
60550 
60550 
60550 
68353258118125

species
111111
 488446 

488446 
488446 
488446 
488446 
488446 
488446 
622237858279

111111
species
6313523243878801339
 87883 

87883 
87883 
87883 
87883 
87883 
87883 

111111
species
8268448396170
 1207504 

1207504 
1207504 
1207504 
1207504 
1207504 
1207504 

111527
6256313132974159846611892
999999
species group

106352954059413521970
 57975 

57975 
57975 
57975 
57975 
57975 
57975 
species
111111


1385591 
1385591 
1385591 
1385591 
1385591 
1385591 
 1385591 
661229323451
111111
species

111111
species

1637831 
1637831 
1637831 
1637831 
1637831 
1637831 
 1637831 
423127185439


1385592 
1385592 
1385592 
1385592 
1385592 
1385592 
 1385592 
572919255092
species
111111

species
111111
 28450 

28450 
28450 
28450 
28450 
28450 
28450 
419620912221296557128165

5572883393278751043

13373 
13373 
13373 
13373 
13373 
13373 
 13373 
species
111111

species
111111

1637841 
1637841 
1637841 
1637841 
1637841 
1637841 
 1637841 
412230166249

 342113 

342113 
342113 
342113 
342113 
342113 
342113 
20010078163286377
111111
species

 1637837 

1637837 
1637837 
1637837 
1637837 
1637837 
1637837 
3429141941106
species
111111

111111
species
76333950142143
 416344 

416344 
416344 
416344 
416344 
416344 
416344 

118577467196208
 41899 

41899 
41899 
41899 
41899 
41899 
41899 
111111
species

99594490148211

640510 
640510 
640510 
640510 
640510 
640510 
 640510 
111111
species

472724223843

1740162 
1740162 
1740162 
1740162 
1740162 
1740162 
 1740162 
111111
species

species
111111
89585056121112
 1804984 

1804984 
1804984 
1804984 
1804984 
1804984 
1804984 

594151367382
 1795874 

1795874 
1795874 
1795874 
1795874 
1795874 
1795874 
species
111111

79734765121226

640511 
640511 
640511 
640511 
640511 
640511 
 640511 
species
111111

species
111111

1097668 
1097668 
1097668 
1097668 
1097668 
1097668 
 1097668 
103614757110158

28067
173706778164326
genus
111111

 28068 

28068 
28068 
28068 
28068 
28068 
28068 
173706778164326
species
111111

genus
333324
152974414
33055

11

994695 


994695 
 994695 
species
11

21


994696 


994696 
 994696 
species
11

species
111111

233181 
233181 
233181 
233181 
233181 
233181 
 233181 
541228

11
 1576550 


1576550 
1576550 
11
species

9235124
 33056 

33056 
33056 
33056 
33056 
33056 
33056 
111111
species

111111
genus
342118214457
327159


327160 
327160 
327160 
327160 
327160 
327160 
 327160 
342118214457
species
111111

206351
190510841064155625242902
order
242226252623

family
777777
93552347165613101547
1499392

111111
genus
168470
114505481162248

species
111111

168471 
168471 
168471 
168471 
168471 
168471 
 168471 
114505481162248

535
544329310356817910
333333
genus


1108595 
1108595 
1108595 
1108595 
1108595 
1108595 
 1108595 
189167118148296319
111111
species

 2059672 

2059672 
2059672 
2059672 
2059672 
2059672 
2059672 
82605853175174
species
111111


536 
536 
536 
536 
536 
536 
 536 
273102134155346417
111111
species

1014634113120169
568394
genus
111111

1014634113120169
 748280 

748280 
748280 
748280 
748280 
748280 
748280 
111111
species

genus
111111
187
6046306110999

6046306110999

1938604 
1938604 
1938604 
1938604 
1938604 
1938604 
 1938604 
species
111111

116524345102121
885864
111111
genus

species
111111
 1906741 

1906741 
1906741 
1906741 
1906741 
1906741 
1906741 
116524345102121

481
97056159390012141355
171519181916
family

87251051683311081116
482
genus
121212121212

 1853276 

1853276 
1853276 
1853276 
1853276 
1853276 
1853276 
8414410
species
111111

22201591152

495 
495 
495 
495 
495 
495 
 495 
111111
species

species
111111
94379063127167
 485 

485 
485 
485 
485 
485 
485 

111111
species
251264
 28091 

28091 
28091 
28091 
28091 
28091 
28091 

 483 

483 
483 
483 
483 
483 
483 
64411153
species
111111

345124

490 
490 
490 
490 
490 
490 
 490 
111111
species

114681621
 1853278 

1853278 
1853278 
1853278 
1853278 
1853278 
1853278 
species
111111

species
111111

655307 
655307 
655307 
655307 
655307 
655307 
 655307 
16186111529

species
111111
11933364

488 
488 
488 
488 
488 
488 
 488 

661400377687851813
 487 

487 
487 
487 
487 
487 
487 
species
111111

303316172

326523 
326523 
326523 
326523 
326523 
326523 
 326523 
species
111111

species
111111

486 
486 
486 
486 
486 
486 
 486 
8251887

111111
genus
291413254179
538

species
111111
291413254179
 539 

539 
539 
539 
539 
539 
539 

526163
 2052837 

2052837 
2052837 
2052837 
2052837 
2052837 
2052837 
111111
species

111
genus
1193515
914

914
 1196083 


1196083 
1196083 
1196083 
species
111

71
11351
genus
1111

1111
species
 72 

72 

72 
72 
72 
11351

5335363549157
59
212222
genus

species
111111
3935193140139

63 
63 
63 
63 
63 
63 
 63 

11111
species
14174918

96942 

96942 
96942 
96942 
96942 
 96942 

105
32257
genus
11

11
species
105
 504 


504 

504 

222220222019
order
562319249288547866
32003

90627
123648662113170
444444
family

111111
genus
935200
35107194551


649841 
649841 
649841 
649841 
649841 
649841 
 649841 
35107194551
111111
species

genus
111111
1778653
34154171745

species
111111
34154171745

1985873 
1985873 
1985873 
1985873 
1985873 
1985873 
 1985873 

genus
111111
402927193356
314343

species
111111
402927193356

63745 
63745 
63745 
63745 
63745 
63745 
 63745 

96
141011171818
genus
111111

species
111111

370405 
370405 
370405 
370405 
370405 
370405 
 370405 
141011171818

611921175485
2008790
family
111111

111111
genus
919
611921175485

111111
species
611921175485

36861 
36861 
36861 
36861 
36861 
36861 
 36861 

family
222222
2008793
190706465178341

111111
genus
104382333105154
378210

111111
species
 1842540 

1842540 
1842540 
1842540 
1842540 
1842540 
1842540 
104382333105154

8632413273187
1054211
genus
111111

8632413273187
 748811 

748811 
748811 
748811 
748811 
748811 
748811 
species
111111

family
976987
206379
82123345285102

5810818384468
914
654654
genus

species
111111
 261292 

261292 
261292 
261292 
261292 
261292 
261292 
4101312

16416
 44577 

44577 
44577 

44577 
44577 
species
1111

111111
species
 916 

916 
916 
916 
916 
916 
916 
7311453

111111
species
99012192810
 915 

915 
915 
915 
915 
915 
915 

6141153
 153948 

153948 
153948 
153948 
153948 
153948 
153948 
species
111111

species
11
163
 44574 

44574 


44574 

genus
322333
241516144134
35798

11111
species
4631628
 1288494 

1288494 

1288494 
1288494 
1288494 
1288494 

634162
 1231 

1231 
1231 

1231 
1231 
1231 
11111
species

141210794
 35799 

35799 
35799 
35799 
35799 
35799 
35799 
111111
species

687655
family
106434492117168
32011

111111
genus
16
824549

species
111111

1662285 
1662285 
1662285 
1662285 
1662285 
1662285 
 1662285 
824549

121
genus
1679002
641

species
111
611
 1581557 

1581557 
1581557 
1581557 

1
species
 1581680 


1581680 
3

81682
331913586383
222222
genus


266009 
266009 
266009 
266009 
266009 
266009 
 266009 
1486281314
species
111111

19117305069
 887061 

887061 
887061 
887061 
887061 
887061 
887061 
111111
species

404
551513174572
111111
genus

551513174572

405 
405 
405 
405 
405 
405 
 405 
species
111111

genus
122211
359407
43131254

424754

359408 
359408 
359408 
359408 
359408 
359408 
 359408 
111111
species

111
species


1055487 
1055487 
1055487 
 1055487 
195

1236
153567115286861277507194403816197271921325
656633689664641623
class

135615
123
order
111

868
123
111
family

869
123
111
genus

123

870 


870 
870 
 870 
111
species

 186490 

186490 
186490 
186490 
186490 
186490 
186490 
22269113
111111
species

135618
13410294196204151
777777
order

777777
family
403
13410294196204151

416
777948111156127
444444
genus

111111
species
 1727196 

1727196 
1727196 
1727196 
1727196 
1727196 
1727196 
2795224640

65310725

421 
421 
421 
421 
421 
421 
 421 
species
111111

species
111111
 1538553 

1538553 
1538553 
1538553 
1538553 
1538553 
1538553 
133712364217

111111
species
312828436145

702114 
702114 
702114 
702114 
702114 
702114 
 702114 

27614283716
413
111111
genus

111111
species
27614283716

414 
414 
414 
414 
414 
414 
 414 

genus
111111
762296
81394353

species
111111
81394353

1704499 
1704499 
1704499 
1704499 
1704499 
1704499 
 1704499 

genus
111111
224231465
39773

111111
species
 271065 

271065 
271065 
271065 
271065 
271065 
271065 
224231465

1608298
143151
genus
111111


1076588 
1076588 
1076588 
1076588 
1076588 
1076588 
 1076588 
143151
species
111111

7251536
 2169539 

2169539 
2169539 
2169539 
2169539 
2169539 
2169539 
111111
species

1240482
219911
12111
order

12111
family
219911
1240483

1193503
215911
genus
11111

215911
 1196095 

1196095 

1196095 
1196095 
1196095 
1196095 
species
11111

4
1335631
1
genus


1267021 
 1267021 
4
1
species


410330 
410330 
410330 
 410330 
6154
111
species

151516161513
order
1706369
181104188214302194

777776
family
60428210914775
1706371

111111
genus
44420275
316625

44420275
 86304 

86304 
86304 
86304 
86304 
86304 
86304 
species
111111

1061832103
447467
111111
genus

 447471 

447471 
447471 
447471 
447471 
447471 
447471 
1061832103
111111
species

genus
11111
11611
2036021

11611
 1737490 

1737490 
1737490 
1737490 
1737490 
1737490 
11111
species

2425
1531261010
genus
111111

111111
species
1531261010

2426 
2426 
2426 
2426 
2426 
2426 
 2426 

302842509957
10
333333
genus


1945512 
1945512 
1945512 
1945512 
1945512 
1945512 
 1945512 
782393037
111111
species


155077 
155077 
155077 
155077 
155077 
155077 
 155077 
1024272413
111111
species

13181514457
 1987723 

1987723 
1987723 
1987723 
1987723 
1987723 
1987723 
111111
species

222222
family
1706372
151417203927

151417203927
1217416
222222
genus


930805 
930805 
930805 
930805 
930805 
930805 
 930805 
48363119
species
111111

111111
species
 930806 

930806 
930806 
930806 
930806 
930806 
930806 
116141488

family
444443
1706373
874157659772

874157659772
48073
genus
444443

species
111111
261319223228

260552 
260552 
260552 
260552 
260552 
260552 
 260552 

 252514 

252514 
252514 
252514 
252514 
252514 
252514 
3111105715
111111
species

species
11111
545108
 359370 

359370 
359370 
359370 
359370 
359370 

species
111111
 1769779 

1769779 
1769779 
1769779 
1769779 
1769779 
1769779 
251323285029

223322
family
19732201920
1706375

1434050
12161233
11111
genus

 1470434 

1470434 

1470434 
1470434 
1470434 
1470434 
12161233
11111
species

111
genus
4146
1084558

 716816 


716816 
716816 
716816 
4146
species
111

genus
111111
630749
73221617

111111
species

1620392 
1620392 
1620392 
1620392 
1620392 
1620392 
 1620392 
73221617

135624
213411851333192828443208
222222222222
order

213411851333192828443208
84642
family
222222222222

133254287
43947
111111
genus

111111
species

43948 
43948 
43948 
43948 
43948 
43948 
 43948 
133254287

genus
222222
101317222428
225143

5575159
 1903694 

1903694 
1903694 
1903694 
1903694 
1903694 
1903694 
111111
species

581017919

1416627 
1416627 
1416627 
1416627 
1416627 
1416627 
 1416627 
111111
species

129577
371115474623
genus
111111

371115474623

511062 
511062 
511062 
511062 
511062 
511062 
 511062 
species
111111

202811271245181126933104
642
genus
171717171717

85248451582312191416

644 
644 
644 
644 
644 
644 
 644 
111111
species

111111
species
253722186551

558964 
558964 
558964 
558964 
558964 
558964 
 558964 


651 
651 
651 
651 
651 
651 
 651 
31826313633
species
111111

441715205429

2033033 
2033033 
2033033 
2033033 
2033033 
2033033 
 2033033 
species
111111

 648 

648 
648 
648 
648 
648 
648 
88476584143170
species
111111

species
111111
501910204724
 1758179 

1758179 
1758179 
1758179 
1758179 
1758179 
1758179 

272026226340

948519 
948519 
948519 
948519 
948519 
948519 
 948519 
species
111111

111111
species

1636607 
1636607 
1636607 
1636607 
1636607 
1636607 
 1636607 
302029194413


1920107 
1920107 
1920107 
1920107 
1920107 
1920107 
 1920107 
412028282854
species
111111

species
111111

2033032 
2033032 
2033032 
2033032 
2033032 
2033032 
 2033032 
1621129784163174

111111
species

652 
652 
652 
652 
652 
652 
 652 
20127336239

160119138185215320
 654 

654 
654 
654 
654 
654 
654 
111111
species

species
111111
511226514849

1636608 
1636608 
1636608 
1636608 
1636608 
1636608 
 1636608 

57716354963

1636609 
1636609 
1636609 
1636609 
1636609 
1636609 
 1636609 
species
111111

111111
species

196024 
196024 
196024 
196024 
196024 
196024 
 196024 
431517285839

 1636606 

1636606 
1636606 
1636606 
1636606 
1636606 
1636606 
503422424581
111111
species

111111
species
297144186288354509

645 
645 
645 
645 
645 
645 
 645 

genus
111111
347533
463131445346

463131445346

347534 
347534 
347534 
347534 
347534 
347534 
 347534 
species
111111

1111
genus
655184
1722

species
11
22


1427364 

1427364 
 1427364 

11
species
 1705394 

1705394 

1705394 
17

111111
order
1463693
1775403

1463693
568386
family
111111

genus
111111
469322
1463693

111111
species
1463693
 465721 

465721 
465721 
465721 
465721 
465721 
465721 

151316141312
order
227209319386170110
72273

family
657655
34064
769899461915

1869285
21
genus
11

21
 594679 


594679 
594679 
11
species

262
769897451915
656555
genus

111111
species
 657445 

657445 
657445 
657445 
657445 
657445 
657445 
1128341812

 1542390 

1542390 
1542390 
1542390 

1542390 
1542390 
327611
species
11111

22

549298 

549298 
 549298 
species
11

11
species
181
 622488 

622488 
622488 

species
111111
 28110 

28110 
28110 
28110 
28110 
28110 
28110 
63121244

1
species
2
 573570 


573570 

5221


954 
954 
954 
954 
 954 
1111
species

36393811117

263 
263 
263 
263 
263 
263 
 263 
111111
species

777766
family
135616
12810320933914694

933
111254
111121
genus

species
111
 147268 

147268 

147268 

147268 
113

1224


92245 

92245 
92245 
92245 
 92245 
1111
species

212222
genus
243277820
40222

species
11111
 754477 

754477 

754477 
754477 
754477 
754477 
1611112

 754476 

754476 
754476 
754476 
754476 
754476 
754476 
83166718
species
111111

12311
28884
genus
11111

12311


39765 
39765 
39765 
39765 
39765 
 39765 
11111
species

1237
868617332213268
111111
genus

species
111111
 1238 

1238 
1238 
1238 
1238 
1238 
1238 
868617332213268

33221
genus
34067
1712651


1329899 
1329899 
1329899 
1329899 
 1329899 
11034
species
1111

111
species
711

728003 
728003 

728003 
 728003 

1111
species
9131

385025 
385025 
385025 


385025 
 385025 

family
212121
135617
23811151

1111
genus
18431
40751

1111
species
18431

40754 

40754 

40754 
40754 
 40754 

11111
genus
1021
58712


288004 
288004 
288004 
288004 
288004 
 288004 
58712
11111
species

order
252427252626
135613
639415316379748708

345341
1738654
family
111111

345341
1738655
genus
111111

345341
 1548547 

1548547 
1548547 
1548547 
1548547 
1548547 
1548547 
111111
species

family
8710899
1046
250175114132285350

13724
2655102745
genus
111111

species
111111
2655102745

73141 
73141 
73141 
73141 
73141 
73141 
 73141 

1111
genus
1980513
1131

1111
species
1131
 1630141 

1630141 

1630141 
1630141 

1630141 

111111
genus
5110918397962
85076

 37487 

37487 
37487 
37487 
37487 
37487 
37487 
5110918397962
species
111111

85072
491882062104
111111
genus

491882062104
 1049 

1049 
1049 
1049 
1049 
1049 
1049 
111111
species

43513151936
156885
genus
111111

111111
species

80679 
80679 
80679 
80679 
80679 
80679 
 80679 
43513151936

111111
genus
251010173333
53392

251010173333

1166950 
1166950 
1166950 
1166950 
1166950 
1166950 
 1166950 
111111
species


1978339 
1978339 
1978339 
1978339 
1978339 
1978339 
 1978339 
512523275766
species
111111

genus
113132
4336183
1227

1111
species
4162
 133539 


133539 
133539 
133539 
133539 

species
11111

473531 
473531 
473531 

473531 
473531 
 473531 
431511

species
11
171
 1229 


1229 

1229 

449719
511121565
111111
family

437504
511121565
genus
111111

511121565

437505 
437505 
437505 
437505 
437505 
437505 
 437505 
111111
species

family
111111
1676141
673114204827

genus
111111
673114204827
1676142

species
111111
673114204827

1579979 
1579979 
1579979 
1579979 
1579979 
1579979 
 1579979 

255526
33271727579
family
222222

109262
33271727579
222222
genus

species
111111
1819815195
 1860122 

1860122 
1860122 
1860122 
1860122 
1860122 
1860122 

species
111111
158912384
 927 

927 
927 
927 
927 
927 
927 

121212121212
family
72276
281167154182348316

85108
171313102318
genus
222222

111111
species
614234
 1052 

1052 
1052 
1052 
1052 
1052 
1052 

111111
species
 1053 

1053 
1053 
1053 
1053 
1053 
1053 
1112982014

26158262421
133193
111111
genus

species
111111
26158262421

351052 
351052 
351052 
351052 
351052 
351052 
 351052 

106633
1611177798223195
555555
genus

 108010 

108010 
108010 
108010 
108010 
108010 
108010 
451010235159
111111
species

species
111111
 106634 

106634 
106634 
106634 
106634 
106634 
106634 
354018185838

species
111111
20229162029

186931 
186931 
186931 
186931 
186931 
186931 
 186931 

species
111111
382526173944

1033854 
1033854 
1033854 
1033854 
1033854 
1033854 
 1033854 

 396595 

396595 
396595 
396595 
396595 
396595 
396595 
232014245525
species
111111

241224112932
1335745
222222
genus


1335746 
1335746 
1335746 
1335746 
1335746 
1335746 
 1335746 
174732120
species
111111

78178812
 1335757 

1335757 
1335757 
1335757 
1335757 
1335757 
1335757 
species
111111

25121273640
1051
genus
111111

species
111111
25121273640
 1442136 

1442136 
1442136 
1442136 
1442136 
1442136 
1442136 

genus
111111
1765964
28911101310

111111
species
28911101310
 160660 

160660 
160660 
160660 
160660 
160660 
160660 

order
7710674
118969
136127194798663

111111
family
315258267
118968

315258267
776
genus
111111

315258267

777 
777 
777 
777 
777 
777 
 777 
111111
species

669563
family
444
105122169716056

genus
558453
445
94120166685956

111
 452 

452 

452 
452 
111
species

4
 45067 


45067 
1
species


1867846 
 1867846 
1
1
species

22313

29423 
29423 
29423 
29423 
29423 
 29423 
11111
species

312
 96230 

96230 
96230 
96230 
species
111

59231
 450 

450 
450 
450 
450 
450 
species
11111

 446 

446 
446 
446 
446 
446 
446 
83107152635354
111111
species

11
species
11
 449 


449 

449 

1
species
1


66969 
 66969 

111


2005262 


2005262 
2005262 
 2005262 
111
species

genus
11111
465
112331

11111
species
112331
 451 

451 
451 
451 
451 
451 

212024222323
order
6124011109626307533
135625

family
212024222323
712
6124011109626307533

110772009169114
745
genus
111122

110772009168113
 747 

747 
747 
747 
747 
747 
747 
species
111111

11
 754 


754 
754 
11
species

535555
genus
7315101503846
713

111111
species
 67854 

67854 
67854 
67854 
67854 
67854 
67854 
116212


715 
715 
715 
715 
715 
715 
 715 
451265142917
111111
species

species
111111
2221819317

716 
716 
716 
716 
716 
716 
 716 

species
11111
271012

189834 

189834 
189834 
189834 
189834 
 189834 

35548
 718 

718 

718 
718 
718 
718 
11111
species

476528
1462823814
111111
genus

 47735 

47735 
47735 
47735 
47735 
47735 
47735 
1462823814
111111
species

genus
567655
2111444882868154
724

1111
species
1371
 712310 

712310 
712310 
712310 

712310 

111111
species

727 
727 
727 
727 
727 
727 
 727 
14584238944928

 730 

730 
730 
730 
730 
730 
730 
51394726147
111111
species


249188 

249188 
249188 

249188 
 249188 
2412
1111
species

 197575 


197575 
197575 
197575 
197575 
197575 
3175156137
species
11111

111
species
 726 


726 
726 
726 
1115

111111
species
 729 

729 
729 
729 
729 
729 
729 
121464410

genus
1111
155493
1442

1111
species

750 

750 

750 
750 
 750 
1442

111111
genus
214906
1514391682

1514391682
 731 

731 
731 
731 
731 
731 
731 
species
111111

2094023
10203934186
111111
genus

111111
species
10203934186
 738 

738 
738 
738 
738 
738 
738 

13213
697331
11111
genus

 157673 


157673 
157673 
157673 
157673 
157673 
13213
11111
species

75984
83821367440111
333333
genus

111111
species
 85404 

85404 
85404 
85404 
85404 
85404 
85404 
1516233511

5146101633472
 75985 

75985 
75985 
75985 
75985 
75985 
75985 
111111
species

111111
species
1720128128
 1432056 

1432056 
1432056 
1432056 
1432056 
1432056 
1432056 

416916
9542715040181
genus
333333

221413

739 
739 
739 
739 
739 
739 
 739 
111111
species

111111
species
 732 

732 
732 
732 
732 
732 
732 
3326417821


714 
714 
714 
714 
714 
714 
 714 
6014293931157
111111
species

1541743
2
genus
1

species
1
2
 386487 


386487 

129127132132128119
order
1030368118066083266015634891017446441713
72274

909090909090
family
135621
1029651117996171058715229431012252441029

878787878787
genus
1028032117929070971315211941010145440055
286

 157783 

157783 
157783 
157783 
157783 
157783 
157783 
9043413098041194368
111111
species


1259844 
1259844 
1259844 
1259844 
1259844 
1259844 
 1259844 
92241746611962645336
111111
species

 1207075 

1207075 
1207075 
1207075 
1207075 
1207075 
1207075 
296719283202546327421712
111111
species

488758398442332858896921393603146768
136843
111111111111
species group


200450 
200450 
200450 
200450 
200450 
200450 
 200450 
2553267155121089644648664
111111
species

111111
species
323023742880709330452083

75612 
75612 
75612 
75612 
75612 
75612 
 75612 

species
111111
 47878 

47878 
47878 
47878 
47878 
47878 
47878 
608957421385556200103851559

species
111111
137788792223501653748

47879 
47879 
47879 
47879 
47879 
47879 
 47879 

111111
species
28681085336951547814984579

47883 
47883 
47883 
47883 
47883 
47883 
 47883 

 46679 

46679 
46679 
46679 
46679 
46679 
46679 
13421087139552672296593
111111
species

 200451 

200451 
200451 
200451 
200451 
200451 
200451 
209918723822137645634599
111111
species

111111
species
106376900923624587141845892
 380021 

380021 
380021 
380021 
380021 
380021 
380021 

111111
species

294 
294 
294 
294 
294 
294 
 294 
453942358059283249633516323579132631

111111
species
227357444109144808900720
 76758 

76758 
76758 
76758 
76758 
76758 
76758 

species
111111

76761 
76761 
76761 
76761 
76761 
76761 
 76761 
234822534183152224295700

111111
species

1931241 
1931241 
1931241 
1931241 
1931241 
1931241 
 1931241 
10139468313461

species
111111
465199252618613153
 1856685 

1856685 
1856685 
1856685 
1856685 
1856685 
1856685 

235926273884309404657633
 219572 

219572 
219572 
219572 
219572 
219572 
219572 
species
111111

686308347893975261

2049589 
2049589 
2049589 
2049589 
2049589 
2049589 
 2049589 
111111
species


1294143 
1294143 
1294143 
1294143 
1294143 
1294143 
 1294143 
427248300549953224
species
111111

species
111111
381924054234162225859731

1415630 
1415630 
1415630 
1415630 
1415630 
1415630 
 1415630 

species
111111
2875150240817192471452

216142 
216142 
216142 
216142 
216142 
216142 
 216142 

111111
species
287124121309479138
 2213057 

2213057 
2213057 
2213057 
2213057 
2213057 
2213057 

species
111111
7033753207101355193
 2083051 

2083051 
2083051 
2083051 
2083051 
2083051 
2083051 

12697828425397343806217740149332
 198620 

198620 
198620 
198620 
198620 
198620 
198620 
species
111111

species
111111

157782 
157782 
157782 
157782 
157782 
157782 
 157782 
139761353914322427512

 1898684 

1898684 
1898684 
1898684 
1898684 
1898684 
1898684 
415225249486825233
species
111111

species
111111
 101564 

101564 
101564 
101564 
101564 
101564 
101564 
454194202500685172

species
111111

1827300 
1827300 
1827300 
1827300 
1827300 
1827300 
 1827300 
4924723932531463342479536

species
111111
6666255023760186684861681

2219057 
2219057 
2219057 
2219057 
2219057 
2219057 
 2219057 

species
111111
8974523498372195370
 1028989 

1028989 
1028989 
1028989 
1028989 
1028989 
1028989 

111111
species

104087 
104087 
104087 
104087 
104087 
104087 
 104087 
8975657885111985687425603

species
111111

1853130 
1853130 
1853130 
1853130 
1853130 
1853130 
 1853130 
299517705514693729361600

111111
species

1583341 
1583341 
1583341 
1583341 
1583341 
1583341 
 1583341 
42983412111320555409756

111111
species
 69328 

69328 
69328 
69328 
69328 
69328 
69328 
6833603127931388207


65741 
65741 
65741 
65741 
65741 
65741 
 65741 
534246274597970311
species
111111

species
111111
6704337521336924304

1788301 
1788301 
1788301 
1788301 
1788301 
1788301 
 1788301 

species group
666666
136845
5942728156276797194316086621537

111111
species
3735172116415895104151414
 76759 

76759 
76759 
76759 
76759 
76759 
76759 

species
111111
347416771536417458731312

78327 
78327 
78327 
78327 
78327 
78327 
 78327 


303 
303 
303 
303 
303 
303 
 303 
4980323732234285919413960417847
species
111111

8293153849772169277

70775 
70775 
70775 
70775 
70775 
70775 
 70775 
111111
species

124658554813652224526
 47880 

47880 
47880 
47880 
47880 
47880 
47880 
111111
species

340126142338581161
 47885 

47885 
47885 
47885 
47885 
47885 
47885 
species
111111

170798321822872
 1981174 

1981174 
1981174 
1981174 
1981174 
1981174 
1981174 
111111
species


1283291 
1283291 
1283291 
1283291 
1283291 
1283291 
 1283291 
348528852829738942162365
111111
species

111111
species

2018067 
2018067 
2018067 
2018067 
2018067 
2018067 
 2018067 
5780776639341573121487654

241123194325150784418792
 2083055 

2083055 
2083055 
2083055 
2083055 
2083055 
2083055 
species
111111

2266215212583147133725890

2067572 
2067572 
2067572 
2067572 
2067572 
2067572 
 2067572 
111111
species

136842
264241771023277580383371616643
333333
species group

111111
species
1564968169734973046700
 296 

296 
296 
296 
296 
296 
296 

111111
species

587753 
587753 
587753 
587753 
587753 
587753 
 587753 
242461634320944533302944615669

61439963612111224274
 47884 

47884 
47884 
47884 
47884 
47884 
47884 
111111
species

 1930532 

1930532 
1930532 
1930532 
1930532 
1930532 
1930532 
410180197558707175
111111
species

6553503187852168197

2083052 
2083052 
2083052 
2083052 
2083052 
2083052 
 2083052 
111111
species

 515393 

515393 
515393 
515393 
515393 
515393 
515393 
230719405098150044267799
111111
species

111111
species
66726624110992066249

1338689 
1338689 
1338689 
1338689 
1338689 
1338689 
 1338689 

136846
604226862656673785043110
species group
222222

570725442527644180632911
578833
species subgroup
111111

570725442527644180632911
 316 

316 
316 
316 
316 
316 
316 
111111
species

species
111111
335142129296441199

74829 
74829 
74829 
74829 
74829 
74829 
 74829 

 237609 

237609 
237609 
237609 
237609 
237609 
237609 
116057253613441613452
111111
species

111111
species
1430954112830852283745
 1500686 

1500686 
1500686 
1500686 
1500686 
1500686 
1500686 

116953447211371890387

1649877 
1649877 
1649877 
1649877 
1649877 
1649877 
 1649877 
species
111111

136849
120170586970121270582119865920982
888888
species group

species subgroup
222222
370230601530308067201284
251698

species
111111

47877 
47877 
47877 
47877 
47877 
47877 
 47877 
1743140772015323180627

111111
species

29438 
29438 
29438 
29438 
29438 
29438 
 29438 
1959165381015483540657

9637223697932913299
 46257 

46257 
46257 
46257 
46257 
46257 
46257 
species
111111

111111
species subgroup
251695
109333578798116239469257740416871

species
111111

317 
317 
317 
317 
317 
317 
 317 
109333578798116239469257740416871

111111
species
130682252512912653605
 33069 

33069 
33069 
33069 
33069 
33069 
33069 

 251701 

251701 
251701 
251701 
251701 
251701 
251701 
311126861429354853701177
species
111111

111111
species
 1206777 

1206777 
1206777 
1206777 
1206777 
1206777 
1206777 
70840369914571183308

111111
species
 36746 

36746 
36746 
36746 
36746 
36746 
36746 
104747947911172416438

species
111111
6332873047492009272
 2069256 

2069256 
2069256 
2069256 
2069256 
2069256 
2069256 

246523235357223414703719

244566 
244566 
244566 
244566 
244566 
244566 
 244566 
species
111111


1534110 
1534110 
1534110 
1534110 
1534110 
1534110 
 1534110 
607353073833712943694837
species
111111

19872400256891013815696
 2083054 

2083054 
2083054 
2083054 
2083054 
2083054 
2083054 
species
111111


2083053 
2083053 
2083053 
2083053 
2083053 
2083053 
 2083053 
7763363667681392217
species
111111


312306 
312306 
312306 
312306 
312306 
312306 
 312306 
94954245711511971341
species
111111

species group
777777
70168389464342410515110703238310
136841


53408 
53408 
53408 
53408 
53408 
53408 
 53408 
137460767416842207739
species
111111

111111
species
 53412 

53412 
53412 
53412 
53412 
53412 
53412 
6043393548621120372


287 
287 
287 
287 
287 
287 
 287 
638163589740225968839625234929
111111
species

1232139
133159964117262270507
222222
species subgroup

487239232682881140
 1149133 

1149133 
1149133 
1149133 
1149133 
1149133 
1149133 
species
111111

 330 

330 
330 
330 
330 
330 
330 
84436040910441389367
111111
species

111111
species

300 
300 
300 
300 
300 
300 
 300 
261712971299345744731535

species
111111
426207231539710228
 43263 

43263 
43263 
43263 
43263 
43263 
43263 

1472927111028092243714
 1500687 

1500687 
1500687 
1500687 
1500687 
1500687 
1500687 
111111
species

species
111111
106350042411111793377

1636610 
1636610 
1636610 
1636610 
1636610 
1636610 
 1636610 

111111
species
283322152529607629281852
 1659194 

1659194 
1659194 
1659194 
1659194 
1659194 
1659194 

183410891284356428941014

1611770 
1611770 
1611770 
1611770 
1611770 
1611770 
 1611770 
111111
species


930166 
930166 
930166 
930166 
930166 
930166 
 930166 
91195811697019758113815517
species
111111

111111
species

237610 
237610 
237610 
237610 
237610 
237610 
 237610 
378135146342494307

327425013006745530472186
 2201356 

2201356 
2201356 
2201356 
2201356 
2201356 
2201356 
111111
species

158665980116822054941
351
subfamily
222222

158665980116822054941
352
222222
genus

 353 

353 
353 
353 
353 
353 
353 
674297327808910402
species
111111

9123624748741144539
 354 

354 
354 
354 
354 
354 
354 
species
111111

111111
genus
331273675333
1849530

species
111111

1697053 
1697053 
1697053 
1697053 
1697053 
1697053 
 1697053 
331273675333

family
393742423829
717699122073405465194684
468

genus
455544
475
1682784872439092

111111
species
27291132682
 480 

480 
480 
480 
480 
480 
480 

11111
species
13111


476 
476 
476 
476 
476 
 476 


29433 
29433 
29433 
29433 
 29433 
23173
species
1111

1302412791927078
 34062 

34062 
34062 
34062 
34062 
34062 
34062 
111111
species

9475211111

386891 
386891 
386891 
386891 
386891 
386891 
 386891 
species
111111

genus
118111187
64152492993437
497

species
1111
222614

1720344 
1720344 
1720344 
1720344 
 1720344 

251125
 1699624 

1699624 
1699624 
1699624 
1699624 
1111
species

111111
species
42251461

571800 
571800 
571800 
571800 
571800 
571800 
 571800 

 349106 

349106 
349106 
349106 
349106 
349106 
349106 
101131325
111111
species

111
species

45610 

45610 
45610 
 45610 
936132


261164 
261164 
261164 
261164 
261164 
261164 
 261164 
161101576
111111
species

species
111111
411311101

2203895 
2203895 
2203895 
2203895 
2203895 
2203895 
 2203895 

species
11111
72113216

1028416 

1028416 
1028416 
1028416 
1028416 
 1028416 

42454357
 330922 

330922 
330922 
330922 
330922 
330922 
330922 
111111
species

111111
species
3119511
 1699622 

1699622 
1699622 
1699622 
1699622 
1699622 
1699622 

330141

334543 

334543 
334543 
334543 
 334543 
species
1111

242426262618
genus
469
485406121337400045070555

species
111111
 1636603 

1636603 
1636603 
1636603 
1636603 
1636603 
1636603 
7737414447017

11111
species
461195254119
 1608473 

1608473 
1608473 
1608473 
1608473 
1608473 

319693941094
 40216 

40216 
40216 
40216 
40216 
40216 
40216 
111111
species


40215 
40215 
40215 
40215 
40215 
40215 
 40215 
2623100753950663
111111
species

111111
species

1758189 
1758189 
1758189 
1758189 
1758189 
1758189 
 1758189 
1514265021202


1324350 
1324350 
1324350 
1324350 
1324350 
 1324350 
3163718449
11111
species


62977 
62977 
62977 
62977 
62977 
62977 
 62977 
11532246111
111111
species

111111
species

1871111 
1871111 
1871111 
1871111 
1871111 
1871111 
 1871111 
241742366302

1111
species
160516923


487316 
487316 
487316 
487316 
 487316 

1111582074690513
 108981 

108981 
108981 
108981 
108981 
108981 
108981 
species
111111

11111
species
2168034860

1407071 
1407071 
1407071 
1407071 
1407071 
 1407071 

111111
species
25495252322

106648 
106648 
106648 
106648 
106648 
106648 
 106648 

 756892 

756892 
756892 
756892 
756892 
756892 
756892 
53158926820211
species
111111

34127862936215342008486
909768
545554
species group

28825648851177931764420
 470 

470 
470 
470 
470 
470 
470 
111111
species


1785128 

1785128 
1785128 
1785128 
 1785128 
16141909
1111
species

species
111111
 48296 

48296 
48296 
48296 
48296 
48296 
48296 
18198573256415437

 471 

471 
471 
471 
471 
471 
471 
3133612573027
species
111111

species
111111
3121537730512

106654 
106654 
106654 
106654 
106654 
106654 
 106654 

131796424421742
 40214 

40214 
40214 
40214 
40214 
40214 
40214 
species
111111

species
111111

2079596 
2079596 
2079596 
2079596 
2079596 
2079596 
 2079596 
53100125510410


1646498 
1646498 
1646498 
1646498 
1646498 
 1646498 
962407458460
species
11111

species
111111

1808001 
1808001 
1808001 
1808001 
1808001 
1808001 
 1808001 
5414992992181

 1148157 

1148157 

1148157 
1148157 
1148157 
110532786
1111
species

6837121632184
 2004644 

2004644 
2004644 
2004644 
2004644 
2004644 
2004644 
species
111111

1621103462780457
 29430 

29430 
29430 
29430 
29430 
29430 
29430 
111111
species


1789224 
1789224 
1789224 
1789224 
 1789224 
32357710
species
1111

1273155
372023586720
111111
genus

372023586720

585455 
585455 
585455 
585455 
585455 
585455 
 585455 
species
111111

species
11111

113267 
113267 
113267 
113267 
113267 
 113267 
922724

111111
species
 1248727 

1248727 
1248727 
1248727 
1248727 
1248727 
1248727 
641313313313

776885817067
order
135622
8674991017105710261355

72275
361202359436566428
family
212025242222

6141021112
1751872
genus
111111

species
111111
6141021112

1526571 
1526571 
1526571 
1526571 
1526571 
1526571 
 1526571 

genus
12212
89404
31315330


983545 

983545 
983545 
983545 
983545 
 983545 
3413329
species
11111

111
species


300231 
300231 

300231 
 300231 
921

1621534
41111
genus
1111

1111
species
41111
 326544 


326544 
326544 
326544 
326544 

genus
999999
2742
256145129300437235

111111
species
152016223021

1749259 
1749259 
1749259 
1749259 
1749259 
1749259 
 1749259 


1420917 
1420917 
1420917 
1420917 
1420917 
1420917 
 1420917 
6225245710558
species
111111

species
111111
401412343719

1420916 
1420916 
1420916 
1420916 
1420916 
1420916 
 1420916 


490759 
490759 
490759 
490759 
490759 
490759 
 490759 
111554642
species
111111

111111
species
2587312832

1671721 
1671721 
1671721 
1671721 
1671721 
1671721 
 1671721 

111111
species
281212427122

1874317 
1874317 
1874317 
1874317 
1874317 
1874317 
 1874317 

 2743 

2743 
2743 
2743 
2743 
2743 
2743 
463627805245
species
111111

111111
species
1851017176

330734 
330734 
330734 
330734 
330734 
330734 
 330734 


1033846 
1033846 
1033846 
1033846 
1033846 
1033846 
 1033846 
111016133330
species
111111

15231033
261825
111111
genus

species
111111
15231033

680279 
680279 
680279 
680279 
680279 
680279 
 680279 

genus
111111
288793
23105612

 2183582 

2183582 
2183582 
2183582 
2183582 
2183582 
2183582 
23105612
species
111111

773717887105135
226
genus
779887

1511514
 2058133 

2058133 
2058133 
2058133 
2058133 
2058133 
2058133 
species
111111

111111
species
7742151635
 28108 

28108 
28108 
28108 
28108 
28108 
28108 

species
11111
 1917158 

1917158 
1917158 
1917158 

1917158 
1917158 
458315

111111
species

314275 
314275 
314275 
314275 
314275 
314275 
 314275 
431280466864

11111
species
12121


715451 
715451 
715451 
715451 
715451 
 715451 


287094 
287094 
287094 
287094 
 287094 
1126
species
1111

 589873 

589873 

589873 
589873 
589873 
589873 
546215
species
11111

species
111


1777491 
1777491 
1777491 
 1777491 
343

 233316 

233316 
233316 
233316 
233316 
233316 
233316 
210364101
111111
species

1172191
21126
1111
genus

 2172099 

2172099 
2172099 
2172099 
2172099 
21126
1111
species

746443
family
43193618146
267889

genus
645343
41193016146
28228

1531

1967665 

1967665 

1967665 
 1967665 
111
species

species
11111
125324

58049 
58049 
58049 
58049 
58049 
 58049 

species
1111
71132
 1816219 

1816219 
1816219 
1816219 

1816219 

 1816218 

1816218 


1816218 
1816218 
1816218 
1171
species
1111


2161872 
2161872 
2161872 


2161872 
 2161872 
31101
1111
species

 28229 

28229 
28229 
28229 
28229 

28229 
3121134
11111
species

1518149
262
genus
111

111
species
262

1763536 

1763536 
1763536 
 1763536 

family
171423211413
267888
141882392078471

53246
141882392078471
genus
171423211413

species
1111
 152297 

152297 

152297 
152297 
152297 
120133

111111
species

288 
288 
288 
288 
288 
288 
 288 
33166122

13


28107 
28107 
 28107 
species
11

11111
species
1710212
 1720343 


1720343 
1720343 
1720343 
1720343 
1720343 

 1348114 

1348114 

1348114 
1348114 
1348114 
1348114 
211561
11111
species

 43657 

43657 
43657 
43657 
43657 
43657 
43657 
812161353
species
111111

1111
species


283699 
283699 
283699 

283699 
 283699 
14315

111111
species
 247523 

247523 
247523 
247523 
247523 
247523 
247523 
419191

 1761891 

1761891 

1761891 
1761891 
1761891 
6431
1111
species

111
species
421
 43662 


43662 

43662 
43662 

122
 166935 


166935 
166935 
166935 
species
111

538264

1514074 
1514074 
1514074 
1514074 

1514074 
 1514074 
species
11111

111
species
 43658 

43658 

43658 
43658 
758

51021

43659 

43659 
43659 

43659 
 43659 
species
1111

11642
 234831 

234831 
234831 
234831 
234831 
234831 
11111
species

332044361846
 298657 

298657 
298657 
298657 
298657 
298657 
298657 
111111
species

species
111111
1210511

314281 
314281 
314281 
314281 
314281 
314281 
 314281 

species
111

28109 

28109 
28109 
 28109 
1685

species
11111
32093320

161398 
161398 
161398 
161398 
161398 
 161398 

11111
species
11132

267375 
267375 
267375 
267375 
267375 
 267375 

11111
species

228 
228 
228 
228 

228 
 228 
1952031


394751 
394751 
394751 
 394751 
1154
species
111

26523
 176102 

176102 

176102 
176102 

176102 
species
1111

267891
214228118
family
122222

214228118
58050
122222
genus


69539 
69539 
69539 
69539 
69539 
 69539 
211622
species
11111

111111
species
 80854 

80854 
80854 
80854 
80854 
80854 
80854 
21211296

1937221496
267894
222221
family

genus
222221
67572
1937221496

122991116

357794 
357794 
357794 
357794 
357794 
357794 
 357794 
111111
species

11111
species
781338

314282 
314282 
314282 
314282 
314282 
 314282 

242114283318252729
267890
242122232122
family

genus
242122232122
22
242114283318252729


70864 
70864 
70864 
70864 
70864 
70864 
 70864 
228721
111111
species

11731022

351745 
351745 
351745 
351745 
351745 
351745 
 351745 
111111
species

species
111111
5832114115104549

62322 
62322 
62322 
62322 
62322 
62322 
 62322 

species
111111
 225848 

225848 
225848 
225848 
225848 
225848 
225848 
263412125

45321

56812 

56812 
56812 
56812 
56812 
 56812 
species
11111

111111
species
9310722
 94122 

94122 
94122 
94122 
94122 
94122 
94122 

11111
species

60481 

60481 
60481 
60481 
60481 
 60481 
85625

111111
species
191020171547

24 
24 
24 
24 
24 
24 
 24 

 2018305 

2018305 
2018305 
2018305 
2018305 
2018305 
2018305 
11131041
111111
species

species
111111
17142091

192073 
192073 
192073 
192073 
192073 
192073 
 192073 

871772127

60478 
60478 
60478 
60478 
60478 
60478 
 60478 
111111
species

species
11111
431147

60961 
60961 

60961 
60961 
60961 
 60961 

1415191

2029986 
2029986 
2029986 
2029986 
2029986 
2029986 
 2029986 
species
111111

11111
species
 60480 

60480 
60480 
60480 
60480 
60480 
544516

5552339

260364 
260364 
260364 
260364 
260364 
260364 
 260364 
species
111111

111111
species
 43661 

43661 
43661 
43661 
43661 
43661 
43661 
4189418


359303 
359303 
359303 
359303 
359303 
359303 
 359303 
1941891713
111111
species


60217 
60217 
60217 
60217 
60217 
60217 
 60217 
1023645
species
111111

species
111
224

404011 
404011 
404011 
 404011 

13399926

271097 
271097 
271097 
271097 
271097 
271097 
 271097 
species
111111

11111
species
121553
 271098 

271098 
271098 
271098 
271098 

271098 

1111
species
 93973 

93973 
93973 

93973 

93973 
14491

11111
species
27764
 2059264 

2059264 

2059264 
2059264 
2059264 
2059264 

species
111111
2121051

70863 
70863 
70863 
70863 
70863 
70863 
 70863 

267893
231237144441
family
444443

231237144441
135575
444443
genus

species
11111
33622
 2100422 

2100422 
2100422 
2100422 
2100422 
2100422 

111111
species
541451815

1096243 
1096243 
1096243 
1096243 
1096243 
1096243 
 1096243 

species
111111
421021015
 2055892 

2055892 
2055892 
2055892 
2055892 
2055892 
2055892 

113751411
 135577 

135577 
135577 
135577 
135577 
135577 
135577 
111111
species

family
111111
267892
361319424666

44011
361319424666
111111
genus

361319424666

44012 
44012 
44012 
44012 
44012 
44012 
 44012 
species
111111

442621316376
1934945
111111
order

family
111111
442621316376
1934946

442621316376
1934947
111111
genus

species
111111

1810504 
1810504 
1810504 
1810504 
1810504 
1810504 
 1810504 
442621316376


83406 
83406 
83406 
83406 
83406 
83406 
 83406 
13651977
species
111111

215684769762164523225093545311214866
91347
order
204203206202201201

6403548048549592359
1903414
151416161514
family

222222
genus
198112232212199635
583

 584 

584 
584 
584 
584 
584 
584 
193111228203197632
species
111111

species
111111
 585 

585 
585 
585 
585 
585 
585 
514923

344443
genus
58511609779187
586

72439372491

587 
587 
587 
587 
587 
587 
 587 
111111
species

152312
 126385 


126385 
126385 
126385 
126385 
126385 
11111
species

111111
species
 588 

588 
588 
588 
588 
588 
588 
422597514884

 333962 

333962 
333962 
333962 
333962 
333962 
911974
11111
species

5721807092219
626
535555
genus

101512942
 40577 

40577 

40577 
40577 
40577 
40577 
species
11111

739156
 628 

628 
628 
628 
628 
628 
628 
111111
species

11111
species

351679 

351679 
351679 
351679 
351679 
 351679 
2781515

2412233557131

40576 
40576 
40576 
40576 
40576 
40576 
 40576 
111111
species

1462614625

351671 
351671 
351671 
351671 
351671 
351671 
 351671 
species
111111

genus
1111
637
5411


634113 
634113 
634113 
634113 
 634113 
5411
1111
species

111111
genus
2741552744225031180
581

 582 

582 
582 
582 
582 
582 
582 
2741552744225031180
111111
species

4811575286138
29487
333333
genus

739163436
 230089 

230089 
230089 
230089 
230089 
230089 
230089 
111111
species

111111
species
15523101822

291112 
291112 
291112 
291112 
291112 
291112 
 291112 

26325263480

2218628 
2218628 
2218628 
2218628 
2218628 
2218628 
 2218628 
111111
species

10676206236186567
447792
genus
222222

 1756993 

1756993 
1756993 
1756993 
1756993 
1756993 
1756993 
5934100137103259
species
111111

species
111111

1972431 
1972431 
1972431 
1972431 
1972431 
1972431 
 1972431 
47421069983308

84991098312315247311835538343
1903411
333333333333
family

629
145384522423069392010008
131313131313
genus

111111
species
17851602050

29483 
29483 
29483 
29483 
29483 
29483 
 29483 

 419257 

419257 
419257 
419257 
419257 
419257 
419257 
919432612118
species
111111

111111
species
 263819 

263819 
263819 
263819 
263819 
263819 
263819 
34143838118131

species
111111
 29484 

29484 
29484 
29484 
29484 
29484 
29484 
531998134134625

2429363438

29485 
29485 
29485 
29485 
29485 
29485 
 29485 
111111
species

111111
species
34423458377513472232

630 
630 
630 
630 
630 
630 
 630 

species
111111

28152 
28152 
28152 
28152 
28152 
28152 
 28152 
107282440372

111111
species
 29486 

29486 
29486 
29486 
29486 
29486 
29486 
8771170230163627

10631382980

1839800 
1839800 
1839800 
1839800 
1839800 
1839800 
 1839800 
111111
species

8394311073159018615470
1649845
species group
333333


633 
633 
633 
633 
633 
633 
 633 
163872122694121210
species
111111

111111
species
657339849128414134210

632 
632 
632 
632 
632 
632 
 632 

111111
species

367190 
367190 
367190 
367190 
367190 
367190 
 367190 
19512373650

species
111111
48329811853365
 631 

631 
631 
631 
631 
631 
631 

222222
genus
24297923907682425814
34037

 741091 

741091 
741091 
741091 
741091 
741091 
741091 
14349912073888232450
111111
species

9948011833794193364
 34038 

34038 
34038 
34038 
34038 
34038 
34038 
species
111111

111111
genus
1565532
1282356471950238421

111111
species
1282356471950238421

1805933 
1805933 
1805933 
1805933 
1805933 
1805933 
 1805933 

161616161616
genus
664888997014119861369726998
613

111111
species
 104623 

104623 
104623 
104623 
104623 
104623 
104623 
2416399475160

 1758196 

1758196 
1758196 
1758196 
1758196 
1758196 
1758196 
78426768141173
111111
species

species
111111
 614 

614 
614 
614 
614 
614 
614 
177442269425477836

12887122199235597

61651 
61651 
61651 
61651 
61651 
61651 
 61651 
species
111111

species
111111
444990119111166

768493 
768493 
768493 
768493 
768493 
768493 
 768493 

111111
species
 47917 

47917 
47917 
47917 
47917 
47917 
47917 
155168238399530790


615 
615 
615 
615 
615 
615 
 615 
4617362645667870923316972
111111
species

81309989131183325

28151 
28151 
28151 
28151 
28151 
28151 
 28151 
species
111111

703083102154363

1759437 
1759437 
1759437 
1759437 
1759437 
1759437 
 1759437 
species
111111

 488142 

488142 
488142 
488142 
488142 
488142 
488142 
883366104110223
species
111111

9354102179216469
 2033438 

2033438 
2033438 
2033438 
2033438 
2033438 
2033438 
111111
species

 1327989 

1327989 
1327989 
1327989 
1327989 
1327989 
1327989 
794155101146452
species
111111

 61652 

61652 
61652 
61652 
61652 
61652 
61652 
178131213361449706
species
111111

111111
species
8152118155157400
 671990 

671990 
671990 
671990 
671990 
671990 
671990 

species
111111

82996 
82996 
82996 
82996 
82996 
82996 
 82996 
682978828159013814157

species
111111
7351698999209
 768490 

768490 
768490 
768490 
768490 
768490 
768490 

1745211
2825224475102
111111
genus

111111
species
 1639108 

1639108 
1639108 
1639108 
1639108 
1639108 
1639108 
2825224475102

102102102101100100
family
543
454554496214792371096130319186297

1215131
1682492
genus
111111

 1410383 

1410383 
1410383 
1410383 
1410383 
1410383 
1410383 
1215131
species
111111

111111
genus
568987
7531227952761357631164


138072 
138072 
138072 
138072 
138072 
138072 
 138072 
7531227952761357631164
species
111111

213


1199245 
1199245 
 1199245 
species
11

11111
species
 134287 

134287 
134287 
134287 
134287 

134287 
15511

genus
222222
160674
511411898105312062595

species
111111

54291 
54291 
54291 
54291 
54291 
54291 
 54291 
46635379993710982366

 575 

575 
575 
575 
575 
575 
575 
455899116108229
111111
species

15
1906659
genus
11

11
species
 1778263 


1778263 
1778263 
15

genus
171717171717
154115983572306641918514
544

species
111111
3836687789149

2013114 
2013114 
2013114 
2013114 
2013114 
2013114 
 2013114 

species group
888888
8809912135177525665186
1344959

species
111111

2077149 
2077149 
2077149 
2077149 
2077149 
2077149 
 2077149 
21847448128140

species
111111

133448 
133448 
133448 
133448 
133448 
133448 
 133448 
3523685484140

8356142136187651

57706 
57706 
57706 
57706 
57706 
57706 
 57706 
111111
species

29488590138229

2066049 
2066049 
2066049 
2066049 
2066049 
2066049 
 2066049 
111111
species

5787153104174366
 67827 

67827 
67827 
67827 
67827 
67827 
67827 
111111
species


2077147 
2077147 
2077147 
2077147 
2077147 
2077147 
 2077147 
3527575979183
species
111111

111111
species

2077148 
2077148 
2077148 
2077148 
2077148 
2077148 
 2077148 
233373649454

species
111111

546 
546 
546 
546 
546 
546 
 546 
5976331483122016823423

3928796484230
 1702170 

1702170 
1702170 
1702170 
1702170 
1702170 
1702170 
111111
species

 1703250 

1703250 
1703250 
1703250 
1703250 
1703250 
1703250 
2029616276101
species
111111

species
111111
202233574449623989

545 
545 
545 
545 
545 
545 
 545 

111111
species
3232836182247
 67824 

67824 
67824 
67824 
67824 
67824 
67824 

species
111111
153110287287321752

35703 
35703 
35703 
35703 
35703 
35703 
 35703 

111111
species
3535807775234
 2019568 

2019568 
2019568 
2019568 
2019568 
2019568 
2019568 

111111
species
 67825 

67825 
67825 
67825 
67825 
67825 
67825 
6963109121178302

 1920110 

1920110 
1920110 
1920110 
1920110 
1920110 
1920110 
7341969397324
111111
species

590
6373470512379115771454532074
222222
genus

11174130135156509

54736 
54736 
54736 
54736 
54736 
54736 
 54736 
species
111111

6262463112249114421438931565
 28901 

28901 
28901 
28901 
28901 
28901 
28901 
111111
species

153119116
409304
genus
111111

species
111111
153119116
 168169 

168169 
168169 
168169 
168169 
168169 
168169 

1335483
4222448393871
genus
111111


563 
563 
563 
563 
563 
563 
 563 
4222448393871
111111
species

species
111111
 2052938 

2052938 
2052938 
2052938 
2052938 
2052938 
2052938 
3724266754190

3081454333913661843

891974 
891974 
891974 
891974 
891974 
891974 
 891974 
111111
species

3547131122107323

693444 
693444 
693444 
693444 
693444 
693444 
 693444 
111111
species

genus
333333
22115532092465230511035
83654

9157289815111458348
 83655 

83655 
83655 
83655 
83655 
83655 
83655 
111111
species

species
111111
 1920116 

1920116 
1920116 
1920116 
1920116 
1920116 
1920116 
6951887114610844397

species
111111
61463323168749290
 1920114 

1920114 
1920114 
1920114 
1920114 
1920114 
1920114 

1111
genus
1906660
22201

22201


1778264 
1778264 
1778264 
1778264 
 1778264 
1111
species

111111
genus
158483
2562134655607781825

2562134655607781825

158822 
158822 
158822 
158822 
158822 
158822 
 158822 
species
111111

genus
454222
203804
173613232

111
species
311
 251535 


251535 

251535 
251535 


101534 
101534 
101534 
101534 
101534 
101534 
 101534 
252121
111111
species

101461
 251542 

251542 
251542 
251542 


251542 
species
1111

species
111
231
 1505597 

1505597 
1505597 
1505597 

3114
 203907 

203907 
203907 
203907 
species
111

50851013632183109031983725944
547
genus
242424242424


1914861 
1914861 
1914861 
1914861 
1914861 
1914861 
 1914861 
10579161226190397
species
111111

354276
450882462172395871830722884
141414141414
species group

111111
species

550 
550 
550 
550 
550 
550 
 550 
285459011597962591287414273


1915310 
1915310 
1915310 
1915310 
1915310 
1915310 
 1915310 
5082182114163227
111111
species

111111
species
 299767 

299767 
299767 
299767 
299767 
299767 
299767 
6461159135133247

111111
species

2077137 
2077137 
2077137 
2077137 
2077137 
2077137 
 2077137 
3860148111121205

436726883184247
 1812935 

1812935 
1812935 
1812935 
1812935 
1812935 
1812935 
111111
species

 1812934 

1812934 
1812934 
1812934 
1812934 
1812934 
1812934 
1562185513495101214
111111
species


2027919 
2027919 
2027919 
2027919 
2027919 
2027919 
 2027919 
113166329213354438
species
111111

2723789505538061281
 61645 

61645 
61645 
61645 
61645 
61645 
61645 
species
111111

111111
species
 158836 

158836 
158836 
158836 
158836 
158836 
158836 
6408862225115422493432


1296536 
1296536 
1296536 
1296536 
1296536 
1296536 
 1296536 
6899238128238323
species
111111

species
111111
52110179133228243

69218 
69218 
69218 
69218 
69218 
69218 
 69218 

5068207151148226

2077136 
2077136 
2077136 
2077136 
2077136 
2077136 
 2077136 
species
111111

379715995169277

1870930 
1870930 
1870930 
1870930 
1870930 
1870930 
 1870930 
111111
species

7153149109130251
 208224 

208224 
208224 
208224 
208224 
208224 
208224 
species
111111

species
111111
4679158107137180

1868135 
1868135 
1868135 
1868135 
1868135 
1868135 
 1868135 

4830137127114350
 1692238 

1692238 
1692238 
1692238 
1692238 
1692238 
1692238 
species
111111

111111
species
50107182155175285

881260 
881260 
881260 
881260 
881260 
881260 
 881260 

24661136079127
 2051905 

2051905 
2051905 
2051905 
2051905 
2051905 
2051905 
species
111111

111111
species
6012429080136240370

399742 
399742 
399742 
399742 
399742 
399742 
 399742 

species
111111

1827481 
1827481 
1827481 
1827481 
1827481 
1827481 
 1827481 
476415890129140

111111
species
8954137165134543

1166130 
1166130 
1166130 
1166130 
1166130 
1166130 
 1166130 

111111
species
 1560339 

1560339 
1560339 
1560339 
1560339 
1560339 
1560339 
6382146111166347

species
111111
 1977566 

1977566 
1977566 
1977566 
1977566 
1977566 
1977566 
4587188139166321

genus
444444
1330545
3616956495948029471909

species
111111
 2153385 

2153385 
2153385 
2153385 
2153385 
2153385 
2153385 
64220657171196578

111111
species
 69220 

69220 
69220 
69220 
69220 
69220 
69220 
7261148103134372

155651848179372417642
 61646 

61646 
61646 
61646 
61646 
61646 
61646 
species
111111

 1907578 

1907578 
1907578 
1907578 
1907578 
1907578 
1907578 
70157610156200317
species
111111

species
111
 1835721 


1835721 
1835721 
1835721 
111

111111
genus
2172100
23117231497

species
111111

2172103 
2172103 
2172103 
2172103 
2172103 
2172103 
 2172103 
23117231497

genus
444444
466365832113510163336
1330547

 1158459 

1158459 
1158459 
1158459 
1158459 
1158459 
1158459 
13397246333302875
species
111111

1591483374273661100
 283686 

283686 
283686 
283686 
283686 
283686 
283686 
111111
species

111111
species

497725 
497725 
497725 
497725 
497725 
497725 
 497725 
8658110175159421

8862139200189940
 208223 

208223 
208223 
208223 
208223 
208223 
208223 
111111
species

570
13409925921534233874190159032
genus
999999

species
111111
 1905288 

1905288 
1905288 
1905288 
1905288 
1905288 
1905288 
68549111698339

111111
species

548 
548 
548 
548 
548 
548 
 548 
7605541444129616563526

3429757559265
 2026240 

2026240 
2026240 
2026240 
2026240 
2026240 
2026240 
111111
species

species
111111
10032682915297172222317043686

573 
573 
573 
573 
573 
573 
 573 

111111
species

1934254 
1934254 
1934254 
1934254 
1934254 
1934254 
 1934254 
66319591124329

111111
species

571 
571 
571 
571 
571 
571 
 571 
104876920701871111764867

111111
species
 244366 

244366 
244366 
244366 
244366 
244366 
244366 
4283137938608391801

species
111111
 1463165 

1463165 
1463165 
1463165 
1463165 
1463165 
1463165 
4303045347639671908

species
111111

1134687 
1134687 
1134687 
1134687 
1134687 
1134687 
 1134687 
5433761135109338122311

929812
7031101140184323
genus
111111

111111
species

929813 
929813 
929813 
929813 
929813 
929813 
 929813 
7031101140184323

620
115653843
genus
321232

11
species
31
 1813821 

1813821 


1813821 

species
11111
 623 

623 
623 

623 
623 
623 
23351


622 
622 
622 
622 
622 
622 
 622 
62623242
species
111111

561
7830617016096145051804538302
genus
444444

species
111111
 208962 

208962 
208962 
208962 
208962 
208962 
208962 
133108310299291650

111111
species

1499973 
1499973 
1499973 
1499973 
1499973 
1499973 
 1499973 
131038243350

species
111111

564 
564 
564 
564 
564 
564 
 564 
151716283373

111111
species

562 
562 
562 
562 
562 
562 
 562 
7669603515732141541768837529

3621
401618
genus
1111

3621

401619 
401619 
401619 


401619 
 401619 
species
1111

777777
genus
413496
8695631418167420074798

 413497 

413497 
413497 
413497 
413497 
413497 
413497 
8954154166160572
species
111111

36324658467810101838

28141 
28141 
28141 
28141 
28141 
28141 
 28141 
111111
species


535744 
535744 
535744 
535744 
535744 
535744 
 535744 
6746102155154446
111111
species


1163710 
1163710 
1163710 
1163710 
1163710 
1163710 
 1163710 
57369112590328
species
111111

6348120135173413
 413502 

413502 
413502 
413502 
413502 
413502 
413502 
111111
species

874591124134272

413501 
413501 
413501 
413501 
413501 
413501 
 413501 
111111
species

111111
species
 413503 

413503 
413503 
413503 
413503 
413503 
413503 
14388276291286929

species
111111
7034101150166263
 1920109 

1920109 
1920109 
1920109 
1920109 
1920109 
1920109 


2066051 
2066051 
2066051 
2066051 
2066051 
2066051 
 2066051 
4038969696181
species
111111

1
genus
1
1906661

species
1
1

1070130 
 1070130 

genus
11111
1048757
69415


1048758 
1048758 
1048758 
1048758 

1048758 
 1048758 
69415
11111
species

111
genus
121
1906657

111
species
121
 1778262 


1778262 
1778262 
1778262 

1330546
2862624686207391393
genus
222222

species
111111
 61647 

61647 
61647 
61647 
61647 
61647 
61647 
162133215317417785

 1334193 

1334193 
1334193 
1334193 
1334193 
1334193 
1334193 
124129253303322608
111111
species

species
111111
3636105125132224

1920128 
1920128 
1920128 
1920128 
1920128 
1920128 
 1920128 

1903410
15829281998259330746757
family
151516151515

6004471072119112742998
122277
555555
genus


2042057 
2042057 
2042057 
2042057 
2042057 
2042057 
 2042057 
6081104138169305
species
111111

111111
species
 554 

554 
554 
554 
554 
554 
554 
2311634655515261177

111111
species

55208 
55208 
55208 
55208 
55208 
55208 
 55208 
289286450196

 1905730 

1905730 
1905730 
1905730 
1905730 
1905730 
1905730 
10671196177220701
species
111111

 29471 

29471 
29471 
29471 
29471 
29471 
29471 
175123279261309619
species
111111

334333
genus
84565
18267124122166284

4820215350121

1239307 
1239307 
1239307 
1239307 
1239307 
1239307 
 1239307 
111111
species

 1929246 


1929246 
3
species
1

1113567264795

63612 
63612 
63612 
63612 
63612 
63612 
 63612 
species
111111

111111
species

1486991 
1486991 
1486991 
1486991 
1486991 
1486991 
 1486991 
231233436968

genus
111111
71655
3012394865132

species
111111
 1109412 

1109412 
1109412 
1109412 
1109412 
1109412 
1109412 
3012394865132

666666
genus
770402763123215693343
204037

species
111111
532449118100222

2037915 
2037915 
2037915 
2037915 
2037915 
2037915 
 2037915 

822671100109234
 1778540 

1778540 
1778540 
1778540 
1778540 
1778540 
1778540 
111111
species

species
111111
13065135234295696
 204042 

204042 
204042 
204042 
204042 
204042 
204042 

111111
species

1089444 
1089444 
1089444 
1089444 
1089444 
1089444 
 1089444 
3421823285226351390

111111
species
9877120169243568

204038 
204038 
204038 
204038 
204038 
204038 
 204038 

 204039 

204039 
204039 
204039 
204039 
204039 
204039 
65286089187233
species
111111

4116455246121
702
genus
111111

species
111111

703 
703 
703 
703 
703 
703 
 703 
4116455246121

family
232323212223
1587361932352250221760200334978232
1903409

67231
51228
11111
genus

11111
species

51229 
51229 
51229 

51229 
51229 
 51229 
67231

590015811898974871207559948
551
777667
genus

 215689 

215689 
215689 
215689 
215689 
215689 
215689 
14356133151274430
species
111111

3661243892808391106

79967 
79967 
79967 
79967 
79967 
79967 
 79967 
species
111111


1922217 
1922217 
1922217 


1922217 
 1922217 
61341
species
1111

111111
species

182337 
182337 
182337 
182337 
182337 
182337 
 182337 
903784172025391177232968

111111
species
 338565 

338565 
338565 
338565 
338565 
338565 
338565 
358160180149324880


1619313 
1619313 
1619313 
1619313 
1619313 
1619313 
 1619313 
42637259059626763000
species
111111

4751724914069191563

552 
552 
552 
552 
552 
552 
 552 
species
111111

11136128182247622
82986
genus
222222

111111
species
3813376568241

82987 
82987 
82987 
82987 
82987 
82987 
 82987 

species
111111
732391117179381
 53336 

53336 
53336 
53336 
53336 
53336 
53336 

genus
121212121212
53335
152337167253280721401979292967625

164481022002621314
 1891675 

1891675 
1891675 
1891675 
1891675 
1891675 
1891675 
111111
species

species
111111
173611372623451039

1484157 
1484157 
1484157 
1484157 
1484157 
1484157 
 1484157 

1654067
843828658159989800130766521298
111111
species group

 549 

549 
549 
549 
549 
549 
549 
843828658159989800130766521298
species
111111

13244135224367971
 665913 

665913 
665913 
665913 
665913 
665913 
665913 
species
111111

2401576314076001735
 1076550 

1076550 
1076550 
1076550 
1076550 
1076550 
1076550 
species
111111

 553 

553 
553 
553 
553 
553 
553 
391761916235268344523560
111111
species

 665914 

665914 
665914 
665914 
665914 
665914 
665914 
179641542673271015
species
111111

species
111111

592316 
592316 
592316 
592316 
592316 
592316 
 592316 
3471224074833871942


66269 
66269 
66269 
66269 
66269 
66269 
 66269 
246431663894601951
species
111111

152913228

1235990 
1235990 
1235990 
1235990 
1235990 
1235990 
 1235990 
111111
species


470934 
470934 
470934 
470934 
470934 
470934 
 470934 
6217967861319210810841974409684
species
111111

 1484158 

1484158 
1484158 
1484158 
1484158 
1484158 
1484158 
363942494083573108
species
111111

111111
genus
382974324723736
32199

species
111111
382974324723736
 9 

9 
9 
9 
9 
9 
9 

331344366147
1903416
222222
family

14527162930
82984
genus
111111

species
111111
14527162930
 82985 

82985 
82985 
82985 
82985 
82985 
82985 

19817203217
82980
111111
genus


158841 
158841 
158841 
158841 
158841 
158841 
 158841 
19817203217
species
111111

111111111111
family
592321867115111972143
1903412

568
8583242228235479
333333
genus


1848580 
1848580 
1848580 
1848580 
1848580 
1848580 
 1848580 
2024603863160
111111
species

111111
species
71149531668
 546367 

546367 
546367 
546367 
546367 
546367 
546367 

5848133137156251
 569 

569 
569 
569 
569 
569 
569 
111111
species

82982
304366881149
genus
111111

 82983 

82983 
82983 
82983 
82983 
82983 
82983 
304366881149
111111
species

777777
genus
635
4772345898558811515

111111
species
1282767139197303
 1263550 

1263550 
1263550 
1263550 
1263550 
1263550 
1263550 

181311533592

93378 
93378 
93378 
93378 
93378 
93378 
 93378 
111111
species

 67780 

67780 
67780 
67780 
67780 
67780 
67780 
1016378132199335
species
111111

111111
species
 1578828 

1578828 
1578828 
1578828 
1578828 
1578828 
1578828 
423123622866154

111111
species
 1650654 

1650654 
1650654 
1650654 
1650654 
1650654 
1650654 
2723367389127

species
111111
11653115171211350

636 
636 
636 
636 
636 
636 
 636 

species
111111
 1821960 

1821960 
1821960 
1821960 
1821960 
1821960 
1821960 
4524465984154

111111
order
742030
391721414138

391721414138
742031
111111
family

genus
111111
391721414138
180541

111111
species
391721414138
 2183911 

2183911 
2183911 
2183911 
2183911 
2183911 
2183911 

6415241
1524249
genus
111111

6415241

1249552 
1249552 
1249552 
1249552 
1249552 
1249552 
 1249552 
species
111111

28227726660222125150434239002255475
135614
414141414141
order

1775411
131382586241212901056
888888
family

333333
genus
510339390189612489
231454

14411811663161176

1379159 
1379159 
1379159 
1379159 
1379159 
1379159 
 1379159 
species
111111

species
111111
23413915765306200

445710 
445710 
445710 
445710 
445710 
445710 
 445710 

1328211761145113
 231455 

231455 
231455 
231455 
231455 
231455 
231455 
species
111111

111111
genus
1391159148177116
242605

111111
species
 242606 

242606 
242606 
242606 
242606 
242606 
242606 
1391159148177116

genus
111111
2233801
251939161618

111111
species
 2021234 

2021234 
2021234 
2021234 
2021234 
2021234 
2021234 
251939161618

genus
111111
6210370198052
70411

6210370198052
 81475 

81475 
81475 
81475 
81475 
81475 
81475 
111111
species

323413
2439712972243173
genus
111111

species
111111

323415 
323415 
323415 
323415 
323415 
323415 
 323415 
2439712972243173

33415214368162208
75309
111111
genus

species
111111
33415214368162208

666685 
666685 
666685 
666685 
666685 
666685 
 666685 

32033
28096426577722038950022237712254419
family
333333333333

genus
222222
867677603207614489
83614

111111
species
 2006110 

2006110 
2006110 
2006110 
2006110 
2006110 
2006110 
49540128886330264

 2172536 

2172536 
2172536 
2172536 
2172536 
2172536 
2172536 
372276315121284225
species
111111

68
408234113136101531342034
555555
genus

species
111111
539350392105408308

1605891 
1605891 
1605891 
1605891 
1605891 
1605891 
 1605891 

1062939836273815552
 84531 

84531 
84531 
84531 
84531 
84531 
84531 
species
111111

 69 

69 
69 
69 
69 
69 
69 
820429493140496327
111111
species

species
111111

262324 
262324 
262324 
262324 
262324 
262324 
 262324 
471434375205354274

111111
species
1190125910402921061573

435897 
435897 
435897 
435897 
435897 
435897 
 435897 

111111
genus
2370
228274314125304122

228274314125304122

2371 
2371 
2371 
2371 
2371 
2371 
 2371 
species
111111

genus
222222
29452013199756119921353
83618

species
111111

415229 
415229 
415229 
415229 
415229 
415229 
 415229 
695457432147403308

111111
species
22501556156541415891045

314722 
314722 
314722 
314722 
314722 
314722 
 314722 

genus
888888
40323
17434618249714706827702158299195987

1468981937607192612948115396
 216778 

216778 
216778 
216778 
216778 
216778 
216778 
111111
species

1835141511702741735803
 1793721 

1793721 
1793721 
1793721 
1793721 
1793721 
1793721 
111111
species

111111
species
936164204404172148425377999

1904944 
1904944 
1904944 
1904944 
1904944 
1904944 
 1904944 

species
111111
37162480238159526331491
 2005046 

2005046 
2005046 
2005046 
2005046 
2005046 
2005046 


128780 
128780 
128780 
128780 
128780 
128780 
 128780 
49523476301384831182113
111111
species

111111
species
 1827305 

1827305 
1827305 
1827305 
1827305 
1827305 
1827305 
47313686330074931451809

species group
111111
995085
1336079764087962208259045265640

1336079764087962208259045265640

40324 
40324 
40324 
40324 
40324 
40324 
 40324 
111111
species

species
111111
1455140312183371731736
 83617 

83617 
83617 
83617 
83617 
83617 
83617 

151515151515
genus
984967690567271204127336954434
338

 90270 

90270 
90270 
90270 
90270 
90270 
90270 
152210319693131111632
species
111111

671509490153485304
 56454 

56454 
56454 
56454 
56454 
56454 
56454 
species
111111

 442694 

442694 
442694 
442694 
442694 
442694 
442694 
757622510163623431
111111
species

479363847433567101363541128101
643453
species group
111111

species
111111

346 
346 
346 
346 
346 
346 
 346 
479363847433567101363541128101


1985254 
1985254 
1985254 
1985254 
1985254 
1985254 
 1985254 
150710097212551075629
111111
species

1711123312883381199906
 56458 

56458 
56458 
56458 
56458 
56458 
56458 
species
111111

species
111111
1768122911223681422807

456327 
456327 
456327 
456327 
456327 
456327 
 456327 

1143882887404230782285926
 339 

339 
339 
339 
339 
339 
339 
species
111111

111111
species

56460 
56460 
56460 
56460 
56460 
56460 
 56460 
153795510412871126704


56459 
56459 
56459 
56459 
56459 
56459 
 56459 
358289200106253143
111111
species

49583515326091432512208
 343 

343 
343 
343 
343 
343 
343 
species
111111

species
111111

347 
347 
347 
347 
347 
347 
 347 
21702180401513245541713212436

111111
species
1974135012233931624945

48664 
48664 
48664 
48664 
48664 
48664 
 48664 

111111
species
47224923589317173
 29447 

29447 
29447 
29447 
29447 
29447 
29447 

species
111111

53413 
53413 
53413 
53413 
53413 
53413 
 53413 
1851121093611289


2070539 
2070539 
 2070539 
218
11
species

order
333333
1692040
14149475512493

14149475512493
1692041
family
333333

genus
111111
702823166942
1744881

702823166942

1620215 
1620215 
1620215 
1620215 
1620215 
1620215 
 1620215 
species
111111

111111
genus
3132182630
986106

3132182630
 1281578 

1281578 
1281578 
1281578 
1281578 
1281578 
1281578 
111111
species

genus
111111
401822212921
1692042


1675686 
1675686 
1675686 
1675686 
1675686 
1675686 
 1675686 
401822212921
111111
species

order
403844403837
135619
119761789710321520991

111111
family
4472202
191033

141450
4472202
111111
genus

species
111111
4472202

141451 
141451 
141451 
141451 
141451 
141451 
 141451 

111111
family
224379
1431291119

1431291119
158481
111111
genus

111111
species
1431291119

158327 
158327 
158327 
158327 
158327 
158327 
 158327 

135620
10842114134126129
family
657666

187492
25425192748
genus
111111

species
111111

187493 
187493 
187493 
187493 
187493 
187493 
 187493 
25425192748

17819281111
1537406
111111
genus


1249553 
1249553 
1249553 
1249553 
1249553 
1249553 
 1249553 
17819281111
111111
species

genus
111111
452520697220
48075

species
111111
452520697220

1821621 
1821621 
1821621 
1821621 
1821621 
1821621 
 1821621 

17535181248
28253
genus
223322

111
species


119864 
119864 
119864 
 119864 
3112


936476 
936476 
936476 
936476 
936476 
936476 
 936476 
102166326
species
111111

 400668 

400668 

400668 
400668 
400668 
400668 
7810922
species
11111

1111
genus
41542
188907

species
1111
41542

188908 

188908 

188908 
188908 
 188908 

666666
family
17050123148203117
224372

101201461
2025617
111111
genus

111111
species
 1917421 

1917421 
1917421 
1917421 
1917421 
1917421 
1917421 
101201461

555555
genus
59753
16049103134197116

species
111111
 59754 

59754 
59754 
59754 
59754 
59754 
59754 
1027873

 1113728 

1113728 
1113728 
1113728 
1113728 
1113728 
1113728 
811313293
111111
species

species
111111
 285091 

285091 
285091 
285091 
285091 
285091 
285091 
332526225023

 1094342 

1094342 
1094342 
1094342 
1094342 
1094342 
1094342 
56319373938
111111
species

111111
species

1306787 
1306787 
1306787 
1306787 
1306787 
1306787 
 1306787 
531838547249

222222
family
1381028514
255527

230494
7634333
111111
genus

111111
species
 1336806 

1336806 
1336806 
1336806 
1336806 
1336806 
1336806 
7634333

1445504
62724181
111111
genus

111111
species

1445505 
1445505 
1445505 
1445505 
1445505 
1445505 
 1445505 
62724181

222123212020
family
8835076087001102714
28256

111111
genus
376488
816612367941

816612367941
 376489 

376489 
376489 
376489 
376489 
376489 
376489 
111111
species

genus
222222
504090
625541589577

342418134161
 698828 

698828 
698828 
698828 
698828 
698828 
698828 
111111
species


157779 
157779 
157779 
157779 
157779 
157779 
 157779 
283123455416
111111
species

genus
111111
6629305610137
42054

 158080 

158080 
158080 
158080 
158080 
158080 
158080 
6629305610137
111111
species

genus
111111
404432
722745506628

 1771309 

1771309 
1771309 
1771309 
1771309 
1771309 
1771309 
722745506628
species
111111

2745
436256316410658456
131314131313
genus

species
111111
652927556738
 1897729 

1897729 
1897729 
1897729 
1897729 
1897729 
1897729 

 213554 

213554 
213554 
213554 
213554 
213554 
213554 
644674
species
111111


1610576 
1610576 
1610576 
1610576 
1610576 
1610576 
 1610576 
12713172022
111111
species

1


1962264 
 1962264 
1
species

111111
species
522227508649
 2746 

2746 
2746 
2746 
2746 
2746 
2746 

species
111111
232835172358

115561 
115561 
115561 
115561 
115561 
115561 
 115561 

4454454111960
 475662 

475662 
475662 
475662 
475662 
475662 
475662 
species
111111

111111
species
582755626540

2136172 
2136172 
2136172 
2136172 
2136172 
2136172 
 2136172 

species
111111
7033436212075
 1883416 

1883416 
1883416 
1883416 
1883416 
1883416 
1883416 

111111
species
11118111913

1504981 
1504981 
1504981 
1504981 
1504981 
1504981 
 1504981 


507626 
507626 
507626 
507626 
507626 
507626 
 507626 
542219339571
111111
species

111111
species
 1971364 

1971364 
1971364 
1971364 
1971364 
1971364 
1971364 
7217111

species
111111
 1178482 

1178482 
1178482 
1178482 
1178482 
1178482 
1178482 
281023312023

111111
species
67151862
 1118153 

1118153 
1118153 
1118153 
1118153 
1118153 
1118153 

22
1495768
genus
11

 1495769 

1495769 

1495769 
22
species
11

genus
1111
3231
114185

3231

114186 
114186 
114186 
114186 
 114186 
1111
species

1121929559256
204286
genus
111111


28258 
28258 
28258 
28258 
28258 
28258 
 28258 
1121929559256
species
111111

111111
genus
4953130341119
235572

4953130341119

91844 
91844 
91844 
91844 
91844 
91844 
 91844 
species
111111

1920240
53231176
224321
family

224321
genus
261963
53231176

4136
 261964 

261964 
261964 
261964 
261964 
1111
species

 1561924 


1561924 
1561924 
1561924 
943
111
species


914150 
914150 
 914150 
25
11
species

16146
 1144748 

1144748 

1144748 
1144748 
1144748 
1144748 
11111
species

111111
genus
349742
1510994640

species
111111
1510994640
 1543721 

1543721 
1543721 
1543721 
1543721 
1543721 
1543721 

343239363335
order
135623
7935691459141211101654

641
7935691459141211101654
343239363335
family

262160635236
657
genus
233333

11111
species


74109 
74109 
74109 
74109 
74109 
 74109 
214633

species
111111
 38293 

38293 
38293 
38293 
38293 
38293 
38293 
8537242910

111111
species
18149332023
 1295392 

1295392 
1295392 
1295392 
1295392 
1295392 
1295392 

246861
1331015922
111111
genus

111111
species

673 
673 
673 
673 
673 
673 
 673 
1331015922

511678
1022261672
233222
genus


80852 
80852 
80852 


80852 
 80852 
1531
1111
species


668 
668 
668 
668 
668 
668 
 668 
912161251
111111
species

1111
species


40269 
40269 
40269 
40269 
 40269 
5742

293
2042066
111
genus

111
species
293

1755811 
1755811 
1755811 
 1755811 

282431302729
genus
662
7425141360131810421594

11111
species
 687 

687 

687 
687 
687 
687 
3618512

species
111111

672 
672 
672 
672 
672 
672 
 672 
954913520093153

1891919
2118
111
species group

 29498 


29498 
29498 

29498 
2118
species
111

113656

2014742 

2014742 
2014742 
2014742 
2014742 
 2014742 
11111
species

11111
species
 553239 

553239 
553239 
553239 
553239 

553239 
84574

111111
species
98511518
 1435069 

1435069 
1435069 
1435069 
1435069 
1435069 
1435069 

135414626
 52443 

52443 
52443 
52443 
52443 
52443 
52443 
111111
species

 676 

676 
676 
676 
676 
676 
676 
4729436069150
species
111111

species
111111
114331

212663 
212663 
212663 
212663 
212663 
212663 
 212663 


674 
674 
674 
674 
674 
674 
 674 
4419152610
111111
species

11111
species
 673372 

673372 

673372 
673372 
673372 
673372 
1211131

species group
88101089
717610
235174559382278441


696485 
696485 
696485 
696485 
696485 
696485 
 696485 
1182810208
species
111111

898015614986142

670 
670 
670 
670 
670 
670 
 670 
111111
species

111111
species
 680 

680 
680 
680 
680 
680 
680 
4040120815141

1111
species


150340 
150340 
150340 

150340 
 150340 
21291

1111
species

766224 

766224 
766224 

766224 
 766224 
3614

species
111111
2872812136
 50719 

50719 
50719 
50719 
50719 
50719 
50719 

 663 

663 
663 
663 
663 
663 
663 
311784507693
111111
species


691 
691 
691 
691 
691 
691 
 691 
26567341460
111111
species

species
111
1667
 190895 


190895 
190895 
190895 

17542301186

669 
669 
669 
669 
669 
669 
 669 
111111
species

342890844652
 55601 

55601 
55601 
55601 
55601 
55601 
55601 
111111
species

species
111
428


689 


689 
689 
 689 

species
11111
381544

76258 

76258 
76258 
76258 
76258 
 76258 

species
111111
13818283245
 190893 

190893 
190893 
190893 
190893 
190893 
190893 

111111
species
991413252

29494 
29494 
29494 
29494 
29494 
29494 
 29494 

6693814
 45658 

45658 
45658 
45658 
45658 
45658 
45658 
111111
species

11111
species
33582

28173 
28173 
28173 
28173 
28173 
 28173 

species
1111
55516

2025808 

2025808 
2025808 

2025808 
 2025808 

8929
 1116375 

1116375 

1116375 
1116375 

1116375 
species
1111

111111
species
 666 

666 
666 
666 
666 
666 
666 
242171367435398619

111111
species
 1534743 

1534743 
1534743 
1534743 
1534743 
1534743 
1534743 
29108143

291819103435
1807140
class
333233

333233
order
291819103435
225057

225058
291819103435
family
333233

119977
291819103435
333233
genus

species
11111
 160808 

160808 
160808 
160808 

160808 
160808 
61172

18664728

33059 
33059 
33059 
33059 
33059 
33059 
 33059 
111111
species

511126205
 920 

920 
920 
920 
920 
920 
920 
species
111111

67819
53785
phylum
11111

53785
1663419
11111
class

53785
1663425
11111
order

1663426
53785
family
11111

1005038
53785
11111
genus


1005039 
1005039 

1005039 
1005039 
1005039 
 1005039 
53785
11111
species

68297
1
phylum
1

1
203486
class
1

order
1
1
203487

family
1
203488
1

genus
1
1
13

species
1
1


513050 
 513050 

phylum
8115784
200918
34442912187

class
8115784
34442912187
188708

2
order
1643946
21

2
family
21
1643948

20
1184396
1
genus

species
1
20


1184387 
 1184387 

651456
1
1
genus

species
1
1


1330330 
 1330330 

2813196176
2419
order
663573

family
442121
1643950
241115241

221111
genus
2420
1732231


2421 
2421 
2421 
 2421 
1512
111
species


1437364 
 1437364 
2
species
1

231
 1462747 


1462747 


1462747 
1462747 
111
species

 46541 

46541 
2
1
species

genus
2211
78131
2422

67131

93466 
93466 
93466 

93466 
 93466 
species
1111

1
 2424 


2424 
species
1

 2423 

2423 
1
species
1

188709
4244135
family
221452

11429
2335
genus
11123

11
species
14


1508420 


1508420 
 1508420 

species
1
1


2336 
 2336 

1
species
1
 1508419 

1508419 


126738 
 126738 
1
1
species

1
 1157947 


1157947 
1
species

1
species


93929 
 93929 
4

4


126740 
 126740 
1
species

31245
1643951
genus
11222

11


177758 

177758 
 177758 
species
11

1
 119394 


119394 
1
species

1111
species
3134

57487 


57487 
57487 
57487 
 57487 

1
species


38322 
 38322 
1

61010611
1643947
232211
order

691041
160798
genus
22211

132
 149715 

149715 
149715 
149715 
species
111

 1545835 

1545835 
1545835 
1545835 
1545835 
1545835 
56841
11111
species

121
1511648
genus
111

species
111
121
 1006576 


1006576 

1006576 

1006576
