# Supplementary material for: Characterisation of the Carpinus betulus L. Phyllomicrobiome in Urban and Forest Areas
Source: Front Microbiol. 2019 May 29;10:1110. doi: 10.3389/fmicb.2019.01110 (PMC6549492; doi:10.3389/fmicb.2019.01110)
Supplement: Supplementary file 2 [file Data_Sheet_1.ZIP › CLARK_epBi.html]

Javascript must be enabled to view this page.

members
magnitude
magnitudeUnassigned
count
unassigned
taxon
rank

epBi41
epBi42
epBi43
epBi51
epBi53
epBi32

256424752608259026982604
249897728330281940039236542821167961775638

249924152536252426302544
2
superkingdom
249858928326161939561236508621164501775109

1077887
phylum
784946442534
1090

class
784946442534
191410
1077887

order
784946442534
191411
1077887

1077887
784946442534
family
191412

11
genus
1711
100715

species

100716 
100716 
1711
 100716 
11

333333
genus
462616291226
256319

111111
species

274539 
274539 
274539 
274539 
274539 
274539 
41161019521
 274539 

111111
species

274537 
274537 
274537 
274537 
274537 
274537 
431554
 274537 

species

1097 
1097 
1097 
1097 
1097 
1097 
175521
 1097 
111111

1091
4420751
genus
212221

species
3419641

1096 
1096 
1096 
1096 
1096 
1096 
 1096 
111111

111
species


1094 
1094 
1094 
111
 1094 

1
 1092 
1

1092 
species

21212
genus
73746
1099

1111
 34090 
1312

34090 

34090 
34090 

34090 
species

6644

1100 


1100 
1100 
1100 
species
 1100 
1111

1101
genus
487141
221121

 281093 

281093 
281093 
281093 
281093 
281093 
35713
species
11111

1111
 1102 

1102 
1102 


1102 
1102 
1311
species

3716927913
phylum
200918
755534

class
3716927913
188708
755534

21131
421174
order
1643947

160798
32144
genus
11121

11


149715 
149715 
species
 149715 
11

1111
 1545835 
species
3234

1545835 
1545835 

1545835 

1545835 

1511648
genus
113
11

 1006576 

1006576 


1006576 
113
species
11

443233
2419
321471099
order

322121
3193885
family
188709

21112
2335
32188
genus

 126740 
26

126740 


126740 
species
11

11
 2336 
species

2336 
2336 
12

1
2


93929 
species
 93929 

1
species


126738 
1
 126738 

1
species


93930 
8
 93930 

1111
28725
genus
1643951

 57487 

57487 
57487 
57487 


57487 
28725
species
1111

121112
1643950
family
154214

2420
genus
11
11

1
species
1

1437364 
 1437364 

species
1


46541 
 46541 
1

44214
genus
2422
11112


93466 
93466 
93466 
93466 
93466 
44213
species
 93466 
11111

1
species


2424 
1
 2424 

11
order
1643946
11

1643948
family
11
11

genus
1
1184396
1

1


1184387 
1
species
 1184387 

1
1
genus
651456

 651457 
species
1

651457 
1

12
95818
14
phylum

 1476577 


1476577 
1476577 
12
species
11

1


2056494 
2
species
 2056494 

11
phylum
14
200940

67799
14
class
11

14
order
188710
11

family
14
188711
11

444090
1
genus
1

1


1653476 
species
 1653476 
1

1
241192
genus
4

1
 171695 
4


171695 
species

1
456828
1
phylum

1
456826
1
genus

species
1


456827 
 456827 
1

142182
713332315639
phylum
333333

333333
class
713332315639
219685

713332315639
order
219686
333333

713332315639
family
219687
333333

222222
157216229
genus
173479


173480 
173480 
173480 
173480 
173480 
173480 
1239487
species
 173480 
111111

111111
species
34122142

1379270 
1379270 
1379270 
1379270 
1379270 
1379270 
 1379270 

genus
562611253430
1706036
111111

species
562611253430

861299 
861299 
861299 
861299 
861299 
861299 
 861299 
111111

242225242420
218192240342397179
phylum
1297

242225242420
218192240342397179
class
188787

141314141414
166154175255337144
order
118964

11111
family
44553
332247

11111
44553
genus
332248

 332249 
species
44553

332249 

332249 
332249 
332249 
332249 
11111

131313131313
183710
family
162154171250332141

1298
genus
162154171250332141
131313131313

111111

2202254 
2202254 
2202254 
2202254 
2202254 
2202254 
81622212332
species
 2202254 

111111
 432329 
species
2111422171

432329 
432329 
432329 
432329 
432329 
432329 

111111
 1299 
species

1299 
1299 
1299 
1299 
1299 
1299 
14432293111

111111

1768108 
1768108 
1768108 
1768108 
1768108 
1768108 
263116217222
species
 1768108 

 2080419 
species

2080419 
2080419 
2080419 
2080419 
2080419 
2080419 
1071111207
111111

 1182568 
228561

1182568 
1182568 
1182568 
1182568 
1182568 
1182568 
species
111111

111111
species

1182571 
1182571 
1182571 
1182571 
1182571 
1182571 
10714243422
 1182571 

species

310783 
310783 
310783 
310783 
310783 
310783 
1114111114
 310783 
111111

species
10514325623

502394 
502394 
502394 
502394 
502394 
502394 
 502394 
111111

 55148 
species
16215103

55148 
55148 
55148 
55148 
55148 
55148 
111111

 68909 
species

68909 
68909 
68909 
68909 
68909 
68909 
832291
111111

111111
117836265

309887 
309887 
309887 
309887 
309887 
309887 
species
 309887 

111111

1309411 
1309411 
1309411 
1309411 
1309411 
1309411 
3053721179
species
 1309411 

1091110106
68933
523865876035
order

188786
523865876035
family
1091110106

11111
186191
genus
651696

11111
 186192 
species
651696

186192 
186192 
186192 
186192 
186192 

111111
208447
genus
174910213

111111
 187137 

187137 
187137 
187137 
187137 
187137 
187137 
174910213
species

112222
65551
genus
268161210

 277 
31115


277 
277 
277 
277 
species
1111

 52022 
species

52022 
52022 
52022 
52022 
52022 
52022 
2655115
111111

767663
270
272332524012
genus


1111069 
1111069 
1111069 
1111069 
1111069 
1111069 
6352101
species
 1111069 
111111

111111
species

274 
274 
274 
274 
274 
274 
121214271910
 274 

11111
32525

37636 
37636 
37636 
37636 
37636 
species
 37636 

111111
 56957 
142411

56957 
56957 
56957 
56957 
56957 
56957 
species

species

456163 
456163 
456163 
456163 
456163 
113133
 456163 
11111

11111
 56956 
21142

56956 
56956 
56956 
56956 
56956 
species

11
species
22

271 

271 
 271 

74152
phylum
1414
1112

class
1414
447830
1112

1783344
order
1414
1112

1112
family
1414
1783343

1408194
genus
1414
1112

species


1408281 
1408281 
1408281 
1408281 
1413
 1408281 
1111

 1408204 
species
1


1408204 
1

phylum
301287243381266368
1117
496159566770

432313422821
class
307596
222222

222222
order
432313422821
307595

222222
432313422821
family
1890422

222222
432313422821
genus
33071

 33072 

33072 
33072 
33072 
33072 
33072 
33072 
2156341111
species
111111

111111
 1416614 
2218781710

1416614 
1416614 
1416614 
1416614 
1416614 
1416614 
species

1890424
order
1167611514510692
212127232925

111
family
1890429
111

111
111
genus
155977

111

155978 


155978 
155978 
111
species
 155978 

11111
1890436
32132
family

11111
genus
32132
1152

11111
 82654 
species

82654 
82654 
82654 

82654 
82654 
32132

21121
family
79576
1890428

21121
79576
genus
1142

1


2116702 
2
species
 2116702 


1148 
1148 
1148 

1148 
1148 
69556
species
 1148 
11111

1

1147 
species
 1147 
1

1213
family
2712239
121121

1218
genus
2712239
121121

111111
 1219 

1219 
1219 
1219 
1219 
1219 
1219 
2612229
species

1
 1501268 
1


1501268 
species

1


1501269 
1
species
 1501269 

121219181918
1890426
7347911138562
family

13034
genus
5412
1111

 292566 
species
5412


292566 
292566 

292566 
292566 
1111

10916151615
1129
genus
593854604847

 321327 
species

321327 

321327 

321327 
121
111

species
11


316278 

316278 
 316278 
11

 64471 
12


64471 


64471 
species
11

11111
 110662 
species

110662 

110662 
110662 
110662 
110662 
73517

 32051 


32051 
32051 
32051 
32051 
32051 
111175
species
11111

 374982 


374982 
1
species
1


1827144 


1827144 
12
species
 1827144 
11

 316279 


316279 
316279 
12
species
11

 84588 
species


84588 
84588 
84588 
84588 
22594
1111

 32046 
314114


32046 
32046 
32046 
32046 
32046 
species
11111


166314 
166314 
166314 
166314 
4113
species
 166314 
1111

11111
species

374981 

374981 
374981 
374981 
374981 
51679
 374981 

1111
 195253 


195253 
195253 

195253 
195253 
1113
species

 1350461 
1


1350461 
species
1

1
 195498 
2

195498 
species

11111
251551

1916956 
1916956 

1916956 
1916956 
1916956 
species
 1916956 

11111
species

29410 
29410 
29410 
29410 

29410 
51582
 29410 


1280380 

1280380 
1280380 
1280380 
4221
species
 1280380 
1111

111111

585425 
585425 
585425 
585425 
585425 
585425 
436712
species
 585425 

11
 1173263 
species


1173263 
1173263 
11

1
 32049 
1


32049 
species

236934

321332 
321332 
321332 
321332 
321332 
321332 
species
 321332 
111111

111111
species
431311

585423 
585423 
585423 
585423 
585423 
585423 
 585423 

222221
167375
genus
14433523611

 59930 
species
8128507

59930 
59930 
59930 
59930 
59930 
11111

 1851505 

1851505 
1851505 
1851505 
1851505 
1851505 
1851505 
63522911
species
111111

11
146785
genus
12

11
species


146786 

146786 
12
 146786 

455344
1890438
family
3011179713

344333
47251
279129611
genus

111111
 1184 
species
225213

1184 
1184 
1184 
1184 
1184 
1184 

 111781 
311116

111781 
111781 
111781 
111781 
111781 
111781 
species
111111

111111
 1080068 
species
2255642

1080068 
1080068 
1080068 
1080068 
1080068 
1080068 

 1752064 


1752064 
1752064 
11
species
11

11111
genus
32512
170610

11111
species

1209493 
1209493 
1209493 

1209493 
1209493 
32512
 1209493 

11
1890505
11
order

1890528
family
11
11

54298
genus
11
11

 54299 
species
11


54299 
54299 
11

1301283
subclass
82116488963140
111511121216

352457
152719392870
order
1118

11
1890452
11
family

102234
11
genus
11

11
11


379064 
379064 
species
 379064 

1111
family
1890450
1111

1111
76023
genus
1111

 65093 

65093 
65093 


65093 
65093 
1111
species
1111

111112
1890449
121818121453
family

111112
121818121453
genus
1125

111111
 1126 
species
121818121452

1126 
1126 
1126 
1126 
1126 
1126 

1
 1967666 


1967666 
1
species

131224
1890464
281261216
family

genus
113
268175
111

111
 2005460 
113


2005460 

2005460 

2005460 
species

111112
genus
261251111
669357

 1617448 
species

1617448 
1617448 
1617448 

1617448 
1617448 
2611110
11111

11
 1615909 
251


1615909 

1615909 
species

112
genus
102231
111

 1173026 
112


1173026 


1173026 
1173026 
species
111

8109879
order
678929503570
1150

1892252
family
115141616
221213

35823
411167
genus
11111

11111
 2153484 
species


2153484 
2153484 
2153484 
2153484 
2153484 
411167

1111
44471
genus
4138

1111
 1173027 
species

1173027 
1173027 

1173027 

1173027 
4138

11
1205
genus
71

11
 1206 
71

1206 


1206 
species

111111
1892251
45112123
family

111111
63132
45112123
genus

111111
species

1173025 
1173025 
1173025 
1173025 
1173025 
1173025 
45112123
 1173025 

2111
family
1892255
1111

genus
2111
241421
1111

 241425 
2111


241425 
241425 
241425 
241425 
species
1111

1892249
family
659192616
233112

233112
genus
659192616
43988

215


41431 
41431 


41431 
species
 41431 
111


395961 
5
species
 395961 
1

 43989 
1561626

43989 
43989 
43989 
43989 
species
1111

11


65393 

65393 
21
species
 65393 

11
species
11


497965 


497965 
 497965 

4618771625
family
1892254
333333

genus
28422717
1155738
111111

 1155739 
28422717

1155739 
1155739 
1155739 
1155739 
1155739 
1155739 
species
111111

222222
18145598
genus
1158

 118323 

118323 
118323 
118323 
118323 
118323 
118323 
1552345
species
111111

111111
393253

482564 
482564 
482564 
482564 
482564 
482564 
species
 482564 

111111
 718217 
species
316117220

718217 
718217 
718217 
718217 
718217 
718217 

order
4259
52604
2123

1890500
1235
family
1111

44474
1235
genus
1111

1235


54308 
54308 

54308 
54308 
species
 54308 
1111

1890498
family
324
112

112
102115
genus
324

1
species


1807358 
3
 1807358 

111
321


102116 


102116 
102116 
species
 102116 

575153986286
order
1161
141916182123

1892263
family
111125
111111

111111
genus
111125
1190

111125

1752063 
1752063 
1752063 
1752063 
1752063 
1752063 
species
 1752063 
111111

 1940762 
species
13


1940762 
1940762 
11

1162
443642664056
family
81111101313

264688
313341
genus
112221

1111
 1164 


1164 
1164 
1164 
1164 
1211
species

species
31213

264691 
264691 
264691 
264691 
264691 
 264691 
11111

genus
7237510
1163
31233

11111
 46234 


46234 
46234 
46234 
46234 
46234 
421235
species

111
 1165 
species


1165 


1165 
1165 
111

 1647413 


1647413 

1647413 
1647413 
1647413 
22514
species
1111

778689
1177
412837263145
genus

11
 103690 
53


103690 
103690 
species

95101016

1751286 
1751286 
1751286 
1751286 
1751286 
1751286 
species
 1751286 
111111

species

1261031 
1261031 
1261031 
1261031 
1261031 
1261031 
114124
 1261031 
111111

species
122123

1618022 
1618022 
1618022 

1618022 
1618022 
 1618022 
11111

 1869241 
species

1869241 
1869241 
1869241 
1869241 
1869241 
1869241 
135114
111111

species


317936 
317936 

317936 
317936 
1322
 317936 
1111

species

1306274 

1306274 
1306274 
1306274 
1306274 
2211032
 1306274 
11111

 272131 
species

272131 
272131 
272131 
272131 
272131 
272131 
615111514
111111

11111
 28072 
11137

28072 
28072 
28072 
28072 

28072 
species

1892259
111
family
111

genus
111
159191
111

111
 70799 
111

70799 


70799 

70799 
species

474667
1185
family
111410301921

11111
431105
genus
373984

 373994 

373994 
373994 

373994 
373994 
373994 
431105
species
11111

7111029916
genus
1186
364556

1111
species
1341


99598 
99598 

99598 
99598 
 99598 

 1954171 
1111


1954171 

1954171 
1954171 
1954171 
species
1111

1111
 2005469 
species

2005469 
2005469 

2005469 
2005469 
2261

 1337936 


1337936 

1337936 

1337936 
346
species
111

11
 2005462 
species


2005462 


2005462 
31

species
3211515

32054 
32054 
32054 
32054 
32054 
32054 
 32054 
111111

111111
 2005461 

2005461 
2005461 
2005461 
2005461 
2005461 
2005461 
223322
species

111111
1930617
7339718
phylum

111111
class
7339718
1962850

111111
1962852
7339718
order

111111
1962854
7339718
family

genus
7339718
187144
111111

 187145 
species

187145 
187145 
187145 
187145 
187145 
187145 
7339718
111111

111111
256845
3312217137
phylum

1313211
3312217137
class
111111

3312217137
order
278082
111111

species
3312217137

2094242 
2094242 
2094242 
2094242 
2094242 
2094242 
 2094242 
111111

778778
74201
6828471035565
phylum

414999
381524604323
class
444444

415001
711336
order
111111

111111
711336
family
415002

111111
genus
711336
442430

 395922 
species
711336

395922 
395922 
395922 
395922 
395922 
395922 
111111

311423574017
order
415000
333333

311423574017
family
134623
333333

 794903 
species
127111283

794903 
794903 
794903 
794903 
794903 
794903 
111111

111111
178440
613672
genus

 107709 

107709 
107709 
107709 
107709 
107709 
107709 
613672
species
111111

111111
genus
1369392512
1961799

species
1369392512

1838286 
1838286 
1838286 
1838286 
1838286 
1838286 
 1838286 
111111

class
11
134549
11

11
genus
134550
11


1704307 


1704307 
11
species
 1704307 
11

class
2411152069
203494
222222

48461
2411152069
order
222222

1647988
2411152069
family
222222

239934
2411152069
genus
222222

111111
 239935 

239935 
239935 
239935 
239935 
239935 
239935 
229101756
species

111111
225313

1679444 
1679444 
1679444 
1679444 
1679444 
1679444 
species
 1679444 

111111
species

1637999 
1637999 
1637999 
1637999 
1637999 
1637999 
62723632
 1637999 

869569
phylum
142149352533
32066

869569
142149352533
class
203490

203491
order
142149352533
869569

family
111343152025
203492
535346

167639
214
genus
111

111
species

167642 

167642 

167642 
214
 167642 

genus
91342151625
848
434336

species
3


861 
 861 
1

111111

860 
860 
860 
860 
860 
860 
191410911
species
 860 

1


856 
species
 856 
1

11


849 


849 
151
species
 849 

 851 
species
5310147

851 
851 
851 
851 
851 
851 
111111

111
species

850 

850 


850 
233
 850 

1142

1583098 
1583098 

1583098 

1583098 
species
 1583098 
1111

334223
3862058
family
1129771

32067
2722056
genus
222222

111111
species

40542 
40542 
40542 
40542 
40542 
40542 
161114
 40542 

1
species


712357 
1
 712357 

11111
 712368 
species

712368 
712368 

712368 
712368 
712368 
111942

168808
genus
1
1

1
1


187101 
species
 187101 

1132
genus
34104
1111

1111
species

34105 
34105 
34105 


34105 
1132
 34105 

12881010
phylum
134625
111111

12881010
class
1921781
111111

1921782
order
12881010
111111

111111
1921783
family
12881010

111111
1921784
genus
12881010

111111
 1307763 
species
12881010

1307763 
1307763 
1307763 
1307763 
1307763 
1307763 

1239
309539231309644773380032147
phylum
277261324291356332

class
726061884948
909932
91214131210

order
346321
1843488
122221

122221
346321
family
909930

122221
346321
genus
904

 905 
species
322211

905 
905 
905 
905 
905 
905 
111111

species


187327 
187327 
187327 
187327 
2411
 187327 
1111

255444
1843489
384534463230
order

255444
31977
family
384534463230

111111
909928
101016161010
genus


1702287 
1702287 
1702287 
1702287 
1702287 
1702287 
101016161010
species
 1702287 
111111

1211
6561
genus
29465

1
 248315 
species
1


248315 

 29466 


29466 
29466 
29466 
29466 
6461
species
1111

21112
39948
genus
41722

 39950 
11


39950 


39950 
species
11

 2161821 
species
31721


2161821 
2161821 
2161821 
2161821 
2161821 
11111

906
genus
282512171918
111111

282512171918

907 
907 
907 
907 
907 
907 
species
 907 
111111

order
311121391517
909929
657765

family
425132
1843490
112122

112122
genus
425132
365348

1111
 484770 
species


484770 
484770 
484770 
484770 
1121

11111
 365349 
species
42411

365349 
365349 
365349 

365349 
365349 

27916381215
family
1843491
545643

genus
27916371215
970
545543

11111
 69823 

69823 
69823 
69823 
69823 
69823 
111123
species

11111
 712538 
species

712538 
712538 
712538 
712538 
712538 
102763

331323

1884263 
1884263 
1884263 
1884263 
1884263 
1884263 
species
 1884263 
111111

 971 
13622411

971 
971 
971 
971 
971 
971 
species
111111

species
2141

713030 

713030 
713030 

713030 
 713030 
1111

1
158846
genus
1


158847 
1
species
 158847 
1

233431
class
2392443
1737404

1
genus
2
1582879

 1852374 
species
2


1852374 
1

1737405
2392423
order
233421

233321
family
239623
1570339

162289
genus
1
1

species
1


1912856 
 1912856 
1

150022
16413
genus
11111

11111
species


1260 
1260 
1260 
1260 
1260 
16413
 1260 

1111
165779
genus
1211


1870984 
1870984 
11
species
 1870984 
11

11
species
12

33034 

33034 
 33034 

11
genus
11
543311

11
 33033 
species
11


33033 

33033 

genus
11
1161127
11

 1852373 
species
11

1852373 

1852373 
11

2042895
18
family
1

18
genus
1505664
1

species
18


1556 
 1556 
1

1111
genus
1111
1930845

 1871025 
1111


1871025 

1871025 
1871025 
1871025 
species
1111

324113
15363612
class
526524

526525
15363612
order
324113

324113
15363612
family
128827

1729679
1124
genus
1111

1111

1702221 
1702221 
1702221 


1702221 
1124
species
 1702221 

1
species
6

1834207 
 1834207 

823365
genus
1647
112111

 1648 
822365

1648 
1648 
1648 
1648 
1648 
1648 
species
111111


1514105 
1
species
 1514105 
1

191303
13
genus
11

 1712675 
13


1712675 


1712675 
species
11

class
222831111180535463293430972
91061
186182236200267246

64699371105102
50651889635412619628473
order
186826

186828
family
179825952507327457
217166

11
11
genus
191769

 1911586 
species
11


1911586 


1911586 
11

1221
2463
genus
1470540

 708126 

708126 

708126 

708126 
224
species
111

111
species
223


1903686 

1903686 
1903686 
 1903686 

114144
2747
genus
159825452506727453

111
species
53334


208596 

208596 
208596 
 208596 

111
 1564681 
species
43126


1564681 

1564681 
1564681 

 147709 
51322


147709 

147709 
147709 
species
111

111111
species

2751 
2751 
2751 
2751 
2751 
2751 
159824052499027371
 2751 

family
6875158
186827
334255

1375
6875158
genus
334255

1
species


119206 
2
 119206 

 87541 
species
151


87541 

87541 
87541 
111


51665 
51665 
51665 
51665 
51665 
51665 
354353
species
 51665 
111111

11111
species
12221

1377 
1377 

1377 
1377 
1377 
 1377 

1111
1112


1376 
1376 

1376 
1376 
species
 1376 

species

128944 

128944 


128944 
211
 128944 
111

81850
family
411213201830
745899

1243
389791018
genus
522444

111111
species
18565513

1245 
1245 
1245 
1245 
1245 
1245 
 1245 

11
 33964 
species
21


33964 
33964 

species

1252 

1252 
1252 

1252 
6111
 1252 
1111

species

1244 
1244 

1244 

1244 
1413
 1244 
1111

11
 1511761 

1511761 


1511761 
41
species

 1246 
species

1246 


1246 
93
11

1
species
1


136609 
 136609 

111
46254
121
genus

111
 1247 
121


1247 
1247 
1247 
species

46255
genus
33610611
223344

111111

137591 
137591 
137591 
137591 
137591 
137591 
224734
species
 137591 

 759620 
species
111

759620 


759620 
759620 
111

11111
species


165096 
165096 
165096 
165096 
165096 
11215
 165096 

 1631871 
species
11


1631871 
1631871 
11

11


1249 


1249 
species
 1249 
11

81852
1227115592477354
family
10101591616

1350
1227015191467335
genus
1091281212

11111

1353 
1353 
1353 

1353 
1353 
1412122215
species
 1353 

 2005703 
species


2005703 

2005703 
2005703 
142
111

11111
 53346 

53346 
53346 
53346 

53346 
53346 
11178
species

111111

1351 
1351 
1351 
1351 
1351 
1351 
1943725172132
species
 1351 

species

2057791 
2057791 
2057791 
2057791 
2057791 
2057791 
181444125
 2057791 
111111

111111

2060307 
2060307 
2060307 
2060307 
2060307 
2060307 
1981114195
species
 2060307 


1352 
1352 
1352 
1352 
1352 
1352 
14154937164125
species
 1352 
111111

111111
 417368 
112147

417368 
417368 
417368 
417368 
417368 
417368 
species

 37734 

37734 
37734 
37734 
37734 
37734 
37734 
3413156357
species
111111

11111
species
23123


44008 
44008 
44008 
44008 
44008 
 44008 

1111
 53345 
species
11157

53345 

53345 

53345 
53345 


1354 

1354 
1354 
1354 
1354 
11531119
species
 1354 
11111

11
44
genus
51668

11
 51669 


51669 
51669 
44
species

111
genus
214
33969

 33970 
species


33970 

33970 
33970 
214
111

2737
genus
121511
12122

111
 633807 
species


633807 

633807 
633807 
143

species


519472 
519472 
519472 
519472 
519472 
11118
 519472 
11111

108299319283271280
family
1300
192831343231

212112
1357
genus
2052694651

1
 1364 
species
4

1364 

 1358 
species

1358 
1358 
1358 
1358 
1358 
1358 
1652494650
111111

2


1363 
species
 1363 
1

1
 1366 
species


1366 
1

172729333129
1301
genus
88294293274225229

 1346 
8


1346 
species
1

119603
133151115
species group
122222

11111
 1334 
species
221354


1334 
1334 
1334 
1334 
1334 

1112611

1336 
1336 
1336 
1336 
1336 
1336 
species
 1336 
111111

1111
15715

1156431 
1156431 
1156431 
1156431 
species
 1156431 


1303 
1303 
1303 
1303 
1303 
1303 
11313212
species
 1303 
111111

1


1335 
1
species
 1335 

111111
 1318 

1318 
1318 
1318 
1318 
1318 
1318 
1447411
species

11111
32334

1309 

1309 
1309 
1309 
1309 
species
 1309 

 1156433 


1156433 
1156433 
1156433 
1156433 
104112
species
1111

species
331


1902136 
1902136 

1902136 
 1902136 
111

11111
21213

1310 
1310 

1310 
1310 
1310 
species
 1310 

111
 1759399 
species


1759399 
1759399 
1759399 
731

111111
812984472311

1313 
1313 
1313 
1313 
1313 
1313 
species
 1313 

species
11715292328

1307 
1307 
1307 
1307 
1307 
1307 
 1307 
111111

species

1311 
1311 
1311 
1311 
1311 
1311 
23161452938
 1311 
111111

11111
 1305 
10151453


1305 
1305 
1305 
1305 
1305 
species

11111
 28037 
species


28037 
28037 
28037 
28037 
28037 
9131122

11111
 1304 
species
14121443


1304 
1304 
1304 
1304 
1304 

species
11


1348 
1348 
 1348 
11

 1326 


1326 
2
species
1

111111
101226151630

1308 
1308 
1308 
1308 
1308 
1308 
species
 1308 


1314 
1314 
1314 
1314 
1314 
1314 
21829363834
species
 1314 
111111

 150055 
1432

150055 

150055 

150055 
150055 
species
1111

 1811193 
species
1


1811193 
1

 82348 
species


82348 
4
1

1
 1888195 
species


1888195 
1

species


1349 
1349 
1349 
1349 
1349 
13686
 1349 
11111

11111
 102684 

102684 

102684 
102684 
102684 
102684 
12142
species

11
species
11

1825069 


1825069 
 1825069 

132232
671232
17411128
species group

 76860 
species


76860 


76860 
12
11

 1328 
species


1328 
1328 
1328 
1328 
1328 
21317
11111

species
143891

1338 
1338 
1338 
1338 
1338 
1338 
 1338 
111111

 712633 
87322


712633 
712633 
712633 
712633 
712633 
species
11111

11111
species
12135

45634 
45634 
45634 
45634 
45634 
 45634 

 1302 
species


1302 
1302 
1302 
1302 
1302 
5143157
11111

11
species


197614 

197614 
41
 197614 

111111
species

315405 
315405 
315405 
315405 
315405 
315405 
121173
 315405 

11
11


59310 
59310 
species
 59310 

1111
3521


257758 
257758 
257758 
257758 
species
 257758 

 113107 
species
154511


113107 
113107 
113107 
113107 
113107 
11111

111
 1340 
species


1340 

1340 
1340 
121

211


1993866 

1993866 
1993866 
species
 1993866 
111

33958
212119208136341343
family
232330173634

222227173230
210117202136323329
genus
1578

11
 60520 

60520 


60520 
11
species

11


1589 

1589 
11
species
 1589 

species


83683 

83683 
13
 83683 
11

111
 1138822 
713

1138822 


1138822 
1138822 
species

111
 1545702 
123


1545702 

1545702 
1545702 
species

 303541 
1

303541 
species
1

1


52242 
1
species
 52242 

111
 1596 


1596 
1596 

1596 
121
species

11111
species
2621411

1624 

1624 
1624 
1624 
1624 
 1624 

111
species


53444 


53444 
53444 
143
 53444 

species
2345902537102

1599 
1599 
1599 
1599 
1599 
1599 
 1599 
111111

11
31


293371 
293371 
species
 293371 

species


1581 
1581 

1581 
1581 
1161
 1581 
1111

222222
655183
species group
6196191127

127212726

1597 
1597 
1597 
1597 
1597 
1597 
species
 1597 
111111

species

1582 
1582 
1582 
1582 
1582 
1582 
4924741
 1582 
111111

11111
 28038 

28038 
28038 
28038 

28038 
28038 
1141819
species

111111
14438177

1613 
1613 
1613 
1613 
1613 
1613 
species
 1613 


392416 
1
species
 392416 
1

 1622 
species
1

1622 
1

111111
2441136

1598 
1598 
1598 
1598 
1598 
1598 
species
 1598 

 33959 
116952

33959 
33959 
33959 
33959 
33959 
33959 
species
111111

11
 1218494 


1218494 
1218494 
11
species

111
 1610 
species
121


1610 

1610 
1610 

1


1625 
1
species
 1625 

 1587 
1210112411

1587 
1587 
1587 
1587 
1587 
1587 
species
111111

11
23


1847728 
1847728 
species
 1847728 

 47715 
species
534187

47715 
47715 
47715 
47715 
47715 
47715 
111111

1
3


97478 
species
 97478 

1
 2108362 
species
2


2108362 

11111
 1604 
species
11182

1604 
1604 
1604 
1604 
1604 

 1007676 
species


1007676 


1007676 
22
11

11111

375175 
375175 
375175 

375175 
375175 
122363
species
 375175 

1111
 1218493 
1111


1218493 
1218493 

1218493 
1218493 
species

111
 637971 

637971 
637971 
637971 
1672
species

 267363 
114


267363 
267363 


267363 
species
111


1590 
1590 
1590 
1590 
1590 
1590 
13729195745
species
 1590 
111111

111111
 1579 
species
5533212

1579 
1579 
1579 
1579 
1579 
1579 

111111
species

1584 
1584 
1584 
1584 
1584 
1584 
33612153926
 1584 

1111
species


240427 
240427 

240427 
240427 
1129
 240427 

 1623 
18196

1623 
1623 


1623 
1623 
species
1111

111111
978111711

1580 
1580 
1580 
1580 
1580 
1580 
species
 1580 

1253
genus
2261814
11344

 1255 


1255 
1255 

1255 
1255 
2294
species
1111

 1254 
species


1254 

1254 
1254 
266
111

111
 187452 
222


187452 

187452 
187452 
species

111
 51663 
212

51663 


51663 
51663 
species

122113143129162144
order
172225932842300567382499
1385

1378
111
genus
111

 29391 
species


29391 
1
1

11
11


1785995 
1785995 
species
 1785995 

608583768348
family
186823
444554

29330
genus
13101212334
111111

 405212 
species

405212 
405212 
405212 
405212 
405212 
405212 
13101212334
111111

111221
1129704
genus
23431541

 33943 
23631

33943 

33943 
33943 
33943 
33943 
species
11111

 2055160 
species


2055160 

2055160 
2055160 
3491
111

454168494643
genus
432330
222222

111111

1214604 
1214604 
1214604 
1214604 
1214604 
1214604 
252847172227
species
 1214604 

111111
species
201321322416

1903704 
1903704 
1903704 
1903704 
1903704 
1903704 
 1903704 

152624282224
93115212391698348195
family
90964

112
genus
2263
69965

111
species


69966 
69966 

69966 
2262
 69966 

1


1855823 
species
 1855823 
1

1
2
genus
45669

2


407035 
species
 407035 
1

93114912371671347192
genus
1279
152423262122

 70255 
species


70255 
70255 
70255 
70255 
6141
1111

 1282 

1282 
1282 
1282 
1282 
1282 
1282 
123353113364716
species
111111

 170573 


170573 
170573 


170573 
122
species
111

species

29388 
29388 
29388 
29388 
29388 
29388 
758566090612612
 29388 
111111

11111
 29382 

29382 
29382 

29382 
29382 
29382 
21212
species


1280 
1280 
1280 
1280 
1280 
1280 
4212814125411796
species
 1280 
111111

 29384 
222

29384 
29384 

29384 
species
111


1286 
1286 
1286 
1286 
1286 
871424
species
 1286 
11111


70258 
70258 
21
species
 70258 
11

species


283734 
283734 
283734 
283734 
283734 
242112
 283734 
11111

 643214 
species
15


643214 
643214 
11

 45972 
5612722

45972 
45972 
45972 
45972 
45972 
45972 
species
111111

1
species


985762 
1
 985762 

11111
species
11127

308354 
308354 
308354 

308354 
308354 
 308354 

111111
species
39221385

1292 
1292 
1292 
1292 
1292 
1292 
 1292 

1111
species

985002 
985002 

985002 
985002 
1171
 985002 

species


28035 
28035 
28035 
28035 
28035 
10614710
 28035 
11111


214473 
214473 
214473 
214473 
2263
species
 214473 
1111

111111
species

1296 
1296 
1296 
1296 
1296 
1296 
111112
 1296 

 1715860 


1715860 
1
species
1

species
151721


246432 
246432 
246432 
246432 
246432 
 246432 
11111

1111
species
1111


1294 
1294 
1294 
1294 
 1294 


1288 
1288 
1288 

1288 
4111
species
 1288 
1111


1290 
1290 
1290 
1290 
1290 
1290 
424172939
species
 1290 
111111

 1283 
species

1283 
1283 
1283 
1283 
1283 
1283 
5162222910
111111

1111
2211


1295 

1295 
1295 
1295 
species
 1295 

 1281 
species

1281 

1281 

1281 
1281 
1321
1111

 29379 
131


29379 
29379 
29379 
species
111

species
2


1284 
 1284 
1

11
 61015 


61015 

61015 
13
species

 155085 
species


155085 
1
1

111111
 29385 
species

29385 
29385 
29385 
29385 
29385 
29385 
5731092

2005363
genus
111
111

 1849491 
111


1849491 

1849491 
1849491 
species
111

186822
family
4533172802975019520
373334324136

332931273632
44249
4163032542634973504
genus


159743 
159743 
159743 
11311
species
 159743 
111

 1536775 

1536775 

1536775 
1536775 
1536775 
1536775 
212316
species
11111

 1536773 
species

1536773 
1536773 
1536773 

1536773 
1536773 
422217
11111

11111
 324057 
species

324057 
324057 
324057 
324057 
324057 
322148

 1870819 
species
114392

1870819 

1870819 
1870819 
1870819 
1870819 
11111

111111
 1464 
species

1464 
1464 
1464 
1464 
1464 
1464 
721443447

111111
species

1126833 
1126833 
1126833 
1126833 
1126833 
1126833 
5259510
 1126833 

1695917
species group
2044880
11111

1695917

483937 
483937 

483937 
483937 
483937 
species
 483937 
11111


61624 
61624 
61624 
61624 
61624 
61624 
69134697812963
species
 61624 
111111

111111
 1870820 
425274

1870820 
1870820 
1870820 
1870820 
1870820 
1870820 
species

1111
22147


1536770 
1536770 
1536770 
1536770 
species
 1536770 

11111
 160799 
11383

160799 
160799 
160799 

160799 
160799 
species


59893 

59893 
18
species
 59893 
11

111111
species

1536774 
1536774 
1536774 
1536774 
1536774 
1536774 
12231964
 1536774 

 169760 
species

169760 
169760 
169760 
169760 
169760 
169760 
7151104
111111

111111
143351522

44251 
44251 
44251 
44251 
44251 
44251 
species
 44251 

species

172713 
172713 
172713 

172713 
172713 
21243
 172713 
11111

 528191 
species

528191 
528191 
528191 
528191 
528191 
528191 
204564083201
111111

3110367

1566358 
1566358 

1566358 
1566358 
1566358 
species
 1566358 
11111

11111
785126

365617 
365617 
365617 

365617 
365617 
species
 365617 

 1536769 

1536769 
1536769 
1536769 
1536769 
1536769 
1536769 
15251691
species
111111

11111
 1462996 
species
2112511

1462996 
1462996 
1462996 
1462996 
1462996 

111111

189425 
189425 
189425 
189425 
189425 
189425 
728183
species
 189425 

111111
39181793415

1616788 
1616788 
1616788 
1616788 
1616788 
1616788 
species
 1616788 

1111
species
5243

1178515 

1178515 

1178515 
1178515 
 1178515 

11111
111192

2069255 
2069255 

2069255 
2069255 
2069255 
species
 2069255 

111111
species
91121297

1536772 
1536772 
1536772 
1536772 
1536772 
1536772 
 1536772 

111111
 414771 
122836253

414771 
414771 
414771 
414771 
414771 
414771 
species

 1532905 
species

1532905 
1532905 
1532905 
1532905 
1532905 
1532905 
36317514
111111

111111
species

162209 
162209 
162209 
162209 
162209 
162209 
235244
 162209 

2311131

1536771 
1536771 
1536771 

1536771 
1536771 
species
 1536771 
11111

 1695218 
species

1695218 
1695218 
1695218 
1695218 
1695218 
1026499
11111

111
species
453

1763538 


1763538 
1763538 
 1763538 

 189426 
species

189426 
189426 
189426 
189426 
189426 
189426 
8244456
111111

 1406 
922658420550

1406 
1406 
1406 
1406 
1406 
1406 
species
111111

11111
72268

481743 
481743 
481743 

481743 
481743 
species
 481743 

genus
102117255
55080
221332

 54913 
6111103

54913 
54913 

54913 
54913 
54913 
species
11111

411372

1465 
1465 
1465 
1465 
1465 
1465 
species
 1465 
111111

38


1393 
1393 
species
 1393 
11

111111
55079
142351
genus

 1500254 
species
142351

1500254 
1500254 
1500254 
1500254 
1500254 
1500254 
111111

111111
76632
26823141610
genus

111111
 377615 
species
26823141610

377615 
377615 
377615 
377615 
377615 
377615 

97981511
family
934925488639
186818

815198
genus
648800
11122

11111
 76853 
species

76853 

76853 
76853 
76853 
76853 
815153

species


2048654 
2048654 
45
 2048654 
11

312231
1569
74392010
genus

11
species
11

1930546 


1930546 
 1930546 

111


1930764 
1930764 
1930764 
111
species
 1930764 

 1571 
species
54281810

1571 
1571 
1571 
1571 
1571 
1571 
111111

 1476 
1

1476 
species
1

1111
genus
173125
648802

species
173125

241244 

241244 

241244 
241244 
 241244 
1111

32182
genus
157226
111

111
 1508404 
32182

1508404 
1508404 


1508404 
species

345596
1372
genus
292618343514

 414778 


414778 
1
species
1

11
11


1302659 


1302659 
species
 1302659 

111111
species

1038856 
1038856 
1038856 
1038856 
1038856 
1038856 
25188414
 1038856 

111
species
221


161360 

161360 
161360 
 161360 

11
species
11


1215089 


1215089 
 1215089 

111111

200991 
200991 
200991 
200991 
200991 
200991 
25312205
species
 200991 

 1598147 
species

1598147 


1598147 
24
11

 1526927 
species
31


1526927 
1526927 
11

species


2213202 

2213202 
52
 2213202 
11

1
 192421 
2


192421 
species

1
2


1374 
species
 1374 

1111
species


2058136 
2058136 
2058136 
2058136 
24111
 2058136 

1649
genus
1
1

species
1


1750719 
 1750719 
1

666664
genus
226172116801716
33986

 132920 

132920 
132920 
132920 
132920 
132920 
132920 
29272012263
species
111111

 360911 
species
11334

360911 
360911 
360911 
360911 
360911 
11111

111111
 1224749 
271386141

1224749 
1224749 
1224749 
1224749 
1224749 
1224749 
species


1849031 
1849031 
1849031 
1849031 
1849031 
1849031 
121799161
species
 1849031 
111111

11111
212010516

1399115 
1399115 
1399115 
1399115 
1399115 
species
 1399115 

111111
 332410 
species
136946645951

332410 
332410 
332410 
332410 
332410 
332410 

7187549487317641412
family
186817
483359466557

324145
genus
75671620
400634

111
species
211


28031 
28031 


28031 
 28031 

species
111


1145276 

1145276 
1145276 
 1145276 
111

1132

2169540 

2169540 

2169540 
2169540 
species
 2169540 
1111

species
412

2072025 


2072025 
2072025 
 2072025 
111

 1421 
23371114

1421 
1421 
1421 
1421 
1421 
1421 
species
111111

111111
genus
221142
182709

111111
 182710 
species
221142

182710 
182710 
182710 
182710 
182710 
182710 

6267048876766491321
genus
1386
272034273831

1
species
1


1547283 
 1547283 

1
 1446792 
species


1446792 
1

species
158314

79880 
79880 
79880 
79880 

79880 
 79880 
11111

species
11


86664 


86664 
 86664 
11

 324767 

324767 

324767 

324767 
324767 
1231
species
1111

species


2009331 
3
 2009331 
1

545455
86661
674110575162162
species group

species
332732215

1405 
1405 
1405 
1405 
1405 
1405 
 1405 
111111

111111
 1396 
species
14518366126

1396 
1396 
1396 
1396 
1396 
1396 

111111
species
333033314190

1428 
1428 
1428 
1428 
1428 
1428 
 1428 

1111
species
9293

580165 

580165 

580165 
580165 
 580165 


1392 
1392 
1392 
1392 
1392 
1392 
832552928
species
 1392 
111111

 1574141 
species
1


1574141 
1

12


1827146 
1827146 
species
 1827146 
11

 1413 
8121185

1413 

1413 
1413 
1413 
1413 
species
11111

1111
 1478 

1478 

1478 
1478 
1478 
2415
species

1
 2093834 
1


2093834 
species

species


1127744 
12
 1127744 
1


666686 
666686 
11
species
 666686 
11

 1837130 


1837130 
1837130 
1837130 
111
species
111

 1398 
species
492510383990

1398 
1398 
1398 
1398 
1398 
1398 
111111

1
 1839798 
2


1839798 
species

111111
 1404 

1404 
1404 
1404 
1404 
1404 
1404 
282304547195194866
species

species
111

86665 

86665 

86665 
 86665 
111


658666 


658666 
81
species
 658666 
11

111111
21021924179

1408 
1408 
1408 
1408 
1408 
1408 
species
 1408 


35841 


35841 
12
species
 35841 
11

111
1792192
125
species group

 293387 


293387 
293387 

293387 
125
species
111

111
 1479 
141

1479 


1479 
1479 
species

32


756828 
756828 
species
 756828 
11

111
 33932 
131

33932 


33932 
33932 
species

1


412384 
6
species
 412384 

11
species


199441 

199441 
11
 199441 

1111
 1664069 


1664069 
1664069 
1664069 
1664069 
1631
species

1
 2026248 
4


2026248 
species

1
 563169 


563169 
1
species

111
species


79883 

79883 
79883 
128
 79883 

11


1892404 


1892404 
13
species
 1892404 

11
 1565991 
species


1565991 


1565991 
42

653685
species group
184218152287163155
767866

species
1

119858 
 119858 
1


1402 
1402 
1402 
1402 
1402 
1402 
51201072816
species
 1402 
111111

1111
11911


1452 
1452 
1452 
1452 
species
 1452 

1938374
species subgroup
6978551246584
332322

 1390 
species

1390 
1390 
1390 
1390 
1390 
1390 
16182491324
111111

species
611

659243 
659243 

659243 
 659243 
111

111111
species

492670 
492670 
492670 
492670 
492670 
492670 
4759311145260
 492670 

111111
 1423 
species

1423 
1423 
1423 
1423 
1423 
1423 
587871865244

11
 72361 
species


72361 
72361 
11

111111
species
54214501710

1648923 
1648923 
1648923 
1648923 
1648923 
1648923 
 1648923 

1
 1628753 
1


1628753 
species

 1471 
species
122

1471 

1471 

1471 
111

 632773 


632773 
632773 
31
species
11

11
 98228 
22

98228 


98228 
species

11


79885 

79885 
31
species
 79885 

1
 1178537 
species


1178537 
1

1111
species
23063


561879 
561879 
561879 
561879 
 561879 

species
1


300825 
 300825 
1

1
1


135735 
species
 135735 


1774743 
1
species
 1774743 
1

11

2052936 


2052936 
11
species
 2052936 

species


665099 

665099 
665099 
114
 665099 
111

1
1
genus
459532

1
species

586416 
1
 586416 

21143
84406
genus
31855

1
2


2017483 
species
 2017483 

111
 1482 

1482 


1482 
1482 
181
species

 163877 
113


163877 

163877 
163877 
species
111

11

1911587 


1911587 
21
species
 1911587 

1


1473 
1
species
 1473 

species
1


403957 
 403957 
1

1055323
2
genus
1

1
 33936 
species
2


33936 

212144
genus
31431310
150247

 198467 
species


198467 

198467 
37
11

111111
 33934 

33934 
33934 
33934 
33934 
33934 
33934 
211326
species

11


1490052 
1490052 
11
species
 1490052 

111
 1490057 

1490057 


1490057 
1490057 
132
species

1


294699 
1
species
 294699 

1906945
genus
21101
1111


1426 
1426 

1426 
1426 
21101
species
 1426 
1111

genus
123
200903
111

111
species


2213194 

2213194 
2213194 
123
 2213194 

111
351195
genus
152

111
 1230341 
152

1230341 

1230341 

1230341 
species

1
genus
3
175304

1
 1472767 
species
3


1472767 

11111
11611
genus
1329200

species
61


1221500 


1221500 
 1221500 
11

 255247 

255247 
255247 

255247 
111
species
111

97111198
733931315043
genus
129337

111

471223 


471223 
471223 
111
species
 471223 

111
species
112

1233873 

1233873 
1233873 
 1233873 

11
species


581103 
581103 
11
 581103 

443244
species group
56271193837
1505648

111111
species

169283 
169283 
169283 
169283 
169283 
169283 
12114528
 169283 

 1462 
species

1462 
1462 


1462 
1462 
15152
1111

 33941 

33941 
33941 
33941 
33941 
33941 
33941 
21285164
species
111111

11111
 33938 
species
2219223

33938 
33938 
33938 

33938 
33938 

 1422 
species
11711


1422 
1422 
1422 
1422 
1422 
11111

9106431

129338 
129338 
129338 
129338 
129338 
129338 
species
 129338 
111111

species
311


544556 
544556 
544556 
 544556 
111

 33940 

33940 

33940 
33940 
33940 
33940 
24163
species
11111

111
 691437 
species
113


691437 
691437 
691437 

 1921421 


1921421 
1921421 
32
species
11

11
species

550542 


550542 
41
 550542 

genus
14
29331
11

 1449 
14


1449 


1449 
species
11

11211
142102
genus
45667

 1570 
141102

1570 

1570 
1570 
1570 
1570 
species
11111

1
 402384 


402384 
1
species

111111
186824
214422
family

2442
genus
1677050
1111

1111
species
2442

1471761 

1471761 
1471761 
1471761 
 1471761 

292635
genus
12
11


2071623 


2071623 
12
species
 2071623 
11

186821
family
4534
11

 85683 


85683 
85683 
4534
species
11

225266
771811370264276
family
186820

1111
21512
genus
2755

 2756 
species
21512


2756 
2756 
2756 
2756 
1111

1637
genus
771811169259264
224155


1638 
1638 
1638 

1638 
1638 
2151328
species
 1638 
11111

 1643 


1643 
1643 
411
species
11

111
 1640 
135


1640 

1640 
1640 
species

 1642 


1642 

1642 
1642 
122
species
111

111111
 1639 
species

1639 
1639 
1639 
1639 
1639 
1639 
751710469237218

766066717170
186801
class
77774012118068001106

81099126
68295
order
302328462950

111
227387
family
141

141
genus
227388
111

 184064 
species
141


184064 
184064 
184064 
111

family
533641
543371
433341

291988
213
genus
111

 291990 
species
213

291990 

291990 
291990 
111

332241
332341
genus
44000

11
species
11


31899 

31899 
 31899 

 717609 


717609 

717609 
12
species
11

111
 413889 

413889 


413889 
413889 
111
species

11

52766 


52766 
species
 52766 
11

11
 44001 
species
11


44001 

44001 

 55205 
species
1111

55205 
55205 
55205 

55205 
1111

543372
family
4512
1111

1111
genus
4512
252965

 252966 
species
4512

252966 
252966 

252966 

252966 
1111

186814
family
211524352447
365474

genus
3
42837
1

1


42838 
3
species
 42838 

111111
44260
13714311528
genus

111111
13714311528

1525 
1525 
1525 
1525 
1525 
1525 
species
 1525 

1
2
genus
499228

1
 499229 


499229 
2
species

8792619
genus
1754
243253

species
1

399726 
 399726 
1

 496866 
11


496866 


496866 
species
11

1
species


108150 
1
 108150 

11
 29323 
21


29323 


29323 
species

111111
7271217

46354 
46354 
46354 
46354 
46354 
46354 
species
 46354 

11
 583357 


583357 
583357 
11
species

1


573062 
1
species
 573062 

1111
 2325 
species


2325 
2325 

2325 
2325 
2111

140458
genus
11
11

11
 85874 
11


85874 
85874 
species

1
485256
order
1

1
family
1
485255

375928
genus
1
1

1
species
1


375929 
 375929 

654955625861
order
74271511737607701052
186802

1111
family
32121
543347

178898
genus
32121
1111

 178899 

178899 
178899 

178899 

178899 
32121
species
1111

343233
family
914332616
186806

232122
1730
genus
810211610

 39488 
species

39488 
39488 
39488 
39488 
39488 
39488 
741124
111111

species


39485 
1
 39485 
1

 1736 
species

1736 
1736 
1736 

1736 
1736 
151146
11111

111111
33951
1412106
genus

111111
 33952 

33952 
33952 
33952 
33952 
33952 
33952 
1412106
species

11221
family
12441
543314

111
113

143393 

143393 
143393 
species
 143393 

2311
genus
86331
1111

species
2311


114527 
114527 
114527 

114527 
 114527 
1111

111111
990719
164276740276185136
family

990721
genus
164276740276185136
111111

164276740276185136

1805714 
1805714 
1805714 
1805714 
1805714 
1805714 
species
 1805714 
111111

111
1392389
genus
235

 1297617 


1297617 
1297617 
1297617 
235
species
111

26353113
family
216572
111111

genus
26353113
459786
111111


351091 
351091 
351091 
351091 
351091 
351091 
26353113
species
 351091 
111111

1068899
541000
632931404732
family

211
711
genus
1263

11

1160721 

1160721 
41
species
 1160721 

31

1264 


1264 
species
 1264 
11

1637257
genus
1
1

1
species


884684 
1
 884684 

 1572656 
species
11151


1572656 
1572656 
1572656 
1572656 
1572656 
11111

634555
471922152321
genus
1508657

11111
 29343 

29343 
29343 

29343 
29343 
29343 
24111
species

11111
53611

1834198 
1834198 
1834198 

1834198 
1834198 
species
 1834198 

 1521 
species
13411

1521 

1521 
1521 
1521 
1521 
11111

1111
 84032 

84032 

84032 
84032 
84032 
1112
species

species

1515 
1515 
1515 
1515 
1515 
1515 
26121281810
 1515 
111111

 1510 

1510 


1510 

1510 
1218
species
111

3531887
genus
216851
111111

 853 

853 
853 
853 
853 
853 
853 
3531887
species
111111

253238
genus
6446102
111111

species
6446102

253239 
253239 
253239 
253239 
253239 
253239 
 253239 
111111

12121212910
family
526241464562
186803

841
1121
genus
1111

1111
 301301 
species


301301 

301301 
301301 
301301 
1121

11111
 712991 
species
11141

712991 

712991 
712991 
712991 
712991 

572511
252510151316
genus
232212

111
species

1912897 
1912897 


1912897 
211
 1912897 

111
 1322 
species


1322 
1322 
1322 
128

111111
 1796616 
species
2323871315

1796616 
1796616 
1796616 
1796616 
1796616 
1796616 

2039240
genus
2
1

1
species


28446 
2
 28446 

1
genus
830
1


185008 
1
species
 185008 
1

11111
genus
31312
698776

11111
 29360 
species

29360 
29360 
29360 
29360 

29360 
31312

111111
207244
31569619
genus

 649756 

649756 
649756 
649756 
649756 
649756 
649756 
31569619
species
111111

genus
1
1663717
1

 1679721 
species

1679721 
1
1

554554
genus
161616182023
1506553

species
21111

1871021 
1871021 
1871021 
1871021 
1871021 
 1871021 
11111

11111
species
52238

66219 
66219 

66219 
66219 
66219 
 66219 

111111

84030 
84030 
84030 
84030 
84030 
84030 
5101010410
species
 84030 

111111
 208479 

208479 
208479 
208479 
208479 
208479 
208479 
223412
species

111111
species
2121113

1834196 
1834196 
1834196 
1834196 
1834196 
1834196 
 1834196 

1111

39491 
39491 
39491 
39491 
3421
species
 39491 

111111
543349
family
541119164

111111
2733
genus
541119164

 2734 
species

2734 
2734 
2734 
2734 
2734 
2734 
541119164
111111

201015152120
family
297283304224352693
31979

49082
genus
113
111


1508644 
1508644 
13
species
 1508644 
11

 49118 

49118 
1
species
1

16813101615
1485
genus
285275297199333679

 29341 
species
21


29341 

29341 
11


1491 
1491 
1491 
1491 
1491 
1491 
221244223144245547
species
 1491 
111111

11
 46867 
species
12

46867 


46867 

1
 84023 
2


84023 
species

1111
species

217159 
217159 
217159 

217159 
1121
 217159 

111111
 1502 
11221161918

1502 
1502 
1502 
1502 
1502 
1502 
species

111111
 169679 
5131513

169679 
169679 
169679 
169679 
169679 
169679 
species


1548 
1548 
251
species
 1548 
11

 1493 
114


1493 

1493 
1493 
species
111

species


1561 

1561 
1561 
141
 1561 
111


1520 
1520 
1520 

1520 
1520 
12321
species
 1520 
11111

1
 332101 
species


332101 
1

11
 238834 
12

238834 

238834 
species

1
 1494 
species


1494 
1

 1542 
1


1542 
species
1

1
species
2

1538 
 1538 

11111
 755731 
13112

755731 
755731 

755731 
755731 
755731 
species

 1042156 
16111

1042156 
1042156 
1042156 
1042156 

1042156 
species
11111

1
 1216932 
species

1216932 
1

species

1534 


1534 
12
 1534 
11

 84022 


84022 
2
species
1

1
species


394958 
3
 394958 

11
 1492 
species
23

1492 


1492 


1488 

1488 
161
species
 1488 
11

11111
14181

1497 

1497 
1497 
1497 
1497 
species
 1497 

1


36745 
species
 36745 
1

1
species


1509 
6
 1509 


1501 
1501 
1501 
1501 
1501 
1501 
19163353582
species
 1501 
111111

 2082193 
1013


2082193 
2082193 
2082193 
species
111

11111
genus
2610125
390805

11111
 1424294 
2610125

1424294 

1424294 
1424294 
1424294 
1424294 
species

genus
87213
1981033
11111

species
87213

2086584 
2086584 

2086584 
2086584 
2086584 
 2086584 
11111

111112
111223
genus
114627

1
 208226 
species


208226 
2

111111
species

461876 
461876 
461876 
461876 
461876 
461876 
111221
 461876 

111111
539000
544191628
family

111111
73918
544191628
genus

111111
 73919 
544191628

73919 
73919 
73919 
73919 
73919 
73919 
species

68298
family
1
1

genus
1
862
1

 863 
species
1

863 
1

471726159
genus
946234
111111

111111
471726159

292800 
292800 
292800 
292800 
292800 
292800 
species
 292800 

313232
26114681316
family
186804

11
44259
22
genus

11
22


143361 
143361 
species
 143361 

1
1849828
genus
1

 1505 
species


1505 
1
1

 2081703 

2081703 

2081703 
71
species
11

112
genus
1481960
111

111
species
112

1731 


1731 
1731 
 1731 

1811267914
genus
1870884
111111


1496 
1496 
1496 
1496 
1496 
1496 
1811267914
species
 1496 
111111

111111
family
541736
31984

111111
2697
genus
541736

111111

35701 
35701 
35701 
35701 
35701 
35701 
541736
species
 35701 

381411303645
family
186807
8851369

genus
151
278993
111

111

863643 

863643 


863643 
151
species
 863643 

34671
genus
36853
11111

11111
 49338 
34671

49338 
49338 

49338 
49338 
49338 
species

genus
22
2282740
11

 102134 


102134 

102134 
22
species
11

51514
genus
11
11

11


51515 

51515 
species
 51515 
11

111
51196
genus
113

111
 51197 
species
113


51197 

51197 

51197 

79206
genus
274152535
221233

11111
 1563 
species
2614618

1563 
1563 

1563 
1563 
1563 

1111

79209 
79209 


79209 
79209 
13189
species
 79209 

1111
 885581 
1118


885581 
885581 
885581 
885581 
species

1562
genus
23544
12322

111
 1833852 
species
331


1833852 
1833852 
1833852 

111
223

1564 

1564 


1564 
species
 1564 


1565 
1565 
1565 
111
species
 1565 
111

 59610 
species
1


59610 
1

1111
genus
2282742
1111

1111
 58138 
1111

58138 
58138 

58138 

58138 
species

1111
471826
genus
1121


471827 
471827 
471827 
471827 
1121
species
 471827 
1111

12
genus
38
56112

11
 1147129 
species

1147129 


1147129 
31

 1131462 
7


1131462 
species
1

521013
order
53433
31212

191
family
972
111

32636
genus
1
1

1
 31909 

31909 
1
species

91
genus
2330
11

1
 656519 
9


656519 
species

1
 2331 
1


2331 
species

21111
53434
42112
family

111
112
genus
42417

species

42422 


42422 
42422 
112
 42422 
111

111
28186
321
genus

111
 28187 
321

28187 
28187 
28187 
species

1676648
154965
class
111111

1676649
order
154965
111111

111111
1676650
154965
family

111111
154965
genus
1676651

111111
 1555112 
154965

1555112 
1555112 
1555112 
1555112 
1555112 
1555112 
species

111
phylum
1731
1134404

1731
class
795747
111

795748
order
1731
111

111
1731
family
795749

111
genus
1731
795750

111
1731


591197 
591197 
591197 
species
 591197 

200783
phylum
3531152
341122

187857
class
3531152
341122

141111
32069
153191
order

3311
family
224027
3111

1
genus
1
182899

species
1


309805 
 309805 
1

212790
2311
genus
2111

1111
1311


436114 
436114 
436114 

436114 
species
 436114 

1
1


309806 
species
 309806 

111
129
family
64898

111
genus
129
939

111
 940 
129

940 
940 


940 
species

261
order
1485951
211

211
family
261
558314

64159
1
genus
1

1
species
1

64160 
 64160 

111
171868
161
genus

species

228745 


228745 
228745 
161
 228745 
111

phylum
8134851138182968165456530
976
168114134165189154

894758789377
117743
73322417671944143716011
class

order
73322417671944143716011
200644
894758789377

222241
39782
92316114
family

222241
34098
genus
92316114


624186 
1
species
 624186 
1

 367806 
2


367806 
species
1

17


1653831 


1653831 
species
 1653831 
11

 164514 
712144

164514 
164514 
164514 
164514 

164514 
species
11111

species

1316444 


1316444 
1316444 
221
 1316444 
111

 1186051 


1186051 
1
species
1

11
1853230
family
241

11
genus
241
332102

species
241


191579 

191579 
 191579 
11

111111
genus
533731193851
336809

111111
 336810 
species

336810 
336810 
336810 
336810 
336810 
336810 
533731193851

1940138
11
genus
11

11

1415657 


1415657 
11
species
 1415657 

246874
family
19
11

267986
genus
19
11


253245 
253245 
19
species
 253245 
11

49546
family
72692027321876143225954
854454738873

1111
417127
genus
4133

species
4133

398743 
398743 

398743 

398743 
 398743 
1111

genus
181427275
104267
412233

species
112262

669041 
669041 
669041 
669041 

669041 
 669041 
11111

species
511

584609 


584609 
584609 
 584609 
111

 1850252 
species
11252

1850252 


1850252 
1850252 
111

 107401 

107401 

107401 
107401 
107401 
1211
species
1111

61311
genus
1209327
1111

1111
61311

1803846 
1803846 

1803846 
1803846 
species
 1803846 

311221
104264
genus
101169144

1111
species
54194

979 


979 
979 
979 
 979 

11
species
31

59600 
59600 
 59600 

 76594 
21285

76594 

76594 
76594 
76594 
species
1111

71
genus
1204360
11

species

762954 
762954 
71
 762954 
11

2131
182161
genus
326319

 313590 
12

313590 
species
1

 983548 
species


983548 
983548 
211
11

611

2173169 


2173169 
2173169 
species
 2173169 
111

species
4


326320 
 326320 
1

11
genus
76
143222

species
76


1729720 
1729720 
 1729720 
11

412343
2123391612
genus
52959

812813

1774273 

1774273 
1774273 
1774273 
1774273 
species
 1774273 
11111

111
121

996801 
996801 


996801 
species
 996801 

111
587

1529069 


1529069 
1529069 
species
 1529069 

 2058137 
species


2058137 
2058137 
46
11

1111
 313598 
species

313598 

313598 
313598 

313598 
7272

312342
genus
91218429
292691

 411153 
species
11127

411153 

411153 
411153 
411153 
1111

111


2126553 

2126553 
2126553 
143
species
 2126553 


1913577 


1913577 
1913577 
1913577 
22245
species
 1913577 
1111

species
6184

1486245 

1486245 

1486245 
1486245 
 1486245 
1111

4
genus
1247519
1

1
 1383885 
4


1383885 
species

111
393005
genus
21010

111
 2069432 

2069432 


2069432 
2069432 
21010
species

1111
336276
11713
genus

 2058135 
species
11713


2058135 

2058135 
2058135 
2058135 
1111

286104
genus
51415
1111

 1936080 


1936080 
14
species
1

111
 754409 
515

754409 


754409 
754409 
species

321422
76831
genus
65668139

 1583100 
species
111

1583100 
1583100 

1583100 
111

111
 480520 

480520 


480520 

480520 
4162
species

11


76832 
76832 
219
species
 76832 

111111
 1458492 
species
1463047

1458492 
1458492 
1458492 
1458492 
1458492 
1458492 

genus
183
216431
111

species
183

313588 


313588 

313588 
 313588 
111

16322202
genus
252356
32242

1111
species
1175

1836467 

1836467 
1836467 
1836467 
 1836467 

111
 1178778 
species

1178778 


1178778 
1178778 
7131

 1644130 
821511

1644130 

1644130 
1644130 
1644130 
1644130 
species
11111

 313603 
1


313603 
species
1

225842
genus
711627
111211


1336795 


1336795 
71
species
 1336795 
11


1336794 
1336794 
1336794 
1336794 
1336794 
11527
species
 1336794 
11111

1
261827
1
genus

1
species
1


1736674 
 1736674 

112040
genus
6681
1111


63186 


63186 
63186 
63186 
6681
species
 63186 
1111

species

1150389 

1150389 
1150389 
1150389 
2151
 1150389 
1111

492718267284103363012
genus
59732
989999

111111
 536441 
species

536441 
536441 
536441 
536441 
536441 
536441 
5721410840

111111
 1721091 
53013421637269

1721091 
1721091 
1721091 
1721091 
1721091 
1721091 
species

111111
 878220 
species
3633182851257

878220 
878220 
878220 
878220 
878220 
878220 


1685010 
1685010 
1685010 
1685010 
1685010 
1685010 
866350261314578
species
 1685010 
111111

111111
2061881037137

1265445 
1265445 
1265445 
1265445 
1265445 
1265445 
species
 1265445 

 558152 
species
4393285725261

558152 

558152 
558152 
558152 
558152 
11111

111111
 1324352 

1324352 
1324352 
1324352 
1324352 
1324352 
1324352 
32311646691156
species

 253 
species
1645480773291988

253 
253 
253 
253 
253 
253 
111111

111111
 2015076 

2015076 
2015076 
2015076 
2015076 
2015076 
2015076 
49832834682326
species

1479141413
237
3025042478394117
genus

 2162713 
species
6611

2162713 


2162713 
2162713 
111

species
51111028332

96345 
96345 
96345 
96345 
96345 
96345 
 96345 
111111

11111
 55197 
212182

55197 
55197 

55197 
55197 
55197 
species


2201181 

2201181 
2201181 
2201181 
11115
species
 2201181 
1111

 1981981 
species
856432345

1981981 
1981981 
1981981 
1981981 
1981981 
1981981 
111111

11111

1306519 

1306519 
1306519 
1306519 
1306519 
5516146
species
 1306519 

 1763534 

1763534 
1763534 

1763534 

1763534 
16118
species
1111

11111
 2183896 
species

2183896 

2183896 
2183896 
2183896 
2183896 
213094

species


2175091 

2175091 
2175091 
2175091 
117176
 2175091 
1111

species

312277 


312277 
312277 
312277 
54114
 312277 
1111

1111
species
1711253

1492737 


1492737 
1492737 
1492737 
 1492737 

 986 
species

986 
986 
986 
986 
986 
986 
302181588220
111111

1111
42293

2172098 

2172098 

2172098 
2172098 
species
 2172098 

11111
species
141151

1355330 

1355330 
1355330 
1355330 
1355330 
 1355330 

 996 
species

996 
996 
996 
996 
996 
996 
271951025123
111111

711
genus
178469
111

 616991 

616991 


616991 
616991 
711
species
111

308865
genus
131812833902550942
535555

111111
species
301224624

1756149 
1756149 
1756149 
1756149 
1756149 
1756149 
 1756149 

11111
 172045 
species
724910047

172045 

172045 
172045 
172045 
172045 


238 
238 
238 
238 
238 
238 
1412135224593
species
 238 
111111

11111
species

1756150 

1756150 
1756150 
1756150 
1756150 
581164049
 1756150 

111111

1117645 
1117645 
1117645 
1117645 
1117645 
1117645 
10179633112119729
species
 1117645 

1111
1518147
3349
genus

1111
species

1790137 

1790137 

1790137 
1790137 
3349
 1790137 

11111
genus
1736214
1649495

11111
 1936081 
species

1936081 
1936081 
1936081 
1936081 

1936081 
1736214

1111
83612
61563
genus

1111
 57029 
species

57029 


57029 
57029 
57029 
61563

28250
genus
374321057591245
111111

 28251 
374321057591245

28251 
28251 
28251 
28251 
28251 
28251 
species
111111

 531844 
species
29268127

531844 
531844 
531844 

531844 
531844 
11111

1111
genus
1142
153265

1111
 101385 
species

101385 

101385 

101385 
101385 
1142

111111
1013
1415758916
genus

 1014 
1415758916

1014 
1014 
1014 
1014 
1014 
1014 
species
111111

21112
612448
genus
358023

1111
species

1622118 

1622118 

1622118 
1622118 
2146
 1622118 

 1850246 
4242

1850246 


1850246 

1850246 
species
111

11
252306
genus
12

 252307 
12

252307 
252307 
species
11

291183
142120117
genus
211222


983544 

983544 


983544 
1211
species
 983544 
111

11
 1486034 


1486034 
1486034 
82
species

11111
species

2057808 
2057808 

2057808 
2057808 
2057808 
221296
 2057808 

111111
34084
355266863609446
genus

 34085 
355266863609446

34085 
34085 
34085 
34085 
34085 
34085 
species
111111

389486
27
genus
11

11
 1453352 

1453352 


1453352 
27
species

genus
771414616138
1016
8886127

 1945657 
species


1945657 


1945657 
1945657 
1101
111

 1316593 
species
13


1316593 

1316593 
11


1018 

1018 
1018 
1018 
5131
species
 1018 
1111

11111
2436413


1019 
1019 
1019 
1019 
1019 
species
 1019 

1111
 45243 

45243 

45243 

45243 
45243 
6252
species

1111

1945658 

1945658 

1945658 
1945658 
1252
species
 1945658 


28188 
28188 

28188 
28188 
28188 
311625
species
 28188 
11111

11
species
22


209053 


209053 
 209053 

 1848904 
species


1848904 
1848904 
28
11

 1017 
51118

1017 
1017 
1017 
1017 
1017 
species
11111

 327575 
species

327575 
327575 
327575 

327575 
3314
1111

11111
species
432114

1316596 
1316596 
1316596 

1316596 
1316596 
 1316596 

1111
species
501111

28189 

28189 

28189 
28189 
 28189 

363408
15136156
genus
31244

11


328515 
328515 
22
species
 328515 

11111

2058134 

2058134 
2058134 
2058134 
2058134 
512092
species
 2058134 

111
species
731

319236 


319236 
319236 
 319236 

1111
 1476901 
species

1476901 


1476901 
1476901 
1476901 
31611

111500
1111
genus
1111

1111


516051 
516051 
516051 
516051 
1111
species
 516051 

order
8089971009357
1100069
444444

444444
563843
family
8089971009357

genus
463045444737
29548
111111

species
463045444737

29549 
29549 
29549 
29549 
29549 
29549 
 29549 
111111

 2026787 
9221321209

2026787 
2026787 
2026787 
2026787 
2026787 
2026787 
species
111111

111111

1779382 
1779382 
1779382 
1779382 
1779382 
1779382 
8292973
species
 1779382 

146918
genus
1783726198
111111

111111
 146919 
species
1783726198

146919 
146919 
146919 
146919 
146919 
146919 

196158115288227132
class
200643
242324243118

111242
1970189
21125122
order

1573805
2411
family
111

111
 1717717 


1717717 
1717717 
1717717 
2411
species

4
family
558415
1

1
1193324
4
genus

1
 889453 
species


889453 
4

1970190
family
2111
1111

1111
genus
2111
1970191

species

1307839 
1307839 

1307839 
1307839 
2111
 1307839 
1111

family
161
1471398
111

genus
161
1471399
111

111
species
161


1168034 

1168034 
1168034 
 1168034 

232223222716
order
194157114263215130
171549

171550
1726414
family
112111

11111
239759
genus
172544

 214856 
species
172544

214856 
214856 
214856 
214856 

214856 
11111

11
1611681
genus
11

 1433126 
species
11


1433126 

1433126 
11

171551
family
763632766553
523251

322131
genus
673630706353
836


393921 
393921 


393921 
241
species
 393921 
111

111
 28123 
species
121

28123 

28123 

28123 

111111
species
643228706153

837 
837 
837 
837 
837 
837 
 837 

111
161
genus
307628

111
species

1642646 


1642646 
1642646 
161
 1642646 

111
genus
821
1784836

111

1562970 

1562970 

1562970 
821
species
 1562970 

675775
815
598122676735
family

genus
598122676735
816
675775

111111
 246787 
524111

246787 
246787 
246787 
246787 
246787 
246787 
species

111111
species

817 
817 
817 
817 
817 
817 
485515301430
 817 

 47678 

47678 
47678 

47678 
126
species
111

11
15


357276 


357276 
species
 357276 

1
 28119 
species


28119 
3


28116 
28116 
2230
species
 28116 
11

 290053 
1


290053 
species
1

111
species

818 
818 


818 
117
 818 

351

1796613 
1796613 


1796613 
species
 1796613 
111

1
 376805 
species
1


376805 

111
species
141


28113 
28113 
28113 
 28113 

 821 
species

821 
821 
821 
821 
821 
821 
1151192
111111

11
2005520
12
family

12
genus
294702
11

11
12


1642647 
1642647 
species
 1642647 

23221
family
2005473
11111

11111
genus
23221
1918540

species

1796646 

1796646 
1796646 
1796646 
1796646 
23221
 1796646 
11111

family
2313129
1853231
11111

283168
genus
2313129
11111

11111

28118 

28118 
28118 
28118 
28118 
2313129
species
 28118 

6211
genus
511434
1111

1111

511435 

511435 
511435 
511435 
6211
species
 511435 

2005519
141
family
111

141
genus
397864
111

111
 397865 

397865 
397865 
397865 
141
species

11
species
31


1400053 
1400053 
 1400053 

332221
family
911142071
2005525

58142071
genus
195950
222221

 28112 

28112 
28112 
28112 
28112 
28112 
28112 
3421461
species
111111

11111
 712710 
species
241261

712710 
712710 
712710 
712710 
712710 

43
genus
375288
11

43

823 
823 
species
 823 
11

171552
family
222130795624
476674

476674
genus
222130795624
838

111
 76123 
151


76123 

76123 
76123 
species

11111
 28132 
species

28132 
28132 
28132 
28132 

28132 
288241

 839 
species
432

839 

839 

839 
111

1
species
12


1177574 
 1177574 

13353163

28131 
28131 
28131 
28131 
28131 
28131 
species
 28131 
111111

 52227 
species
34412819

52227 
52227 
52227 
52227 
52227 
52227 
111111

 28129 
species


28129 
28129 
28129 
28129 
28129 
293241
11111

1111
species


589436 
589436 
589436 
589436 
2143
 589436 

1
1


652716 
species
 652716 

211
family
2005523
111

genus
211
346096
111

211


185300 
185300 
185300 
species
 185300 
111

1937959
class
542122010
121222

order
542122010
1936988
121222

1111
89374
family
28107

28107
genus
1007
1111

1111
 1008 
species


1008 

1008 
1008 
1008 
28107

111111
5224103
family
1937961

2349
5224103
genus
111111

5224103

2350 
2350 
2350 
2350 
2350 
2350 
species
 2350 
111111

class
264267188416352232
768503
271927353632

271927353632
768507
order
264267188416352232

11296
family
1937968
1111

1111
1937972
11296
genus

1111
 999 
species
11296


999 
999 
999 
999 

74711128
89373
family
304221106241

genus
22712
978
1111

1111
species

985 


985 
985 
985 
22712
 985 

32102
genus
861914
1111

 1834519 

1834519 


1834519 
1834519 
1834519 
32102
species
1111

genus
212
319458
111

111
species
212

316068 


316068 
316068 
 316068 

genus
82
2173039
11

11
 1784714 


1784714 
1784714 
82
species

1664383
23421
genus
1111


2183547 
2183547 
2183547 
2183547 
23421
species
 2183547 
1111

151923
genus
105
1111

1111
species
151923

106 
106 


106 
106 
 106 

11111
genus
12337
120831

11111
12337


94254 
94254 
94254 
94254 
94254 
species
 94254 

325553
821835336
genus
107

 1211326 

1211326 
1211326 
1211326 
1211326 
1211326 
1211326 
314623
species
111111

species
14213


2057025 
2057025 
2057025 
2057025 
 2057025 
1111

species
152142

1178516 

1178516 
1178516 
1178516 
1178516 
 1178516 
11111

species
13101


1379870 
1379870 
1379870 
1379870 
 1379870 
1111

1111
species
4434

564064 

564064 
564064 
564064 
 564064 

200667
family
332471212
211333

869806
22015
genus
1111

1111
species
22015

1006 


1006 
1006 
1006 
 1006 

59739
genus
12116
11111

 1191459 
species
12116

1191459 

1191459 
1191459 
1191459 
1191459 
11111

1111
 1257021 
species
326101


1257021 

1257021 
1257021 
1257021 

1853232
family
208255157179242160
121113121313

888888
89966
genus
190246123132213150

species

1484116 
1484116 
1484116 
1484116 
1484116 
1484116 
408546368034
 1484116 
111111

 1850093 

1850093 
1850093 
1850093 
1850093 
1850093 
1850093 
31321674836
species
111111


1411621 
1411621 
1411621 
1411621 
1411621 
1411621 
21295122914
species
 1411621 
111111

 1446467 
species
1068696

1446467 
1446467 
1446467 
1446467 
1446467 
1446467 
111111

 1484118 
species
58302517105

1484118 
1484118 
1484118 
1484118 
1484118 
1484118 
111111

111111
 1356852 
13816251636

1356852 
1356852 
1356852 
1356852 
1356852 
1356852 
species

 1385663 

1385663 
1385663 
1385663 
1385663 
1385663 
1385663 
294286
species
111111

 1385664 
15473271313

1385664 
1385664 
1385664 
1385664 
1385664 
1385664 
species
111111

212122
323449
63916123
genus

11111
species

388950 
388950 
388950 

388950 
388950 
43181
 388950 

 400092 
species
281642

400092 

400092 
400092 
400092 
400092 
11111

genus
1262531177
1379908
223333


1379910 
1379910 
1379910 
1379910 
1379910 
1379910 
65146112
species
 1379910 
111111

111111
 512763 
species

512763 
512763 
512763 
512763 
512763 
512763 
6182444

species


1379909 
1379909 
1379909 
1379909 
3121
 1379909 
1111

111
1853234
family
1311

59740
genus
1311
111


1085624 


1085624 
1085624 
1311
species
 1085624 
111

413644
family
914622310
563798

1111
246875
genus
12121


1727163 

1727163 
1727163 
1727163 
12121
species
 1727163 
1111

68288
genus
31047
1212

 104 
species
383

104 


104 

104 
111


320787 
320787 
320787 
244
species
 320787 
111

390846
51239172
genus
212211

111111
 1795355 
11122172

1795355 
1795355 
1795355 
1795355 
1795355 
1795355 
species

111

390884 

390884 
390884 
4117
species
 390884 

111
111
genus
232244

species
111


232259 
232259 
232259 
 232259 
111

122222
1501348
family
142632

273135
41532
genus
21122

1111
 247481 
species
3521


247481 

247481 
247481 
247481 

 249402 
1111


249402 
249402 

249402 
249402 
species
1111

genus
111
281119
111

111
species
111

281120 

281120 
281120 
 281120 

151513161615
2377919580140142968
class
117747

151513161615
200666
order
2377919580140142968

151513161615
2377919580140142968
family
84566

84567
genus
163393478742122620
554555

species

363852 
363852 
363852 
363852 
363852 
363852 
1625879432
 363852 
111111

species

188932 
188932 
188932 
188932 
188932 
188932 
882620734439725
 188932 
111111

111111

1727164 
1727164 
1727164 
1727164 
1727164 
1727164 
295625021187
species
 1727164 

 984 

984 
984 

984 
984 
984 
72667372
species
11111

111111
23431251564

430522 
430522 
430522 
430522 
430522 
430522 
species
 430522 

545555
28453
45241484747027
genus

111111
 743722 
species
72129181

743722 
743722 
743722 
743722 
743722 
743722 

11111
 1010 
545251

1010 

1010 
1010 
1010 
1010 
species

111111
 2003121 
species

2003121 
2003121 
2003121 
2003121 
2003121 
2003121 
1676188139

111111
 1538644 
961341622410

1538644 
1538644 
1538644 
1538644 
1538644 
1538644 
species

111111
species

1933220 
1933220 
1933220 
1933220 
1933220 
1933220 
89343106
 1933220 

423349
5101225710510
genus
233333

479147964

1300914 
1300914 
1300914 
1300914 
1300914 
1300914 
species
 1300914 
111111

 1550579 
species
215365


1550579 
1550579 
1550579 
1550579 
1550579 
11111

species

1234841 
1234841 
1234841 
1234841 
1234841 
1234841 
1125731
 1234841 
111111

11111
1649482
genus
4111831

4111831

151895 
151895 

151895 
151895 
151895 
species
 151895 
11111

11111
 1986952 
1724201810

1986952 
1986952 

1986952 
1986952 
1986952 
species

3311297
genus
929509
11111

11111
species
3311297

995 
995 
995 
995 
995 
 995 

847676
class
201317685320
1853228

1853229
201317685320
order
847676

563835
family
201317685320
847676

111111
354354
58134329
genus

 354356 
58134329

354356 
354356 
354356 
354356 
354356 
354356 
species
111111

genus
12
398041
11

11
12

1492898 


1492898 
species
 1492898 

genus
5461143
379899
222122

111111

1176587 
1176587 
1176587 
1176587 
1176587 
1176587 
4141132
species
 1176587 

 446683 

446683 
446683 
446683 

446683 
446683 
13211
species
11111

6123032
genus
79328
212212


2029983 

2029983 
2029983 

2029983 
41291
species
 2029983 
1111

111111
 79329 

79329 
79329 
79329 
79329 
79329 
79329 
211131
species

1121
genus
649460
1111

1111
 477680 

477680 

477680 
477680 
477680 
1121
species

27116
genus
1769012
11111

species

1850526 

1850526 
1850526 
1850526 
1850526 
27116
 1850526 
11111

12513811117720589
phylum
57723
999998

204432
class
751016711016462
666665

204433
order
751016711016462
666665

204434
family
751016711016462
666665

222222
genus
293117426526
392733

111111

870903 
870903 
870903 
870903 
870903 
870903 
9176153916
species
 870903 

111111
 392734 

392734 
392734 
392734 
392734 
392734 
392734 
201411272610
species

222222
332635365616
genus
940557

271827224112

940615 
940615 
940615 
940615 
940615 
940615 
species
 940615 
111111

 940614 
68814154

940614 
940614 
940614 
940614 
940614 
940614 
species
111111

11111
33973
1036511
genus

11111
 33075 
species

33075 
33075 
33075 
33075 
33075 
1036511

658061
3419273220
genus
111111

111111
3419273220

658062 
658062 
658062 
658062 
658062 
658062 
species
 658062 

111111
class
951242167
332159

332160
951242167
order
111111

111111
332161
family
951242167

111111
951242167
genus
332162

 332163 
species

332163 
332163 
332163 
332163 
332163 
332163 
951242167
111111

class
312541
1562566
111111

111111
genus
312541
458032

 458033 
species

458033 
458033 
458033 
458033 
458033 
458033 
312541
111111

1813735
383130202119
class
111111

111111
2211325
family
383130202119

111111
2004797
genus
383130202119

383130202119

1855912 
1855912 
1855912 
1855912 
1855912 
1855912 
species
 1855912 
111111

508458
31724726
phylum
113222

31724726
class
649775
113222

113222
order
31724726
649776

649777
31724726
family
113222

1111
508459
14312
genus

14312


1197717 
1197717 
1197717 
1197717 
species
 1197717 
1111

336260
1
genus
1

 336261 
1


336261 
species
1

81461
3179414
genus
111111

 81462 
3179414

81462 
81462 
81462 
81462 
81462 
81462 
species
111111

67819
phylum
16810955
111111

111111
1663419
class
16810955

111111
16810955
order
1663425

111111
1663426
16810955
family

genus
16810955
1005038
111111

 1005039 
16810955

1005039 
1005039 
1005039 
1005039 
1005039 
1005039 
species
111111

4204605763733181010
phylum
544448
303038263145

31969
class
4194605763733171010
293038263045

222228182526
399437536358296899
order
2085

222228182526
399437536358296899
family
2092

202027172324
2093
genus
396434534357292894

1111
species
4331

2115 
2115 

2115 
2115 
 2115 

 2098 
1101

2098 

2098 

2098 
species
111

11111
 29501 
species
22221

29501 
29501 
29501 

29501 
29501 

 29555 
1


29555 
species
1

species

171279 
171279 
171279 
171279 
171279 
171279 
3953898335161
 171279 
111111

1
 136241 
1


136241 
species

111111
 171632 

171632 
171632 
171632 
171632 
171632 
171632 
29929827
species

111111
species
724153

57372 
57372 
57372 
57372 
57372 
57372 
 57372 

11
 29554 
species
11

29554 


29554 

 28903 
435335533854

28903 
28903 
28903 
28903 
28903 
28903 
species
111111

656088
species group
2626177813
223132

11
species
11


2105 

2105 
 2105 

11111
species

2095 
2095 
2095 

2095 
2095 
21337
 2095 

 2102 
species
242513746

2102 
2102 
2102 
2102 
2102 
2102 
111111

species
31722

2113 
2113 
2113 
2113 

2113 
 2113 
11111

 2097 


2097 
2097 


2097 
111
species
111


2112 
2112 
13
species
 2112 
11

 2104 
species
12642

2104 

2104 
2104 
2104 
2104 
11111

species
11


2110 

2110 
 2110 
11

1111
 2109 
species
1111


2109 
2109 
2109 
2109 

111
 171284 
212

171284 


171284 
171284 
species

 984991 
species


984991 
1
1

13143142235

2100 
2100 
2100 
2100 
2100 
2100 
species
 2100 
111111

 92401 
species
1


92401 
1

species
21


2111 


2111 
 2111 
11

1


2123 
2
species
 2123 

111111
 2099 

2099 
2099 
2099 
2099 
2099 
2099 
921191616067286
species

111111
 2128 
species
665152505089

2128 
2128 
2128 
2128 
2128 
2128 

111
 86660 
species
211


86660 
86660 


86660 

species


50052 

50052 
21
 50052 
11

 2096 
24161212933

2096 
2096 
2096 
2096 
2096 
2096 
species
111111

111111
 92400 

92400 
92400 
92400 
92400 
92400 
92400 
4583220
species

111111
 45362 
species
10657212

45362 
45362 
45362 
45362 
45362 
45362 

species


28227 
28227 
11
 28227 
11

111111
species

2107 
2107 
2107 
2107 
2107 
2107 
5668803533144
 2107 

221122
2129
332145
genus

111111
 2130 

2130 
2130 
2130 
2130 
2130 
2130 
222122
species

1111
 134821 
species

134821 
134821 


134821 
134821 
1123

5786415
172134131793
order
186328

4786312
2131
family
162134131387

2132
genus
162134131387
4786312

11111

2136 
2136 
2136 
2136 

2136 
11436
species
 2136 

111
species
211

2145 

2145 


2145 
 2145 

11
 47834 
16


47834 


47834 
species

2121


216935 
216935 
216935 

216935 
species
 216935 
1111

species
11


2137 


2137 
 2137 
11

111111

216936 
216936 
216936 
216936 
216936 
216936 
86113114
species
 216936 


216934 
1
species
 216934 
1

11
 1114980 


1114980 


1114980 
11
species

1
species


2144 
1
 2144 


216946 
216946 
216946 
216946 
216946 
216946 
591221147
species
 216946 
111111

1111
 362837 
species


362837 
362837 
362837 

362837 
1324

11
species
12


216933 
216933 
 216933 

1
 216937 
species
2


216937 

 315358 
species
2


315358 
1

113
33925
family
146

145
genus
46239
112

1
1


219745 
species
 219745 

111
species

2151 


2151 
2151 
144
 2151 

46238
genus
1
1

 215578 
species
1


215578 
1

212214
186329
3262418
order

2146
family
3262418
212214

genus
112
2147
111

species

61635 


61635 
11
 61635 
11

1
 38986 
2


38986 
species

112113
2261416
genus
33926

11
species group
85632
11

11


59748 


59748 
species
 59748 
11

1
2
species group
85630

species
2


37692 
 37692 
1

111111
species group
2251413
85620

 229545 
225413

229545 
229545 
229545 

229545 
229545 
species
11111

species
1


202462 
 202462 
1

1
1

1911684 
species
 1911684 

1
 1911683 
species


1911683 
1

2138240
11
phylum
11

2138243
class
11
11

11
order
2138246
11

11
2138247
11
family

68335
genus
11
11

11
 35786 
species


35786 
35786 
11

11
68297
phylum
11

203486
class
11
11

11
203487
11
order

11
11
family
203488

11
11
genus
13

11


513050 
513050 
species
 513050 
11

200938
phylum
6743134
111111

111111
118001
6743134
class

111111
6743134
order
189769

111111
189770
6743134
family

111111
6743134
genus
393029

 936456 

936456 
936456 
936456 
936456 
936456 
936456 
6743134
species
111111

525255
204428
phylum
1841732017

525255
204429
1841732017
class

111
1963360
order
114

11
family
11
92713

11
83551
11
genus

 83552 
11

83552 


83552 
species
11

4
family
92714
1

71666
4
genus
1

1


71667 
4
species
 71667 

425244
1741731913
order
51291

425244
809
family
1741731913

1741731913
genus
810
425244

11
 83554 
species
52

83554 

83554 

111111
 813 

813 
813 
813 
813 
813 
813 
5311279
species

111
 85991 
123


85991 
85991 

85991 
species

1
 1457153 
species
1


1457153 

1


83557 
7
species
 83557 

1
species

83559 
6
 83559 

species

83558 

83558 
83558 
83558 
83558 
11122
 83558 
11111

11
species


83560 


83560 
11
 83560 

131211
200930
131213
phylum

68337
131213
class
131211

131211
191393
order
131213

131211
191394
family
131213

1111
genus
1113
2351

species

2352 
2352 
2352 


2352 
1113
 2352 
1111

11
11
genus
53572

species
11


197162 
197162 
 197162 
11

11
genus
11
545865

 477976 
11


477976 

477976 
species
11

genus
1
117999
1

1
 118000 
1


118000 
species

200795
phylum
5743931068550
91114111211

23213
292625
771027
class

23213
292629
771027
order

23213
292628
771027
family

11111
233189
63525
genus

11111
 167964 


167964 
167964 
167964 
167964 
167964 
63525
species

1111
1151
genus
2019482

1111
species
1151


1986204 
1986204 
1986204 

1986204 
 1986204 

species
31


1889813 


1889813 
 1889813 
11

222122
861414186
class
189775

111111
85000
subclass
651314114

85001
order
651314114
111111

111111
255728
suborder
651314114

651314114
family
85002
111111

genus
651314114
2056
111111

111111
 2057 
species

2057 
2057 
2057 
2057 
2057 
2057 
651314114

21172
order
189776
11111

21172
family
189777
11111

499
21172
genus
11111

 500 

500 
500 
500 

500 
500 
21172
species
11111

434443
class
15919351212
32061

order
15919351212
32064
434443

1508595
suborder
5510785
222221

1508635
5510785
family
222221

222221
120961
genus
5510785

11111
species

357808 
357808 
357808 
357808 
357808 
33511
 357808 

species

120962 
120962 
120962 
120962 
120962 
120962 
225675
 120962 
111111

212222
1508594
10492847
suborder

10492847
family
1106
212222

1107
genus
10492847
212222

 152260 
421031

152260 

152260 
152260 
152260 
152260 
species
11111

111111
 1108 
6471816

1108 
1108 
1108 
1108 
1108 
1108 
species

class
23923222012
301297
123231

23817171212
order
1202465
111111

111111
23817171212
family
1202464

111111
genus
23817171212
61434

species

61435 
61435 
61435 
61435 
61435 
61435 
23817171212
 61435 
111111

1212
670486
1658
genus

11


552810 

552810 
16
species
 552810 

 1839801 


1839801 
1839801 
1839801 
1839801 
1552
species
1111

class
661017308
475962
111111

111111
475963
661017308
order

family
661017308
475964
111111

genus
661017308
233191
111111

 133453 
species

133453 
133453 
133453 
133453 
133453 
133453 
661017308
111111

class
5620835
1382928
111111

1382929
order
5620835
111111

111111
5620835
family
1382930

5620835
genus
1988031
111111

111111
 1806508 
species

1806508 
1806508 
1806508 
1806508 
1806508 
1806508 
5620835

534555
phylum
211427212516
40117

534555
203693
211427212516
class

534555
189778
order
211427212516

189779
family
211427212516
534555

28261
8
genus
1

1
species
8


28262 
 28262 

1234
201319202413
genus
423443

species
42238

1715989 

1715989 
1715989 
1715989 
1715989 
 1715989 
11111

 330214 
species

330214 
330214 
330214 
330214 
330214 
330214 
1199972
111111

111111
species
3488133

42253 
42253 
42253 
42253 
42253 
42253 
 42253 

111
species

1325564 


1325564 
1325564 
211
 1325564 

179
genus
11113
11112

11
261385
11
species group

11
 1660083 
species
11


1660083 
1660083 

261386
1112
species group
1111

1111
1112

178606 
178606 

178606 

178606 
species
 178606 

phylum
535292694478
203691
151720151918

203692
class
535292694478
151720151918

1643686
order
59318610
144332

144332
59318610
family
143786

144332
59318610
genus
29521

111
331


84378 
84378 

84378 
species
 84378 

1111
 52584 
species


52584 
52584 
52584 

52584 
1332

 84377 
species
1111


84377 
84377 
84377 
84377 
1111

111111
 159 
species

159 
159 
159 
159 
159 
159 
5424448

136
313749423256
order
91013101212

768898
137
family
283242342748

146
genus
2511531
222121

1111
1112

46355 
46355 
46355 

46355 
species
 46355 

111111
1410511

154 
154 
154 
154 
154 
154 
species
 154 

331
genus
1616789
111

 1307761 


1307761 
1307761 

1307761 
331
species
111

111111
8357220
genus
399320

111111
 273376 

273376 
273376 
273376 
273376 
273376 
273376 
8357220
species

genus
3
1911556
1

1
 55206 
species


55206 
3

434555
157
182423191926
genus

species

88058 

88058 
88058 
88058 
88058 
21116
 88058 
11111

 160 
species
111

160 


160 
160 
111

11111
 81028 
15164

81028 
81028 
81028 

81028 
81028 
species

111111
14182015713

1539298 
1539298 
1539298 
1539298 
1539298 
1539298 
species
 1539298 

2


221027 
species
 221027 
1

111
species
111


215591 
215591 
215591 
 215591 

11
 150829 
species
11


150829 

150829 

1
 409322 
species
4


409322 

245234
family
357858
1643685

138
genus
11426
11313

 40834 
11222

40834 
40834 
40834 

40834 
40834 
species
11111

1


47466 
species
 47466 
1

species
2


44449 
 44449 
1

species


142 


142 
12
 142 
11

132221
64895
243832
genus

1


64897 
species
 64897 
1

1


139 
species
 139 
1

 664662 


664662 
1
species
1

11
species
21


62088 
62088 
 62088 

1
species


29519 
6
 29519 

 29518 
species

29518 
29518 
29518 

29518 
29518 
22222
11111

533244
family
1761219612
170

338321
3131531
genus
111111

111111
3131531

29510 
29510 
29510 
29510 
29510 
29510 
species
 29510 

422133
14594311
genus
171

111
 172 
179

172 

172 


172 
species

species
24411

173 
173 

173 
173 
173 
 173 
11111

111
 174 
species

174 
174 


174 
111

1111
 28452 
species
10211

28452 

28452 

28452 
28452 

1111
5642
phylum
65842

204430
5642
class
1111

1111
218872
order
5642

1111
204431
5642
family

1111
832
5642
genus

1111
species


833 
833 
833 
833 
5642
 833 

131213141314
203682
169152136288239149
phylum

121112121212
118110116237192106
class
203683

order
115110116229189105
112
111112111111

126
57505311810956
family
667666

genus
213332
1936111
111111

111111
 1891926 
213332

1891926 
1891926 
1891926 
1891926 
1891926 
1891926 
species

111111
genus
45529536
265488

111111
 265606 

265606 
265606 
265606 
265606 
265606 
265606 
45529536
species

11
31
genus
1676125

 1331910 
31


1331910 
1331910 
species
11

genus
192136597211
118
222222

species

1636152 
1636152 
1636152 
1636152 
1636152 
1636152 
10133035559
 1636152 
111111

98624172

1632864 
1632864 
1632864 
1632864 
1632864 
1632864 
species
 1632864 
111111

111111
49226265
genus
1649490

111111
species

119 
119 
119 
119 
119 
119 
49226265
 119 

123
2814432
genus
11111

11111
 125 

125 
125 
125 

125 
125 
2814432
species

1763524
321516683627
family
333333

127
316322
genus
111111

111111
 128 

128 
128 
128 
128 
128 
128 
316322
species

111111
1763521
genus
9105361624

9105361624

1387353 
1387353 
1387353 
1387353 
1387353 
1387353 
species
 1387353 
111111

111111
204529181
genus
466152

 466153 

466153 
466153 
466153 
466153 
466153 
466153 
204529181
species
111111

222222
family
264547434422
1914233

222222
113
genus
264547434422

111111
 114 
181429201212

114 
114 
114 
114 
114 
114 
species

 1630693 
species
83118233210

1630693 
1630693 
1630693 
1630693 
1630693 
1630693 
111111

1127829
order
3831
1111

1111
1127830
3831
family

380738
genus
3831
1111

1111
 174633 
species

174633 


174633 
174633 
174633 
3831

666505
class
514220514743
111212

1
species


1940790 
1
 1940790 

1
 1941349 
2


1941349 
species

666506
514220504741
order
111111

666507
family
514220504741
111111

111111
genus
514220504741
666508


547188 
547188 
547188 
547188 
547188 
547188 
514220504741
species
 547188 
111111

262406
11421
genus
11111

11111
 166501 
11421

166501 

166501 
166501 
166501 
166501 
species

1224
phylum
242406127221431730946207847619914061707147
137613521357138013741341

65341250339
class
1807140
333333

333333
225057
order
65341250339

333333
225058
family
65341250339

333333
genus
65341250339
119977

 33059 
75332185

33059 
33059 
33059 
33059 
33059 
33059 
species
111111

111111

920 
920 
920 
920 
920 
920 
5728812123
species
 920 

111111
species

160808 
160808 
160808 
160808 
160808 
160808 
111631
 160808 

808286838676
68525
subphylum
149312671347194318161351

202121252118
class
186167146333150215
29547

269258
11
genus
11

11
 387092 
species


387092 

387092 
11

265570
32
genus
21

1
 206403 
species

206403 
2

11
species
12

387093 

387093 
 387093 

181920242117
183165144332150214
order
213849

111317161513
family
5930421495760
72294

881110108
genus
531232993941
194

111

497724 
497724 
497724 
111
species
 497724 

1111
 198 


198 
198 
198 
198 
1223
species


199 
199 
199 
142
species
 199 
111

11111
 195 
species
112143

195 

195 
195 
195 
195 

 1660074 


1660074 
1
species
1

111111
 1965231 
species

1965231 
1965231 
1965231 
1965231 
1965231 
1965231 
524212

111
 200 
species
171

200 


200 
200 

111111
species

197 
197 
197 
197 
197 
197 
26211661923
 197 

111
 1244531 
121


1244531 
1244531 


1244531 
species

species
111

76517 

76517 

76517 
 76517 
111

 374106 
species


374106 
1
1

 75658 
species
712


75658 
75658 
75658 
111

 196 
species

196 
196 
196 

196 
196 
53323
11111

1
2


1031542 
species
 1031542 

 206 
species


206 
1
1

species
101


28898 
28898 
 28898 
11

 201 
species

201 


201 

201 
314
111


824 
824 
17
species
 824 
11

234232
587311516
genus
28196

111111
461261115

28197 
28197 
28197 
28197 
28197 
28197 
species
 28197 

111
species
112


944547 
944547 

944547 
 944547 


28199 
28199 
28199 
28199 
28199 
14521
species
 28199 
11111

11

1850254 

1850254 
11
species
 1850254 

122423
57665
11031933
genus

 194424 
species
1


194424 
1

1
 366522 


366522 
1
species

 220622 


220622 
220622 
220622 

220622 
82141
species
1111

1111


1986224 
1986224 
1986224 
1986224 
2112
species
 1986224 

11
species

65553 


65553 
13
 65553 

1
 44674 
species


44674 
1

 66821 
species
1


66821 
1

5213121
genus
269260
11111

 269261 
species

269261 
269261 
269261 
269261 
269261 
5213121
11111

1191149917192154
family
72293
652754

1152
genus
202746
111

152


39766 
39766 
species
 39766 
11

1
1


202747 
species
 202747 

843
11
genus
11

11
 844 
species

844 


844 
11

286130
12
genus
11


148813 
148813 
12
species
 148813 
11

genus
1171119915689154
209
432634

11
 135569 
species
11

135569 


135569 

1
species
1


32025 
 32025 

species

210 
210 
210 
210 
210 
210 
1081079714981145
 210 
111111


76936 
1
species
 76936 
1

111
 1591088 

1591088 


1591088 
1591088 
131
species

species
1


222136 
 222136 
1

111111
species

138563 
138563 
138563 
138563 
138563 
138563 
732357
 138563 

11
11


214 

214 
species
 214 

order
11
235899
11

224467
11
family
11

11
genus
191291
11

species
11


244787 


244787 
 244787 
11

class
130711001201161016661136
28221
606165586558

11
order
213113
11

11
11
family
117942

11
genus
84404
11

11
 84405 
species

84405 


84405 
11

598696
order
383946626154
213118

family
131910301923
213121
244343

1242488
genus
427922
111111

 427923 
species
1242488

427923 
427923 
427923 
427923 
427923 
427923 
111111

11111
genus
131723
109168

11111
 84980 


84980 
84980 
84980 
84980 
84980 
131723
species

111111
893
11219812
genus

 1986146 
species
11219812

1986146 
1986146 
1986146 
1986146 
1986146 
1986146 
111111

111
genus
151
53318

111
 65555 


65555 
65555 

65555 
151
species

213119
252036324231
family
354353

222122
896
genus
171230172426

 181663 
647128

181663 
181663 
181663 

181663 
181663 
species
11111

111111
 897 
species
11823171218

897 
897 
897 
897 
897 
897 

111111
218207
82511155
genus

111111
species
82511155

259354 
259354 
259354 
259354 
259354 
259354 
 259354 

11
52
genus
28222


28223 


28223 
52
species
 28223 
11

1111
genus
1141
2295

 2296 
species


2296 
2296 
2296 
2296 
1141
1111

order
662590758845872519
29
181818181818

80812
189108134220205186
suborder
333333

111111
1055686
282333325326
family

1055688
282333325326
genus
111111

111111
 927083 
282333325326

927083 
927083 
927083 
927083 
927083 
927083 
species

49
family
16185101188152160
222222

201418263012
genus
50
111111

111111
species

52 
52 
52 
52 
52 
52 
201418263012
 52 

111111
1417183162122148
genus
39643

111111
 56 

56 
56 
56 
56 
56 
56 
1417183162122148
species

111111
262335384424
suborder
224462

224464
262335384424
family
111111

162027
genus
262335384424
111111

 80816 
species
262335384424

80816 
80816 
80816 
80816 
80816 
80816 
111111

suborder
447459589587623309
80811
141414141414

31
195206289240288157
family
666666

555555
32
genus
164136243207237109

111111
 1297742 
261624263620

1297742 
1297742 
1297742 
1297742 
1297742 
1297742 
species

111111

33 
33 
33 
33 
33 
33 
6966997610129
species
 33 

 35 
species

35 
35 
35 
35 
35 
35 
301973422618
111111

111111
 34 
species

34 
34 
34 
34 
34 
34 
20112629339

111111

83455 
83455 
83455 
83455 
83455 
83455 
192421344133
species
 83455 

317046335148
genus
83461
111111

species

184914 
184914 
184914 
184914 
184914 
184914 
317046335148
 184914 
111111

444444
39
138122134143173104
family

111111
352019362421
genus
44

111111
species

83453 
83453 
83453 
83453 
83453 
83453 
352019362421
 83453 

42
252550649042
genus
111111

252550649042

43 
43 
43 
43 
43 
43 
species
 43 
111111

40
676530264017
genus
111111

111111
 41 
species
676530264017

41 
41 
41 
41 
41 
41 

47
111235171924
genus
111111

111111
 48 
111235171924

48 
48 
48 
48 
48 
48 
species

family
126232182
1524213
111111

111111
genus
126232182
1524214

 1391653 
species
126232182

1391653 
1391653 
1391653 
1391653 
1391653 
1391653 
111111

10212514318315446
family
1524215
333333

161492
genus
10212514318315446
333333

111111
species
7854731069422

161493 
161493 
161493 
161493 
161493 
161493 
 161493 

136327514016

447217 
447217 
447217 
447217 
447217 
447217 
species
 447217 
111111

111111
 404589 
species
1184326208

404589 
404589 
404589 
404589 
404589 
404589 

69541
205154125291271171
order
151617161716

666666
213421
family
844533959446

18
361719494019
genus
444444

111111
574332

29542 
29542 
29542 
29542 
29542 
29542 
species
 29542 


19 
19 
19 
19 
19 
19 
12223210
species
 19 
111111

61516173

1842532 
1842532 
1842532 
1842532 
1842532 
1842532 
species
 1842532 
111111

111111
24787184

29543 
29543 
29543 
29543 
29543 
29543 
species
 29543 

222222
482814465427
genus
890

111111
 1823759 
species

1823759 
1823759 
1823759 
1823759 
1823759 
1823759 
38167324023

111111
species

1603606 
1603606 
1603606 
1603606 
1603606 
1603606 
1012714144
 1603606 

family
12110992196177125
213422
91011101110

89109109
28231
genus
11710888195165121


443143 
443143 
443143 
443143 
443143 
443143 
7425441210
species
 443143 
111111

 1340425 
species
19176412839

1340425 
1340425 
1340425 
1340425 
1340425 
1340425 
111111

species
31


1203471 

1203471 
 1203471 
11

111111
 443144 
1651320118

443144 
443144 
443144 
443144 
443144 
443144 
species

111111
 35554 
1075333616

35554 
35554 
35554 
35554 
35554 
35554 
species

231078921

28232 
28232 
28232 
28232 
28232 
28232 
species
 28232 
111111


345632 
345632 
345632 
345632 
345632 
345632 
123763
species
 345632 
111111

 313985 
species

313985 
313985 
313985 
313985 
313985 
313985 
13171118
111111

111111

225194 
225194 
225194 
225194 
225194 
225194 
28617274712
species
 225194 

11111
species
1121444


351604 
351604 
351604 
351604 
351604 
 351604 

111111
4141124
genus
392332

species

483547 
483547 
483547 
483547 
483547 
483547 
4141124
 483547 
111111

324221
4349201
order
213462

213491
family
213468
213111

genus
11
60892
11

 60893 

60893 

60893 
11
species
11

11
43773
genus
19

11
species
19


316277 

316277 
 316277 

11111
11141
genus
2357

 2358 
species
11141

2358 
2358 
2358 
2358 

2358 
11111

11111
221511
family
213465

11111
29526
genus
221511

11111
 119484 
221511

119484 
119484 
119484 
119484 
119484 
species

1551504
2
genus
1

1
 673862 
species
2


673862 

1779134
order
186111326
111111

186111326
family
1779135
111111

111111
186111326
genus
1779136

111111
 1548548 
species

1548548 
1548548 
1548548 
1548548 
1548548 
1548548 
186111326

111111
453227
2518120515
order

111111
family
2518120515
453228

111111
2518120515
genus
453229

111111
 453230 
species

453230 
453230 
453230 
453230 
453230 
453230 
2518120515

213115
order
354290256370434368
161416141614

1463310
family
213117
111111

111111
45662
genus
1463310

 45663 
species
1463310

45663 
45663 
45663 
45663 
45663 
45663 
111111

131114111311
194924
family
305244238313381318

333332
genus
282130545211
2035811

species
42523

57320 
57320 
57320 
57320 
57320 
 57320 
11111

species

182210 
182210 
182210 
182210 
182210 
182210 
4121338205
 182210 
111111

111111
 1716143 
species
2071214296

1716143 
1716143 
1716143 
1716143 
1716143 
1716143 

111111
genus
3582134
41707

111111
species

29546 
29546 
29546 
29546 
29546 
29546 
3582134
 29546 

9710798
872
genus
274218200257328273

 880 
species

880 
880 
880 
111
111

species
31


1725232 

1725232 
 1725232 
11

111111
 901 

901 
901 
901 
901 
901 
901 
15104131811
species


881 
881 
881 
881 
881 
881 
8361468414350
species
 881 
111111


44742 
44742 
44742 
44742 
44742 
44742 
107108897685130
species
 44742 
111111

 876 

876 
876 
876 
876 
876 
876 
16195293145
species
111111

111111
species
291016231817

873 
873 
873 
873 
873 
873 
 873 

11111
species

631220 

631220 
631220 
631220 
631220 
791144
 631220 

111111
 184917 
15921212315

184917 
184917 
184917 
184917 
184917 
184917 
species

species
1651

58180 

58180 

58180 
58180 
 58180 
1111

221222
213116
484212545040
family

221222
898
genus
484212545040

474112524238

899 
899 
899 
899 
899 
899 
species
 899 
111111

11111
 132132 
species

132132 
132132 

132132 
132132 
132132 
11282

class
197031861012723210202877615368
28211
374366370378377364

676768696768
204455
252324341710255329471682
order

646365656564
31989
249823861685251829181636
family

2433
26817333233
genus
222222

species
1359251724

2434 
2434 
2434 
2434 
2434 
2434 
 2434 
111111

111111
species

42443 
42443 
42443 
42443 
42443 
42443 
13388159
 42443 

222222
354203
6367587016851
genus

111111
293923449419

1792508 
1792508 
1792508 
1792508 
1792508 
1792508 
species
 1792508 

 311180 
species
342835267432

311180 
311180 
311180 
311180 
311180 
311180 
111111

 2033435 
1811914105

2033435 
2033435 
2033435 
2033435 
2033435 
2033435 
species
111111

11111
1579315
genus
478711

11111
species
478711

1579316 
1579316 

1579316 
1579316 
1579316 
 1579316 

360528
361723163118
genus
111111


1250539 
1250539 
1250539 
1250539 
1250539 
1250539 
361723163118
species
 1250539 
111111

111111
191028
genus
5761886327

111111
5761886327

133924 
133924 
133924 
133924 
133924 
133924 
species
 133924 

111111
1759396
9228191
genus

 1920883 
species

1920883 
1920883 
1920883 
1920883 
1920883 
1920883 
9228191
111111

genus
78410469
1649279
111111

111111
78410469

379347 
379347 
379347 
379347 
379347 
379347 
species
 379347 

genus
584535565649
263377
111111

 1229727 

1229727 
1229727 
1229727 
1229727 
1229727 
1229727 
584535565649
species
111111

11111
52336
genus
119541

species

441209 
441209 
441209 
441209 
441209 
52336
 441209 
11111

36213527227
genus
1609958
111111

 1609966 
36213527227

1609966 
1609966 
1609966 
1609966 
1609966 
1609966 
species
111111

genus
551440248509553355
302485
434444

111111
species

221822 
221822 
221822 
221822 
221822 
221822 
343227146291319245
 221822 

111111
 1580596 
species
828536985163

1580596 
1580596 
1580596 
1580596 
1580596 
1580596 

 1844006 
species
83844

1844006 

1844006 
1844006 
1844006 
1844006 
11111

 60890 
1181286311217943

60890 
60890 
60890 
60890 
60890 
60890 
species
111111

111111
204456
6248135816
genus

111111
 2169400 

2169400 
2169400 
2169400 
2169400 
2169400 
2169400 
6248135816
species

159345
genus
151524222317
111111

111111
species

159346 
159346 
159346 
159346 
159346 
159346 
151524222317
 159346 

222222
92944
16616499150111120
genus

111111
species

92947 
92947 
92947 
92947 
92947 
92947 
301919212024
 92947 

111111
 92945 
species

92945 
92945 
92945 
92945 
92945 
92945 
136145801299196

58842
393141524944
genus
111111

species
393141524944

2009329 
2009329 
2009329 
2009329 
2009329 
2009329 
 2009329 
111111

111111
genus
4982136134
74032

111111
species
4982136134

74033 
74033 
74033 
74033 
74033 
74033 
 74033 

53945
19366141817
genus
223322

111
 1458307 
species
121


1458307 
1458307 
1458307 

111111
species
1335481716

1217908 
1217908 
1217908 
1217908 
1217908 
1217908 
 1217908 

species

53946 
53946 
53946 
53946 

53946 
61141
 53946 
11111

111111
188905
162516810
genus

 290400 
162516810

290400 
290400 
290400 
290400 
290400 
290400 
species
111111

111111
14189181010
genus
2211641

14189181010

245188 
245188 
245188 
245188 
245188 
245188 
species
 245188 
111111

333333
34008
121159928917272
genus

111111
 1564506 
species

1564506 
1564506 
1564506 
1564506 
1564506 
1564506 
42251143211

 35806 
species

35806 
35806 
35806 
35806 
35806 
35806 
7292606710251
111111

 308754 
74221183810

308754 
308754 
308754 
308754 
308754 
308754 
species
111111

111111
56910145
genus
309512

 215813 

215813 
215813 
215813 
215813 
215813 
215813 
56910145
species
111111

664217254623
genus
60136
222222

111111
species
4527515399

1402135 
1402135 
1402135 
1402135 
1402135 
1402135 
 1402135 

111111

1917485 
1917485 
1917485 
1917485 
1917485 
1917485 
21151210714
species
 1917485 

111111
524032376531
genus
227873

111111
species
524032376531

121719 
121719 
121719 
121719 
121719 
121719 
 121719 

111111
genus
556031395521
367771

species

42444 
42444 
42444 
42444 
42444 
42444 
556031395521
 42444 
111111

737134367352
genus
478070
333333

15269142010

1674922 
1674922 
1674922 
1674922 
1674922 
1674922 
species
 1674922 
111111

species
251810122114

187304 
187304 
187304 
187304 
187304 
187304 
 187304 
111111

 2021862 
species
332715103228

2021862 
2021862 
2021862 
2021862 
2021862 
2021862 
111111

1060
genus
330316185252374182
444444

111111
 1063 

1063 
1063 
1063 
1063 
1063 
1063 
270235133187186136
species

 1061 
20171317374

1061 
1061 
1061 
1061 
1061 
1061 
species
111111

 1850250 
species
122512189517

1850250 
1850250 
1850250 
1850250 
1850250 
1850250 
111111

111111
 2033869 

2033869 
2033869 
2033869 
2033869 
2033869 
2033869 
283927305625
species

111111
299261
genus
122035322830

111111
species
122035322830

299262 
299262 
299262 
299262 
299262 
299262 
 299262 

 1904441 
86165

1904441 
1904441 
1904441 

1904441 
1904441 
species
11111

111111
332214315936
genus
1097466

111111
 1335048 
species

1335048 
1335048 
1335048 
1335048 
1335048 
1335048 
332214315936

334444
genus
10364499411879
875170

 1397108 
species


1397108 
1397108 
1397108 
1397108 
61821
1111

111111
 1411902 

1411902 
1411902 
1411902 
1411902 
1411902 
1411902 
26101314344
species

111111
species
261720193848

1758178 
1758178 
1758178 
1758178 
1758178 
1758178 
 1758178 

111111
 1208324 
species
513710434426

1208324 
1208324 
1208324 
1208324 
1208324 
1208324 

genus
5113515
1284657
111111

111111
5113515

1284658 
1284658 
1284658 
1284658 
1284658 
1284658 
species
 1284658 

111111
285107
genus
81811303134

111111
 1915078 
species
81811303134

1915078 
1915078 
1915078 
1915078 
1915078 
1915078 

genus
20141662711
74030
222222

111111
1511144253

215743 
215743 
215743 
215743 
215743 
215743 
species
 215743 

111111
species
532228

391613 
391613 
391613 
391613 
391613 
391613 
 391613 

222222
97050
genus
43273133615

111111
2313171436

292414 
292414 
292414 
292414 
292414 
292414 
species
 292414 

111111
2014141939

89184 
89184 
89184 
89184 
89184 
89184 
species
 89184 

331511451661475198
genus
265
999999

111111

266 
266 
266 
266 
266 
266 
522963347023
species
 266 

111111
 147645 
10623218933115065

147645 
147645 
147645 
147645 
147645 
147645 
species

111111
 1945662 
16352841339

1945662 
1945662 
1945662 
1945662 
1945662 
1945662 
species

 1499308 
species
155937567718

1499308 
1499308 
1499308 
1499308 
1499308 
1499308 
111111

species

2065379 
2065379 
2065379 
2065379 
2065379 
2065379 
354139273615
 2065379 
111111

 1529068 
species
33141783223

1529068 
1529068 
1529068 
1529068 
1529068 
1529068 
111111

 1077935 
species

1077935 
1077935 
1077935 
1077935 
1077935 
1077935 
182938572525
111111

species

34003 
34003 
34003 
34003 
34003 
34003 
174720682115
 34003 
111111

111111
39252039315

34004 
34004 
34004 
34004 
34004 
34004 
species
 34004 

392221576123
genus
436357
111111

111111
species

2109625 
2109625 
2109625 
2109625 
2109625 
2109625 
392221576123
 2109625 

69657
254825352946
family
343424

2723
genus
21914
1111

1111
 2724 
21914

2724 
2724 

2724 

2724 
species

122212
85
genus
15411521920

1111
species
1984


1906738 
1906738 
1906738 

1906738 
 1906738 

species
1540613916

81032 
81032 
81032 
81032 
81032 
81032 
 81032 
111111

111111
74317
genus
861052012


74318 
74318 
74318 
74318 
74318 
74318 
861052012
species
 74318 
111111

order
6507064948911016630
204458
151515151515

76892
family
6507064948911016630
151515151515

75
407441287489632450
genus
555555

111111
 88688 

88688 
88688 
88688 
88688 
88688 
88688 
9014296169181156
species

species

155892 
155892 
155892 
155892 
155892 
155892 
158198117178327163
 155892 
111111

111111
species

366602 
366602 
366602 
366602 
366602 
366602 
856221817198
 366602 

 69395 
441821152518

69395 
69395 
69395 
69395 
69395 
69395 
species
111111


69666 
69666 
69666 
69666 
69666 
69666 
302132462815
species
 69666 
111111

222222
20
6661627910872
genus

111111
 2201350 

2201350 
2201350 
2201350 
2201350 
2201350 
2201350 
131833163348
species

 284016 
species
534329637524

284016 
284016 
284016 
284016 
284016 
284016 
111111

 1759059 

1759059 
1759059 
1759059 
1759059 
1759059 
1759059 
410517262
species
111111

16016412123120081
genus
41275
666666


588932 
588932 
588932 
588932 
588932 
588932 
331016221711
species
 588932 
111111

species

1938605 
1938605 
1938605 
1938605 
1938605 
1938605 
271316516320
 1938605 
111111

111111
 1532555 

1532555 
1532555 
1532555 
1532555 
1532555 
1532555 
353112263712
species

species

74313 
74313 
74313 
74313 
74313 
74313 
176315643511
 74313 
111111

 1827469 
332027322612

1827469 
1827469 
1827469 
1827469 
1827469 
1827469 
species
111111

111111
152735362215

41276 
41276 
41276 
41276 
41276 
41276 
species
 41276 

genus
133019755025
76890
111111

111111
 78587 
133019755025

78587 
78587 
78587 
78587 
78587 
78587 
species

11111
genus
734510
213485

species

349221 
349221 
349221 
349221 
349221 
734510
 349221 
11111

149149150151152151
order
1117110909754312195180179707
356

222222
119043
family
413035516810

111111
genus
1412811256
444432

111111
species
1412811256

1922226 
1922226 
1922226 
1922226 
1922226 
1922226 
 1922226 

256616
27182740434
genus
111111

111111
 256618 
species

256618 
256618 
256618 
256618 
256618 
256618 
27182740434

family
107110846079611194834
118882
151513151715

234
genus
9529255328841109757
11119111311

species

1885919 
1885919 
1885919 
1885919 
1885919 
1885919 
14181391312
 1885919 
111111

111111
species
1481246699120133

29461 
29461 
29461 
29461 
29461 
29461 
 29461 

111111
 235 
1171758616110277

235 
235 
235 
235 
235 
235 
species

 120576 
species
826132447

120576 
120576 
120576 
120576 
120576 
120576 
111111

111111
species

1149952 
1149952 
1149952 
1149952 
1149952 
1149952 
1322221
 1149952 

111111
 29459 
601532337521743459

29459 
29459 
29459 
29459 
29459 
29459 
species

species

36855 
36855 
36855 
36855 
36855 
36855 
292412575349
 36855 
111111

 1844051 
5224

1844051 

1844051 
1844051 
1844051 
species
1111

 1891098 
species
141173

1891098 
1891098 

1891098 
1891098 
1891098 
11111

1111
species
141123

444163 
444163 


444163 
444163 
 444163 

111
74210


120577 


120577 
120577 
species
 120577 


981386 
981386 
981386 
981386 
981386 
21613
species
 981386 
11111

species

236 


236 
236 
226
 236 
111

11915975778577
genus
528
444444

111111
species
1758284

419475 
419475 
419475 
419475 
419475 
419475 
 419475 

111111
 571256 
292699179

571256 
571256 
571256 
571256 
571256 
571256 
species

 529 
421926455258

529 
529 
529 
529 
529 
529 
species
111111

111111
 271865 
species

271865 
271865 
271865 
271865 
271865 
271865 
31109322186

222222
45404
383920517340
family

111111
1912713233
genus
532

 533 
species

533 
533 
533 
533 
533 
533 
1912713233
111111

111111
120652
genus
192713385037

111111

199596 
199596 
199596 
199596 
199596 
199596 
192713385037
species
 199596 

1734920
genus
432113334446
111111

111111
species
432113334446

1235591 
1235591 
1235591 
1235591 
1235591 
1235591 
 1235591 

111111
1572860
313126206629
genus

111111
species

1482074 
1482074 
1482074 
1482074 
1482074 
1482074 
313126206629
 1482074 

111111111111
45401
401382291357631289
family

genus
170101159111231118
59282
111111

111111
 1079 
species
170101159111231118

1079 
1079 
1079 
1079 
1079 
1079 

111111
119044
525244
genus

111111

1608628 
1608628 
1608628 
1608628 
1608628 
1608628 
525244
species
 1608628 

111111
6166356211857
genus
29407

species

674703 
674703 
674703 
674703 
674703 
674703 
6166356211857
 674703 
111111

genus
77113399512960
46913
333333

111111
species
386512285837

1643450 
1643450 
1643450 
1643450 
1643450 
1643450 
 1643450 


2083786 
2083786 
2083786 
2083786 
2083786 
2083786 
2112615269
species
 2083786 
111111

 1736675 

1736675 
1736675 
1736675 
1736675 
1736675 
1736675 
183621524514
species
111111

111111
genus
68518207
1068

 1069 

1069 
1069 
1069 
1069 
1069 
1069 
68518207
species
111111

1082930
genus
4176122912
111111

111111

531813 
531813 
531813 
531813 
531813 
531813 
4176122912
species
 531813 

333333
81
genus
7875425710031

 53399 
species

53399 
53399 
53399 
53399 
53399 
53399 
3140926539
111111

111111
 1427356 
species

1427356 
1427356 
1427356 
1427356 
1427356 
1427356 
222215133316

111111
 717785 
25131818146

717785 
717785 
717785 
717785 
717785 
717785 
species

333333
1104825509849
family
31993

425
genus
241712206113
111111

 426 
species

426 
426 
426 
426 
426 
426 
241712206113
111111

222222
133
863113303736
genus

species

187303 
187303 
187303 
187303 
187303 
187303 
5232102126
 187303 
111111

342811201610

655015 
655015 
655015 
655015 
655015 
655015 
species
 655015 
111111

191919191919
119045
175018281355230831301589
family

2282523
6386474108301016537
genus
333333

111111
 223967 

223967 
223967 
223967 
223967 
223967 
223967 
149159120227212148
species

111111

408 
408 
408 
408 
408 
408 
463462252548738374
species
 408 

111111
species
262638556615

29429 
29429 
29429 
29429 
29429 
29429 
 29429 

222222
186650
genus
5768566714654

111111

2082949 
2082949 
2082949 
2082949 
2082949 
2082949 
162423284113
species
 2082949 

 1882682 
species
4144333910541

1882682 
1882682 
1882682 
1882682 
1882682 
1882682 
111111

141414141414
407
1055111388914111968998
genus

111111
 270351 

270351 
270351 
270351 
270351 
270351 
270351 
92969613220276
species

 334852 

334852 
334852 
334852 
334852 
334852 
334852 
4381588313135
species
111111

 418223 
species

418223 
418223 
418223 
418223 
418223 
418223 
867636141149110
111111

 2202825 

2202825 
2202825 
2202825 
2202825 
2202825 
2202825 
3938615911348
species
111111

111111
 1826873 
4450548014685

1826873 
1826873 
1826873 
1826873 
1826873 
1826873 
species

 426117 
130945611610975

426117 
426117 
426117 
426117 
426117 
426117 
species
111111

 31998 
7950459317855

31998 
31998 
31998 
31998 
31998 
31998 
species
111111

species
6813069109149129

2202828 
2202828 
2202828 
2202828 
2202828 
2202828 
 2202828 
111111

111111
746411210214471

2202827 
2202827 
2202827 
2202827 
2202827 
2202827 
species
 2202827 

111111
 114616 
species
114107509313561

114616 
114616 
114616 
114616 
114616 
114616 

111111
 2067957 
species

2067957 
2067957 
2067957 
2067957 
2067957 
2067957 
799234648429

111111
 2202826 
97869015017185

2202826 
2202826 
2202826 
2202826 
2202826 
2202826 
species

 1479019 
species

1479019 
1479019 
1479019 
1479019 
1479019 
1479019 
60919712916797
111111

111111
505831609042

925818 
925818 
925818 
925818 
925818 
925818 
species
 925818 

69277
family
7728424947881190650
141414141414

888888
566450324475762392
genus
68287

111111
 71433 
6336234111172

71433 
71433 
71433 
71433 
71433 
71433 
species


2066070 
2066070 
2066070 
2066070 
2066070 
2066070 
3727293113330
species
 2066070 
111111

111111
 39645 
species
11611310019816992

39645 
39645 
39645 
39645 
39645 
39645 

species
1087727426464

536018 
536018 
536018 
536018 
536018 
536018 
 536018 
111111

111111
species
465531265477

1670800 
1670800 
1670800 
1670800 
1670800 
1670800 
 1670800 

835659477025

381 
381 
381 
381 
381 
381 
species
 381 
111111

111111
species

278153 
278153 
278153 
278153 
278153 
278153 
543920408111
 278153 


593909 
593909 
593909 
593909 
593909 
593909 
594735508021
species
 593909 
111111

111111
449972
genus
106109816

111111
species
106109816

266779 
266779 
266779 
266779 
266779 
266779 
 266779 

111111
245876
20403292037
genus

111111

472175 
472175 
472175 
472175 
472175 
472175 
20403292037
species
 472175 

222222
141319146253348164
genus
31988

 83263 

83263 
83263 
83263 
83263 
83263 
83263 
6049578710646
species
111111

111111
species
8127089166242118

374606 
374606 
374606 
374606 
374606 
374606 
 374606 

111111
28100
251064624
genus

species

1867719 
1867719 
1867719 
1867719 
1867719 
1867719 
251064624
 1867719 
111111

10175184617
genus
274591
111111

111111
 1620421 

1620421 
1620421 
1620421 
1620421 
1620421 
1620421 
10175184617
species

636555
401616361426
family
772

773
genus
401616361426
636555

 38323 
species
81

38323 


38323 
11

1
 33045 
1


33045 
species

1111
species

1933904 

1933904 
1933904 

1933904 
2211
 1933904 

1
species


774 
1
 774 

 1933912 


1933912 
1933912 
15
species
11

11
 388640 
12


388640 


388640 
species

1
1


515256 
species
 515256 

 1933907 
species
1


1933907 
1

111
 85701 
species


85701 
85701 
85701 
211

13652

1686310 
1686310 
1686310 
1686310 
1686310 
species
 1686310 
11111

 803 
species
1311528917

803 
803 
803 
803 
803 
803 
111111

 1318743 
species

1318743 
5
1

 33047 
species
11

33047 
1

414444444344
82115
413540152956440772733875
family

122223
34019
165466
genus

species


1273132 
1273132 

1273132 
1273132 
3141
 1273132 
1111

1
 34020 


34020 
1
species

1
2


556287 
species
 556287 

 34021 
134224

34021 
34021 
34021 
34021 
34021 
34021 
species
111111

111111
genus
59126548419999
1525371

111111
 399 
59126548419999

399 
399 
399 
399 
399 
399 
species

242626262525
211020811581231037232068
genus
379

 1703961 
2222


1703961 
1703961 
1703961 
1703961 
species
1111

111111
 1981173 
species

1981173 
1981173 
1981173 
1981173 
1981173 
1981173 
386626706741


1125847 
1125847 
1125847 
1125847 
1125847 
1125847 
382220305018
species
 1125847 
111111

1111
species
1461


1703966 
1703966 
1703966 

1703966 
 1703966 

species
5317664126921059625

384 
384 
384 
384 
384 
384 
 384 
111111

111111
414126334427

1703960 
1703960 
1703960 
1703960 
1703960 
1703960 
species
 1703960 

202926537967

1571470 
1571470 
1571470 
1571470 
1571470 
1571470 
species
 1571470 
111111

species
662020448219

1703968 
1703968 
1703968 
1703968 
1703968 
1703968 
 1703968 
111111

111111
species

29449 
29449 
29449 
29449 
29449 
29449 
338251216342436338
 29449 

111111
 1301032 
species
272720435427

1301032 
1301032 
1301032 
1301032 
1301032 
1301032 

 2020311 
species
252547467416

2020311 
2020311 
2020311 
2020311 
2020311 
2020311 
111111

 1703962 
species

1703962 
1703962 
1703962 
1703962 
1703962 
1703962 
143422241313
111111

111111

2020313 
2020313 
2020313 
2020313 
2020313 
2020313 
131217284420
species
 2020313 

111111
 2020312 

2020312 
2020312 
2020312 
2020312 
2020312 
2020312 
868625438524
species

33214204

1703967 
1703967 
1703967 
1703967 
1703967 
1703967 
species
 1703967 
111111

111111

398 
398 
398 
398 
398 
398 
381025475538
species
 398 

species
30818335419

1914541 
1914541 
1914541 
1914541 
1914541 
1914541 
 1914541 
111111

111111
314540217452

1869170 
1869170 
1869170 
1869170 
1869170 
1869170 
species
 1869170 


2048897 
2048897 
2048897 
2048897 
2048897 
2048897 
141851256328
species
 2048897 
111111

species
6850222511833

424182 
424182 
424182 
424182 
424182 
424182 
 424182 
111111

 56730 
species
495564749484

56730 
56730 
56730 
56730 
56730 
56730 
111111

 1703965 
species

1703965 
1703965 
1703965 
1703965 
1703965 
1703965 
15104192121
111111

111111
263635258722

2028343 
2028343 
2028343 
2028343 
2028343 
2028343 
species
 2028343 


1703964 
1703964 
1703964 
1703964 
1703964 
1703964 
251218231722
species
 1703964 
111111

111111
 396 
species

396 
396 
396 
396 
396 
396 
522381385498920474

111111

1703969 
1703969 
1703969 
1703969 
1703969 
1703969 
5271345011135
species
 1703969 

genus
387373253319820393
357
666666

222222
species group
241203179208522274
1183400

 1176649 
species

1176649 
1176649 
1176649 
1176649 
1176649 
1176649 
472629189620
111111

194177150190426254

358 
358 
358 
358 
358 
358 
species
 358 
111111

species

861208 
861208 
861208 
861208 
861208 
861208 
216514398125
 861208 
111111

111111
 373 
species
595117126827

373 
373 
373 
373 
373 
373 

 1842536 
species

1842536 
1842536 
1842536 
1842536 
1842536 
1842536 
18132524595
111111

 359 
species

359 
359 
359 
359 
359 
359 
484118369062
111111

111111
323620
624032738143
genus


879274 
879274 
879274 
879274 
879274 
879274 
624032738143
species
 879274 
111111

666666
28105
14041297968154122741185
genus

111111
species

382 
382 
382 
382 
382 
382 
97694666910461500825
 382 

 1842534 

1842534 
1842534 
1842534 
1842534 
1842534 
1842534 
8271565915047
species
111111

111111
species
362728255117

110321 
110321 
110321 
110321 
110321 
110321 
 110321 

 380 
species

380 
380 
380 
380 
380 
380 
210127127269359204
111111

 794846 
species

794846 
794846 
794846 
794846 
794846 
794846 
166616705521
111111

111111
 194963 
8460727215971

194963 
194963 
194963 
194963 
194963 
194963 
species

106591
11292637617081
genus
222222

111111
species
644739467741

106592 
106592 
106592 
106592 
106592 
106592 
 106592 

111111
species

716925 
716925 
716925 
716925 
716925 
716925 
484524309340
 716925 

232323232323
41294
family
209319771360262831821849

111111
40136
genus
5570466012445

5570466012445

40137 
40137 
40137 
40137 
40137 
40137 
species
 40137 
111111

111111
1649510
41126315130
genus

41126315130

1333996 
1333996 
1333996 
1333996 
1333996 
1333996 
species
 1333996 
111111

85413
197227113255373161
genus
444444

111111
 1792307 
species
552236596563

1792307 
1792307 
1792307 
1792307 
1792307 
1792307 

111111
 2015316 
4243233810231

2015316 
2015316 
2015316 
2015316 
2015316 
2015316 
species

455126659648

1842539 
1842539 
1842539 
1842539 
1842539 
1842539 
species
 1842539 
111111

111111
species
55111289311019

1526658 
1526658 
1526658 
1526658 
1526658 
1526658 
 1526658 

141414141414
374
12081232841162918611196
genus

 1355477 
species
338297231400545369

1355477 
1355477 
1355477 
1355477 
1355477 
1355477 
111111

111111
species
77128568711379

44255 
44255 
44255 
44255 
44255 
44255 
 44255 

 1404367 
5087309011049

1404367 
1404367 
1404367 
1404367 
1404367 
1404367 
species
111111

 1223566 

1223566 
1223566 
1223566 
1223566 
1223566 
1223566 
404246674030
species
111111

species
382932727329

1274631 
1274631 
1274631 
1274631 
1274631 
1274631 
 1274631 
111111

111111

335659 
335659 
335659 
335659 
335659 
335659 
633433104106102
species
 335659 

 376 
345749706254

376 
376 
376 
376 
376 
376 
species
111111

111111
 2057741 
species

2057741 
2057741 
2057741 
2057741 
2057741 
2057741 
76100699910966


115808 
115808 
115808 
115808 
115808 
115808 
77333012010622
species
 115808 
111111

111111
 375 
species

375 
375 
375 
375 
375 
375 
201185127176256107


114615 
114615 
114615 
114615 
114615 
114615 
437650999063
species
 114615 
111111

 1404768 
species
7773257310274

1404768 
1404768 
1404768 
1404768 
1404768 
1404768 
111111

111111
 288000 
6663277773103

288000 
288000 
288000 
288000 
288000 
288000 
species

111111

931866 
931866 
931866 
931866 
931866 
931866 
282836957649
species
 931866 

111111
genus
554415311601717388
1073

species
554415311601717388

1076 
1076 
1076 
1076 
1076 
1076 
 1076 
111111

222222
genus
382143525629
911

111111
species
101229354325

912 
912 
912 
912 
912 
912 
 912 

species
2891417134

913 
913 
913 
913 
913 
913 
 913 
111111

222222
1484898
303318282825
genus

 1384459 
species

1384459 
1384459 
1384459 
1384459 
1384459 
1384459 
242611131714
111111

111111
677151111

2170729 
2170729 
2170729 
2170729 
2170729 
2170729 
species
 2170729 

444444
255475
292307128171743152
family

414371
6270443259138
genus
111111

111111
 1349819 
species
6270443259138

1349819 
1349819 
1349819 
1349819 
1349819 
1349819 

23023784139152114
genus
293088
333333

 1486262 
1096118425330

1486262 
1486262 
1486262 
1486262 
1486262 
1486262 
species
111111

 293089 

293089 
293089 
293089 
293089 
293089 
293089 
896037505362
species
111111

111111
 686597 

686597 
686597 
686597 
686597 
686597 
686597 
3211629474622
species

2036754
family
133135591119575
222222

133135591119575
genus
28209
222222

 1702325 
species
9610744736459

1702325 
1702325 
1702325 
1702325 
1702325 
1702325 
111111

111111
species

444444 
444444 
444444 
444444 
444444 
444444 
372815383116
 444444 

333333
335928
family
191121140195188169

279
genus
382560364338
111111

species
382560364338

280 
280 
280 
280 
280 
280 
 280 
111111

11259368356107
genus
6
111111

species
11259368356107

7 
7 
7 
7 
7 
7 
 7 
111111

413744768924
genus
152053
111111

111111
species
413744768924

921 
921 
921 
921 
921 
921 
 921 

766
1489110796297111
order
251918232414

species

1528098 

1528098 
11
 1528098 
11

241527254567
family
942
8751487

1
33993
genus
1

 950 


950 
1
species
1

332843
13615101733
tribe
952

13615101733
genus
953
332843

1
 246273 


246273 
1
species

111


80849 

80849 
80849 
species
 80849 
111


77038 
77038 
77038 
77038 
77038 
77038 
92103149
species
 77038 
111111

111111
species
1351120

77551 
77551 
77551 
77551 
77551 
77551 
 77551 

111
species
314

169402 


169402 

169402 
 169402 

 163164 
species
1


163164 
1

 100901 
species
11


100901 
100901 
11

 66084 
species
1


66084 
1

342423
943
99713933
genus

342323
species group
99712933
106178

species

779 
779 


779 
134
 779 
111

11


35795 

35795 
species
 35795 
11

 944 
species
6468827

944 
944 
944 
944 
944 
944 
111111


945 
945 
945 
945 
945 
945 
211312
species
 945 
111111

species
1


391036 
 391036 
1

768
genus
252181
21211

 769 
species
11

769 


769 
11

1
106179
species group
1

1


948 
species
 948 
1

1111
15118

770 

770 
770 
770 
species
 770 

1612129167
family
12376797125244
775

1
genus
2115980
1

1
 2115978 


2115978 
1
species

1612119167
33988
tribe
12376787125244

111111
1121528
genus
69474

 784 
1121528

784 
784 
784 
784 
784 
784 
species
111111

1511108156
780
genus
12275765625036

111221
1129742
species group
334137191566

 788 
species


788 
788 
162
11

species
33413731546

33990 
33990 
33990 
33990 
33990 
33990 
 33990 
111111

702220364227
species group
114277
12875104

 35790 
species
92136

35790 
35790 

35790 
35790 
1111

 42862 
species

42862 
42862 
42862 

42862 
4324
1111

11
 786 
11


786 
786 
species

111111
 33992 

33992 
33992 
33992 
33992 
33992 
33992 
1051011023
species

 109232 
species
31

109232 


109232 
11

1
 787 
1

787 
species

 783 
species

783 
783 
783 
783 
783 
783 
22331091
111111

 35794 
1211

35794 
35794 
35794 


35794 
species
1111

22

35791 


35791 
species
 35791 
11

6421

47589 
47589 
47589 

47589 
species
 47589 
1111

11
11

781 

781 
species
 781 

1


226665 
1
species
 226665 

2


35788 
species
 35788 
1

species

33989 


33989 
33989 
33989 
9162
 33989 
1111

111
 35792 
species

35792 


35792 
35792 
2112

species

337479 
337479 
337479 

337479 
16111850
 337479 
1111

311123
species group
114292
111121

 782 
311113

782 
782 
782 
782 
782 
782 
species
111111

1
 785 
species


785 
1

2431034
order
1921002
221322

1
family
2
1777752

1
1521255
2
genus

1
 91604 


91604 
2
species

111111
113723
family
44746

 86106 
113723

86106 
86106 
86106 
86106 
86106 
86106 
species
111111

13111
family
2100208
11111

genus
13111
1509243
11111

11111
13111

1509244 
1509244 

1509244 
1509244 
1509244 
species
 1509244 

54526
order
161
121

121
family
161
1655514

1
5
genus
198251

1
 198252 


198252 
5
species

111
 744985 


744985 
744985 


744985 
111
species

444143434441
204441
195420281254215629701683
order

807766454872974682
family
433
232122232421

444444
genus
11312544160139157
1434011

111111
species

265960 
265960 
265960 
265960 
265960 
265960 
369130185
 265960 

species
19288354943

33995 
33995 
33995 
33995 
33995 
33995 
 33995 
111111

111111
 1177712 
species
13206342942

1177712 
1177712 
1177712 
1177712 
1177712 
1177712 

111111
species

28448 
28448 
28448 
28448 
28448 
28448 
456829614367
 28448 

89583
genus
894864486750
111111

111111
 33996 
species
894864486750

33996 
33996 
33996 
33996 
33996 
33996 

91914
13991176
genus
111111

111111
 91915 
13991176

91915 
91915 
91915 
91915 
91915 
91915 
species

genus
289484714
320496
111111


320497 
320497 
320497 
320497 
320497 
320497 
289484714
species
 320497 
111111

222222
6685384115870
genus
441

111111
 442 

442 
442 
442 
442 
442 
442 
5869331714339
species


318683 
318683 
318683 
318683 
318683 
318683 
8165241531
species
 318683 
111111

111111
153497
genus
271815611342

111111

153496 
153496 
153496 
153496 
153496 
153496 
271815611342
species
 153496 

15112690156201139
genus
364409
111111

 364410 
species

364410 
364410 
364410 
364410 
364410 
364410 
15112690156201139
111111

1111
1602345
1151
genus

 1510841 
species

1510841 


1510841 
1510841 
1510841 
1151
1111

767785
7249741097069
genus
434


438 
438 
438 
438 
438 
438 
352525642813
species
 438 
111111

111111
species

104102 
104102 
104102 
104102 
104102 
104102 
3194104
 104102 

111111
species
94413733

481146 
481146 
481146 
481146 
481146 
481146 
 481146 

151157
16611111812
subgenus
111111

111111

435 
435 
435 
435 
435 
435 
16611111812
species
 435 

1111
 1633874 
species
2111


1633874 
1633874 
1633874 
1633874 

111111
311231147

446692 
446692 
446692 
446692 
446692 
446692 
species
 446692 

5151

1076596 

1076596 
1076596 
1076596 
species
 1076596 
1111

11
11

65959 


65959 
species
 65959 

812924755934
genus
522
222222

111111
 524 

524 
524 
524 
524 
524 
524 
492313392714
species

111111
 62140 
species

62140 
62140 
62140 
62140 
62140 
62140 
32611363220

125216
16626892202208100
genus
222222

111111
6897388111177

257708 
257708 
257708 
257708 
257708 
257708 
species
 257708 

 2018065 
98171541219723

2018065 
2018065 
2018065 
2018065 
2018065 
2018065 
species
111111

41295
family
11471262800128419961001
212021202020

6157252338
genus
1182780
111111

111111
species

1288970 
1288970 
1288970 
1288970 
1288970 
1288970 
6157252338
 1288970 

222222
genus
1238677131173110
1081

species
896142909575

1085 
1085 
1085 
1085 
1085 
1085 
 1085 
111111

111111
 34018 
species
342535417835

34018 
34018 
34018 
34018 
34018 
34018 

5013681578457
genus
171436
111111

 171437 
5013681578457

171437 
171437 
171437 
171437 
171437 
171437 
species
111111

genus
11211
1804663
11111

11111
species

1549855 

1549855 
1549855 
1549855 
1549855 
11211
 1549855 

111111
1543705
861764915115389
genus

 28077 
species
861764915115389

28077 
28077 
28077 
28077 
28077 
28077 
111111

191
genus
5995724156531069531
666666

 682998 
species

682998 
682998 
682998 
682998 
682998 
682998 
618747928437
111111

10277577413255

2202148 
2202148 
2202148 
2202148 
2202148 
2202148 
species
 2202148 
111111

 664962 
species

664962 
664962 
664962 
664962 
664962 
664962 
105587911311791
111111

111111
 192 
species

192 
192 
192 
192 
192 
192 
1601339013223394

111111
 193 
species
10512887136324147

193 
193 
193 
193 
193 
193 


528244 
528244 
528244 
528244 
528244 
528244 
668955106179107
species
 528244 
111111

1612157
15191115256
genus
111111

 1084 

1084 
1084 
1084 
1084 
1084 
1084 
15191115256
species
111111

1543704
301117225320
genus
111111

111111
 1612173 
301117225320

1612173 
1612173 
1612173 
1612173 
1612173 
1612173 
species

genus
488045576718
168934
222222

111111
species
2913711286

2048283 
2048283 
2048283 
2048283 
2048283 
2048283 
 2048283 

111111
 220697 
species
196738463912

220697 
220697 
220697 
220697 
220697 
220697 

1263978
211
genus
111

 1263979 

1263979 
1263979 
1263979 
211
species
111

13134
genus
18716696171348131
444444

111111

55518 
55518 
55518 
55518 
55518 
55518 
7957445411738
species
 55518 

species
334613338022

1663591 
1663591 
1663591 
1663591 
1663591 
1663591 
 1663591 
111111

111111
 84159 
species

84159 
84159 
84159 
84159 
84159 
84159 
322823408215

433516446956

1639348 
1639348 
1639348 
1639348 
1639348 
1639348 
species
 1639348 
111111

111111
241753466914
genus
1632780

111111
 1868589 
241753466914

1868589 
1868589 
1868589 
1868589 
1868589 
1868589 
species

11111
genus
21251
767891

 767892 
21251

767892 
767892 
767892 
767892 

767892 
species
11111

204457
316723761521301133861504
order
676667686867

494849494948
289921001279259829711278
family
41297

111111
genus
601625334010
541

111111
species

542 
542 
542 
542 
542 
542 
601625334010
 542 

444444
genus
189197106187215124
165696

111111
 48935 

48935 
48935 
48935 
48935 
48935 
48935 
228010382415
species

111111
 158500 
species

158500 
158500 
158500 
158500 
158500 
158500 
11162477110940

111111
 702113 
353224675357

702113 
702113 
702113 
702113 
702113 
702113 
species

 205844 

205844 
205844 
205844 
205844 
205844 
205844 
212325112912
species
111111

141414141414
165695
624431257568684321
genus

111111
 120107 
462512234217

120107 
120107 
120107 
120107 
120107 
120107 
species

 332055 
species
375519298033

332055 
332055 
332055 
332055 
332055 
332055 
111111

 627192 
species

627192 
627192 
627192 
627192 
627192 
627192 
34141038338
111111

111111
species
744114302710

1843368 
1843368 
1843368 
1843368 
1843368 
1843368 
 1843368 


76947 
76947 
76947 
76947 
76947 
76947 
1316158276
species
 76947 
111111

111111
species

1332080 
1332080 
1332080 
1332080 
1332080 
1332080 
20151219279
 1332080 

111111
 332056 
species

332056 
332056 
332056 
332056 
332056 
332056 
402829474652

111111
species

46429 
46429 
46429 
46429 
46429 
46429 
3813726489
 46429 

111111
 1855519 
318926144

1855519 
1855519 
1855519 
1855519 
1855519 
1855519 
species

1601467315113976

13690 
13690 
13690 
13690 
13690 
13690 
species
 13690 
111111

111111
 407020 
139412186

407020 
407020 
407020 
407020 
407020 
407020 
species

111111

484429 
484429 
484429 
484429 
484429 
484429 
4523126310014
species
 484429 

111111
 1673076 

1673076 
1673076 
1673076 
1673076 
1673076 
1673076 
411215643465
species

111111

1315974 
1315974 
1315974 
1315974 
1315974 
1315974 
322626324912
species
 1315974 

72173
15228144512
genus
111111

111111
 1634516 

1634516 
1634516 
1634516 
1634516 
1634516 
1634516 
15228144512
species

1649486
542324396526
genus
111111

111111
 1850238 

1850238 
1850238 
1850238 
1850238 
1850238 
1850238 
542324396526
species

141414141414
13687
genus
148996964912621449467

111111
species
65262876779

1609977 
1609977 
1609977 
1609977 
1609977 
1609977 
 1609977 

 1030157 
species

1030157 
1030157 
1030157 
1030157 
1030157 
1030157 
231520373813
111111

111111

745310 
745310 
745310 
745310 
745310 
745310 
35344638419
species
 745310 

species

160791 
160791 
160791 
160791 
160791 
160791 
1579410211915946
 160791 
111111

species
807045687546

1938607 
1938607 
1938607 
1938607 
1938607 
1938607 
 1938607 
111111

 1390395 

1390395 
1390395 
1390395 
1390395 
1390395 
1390395 
131686513213248
species
111111

111111
 1921510 

1921510 
1921510 
1921510 
1921510 
1921510 
1921510 
442815114817
species

 397260 

397260 
397260 
397260 
397260 
397260 
397260 
805350557556
species
111111

127974613712415

1560345 
1560345 
1560345 
1560345 
1560345 
1560345 
species
 1560345 
111111

111111
 1327635 
species
846841578127

1327635 
1327635 
1327635 
1327635 
1327635 
1327635 

111111
 152682 

152682 
152682 
152682 
152682 
152682 
152682 
2571736118222537
species

111111
 93064 
species

93064 
93064 
93064 
93064 
93064 
93064 
717529779260

species
2181075415219137

1549858 
1549858 
1549858 
1549858 
1549858 
1549858 
 1549858 
111111

species

1961362 
1961362 
1961362 
1961362 
1961362 
1961362 
11761471219147
 1961362 
111111

434443
genus
544722552519
1434046


1806885 

1806885 
1806885 
1806885 
1806885 
118653
species
 1806885 
11111

 2077182 

2077182 
2077182 
2077182 
2077182 
2077182 
2077182 
291241269
species
111111

species

1922222 
1922222 
1922222 
1922222 
1922222 
2334144
 1922222 
11111

 266812 
122623107

266812 
266812 
266812 
266812 
266812 
266812 
species
111111

genus
367363179384400257
165697
999999

species
531815323931

1866325 
1866325 
1866325 
1866325 
1866325 
1866325 
 1866325 
111111

 1357916 
species

1357916 
1357916 
1357916 
1357916 
1357916 
1357916 
39273630297
111111

species
1241965913313096

33050 
33050 
33050 
33050 
33050 
33050 
 33050 
111111

 1515612 
species
393517426645

1515612 
1515612 
1515612 
1515612 
1515612 
1515612 
111111

111111
 292913 
3466121711

292913 
292913 
292913 
292913 
292913 
292913 
species

 1913578 
species
312131

1913578 
1913578 
1913578 
1913578 
1913578 
1913578 
111111

 117207 
191513513333

117207 
117207 
117207 
117207 
117207 
117207 
species
111111

 33052 

33052 
33052 
33052 
33052 
33052 
33052 
13179123519
species
111111

111111

267128 
267128 
267128 
267128 
267128 
267128 
434822714814
species
 267128 

111111
genus
47329564842
150203

 1842535 
47329564842

1842535 
1842535 
1842535 
1842535 
1842535 
1842535 
species
111111

181818191919
268276242413415226
family
335929

361177
genus
8573959214857
667777

111111
141613121030

645517 
645517 
645517 
645517 
645517 
645517 
species
 645517 

 1982042 

1982042 
1982042 
1982042 
1982042 
1982042 
1982042 
7131418185
species
111111

 692370 
151717303811

692370 
692370 
692370 
692370 
692370 
692370 
species
111111

111111
 543877 
species
1512819405

543877 
543877 
543877 
543877 
543877 
543877 

 1267766 
1941461

1267766 
1267766 
1267766 
1267766 
1267766 
1267766 
species
111111

111111
species
1511357334

361183 
361183 
361183 
361183 
361183 
361183 
 361183 

7231


476157 
476157 
476157 
476157 
species
 476157 
1111

333333
genus
478731889729
1111

 1112 

1112 
1112 
1112 
1112 
1112 
1112 
22201726155
species
111111

 1896196 
species
96626187

1896196 
1896196 
1896196 
1896196 
1896196 
1896196 
111111

species
16618366417

2023229 
2023229 
2023229 
2023229 
2023229 
2023229 
 2023229 
111111

723734775536
genus
1295327
222222

 450378 

450378 
450378 
450378 
450378 
450378 
450378 
442925624031
species
111111

species
288915155

1348774 
1348774 
1348774 
1348774 
1348774 
1348774 
 1348774 
111111

1041
genus
647982156115104
776777

111111
 39960 
102123465325

39960 
39960 
39960 
39960 
39960 
39960 
species

species

2011159 
2011159 
2011159 
2011159 
2011159 
2011159 
1237472
 2011159 
111111

111111
species

266951 
266951 
266951 
266951 
266951 
266951 
136148415
 266951 

111111
 502682 

502682 
502682 
502682 
502682 
502682 
502682 
6342057628
species

111111
 192812 
151014271826

192812 
192812 
192812 
192812 
192812 
192812 
species

7448153

1648404 
1648404 
1648404 
1648404 
1648404 
1648404 
species
 1648404 
111111

 1922225 

1922225 
1922225 

1922225 
1922225 
1922225 
116125
species
11111

991903
533623475324
genus
111111

111111

991904 
991904 
991904 
991904 
991904 
991904 
533623475324
species
 991904 

255473
order
232246
111111

111111
255474
232246
family

genus
232246
208215
111111

111111
species

208216 
208216 
208216 
208216 
208216 
208216 
232246
 208216 

1191478
order
11341
11111

family
11341
1191479
11111

11341
genus
162171
11111

11111
species


1124597 
1124597 
1124597 
1124597 
1124597 
11341
 1124597 

654634640650638628
232484625752421655651195496116863481590923
class
1236

111111
1775403
19255126223
order

111111
family
19255126223
568386

19255126223
genus
469322
111111


465721 
465721 
465721 
465721 
465721 
465721 
19255126223
species
 465721 
111111

 2169539 
species
2866201421

2169539 
2169539 
2169539 
2169539 
2169539 
2169539 
111111

1273155
genus
6585705224390
111111

6585705224390

585455 
585455 
585455 
585455 
585455 
585455 
species
 585455 
111111

655184
genus
211
211

species
11

1427364 


1427364 
 1427364 
11

 1705394 
11

1705394 
1705394 
species
11

777777
135618
270353128216374266
order

777777
family
270353128216374266
403

444444
416
21425464163288208
genus

 702114 
6880246711286

702114 
702114 
702114 
702114 
702114 
702114 
species
111111

species
20229185612

1538553 
1538553 
1538553 
1538553 
1538553 
1538553 
 1538553 
111111


421 
421 
421 
421 
421 
421 
5023395119
species
 421 
111111

 1727196 
species
7612928696991

1727196 
1727196 
1727196 
1727196 
1727196 
1727196 
111111

111111
39773
genus
1173928252

 271065 

271065 
271065 
271065 
271065 
271065 
271065 
1173928252
species
111111

111111
762296
21102110248
genus

111111
 1704499 

1704499 
1704499 
1704499 
1704499 
1704499 
1704499 
21102110248
species

111111
genus
241634153748
413

111111
species

414 
414 
414 
414 
414 
414 
241634153748
 414 

111111
1934945
order
5710821827534

1934946
5710821827534
family
111111

111111
1934947
5710821827534
genus

111111
 1810504 

1810504 
1810504 
1810504 
1810504 
1810504 
1810504 
5710821827534
species

222222222222
135624
489833952404331135753016
order

222222222222
84642
489833952404331135753016
family

111111
5183366410661
genus
129577

111111
 511062 
species

511062 
511062 
511062 
511062 
511062 
511062 
5183366410661

761323281921
genus
225143
222222

species

1903694 
1903694 
1903694 
1903694 
1903694 
1903694 
2331018108
 1903694 
111111

 1416627 
53101310913

1416627 
1416627 
1416627 
1416627 
1416627 
1416627 
species
111111

43947
genus
291347641243
111111

species
291347641243

43948 
43948 
43948 
43948 
43948 
43948 
 43948 
111111

genus
465132152243309433322811
642
171717171717

111111
species
488226204370286318

654 
654 
654 
654 
654 
654 
 654 

 2033033 
species
824950477329

2033033 
2033033 
2033033 
2033033 
2033033 
2033033 
111111

111111

948519 
948519 
948519 
948519 
948519 
948519 
1014335395243
species
 948519 


645 
645 
645 
645 
645 
645 
736523381410440392
species
 645 
111111


558964 
558964 
558964 
558964 
558964 
558964 
812832385757
species
 558964 
111111

111111
species
848357698234

196024 
196024 
196024 
196024 
196024 
196024 
 196024 

111111
 1920107 
species
1123431443249

1920107 
1920107 
1920107 
1920107 
1920107 
1920107 

111111
species

1758179 
1758179 
1758179 
1758179 
1758179 
1758179 
776314287625
 1758179 

 2033032 
species

2033032 
2033032 
2033032 
2033032 
2033032 
2033032 
200220117168197146
111111

species
522824547163

1636607 
1636607 
1636607 
1636607 
1636607 
1636607 
 1636607 
111111

111111
species

651 
651 
651 
651 
651 
651 
814032764020
 651 

111111
703656768160

1636606 
1636606 
1636606 
1636606 
1636606 
1636606 
species
 1636606 

species
795351488153

1636608 
1636608 
1636608 
1636608 
1636608 
1636608 
 1636608 
111111

111111
 648 
species
2441308212011583

648 
648 
648 
648 
648 
648 

111111

652 
652 
652 
652 
652 
652 
546841448940
species
 652 

species
20411500995137614791336

644 
644 
644 
644 
644 
644 
 644 
111111

111111

1636609 
1636609 
1636609 
1636609 
1636609 
1636609 
699141878163
species
 1636609 

111111
9171556110680
genus
347533

111111
 347534 

347534 
347534 
347534 
347534 
347534 
347534 
9171556110680
species

 2070539 
species
311

2070539 

2070539 


2070539 
111

72274
105865923462881131114172412614589671316174
order
123125125121123114

333535313324
468
family
655642523708606375

687786
552519163620
genus
497

1111
 1720344 
species


1720344 
1720344 
1720344 
1720344 
2313

11
species


571800 
571800 
11
 571800 

111
 1699622 
211


1699622 


1699622 
1699622 
species

111111
 334543 
1113162

334543 
334543 
334543 
334543 
334543 
334543 
species

species
734192

2203895 
2203895 
2203895 
2203895 
2203895 
2203895 
 2203895 
111111

 261164 
species

261164 
5
1


1699624 
1
species
 1699624 
1

 330922 
species

330922 
330922 
330922 
330922 
330922 
330922 
20142743
111111

 349106 
12217


349106 
349106 
349106 
349106 
349106 
species
11111

11
41

45610 


45610 
species
 45610 

111111
1816115

1028416 
1028416 
1028416 
1028416 
1028416 
1028416 
species
 1028416 

232323202114
genus
503460348488426271
469

11111
3517111514

40215 
40215 
40215 
40215 
40215 
species
 40215 

species
1033

487316 
487316 
487316 
 487316 
111


1324350 
1324350 

1324350 

1324350 
3211
species
 1324350 
1111

 62977 
species
11


62977 

62977 
11

555444
909768
282253231316333210
species group

 106654 
20135242314

106654 
106654 
106654 
106654 
106654 
106654 
species
111111

1111
3111

1785128 
1785128 
1785128 


1785128 
species
 1785128 

11111
species

471 
471 
471 
471 
471 
12559
 471 

111111
species

470 
470 
470 
470 
470 
470 
241226211275181187
 470 

17119121208

48296 
48296 
48296 
48296 
48296 
48296 
species
 48296 
111111


2004644 

2004644 
2004644 
2004644 
62152
species
 2004644 
1111

 1789224 
species
11

1789224 
1789224 
11


40216 
40216 
40216 
40216 
40216 
40216 
7620225
species
 40216 
111111

111111
species

108981 
108981 
108981 
108981 
108981 
108981 
4110031511835
 108981 

1111
 1148157 
1411

1148157 
1148157 
1148157 

1148157 
species

111111
27123354

1808001 
1808001 
1808001 
1808001 
1808001 
1808001 
species
 1808001 

species
1314101856

29430 
29430 
29430 
29430 
29430 
29430 
 29430 
111111


1646498 

1646498 
1646498 
1646498 
7132
species
 1646498 
1111

1111
species

106648 
106648 


106648 
106648 
2133
 106648 

species
13125

1758189 
1758189 
1758189 
1758189 
1758189 
 1758189 
11111

 40214 
species
10111121161

40214 
40214 
40214 
40214 
40214 
40214 
111111

513147

2079596 
2079596 
2079596 
2079596 
2079596 
species
 2079596 
11111

11111
 1608473 
species
54211


1608473 
1608473 
1608473 
1608473 
1608473 

 1636603 

1636603 
1636603 
1636603 
1636603 
1636603 
1636603 
221781152
species
111111


1871111 
1871111 
1871111 
1871111 
14214
species
 1871111 
1111

1674953

756892 
756892 
756892 
756892 
756892 
756892 
species
 756892 
111111

1111
 1407071 
1211


1407071 
1407071 
1407071 
1407071 
species

9715715620414484
genus
475
445444

 480 
19183172534

480 
480 
480 
480 
480 
480 
species
111111

111111
species
411211411646835

34062 
34062 
34062 
34062 
34062 
34062 
 34062 

111111
 29433 

29433 
29433 
29433 
29433 
29433 
29433 
315371
species

1
species


476 
1
 476 

 386891 

386891 
386891 
386891 
386891 
386891 
386891 
34176204414
species
111111

909090909090
135621
family
105800423456461130591172341814583611315799

286
genus
105504823416471127970171996414549561312285
878787878787

species
103713390011134248191777512401

2067572 
2067572 
2067572 
2067572 
2067572 
2067572 
 2067572 
111111

111111
 2213057 
650675318628651780

2213057 
2213057 
2213057 
2213057 
2213057 
2213057 
species

 104087 

104087 
104087 
104087 
104087 
104087 
104087 
102062120610757167571358311219
species
111111

111111
 1611770 
species

1611770 
1611770 
1611770 
1611770 
1611770 
1611770 
356960122609443545944231

species

1788301 
1788301 
1788301 
1788301 
1788301 
1788301 
172625851268182317581786
 1788301 
111111

111111
 2049589 
14912009816158013911923

2049589 
2049589 
2049589 
2049589 
2049589 
2049589 
species

 1534110 
species
423163016139674449824158

1534110 
1534110 
1534110 
1534110 
1534110 
1534110 
111111

111111
254831781421251824752629

157782 
157782 
157782 
157782 
157782 
157782 
species
 157782 

136842
477048800541190673726018355910
species group
333333

111111
 47884 
species

47884 
47884 
47884 
47884 
47884 
47884 
149224081267181314861774

species

587753 
587753 
587753 
587753 
587753 
587753 
426127958836867609345439349631
 587753 
111111

111111
 296 

296 
296 
296 
296 
296 
296 
360060093056462543044505
species

species
802919457655768996

237610 
237610 
237610 
237610 
237610 
237610 
 237610 
111111

111111
species
251377107218232197

1931241 
1931241 
1931241 
1931241 
1931241 
1931241 
 1931241 

 1500687 

1500687 
1500687 
1500687 
1500687 
1500687 
1500687 
298551592192387238393059
species
111111

species
292052712835482742883644

1853130 
1853130 
1853130 
1853130 
1853130 
1853130 
 1853130 
111111

136843
4433161251138591275907527711637503680
species group
111111111111

111111
 380021 
species

380021 
380021 
380021 
380021 
380021 
380021 
202023960217770301512666223822

111111
 294 
340608947825467796707298547592379093

294 
294 
294 
294 
294 
294 
species

 47879 

47879 
47879 
47879 
47879 
47879 
47879 
218137341697291526432203
species
111111

species

46679 
46679 
46679 
46679 
46679 
46679 
346583103702570148174105
 46679 
111111

87482731112184176831430712974

76758 
76758 
76758 
76758 
76758 
76758 
species
 76758 
111111

111111
species

47878 
47878 
47878 
47878 
47878 
47878 
281329084233406599574940130839
 47878 

111111
species

75612 
75612 
75612 
75612 
75612 
75612 
340264863237603341724136
 75612 

111111
 200451 
species
77953107413776150771147010086

200451 
200451 
200451 
200451 
200451 
200451 

species

76761 
76761 
76761 
76761 
76761 
76761 
91842554211102169981515911591
 76761 
111111

111111
 200450 
species
113954073615195271152122614090

200450 
200450 
200450 
200450 
200450 
200450 

111111
species
82042967611410185991418810741

47883 
47883 
47883 
47883 
47883 
47883 
 47883 

111111
species

2018067 
2018067 
2018067 
2018067 
2018067 
2018067 
91833762012215217001501111601
 2018067 

241824601010228425112290

1028989 
1028989 
1028989 
1028989 
1028989 
1028989 
species
 1028989 
111111

species

2201356 
2201356 
2201356 
2201356 
2201356 
2201356 
373675883505584647064000
 2201356 
111111

111111
species

1415630 
1415630 
1415630 
1415630 
1415630 
1415630 
103152914912091183441721512228
 1415630 

 198620 

198620 
198620 
198620 
198620 
198620 
198620 
124112128023904287081482612129
species
111111

111111
species

244566 
244566 
244566 
244566 
244566 
244566 
106693180613997214911760312824
 244566 

111111
species
198224421064225821822852

237609 
237609 
237609 
237609 
237609 
237609 
 237609 

111111
species
297858122332399938263435

1500686 
1500686 
1500686 
1500686 
1500686 
1500686 
 1500686 

111111
439078373751594253024509

1283291 
1283291 
1283291 
1283291 
1283291 
1283291 
species
 1283291 

 216142 
species

216142 
216142 
216142 
216142 
216142 
216142 
276525481756293925674328
111111

species

930166 
930166 
930166 
930166 
930166 
930166 
154382769613210223111913721040
 930166 
111111

111111
 2083055 

2083055 
2083055 
2083055 
2083055 
2083055 
2083055 
95793075911467198481666811218
species

111111
 515393 

515393 
515393 
515393 
515393 
515393 
515393 
114533940911784259232230413401
species

111111
 312306 

312306 
312306 
312306 
312306 
312306 
312306 
14692273999170019971834
species

11281595650120911501491

2069256 
2069256 
2069256 
2069256 
2069256 
2069256 
species
 2069256 
111111

111111

157783 
157783 
157783 
157783 
157783 
157783 
13151662845175313201933
species
 157783 

 2083052 

2083052 
2083052 
2083052 
2083052 
2083052 
2083052 
12311403722126312831324
species
111111

666666
136845
829401261425649410490996036105970
species group

species
13151851781145614891779

70775 
70775 
70775 
70775 
70775 
70775 
 70775 
111111

612181793737690666637429

76759 
76759 
76759 
76759 
76759 
76759 
species
 76759 
111111

111111
522583563785694261166855

78327 
78327 
78327 
78327 
78327 
78327 
species
 78327 

 303 
species
6730710418646605866577881386348

303 
303 
303 
303 
303 
303 
111111

111111

47880 
47880 
47880 
47880 
47880 
47880 
219528891266225522312579
species
 47880 


47885 
47885 
47885 
47885 
47885 
47885 
777681320693724980
species
 47885 
111111

species group
11942118847485485146751147526136964
136841
777777

111111
species
11801428625118114291260

53412 
53412 
53412 
53412 
53412 
53412 
 53412 

111111
 53408 
species
264236771651317831083137

53408 
53408 
53408 
53408 
53408 
53408 

111111

43263 
43263 
43263 
43263 
43263 
43263 
921120559510129351275
species
 43263 

111111
 287 
species
10729417172078030132911133690122825

287 
287 
287 
287 
287 
287 

 300 

300 
300 
300 
300 
300 
300 
511270053055565557425506
species
111111

222222
1232139
227234391529281426222961
species subgroup

111111
species

330 
330 
330 
330 
330 
330 
15022233964200215822144
 330 

77012065658121040817

1149133 
1149133 
1149133 
1149133 
1149133 
1149133 
species
 1149133 
111111

 101564 
species

101564 
101564 
101564 
101564 
101564 
101564 
750885443877859863
111111

111111
 2083051 
species
10131802679149112651439

2083051 
2083051 
2083051 
2083051 
2083051 
2083051 

 1338689 

1338689 
1338689 
1338689 
1338689 
1338689 
1338689 
8871502815132110561120
species
111111

species

1207075 
1207075 
1207075 
1207075 
1207075 
1207075 
334753123084504140233275
 1207075 
111111

species

1636610 
1636610 
1636610 
1636610 
1636610 
1636610 
14311983993210118072355
 1636610 
111111

111111

1659194 
1659194 
1659194 
1659194 
1659194 
1659194 
314866803430565142063764
species
 1659194 

111111
903115050811228941101

1930532 
1930532 
1930532 
1930532 
1930532 
1930532 
species
 1930532 

111111
 2219057 
species
94723045912837197261675911383

2219057 
2219057 
2219057 
2219057 
2219057 
2219057 

111111
397558204478354435

1981174 
1981174 
1981174 
1981174 
1981174 
1981174 
species
 1981174 

 219572 

219572 
219572 
219572 
219572 
219572 
219572 
219068335224888483363745227550
species
111111

111111
species
14712130981178418792176

1259844 
1259844 
1259844 
1259844 
1259844 
1259844 
 1259844 


1583341 
1583341 
1583341 
1583341 
1583341 
1583341 
70245897125254087459826796
species
 1583341 
111111

222222
10592119445435103671151212622
species group
136846

1004811421517798881106411987
species subgroup
578833
111111

 316 

316 
316 
316 
316 
316 
316 
1004811421517798881106411987
species
111111

111111
 74829 

74829 
74829 
74829 
74829 
74829 
74829 
544523258479448635
species

 65741 

65741 
65741 
65741 
65741 
65741 
65741 
997127052596110451124
species
111111

111111
 2083053 
12771525717146512931673

2083053 
2083053 
2083053 
2083053 
2083053 
2083053 
species

 2083054 
56941690969741306787567189

2083054 
2083054 
2083054 
2083054 
2083054 
2083054 
species
111111

species

1649877 
1649877 
1649877 
1649877 
1649877 
1649877 
169324431345201620072011
 1649877 
111111

 1898684 
94111885098091040908

1898684 
1898684 
1898684 
1898684 
1898684 
1898684 
species
111111

species group
13328513622610843993949114723219083
136849
888888

251695
106392108692951577329790080192373
species subgroup
111111

111111
 317 
species
106392108692951577329790080192373

317 
317 
317 
317 
317 
317 

111111
285231411018209128002169

36746 
36746 
36746 
36746 
36746 
36746 
species
 36746 

111111
337334081507272130542979

33069 
33069 
33069 
33069 
33069 
33069 
species
 33069 

species

1206777 
1206777 
1206777 
1206777 
1206777 
1206777 
157819351253152318221777
 1206777 
111111

222222
251698
species subgroup
803080854417601876148536

species

47877 
47877 
47877 
47877 
47877 
47877 
400638622060281536584334
 47877 
111111

111111
 29438 
species
402442232357320339564202

29438 
29438 
29438 
29438 
29438 
29438 

111111

46257 
46257 
46257 
46257 
46257 
46257 
26012267948201022292608
species
 46257 

111111
species
845986984139628971248641

251701 
251701 
251701 
251701 
251701 
251701 
 251701 

111111
 69328 
11171531705145813851398

69328 
69328 
69328 
69328 
69328 
69328 
species

111111
species

1827300 
1827300 
1827300 
1827300 
1827300 
1827300 
8464297701106418869147619917
 1827300 

111111
 1294143 
9491468591108210361046

1294143 
1294143 
1294143 
1294143 
1294143 
1294143 
species


1856685 
1856685 
1856685 
1856685 
1856685 
1856685 
6999734809539201073
species
 1856685 
111111

111111
713339593658
genus
1849530

111111
species

1697053 
1697053 
1697053 
1697053 
1697053 
1697053 
713339593658
 1697053 

351
subfamily
288539662582339533693456
222222

352
genus
288539662582339533693456
222222

111111
 354 
171926621852198720472029

354 
354 
354 
354 
354 
354 
species

111111

353 
353 
353 
353 
353 
353 
11661304730140813221427
species
 353 

135615
1
order
1

868
family
1
1

1
1
genus
869

 870 
species
1


870 
1

genus
4420724
1608298
111111

 1076588 
species
4420724

1076588 
1076588 
1076588 
1076588 
1076588 
1076588 
111111

111111
genus
141178305
1524249

111111
 1249552 

1249552 
1249552 
1249552 
1249552 
1249552 
1249552 
141178305
species

1
species


410330 
2
 410330 

856763
85737114215577
order
118969

645552
444
65675611113064
family

544452
445
63675511013064
genus

11
 450 
species
11

450 


450 

1
 28087 


28087 
2
species

1
species


96230 
4
 96230 


452 


452 
110
species
 452 
11

 1867846 
species

1867846 

1867846 
11
11

 449 
species
1231

449 

449 
449 
449 
1111

species
1


2005262 
 2005262 
1

 29423 

29423 
29423 
29423 
29423 
29423 
102143
species
11111

species

446 
446 
446 
446 
446 
446 
5062519911563
 446 
111111

465
genus
211
111

111
species
211

451 

451 
451 
 451 

211211
family
20615312513
118968

211211
776
genus
20615312513

11
species
11

325775 


325775 
 325775 

111111

777 
777 
777 
777 
777 
777 
19615302513
species
 777 

373535373736
135623
order
302116071583198412921021

373535373736
302116071583198412921021
family
641

246861
53312219276
genus
111111

111111
 673 
species
53312219276

673 
673 
673 
673 
673 
673 

11
2042066
11
genus

11
 1755811 
species
11


1755811 

1755811 

233333
657
genus
452157503744

111111
 1295392 

1295392 
1295392 
1295392 
1295392 
1295392 
1295392 
21629292423
species

species


74109 
74109 
74109 
74109 
74109 
23335
 74109 
11111

111111
 38293 

38293 
38293 
38293 
38293 
38293 
38293 
241325181016
species

genus
1810814207
511678
333223

 80852 
species
13211

80852 
80852 
80852 

80852 
80852 
11111

11111
 40269 
91412

40269 
40269 
40269 
40269 

40269 
species

111111
 668 
86213194

668 
668 
668 
668 
668 
668 
species

312827313029
662
genus
29051545149519011207964

111111
 52443 
1335718619

52443 
52443 
52443 
52443 
52443 
52443 
species

111111
 2014742 
species

2014742 
2014742 
2014742 
2014742 
2014742 
2014742 
29861795

species

190893 
190893 
190893 
190893 
190893 
190893 
65728133213
 190893 
111111

111
 2025808 
species
1011

2025808 
2025808 


2025808 

111111

672 
672 
672 
672 
672 
672 
30684163312110123
species
 672 


55601 
55601 
55601 
55601 
55601 
55601 
1571141021087488
species
 55601 
111111

11111
species group
2166136
1891919

11111
 29498 
2166136


29498 
29498 
29498 
29498 
29498 
species

109910910
species group
804288374459432254
717610

111111
species
299111958

696485 
696485 
696485 
696485 
696485 
696485 
 696485 


663 
663 
663 
663 
663 
663 
1385186766755
species
 663 
111111

11111
21322

766224 
766224 

766224 
766224 
766224 
species
 766224 

 669 

669 
669 
669 
669 
669 
669 
855848654430
species
111111

111111
 190895 

190895 
190895 
190895 
190895 
190895 
190895 
25113106
species

 691 
species

691 
691 
691 
691 
691 
691 
872435545618
111111

111111
 670 

670 
670 
670 
670 
670 
670 
31910111516916473
species

111111
species
3682611164

50719 
50719 
50719 
50719 
50719 
50719 
 50719 

1111
species

150340 

150340 
150340 

150340 
13525
 150340 

 680 
species

680 
680 
680 
680 
680 
680 
933137576853
111111

111111
species

1534743 
1534743 
1534743 
1534743 
1534743 
1534743 
2016312173
 1534743 

species

553239 


553239 
76
 553239 
11

11111
species
51144

689 

689 
689 
689 
689 
 689 

111111
1042834571721394366

666 
666 
666 
666 
666 
666 
species
 666 


687 
687 
687 
687 
687 
687 
25423254
species
 687 
111111

111111

28173 
28173 
28173 
28173 
28173 
28173 
653312926
species
 28173 

111111
species

1435069 
1435069 
1435069 
1435069 
1435069 
1435069 
321013724
 1435069 

 674 
102111196

674 
674 
674 
674 
674 
674 
species
111111

species

76258 
76258 

76258 
76258 
76258 
141012
 76258 
11111


1116375 
1116375 
1116375 
1116375 
1116375 
1116375 
582821364
species
 1116375 
111111

111
 212663 
species

212663 


212663 
212663 
191

111111
species

29494 
29494 
29494 
29494 
29494 
29494 
52827211712
 29494 

species

676 
676 
676 
676 
676 
676 
183551171095937
 676 
111111

111111
 673372 
species
14641714

673372 
673372 
673372 
673372 
673372 
673372 

661520124

45658 
45658 
45658 
45658 
45658 
45658 
species
 45658 
111111

 83406 
species

83406 
83406 
83406 
83406 
83406 
83406 
732423211
111111

species


113267 

113267 

113267 
213
 113267 
111

91347
order
982442181466364524200687157480177510
203197200201198202

family
17452811555138
1903416
222222

111111
11029491012722
genus
82980

11029491012722

158841 
158841 
158841 
158841 
158841 
158841 
species
 158841 
111111

82984
genus
642332542416
111111

111111
 82985 

82985 
82985 
82985 
82985 
82985 
82985 
642332542416
species

111111
702
genus
212150861378761

 703 

703 
703 
703 
703 
703 
703 
212150861378761
species
111111

232121212221
1903409
family
596306102519173482417512658098657

53335
genus
48860317921191166361604034000
121212111212

111111
1085167276145132243

66269 
66269 
66269 
66269 
66269 
66269 
species
 66269 

111111
 665914 
species

665914 
665914 
665914 
665914 
665914 
665914 
870266371189210254

111111
species
760143262181151152

1891675 
1891675 
1891675 
1891675 
1891675 
1891675 
 1891675 

111111
 470934 
species

470934 
470934 
470934 
470934 
470934 
470934 
195447727490962359238219379


1235990 
1235990 
1235990 

1235990 
1235990 
31327
species
 1235990 
11111

 592316 
1340297450287178295

592316 
592316 
592316 
592316 
592316 
592316 
species
111111

species

665913 
665913 
665913 
665913 
665913 
665913 
669189336186169265
 665913 
111111

species group
270833763949861255127110962
1654067
111111

species

549 
549 
549 
549 
549 
549 
270833763949861255127110962
 549 
111111

 553 
141341314218811889911789

553 
553 
553 
553 
553 
553 
species
111111

 1484157 

1484157 
1484157 
1484157 
1484157 
1484157 
1484157 
891215354195187185
species
111111

111111
 1076550 
species
1344250496210209258

1076550 
1076550 
1076550 
1076550 
1076550 
1076550 

111111
 1484158 
species

1484158 
1484158 
1484158 
1484158 
1484158 
1484158 
1227166298166158211

111111
genus
13821281794
32199

 9 

9 
9 
9 
9 
9 
9 
13821281794
species
111111

766776
10692484478154069351022039564418
genus
551

 215689 
species
568188346100184165

215689 
215689 
215689 
215689 
215689 
215689 
111111


1922217 


1922217 
1922217 
131
species
 1922217 
111

111111
 1619313 
1387201333215187227

1619313 
1619313 
1619313 
1619313 
1619313 
1619313 
species

111111
 79967 

79967 
79967 
79967 
79967 
79967 
79967 
16045611103467452538
species


182337 
182337 
182337 
182337 
182337 
182337 
10054682625150620334401874862660
species
 182337 
111111

111111
19796991257643617592

552 
552 
552 
552 
552 
552 
species
 552 

111111
species
839204410234206236

338565 
338565 
338565 
338565 
338565 
338565 
 338565 

genus
763112276260128145
82986
222222

111111
2693985724135

82987 
82987 
82987 
82987 
82987 
82987 
species
 82987 

111111
species

53336 
53336 
53336 
53336 
53336 
53336 
4947319118887110
 53336 

3
genus
51228
1

1
 51229 
species
3

51229 

161615161515
1903414
family
367777917371581959757

111111
581
1858478864832557358
genus

111111
1858478864832557358

582 
582 
582 
582 
582 
582 
species
 582 

genus
31925111825057
29487
333333

 291112 
761281167

291112 
291112 
291112 
291112 
291112 
291112 
species
111111

111111
1691552482627

2218628 
2218628 
2218628 
2218628 
2218628 
2218628 
species
 2218628 

111111
 230089 
species
74931231823

230089 
230089 
230089 
230089 
230089 
230089 

222222
583
genus
752108335304129159

 585 
species
2821494

585 
585 
585 
585 
585 
585 
111111

111111
 584 
724106334300120155

584 
584 
584 
584 
584 
584 
species

444444
586
3818624922214993
genus

5562517124

126385 
126385 
126385 
126385 
126385 
126385 
species
 126385 
111111

111111
 588 
species

588 
588 
588 
588 
588 
588 
1946613715310352

111111
 587 
1141170432021

587 
587 
587 
587 
587 
587 
species

 333962 

333962 
333962 
333962 
333962 
333962 
333962 
1831791416
species
111111

111
genus
637
111

111

634113 
634113 

634113 
species
 634113 
111

555555
366811781407490
genus
626

111111
species

351679 
351679 
351679 
351679 
351679 
351679 
1753430735
 351679 

111111
 351671 
species
50162124163

351671 
351671 
351671 
351671 
351671 
351671 


40577 
40577 
40577 
40577 
40577 
40577 
661137301415
species
 40577 
111111

species

40576 
40576 
40576 
40576 
40576 
40576 
2114562382325
 40576 
111111

111111
species

628 
628 
628 
628 
628 
628 
22424181412
 628 

99961009997101
2171715146890898732575847747770
family
543

111
genus
7162
1048757

7162

1048758 


1048758 

1048758 
species
 1048758 
111

11111
genus
21152
1682492

 1410383 
species
21152


1410383 
1410383 
1410383 
1410383 
1410383 
11111

111111
 891974 

891974 
891974 
891974 
891974 
891974 
891974 
1440211307212175214
species

444444
1330547
genus
346263113991161754575


283686 
283686 
283686 
283686 
283686 
283686 
1283185497454279216
species
 283686 
111111

111111
 208223 
species
5539417616085101

208223 
208223 
208223 
208223 
208223 
208223 

111111
 497725 
species

497725 
497725 
497725 
497725 
497725 
497725 
557110234168152107

111111
 1158459 
species

1158459 
1158459 
1158459 
1158459 
1158459 
1158459 
1069242492379238151

species
3647613520410370

2066051 
2066051 
2066051 
2066051 
2066051 
2066051 
 2066051 
111111

25950988411859
genus
1335483
111111

111111
species
25950988411859

563 
563 
563 
563 
563 
563 
 563 

1


1835721 
1
species
 1835721 

2136411933938597376
genus
1330545
444444

111111
44611520124114597

2153385 
2153385 
2153385 
2153385 
2153385 
2153385 
species
 2153385 

111111
 69220 

69220 
69220 
69220 
69220 
69220 
69220 
4224513113611086
species

111111
species
3818321922711558

1907578 
1907578 
1907578 
1907578 
1907578 
1907578 
 1907578 

111111

61646 
61646 
61646 
61646 
61646 
61646 
887168382334227135
species
 61646 

222222
1330546
genus
1676550720672648492


61647 
61647 
61647 
61647 
61647 
61647 
778341389356392297
species
 61647 
111111

111111
species
898209331316256195

1334193 
1334193 
1334193 
1334193 
1334193 
1334193 
 1334193 

111
genus
336
409304

 168169 
species

168169 


168169 
168169 
336
111

1906657
genus
232211
11111

 1778262 
species

1778262 

1778262 
1778262 
1778262 
1778262 
232211
11111

11211
genus
11212
203804

11
11

251535 


251535 
species
 251535 

1
 1505597 


1505597 
1
species

species


101534 

101534 
11
 101534 
11

1
species
2


251542 
 251542 

570
genus
645891879527535224101980916517
999999

 1463165 

1463165 
1463165 
1463165 
1463165 
1463165 
1463165 
2325799978963740588
species
111111

 1934254 
species
31773135120102101

1934254 
1934254 
1934254 
1934254 
1934254 
1934254 
111111

 571 
species

571 
571 
571 
571 
571 
571 
542412402477185116171284
111111

111111
 244366 
species
23288251081822784634

244366 
244366 
244366 
244366 
244366 
244366 

111111
 1134687 

1134687 
1134687 
1134687 
1134687 
1134687 
1134687 
364369214761116971666
species

species

573 
573 
573 
573 
573 
573 
452421374519302156111408712195
 573 
111111

 1905288 
37311215412812573

1905288 
1905288 
1905288 
1905288 
1905288 
1905288 
species
111111

111111
species

548 
548 
548 
548 
548 
548 
47861258181317181244916
 548 

111111
 2026240 
species
151511198113960

2026240 
2026240 
2026240 
2026240 
2026240 
2026240 

1906659
2511
genus
1111

1111
 1778263 

1778263 

1778263 
1778263 

1778263 
2511
species

111111
 1920128 

1920128 
1920128 
1920128 
1920128 
1920128 
1920128 
3448621720211883
species

1138025405244445831292294
genus
544
171717171717

111111
 1702170 
28156123607637

1702170 
1702170 
1702170 
1702170 
1702170 
1702170 
species

 2013114 

2013114 
2013114 
2013114 
2013114 
2013114 
2013114 
2517616310610587
species
111111

 67824 
species
369471241226642

67824 
67824 
67824 
67824 
67824 
67824 
111111

111111
 1703250 
254371021028265

1703250 
1703250 
1703250 
1703250 
1703250 
1703250 
species

 67825 
species
3757012712312483

67825 
67825 
67825 
67825 
67825 
67825 
111111

 35703 
species
844257472381249173

35703 
35703 
35703 
35703 
35703 
35703 
111111

111111
 545 

545 
545 
545 
545 
545 
545 
1846319791623478346
species

111111
species
30525971209174

2019568 
2019568 
2019568 
2019568 
2019568 
2019568 
 2019568 

888888
1344959
653416053142271318201347
species group

 67827 
species
5869625921814294

67827 
67827 
67827 
67827 
67827 
67827 
111111

111111

546 
546 
546 
546 
546 
546 
41531042199217621174870
species
 546 

18633115646036

2077149 
2077149 
2077149 
2077149 
2077149 
2077149 
species
 2077149 
111111

 2077148 
19315215113010170

2077148 
2077148 
2077148 
2077148 
2077148 
2077148 
species
111111

111111
620134270221150121

57706 
57706 
57706 
57706 
57706 
57706 
species
 57706 

111111
 133448 

133448 
133448 
133448 
133448 
133448 
133448 
298671281235259
species

111111
 2066049 

2066049 
2066049 
2066049 
2066049 
2066049 
2066049 
292301291219648
species

111111
2065198744549

2077147 
2077147 
2077147 
2077147 
2077147 
2077147 
species
 2077147 

321481031083840

1920110 
1920110 
1920110 
1920110 
1920110 
1920110 
species
 1920110 
111111

species
3094511011910745

1920109 
1920109 
1920109 
1920109 
1920109 
1920109 
 1920109 
111111


2052938 
2052938 
2052938 
2052938 
2052938 
2052938 
1624057633647
species
 2052938 
111111

613512282448213715781180
genus
413496
777777

species

413503 
413503 
413503 
413503 
413503 
413503 
1071205404324225166
 413503 
111111

111111
 535744 

535744 
535744 
535744 
535744 
535744 
535744 
690135198174155103
species

 1163710 

1163710 
1163710 
1163710 
1163710 
1163710 
1163710 
346851371308663
species
111111

111111
 28141 
species

28141 
28141 
28141 
28141 
28141 
28141 
250452110661002681497

111111
43174170157155119

413502 
413502 
413502 
413502 
413502 
413502 
species
 413502 

111111

413497 
413497 
413497 
413497 
413497 
413497 
599115277177108107
species
 413497 

111111

413501 
413501 
413501 
413501 
413501 
413501 
49493196173168125
species
 413501 

561
4538399101927015248114469708
genus
444444

species

564 
564 
564 
564 
564 
564 
531216251215
 564 
111111

111111

1499973 
1499973 
1499973 
1499973 
1499973 
1499973 
1061796474036
species
 1499973 

 208962 
species
1146157424397254167

208962 
208962 
208962 
208962 
208962 
208962 
111111

111111
 562 
species

562 
562 
562 
562 
562 
562 
4407897241873414779111409490

genus
111
1906661
111

species
111


1070130 
1070130 


1070130 
 1070130 
111

genus
347026965139351110784466680
547
242424242424

species
430661391057775

1692238 
1692238 
1692238 
1692238 
1692238 
1692238 
 1692238 
111111

 1166130 
species

1166130 
1166130 
1166130 
1166130 
1166130 
1166130 
51310316716310383
111111

111111
species
408521351419762

1868135 
1868135 
1868135 
1868135 
1868135 
1868135 
 1868135 

111111
species
1283192857626

2051905 
2051905 
2051905 
2051905 
2051905 
2051905 
 2051905 

354276
30573618712366973074355942
species group
141414141414

111111
species
287691131048254

1870930 
1870930 
1870930 
1870930 
1870930 
1870930 
 1870930 

111111
species
1984363769655366368

61645 
61645 
61645 
61645 
61645 
61645 
 61645 

111111
species

2077136 
2077136 
2077136 
2077136 
2077136 
2077136 
4049015714811779
 2077136 

111111
species

1915310 
1915310 
1915310 
1915310 
1915310 
1915310 
4739414812611357
 1915310 

 1296536 
43010520216810684

1296536 
1296536 
1296536 
1296536 
1296536 
1296536 
species
111111

111111
species

1812935 
1812935 
1812935 
1812935 
1812935 
1812935 
38010116218513375
 1812935 

species

2027919 
2027919 
2027919 
2027919 
2027919 
2027919 
717127265243196105
 2027919 
111111

111111
 158836 
399278115571332957820

158836 
158836 
158836 
158836 
158836 
158836 
species

species
1906440098020588046683691

550 
550 
550 
550 
550 
550 
 550 
111111

species
1243229423422346285

1812934 
1812934 
1812934 
1812934 
1812934 
1812934 
 1812934 
111111

111111
species

2077137 
2077137 
2077137 
2077137 
2077137 
2077137 
349421321016568
 2077137 

species

69218 
69218 
69218 
69218 
69218 
69218 
4426716611585105
 69218 
111111

111111
 208224 
species

208224 
208224 
208224 
208224 
208224 
208224 
383441361349279

111111
 299767 
species
4256611611710972

299767 
299767 
299767 
299767 
299767 
299767 

species

399742 
399742 
399742 
399742 
399742 
399742 
35895232146141101
 399742 
111111

species
315791211288879

1977566 
1977566 
1977566 
1977566 
1977566 
1977566 
 1977566 
111111

 881260 

881260 
881260 
881260 
881260 
881260 
881260 
4521031621598690
species
111111

111111
 1560339 
species
4017216510910072

1560339 
1560339 
1560339 
1560339 
1560339 
1560339 

111111
 1914861 

1914861 
1914861 
1914861 
1914861 
1914861 
1914861 
856112243219174115
species

 1827481 
species
268651131226935

1827481 
1827481 
1827481 
1827481 
1827481 
1827481 
111111

111111
158483
23124061780952645682
genus

111111
 158822 
23124061780952645682

158822 
158822 
158822 
158822 
158822 
158822 
species

111111
316621951329447

693444 
693444 
693444 
693444 
693444 
693444 
species
 693444 

111111
8953612
genus
568987

8953612

138072 
138072 
138072 
138072 
138072 
138072 
species
 138072 
111111

11111
 1199245 
91331

1199245 
1199245 

1199245 
1199245 
1199245 
species

111111
2172100
921119461316
genus

111111
 2172103 
921119461316

2172103 
2172103 
2172103 
2172103 
2172103 
2172103 
species

355258047142471085689377544
genus
590
222222

111111
 54736 

54736 
54736 
54736 
54736 
54736 
54736 
54411720820713699
species

349817930140391064988017445

28901 
28901 
28901 
28901 
28901 
28901 
species
 28901 
111111

11
genus
21
401618

21


401619 


401619 
species
 401619 
11

223222
32922635441
genus
620

1111
 623 
2233


623 
623 
623 
623 
species

111111
 622 
species

622 
622 
622 
622 
622 
622 
20819615138

 1813821 
1211

1813821 
1813821 
1813821 
species
111

222222
160674
genus
432783513821359998712

111111
601751661908686

575 
575 
575 
575 
575 
575 
species
 575 

 54291 

54291 
54291 
54291 
54291 
54291 
54291 
372676012161169912626
species
111111

333333
83654
1486314505491414221
genus

111111
 1920114 
species
45210317512713088

1920114 
1920114 
1920114 
1920114 
1920114 
1920114 

111111
 83655 
species

83655 
83655 
83655 
83655 
83655 
83655 
4259416517812168

60911716518616365

1920116 
1920116 
1920116 
1920116 
1920116 
1920116 
species
 1920116 
111111

694223312293237124
genus
929812
111111


929813 
929813 
929813 
929813 
929813 
929813 
694223312293237124
species
 929813 
111111

 134287 

134287 
134287 
22
species
11

12892214
genus
1906660
111111

species
12892214

1778264 
1778264 
1778264 
1778264 
1778264 
1778264 
 1778264 
111111

1903411
family
1466902276890448760706553926656
333333333333

111111
genus
2593916217511956
1745211

2593916217511956

1639108 
1639108 
1639108 
1639108 
1639108 
1639108 
species
 1639108 
111111

20456291113063897055334277
genus
629
131313131313

 630 

630 
630 
630 
630 
630 
630 
55667714416252114831425
species
111111

111111
 1839800 
13321108984819

1839800 
1839800 
1839800 
1839800 
1839800 
1839800 
species

111111
species
1316250704728405235

29486 
29486 
29486 
29486 
29486 
29486 
 29486 

111111
252491548711757

263819 
263819 
263819 
263819 
263819 
263819 
species
 263819 

333333
1649845
1045915175145443427541944
species group


633 
633 
633 
633 
633 
633 
1848375884686486412
species
 633 
111111

species

632 
632 
632 
632 
632 
632 
849011124195367822291502
 632 
111111

111111
1213066703930

367190 
367190 
367190 
367190 
367190 
367190 
species
 367190 

922981015310219235

29484 
29484 
29484 
29484 
29484 
29484 
species
 29484 
111111

 419257 

419257 
419257 
419257 
419257 
419257 
419257 
1804818110012156
species
111111

111111
 29485 

29485 
29485 
29485 
29485 
29485 
29485 
181241611426035
species

111111
 28152 
1672570510663155

28152 
28152 
28152 
28152 
28152 
28152 
species

species
10168333534619583

631 
631 
631 
631 
631 
631 
 631 
111111

111111
 29483 
26425139986833

29483 
29483 
29483 
29483 
29483 
29483 
species

34037
4989541993885029255125128010
genus
222222

111111
 741091 
species

741091 
741091 
741091 
741091 
741091 
741091 
258582103191911470563943948

 34038 
species
240372096196591455061184062

34038 
34038 
34038 
34038 
34038 
34038 
111111

111111
genus
201001382131641180345732072
1565532

111111
201001382131641180345732072

1805933 
1805933 
1805933 
1805933 
1805933 
1805933 
species
 1805933 

161616161616
genus
559801423725209258674280212241
613

111111
1973476860800942347

47917 
47917 
47917 
47917 
47917 
47917 
species
 47917 

111111
 2033438 

2033438 
2033438 
2033438 
2033438 
2033438 
2033438 
1072164329356321142
species

species
2694432281433764653477

614 
614 
614 
614 
614 
614 
 614 
111111

species

1327989 
1327989 
1327989 
1327989 
1327989 
1327989 
468100233210257106
 1327989 
111111

111111
 61651 
species

61651 
61651 
61651 
61651 
61651 
61651 
928272420397617202

111111
42415321327256794

768493 
768493 
768493 
768493 
768493 
768493 
species
 768493 

111111
species

61652 
61652 
61652 
61652 
61652 
61652 
1399424724652574326
 61652 

111111
13836843233604998131

28151 
28151 
28151 
28151 
28151 
28151 
species
 28151 

111111
 615 
species

615 
615 
615 
615 
615 
615 
3451691111456814701196348264

111111
355561741609369

104623 
104623 
104623 
104623 
104623 
104623 
species
 104623 

111111
 82996 
species

82996 
82996 
82996 
82996 
82996 
82996 
814815483463334285021401

111111

671990 
671990 
671990 
671990 
671990 
671990 
707273299282286138
species
 671990 

 488142 
species

488142 
488142 
488142 
488142 
488142 
488142 
40580135224248135
111111

 1758196 
507107205194250166

1758196 
1758196 
1758196 
1758196 
1758196 
1758196 
species
111111

 768490 
466168236297638142

768490 
768490 
768490 
768490 
768490 
768490 
species
111111

111111
 1759437 
species
535189213244222101

1759437 
1759437 
1759437 
1759437 
1759437 
1759437 

661189341332156147
genus
447792
222222


1756993 
1756993 
1756993 
1756993 
1756993 
1756993 
3001081801698055
species
 1756993 
111111

111111

1972431 
1972431 
1972431 
1972431 
1972431 
1972431 
361811611637692
species
 1972431 

4201815186218371701850
family
1903412
111111111111

genus
2189576876897774554
635
777777

111111
species

1263550 
1263550 
1263550 
1263550 
1263550 
1263550 
3667920512616688
 1263550 


636 
636 
636 
636 
636 
636 
513138226277204146
species
 636 
111111

111111
 67780 

67780 
67780 
67780 
67780 
67780 
67780 
466137220166179133
species

species

93378 
93378 
93378 
93378 
93378 
93378 
109628303310
 93378 
111111

 1821960 

1821960 
1821960 
1821960 
1821960 
1821960 
1821960 
174102671076231
species
111111

111111
species

1650654 
1650654 
1650654 
1650654 
1650654 
1650654 
30356641224968
 1650654 

111111
 1578828 
species
2585866698178

1578828 
1578828 
1578828 
1578828 
1578828 
1578828 

333333
1680189839838823238
genus
568

111111
species

546367 
546367 
546367 
546367 
546367 
546367 
304321592069749
 546367 

species

569 
569 
569 
569 
569 
569 
1100124530491577147
 569 
111111

species
2763315014114942

1848580 
1848580 
1848580 
1848580 
1848580 
1848580 
 1848580 
111111

111111
82982
genus
3325014710210458

species

82983 
82983 
82983 
82983 
82983 
82983 
3325014710210458
 82983 
111111

161515161516
1903410
family
1335027265589556739302574

111111
genus
3316211216212565
71655

 1109412 
3316211216212565

1109412 
1109412 
1109412 
1109412 
1109412 
1109412 
species
111111

666666
204037
583514282441247219491207
genus

species

1778540 
1778540 
1778540 
1778540 
1778540 
1778540 
3438418816115362
 1778540 
111111

111111
36912121321816195

2037915 
2037915 
2037915 
2037915 
2037915 
2037915 
species
 2037915 

433116196182174105

204039 
204039 
204039 
204039 
204039 
204039 
species
 204039 
111111

 204038 
species

204038 
204038 
204038 
204038 
204038 
204038 
1099249378393359209
111111

species

1089444 
1089444 
1089444 
1089444 
1089444 
1089444 
24756769971127817520
 1089444 
111111

species

204042 
204042 
204042 
204042 
204042 
204042 
1116182469391285216
 204042 
111111

genus
50211218721717094
84565
433434

 1239307 

1239307 
1239307 
1239307 
1239307 
1239307 
1239307 
2284296765633
species
111111

 1486991 

1486991 
1486991 
1486991 
1486991 
1486991 
1486991 
15242551068040
species
111111

 63612 
1202836343420

63612 
63612 
63612 
63612 
63612 
63612 
species
111111

211

1929246 


1929246 

1929246 
species
 1929246 
111

555555
668211242849271616861208
genus
122277

 554 
species
290452113351286695481

554 
554 
554 
554 
554 
554 
111111

111111

29471 
29471 
29471 
29471 
29471 
29471 
1582254660592492353
species
 29471 

111111
 55208 
species
317381051026039

55208 
55208 
55208 
55208 
55208 
55208 

 2042057 
758152339396164164

2042057 
2042057 
2042057 
2042057 
2042057 
2042057 
species
111111

111111
species
1121159410340275171

1905730 
1905730 
1905730 
1905730 
1905730 
1905730 
 1905730 

252624262727
order
7496835956061093698
135613

89791010
1046
family
212249215191462225

221233
genus
513132313
1227

111111
species

473531 
473531 
473531 
473531 
473531 
473531 
411111
 473531 

species
112215

1229 
1229 


1229 
1229 
 1229 
1111

111
species
217


133539 
133539 
133539 
 133539 

1980513
genus
7222
1111

1111
 1630141 


1630141 

1630141 
1630141 
1630141 
7222
species

genus
657457369237
85076
111111

 37487 
species

37487 
37487 
37487 
37487 
37487 
37487 
657457369237
111111

111111

1978339 
1978339 
1978339 
1978339 
1978339 
1978339 
372833274831
species
 1978339 

156885
genus
10104974411
111111

111111
species

80679 
80679 
80679 
80679 
80679 
80679 
10104974411
 80679 

402431316117
genus
53392
111111

111111

1166950 
1166950 
1166950 
1166950 
1166950 
1166950 
402431316117
species
 1166950 

13724
genus
2161224411053
111111

111111
2161224411053

73141 
73141 
73141 
73141 
73141 
73141 
species
 73141 

111111
343222418261
genus
85072

111111
species

1049 
1049 
1049 
1049 
1049 
1049 
343222418261
 1049 

2326141012
family
1738654
111111

111111
1738655
2326141012
genus

111111
species

1548547 
1548547 
1548547 
1548547 
1548547 
1548547 
2326141012
 1548547 

255526
family
629943778259
222222

109262
629943778259
genus
222222

111111
species
348426433633

927 
927 
927 
927 
927 
927 
 927 

111111
 1860122 
species
281517344626

1860122 
1860122 
1860122 
1860122 
1860122 
1860122 

71571014
family
449719
111111

437504
71571014
genus
111111

111111
 437505 
species
71571014

437505 
437505 
437505 
437505 
437505 
437505 

121212121212
family
390247293286446347
72276

190140165173304207
genus
106633
555555


108010 
108010 
108010 
108010 
108010 
108010 
242331385036
species
 108010 
111111

species

1033854 
1033854 
1033854 
1033854 
1033854 
1033854 
594636407867
 1033854 
111111

111111
462936366359

396595 
396595 
396595 
396595 
396595 
396595 
species
 396595 

111111
species
222620297516

186931 
186931 
186931 
186931 
186931 
186931 
 186931 

 106634 
391642303829

106634 
106634 
106634 
106634 
106634 
106634 
species
111111

111111
133193
513247364240
genus

111111
513247364240

351052 
351052 
351052 
351052 
351052 
351052 
species
 351052 

genus
25518121017
1765964
111111

111111
species

160660 
160660 
160660 
160660 
160660 
160660 
25518121017
 160660 

1051
473317182025
genus
111111

 1442136 
species
473317182025

1442136 
1442136 
1442136 
1442136 
1442136 
1442136 
111111

222222
85108
genus
342524374440

 1052 
43991511

1052 
1052 
1052 
1052 
1052 
1052 
species
111111

111111
species
302215282929

1053 
1053 
1053 
1053 
1053 
1053 
 1053 

222222
1335745
431222102618
genus

111111
2278693

1335757 
1335757 
1335757 
1335757 
1335757 
1335757 
species
 1335757 

111111
 1335746 
species

1335746 
1335746 
1335746 
1335746 
1335746 
1335746 
2151441715

558533318341
family
1676141
111111

genus
558533318341
1676142
111111

111111
species
558533318341

1579979 
1579979 
1579979 
1579979 
1579979 
1579979 
 1579979 

order
13111
1240482
11111

11111
1240483
13111
family

11111
1193503
13111
genus

11111

1196095 
1196095 
1196095 
1196095 

1196095 
13111
species
 1196095 

1692040
701396614913689
order
333333

1692041
family
701396614913689
333333

111111
986106
genus
44723492121

 1281578 
species
44723492121

1281578 
1281578 
1281578 
1281578 
1281578 
1281578 
111111

1692042
396620616536
genus
111111

111111
species

1675686 
1675686 
1675686 
1675686 
1675686 
1675686 
396620616536
 1675686 

1744881
genus
272623395032
111111

111111
species
272623395032

1620215 
1620215 
1620215 
1620215 
1620215 
1620215 
 1620215 

806978787574
135622
179716551000181914401169
order

135656
family
267894
122221

67572
135656
genus
122221

 314282 
1123


314282 
314282 
314282 
314282 
species
1111

species

357794 
357794 
357794 
357794 
357794 
357794 
124426
 357794 
111111

232224232223
267890
625527313821503270
family

625527313821503270
genus
22
232224232223

 56812 

56812 
56812 
56812 


56812 
8621
species
1111

 62322 

62322 
62322 
62322 
62322 
62322 
62322 
180929013215081
species
111111

 60478 
species
3097131810

60478 
60478 
60478 
60478 
60478 
60478 
111111

111111
species
357375755

351745 
351745 
351745 
351745 
351745 
351745 
 351745 

 60217 


60217 
60217 
60217 
60217 
60217 
57373
species
11111

1111
3415

192073 

192073 
192073 
192073 
species
 192073 


70863 
70863 
70863 
70863 
70863 
70863 
242742139
species
 70863 
111111

 60481 

60481 
60481 
60481 
60481 
60481 
60481 
53391021915
species
111111

 94122 

94122 
94122 
94122 
94122 
94122 
94122 
114316312811
species
111111

111111
 260364 
283919291517

260364 
260364 
260364 
260364 
260364 
260364 
species

111111

225848 
225848 
225848 
225848 
225848 
225848 
194218112121
species
 225848 

 2059264 
16526512

2059264 
2059264 
2059264 
2059264 
2059264 
2059264 
species
111111

 93973 
species
235511

93973 
93973 
93973 
93973 
93973 
93973 
111111

 404011 
4382

404011 

404011 
404011 

404011 
species
1111

111111
 271098 
species
5241110222

271098 
271098 
271098 
271098 
271098 
271098 

 70864 
species
4842020159

70864 
70864 
70864 
70864 
70864 
70864 
111111

 60480 

60480 
60480 
60480 
60480 
60480 
60480 
1133353713
species
111111

 60961 
species

60961 
60961 
60961 
60961 
60961 
60961 
103231045
111111

 43661 

43661 
43661 
43661 
43661 
43661 
43661 
1462817339
species
111111

473324232515

359303 
359303 
359303 
359303 
359303 
359303 
species
 359303 
111111

15214511

2029986 
2029986 
2029986 
2029986 
2029986 
2029986 
species
 2029986 
111111

 2018305 
121651633

2018305 
2018305 
2018305 
2018305 
2018305 
2018305 
species
111111

111111
7748129143

271097 
271097 
271097 
271097 
271097 
271097 
species
 271097 

 24 
species

24 
24 
24 
24 
24 
24 
5171221812
111111

72275
family
820877464756713664
242223232223

11
21
genus
1621534

11
21

326544 


326544 
species
 326544 

24621358
genus
89404
222122

11111
 300231 
species

300231 
300231 
300231 

300231 
300231 
63125

 983545 
18311333

983545 
983545 
983545 
983545 
983545 
983545 
species
111111

261825
155358112
genus
111111

111111
species
155358112

680279 
680279 
680279 
680279 
680279 
680279 
 680279 

111111
1751872
genus
28841176

species
28841176

1526571 
1526571 
1526571 
1526571 
1526571 
1526571 
 1526571 
111111

11111
1172191
genus
1273181


2172099 
2172099 
2172099 
2172099 
2172099 
1273181
species
 2172099 
11111

genus
523668293542601574
2742
999999

111111
species
45109421064785

1671721 
1671721 
1671721 
1671721 
1671721 
1671721 
 1671721 

111111
 2743 
species
726581788798

2743 
2743 
2743 
2743 
2743 
2743 

111111

490759 
490759 
490759 
490759 
490759 
490759 
2011011818852
species
 490759 

111111
 1033846 
species

1033846 
1033846 
1033846 
1033846 
1033846 
1033846 
406120504043

 1749259 
373230543355

1749259 
1749259 
1749259 
1749259 
1749259 
1749259 
species
111111

111111
 330734 
species

330734 
330734 
330734 
330734 
330734 
330734 
261313492434

111111
species
121134456011293

1420916 
1420916 
1420916 
1420916 
1420916 
1420916 
 1420916 

111111
521711214428

1874317 
1874317 
1874317 
1874317 
1874317 
1874317 
species
 1874317 

111111
 1420917 

1420917 
1420917 
1420917 
1420917 
1420917 
1420917 
110127404312686
species

111111
genus
389352494
288793

111111
 2183582 
389352494

2183582 
2183582 
2183582 
2183582 
2183582 
2183582 
species

878879
genus
192124124897970
226

 715451 
species
11


715451 

715451 
11

111111
 233316 
species
3120423125

233316 
233316 
233316 
233316 
233316 
233316 

species
1011872232933

314275 
314275 
314275 
314275 
314275 
314275 
 314275 
111111

 1917158 
species

1917158 
1917158 
1917158 
1917158 

1917158 
72331
11111

species

1777491 
1777491 
1777491 
1777491 
1777491 
1777491 
228175
 1777491 
111111

11111
 287094 
species

287094 
287094 
287094 

287094 
287094 
21516

species
1731772

589873 
589873 
589873 
589873 
589873 
589873 
 589873 
111111

 28108 
species

28108 
28108 
28108 
28108 
28108 
28108 
407428202116
111111

11111
 2058133 

2058133 

2058133 
2058133 
2058133 
2058133 
81121
species

546663
267889
221029341715
family

genus
191025231615
28228
445553

111111
species
112113

28229 
28229 
28229 
28229 
28229 
28229 
 28229 

14172411

1816219 
1816219 
1816219 
1816219 
1816219 
1816219 
species
 1816219 
111111

 1967665 
species

1967665 

1967665 

1967665 
365
111

1111
1351

1816218 
1816218 
1816218 
1816218 
species
 1816218 

11111
 58049 
551841


58049 
58049 
58049 
58049 
58049 
species

11
species


2161872 
2161872 
12
 2161872 

1111
1518149
genus
34111

1111
 1763536 
species

1763536 

1763536 
1763536 
1763536 
34111

111111
267892
3414651592449
family

genus
3414651592449
44011
111111

 44012 
species
3414651592449

44012 
44012 
44012 
44012 
44012 
44012 
111111

family
2135893104131136
267888
201316171618

2135893104131136
genus
53246
201316171618

 228 

228 
228 
228 
228 
228 
228 
4345119
species
111111

11111
species

43658 

43658 
43658 
43658 
43658 
253571
 43658 

11111
species
12121

1761891 
1761891 
1761891 
1761891 

1761891 
 1761891 

 152297 
species
2221616

152297 

152297 
152297 
152297 
152297 
11111

species

28107 
28107 
28107 
28107 
28107 
28107 
1776591
 28107 
111111

111111
species
1555232

288 
288 
288 
288 
288 
288 
 288 

 43659 
species

43659 


43659 
43659 
171
111

11111
 283699 

283699 
283699 
283699 

283699 
283699 
11111
species

111111
species
12818722

161398 
161398 
161398 
161398 
161398 
161398 
 161398 

11111
861041

43662 
43662 

43662 
43662 
43662 
species
 43662 

111111
 298657 

298657 
298657 
298657 
298657 
298657 
298657 
291010162650
species

 234831 
species
2


234831 
1


43657 
43657 
43657 
43657 
43657 
43657 
5851013212
species
 43657 
111111

111
 394751 

394751 
394751 


394751 
222
species

 176102 

176102 
176102 
176102 
176102 
176102 
176102 
212283
species
111111

11111

314281 

314281 
314281 
314281 
314281 
133216
species
 314281 

1
 166935 
species

166935 
1

111111
 247523 
species

247523 
247523 
247523 
247523 
247523 
247523 
449213

species


1720343 

1720343 
218
 1720343 
11

species
911

267375 


267375 
267375 
 267375 
111

 1348114 
species
1141411

1348114 
1348114 
1348114 
1348114 

1348114 
11111

1111

1514074 

1514074 
1514074 

1514074 
42024
species
 1514074 

444444
593126273524
family
267893

444444
genus
593126273524
135575

species

2100422 
2100422 
2100422 
2100422 
2100422 
2100422 
19108957
 2100422 
111111

111111
3213761710

1096243 
1096243 
1096243 
1096243 
1096243 
1096243 
species
 1096243 

315832

2055892 
2055892 
2055892 
2055892 
2055892 
2055892 
species
 2055892 
111111

species

135577 
135577 
135577 
135577 
135577 
135577 
5764105
 135577 
111111

212221
family
2331912125
267891

2331912125
genus
58050
212221

11111
18118115

80854 

80854 
80854 
80854 
80854 
species
 80854 

species

69539 
69539 
69539 
69539 
69539 
53841
 69539 
11111

order
217218671129219319942261
135619
394039404141

222222
255527
family
665818293812

111111
genus
31275776
1445504

111111
 1445505 
species
31275776

1445505 
1445505 
1445505 
1445505 
1445505 
1445505 

111111
230494
genus
35311322316

 1336806 

1336806 
1336806 
1336806 
1336806 
1336806 
1336806 
35311322316
species
111111

family
15301368823154514891779
28256
202221212120

111111
1438281105117195
genus
204286

111111
species
1438281105117195

28258 
28258 
28258 
28258 
28258 
28258 
 28258 

111
genus
211
114185

 114186 
211


114186 
114186 

114186 
species
111

111111
42054
945242536966
genus

111111
 158080 
species

158080 
158080 
158080 
158080 
158080 
158080 
945242536966

111111
73752212110
genus
235572

111111
species

91844 
91844 
91844 
91844 
91844 
91844 
73752212110
 91844 

genus
8659014809439611177
2745
131313131313

 2136172 
species
1721835914674292

2136172 
2136172 
2136172 
2136172 
2136172 
2136172 
111111

5357226870120

115561 
115561 
115561 
115561 
115561 
115561 
species
 115561 
111111

 2746 

2746 
2746 
2746 
2746 
2746 
2746 
1107668112140106
species
111111

111111
 1178482 
species
803421437767

1178482 
1178482 
1178482 
1178482 
1178482 
1178482 

 1504981 

1504981 
1504981 
1504981 
1504981 
1504981 
1504981 
332815563476
species
111111

 1610576 
species
11192230276

1610576 
1610576 
1610576 
1610576 
1610576 
1610576 
111111


507626 
507626 
507626 
507626 
507626 
507626 
7212266160147105
species
 507626 
111111

species
1143242

1971364 
1971364 
1971364 
1971364 
1971364 
1971364 
 1971364 
111111

956848123129190

475662 
475662 
475662 
475662 
475662 
475662 
species
 475662 
111111

122125667214391

1883416 
1883416 
1883416 
1883416 
1883416 
1883416 
species
 1883416 
111111


1897729 
1897729 
1897729 
1897729 
1897729 
1897729 
891006084104106
species
 1897729 
111111

 213554 

213554 
213554 
213554 
213554 
213554 
213554 
879132348
species
111111

 1118153 
96172488

1118153 
1118153 
1118153 
1118153 
1118153 
1118153 
species
111111

genus
176156631159697
376488
111111

111111
176156631159697

376489 
376489 
376489 
376489 
376489 
376489 
species
 376489 

137715283131126
genus
404432
111111

111111
species
137715283131126

1771309 
1771309 
1771309 
1771309 
1771309 
1771309 
 1771309 

genus
12
1495768
11

species


1495769 

1495769 
12
 1495769 
11

222222
108665222393108
genus
504090

 698828 
species

698828 
698828 
698828 
698828 
698828 
698828 
442715454335
111111

species
6439371785073

157779 
157779 
157779 
157779 
157779 
157779 
 157779 
111111

91852189
family
191033
111111

genus
91852189
141450
111111

 141451 
91852189

141451 
141451 
141451 
141451 
141451 
141451 
species
111111

666666
224372
323205159339266283
family

2025617
314785
genus
111111

species

1917421 
1917421 
1917421 
1917421 
1917421 
1917421 
314785
 1917421 
111111

320204155332258278
genus
59753
555555

 285091 
species

285091 
285091 
285091 
285091 
285091 
285091 
994742726028
111111

111111
 1094342 
79504210243127

1094342 
1094342 
1094342 
1094342 
1094342 
1094342 
species

111111
 59754 
species
910726815

59754 
59754 
59754 
59754 
59754 
59754 

111111
species
12594488113082

1306787 
1306787 
1306787 
1306787 
1306787 
1306787 
 1306787 

species

1113728 
1113728 
1113728 
1113728 
1113728 
1113728 
8316511726
 1113728 
111111

1920240
35818136
family
221234

261963
genus
35818136
221234

111
species
182

914150 


914150 
914150 
 914150 

1111
species
4852


1561924 
1561924 
1561924 

1561924 
 1561924 

31


261964 
261964 
species
 261964 
11

211321

1144748 
1144748 

1144748 
1144748 
1144748 
species
 1144748 
11111

767777
135620
161150111218147144
family

323333
28253
genus
2168311922

 400668 
species

400668 
400668 
400668 
400668 
400668 
400668 
514386
111111

11111
species

119864 

119864 
119864 
119864 
119864 
211573
 119864 

145313413

936476 
936476 
936476 
936476 
936476 
936476 
species
 936476 
111111

187492
17518531513
genus
111111

111111
17518531513

187493 
187493 
187493 
187493 
187493 
187493 
species
 187493 

111111
8010944739090
genus
48075

111111
8010944739090

1821621 
1821621 
1821621 
1821621 
1821621 
1821621 
species
 1821621 

1537406
genus
182831401918
111111

111111
182831401918

1249553 
1249553 
1249553 
1249553 
1249553 
1249553 
species
 1249553 

111111
188907
genus
252102141

111111
 188908 
species

188908 
188908 
188908 
188908 
188908 
188908 
252102141

224379
family
80635422328
111111

genus
80635422328
158481
111111

 158327 
species

158327 
158327 
158327 
158327 
158327 
158327 
80635422328
111111

414141414141
135614
2685203580692472179285786487216
order

32033
family
2668203489587237164445672486295
333333333333

genus
150311151626577037612857049777
40323
888888

111111
species

83617 
83617 
83617 
83617 
83617 
83617 
141625025674356540
 83617 

111111
 128780 
species

128780 
128780 
128780 
128780 
128780 
128780 
358653960016310101292

133618921329321502

1793721 
1793721 
1793721 
1793721 
1793721 
1793721 
species
 1793721 
111111

 1904944 

1904944 
1904944 
1904944 
1904944 
1904944 
1904944 
35663145462314329215632
species
111111

96149109825016330252062836087
species group
995085
111111


40324 
40324 
40324 
40324 
40324 
40324 
96149109825016330252062836087
species
 40324 
111111

 1827305 
322235912259967541316

1827305 
1827305 
1827305 
1827305 
1827305 
1827305 
species
111111

62029615289819223524

216778 
216778 
216778 
216778 
216778 
216778 
species
 216778 
111111

111111
 2005046 
species

2005046 
2005046 
2005046 
2005046 
2005046 
2005046 
27374281128133658884

555555
genus
35941111134684615561704
68

111111
 84531 
species

84531 
84531 
84531 
84531 
84531 
84531 
1005414313268459525

111111
species
548183233155245261

69 
69 
69 
69 
69 
69 
 69 

 435897 

435897 
435897 
435897 
435897 
435897 
435897 
1187345441223476447
species
111111

111111
4056014684142199

1605891 
1605891 
1605891 
1605891 
1605891 
1605891 
species
 1605891 

449109213116234272

262324 
262324 
262324 
262324 
262324 
262324 
species
 262324 
111111

151515151515
338
1096681764219122111152553233309
genus

2186337326058

53413 
53413 
53413 
53413 
53413 
53413 
species
 53413 
111111

111111
species
2805311123117161

29447 
29447 
29447 
29447 
29447 
29447 
 29447 

111111
species
10099912751157234

56454 
56454 
56454 
56454 
56454 
56454 
 56454 

 343 

343 
343 
343 
343 
343 
343 
380068981143611601440
species
111111

111111

1985254 
1985254 
1985254 
1985254 
1985254 
1985254 
1359304245251370503
species
 1985254 

111111
 90270 
species
2606163251114338444

90270 
90270 
90270 
90270 
90270 
90270 

111111
 56459 

56459 
56459 
56459 
56459 
56459 
56459 
4671064868162145
species

 442694 
806180192139234303

442694 
442694 
442694 
442694 
442694 
442694 
species
111111

111111
species
13332501703151435583

56458 
56458 
56458 
56458 
56458 
56458 
 56458 

 339 

339 
339 
339 
339 
339 
339 
1515620411890121829433604
species
111111

111111
 456327 
species

456327 
456327 
456327 
456327 
456327 
456327 
1733354349273470693

species
2403841194154243060417597

347 
347 
347 
347 
347 
347 
 347 
111111

111111
529968519849754001209716309
species group
643453

 346 
species
529968519849754001209716309

346 
346 
346 
346 
346 
346 
111111

 48664 
species

48664 
48664 
48664 
48664 
48664 
48664 
2244457455370515733
111111

111111
 56460 
species

56460 
56460 
56460 
56460 
56460 
56460 
1623245252159433502

genus
324241156165147222
2370
111111

111111
species

2371 
2371 
2371 
2371 
2371 
2371 
324241156165147222
 2371 

666251272173213267
genus
83614
222222

111111
species
3448812646118123

2006110 
2006110 
2006110 
2006110 
2006110 
2006110 
 2006110 

111111
 2172536 
species
32216314612795144

2172536 
2172536 
2172536 
2172536 
2172536 
2172536 

22574885713847061016
genus
83618
222222

111111
 314722 

314722 
314722 
314722 
314722 
314722 
314722 
1699400457265513759
species

 415229 
species

415229 
415229 
415229 
415229 
415229 
415229 
55888114119193257
111111

1775411
family
1700911523514841140921
888888

111111
323413
14790155125150122
genus

111111
species

323415 
323415 
323415 
323415 
323415 
323415 
14790155125150122
 323415 

111111
70411
genus
10370236824361

species
10370236824361

81475 
81475 
81475 
81475 
81475 
81475 
 81475 
111111

111111
242605
genus
4362342700501259195

4362342700501259195

242606 
242606 
242606 
242606 
242606 
242606 
species
 242606 
111111

2864926228
genus
2233801
111111

 2021234 
2864926228

2021234 
2021234 
2021234 
2021234 
2021234 
2021234 
species
111111

231454
7203921685572517420
genus
333333

111111
species
26191489196228191

445710 
445710 
445710 
445710 
445710 
445710 
 445710 

111111
species
258222593213144124

231455 
231455 
231455 
231455 
231455 
231455 
 231455 

111111
 1379159 
20179603163145105

1379159 
1379159 
1379159 
1379159 
1379159 
1379159 
species

75309
genus
266119410178149115
111111

111111

666685 
666685 
666685 
666685 
666685 
666685 
266119410178149115
species
 666685 

161616161516
1706369
472520236321543404
order

222222
1706372
344928405269
family

222222
344928405269
genus
1217416

111111
 930806 

930806 
930806 
930806 
930806 
930806 
930806 
224510314158
species

 930805 

930805 
930805 
930805 
930805 
930805 
930805 
1241891111
species
111111

444444
1706373
family
1071687476110121

genus
1071687476110121
48073
444444

 260552 
species
315317303536

260552 
260552 
260552 
260552 
260552 
260552 
111111

111111
 252514 
species

252514 
252514 
252514 
252514 
252514 
252514 
341324272558

111111
 359370 
1137493

359370 
359370 
359370 
359370 
359370 
359370 
species

 1769779 

1769779 
1769779 
1769779 
1769779 
1769779 
1769779 
319926154124
species
111111

412124453866
family
1706375
333333

111111
39391812
genus
1084558

 716816 
39391812

716816 
716816 
716816 
716816 
716816 
716816 
species
111111

630749
2054211751
genus
111111

species

1620392 
1620392 
1620392 
1620392 
1620392 
1620392 
2054211751
 1620392 
111111

1434050
genus
187171533
111111

 1470434 

1470434 
1470434 
1470434 
1470434 
1470434 
1470434 
187171533
species
111111

777767
1706371
290282110160343148
family

111111
genus
277933472713
447467

species
277933472713

447471 
447471 
447471 
447471 
447471 
447471 
 447471 
111111

316625
genus
613934710
111111

111111
species

86304 
86304 
86304 
86304 
86304 
86304 
613934710
 86304 

2425
genus
1662412434
111111

111111

2426 
2426 
2426 
2426 
2426 
2426 
1662412434
species
 2426 

333333
10
1851574996266119
genus

 1987723 

1987723 
1987723 
1987723 
1987723 
1987723 
1987723 
4874102616761
species
111111

species

155077 
155077 
155077 
155077 
155077 
155077 
493823595438
 155077 
111111

111111
 1945512 

1945512 
1945512 
1945512 
1945512 
1945512 
1945512 
884516114520
species

genus
11112
2036021
11111

species
11112

1737490 
1737490 
1737490 
1737490 

1737490 
 1737490 
11111

135625
107033759765673382304
order
232222232317

107033759765673382304
family
712
232222232317

11111
697331
genus
2372321

11111
2372321

157673 
157673 

157673 
157673 
157673 
species
 157673 

111111
266681151499387
genus
745

species
266681151499387

747 
747 
747 
747 
747 
747 
 747 
111111

476528
genus
201415129
11111

11111
 47735 
species

47735 
47735 
47735 
47735 
47735 
201415129

155493
genus
9124131723
111111

 750 

750 
750 
750 
750 
750 
750 
9124131723
species
111111

333333
416916
genus
15639751027851

species

732 
732 
732 
732 
732 
732 
662034263024
 732 
111111

111111
851533734526

714 
714 
714 
714 
714 
714 
species
 714 

111111
species

739 
739 
739 
739 
739 
739 
548331
 739 

1822257543828
genus
75984
333223

111111
 85404 
121152095

85404 
85404 
85404 
85404 
85404 
85404 
species

 75985 

75985 
75985 
75985 
75985 
75985 
75985 
1141935342921
species
111111

1111
 1432056 
species

1432056 
1432056 
1432056 


1432056 
56272

1171161252522
genus
713
535553

11111
131125

67854 
67854 
67854 
67854 
67854 
species
 67854 

111111
 715 
74745141718

715 
715 
715 
715 
715 
715 
species


718 
718 
718 
718 
718 
53651
species
 718 
11111

11111
 189834 
141211

189834 

189834 
189834 
189834 
189834 
species

11111
 716 
species
118213

716 

716 
716 
716 
716 

genus
5383273895
2094023
111111

111111
 738 
species

738 
738 
738 
738 
738 
738 
5383273895

111111
214906
genus
8416131011

111111
 731 
species
8416131011

731 
731 
731 
731 
731 
731 

676773
724
genus
2571225909524410176

111111

727 
727 
727 
727 
727 
727 
20776590461316451
species
 727 

1111
 712310 
species
1161

712310 
712310 

712310 
712310 

111111
 730 

730 
730 
730 
730 
730 
730 
162622139
species

species


726 
726 
726 
726 
3233
 726 
1111


249188 
249188 
249188 
249188 
249188 
102262
species
 249188 
11111

 197575 
species
113127461516

197575 
197575 
197575 
197575 
197575 
197575 
111111

12712303

729 
729 
729 
729 
729 
species
 729 
11111

 1248727 
27803475324

1248727 
1248727 
1248727 
1248727 
1248727 
1248727 
species
111111

349742
genus
38342676422
111111

111111
species

1543721 
1543721 
1543721 
1543721 
1543721 
1543721 
38342676422
 1543721 

111111
742030
781215098169157
order

781215098169157
family
742031
111111

111111
180541
781215098169157
genus

111111

2183911 
2183911 
2183911 
2183911 
2183911 
2183911 
781215098169157
species
 2183911 

1211101599
72273
order
277539319487338315

135616
family
243502254391268264
645645

genus
585122317
40222
211222

1111
 754477 
species
3531

754477 


754477 
754477 
754477 

111111
 754476 
28572016

754476 
754476 
754476 
754476 
754476 
754476 
species

1237
genus
230492243376243238
111111

111111
 1238 
230492243376243238

1238 
1238 
1238 
1238 
1238 
1238 
species

11111
28884
genus
11127

11111

39765 
39765 

39765 
39765 
39765 
11127
species
 39765 

21
genus
34067
21

species


728003 
728003 
11
 728003 
11

1
species


1329899 
1
 1329899 

21111
933
genus
71412

11111
 92245 
31412

92245 
92245 
92245 
92245 

92245 
species

1
species
4

147268 
 147268 

453743
233463936949
family
34064

11
1869285
11
genus

 594679 
11


594679 

594679 
species
11

443643
262
genus
233363926949

species
12


573570 
573570 
 573570 
11

 28110 

28110 

28110 
28110 
41322
species
111

1111
 549298 

549298 
549298 

549298 
549298 
1113
species

111111
 263 
species

263 
263 
263 
263 
263 
263 
163039536138

 657445 

657445 
657445 
657445 
657445 
657445 
657445 
21111338
species
111111

111
 1542390 


1542390 

1542390 

1542390 
123
species

222211
135617
1132312
family

1111
40751
2112
genus

species
2112

40754 
40754 
40754 
40754 
 40754 
1111

genus
921112
1021
111111

 288004 
species

288004 
288004 
288004 
288004 
288004 
288004 
921112
111111

species
11114

186490 
186490 

186490 
186490 
186490 
 186490 
11111

580370
292142114
class
211222

211222
580371
order
292142114

family
292142114
580372
211222

211222
377315
genus
292142114

11111
 1921086 
species
202112

1921086 
1921086 

1921086 
1921086 
1921086 

11111
 1921087 

1921087 

1921087 
1921087 
1921087 
1921087 
9132012
species

class
341919325120
1553900
321232

111121
301719314918
order
213481

111121
301719314918
family
213483

genus
301719314918
958
111121

species
301719314818

959 
959 
959 
959 
959 
959 
 959 
111111

1
 453816 
species
1


453816 

order
42122
2024979
21111

263369
family
12
11

11
146784
genus
12

11
12

960 


960 
species
 960 

family
3212
1652132
1111

1652133
genus
3212
1111

1111
3212

97084 
97084 

97084 
97084 
species
 97084 

778911269696119310046627436199462
class
28216
260264256262265266

1111
genus
1432
1301080

1111
1432

1160784 
1160784 
1160784 


1160784 
species
 1160784 

206351
order
229927295221308441532033
242323242424

777777
9091343633124623441022
family
1499392

111111
7610990141265124
genus
568394

111111
species

748280 
748280 
748280 
748280 
748280 
748280 
7610990141265124
 748280 

genus
5839443757941426634
535
333333

 1108595 
species

1108595 
1108595 
1108595 
1108595 
1108595 
1108595 
221324158333461274
111111

11626485166293142

2059672 
2059672 
2059672 
2059672 
2059672 
2059672 
species
 2059672 
111111

111111
246356132295672218

536 
536 
536 
536 
536 
536 
species
 536 

885864
66114586623983
genus
111111

111111

1906741 
1906741 
1906741 
1906741 
1906741 
1906741 
66114586623983
species
 1906741 

111111
7766456415889
genus
187


1938604 
1938604 
1938604 
1938604 
1938604 
1938604 
7766456415889
species
 1938604 
111111

1071106518125692
genus
168470
111111

111111
 168471 
species
1071106518125692

168471 
168471 
168471 
168471 
168471 
168471 

171616171717
481
139013864588183818091011
family

111
143
genus
71

111
 72 
species
143

72 
72 


72 

111
512
genus
1193515

 1196083 
species
512


1196083 
1196083 

1196083 
111

1111
1111

2052837 

2052837 
2052837 

2052837 
species
 2052837 

112222
59
204021867349
genus

111111
204019807147

63 
63 
63 
63 
63 
63 
species
 63 

1111
species


96942 
96942 
96942 
96942 
2622
 96942 

538
genus
122131334
111111

111111

539 
539 
539 
539 
539 
539 
122131334
species
 539 

1111
genus
4223
32257

 504 
species

504 
504 
504 

504 
4223
1111

121210121212
13521338454617371727955
genus
482

111111

28091 
28091 
28091 
28091 
28091 
28091 
11143211
species
 28091 

111111
3251029149

1853278 
1853278 
1853278 
1853278 
1853278 
1853278 
species
 1853278 

 655307 
15713112213

655307 
655307 
655307 
655307 
655307 
655307 
species
111111

 487 

487 
487 
487 
487 
487 
487 
11541049440714651292779
species
111111

11111

326523 
326523 

326523 
326523 
326523 
253163
species
 326523 

111111
species
861145511521581

485 
485 
485 
485 
485 
485 
 485 

111111
 490 
186854

490 
490 
490 
490 
490 
490 
species


495 
495 
495 
495 
495 
495 
273120507327
species
 495 
111111

111111
 486 

486 
486 
486 
486 
486 
486 
57822163013
species

11111
species

1853276 
1853276 

1853276 
1853276 
1853276 
21222
 1853276 

930718359

488 
488 
488 
488 
488 
488 
species
 488 
111111

 483 
species
89217214

483 
483 
483 
483 
483 
483 
111111

202219192222
32003
order
403606322620895374

family
93119127209343137
2008793
222222

111111
1054211
genus
4143487517564

species
4143487517564

748811 
748811 
748811 
748811 
748811 
748811 
 748811 
111111

378210
52767913416873
genus
111111


1842540 
1842540 
1842540 
1842540 
1842540 
1842540 
52767913416873
species
 1842540 
111111

family
155164102131197100
32011
676577

12222
359407
28717112
genus

 359408 

359408 
359408 
359408 

359408 
359408 
28614101
species
11111

1111
species


1055487 
1055487 

1055487 
1055487 
1311
 1055487 

111111
404
genus
544533455452


405 
405 
405 
405 
405 
405 
544533455452
species
 405 
111111

222222
81682
54107517510139
genus

111111
species

887061 
887061 
887061 
887061 
887061 
887061 
38693355709
 887061 

 266009 
163818203130

266009 
266009 
266009 
266009 
266009 
266009 
species
111111

1679002
genus
21552
11111

1111
2155

1581680 
1581680 

1581680 
1581680 
species
 1581680 

1
 1581557 


1581557 
2
species

111111
16
genus
17416265

111111
 1662285 
17416265

1662285 
1662285 
1662285 
1662285 
1662285 
1662285 
species

153224358225
family
2008790
111111

111111
919
153224358225
genus

 36861 

36861 
36861 
36861 
36861 
36861 
36861 
153224358225
species
111111

786788
206379
442919274636
family

332333
15186211926
genus
35798


1288494 
1288494 

1288494 
1288494 
1288494 
35685
species
 1288494 
11111

111111
 1231 

1231 
1231 
1231 
1231 
1231 
1231 
132124
species

species

35799 
35799 
35799 
35799 
35799 
35799 
1110414917
 35799 
111111

genus
29111362710
914
454455


153948 
153948 
153948 

153948 
1411
species
 153948 
1111

 261292 

261292 


261292 
261292 
511
species
111

111
 44574 


44574 
44574 

44574 
121
species

111111
21751174

915 
915 
915 
915 
915 
915 
species
 915 

11111
21262

44577 
44577 

44577 
44577 
44577 
species
 44577 

111111

916 
916 
916 
916 
916 
916 
112222
species
 916 

family
962625021822776
90627
444444

111111
85310384815
genus
1778653

111111
 1985873 
species

1985873 
1985873 
1985873 
1985873 
1985873 
1985873 
85310384815

genus
14499567112
935200
111111

14499567112

649841 
649841 
649841 
649841 
649841 
649841 
species
 649841 
111111

96
38121762456
genus
111111

111111
 370405 
species
38121762456

370405 
370405 
370405 
370405 
370405 
370405 

314343
genus
363924626343
111111

species
363924626343

63745 
63745 
63745 
63745 
63745 
63745 
 63745 
111111

111111
327159
genus
373836435813

 327160 
species
373836435813

327160 
327160 
327160 
327160 
327160 
327160 
111111

11


543913 
543913 
31
species
 543913 

111
111
genus
189384

111
 189385 
species

189385 
189385 

189385 
111

195197194196198198
80840
order
73901122074547279468826633295321

32012
48726114715374
genus
111111

111111
 926 
species

926 
926 
926 
926 
926 
926 
48726114715374

111111
11512885176249127
genus
65047

111111
 1658665 
species

1658665 
1658665 
1658665 
1658665 
1658665 
1658665 
11512885176249127

11
 1834205 
12

1834205 


1834205 
species

868988888989
119060
family
399793998020080403836874731034

494949494949
32008
genus
238312155611916240813978916605

111111

1468409 
1468409 
1468409 
1468409 
1468409 
1468409 
373269477716
species
 1468409 

111111
 758796 

758796 
758796 
758796 
758796 
758796 
758796 
11513775193254167
species


1795874 
1795874 
1795874 
1795874 
1795874 
1795874 
452640739326972844149
species
 1795874 
111111

 640512 

640512 
640512 
640512 
640512 
640512 
640512 
84146408919397
species
111111

111111
 640510 
species

640510 
640510 
640510 
640510 
640510 
640510 
961896713327282

species group
6992902645198645150216889
87882
222222222222

111111
7310765113200103

1207504 
1207504 
1207504 
1207504 
1207504 
1207504 
species
 1207504 

111111
 152480 
species
258401175378647333

152480 
152480 
152480 
152480 
152480 
152480 

111111
 95485 
species

95485 
95485 
95485 
95485 
95485 
95485 
113116131184353173


60550 
60550 
60550 
60550 
60550 
60550 
9913457183192160
species
 60550 
111111

species
200726201251243647262037

95486 
95486 
95486 
95486 
95486 
95486 
 95486 
111111

1221267211611348

1637869 
1637869 
1637869 
1637869 
1637869 
1637869 
species
 1637869 
111111

111111
 1503054 
species

1503054 
1503054 
1503054 
1503054 
1503054 
1503054 
51896113519254

111111
7659074809171425700

87883 
87883 
87883 
87883 
87883 
87883 
species
 87883 

111111

1637853 
1637853 
1637853 
1637853 
1637853 
1637853 
110107569111482
species
 1637853 

 488732 
species

488732 
488732 
488732 
488732 
488732 
488732 
841087916416072
111111

 152500 
species
554254746644

152500 
152500 
152500 
152500 
152500 
152500 
111111

111111

1503055 
1503055 
1503055 
1503055 
1503055 
1503055 
55131518515252
species
 1503055 

 60552 
7288313866431279512

60552 
60552 
60552 
60552 
60552 
60552 
species
111111

 265293 
1441038212127191

265293 
265293 
265293 
265293 
265293 
265293 
species
111111

111111

488447 
488447 
488447 
488447 
488447 
488447 
13316059111270142
species
 488447 

111111

1637862 
1637862 
1637862 
1637862 
1637862 
1637862 
11393757713338
species
 1637862 

111111
 488729 
10184355118883

488729 
488729 
488729 
488729 
488729 
488729 
species


101571 
101571 
101571 
101571 
101571 
101571 
6638114097971312619
species
 101571 
111111

 482957 
species
160185117239378257

482957 
482957 
482957 
482957 
482957 
482957 
111111

 488731 
species

488731 
488731 
488731 
488731 
488731 
488731 
725564119179101
111111

111111

292 
292 
292 
292 
292 
292 
10561614684149224331136
species
 292 

302027611923852

488446 
488446 
488446 
488446 
488446 
488446 
species
 488446 
111111

111111
species

416344 
416344 
416344 
416344 
416344 
416344 
627884116177109
 416344 

111111
 28095 
10231371581118920621010

28095 
28095 
28095 
28095 
28095 
28095 
species

species

1097668 
1097668 
1097668 
1097668 
1097668 
1097668 
109898010228780
 1097668 
111111

111527
species group
6309799549347565136526173
999999

 1637841 
704526564718

1637841 
1637841 
1637841 
1637841 
1637841 
1637841 
species
111111

111111
 1385591 
species

1385591 
1385591 
1385591 
1385591 
1385591 
1385591 
40493610013162

species

1637831 
1637831 
1637831 
1637831 
1637831 
1637831 
223232535567
 1637831 
111111

 342113 
248374193366577300

342113 
342113 
342113 
342113 
342113 
342113 
species
111111

111111
species

28450 
28450 
28450 
28450 
28450 
28450 
414855793400511093534226
 28450 

111111
5716994436991273533

13373 
13373 
13373 
13373 
13373 
13373 
species
 13373 

 1385592 
species
763066398342

1385592 
1385592 
1385592 
1385592 
1385592 
1385592 
111111

 57975 
species

57975 
57975 
57975 
57975 
57975 
57975 
1091115670711102053891
111111

 1637837 

1637837 
1637837 
1637837 
1637837 
1637837 
1637837 
433131328034
species
111111

111111
 1740163 
6871377816061

1740163 
1740163 
1740163 
1740163 
1740163 
1740163 
species


1740162 
1740162 
1740162 
1740162 
1740162 
1740162 
7819337558123
species
 1740162 
111111

111111
 41899 
18520656150391201

41899 
41899 
41899 
41899 
41899 
41899 
species

 758793 

758793 
758793 
758793 
758793 
758793 
758793 
11012491102261160
species
111111

111111
species

1804984 
1804984 
1804984 
1804984 
1804984 
1804984 
1289461128245169
 1804984 

species
11713166139223148

1678678 
1678678 
1678678 
1678678 
1678678 
1678678 
 1678678 
111111

111111
species
159116579217087

2217913 
2217913 
2217913 
2217913 
2217913 
2217913 
 2217913 

111111
6437883677351224616

337 
337 
337 
337 
337 
337 
species
 337 

species
280931124716551947114

1795043 
1795043 
1795043 
1795043 
1795043 
1795043 
 1795043 
111111

111111
species
18122659151271154

640511 
640511 
640511 
640511 
640511 
640511 
 640511 

444444
3805553025055712115444610
genus
48736

111111
species

329 
329 
329 
329 
329 
329 
499452214431922430
 329 

111111
207344171365593218

105219 
105219 
105219 
105219 
105219 
105219 
species
 105219 

111111
 190721 
species

190721 
190721 
190721 
190721 
190721 
190721 
256355134333596273

 305 
species

305 
305 
305 
305 
305 
305 
284343791986458394333689
111111

93217
genus
171922131041238737081540
999999

111111
 656178 
species
1021488412617873

656178 
656178 
656178 
656178 
656178 
656178 


656179 
656179 
656179 
656179 
656179 
656179 
531093611921877
species
 656179 
111111

111111

445709 
445709 
445709 
445709 
445709 
445709 
16521294268305140
species
 445709 

111111
 93220 

93220 
93220 
93220 
93220 
93220 
93220 
5506113147401225490
species

111111
species
367469237426715338

93218 
93218 
93218 
93218 
93218 
93218 
 93218 

111111
species

93219 
93219 
93219 
93219 
93219 
93219 
10615271189300162
 93219 

 93221 
941735317621594

93221 
93221 
93221 
93221 
93221 
93221 
species
111111

species

573737 
573737 
573737 
573737 
573737 
573737 
10697466525173
 573737 
111111

111111
species

93222 
93222 
93222 
93222 
93222 
93222 
17624210627830193
 93222 

999999
genus
192126381050280548732470
106589

26532889334577304

68895 
68895 
68895 
68895 
68895 
68895 
species
 68895 
111111

111111
 367825 
species

367825 
367825 
367825 
367825 
367825 
367825 
214269117265440301

111111
 106590 

106590 
106590 
106590 
106590 
106590 
106590 
4206422326311260534
species

111111
 248026 
112182106193389177

248026 
248026 
248026 
248026 
248026 
248026 
species

 2036817 
228234109259572214

2036817 
2036817 
2036817 
2036817 
2036817 
2036817 
species
111111

111111
 1389192 
20026394304463228

1389192 
1389192 
1389192 
1389192 
1389192 
1389192 
species

species
198256135326512307

119219 
119219 
119219 
119219 
119219 
119219 
 119219 
111111

species

876364 
876364 
876364 
876364 
876364 
876364 
206339103329441291
 876364 
111111


82541 
82541 
82541 
82541 
82541 
82541 
7812565164219114
species
 82541 
111111

111111
157932
659456772393301144533956
genus

111111
species

29443 
29443 
29443 
29443 
29443 
29443 
659456772393301144533956
 29443 

44013
genus
435103512051
254455

31242


556054 
556054 
556054 
556054 
556054 
species
 556054 
11111

111111

1743172 
1743172 
1743172 
1743172 
1743172 
1743172 
1227415
species
 1743172 

111111
 576611 
species

576611 
576611 
576611 
576611 
576611 
576611 
3256239327

species
43154


1835254 

1835254 
1835254 
1835254 
 1835254 
1111

1111
 576610 
species
1143


576610 
576610 

576610 
576610 

121212121212
genus
210523311165235242601802
1822464

111111
species

134537 
134537 
134537 
134537 
134537 
134537 
1701176916321088
 134537 

111111
 75105 
species

75105 
75105 
75105 
75105 
75105 
75105 
6276403726071391475

species

412963 
412963 
412963 
412963 
412963 
412963 
221320324818
 412963 
111111

22224263149441173

2026199 
2026199 
2026199 
2026199 
2026199 
2026199 
species
 2026199 
111111

 169430 
8018385141251132

169430 
169430 
169430 
169430 
169430 
169430 
species
111111

111111
1241504217822291

948107 
948107 
948107 
948107 
948107 
948107 
species
 948107 

 148447 
species
1389666122148106

148447 
148447 
148447 
148447 
148447 
148447 
111111

111111
 36873 

36873 
36873 
36873 
36873 
36873 
36873 
332363162387543281
species

111111
species
668632125193112

1926494 
1926494 
1926494 
1926494 
1926494 
1926494 
 1926494 

 311230 
species
11612381144292108

311230 
311230 
311230 
311230 
311230 
311230 
111111

107161114157315119

261302 
261302 
261302 
261302 
261302 
261302 
species
 261302 
111111

species
1011575914720699

252970 
252970 
252970 
252970 
252970 
252970 
 252970 
111111

genus
104101109152283128
28067
111111

 28068 

28068 
28068 
28068 
28068 
28068 
28068 
104101109152283128
species
111111

genus
689986195255149
88
111111

111111
species
689986195255149

34029 
34029 
34029 
34029 
34029 
34029 
 34029 

107995611423694
genus
318147
111111

 1768242 
species
107995611423694

1768242 
1768242 
1768242 
1768242 
1768242 
1768242 
111111

242424242424
75682
family
1354455515211912727915075041567

777777
149698
genus
27869022439912349286947095

 1707785 
371128148315703756940

1707785 
1707785 
1707785 
1707785 
1707785 
1707785 
species
111111

species

2072590 
2072590 
2072590 
2072590 
2072590 
2072590 
4091334356209243071142
 2072590 
111111

111111
 1593482 
3921337322182042551045

1593482 
1593482 
1593482 
1593482 
1593482 
1593482 
species

 47229 

47229 
47229 
47229 
47229 
47229 
47229 
4141158197513853494867
species
111111

 1141883 
31688050614122850795

1141883 
1141883 
1141883 
1141883 
1141883 
1141883 
species
111111

 2045208 
species
4581459403177248071092

2045208 
2045208 
2045208 
2045208 
2045208 
2045208 
111111

111111
 1678028 
species

1678028 
1678028 
1678028 
1678028 
1678028 
1678028 
4261573354229852251214

7781400671179817510665229714
genus
29580
666666

7317844165418109

375286 
375286 
375286 
375286 
375286 
375286 
species
 375286 
111111


1236179 
1236179 
1236179 
1236179 
1236179 
1236179 
251411430206
species
 1236179 
111111

111111
 368607 
species

368607 
368607 
368607 
368607 
368607 
368607 
2262118942711901313658859

 1938606 
2246117982762038321449011

1938606 
1938606 
1938606 
1938606 
1938606 
1938606 
species
111111

species

55508 
55508 
55508 
55508 
55508 
55508 
7373553219179196622489
 55508 
111111

species
2438125033552250330439240

1644131 
1644131 
1644131 
1644131 
1644131 
1644131 
 1644131 
111111

555555
1668357214675366284052795
genus
963

species
794170814152168342961377

964 
964 
964 
964 
964 
964 
 964 
111111

species
2105051525111051449

80842 
80842 
80842 
80842 
80842 
80842 
 80842 
111111

species
3036402146781446450

92645 
92645 
92645 
92645 
92645 
92645 
 92645 
111111


2025949 
2025949 
2025949 
2025949 
2025949 
2025949 
15728357340577203
species
 2025949 
111111

111111
species
2044361004501035316

341045 
341045 
341045 
341045 
341045 
341045 
 341045 

202907
11492616840276162851795
genus
333333

4698172689462126632

158899 
158899 
158899 
158899 
158899 
158899 
species
 158899 
111111


279113 
279113 
279113 
279113 
279113 
279113 
3269723018902150581
species
 279113 
111111

 279058 
3548272719252009582

279058 
279058 
279058 
279058 
279058 
279058 
species
111111

846
663023386728
genus
111111

species

847 
847 
847 
847 
847 
847 
663023386728
 847 
111111

222222
303379
genus
9420875294647140

species
22863112526459

1809410 
1809410 
1809410 
1809410 
1809410 
1809410 
 1809410 
111111

 204773 
721224416938381

204773 
204773 
204773 
204773 
204773 
204773 
species
111111

111111
93681
798556141258185
genus


76731 
76731 
76731 
76731 
76731 
76731 
798556141258185
species
 76731 
111111

family
14606192739227178943117615806
506
313129313231

222222
290425
2365277714757
genus

111111
species
144720557931

302406 
302406 
302406 
302406 
302406 
302406 
 302406 

111111
 310575 
species

310575 
310575 
310575 
310575 
310575 
310575 
9187226826

264122545726
genus
305976
111111

111111
species
264122545726

1007105 
1007105 
1007105 
1007105 
1007105 
1007105 
 1007105 

359336
genus
628453125200101
111111

 75697 
species
628453125200101

75697 
75697 
75697 
75697 
75697 
75697 
111111

121121
29574
genus
431467

11
 84590 
species
11


84590 


84590 

 29575 
species
421457

29575 
29575 
29575 
29575 
29575 
29575 
111111

1111
90243
1121
genus

species

90245 


90245 
90245 
90245 
1121
 90245 
1111

3213522
genus
1100891
11111

11111
 643674 
3213522

643674 
643674 

643674 
643674 
643674 
species

genus
10011899145217181
1921582
111111

111111
 1851544 
species

1851544 
1851544 
1851544 
1851544 
1851544 
1851544 
10011899145217181

11554151407411136282440312397
genus
517
171717171717

 1331258 
species
1081343845229103

1331258 
1331258 
1331258 
1331258 
1331258 
1331258 
111111

species
12419883109238151

1416806 
1416806 
1416806 
1416806 
1416806 
1416806 
 1416806 
111111

species

518 
518 
518 
518 
518 
518 
450546281411954408
 518 
111111

6067103716671173686

35814 
35814 
35814 
35814 
35814 
35814 
species
 35814 
111111

 1697043 
species

1697043 
1697043 
1697043 
1697043 
1697043 
1697043 
901024712116490
111111

111111
 463040 
species
6512259178245162

463040 
463040 
463040 
463040 
463040 
463040 

 521 
species
121916477013

521 
521 
521 
521 
521 
521 
111111

111111
 94624 
421265111026564

94624 
94624 
94624 
94624 
94624 
94624 
species

111111

463014 
463014 
463014 
463014 
463014 
463014 
681085414611471
species
 463014 

111111

1416803 
1416803 
1416803 
1416803 
1416803 
1416803 
192191103225317232
species
 1416803 

 463024 
342420567726

463024 
463024 
463024 
463024 
463024 
463024 
species
111111

111111
species

519 
519 
519 
519 
519 
519 
264300168204479265
 519 

111111
 520 

520 
520 
520 
520 
520 
520 
868611719569510413187699350
species

111111
 123899 

123899 
123899 
123899 
123899 
123899 
123899 
9112985193271195
species

111111
 463025 
species
407352207317459252

463025 
463025 
463025 
463025 
463025 
463025 

species

2163011 
2163011 
2163011 
2163011 
2163011 
2163011 
6313382
 2163011 
111111


103855 
103855 
103855 
103855 
103855 
103855 
309357132353571327
species
 103855 
111111

555555
222
genus
273736791511367159002931

111111
 1758194 
species
154255132244435218

1758194 
1758194 
1758194 
1758194 
1758194 
1758194 

111111
 217203 
333376152392622339

217203 
217203 
217203 
217203 
217203 
217203 
species

111111
17182245942231137081755

85698 
85698 
85698 
85698 
85698 
85698 
species
 85698 

 217204 
228230109306499248

217204 
217204 
217204 
217204 
217204 
217204 
species
111111

111111
 32002 

32002 
32002 
32002 
32002 
32002 
32002 
304573176418636371
species

9614110317623983
genus
507
111111

111111
 511 

511 
511 
511 
511 
511 
511 
9614110317623983
species

111111
71211175023

1469502 
1469502 
1469502 
1469502 
1469502 
1469502 
species
 1469502 

424242424242
80864
4595597433397168126745425
family

species
4749256012051

1458425 
1458425 
1458425 
1458425 
1458425 
1458425 
 1458425 
111111

genus
150253117297505172
52972
222222

111111

216465 
216465 
216465 
216465 
216465 
216465 
701528717429795
species
 216465 

species

296591 
296591 
296591 
296591 
296591 
296591 
801013012320877
 296591 
111111

222222
219181
5114370140211119
genus


1658672 
1658672 
1658672 
1658672 
1658672 
1658672 
111417354711
species
 1658672 
111111

111111
4012953105164108

2109914 
2109914 
2109914 
2109914 
2109914 
2109914 
species
 2109914 

444444
34072
109610621084171227381375
genus

111111
 34073 
species

34073 
34073 
34073 
34073 
34073 
34073 
5004715178711307660

111111
 2126319 
species
268213281372526340

2126319 
2126319 
2126319 
2126319 
2126319 
2126319 

111111
 1795631 
105918914026383

1795631 
1795631 
1795631 
1795631 
1795631 
1795631 
species

111111
223287197329642292

436515 
436515 
436515 
436515 
436515 
436515 
species
 436515 

222222
1649468
genus
130234101277410138

 2109913 

2109913 
2109913 
2109913 
2109913 
2109913 
2109913 
6284477920673
species
111111

 2116657 
species
681505419820465

2116657 
2116657 
2116657 
2116657 
2116657 
2116657 
111111

111111
242110112
genus
1436289

 1436290 
242110112

1436290 
1436290 
1436290 
1436290 
1436290 
1436290 
species
111111

111111
genus
4362336416686
352450

111111
 2109915 
4362336416686

2109915 
2109915 
2109915 
2109915 
2109915 
2109915 
species

64521510786106
genus
665874
222222

111111
 1678129 

1678129 
1678129 
1678129 
1678129 
1678129 
1678129 
41028174
species

 1678128 

1678128 
1678128 
1678128 
1678128 
1678128 
1678128 
6042139969102
species
111111

444444
28065
genus
280303201418712274

111111
species

192843 
192843 
192843 
192843 
192843 
192843 
182954348137
 192843 


1842727 
1842727 
1842727 
1842727 
1842727 
1842727 
15114385252368149
species
 1842727 
111111

species

1484693 
1484693 
1484693 
1484693 
1484693 
1484693 
851074110518861
 1484693 
111111

 81479 
species
262421277527

81479 
81479 
81479 
81479 
81479 
81479 
111111

12916
genus
10891366644148731041129
888888

111111
 80867 

80867 
80867 
80867 
80867 
80867 
80867 
10115992151357140
species

111111
142180677225479

1842533 
1842533 
1842533 
1842533 
1842533 
1842533 
species
 1842533 

111111
 1858609 

1858609 
1858609 
1858609 
1858609 
1858609 
1858609 
74735613818271
species


721785 
721785 
721785 
721785 
721785 
721785 
84130368823375
species
 721785 
111111

111111
8196479025598

358220 
358220 
358220 
358220 
358220 
358220 
species
 358220 

111111
200218159293596240

80869 
80869 
80869 
80869 
80869 
80869 
species
 80869 

111111

553814 
553814 
553814 
553814 
553814 
553814 
2964111425201012323
species
 553814 

111111
 232721 
species
1119945135215103

232721 
232721 
232721 
232721 
232721 
232721 

 1458426 
species

1458426 
1458426 
1458426 
1458426 
1458426 
1458426 
154914666254
111111

333333
genus
215253125365596317
47420

 1763535 
species
65100358813161

1763535 
1763535 
1763535 
1763535 
1763535 
1763535 
111111

111111
 1842537 

1842537 
1842537 
1842537 
1842537 
1842537 
1842537 
1019932135263167
species

111111
 795665 
species

795665 
795665 
795665 
795665 
795665 
795665 
49545814220289

111111
364316
genus
7167789714582

species

364317 
364317 
364317 
364317 
364317 
364317 
7167789714582
 364317 
111111

283
genus
390491216512924342
333333

species
264293117310630218

285 
285 
285 
285 
285 
285 
 285 
111111

 225992 
species
473921547932

225992 
225992 
225992 
225992 
225992 
225992 
111111

 1082851 
791597814821592

1082851 
1082851 
1082851 
1082851 
1082851 
1082851 
species
111111

80865
5318633507041570649
genus
444444

111111
 80866 

80866 
80866 
80866 
80866 
80866 
80866 
122279139201475179
species

species

742013 
742013 
742013 
742013 
742013 
742013 
15915179157373121
 742013 
111111

species
9920772200371160

1920191 
1920191 
1920191 
1920191 
1920191 
1920191 
 1920191 
111111

111111
 180282 
species
15122660146351189

180282 
180282 
180282 
180282 
180282 
180282 

111111
201096
genus
145307103324481186

111111
145307103324481186

179636 
179636 
179636 
179636 
179636 
179636 
species
 179636 

genus
13516849163296107
238749
111111

 1546149 
species

1546149 
1546149 
1546149 
1546149 
1546149 
1546149 
13516849163296107
111111

174951
14124893365537236
genus
111111


94132 
94132 
94132 
94132 
94132 
94132 
14124893365537236
species
 94132 
111111


413882 
413882 
413882 
413882 
413882 
413882 
103138102214302178
species
 413882 
111111

111111
1111045015621057
genus
92793

111111
species

1296669 
1296669 
1296669 
1296669 
1296669 
1296669 
1111045015621057
 1296669 

111111
genus
741165318523991
316612

 105560 

105560 
105560 
105560 
105560 
105560 
105560 
741165318523991
species
111111

360378221467750381
genus
212743
111111

111111
 946333 

946333 
946333 
946333 
946333 
946333 
946333 
360378221467750381
species

161616161616
12101456842196927881629
order
206389

2008794
9871159668157322531317
family
121212121212

12960
genus
6417243579371233789
666666

111111
 198107 
11013273171180186

198107 
198107 
198107 
198107 
198107 
198107 
species

 62928 
species

62928 
62928 
62928 
62928 
62928 
62928 
116936015314693
111111

 748247 
83655011510541

748247 
748247 
748247 
748247 
748247 
748247 
species
111111

111111
 41977 
16721398325362255

41977 
41977 
41977 
41977 
41977 
41977 
species

species
701145198266157

418699 
418699 
418699 
418699 
418699 
418699 
 418699 
111111

111111
 2067960 
species

2067960 
2067960 
2067960 
2067960 
2067960 
2067960 
95107257517457

555555
genus
323403301584913482
33057

 59405 
656943107122130

59405 
59405 
59405 
59405 
59405 
59405 
species
111111

 2005884 

2005884 
2005884 
2005884 
2005884 
2005884 
2005884 
62858513025792
species
111111

111111

96773 
96773 
96773 
96773 
96773 
96773 
5915266174210149
species
 96773 

111111

85643 
85643 
85643 
85643 
85643 
85643 
10150508718856
species
 85643 

111111

1134435 
1134435 
1134435 
1134435 
1134435 
1134435 
3647578613655
species
 1134435 

species

2080469 
2080469 
2080469 
2080469 
2080469 
2080469 
2332105210746
 2080469 
111111

family
171932258828
2008795
111111

73029
171932258828
genus
111111

171932258828

259537 
259537 
259537 
259537 
259537 
259537 
species
 259537 
111111

333333
family
206278142371447284
75787

111111

1898103 
1898103 
1898103 
1898103 
1898103 
1898103 
323422388725
species
 1898103 

146937
11816981199234172
genus
111111

111111
 146939 
species

146939 
146939 
146939 
146939 
146939 
146939 
11816981199234172

111111
56753913412687
genus
551759

 551760 
species
56753913412687

551760 
551760 
551760 
551760 
551760 
551760 
111111

3457414512183

1904640 
1904640 
1904640 
1904640 
1904640 
1904640 
species
 1904640 
111111

33055
54113137
genus
121323

species


233181 
233181 
233181 
233181 
233181 
212112
 233181 
11111

 994695 
species
1


994695 
1

1111
 33056 
5224

33056 
33056 


33056 
33056 
species

11
species


994696 

994696 
101
 994696 

616081041271923321970797285127082
phylum
201174
473477487483482474

459464468465466459
1760
614541039821921041967237264826934
class

131313131313
order
1072875156719831261665
85008

131313131313
family
1072875156719831261665
28056

1865
417305642850490232
genus
555555

111111
species

2033844 
2033844 
2033844 
2033844 
2033844 
2033844 
6445731129550
 2033844 

species
106551361589540

196914 
196914 
196914 
196914 
196914 
196914 
 196914 
111111

 649831 
63461281407135

649831 
649831 
649831 
649831 
649831 
649831 
species
111111

 1866 
species
77571071736122

1866 
1866 
1866 
1866 
1866 
1866 
111111

 134676 
species
10710219826716885

134676 
134676 
134676 
134676 
134676 
134676 
111111

genus
12810020831020176
1873
222222

111111
 648999 
6943931539752

648999 
648999 
648999 
648999 
648999 
648999 
species

111111
 47850 
species
595711515710424

47850 
47850 
47850 
47850 
47850 
47850 

168694
genus
3425671215019
222222

111111

168697 
168697 
168697 
168697 
168697 
168697 
18143471258
species
 168697 

species
161133502511

168695 
168695 
168695 
168695 
168695 
168695 
 168695 
111111

333333
673534
451411545597453316
genus

111111
species

2071627 
2071627 
2071627 
2071627 
2071627 
2071627 
152135240206168112
 2071627 

111111
735877767831

2024580 
2024580 
2024580 
2024580 
2024580 
2024580 
species
 2024580 

 2108470 
species

2108470 
2108470 
2108470 
2108470 
2108470 
2108470 
226218228315207173
111111

42341051056722
genus
84593
111111

111111
 1003110 
42341051056722

1003110 
1003110 
1003110 
1003110 
1003110 
1003110 
species

147067
382249556828
genus
111111

382249556828

2006 
2006 
2006 
2006 
2006 
2006 
species
 2006 
111111

111111
1643683
72911114
order

111111
85032
family
72911114

111111
72911114
genus
28048


28049 
28049 
28049 
28049 
28049 
28049 
72911114
species
 28049 
111111

order
5763911159746
1643684
111111

5763911159746
family
85031
111111

111111
53460
genus
5763911159746

111111
 53461 

53461 
53461 
53461 
53461 
53461 
53461 
5763911159746
species

202020202019
85004
1222880169720881367607
order

31953
1222880169720881367607
family
202020202019

111111
113421
genus
196082

111111
species
113421

78258 
78258 
78258 
78258 
78258 
78258 
 78258 

1678
1183866167920791360602
genus
181818181817

22277

1691 
1691 
1691 
1691 
1691 
species
 1691 
11111

 1685 
353259532593407163

1685 
1685 
1685 
1685 
1685 
1685 
species
111111

 1683 
species

1683 
1683 
1683 
1683 
1683 
1683 
311324381013
111111

111111
 1686 
321101510

1686 
1686 
1686 
1686 
1686 
1686 
species

23914729334820195

28025 
28025 
28025 
28025 
28025 
28025 
species
 28025 
111111


216816 
216816 
216816 
216816 
216816 
216816 
248168340423325118
species
 216816 
111111

species

35760 
35760 
35760 
35760 
35760 
35760 
31146053347
 35760 
111111

111111
 1694 
species

1694 
1694 
1694 
1694 
1694 
1694 
282869785228

111111
977015824012081

1681 
1681 
1681 
1681 
1681 
1681 
species
 1681 

111111
 158787 
species
5538641094525

158787 
158787 
158787 
158787 
158787 
158787 

 33905 
734652

33905 
33905 
33905 
33905 
33905 
33905 
species
111111

111111
 1684 

1684 
1684 
1684 
1684 
1684 
1684 
8362319266
species

111111
species
113014301213

1687 
1687 
1687 
1687 
1687 
1687 
 1687 

111111
 28026 
4471671

28026 
28026 
28026 
28026 
28026 
28026 
species

314541

638619 
638619 
638619 
638619 
638619 
638619 
species
 638619 
111111

 1689 
24163641178

1689 
1689 
1689 
1689 
1689 
1689 
species
111111

111111
 1680 
species
322236464614

1680 
1680 
1680 
1680 
1680 
1680 

111111
71312172717

630129 
630129 
630129 
630129 
630129 
630129 
species
 630129 

2701
381315554
genus
111111

111111
381315554

2702 
2702 
2702 
2702 
2702 
2702 
species
 2702 

85013
order
19718230243821688
555555

74712
19718230243821688
family
555555

genus
19718230243821688
1854
555555

 656024 

656024 
656024 
656024 
656024 
656024 
656024 
372548371822
species
111111

111111
species
4629451224719

1859 
1859 
1859 
1859 
1859 
1859 
 1859 

111111
 298653 
species

298653 
298653 
298653 
298653 
298653 
298653 
6140681087111

111111
species
33651021175928

298654 
298654 
298654 
298654 
298654 
298654 
 298654 

 106370 
species
20233954218

106370 
106370 
106370 
106370 
106370 
106370 
111111

 1650658 
221


1650658 
1650658 


1650658 
species
111

202917353382423025691136
order
85010
282828282828

family
202917353382423025691136
2070
282828282828

555555
65496
genus
17810331436422676

111111
 1612552 
372163756620

1612552 
1612552 
1612552 
1612552 
1612552 
1612552 
species

111111
species
411972664511

2072503 
2072503 
2072503 
2072503 
2072503 
2072503 
 2072503 

111111
351682825527

1470176 
1470176 
1470176 
1470176 
1470176 
1470176 
species
 1470176 

111111
 340345 
species
34163172227

340345 
340345 
340345 
340345 
340345 
340345 

 1612551 
313166693811

1612551 
1612551 
1612551 
1612551 
1612551 
1612551 
species
111111

111111
2029
3789991316736
genus

 860235 
species

860235 
860235 
860235 
860235 
860235 
860235 
3789991316736
111111

3831831066736
genus
674734
111111

111111
species

1653480 
1653480 
1653480 
1653480 
1653480 
1653480 
3831831066736
 1653480 

777777
genus
4943997591003604268
1847

111111
 1688404 
species
764610011310741

1688404 
1688404 
1688404 
1688404 
1688404 
1688404 

 1690815 
54431041536143

1690815 
1690815 
1690815 
1690815 
1690815 
1690815 
species
111111

111111
 1096868 
species

1096868 
1096868 
1096868 
1096868 
1096868 
1096868 
79521201508031

111111
species
7444981289436

1641402 
1641402 
1641402 
1641402 
1641402 
1641402 
 1641402 

species
871151191707545

1096856 
1096856 
1096856 
1096856 
1096856 
1096856 
 1096856 
111111

 240495 

240495 
240495 
240495 
240495 
240495 
240495 
4337991408123
species
111111

111111
 445576 
816211914910649

445576 
445576 
445576 
445576 
445576 
445576 
species

111111
2071
genus
5551911255932

species

103731 
103731 
103731 
103731 
103731 
103731 
5551911255932
 103731 
111111

13176365258
genus
1851
111111

111111
 1852 
species
13176365258

1852 
1852 
1852 
1852 
1852 
1852 

222222
40566
genus
1468927427716352

111111
 42197 
species

42197 
42197 
42197 
42197 
42197 
42197 
64341201266325

 40567 
825515415110027

40567 
40567 
40567 
40567 
40567 
40567 
species
111111

262756706435
genus
142577
111111

111111
 530584 

530584 
530584 
530584 
530584 
530584 
530584 
262756706435
species

111111
genus
466611110410544
43356

111111
 43357 

43357 
43357 
43357 
43357 
43357 
43357 
466611110410544
species

666666
1813
847712123916071008456
genus


1814 
1814 
1814 
1814 
1814 
1814 
93801091379347
species
 1814 
111111

420328674906502223

33910 
33910 
33910 
33910 
33910 
33910 
species
 33910 
111111

111111
 31958 

31958 
31958 
31958 
31958 
31958 
31958 
10915620024714056
species

884867887237

1911175 
1911175 
1911175 
1911175 
1911175 
1911175 
species
 1911175 
111111

 208439 

208439 
208439 
208439 
208439 
208439 
208439 
575410510711454
species
111111

species
8046841228739

1896961 
1896961 
1896961 
1896961 
1896961 
1896961 
 1896961 
111111

165301
828615520411346
genus
111111

species
828615520411346

1586287 
1586287 
1586287 
1586287 
1586287 
1586287 
 1586287 
111111

111111
genus
67651381746847
1835


1836 
1836 
1836 
1836 
1836 
1836 
67651381746847
species
 1836 
111111

777777
85012
order
503491800996718256

2004
20015030934127696
family
222222

111111
2000
835714113311249
genus

 2001 
species

2001 
2001 
2001 
2001 
2001 
2001 
835714113311249
111111

111111
83681
genus
1179316820816447

species
1179316820816447

1909395 
1909395 
1909395 
1909395 
1909395 
1909395 
 1909395 
111111

family
274313430588393138
83676
444444

2013
261295408544373130
genus
333333

 280236 
species
212650904831

280236 
280236 
280236 
280236 
280236 
280236 
111111

species
402284724825

53437 
53437 
53437 
53437 
53437 
53437 
 53437 
111111

111111
 2014 
20024727438227774

2014 
2014 
2014 
2014 
2014 
2014 
species

83677
genus
13182244208
111111

 2021 
species

2021 
2021 
2021 
2021 
2021 
2021 
13182244208
111111

2012
family
292861674922
111111

111111
292861674922
genus
2019

species

2020 
2020 
2020 
2020 
2020 
2020 
292861674922
 2020 
111111

85011
order
5706513597851355082203399
909090909090

5706513597851355082203399
family
2062
909090909090

genus
278196512745386151
2063
444444

 2066 

2066 
2066 
2066 
2066 
2066 
2066 
74521421609553
species
111111

 68173 
species
4728781566415

68173 
68173 
68173 
68173 
68173 
68173 
111111

 2018025 
905715418113055

2018025 
2018025 
2018025 
2018025 
2018025 
2018025 
species
111111


1894 
1894 
1894 
1894 
1894 
1894 
67591382489728
species
 1894 
111111

868686868686
1883
5428493992731280578343248
genus

species

1964449 
1964449 
1964449 
1964449 
1964449 
1964449 
313058926339
 1964449 
111111


193462 
193462 
193462 
193462 
193462 
193462 
5133721015218
species
 193462 
111111

111111
species

47716 
47716 
47716 
47716 
47716 
47716 
399958867928
 47716 

241765665213

1901 
1901 
1901 
1901 
1901 
1901 
species
 1901 
111111

 68214 
46501101236427

68214 
68214 
68214 
68214 
68214 
68214 
species
111111

species
7312010617412454

1751294 
1751294 
1751294 
1751294 
1751294 
1751294 
 1751294 
111111

111111
 38300 
3729791096522

38300 
38300 
38300 
38300 
38300 
38300 
species

111111
 1902 
species

1902 
1902 
1902 
1902 
1902 
1902 
67558512813018

 408015 
342659765722

408015 
408015 
408015 
408015 
408015 
408015 
species
111111

 2049881 
species

2049881 
2049881 
2049881 
2049881 
2049881 
2049881 
4147831249632
111111

species

1885 
1885 
1885 
1885 
1885 
1885 
46529416910039
 1885 
111111

111111
5244961385428

33903 
33903 
33903 
33903 
33903 
33903 
species
 33903 

111111
4848981195219

2174846 
2174846 
2174846 
2174846 
2174846 
2174846 
species
 2174846 

111111
species

47763 
47763 
47763 
47763 
47763 
47763 
15812926634422275
 47763 

111111
species
695510110511730

1561022 
1561022 
1561022 
1561022 
1561022 
1561022 
 1561022 

111111
 1930 

1930 
1930 
1930 
1930 
1930 
1930 
63291111618841
species

5233992295842

68202 
68202 
68202 
68202 
68202 
68202 
species
 68202 
111111

 362257 

362257 
362257 
362257 
362257 
362257 
362257 
7342951698334
species
111111

species

1984801 
1984801 
1984801 
1984801 
1984801 
1984801 
4632731368324
 1984801 
111111

111111
 68192 

68192 
68192 
68192 
68192 
68192 
68192 
4839981227320
species

111111
5237751378452

2059884 
2059884 
2059884 
2059884 
2059884 
2059884 
species
 2059884 

111111
 1616117 
7371891319228

1616117 
1616117 
1616117 
1616117 
1616117 
1616117 
species

 1109743 
303295908526

1109743 
1109743 
1109743 
1109743 
1109743 
1109743 
species
111111

 444103 
species
5583751407323

444103 
444103 
444103 
444103 
444103 
444103 
111111

111111
 1849967 
species

1849967 
1849967 
1849967 
1849967 
1849967 
1849967 
521865975129

111111

862751 
862751 
862751 
862751 
862751 
862751 
5143801495922
species
 862751 

111111
 1969 
1299920025316683

1969 
1969 
1969 
1969 
1969 
1969 
species

111111

1783515 
1783515 
1783515 
1783515 
1783515 
1783515 
62421031006646
species
 1783515 

111111
4237871049631

1827580 
1827580 
1827580 
1827580 
1827580 
1827580 
species
 1827580 

 1495638 
species
79175792229673

1495638 
1495638 
1495638 
1495638 
1495638 
1495638 
111111

 379067 

379067 
379067 
379067 
379067 
379067 
379067 
671361241348371
species
111111

 68570 

68570 
68570 
68570 
68570 
68570 
68570 
19218926741127484
species
111111

111111
 75293 

75293 
75293 
75293 
75293 
75293 
75293 
4324471065036
species

111111
43341161305822

1265601 
1265601 
1265601 
1265601 
1265601 
1265601 
species
 1265601 

111111
 1950 
3330581015817

1950 
1950 
1950 
1950 
1950 
1950 
species


1736046 
1736046 
1736046 
1736046 
1736046 
1736046 
5833631027333
species
 1736046 
111111

629295
species subgroup
192163343528286138
333333

111111
 68179 
3645711178635

68179 
68179 
68179 
68179 
68179 
68179 
species

 1911 
species

1911 
1911 
1911 
1911 
1911 
1911 
5742881264844
111111

 1908 
species

1908 
1908 
1908 
1908 
1908 
1908 
997618428515259
111111

 1437453 
4729821197544

1437453 
1437453 
1437453 
1437453 
1437453 
1437453 
species
111111

111111
species
4239103866429

1690221 
1690221 
1690221 
1690221 
1690221 
1690221 
 1690221 

5079721436521

1661694 
1661694 
1661694 
1661694 
1661694 
1661694 
species
 1661694 
111111

 1940 
62301061405757

1940 
1940 
1940 
1940 
1940 
1940 
species
111111

111111
 465541 

465541 
465541 
465541 
465541 
465541 
465541 
5529671606845
species

111111
 1535768 

1535768 
1535768 
1535768 
1535768 
1535768 
1535768 
332278987743
species

 1837283 
species

1837283 
1837283 
1837283 
1837283 
1837283 
1837283 
5325821516418
111111

species
61641511477351

1915 
1915 
1915 
1915 
1915 
1915 
 1915 
111111

111111
species
432186876218

1926 
1926 
1926 
1926 
1926 
1926 
 1926 

1852274
312285498602328122
species group
111111


1888 
1888 
1888 
1888 
1888 
1888 
312285498602328122
species
 1888 
111111

38104851707520

206662 
206662 
206662 
206662 
206662 
206662 
species
 206662 
111111

111111
 1935 

1935 
1935 
1935 
1935 
1935 
1935 
6962861243530
species

 2135430 
species
56251061257729

2135430 
2135430 
2135430 
2135430 
2135430 
2135430 
111111

111111
 42239 

42239 
42239 
42239 
42239 
42239 
42239 
62501161119818
species


1649184 
1649184 
1649184 
1649184 
1649184 
1649184 
52488314610144
species
 1649184 
111111

 1912 
species

1912 
1912 
1912 
1912 
1912 
1912 
313225464547352214
111111

111111
 1169025 
species

1169025 
1169025 
1169025 
1169025 
1169025 
1169025 
38401041128523

111111
 1971 
4369881167657

1971 
1971 
1971 
1971 
1971 
1971 
species

 1907 
species
53381311127323

1907 
1907 
1907 
1907 
1907 
1907 
111111

111111
species
6759951578730

1355015 
1355015 
1355015 
1355015 
1355015 
1355015 
 1355015 

 1889 
species

1889 
1889 
1889 
1889 
1889 
1889 
1158623130616547
111111

 92644 

92644 
92644 
92644 
92644 
92644 
92644 
473457879921
species
111111

111111

1841249 
1841249 
1841249 
1841249 
1841249 
1841249 
62511361377022
species
 1841249 

111111
 1725411 
species

1725411 
1725411 
1725411 
1725411 
1725411 
1725411 
554010812711627

111111
 1262452 

1262452 
1262452 
1262452 
1262452 
1262452 
1262452 
363049774013
species

111111

68249 
68249 
68249 
68249 
68249 
68249 
45521121508261
species
 68249 

111111
species
372973915622

164348 
164348 
164348 
164348 
164348 
164348 
 164348 


29303 
29303 
29303 
29303 
29303 
29303 
9711717818519256
species
 29303 
111111

111111

234612 
234612 
234612 
234612 
234612 
234612 
4246761326425
species
 234612 

111111
species

2202000 
2202000 
2202000 
2202000 
2202000 
2202000 
6934881428940
 2202000 

111111
species

1961713 
1961713 
1961713 
1961713 
1961713 
1961713 
281564645411
 1961713 

111111

1812480 
1812480 
1812480 
1812480 
1812480 
1812480 
40641121186534
species
 1812480 

species

565560 
565560 
565560 
565560 
565560 
565560 
5350941038422
 565560 
111111

111111

1914 
1914 
1914 
1914 
1914 
1914 
5940881486930
species
 1914 

111111
species
4055941179218

1642299 
1642299 
1642299 
1642299 
1642299 
1642299 
 1642299 

111111
species

2094021 
2094021 
2094021 
2094021 
2094021 
2094021 
4848741399335
 2094021 

 553510 
species
482874886419

553510 
553510 
553510 
553510 
553510 
553510 
111111

111111
 1972846 
species

1972846 
1972846 
1972846 
1972846 
1972846 
1972846 
8049691076127

111111
 1916 

1916 
1916 
1916 
1916 
1916 
1916 
38411241555520
species

111111
species

67267 
67267 
67267 
67267 
67267 
67267 
4541801057522
 67267 

species

146923 
146923 
146923 
146923 
146923 
146923 
5167621087213
 146923 
111111

 285473 

285473 
285473 
285473 
285473 
285473 
285473 
462570996922
species
111111

111111
 1038928 

1038928 
1038928 
1038928 
1038928 
1038928 
1038928 
3248421059432
species

111111
species
443072926140

68280 
68280 
68280 
68280 
68280 
68280 
 68280 

111111
 2184053 
species

2184053 
2184053 
2184053 
2184053 
2184053 
2184053 
46421111167857

111111

42684 
42684 
42684 
42684 
42684 
42684 
6265921449325
species
 42684 

111111
 54571 

54571 
54571 
54571 
54571 
54571 
54571 
163114288404228112
species

149150153151151148
order
7341100214016615671104854886
85007

family
775984176821811479504
1653
525254535250

525254535250
1716
775984176821811479504
genus

111111
 146827 
species

146827 
146827 
146827 
146827 
146827 
146827 
8162635234


225326 
225326 
225326 
225326 
225326 
225326 
202950832710
species
 225326 
111111

 42817 
species

42817 
42817 
42817 
42817 
42817 
42817 
3321114
111111

111111

1697 
1697 
1697 
1697 
1697 
1697 
7371367
species
 1697 

species

161896 
161896 
161896 
161896 
161896 
161896 
19213045302
 161896 
111111

 258224 
415171

258224 
258224 
258224 
258224 
258224 
258224 
species
111111

species
253052683310

349751 
349751 
349751 
349751 
349751 
349751 
 349751 
111111

species
8541641

160386 
160386 
160386 
160386 
160386 
160386 
 160386 
111111

111111
species

152794 
152794 
152794 
152794 
152794 
152794 
5102620179
 152794 

 1717 
species
293860787241

1717 
1717 
1717 
1717 
1717 
1717 
111111

111111

1737425 
1737425 
1737425 
1737425 
1737425 
1737425 
782227194
species
 1737425 

species
36551

1050174 
1050174 
1050174 
1050174 
1050174 
 1050174 
11111

11111
 1652495 

1652495 
1652495 
1652495 
1652495 
1652495 
2410132
species

species
302975996929

43771 
43771 
43771 
43771 
43771 
43771 
 43771 
111111

 1718 

1718 
1718 
1718 
1718 
1718 
1718 
8111119029716757
species
111111

111111
species

1404244 
1404244 
1404244 
1404244 
1404244 
1404244 
13152833136
 1404244 

 1721 
species

1721 
1721 
1721 
1721 

1721 
15661
11111

31427141

1487956 
1487956 
1487956 
1487956 
1487956 
1487956 
species
 1487956 
111111

 38301 
species

38301 
38301 
38301 
38301 
38301 
38301 
1412207364
111111

111111
 156978 
29166057528

156978 
156978 
156978 
156978 
156978 
156978 
species

11111


1724 
1724 
1724 
1724 
1724 
110792
species
 1724 

111111
 191493 

191493 
191493 
191493 
191493 
191493 
191493 
21804376309
species

 161879 

161879 


161879 
161879 
161879 
2432
species
1111

111111
 108486 
4461443

108486 
108486 
108486 
108486 
108486 
108486 
species

species
9143224115

1072256 
1072256 
1072256 
1072256 
1072256 
1072256 
 1072256 
111111

111111
 1231000 

1231000 
1231000 
1231000 
1231000 
1231000 
1231000 
71016164417
species

1062822145

136857 
136857 
136857 
136857 
136857 
136857 
species
 136857 
111111

111111
 169292 

169292 
169292 
169292 
169292 
169292 
169292 
316291672
species

 571915 
22

571915 

571915 
species
11

 35757 
11153

35757 
35757 
35757 
35757 
35757 
species
11111

species
183932734127

65058 
65058 
65058 
65058 
65058 
65058 
 65058 
111111

111111
species

161899 
161899 
161899 
161899 
161899 
161899 
651015113
 161899 

11111
 161895 
13138

161895 
161895 
161895 
161895 
161895 
species

111111
 28028 
species
251911372

28028 
28028 
28028 
28028 
28028 
28028 

111111
species

1719 
1719 
1719 
1719 
1719 
1719 
98932382759838
 1719 

species
1783320139

2079535 
2079535 
2079535 
2079535 
2079535 
2079535 
 2079535 
111111

111111
species

191610 
191610 
191610 
191610 
191610 
191610 
131114174510
 191610 

species

1727 
1727 
1727 
1727 
1727 
1727 
214573757013
 1727 
111111

111111
species
365167794330

1223514 
1223514 
1223514 
1223514 
1223514 
1223514 
 1223514 

111111
421422121

203263 
203263 
203263 
203263 
203263 
203263 
species
 203263 

 187491 

187491 
187491 
187491 
187491 
187491 
187491 
91123312511
species
111111

111111
 702967 

702967 
702967 
702967 
702967 
702967 
702967 
416913111
species

 92706 
3278193

92706 
92706 
92706 
92706 
92706 
92706 
species
111111

species
161543423011

38305 
38305 
38305 
38305 
38305 
38305 
 38305 
111111

111111
 1705 
species
4142231173

1705 
1705 
1705 
1705 
1705 
1705 

21154039337

1121358 
1121358 
1121358 
1121358 
1121358 
1121358 
species
 1121358 
111111

4312141


1408191 
1408191 
1408191 
1408191 
1408191 
species
 1408191 
11111

20224961205

43770 
43770 
43770 
43770 
43770 
43770 
species
 43770 
111111

111111
 401472 

401472 
401472 
401472 
401472 
401472 
401472 
19223029306
species

111111
 575200 
242534376327

575200 
575200 
575200 
575200 
575200 
575200 
species

 89154 
species
8153441257

89154 
89154 
89154 
89154 
89154 
89154 
111111

111111
species

2080740 
2080740 
2080740 
2080740 
2080740 
2080740 
201726422116
 2080740 

111111
 1230998 

1230998 
1230998 
1230998 
1230998 
1230998 
1230998 
202570663013
species


38289 
38289 
38289 
38289 
38289 
38289 
211129344515
species
 38289 
111111

 35755 


35755 


35755 
11
species
11

331335392512
family
316606
111111

genus
331335392512
286801
111111

111111
331335392512

286802 
286802 
286802 
286802 
286802 
286802 
species
 286802 

family
1914393130816409725901151
85025
323232323232

genus
450471726883786270
1817
777777

111111
 37329 

37329 
37329 
37329 
37329 
37329 
37329 
765816018615459
species

111111
 37332 

37332 
37332 
37332 
37332 
37332 
37332 
7512113822217456
species


37326 
37326 
37326 
37326 
37326 
37326 
12911113617214643
species
 37326 
111111

111111
 2213200 
species
352064593515

2213200 
2213200 
2213200 
2213200 
2213200 
2213200 

 455432 
species

455432 
455432 
455432 
455432 
455432 
455432 
6663537212838
111111

 37330 
294838416427

37330 
37330 
37330 
37330 
37330 
37330 
species
111111

111111
species

135487 
135487 
135487 
135487 
135487 
135487 
40501371318532
 135487 

252525252525
1827
genus
146434603009032141804881

111111
 1833 

1833 
1833 
1833 
1833 
1833 
1833 
15320022311229813776
species

species
166189247512

1045808 
1045808 
1045808 
1045808 
1045808 
1045808 
 1045808 
111111

111111
 679318 
161231341126

679318 
679318 
679318 
679318 
679318 
679318 
species


191292 
191292 
191292 
191292 
191292 
191292 
5026829710554
species
 191292 
111111

111111
 132919 
48471071138428

132919 
132919 
132919 
132919 
132919 
132919 
species

209242380468247143

37919 
37919 
37919 
37919 
37919 
37919 
species
 37919 
111111

111111
 1500843 
species

1500843 
1500843 
1500843 
1500843 
1500843 
1500843 
423559795415

111111
 1830 
species
14213832036323175

1830 
1830 
1830 
1830 
1830 
1830 

 1828 
4249741286937

1828 
1828 
1828 
1828 
1828 
1828 
species
111111

 38310 
species

38310 
38310 
38310 
38310 
38310 
38310 
262245543810
111111

111111
species

1653479 
1653479 
1653479 
1653479 
1653479 
1653479 
3675471204817
 1653479 

species

334542 
334542 
334542 
334542 
334542 
334542 
5651108554338
 334542 
111111

111111
 1033922 
species
453385996480

1033922 
1033922 
1033922 
1033922 
1033922 
1033922 

111111
 1564114 
species
622553814217

1564114 
1564114 
1564114 
1564114 
1564114 
1564114 

 1723645 
14381030452118

1723645 
1723645 
1723645 
1723645 
1723645 
1723645 
species
111111

 1727214 
species

1727214 
1727214 
1727214 
1727214 
1727214 
1727214 
28971005622316
111111

111111
 1990687 

1990687 
1990687 
1990687 
1990687 
1990687 
1990687 
335668944317
species

1088319520810828

43767 
43767 
43767 
43767 
43767 
43767 
species
 43767 
111111

 1570939 
species

1570939 
1570939 
1570939 
1570939 
1570939 
1570939 
3117501605713
111111

11212615818210465

103816 
103816 
103816 
103816 
103816 
103816 
species
 103816 
111111

382975945938

1829 
1829 
1829 
1829 
1829 
1829 
species
 1829 
111111

111111
392970543713

935199 
935199 
935199 
935199 
935199 
935199 
species
 935199 

 1805827 
species
5470941038939

1805827 
1805827 
1805827 
1805827 
1805827 
1805827 
111111

species
4643861086524

1653478 
1653478 
1653478 
1653478 
1653478 
1653478 
 1653478 
111111

species

1807790 
1807790 
1807790 
1807790 
1807790 
1807790 
1854887693012
 1807790 
111111

408938665851765353932896
family
1762
495051505150

670516
812608110513191059431
genus
555555

111111
 1578165 
species
161412371521

1578165 
1578165 
1578165 
1578165 
1578165 
1578165 

species

1520670 
1520670 
1520670 
1520670 
1520670 
1520670 
1041116117
 1520670 
111111

 36809 

36809 
36809 
36809 
36809 
36809 
36809 
74455810311194981382
species
111111

111111
 83262 

83262 
83262 
83262 
83262 
83262 
83262 
291433402612
species

111111
 1774 
species

1774 
1774 
1774 
1774 
1774 
1774 
13181832269

genus
104317315
697025
111111

 639313 

639313 
639313 
639313 
639313 
639313 
639313 
104317315
species
111111

2238561034654
genus
1073531
222222

111111
 1788 
species

1788 
1788 
1788 
1788 
1788 
1788 
7122451194


875328 
875328 
875328 
875328 
875328 
875328 
152632522750
species
 875328 
111111

1866885
genus
851681113413931018481
131313131313

111111
2665591003825

110539 
110539 
110539 
110539 
110539 
110539 
species
 110539 

111111
species

36814 
36814 
36814 
36814 
36814 
36814 
261925433313
 36814 

species
352950715228

1800 
1800 
1800 
1800 
1800 
1800 
 1800 
111111


1791 
1791 
1791 
1791 
1791 
1791 
373654664129
species
 1791 
111111

species
82325506511

758802 
758802 
758802 
758802 
758802 
758802 
 758802 
111111

species
353270404580377201

1772 
1772 
1772 
1772 
1772 
1772 
 1772 
111111

 134601 
5440110954932

134601 
134601 
134601 
134601 
134601 
134601 
species
111111

111111
species

1771 
1771 
1771 
1771 
1771 
1771 
513870737921
 1771 

 1766 

1766 
1766 
1766 
1766 
1766 
1766 
21841466819
species
111111

species

1804 
1804 
1804 
1804 
1804 
1804 
92661061229239
 1804 
111111

111111
 1797 

1797 
1797 
1797 
1797 
1797 
1797 
44244343212
species

 1795 
311563211618

1795 
1795 
1795 
1795 
1795 
1795 
species
111111

 1810 
species

1810 
1810 
1810 
1810 
1810 
1810 
734884838743
111111

239425353553482132391925
genus
1763
282930293029

111111

1781 
1781 
1781 
1781 
1781 
1781 
322141492840
species
 1781 

 1168287 
species

1168287 
1168287 
1168287 
1168287 
1168287 
1168287 
13101524217
111111

species

1570328 
1570328 
1570328 
1570328 
1570328 
1570328 
212336763812
 1570328 
111111

111111
species

164757 
164757 
164757 
164757 
164757 
164757 
612866642923
 164757 

111111
species
151218503413

212767 
212767 
212767 
212767 
212767 
212767 
 212767 

111111
 261524 
species

261524 
261524 
261524 
261524 
261524 
261524 
14921154


1920667 
1920667 
1920667 
1920667 
1920667 
1920667 
203746885325
species
 1920667 
111111

111111
 482462 

482462 
482462 
482462 
482462 
482462 
482462 
495686854165
species

492881865839

164756 
164756 
164756 
164756 
164756 
164756 
species
 164756 
111111

111111

1561223 
1561223 
1561223 
1561223 
1561223 
1561223 
20102329164
species
 1561223 

5107121


1769 
1769 
1769 
1769 
1769 
species
 1769 
11111

 1809 

1809 
1809 
1809 
1809 
1809 
1809 
20153139278
species
111111

 29311 
1271810102

29311 
29311 
29311 
29311 
29311 
29311 
species
111111

111111
 1273687 
species
27585277289

1273687 
1273687 
1273687 
1273687 
1273687 
1273687 

species
492237663224

1545728 
1545728 
1545728 
1545728 
1545728 
1545728 
 1545728 
111111

223232
77643
species group
8971036126218271325663

111111
 1765 
species

1765 
1765 
1765 
1765 
1765 
1765 
142232315813

 78331 
species
11


78331 

78331 
11

111111
 1773 
species
8831014122917961266650

1773 
1773 
1773 
1773 
1773 
1773 

923932142418671173876
species group
120793
666666

111111
5611252783734

339268 
339268 
339268 
339268 
339268 
339268 
species
 339268 

 701042 
species

701042 
701042 
701042 
701042 
701042 
701042 
161624462037
111111

 222805 

222805 
222805 
222805 
222805 
222805 
222805 
181260299418237215
species
111111

 1764 
413356700923588404

1764 
1764 
1764 
1764 
1764 
1764 
species
111111

223157329372257169

1767 
1767 
1767 
1767 
1767 
1767 
species
 1767 
111111

343120303417

1138383 
1138383 
1138383 
1138383 
1138383 
1138383 
species
 1138383 
111111

111111

189918 
189918 
189918 
189918 
189918 
189918 
27275177368
species
 189918 

111111
species
369649596419

1682113 
1682113 
1682113 
1682113 
1682113 
1682113 
 1682113 

111111
2249310
191420474022
species group

111111
 722731 
species
191420474022

722731 
722731 
722731 
722731 
722731 
722731 

species
293946486221

1768 
1768 
1768 
1768 
1768 
1768 
 1768 
111111

111111
species
303665595831

1879023 
1879023 
1879023 
1879023 
1879023 
1879023 
 1879023 

111111
44196766399

2051552 
2051552 
2051552 
2051552 
2051552 
2051552 
species
 2051552 

85026
family
303623792746503174
777777

303623792746503174
genus
2053
777777

111111
 2059875 
313776796320

2059875 
2059875 
2059875 
2059875 
2059875 
2059875 
species


2055 
2055 
2055 
2055 
2055 
2055 
9717523219714648
species
 2055 
111111

111111
182963644711

84595 
84595 
84595 
84595 
84595 
84595 
species
 84595 

 1136941 
414858735617

1136941 
1136941 
1136941 
1136941 
1136941 
1136941 
species
111111

111111
 337191 

337191 
337191 
337191 
337191 
337191 
337191 
46831051125845
species

 2054 
species
2722021417710620

2054 
2054 
2054 
2054 
2054 
2054 
111111

species

1004901 
1004901 
1004901 
1004901 
1004901 
1004901 
433144442713
 1004901 
111111

111111
genus
7113149190901
1847725


1528099 
1528099 
1528099 
1528099 
1528099 
1528099 
7113149190901
species
 1528099 
111111

85028
family
1089818923713960
222222

genus
1089818923713960
2060
222222

 2061 
species

2061 
2061 
2061 
2061 
2061 
2061 
443775824526
111111

111111
 57704 
species

57704 
57704 
57704 
57704 
57704 
57704 
64611141559434

85029
11239356652826688
family
555555

genus
11239356652826688
37914
555555

species

499555 
499555 
499555 
499555 
499555 
499555 
222485472712
 499555 
111111

111111

546160 
546160 
546160 
546160 
546160 
546160 
19951461626520
species
 546160 

111111
 712270 
352162372029226

712270 
712270 
712270 
712270 
712270 
712270 
species

 2052657 

2052657 
2052657 
2052657 
2052657 
2052657 
2052657 
201836392811
species
111111

111111
species
164062785419

139021 
139021 
139021 
139021 
139021 
139021 
 139021 

414714
60417713012741
order
111111

111111
414877
60417713012741
family

111111
414878
60417713012741
genus

111111
60417713012741

304895 
304895 
304895 
304895 
304895 
304895 
species
 304895 

335553
1361829187
order
2039638

335553
2162846
1361829187
family

122
2039639
1144
genus

11
 1884916 


1884916 
1884916 
122
species


1884915 
1884915 
1884915 
122
species
 1884915 
111

334333
622681
genus
1361715147

131421

1884913 
1884913 
1884913 
1884913 
1884913 
1884913 
species
 1884913 
111111

 1884914 
729935

1884914 
1884914 
1884914 
1884914 
1884914 
1884914 
species
111111

1
species


1884907 
1
 1884907 

111111
 1884905 
species
516291

1884905 
1884905 
1884905 
1884905 
1884905 
1884905 

333333
order
241184569456393124
1643682

333333
241184569456393124
family
85030

88138
genus
967115717416130
111111

111111
 477641 

477641 
477641 
477641 
477641 
477641 
477641 
967115717416130
species

111111
38501
genus
5854821369634

 138336 
5854821369634

138336 
138336 
138336 
138336 
138336 
138336 
species
111111

111111
875933014613660
genus
1860

 1861 
875933014613660

1861 
1861 
1861 
1861 
1861 
1861 
species
111111

111111
85014
order
352545533611

111111
85034
352545533611
family

111111
352545533611
genus
283810

111111
352545533611

283811 
283811 
283811 
283811 
283811 
283811 
species
 283811 

85006
392831868664406686643095314395
order
991001009910099

444444
family
635422113912701062284
85016

635422113912701062284
genus
1707
444444

 1708 
221157405419361100

1708 
1708 
1708 
1708 
1708 
1708 
species
111111

 2003551 
species

2003551 
2003551 
2003551 
2003551 
2003551 
2003551 
15311927033626966
111111

111111
 1711 
species
1248326228325565

1711 
1711 
1711 
1711 
1711 
1711 


11 
11 
11 
11 
11 
11 
1376320223217753
species
 11 
111111

2038
genus
1541627312
111111


2039 
2039 
2039 
2039 
2039 
2039 
1541627312
species
 2039 
111111

family
1721611810821597072232877
145360
111111

111111
1721611810821597072232877
genus
60919

111111
 60920 
species
1721611810821597072232877

60920 
60920 
60920 
60920 
60920 
60920 

85018
762217135
family
111111

111111
1862
762217135
genus

111111
 1863 
species

1863 
1863 
1863 
1863 
1863 
1863 
762217135

111111
family
727916216512148
85019

727916216512148
genus
1696
111111

 1703 

1703 
1703 
1703 
1703 
1703 
1703 
727916216512148
species
111111

family
276335586693478149
85021
444444

111111
53457
8415820421917539
genus

 857417 
species

857417 
857417 
857417 
857417 
857417 
857417 
8415820421917539
111111

265976
73481071318526
genus
111111

111111
 1758689 

1758689 
1758689 
1758689 
1758689 
1758689 
1758689 
73481071318526
species

111111
genus
796214019512156
267408

111111

1658671 
1658671 
1658671 
1658671 
1658671 
1658671 
796214019512156
species
 1658671 

53357
40671351489728
genus
111111

111111
 53358 
species

53358 
53358 
53358 
53358 
53358 
53358 
40671351489728

1268
family
15322007330354832808857
313131313131

111111
1868332
671242325
genus

 556325 

556325 
556325 
556325 
556325 
556325 
556325 
671242325
species
111111

12312326535816446
genus
596707
111111

111111
 37927 
species

37927 
37927 
37927 
37927 
37927 
37927 
12312326535816446

57493
genus
262257568714413171
444444

111111
 71999 
species
5610116919513871

71999 
71999 
71999 
71999 
71999 
71999 

111111
species

1702043 
1702043 
1702043 
1702043 
1702043 
1702043 
4239761277115
 1702043 

 72000 
species
916417120812044

72000 
72000 
72000 
72000 
72000 
72000 
111111

 446860 
species
73531521848441

446860 
446860 
446860 
446860 
446860 
446860 
111111

1663
genus
697759129524021177367
151515151515

781281272949534

1849032 
1849032 
1849032 
1849032 
1849032 
1849032 
species
 1849032 
111111

111111
 1690248 
46357311710624

1690248 
1690248 
1690248 
1690248 
1690248 
1690248 
species

111111

1618207 
1618207 
1618207 
1618207 
1618207 
1618207 
16720392114
species
 1618207 

3534621424214

2020486 
2020486 
2020486 
2020486 
2020486 
2020486 
species
 2020486 
111111


1704044 
1704044 
1704044 
1704044 
1704044 
1704044 
704210214313226
species
 1704044 
111111

 1494608 
3738751155718

1494608 
1494608 
1494608 
1494608 
1494608 
1494608 
species
111111

111111
species

37928 
37928 
37928 
37928 
37928 
37928 
432462116738
 37928 

 1771959 
2325451123013

1771959 
1771959 
1771959 
1771959 
1771959 
1771959 
species
111111

111111
4751731797522

2079227 
2079227 
2079227 
2079227 
2079227 
2079227 
species
 2079227 

928817526915075

656366 
656366 
656366 
656366 
656366 
656366 
species
 656366 
111111

111111
 290399 
species
55831002498723

290399 
290399 
290399 
290399 
290399 
290399 

111111
3347771355918

1806905 
1806905 
1806905 
1806905 
1806905 
1806905 
species
 1806905 

111111

1118963 
1118963 
1118963 
1118963 
1118963 
1118963 
4242761425140
species
 1118963 

111111

1357915 
1357915 
1357915 
1357915 
1357915 
1357915 
526912821111122
species
 1357915 

28461001398816

1652545 
1652545 
1652545 
1652545 
1652545 
1652545 
species
 1652545 
111111

111111
1645
7101826710
genus

species
7101826710

1646 
1646 
1646 
1646 
1646 
1646 
 1646 
111111

222222
genus
421141393308526
32207

111111
 43675 
30104983078021

43675 
43675 
43675 
43675 
43675 
43675 
species

 2047 
species
1210412355

2047 
2047 
2047 
2047 
2047 
2047 
111111

111111
genus
3042651254116
1742992

species

43663 
43663 
43663 
43663 
43663 
43663 
3042651254116
 43663 
111111

333333
1742993
168238319735482101
genus

 361575 
species
3245801496912

361575 
361575 
361575 
361575 
361575 
361575 
111111

111111
 85085 
species

85085 
85085 
85085 
85085 
85085 
85085 
667812736831651

 121292 
species
701151122189738

121292 
121292 
121292 
121292 
121292 
121292 
111111

111111
1269
genus
13340252361928684

 1270 
species
13340252361928684

1270 
1270 
1270 
1270 
1270 
1270 
111111

1742989
genus
64559913212131
222222

111111
 1933880 

1933880 
1933880 
1933880 
1933880 
1933880 
1933880 
252241755411
species

species
393358576720

256701 
256701 
256701 
256701 
256701 
256701 
 256701 
111111

111111
85022
family
84151127011141

111111
43673
84151127011141
genus

 43674 

43674 
43674 
43674 
43674 
43674 
43674 
84151127011141
species
111111

85020
332261720711442216
family
777777

111111
472568
genus
691211115

111111
species
691211115

472569 
472569 
472569 
472569 
472569 
472569 
 472569 

555555
317245684682416207
genus
43668

species
756818117510574

43669 
43669 
43669 
43669 
43669 
43669 
 43669 
111111

 2017484 
663382938622

2017484 
2017484 
2017484 
2017484 
2017484 
2017484 
species
111111

 1331682 
species
91681891817845

1331682 
1331682 
1331682 
1331682 
1331682 
1331682 
111111

 1903186 
species

1903186 
1903186 
1903186 
1903186 
1903186 
1903186 
49431441197749
111111

species

2017485 
2017485 
2017485 
2017485 
2017485 
2017485 
3633881147017
 2017485 
111111

111111
genus
972418154
36739

111111
 1630135 
972418154

1630135 
1630135 
1630135 
1630135 
1630135 
1630135 
species

222222
125316
263172433517434128
family

111111
1288820224318260
genus
947525

111111
 2171623 

2171623 
2171623 
2171623 
2171623 
2171623 
2171623 
1288820224318260
species

84756
genus
1358423127425268
111111

species

84757 
84757 
84757 
84757 
84757 
84757 
1358423127425268
 84757 
111111

555555
1022409163816121505400
family
85017

367172657659534165
genus
254250
222222

 372663 
1726832130721681

372663 
372663 
372663 
372663 
372663 
372663 
species
111111

111111
species

139208 
139208 
139208 
139208 
139208 
139208 
19510433635231884
 139208 

222222
491172761706749181
genus
157920

 1710 
species

1710 
1710 
1710 
1710 
1710 
1710 
2389443934336091
111111

111111
 1980001 
2537832236338990

1980001 
1980001 
1980001 
1980001 
1980001 
1980001 
species

111111
186188
1646522024722254
genus

111111

186189 
186189 
186189 
186189 
186189 
186189 
1646522024722254
species
 186189 

85023
17675145064496151651164309315
family
383939383938

518733
genus
286303737968309130
111111

 412690 
286303737968309130

412690 
412690 
412690 
412690 
412690 
412690 
species
111111

genus
630144879145931128204528
2034
333333

 1905847 
22071635341332499411686

1905847 
1905847 
1905847 
1905847 
1905847 
1905847 
species
111111

111111

1561023 
1561023 
1561023 
1561023 
1561023 
1561023 
16521120219026028021024
species
 1561023 

111111
 69373 

69373 
69373 
69373 
69373 
69373 
69373 
244217323542346010771818
species

222222
genus
212145975514
1705353

111111
 708131 
6122740264

708131 
708131 
708131 
708131 
708131 
708131 
species

111111

1987356 
1987356 
1987356 
1987356 
1987356 
1987356 
15918572910
species
 1987356 

111111
1434032
117181846
genus

111111
 1159327 
species

1159327 
1159327 
1159327 
1159327 
1159327 
1159327 
117181846

76634
256241575741208112
genus
111111

111111
species

2079792 
2079792 
2079792 
2079792 
2079792 
2079792 
256241575741208112
 2079792 

131313131313
33882
42742915156811203636211419
genus

111111
21323967670423483

36805 
36805 
36805 
36805 
36805 
36805 
species
 36805 

111111
 1938334 
2001601450112135681

1938334 
1938334 
1938334 
1938334 
1938334 
1938334 
species

species
21022462175029593

162426 
162426 
162426 
162426 
162426 
162426 
 162426 
111111

111111
 1714373 
species
215226683787261105

1714373 
1714373 
1714373 
1714373 
1714373 
1714373 

111111
 1916917 
species
22521527691199294166

1916917 
1916917 
1916917 
1916917 
1916917 
1916917 

111111
species
270253883889263105

2033 
2033 
2033 
2033 
2033 
2033 
 2033 

111111
21323169680026884

300019 
300019 
300019 
300019 
300019 
300019 
species
 300019 

 2103230 
species

2103230 
2103230 
2103230 
2103230 
2103230 
2103230 
25923413621157289115
111111

111111
species

84292 
84292 
84292 
84292 
84292 
84292 
20919055756420778
 84292 


1795053 
1795053 
1795053 
1795053 
1795053 
1795053 
20520012481065284107
species
 1795053 
111111

111111
 367477 

367477 
367477 
367477 
367477 
367477 
367477 
2102401155853268111
species

111111
 1906742 

1906742 
1906742 
1906742 
1906742 
1906742 
1906742 
1596272829972325140
species

111111
 1696072 

1696072 
1696072 
1696072 
1696072 
1696072 
1696072 
24923127521175277151
species

29832574105421532438381386
genus
1573
555555

111111
 31964 
33131511521766442146

31964 
31964 
31964 
31964 
31964 
31964 
species

 31963 
36127612911865459157

31963 
31963 
31963 
31963 
31963 
31963 
species
111111

111111
37635014201921466173

28447 
28447 
28447 
28447 
28447 
28447 
species
 28447 

species

1874630 
1874630 
1874630 
1874630 
1874630 
1874630 
41333212361872428180
 1874630 
111111

111111
species
15021301544379002043730

33014 
33014 
33014 
33014 
33014 
33014 
 33014 

111111
3193077581144450161
genus
1759331

3193077581144450161

1619308 
1619308 
1619308 
1619308 
1619308 
1619308 
species
 1619308 
111111

222222
genus
66669818642460841295
33877

111111
species
3032958631064358132

2080742 
2080742 
2080742 
2080742 
2080742 
2080742 
 2080742 

species
36340310011396483163

453304 
453304 
453304 
453304 
453304 
453304 
 453304 
111111

111111
337004
genus
15316243051520966

 279828 

279828 
279828 
279828 
279828 
279828 
279828 
15316243051520966
species
111111

genus
59550913372203734277
69578
222222

111111
species

1978566 
1978566 
1978566 
1978566 
1978566 
1978566 
2952356071023339107
 1978566 

3002747301180395170

670052 
670052 
670052 
670052 
670052 
670052 
species
 670052 
111111

6371108112230101832379
genus
447237
111111

111111
 1795630 

1795630 
1795630 
1795630 
1795630 
1795630 
1795630 
6371108112230101832379
species

122121
529883
genus
43105113

 529884 


529884 
529884 

529884 
145
species
111

 535712 
species

535712 
535712 
535712 
535712 
535712 
535712 
426563
111111

genus
819423932712632
235888
111111

111111
819423932712632

2079791 
2079791 
2079791 
2079791 
2079791 
2079791 
species
 2079791 

genus
4313637761176464194
33886
222222

325275591896329136

33888 
33888 
33888 
33888 
33888 
33888 
species
 33888 
111111

111111
species
1068818528013558

145458 
145458 
145458 
145458 
145458 
145458 
 145458 

111111
110932
65771416822316908313
genus


1575 
1575 
1575 
1575 
1575 
1575 
65771416822316908313
species
 1575 
111111

145357
family
15435249347829573
333333

111111
genus
767312915410527
57495

111111
 1274 
species
767312915410527

1274 
1274 
1274 
1274 
1274 
1274 

57499
genus
302091941568213
111111

111111
 1276 

1276 
1276 
1276 
1276 
1276 
1276 
302091941568213
species

745364
487017016810833
genus
111111

487017016810833

571913 
571913 
571913 
571913 
571913 
571913 
species
 571913 
111111

order
302865170682298699015267978
85009
222222222222

family
248664709671498565314444672
31957
141414141414

333333
1912216
genus
171663359649778293313045265

111111
 33010 
42921341724916

33010 
33010 
33010 
33010 
33010 
33010 
species

111111
 33011 
species
7393240261

33011 
33011 
33011 
33011 
33011 
33011 

166763228648118272112970248

1747 
1747 
1747 
1747 
1747 
1747 
species
 1747 
111111

111111
genus
64451181578817
29404

species
64451181578817

29405 
29405 
29405 
29405 
29405 
29405 
 29405 
111111

222222
33494912741577657182
genus
1743

species

671223 
671223 
671223 
671223 
671223 
671223 
237096136771237
 671223 
111111

111111
 1744 

1744 
1744 
1744 
1744 
1744 
1744 
311240661900534175
species

203133
35131321
genus
111111

 1871034 
species

1871034 
1871034 
1871034 
1871034 
1871034 
1871034 
35131321
111111

72763
187178421528320106
genus
555555

 1909732 
392988916514

1909732 
1909732 
1909732 
1909732 
1909732 
1909732 
species
111111

111111
 1610493 
species
293076766419

1610493 
1610493 
1610493 
1610493 
1610493 
1610493 

 399497 
species
50551171678736

399497 
399497 
399497 
399497 
399497 
399497 
111111

111111
 1332264 
5242961298220

1332264 
1332264 
1332264 
1332264 
1332264 
1332264 
species

species
172244652217

2161816 
2161816 
2161816 
2161816 
2161816 
2161816 
 2161816 
111111

1912215
genus
16215431542027273
111111

16215431542027273

1748 
1748 
1748 
1748 
1748 
1748 
species
 1748 
111111

111111
201931256028
genus
1912217


1750 
1750 
1750 
1750 
1750 
1750 
201931256028
species
 1750 
111111

85015
54246110801337823306
family
888888

111111
2044
888618520914984
genus

species
888618520914984

2045 
2045 
2045 
2045 
2045 
2045 
 2045 
111111

genus
5324771036625
116071
111111

 75385 
species
5324771036625

75385 
75385 
75385 
75385 
75385 
75385 
111111

111111
182639
60451501669743
genus

111111
species

182640 
182640 
182640 
182640 
182640 
182640 
60451501669743
 182640 

genus
17217235544627581
1839
333333


196162 
196162 
196162 
196162 
196162 
196162 
83881401838826
species
 196162 
111111

111111
 2045452 
species
2938991006523

2045452 
2045452 
2045452 
2045452 
2045452 
2045452 

111111

450734 
450734 
450734 
450734 
450734 
450734 
604611616312232
species
 450734 

2040
16913431341323673
genus
222222

111111
species
886614918512231

2041 
2041 
2041 
2041 
2041 
2041 
 2041 

111111
 2079793 
species
816816422811442

2079793 
2079793 
2079793 
2079793 
2079793 
2079793 

order
28115226735229583
622452
111111

28115226735229583
family
83778
111111

111111
28115226735229583
genus
33981

111111
 131568 
species
28115226735229583

131568 
131568 
131568 
131568 
131568 
131568 

131514151514
2037
order
322291608866525164

131514151514
family
322291608866525164
2049

11111
genus
32675
28263

11111
 28264 
species

28264 
28264 
28264 
28264 
28264 
32675

671916164
genus
1653174
222222

111111
 59505 

59505 
59505 
59505 
59505 
59505 
59505 
544461
species

 2171980 
species
131512103

2171980 
2171980 
2171980 
2171980 
2171980 
2171980 
111111

9109101010
242239484690412132
genus
1654

3568881137917

544580 
544580 
544580 
544580 
544580 
544580 
species
 544580 
111111


1912795 

1912795 
1912795 
1912795 
7263
species
 1912795 
1111

111111
 111015 
53351071569029

111015 
111015 
111015 
111015 
111015 
111015 
species


2081702 
2081702 
2081702 
2081702 
2081702 
2081702 
19162736277
species
 2081702 
111111

342650815413

1960083 
1960083 
1960083 
1960083 
1960083 
1960083 
species
 1960083 
111111

111111

1852377 
1852377 
1852377 
1852377 
1852377 
1852377 
1445231615
species
 1852377 

111111
species

2079536 
2079536 
2079536 
2079536 
2079536 
2079536 
252877104268
 2079536 

species

52773 
52773 
52773 
52773 
52773 
52773 
7152925189
 52773 
111111

111111
 712122 
282343795818

712122 
712122 
712122 
712122 
712122 
712122 
species

 1851395 
species
271758713813

1851395 
1851395 
1851395 
1851395 
1851395 
1851395 
111111

111111
7141971449020
genus
1069494


1661 
1661 
1661 
1661 
1661 
1661 
7141971449020
species
 1661 
111111

11111
22928
genus
2050

11111
species
22928


2051 
2051 
2051 
2051 
2051 
 2051 

191935462215
order
622450
111111

111111
622451
family
191935462215

1849
genus
191935462215
111111

 414996 
species

414996 
414996 
414996 
414996 
414996 
414996 
191935462215
111111

2216571092526
class
84995
212222

212222
order
2216571092526
84996

212222
2216571092526
family
84997

212222
42255
2216571092526
genus

111111
species

49319 
49319 
49319 
49319 
49319 
49319 
201650942223
 49319 

11111
 42256 

42256 

42256 
42256 
42256 
42256 
271533
species

111111
84992
641316146
class

111111
84993
order
641316146

111111
641316146
family
84994

111111
641316146
genus
53634

 53635 
species
641316146

53635 
53635 
53635 
53635 
53635 
53635 
111111


1848255 
1
species
 1848255 
1

84998
6984911289879
class
101013131111

84999
161412262313
order
456665

233334
3761599
family
1643824

133925
3751598
genus
232333

111111

1805478 
1805478 
1805478 
1805478 
1805478 
1805478 
133634
species
 1805478 

111111
species

712411 
712411 
712411 
712411 
712411 
712411 
222721
 712411 

1111
 133926 


133926 

133926 
133926 
133926 
2243
species

11
genus
11
1380

species


1382 


1382 
11
 1382 
11

family
137611144
84107
223331

11111
 1531429 
species
83293

1531429 
1531429 
1531429 
1531429 
1531429 

111111
102106
543174
genus

species

74426 
74426 
74426 
74426 
74426 
74426 
543174
 74426 
111111

111
genus
114
33870

111
 33871 
species
114


33871 
33871 
33871 

657756
1643822
5370791027566
order

657756
1643826
5370791027566
family

222222
genus
14621431730
644652

 1841863 
species
541627725

1841863 
1841863 
1841863 
1841863 
1841863 
1841863 
111111

111111
92516105

1335613 
1335613 
1335613 
1335613 
1335613 
1335613 
species
 1335613 

genus
1771
447020
1111

1111
 446660 
1771

446660 

446660 
446660 

446660 
species

84162
9
genus
1

species


84163 
9
 84163 
1

222211
84111
genus
24164133378

1111
 502558 

502558 
502558 
502558 
502558 
1171
species

species
23153432378

84112 
84112 
84112 
84112 
84112 
84112 
 84112 
111111

111
79603
221
genus

111
species
221


79604 

79604 
79604 
 79604 

111111
14488101926
genus
84108

14488101926

84110 
84110 
84110 
84110 
84110 
84110 
species
 84110 
111111

species
11


1848755 

1848755 
 1848755 
11

class
5741651026537
1497346
111111

5741651026537
order
588673
111111

family
5741651026537
320583
111111

111111
genus
5741651026537
191494

 191495 
species

191495 
191495 
191495 
191495 
191495 
191495 
5741651026537
111111

1


1848756 
species
 1848756 
1

656072666860
superkingdom
388412478342346529
2157

423234
28889
77114421
phylum

77114421
class
183924
423234

871006
1
order
1

1
family
255472
1

200414
1
genus
1

1


200415 
1
species
 200415 

312112
2281
313113
order

313113
family
118883
312112

genus
11
69655
11

11
species


111955 

111955 
11
 111955 

31211
genus
31312
2284

1
species

43080 
1
 43080 

 2285 

2285 

2285 

2285 
2285 
1212
species
1111

111

2287 
2287 
2287 
111
species
 2287 

111112
order
4683218
114380

4683218
family
2307
111112

1
genus
2308
1

1
 1273541 
species
1


1273541 

4683217
genus
54251
111111

111111
species
4683217

54252 
54252 
54252 
54252 
54252 
54252 
 54252 

phylum
5273
651137
2221

1
31932
1
order

1
family
1
338190

1
genus
1
338191

1
 1229909 
species
1


1229909 

1111
1825023
4133
genus


1846278 
1846278 
1846278 
1846278 
4133
species
 1846278 
1111

class
14
1643678
11

4
order
1033996
1

1
family
4
1033997

497726
4
genus
1

species


1034015 
4
 1034015 
1

1968909
order
1
1

1968910
1
family
1

1
1
genus
498374

1
 2045011 


2045011 
1
species

615667626456
phylum
381400465331339508
28890

322522
183968
4881025
class

322522
order
4881025
2258

family
4881025
2259
322522

211321
genus
311821
2263

 54077 
species
1


54077 
1

1
 72803 


72803 
1
species

species
11

342948 


342948 
 342948 
11

11111
species

2008440 
2008440 
2008440 
2008440 

2008440 
21161
 2008440 

1
 311400 
1


311400 
species

17724
genus
2260
11121

species

2261 
2261 
2261 
2261 

2261 
17714
 2261 
11111

1
 53953 
species
1


53953 

313137343226
183963
15021121016515192
class

order
538077684831
2235
11131515149

1963268
497652373228
family
811111097

12221
431093
genus
146825

 146826 
species
41543

146826 
146826 
146826 
146826 

146826 
11111

111
species
255


430914 
430914 
430914 
 430914 

111111
141333214
genus
1073987

 1932360 

1932360 
1932360 
1932360 
1932360 
1932360 
1932360 
141333214
species
111111

344443
24182117226
genus
2237


2238 
2238 
2238 
2238 
2238 
24632
species
 2238 
11111

21821


1592728 
1592728 
1592728 
1592728 
1592728 
species
 1592728 
11111

111111

51589 
51589 
51589 
51589 
51589 
51589 
1029331
species
 51589 

111111
species
121053154

1932004 
1932004 
1932004 
1932004 
1932004 
1932004 
 1932004 

1111
 1679096 


1679096 
1679096 
1679096 
1679096 
2223
species

222121
genus
63513143
63743

 2257 
534313

2257 
2257 
2257 
2257 
2257 
species
11111

11111
 416273 
species
111013

416273 
416273 
416273 

416273 
416273 

203135
153512
genus
111111

111111
 57705 

57705 
57705 
57705 
57705 
57705 
57705 
153512
species

324552
2236
family
442531163

212028122
genus
2239
212331

species


751944 
751944 
751944 
751944 
71332
 751944 
1111

 1407499 

1407499 
1407499 
1407499 
1407499 
1407499 
1113127
species
11111

111
species
132

2242 


2242 
2242 
 2242 

1980514
genus
23111
11111

11111
species
23111

1873524 
1873524 
1873524 
1873524 
1873524 
 1873524 

1111
4231
genus
1656823

1111
species


1604004 
1604004 
1604004 
1604004 
4231
 1604004 

1644060
order
779676465941
11101210109

11101210109
1644061
779676465941
family

11111

1902251 
1902251 
1902251 

1902251 
1902251 
9101115
species
 1902251 

353799
182512
genus
11111

species

353800 
353800 
353800 
353800 
353800 
182512
 353800 
11111

genus
452172522
63742
111111

111111

13769 
13769 
13769 
13769 
13769 
13769 
452172522
species
 13769 

519434
genus
1269201
111111

species
519434

1333523 
1333523 
1333523 
1333523 
1333523 
1333523 
 1333523 
111111

203193
21225397
genus
111111

 229731 

229731 
229731 
229731 
229731 
229731 
229731 
21225397
species
111111

11111
29287
112414
genus

11111

29288 

29288 
29288 
29288 
29288 
112414
species
 29288 

111111
387342
genus
21131122

 387343 
21131122

387343 
387343 
387343 
387343 
387343 
387343 
species
111111

1111
2256
genus
2221

1111
 44930 
2221

44930 
44930 
44930 
44930 
species

105113121
genus
88723
222121

 406552 
species

406552 
406552 
406552 
406552 
406552 
341033
11111

11111
 69525 
species
71191

69525 
69525 
69525 

69525 
69525 

genus
5112646
121871
112212

111
 588898 
species
312


588898 
588898 

588898 

111111
 62320 

62320 
62320 
62320 
62320 
62320 
62320 
519544
species

9810988
203557514420
order
1644055

11111
species

756883 
756883 
756883 
756883 
756883 
251621
 756883 

444444
1963271
family
1162222176

111111
genus
215651
1209988

111111
species
215651

1048396 
1048396 
1048396 
1048396 
1048396 
1048396 
 1048396 

111111
genus
3346103
1644057

111111
 755307 
3346103

755307 
755307 
755307 
755307 
755307 
755307 
species

11
21
genus
1450140

 1073996 
21

1073996 


1073996 
species
11

122221
42131021
genus
56688

1111
1141


2247 
2247 
2247 
2247 
species
 2247 

111111

29284 
29284 
29284 
29284 
29284 
29284 
4112611
species
 29284 

435434
1644056
72419272614
family

1321124
genus
60846
111111

111111
 60847 
1321124

60847 
60847 
60847 
60847 
60847 
60847 
species

2
genus
293431
1

species
2


293091 
 293091 
1

genus
62115261410
2251
323323


2246 
2246 
2246 
2246 
2246 
2246 
11741494
species
 2246 
111111

species

2252 

2252 
2252 

2252 
4142
 2252 
1111

111111
 35746 
1410854

35746 
35746 
35746 
35746 
35746 
35746 
species

183980
class
1
1

order
1
2231
1

1
family
2232
1

2233
genus
1
1

species
1


2234 
 2234 
1

434344
183939
202233102052
class

order
202233102052
2182
434344

434344
2183
202233102052
family

2184
192233101952
genus
334334

111111
 2188 

2188 
2188 
2188 
2188 
2188 
2188 
86154426
species

111111
 42879 

42879 
42879 
42879 
42879 
42879 
42879 
1142314
species

species
10151341211

39152 
39152 
39152 
39152 
39152 
39152 
 39152 
111111

11
species
11


2187 


2187 
 2187 

155862
genus
11
11

 155863 

155863 


155863 
11
species
11

131317151817
103761106498199
class
224756

2191
412926222653
order
645473

111111
family
1771681335
88404

genus
1771681335
2192
111111

 83984 
1771681335

83984 
83984 
83984 
83984 
83984 
83984 
species
111111

2232
family
1198451
1211

111
395331
genus
132

 358766 
species


358766 
358766 
13
11

1


882104 
2
species
 882104 

475087
21
genus
11

11
species
21

475088 

475088 
 475088 

family
22208111018
2194
422242

45989
genus
10112453
311131

 86622 

86622 


86622 
22
species
11

 2198 
711213

2198 
2198 
2198 

2198 
2198 
species
11111

111
species
142

83986 


83986 
83986 
 83986 

genus
12967515
230355
111111

 54120 
species

54120 
54120 
54120 
54120 
54120 
54120 
12967515
111111

11
family
21
196137

21
genus
2202
11

11


2203 


2203 
21
species
 2203 

221
order
11118
570264

family
11118
570265
221

221
570266
11118
genus

species


1175445 
1175445 
81
 1175445 
11

 1175444 
3108


1175444 
1175444 
1175444 
species
111

79129913
94695
order
6247843161138

79118913
2206
family
6247832961138

1112
2225
1512
genus

1
species


29291 
1
 29291 

 2226 
1511


2226 
2226 
2226 

2226 
species
1111

6245762861135
genus
2207
7797910

11111
44225


1434103 
1434103 
1434103 
1434103 
1434103 
species
 1434103 

11
 1434099 
species


1434099 


1434099 
21

species
2152


38027 
38027 
38027 
38027 
 38027 
1111

11111
 1434100 
species

1434100 
1434100 

1434100 
1434100 
1434100 
53322

 1434102 
696117

1434102 
1434102 
1434102 

1434102 
1434102 
species
11111

111111
species
36183282183

2208 
2208 
2208 
2208 
2208 
2208 
 2208 

species
1111

2214 
2214 


2214 
2214 
 2214 
1111

11
 418008 

418008 


418008 
11
species

species
3


2215 
 2215 
1

species


2210 
2210 

2210 
611
 2210 
111

 170861 
7516774

170861 
170861 
170861 
170861 
170861 
170861 
species
111111

111111
 2209 
species

2209 
2209 
2209 
2209 
2209 
2209 
65561129

11
11
genus
2220

11


420950 


420950 
11
species
 420950 

101191
genus
2
1

species


101192 
2
 101192 
1

11
143067
family
12

11
12
genus
2222

1
species


301375 
2
 301375 

1
 2223 


2223 
1
species

8970967855156
class
183925
755366

755366
order
8970967855156
2158

2178
1
family
1

1
1
genus
2179

1

2180 
1
species
 2180 

655366
family
8870967855156
2159

genus
534456412980
2172
433234

1
 224719 
species
2


224719 

111111
species

83816 
83816 
83816 
83816 
83816 
83816 
474269
 83816 

 294671 

294671 
294671 
294671 

294671 
294671 
41215
species
11111

1

230361 
1
species
 230361 

111111

2173 
2173 
2173 
2173 
2173 
2173 
443650392264
species
 2173 

11121
11121
genus
2160

1
 877455 
species


877455 
1

1
species


59277 
1
 59277 

1
species
1


1911685 
 1911685 

species
111

2162 
2162 


2162 
 2162 
111

111111
145260
genus
342539372475

species

145261 
145261 
145261 
145261 
145261 
145261 
342539372475
 145261 
111111

class
151383134
183967
322121

order
141183114
2301
212111

 1054217 
species
8

1054217 
1

112111
family
61183114
46659

61183114
genus
2302
112111

111111
 50339 
species

50339 
50339 
50339 
50339 
50339 
50339 
61173114

 2303 
species
1


2303 
1

species


1495144 
2
 1495144 
1

11
order
12
1235850

11
12
family
1577788

1291539
12
genus
11

 1291540 
species

1291540 
1291540 
12
11
