# Supplementary material for: Characterisation of the Carpinus betulus L. Phyllomicrobiome in Urban and Forest Areas
Source: Front Microbiol. 2019 May 29;10:1110. doi: 10.3389/fmicb.2019.01110 (PMC6549492; doi:10.3389/fmicb.2019.01110)
Supplement: Supplementary file 2 [file Data_Sheet_1.ZIP › CLARK_epBo.html]

Javascript must be enabled to view this page.

members
magnitude
magnitudeUnassigned
count
unassigned
taxon
rank

epBo13
epBo14
epBo23
epBo24
epBo32
epBo33

212294721959951082361150317325204151794425
271723722571272725072501

134663311681136662991
2157
superkingdom
725670907074

1465111316
28889
531642
phylum

1465111316
class
531642
183924

871006
order
1
20

20
1
family
255472

200414
genus
1
20

20
1
species
 200415 


200415 

93695
114380
21111
order

family
1
2272
5

5
54258
genus
1


54259 
5
 54259 
1
species

43695
family
11111
2307

43695
54251
genus
11111


54252 
54252 

54252 
54252 
54252 
11111
species
 54252 
43695

32321
order
2281
1372321

118883
32321
family
1372321

41980
1
genus
1


1006005 
1
 1006005 
1
species

1341221
2284
genus
11221

11
species
 2285 
1341

2285 


2285 


43080 

43080 
43080 
43080 
1111
1111
species
 43080 


2287 
1
species
1
 2287 

69655
genus
111
211

 111955 
111
species
211

111955 
111955 

111955 

2266
order
12
12

1
114378
1
family

1
genus
1
2268


1483572 
species
1
 1483572 
1

family
11
2267
11

genus
11
2270
11

11
species
11
 184117 


184117 
184117 

4265
phylum
2133
651137

2
genus
1
1593364


1410606 
2
 1410606 
species
1

3
genus
1
1825023


1846278 
3
1
species
 1846278 

class
2121
1643678
4241

1033996
order
1111
1231

1231
family
1111
1033997

1231
497726
1111
genus

1111
species
 1034015 
1231

1034015 
1034015 

1034015 

1034015 

31
11
order
1968909

31
family
11
1968910

genus
11
498374
31


2045011 


2045011 
 2045011 
11
species
31

1
order
1
31932

338190
family
1
1

1
1
genus
338191

1
1
species
 1580092 


1580092 

28890
655269816669
phylum
119662611671119631980

183939
class
444435
232814183016

2182
444435
order
232814183016

196117
12
family
33

196118
genus
12
33

2
species
1
 67760 


67760 

31
 73913 
species
11

73913 


73913 

2183
family
344433
202814183013

3211
genus
1111
155862

1111
species
 155863 
3211


155863 
155863 
155863 
155863 

202512172913
2184
genus
333323


42879 
42879 
42879 
42879 

42879 
 42879 
11111
species
31114


2188 
2188 
2188 
2188 
2188 
2188 
111111
species
 2188 
51047195


39152 
39152 
39152 
39152 
39152 
39152 
111111
species
 39152 
121479104

183963
282336333532
class
9488143160151138

171836434232
order
858899
1644055

7526281113
1963271
family
423445

21952
genus
11111
1209988


1048396 
1048396 

1048396 
1048396 
1048396 
21952
species
11111
 1048396 

genus
11
1450140
11

 1073996 
species
11
11


1073996 
1073996 

22212
genus
56688
421012


29284 

29284 
29284 

29284 
1111
species
 29284 
3171

11311
species
11111
 2247 

2247 

2247 
2247 
2247 
2247 

1424948
genus
111111
1644057

1424948
 755307 
species
111111

755307 
755307 
755307 
755307 
755307 
755307 

family
434343
1644056
10137121916

293431
genus
1
1


293091 
1
1
species
 293091 

113
genus
111
60846

113
111
species
 60847 

60847 

60847 

60847 

9136121516
2251
genus
333323


2252 
2252 
2252 
2252 
2252 
2252 
111111
species
 2252 
112282

 2246 
species
11111
46162

2246 
2246 
2246 
2246 

2246 


35746 
35746 
35746 
35746 
35746 
35746 
4634712
111111
species
 35746 

 756883 
species
1111
33123


756883 
756883 
756883 
756883 

575363554144
1644060
order
111112101010

575363554144
1644061
111112101010
family

1013853
111111
genus
29287


29288 
29288 
29288 
29288 
29288 
29288 
1013853
 29288 
111111
species

427565
222122
genus
121871


588898 
588898 
588898 

588898 
588898 
 588898 
species
11111
11212

 62320 
111111
species
315553

62320 
62320 
62320 
62320 
62320 
62320 

1361
1111
genus
2256


44930 
44930 

44930 
44930 
 44930 
1111
species
1361

146453
111111
species
 1902251 

1902251 
1902251 
1902251 
1902251 
1902251 
1902251 

2122
genus
88723
4197

species
1111
 406552 
3111

406552 
406552 
406552 
406552 


69525 

69525 
69525 
 69525 
species
111
186

387342
111111
genus
4385312

111111
species
 387343 
4385312

387343 
387343 
387343 
387343 
387343 
387343 

111111
genus
1269201
7121676

 1333523 
111111
species
7121676

1333523 
1333523 
1333523 
1333523 
1333523 
1333523 

91017427
203193
111111
genus

111111
species
 229731 
91017427

229731 
229731 
229731 
229731 
229731 
229731 

353799
genus
111111
14104824


353800 
353800 
353800 
353800 
353800 
353800 
 353800 
species
111111
14104824

genus
111111
63742
495853


13769 
13769 
13769 
13769 
13769 
13769 
495853
species
111111
 13769 

order
9716151613
2235
201744626862

741010108
family
1963268
10930353843

203135
1111
genus
1141

1141
species
1111
 57705 

57705 

57705 
57705 

57705 

1073987
genus
111111
147229

 1932360 
111111
species
147229

1932360 
1932360 
1932360 
1932360 
1932360 
1932360 

2810182
63743
12221
genus


416273 

416273 
416273 
416273 
416273 
263162
 416273 
species
11111


2257 
2257 
2257 
272
species
111
 2257 

236111217
genus
223343
2237

51
species
11
 2238 


2238 
2238 

species
111111
 51589 
122354

51589 
51589 
51589 
51589 
51589 
51589 


1932004 
1932004 
1932004 
1932004 
3344
 1932004 
species
1111

 1592728 
11111
species
11129

1592728 
1592728 
1592728 

1592728 
1592728 


1679096 
1679096 
1679096 
241
111
species
 1679096 

212222
genus
146825
4264514


146826 
146826 
146826 
146826 
146826 
146826 
 146826 
species
111111
122114


430914 

430914 
430914 
430914 
430914 
343410
11111
species
 430914 

10814273019
2236
236565
family

genus
223333
2239
1074201112


1407499 
1407499 
1407499 
1407499 
1407499 
111111
 1407499 
species
11111


2242 

2242 
2242 
2242 
2242 
11111
species
 2242 
11551

species
111111
 751944 
9624510

751944 
751944 
751944 
751944 
751944 
751944 

41131
1111
genus
1980514


1873524 
1873524 
1873524 
1873524 
41131
 1873524 
species
1111

1111
genus
332246
1361

species
1111
 413810 
1361


413810 
413810 
413810 
413810 

356
111
genus
1656823


1604004 

1604004 
1604004 
species
111
 1604004 
356

183980
1111
class
1121

1121
2231
1111
order

2232
1111
family
1121

2233
genus
1
1


2234 
1
 2234 
species
1

12
54260
11
genus

12
 54261 
11
species


54261 
54261 

1
genus
190818
1

1
species
 113653 
1

113653 

class
121418221719
224756
115321386416166306

3413
570264
order
1112

3413
family
1112
570265

3413
570266
genus
1112


1175445 
1175445 
1175445 
1175445 
 1175445 
1111
species
3411

1
species
 1175444 
2


1175444 

6121747806463
94695
81012131011
order

143067
family
111
111

111
genus
111
2222


2223 
species
1
 2223 
1

species
11
 301375 
11


301375 


301375 

6121746796462
2206
family
81011121010

4413
112
genus
2225

4412
 2226 
111
species


2226 


2226 
2226 


29291 
1
species
 29291 
1

12
genus
11
2220


420950 

420950 
12
11
species
 420950 

2175
genus
11
12

 2177 
species
11
12


2177 
2177 

2207
8981088
genus
6117343766159


1434103 
1434103 
1434103 
1434103 
1434103 
1434103 
225415
111111
species
 1434103 

 170861 
species
111111
268567

170861 
170861 
170861 
170861 
170861 
170861 

2111
1111
species
 2210 

2210 
2210 

2210 

2210 


2209 
2209 
2209 
2209 
2209 
2209 
91811151011
 2209 
species
111111

111
species
111
 2214 


2214 

2214 
2214 


38027 

38027 
38027 
38027 
38027 
31132
 38027 
11111
species


2215 
1
 2215 
1
species


1434100 
1434100 
1434100 
1434100 
1434100 
1434100 
144631
 1434100 
species
111111

 2208 
species
111111
212811193624

2208 
2208 
2208 
2208 
2208 
2208 


1434099 
1434099 
11
 1434099 
species
11

2111222318
 1434102 
111111
species

1434102 
1434102 
1434102 
1434102 
1434102 
1434102 

1
genus
1
101191

1
 101192 
species
1


101192 

196136
1
genus
1

species
1
 39669 
1


39669 

order
445866
2191
54104336332101240

2194
223444
family
25461411455186

genus
111111
230355
23451351314781


54120 
54120 
54120 
54120 
54120 
54120 
 54120 
species
111111
23451351314781

112333
genus
45989
2161445

 86622 
species
111
321


86622 
86622 
86622 


83986 

83986 
83986 
83986 
83986 
 83986 
11111
species
241012

12112
species
11111
 2198 


2198 
2198 
2198 
2198 
2198 

113
196137
family
111

113
genus
111
2202

 2203 
species
111
113


2203 

2203 

2203 

1141
1121
family
1198451

1121
genus
395331
1141

21
species
11
 882104 


882104 
882104 

112
species
111
 358766 

358766 

358766 
358766 

88404
family
111111
285719418249151

285719418249151
111111
genus
2192

 83984 
111111
species
285719418249151

83984 
83984 
83984 
83984 
83984 
83984 

895175541457251467
183925
class
975756

895175541457251467
2158
order
975756

975756
family
2159
895175541457251467

genus
11111
2160
31111


2162 


2162 
2162 
311
111
species
 2162 

 877455 
11
species
11


877455 


877455 

10
2316
genus
1

 2317 
1
species
10

2317 

genus
121212
145260
3977241179126231

species
11
 145263 
11


145263 

145263 

 145262 
species
1
1


145262 


145261 
145261 
145261 
145261 
145261 
145261 
3976241178126230
 145261 
111111
species

84397300277124235
2172
genus
644433


294671 
294671 
294671 
294671 
294671 
species
11111
 294671 
41123


1609968 
1
species
 1609968 
38


2173 
2173 
2173 
2173 
2173 
2173 
 2173 
species
111111
27290296273115233


230361 
230361 
230361 
49111
111
species
 230361 

352161
 83816 
species
111111

83816 
83816 
83816 
83816 
83816 
83816 


224719 


224719 

224719 
3511
 224719 
111
species

44738272022
class
624944
183968

2258
order
624944
44738272022

44738272022
624944
family
2259

40633191819
311121
genus
2260

species
111111
 2261 
5633191719

2261 
2261 
2261 
2261 
2261 
2261 

341
 1183377 
species
11

1183377 


1183377 

 53953 
species
1
1

53953 

415823
genus
313823
2263


2016361 

2016361 
12
 2016361 
species
11


246969 

246969 
 246969 
species
11
11


55802 
 55802 
species
1
1

2
 195522 
species
1

195522 


342948 
342948 
11
11
species
 342948 


2264 
1
species
 2264 
1


163003 
163003 
 163003 
11
species
11


2265 
 2265 
species
1
1

species
11
 54262 
11


54262 

54262 


1674923 
species
1
 1674923 
1


71998 
1
1
species
 71998 

 2008440 
species
11
21


2008440 
2008440 

 49899 
species
1
1


49899 

 122420 
1
species
1


122420 

24643401331
183967
class
511523

1
1
order
1235850

1577788
1
family
1

1291539
1
genus
1

 1291540 
1
species
1


1291540 

 1495144 
species
11
12


1495144 

1495144 

24643381329
order
511322
2301

4111
2111
family
90142

2111
genus
74968
4111


97393 


97393 
97393 
97393 
species
1111
 97393 
1111

3
 74969 
species
1

74969 

1
family
1747776
1

1747777
genus
1
1


1673428 
 1673428 
species
1
1

46659
211111
family
19643321228

2302
211111
genus
19643321228

1
 2303 
species
1

2303 

18643321228
 50339 
species
111111

50339 
50339 
50339 
50339 
50339 
50339 


1054217 
5
 1054217 
species
1

212160121953621081193150203725197531793434
2
264523162501263724372427
superkingdom

1042223
508458
phylum
32212

32212
class
649775
1042223

1042223
order
32212
649776

649777
32212
family
1042223

81466
genus
1
34

 81468 
1
species
34

81468 

336260
genus
11
61


336261 

336261 
61
species
11
 336261 

genus
1
49894
1

1
 97477 
1
species


97477 

12
508459
11
genus


1197717 
1197717 
12
 1197717 
11
species

81461
1111
genus
64112

 81462 
species
1111
64112

81462 

81462 
81462 

81462 

8184181
111111
phylum
200938

8184181
class
111111
118001

189769
111111
order
8184181

8184181
189770
111111
family

8184181
111111
genus
393029

species
111111
 936456 
8184181

936456 
936456 
936456 
936456 
936456 
936456 

967777
phylum
74201
37275313216597116

class
212222
203494
32321437361625

32321437361625
order
212222
48461

32321437361625
1647988
family
212222

genus
212222
239934
32321437361625

 239935 
species
111111
30931434321416

239935 
239935 
239935 
239935 
239935 
239935 


1679444 

1679444 
1679444 
1679444 
1679444 
 1679444 
11111
species
1393429

13
2
class
1955630

717963
order
2
13

13
717964
family
2

511745
2
genus
13

 511746 
1
species
12

511746 

1
 591154 
1
species

591154 

1
134549
class
1

1
1
genus
134550


1704307 
1
species
1
 1704307 

47428671197058
414999
class
434444

111111
order
415001
1288974

1288974
415002
family
111111

442430
111111
genus
1288974

1288974
species
111111
 395922 

395922 
395922 
395922 
395922 
395922 
395922 

415000
323333
order
46220591106354

46220591106354
323333
family
134623

111111
genus
178440
421918301819


107709 
107709 
107709 
107709 
107709 
107709 
111111
species
 107709 
421918301819


794903 
794903 
794903 
794903 
794903 
794903 
111111
species
 794903 
91836811

41133443724
1961799
genus
11111

species
11111
 1838286 
41133443724

1838286 

1838286 
1838286 
1838286 
1838286 

81028101133
111111
species
 1637999 

1637999 
1637999 
1637999 
1637999 
1637999 
1637999 

111
phylum
95818
611


1476577 


1476577 
species
11
 1476577 
61

 2056494 
1
species
1


2056494 

1224
phylum
136913111361140013171311
159758521556091002230133684823291181656562

126069774481291351603876842147098
28216
class
261252263268258252

 543913 
species
111
313

543913 
543913 

543913 


1904640 
1904640 
1904640 
1904640 
1904640 
1904640 
8144381065441
 1904640 
111111
species

80840
198193197197196193
order
121856723961239521539896400744721

1066513728011334
316612
111111
genus


105560 
105560 
105560 
105560 
105560 
105560 
species
111111
 105560 
1066513728011334

63391011888661
32012
111111
genus

63391011888661
species
111111
 926 

926 
926 
926 
926 
926 
926 

93681
111111
genus
13316914424915272


76731 
76731 
76731 
76731 
76731 
76731 
species
111111
 76731 
13316914424915272


1834205 

1834205 

1834205 
122
111
species
 1834205 

6120685289531753853942685
424242424242
family
80864

174951
111111
genus
233187345695208141


94132 
94132 
94132 
94132 
94132 
94132 
species
111111
 94132 
233187345695208141


1458425 
1458425 
1458425 
1458425 
1458425 
1458425 
78801021417528
 1458425 
111111
species

genus
444444
80865
6369569711545646222


742013 
742013 
742013 
742013 
742013 
742013 
14623627149416774
species
111111
 742013 

16727422137515557
species
111111
 1920191 

1920191 
1920191 
1920191 
1920191 
1920191 
1920191 


180282 
180282 
180282 
180282 
180282 
180282 
species
111111
 180282 
15532529033015462


80866 
80866 
80866 
80866 
80866 
80866 
species
111111
 80866 
16812118934617029

364316
genus
111111
93561842238959


364317 
364317 
364317 
364317 
364317 
364317 
 364317 
111111
species
93561842238959

270260357733300130
47420
genus
333333

1191491282869253
species
111111
 795665 

795665 
795665 
795665 
795665 
795665 
795665 

111111
species
 1763535 
89511041597525

1763535 
1763535 
1763535 
1763535 
1763535 
1763535 


1842537 
1842537 
1842537 
1842537 
1842537 
1842537 
 1842537 
species
111111
626012528813352

genus
444444
34072
12081362223460521242676

3523383801145273158
 2126319 
species
111111

2126319 
2126319 
2126319 
2126319 
2126319 
2126319 

111111
species
 34073 
55177911392918675350

34073 
34073 
34073 
34073 
34073 
34073 


1795631 
1795631 
1795631 
1795631 
1795631 
1795631 
1284430492912749
 1795631 
species
111111

species
111111
 436515 
1772014111060167119

436515 
436515 
436515 
436515 
436515 
436515 

23422434056920969
222222
genus
52972

1201461512339532
species
111111
 296591 

296591 
296591 
296591 
296591 
296591 
296591 


216465 
216465 
216465 
216465 
216465 
216465 
 216465 
111111
species
1147818933611437

219181
genus
222222
20012918832613953

1654515326911344
species
111111
 2109914 

2109914 
2109914 
2109914 
2109914 
2109914 
2109914 


1658672 
1658672 
1658672 
1658672 
1658672 
1658672 
35843557269
 1658672 
111111
species

352450
111111
genus
1071351131438358


2109915 
2109915 
2109915 
2109915 
2109915 
2109915 
species
111111
 2109915 
1071351131438358

283
genus
333333
510418621917392168

15017111117417549
 1082851 
111111
species

1082851 
1082851 
1082851 
1082851 
1082851 
1082851 

111111
species
 285 
32822043465520195

285 
285 
285 
285 
285 
285 


225992 
225992 
225992 
225992 
225992 
225992 
322776881624
species
111111
 225992 


1458426 
1458426 
1458426 
1458426 
1458426 
1458426 
7240581044938
111111
species
 1458426 

201096
genus
111111
248221325661209111

248221325661209111
 179636 
species
111111

179636 
179636 
179636 
179636 
179636 
179636 

genus
888888
12916
14431634199135631082536

 1858609 
111111
species
41871492136522

1858609 
1858609 
1858609 
1858609 
1858609 
1858609 


358220 
358220 
358220 
358220 
358220 
358220 
 358220 
species
111111
922591562457154

991291322349881
111111
species
 232721 

232721 
232721 
232721 
232721 
232721 
232721 


80867 
80867 
80867 
80867 
80867 
80867 
 80867 
111111
species
220372211411160100

species
111111
 721785 
92941152315917

721785 
721785 
721785 
721785 
721785 
721785 


80869 
80869 
80869 
80869 
80869 
80869 
248237509832265124
species
111111
 80869 

 1842533 
species
111111
671051692679122

1842533 
1842533 
1842533 
1842533 
1842533 
1842533 


553814 
553814 
553814 
553814 
553814 
553814 
5843515501130273116
 553814 
species
111111

444444
genus
28065
313279544829315192


1842727 
1842727 
1842727 
1842727 
1842727 
1842727 
 1842727 
111111
species
142152242522193133


1484693 
1484693 
1484693 
1484693 
1484693 
1484693 
species
111111
 1484693 
100391311614433


192843 
192843 
192843 
192843 
192843 
192843 
 192843 
species
111111
532399715814


81479 
81479 
81479 
81479 
81479 
81479 
186572752012
species
111111
 81479 

1436289
111111
genus
466253058


1436290 
1436290 
1436290 
1436290 
1436290 
1436290 
466253058
 1436290 
111111
species

238749
111111
genus
11520815124210346

 1546149 
111111
species
11520815124210346

1546149 
1546149 
1546149 
1546149 
1546149 
1546149 

665874
genus
222222
5732781168344


1678128 
1678128 
1678128 
1678128 
1678128 
1678128 
523071957140
111111
species
 1678128 

52721124
111111
species
 1678129 

1678129 
1678129 
1678129 
1678129 
1678129 
1678129 

257625326649165106
genus
222222
1649468


2116657 
2116657 
2116657 
2116657 
2116657 
2116657 
 2116657 
111111
species
1235031683317150

111111
species
 2109913 
1341221583189456

2109913 
2109913 
2109913 
2109913 
2109913 
2109913 

20527820530712789
genus
111111
65047


1658665 
1658665 
1658665 
1658665 
1658665 
1658665 
 1658665 
111111
species
20527820530712789

212743
111111
genus
614766593900389231


946333 
946333 
946333 
946333 
946333 
946333 
111111
species
 946333 
614766593900389231

233326170349155117
 413882 
111111
species

413882 
413882 
413882 
413882 
413882 
413882 

1821662393729828
genus
111111
28067


28068 
28068 
28068 
28068 
28068 
28068 
1821662393729828
111111
species
 28068 

119060
family
888789898886
335083547946969524193353230446

44013
435542
genus
20125964513

11
species
11
 576610 


576610 
576610 


556054 

556054 
556054 
556054 
1351
 556054 
species
1111

14549541
 576611 
11111
species

576611 
576611 
576611 
576611 
576611 

species
111111
 1743172 
4643211

1743172 
1743172 
1743172 
1743172 
1743172 
1743172 


1835254 
1835254 
1835254 
1835254 
1835254 
1835254 
111111
species
 1835254 
112112

188541857925234287172040422230
genus
494949494949
32008


640510 
640510 
640510 
640510 
640510 
640510 
73661101595729
 640510 
111111
species

species
111111
 337 
640653736849567362

337 
337 
337 
337 
337 
337 


1795874 
1795874 
1795874 
1795874 
1795874 
1795874 
22412318922015016386
111111
species
 1795874 


41899 
41899 
41899 
41899 
41899 
41899 
1547321423719273
111111
species
 41899 

6753100525580
111111
species
 1740162 

1740162 
1740162 
1740162 
1740162 
1740162 
1740162 

111111
species
 758793 
11390175146120151

758793 
758793 
758793 
758793 
758793 
758793 


1468409 
1468409 
1468409 
1468409 
1468409 
1468409 
species
111111
 1468409 
351653724714

72166571101761164569384325
species group
999999
111527

813484857531
 1385592 
species
111111

1385592 
1385592 
1385592 
1385592 
1385592 
1385592 

 342113 
species
111111
286318350456302212

342113 
342113 
342113 
342113 
342113 
342113 

10821317160820051115583
111111
species
 57975 

57975 
57975 
57975 
57975 
57975 
57975 


1385591 
1385591 
1385591 
1385591 
1385591 
1385591 
 1385591 
species
111111
311676745522

6215461844026
 1637841 
species
111111

1637841 
1637841 
1637841 
1637841 
1637841 
1637841 


1637831 
1637831 
1637831 
1637831 
1637831 
1637831 
394445654934
 1637831 
species
111111

 13373 
111111
species
74653810251194583347

13373 
13373 
13373 
13373 
13373 
13373 


1637837 
1637837 
1637837 
1637837 
1637837 
1637837 
species
111111
 1637837 
393177712515


28450 
28450 
28450 
28450 
28450 
28450 
 28450 
species
111111
485041196850761146943055

species group
222222222222
87882
79968679106461214777944159

79164911291268715571
 87883 
111111
species

87883 
87883 
87883 
87883 
87883 
87883 

1056015412810196
111111
species
 60550 

60550 
60550 
60550 
60550 
60550 
60550 

55142991327732
111111
species
 488729 

488729 
488729 
488729 
488729 
488729 
488729 


1637862 
1637862 
1637862 
1637862 
1637862 
1637862 
species
111111
 1637862 
73711661449747

species
111111
 1207504 
95115154140145113

1207504 
1207504 
1207504 
1207504 
1207504 
1207504 

 482957 
111111
species
315414381367212155

482957 
482957 
482957 
482957 
482957 
482957 

 152500 
species
111111
102381131828736

152500 
152500 
152500 
152500 
152500 
152500 


95485 
95485 
95485 
95485 
95485 
95485 
198119269267197118
 95485 
111111
species


1503054 
1503054 
1503054 
1503054 
1503054 
1503054 
111111
species
 1503054 
1962541982207542

10571371147917611334614
species
111111
 292 

292 
292 
292 
292 
292 
292 

 488446 
species
111111
100110891035351

488446 
488446 
488446 
488446 
488446 
488446 


1637869 
1637869 
1637869 
1637869 
1637869 
1637869 
11311113713312939
species
111111
 1637869 

102681241638952
 265293 
species
111111

265293 
265293 
265293 
265293 
265293 
265293 


95486 
95486 
95486 
95486 
95486 
95486 
228821933048361619981094
111111
species
 95486 


488732 
488732 
488732 
488732 
488732 
488732 
 488732 
111111
species
916615913710955

111111
species
 101571 
7238689671081840329

101571 
101571 
101571 
101571 
101571 
101571 

11722931393840
species
111111
 1503055 

1503055 
1503055 
1503055 
1503055 
1503055 
1503055 

 1637853 
species
111111
1309613014814342

1637853 
1637853 
1637853 
1637853 
1637853 
1637853 


488731 
488731 
488731 
488731 
488731 
488731 
 488731 
111111
species
79861561719575

7967809641219773342
 60552 
species
111111

60552 
60552 
60552 
60552 
60552 
60552 


152480 
152480 
152480 
152480 
152480 
152480 
369946489472386151
species
111111
 152480 


488447 
488447 
488447 
488447 
488447 
488447 
10110014815610165
 488447 
111111
species


1795043 
1795043 
1795043 
1795043 
1795043 
1795043 
177991802899845207
111111
species
 1795043 


1804984 
1804984 
1804984 
1804984 
1804984 
1804984 
14011816013878130
species
111111
 1804984 


2217913 
2217913 
2217913 
2217913 
2217913 
2217913 
17313519315512961
 2217913 
species
111111


1678678 
1678678 
1678678 
1678678 
1678678 
1678678 
 1678678 
species
111111
14514821623513291

species
111111
 1740163 
4532841423529

1740163 
1740163 
1740163 
1740163 
1740163 
1740163 


28095 
28095 
28095 
28095 
28095 
28095 
10431104127913331314675
species
111111
 28095 

species
111111
 758796 
1248416320683146

758796 
758796 
758796 
758796 
758796 
758796 


1097668 
1097668 
1097668 
1097668 
1097668 
1097668 
1011598816613247
 1097668 
species
111111

 640512 
111111
species
871791401226681

640512 
640512 
640512 
640512 
640512 
640512 


416344 
416344 
416344 
416344 
416344 
416344 
126531441685582
 416344 
species
111111

175144188236125102
species
111111
 640511 

640511 
640511 
640511 
640511 
640511 
640511 

157932
genus
111111
295656505620421837802932


29443 
29443 
29443 
29443 
29443 
29443 
111111
species
 29443 
295656505620421837802932

48736
444444
genus
554253927417948942381905


305 
305 
305 
305 
305 
305 
457046735963773133321505
 305 
species
111111

 329 
species
111111
416234647792315140

329 
329 
329 
329 
329 
329 


105219 
105219 
105219 
105219 
105219 
105219 
111111
species
 105219 
334255452490313100

species
111111
 190721 
222230355476278160

190721 
190721 
190721 
190721 
190721 
190721 

93217
genus
999999
18641465288532241416937


573737 
573737 
573737 
573737 
573737 
573737 
 573737 
111111
species
957216519410956

species
111111
 656178 
88671661797737

656178 
656178 
656178 
656178 
656178 
656178 


93219 
93219 
93219 
93219 
93219 
93219 
111111
species
 93219 
7479207175115109

111111
species
 93221 
1411321891658379

93221 
93221 
93221 
93221 
93221 
93221 


93222 
93222 
93222 
93222 
93222 
93222 
 93222 
111111
species
1837829834414159


93218 
93218 
93218 
93218 
93218 
93218 
479328484613288194
 93218 
111111
species


445709 
445709 
445709 
445709 
445709 
445709 
13213328027212263
 445709 
111111
species

species
111111
 656179 
70581331816037

656179 
656179 
656179 
656179 
656179 
656179 


93220 
93220 
93220 
93220 
93220 
93220 
species
111111
 93220 
6025189631101421303

genus
999999
106589
237824062997375119321281


876364 
876364 
876364 
876364 
876364 
876364 
 876364 
species
111111
296318309374177145

192268275394243149
species
111111
 1389192 

1389192 
1389192 
1389192 
1389192 
1389192 
1389192 


367825 
367825 
367825 
367825 
367825 
367825 
 367825 
species
111111
234331323332253161


119219 
119219 
119219 
119219 
119219 
119219 
322201448484231168
111111
species
 119219 

species
111111
 82541 
1496915024410453

82541 
82541 
82541 
82541 
82541 
82541 


106590 
106590 
106590 
106590 
106590 
106590 
 106590 
111111
species
521498622884383288

 2036817 
111111
species
18226227934321692

2036817 
2036817 
2036817 
2036817 
2036817 
2036817 


68895 
68895 
68895 
68895 
68895 
68895 
289365289325168162
 68895 
111111
species


248026 
248026 
248026 
248026 
248026 
248026 
1939430237115763
 248026 
111111
species

189419752757295617571148
genus
121212121212
1822464


412963 
412963 
412963 
412963 
412963 
412963 
552185652659
species
111111
 412963 


261302 
261302 
261302 
261302 
261302 
261302 
 261302 
species
111111
112541381657344

111111
species
 75105 
551619825837512322

75105 
75105 
75105 
75105 
75105 
75105 

831671871847854
species
111111
 948107 

948107 
948107 
948107 
948107 
948107 
948107 


2026199 
2026199 
2026199 
2026199 
2026199 
2026199 
12413117120715868
 2026199 
species
111111


134537 
134537 
134537 
134537 
134537 
134537 
111111
species
 134537 
704313814597112

species
111111
 311230 
14414118319813876

311230 
311230 
311230 
311230 
311230 
311230 


36873 
36873 
36873 
36873 
36873 
36873 
309449377421283193
 36873 
111111
species

111111
species
 1926494 
84321811458638

1926494 
1926494 
1926494 
1926494 
1926494 
1926494 

13512418218410352
111111
species
 148447 

148447 
148447 
148447 
148447 
148447 
148447 

10511015517210886
 169430 
111111
species

169430 
169430 
169430 
169430 
169430 
169430 

122841352339544
species
111111
 252970 

252970 
252970 
252970 
252970 
252970 
252970 

genus
111111
318147
8884821423424


1768242 
1768242 
1768242 
1768242 
1768242 
1768242 
 1768242 
species
111111
8884821423424


1469502 
1469502 
1469502 
1469502 
1469502 
1469502 
334122384516
111111
species
 1469502 

868519332713234
111111
genus
92793

868519332713234
species
111111
 1296669 

1296669 
1296669 
1296669 
1296669 
1296669 
1296669 

156145214316144100
genus
111111
88


34029 
34029 
34029 
34029 
34029 
34029 
111111
species
 34029 
156145214316144100

506
family
322930313030
52915215591920723988187078494

111111
genus
1100891
1611166626


643674 
643674 
643674 
643674 
643674 
643674 
1611166626
111111
species
 643674 

1
genus
1
1472344

1
 1472345 
species
1

1472345 

517
171617171717
genus
48882172211501118928152176655


1416803 
1416803 
1416803 
1416803 
1416803 
1416803 
 1416803 
111111
species
184215273281152210

 463014 
species
111111
66571151304167

463014 
463014 
463014 
463014 
463014 
463014 

species
111111
 1697043 
6378102817158

1697043 
1697043 
1697043 
1697043 
1697043 
1697043 

 519 
species
111111
240275311354431135

519 
519 
519 
519 
519 
519 


35814 
35814 
35814 
35814 
35814 
35814 
7061232734919666360
111111
species
 35814 

 1416806 
111111
species
1139514717512681

1416806 
1416806 
1416806 
1416806 
1416806 
1416806 

288260372337303174
species
111111
 103855 

103855 
103855 
103855 
103855 
103855 
103855 


521 
521 
521 
521 
521 
521 
species
111111
 521 
10124368243

 463024 
species
111111
4934771274916

463024 
463024 
463024 
463024 
463024 
463024 


94624 
94624 
94624 
94624 
94624 
94624 
 94624 
111111
species
981101372215017


123899 
123899 
123899 
123899 
123899 
123899 
229263172201150106
 123899 
species
111111


463025 
463025 
463025 
463025 
463025 
463025 
248331350375278162
 463025 
species
111111

 520 
111111
species
45869135491140614671122384939

520 
520 
520 
520 
520 
520 


518 
518 
518 
518 
518 
518 
432434427604390209
 518 
species
111111


2163011 

2163011 
2163011 
2163011 
2163011 
 2163011 
species
11111
3107114

16618418424114087
111111
species
 463040 

463040 
463040 
463040 
463040 
463040 
463040 


1331258 
1331258 
1331258 
1331258 
1331258 
1331258 
 1331258 
species
111111
118921511369727

1921582
genus
111111
8716519420919583


1851544 
1851544 
1851544 
1851544 
1851544 
1851544 
111111
species
 1851544 
8716519420919583

genus
111111
305976
282943382921


1007105 
1007105 
1007105 
1007105 
1007105 
1007105 
 1007105 
species
111111
282943382921

10012513114511374
507
genus
111111

 511 
species
111111
10012513114511374

511 
511 
511 
511 
511 
511 

290425
222222
genus
272557522830

species
111111
 310575 
16102925246

310575 
310575 
310575 
310575 
310575 
310575 


302406 
302406 
302406 
302406 
302406 
302406 
 302406 
species
111111
11152827424

352239183629445529701527
genus
555555
222


217204 
217204 
217204 
217204 
217204 
217204 
111111
species
 217204 
252209282365254117


85698 
85698 
85698 
85698 
85698 
85698 
species
111111
 85698 
23462605226929581833976


32002 
32002 
32002 
32002 
32002 
32002 
345398390482410152
 32002 
species
111111

 217203 
species
111111
379480483421258180

217203 
217203 
217203 
217203 
217203 
217203 

species
111111
 1758194 
200226205229215102

1758194 
1758194 
1758194 
1758194 
1758194 
1758194 

90243
genus
11
12

 90245 
species
11
12

90245 


90245 

29574
genus
111111
13822721

111111
species
 29575 
13822721

29575 
29575 
29575 
29575 
29575 
29575 

1136312414614777
359336
111111
genus

species
111111
 75697 
1136312414614777

75697 
75697 
75697 
75697 
75697 
75697 

274136342467215657648972290
75682
242424242424
family

149698
777777
genus
19174227432504362231637748

species
111111
 2072590 
13383272378331924394

2072590 
2072590 
2072590 
2072590 
2072590 
2072590 

21322713778438623981
111111
species
 1707785 

1707785 
1707785 
1707785 
1707785 
1707785 
1707785 


47229 
47229 
47229 
47229 
47229 
47229 
97443791554114356343137
111111
species
 47229 


1678028 
1678028 
1678028 
1678028 
1678028 
1678028 
111745822033176177103
 1678028 
species
111111


2045208 
2045208 
2045208 
2045208 
2045208 
2045208 
 2045208 
species
111111
16672973002384614890

111111
species
 1141883 
205822736494088186168

1141883 
1141883 
1141883 
1141883 
1141883 
1141883 


1593482 
1593482 
1593482 
1593482 
1593482 
1593482 
11183151953305230175
 1593482 
111111
species

963
555555
genus
27091514433255011583793


92645 
92645 
92645 
92645 
92645 
92645 
 92645 
111111
species
5233987821040377150

1685929043114949
 2025949 
111111
species

2025949 
2025949 
2025949 
2025949 
2025949 
2025949 


964 
964 
964 
964 
964 
964 
 964 
111111
species
137375122582791796341

 80842 
species
111111
264165506536172183

80842 
80842 
80842 
80842 
80842 
80842 


341045 
341045 
341045 
341045 
341045 
341045 
species
111111
 341045 
3811414967038970

303379
222222
genus
1621223103783127


1809410 
1809410 
1809410 
1809410 
1809410 
1809410 
111111
species
 1809410 
61949715888

101282132202319
 204773 
species
111111

204773 
204773 
204773 
204773 
204773 
204773 

169389726223808854354
genus
333333
202907

5332738781337352133
species
111111
 158899 

158899 
158899 
158899 
158899 
158899 
158899 

6083098811209290113
111111
species
 279058 

279058 
279058 
279058 
279058 
279058 
279058 


279113 
279113 
279113 
279113 
279113 
279113 
5523158631262212108
 279113 
111111
species

666666
genus
29580
36371531693610626759367

7063681404213415666
 55508 
species
111111

55508 
55508 
55508 
55508 
55508 
55508 


1236179 
1236179 
1236179 
1236179 
1236179 
1236179 
 1236179 
species
111111
2654391724

94543417482796195153
111111
species
 1644131 

1644131 
1644131 
1644131 
1644131 
1644131 
1644131 


1938606 
1938606 
1938606 
1938606 
1938606 
1938606 
9662341718273515058
111111
species
 1938606 

111111
species
 375286 
54361232183013

375286 
375286 
375286 
375286 
375286 
375286 

9404051904272622673
species
111111
 368607 

368607 
368607 
368607 
368607 
368607 
368607 

3841740331
genus
111111
846

111111
species
 847 
3841740331

847 
847 
847 
847 
847 
847 

1211
1111
genus
1301080


1160784 
1160784 


1160784 
1160784 
1211
 1160784 
1111
species

2
1381133
1
genus

species
1
 669502 
2


669502 

111111
genus
327159
389643695111


327160 
327160 
327160 
327160 
327160 
327160 
species
111111
 327160 
389643695111

order
191820232016
32003
476457636701476218

32011
family
566854
861001091475856

606965512425
genus
111111
404


405 
405 
405 
405 
405 
405 
111111
species
 405 
606965512425

2151410
11121
genus
359407


1055487 
1055487 

1055487 
212
111
species
 1055487 

51210
 359408 
species
111


359408 
359408 
359408 

1679002
genus
1121
2163

 1581557 
species
11
11


1581557 
1581557 

253
 1581680 
111
species


1581680 

1581680 

1581680 

202731581628
222222
genus
81682


266009 
266009 
266009 
266009 
266009 
266009 
 266009 
111111
species
7191431321

1381727137
species
111111
 887061 

887061 
887061 
887061 
887061 
887061 
887061 

417188
genus
11111
16

417188
11111
species
 1662285 

1662285 
1662285 
1662285 
1662285 
1662285 

family
444444
90627
12012014517415825

935200
111111
genus
13113443345


649841 
649841 
649841 
649841 
649841 
649841 
 649841 
111111
species
13113443345

44532739116
genus
111111
96


370405 
370405 
370405 
370405 
370405 
370405 
44532739116
 370405 
species
111111

genus
111111
314343
48483966919


63745 
63745 
63745 
63745 
63745 
63745 
48483966919
 63745 
111111
species

1584526225
111111
genus
1778653


1985873 
1985873 
1985873 
1985873 
1985873 
1985873 
1584526225
 1985873 
species
111111

206379
family
757885
393173545725

424552
genus
914
1992526318

1119
 44574 
1111
species

44574 

44574 
44574 
44574 


916 

916 
916 
916 
4368
species
1111
 916 


153948 
153948 
153948 
153948 
153948 
 153948 
species
11111
318410

species
111111
 915 
118131337

915 
915 
915 
915 
915 
915 


44577 
44577 
 44577 
11
species
11

 261292 
1
species
2


261292 

333333
genus
35798
202248282617

111111
species
 35799 
912201265

35799 
35799 
35799 
35799 
35799 
35799 

111111
species
 1288494 
44713146

1288494 
1288494 
1288494 
1288494 
1288494 
1288494 


1231 
1231 
1231 
1231 
1231 
1231 
111111
species
 1231 
7621366

111111
family
2008790
585660663114

111111
genus
919
585660663114

 36861 
species
111111
585660663114

36861 
36861 
36861 
36861 
36861 
36861 

17315024926017298
family
222222
2008793

genus
111111
1054211
108721141347832

111111
species
 748811 
108721141347832

748811 
748811 
748811 
748811 
748811 
748811 

378210
genus
111111
65781351269466


1842540 
1842540 
1842540 
1842540 
1842540 
1842540 
65781351269466
 1842540 
111111
species

4151113
33055
22313
genus


233181 

233181 
233181 
3148
111
species
 233181 

1
species
1
 994692 


994692 


994695 
1
 994695 
species
1


994696 


994696 
11
species
11
 994696 

12
 33056 
species
11

33056 


33056 


1576550 
1576550 
11
 1576550 
11
species

15912253203024461448941
161616161616
order
206389

13391911170619761174825
family
121212121212
2008794

12960
genus
666666
74110739341051682465

111111
species
 62928 
14530214517512460

62928 
62928 
62928 
62928 
62928 
62928 

176262248284149121
 41977 
111111
species

41977 
41977 
41977 
41977 
41977 
41977 

10017213518513957
species
111111
 418699 

418699 
418699 
418699 
418699 
418699 
418699 

17020610817210342
111111
species
 2067960 

2067960 
2067960 
2067960 
2067960 
2067960 
2067960 

11710021910975149
 198107 
111111
species

198107 
198107 
198107 
198107 
198107 
198107 

 748247 
species
111111
3331791269236

748247 
748247 
748247 
748247 
748247 
748247 


2080469 
2080469 
2080469 
2080469 
2080469 
2080469 
489345656133
species
111111
 2080469 

genus
555555
33057
550745727860431327


1134435 
1134435 
1134435 
1134435 
1134435 
1134435 
species
111111
 1134435 
691981271729651


85643 
85643 
85643 
85643 
85643 
85643 
111111
species
 85643 
1632151281923730

931082181818996
111111
species
 96773 

96773 
96773 
96773 
96773 
96773 
96773 


59405 
59405 
59405 
59405 
59405 
59405 
1131128317710897
 59405 
species
111111


2005884 
2005884 
2005884 
2005884 
2005884 
2005884 
species
111111
 2005884 
11211217113810153

75787
333333
family
203210285381222109

10512311515710647
146937
111111
genus


146939 
146939 
146939 
146939 
146939 
146939 
10512311515710647
 146939 
111111
species

83481141794449
551759
genus
111111

111111
species
 551760 
83481141794449

551760 
551760 
551760 
551760 
551760 
551760 


1898103 
1898103 
1898103 
1898103 
1898103 
1898103 
species
111111
 1898103 
153956457213

491323989527
2008795
family
111111

491323989527
111111
genus
73029

species
111111
 259537 
491323989527

259537 
259537 
259537 
259537 
259537 
259537 

14
11
genus
189384


189385 

189385 
14
11
species
 189385 

201821992417306023831162
order
212125252221
206351

141418181514
family
481
9651143113915321165591

genus
122211
59
5310430766710

5310316666710
species
111111
 63 

63 
63 
63 
63 
63 
63 

11410
 96942 
species
111


96942 
96942 
96942 


2052837 

2052837 
2052837 
522
 2052837 
111
species

111012121111
genus
482
8641000104314021083576

116752
 326523 
species
111111

326523 
326523 
326523 
326523 
326523 
326523 

131061
 490 
11111
species

490 

490 
490 
490 
490 

6928307151034820478
species
111111
 487 

487 
487 
487 
487 
487 
487 


1853278 
1853278 
1853278 
1853278 
1853278 
1853278 
111111
species
 1853278 
15193024925

 485 
species
111111
864219121513028

485 
485 
485 
485 
485 
485 


486 

486 
486 
486 
486 
351565
11111
species
 486 

111111
species
 495 
332232333617

495 
495 
495 
495 
495 
495 


488 
488 
488 
488 
488 
488 
species
111111
 488 
521414142

9801427343
species
111111
 655307 

655307 
655307 
655307 
655307 
655307 
655307 


28091 
28091 
28091 
28091 
28091 
28091 
111111
species
 28091 
12258105

 1853276 
species
1111
71149

1853276 
1853276 
1853276 
1853276 


483 
483 
483 
483 
483 
11461310
11111
species
 483 

71
genus
111
541

541
111
species
 72 


72 
72 
72 

32257
genus
111
232

111
species
 504 
232

504 

504 
504 

genus
111111
538
46135344123

111111
species
 539 
46135344123

539 
539 
539 
539 
539 
539 

264
11
genus
1193515


1196083 

1196083 
species
11
 1196083 
264

10531056127815281218571
1499392
family
777777

624712694912735335
333333
genus
535

species
111111
 536 
226277310416226108

536 
536 
536 
536 
536 
536 


1108595 
1108595 
1108595 
1108595 
1108595 
1108595 
111111
species
 1108595 
223183244346351169


2059672 
2059672 
2059672 
2059672 
2059672 
2059672 
17525214015015858
species
111111
 2059672 

187
111111
genus
9493113989853

9493113989853
species
111111
 1938604 

1938604 
1938604 
1938604 
1938604 
1938604 
1938604 

718515918010462
genus
111111
568394

718515918010462
 748280 
species
111111

748280 
748280 
748280 
748280 
748280 
748280 

628912812311353
genus
111111
885864


1906741 
1906741 
1906741 
1906741 
1906741 
1906741 
628912812311353
 1906741 
111111
species

2027718421516868
111111
genus
168470


168471 
168471 
168471 
168471 
168471 
168471 
 168471 
111111
species
2027718421516868

class
632613632646597596
1236
14367402056633837717112712522029401581344

116131
12121
order
1240482

family
12121
1240483
116131

1335631
genus
111
161


1267021 
1267021 


1267021 
 1267021 
species
111
161

10121
1193503
1111
genus

10121
species
1111
 1196095 


1196095 
1196095 

1196095 
1196095 

order
777777
135618
175272218286214181

403
family
777777
175272218286214181

3465856
111111
genus
762296

111111
species
 1704499 
3465856

1704499 
1704499 
1704499 
1704499 
1704499 
1704499 

413
111111
genus
344540403038

344540403038
 414 
111111
species

414 
414 
414 
414 
414 
414 

39773
genus
111111
5348127


271065 
271065 
271065 
271065 
271065 
271065 
5348127
 271065 
species
111111

133220168180167130
416
genus
444444


1727196 
1727196 
1727196 
1727196 
1727196 
1727196 
species
111111
 1727196 
424770545569


421 
421 
421 
421 
421 
421 
species
111111
 421 
12171661817

6611553808138
111111
species
 702114 

702114 
702114 
702114 
702114 
702114 
702114 

111111
species
 1538553 
13412940136

1538553 
1538553 
1538553 
1538553 
1538553 
1538553 

135614
order
414141414141
431378917972155622924324587349878

1965322099313622033454
888888
family
1775411

95282543212
111111
genus
2233801


2021234 
2021234 
2021234 
2021234 
2021234 
2021234 
111111
species
 2021234 
95282543212

6261253415624889173
genus
333333
231454


1379159 
1379159 
1379159 
1379159 
1379159 
1379159 
111111
species
 1379159 
17620311918125023


231455 
231455 
231455 
231455 
231455 
231455 
19554510715836340
species
111111
 231455 


445710 
445710 
445710 
445710 
445710 
445710 
255505189285276110
111111
species
 445710 

4107489919031845
75309
genus
111111

4107489919031845
 666685 
111111
species

666685 
666685 
666685 
666685 
666685 
666685 

37746113620025195
genus
111111
323413


323415 
323415 
323415 
323415 
323415 
323415 
37746113620025195
species
111111
 323415 

242605
genus
111111
25937226917236291


242606 
242606 
242606 
242606 
242606 
242606 
111111
species
 242606 
25937226917236291

1983584913319248
70411
genus
111111

111111
species
 81475 
1983584913319248

81475 
81475 
81475 
81475 
81475 
81475 

429413914752145692910704567019424
32033
family
333333333333

83618
genus
222222
4295938628622923415218

 314722 
111111
species
3079684921718802567153

314722 
314722 
314722 
314722 
314722 
314722 


415229 
415229 
415229 
415229 
415229 
415229 
 415229 
111111
species
121625376941284865

1542643235529842855941264625989
338
genus
151515151515

 56454 
111111
species
975235546523114724

56454 
56454 
56454 
56454 
56454 
56454 


48664 
48664 
48664 
48664 
48664 
48664 
 48664 
111111
species
2865559725617202059142


343 
343 
343 
343 
343 
343 
 343 
111111
species
79561684537841075561237


90270 
90270 
90270 
90270 
90270 
90270 
 90270 
111111
species
2068538118812561846111

species
111111
 56460 
222740071491127173356

56460 
56460 
56460 
56460 
56460 
56460 

species
111111
 53413 
204627161311876

53413 
53413 
53413 
53413 
53413 
53413 


29447 
29447 
29447 
29447 
29447 
29447 
63410822530153217
111111
species
 29447 

2728510128817071956169
 456327 
species
111111

456327 
456327 
456327 
456327 
456327 
456327 


56458 
56458 
56458 
56458 
56458 
56458 
 56458 
species
111111
299162331191449220480

 339 
species
111111
17213357561010917613375596

339 
339 
339 
339 
339 
339 


1985254 
1985254 
1985254 
1985254 
1985254 
1985254 
 1985254 
111111
species
178935022129611433152


347 
347 
347 
347 
347 
347 
3360969683222319998284181412
species
111111
 347 

52111015421048739
species
111111
 56459 

56459 
56459 
56459 
56459 
56459 
56459 

643453
species group
111111
77465163819469942204645802857


346 
346 
346 
346 
346 
346 
77465163819469942204645802857
111111
species
 346 

 442694 
111111
species
1019246317972494491

442694 
442694 
442694 
442694 
442694 
442694 

1203236914963293682
222222
genus
83614

61212528132141238
 2006110 
111111
species

2006110 
2006110 
2006110 
2006110 
2006110 
2006110 

59111176831152444
 2172536 
111111
species

2172536 
2172536 
2172536 
2172536 
2172536 
2172536 

77391472968538935103471
555555
genus
68


1605891 
1605891 
1605891 
1605891 
1605891 
1605891 
92619489242351279
 1605891 
111111
species

species
111111
 435897 
2437467615213951519105

435897 
435897 
435897 
435897 
435897 
435897 


69 
69 
69 
69 
69 
69 
111111
species
 69 
12182293107554102150

 262324 
species
111111
960179412137990079

262324 
262324 
262324 
262324 
262324 
262324 


84531 
84531 
84531 
84531 
84531 
84531 
 84531 
111111
species
2198401821311421151158

2370
genus
111111
6401128120401435154


2371 
2371 
2371 
2371 
2371 
2371 
species
111111
 2371 
6401128120401435154

26127256358834871982583203502510
40323
888888
genus


83617 
83617 
83617 
83617 
83617 
83617 
species
111111
 83617 
28486321921522177956


2005046 
2005046 
2005046 
2005046 
2005046 
2005046 
 2005046 
species
111111
5520111781642788330463


1904944 
1904944 
1904944 
1904944 
1904944 
1904944 
 1904944 
111111
species
31578667841887519711962127


128780 
128780 
128780 
128780 
128780 
128780 
 128780 
111111
species
7359153421513487511897

 1827305 
species
111111
6478143491073278434790

1827305 
1827305 
1827305 
1827305 
1827305 
1827305 

28476259441330181516
 1793721 
111111
species

1793721 
1793721 
1793721 
1793721 
1793721 
1793721 

995085
111111
species group
18487539986925481021741561581899


40324 
40324 
40324 
40324 
40324 
40324 
18487539986925481021741561581899
 40324 
species
111111


216778 
216778 
216778 
216778 
216778 
216778 
 216778 
species
111111
19767434861938482135867162

135622
726871796056
order
2489131297012201037751

111111
family
267892
6415522242828

44011
111111
genus
6415522242828

 44012 
111111
species
6415522242828

44012 
44012 
44012 
44012 
44012 
44012 

267889
425533
family
61102928713

61102728713
28228
424533
genus

species
11111
 28229 
12312

28229 

28229 
28229 
28229 
28229 

species
11111
 58049 
4616357

58049 

58049 
58049 
58049 
58049 


1816218 
1816218 
1816218 
1816218 
1816218 
1816218 
8231014
 1816218 
111111
species

1
 2161872 
1
species


2161872 

68611
species
1111
 1816219 

1816219 
1816219 
1816219 
1816219 

genus
1
1518149
2

2
1
species
 1763536 


1763536 

family
21222
267891
7923195

7923195
genus
21222
58050


80854 
80854 
80854 
80854 
80854 
 80854 
11111
species
112133


69539 

69539 
69539 
69539 
species
1111
 69539 
682162

427627543477649486
family
192021222019
72275

1111
genus
1172191
5811


2172099 
2172099 


2172099 
2172099 
5811
species
1111
 2172099 

111111
genus
1751872
1514914182

1514914182
 1526571 
111111
species

1526571 
1526571 
1526571 
1526571 
1526571 
1526571 

112221
genus
89404
5153314


300231 
300231 
300231 
300231 
300231 
300231 
5142114
111111
species
 300231 


983545 
983545 
983545 
112
111
species
 983545 

288793
genus
11111
1823102111

1823102111
 2183582 
species
11111

2183582 
2183582 
2183582 
2183582 
2183582 

genus
577856
226
49461091615826

16
 1777491 
11
species


1777491 

1777491 


28108 
28108 
28108 
28108 
28108 
28108 
1671941244
 28108 
species
111111


589873 
589873 
589873 
589873 
1111
species
 589873 
14441


2058133 
2058133 
2058133 
2058133 
2058133 
124211
species
11111
 2058133 

 287094 
1111
species
2237

287094 
287094 
287094 
287094 

 314275 
111111
species
25285049197

314275 
314275 
314275 
314275 
314275 
314275 

 1917158 
11111
species
11162

1917158 
1917158 
1917158 
1917158 

1917158 

species
111111
 233316 
561161011

233316 
233316 
233316 
233316 
233316 
233316 

261825
11111
genus
12214

 680279 
species
11111
12214

680279 

680279 
680279 
680279 
680279 

334535408276557439
2742
999999
genus

252241354539
111111
species
 1033846 

1033846 
1033846 
1033846 
1033846 
1033846 
1033846 

3570865212359
 1420917 
species
111111

1420917 
1420917 
1420917 
1420917 
1420917 
1420917 

species
111111
 1874317 
111029304014

1874317 
1874317 
1874317 
1874317 
1874317 
1874317 

305148241237
111111
species
 490759 

490759 
490759 
490759 
490759 
490759 
490759 

 1671721 
species
111111
276345284385

1671721 
1671721 
1671721 
1671721 
1671721 
1671721 


2743 
2743 
2743 
2743 
2743 
2743 
 2743 
111111
species
432937438138


1420916 
1420916 
1420916 
1420916 
1420916 
1420916 
68109491710454
species
111111
 1420916 

503427163447
111111
species
 330734 

330734 
330734 
330734 
330734 
330734 
330734 


1749259 
1749259 
1749259 
1749259 
1749259 
1749259 
4514746317566
 1749259 
111111
species

383395231503222132
267890
232421242018
family

383395231503222132
22
genus
232421242018

960836322
111111
species
 60478 

60478 
60478 
60478 
60478 
60478 
60478 

 351745 
species
1111
1311381

351745 
351745 

351745 
351745 


62322 
62322 
62322 
62322 
62322 
62322 
111111
species
 62322 
185104791039048

 260364 
species
11111
91682

260364 
260364 
260364 
260364 
260364 

111111
species
 24 
885863

24 
24 
24 
24 
24 
24 


60480 
60480 
60480 
60480 
60480 
60480 
 60480 
111111
species
11411731


2029986 
2029986 
2029986 
2029986 
species
1111
 2029986 
2521

895123
 70863 
111111
species

70863 
70863 
70863 
70863 
70863 
70863 

 60961 
species
111111
2221315

60961 
60961 
60961 
60961 
60961 
60961 


60481 
60481 
60481 
60481 
60481 
60481 
331951537
 60481 
species
111111

34710212
 60217 
species
111111

60217 
60217 
60217 
60217 
60217 
60217 

species
111
 70864 
225

70864 
70864 

70864 


93973 
93973 
93973 
93973 
93973 
93973 
species
111111
 93973 
771232

31132
 56812 
species
11111

56812 
56812 
56812 
56812 
56812 


2059264 
2059264 
2059264 
2059264 
2059264 
2059264 
423242
 2059264 
111111
species


192073 
192073 
192073 
192073 
192073 
192073 
2431562
 192073 
111111
species

756976
111111
species
 94122 

94122 
94122 
94122 
94122 
94122 
94122 

species
111111
 2018305 
237314

2018305 
2018305 
2018305 
2018305 
2018305 
2018305 


271097 
271097 
271097 
271097 
271097 
271097 
111111
species
 271097 
2734262745


225848 
225848 
225848 
225848 
225848 
225848 
15192019310
 225848 
species
111111

85544
species
11111
 271098 

271098 
271098 

271098 
271098 
271098 


359303 
359303 
359303 
359303 
359303 
359303 
 359303 
species
111111
326238532723


43661 
43661 
43661 
43661 

43661 
33483
 43661 
species
11111

species
111
 404011 
122


404011 
404011 
404011 

267893
444444
family
7962740301712

7962740301712
135575
genus
444444

3713565
species
111111
 2100422 

2100422 
2100422 
2100422 
2100422 
2100422 
2100422 


1096243 
1096243 
1096243 
1096243 
1096243 
1096243 
121717425
111111
species
 1096243 

species
111111
 2055892 
77621471

2055892 
2055892 
2055892 
2055892 
2055892 
2055892 

5191721
species
111111
 135577 

135577 
135577 
135577 
135577 
135577 
135577 

family
112222
267894
22356115

22356115
genus
112222
67572


314282 
314282 
314282 
314282 
species
1111
 314282 
2113

 357794 
111111
species
22335102

357794 
357794 
357794 
357794 
357794 
357794 

267888
1815151989
family
65793971339875

genus
1815151989
53246
65793971339875

species
111111
 298657 
5794044193830

298657 
298657 
298657 
298657 
298657 
298657 


1348114 
1348114 
1348114 
1348114 

1348114 
 1348114 
species
11111
441122

42137131
 152297 
111111
species

152297 
152297 
152297 
152297 
152297 
152297 

11
species
 394751 
115

394751 


394751 


247523 
247523 
247523 
247523 
1111
species
 247523 
11417


43657 
43657 
43657 
43657 
43657 
43657 
8161166
 43657 
111111
species

11
 283699 
11
species


283699 
283699 

 28109 
species
1
1


28109 


288 
288 
288 
288 
 288 
species
1111
1453


43658 


43658 

43658 
 43658 
species
111
116


1761891 
1761891 
1761891 
 1761891 
species
111
114

1
species
 43659 
1


43659 


1720343 
1720343 
1720343 
1720343 
1115
 1720343 
1111
species


314281 
314281 
314281 
314281 
314281 
57212
 314281 
11111
species

1
species
 234831 
1

234831 

31
 28107 
11
species


28107 

28107 

species
111111
 161398 
310132231

161398 
161398 
161398 
161398 
161398 
161398 


267375 


267375 

267375 
1111
 267375 
111
species

 228 
111111
species
3791522

228 
228 
228 
228 
228 
228 

species
11
 166935 
166

166935 


166935 


176102 
176102 
176102 
176102 
176102 
176102 
 176102 
species
111111
4545106


1514074 
1514074 
1514074 
1514074 
1514074 
33441
 1514074 
species
11111

1
species
1
 43662 


43662 


1248727 
1248727 
1248727 
1248727 
1248727 
1248727 
264017234638
 1248727 
species
111111

495637183316
genus
111111
349742

 1543721 
111111
species
495637183316

1543721 
1543721 
1543721 
1543721 
1543721 
1543721 

 2070539 
1111
species
1121


2070539 
2070539 
2070539 
2070539 

655184
genus
111
215

 1427364 
111
species
215


1427364 


1427364 
1427364 

92911491348381
333333
order
1692040

333333
family
1692041
92911491348381

genus
111111
986106
13626271114

111111
species
 1281578 
13626271114

1281578 
1281578 
1281578 
1281578 
1281578 
1281578 

345759583739
1692042
genus
111111

 1675686 
111111
species
345759583739

1675686 
1675686 
1675686 
1675686 
1675686 
1675686 

1744881
genus
111111
452864493528


1620215 
1620215 
1620215 
1620215 
1620215 
1620215 
452864493528
 1620215 
species
111111

3817852214531186293503573218322709
198194195197194191
order
91347

543
959596969694
family
638364536732095657522040611820

111
genus
1048757
112

111
species
 1048758 
112


1048758 
1048758 
1048758 


693444 
693444 
693444 
693444 
693444 
693444 
704437913910
species
111111
 693444 

1906660
genus
11111
131310191

 1778264 
11111
species
131310191

1778264 
1778264 
1778264 
1778264 

1778264 

 891974 
111111
species
2412251422615541

891974 
891974 
891974 
891974 
891974 
891974 


1920109 
1920109 
1920109 
1920109 
1920109 
1920109 
1024642953611
 1920109 
111111
species


1835721 
 1835721 
species
1
1

311117
1682492
11111
genus

11111
species
 1410383 
311117

1410383 
1410383 
1410383 

1410383 
1410383 

83654
333333
genus
33721517536517039


1920114 
1920114 
1920114 
1920114 
1920114 
1920114 
 1920114 
species
111111
13490561209510

 1920116 
species
111111
11070761334417

1920116 
1920116 
1920116 
1920116 
1920116 
1920116 

species
111111
 83655 
9355431123112

83655 
83655 
83655 
83655 
83655 
83655 

4926419575
1335483
genus
111111


563 
563 
563 
563 
563 
563 
species
111111
 563 
4926419575

18087951495035
929812
111111
genus

111111
species
 929813 
18087951495035

929813 
929813 
929813 
929813 
929813 
929813 

2635176213803446780391
544
genus
171717171716

151910117332129541181
888887
species group
1344959


2066049 
2066049 
2066049 
2066049 
2066049 
2066049 
72374187163
species
111111
 2066049 


2077147 
2077147 
2077147 
2077147 
2077147 
2077147 
493725514010
 2077147 
species
111111


133448 
133448 
133448 
133448 
133448 
133448 
111111
species
 133448 
6734329052


57706 
57706 
57706 
57706 
57706 
57706 
111111
species
 57706 
10786421272611

402524604
 2077148 
11111
species

2077148 
2077148 
2077148 
2077148 
2077148 

13583581866211
 67827 
111111
species

67827 
67827 
67827 
67827 
67827 
67827 


2077149 
2077149 
2077149 
2077149 
2077149 
2077149 
111111
species
 2077149 
4128329261

111111
species
 546 
10086814791436382143

546 
546 
546 
546 
546 
546 

73634672298
111111
species
 1703250 

1703250 
1703250 
1703250 
1703250 
1703250 
1703250 

6641296823
 1702170 
111111
species

1702170 
1702170 
1702170 
1702170 
1702170 
1702170 

44330324947373107
species
111111
 545 

545 
545 
545 
545 
545 
545 


67825 
67825 
67825 
67825 
67825 
67825 
10773691212723
 67825 
111111
species

8261571121314
111111
species
 2013114 

2013114 
2013114 
2013114 
2013114 
2013114 
2013114 


67824 
67824 
67824 
67824 
67824 
67824 
35331766235
 67824 
species
111111

73355089183
111111
species
 1920110 

1920110 
1920110 
1920110 
1920110 
1920110 
1920110 


35703 
35703 
35703 
35703 
35703 
35703 
 35703 
species
111111
194113882533318

432942632129
species
111111
 2019568 

2019568 
2019568 
2019568 
2019568 
2019568 
2019568 

111
203804
genus
111

111
species
111
 1505597 

1505597 
1505597 


1505597 

57439130660413777
1330545
genus
444444

species
111111
 1907578 
10766351153512

1907578 
1907578 
1907578 
1907578 
1907578 
1907578 


2153385 
2153385 
2153385 
2153385 
2153385 
2153385 
 2153385 
111111
species
10885701292614

814461902613
species
111111
 69220 

69220 
69220 
69220 
69220 
69220 
69220 


61646 
61646 
61646 
61646 
61646 
61646 
111111
species
 61646 
2781961402705038

89514411258
111111
species
 1920128 

1920128 
1920128 
1920128 
1920128 
1920128 
1920128 

29292734317
111111
species
 2052938 

2052938 
2052938 
2052938 
2052938 
2052938 
2052938 

3121
1906659
genus
1111


1778263 

1778263 
1778263 
1778263 
3121
species
1111
 1778263 

568987
genus
111111
88109149

88109149
species
111111
 138072 

138072 
138072 
138072 
138072 
138072 
138072 

111
111
genus
409304

111
 168169 
111
species


168169 
168169 


168169 

2018171448
620
genus
222221

2132
 623 
1111
species

623 
623 
623 

623 


1813821 
 1813821 
1
species
6

181714828
111111
species
 622 

622 
622 
622 
622 
622 
622 


2066051 
2066051 
2066051 
2066051 
2066051 
2066051 
1127051108145
111111
species
 2066051 

1615101294
2172100
genus
111111


2172103 
2172103 
2172103 
2172103 
2172103 
2172103 
1615101294
 2172103 
111111
species

777777
genus
413496
153510057941621356221


413502 
413502 
413502 
413502 
413502 
413502 
 413502 
species
111111
10786631143125


1163710 
1163710 
1163710 
1163710 
1163710 
1163710 
 1163710 
111111
species
78543190374

2721711002164924
 413503 
111111
species

413503 
413503 
413503 
413503 
413503 
413503 


413497 
413497 
413497 
413497 
413497 
413497 
species
111111
 413497 
16487991941522

64141132972115878
 28141 
species
111111

28141 
28141 
28141 
28141 
28141 
28141 

111111
species
 535744 
129107981583428

535744 
535744 
535744 
535744 
535744 
535744 

 413501 
species
111111
14489741283240

413501 
413501 
413501 
413501 
413501 
413501 

547
genus
242424242424
958166984618986822561487

845658434066872419641319
141414141414
species group
354276

12339375891119303233
 158836 
111111
species

158836 
158836 
158836 
158836 
158836 
158836 


2027919 
2027919 
2027919 
2027919 
2027919 
2027919 
146108931897442
111111
species
 2027919 

species
111111
 1915310 
12111862894121

1915310 
1915310 
1915310 
1915310 
1915310 
1915310 


61645 
61645 
61645 
61645 
61645 
61645 
111111
species
 61645 
381229232448180107


1296536 
1296536 
1296536 
1296536 
1296536 
1296536 
1206841124159
 1296536 
111111
species

1024966952925
 208224 
111111
species

208224 
208224 
208224 
208224 
208224 
208224 

85755689913
species
111111
 1812935 

1812935 
1812935 
1812935 
1812935 
1812935 
1812935 


2077136 
2077136 
2077136 
2077136 
2077136 
2077136 
855138993612
 2077136 
species
111111


69218 
69218 
69218 
69218 
69218 
69218 
 69218 
species
111111
10461501243541


550 
550 
550 
550 
550 
550 
 550 
species
111111
54943759251756561123705


2077137 
2077137 
2077137 
2077137 
2077137 
2077137 
111111
species
 2077137 
805828951324

714250801821
111111
species
 1870930 

1870930 
1870930 
1870930 
1870930 
1870930 
1870930 

4452621691712
species
111111
 299767 

299767 
299767 
299767 
299767 
299767 
299767 


1812934 
1812934 
1812934 
1812934 
1812934 
1812934 
species
111111
 1812934 
3902361823487154

 1914861 
species
111111
11185901471818

1914861 
1914861 
1914861 
1914861 
1914861 
1914861 

12012141902817
 1868135 
species
111111

1868135 
1868135 
1868135 
1868135 
1868135 
1868135 

species
111111
 881260 
188102791382424

881260 
881260 
881260 
881260 
881260 
881260 

657441961713
 1827481 
species
111111

1827481 
1827481 
1827481 
1827481 
1827481 
1827481 

884739633022
species
111111
 1977566 

1977566 
1977566 
1977566 
1977566 
1977566 
1977566 

 399742 
species
111111
12187742102811

399742 
399742 
399742 
399742 
399742 
399742 


2051905 
2051905 
2051905 
2051905 
2051905 
2051905 
658517614011
species
111111
 2051905 


1560339 
1560339 
1560339 
1560339 
1560339 
1560339 
168130781184031
111111
species
 1560339 


1692238 
1692238 
1692238 
1692238 
1692238 
1692238 
species
111111
 1692238 
97563995137

10268541265414
 1166130 
111111
species

1166130 
1166130 
1166130 
1166130 
1166130 
1166130 

genus
222222
590
11406750249171043228941760


28901 
28901 
28901 
28901 
28901 
28901 
11272741848391027428771731
111111
species
 28901 


54736 
54736 
54736 
54736 
54736 
54736 
111111
species
 54736 
13484781581729

genus
111111
158483
518405586720100149


158822 
158822 
158822 
158822 
158822 
158822 
518405586720100149
 158822 
111111
species

1330546
genus
222222
629532364603330218

 61647 
111111
species
387353244353231126

61647 
61647 
61647 
61647 
61647 
61647 


1334193 
1334193 
1334193 
1334193 
1334193 
1334193 
111111
species
 1334193 
2421791202509992


134287 
1
species
 134287 
1

999999
genus
570
2105215912115042167592594898


548 
548 
548 
548 
548 
548 
 548 
111111
species
13619847291476324167

111111
species
 1905288 
107513086238

1905288 
1905288 
1905288 
1905288 
1905288 
1905288 

 244366 
species
111111
725509363777318177

244366 
244366 
244366 
244366 
244366 
244366 

154961173384631576172593897
 573 
species
111111

573 
573 
573 
573 
573 
573 


1463165 
1463165 
1463165 
1463165 
1463165 
1463165 
 1463165 
species
111111
650453386731280164

855361912244
 1934254 
species
111111

1934254 
1934254 
1934254 
1934254 
1934254 
1934254 

species
111111
 2026240 
41502755711

2026240 
2026240 
2026240 
2026240 
2026240 
2026240 

937683602970433123
 1134687 
species
111111

1134687 
1134687 
1134687 
1134687 
1134687 
1134687 

 571 
species
111111
165013968431728593307

571 
571 
571 
571 
571 
571 

743504388902154118
1330547
genus
444444

 1158459 
species
111111
224168752952324

1158459 
1158459 
1158459 
1158459 
1158459 
1158459 

 283686 
111111
species
2441711403614845

283686 
283686 
283686 
283686 
283686 
283686 


208223 
208223 
208223 
208223 
208223 
208223 
111111
species
 208223 
1196755116305

species
111111
 497725 
156981181305344

497725 
497725 
497725 
497725 
497725 
497725 


1199245 
1199245 
species
11
 1199245 
11

11
genus
11
1906657


1778262 
1778262 
11
 1778262 
species
11

12852907159731342833502084
444443
genus
561

20172331
species
11111
 1499973 

1499973 
1499973 
1499973 
1499973 
1499973 


564 
564 
564 
564 
564 
564 
25653141
 564 
111111
species

12616889658401310632842056
species
111111
 562 

562 
562 
562 
562 
562 
562 


208962 
208962 
208962 
208962 
208962 
208962 
1911521262586127
 208962 
species
111111

10107355601084351222
160674
222222
genus


575 
575 
575 
575 
575 
575 
species
111111
 575 
172131661331853

 54291 
species
111111
838604494951333169

54291 
54291 
54291 
54291 
54291 
54291 

1903409
family
232121222122
2918471572757269825108423594912

genus
222222
82986
22212077213397


53336 
53336 
53336 
53336 
53336 
53336 
 53336 
111111
species
18110053173255

41202440142
111111
species
 82987 

82987 
82987 
82987 
82987 
82987 
82987 

53335
genus
121111121212
959976053636861512801203


1235990 


1235990 
1235990 
1235990 
2111
species
1111
 1235990 

species
111111
 1891675 
2231371132712918

1891675 
1891675 
1891675 
1891675 
1891675 
1891675 

4592901624855022
 1076550 
species
111111

1076550 
1076550 
1076550 
1076550 
1076550 
1076550 

 592316 
111111
species
3742431663534470

592316 
592316 
592316 
592316 
592316 
592316 


1484157 
1484157 
1484157 
1484157 
1484157 
1484157 
 1484157 
species
111111
2732061313554823


470934 
470934 
470934 
470934 
470934 
470934 
species
111111
 470934 
4273299213413470374484

196412326721763178145
111111
species
 553 

553 
553 
553 
553 
553 
553 


66269 
66269 
66269 
66269 
66269 
66269 
 66269 
111111
species
186104128193271

1941661121864864
 1484158 
111111
species

1484158 
1484158 
1484158 
1484158 
1484158 
1484158 

species group
111111
1654067
9121684427868371243

 549 
species
111111
9121684427868371243

549 
549 
549 
549 
549 
549 


665913 
665913 
665913 
665913 
665913 
665913 
 665913 
111111
species
3222322253166070

4173191593545062
species
111111
 665914 

665914 
665914 
665914 
665914 
665914 
665914 

genus
776766
551
2820031495406897424224010233689


552 
552 
552 
552 
552 
552 
128576851213567564
 552 
species
111111

113961342812044333
 79967 
111111
species

79967 
79967 
79967 
79967 
79967 
79967 


1619313 
1619313 
1619313 
1619313 
1619313 
1619313 
 1619313 
111111
species
4531981903614421

112
species
111
 1922217 

1922217 
1922217 

1922217 


338565 
338565 
338565 
338565 
338565 
338565 
species
111111
 338565 
6092921706011950

 182337 
111111
species
278186147479675282383308293500

182337 
182337 
182337 
182337 
182337 
182337 


215689 
215689 
215689 
215689 
215689 
215689 
 215689 
species
111111
3301891463861321

712
111
genus
51228

712
species
111
 51229 

51229 

51229 


51229 

32199
111111
genus
161010161711


9 
9 
9 
9 
9 
9 
161010161711
species
111111
 9 

702
111111
genus
48383657849

48383657849
species
111111
 703 

703 
703 
703 
703 
703 
703 

8805614381053285123
1903414
161414151514
family

434436725319
545555
genus
626

 628 
11111
species
751021

628 

628 
628 
628 
628 

 351671 
111111
species
43511141

351671 
351671 
351671 
351671 
351671 
351671 


351679 
351679 
351679 
351679 
351679 
351679 
 351679 
species
111111
4471213


40576 
40576 
40576 
40576 
40576 
40576 
172915252813
 40576 
species
111111

 40577 
species
111111
11841481

40577 
40577 
40577 
40577 
40577 
40577 

111
637
111
genus


634113 


634113 
634113 
111
 634113 
111
species

50833524259414850
581
genus
111111

 582 
111111
species
50833524259414850

582 
582 
582 
582 
582 
582 

53282165176
genus
333323
29487

species
111111
 230089 
167719132

230089 
230089 
230089 
230089 
230089 
230089 

11111
species
 291112 
135162

291112 
291112 
291112 
291112 

291112 


2218628 
2218628 
2218628 
2218628 
2218628 
2218628 
361893042
 2218628 
species
111111

10767631491137
586
444443
genus


587 
587 
587 
587 
587 
587 
15921722
species
111111
 587 


588 
588 
588 
588 
588 
588 
 588 
111111
species
784248100320

11111
species
 126385 
44284

126385 
126385 
126385 
126385 
126385 

10121124215
111111
species
 333962 

333962 
333962 
333962 
333962 
333962 
333962 

16887761735510
221221
genus
583

16386761695410
species
111111
 584 

584 
584 
584 
584 
584 
584 

species
1111
 585 
5141

585 
585 

585 
585 

3067229817603343987727
151515151515
family
1903410

8257308997
71655
genus
111111

8257308997
111111
species
 1109412 

1109412 
1109412 
1109412 
1109412 
1109412 
1109412 

147981141605837
84565
333333
genus

111111
species
 63612 
361318331111

63612 
63612 
63612 
63612 
63612 
63612 


1239307 
1239307 
1239307 
1239307 
1239307 
1239307 
species
111111
 1239307 
62484176174

111111
species
 1486991 
493755513022

1486991 
1486991 
1486991 
1486991 
1486991 
1486991 

555555
genus
122277
152910627341662277266

3024144564
 55208 
111111
species

55208 
55208 
55208 
55208 
55208 
55208 

111111
species
 1905730 
234185853182755

1905730 
1905730 
1905730 
1905730 
1905730 
1905730 


554 
554 
554 
554 
554 
554 
 554 
species
111111
62542226372312345

 2042057 
111111
species
235156941812660

2042057 
2042057 
2042057 
2042057 
2042057 
2042057 


29471 
29471 
29471 
29471 
29471 
29471 
40527527839595102
species
111111
 29471 

666666
genus
204037
130910818821432643417


204042 
204042 
204042 
204042 
204042 
204042 
111111
species
 204042 
2181421412718844

963759913337
 2037915 
111111
species

2037915 
2037915 
2037915 
2037915 
2037915 
2037915 


204038 
204038 
204038 
204038 
204038 
204038 
111111
species
 204038 
183138144221153113


1089444 
1089444 
1089444 
1089444 
1089444 
1089444 
616534385599264153
111111
species
 1089444 


204039 
204039 
204039 
204039 
204039 
204039 
species
111111
 204039 
1021641161307850


1778540 
1778540 
1778540 
1778540 
1778540 
1778540 
9466371202720
species
111111
 1778540 

9767416481236453330
1903412
111111111111
family

74413111311
genus
111111
82982

 82983 
species
111111
74413111311

82983 
82983 
82983 
82983 
82983 
82983 

677551515780416302
635
777777
genus


1821960 
1821960 
1821960 
1821960 
1821960 
1821960 
 1821960 
111111
species
733039781811


636 
636 
636 
636 
636 
636 
 636 
species
111111
17012312617410659


1650654 
1650654 
1650654 
1650654 
1650654 
1650654 
 1650654 
species
111111
694975714151


93378 
93378 
93378 
93378 
93378 
93378 
183772485
111111
species
 93378 

 1263550 
111111
species
121841211816690

1263550 
1263550 
1263550 
1263550 
1263550 
1263550 

111111
species
 1578828 
4955561086958

1578828 
1578828 
1578828 
1578828 
1578828 
1578828 


67780 
67780 
67780 
67780 
67780 
67780 
1771739114410828
111111
species
 67780 

568
genus
333333
2251491023433627


1848580 
1848580 
1848580 
1848580 
1848580 
1848580 
6631158522
 1848580 
species
111111

 546367 
species
111111
2712154633

546367 
546367 
546367 
546367 
546367 
546367 


569 
569 
569 
569 
569 
569 
111111
species
 569 
132106722123122

1903416
222211
family
57263476137

82984
genus
1111
248736

1111
species
 82985 
248736

82985 
82985 
82985 
82985 

33182740137
82980
111111
genus


158841 
158841 
158841 
158841 
158841 
158841 
111111
species
 158841 
33182740137

447792
222222
genus
191109942185714

80484594164
 1756993 
111111
species

1756993 
1756993 
1756993 
1756993 
1756993 
1756993 


1972431 
1972431 
1972431 
1972431 
1972431 
1972431 
11161491244110
111111
species
 1972431 

2088315038108262753876154727
family
333333333231
1903411

8673122136109157116
222222
genus
34037


34038 
34038 
34038 
34038 
34038 
34038 
111111
species
 34038 
31914310231227351

111111
species
 741091 
54816911129878465

741091 
741091 
741091 
741091 
741091 
741091 

1565532
111111
genus
55419117516015936

55419117516015936
 1805933 
species
111111

1805933 
1805933 
1805933 
1805933 
1805933 
1805933 

111111
genus
1745211
1115939862015


1639108 
1639108 
1639108 
1639108 
1639108 
1639108 
111111
species
 1639108 
1115939862015

629
131313131211
genus
4427252017304692497366

111111
species
 29484 
171825620936

29484 
29484 
29484 
29484 
29484 
29484 


1839800 
1839800 
1839800 
1839800 
1839800 
331412371
species
11111
 1839800 


28152 
28152 
28152 
28152 
28152 
28152 
5232135125
species
111111
 28152 


29485 
29485 
29485 
29485 
 29485 
species
1111
2020757

1720186076
species
111111
 29483 

29483 
29483 
29483 
29483 
29483 
29483 

14066464131324112104
species
111111
 630 

630 
630 
630 
630 
630 
630 


29486 
29486 
29486 
29486 
29486 
29486 
 29486 
111111
species
233200802835715


419257 
419257 
419257 
419257 
419257 
419257 
57985278517
species
111111
 419257 

1649845
species group
333333
230913319822418262191


633 
633 
633 
633 
633 
633 
 633 
species
111111
3582722623774493


367190 
367190 
367190 
367190 
367190 
367190 
2936133222
 367190 
species
111111

 632 
111111
species
19221023707200921696

632 
632 
632 
632 
632 
632 


263819 
263819 
263819 
263819 
263819 
263819 
 263819 
111111
species
665749672016

111111
species
 631 
632048108286

631 
631 
631 
631 
631 
631 

613
161616161616
genus
149241195686691505068824194


82996 
82996 
82996 
82996 
82996 
82996 
 82996 
111111
species
165512007871724560271

2321141322505242
 2033438 
111111
species

2033438 
2033438 
2033438 
2033438 
2033438 
2033438 

 671990 
111111
species
1959413927510247

671990 
671990 
671990 
671990 
671990 
671990 


47917 
47917 
47917 
47917 
47917 
47917 
111111
species
 47917 
43927921157312131


768493 
768493 
768493 
768493 
768493 
768493 
species
111111
 768493 
11160461295023

10892891489447
111111
species
 1759437 

1759437 
1759437 
1759437 
1759437 
1759437 
1759437 

2371621162213060
111111
species
 28151 

28151 
28151 
28151 
28151 
28151 
28151 


615 
615 
615 
615 
615 
615 
species
111111
 615 
1001085605912985750883044


61651 
61651 
61651 
61651 
61651 
61651 
 61651 
species
111111
26520515623915158

111111
species
 61652 
420367304457179125

61652 
61652 
61652 
61652 
61652 
61652 

17687921535339
 768490 
species
111111

768490 
768490 
768490 
768490 
768490 
768490 

1301431401127554
 1758196 
111111
species

1758196 
1758196 
1758196 
1758196 
1758196 
1758196 


614 
614 
614 
614 
614 
614 
 614 
species
111111
587316286543138132


488142 
488142 
488142 
488142 
488142 
488142 
14315415114398151
 488142 
111111
species


104623 
104623 
104623 
104623 
104623 
104623 
species
111111
 104623 
733535722012


1327989 
1327989 
1327989 
1327989 
1327989 
1327989 
 1327989 
111111
species
14388731547158

287835212770266733921997
order
222222222122
135624

family
222222222122
84642
287835212770266733921997

5363332610520
genus
111111
129577

5363332610520
 511062 
111111
species

511062 
511062 
511062 
511062 
511062 
511062 

272433002628249331861888
642
171717171717
genus


1636608 
1636608 
1636608 
1636608 
1636608 
1636608 
species
111111
 1636608 
492466604414

222250233257291240
 654 
species
111111

654 
654 
654 
654 
654 
654 

146161171171132119
 2033032 
species
111111

2033032 
2033032 
2033032 
2033032 
2033032 
2033032 


1636606 
1636606 
1636606 
1636606 
1636606 
1636606 
 1636606 
111111
species
343832404053

255547319513
species
111111
 652 

652 
652 
652 
652 
652 
652 


1636607 
1636607 
1636607 
1636607 
1636607 
1636607 
646928413741
 1636607 
species
111111

539842605945
 1636609 
species
111111

1636609 
1636609 
1636609 
1636609 
1636609 
1636609 

111111
species
 644 
12811683117610171486862

644 
644 
644 
644 
644 
644 

362409405366375268
species
111111
 645 

645 
645 
645 
645 
645 
645 


558964 
558964 
558964 
558964 
558964 
558964 
6582596010530
 558964 
species
111111


948519 
948519 
948519 
948519 
948519 
948519 
111111
species
 948519 
479541566743


1758179 
1758179 
1758179 
1758179 
1758179 
1758179 
222842324914
111111
species
 1758179 


1920107 
1920107 
1920107 
1920107 
1920107 
1920107 
484029395736
111111
species
 1920107 


196024 
196024 
196024 
196024 
196024 
196024 
483470558835
 196024 
111111
species


2033033 
2033033 
2033033 
2033033 
2033033 
2033033 
 2033033 
species
111111
412235375913


651 
651 
651 
651 
651 
651 
12311142364014
 651 
species
111111

111111
species
 648 
9410111013516248

648 
648 
648 
648 
648 
648 

2318123514
225143
222212
genus

1384173
11111
species
 1416627 

1416627 
1416627 
1416627 
1416627 

1416627 

101081811
species
111111
 1903694 

1903694 
1903694 
1903694 
1903694 
1903694 
1903694 

genus
111111
347533
5911063797954


347534 
347534 
347534 
347534 
347534 
347534 
5911063797954
111111
species
 347534 

193034342131
43947
genus
111111


43948 
43948 
43948 
43948 
43948 
43948 
 43948 
species
111111
193034342131

genus
111111
1273155
63159374212620

111111
species
 585455 
63159374212620

585455 
585455 
585455 
585455 
585455 
585455 

6266821
111111
species
 2169539 

2169539 
2169539 
2169539 
2169539 
2169539 
2169539 

species
111
 186490 
212

186490 

186490 
186490 

111111
genus
1608298
853283

 1076588 
111111
species
853283

1076588 
1076588 
1076588 
1076588 
1076588 
1076588 

71210016622026792
1210101499
order
72273

135617
121
family
223

11
genus
40751
13


40754 
40754 
13
species
11
 40754 

21
1021
11
genus


288004 
288004 
 288004 
species
11
21

4257511616518739
135616
family
754654

281688713417433
111111
genus
1237

281688713417433
111111
species
 1238 

1238 
1238 
1238 
1238 
1238 
1238 

genus
11
28884
31


39765 
39765 
 39765 
species
11
31

12218
34067
211
genus


728003 


728003 
11
species
 728003 
58

117
1
species
 385025 

385025 


1329899 
 1329899 
1
species
1

416843
933
genus
112221

411513
 92245 
species
111111

92245 
92245 
92245 
92245 
92245 
92245 


147268 
147268 
147268 
533
111
species
 147268 

155232213
genus
221212
40222


754477 
754477 

754477 

754477 
 754477 
species
1111
3281

 754476 
species
111111
123231412

754476 
754476 
754476 
754476 
754476 
754476 

family
544745
34064
2872348528053

2872348508053
262
544645
genus

species
1111
 28110 
2176112

28110 


28110 
28110 
28110 


263 
263 
263 
263 
263 
263 
 263 
species
111111
521641276431


1542390 
1
species
1
 1542390 


549298 
549298 
549298 
549298 
549298 
11111
species
 549298 
32142


573570 
573570 
573570 

573570 
1111
species
 573570 
2141

species
111111
 657445 
14358138

657445 
657445 
657445 
657445 
657445 
657445 

1
species
 622488 
1


622488 


1547445 
 1547445 
1
species
1

2
1869285
genus
1

2
 594679 
species
1


594679 

8761042807862922466
order
262627232526
135613

72276
121212121212
family
446554371446447228

133193
111111
genus
8912519483822


351052 
351052 
351052 
351052 
351052 
351052 
8912519483822
species
111111
 351052 

152855453823
genus
111111
1051

 1442136 
species
111111
152855453823

1442136 
1442136 
1442136 
1442136 
1442136 
1442136 

genus
222222
85108
543745274635

 1053 
111111
species
403242224130

1053 
1053 
1053 
1053 
1053 
1053 

1453555
111111
species
 1052 

1052 
1052 
1052 
1052 
1052 
1052 

222222
genus
1335745
951926127

3491645
111111
species
 1335757 

1335757 
1335757 
1335757 
1335757 
1335757 
1335757 

61101082
 1335746 
species
111111

1335746 
1335746 
1335746 
1335746 
1335746 
1335746 

14620231915
genus
111111
1765964


160660 
160660 
160660 
160660 
160660 
160660 
14620231915
species
111111
 160660 

106633
555555
genus
265353213277294126


108010 
108010 
108010 
108010 
108010 
108010 
 108010 
species
111111
656055705035

79169525613716
111111
species
 1033854 

1033854 
1033854 
1033854 
1033854 
1033854 
1033854 


396595 
396595 
396595 
396595 
396595 
396595 
311839364044
species
111111
 396595 


186931 
186931 
186931 
186931 
186931 
186931 
 186931 
species
111111
426135453315


106634 
106634 
106634 
106634 
106634 
106634 
 106634 
111111
species
484532703416

2113122024
449719
111111
family

437504
111111
genus
2113122024


437505 
437505 
437505 
437505 
437505 
437505 
111111
species
 437505 
2113122024

51534
1738654
family
11111

51534
11111
genus
1738655


1548547 
1548547 
1548547 
1548547 
1548547 
51534
species
11111
 1548547 

family
222222
255526
8611838487120

8611838487120
genus
222222
109262


927 
927 
927 
927 
927 
927 
111111
species
 927 
56612023318

305718254012
 1860122 
111111
species

1860122 
1860122 
1860122 
1860122 
1860122 
1860122 

1676141
family
111111
284872445014

284872445014
1676142
111111
genus


1579979 
1579979 
1579979 
1579979 
1579979 
1579979 
284872445014
 1579979 
111111
species

family
99106810
1046
290308309301348200

7107637
genus
33323
1227

173323
 133539 
11111
species

133539 
133539 
133539 

133539 
133539 

1111
species
 1229 
4121

1229 
1229 
1229 


1229 

 473531 
species
11111
222313

473531 
473531 
473531 

473531 
473531 

111111
genus
85072
475165599447


1049 
1049 
1049 
1049 
1049 
1049 
475165599447
species
111111
 1049 


1978339 
1978339 
1978339 
1978339 
1978339 
1978339 
497164503935
111111
species
 1978339 

263629376014
111111
genus
13724

263629376014
 73141 
species
111111

73141 
73141 
73141 
73141 
73141 
73141 

311648481814
111111
genus
156885


80679 
80679 
80679 
80679 
80679 
80679 
311648481814
species
111111
 80679 

13
1980513
11
genus

species
11
 1630141 
13


1630141 


1630141 

752041313814
53392
111111
genus

752041313814
111111
species
 1166950 

1166950 
1166950 
1166950 
1166950 
1166950 
1166950 

5510454769336
85076
111111
genus


37487 
37487 
37487 
37487 
37487 
37487 
5510454769336
 37487 
species
111111

7047464300436327236
order
221818221618
135625

7047464300436327236
712
221818221618
family

1116194028397
75984
genus
333323


1432056 
1432056 
1432056 
1432056 

1432056 
 1432056 
species
11111
12421


75985 
75985 
75985 
75985 
75985 
75985 
1109123516364
 75985 
111111
species


85404 
85404 
85404 
85404 
85404 
85404 
6511032
 85404 
111111
species

5449301022713
genus
121111
745


754 
1
 754 
1
species

 747 
111111
species
5448301022713

747 
747 
747 
747 
747 
747 

724
644654
genus
153299114157212153


727 
727 
727 
727 
727 
727 
125265929411476
 727 
111111
species

 729 
111111
species
32192314

729 
729 
729 
729 
729 
729 

172816356858
species
111111
 197575 

197575 
197575 
197575 
197575 
197575 
197575 


730 
730 
730 
730 
730 
730 
5451565
species
111111
 730 

211
 249188 
species
111

249188 


249188 
249188 

13
species
11
 712310 

712310 


712310 

1515722
214906
genus
111111


731 
731 
731 
731 
731 
731 
 731 
species
111111
1515722

432523
genus
713
2619224658

2321
 67854 
1111
species

67854 
67854 

67854 

67854 

 715 
111111
species
51551746

715 
715 
715 
715 
715 
715 


718 


718 
11
species
11
 718 


716 
716 
716 
716 
716 
18117231
 716 
11111
species

31
 189834 
species
11


189834 

189834 

genus
333332
416916
41193352392723

species
11111
 739 
31252

739 
739 
739 
739 
739 


732 
732 
732 
732 
732 
732 
1161012134
 732 
species
111111

41052640221219
 714 
species
111111

714 
714 
714 
714 
714 
714 

2094023
genus
11111
182314144


738 
738 
738 
738 

738 
species
11111
 738 
182314144

23403
476528
1111
genus

23403
 47735 
1111
species

47735 

47735 
47735 

47735 

genus
1111
697331
1533221


157673 

157673 

157673 
157673 
 157673 
1111
species
1533221

155493
111111
genus
11211831322


750 
750 
750 
750 
750 
750 
11211831322
 750 
species
111111

227923952064138625672126
order
393939403737
135619

720174425
191033
111111
family

720174425
genus
111111
141450

111111
species
 141451 
720174425

141451 
141451 
141451 
141451 
141451 
141451 

666666
family
224372
216296262148300241

59753
genus
555555
214293259144294239


59754 
59754 
59754 
59754 
59754 
59754 
668728
species
111111
 59754 


1094342 
1094342 
1094342 
1094342 
1094342 
1094342 
639484498990
 1094342 
111111
species

species
111111
 1306787 
106113544513446

1306787 
1306787 
1306787 
1306787 
1306787 
1306787 

 285091 
111111
species
175066323026

285091 
285091 
285091 
285091 
285091 
285091 

 1113728 
species
111111
223047113969

1113728 
1113728 
1113728 
1113728 
1113728 
1113728 

233462
genus
111111
2025617

233462
species
111111
 1917421 

1917421 
1917421 
1917421 
1917421 
1917421 
1917421 

667757
family
135620
101157167143217201

191418151919
28253
333323
genus

6511011
 400668 
species
111111

400668 
400668 
400668 
400668 
400668 
400668 


936476 
936476 
936476 
936476 
936476 
936476 
1181441814
species
111111
 936476 

11111
species
 119864 
21314

119864 
119864 
119864 
119864 

119864 

1537406
111111
genus
51510152812


1249553 
1249553 
1249553 
1249553 
1249553 
1249553 
51510152812
 1249553 
species
111111

111111
genus
48075
629211496151143


1821621 
1821621 
1821621 
1821621 
1821621 
1821621 
 1821621 
111111
species
629211496151143

187492
genus
111111
153624161925

 187493 
species
111111
153624161925

187493 
187493 
187493 
187493 
187493 
187493 

112
111
genus
188907

species
111
 188908 
112


188908 
188908 

188908 

255527
222222
family
5992638183614

518238134
111111
genus
1445504

518238134
 1445505 
111111
species

1445505 
1445505 
1445505 
1445505 
1445505 
1445505 

594815102310
genus
111111
230494


1336806 
1336806 
1336806 
1336806 
1336806 
1336806 
594815102310
111111
species
 1336806 

224379
111111
family
278540241035

111111
genus
158481
278540241035


158327 
158327 
158327 
158327 
158327 
158327 
278540241035
111111
species
 158327 

4324141
1920240
221221
family

4324141
221221
genus
261963

211
 261964 
11
species


261964 


261964 


1561924 
1561924 

1561924 
1561924 
1561924 
 1561924 
species
11111
31331


1144748 

1144748 
1144748 
121
species
111
 1144748 

132518081538104519861609
28256
family
212121212019

204286
genus
111111
909813771228154

909813771228154
species
111111
 28258 

28258 
28258 
28258 
28258 
28258 
28258 

42054
genus
111111
10114360737232

species
111111
 158080 
10114360737232

158080 
158080 
158080 
158080 
158080 
158080 

genus
111111
235572
182221355838

182221355838
species
111111
 91844 

91844 
91844 
91844 
91844 
91844 
91844 

404432
111111
genus
1271881127922693

111111
species
 1771309 
1271881127922693

1771309 
1771309 
1771309 
1771309 
1771309 
1771309 

1097412069160182
genus
222222
504090

 157779 
species
111111
87507231123137

157779 
157779 
157779 
157779 
157779 
157779 

111111
species
 698828 
222448383745

698828 
698828 
698828 
698828 
698828 
698828 

790118696862010511025
131414131312
genus
2745

 1971364 
11111
species
141261

1971364 
1971364 
1971364 
1971364 
1971364 

species
111111
 475662 
9515014769129211

475662 
475662 
475662 
475662 
475662 
475662 


1610576 
1610576 
1610576 
1610576 
1610576 
1610576 
species
111111
 1610576 
375029233410

 2746 
111111
species
931551166213394

2746 
2746 
2746 
2746 
2746 
2746 

941415912
species
111111
 213554 

213554 
213554 
213554 
213554 
213554 
213554 

7079866366105
 115561 
species
111111

115561 
115561 
115561 
115561 
115561 
115561 

 1118153 
species
111111
1914441

1118153 
1118153 
1118153 
1118153 
1118153 
1118153 

871821018719155
111111
species
 1883416 

1883416 
1883416 
1883416 
1883416 
1883416 
1883416 

533547197391
species
111111
 1504981 

1504981 
1504981 
1504981 
1504981 
1504981 
1504981 


1897729 
1897729 
1897729 
1897729 
1897729 
1897729 
 1897729 
species
111111
9114510666111101


507626 
507626 
507626 
507626 
507626 
507626 
 507626 
111111
species
60161119645390


1178482 
1178482 
1178482 
1178482 
1178482 
1178482 
 1178482 
111111
species
415449616460


2136172 
2136172 
2136172 
2136172 
2136172 
2136172 
 2136172 
species
111111
13416513781183195

11
species
11
 1962264 


1962264 
1962264 

31
114185
genus
11


114186 


114186 
31
 114186 
species
11

376488
genus
111111
87971209719185

87971209719185
 376489 
species
111111

376489 
376489 
376489 
376489 
376489 
376489 

467957
order
118969
1757908012611324

346745
family
444
16508263926614

10658262906614
445
genus
245645


449 
 449 
1
species
1


66969 
66969 


66969 
 66969 
species
111
111


96230 
1
species
1
 96230 

581
111
species
 452 


452 
452 
452 


29423 
29423 
29423 
29423 
 29423 
1111
species
1342

species
1
 450 
1


450 


1867846 
1867846 
 1867846 
species
11
11


446 
446 
446 
446 
446 
446 
 446 
111111
species
10647856775510


45067 
2
 45067 
species
1


28087 

28087 
28087 
28087 
1111
species
 28087 
1121

111
genus
465
58512

111
species
 451 
58512

451 

451 
451 

107817344710
121212
family
118968

776
121212
genus
107817344710


777 
777 
777 
777 
777 
777 
10771733479
 777 
species
111111


2054173 

2054173 
 2054173 
11
species
11


325775 
 325775 
species
1
1

60357390605869460047539417013991541667
72274
order
126121126127120122

468
family
363136373032
1656429600879545465

497
genus
10411948
651743231610

4411
 1699624 
species
111

1699624 

1699624 


1699624 

 2203895 
species
111111
326152

2203895 
2203895 
2203895 
2203895 
2203895 
2203895 

 1028416 
111111
species
2111111

1028416 
1028416 
1028416 
1028416 
1028416 
1028416 

species
11111
 45610 
24422

45610 
45610 
45610 
45610 

45610 


349106 

349106 
349106 
111
species
 349106 
221

43
 571800 
species
11


571800 
571800 


330922 
330922 
330922 
330922 
330922 
330922 
 330922 
111111
species
4108691

species
1111
 1699622 
4271

1699622 

1699622 
1699622 

1699622 

111
species
 261164 
131

261164 

261164 


261164 


1720344 

1720344 
1720344 

1720344 
2111
 1720344 
species
1111

 334543 
species
1111
1111

334543 

334543 
334543 
334543 

469
genus
222220232220
1504293480647282281


1808001 
1808001 
1808001 
1808001 
1808001 
1808001 
 1808001 
species
111111
2123535

 1758189 
species
1111
1144

1758189 

1758189 
1758189 
1758189 

 1407071 
species
11111
13283

1407071 

1407071 
1407071 
1407071 
1407071 


40215 
40215 
40215 
40215 
40215 
40215 
484162018
111111
species
 40215 


1646498 
1646498 
1646498 
1646498 
1646498 
1646498 
422263
species
111111
 1646498 


1789224 
1789224 
1789224 
113
 1789224 
species
111

 29430 
111111
species
3143101110

29430 
29430 
29430 
29430 
29430 
29430 

5594813
 2004644 
species
111111

2004644 
2004644 
2004644 
2004644 
2004644 
2004644 


108981 
108981 
108981 
108981 
108981 
108981 
41515121432
 108981 
111111
species

1513
1111
species
 2079596 


2079596 
2079596 
2079596 
2079596 


1608473 
1608473 
1608473 
1608473 
1608473 
2311110
11111
species
 1608473 

5431611
species
111111
 40216 

40216 
40216 
40216 
40216 
40216 
40216 


1871111 

1871111 
1871111 
1871111 
1871111 
11111
species
 1871111 
12261


106648 
106648 

106648 
106648 
106648 
species
11111
 106648 
34332

94116106
species
111111
 756892 

756892 
756892 
756892 
756892 
756892 
756892 


1636603 
1636603 
1636603 
1636603 
1636603 
1636603 
111111
species
 1636603 
575495


40214 
40214 
40214 
40214 
40214 
40214 
4212652011
 40214 
111111
species


62977 
62977 

62977 
62977 
62977 
 62977 
species
11111
11111

1351
 1324350 
species
1111


1324350 
1324350 
1324350 

1324350 

species group
553344
909768
1449199377484142168

111111
species
 48296 
1591655197

48296 
48296 
48296 
48296 
48296 
48296 

2111
1111
species
 471 

471 
471 


471 
471 

83442433
species
111111
 106654 

106654 
106654 
106654 
106654 
106654 
106654 

 1785128 
species
11
11

1785128 
1785128 


470 
470 
470 
470 
470 
470 
1423154357405119157
 470 
111111
species


1148157 
1148157 

1148157 
1148157 
1148157 
12131
 1148157 
species
11111

475
genus
455544
8711977209247174

658228179177113
 34062 
111111
species

34062 
34062 
34062 
34062 
34062 
34062 


476 
476 
476 
476 

476 
11111
species
 476 
111121


386891 
386891 
386891 
386891 
386891 
386891 
498556
 386891 
species
111111

8121
 29433 
1111
species


29433 
29433 
29433 
29433 


480 
480 
480 
480 
480 
480 
 480 
111111
species
171939116454

909090909090
family
135621
60191790562969400047451517008541541202

230931693046123944743064
351
222222
subfamily

230931693046123944743064
352
222222
genus

species
111111
 353 
10101382132251017881348

353 
353 
353 
353 
353 
353 


354 
354 
354 
354 
354 
354 
12991787172472926861716
 354 
111111
species

1849530
genus
111111
921927598517


1697053 
1697053 
1697053 
1697053 
1697053 
1697053 
921927598517
 1697053 
111111
species

genus
878787878787
286
59951690244169092747321716962951538121


1856685 
1856685 
1856685 
1856685 
1856685 
1856685 
4557288113481233733
 1856685 
species
111111

9381224150457020831637
species
111111
 157783 

157783 
157783 
157783 
157783 
157783 
157783 


1259844 
1259844 
1259844 
1259844 
1259844 
1259844 
10461210143148523891475
species
111111
 1259844 


219572 
219572 
219572 
219572 
219572 
219572 
species
111111
 219572 
8853201925181390425311930

 1898684 
111111
species
5867349343201170684

1898684 
1898684 
1898684 
1898684 
1898684 
1898684 


1853130 
1853130 
1853130 
1853130 
1853130 
1853130 
 1853130 
111111
species
17781990233584940422051

9955113981319042251958112988
111111
species
 930166 

930166 
930166 
930166 
930166 
930166 
930166 

 1583341 
species
111111
33133862567463166652463132

1583341 
1583341 
1583341 
1583341 
1583341 
1583341 

228339266580142695190379830512134270
species group
111111111111
136843


200450 
200450 
200450 
200450 
200450 
200450 
 200450 
111111
species
5037226724351090182922044


76758 
76758 
76758 
76758 
76758 
76758 
111111
species
 76758 
967717922132992154821998


75612 
75612 
75612 
75612 
75612 
75612 
 75612 
111111
species
17692245240794738632143

 380021 
111111
species
11332127341403351342951413236

380021 
380021 
380021 
380021 
380021 
380021 


47883 
47883 
47883 
47883 
47883 
47883 
111111
species
 47883 
408117221989953154311784

111111
species
 200451 
382321572305925139402469

200451 
200451 
200451 
200451 
200451 
200451 


47879 
47879 
47879 
47879 
47879 
47879 
12811664184570530391673
111111
species
 47879 


47878 
47878 
47878 
47878 
47878 
47878 
10981434651132403437235205
species
111111
 47878 


294 
294 
294 
294 
294 
294 
species
111111
 294 
17411423402810603317551766731099508

 76761 
species
111111
4572214824711055153222422

76761 
76761 
76761 
76761 
76761 
76761 


46679 
46679 
46679 
46679 
46679 
46679 
16721477193265845961788
 46679 
111111
species


1028989 
1028989 
1028989 
1028989 
1028989 
1028989 
13832107244683122761943
species
111111
 1028989 


198620 
198620 
198620 
198620 
198620 
198620 
 198620 
111111
species
6646835884883158151166991

5548217252831138731
 237610 
species
111111

237610 
237610 
237610 
237610 
237610 
237610 

7169739463161262808
 1294143 
111111
species

1294143 
1294143 
1294143 
1294143 
1294143 
1294143 


1649877 
1649877 
1649877 
1649877 
1649877 
1649877 
111111
species
 1649877 
10881410161260826301712

9001133132545220731199
species
111111
 1788301 

1788301 
1788301 
1788301 
1788301 
1788301 
1788301 

18292264259790839832335
 1659194 
111111
species

1659194 
1659194 
1659194 
1659194 
1659194 
1659194 


1500687 
1500687 
1500687 
1500687 
1500687 
1500687 
16941978228278535861994
111111
species
 1500687 

5187919603391300777
 1930532 
species
111111

1930532 
1930532 
1930532 
1930532 
1930532 
1930532 


2083051 
2083051 
2083051 
2083051 
2083051 
2083051 
 2083051 
species
111111
709956102035114941132

69797310273831477859
 69328 
species
111111

69328 
69328 
69328 
69328 
69328 
69328 

704012943832089891504891223331020189
136849
888888
species group

148029162705103519623006
species
111111
 46257 

46257 
46257 
46257 
46257 
46257 
46257 


33069 
33069 
33069 
33069 
33069 
33069 
178131103401132529394328
 33069 
species
111111

5508526607317825313953198234986974
251695
111111
species subgroup


317 
317 
317 
317 
317 
317 
5508526607317825313953198234986974
 317 
species
111111

251698
species subgroup
222222
489310140117163566733013523

 29438 
111111
species
245754535941179238127589

29438 
29438 
29438 
29438 
29438 
29438 

species
111111
 47877 
243646875775177435185934

47877 
47877 
47877 
47877 
47877 
47877 


251701 
251701 
251701 
251701 
251701 
251701 
482689079100349174059590
 251701 
111111
species


1206777 
1206777 
1206777 
1206777 
1206777 
1206777 
9161058134544721041044
species
111111
 1206777 


36746 
36746 
36746 
36746 
36746 
36746 
 36746 
111111
species
142021792469109423591724

111111
species
 1931241 
152267204109212179

1931241 
1931241 
1931241 
1931241 
1931241 
1931241 


237609 
237609 
237609 
237609 
237609 
237609 
13141730197364025642045
111111
species
 237609 

 1500686 
species
111111
16781869231572541841899

1500686 
1500686 
1500686 
1500686 
1500686 
1500686 

 1827300 
111111
species
391218632144940147111719

1827300 
1827300 
1827300 
1827300 
1827300 
1827300 


1283291 
1283291 
1283291 
1283291 
1283291 
1283291 
229630183470116451652873
 1283291 
species
111111


2213057 
2213057 
2213057 
2213057 
2213057 
2213057 
species
111111
 2213057 
517701662217838759


65741 
65741 
65741 
65741 
65741 
65741 
6248659384041347838
 65741 
111111
species

4207201323801070154292034
 2083055 
species
111111

2083055 
2083055 
2083055 
2083055 
2083055 
2083055 

7550100321082642701492610110
species group
222222
136846


74829 
74829 
74829 
74829 
74829 
74829 
493726510200748470
 74829 
111111
species

70579306103164070141789640
species subgroup
111111
578833

70579306103164070141789640
111111
species
 316 

316 
316 
316 
316 
316 
316 

136842
species group
333333
277052968934311114396637731462


47884 
47884 
47884 
47884 
47884 
47884 
 47884 
species
111111
7941024126552617591108


587753 
587753 
587753 
587753 
587753 
587753 
24765259902985698445982827449
111111
species
 587753 

214626753190106947902905
111111
species
 296 

296 
296 
296 
296 
296 
296 


2083054 
2083054 
2083054 
2083054 
2083054 
2083054 
29942121219593188252330
species
111111
 2083054 

136841
species group
777777
79948999711000424151618377399792

17102287255189332922303
1232139
222222
species subgroup


1149133 
1149133 
1149133 
1149133 
1149133 
1149133 
 1149133 
111111
species
6188608352871272635

10921427171660620201668
111111
species
 330 

330 
330 
330 
330 
330 
330 

 43263 
species
111111
6998949693461469900

43263 
43263 
43263 
43263 
43263 
43263 


53412 
53412 
53412 
53412 
53412 
53412 
111111
species
 53412 
72193611034621494930

325441134936184771574370
species
111111
 300 

300 
300 
300 
300 
300 
300 

7160289326878053704616640088822
species
111111
 287 

287 
287 
287 
287 
287 
287 

19622415267892239612467
 53408 
species
111111

53408 
53408 
53408 
53408 
53408 
53408 

111111
species
 2201356 
19832334259698246412380

2201356 
2201356 
2201356 
2201356 
2201356 
2201356 


2083053 
2083053 
2083053 
2083053 
2083053 
2083053 
8551027119745817341210
 2083053 
111111
species

 1534110 
111111
species
20962536283690547632567

1534110 
1534110 
1534110 
1534110 
1534110 
1534110 

206276405141467337
111111
species
 1981174 

1981174 
1981174 
1981174 
1981174 
1981174 
1981174 


2049589 
2049589 
2049589 
2049589 
2049589 
2049589 
11641467141952518911516
 2049589 
species
111111

species
111111
 2067572 
20070248925571032176282300

2067572 
2067572 
2067572 
2067572 
2067572 
2067572 

15941915218481836691966
 157782 
species
111111

157782 
157782 
157782 
157782 
157782 
157782 


1338689 
1338689 
1338689 
1338689 
1338689 
1338689 
68775310853621374987
111111
species
 1338689 

species
111111
 2069256 
770908116345815341349

2069256 
2069256 
2069256 
2069256 
2069256 
2069256 

11131396172055621921866
 1636610 
111111
species

1636610 
1636610 
1636610 
1636610 
1636610 
1636610 

4384201224341046164082078
species
111111
 2018067 

2018067 
2018067 
2018067 
2018067 
2018067 
2018067 

 1415630 
species
111111
4603188123021107168191993

1415630 
1415630 
1415630 
1415630 
1415630 
1415630 

5391639573702791125316216
111111
species
 104087 

104087 
104087 
104087 
104087 
104087 
104087 


1611770 
1611770 
1611770 
1611770 
1611770 
1611770 
20362224267796248712421
111111
species
 1611770 


2083052 
2083052 
2083052 
2083052 
2083052 
2083052 
63980910723701643989
 2083052 
species
111111


101564 
101564 
101564 
101564 
101564 
101564 
 101564 
species
111111
530604723266920634


216142 
216142 
216142 
216142 
216142 
216142 
species
111111
 216142 
2309252833535204342134200


312306 
312306 
312306 
312306 
312306 
312306 
9891200146957224341390
species
111111
 312306 

5450065497763662642212297376426
species group
666666
136845


47880 
47880 
47880 
47880 
47880 
47880 
13611884214889734622005
 47880 
111111
species


78327 
78327 
78327 
78327 
78327 
78327 
369145284793170680815240
111111
species
 78327 

404556275976223487126213
111111
species
 76759 

76759 
76759 
76759 
76759 
76759 
76759 

species
111111
 47885 
5176847292961070780

47885 
47885 
47885 
47885 
47885 
47885 

species
111111
 303 
439475177461497208999979060929

303 
303 
303 
303 
303 
303 


70775 
70775 
70775 
70775 
70775 
70775 
species
111111
 70775 
9391000122339018581259

4392197025701122162282537
 2219057 
111111
species

2219057 
2219057 
2219057 
2219057 
2219057 
2219057 


515393 
515393 
515393 
515393 
515393 
515393 
 515393 
111111
species
7597230925391143179112140

4566305027541093180353258
 244566 
111111
species

244566 
244566 
244566 
244566 
244566 
244566 


1207075 
1207075 
1207075 
1207075 
1207075 
1207075 
111111
species
 1207075 
17472067209582243321751

742030
order
111111
8691775481106

111111
family
742031
8691775481106

111111
genus
180541
8691775481106

 2183911 
111111
species
8691775481106

2183911 
2183911 
2183911 
2183911 
2183911 
2183911 

688725637327
111111
order
1934945

688725637327
1934946
111111
family

688725637327
1934947
111111
genus

688725637327
species
111111
 1810504 

1810504 
1810504 
1810504 
1810504 
1810504 
1810504 

221
order
111
135615

221
111
family
868

869
111
genus
221


870 

870 


870 
111
species
 870 
221

1512
111
species
 113267 


113267 
113267 

113267 

4147161
111111
genus
1524249

4147161
species
111111
 1249552 

1249552 
1249552 
1249552 
1249552 
1249552 
1249552 

1775403
111111
order
234917143916

111111
family
568386
234917143916

469322
genus
111111
234917143916

234917143916
 465721 
111111
species

465721 
465721 
465721 
465721 
465721 
465721 

10249118601056794570
order
312935352928
135623

10249118601056794570
641
family
312935352928

1111619155
111111
genus
246861

 673 
111111
species
1111619155

673 
673 
673 
673 
673 
673 

1112
2042066
1111
genus


1755811 
1755811 
1755811 

1755811 
species
1111
 1755811 
1112

9768678011008756541
262229282521
genus
662

 689 
1111
species
1311


689 
689 
689 
689 

1111
species
 1435069 
1511115

1435069 

1435069 
1435069 
1435069 

32
 76258 
11
species


76258 
76258 

2419
 553239 
1111
species


553239 
553239 
553239 
553239 


29494 
29494 
29494 
29494 
29494 
29494 
species
111111
 29494 
182619131710


674 
674 
674 
674 
674 
674 
species
111111
 674 
336932154


52443 
52443 
52443 
52443 
52443 
52443 
species
111111
 52443 
6121072412


687 

687 
687 
 687 
111
species
828

 1534743 
species
111111
359783

1534743 
1534743 
1534743 
1534743 
1534743 
1534743 

11111
species
 673372 
43431

673372 
673372 
673372 
673372 

673372 

 1116375 
species
111111
31116151

1116375 
1116375 
1116375 
1116375 
1116375 
1116375 


676 
676 
676 
676 
676 
676 
 676 
species
111111
554175652619

 666 
species
111111
420331307383324187

666 
666 
666 
666 
666 
666 

species
1
 212663 
2

212663 

21597311
 45658 
species
111111

45658 
45658 
45658 
45658 
45658 
45658 


672 
672 
672 
672 
672 
672 
8495851163562
 672 
species
111111

species
111111
 55601 
915534517028

55601 
55601 
55601 
55601 
55601 
55601 

879887
species group
717610
194189175283184198


670 
670 
670 
670 
670 
670 
312954718227
 670 
species
111111


190895 

190895 
190895 
190895 
2193
1111
species
 190895 

574841561236
 663 
111111
species

663 
663 
663 
663 
663 
663 

4511766111
species
111111
 691 

691 
691 
691 
691 
691 
691 


150340 
species
1
 150340 
1

414510126
 50719 
111111
species

50719 
50719 
50719 
50719 
50719 
50719 


680 
680 
680 
680 
680 
680 
 680 
species
111111
346234303877


669 
669 
669 
669 
669 
669 
111111
species
 669 
10181983530


696485 
696485 
696485 
696485 
696485 
696485 
species
111111
 696485 
117133321

1
1
species
 2025808 

2025808 

species group
111
1891919
2324


29498 
29498 
29498 
species
111
 29498 
2324


2014742 
2014742 
2014742 
2014742 
2014742 
 2014742 
species
11111
62434


28173 
28173 
28173 
28173 
28173 
28173 
 28173 
species
111111
1615421


190893 
190893 
190893 
190893 
190893 
190893 
111111
species
 190893 
11151316103

2214102
121212
genus
511678


668 
668 
668 
668 
668 
species
11111
 668 
112101


80852 
80852 

80852 

80852 
2121
 80852 
species
1111

657
genus
333323
353051241320


1295392 
1295392 
1295392 
1295392 
1295392 
1295392 
862491114
111111
species
 1295392 


74109 
74109 
74109 
74109 

74109 
11223
species
11111
 74109 


38293 
38293 
38293 
38293 
38293 
38293 
2623251323
 38293 
111111
species

321436307317446308
1706369
order
161516161515

212638221668
333322
family
1706375

630749
genus
111111
10152861049

10152861049
 1620392 
species
111111

1620392 
1620392 
1620392 
1620392 
1620392 
1620392 

477719
1084558
11111
genus

 716816 
species
11111
477719

716816 
716816 
716816 
716816 

716816 

1434050
11111
genus
74396

74396
species
11111
 1470434 

1470434 
1470434 
1470434 
1470434 
1470434 

1181119313074104
family
444444
1706373

genus
444444
48073
1181119313074104


1769779 
1769779 
1769779 
1769779 
1769779 
1769779 
506848533226
111111
species
 1769779 

41252453753
 252514 
111111
species

252514 
252514 
252514 
252514 
252514 
252514 

241114222922
species
111111
 260552 

260552 
260552 
260552 
260552 
260552 
260552 


359370 
359370 
359370 
359370 
359370 
359370 
 359370 
111111
species
377263

514328347135
family
222222
1706372

514328347135
1217416
genus
222222

 930805 
species
111111
8415123112

930805 
930805 
930805 
930805 
930805 
930805 


930806 
930806 
930806 
930806 
930806 
930806 
433913224023
 930806 
111111
species

131256148131285101
1706371
family
767777

316625
111111
genus
1325915352


86304 
86304 
86304 
86304 
86304 
86304 
 86304 
111111
species
1325915352

81571754
2425
genus
111111


2426 
2426 
2426 
2426 
2426 
2426 
species
111111
 2426 
81571754

44532
genus
11111
2036021


1737490 

1737490 
1737490 
1737490 
1737490 
11111
species
 1737490 
44532

447467
111111
genus
3596121126


447471 
447471 
447471 
447471 
447471 
447471 
 447471 
111111
species
3596121126

genus
333333
10
712071228213087

286945213417
species
111111
 1987723 

1987723 
1987723 
1987723 
1987723 
1987723 
1987723 

111111
species
 155077 
189533365254

155077 
155077 
155077 
155077 
155077 
155077 


1945512 
1945512 
1945512 
1945512 
1945512 
1945512 
254344254416
 1945512 
111111
species

11111
species
 83406 
1672142

83406 
83406 
83406 

83406 
83406 

class
423231
1553900
341223241310

2024979
21212
order
32335

22234
family
11111
1652132

22234
1652133
genus
11111

 97084 
11111
species
22234

97084 
97084 
97084 
97084 
97084 

111
263369
111
family

146784
111
genus
111


960 

960 

960 
 960 
species
111
111

31102021810
order
211111
213481

31102021810
family
211111
213483

31102021810
genus
211111
958

 453816 
species
1
6

453816 

 959 
species
111111
25102021810

959 
959 
959 
959 
959 
959 

977383867777
subphylum
68525
621424401604211619771534

1581359183188195282
29547
321419211315
class

genus
11
269258
31


387092 


387092 
species
11
 387092 
31

21112
genus
265570
25494

species
11111
 387093 
15493

387093 

387093 
387093 
387093 
387093 

11
species
11
 206403 

206403 


206403 

11113
235899
order
11111

11113
224467
11111
family

191291
genus
11111
11113


244787 
244787 
244787 
244787 

244787 
 244787 
11111
species
11113

order
281317191211
213849
1575358177183186274

311215
269260
genus
11111


269261 
269261 
269261 
269261 
269261 
species
11111
 269261 
311215

family
179101277
72294
10433536383729

9483222233214
194
868744
genus


196 


196 
species
11
 196 
13


522485 
 522485 
1
species
11

41
11
species
 1244531 

1244531 
1244531 


201 

201 
201 
201 
1221
 201 
species
1111

2331
 200 
1111
species

200 
200 
200 


200 

3
 198 
1
species


198 

1
 199 
species
1


199 


824 
824 
824 
221
 824 
111
species


206 


206 
11
 206 
11
species

11111
species
 195 
11212

195 
195 
195 
195 
195 

3311
111
species
 1965231 

1965231 
1965231 
1965231 

111111
species
 197 
8952110112811

197 
197 
197 
197 
197 
197 

511
 76517 
111
species


76517 
76517 


76517 

57665
genus
62311
352311


1581011 
1
species
1
 1581011 

species
111
 220622 
111

220622 
220622 


220622 


1986224 
1986224 

1986224 
211
111
species
 1986224 


65553 
 65553 
1
species
29

1
species
 44674 
1

44674 


366522 


366522 
 366522 
species
11
11

1
species
1
 66821 

66821 

1
 194424 
species
1


194424 

28196
genus
312222
6011412414

5218235
 28197 
111111
species

28197 
28197 
28197 
28197 
28197 
28197 

261019
 28199 
species
11111

28199 

28199 
28199 
28199 
28199 

1
species
 944547 
6

944547 

72293
family
1036644
529312139144144245

843
genus
11
502

 844 
11
species
502

844 

844 

361
286130
genus
11


148813 


148813 
361
 148813 
species
11

genus
634542
209
200312136142144243

1
species
 213 
2

213 


1591088 
1591088 
1591088 
1591088 
1591088 
 1591088 
11111
species
95413

11
species
 37372 
11

37372 

37372 


214 


214 
214 
341
species
111
 214 

182267123123136226
111111
species
 210 

210 
210 
210 
210 
210 
210 

340813417
species
111111
 138563 

138563 
138563 
138563 
138563 
138563 
138563 

 135569 
1
species
1


135569 

243121
genus
2111
202746


39766 

39766 
39766 
species
111
 39766 
23912

41
11
species
 202747 

202747 


202747 

28221
class
655964656462
463320811421192817821252

2075411131412
111111
order
1779134

2075411131412
1779135
111111
family

genus
111111
1779136
2075411131412

111111
species
 1548548 
2075411131412

1548548 
1548548 
1548548 
1548548 
1548548 
1548548 

3411437248
order
111111
453227

453228
family
111111
3411437248

111111
genus
453229
3411437248


453230 
453230 
453230 
453230 
453230 
453230 
3411437248
111111
species
 453230 

334375195267296156
69541
171517171716
order

96141639213548
family
656666
213421

69109347111512
434444
genus
18


29542 
29542 
29542 
29542 
29542 
29542 
species
111111
 29542 
2411528123

 19 
11111
species
332214

19 

19 
19 
19 
19 


29543 
29543 
29543 
29543 
29543 
29543 
111111
species
 29543 
15791428792

272921333
species
111111
 1842532 

1842532 
1842532 
1842532 
1842532 
1842532 
1842532 

222222
genus
890
273229212036

11818669
111111
species
 1603606 

1603606 
1603606 
1603606 
1603606 
1603606 
1603606 

111111
species
 1823759 
162411151427

1823759 
1823759 
1823759 
1823759 
1823759 
1823759 

111011111110
family
213422
238234132175161108

28231
1091010109
genus
228230126164147106

1584814915
 443144 
species
111111

443144 
443144 
443144 
443144 
443144 
443144 

401051133
111111
species
 351604 

351604 
351604 
351604 
351604 
351604 
351604 

111111
species
 28232 
28122613382

28232 
28232 
28232 
28232 
28232 
28232 


313985 
313985 
313985 
313985 
313985 
313985 
species
111111
 313985 
61821421

4153
 1203471 
species
1111

1203471 

1203471 
1203471 
1203471 


225194 
225194 
225194 
225194 
225194 
225194 
1421181298
species
111111
 225194 


35554 
35554 
35554 
35554 
35554 
35554 
642514284722
111111
species
 35554 


1340425 
1340425 
1340425 
1340425 
1340425 
1340425 
 1340425 
species
111111
472229201726

41216241517
species
111111
 443143 

443143 
443143 
443143 
443143 
443143 
443143 


345632 
345632 
345632 
345632 
345632 
345632 
 345632 
species
111111
626723412

genus
111111
392332
104611142


483547 
483547 
483547 
483547 
483547 
483547 
 483547 
111111
species
104611142

213118
789897
order
28733415413133

151522164316
family
334444
213121

genus
111111
893
6710767

species
111111
 1986146 
6710767

1986146 
1986146 
1986146 
1986146 
1986146 
1986146 

111111
genus
427922
5676296


427923 
427923 
427923 
427923 
427923 
427923 
5676296
 427923 
111111
species

11111
genus
53318
44172


65555 

65555 
65555 
65555 
65555 
44172
 65555 
11111
species

21211
109168
genus
11111


84980 
84980 
84980 
84980 
84980 
11111
species
 84980 
21211

2721819388817
family
455453
213119

111111
genus
218207
491711458


259354 
259354 
259354 
259354 
259354 
259354 
491711458
 259354 
111111
species

111
genus
111
28222


28223 
28223 

28223 
 28223 
species
111
111

genus
222221
896
22215623408


897 
897 
897 
897 
897 
897 
21114512298
111111
species
 897 

 181663 
species
11111
11111111

181663 
181663 
181663 
181663 
181663 

2295
111111
genus
115421


2296 
2296 
2296 
2296 
2296 
2296 
115421
species
111111
 2296 

10899538171106911680
181818181818
order
29

396239694826
suborder
111111
224462

224464
family
111111
396239694826

genus
111111
162027
396239694826


80816 
80816 
80816 
80816 
80816 
80816 
396239694826
 80816 
111111
species

80812
333333
suborder
208271208271243211

49
222222
family
195137168230182185

50
genus
111111
254814252612


52 
52 
52 
52 
52 
52 
111111
species
 52 
254814252612

17089154205156173
111111
genus
39643

17089154205156173
111111
species
 56 

56 
56 
56 
56 
56 
56 

1313440416126
family
111111
1055686

1313440416126
1055688
genus
111111

1313440416126
111111
species
 927083 

927083 
927083 
927083 
927083 
927083 
927083 

842620570766620443
80811
suborder
141414141414

460184172228152175
39
444444
family

252822334915
111111
genus
47

 48 
111111
species
252822334915

48 
48 
48 
48 
48 
48 

3516435443371
genus
111111
44

 83453 
111111
species
3516435443371

83453 
83453 
83453 
83453 
83453 
83453 

111111
genus
40
24183463149

 41 
species
111111
24183463149

41 
41 
41 
41 
41 
41 

607481885680
111111
genus
42

111111
species
 43 
607481885680

43 
43 
43 
43 
43 
43 

288357239297295149
31
666666
family

32
genus
555555
24332819626725697

 33 
111111
species
578788757930

33 
33 
33 
33 
33 
33 

404037326314
 34 
species
111111

34 
34 
34 
34 
34 
34 

species
111111
 35 
192924411016

35 
35 
35 
35 
35 
35 

468110622922
species
111111
 1297742 

1297742 
1297742 
1297742 
1297742 
1297742 
1297742 


83455 
83455 
83455 
83455 
83455 
83455 
111111
species
 83455 
819137577515

452943303952
83461
genus
111111


184914 
184914 
184914 
184914 
184914 
184914 
 184914 
111111
species
452943303952

family
333333
1524215
8875146213154107

genus
333333
161492
8875146213154107

 161493 
111111
species
4240741008356

161493 
161493 
161493 
161493 
161493 
161493 


404589 
404589 
404589 
404589 
404589 
404589 
82114681936
 404589 
species
111111

381458455215
 447217 
111111
species

447217 
447217 
447217 
447217 
447217 
447217 

6413281912
1524213
family
111111

1524214
111111
genus
6413281912


1391653 
1391653 
1391653 
1391653 
1391653 
1391653 
species
111111
 1391653 
6413281912

213462
422343
order
332711149

316566
213465
111111
family

genus
111111
29526
316566


119484 
119484 
119484 
119484 
119484 
119484 
 119484 
111111
species
316566

3011683
311232
family
213468

2357
genus
1111
2141

2141
1111
species
 2358 

2358 


2358 
2358 
2358 

11111
genus
60892
261522


60893 
60893 

60893 
60893 
60893 
261522
species
11111
 60893 

43773
genus
111
212


316277 

316277 

316277 
212
111
species
 316277 

758703344438392353
order
151414151415
213115

564614290404358314
family
121111131212
194924

495477262351316286
872
878988
genus


901 
901 
901 
901 
901 
901 
32712137
species
111111
 901 


44742 
44742 
44742 
44742 
44742 
44742 
124383144178162178
species
111111
 44742 


873 
873 
873 
873 
873 
873 
 873 
111111
species
10717102320

12148312717
 184917 
111111
species

184917 
184917 
184917 
184917 
184917 
184917 


631220 
631220 
631220 
631220 
631220 
631220 
 631220 
111111
species
68720210

species
111111
 881 
3213852676028

881 
881 
881 
881 
881 
881 


876 
876 
876 
876 
876 
876 
111111
species
 876 
122522112624

752032
species
11111
 58180 

58180 

58180 
58180 
58180 
58180 


880 
2
1
species
 880 

genus
111111
41707
810210511


29546 
29546 
29546 
29546 
29546 
29546 
810210511
 29546 
111111
species

genus
332333
2035811
6112726433717

1538108155
111111
species
 182210 

182210 
182210 
182210 
182210 
182210 
182210 


1716143 
1716143 
1716143 
1716143 
1716143 
1716143 
202016321910
species
111111
 1716143 


57320 
57320 

57320 
57320 
57320 
2669332
 57320 
11111
species

family
222222
213116
428852343432

222222
genus
898
428852343432

 899 
species
111111
367642213323

899 
899 
899 
899 
899 
899 

612101319
 132132 
111111
species

132132 
132132 
132132 
132132 
132132 
132132 

152127
family
1111
213117

152127
45662
1111
genus


45663 
45663 
45663 


45663 
152127
 45663 
1111
species

1551504
1111
genus
1111


673862 

673862 
673862 

673862 
1111
 673862 
species
1111

2211
213113
order
111

111
family
117942
2211

2211
84404
111
genus

2211
 84405 
species
111

84405 

84405 
84405 

580370
class
112221
1382081

580371
112221
order
1382081

1382081
family
112221
580372

1382081
377315
genus
112221

1111
species
 1921087 
15167

1921087 

1921087 
1921087 
1921087 

33411
species
11111
 1921086 


1921086 
1921086 
1921086 
1921086 
1921086 

1991842506611
1807140
class
333333

1991842506611
order
333333
225057

333333
family
225058
1991842506611

1991842506611
119977
genus
333333


33059 
33059 
33059 
33059 
33059 
33059 
19412214246
111111
species
 33059 

 920 
111111
species
3161720352

920 
920 
920 
920 
920 
920 

 160808 
111111
species
2131673

160808 
160808 
160808 
160808 
160808 
160808 

28211
371367375393377381
class
283281905533701471265569326564

676868686868
order
204457
7557251011905142592534510375

62438793612471973850
family
191919191919
335929

18142330444743318
777777
genus
361177

368736511549
111111
species
 543877 

543877 
543877 
543877 
543877 
543877 
543877 


1267766 
1267766 
1267766 
1267766 
1267766 
1267766 
19524535725
 1267766 
111111
species

51413133
 476157 
species
111111

476157 
476157 
476157 
476157 
476157 
476157 


1982042 
1982042 
1982042 
1982042 
1982042 
1982042 
111111
species
 1982042 
258537211650


645517 
645517 
645517 
645517 
645517 
645517 
species
111111
 645517 
4011616714869

111111
species
 692370 
3447310621689

692370 
692370 
692370 
692370 
692370 
692370 


361183 
361183 
361183 
361183 
361183 
361183 
22542687833
111111
species
 361183 

8846121196261128
222222
genus
1295327


450378 
450378 
450378 
450378 
450378 
450378 
species
111111
 450378 
66418814416791


1348774 
1348774 
1348774 
1348774 
1348774 
1348774 
22533529437
 1348774 
species
111111

155140194238369171
1111
333333
genus

313477010646
species
111111
 2023229 

2023229 
2023229 
2023229 
2023229 
2023229 
2023229 

71121596210147
species
111111
 1896196 

1896196 
1896196 
1896196 
1896196 
1896196 
1896196 

 1112 
111111
species
53168810616278

1112 
1112 
1112 
1112 
1112 
1112 

1041
777777
genus
200159291369600233


2011159 
2011159 
2011159 
2011159 
2011159 
2011159 
25328475028
 2011159 
species
111111


1648404 
1648404 
1648404 
1648404 
1648404 
1648404 
245522272912
111111
species
 1648404 

 1922225 
111111
species
8113203

1922225 
1922225 
1922225 
1922225 
1922225 
1922225 


266951 
266951 
266951 
266951 
266951 
266951 
 266951 
species
111111
12326609424

 502682 
111111
species
311842517831

502682 
502682 
502682 
502682 
502682 
502682 


192812 
192812 
192812 
192812 
192812 
192812 
2528655710467
111111
species
 192812 


39960 
39960 
39960 
39960 
39960 
39960 
111111
species
 39960 
755110712422568

693321231096913012233729525
41297
484949494949
family

8473691258161329211080
165697
899999
genus


117207 
117207 
117207 
117207 
117207 
117207 
87608512218579
 117207 
111111
species


1866325 
1866325 
1866325 
1866325 
1866325 
1866325 
species
111111
 1866325 
8328155176343139

7811136169358118
 267128 
111111
species

267128 
267128 
267128 
267128 
267128 
267128 

8915108175212126
111111
species
 1515612 

1515612 
1515612 
1515612 
1515612 
1515612 
1515612 

 33050 
111111
species
3001534715261086365

33050 
33050 
33050 
33050 
33050 
33050 

948010416120274
 33052 
111111
species

33052 
33052 
33052 
33052 
33052 
33052 


1357916 
1357916 
1357916 
1357916 
1357916 
1357916 
809137181330111
 1357916 
111111
species


292913 
292913 
292913 
292913 
292913 
292913 
36116110020149
 292913 
111111
species

213419
11111
species
 1913578 


1913578 
1913578 
1913578 
1913578 
1913578 

111111
genus
150203
117122123185291149


1842535 
1842535 
1842535 
1842535 
1842535 
1842535 
117122123185291149
 1842535 
species
111111

394678366627516140615663
13687
141414141414
genus


1327635 
1327635 
1327635 
1327635 
1327635 
1327635 
27175325395762332
111111
species
 1327635 


1921510 
1921510 
1921510 
1921510 
1921510 
1921510 
562961689737
species
111111
 1921510 

661611516320893
111111
species
 1030157 

1030157 
1030157 
1030157 
1030157 
1030157 
1030157 


1549858 
1549858 
1549858 
1549858 
1549858 
1549858 
 1549858 
species
111111
58141153111272420900

 1609977 
111111
species
23941345541799334

1609977 
1609977 
1609977 
1609977 
1609977 
1609977 

12644144195289113
 745310 
111111
species

745310 
745310 
745310 
745310 
745310 
745310 

16831281340606254
 93064 
111111
species

93064 
93064 
93064 
93064 
93064 
93064 


1390395 
1390395 
1390395 
1390395 
1390395 
1390395 
species
111111
 1390395 
300754856931198457


160791 
160791 
160791 
160791 
160791 
160791 
344111481594971386
 160791 
111111
species

 397260 
species
111111
19998275374655302

397260 
397260 
397260 
397260 
397260 
397260 

664871241130827581051
 152682 
111111
species

152682 
152682 
152682 
152682 
152682 
152682 

390455126871217528
 1560345 
111111
species

1560345 
1560345 
1560345 
1560345 
1560345 
1560345 


1938607 
1938607 
1938607 
1938607 
1938607 
1938607 
21027308380760328
111111
species
 1938607 

332635586511321548
species
111111
 1961362 

1961362 
1961362 
1961362 
1961362 
1961362 
1961362 

genus
444444
165696
3622415676871303467

 48935 
111111
species
45186510015173

48935 
48935 
48935 
48935 
48935 
48935 


158500 
158500 
158500 
158500 
158500 
158500 
species
111111
 158500 
215120316379856227

species
111111
 702113 
6389100114154110

702113 
702113 
702113 
702113 
702113 
702113 


205844 
205844 
205844 
205844 
205844 
205844 
3914869414257
 205844 
111111
species

11011169239401146
1649486
111111
genus


1850238 
1850238 
1850238 
1850238 
1850238 
1850238 
 1850238 
111111
species
11011169239401146

5713938917771
72173
111111
genus


1634516 
1634516 
1634516 
1634516 
1634516 
1634516 
 1634516 
111111
species
5713938917771

541
genus
111111
22811341285682359


542 
542 
542 
542 
542 
542 
22811341285682359
species
111111
 542 

1434046
genus
444444
62238917519188


1806885 
1806885 
1806885 
1806885 
1806885 
1806885 
 1806885 
species
111111
81113484818


2077182 
2077182 
2077182 
2077182 
2077182 
2077182 
27639606631
 2077182 
species
111111


1922222 
1922222 
1922222 
1922222 
1922222 
1922222 
62771110
111111
species
 1922222 


266812 
266812 
266812 
266812 
266812 
266812 
 266812 
species
111111
21430606629

genus
141414141414
165695
12045501667222333451502

1065710212822285
 332055 
111111
species

332055 
332055 
332055 
332055 
332055 
332055 


484429 
484429 
484429 
484429 
484429 
484429 
 484429 
species
111111
10974122199254104


46429 
46429 
46429 
46429 
46429 
46429 
111111
species
 46429 
5137122115201115

747512015526497
111111
species
 1843368 

1843368 
1843368 
1843368 
1843368 
1843368 
1843368 


407020 
407020 
407020 
407020 
407020 
407020 
111111
species
 407020 
3177211912557


1855519 
1855519 
1855519 
1855519 
1855519 
1855519 
7064498911077
 1855519 
111111
species

25787410550833338
 13690 
111111
species

13690 
13690 
13690 
13690 
13690 
13690 


627192 
627192 
627192 
627192 
627192 
627192 
 627192 
111111
species
74229815018865

 1315974 
species
111111
9548158122255133

1315974 
1315974 
1315974 
1315974 
1315974 
1315974 

6614101132162107
111111
species
 1673076 

1673076 
1673076 
1673076 
1673076 
1673076 
1673076 


332056 
332056 
332056 
332056 
332056 
332056 
 332056 
species
111111
11633100142220106


76947 
76947 
76947 
76947 
76947 
76947 
 76947 
species
111111
192517112756


120107 
120107 
120107 
120107 
120107 
120107 
8722111167273113
 120107 
species
111111

 1332080 
species
111111
498518411149

1332080 
1332080 
1332080 
1332080 
1332080 
1332080 

1191478
order
1111
1367

family
1111
1191479
1367

1111
genus
162171
1367

 1124597 
1111
species
1367


1124597 
1124597 
1124597 
1124597 

order
112322
1921002
2412663

241312
11111
family
2100208

241312
1509243
11111
genus


1509244 

1509244 
1509244 
1509244 
1509244 
 1509244 
11111
species
241312

family
1
1777752
1

1
genus
1
1521255


91604 
1
species
 91604 
1

11251
44746
11111
family

 86106 
species
11111
11251


86106 
86106 
86106 
86106 
86106 

128112811219486103
766
order
182321272021

family
77711912
942
373039424250

1121
genus
768
5121


769 
1
 769 
species
1

51
species group
11
106179

11
species
 948 
51

948 


948 

11
species
11
 770 


770 
770 

12
genus
11
33993


951 
 951 
species
1
2


33994 
1
1
species
 33994 

102323151131
343447
tribe
952

genus
343447
953
102323151131

 77038 
111111
species
4151911418

77038 
77038 
77038 
77038 
77038 
77038 

 66084 
species
1
1


66084 


169402 
169402 

169402 

169402 
 169402 
species
1111
1211

1111
species
 246273 
1121


246273 
246273 

246273 
246273 

 100901 
species
1
2


100901 

553135
 77551 
111111
species

77551 
77551 
77551 
77551 
77551 
77551 

111
species
 163164 
223


163164 
163164 
163164 

333444
genus
943
22715242918


391036 
 391036 
species
1
1

333443
species group
106178
22715242917

843979
111111
species
 944 

944 
944 
944 
944 
944 
944 


35795 
35795 
72
 35795 
11
species

132113186
species
111111
 945 

945 
945 
945 
945 
945 
945 

111111
species
 779 
111522

779 
779 
779 
779 
779 
779 

1328881
family
1
1

1
genus
1
1802983


1802984 
1
species
 1802984 
1

775
family
10151216119
116197701524453

33988
9141115119
tribe
116096651424453

780
genus
8131014108
115782451363944

11472926192030
588976
species group
114277

86112
 35794 
species
111

35794 
35794 


35794 


35788 
1
species
 35788 
1


42862 
42862 
42862 
42862 
237623
species
1111
 42862 

species
11
 786 
12


786 
786 


35792 
species
1
 35792 
2


33992 
33992 
33992 
33992 
33992 
33992 
87134917
 33992 
species
111111

 33989 
species
11111
41231


33989 
33989 
33989 
33989 
33989 


783 
783 
783 
783 
783 
783 
 783 
111111
species
243323


781 

781 
species
11
 781 
12


35790 
35790 
35790 
35790 
35790 
35790 
species
111111
 35790 
3941211

21
11
species
 33991 


33991 

33991 


35791 
1
 35791 
species
1

 109232 
species
1
1


109232 

326
species
111
 47589 


47589 

47589 
47589 


337479 
337479 

337479 
337479 
 337479 
species
1111
114124

species group
121211
114292
6881347


785 

785 
21
 785 
species
11

6681247
 782 
species
111111

782 
782 
782 
782 
782 
782 

121211
species group
1129742
3311192117

3301191117
 33990 
species
111111

33990 
33990 
33990 
33990 
33990 
33990 

11
 788 
11
species


788 

788 

31420659
69474
genus
111111

111111
species
 784 
31420659

784 
784 
784 
784 
784 
784 

11510
2115980
genus
1111


2115978 
2115978 
2115978 
2115978 
 2115978 
1111
species
11510

 1528098 
111
species
8312

1528098 
1528098 
1528098 

356
149144152160154155
order
139251099315002235612162011457

136641544950
772
2261167
family

136641544950
2261167
genus
773

1
 774 
species
1


774 


803 
803 
803 
803 
803 
803 
113822123631
species
111111
 803 


1686310 
1686310 
1686310 
1686310 
1686310 
1686310 
111111
species
 1686310 
2281313513

12
species
11
 85701 


85701 
85701 

2112
species
1111
 1933912 


1933912 
1933912 
1933912 
1933912 


1933906 
1
 1933906 
species
1

 1933904 
species
11
11


1933904 

1933904 


33047 
33047 
11
species
 33047 
74


515256 


515256 
 515256 
11
species
11

 1933907 
species
11
41


1933907 

1933907 


1318743 
 1318743 
species
1
2


1933910 
 1933910 
1
species
1


38323 
38323 
38323 
38323 
21021
1111
species
 38323 

82115
family
434244454445
713339955112743658004414

323620
genus
111111
148211951498591


879274 
879274 
879274 
879274 
879274 
879274 
 879274 
species
111111
148211951498591

379
252526262626
genus
188520522717380128682289


2020311 
2020311 
2020311 
2020311 
2020311 
2020311 
 2020311 
species
111111
271846807528


1703968 
1703968 
1703968 
1703968 
1703968 
1703968 
221242473219
 1703968 
111111
species


424182 
424182 
424182 
424182 
424182 
424182 
 424182 
species
111111
107618251721


1301032 
1301032 
1301032 
1301032 
1301032 
1301032 
541657474324
 1301032 
species
111111


396 
396 
396 
396 
396 
396 
4926066931051848644
species
111111
 396 

species
111111
 1869170 
157029344426

1869170 
1869170 
1869170 
1869170 
1869170 
1869170 

 1571470 
111111
species
442962704344

1571470 
1571470 
1571470 
1571470 
1571470 
1571470 

652349723219
species
111111
 2048897 

2048897 
2048897 
2048897 
2048897 
2048897 
2048897 

1628
species
1111
 1703966 


1703966 
1703966 
1703966 
1703966 

273252602623
 2020313 
111111
species

2020313 
2020313 
2020313 
2020313 
2020313 
2020313 


2028343 
2028343 
2028343 
2028343 
2028343 
2028343 
111111
species
 2028343 
31923613042

 29449 
species
111111
263338394471403326

29449 
29449 
29449 
29449 
29449 
29449 

272569847049
species
111111
 1703969 

1703969 
1703969 
1703969 
1703969 
1703969 
1703969 


1703961 
1703961 
1703961 
1703961 
1703961 
1703961 
111111
species
 1703961 
113236


384 
384 
384 
384 
384 
384 
 384 
species
111111
5045437901046705662


1703967 
1703967 
1703967 
1703967 
1703967 
1703967 
species
111111
 1703967 
341610351712


1125847 
1125847 
1125847 
1125847 
1125847 
1125847 
111111
species
 1125847 
182431554428

species
111111
 1914541 
112053446912

1914541 
1914541 
1914541 
1914541 
1914541 
1914541 

332644414463
species
111111
 1703960 

1703960 
1703960 
1703960 
1703960 
1703960 
1703960 

111111
species
 1703964 
181325545235

1703964 
1703964 
1703964 
1703964 
1703964 
1703964 

10817312114
 1703965 
111111
species

1703965 
1703965 
1703965 
1703965 
1703965 
1703965 

27611231711
111111
species
 1703962 

1703962 
1703962 
1703962 
1703962 
1703962 
1703962 

 56730 
species
111111
4530691185090

56730 
56730 
56730 
56730 
56730 
56730 

111111
species
 1981173 
6070601268722

1981173 
1981173 
1981173 
1981173 
1981173 
1981173 

 2020312 
species
111111
282732697032

2020312 
2020312 
2020312 
2020312 
2020312 
2020312 

191437492429
 398 
111111
species

398 
398 
398 
398 
398 
398 

1525371
111111
genus
69508619414969

111111
species
 399 
69508619414969

399 
399 
399 
399 
399 
399 

666666
genus
357
3704296401501524318

species group
222222
1183400
202225261339359196


358 
358 
358 
358 
358 
358 
174175222269330176
 358 
111111
species


1176649 
1176649 
1176649 
1176649 
1176649 
1176649 
 1176649 
111111
species
285039702920


359 
359 
359 
359 
359 
359 
 359 
111111
species
34452742253944


861208 
861208 
861208 
861208 
861208 
861208 
222741573135
species
111111
 861208 

211546356227
 373 
111111
species

373 
373 
373 
373 
373 
373 


1842536 
1842536 
1842536 
1842536 
1842536 
1842536 
111111
species
 1842536 
14211453316

3861826
34019
genus
212323


1273132 


1273132 
 1273132 
11
species
24


34021 
34021 
34021 
34021 

34021 
185123
 34021 
11111
species

 556287 
111
species
212


556287 
556287 
556287 


34020 
1
 34020 
species
1


309868 


309868 
11
11
species
 309868 

124412871643256720381498
28105
666666
genus

 382 
species
111111
847876110617041385951

382 
382 
382 
382 
382 
382 


110321 
110321 
110321 
110321 
110321 
110321 
111111
species
 110321 
171434773114

111111
species
 194963 
778095175110107

194963 
194963 
194963 
194963 
194963 
194963 


380 
380 
380 
380 
380 
380 
228265276412433354
111111
species
 380 


794846 
794846 
794846 
794846 
794846 
794846 
 794846 
species
111111
331138852224

species
111111
 1842534 
4241941145748

1842534 
1842534 
1842534 
1842534 
1842534 
1842534 

8091164206134143
106591
222222
genus

species
111111
 716925 
365588877082

716925 
716925 
716925 
716925 
716925 
716925 


106592 
106592 
106592 
106592 
106592 
106592 
 106592 
111111
species
4436761196461

10776179218124129
2036754
family
222222

222222
genus
28209
10776179218124129


444444 
444444 
444444 
444444 
444444 
444444 
111111
species
 444444 
31876917830


1702325 
1702325 
1702325 
1702325 
1702325 
1702325 
76681031274699
111111
species
 1702325 

255475
family
444444
1651272521720349251

414371
111111
genus
5057102151811768

 1349819 
111111
species
5057102151811768

1349819 
1349819 
1349819 
1349819 
1349819 
1349819 

11570150202232183
293088
333333
genus

30427505125
 686597 
111111
species

686597 
686597 
686597 
686597 
686597 
686597 

4832619310188
 293089 
111111
species

293089 
293089 
293089 
293089 
293089 
293089 


1486262 
1486262 
1486262 
1486262 
1486262 
1486262 
373462598070
species
111111
 1486262 

41294
family
232323232323
224724043323445137202193

genus
141414141414
374
141514112004250621531260


376 
376 
376 
376 
376 
376 
 376 
species
111111
452473979157


2057741 
2057741 
2057741 
2057741 
2057741 
2057741 
1088613111815291
 2057741 
111111
species

 1223566 
111111
species
544481969637

1223566 
1223566 
1223566 
1223566 
1223566 
1223566 

20273901297359
111111
species
 114615 

114615 
114615 
114615 
114615 
114615 
114615 

species
111111
 931866 
62227813910793

931866 
931866 
931866 
931866 
931866 
931866 


115808 
115808 
115808 
115808 
115808 
115808 
4644861138441
species
111111
 115808 


1404367 
1404367 
1404367 
1404367 
1404367 
1404367 
673710012111777
 1404367 
species
111111


1404768 
1404768 
1404768 
1404768 
1404768 
1404768 
806614911414476
111111
species
 1404768 


288000 
288000 
288000 
288000 
288000 
288000 
42319414510880
111111
species
 288000 

 1355477 
species
111111
412487602694598333

1355477 
1355477 
1355477 
1355477 
1355477 
1355477 


375 
375 
375 
375 
375 
375 
129252256451288144
 375 
111111
species


1274631 
1274631 
1274631 
1274631 
1274631 
1274631 
457056754424
species
111111
 1274631 

5611299125137101
 44255 
species
111111

44255 
44255 
44255 
44255 
44255 
44255 

67631098911447
 335659 
species
111111

335659 
335659 
335659 
335659 
335659 
335659 

444444
genus
85413
182231364528356247

4852781459654
111111
species
 1526658 

1526658 
1526658 
1526658 
1526658 
1526658 
1526658 

species
111111
 1842539 
531231211538879

1842539 
1842539 
1842539 
1842539 
1842539 
1842539 

 2015316 
111111
species
3823931088947

2015316 
2015316 
2015316 
2015316 
2015316 
2015316 

 1792307 
111111
species
4333721228367

1792307 
1792307 
1792307 
1792307 
1792307 
1792307 

868910417418480
40136
111111
genus

868910417418480
species
111111
 40137 

40137 
40137 
40137 
40137 
40137 
40137 

172245544332
1649510
genus
111111


1333996 
1333996 
1333996 
1333996 
1333996 
1333996 
 1333996 
species
111111
172245544332

222222
genus
911
278461028248

111111
species
 912 
16424595231

912 
912 
912 
912 
912 
912 

 913 
species
111111
11422433017

913 
913 
913 
913 
913 
913 

111111
genus
1073
5206437601087902526


1076 
1076 
1076 
1076 
1076 
1076 
5206437601087902526
 1076 
111111
species

119045
191919191919
family
187520093129527633002232

116611911838313919221326
141414141414
genus
407

41117871517258
species
111111
 925818 

925818 
925818 
925818 
925818 
925818 
925818 

9518912421815282
 114616 
species
111111

114616 
114616 
114616 
114616 
114616 
114616 

712313218412281
species
111111
 2202825 

2202825 
2202825 
2202825 
2202825 
2202825 
2202825 

111111
species
 2202826 
82111132268164134

2202826 
2202826 
2202826 
2202826 
2202826 
2202826 


334852 
334852 
334852 
334852 
334852 
334852 
111111
species
 334852 
80499620619892


31998 
31998 
31998 
31998 
31998 
31998 
99145128243125108
species
111111
 31998 


270351 
270351 
270351 
270351 
270351 
270351 
 270351 
111111
species
909215129214791

9577175263169132
 1479019 
111111
species

1479019 
1479019 
1479019 
1479019 
1479019 
1479019 

 2067957 
111111
species
4621861596547

2067957 
2067957 
2067957 
2067957 
2067957 
2067957 

975712719715998
 2202828 
111111
species

2202828 
2202828 
2202828 
2202828 
2202828 
2202828 


426117 
426117 
426117 
426117 
426117 
426117 
711111151599576
species
111111
 426117 


418223 
418223 
418223 
418223 
418223 
418223 
6875179259150123
 418223 
111111
species


1826873 
1826873 
1826873 
1826873 
1826873 
1826873 
9475147275132114
111111
species
 1826873 

1374915926517290
111111
species
 2202827 

2202827 
2202827 
2202827 
2202827 
2202827 
2202827 

10311515220115668
genus
222222
186650

14542833311
 2082949 
species
111111

2082949 
2082949 
2082949 
2082949 
2082949 
2082949 

species
111111
 1882682 
8911011011812357

1882682 
1882682 
1882682 
1882682 
1882682 
1882682 

2282523
333333
genus
606703113919361222838


408 
408 
408 
408 
408 
408 
 408 
species
111111
4075357791349881555

153146286428263246
111111
species
 223967 

223967 
223967 
223967 
223967 
223967 
223967 


29429 
29429 
29429 
29429 
29429 
29429 
4622741597837
 29429 
species
111111

genus
111111
1734920
172150305521

species
111111
 1235591 
172150305521

1235591 
1235591 
1235591 
1235591 
1235591 
1235591 

181744454736
genus
222222
1484898


1384459 
1384459 
1384459 
1384459 
1384459 
1384459 
111226251511
111111
species
 1384459 

7518203225
 2170729 
111111
species

2170729 
2170729 
2170729 
2170729 
2170729 
2170729 

531344439734698312
45401
family
111111111111

81
genus
333333
16849621229457


717785 
717785 
717785 
717785 
717785 
717785 
61625223222
species
111111
 717785 

131813422119
species
111111
 1427356 

1427356 
1427356 
1427356 
1427356 
1427356 
1427356 


53399 
53399 
53399 
53399 
53399 
53399 
111111
species
 53399 
1491524584116

14916352616
1068
genus
111111


1069 
1069 
1069 
1069 
1069 
1069 
species
111111
 1069 
14916352616

333333
genus
46913
79808519818772


1643450 
1643450 
1643450 
1643450 
1643450 
1643450 
302725626824
 1643450 
111111
species

111111
species
 1736675 
294037779635

1736675 
1736675 
1736675 
1736675 
1736675 
1736675 


2083786 
2083786 
2083786 
2083786 
2083786 
2083786 
 2083786 
species
111111
201323592313

119044
genus
111111
121016124


1608628 
1608628 
1608628 
1608628 
1608628 
1608628 
121016124
 1608628 
111111
species

59282
genus
111111
210180193242243126

 1079 
species
111111
210180193242243126

1079 
1079 
1079 
1079 
1079 
1079 

1082930
genus
111111
1911930128

1911930128
species
111111
 531813 

531813 
531813 
531813 
531813 
531813 
531813 

4023549112429
29407
111111
genus


674703 
674703 
674703 
674703 
674703 
674703 
4023549112429
 674703 
111111
species

222222
family
119043
11433342779

7152028402
111111
genus
256616


256618 
256618 
256618 
256618 
256618 
256618 
species
111111
 256618 
7152028402

4281314377
111111
genus
444432


1922226 
1922226 
1922226 
1922226 
1922226 
1922226 
111111
species
 1922226 
4281314377

333333
family
335928
152124275352254216

111111
genus
152053
4524871617158

4524871617158
111111
species
 921 

921 
921 
921 
921 
921 
921 

6
111111
genus
706111410676106

706111410676106
 7 
111111
species

7 
7 
7 
7 
7 
7 

3739748510752
279
genus
111111


280 
280 
280 
280 
280 
280 
 280 
species
111111
3739748510752

83084496912605629783
118882
family
171315171716

8010879145428569
genus
444444
528

 571256 
111111
species
935132766

571256 
571256 
571256 
571256 
571256 
571256 


419475 
419475 
419475 
419475 
419475 
419475 
17625201825
 419475 
species
111111


271865 
271865 
271865 
271865 
271865 
271865 
111111
species
 271865 
122163537268


529 
529 
529 
529 
529 
529 
4297337710150
 529 
species
111111

75073689011151344714
genus
13911131312
234

648111716712158
111111
species
 29461 

29461 
29461 
29461 
29461 
29461 
29461 


1885919 
1885919 
1885919 
1885919 
1885919 
1885919 
791621272
species
111111
 1885919 


236 


236 
236 
236 
1961
 236 
species
1111


29459 
29459 
29459 
29459 
29459 
29459 
111111
species
 29459 
506526553683993486

species
111111
 981386 
224711

981386 
981386 
981386 
981386 
981386 
981386 


120577 

120577 
120577 
120577 
120577 
11431
 120577 
species
11111


1149952 

1149952 
1149952 
1149952 
1149952 
321143
 1149952 
11111
species


1844051 

1844051 
1844051 
1844051 
1844051 
62542
species
11111
 1844051 

species
111111
 444163 
5828912

444163 
444163 
444163 
444163 
444163 
444163 


36855 
36855 
36855 
36855 
36855 
36855 
333371363948
 36855 
111111
species

111111
species
 235 
1127311415312594

235 
235 
235 
235 
235 
235 

6287116
111111
species
 120576 

120576 
120576 
120576 
120576 
120576 
120576 


1891098 
1891098 

1891098 
1891098 
4241
1111
species
 1891098 

62237416612952
family
333333
31993

222222
genus
133
181043977135


187303 
187303 
187303 
187303 
187303 
187303 
12415384513
 187303 
111111
species


655015 
655015 
655015 
655015 
655015 
655015 
 655015 
111111
species
6628592622

441331695817
111111
genus
425

441331695817
species
111111
 426 

426 
426 
426 
426 
426 
426 

family
222222
45404
618169936056

263147573842
111111
genus
120652

 199596 
species
111111
263147573842

199596 
199596 
199596 
199596 
199596 
199596 

355022362214
genus
111111
532

355022362214
 533 
111111
species

533 
533 
533 
533 
533 
533 

141414141414
family
69277
68480494715941247673

8221233275
449972
genus
111111

111111
species
 266779 
8221233275

266779 
266779 
266779 
266779 
266779 
266779 

4575406761133829434
68287
888888
genus

 2066070 
species
111111
2923771206841

2066070 
2066070 
2066070 
2066070 
2066070 
2066070 


381 
381 
381 
381 
381 
381 
 381 
111111
species
2741791415945

4358401329155
 1670800 
species
111111

1670800 
1670800 
1670800 
1670800 
1670800 
1670800 

 39645 
species
111111
146187200295270125

39645 
39645 
39645 
39645 
39645 
39645 

 71433 
111111
species
5334651286846

71433 
71433 
71433 
71433 
71433 
71433 


536018 
536018 
536018 
536018 
536018 
536018 
 536018 
111111
species
4748808511648


593909 
593909 
593909 
593909 
593909 
593909 
55748011410235
111111
species
 593909 


278153 
278153 
278153 
278153 
278153 
278153 
5775551185539
 278153 
species
111111

111111
genus
245876
354526161526

111111
species
 472175 
354526161526

472175 
472175 
472175 
472175 
472175 
472175 

7415261410
28100
111111
genus

111111
species
 1867719 
7415261410

1867719 
1867719 
1867719 
1867719 
1867719 
1867719 

274591
genus
111111
9114312211


1620421 
1620421 
1620421 
1620421 
1620421 
1620421 
9114312211
species
111111
 1620421 

168192204355340187
222222
genus
31988

791506511311771
 83263 
species
111111

83263 
83263 
83263 
83263 
83263 
83263 


374606 
374606 
374606 
374606 
374606 
374606 
8942139242223116
 374606 
111111
species

111111
genus
1572860
191566908230


1482074 
1482074 
1482074 
1482074 
1482074 
1482074 
species
111111
 1482074 
191566908230

204441
444141434345
order
232422252579316129351800

433
232121232324
family
942850103211961171817

655010714214680
522
genus
222222

111111
species
 524 
403047925130

524 
524 
524 
524 
524 
524 


62140 
62140 
62140 
62140 
62140 
62140 
species
111111
 62140 
252060509550

3360533324
genus
111111
320496


320497 
320497 
320497 
320497 
320497 
320497 
3360533324
species
111111
 320497 

11831
11111
genus
1602345

species
11111
 1510841 
11831

1510841 
1510841 

1510841 
1510841 
1510841 

2308715613211281
441
222222
genus


318683 
318683 
318683 
318683 
318683 
318683 
143336221936
111111
species
 318683 


442 
442 
442 
442 
442 
442 
216541201109345
111111
species
 442 

3710767
111111
genus
91914


91915 
91915 
91915 
91915 
91915 
91915 
3710767
111111
species
 91915 

434
756778
genus
98117961488582


65959 
1
 65959 
species
1


438 
438 
438 
438 
438 
438 
563635714331
species
111111
 438 


481146 
481146 
481146 
481146 
481146 
481146 
 481146 
species
111111
8152518523

11722
 1076596 
11111
species

1076596 

1076596 
1076596 
1076596 
1076596 

 446692 
111111
species
141861472

446692 
446692 
446692 
446692 
446692 
446692 


1633874 


1633874 
1633874 
1633874 
1451
 1633874 
species
1111


104102 
104102 
104102 
104102 
104102 
104102 
1139717114
111111
species
 104102 

7922171218
151157
111111
subgenus


435 
435 
435 
435 
435 
435 
species
111111
 435 
7922171218

111111
genus
89583
10610512015311461


33996 
33996 
33996 
33996 
33996 
33996 
 33996 
111111
species
10610512015311461

genus
111111
364409
153148235205192195


364410 
364410 
364410 
364410 
364410 
364410 
153148235205192195
species
111111
 364410 

genus
222222
125216
148134173205277139

 2018065 
species
111111
84818110611859

2018065 
2018065 
2018065 
2018065 
2018065 
2018065 

 257708 
111111
species
6453929915980

257708 
257708 
257708 
257708 
257708 
257708 

1434011
genus
444444
8912194130170130

species
111111
 28448 
437933524658

28448 
28448 
28448 
28448 
28448 
28448 

111111
species
 1177712 
262031262927

1177712 
1177712 
1177712 
1177712 
1177712 
1177712 

 265960 
111111
species
152526425

265960 
265960 
265960 
265960 
265960 
265960 

 33995 
species
111111
52025265340

33995 
33995 
33995 
33995 
33995 
33995 

162036333437
genus
111111
153497


153496 
153496 
153496 
153496 
153496 
153496 
162036333437
species
111111
 153496 

41295
212020202021
family
13821375154719651764983

15322443016
1612157
111111
genus

15322443016
111111
species
 1084 

1084 
1084 
1084 
1084 
1084 
1084 

666666
genus
191
70379384210901029601


192 
192 
192 
192 
192 
192 
 192 
species
111111
13491203280202148

218302218243245165
111111
species
 193 

193 
193 
193 
193 
193 
193 


664962 
664962 
664962 
664962 
664962 
664962 
675411612411982
 664962 
species
111111

 2202148 
species
111111
48479312111667

2202148 
2202148 
2202148 
2202148 
2202148 
2202148 


682998 
682998 
682998 
682998 
682998 
682998 
931386012112340
species
111111
 682998 


528244 
528244 
528244 
528244 
528244 
528244 
14316115220122499
111111
species
 528244 

222222
genus
168934
13181426268


2048283 
2048283 
2048283 
2048283 
2048283 
2048283 
 2048283 
species
111111
585634


220697 
220697 
220697 
220697 
220697 
220697 
111111
species
 220697 
810920234

10393791245463
171436
genus
111111


171437 
171437 
171437 
171437 
171437 
171437 
10393791245463
111111
species
 171437 

262833787813
1543704
111111
genus


1612173 
1612173 
1612173 
1612173 
1612173 
1612173 
111111
species
 1612173 
262833787813

genus
111111
1804663
2531421


1549855 
1549855 
1549855 
1549855 
1549855 
1549855 
2531421
111111
species
 1549855 

1263978
11
genus
11

11
 1263979 
species
11

1263979 


1263979 

1543705
111111
genus
11715381917545

species
111111
 28077 
11715381917545

28077 
28077 
28077 
28077 
28077 
28077 

75411245
1182780
111111
genus

species
111111
 1288970 
75411245

1288970 
1288970 
1288970 
1288970 
1288970 
1288970 

genus
444444
13134
206150279292250115


1639348 
1639348 
1639348 
1639348 
1639348 
1639348 
514447646025
 1639348 
111111
species

551877704828
111111
species
 1663591 

1663591 
1663591 
1663591 
1663591 
1663591 
1663591 


84159 
84159 
84159 
84159 
84159 
84159 
282042726819
 84159 
111111
species


55518 
55518 
55518 
55518 
55518 
55518 
7268113867443
111111
species
 55518 

18979192205196115
1081
genus
222222


1085 
1085 
1085 
1085 
1085 
1085 
 1085 
111111
species
119449213010770

species
111111
 34018 
7035100758945

34018 
34018 
34018 
34018 
34018 
34018 

325175865438
genus
111111
991903

325175865438
 991904 
111111
species

991904 
991904 
991904 
991904 
991904 
991904 

order
3211
54526
75211

1655514
family
3211
75211

species
111
 744985 
111

744985 
744985 


744985 

7411
211
genus
198251


1002672 
1002672 
species
11
 1002672 
361


198252 


198252 
381
 198252 
11
species

121327131430
111111
order
255473

255474
family
111111
121327131430

121327131430
208215
genus
111111


208216 
208216 
208216 
208216 
208216 
208216 
121327131430
species
111111
 208216 

124151765
213485
111111
genus

124151765
 349221 
111111
species

349221 
349221 
349221 
349221 
349221 
349221 

2291911
767891
genus
111111


767892 
767892 
767892 
767892 
767892 
767892 
2291911
111111
species
 767892 

151515151515
order
204458
836870113215961628706

family
151515151515
76892
836870113215961628706

272068431828
76890
genus
111111

272068431828
 78587 
species
111111

78587 
78587 
78587 
78587 
78587 
78587 

genus
666666
41275
184220205480508186

173925708227
 588932 
species
111111

588932 
588932 
588932 
588932 
588932 
588932 


41276 
41276 
41276 
41276 
41276 
41276 
531126908724
 41276 
species
111111


74313 
74313 
74313 
74313 
74313 
74313 
211536835239
species
111111
 74313 

235139858426
 1938605 
111111
species

1938605 
1938605 
1938605 
1938605 
1938605 
1938605 


1827469 
1827469 
1827469 
1827469 
1827469 
1827469 
538139858749
 1827469 
species
111111


1532555 
1532555 
1532555 
1532555 
1532555 
1532555 
 1532555 
111111
species
1723406711621

543523706910958447
75
genus
555555

species
111111
 69666 
3221531028636

69666 
69666 
69666 
69666 
69666 
69666 

238128306385417175
 155892 
111111
species

155892 
155892 
155892 
155892 
155892 
155892 


366602 
366602 
366602 
366602 
366602 
366602 
75608912311973
species
111111
 366602 


88688 
88688 
88688 
88688 
88688 
88688 
162131214216254147
species
111111
 88688 


69395 
69395 
69395 
69395 
69395 
69395 
 69395 
111111
species
3618344848216

646613214312839
20
222222
genus

 284016 
111111
species
454410110710126

284016 
284016 
284016 
284016 
284016 
284016 


2201350 
2201350 
2201350 
2201350 
2201350 
2201350 
 2201350 
species
111111
192231362713


1759059 
1759059 
1759059 
1759059 
1759059 
1759059 
18412120166
 1759059 
111111
species

219122382777407839032013
order
696770706969
204455

88667315221436
69657
444444
family

215234
genus
111111
2723

215234
 2724 
species
111111

2724 
2724 
2724 
2724 
2724 
2724 

6358499213418
74317
genus
111111

6358499213418
111111
species
 74318 

74318 
74318 
74318 
74318 
74318 
74318 

85
genus
222222
23719587714


1906738 
1906738 
1906738 
1906738 
1906738 
1906738 
 1906738 
species
111111
142829292


81032 
81032 
81032 
81032 
81032 
81032 
9511294812
 81032 
species
111111

31989
656366666565
family
210321722704392636891977

402437429543444338
444444
genus
302485

 221822 
species
111111
257344286369312251

221822 
221822 
221822 
221822 
221822 
221822 


1844006 
1844006 
1844006 
1844006 
1844006 
1844006 
223334
111111
species
 1844006 


60890 
60890 
60890 
60890 
60890 
60890 
90598910910133
 60890 
species
111111

533251622850
111111
species
 1580596 

1580596 
1580596 
1580596 
1580596 
1580596 
1580596 

genus
111111
227873
364643747030


121719 
121719 
121719 
121719 
121719 
121719 
species
111111
 121719 
364643747030

genus
111111
1649279
51839167


379347 
379347 
379347 
379347 
379347 
379347 
 379347 
species
111111
51839167

11171998
11111
genus
119541


441209 

441209 
441209 
441209 
441209 
11111
species
 441209 
11171998

221015353228
genus
222222
2433

111111
species
 2434 
11614303027

2434 
2434 
2434 
2434 
2434 
2434 


42443 
42443 
42443 
42443 
42443 
42443 
1141521
species
111111
 42443 

111111
genus
436357
2226854012059


2109625 
2109625 
2109625 
2109625 
2109625 
2109625 
 2109625 
species
111111
2226854012059


2033435 
2033435 
2033435 
2033435 
2033435 
2033435 
 2033435 
species
111111
3413283921

97050
222221
genus
232538513511

41911165
11111
species
 292414 

292414 
292414 
292414 
292414 
292414 

 89184 
111111
species
19627353011

89184 
89184 
89184 
89184 
89184 
89184 

74032
111111
genus
618824177

species
111111
 74033 
618824177

74033 
74033 
74033 
74033 
74033 
74033 


1904441 
1904441 
1904441 
1904441 
1904441 
1904441 
111111
species
 1904441 
6210595

111111
genus
367771
20939617428

species
111111
 42444 
20939617428

42444 
42444 
42444 
42444 
42444 
42444 

genus
222222
354203
905611319111664

 311180 
111111
species
393851826728

311180 
311180 
311180 
311180 
311180 
311180 


1792508 
1792508 
1792508 
1792508 
1792508 
1792508 
5118621094936
species
111111
 1792508 

685131
1284657
genus
111111


1284658 
1284658 
1284658 
1284658 
1284658 
1284658 
 1284658 
species
111111
685131

686670615691
58842
genus
111111


2009329 
2009329 
2009329 
2009329 
2009329 
2009329 
686670615691
111111
species
 2009329 

299261
111111
genus
201731253753


299262 
299262 
299262 
299262 
299262 
299262 
 299262 
species
111111
201731253753

474147463112
genus
111111
191028

species
111111
 133924 
474147463112

133924 
133924 
133924 
133924 
133924 
133924 

106110151212
111111
genus
2211641

 245188 
species
111111
106110151212

245188 
245188 
245188 
245188 
245188 
245188 

111727443819
genus
111111
360528

 1250539 
species
111111
111727443819

1250539 
1250539 
1250539 
1250539 
1250539 
1250539 

265
genus
999999
327401394804815237

93938518
species
111111
 1529068 

1529068 
1529068 
1529068 
1529068 
1529068 
1529068 

11211410929136466
 147645 
111111
species

147645 
147645 
147645 
147645 
147645 
147645 

302246998827
111111
species
 34004 

34004 
34004 
34004 
34004 
34004 
34004 


1945662 
1945662 
1945662 
1945662 
1945662 
1945662 
343717724022
111111
species
 1945662 

111111
species
 1077935 
213545777337

1077935 
1077935 
1077935 
1077935 
1077935 
1077935 


1499308 
1499308 
1499308 
1499308 
1499308 
1499308 
111111
species
 1499308 
305127695517


2065379 
2065379 
2065379 
2065379 
2065379 
2065379 
species
111111
 2065379 
292262344216


34003 
34003 
34003 
34003 
34003 
34003 
species
111111
 34003 
111636324427


266 
266 
266 
266 
266 
266 
5110143925817
111111
species
 266 

752328916
111111
genus
188905


290400 
290400 
290400 
290400 
290400 
290400 
752328916
species
111111
 290400 

19315322910
204456
111111
genus

 2169400 
species
111111
19315322910

2169400 
2169400 
2169400 
2169400 
2169400 
2169400 

323550517842
60136
212222
genus

species
111111
 1917485 
203523375836

1917485 
1917485 
1917485 
1917485 
1917485 
1917485 

122714206
 1402135 
11111
species

1402135 

1402135 
1402135 
1402135 
1402135 

8710256928835
111111
genus
263377

8710256928835
111111
species
 1229727 

1229727 
1229727 
1229727 
1229727 
1229727 
1229727 

333333
genus
34008
12493163282316176


35806 
35806 
35806 
35806 
35806 
35806 
654590172208116
111111
species
 35806 


308754 
308754 
308754 
308754 
308754 
308754 
472048665231
 308754 
111111
species

122825445629
111111
species
 1564506 

1564506 
1564506 
1564506 
1564506 
1564506 
1564506 

313671
1759396
111111
genus


1920883 
1920883 
1920883 
1920883 
1920883 
1920883 
species
111111
 1920883 
313671

52573210
1579315
genus
111111

 1579316 
species
111111
52573210

1579316 
1579316 
1579316 
1579316 
1579316 
1579316 

1609958
111111
genus
201231244525


1609966 
1609966 
1609966 
1609966 
1609966 
1609966 
201231244525
 1609966 
111111
species

285107
111111
genus
242063484143


1915078 
1915078 
1915078 
1915078 
1915078 
1915078 
 1915078 
111111
species
242063484143

6102327154
genus
111111
159345


159346 
159346 
159346 
159346 
159346 
159346 
 159346 
species
111111
6102327154

genus
223323
53945
644232411

1232
 1458307 
species
1111

1458307 

1458307 
1458307 

1458307 

53111167
 1217908 
species
111111

1217908 
1217908 
1217908 
1217908 
1217908 
1217908 


53946 
53946 
53946 
53946 
53946 
11982
species
11111
 53946 

169132142200206126
92944
222222
genus


92947 
92947 
92947 
92947 
92947 
92947 
111111
species
 92947 
221933234919

147113109177157107
 92945 
111111
species

92945 
92945 
92945 
92945 
92945 
92945 

genus
333333
478070
4633881228939


2021862 
2021862 
2021862 
2021862 
2021862 
2021862 
211536374712
111111
species
 2021862 


187304 
187304 
187304 
187304 
187304 
187304 
 187304 
species
111111
201144532714

578321513
species
111111
 1674922 

1674922 
1674922 
1674922 
1674922 
1674922 
1674922 

252318436643597283
genus
444444
1060


1063 
1063 
1063 
1063 
1063 
1063 
188255300453432198
species
111111
 1063 


1850250 
1850250 
1850250 
1850250 
1850250 
1850250 
341756589328
 1850250 
111111
species

81233482112
 1061 
111111
species

1061 
1061 
1061 
1061 
1061 
1061 

223447845145
 2033869 
species
111111

2033869 
2033869 
2033869 
2033869 
2033869 
2033869 

875170
genus
444444
53107911079170


1758178 
1758178 
1758178 
1758178 
1758178 
1758178 
 1758178 
species
111111
192531182134


1411902 
1411902 
1411902 
1411902 
1411902 
1411902 
11151228139
 1411902 
species
111111


1208324 
1208324 
1208324 
1208324 
1208324 
1208324 
176546565424
 1208324 
species
111111


1397108 
1397108 
1397108 
1397108 
1397108 
1397108 
111111
species
 1397108 
622533

18112837158
74030
genus
222222


215743 
215743 
215743 
215743 
215743 
215743 
159192376
 215743 
species
111111

3291482
species
111111
 391613 

391613 
391613 
391613 
391613 
391613 
391613 

373251644433
1097466
genus
111111


1335048 
1335048 
1335048 
1335048 
1335048 
1335048 
373251644433
 1335048 
111111
species

13428312014
309512
111111
genus

 215813 
species
111111
13428312014

215813 
215813 
215813 
215813 
215813 
215813 

5717631298832
genus
111111
1632780


1868589 
1868589 
1868589 
1868589 
1868589 
1868589 
 1868589 
species
111111
5717631298832

23914771213326
phylum
1259948
32066

class
1259948
203490
23914771213326

order
1259948
203491
23914771213326

210733571412
1129771
family
614524

2
32068
genus
1


826 
2
species
1
 826 

69732561411
313423
genus
32067

111
species
 712357 
131


712357 
712357 
712357 

11201
 712368 
1111
species

712368 

712368 
712368 

712368 

573021119
 40542 
111111
species

40542 
40542 
40542 
40542 
40542 
40542 


1785996 

1785996 
1785996 
 1785996 
species
111
63114

1351
11
genus
34104

1351
 34105 
11
species

34105 


34105 

168808
genus
111
411


187101 

187101 


187101 
 187101 
species
111
411

family
645424
203492
29744641914

1676461914
848
544324
genus


861 


861 
11
species
11
 861 

 849 
11
species
11

849 
849 


850 


850 
12
 850 
11
species


1583098 
1583098 
1583098 
1583098 
2211
species
1111
 1583098 


860 
860 
860 
860 
species
1111
 860 
18137

species
11
 856 
11


856 
856 

11333764
111111
species
 851 

851 
851 
851 
851 
851 
851 

genus
111
167639
133818


167642 

167642 
167642 
133818
 167642 
111
species

200783
72533
phylum
38041694

class
72533
187857
38041694

order
1111
1485951
7321

7321
558314
family
1111

7321
171868
1111
genus

 228745 
species
1111
7321

228745 

228745 
228745 
228745 

37311484
order
61423
32069

family
2122
224027
308183

112
genus
212790
28473


309806 
1
species
1
 309806 


436114 


436114 

436114 
28472
species
111
 436114 

111
genus
182899
2411


309805 

309805 
309805 
2411
111
species
 309805 

3221
family
64898
53681

53
168657
3
genus

23
1
species
 380749 

380749 


547145 
species
1
 547145 
22


547146 
 547146 
1
species
8

genus
11
939
27


940 
940 
 940 
species
11
27

111
genus
75905
411


136094 
136094 
136094 
species
111
 136094 
411

12
1
genus
412592


412593 
12
species
1
 412593 

675979736569
phylum
1117
1245312456411383456

11
1890505
order
11

11
11
family
1890528

11
54298
11
genus


54299 

54299 
11
species
 54299 
11

species
111111
 718217 
612385982

718217 
718217 
718217 
718217 
718217 
718217 

221924221822
order
1161
1376287907492


1940762 
1940762 

1940762 
1940762 
 1940762 
1111
species
5112

12211
11111
family
1892259

genus
11111
159191
12211


70799 
70799 
70799 
70799 
70799 
12211
 70799 
11111
species

1185
family
768878
433439332132

4166116
373984
11111
genus


373994 
373994 
373994 
373994 

373994 
 373994 
species
11111
4166116

657777
genus
1186
391833222126

111111
species
 32054 
252325

32054 
32054 
32054 
32054 
32054 
32054 

 1337936 
111111
species
1551122

1337936 
1337936 
1337936 
1337936 
1337936 
1337936 

33361
species
11111
 1954171 


1954171 
1954171 
1954171 
1954171 
1954171 


2005461 
2005461 
2005461 
2005461 
2005461 
2005461 
111111
species
 2005461 
222517

310376
 99598 
11111
species

99598 

99598 
99598 
99598 
99598 

species
111111
 2005469 
2313523

2005469 
2005469 
2005469 
2005469 
2005469 
2005469 


2005462 

2005462 
2005462 
2005462 
2005462 
152212
 2005462 
11111
species

2211
1892263
family
1111

genus
1111
1190
2211

1111
species
 1752063 
2211

1752063 

1752063 
1752063 

1752063 

922243545156
1162
14111312911
family

30211968
312213
genus
1163


1165 


1165 
11
 1165 
11
species


46234 
46234 
46234 
46234 
46234 
46234 
 46234 
species
111111
2629563

3244
 1647413 
species
1111

1647413 

1647413 
1647413 

1647413 

581824444448
999978
genus
1177

111226
 1618022 
111111
species

1618022 
1618022 
1618022 
1618022 
1618022 
1618022 


1261031 
1261031 
1261031 
1261031 
1261031 
1261031 
111111
species
 1261031 
315219


1869241 
1869241 
1869241 
1869241 

1869241 
21145
species
11111
 1869241 

22141134
111111
species
 1306274 

1306274 
1306274 
1306274 
1306274 
1306274 
1306274 


272131 
272131 
272131 
272131 
272131 
272131 
32442312
 272131 
species
111111


28072 
28072 
28072 
28072 
28072 
28072 
222354
 28072 
111111
species

1811114
111111
species
 317936 

317936 
317936 
317936 
317936 
317936 
317936 

 1751286 
species
111111
472194

1751286 
1751286 
1751286 
1751286 
1751286 
1751286 

species
1111
 103690 
32416

103690 
103690 
103690 
103690 

21211
genus
264688
42811


1164 
1164 
1164 
1164 
1164 
 1164 
11111
species
12411

 264691 
species
11
34

264691 

264691 

1301283
subclass
121319171516
587418911068131

262764553346
338654
order
1118

113212
family
1890464
2382315

112111
genus
669357
2371310


1617448 
1617448 
1617448 
1617448 
1617448 
1617448 
111111
species
 1617448 
2351310

2
species
1
 1615909 


1615909 

5
1
genus
268175

5
1
species
 2005460 


2005460 

102231
genus
11
11


1173026 
1173026 
 1173026 
species
11
11

1433
1890450
family
1212

131
genus
111
76023

 65093 
species
111
131


65093 
65093 
65093 

1453359
111
genus
132

132
 713887 
species
111


713887 
713887 

713887 

1890449
112222
family
232351342731

1125
112222
genus
232351342731


1126 
1126 
1126 
1126 
1126 
1126 
111111
species
 1126 
232350312630

 1967666 
species
1111
1311


1967666 
1967666 
1967666 
1967666 

family
111
1890452
1116

1116
102234
genus
111

 379064 
species
111
1116

379064 

379064 
379064 

order
91011111012
1150
3247125553585

71028151110
323333
family
1892252

5521865
111111
genus
44471


1173027 
1173027 
1173027 
1173027 
1173027 
1173027 
5521865
 1173027 
species
111111

11212
1205
genus
11111


1206 

1206 
1206 
1206 
1206 
 1206 
species
11111
11212

156543
genus
111111
35823

156543
 2153484 
111111
species

2153484 
2153484 
2153484 
2153484 
2153484 
2153484 

131
family
111
1892255

241421
111
genus
131


241425 
241425 
241425 
 241425 
111
species
131

244324
family
1892249
273115620

43988
244324
genus
273115620

 395961 
species
111111
1321939

395961 
395961 
395961 
395961 
395961 
395961 


41431 
41431 
41431 
41431 
41431 
41431 
118338
 41431 
111111
species

212
 65393 
111
species


65393 
65393 


65393 


497965 
497965 
497965 

497965 
1131
 497965 
species
1111

59187327
family
111111
1892251

59187327
63132
111111
genus

59187327
 1173025 
species
111111

1173025 
1173025 
1173025 
1173025 
1173025 
1173025 

family
333333
1892254
182148171227

1158
genus
222222
15164312918

 482564 
111111
species
111333976

482564 
482564 
482564 
482564 
482564 
482564 

43103212
species
111111
 118323 

118323 
118323 
118323 
118323 
118323 
118323 

355539
111111
genus
1155738


1155739 
1155739 
1155739 
1155739 
1155739 
1155739 
 1155739 
111111
species
355539

1001136139174145128
1890424
order
272331292826

3413437
1890436
111111
family

3413437
1152
genus
111111


82654 
82654 
82654 
82654 
82654 
82654 
3413437
 82654 
species
111111

99197119
family
334443
1890438

genus
223333
47251
5818599

 1080068 
11111
species
710354


1080068 
1080068 
1080068 
1080068 
1080068 

11
11
species
 1752064 


1752064 

1752064 


1184 

1184 
1184 
1184 
species
1111
 1184 
4312

 111781 
11111
species
11524

111781 
111781 
111781 

111781 
111781 

11111
genus
170610
41122

11111
species
 1209493 
41122

1209493 
1209493 
1209493 
1209493 
1209493 

111
family
1890429
111

155977
genus
111
111

111
 155978 
species
111

155978 


155978 

155978 

941078913511190
family
191723202119
1890426

17826305426
genus
222222
167375

32420165
 59930 
species
111111

59930 
59930 
59930 
59930 
59930 
59930 


1851505 
1851505 
1851505 
1851505 
1851505 
1851505 
species
111111
 1851505 
14622103821

314541
13034
genus
111111


292566 
292566 
292566 
292566 
292566 
292566 
111111
species
 292566 
314541

1129
151319171715
genus
7397581005260

111111
species
 110662 
2161158

110662 
110662 
110662 
110662 
110662 
110662 


374981 
374981 
374981 
374981 
374981 
374981 
species
111111
 374981 
8614112

 32049 
species
1111
1114


32049 
32049 
32049 

32049 

21111
 1173263 
species
11111

1173263 
1173263 
1173263 
1173263 
1173263 

 33070 
11
species
11


33070 

33070 


195498 
1
species
 195498 
1


585425 
585425 
585425 
585425 
585425 
585425 
253573
111111
species
 585425 


29410 
29410 
29410 
29410 
29410 
29410 
 29410 
111111
species
11101076


1350461 
 1350461 
species
1
2

 374982 
1
species
1

374982 


195253 
195253 

195253 
195253 
5432
 195253 
1111
species


195248 
195248 
195248 
211
species
111
 195248 


321327 

321327 
321327 
321327 
321327 
 321327 
species
11111
12612

species
11
 84588 
104


84588 

84588 


1916956 
1916956 
1916956 
1916956 
1916956 
11331
 1916956 
species
11111


64471 
64471 
64471 
64471 
 64471 
1111
species
81311


316279 

316279 
316279 
233
111
species
 316279 


32051 
32051 
32051 
32051 
32051 
32051 
111111
species
 32051 
345461045

4261026
 32046 
species
111111

32046 
32046 
32046 
32046 
32046 
32046 

322
species
111
 1280380 


1280380 
1280380 
1280380 


321332 

321332 
321332 
321332 
 321332 
species
1111
47145

1211
1111
species
 316278 

316278 
316278 
316278 
316278 

111
 166314 
111
species


166314 

166314 
166314 

252951
 585423 
species
111111

585423 
585423 
585423 
585423 
585423 
585423 

genus
11111
146785
11113

11111
species
 146786 
11113

146786 
146786 
146786 

146786 
146786 

5771446
1213
112211
family

1218
genus
112211
5771446

5751246
111111
species
 1219 

1219 
1219 
1219 
1219 
1219 
1219 

 1501268 
species
1
2


1501268 


1501269 
1
species
 1501269 
2

889911131615
family
211111
1890428

genus
211111
1142
889911131615

2
species
1
 1147 

1147 

887911131615
species
111111
 1148 

1148 
1148 
1148 
1148 
1148 
1148 

392736233020
307596
class
222222

307595
order
222222
392736233020

1890422
family
222222
392736233020

222222
genus
33071
392736233020


1416614 
1416614 
1416614 
1416614 
1416614 
1416614 
29191311156
111111
species
 1416614 

 33072 
111111
species
10823121514

33072 
33072 
33072 
33072 
33072 
33072 

211212
order
52604
311673

family
1121
1890498
1161

1161
102115
genus
1121


1807358 
species
1
 1807358 
2


102116 
102116 

102116 

102116 
1141
species
1111
 102116 

2172
1111
family
1890500

2172
genus
1111
44474

1111
species
 54308 
2172

54308 

54308 

54308 
54308 

142182
phylum
333333
84249912610345

class
333333
219685
84249912610345

order
333333
219686
84249912610345

family
333333
219687
84249912610345

1706036
genus
111111
601249665232


861299 
861299 
861299 
861299 
861299 
861299 
111111
species
 861299 
601249665232

222222
genus
173479
241250605113


173480 
173480 
173480 
173480 
173480 
173480 
111111
species
 173480 
22103347126


1379270 
1379270 
1379270 
1379270 
1379270 
1379270 
species
111111
 1379270 
221713397

18776732406628599631
453331323432
phylum
544448


1911684 


1911684 
51
species
11
 1911684 

31969
433331313432
class
18762732406627599631

24932565
186329
522222
order

24932565
2146
family
522222

32222
genus
33926
53265

85630
1111
species group
2121

 37692 
species
1111
2121

37692 
37692 


37692 
37692 

species group
1
85632
1

1
 59748 
1
species

59748 

22244
85620
11211
species group

22144
11111
species
 229545 

229545 
229545 
229545 

229545 
229545 


100379 
1
species
1
 100379 

22
genus
2147
2445


61635 
 61635 
species
1
242


35623 
1
 35623 
species
1

species
11
 2148 
24

2148 


2148 

17824690385572547594
2085
252325212323
order

17824690385572547594
2092
family
252325212323

2093
242124192121
genus
17796687384568544590

121111
11111
species
 2115 

2115 
2115 
2115 

2115 
2115 

 2104 
111111
species
9423224

2104 
2104 
2104 
2104 
2104 
2104 


2128 
2128 
2128 
2128 
2128 
2128 
7819657896386
species
111111
 2128 


45362 
45362 
45362 
45362 
45362 
45362 
318151367
species
111111
 45362 


171632 
171632 
171632 
171632 
171632 
171632 
 171632 
species
111111
4003214253325

2
 29553 
1
species


29553 


2109 

2109 
2109 
111
species
 2109 
111

21241
 171284 
11111
species

171284 
171284 
171284 
171284 
171284 


2110 

2110 
11
species
 2110 
125

 1749074 
species
1
4

1749074 

344621536858
111111
species
 171279 

171279 
171279 
171279 
171279 
171279 
171279 

species
111111
 2107 
4740324310276

2107 
2107 
2107 
2107 
2107 
2107 

182537173027
 2096 
species
111111

2096 
2096 
2096 
2096 
2096 
2096 


2111 
 2111 
species
1
1

 2100 
species
111111
15914141412

2100 
2100 
2100 
2100 
2100 
2100 


29501 
29501 
29501 
29501 

29501 
882231
species
11111
 29501 

354511
 2113 
species
111111

2113 
2113 
2113 
2113 
2113 
2113 


92401 

92401 
92401 

92401 
5322
1111
species
 92401 


57372 
57372 
57372 
57372 
57372 
57372 
 57372 
species
111111
61421075

1131
 2112 
1111
species

2112 

2112 

2112 
2112 


2099 
2099 
2099 
2099 
2099 
2099 
species
111111
 2099 
9210760101134100


86660 


86660 
 86660 
11
species
12


2123 


2123 
 2123 
11
species
11

121
 2097 
111
species


2097 

2097 
2097 

 28903 
111111
species
1311689014261159

28903 
28903 
28903 
28903 
28903 
28903 

1676291612107
656088
species group
312121

 2095 
111
species
114

2095 

2095 

2095 

1
 2105 
1
species

2105 


2102 
2102 
2102 
2102 
2102 
2102 
167609151267
 2102 
111111
species


2098 
2098 

2098 
211
species
111
 2098 


92400 
92400 
92400 
92400 
92400 
92400 
species
111111
 92400 
566735


29554 
 29554 
1
species
5

2831434
121222
genus
2129


2130 
2130 
2130 
2130 
2130 
 2130 
species
11111
11111


134821 
134821 

134821 
134821 
134821 
282323
 134821 
species
11111

186328
1384897
order
6893919504632

2131
family
973686
883815474431

883815474431
973686
genus
2132


2144 
2144 


2144 
311
111
species
 2144 


2145 
 2145 
species
1
1

111
species
 2136 
1629


2136 

2136 
2136 

111111
species
 216946 
12131361722

216946 
216946 
216946 
216946 
216946 
216946 


216942 
1
 216942 
1
species

 216934 
11
species
11


216934 


216934 

 47834 
species
11
21

47834 


47834 


315358 
4
 315358 
species
1

11
species
 216933 
11


216933 


216933 


216935 
216935 

216935 
216935 
62251
1111
species
 216935 


2137 
1
 2137 
species
1


216936 
216936 
216936 
216936 
216936 
216936 
7313115
111111
species
 216936 

522831
species
11111
 362837 

362837 
362837 

362837 
362837 
362837 


216937 
216937 
31
species
11
 216937 

60114321
33925
family
411211

genus
411211
46239
60114321

9
species
1
 138853 

138853 

 2151 
11111
species
5534121

2151 

2151 
2151 
2151 
2151 


2149 
1
 2149 
1
species


324078 
1
species
 324078 
1


219745 
2
species
1
 219745 


225999 
38
1
species
 225999 

9
1912503
1
genus

1
species
 1541959 
9

1541959 

572813346266167139956180733127974
phylum
485465479485488487
201174


1848755 


1848755 
591
 1848755 
species
11

221184794740
1497346
class
111111

111111
order
588673
221184794740

221184794740
320583
111111
family

221184794740
111111
genus
191494


191495 
191495 
191495 
191495 
191495 
191495 
221184794740
 191495 
111111
species

111111
class
84992
6224116

6224116
order
111111
84993

84994
111111
family
6224116

6224116
53634
genus
111111

 53635 
species
111111
6224116

53635 
53635 
53635 
53635 
53635 
53635 


1848754 
177
1
species
 1848754 

24352127131165122
class
14712121312
84998

102929443748
84999
order
735565

35414242131
family
322232
84107


1531429 
1531429 
1531429 
1531429 
1531429 
1531429 
species
111111
 1531429 
43851119

221619912
102106
genus
111111


74426 
74426 
74426 
74426 
74426 
74426 
species
111111
 74426 
221619912

91
genus
11
33870


33871 


33871 
 33871 
species
11
91

67515201617
1643824
family
413333

24
genus
1
1380

1
species
 1382 
24

1382 

43515201617
133925
313333
genus

369822
species
11111
 712411 

712411 

712411 
712411 
712411 
712411 

4557914
111111
species
 1805478 

1805478 
1805478 
1805478 
1805478 
1805478 
1805478 


133926 

133926 
133926 
133926 
133926 
 133926 
species
11111
31551

1643822
747777
order
14143988712874

747777
family
1643826
14143988712874

11814126
447020
genus
11111


446660 

446660 
446660 
446660 
446660 
species
11111
 446660 
11814126

79603
11111
genus
199121


79604 

79604 
79604 
79604 
79604 
199121
11111
species
 79604 

222222
genus
644652
182340232243

9221388
 1335613 
111111
species

1335613 
1335613 
1335613 
1335613 
1335613 
1335613 

92138101435
species
111111
 1841863 

1841863 
1841863 
1841863 
1841863 
1841863 
1841863 

84108
genus
111111
92673410

92673410
species
111111
 84110 

84110 
84110 
84110 
84110 
84110 
84110 

941825425814
212222
genus
84111

 502558 
11111
species
31212

502558 

502558 
502558 
502558 
502558 


84112 
84112 
84112 
84112 
84112 
84112 
species
111111
 84112 
911824405712

class
222222
84995
365152244639

84996
222222
order
365152244639

365152244639
84997
222222
family

365152244639
genus
222222
42255


42256 
42256 
42256 
42256 
42256 
42256 
1216121062
species
111111
 42256 


49319 
49319 
49319 
49319 
49319 
49319 
 49319 
111111
species
243540144037

3
species
1
 1848255 

1848255 

464454463469471470
class
1760
567353334665902139718180474127756

order
191818202020
85004
17286521556150713971949

17286521556150713971949
191818202020
family
31953

203795612
2701
111111
genus

111111
species
 2702 
203795612

2702 
2702 
2702 
2702 
2702 
2702 

genus
171617181818
1678
15226441547149813831934

8721821
species
111111
 1686 

1686 
1686 
1686 
1686 
1686 
1686 

 638619 
species
111111
2118320

638619 
638619 
638619 
638619 
638619 
638619 


630129 
630129 
630129 
630129 
630129 
630129 
 630129 
111111
species
9193331823

11111
species
 1691 
12358

1691 

1691 
1691 
1691 
1691 

190113397453395574
species
111111
 1685 

1685 
1685 
1685 
1685 
1685 
1685 

8618412565
111111
species
 1689 

1689 
1689 
1689 
1689 
1689 
1689 


33905 
33905 

33905 
33905 
33905 
 33905 
11111
species
13665

species
111111
 1687 
3599216

1687 
1687 
1687 
1687 
1687 
1687 

4821221519
 1683 
111111
species

1683 
1683 
1683 
1683 
1683 
1683 


28026 
28026 
28026 
28026 
21322
 28026 
species
1111

 158787 
species
111111
151030284445

158787 
158787 
158787 
158787 
158787 
158787 

148225374288275440
 216816 
species
111111

216816 
216816 
216816 
216816 
216816 
216816 


1680 
1680 
1680 
1680 
1680 
1680 
 1680 
111111
species
121265394664


1694 
1694 
1694 
1694 
1694 
1694 
261574658144
111111
species
 1694 

941036443758
 35760 
species
111111

35760 
35760 
35760 
35760 
35760 
35760 


28025 
28025 
28025 
28025 
28025 
28025 
111111
species
 28025 
960102309321273304


1684 
1684 
1684 
1684 
1684 
1684 
 1684 
species
111111
7814191818

34100160135111218
111111
species
 1681 

1681 
1681 
1681 
1681 
1681 
1681 

196082
11111
genus
31483

 78258 
species
11111
31483

78258 
78258 

78258 
78258 
78258 

2037
141214141415
order
228140480578515920

family
141214141415
2049
228140480578515920

70121122933
1653174
222222
genus

16181667
111111
species
 2171980 

2171980 
2171980 
2171980 
2171980 
2171980 
2171980 


59505 
59505 
59505 
59505 
59505 
59505 
541136326
 59505 
species
111111

113109400485430728
genus
10999910
1654

111111
species
 1851395 
221049635552

1851395 
1851395 
1851395 
1851395 
1851395 
1851395 

24218110495210
111111
species
 111015 

111015 
111015 
111015 
111015 
111015 
111015 


52773 
52773 
52773 
52773 
52773 
52773 
5211292125
species
111111
 52773 

161421828
111111
species
 1852377 

1852377 
1852377 
1852377 
1852377 
1852377 
1852377 


2079536 
2079536 
2079536 
2079536 
2079536 
2079536 
111111
species
 2079536 
61637343676

species
111111
 1960083 
121556535889

1960083 
1960083 
1960083 
1960083 
1960083 
1960083 

111111
species
 712122 
21948633985

712122 
712122 
712122 
712122 
712122 
712122 


1912795 


1912795 
 1912795 
species
11
11

1522767682120
 544580 
species
111111

544580 
544580 
544580 
544580 
544580 
544580 


2081702 
2081702 
2081702 
2081702 
2081702 
2081702 
6828423642
111111
species
 2081702 

111111
genus
1069494
2219666467130

species
111111
 1661 
2219666467130

1661 
1661 
1661 
1661 
1661 
1661 

2324526
28263
11111
genus

2324526
species
11111
 28264 

28264 

28264 
28264 
28264 
28264 

1343
2050
genus
1111


2051 
2051 
2051 
2051 
1343
 2051 
species
1111

85008
order
131313131313
6977131323152015131762

family
131313131313
28056
6977131323152015131762

genus
555555
1865
253208541674558766

3635579469121
 1866 
species
111111

1866 
1866 
1866 
1866 
1866 
1866 

4181111118102183
 196914 
species
111111

196914 
196914 
196914 
196914 
196914 
196914 


2033844 
2033844 
2033844 
2033844 
2033844 
2033844 
 2033844 
111111
species
3025829584100

111111
species
 649831 
4324108128133131

649831 
649831 
649831 
649831 
649831 
649831 


134676 
134676 
134676 
134676 
134676 
134676 
10343183239170231
111111
species
 134676 

genus
222222
1873
7281182198152204


648999 
648999 
648999 
648999 
648999 
648999 
 648999 
111111
species
4426821106777

28551008885127
 47850 
species
111111

47850 
47850 
47850 
47850 
47850 
47850 

1283596079101
84593
111111
genus

1283596079101
 1003110 
species
111111

1003110 
1003110 
1003110 
1003110 
1003110 
1003110 

genus
333333
673534
333323486511654585


2071627 
2071627 
2071627 
2071627 
2071627 
2071627 
111111
species
 2071627 
144136179208264197

species
111111
 2024580 
222462516178

2024580 
2024580 
2024580 
2024580 
2024580 
2024580 


2108470 
2108470 
2108470 
2108470 
2108470 
2108470 
167163245252329310
species
111111
 2108470 

2718557770106
168694
genus
222222


168695 
168695 
168695 
168695 
168695 
168695 
 168695 
111111
species
11817422075

 168697 
species
111111
161038355031

168697 
168697 
168697 
168697 
168697 
168697 


1650658 
1650658 
1650658 
233
 1650658 
111
species

152146153153155152
order
85007
5812595610216122381192413810

311135735370618956966876
515051525252
family
1762

1073531
genus
222222
3226841125081

12735623021
 1788 
111111
species

1788 
1788 
1788 
1788 
1788 
1788 


875328 
875328 
875328 
875328 
875328 
875328 
201949502060
 875328 
111111
species

523712969113710751281
1866885
131313131313
genus

species
111111
 1800 
362245714162

1800 
1800 
1800 
1800 
1800 
1800 

282266547275
 1791 
species
111111

1791 
1791 
1791 
1791 
1791 
1791 


1771 
1771 
1771 
1771 
1771 
1771 
111111
species
 1771 
308341556676

 1810 
species
111111
505470738456

1810 
1810 
1810 
1810 
1810 
1810 


1772 
1772 
1772 
1772 
1772 
1772 
species
111111
 1772 
219340383446367543


1797 
1797 
1797 
1797 
1797 
1797 
10745414247
species
111111
 1797 


110539 
110539 
110539 
110539 
110539 
110539 
species
111111
 110539 
211038503780


134601 
134601 
134601 
134601 
134601 
134601 
289159769485
 134601 
species
111111

111111
species
 36814 
91011325743

36814 
36814 
36814 
36814 
36814 
36814 

181937514040
 1766 
111111
species

1766 
1766 
1766 
1766 
1766 
1766 


1804 
1804 
1804 
1804 
1804 
1804 
species
111111
 1804 
4939101127110107


1795 
1795 
1795 
1795 
1795 
1795 
8616201527
 1795 
species
111111

17957415040
 758802 
species
111111

758802 
758802 
758802 
758802 
758802 
758802 

7633211324
697025
111111
genus

111111
species
 639313 
7633211324

639313 
639313 
639313 
639313 
639313 
639313 

54563394511049341293
670516
555555
genus

391643553759
 83262 
species
111111

83262 
83262 
83262 
83262 
83262 
83262 


36809 
36809 
36809 
36809 
36809 
36809 
111111
species
 36809 
4735868409828441143


1520670 
1520670 
1520670 
1520670 
1520670 
1520670 
131729332532
species
111111
 1520670 


1578165 
1578165 
1578165 
1578165 
1578165 
1578165 
151124201239
111111
species
 1578165 


1774 
1774 
1774 
1774 
1774 
1774 
111111
species
 1774 
539141620

200421963339381536244197
1763
genus
302930313131


164757 
164757 
164757 
164757 
164757 
164757 
273133463378
 164757 
111111
species


261524 
261524 
261524 
261524 
261524 
261524 
species
111111
 261524 
13272610198


1561223 
1561223 
1561223 
1561223 
1561223 
1561223 
111111
species
 1561223 
263713167024

141435235067
111111
species
 1781 

1781 
1781 
1781 
1781 
1781 
1781 

77643
species group
323444
7418361167151713101441


1773 
1773 
1773 
1773 
1773 
1773 
7048301147147912811419
species
111111
 1773 

221
 78331 
species
111


78331 
78331 
78331 


33894 

33894 
33894 
33894 
33894 
11222
 33894 
species
11111


1765 
1765 
1765 
1765 
1765 
1765 
36619342519
111111
species
 1765 

212052814389
 1682113 
111111
species

1682113 
1682113 
1682113 
1682113 
1682113 
1682113 


1769 
1769 
1769 
1769 
1769 
1769 
 1769 
111111
species
92820104

81344473357
 189918 
species
111111

189918 
189918 
189918 
189918 
189918 
189918 

111111
species
 1879023 
1817565747121

1879023 
1879023 
1879023 
1879023 
1879023 
1879023 

111111
species
 482462 
312972537337

482462 
482462 
482462 
482462 
482462 
482462 


212767 
212767 
212767 
212767 
212767 
212767 
81940254219
 212767 
species
111111

 164756 
species
111111
868587989888

164756 
164756 
164756 
164756 
164756 
164756 

 1273687 
111111
species
201728396329

1273687 
1273687 
1273687 
1273687 
1273687 
1273687 

111111
species
 2051552 
112842395860

2051552 
2051552 
2051552 
2051552 
2051552 
2051552 

9521292315
species
111111
 1168287 

1168287 
1168287 
1168287 
1168287 
1168287 
1168287 

452047493075
species
111111
 1545728 

1545728 
1545728 
1545728 
1545728 
1545728 
1545728 

 1809 
species
111111
385315171925

1809 
1809 
1809 
1809 
1809 
1809 


1768 
1768 
1768 
1768 
1768 
1768 
251657523361
 1768 
111111
species

111111
species group
2249310
131134241955


722731 
722731 
722731 
722731 
722731 
722731 
131134241955
111111
species
 722731 

7638771379147114751688
666666
species group
120793

 222805 
species
111111
160207260296301320

222805 
222805 
222805 
222805 
222805 
222805 

391950353756
species
111111
 701042 

701042 
701042 
701042 
701042 
701042 
701042 

393413710752788833
 1764 
species
111111

1764 
1764 
1764 
1764 
1764 
1764 

141933242241
species
111111
 1138383 

1138383 
1138383 
1138383 
1138383 
1138383 
1138383 


339268 
339268 
339268 
339268 
339268 
339268 
 339268 
111111
species
335757442347

124162269320304391
species
111111
 1767 

1767 
1767 
1767 
1767 
1767 
1767 


1570328 
1570328 
1570328 
1570328 
1570328 
1570328 
6815213249
 1570328 
species
111111


1920667 
1920667 
1920667 
1920667 
1920667 
1920667 
712863733888
species
111111
 1920667 

1358619
111111
species
 29311 

29311 
29311 
29311 
29311 
29311 
29311 

121912872565271827073841
family
323232323232
85025

genus
252525252525
1827
89810381975206920402957

3743807153141
111111
species
 1033922 

1033922 
1033922 
1033922 
1033922 
1033922 
1033922 

 1833 
species
111111
91130138163231623

1833 
1833 
1833 
1833 
1833 
1833 


38310 
38310 
38310 
38310 
38310 
38310 
9625424961
 38310 
111111
species

111111
species
 1828 
362469526994

1828 
1828 
1828 
1828 
1828 
1828 


1727214 
1727214 
1727214 
1727214 
1727214 
1727214 
 1727214 
species
111111
104424243260

species
111111
 1500843 
206267695286

1500843 
1500843 
1500843 
1500843 
1500843 
1500843 


1830 
1830 
1830 
1830 
1830 
1830 
8398192253213277
111111
species
 1830 


1564114 
1564114 
1564114 
1564114 
1564114 
1564114 
3322102599870
 1564114 
111111
species


679318 
679318 
679318 
679318 
679318 
679318 
1992217732
111111
species
 679318 


191292 
191292 
191292 
191292 
191292 
191292 
607982786785
 191292 
111111
species

14923562654
species
111111
 1723645 

1723645 
1723645 
1723645 
1723645 
1723645 
1723645 


132919 
132919 
132919 
132919 
132919 
132919 
202642617953
 132919 
species
111111


103816 
103816 
103816 
103816 
103816 
103816 
species
111111
 103816 
5843113117107151

142581497955
 1653479 
111111
species

1653479 
1653479 
1653479 
1653479 
1653479 
1653479 

 1807790 
111111
species
171987785475

1807790 
1807790 
1807790 
1807790 
1807790 
1807790 

302276667066
 1570939 
111111
species

1570939 
1570939 
1570939 
1570939 
1570939 
1570939 

48439613792194
 43767 
111111
species

43767 
43767 
43767 
43767 
43767 
43767 

131263644948
111111
species
 1990687 

1990687 
1990687 
1990687 
1990687 
1990687 
1990687 


1829 
1829 
1829 
1829 
1829 
1829 
2665736563100
111111
species
 1829 


334542 
334542 
334542 
334542 
334542 
334542 
 334542 
111111
species
4428302552


1653478 
1653478 
1653478 
1653478 
1653478 
1653478 
391468665680
 1653478 
111111
species

 1045808 
111111
species
81719273146

1045808 
1045808 
1045808 
1045808 
1045808 
1045808 


935199 
935199 
935199 
935199 
935199 
935199 
192241638758
 935199 
111111
species

 37919 
111111
species
125113305279292319

37919 
37919 
37919 
37919 
37919 
37919 

111111
species
 1805827 
658759835977

1805827 
1805827 
1805827 
1805827 
1805827 
1805827 

321249590649667884
genus
777777
1817

111111
species
 37332 
10832138119154138

37332 
37332 
37332 
37332 
37332 
37332 

3948599278159
111111
species
 135487 

135487 
135487 
135487 
135487 
135487 
135487 

111111
species
 37326 
3536140121133113

37326 
37326 
37326 
37326 
37326 
37326 

312860696748
species
111111
 455432 

455432 
455432 
455432 
455432 
455432 
455432 


37330 
37330 
37330 
37330 
37330 
37330 
2243493732113
 37330 
species
111111

111111
species
 37329 
7650107131145204

37329 
37329 
37329 
37329 
37329 
37329 


2213200 
2213200 
2213200 
2213200 
2213200 
2213200 
species
111111
 2213200 
1012378058109

171110204619777414
555555
family
85029

171110204619777414
37914
555555
genus


546160 
546160 
546160 
546160 
546160 
546160 
 546160 
111111
species
32454114117575

species
111111
 2052657 
171822418185

2052657 
2052657 
2052657 
2052657 
2052657 
2052657 


499555 
499555 
499555 
499555 
499555 
499555 
111111
species
 499555 
41820385762


712270 
712270 
712270 
712270 
712270 
712270 
641955339393115
species
111111
 712270 

172066607177
111111
species
 139021 

139021 
139021 
139021 
139021 
139021 
139021 

family
777777
85026
302271461966941638

genus
777777
2053
302271461966941638


2055 
2055 
2055 
2055 
2055 
2055 
111111
species
 2055 
9056118247273160

 1004901 
species
111111
162434434350

1004901 
1004901 
1004901 
1004901 
1004901 
1004901 


2059875 
2059875 
2059875 
2059875 
2059875 
2059875 
201060696193
species
111111
 2059875 

 1136941 
species
111111
312570877169

1136941 
1136941 
1136941 
1136941 
1136941 
1136941 


337191 
337191 
337191 
337191 
337191 
337191 
4257609310285
111111
species
 337191 


84595 
84595 
84595 
84595 
84595 
84595 
 84595 
111111
species
251354535372


2054 
2054 
2054 
2054 
2054 
2054 
788665374338109
 2054 
111111
species

53143410520964
1847725
genus
111111


1528099 
1528099 
1528099 
1528099 
1528099 
1528099 
53143410520964
species
111111
 1528099 

6370137149130171
family
222222
85028

6370137149130171
genus
222222
2060


2061 
2061 
2061 
2061 
2061 
2061 
 2061 
111111
species
302255475883

 57704 
species
111111
3348821027288

57704 
57704 
57704 
57704 
57704 
57704 

534854535552
family
1653
8706051428146814411745

1716
genus
534854535552
8706051428146814411745

101850605068
111111
species
 225326 

225326 
225326 
225326 
225326 
225326 
225326 

112715
 258224 
11111
species


258224 
258224 
258224 
258224 
258224 


152794 
152794 
152794 
152794 
152794 
152794 
 152794 
species
111111
1083151218

11111
species
 1408191 
31454

1408191 

1408191 
1408191 
1408191 
1408191 

3131436
species
111111
 92706 

92706 
92706 
92706 
92706 
92706 
92706 


43771 
43771 
43771 
43771 
43771 
43771 
281583525252
111111
species
 43771 

 1718 
111111
species
261108135173141158

1718 
1718 
1718 
1718 
1718 
1718 


156978 
156978 
156978 
156978 
156978 
156978 
species
111111
 156978 
353836423271

67891115
 1231000 
species
111111

1231000 
1231000 
1231000 
1231000 
1231000 
1231000 

 2079535 
111111
species
11414152057

2079535 
2079535 
2079535 
2079535 
2079535 
2079535 


35755 
1
species
1
 35755 


2080740 
2080740 
2080740 
2080740 
2080740 
2080740 
171239303537
 2080740 
species
111111


28028 
28028 
28028 
28028 
28028 
28028 
111111
species
 28028 
7254416

116382
 161879 
11111
species

161879 

161879 
161879 
161879 
161879 

 1404244 
species
111111
171715109

1404244 
1404244 
1404244 
1404244 
1404244 
1404244 


349751 
349751 
349751 
349751 
349751 
349751 
91040413656
 349751 
111111
species


38301 
38301 
38301 
38301 
38301 
38301 
6182281523
 38301 
111111
species

species
111111
 1717 
4744788465109

1717 
1717 
1717 
1717 
1717 
1717 

 38305 
111111
species
5546473654

38305 
38305 
38305 
38305 
38305 
38305 


161899 
161899 
161899 
161899 
161899 
161899 
 161899 
111111
species
54104915

451229547647
 1230998 
species
111111

1230998 
1230998 
1230998 
1230998 
1230998 
1230998 

 161896 
species
111111
14122274039

161896 
161896 
161896 
161896 
161896 
161896 


702967 
702967 
702967 
702967 
702967 
702967 
111111
species
 702967 
4512191118

111111
species
 1072256 
541817119

1072256 
1072256 
1072256 
1072256 
1072256 
1072256 


187491 
187491 
187491 
187491 
187491 
187491 
111111
species
 187491 
243621233125


161895 
161895 
161895 
161895 
161895 
161895 
 161895 
species
111111
5121554

species
111111
 1719 
3537170152149181

1719 
1719 
1719 
1719 
1719 
1719 


1121358 
1121358 
1121358 
1121358 
1121358 
1121358 
12629304465
 1121358 
111111
species

151016383134
 43770 
species
111111

43770 
43770 
43770 
43770 
43770 
43770 


571915 

571915 

571915 
121
species
111
 571915 


1050174 
1050174 
1050174 
1050174 
1050174 
1050174 
4361482
 1050174 
species
111111


89154 
89154 
89154 
89154 
89154 
89154 
28227361818
species
111111
 89154 

211128
 1652495 
species
11111

1652495 

1652495 
1652495 
1652495 
1652495 


146827 
146827 
146827 
146827 
146827 
146827 
111111
species
 146827 
162014232013

species
111111
 1697 
4324148

1697 
1697 
1697 
1697 
1697 
1697 


42817 
42817 
42817 
42817 
42817 
42817 
 42817 
111111
species
226749

 1721 
species
111111
3211265

1721 
1721 
1721 
1721 
1721 
1721 

312824
 1724 
11111
species

1724 

1724 
1724 
1724 
1724 


65058 
65058 
65058 
65058 
65058 
65058 
 65058 
species
111111
213326222430


1705 
1705 
1705 
1705 
1705 
1705 
species
111111
 1705 
7837252637


1487956 
1487956 
1487956 
1487956 
1487956 
1487956 
223536
 1487956 
species
111111

111111
species
 136857 
11416222724

136857 
136857 
136857 
136857 
136857 
136857 

 1223514 
species
111111
10616292335

1223514 
1223514 
1223514 
1223514 
1223514 
1223514 


38289 
38289 
38289 
38289 
38289 
38289 
12737455527
 38289 
111111
species


575200 
575200 
575200 
575200 
575200 
575200 
121025343532
 575200 
species
111111


160386 
160386 
160386 
160386 
160386 
160386 
1112233
111111
species
 160386 

111111
species
 1737425 
6210191132

1737425 
1737425 
1737425 
1737425 
1737425 
1737425 

151743394554
 191493 
111111
species

191493 
191493 
191493 
191493 
191493 
191493 


108486 
108486 
108486 
108486 
108486 
108486 
111111
species
 108486 
546231721

species
111111
 169292 
351412223

169292 
169292 
169292 
169292 
169292 
169292 


1727 
1727 
1727 
1727 
1727 
1727 
4412102365466
111111
species
 1727 

 191610 
species
111111
12612202822

191610 
191610 
191610 
191610 
191610 
191610 

 203263 
species
111111
2223122320

203263 
203263 
203263 
203263 
203263 
203263 

6742352939
species
111111
 401472 

401472 
401472 
401472 
401472 
401472 
401472 

14261
species
1111
 35757 

35757 

35757 
35757 
35757 

111111
family
316606
232617242361

111111
genus
286801
232617242361


286802 
286802 
286802 
286802 
286802 
286802 
111111
species
 286802 
232617242361

128071097635528323463016459456
order
10099100100100100
85006

family
444444
85016
2002307577266692684

2002307577266692684
1707
444444
genus


1708 
1708 
1708 
1708 
1708 
1708 
 1708 
111111
species
4966215204224964

 11 
111111
species
5340183158117562

11 
11 
11 
11 
11 
11 

111111
species
 1711 
4166160174156561

1711 
1711 
1711 
1711 
1711 
1711 

 2003551 
111111
species
5758199190172597

2003551 
2003551 
2003551 
2003551 
2003551 
2003551 

10557902429620254142400225336
family
393839393939
85023

235888
111111
genus
5249281191208196

5249281191208196
species
111111
 2079791 

2079791 
2079791 
2079791 
2079791 
2079791 
2079791 

337004
genus
111111
11689479336354278


279828 
279828 
279828 
279828 
279828 
279828 
 279828 
species
111111
11689479336354278

33877
genus
222222
3823681562118912511191


453304 
453304 
453304 
453304 
453304 
453304 
 453304 
111111
species
227216917684715678

155152645505536513
 2080742 
species
111111

2080742 
2080742 
2080742 
2080742 
2080742 
2080742 

5181085
212222
genus
529883

317574
 535712 
species
111111

535712 
535712 
535712 
535712 
535712 
535712 


529884 

529884 
529884 
529884 
529884 
species
11111
 529884 
21511

111111
genus
110932
3843451481125011701156


1575 
1575 
1575 
1575 
1575 
1575 
species
111111
 1575 
3843451481125011701156

222222
genus
1705353
15989505363


1987356 
1987356 
1987356 
1987356 
1987356 
1987356 
7649173427
111111
species
 1987356 

 708131 
111111
species
8340331936

708131 
708131 
708131 
708131 
708131 
708131 

genus
222222
33886
217173640668731609


33888 
33888 
33888 
33888 
33888 
33888 
167127440507541445
 33888 
species
111111

111111
species
 145458 
5046200161190164

145458 
145458 
145458 
145458 
145458 
145458 

447237
genus
111111
8345174972321028612524

 1795630 
species
111111
8345174972321028612524

1795630 
1795630 
1795630 
1795630 
1795630 
1795630 

518733
genus
111111
210219654490492563


412690 
412690 
412690 
412690 
412690 
412690 
210219654490492563
 412690 
species
111111

166818445302449346768151
genus
131313131313
33882

 1906742 
111111
species
106155512423489546

1906742 
1906742 
1906742 
1906742 
1906742 
1906742 


1795053 
1795053 
1795053 
1795053 
1795053 
1795053 
305159391316373710
111111
species
 1795053 


1938334 
1938334 
1938334 
1938334 
1938334 
1938334 
species
111111
 1938334 
1171524053192812050

10298415389353387
 1714373 
species
111111

1714373 
1714373 
1714373 
1714373 
1714373 
1714373 


367477 
367477 
367477 
367477 
367477 
367477 
100119361287305547
species
111111
 367477 

111111
species
 2103230 
136180411314291624

2103230 
2103230 
2103230 
2103230 
2103230 
2103230 

122207426420450629
species
111111
 1916917 

1916917 
1916917 
1916917 
1916917 
1916917 
1916917 


162426 
162426 
162426 
162426 
162426 
162426 
129105443349345383
111111
species
 162426 

111111
species
 36805 
124115350364372398

36805 
36805 
36805 
36805 
36805 
36805 


300019 
300019 
300019 
300019 
300019 
300019 
species
111111
 300019 
120127416325421439


1696072 
1696072 
1696072 
1696072 
1696072 
1696072 
111188370355337563
111111
species
 1696072 

123158515388400544
species
111111
 2033 

2033 
2033 
2033 
2033 
2033 
2033 


84292 
84292 
84292 
84292 
84292 
84292 
7381287244259331
111111
species
 84292 

189158691690672566
genus
111111
1759331

189158691690672566
species
111111
 1619308 

1619308 
1619308 
1619308 
1619308 
1619308 
1619308 

111111
genus
76634
7697435288284264


2079792 
2079792 
2079792 
2079792 
2079792 
2079792 
111111
species
 2079792 
7697435288284264

419631465865308246003366
2034
genus
333333


1905847 
1905847 
1905847 
1905847 
1905847 
1905847 
 1905847 
111111
species
15481050184494114701016

 1561023 
species
111111
1080820184899113771047

1561023 
1561023 
1561023 
1561023 
1561023 
1561023 

species
111111
 69373 
156812762173115017531303

69373 
69373 
69373 
69373 
69373 
69373 

4413131143
genus
111111
1434032


1159327 
1159327 
1159327 
1159327 
1159327 
1159327 
4413131143
species
111111
 1159327 

262276120799310691185
69578
genus
222222


670052 
670052 
670052 
670052 
670052 
670052 
 670052 
111111
species
146161666532591612


1978566 
1978566 
1978566 
1978566 
1978566 
1978566 
116115541461478573
species
111111
 1978566 

194717295941846155625176
555555
genus
1573

240180778909676597
 31964 
111111
species

31964 
31964 
31964 
31964 
31964 
31964 


1874630 
1874630 
1874630 
1874630 
1874630 
1874630 
 1874630 
species
111111
253251704925684626


31963 
31963 
31963 
31963 
31963 
31963 
111111
species
 31963 
2612276661093690697


28447 
28447 
28447 
28447 
28447 
28447 
227231756972727662
 28447 
species
111111


33014 
33014 
33014 
33014 
33014 
33014 
9668403037456227852594
 33014 
111111
species

85020
777777
family
182163483513438780

genus
555555
43668
171150459485413744


1903186 
1903186 
1903186 
1903186 
1903186 
1903186 
 1903186 
species
111111
2830808068155


2017485 
2017485 
2017485 
2017485 
2017485 
2017485 
species
111111
 2017485 
1417545773120


2017484 
2017484 
2017484 
2017484 
2017484 
2017484 
111111
species
 2017484 
3428647850121


43669 
43669 
43669 
43669 
43669 
43669 
 43669 
111111
species
4834141144121126


1331682 
1331682 
1331682 
1331682 
1331682 
1331682 
111111
species
 1331682 
4741120126101222

7108141020
472568
111111
genus

 472569 
species
111111
7108141020

472569 
472569 
472569 
472569 
472569 
472569 

36739
genus
111111
4316141516

111111
species
 1630135 
4316141516

1630135 
1630135 
1630135 
1630135 
1630135 
1630135 

family
111111
145360
889619730823320161

889619730823320161
111111
genus
60919


60920 
60920 
60920 
60920 
60920 
60920 
 60920 
111111
species
889619730823320161

family
555555
85017
2322527718867964167

435094180118510
186188
111111
genus


186189 
186189 
186189 
186189 
186189 
186189 
 186189 
species
111111
435094180118510

105983453453522194
222222
genus
157920

52411721641861166
species
111111
 1980001 

1980001 
1980001 
1980001 
1980001 
1980001 
1980001 

111111
species
 1710 
53571731811661028

1710 
1710 
1710 
1710 
1710 
1710 

841043323613261463
254250
222222
genus


139208 
139208 
139208 
139208 
139208 
139208 
species
111111
 139208 
4859224216194743


372663 
372663 
372663 
372663 
372663 
372663 
111111
species
 372663 
3645108145132720

10284334345255723
125316
222222
family

84756
111111
genus
4733171189146299


84757 
84757 
84757 
84757 
84757 
84757 
species
111111
 84757 
4733171189146299

5551163156109424
111111
genus
947525

 2171623 
111111
species
5551163156109424

2171623 
2171623 
2171623 
2171623 
2171623 
2171623 

465810911588160
85019
family
111111

465810911588160
1696
genus
111111

species
111111
 1703 
465810911588160

1703 
1703 
1703 
1703 
1703 
1703 

85022
family
111111
218225116424

43673
111111
genus
218225116424

218225116424
species
111111
 43674 

43674 
43674 
43674 
43674 
43674 
43674 

333333
family
145357
14286312493493549

4432127129159228
57495
111111
genus


1274 
1274 
1274 
1274 
1274 
1274 
4432127129159228
 1274 
species
111111

genus
111111
745364
402197111136195


571913 
571913 
571913 
571913 
571913 
571913 
402197111136195
species
111111
 571913 

57499
genus
111111
583388253198126


1276 
1276 
1276 
1276 
1276 
1276 
583388253198126
 1276 
species
111111

family
111111
85018
34581023

genus
111111
1862
34581023


1863 
1863 
1863 
1863 
1863 
1863 
 1863 
species
111111
34581023

2941481422
2038
111111
genus

 2039 
111111
species
2941481422

2039 
2039 
2039 
2039 
2039 
2039 

10528602538286726523657
family
313131313131
1268

41232282015
genus
111111
1868332


556325 
556325 
556325 
556325 
556325 
556325 
41232282015
 556325 
species
111111

1269
111111
genus
16799173506483287

 1270 
species
111111
16799173506483287

1270 
1270 
1270 
1270 
1270 
1270 

64351039973115
1742989
genus
222222


1933880 
1933880 
1933880 
1933880 
1933880 
1933880 
111111
species
 1933880 
18570675574

463033321841
111111
species
 256701 

256701 
256701 
256701 
256701 
256701 
256701 

1742992
genus
111111
131035473264


43663 
43663 
43663 
43663 
43663 
43663 
131035473264
111111
species
 43663 

169116448453448603
57493
genus
444444


1702043 
1702043 
1702043 
1702043 
1702043 
1702043 
 1702043 
species
111111
3412556357118

 72000 
111111
species
4951157136117170

72000 
72000 
72000 
72000 
72000 
72000 


446860 
446860 
446860 
446860 
446860 
446860 
 446860 
111111
species
5523118121123162


71999 
71999 
71999 
71999 
71999 
71999 
3130118133151153
111111
species
 71999 

456417118110679751693
genus
151515151515
1663

91039323352
species
111111
 1771959 

1771959 
1771959 
1771959 
1771959 
1771959 
1771959 


1849032 
1849032 
1849032 
1849032 
1849032 
1849032 
 1849032 
111111
species
44239510285153


37928 
37928 
37928 
37928 
37928 
37928 
111111
species
 37928 
202457655388

111111
species
 1494608 
192276716583

1494608 
1494608 
1494608 
1494608 
1494608 
1494608 

 1118963 
111111
species
1930724259130

1118963 
1118963 
1118963 
1118963 
1118963 
1118963 


1652545 
1652545 
1652545 
1652545 
1652545 
1652545 
species
111111
 1652545 
2927816863118

51261118397143
species
111111
 290399 

290399 
290399 
290399 
290399 
290399 
290399 


2079227 
2079227 
2079227 
2079227 
2079227 
2079227 
species
111111
 2079227 
3718849287123

 1618207 
species
111111
4417181027

1618207 
1618207 
1618207 
1618207 
1618207 
1618207 

34319678100127
111111
species
 1357915 

1357915 
1357915 
1357915 
1357915 
1357915 
1357915 


1806905 
1806905 
1806905 
1806905 
1806905 
1806905 
121759573789
111111
species
 1806905 


656366 
656366 
656366 
656366 
656366 
656366 
7142193119120248
species
111111
 656366 


1690248 
1690248 
1690248 
1690248 
1690248 
1690248 
 1690248 
111111
species
53976210959109


1704044 
1704044 
1704044 
1704044 
1704044 
1704044 
3536989576117
 1704044 
111111
species


2020486 
2020486 
2020486 
2020486 
2020486 
2020486 
191041363186
111111
species
 2020486 

6459207207187337
596707
genus
111111

 37927 
species
111111
6459207207187337

37927 
37927 
37927 
37927 
37927 
37927 

1742993
genus
333333
8681258260254400


361575 
361575 
361575 
361575 
361575 
361575 
 361575 
111111
species
171146565292


85085 
85085 
85085 
85085 
85085 
85085 
3439107115124159
 85085 
111111
species

111111
species
 121292 
35311058978149

121292 
121292 
121292 
121292 
121292 
121292 

252784182174119
222222
genus
32207


2047 
2047 
2047 
2047 
2047 
2047 
9311193430
111111
species
 2047 

 43675 
species
111111
16247316314089

43675 
43675 
43675 
43675 
43675 
43675 

441718624
1645
genus
111111


1646 
1646 
1646 
1646 
1646 
1646 
species
111111
 1646 
441718624

153107366612498770
444444
family
85021

111111
genus
53457
6133102218221230


857417 
857417 
857417 
857417 
857417 
857417 
 857417 
species
111111
6133102218221230

genus
111111
265976
19217711877139


1758689 
1758689 
1758689 
1758689 
1758689 
1758689 
species
111111
 1758689 
19217711877139

genus
111111
267408
4131105172110244

 1658671 
111111
species
4131105172110244

1658671 
1658671 
1658671 
1658671 
1658671 
1658671 

32228210490157
genus
111111
53357


53358 
53358 
53358 
53358 
53358 
53358 
32228210490157
111111
species
 53358 

order
111111
85014
43753447243

43753447243
111111
family
85034

43753447243
genus
111111
283810

43753447243
 283811 
species
111111

283811 
283811 
283811 
283811 
283811 
283811 

order
111111
1643683
213864

213864
85032
family
111111

213864
28048
111111
genus

213864
 28049 
111111
species

28049 
28049 
28049 
28049 
28049 
28049 

85011
909090909090
order
3983427782349428811113612

2062
family
909090909090
3983427782349428811113612

156279339364317617
genus
444444
2063


2066 
2066 
2066 
2066 
2066 
2066 
111111
species
 2066 
25651099586104


2018025 
2018025 
2018025 
2018025 
2018025 
2018025 
111111
species
 2018025 
22319211863178


1894 
1894 
1894 
1894 
1894 
1894 
569910795128152
 1894 
111111
species

5384315640183
 68173 
111111
species

68173 
68173 
68173 
68173 
68173 
68173 

3827399878959064779412995
genus
868686868686
1883


1961713 
1961713 
1961713 
1961713 
1961713 
1961713 
102344845287
species
111111
 1961713 

5158186194162222
species
111111
 1889 

1889 
1889 
1889 
1889 
1889 
1889 


2059884 
2059884 
2059884 
2059884 
2059884 
2059884 
4857969894151
 2059884 
111111
species


1984801 
1984801 
1984801 
1984801 
1984801 
1984801 
 1984801 
111111
species
3837999983156

989310114266112
 1930 
species
111111

1930 
1930 
1930 
1930 
1930 
1930 

8443959286107
111111
species
 2094021 

2094021 
2094021 
2094021 
2094021 
2094021 
2094021 

2229589589123
 1751294 
species
111111

1751294 
1751294 
1751294 
1751294 
1751294 
1751294 

3878751035672
111111
species
 1964449 

1964449 
1964449 
1964449 
1964449 
1964449 
1964449 

species
111111
 164348 
14294510758103

164348 
164348 
164348 
164348 
164348 
164348 


465541 
465541 
465541 
465541 
465541 
465541 
111111
species
 465541 
3423896557135


1736046 
1736046 
1736046 
1736046 
1736046 
1736046 
 1736046 
111111
species
3899899491175


68249 
68249 
68249 
68249 
68249 
68249 
 68249 
species
111111
33697412067120


1649184 
1649184 
1649184 
1649184 
1649184 
1649184 
species
111111
 1649184 
3019504949152


193462 
193462 
193462 
193462 
193462 
193462 
221159664865
species
111111
 193462 


1495638 
1495638 
1495638 
1495638 
1495638 
1495638 
609012299107112
 1495638 
111111
species

3827589578177
 1616117 
111111
species

1616117 
1616117 
1616117 
1616117 
1616117 
1616117 


1661694 
1661694 
1661694 
1661694 
1661694 
1661694 
 1661694 
species
111111
3223737877166


1725411 
1725411 
1725411 
1725411 
1725411 
1725411 
111111
species
 1725411 
31198410680147

 1915 
111111
species
29209211769126

1915 
1915 
1915 
1915 
1915 
1915 

475177805382
 75293 
species
111111

75293 
75293 
75293 
75293 
75293 
75293 

species
111111
 67267 
18964786169

67267 
67267 
67267 
67267 
67267 
67267 

 565560 
species
111111
241887905477

565560 
565560 
565560 
565560 
565560 
565560 


362257 
362257 
362257 
362257 
362257 
362257 
28237810485147
111111
species
 362257 


1262452 
1262452 
1262452 
1262452 
1262452 
1262452 
111111
species
 1262452 
2610861066293

31265973105166
 234612 
species
111111

234612 
234612 
234612 
234612 
234612 
234612 


1690221 
1690221 
1690221 
1690221 
1690221 
1690221 
 1690221 
111111
species
146667606595

652888106129122
species
111111
 862751 

862751 
862751 
862751 
862751 
862751 
862751 

 2202000 
species
111111
4344948259119

2202000 
2202000 
2202000 
2202000 
2202000 
2202000 

5219799970106
species
111111
 553510 

553510 
553510 
553510 
553510 
553510 
553510 

 379067 
species
111111
396461102109166

379067 
379067 
379067 
379067 
379067 
379067 

111111
species
 42684 
2527989268113

42684 
42684 
42684 
42684 
42684 
42684 


206662 
206662 
206662 
206662 
206662 
206662 
111111
species
 206662 
6824667574102


1837283 
1837283 
1837283 
1837283 
1837283 
1837283 
 1837283 
species
111111
1614547675161


68280 
68280 
68280 
68280 
68280 
68280 
 68280 
111111
species
2329808552138


1912 
1912 
1912 
1912 
1912 
1912 
 1912 
111111
species
212269344430346573

6473187210174393
111111
species
 1969 

1969 
1969 
1969 
1969 
1969 
1969 


33903 
33903 
33903 
33903 
33903 
33903 
521910577104101
 33903 
species
111111

629295
333333
species subgroup
101101269291323622

 1908 
species
111111
5642138153130304

1908 
1908 
1908 
1908 
1908 
1908 

28385762121151
species
111111
 1911 

1911 
1911 
1911 
1911 
1911 
1911 


68179 
68179 
68179 
68179 
68179 
68179 
111111
species
 68179 
1721747672167


2135430 
2135430 
2135430 
2135430 
2135430 
2135430 
species
111111
 2135430 
6724919269133


1971 
1971 
1971 
1971 
1971 
1971 
38538810610176
 1971 
species
111111

 2049881 
111111
species
1824786279112

2049881 
2049881 
2049881 
2049881 
2049881 
2049881 

3051718477104
111111
species
 1935 

1935 
1935 
1935 
1935 
1935 
1935 

 1940 
species
111111
6079718397141

1940 
1940 
1940 
1940 
1940 
1940 

111111
species
 92644 
2348571075482

92644 
92644 
92644 
92644 
92644 
92644 

 54571 
111111
species
85113243267228449

54571 
54571 
54571 
54571 
54571 
54571 


1902 
1902 
1902 
1902 
1902 
1902 
1493110611872153
species
111111
 1902 


1907 
1907 
1907 
1907 
1907 
1907 
2751758578136
 1907 
111111
species


1535768 
1535768 
1535768 
1535768 
1535768 
1535768 
 1535768 
111111
species
243553796096

species
111111
 1841249 
2745847791144

1841249 
1841249 
1841249 
1841249 
1841249 
1841249 


444103 
444103 
444103 
444103 
444103 
444103 
16329968162141
 444103 
111111
species

1917631046272
species
111111
 1038928 

1038928 
1038928 
1038928 
1038928 
1038928 
1038928 

3536861079794
species
111111
 42239 

42239 
42239 
42239 
42239 
42239 
42239 


2174846 
2174846 
2174846 
2174846 
2174846 
2174846 
 2174846 
111111
species
261643774582


2184053 
2184053 
2184053 
2184053 
2184053 
2184053 
361658928299
species
111111
 2184053 

species
111111
 1972846 
2631586760125

1972846 
1972846 
1972846 
1972846 
1972846 
1972846 

86148828787128
111111
species
 1783515 

1783515 
1783515 
1783515 
1783515 
1783515 
1783515 

 1901 
species
111111
381562694164

1901 
1901 
1901 
1901 
1901 
1901 


29303 
29303 
29303 
29303 
29303 
29303 
7157140175157192
 29303 
species
111111


408015 
408015 
408015 
408015 
408015 
408015 
4916476574108
species
111111
 408015 

1620618510280
species
111111
 68214 

68214 
68214 
68214 
68214 
68214 
68214 


1355015 
1355015 
1355015 
1355015 
1355015 
1355015 
 1355015 
species
111111
576212212490134


1914 
1914 
1914 
1914 
1914 
1914 
 1914 
111111
species
503410911685145

52869612282130
 1885 
111111
species

1885 
1885 
1885 
1885 
1885 
1885 


1926 
1926 
1926 
1926 
1926 
1926 
2936688792114
 1926 
111111
species


1561022 
1561022 
1561022 
1561022 
1561022 
1561022 
313013610779122
 1561022 
species
111111

1615527071114
 146923 
species
111111

146923 
146923 
146923 
146923 
146923 
146923 


1265601 
1265601 
1265601 
1265601 
1265601 
1265601 
2046546669109
 1265601 
species
111111

111111
species
 1642299 
191368706388

1642299 
1642299 
1642299 
1642299 
1642299 
1642299 

 1849967 
species
111111
2124708383135

1849967 
1849967 
1849967 
1849967 
1849967 
1849967 


1812480 
1812480 
1812480 
1812480 
1812480 
1812480 
2621547984106
species
111111
 1812480 


1437453 
1437453 
1437453 
1437453 
1437453 
1437453 
 1437453 
111111
species
3533716866139


1827580 
1827580 
1827580 
1827580 
1827580 
1827580 
111111
species
 1827580 
2725886882129

33112748151132
 285473 
111111
species

285473 
285473 
285473 
285473 
285473 
285473 


68202 
68202 
68202 
68202 
68202 
68202 
 68202 
111111
species
4548728961619


68192 
68192 
68192 
68192 
68192 
68192 
species
111111
 68192 
3049426395105

108121244255219388
111111
species
 47763 

47763 
47763 
47763 
47763 
47763 
47763 


68570 
68570 
68570 
68570 
68570 
68570 
 68570 
species
111111
11555260319295327

111111
species
 1916 
2229607276140

1916 
1916 
1916 
1916 
1916 
1916 


1109743 
1109743 
1109743 
1109743 
1109743 
1109743 
111111
species
 1109743 
2342796350119


1169025 
1169025 
1169025 
1169025 
1169025 
1169025 
 1169025 
species
111111
174466747390


47716 
47716 
47716 
47716 
47716 
47716 
2818859366143
species
111111
 47716 


1950 
1950 
1950 
1950 
1950 
1950 
1310676965127
111111
species
 1950 

5010253886785
 38300 
111111
species

38300 
38300 
38300 
38300 
38300 
38300 

1852274
111111
species group
149227360350318491

111111
species
 1888 
149227360350318491

1888 
1888 
1888 
1888 
1888 
1888 

188250313333271366
555555
order
85013

188250313333271366
74712
family
555555

555555
genus
1854
188250313333271366


298654 
298654 
298654 
298654 
298654 
298654 
28361027486104
111111
species
 298654 


1859 
1859 
1859 
1859 
1859 
1859 
202356893792
 1859 
111111
species


106370 
106370 
106370 
106370 
106370 
106370 
111111
species
 106370 
504139453558


298653 
298653 
298653 
298653 
298653 
298653 
6313985856866
 298653 
species
111111


656024 
656024 
656024 
656024 
656024 
656024 
 656024 
species
111111
271131404546

85010
order
282828282828
124613852537309525503615

family
282828282828
2070
124613852537309525503615

65496
555555
genus
9167233251215403

species
111111
 340345 
122143302848

340345 
340345 
340345 
340345 
340345 
340345 


1612551 
1612551 
1612551 
1612551 
1612551 
1612551 
 1612551 
species
111111
131431354750


2072503 
2072503 
2072503 
2072503 
2072503 
2072503 
121148524695
111111
species
 2072503 

species
111111
 1470176 
259525527125

1470176 
1470176 
1470176 
1470176 
1470176 
1470176 


1612552 
1612552 
1612552 
1612552 
1612552 
1612552 
 1612552 
species
111111
291259796785

101329354254
genus
111111
1851


1852 
1852 
1852 
1852 
1852 
1852 
species
111111
 1852 
101329354254

2071
111111
genus
40383511881122


103731 
103731 
103731 
103731 
103731 
103731 
40383511881122
 103731 
species
111111

43439211196106
111111
genus
165301


1586287 
1586287 
1586287 
1586287 
1586287 
1586287 
43439211196106
 1586287 
111111
species

111111
genus
43356
548347739495

548347739495
 43357 
species
111111

43357 
43357 
43357 
43357 
43357 
43357 

315810213198166
1835
genus
111111

315810213198166
species
111111
 1836 

1836 
1836 
1836 
1836 
1836 
1836 

666666
genus
1813
50648597411609861209

species
111111
 1814 
45477210910179

1814 
1814 
1814 
1814 
1814 
1814 

 1911175 
111111
species
792357985579

1911175 
1911175 
1911175 
1911175 
1911175 
1911175 

111111
species
 208439 
3053637982109

208439 
208439 
208439 
208439 
208439 
208439 

 1896961 
111111
species
343185926682

1896961 
1896961 
1896961 
1896961 
1896961 
1896961 

274247574630530721
species
111111
 33910 

33910 
33910 
33910 
33910 
33910 
33910 


31958 
31958 
31958 
31958 
31958 
31958 
4484123152152139
 31958 
species
111111

111111
genus
142577
395636493061


530584 
530584 
530584 
530584 
530584 
530584 
395636493061
 530584 
species
111111

34236814148112
111111
genus
674734

34236814148112
species
111111
 1653480 

1653480 
1653480 
1653480 
1653480 
1653480 
1653480 

genus
777777
1847
264337644727586819


1641402 
1641402 
1641402 
1641402 
1641402 
1641402 
111111
species
 1641402 
537711311172120


1096868 
1096868 
1096868 
1096868 
1096868 
1096868 
3235789191172
 1096868 
111111
species


1096856 
1096856 
1096856 
1096856 
1096856 
1096856 
2135971016793
111111
species
 1096856 

 1688404 
species
111111
2769939574102

1688404 
1688404 
1688404 
1688404 
1688404 
1688404 

22226012680105
 240495 
111111
species

240495 
240495 
240495 
240495 
240495 
240495 


445576 
445576 
445576 
445576 
445576 
445576 
42629510288100
 445576 
111111
species


1690815 
1690815 
1690815 
1690815 
1690815 
1690815 
species
111111
 1690815 
6737108101114127

2611593869076
2029
111111
genus

2611593869076
 860235 
species
111111

860235 
860235 
860235 
860235 
860235 
860235 

10867184213184392
40566
genus
222222


40567 
40567 
40567 
40567 
40567 
40567 
 40567 
111111
species
6539100126122235


42197 
42197 
42197 
42197 
42197 
42197 
4328848762157
111111
species
 42197 

1930718485154
414714
order
111111

414877
111111
family
1930718485154

1930718485154
111111
genus
414878

1930718485154
 304895 
species
111111

304895 
304895 
304895 
304895 
304895 
304895 

302726262739
622450
order
111111

302726262739
111111
family
622451

302726262739
1849
111111
genus


414996 
414996 
414996 
414996 
414996 
414996 
species
111111
 414996 
302726262739

28502827742427667312262729829
85009
222221222222
order

4363958409937781328
85015
888888
family

111111
genus
2044
102152168149123230


2045 
2045 
2045 
2045 
2045 
2045 
111111
species
 2045 
102152168149123230

33159512798228
182639
genus
111111


182640 
182640 
182640 
182640 
182640 
182640 
species
111111
 182640 
33159512798228

4222717463126
116071
genus
111111


75385 
75385 
75385 
75385 
75385 
75385 
species
111111
 75385 
4222717463126

8450295297239381
genus
222222
2040

4922147151105195
species
111111
 2079793 

2079793 
2079793 
2079793 
2079793 
2079793 
2079793 


2041 
2041 
2041 
2041 
2041 
2041 
3528148146134186
111111
species
 2041 

175156211346255363
1839
333333
genus


196162 
196162 
196162 
196162 
196162 
196162 
species
111111
 196162 
36487714793139

33177613192130
111111
species
 450734 

450734 
450734 
450734 
450734 
450734 
450734 


2045452 
2045452 
2045452 
2045452 
2045452 
2045452 
1069158687094
species
111111
 2045452 

28066788234027568012184928501
141413141414
family
31957

72763
genus
555555
237138263366303535

111432465080
 2161816 
111111
species

2161816 
2161816 
2161816 
2161816 
2161816 
2161816 

1411742593064
species
111111
 1610493 

1610493 
1610493 
1610493 
1610493 
1610493 
1610493 


1909732 
1909732 
1909732 
1909732 
1909732 
1909732 
 1909732 
111111
species
1614375647110


399497 
399497 
399497 
399497 
399497 
399497 
3871659770132
 399497 
species
111111

312287108106149
111111
species
 1332264 

1332264 
1332264 
1332264 
1332264 
1332264 
1332264 

3768331582121214051055
1743
genus
222222


1744 
1744 
1744 
1744 
1744 
1744 
 1744 
species
111111
3553246536582504798

 671223 
species
111111
2158546630901257

671223 
671223 
671223 
671223 
671223 
671223 

10286243353240398
1912215
genus
111111

10286243353240398
species
111111
 1748 

1748 
1748 
1748 
1748 
1748 
1748 

23915729522087363811982926405
1912216
333333
genus

52344885127116
species
111111
 33010 

33010 
33010 
33010 
33010 
33010 
33010 


33011 
33011 
33011 
33011 
33011 
33011 
11517274734
111111
species
 33011 


1747 
1747 
1747 
1747 
1747 
1747 
23852725621437352611965526255
 1747 
species
111111

genus
111111
1912217
10823301428


1750 
1750 
1750 
1750 
1750 
1750 
10823301428
 1750 
species
111111

322283785778
29404
111111
genus


29405 
29405 
29405 
29405 
29405 
29405 
322283785778
111111
species
 29405 

22312
11111
genus
203133


1871034 
1871034 

1871034 
1871034 
1871034 
 1871034 
11111
species
22312

1643684
order
111111
27286410189103

85031
111111
family
27286410189103

27286410189103
111111
genus
53460

27286410189103
species
111111
 53461 

53461 
53461 
53461 
53461 
53461 
53461 

145180349478288499
333333
order
1643682

145180349478288499
85030
family
333333

38501
111111
genus
584310613699132

584310613699132
111111
species
 138336 

138336 
138336 
138336 
138336 
138336 
138336 

41106110168103165
genus
111111
1860


1861 
1861 
1861 
1861 
1861 
1861 
41106110168103165
 1861 
species
111111

463113317486202
88138
111111
genus


477641 
477641 
477641 
477641 
477641 
477641 
species
111111
 477641 
463113317486202

order
444667
2039638
918522173135

918522173135
2162846
family
444667

2039639
112212
genus
516248

5146
species
1111
 1884915 


1884915 
1884915 
1884915 
1884915 

1112
 1884916 
1111
species


1884916 
1884916 
1884916 

1884916 


1884634 
species
1
 1884634 
5

913416152727
622681
genus
332455


1884907 
1884907 
11
 1884907 
species
11

 1884914 
species
111111
321161210

1884914 
1884914 
1884914 
1884914 
1884914 
1884914 


573600 

573600 
11
 573600 
11
species


1884904 
1884904 
 1884904 
species
11
12

 1884905 
11111
species
101252

1884905 
1884905 

1884905 
1884905 
1884905 


1884913 

1884913 
1884913 
1884913 
1884913 
 1884913 
species
11111
90056812

147067
genus
111111
2715315333126


2006 
2006 
2006 
2006 
2006 
2006 
2715315333126
species
111111
 2006 

6575186370140366
order
111111
622452

6575186370140366
83778
family
111111

111111
genus
33981
6575186370140366

6575186370140366
 131568 
species
111111

131568 
131568 
131568 
131568 
131568 
131568 

85012
order
777777
3073226688176281065

222222
family
2004
11898211289241377

83681
genus
111111
7674134195169194

species
111111
 1909395 
7674134195169194

1909395 
1909395 
1909395 
1909395 
1909395 
1909395 

4224779472183
genus
111111
2000


2001 
2001 
2001 
2001 
2001 
2001 
4224779472183
111111
species
 2001 

83676
family
444444
176199421460356628

333333
genus
2013
163194390445322580

species
111111
 2014 
111117282292214391

2014 
2014 
2014 
2014 
2014 
2014 

111111
species
 280236 
336658815189

280236 
280236 
280236 
280236 
280236 
280236 


53437 
53437 
53437 
53437 
53437 
53437 
species
111111
 53437 
1911507257100

13531153448
83677
genus
111111

13531153448
 2021 
species
111111

2021 
2021 
2021 
2021 
2021 
2021 

2012
family
111111
132536683160

132536683160
111111
genus
2019

species
111111
 2020 
132536683160

2020 
2020 
2020 
2020 
2020 
2020 

64113
22111
phylum
74152

44113
447830
12111
class

12111
order
1783344
44113

1783343
12111
family
44113

12111
genus
1408194
44113


1408204 
1408204 
21
11
species
 1408204 

1111
species
 1408281 
4213

1408281 
1408281 

1408281 

1408281 

1
class
641853
2

1
order
641854
2

family
1
641876
2

2
genus
1
423604

2
1
species
 423605 

423605 

309431213232183321
phylum
141216121415
203682

261228167149147182
203683
class
131114101313

112
121013101212
order
256223160149143167

1763524
333233
family
11510751434358

genus
11111
127
2477255


128 
128 
128 

128 
128 
 128 
species
11111
2477255

142534282940
1763521
111111
genus

142534282940
 1387353 
species
111111

1387353 
1387353 
1387353 
1387353 
1387353 
1387353 

466152
genus
111111
7751515913


466153 
466153 
466153 
466153 
466153 
466153 
7751515913
 466153 
111111
species

1914233
222222
family
631349473940

631349473940
222222
genus
113

31532382514
 114 
species
111111

114 
114 
114 
114 
114 
114 


1630693 
1630693 
1630693 
1630693 
1630693 
1630693 
3281791426
 1630693 
111111
species

126
family
758677
7810360596169

genus
111111
265488
3115462

3115462
 265606 
species
111111

265606 
265606 
265606 
265606 
265606 
265606 

1361
1649480
genus
1111


120 

120 

120 
120 
1361
1111
species
 120 

16603312
1936111
genus
111111

 1891926 
111111
species
16603312

1891926 
1891926 
1891926 
1891926 
1891926 
1891926 

1676125
genus
1
1

1
species
1
 1331910 


1331910 

341038353055
118
genus
222222


1632864 
1632864 
1632864 
1632864 
1632864 
1632864 
5518101216
 1632864 
species
111111

29520251839
species
111111
 1636152 

1636152 
1636152 
1636152 
1636152 
1636152 
1636152 

genus
111111
123
92238118


125 
125 
125 
125 
125 
125 
species
111111
 125 
92238118

157971
1649490
11111
genus


119 

119 
119 
119 
119 
157971
species
11111
 119 

557415
order
11111
1127829

1127830
family
11111
557415

557415
380738
11111
genus


174633 
174633 
174633 

174633 
174633 
557415
species
11111
 174633 

112212
class
666505
48203468336139

21
11
species
 1941349 


1941349 

1941349 

species
1
 1940790 
2


1940790 

order
111111
666506
48203448136138

48203448136138
666507
111111
family

111111
genus
666508
48203448136138

 547188 
111111
species
48203448136138

547188 
547188 
547188 
547188 
547188 
547188 

21
phylum
68297
22

22
class
21
203486

22
order
21
203487

family
21
203488
22

13
genus
21
22

12
11
species
 14 

14 


14 

species
1
 513050 
1

513050 

251757344850
40117
658757
phylum

251757344850
203693
class
658757

251757344850
189778
658757
order

658757
family
189779
251757344850

179
113213
genus
1171014

1142
261386
species group
1111

1142
species
1111
 178606 

178606 

178606 
178606 

178606 

111
species group
655606
311


180 

180 
180 
311
111
species
 180 

1361
1111
species group
261385

1361
 1660083 
species
1111


1660083 
1660083 
1660083 

1660083 

141633214746
1234
444444
genus


1715989 
1715989 
1715989 
1715989 
1715989 
1715989 
9876121
 1715989 
111111
species


42253 
42253 
42253 
42253 
42253 
42253 
321082038
species
111111
 42253 


330214 
330214 
330214 
330214 
330214 
330214 
species
111111
 330214 
15156104

111153
 1325564 
species
111111

1325564 
1325564 
1325564 
1325564 
1325564 
1325564 

28261
genus
111
10173

10173
111
species
 28262 

28262 

28262 
28262 

435434
phylum
204428
27691514610

204429
class
435434
27691514610

111
order
1963360
121

family
111
92713
121

121
282132
genus
111


389348 

389348 
389348 
species
111
 389348 
121

334334
order
51291
27591313610

27591313610
809
family
334334

810
334334
genus
27591313610


83554 


83554 
species
11
 83554 
11


1457153 
1
 1457153 
species
1

1
 83559 
species
1


83559 


813 
813 
813 
813 
813 
813 
2696101145
 813 
111111
species

 85991 
11
species
11


85991 


85991 

11111
species
 83558 
42113

83558 
83558 
83558 
83558 

83558 


83560 

83560 

83560 
211
species
111
 83560 

phylum
111
456828
131

456826
111
genus
131

131
 456827 
111
species


456827 
456827 
456827 

111
phylum
111
67814

693071
111
class
111

111
order
111
693072

111
693073
family
111

111
693074
111
genus

111
species
111
 693075 


693075 
693075 
693075 

1930617
phylum
111111
3108912

3108912
class
111111
1962850

3108912
111111
order
1962852

family
111111
1962854
3108912

genus
111111
187144
3108912


187145 
187145 
187145 
187145 
187145 
187145 
 187145 
111111
species
3108912

355216249310285268
phylum
1239
25451427983186511855864003

1930845
1111
genus
12101

 1871025 
species
1111
12101

1871025 

1871025 

1871025 
1871025 

909932
class
151012131111
674569731015493

554885242
1843488
212121
order

554885242
909930
212121
family

904
212121
genus
554885242

 905 
111111
species
514084232

905 
905 
905 
905 
905 
905 


187327 

187327 

187327 
 187327 
111
species
40811

1843489
535545
order
7562349493370

7562349493370
31977
535545
family

genus
111111
909928
121010171315

121010171315
species
111111
 1702287 

1702287 
1702287 
1702287 
1702287 
1702287 
1702287 

13114715
39948
212112
genus


39950 
39950 
39950 


39950 
 39950 
1111
species
110131


2161821 

2161821 
2161821 
2161821 
2161821 
211714
species
11111
 2161821 

6091230181243
906
111111
genus


907 
907 
907 
907 
907 
907 
6091230181243
111111
species
 907 

11211
genus
29465
45777

species
1
 248315 
1


248315 

11111
species
 29466 
45677

29466 

29466 
29466 
29466 
29466 

865755
order
909929
4413819501721

family
664654
1843491
4323816451720

519
158846
genus
111

519
 158847 
111
species

158847 
158847 

158847 

genus
554554
970
4273716361720

 1884263 
species
111111
1212142

1884263 
1884263 
1884263 
1884263 
1884263 
1884263 


971 
971 
971 
971 
971 
971 
111111
species
 971 
742656

species
111111
 713030 
415825

713030 
713030 
713030 
713030 
713030 
713030 

4012971227
111111
species
 69823 

69823 
69823 
69823 
69823 
69823 
69823 

1111
species
 712538 
3294

712538 
712538 

712538 
712538 

9351
1843490
family
2111

2111
genus
365348
9351

41
species
11
 484770 

484770 


484770 


365349 

365349 
365349 
 365349 
species
111
535

class
217146158212196187
91061
2456617311432323040722279

1369597132129117
order
1385
1235113691003253134551834

332730343227
family
186822
2213248179281266246

3032854
genus
322322
55080


1465 


1465 
 1465 
11
species
51

2411522
 54913 
species
111111

54913 
54913 
54913 
54913 
54913 
54913 


1393 
1393 
1393 
1393 
1393 
1393 
species
111111
 1393 
121232

402371
55079
genus
11121


1450761 
3
1
species
 1450761 


1500254 
1500254 
1500254 
1500254 

1500254 
402341
species
11111
 1500254 

76632
111111
genus
10524252743


377615 
377615 
377615 
377615 
377615 
377615 
10524252743
111111
species
 377615 

genus
282326282923
44249
2133238150241234198


162209 
162209 
162209 
162209 
162209 
162209 
 162209 
species
111111
2116118

species
11111
 1695218 
287251

1695218 

1695218 
1695218 
1695218 
1695218 

2113132
 365617 
species
111111

365617 
365617 
365617 
365617 
365617 
365617 


1536770 
1536770 


1536770 
132
 1536770 
111
species


528191 
528191 
528191 
528191 
528191 
528191 
species
111111
 528191 
1614915797

1111
species
 414771 
3161


414771 
414771 
414771 
414771 


61624 
61624 
61624 
61624 
61624 
61624 
 61624 
111111
species
61103527673102


1870819 


1870819 
1870819 
 1870819 
111
species
125

154423
2044880
11111
species group


483937 
483937 
483937 
483937 
483937 
 483937 
11111
species
154423

species
111111
 189426 
1058512

189426 
189426 
189426 
189426 
189426 
189426 


1536769 
1536769 
1536769 
1536769 
1536769 
1536769 
 1536769 
species
111111
7574526


1536773 
1536773 
1536773 
1536773 
1536773 
 1536773 
species
11111
462347


1178515 
1178515 
1178515 
1178515 
1178515 
1178515 
111111
species
 1178515 
1231411


324057 


324057 
324057 
324057 
6311
 324057 
species
1111

 1536775 
1
species
4


1536775 

6162810
 1536772 
111111
species

1536772 
1536772 
1536772 
1536772 
1536772 
1536772 


1126833 
species
1
 1126833 
1

 44251 
species
111111
8524172

44251 
44251 
44251 
44251 
44251 
44251 


1536774 
1536774 
1536774 
1536774 
1536774 
1536774 
134335
 1536774 
species
111111

 1870820 
species
11111
101212102

1870820 

1870820 
1870820 
1870820 
1870820 

111111
species
 1616788 
629116234

1616788 
1616788 
1616788 
1616788 
1616788 
1616788 

238
 169760 
111
species


169760 
169760 
169760 


1532905 
1532905 
1532905 

1532905 
1532905 
52939
 1532905 
11111
species

3
species
1
 2069255 


2069255 

13
 1536771 
11
species


1536771 
1536771 


1462996 
1462996 
1462996 
1462996 
1462996 
13144
 1462996 
species
11111

2131
 172713 
1111
species

172713 
172713 

172713 
172713 


160799 

160799 

160799 
 160799 
111
species
154

species
111
 159743 
211

159743 

159743 
159743 


59893 
 59893 
1
species
1

14112
 481743 
species
11111

481743 

481743 
481743 
481743 
481743 

123873719
 1406 
species
111111

1406 
1406 
1406 
1406 
1406 
1406 

11111
species
 189425 
321410

189425 

189425 
189425 
189425 
189425 

12
11
species
 1763538 

1763538 


1763538 

11111
species
 1566358 
32132

1566358 
1566358 
1566358 
1566358 

1566358 

203212
species
11111
 1464 

1464 

1464 
1464 
1464 
1464 

36161648
186824
family
211222

1677050
11111
genus
1916136

 1471761 
11111
species
1916136

1471761 
1471761 

1471761 
1471761 
1471761 

genus
11111
292635
171512


2071623 

2071623 
2071623 
2071623 
2071623 
11111
species
 2071623 
171512

2
1378
2
genus


29391 
1
species
 29391 
1


1785995 
1
 1785995 
1
species

5643238322517
186818
101071377
family

9141316108
1372
genus
653643

species
11
 2213202 
11

2213202 

2213202 

21
 161360 
11
species


161360 

161360 

species
1
 1526927 
1

1526927 

 2058136 
11111
species
21131

2058136 
2058136 
2058136 
2058136 
2058136 


1038856 
1038856 
1038856 
1038856 
1038856 
1038856 
2911363
species
111111
 1038856 


200991 
200991 

200991 
200991 
200991 
 200991 
11111
species
11613

 1374 
11
species
12


1374 

1374 


192421 
192421 

192421 
192421 
 192421 
1111
species
2122

21011
genus
1111
1649


1750719 
1750719 
1750719 

1750719 
21011
 1750719 
species
1111

1147321
648800
genus
22121

 2048654 
111
species
1311

2048654 
2048654 

2048654 

 76853 
11111
species
1016311

76853 
76853 
76853 
76853 

76853 

82
genus
11
157226


1508404 

1508404 
species
11
 1508404 
82

1569
genus
221422
4419413137


1571 
1571 
1571 
1571 
1571 
1571 
108410106
 1571 
111111
species

131
species
111
 1930546 


1930546 
1930546 
1930546 


1476 
1
species
1
 1476 


1930764 
1930764 

1930764 
43111
111
species
 1930764 

186823
444544
family
8972533419255

genus
111211
1129704
112517


33943 
33943 
33943 
33943 
33943 
33943 
112317
111111
species
 33943 


2055160 
 2055160 
1
species
2

genus
111111
29330
14128134815


405212 
405212 
405212 
405212 
405212 
405212 
14128134815
111111
species
 405212 

7552223234333
222222
genus
432330


1903704 
1903704 
1903704 
1903704 
1903704 
1903704 
111111
species
 1903704 
69717159928

 1214604 
111111
species
585814345

1214604 
1214604 
1214604 
1214604 
1214604 
1214604 

9810219343125
321322
family
186820

69231
2755
genus
1111


2756 
2756 

2756 
2756 
 2756 
1111
species
69231

211212
genus
1637
2910019313025

111111
species
 1639 
2410019263024

1639 
1639 
1639 
1639 
1639 
1639 

551
 1638 
111
species

1638 


1638 

1638 

211314232424
family
90964
4321317712551999564

2005363
11
genus
11


1849491 
1849491 
11
 1849491 
species
11

69965
genus
232
362

species
111
 69966 
121

69966 

69966 


69966 


1855823 


1855823 
species
11
 1855823 
31


1898474 

1898474 
21
 1898474 
11
species

17
45669
genus
1

17
 407035 
species
1

407035 

4111307112551999562
genus
171211232422
1279

 70255 
111
species
221


70255 
70255 
70255 

 985002 
11
species
11


985002 


985002 

4315913622160
 1280 
111111
species

1280 
1280 
1280 
1280 
1280 
1280 


1281 

1281 
species
11
 1281 
11


1295 

1295 
1295 
1295 
17121
species
1111
 1295 


1286 


1286 
1286 
1286 
 1286 
species
1111
4173

species
111111
 29388 
23958357831199299

29388 
29388 
29388 
29388 
29388 
29388 


1290 
1290 

1290 
1290 
1290 
55153238
11111
species
 1290 


643214 


643214 
11
 643214 
species
11


1282 
1282 
1282 
1282 
1282 
1282 
111111
species
 1282 
524118202354116

3112617
11111
species
 45972 

45972 
45972 

45972 
45972 
45972 

 283734 
species
111
2442


283734 
283734 
283734 

51113255
 1292 
species
111111

1292 
1292 
1292 
1292 
1292 
1292 


308354 
 308354 
species
1
2


1283 
1283 

1283 
1283 
1283 
213132916
11111
species
 1283 


1294 
species
1
 1294 
1

12
 1715860 
species
11

1715860 


1715860 


61015 
61015 
61015 
 61015 
111
species
124


155085 


155085 
 155085 
species
11
71


1284 
species
1
 1284 
1


29384 
 29384 
1
species
1

 214473 
species
1111
1551

214473 


214473 
214473 
214473 

 1288 
species
111111
511552

1288 
1288 
1288 
1288 
1288 
1288 


29385 
29385 
29385 
29385 
29385 
29385 
 29385 
111111
species
4127131


29379 
29379 
29379 
 29379 
species
111
113


1296 

1296 
species
11
 1296 
21


29382 
29382 
29382 
species
111
 29382 
941

 46127 
1
species
1


46127 

2214143
 28035 
species
11111

28035 
28035 

28035 
28035 
28035 

11111
species
 246432 
11481

246432 
246432 

246432 
246432 
246432 


170573 

170573 

170573 
170573 
1121
 170573 
1111
species

271228153179258221
33986
666666
genus


360911 
360911 
360911 
360911 
360911 
360911 
382637
 360911 
species
111111

21912484100160141
 332410 
species
111111

332410 
332410 
332410 
332410 
332410 
332410 

113423304334
species
111111
 132920 

132920 
132920 
132920 
132920 
132920 
132920 

111111
species
 1224749 
172015162115

1224749 
1224749 
1224749 
1224749 
1224749 
1224749 


1399115 
1399115 
1399115 
1399115 
1399115 
1399115 
141813121616
111111
species
 1399115 

species
111111
 1849031 
7241615158

1849031 
1849031 
1849031 
1849031 
1849031 
1849031 

7840587503703778698
186817
family
573234465045

2
genus
84406
2


403957 
 403957 
species
1
1


163877 
1
 163877 
species
1

131282
11111
genus
1906945

 1426 
species
11111
131282

1426 
1426 

1426 
1426 
1426 

11
genus
200903
14


2213194 


2213194 
14
11
species
 2213194 

332712
1329200
211
genus


1221500 

1221500 
41
11
species
 1221500 

species
11
 255247 
33232

255247 


255247 

14992053494459
129337
genus
114912711

species
1
 544556 
4


544556 

111
species
111
 471223 

471223 

471223 


471223 


1422 

1422 
1422 
1422 
1422 
 1422 
11111
species
444410


33940 

33940 
33940 
33940 
33940 
11111
species
 33940 
1356113147

species
111
 1921421 
541

1921421 


1921421 

1921421 

111
species
 1233873 
812

1233873 

1233873 
1233873 

species group
333433
1505648
1221933212234

11
species
11
 1462 


1462 

1462 


33938 
33938 
33938 
33938 
33938 
33938 
6109347
111111
species
 33938 

species
111111
 169283 
1381021021

169283 
169283 
169283 
169283 
169283 
169283 

103141586
 33941 
species
11111

33941 

33941 
33941 
33941 
33941 

1731
1111
species
 550542 


550542 
550542 
550542 
550542 

122
 691437 
species
111

691437 


691437 

691437 

111111
species
 129338 
112211

129338 
129338 
129338 
129338 
129338 
129338 

12
 581103 
11
species

581103 


581103 

8340101
150247
genus
33141


1490057 
2
1
species
 1490057 

 1490052 
species
11
11


1490052 


1490052 

112
 198467 
111
species

198467 
198467 


198467 

5
 294699 
species
1


294699 


33934 
33934 

33934 
33934 
33934 
514021
 33934 
species
11111

3164
genus
1111
1055323

3164
species
1111
 33936 

33936 
33936 
33936 
33936 

3
29331
genus
1

3
species
1
 1449 

1449 

1386
312020243029
genus
2167553438527703625

11
 666686 
species
11

666686 


666686 

 300825 
species
1
1


300825 


1478 


1478 
1478 
1478 
806241
 1478 
1111
species

111
species
 561879 
111

561879 

561879 


561879 

11
species
11
 1479 

1479 


1479 


35841 
35841 
35841 
35841 
1122
 35841 
species
1111


79883 
79883 


79883 
79883 
2111
1111
species
 79883 


1837130 
species
1
 1837130 
1


412384 
1
species
 412384 
1

species group
555454
86661
36262461278066

49237562714
 1396 
111111
species

1396 
1396 
1396 
1396 
1396 
1396 

 1405 
species
111111
57413711

1405 
1405 
1405 
1405 
1405 
1405 


1392 
1392 
1392 
1392 
1392 
1392 
29752149
 1392 
species
111111

 580165 
1111
species
265121

580165 
580165 
580165 

580165 


1428 
1428 
1428 
1428 
1428 
1428 
 1428 
111111
species
142428563132

1
species
 33932 
3

33932 


1404 
1404 
1404 
1404 
1404 
1404 
 1404 
species
111111
291273209262452281


632773 
632773 
632773 

632773 
632773 
11111
species
 632773 
381111

 79880 
species
111
431

79880 
79880 


79880 

1
species
 859143 
1


859143 


658666 
1
species
 658666 
1


199441 


199441 
11
species
 199441 
72


1856406 
2
species
1
 1856406 

111
111
species
 1892404 


1892404 

1892404 

1892404 

 1413 
species
11
157


1413 

1413 


1547283 
 1547283 
species
1
1

231
 324767 
species
111


324767 
324767 
324767 

270148115113124141
653685
855696
species group

444761668442
1938374
322232
species subgroup

12
11
species
 659243 

659243 


659243 

282246587228
111111
species
 492670 

492670 
492670 
492670 
492670 
492670 
492670 


1390 
1390 
1390 
1390 
1390 
1390 
 1390 
species
111111
15251581014


1423 
1423 
1423 
1423 
1423 
1423 
1999245231190
 1423 
111111
species


72361 


72361 
72361 
72361 
 72361 
1111
species
2112


1648923 
1648923 
1648923 
1648923 
1648923 
1648923 
5461185
species
111111
 1648923 

species
11
 119858 
32

119858 


119858 


1452 
 1452 
species
1
4


1402 
1402 
1402 
1402 
1402 
1402 
175312142
 1402 
111111
species


1664069 


1664069 
11
 1664069 
species
11

11
11
species
 98228 

98228 


98228 


1127744 


1127744 
12
 1127744 
species
11


2093834 
1
 2093834 
species
1

 1565991 
1
species
1


1565991 


2009331 
1
species
1
 2009331 

1
1792192
species group
1

 293387 
1
species
1


293387 

1
 665099 
species
1


665099 


2049935 
 2049935 
species
1
1


1467 


1467 
1467 
1467 
 1467 
species
1111
9111

 264697 
11
species
111

264697 


264697 


1408 
1408 

1408 

1408 
1131
 1408 
1111
species

species
111
 1446792 
281


1446792 

1446792 
1446792 


1398 
1398 
1398 
1398 
1398 
1398 
species
111111
 1398 
356605781863


79885 
1
species
 79885 
1


1774743 
3
species
1
 1774743 


1839798 
1839798 
11
species
11
 1839798 


1471 
 1471 
1
species
1

genus
1111
351195
56111

56111
 1230341 
1111
species

1230341 
1230341 

1230341 

1230341 

11
45667
genus
11


1570 


1570 
11
 1570 
11
species

7628575710
400634
423432
genus

12311
 2169540 
species
11111

2169540 

2169540 
2169540 
2169540 
2169540 


1421 
1421 
1421 
1421 
1421 
1421 
69451359
species
111111
 1421 


2072025 


2072025 
5768
 2072025 
11
species


1145276 
1
1
species
 1145276 

10321
1111
species
 28031 

28031 
28031 
28031 

28031 

11
182709
11
genus


182710 
182710 
11
species
 182710 
11

order
815161806770
186826
12215362429699617445


1993866 
1993866 
 1993866 
species
11
11

41177134383362220
271821332928
family
1300

16104061758
1357
213112
genus


1363 
2
 1363 
species
1

1
species
1
 1366 


1366 

14103761757
 1358 
111111
species

1358 
1358 
1358 
1358 
1358 
1358 

 1364 
11
species
21

1364 


1364 

3956794377345162
1301
genus
251718322826

97421
 1309 
1111
species

1309 


1309 
1309 
1309 


1310 
1310 
1310 

1310 
1310 
 1310 
species
11111
622111


315405 


315405 
226
 315405 
species
11


45634 
45634 
45634 
45634 
45634 
11222
 45634 
11111
species

91215611917
 1314 
111111
species

1314 
1314 
1314 
1314 
1314 
1314 


712633 


712633 
712633 
712633 
2565
 712633 
1111
species

671232
species group
211331
41331273

 1328 
11
species
43


1328 
1328 


1338 
1338 
1338 
1338 
1338 
1338 
4033733
111111
species
 1338 


76860 


76860 
76860 
111
111
species
 76860 


113107 
113107 
113107 
873
111
species
 113107 

 197614 
11
species
282

197614 

197614 

5141043
12212
species group
119603

2341
 1336 
1111
species


1336 
1336 
1336 
1336 

 1334 
1111
species
51272

1334 

1334 
1334 

1334 

137183
11111
species
 1307 


1307 
1307 
1307 
1307 
1307 


1348 
1
species
 1348 
32


28037 
28037 
28037 
28037 
28037 
28037 
 28037 
111111
species
41510124


1811193 
1
1
species
 1811193 

 1313 
species
111111
3113177912229

1313 
1313 
1313 
1313 
1313 
1313 


257758 
257758 


257758 
257758 
 257758 
1111
species
1121


1304 
1304 
1304 
5176
species
111
 1304 

12182
1111
species
 1902136 

1902136 


1902136 
1902136 
1902136 

11111
species
 1303 
119108

1303 
1303 

1303 
1303 
1303 


1305 
1305 
1305 
1305 
1305 
1305 
111111
species
 1305 
724221712

 1839799 
1
species
2


1839799 


1308 
1308 
1308 
1308 
1308 
1308 
9128121319
 1308 
species
111111


150055 
150055 
150055 
150055 
150055 
11111
species
 150055 
11112


1825069 
1
species
 1825069 
23


102684 
102684 
102684 
102684 
102684 
102684 
14421342
species
111111
 102684 


1814128 


1814128 
21
 1814128 
species
11


1302 
1302 
1302 
1302 
1302 
1302 
54111115
 1302 
species
111111


1759399 
1759399 
1759399 
8103
111
species
 1759399 


1318 
1318 
1318 
1318 
1318 
32647
11111
species
 1318 

111
species
 1326 
3911

1326 


1326 
1326 


1156431 
1156431 
1156431 
1156431 
1156431 
1156431 
111111
species
 1156431 
22181510

211178
1111
species
 1156433 

1156433 


1156433 
1156433 
1156433 

 1349 
1
species
1


1349 


1311 
1311 
1311 
1311 
1311 
1311 
7414856
species
111111
 1311 

81852
family
781010910
5799119988361

2737
111
genus
111

1
 633807 
1
species


633807 


519472 
519472 
11
 519472 
11
species

21
11
genus
51668

21
species
11
 51669 


51669 

51669 

1
33969
genus
1


33970 
 33970 
1
species
1

5799117958260
1350
788889
genus


2060307 
2060307 
2060307 
2060307 
2060307 
2060307 
17192491916
species
111111
 2060307 


1353 
1353 
1353 
1353 
1353 
1353 
species
111111
 1353 
8141820138

 417368 
111
species
112


417368 
417368 
417368 

3
 53346 
species
1


53346 


53345 


53345 
11
 53345 
11
species

 2057791 
111111
species
31085134

2057791 
2057791 
2057791 
2057791 
2057791 
2057791 


1354 
1354 
11
species
 1354 
31

11
species
 2005703 
11


2005703 


2005703 

322
 44008 
111
species

44008 

44008 


44008 

species
111111
 1352 
11292429106

1352 
1352 
1352 
1352 
1352 
1352 


1351 

1351 
1351 
1351 
1351 
11111
species
 1351 
510354

111111
species
 37734 
102230251918

37734 
37734 
37734 
37734 
37734 
37734 

family
312322
186827
734638

734638
1375
genus
312322


128944 
7
 128944 
species
1

 51665 
species
11111
23342

51665 
51665 
51665 
51665 
51665 

species
1111
 1376 
2111

1376 

1376 

1376 
1376 

 87541 
species
1
1


87541 


1377 


1377 
31
 1377 
11
species

9281711182428
186828
422211
family

422211
genus
2747
9281711182428


1564681 
1564681 
1564681 
83911
 1564681 
111
species

111111
species
 2751 
81610172428

2751 
2751 
2751 
2751 
2751 
2751 


147709 


147709 
 147709 
species
11
111

70
 208596 
1
species

208596 

81850
family
1266675
537481947238

11
genus
1
46254

11
 1247 
1
species

1247 

270161234195
744442
genus
1243


979982 

979982 
979982 
13611
species
111
 979982 

4
 1246 
1
species

1246 

 1245 
species
111111
1054281763

1245 
1245 
1245 
1245 
1245 
1245 

species
11111
 33964 
21133

33964 
33964 
33964 
33964 
33964 

11
species
 1511761 
12


1511761 


1511761 


1252 
1252 
232
 1252 
11
species

147921382
species
11111
 1244 

1244 

1244 
1244 
1244 
1244 

3
1
species
 136609 

136609 

46255
genus
422233
2662271343

18
1
species
 1631871 

1631871 


1249 
1
 1249 
species
1

21
species
11
 165096 

165096 


165096 

species
111111
 759620 
2013121

759620 
759620 
759620 
759620 
759620 
759620 


137591 
137591 
137591 
137591 
137591 
137591 
species
111111
 137591 
2622141211

family
281620261823
33958
5438158142147121119

genus
271519231621
1578
5405157141144117117

 1579 
species
111111
224111

1579 
1579 
1579 
1579 
1579 
1579 

 1545702 
1
species
2


1545702 


1218493 
1218493 
species
11
 1218493 
21


1604 

1604 

1604 
112
species
111
 1604 


267363 
1
species
1
 267363 


1601 
species
1
 1601 
1


1581 

1581 
1581 
 1581 
species
111
811

 1584 
species
111111
273122413244

1584 
1584 
1584 
1584 
1584 
1584 

species
11
 1623 
22

1623 


1623 

 52242 
species
1
1


52242 

10814212
 1624 
11111
species

1624 

1624 
1624 
1624 
1624 

1
species
 148814 
1

148814 

2112
species
111
 47715 

47715 


47715 
47715 

 1596 
11
species
31


1596 
1596 


53444 
1
species
 53444 
2


1580 
1580 
1580 
1580 
1580 
1580 
111111
species
 1580 
29541366

258782833
111111
species
 1590 

1590 
1590 
1590 
1590 
1590 
1590 


392416 
1
 392416 
species
1

1
 1612 
species
1


1612 


375175 

375175 
375175 
111
species
 375175 
236


1603 
 1603 
1
species
1

species
111
 637971 
3101


637971 
637971 
637971 


1599 
1599 
1599 
1599 
1599 
1599 
6833057435346
111111
species
 1599 


1613 

1613 
1613 
1613 
1613 
373742
 1613 
species
11111


1598 

1598 
1598 

1598 
108212
 1598 
1111
species


1218494 


1218494 
 1218494 
11
species
21

1118
 240427 
species
1111

240427 
240427 

240427 

240427 

 1602 
species
11
31

1602 


1602 


97478 

97478 
97478 

97478 
species
1111
 97478 
1371

 2099788 
11
species
11


2099788 


2099788 


28038 

28038 
28038 
28038 
28038 
11111
species
 28038 
29691612

 267818 
1
species
1


267818 


1587 
1587 


1587 
1587 
2122
species
1111
 1587 

species
1
 1622 
16

1622 


303541 
303541 
14
species
11
 303541 

151459819
655183
species group
122112


1597 
1597 
1597 
1597 
1597 
1597 
111111
species
 1597 
151249817


1582 
1582 


1582 
 1582 
species
111
212

1
 83683 
species
1


83683 

 1610 
111
species
421

1610 

1610 

1610 

72373
species
11111
 33959 

33959 
33959 

33959 
33959 
33959 


1847728 


1847728 
11
11
species
 1847728 

3311342
1253
111322
genus


51663 

51663 
11
species
11
 51663 

11
species
 114090 
12


114090 
114090 


1255 
1255 
1255 
1255 
1255 
1255 
3311121
 1255 
111111
species

172715326
1676648
class
111111

1676649
111111
order
172715326

172715326
1676650
family
111111

111111
genus
1676651
172715326


1555112 
1555112 
1555112 
1555112 
1555112 
1555112 
 1555112 
species
111111
172715326

1737404
class
412333
2212964

1737405
order
412333
2212964

1
family
2042895
1

1
genus
1
1505664


1556 
species
1
 1556 
1

1570339
412332
family
2212963

165779
2112
genus
2123


1870984 

1870984 

1870984 
species
111
 1870984 
111


33034 


33034 
33034 
122
111
species
 33034 

genus
1
543311
1


33033 
1
 33033 
1
species

genus
111
162289
1911


1912856 


1912856 

1912856 
111
species
 1912856 
1911

11632
11111
genus
150022

11111
species
 1260 
11632


1260 
1260 
1260 
1260 
1260 

2131579881662155714371593
class
1125573777062
186801

68281044
53433
order
222332

1211
family
972
31212

31212
genus
1211
2330


656519 
656519 


656519 
species
111
 656519 
3112

 2331 
11
species
11


2331 

2331 

family
12222
53434
378924

1111
1111
genus
42417

1111
 42422 
species
1111


42422 
42422 
42422 
42422 

377813
28186
genus
11111

377813
11111
species
 28187 

28187 

28187 
28187 
28187 
28187 

186802
944659645754
order
2039139231622149713971543

158111077
family
186807
1652232427926

56112
genus
112
115

12
 1147129 
species
11


1147129 

1147129 


55583 


55583 
 55583 
species
11
13

51328
3113
genus
36853


49338 
49338 
49338 
111
species
 49338 
324


233055 


233055 
species
11
 233055 
441

33
11
species
 36854 

36854 


36854 


142877 
 142877 
species
1
4

75131
1562
4121
genus

 1564 
species
111
6221

1564 

1564 
1564 


59610 
2
 59610 
1
species


1833852 
9
1
species
 1833852 

species
111
 1565 
211

1565 
1565 
1565 

45012138219
genus
323222
79206


1563 
1563 
1563 
1563 
1563 
1563 
 1563 
111111
species
91096117


79209 

79209 
79209 
79209 
1111
species
 79209 
6221

 885581 
1111
species
435222

885581 
885581 
885581 


885581 

51514
genus
111
212

212
species
111
 51515 

51515 


51515 
51515 

1111
genus
278993
282132

282132
 863643 
species
1111

863643 

863643 
863643 

863643 

322
111
genus
471826

 471827 
species
111
322


471827 
471827 

471827 

2692111
2282742
11111
genus


58138 
58138 
58138 

58138 
58138 
species
11111
 58138 
2692111

111
genus
51196
111

111
species
111
 51197 


51197 

51197 
51197 

genus
111111
2282740
52211221

species
111111
 102134 
52211221

102134 
102134 
102134 
102134 
102134 
102134 

82911481408192155
family
17913151011
186803

7611112
11111
genus
698776


29360 

29360 
29360 
29360 
29360 
 29360 
species
11111
7611112

111
species
 712991 
35431

712991 


712991 

712991 

5722111
1663717
genus
11111


1679721 
1679721 
1679721 
1679721 
1679721 
5722111
 1679721 
species
11111

7822
21
genus
830

642
 185008 
1
species

185008 


43305 


43305 
 43305 
11
species
1402

566141554341922
572511
323322
genus


1796616 
1796616 
1796616 
1796616 
1796616 
1796616 
species
111111
 1796616 
521171451201718

 1912897 
species
111111
4437121324

1912897 
1912897 
1912897 
1912897 
1912897 
1912897 

 1322 
species
111
6011

1322 

1322 
1322 

13943111
11111
genus
841


301301 

301301 
301301 
301301 
301301 
13943111
 301301 
species
11111


39491 
39491 
39491 

39491 
1922321
 39491 
species
1111

161
11
genus
2039240

161
species
11
 28446 

28446 


28446 

1316141784372
genus
111111
207244

 649756 
111111
species
1316141784372

649756 
649756 
649756 
649756 
649756 
649756 

191801462302657
genus
545535
1506553


1871021 
1871021 
1871021 
1871021 

1871021 
species
11111
 1871021 
21962111

643614418
species
11111
 66219 

66219 

66219 
66219 
66219 
66219 


208479 
208479 
208479 
208479 
208479 
208479 
 208479 
111111
species
429017834


84030 
84030 
84030 
84030 
84030 
84030 
 84030 
species
111111
372493782241


1834196 
1834196 
1834196 
1834196 

1834196 
25342393
species
11111
 1834196 

433333
family
186806
9851915202112

1331125
genus
11111
33951

 33952 
species
11111
1331125

33952 

33952 
33952 
33952 
33952 

971891419197
1730
genus
332222


39488 
39488 
39488 
39488 
39488 
39488 
954847543
 39488 
111111
species


1736 
1736 
1736 
1736 
1736 
1736 
 1736 
111111
species
1634714154

11
species
 39485 
71

39485 
39485 

352143256
111111
genus
946234


292800 
292800 
292800 
292800 
292800 
292800 
 292800 
species
111111
352143256

111111
family
216572
1523412201552

1523412201552
111111
genus
459786

1523412201552
species
111111
 351091 

351091 
351091 
351091 
351091 
351091 
351091 

671092918
111111
family
543349

genus
111111
2733
671092918


2734 
2734 
2734 
2734 
2734 
2734 
species
111111
 2734 
671092918

family
2111
68298
3111

2
129001
1
genus

1
species
 86170 
2

86170 

genus
1111
862
1111


863 
863 
863 


863 
1111
1111
species
 863 

101219341522
539000
111111
family

genus
111111
73918
101219341522

101219341522
species
111111
 73919 

73919 
73919 
73919 
73919 
73919 
73919 

21136427
31984
111111
family

21136427
genus
111111
2697


35701 
35701 
35701 
35701 
35701 
35701 
species
111111
 35701 
21136427

17305162774
family
422211
186804

93
1849828
1
genus

93
 1505 
1
species

1505 

12
1481960
1
genus


1731 
12
species
1
 1731 

811
111
genus
44259


143361 
143361 
143361 
species
111
 143361 
811

1617415674
1870884
111111
genus

111111
species
 1496 
1617415674

1496 
1496 
1496 
1496 
1496 
1496 


2081703 
21
1
species
 2081703 

291014191815
family
31979
27236439974922782880

1485
genus
22910141612
25255438965914775869

species
1111
 1497 
1874125

1497 
1497 


1497 
1497 


332101 
species
1
 332101 
33


1513 
1513 

1513 
1513 
 1513 
1111
species
1111


1534 
1534 
1534 
40021
 1534 
111
species


1491 
1491 
1491 
1491 
1491 
1491 
111111
species
 1491 
13934414925836724793


1561 
1561 
11
 1561 
species
11

34141972623
 1501 
111111
species

1501 
1501 
1501 
1501 
1501 
1501 

211
 238834 
111
species

238834 


238834 
238834 

11
species
 1488 
201

1488 


1488 

111
species
111
 1509 


1509 
1509 
1509 


29341 


29341 
 29341 
11
species
51

427224232
111111
species
 755731 

755731 
755731 
755731 
755731 
755731 
755731 

56766432
11111
species
 1502 

1502 

1502 
1502 
1502 
1502 

13401
 84023 
11
species

84023 


84023 


1548 
1
species
 1548 
3


1492 
1492 
1492 
1492 
1492 
1492 
 1492 
111111
species
474122115


1520 

1520 
1520 
1520 
401222
1111
species
 1520 


1493 


1493 
61
 1493 
11
species

5222154
111111
species
 169679 

169679 
169679 
169679 
169679 
169679 
169679 


394958 


394958 
11
species
 394958 
12231

 46867 
species
11
11

46867 


46867 

 217159 
111
species
29221

217159 

217159 


217159 

 1216932 
11
species
2141

1216932 


1216932 

species
11111
 1042156 
1321211

1042156 
1042156 
1042156 
1042156 
1042156 

 84022 
1
species
8

84022 

2111
genus
114627
663212


208226 
species
1
 208226 
2

 461876 
1111
species
661212

461876 

461876 
461876 

461876 

11
species
 2082193 
242

2082193 


2082193 

2711236
11111
genus
1981033


2086584 
2086584 
2086584 
2086584 
2086584 
2711236
 2086584 
11111
species

1015111
49082
genus
2111


1508644 

1508644 
6811
11
species
 1508644 


49118 


49118 

49118 
33411
species
111
 49118 

84118
genus
11111
390805

84118
 1424294 
11111
species

1424294 

1424294 
1424294 
1424294 
1424294 

592343447544556
541000
1457698
family

genus
1
1890281
11


1677857 
11
 1677857 
species
1

genus
2111
1263
2466112


1264 
1264 
21321
 1264 
species
11


1160721 

1160721 

1160721 
33412
 1160721 
species
111

159282021727
216851
genus
111111


853 
853 
853 
853 
853 
853 
 853 
species
111111
159282021727

236752
genus
11
11


236753 


236753 
 236753 
species
11
11

species
11
 1572656 
1422

1572656 


1572656 

1
genus
1637257
1


884684 
1
species
1
 884684 

545142123282621
genus
724454
1508657


1521 

1521 
1521 
 1521 
111
species
1321

species
11111
 1510 
9037553

1510 
1510 

1510 
1510 
1510 

502941418211116
111111
species
 1515 

1515 
1515 
1515 
1515 
1515 
1515 

1646211
1111
species
 84032 

84032 

84032 

84032 
84032 

781151
 1834198 
11111
species

1834198 

1834198 
1834198 
1834198 
1834198 


288965 
91
species
1
 288965 

14894
 29343 
11
species

29343 


29343 

50843586
genus
111111
253238

50843586
 253239 
111111
species

253239 
253239 
253239 
253239 
253239 
253239 

2405225114
1392389
genus
111111


1297617 
1297617 
1297617 
1297617 
1297617 
1297617 
2405225114
 1297617 
species
111111

11132132
543347
family
11111

11132132
genus
11111
178898


178899 
178899 
178899 
178899 
178899 
11132132
 178899 
11111
species

2408311334243396359
111111
family
990719

2408311334243396359
111111
genus
990721


1805714 
1805714 
1805714 
1805714 
1805714 
1805714 
species
111111
 1805714 
2408311334243396359

3141
family
1111
543314

3141
 143393 
1111
species

143393 


143393 
143393 
143393 

1571110106
order
68295
91476331503646

111
family
227387
111

227388
genus
111
111


184064 


184064 
184064 
species
111
 184064 
111

family
111111
543372
882744232

genus
111111
252965
882744232

882744232
 252966 
species
111111

252966 
252966 
252966 
252966 
252966 
252966 

424311
family
543371
13625322

925322
44000
224311
genus

611
 717609 
111
species

717609 

717609 
717609 

 413889 
species
1
1


413889 


31899 
31899 
11
 31899 
species
11


52766 

52766 
52766 
 52766 
species
111
311


44001 
44001 

44001 
44001 
 44001 
species
1111
1222

1
genus
291988
124


291990 
124
 291990 
1
species

28895
1
genus
3


1517 
3
 1517 
species
1

1835722443042
946574
family
186814

9119
genus
111
499228


499229 
499229 


499229 
9119
 499229 
species
111

140458
genus
111
9111

9111
 85874 
species
111

85874 
85874 


85874 

1754
513242
genus
15657228

species
1
 583357 
1

583357 


108150 
species
1
 108150 
1

11635195
species
111111
 46354 

46354 
46354 
46354 
46354 
46354 
46354 

 496866 
species
1
1

496866 

111
 399726 
111
species

399726 

399726 

399726 


573062 

573062 
 573062 
11
species
11


2325 
2325 
2325 
 2325 
111
species
213

genus
111
129957
4114

4114
species
111
 129958 

129958 

129958 
129958 

421
111
genus
42837


42838 
42838 
42838 
421
111
species
 42838 

64491221615
111111
genus
44260


1525 
1525 
1525 
1525 
1525 
1525 
64491221615
 1525 
111111
species

291
11
order
485256

291
family
11
485255

genus
11
375928
291

291
11
species
 375929 

375929 

375929 

100067820647
526524
532433
class

100067820647
order
532433
526525

100067820647
family
532433
128827

11111
genus
1729679
12151323


1702221 
1702221 
1702221 

1702221 
1702221 
12151323
 1702221 
species
11111


1834207 
1834207 

1834207 
1834207 
1834207 
 1834207 
11111
species
1820116211

191303
genus
11
7224

11
species
 1712675 
7224

1712675 


1712675 

1647
211211
genus
6899552013


1648 
1648 
1648 
1648 
1648 
1648 
 1648 
111111
species
672355513


1514105 


1514105 
species
11
 1514105 
17615

256845
phylum
111111
511891768

1313211
111111
class
511891768

511891768
111111
order
278082


2094242 
2094242 
2094242 
2094242 
2094242 
2094242 
 2094242 
species
111111
511891768

134625
phylum
111111
331311419

331311419
class
111111
1921781

331311419
order
111111
1921782

331311419
111111
family
1921783

1921784
genus
111111
331311419

 1307763 
111111
species
331311419

1307763 
1307763 
1307763 
1307763 
1307763 
1307763 

321421
1134404
11211
phylum

321421
class
11211
795747

order
11211
795748
321421

family
11
1334117
11

genus
11
1134403
11

11
 1134405 
species
11


1134405 

1134405 

32132
family
1111
795749

32132
1111
genus
795750

species
1111
 591197 
32132

591197 

591197 
591197 
591197 

111111
phylum
67819
311587207

311587207
1663419
111111
class

1663425
order
111111
311587207

311587207
1663426
family
111111

311587207
1005038
111111
genus

311587207
species
111111
 1005039 

1005039 
1005039 
1005039 
1005039 
1005039 
1005039 

31
phylum
200940
621

67799
class
31
621

621
order
31
188710

31
family
188711
621

genus
11
241192
91


171695 


171695 
 171695 
species
11
91

53
2
genus
1740

species
1
 1295609 
52

1295609 


1741 
species
1
 1741 
1

1090
11912141110
phylum
496568807546

496568807546
191410
class
11912141110

11912141110
order
191411
496568807546

496568807546
11912141110
family
191412

100715
11
genus
39


100716 


100716 
39
 100716 
11
species

55820197
genus
334434
1101

 281093 
111111
species
31211161

281093 
281093 
281093 
281093 
281093 
281093 

113322
 1974213 
111111
species

1974213 
1974213 
1974213 
1974213 
1974213 
1974213 

111
species
 1102 
131


1102 
1102 

1102 


1868325 
1868325 
1868325 
1868325 
1868325 
1868325 
132313
111111
species
 1868325 

genus
313431
1091
5710101818

species
111
 337090 
111

337090 

337090 
337090 

 1094 
111
species
111

1094 


1094 
1094 


1096 
1096 
1096 
1096 
1096 
1096 
 1096 
111111
species
37871618


1092 
1092 
1092 
111
 1092 
111
species

315042382216
256319
genus
333333


1097 
1097 
1097 
1097 
1097 
1097 
111111
species
 1097 
514642

 274537 
species
111111
173278124

274537 
274537 
274537 
274537 
274537 
274537 


274539 
274539 
274539 
274539 
274539 
274539 
 274539 
species
111111
9173124610

5383165
1099
122222
genus


1100 
1100 
1100 
1100 
1100 
1100 
527164
 1100 
species
111111


34090 
34090 
34090 
34090 
34090 
 34090 
species
11111
112101

342142
11111
phylum
65842

342142
204430
11111
class

342142
11111
order
218872

342142
family
11111
204431

genus
11111
832
342142

 833 
species
11111
342142

833 
833 
833 
833 
833 

8494191349459158235
phylum
271921211922
203691

203692
271921211922
class
8494191349459158235

8046146318387128214
136
order
211215151317

family
445466
1643685
338541515

252278
64895
genus
122233


139 
139 
139 
139 

139 
 139 
11111
species
23111


664662 
3
1
species
 664662 

 29518 
11111
species
21136


29518 
29518 
29518 
29518 
29518 

1
species
 62088 
1


62088 


64897 
species
1
 64897 
1

3133287
138
genus
323233

21112
 142 
11111
species

142 
142 
142 
142 

142 

2821123
 40834 
species
111111

40834 
40834 
40834 
40834 
40834 
40834 

 44449 
species
1
5


44449 

1112
 47466 
1111
species

47466 

47466 

47466 
47466 

8013138313383113199
1781011711
family
137

11
genus
1911556
41

11
species
 55206 
41

55206 


55206 

101610261014
399320
322321
genus


1131707 
1131707 

1131707 
211
 1131707 
111
species


273376 
273376 
273376 
273376 
273376 
273376 
111111
species
 273376 
715910514

 1131703 
1111
species
11155

1131703 

1131703 
1131703 
1131703 

797878295332103179
157
1056557
genus


167 

167 


167 
1121
 167 
111
species


160 


160 
160 
160 
 160 
species
1111
1165


221027 
1
species
 221027 
5

25
 158 
species
1

158 

 53435 
1
species
97

53435 

270121
 215591 
species
1111

215591 
215591 

215591 

215591 


1539298 
1539298 
1539298 
1539298 
1539298 
1539298 
387328531581163
111111
species
 1539298 

 150829 
species
11
110


150829 

150829 


81028 
81028 
81028 
81028 
81028 
81028 
 81028 
species
111111
6762131026

species
111111
 88058 
1223442

88058 
88058 
88058 
88058 
88058 
88058 

757111
 409322 
1111
species

409322 
409322 
409322 


409322 

14
1616789
11
genus


1307761 


1307761 
11
species
 1307761 
14

genus
21222
146
20448215


154 
154 
154 
154 

154 
 154 
species
11111
14441114


46355 

46355 
46355 

46355 
67101
 46355 
species
1111

order
234333
1643686
3531824119

234333
family
143786
3531824119

29521
234333
genus
3531824119


159 
159 
159 
159 
159 
159 
species
111111
 159 
322051765

813
 84378 
species
111


84378 
84378 
84378 

111111
species
 52584 
331423

52584 
52584 
52584 
52584 
52584 
52584 


84377 

84377 
84377 
species
111
 84377 
131

170
442332
family
4131423481912

404584327
171
genus
331221


172 
 172 
species
1
1

 174 
11
species
23


174 

174 


28183 
species
1
 28183 
1

species
11111
 173 
40228401

173 
173 
173 
173 
173 

species
111
 28452 
117


28452 


28452 
28452 

genus
111111
338321
99155175


29510 
29510 
29510 
29510 
29510 
29510 
 29510 
111111
species
99155175

11121
phylum
200930
231222

231222
68337
11121
class

231222
order
11121
191393

11121
family
191394
231222

1
genus
117999
2


118000 
1
species
 118000 
2

2321
111
genus
545865

 477976 
111
species
2321

477976 

477976 
477976 

genus
1
2351
1


2352 
1
1
species
 2352 

1
53572
1
genus

1
 197162 
species
1


197162 

471286314429408265
1297
phylum
221923242521

221923242521
class
188787
471286314429408265

361222262302334218
131214141414
order
118964

family
131113131313
183710
361221251296326213

131113131313
genus
1298
361221251296326213

 1299 
species
111111
317438505018

1299 
1299 
1299 
1299 
1299 
1299 

 2202254 
species
111111
151223274938

2202254 
2202254 
2202254 
2202254 
2202254 
2202254 

31818372013
111111
species
 502394 

502394 
502394 
502394 
502394 
502394 
502394 


2080419 
2080419 
2080419 
2080419 
2080419 
2080419 
413368217
111111
species
 2080419 

4171568
 68909 
11111
species

68909 

68909 
68909 
68909 
68909 


310783 
310783 
310783 
310783 
310783 
310783 
30552093
111111
species
 310783 

species
111111
 432329 
15320161114

432329 
432329 
432329 
432329 
432329 
432329 


1309411 
1309411 
1309411 
1309411 
1309411 
1309411 
111111
species
 1309411 
122419233


1182568 

1182568 
1182568 
1182568 
1182568 
766265
11111
species
 1182568 


1182571 
1182571 
1182571 
1182571 
1182571 
1182571 
434827274738
 1182571 
species
111111


1768108 
1768108 
1768108 
1768108 
1768108 
1768108 
 1768108 
111111
species
133931384049


55148 
55148 
55148 
55148 
55148 
55148 
111111
species
 55148 
344571557


309887 
309887 
309887 
309887 
309887 
309887 
2219181910
 309887 
species
111111

family
11111
332247
111685

11111
genus
332248
111685


332249 
332249 
332249 
332249 
332249 
11111
species
 332249 
111685

11064521277447
order
97910117
68933

11064521277447
family
97910117
188786

535674
genus
270
901731813629


37636 

37636 
37636 
37636 
16497
 37636 
1111
species


456163 
456163 
456163 
456163 
 456163 
1111
species
3945

2
1
species
 271 


271 

571514322014
 274 
species
111111

274 
274 
274 
274 
274 
274 


56956 
56956 
56956 
56956 
56956 
species
11111
 56956 
419161

1111
species
 1111069 
3915

1111069 


1111069 
1111069 
1111069 

 56957 
species
111111
1011615

56957 
56957 
56957 
56957 
56957 
56957 

208447
111111
genus
10410251714


187137 
187137 
187137 
187137 
187137 
187137 
10410251714
111111
species
 187137 

222221
genus
65551
73981331


277 
277 
277 
277 
277 
277 
species
111111
 277 
33831011

 52022 
11111
species
41532

52022 
52022 
52022 
52022 
52022 

3438183
186191
111111
genus


186192 
186192 
186192 
186192 
186192 
186192 
3438183
species
111111
 186192 

162118153185121123
phylum
976
134398116470421697419072441

174737560781424011461731
class
322533342531
768503

order
322533342531
768507
174737560781424011461731

4481081229
645424
family
563798

53231
246875
11111
genus


1727163 
1727163 
1727163 
1727163 
1727163 
 1727163 
species
11111
53231

11111
genus
232244
23261

species
11111
 232259 
23261

232259 
232259 
232259 
232259 

232259 

2212
genus
68288
7226

species
111
 320787 
215

320787 

320787 


320787 

5121
1111
species
 104 

104 

104 
104 

104 

390846
221111
genus
43442112

 1795355 
species
11
4192

1795355 
1795355 


390884 
390884 
390884 
390884 
390884 
390884 
1522112
 390884 
111111
species

family
111
1937968
131

1937972
genus
111
131

111
species
 999 
131


999 

999 

999 

808161232092644
1161212811
family
89373

7773217
105
genus
111111


106 
106 
106 
106 
106 
106 
7773217
111111
species
 106 

6151012
861914
11111
genus


1834519 

1834519 
1834519 
1834519 
1834519 
6151012
species
11111
 1834519 

1111
genus
978
5452

 985 
1111
species
5452

985 

985 
985 

985 

142
319458
genus
111

 316068 
111
species
142


316068 
316068 

316068 

120831
genus
11111
30333633


94254 

94254 
94254 
94254 
94254 
species
11111
 94254 
30333633

genus
111
2173039
312

312
 1784714 
species
111

1784714 

1784714 
1784714 

5248681371817
545545
genus
107


1211326 

1211326 
1211326 

1211326 
15210276
1111
species
 1211326 

 564064 
species
111111
11133182

564064 
564064 
564064 
564064 
564064 
564064 


1178516 
1178516 
1178516 
1178516 
1178516 
1178516 
111111
species
 1178516 
12131814

 2057025 
species
111111
3621174023

2057025 
2057025 
2057025 
2057025 
2057025 
2057025 

84152172
111111
species
 1379870 

1379870 
1379870 
1379870 
1379870 
1379870 
1379870 

111111
genus
1664383
178141221


2183547 
2183547 
2183547 
2183547 
2183547 
2183547 
 2183547 
111111
species
178141221

1501348
family
1111
1241

281119
11
genus
12

 281120 
species
11
12


281120 
281120 

41
genus
11
273135

 247481 
11
species
41


247481 

247481 

1
1853234
family
1

genus
1
59740
1


1085624 
1
species
 1085624 
1

41933659311398411151673
131213131313
family
1853232

3037711213126
1379908
323333
genus


1379910 
1379910 
1379910 
1379910 
1379910 
1379910 
 1379910 
species
111111
143033561717

137164445
species
111111
 1379909 

1379909 
1379909 
1379909 
1379909 
1379909 
1379909 


512763 

512763 
512763 
512763 
512763 
32221104
 512763 
11111
species

38129758361381810791640
888888
genus
89966

111111
species
 1850093 
34237652106187221

1850093 
1850093 
1850093 
1850093 
1850093 
1850093 

6032276943459
 1385664 
species
111111

1385664 
1385664 
1385664 
1385664 
1385664 
1385664 

15610121744871345611
111111
species
 1484116 

1484116 
1484116 
1484116 
1484116 
1484116 
1484116 

10111704143956
111111
species
 1385663 

1385663 
1385663 
1385663 
1385663 
1385663 
1385663 


1356852 
1356852 
1356852 
1356852 
1356852 
1356852 
37395761115105166
 1356852 
111111
species

21612736725193
 1446467 
111111
species

1446467 
1446467 
1446467 
1446467 
1446467 
1446467 

 1411621 
species
111111
28146191413130171

1411621 
1411621 
1411621 
1411621 
1411621 
1411621 

 1484118 
species
111111
354510322533188263

1484118 
1484118 
1484118 
1484118 
1484118 
1484118 

82244557
222222
genus
323449


388950 
388950 
388950 
388950 
388950 
388950 
71172235
 388950 
111111
species


400092 
400092 
400092 
400092 
400092 
400092 
1172322
species
111111
 400092 

200667
212311
family
7211142823

6
1
genus
869806


1006 
 1006 
species
1
6

7114162
11111
species
 1257021 

1257021 
1257021 
1257021 
1257021 
1257021 

651063
1111
genus
59739

651063
 1191459 
1111
species

1191459 

1191459 
1191459 

1191459 

1853228
655835
class
175291384413

1853229
order
655835
175291384413

family
655835
563835
175291384413

11
genus
1769012
13


1850526 


1850526 
13
 1850526 
species
11

106211813
genus
111111
354354


354356 
354356 
354356 
354356 
354356 
354356 
106211813
 354356 
111111
species

23
398041
genus
1

23
 1492898 
species
1


1492898 

222211
genus
379899
23791814


1176587 
1176587 
1176587 
1176587 
species
1111
 1176587 
34312

111111
species
 446683 
2036614

446683 
446683 
446683 
446683 
446683 
446683 

genus
11111
649460
33192183

 477680 
11111
species
33192183

477680 
477680 
477680 
477680 

477680 

1211423
79328
111212
genus


79329 
79329 
79329 
79329 
1121
 79329 
1111
species


2029983 
2029983 

2029983 

2029983 
12132
1111
species
 2029983 

117747
class
141416161114
149341141561549104112

order
141416161114
200666
149341141561549104112

84566
family
141416161114
149341141561549104112

1649482
genus
111
192


151895 
151895 

151895 
192
 151895 
species
111

555544
genus
28453
1448733411113632


1933220 
1933220 
1933220 
1933220 
1933220 
1933220 
 1933220 
111111
species
140335131188


1010 
1010 
1010 
1010 
23319
 1010 
1111
species


743722 
743722 
743722 
743722 
743722 
743722 
 743722 
species
111111
1241615

species
111111
 2003121 
2648221710

2003121 
2003121 
2003121 
2003121 
2003121 
2003121 

 1538644 
111111
species
404191553109

1538644 
1538644 
1538644 
1538644 
1538644 
1538644 

84567
555545
genus
31477933725264


363852 
363852 
363852 
363852 
363852 
363852 
258356733
 363852 
111111
species


430522 
430522 
430522 
430522 
430522 
430522 
5485931
 430522 
111111
species

3064571233957
 188932 
111111
species

188932 
188932 
188932 
188932 
188932 
188932 


984 
984 
984 
984 

984 
619342
species
11111
 984 

155148971
 1727164 
111111
species

1727164 
1727164 
1727164 
1727164 
1727164 
1727164 

11621510221111
423349
genus
223312


1550579 
1550579 
 1550579 
species
11
416

4110990118
 1300914 
species
111111

1300914 
1300914 
1300914 
1300914 
1300914 
1300914 


1234841 
1234841 
1234841 
1234841 

1234841 
11211163
species
11111
 1234841 

929509
genus
111111
15131221

 995 
species
111111
15131221

995 
995 
995 
995 
995 
995 


1986952 
1986952 
1986952 
1986952 
1986952 
1986952 
2132332
 1986952 
111111
species

1937959
122211
class
173311653

order
122211
1936988
173311653

326353
1937961
family
11111

326353
2349
genus
11111


2350 
2350 
2350 
2350 
2350 
326353
 2350 
11111
species

family
1111
89374
17153

17153
1111
genus
1007


1008 
1008 
1008 
1008 
 1008 
1111
species
17153

200643
402023322224
class
9519689306247190149

15411715
1970189
order
411313

21
1573805
11
family


1717717 


1717717 
21
 1717717 
species
11

111
family
558415
10311

1193324
genus
111
10311


889453 


889453 
889453 
10311
111
species
 889453 

4842
1471398
111
family

4842
1471399
111
genus


1168034 


1168034 

1168034 
111
species
 1168034 
4842

family
11111
1970190
11122

11111
genus
1970191
11122


1307839 
1307839 
1307839 
1307839 

1307839 
species
11111
 1307839 
11122

171549
361922292121
order
9504288305240189144

35831081221
2005525
family
232232

genus
11
375288
12


2025876 
2
 2025876 
species
1


823 
1
species
1
 823 

35821081021
195950
222222
genus

12414534
species
111111
 712710 

712710 
712710 
712710 
712710 
712710 
712710 


28112 
28112 
28112 
28112 
28112 
28112 
 28112 
111111
species
234163717

432434
family
171551
180652964519038

836
331333
genus
180642956509034


393921 
393921 

393921 
393921 
393921 
 393921 
species
11111
142112

180612156478721
111111
species
 837 

837 
837 
837 
837 
837 
837 


28123 
28123 

28123 
28123 
28123 
11111
species
 28123 
24121

1784836
1
genus
4

 1562970 
1
species
4


1562970 

111
genus
307628
181


1642646 

1642646 
1642646 
181
 1642646 
111
species

6772428149622625
1238854
family
815

6772428149622625
816
genus
1238854

45022322
11111
species
 1796613 

1796613 
1796613 
1796613 
1796613 
1796613 

11
species
 376805 
132

376805 


376805 

species
11
 47678 
52

47678 

47678 


28113 
7
species
1
 28113 


357276 


357276 

357276 
28651
111
species
 357276 


817 
817 
817 
817 
817 
817 
species
111111
 817 
617412163331821


246787 
246787 
246787 
246787 
246787 
 246787 
species
11111
695163

 818 
species
1111
81152

818 

818 
818 

818 


28116 


28116 
 28116 
species
11
1662

30777711
species
11111
 821 

821 

821 
821 
821 
821 

41
species
11
 28119 

28119 

28119 


290053 

290053 

290053 
54312
species
111
 290053 

171550
family
22222
180425276

175121203
239759
genus
11111


214856 
214856 
214856 
214856 

214856 
11111
species
 214856 
175121203

1611681
11111
genus
53473


1433126 
1433126 
1433126 
1433126 

1433126 
11111
species
 1433126 
53473

11
family
2005520
115

115
11
genus
294702


1642647 


1642647 
11
species
 1642647 
115

29261
2005523
family
111

29261
genus
111
346096

111
species
 185300 
29261

185300 


185300 
185300 

family
1111
2005473
2241110

2241110
1918540
1111
genus


1796646 

1796646 
1796646 
1796646 
1111
species
 1796646 
2241110

54302245724944
family
966776
171552

838
966776
genus
54302245724944


839 
839 
839 
839 
839 
 839 
species
11111
24151


1177574 
1177574 

1177574 
1177574 
1177574 
42333
11111
species
 1177574 

 76123 
species
1111
443111

76123 

76123 
76123 

76123 


28129 
28129 
28129 
28129 
28129 
28129 
111111
species
 28129 
1411853


589436 


589436 
2622
 589436 
species
11

 52227 
111111
species
311230231029

52227 
52227 
52227 
52227 
52227 
52227 


28132 
28132 
28132 
28132 
28132 
28132 
 28132 
species
111111
4128133


652716 
92
1
species
 652716 


28131 
28131 
28131 
28131 
28131 
28131 
 28131 
111111
species
457821024155

1111
family
1853231
389114

283168
1111
genus
389114


28118 
28118 

28118 

28118 
species
1111
 28118 
389114

71165
2005519
family
1111

71165
397864
1111
genus

1111
species
 397865 
71165

397865 

397865 
397865 

397865 

511434
genus
11
91

91
 511435 
species
11

511435 


511435 


1400053 
1400053 


1400053 
1400053 
1111
species
 1400053 
2353111

1074113514111193
1100069
order
444444

563843
444444
family
1074113514111193

60918421210
146918
111111
genus


146919 
146919 
146919 
146919 
146919 
146919 
60918421210
 146919 
111111
species

genus
111111
29548
262059433641


29549 
29549 
29549 
29549 
29549 
29549 
262059433641
111111
species
 29549 


2026787 
2026787 
2026787 
2026787 
2026787 
2026787 
 2026787 
species
111111
12740253827


1779382 
1779382 
1779382 
1779382 
1779382 
1779382 
9518312515
111111
species
 1779382 

22222483343707347340
117743
654870895544
class

22222483343707347340
654870895544
order
200644

21158397307642303313
49546
family
604464845343

1111
genus
143222
1181

 1729720 
1111
species
1181


1729720 
1729720 
1729720 
1729720 

316829714666
34084
111111
genus

316829714666
 34085 
species
111111

34085 
34085 
34085 
34085 
34085 
34085 

genus
13
326319
15


326320 
 326320 
1
species
1

2
1
species
 2173169 


2173169 

2
 313590 
1
species


313590 


983548 
1
species
1
 983548 

1016
5410994
genus
3926412241810

1111
species
 1017 
12112

1017 

1017 
1017 
1017 


1705617 
1705617 
11
species
 1705617 
12

2845112
 1945658 
species
1111

1945658 
1945658 
1945658 

1945658 

1
species
1
 1945657 


1945657 


209053 

209053 
209053 
112
 209053 
111
species


45243 
45243 
45243 
45243 
45243 
45243 
106211213
species
111111
 45243 


1848904 
1848904 
11
 1848904 
11
species


28188 
28188 
28188 
231
species
111
 28188 


1316593 
1316593 
11
species
 1316593 
21


1019 

1019 
1019 
1019 
1019 
 1019 
species
11111
61263


28189 
1
species
 28189 
10

11322
species
11111
 1316596 


1316596 
1316596 
1316596 
1316596 
1316596 

 327575 
111
species
111

327575 


327575 
327575 

1713
389486
1111
genus


1453352 

1453352 
1453352 

1453352 
1713
 1453352 
1111
species

11424
genus
11111
336276


2058135 

2058135 
2058135 
2058135 
2058135 
 2058135 
11111
species
11424

111500
genus
1
2

 516051 
1
species
2

516051 

11
genus
1247519
141


1383885 

1383885 
11
species
 1383885 
141

52223
252356
4213
genus

3
 313603 
1
species

313603 

121
 1836467 
species
111

1836467 


1836467 

1836467 

211
111
species
 1644130 

1644130 

1644130 


1644130 

 1178778 
111
species
4611

1178778 

1178778 


1178778 

1209327
111
genus
231

species
111
 1803846 
231


1803846 

1803846 
1803846 

4118
1111
genus
1649495

 1936081 
species
1111
4118

1936081 
1936081 
1936081 
1936081 

genus
1121
291183
1222


2057808 
1
species
 2057808 
2


1486034 

1486034 
11
species
 1486034 
11


983544 
983544 
 983544 
11
species
21

species
11111
 531844 
21152

531844 
531844 
531844 
531844 
531844 

57739857
genus
312321
76831

 1583100 
species
111
40033

1583100 

1583100 
1583100 

111
species
 480520 
411

480520 


480520 
480520 


1458492 
1458492 
1458492 
1458492 
1458492 
1458492 
17336447
111111
species
 1458492 

genus
111
216431
211


313588 


313588 
313588 
211
 313588 
species
111

genus
111111
252306
133112

 252307 
111111
species
133112

252307 
252307 
252307 
252307 
252307 
252307 

8
1
genus
417127


398743 
 398743 
species
1
8

225842
111211
genus
112321

1
species
 1336795 
1


1336795 

112221
 1336794 
species
111111

1336794 
1336794 
1336794 
1336794 
1336794 
1336794 

3137
286104
1111
genus

313
 1936080 
species
111

1936080 
1936080 
1936080 

species
1
 754409 
7


754409 

1637534581133840
237
genus
1389151010

673315
 1355330 
species
111111

1355330 
1355330 
1355330 
1355330 
1355330 
1355330 


312277 


312277 
11
 312277 
species
11


986 
986 
986 
986 
986 
986 
12191981311
species
111111
 986 

species
111111
 96345 
327431063

96345 
96345 
96345 
96345 
96345 
96345 

 2201181 
111
species
814

2201181 

2201181 
2201181 


1306519 


1306519 

1306519 
 1306519 
111
species
141

 2175091 
species
11111
32816

2175091 

2175091 
2175091 
2175091 
2175091 


2172098 
2172098 
61
 2172098 
11
species


996 
996 
996 
996 
996 
996 
 996 
111111
species
15744525421010


55197 
55197 
55197 
55197 
55197 
26113
11111
species
 55197 

 1763534 
111
species
121


1763534 

1763534 
1763534 


1492737 
1492737 

1492737 

1492737 
2111
 1492737 
1111
species


2183896 
2183896 
2183896 
2183896 
2183896 
2183896 
111111
species
 2183896 
14812211


2162713 


2162713 
2162713 
2162713 
101811
 2162713 
species
1111


1981981 

1981981 
1981981 

1981981 
species
1111
 1981981 
2231

178469
11
genus
11

11
species
 616991 
11


616991 


616991 

genus
11111
112040
11271


63186 
63186 
63186 
63186 
63186 
species
11111
 63186 
11271

52959
genus
112423
1251323

321
species
111
 1774273 


1774273 
1774273 

1774273 


1529069 
2
1
species
 1529069 


313598 
1
species
 313598 
1

1111
species
 996801 
1511

996801 


996801 
996801 
996801 


2058137 

2058137 
2058137 
2058137 
species
1111
 2058137 
2511

1013
111111
genus
143113184025


1014 
1014 
1014 
1014 
1014 
1014 
143113184025
species
111111
 1014 

23
genus
11
153265

11
species
 101385 
23


101385 
101385 

6115121
11241
genus
363408

 328515 
species
111
1139


328515 
328515 
328515 


2058134 

2058134 
2058134 
2058134 
6211
 2058134 
1111
species


319236 
1
1
species
 319236 


1476901 
 1476901 
1
species
1

59732
699897
genus
192623252517

 1265445 
111
species
111


1265445 
1265445 

1265445 

species
111111
 878220 
234413

878220 
878220 
878220 
878220 
878220 
878220 

species
11111
 2015076 
13225


2015076 
2015076 
2015076 
2015076 
2015076 

111111
species
 1721091 
131123

1721091 
1721091 
1721091 
1721091 
1721091 
1721091 

 558152 
1111
species
7231


558152 
558152 
558152 
558152 

 1324352 
111111
species
211231

1324352 
1324352 
1324352 
1324352 
1324352 
1324352 


536441 
536441 
536441 
536441 
536441 
536441 
species
111111
 536441 
321542

 253 
111111
species
1037682

253 
253 
253 
253 
253 
253 

153231
 1685010 
111111
species

1685010 
1685010 
1685010 
1685010 
1685010 
1685010 

531
393005
111
genus

species
111
 2069432 
531


2069432 
2069432 

2069432 

104267
genus
3211
28211

 107401 
species
11
141

107401 
107401 


584609 
584609 
 584609 
11
species
11


1850252 


1850252 
 1850252 
11
species
121


669041 
 669041 
1
species
2


1150389 

1150389 
1150389 
2911
species
111
 1150389 

104264
122321
genus
1221021


979 
979 
979 
979 
979 
11711
 979 
species
11111

1112
 76594 
1111
species

76594 
76594 
76594 
76594 

 59600 
11
species
11


59600 
59600 

111111
genus
28250
392046715295113

392046715295113
111111
species
 28251 

28251 
28251 
28251 
28251 
28251 
28251 

3381
1111
genus
1518147


1790137 

1790137 
1790137 

1790137 
 1790137 
species
1111
3381

292691
2141
genus
21151

13
 2126553 
species
11


2126553 

2126553 

 1913577 
11
species
12

1913577 


1913577 

 411153 
species
1
4


411153 


1486245 


1486245 
1486245 
161
111
species
 1486245 

21
261827
genus
11

species
11
 1736674 
21


1736674 
1736674 

11
11
genus
358023


1622118 
1
 1622118 
species
1

1
1
species
 1850246 


1850246 

41
83612
genus
11

 57029 
11
species
41

57029 


57029 

335543
genus
308865
18733981713

 1756150 
species
111
111


1756150 
1756150 
1756150 


1756149 
1756149 
1756149 
423
 1756149 
species
111


172045 

172045 
172045 
172045 
172045 
45411
 172045 
11111
species


1117645 
1117645 
1117645 
1117645 
1117645 
1117645 
1332135107
 1117645 
species
111111

1325635
species
111111
 238 

238 
238 
238 
238 
238 
238 

1853230
11
family
13

332102
genus
11
13

13
 191579 
species
11


191579 
191579 

611
111
family
246874

611
267986
genus
111


253245 
253245 
253245 
 253245 
species
111
611

6988227554227
111111
genus
336809


336810 
336810 
336810 
336810 
336810 
336810 
111111
species
 336810 
6988227554227

21
1755828
11
family

21
11
genus
1755829

21
 242600 
11
species


242600 
242600 

32221
family
39782
3603562

3603562
34098
genus
32221

22
 1653831 
species
11

1653831 
1653831 


367806 
1
 367806 
species
1

1111
species
 1316444 
354322

1316444 

1316444 
1316444 
1316444 

 164514 
species
111
424

164514 

164514 
164514 

200918
736938
phylum
11420191214

11420191214
class
736938
188708

1643946
211
order
211

1643948
family
211
211

genus
11
651456
11

 651457 
species
1
1


651457 

 1330330 
species
1
1

1330330 

genus
11
1184396
11


1184387 


1184387 
 1184387 
11
species
11

21664
order
11312
1643947

2
genus
1
1511648

1
species
 1006576 
2


1006576 

2562
160798
1211
genus


149715 
 149715 
1
species
2

2362
 1545835 
species
1111

1545835 


1545835 
1545835 
1545835 

genus
11
28236
11

11
 69499 
species
11


69499 
69499 

order
425526
2419
731912610

22223
1643950
22112
family

1222
2422
genus
1111


93466 
93466 
93466 
93466 
1222
species
1111
 93466 

211
2420
genus
211

 1462747 
111
species
111

1462747 

1462747 


1462747 


46541 
 46541 
1
species
1

53171047
188709
family
223414

1631
2335
genus
1121

 126740 
1111
species
1621

126740 

126740 
126740 

126740 


93930 
species
1
 93930 
1

4311746
1643951
genus
122213


57487 
57487 
57487 
57487 
57487 
57487 
428643
 57487 
111111
species

 119394 
species
1
1


119394 


38322 


38322 
31
 38322 
11
species


177758 


177758 
species
11
 177758 
12

12
genus
11
262406


166501 


166501 
12
 166501 
species
11

phylum
989999
57723
95576183212161134

332159
111111
class
86221416108

86221416108
332160
order
111111

111111
family
332161
86221416108

86221416108
332162
111111
genus

species
111111
 332163 
86221416108

332163 
332163 
332163 
332163 
332163 
332163 

352628353116
1813735
class
111111

family
111111
2211325
352628353116

2004797
111111
genus
352628353116

species
111111
 1855912 
352628353116

1855912 
1855912 
1855912 
1855912 
1855912 
1855912 

class
11111
1562566
431494

458032
genus
11111
431494

431494
 458033 
11111
species

458033 

458033 
458033 
458033 
458033 

5448138147111106
666666
class
204432

666666
order
204433
5448138147111106

5448138147111106
204434
family
666666

55410208
33973
genus
111111


33075 
33075 
33075 
33075 
33075 
33075 
species
111111
 33075 
55410208

658061
genus
111111
771217916


658062 
658062 
658062 
658062 
658062 
658062 
species
111111
 658062 
771217916

161855325628
940557
genus
222222

371913106
species
111111
 940614 

940614 
940614 
940614 
940614 
940614 
940614 


940615 
940615 
940615 
940615 
940615 
940615 
 940615 
species
111111
131136194622

genus
222222
392733
261867882654


870903 
870903 
870903 
870903 
870903 
870903 
6102729126
species
111111
 870903 

 392734 
111111
species
20840592528

392734 
392734 
392734 
392734 
392734 
392734 

42446591161217652
phylum
12111412118
200795

423973040561714
301297
class
333332

56111721
genus
222211
670486

 552810 
11111
species
41142

552810 
552810 
552810 
552810 
552810 

1510131
11111
species
 1839801 

1839801 
1839801 
1839801 
1839801 

1839801 

423922429391513
1202465
order
111121

1202464
family
111121
423922429391513

423922429391513
61434
111121
genus


61435 
61435 
61435 
61435 
61435 
61435 
423922429391413
111111
species
 61435 

 1522671 
species
1
1


1522671 

475962
111111
class
11113757

111111
order
475963
11113757

475964
family
111111
11113757

233191
111111
genus
11113757

111111
species
 133453 
11113757

133453 
133453 
133453 
133453 
133453 
133453 

2115516249
32061
434343
class

32064
434343
order
2115516249

632657
1508595
222222
suborder

632657
222222
family
1508635

632657
222222
genus
120961

511522
111111
species
 357808 

357808 
357808 
357808 
357808 
357808 
357808 

111111
species
 120962 
121135

120962 
120962 
120962 
120962 
120962 
120962 

212121
suborder
1508594
1512310192

1512310192
family
212121
1106

genus
212121
1107
1512310192

131221072
 1108 
111111
species

1108 
1108 
1108 
1108 
1108 
1108 


152260 

152260 

152260 
 152260 
111
species
2112

12321
class
292625
48533

48533
292629
order
12321

family
12321
292628
48533


1889813 
1889813 
12
 1889813 
11
species

441
genus
111
2019482


1986204 
1986204 
1986204 
441
 1986204 
species
111

4313
1111
genus
233189


167964 
167964 
167964 
167964 
 167964 
1111
species
4313

632622154
189775
212211
class

189776
order
111
421

family
111
189777
421

499
111
genus
421


500 

500 
500 
 500 
species
111
421

232421154
85000
111111
subclass

111111
order
85001
232421154

255728
111111
suborder
232421154

family
111111
85002
232421154

genus
111111
2056
232421154

232421154
111111
species
 2057 

2057 
2057 
2057 
2057 
2057 
2057 

1382928
class
111111
7227171218

order
111111
1382929
7227171218

1382930
family
111111
7227171218

7227171218
1988031
genus
111111


1806508 
1806508 
1806508 
1806508 
1806508 
1806508 
7227171218
species
111111
 1806508
