# Supplementary material for: Characterisation of the Carpinus betulus L. Phyllomicrobiome in Urban and Forest Areas
Source: Front Microbiol. 2019 May 29;10:1110. doi: 10.3389/fmicb.2019.01110 (PMC6549492; doi:10.3389/fmicb.2019.01110)
Supplement: Supplementary file 3 [file Data_Sheet_2.ZIP › kaiju.out_all_Bi.html]

Javascript must be enabled to view this page.

magnitude
magnitudeUnassigned

kaiju.out.epBi32
kaiju.out.epBi41
kaiju.out.epBi51
kaiju.out.epBi53
kaiju.out.epBi43
kaiju.out.epBi42

381992652777085519099506562237537485313338

21111872125525371390690
381992652777085519099506562237537485313338

11018812912
13884776819151428409

5
181119503

3111443

112

113

1

141

14

1

1141931

1

1

11

1

33

1

1

151

888210473387874226
424

1041901
1

312

312

1
2265

22

1

10

7

138

1

6

511221

10

511121

11231

11231

11231

5181210634
221

53132

53132

1222
41586602

1

1

1232

11

1221

3953562

22145

22145

1221

1221

11

11

36281

112

35161

322

322

322

31362
585112351303271100

151212417
64352748815464

1

1

1

22

22

22

113

113

57271664211138
141512124

1

1

11

1

1

1

1

1

1

2

1

12

1

1

1

1

6138

11

5

1

1

1

1

151

2

2122

11

1

21411

121

5

111

9

41

1

2

12

3

112

1

1

2

1

52

2

3

1111

51411

13

1

2

1

21

1

331

1

4

311

6

2

1

1

11

1

1

1

42

1

1

1

1

2

11

1

1

1

12

1

3

211

1161

4

2

16

6

1

1

1

1

1

1

1

1

1

1

4

1

1

1

1

1

11

11

11

1

1

1

1

1

1

3

3

3

1

1

1

1

2

1

1

1

1

1

1

15

15

1

1

5

1511

141

1

4

1

11

1

13

1

12

321
31

1

1

12
425

1

1

1

1

22

22

1

1

1

1

2

2

2

31

29

29

29

1

1

1

1

14

14

14

137

1

1

1

1

6

1

3111
29348411064426

1

1

1

2

2

2

1

1

1

1

111

111

1

11

1

1

1

312

2

1

3

3

3

28245361021523
50323

1

1

3171113

6

1

22

1

39715243

1

11

1

1

1

1

1

2

11

1

14951

1

2

1

31133

2

1

1

1

162

4

12

2

161

1116725

3

32

2

2

115

15

15

1

1

12121
1

114

114

4

1

1

11
1

1

1

6

6

6

1

1

1

1

1

2242833106608
14211

2

2

2

42
91818221

1171

1171

1

1

17

2
834118

191
832118

11

33

4

1

1

72

16112

145

1612

2

1

13
43

3

2

2

2

152

152
3

1

12

1

213

12

12

1

1

11

11

2611

2611

1

1

1

2163
8

1

43

4

1

1

21531

21531

11515820532
12131

5

43

1111

4

6

1

1

9

4

11

1

1

1

4215

1

14

3225

421

681

1

361521

2

2

2

2111
1

1

1

1

1

11

11

1

1

1

16111

1

1

1

1

1

1

311

311

2

2

3312

22

22

22

131

131

14742381804

1
1035293

2131

324242

511

111

3

1

13711861711

13011861701

1181135521

1251118

1251118

61

1

1
10753112

1

1

7125
2

12

1

1

4

1

3

1

12

362361
131

1

513

4

1

1

1

11

11

1

1

11

121

1

12

1

1

1

1622393619

3121
121

2

1

1

1173014

1173014

11714

30

1
1243743

222111

411

521522

1

1

11

11

11

42526

2212

2212

121
2

1

1

1

12

2411

211

211
1

2

1

11
31

2

13

1

3

81164

1

1

1

11

1
11

1

13

3

3

1

1

813

73

73

1

1

1

1

1

1

18114202412

1684191612

2

2

431

21

21
1

11

111

111

11

11

7519105

22

22

22

21152
111

113

113

11

1

1

1

252

21

42

221

221

121

1

121111

1

11

121

1

1

533764

1

1

1

221452

1

1

11

11142

1

21

111

1

11

11

2

2

1

1

1211

11

11

1

2218

12

1

1

1

1

1

1

216

11

15

1

1

21212
11

111

1

11

2

2
1

1

1

1

1

1485068169
1111

11
1064467667

1112
112

1

12

12

12

21

1

1

1

1

111

1

1

1

1

114037063

114037063

34311
4

11

22

11

1

1

1
213

212

1

2

2

1

1

1

1

1512

111

14

1

1

1

1

1

1

1

1
211393934

11
121111

411
11111

61

1

1
453162

3211

11

12341
111

1

1

1

1

11

2

83

83

1
11

1

24521

112

2

1

111

12

1

2

2

211

211
1

2
21

1

3

1

1

1

2

2

2

262317512316

4327103

1
1141

21

21

21

1

2

11

11

1

1

1

1

1

1

1

1

11161

111

111

1

1

11

1

1

11

11

11

14

14

1

2

1

1

1

1

1

22141

111

1

1

1

1

1

1

1

1

1

1113

13

13

11

222015441313

11

11

11

1

1

1

11

11

11

1
11

1

21

2

2

1

1

11

11

11

45543

2
1

1

1

1

11131

1

1

1

1

1112

1112

14412

1
14412

112

112

12

1

43

3

3

13

1312327411

323323

1

322322
321221

111

111

1

1

1

1

1

1

9102418

9102418

111

1

1

1

11

11

114

1

1

1

11

1

1

1

1

1

1

3

3

3

112

2

2

11

11

1

1

111

111

111

1

1

1

11

1

1

1

1

1

124

124

1

1

1

1

11

1

1

4

1
4

3

4235435

115

15

1

1

3

3

3

2

2

1

1

1

1

122

1

1

121
1

1

1

1

1

1

1

1

1

1111

1111

11

11

1

11
11125

1

1

1

115

11

11

5

5

111301

111

1

1

1

129

1

29

1

1

1

162856957170108

383143

383142

383142

1

1

7054613813091
53215

614541299346

21111

1

2111

2111

93103612
13213

1111

1111

623146

11

11
1

1

111

1

11

612125

111

111

111

5114
313

211

111

111

11
123

1

1

1

1

1

1

2

4
20201476814

21

1

11

11

663347

563145

563145

122

2312

2312

101483577
129

11

81351226
81331226

2

2

1

1

2261
1231

1

1

1

3

451235
171615181312

111

211
7541034

6211023

1121
11

12

1

347452

347321

22531

1

12111

131

132

21

135147
1314

2

2

9212

31

1

2134

2134

214
2134

3

251853340

2

1

11

221843240
1241

1121912

224

224

11011620
1

41612

1698

2213

11
53275183313

21

21

11

11

1

1

21
1

11

2

2

46243153012
33131

1

4

1

11
4120214239

2191464

3

2

4

1

741432

1

1

622
522

1

3

11

4334

1

1

11

1211

121

1

1

1

112131

112131

1

11221
112121

1

1

5253173

3143172

11

1

1

1

1

1

2142162

213282

11

23181

23181

1

18

1

6
8

2

1

1

1

1

1

1

1

1

1

111

11

11

1

1

91141

91141

2

1

1

1

1

1

2

4

1

1

1

1

1

1

1

2

2

1

31761
16513410136222534

1

7

8

1

23

1

2

9

1

4

1

67516987

1

11

4

1

636463563724

636463563723

1

4

12

1

1

1

1

11712182

1

1

1

21

1

1

1

1

781145262

1

1185942

1

1

1

17

11

131

1

1

82821222911779104081820113461
381642752753595517163506217037509305312239

407452194294680553825394
291322202419

241030101314

65102611

414

1252

125247

111514

11414

11414

11

11

1434733
51

11131

1

41112

4232

311

311

172966
346447023390573046384740

21

21

21

2
21

1

1

141

141

12

2

1

5313
464648346626

221821
116

1

1

1

1

1

11

1

10102242

1

71021

71021

2

1

11121

11121

11121

2131

1

11

22

231632195620
121

131

2

1122

53635

2412

21

1

11

1111

11

211

10

213

1

1231

1

1

3

12

11121

14134

21

1

1

243

1112

21

11

4

4

1

1

2

4137313

1

11
413733

1

13

2

1121

1

1

1

91

12

28121591518

1
28121591518

28111591518
12121

1844346

1

1324135

11

21111
1

11111

1

1

1

112

1

111

8695711
33421

1

1

1

11

1111

112

1

11

11

11

1

11

111

1

1

11

1

1

1

157706897921718910
1151611105

422
39638722754588839

334513

334513

12212

11231

12

9615666708552809

9615666708552809
24

3322
3422

1

111
1

11

1609665700539808

2

2

122

1151
151

1

10828101816

111

111

1

1

3313243
112

1

111

111

81

21311

1121

12
1121

11

311

311

116232

1612
612

1

1112

11119

11119

313

313

12
113341

2

1

1

11

11

1

11

131222291711

11
12122

1

121

1

2212

2212

4251713
1

2122

1121

13312

2113

2113

115321

1521

12

1

1
3411

3

3

2111

3211
345493

11

1

3

21

112
11

11

1

121

1

2

492961775531

492961775531
854471

624464
1

1

1412

11

1

11

11

2223

42171

1

111

3215

6113634

11

2111

1111

312412

121
2589116

112

111
11

1

3
1323

123

12
2

1

1

113333
1222

11113

11
111

1

2
426241

1112

1311
131

1

11211

1322

122

12

211

211

21113
13

110
1110

1

1

1

221
4261568

11

11

11

1

4

1
11

1

121

111
11

1

121

2163

12134
2

1114

11

833963
11

111

52411
4211

14

31

122

1

121

416411
1

1221
212211

21

241

21321

21321

2
583498796535

291648333114
12

1
2131

111

12

6312

6312

1431

1231
1431

2

111

111

31312

31312

1
1222

222

1484019167
51796

1

111

182

182

1

111
11

1

1

1

1

1

21

1

1

12

2

1

11
111

1

2
1

1

14

11

1

1

212

13

1

1

1

1

221

11

411442
111

21131

1

13

291850443421
11211

11
634452

12
1121

11

211

21322
2132

2

111

2
521011

221

124

221

41362

41362

6213

6213

11
235516

11

22311

1111

113

151

121

3

94152156
6241233

211

1

1

1

2
12

1

1

11

4

2

111
11

1

111

11

11

1

1

11

212110

13

11217

2165

112
2164

131

121

1

112346

12245

12245

111

111

314038632208464736493561

309138282157456936203527
5

13415963

111
13415963

1

1

111

111

111

135131
15131

12

121

9174

1
307338192136454436113520

1

1

1111
1313

1

2

1

341011

341011

152
23221135

1131

1

2122

121

311

1121
1122

1

24933
13

3

1163

3

606978728369
306538082097449735983511

112

322
422

1

1211
13231

1

13

1433
132

41

463

1960201332207353255

8181122299610183

6111516712

1

1

1

1

12

2

15840152210

111

90734051453114929673062

4161

282532164316

21

1444

12141
12131

1

4252

4252

1312

1

1

1311

1311

211

11

5431032

11

11

5421031
5121

1

1

22721

731912

731912

1
731912

251

231

231

41111

324132

112
32432

322

21

1

1

1

393046682530
263541

1
201528351515

2
214312

1111

1312

1321
1381619911

31

1

221

133

915827

1

311

25111

13

131103

131103

23121

23121

21

21

12
512

41

21121

21121

21121

2132012

2132012

11

11

1122

1211

1

1

16

7

121

121
11

11

136107310

925216

92223

313

142111

1121

311

33413

33413

111021810

111021810

111021810

131

13

1

811177

5132

113

112

113

2613

2613

6751355

6751355

344831

31461
113

131

1

1

1

11

1
3221

11

2111

331524

33153

12111

2112

3

21

21

231232192211

231232192211
11

211032182110

5314542
321

1

1

1

21

1111

1

11

111

19

1

3228
211

118

1

42344
12513888

11

22112

1

511

13121

21

1

1

51

1

1

12231
1112

1

11

1

2111

211

1

1221

1
1221

11

21

12823
8910834108138

1

1

1

11

11

1

1

1

259427103138

1

1

1

1

1

22

1

422466373649
1

16714543

15314533

1438333

1

1438233

62

62

1

1

141

1

131

23122
11

11

11

1

1

1

1

11
231452313044

4623112338

3522102338
35614

2242234

1111
1

11

11

1

148281845

148281845

11

131

11

5143

13131

31

23

12

11

111

2

10

11

1

13

1

1

1

1

11

11

11

412

412

9881158

2323
9881158

111

1

1

1

21

1

11

12

2

1

11

1

1

2

1

11

3

1

111

1

1

12

6553259

1

4

1

18

11

422154

1111

311

1
5224678031009656582

131

131

1809220432889106
14410222

114531352943478
12

11
131

3

141621
113491342923478

127312

1263

12

1

21
98461212873375

1

31211
97431182873374

94421162863274

11

1

1

1

1

1

1

1

1

161914131610
111

1

1

81089117
521513

1

11111

14

1

2

1

211

1

1

1

11

1

2411

3

11

11

11
413311

111

12

11

111

1
1423

1

1411

1

11
12111

11

1

1

61
831971811

1

1

1

1

1

1

1174169

1

1

1

1

1

1

28132512195

28102512185

28102512185
11

20712672
3123

1

2
926141

924141

212121

6321

6321

82126113

82126113

31

31

31

315364571648558456

211121
11

2112

112121

11
112121

1

11111

1
312362568646554454

132171211

52362
1

11

11

31212
1

1

3121

213

213

23

1
23

1

11

1

81266

13

12

1

1

1

1

5212
522

1

1
1321

11

11

22

22

433

13

33
23

1

1
22741

331

331

33

1

2231

2231
1

122

11

11

11

11

11

278350526620524438
1

11
277350526619524438

22211
1

111

1111

272347523615519437
21211

2111

1

13

266342521611514436

1

112

1

3134

311

22

11

1

1

2072271513

111
186147158

11
21411

1

1

3

1

1

21

21

4215
211

11

1

1

11

2

1035635
62123

1111

11

11

111

2

2

11

1

1

11

2

2

111
2124

4

11

2185

1
2185

1

11

11

133
143

1

11

11

11

11
20102525720

21411

21411

2311
117111178

12

111

1

1

11

1

11

1

12

11

22

221

1

1

11

111

11

1

11

1121

111

621311

521311

1

6162
1

662

201436222122

536821

1

1

536721
2232

22

1

1

1

3

1

1

11

1

2

2

2

1

1

1

1

115

115

13

13

1

1

1

1

1

1

312111

312111
1

2

1

1

21

1

212212
324683

1

1

1

1

111

11

14

2

13

1
13

1

1

1

3141116
68207317

1

1

1

11

1

1

12

1

2

2

1

1

11

211

3

373487151848385453483498730636390455228293
6101110542679619637879282873053

7159998617662

21
191824111816

47255

47255

47255

47255
213

14122
13122

1

23

11

7410461
151120111310

1

11231

1

3

221321

135113

1

2

1

3111

5241757515846

511233
522655

41

1112

282437454030
4739736915341

244712

12

12

159188973

22148136

5332102
90752141983647694274152127357811

4421713
1310132588

214

211

1

1

1

3511

2311

62223

51061728

24131
51061728

382426

1

11

11111
261643161611

22132715115

1

21511

14510223

211022

15122

414

3215145

11

1

5

112

21111
21141

3

911245
24210117518315077

27735201311
14156103967246

1

111

22101

2

213

12121

11

21

42162024147

11

1

1

671112

53222

12111

15352

1

11

2311

1121

4451

5122

1329813

1

1

926385

33223

61441

824772

11451

11

11

914471847426

914471847426

21

2

1

914469847326
1

361733252711

361733252711

1241

1241

3231
542536554514

421733

8435113

6121

1423674

5131622111

52291

4523

523441

2282

2282

103212526510
90342141809647351273815125157659

90251141646646843273609124177563
12741906111683394230295

225917827618357220292025

225917827618357220292025
2735126643828

4310733

4310733

4310733

61024643

448522

448522

2616121

2616121

237222

237222

237222

201016712

201016712
136942

32

71531

192938312754
611779168710387211219

74561579

74561579

4536019408056001040
8712415191107180

321

111628191721

8211966

395416711

121032171222

82150111141180365

497

611819611

7973568

2912212

21424

331323581327
341323621329

142

29814211116

175385813535

871420716

111082291423

101814221419
8189221219

252

17142712811
17142712812

1

143028184253

961017158
961016157

11

221661201022

172862374370

691519822

519916935

1112725317

162437171831

715844

2410311
2935256672135

36211347

23291512

1232316319

510138945

59351181

815431979
73833241014145

24111024

15612

97241175

7437823

3015531173

21156952

2

1

4318633

89541565

2152059

11

302773192536

302773192536

221266351614
872623418718311026479

23
3725106352113

210401445

29104712136

6517642

30710145393

30710145393

615801965

615801965

161310856166

1222252

44572732

117292782

148781734013192

148781734013192

2114761885

2114761885

22
423912156922

6146823311

34255133611

4240306921535
11201082088

621011113

6327513

1881291927

17323734

2516922556
1063324

110111

5211211

10768911

1169016643545164
2927222631313

37771533

1272444735

194821763

210763359

1

1

582171721

1261031074

2911822

74491113

211033572715

2479912

2389424

1522
147040105

13332942
13332952

1

32933

162611421
152611421

1

66643

66643

454313

454313

5022132471823
1131

261310225815

2392919107
2292919107

1

54291126

54291126

271811320812

271811320812

412151468

412151468

13101372359

13101372359

236264282

216234062
196234062

2

2322

311399775320
211149797495241133

218821686

218821686

212570472622

19126813913

611181733

30221421907122

9419211211

4317652

534531

48271353

2215361596

52104

1716157512913

17101820811

712512149

712512149

2413
165985432027961

3019428410

3019428410

111
1322320201

8110131

541

185181

23139151

23139151
22139141

11

8166430104
12671

1151

210812

25921

11842

173164

16422
2722214157

651613

23311

18816292
18816302

1

11

13512616314

13512616314
13511616314

10

7161651762
31

53331

1614895

141442

7331946

7331946

1221
112181222

5821

52882

45106430911

3221

72613

356642467

67211853

443923

443923

231893

231893
131793

11

69303031263362

43182961
7769661115

312833414

32341

122141657

122141657

371521031923

371521031923

1361013817

1361013817

13132424
42515549523485102

532092

532092

56313

56313

193612411183446
316281666

61395

321122

451431
461431

1

24142413

76

32412

417851

8421912

8631172

32432

1422

335314

21121

165612

241111

3243

41113

53625

73443

126811

1039963

99311729923

99311729923
87281628820

1231113

40207423129
53711

72875

12341044

511

4612213

7543311
753231

111

6227761

6227761

466141
112

32111

2

4211

2638363

2638363

26423

26423

32166337712
12

2212471946

103161536
103161636

1

1415511144

1415511144

1415511144

381451303157

1331981013
311245213053

961051121
1061451123

142

83118617
83128917

13

423814

4211

2363

331

331

664823127961759354434

664823127961759354434

1523158461220
664823127961759354434

698410401303536
28561417

25243911866

152017234143

122130840910
122130839910

1

151111324510

30114149510

30114149510

2118127521320

2118127521320

5343386731117

5343386731117

155191238

155191238

121022
3748209893524

51346321410

65421554

156932084

1022182084

1
1822103362221

1386520714

4143816157

17316631132220
2483639579927150174

1

1

221646569616

1022117646119

15167525198

273131547411

3127479872025

252063964616

72611314975

201826745610

31119622105

5152075624
513190523

217421

18163466389

6191093185

81015342711

10487406988

141816075396

10183342076

4526611
4053409681117

314981644
312971544

211

117761721

317721324

22211

1781371416

111
203114944512

913691336

1017803125

81767381114

81767381114

54773431162034

1436186481010

125171048

4212614

56151636

33873

14251022623

233

4530782
335232

2433

12122

4316103

4316103

4107817

4107817

11142

11142

611733

611733

17882511210
3131

41172

72

77772887

33611

4124

3121

13

9757189953741

9757189953741

5424916

311585

311585

239111

239111
23911

1

8348115662424
1

485124

485124

7840110652220
12

124152566

407521465

655533

3424853

1010931

6105822

11319

11319

844719311

3112214

3112214

53351727

53351727

255258365854452205416672750
1817788711127

25111264
123844

41

1

13431

253258145846462197116502719
5113833779

112915851875380

465124510
112915851875380

169382756

4

333

2896431105

22812

333424

142

2310333

12

8242945

1

63011519510

1

191321439814

1894323616

21

21

241557125836762174615902630
692451128080449126

443744072612

1411241512

199971413

11173194347

1054377655418116531819857
1897991017934007197161

161551261024

94058213517

31268681621512

233911161801223

30592279075

197129210146

401512466729

112

348637623

359167713992826

50352727112631836

2320645213869

433883145154292111141

191418466823

183623612752412

17533541261211

552222032

15632725559

11324261531210

1

512

196014501971815

3221342695402421
3221242015382421

1682

18302541072418

145941062881511

417446722301010

7296261401812

19982014834

3814818875074812

61095591730

5610028014053

15337771661615
15337461661615

31

32013696026

12586831411118

2264400961210

8431123156228

451998054213403131

1928718163149

122411734617

23112105095106

3761675115813

134547784104

5113453322

113742899611

21233234159

21193224059
21233234159

411

4159063317
396716792772568

21204281161031

14291132951117
14321161981220

329313

2082034481977081
5167212540846173230

991382331

31293565826
34334106126

72

34471

1810141

1622823337

1612162

710591042

7261052421

17174738313

1654644748

2272112

55431037

727711153

28281135

194425074921

94441511

532780691411

125516

2318311043

4187944

124246

346361

41848241010

5279932216
5269932216

1

509290541412

122881689

122881689

11446111

5343310972132
457727

2291372775

118541897

612362226

109762317

21
1521237491928

5117226714

1010163221214
1010156221214

7

4443396681016

4443396681016

2429195571212

2429195571212

106124153853493273
5367071209529044511240

39305251691634

10222712334

15236561121624

2023289662148

142016521221324

3734223863799

28364901072267

31414713512359

356891025239119
356993825540119

12831

161821062839

172411844746

3

16815554711

15407281321235

221414991052447

24339231141843

17385101212053

36414822152355

51415235710

325448319340118

11261212

821891302362552224003773311258
3857328671

1712712165

421

212

2

1511

12

8

11612

44861

42351041

15732743411196
821341301672541923993073181252

21153

21153

21153

132

132

132

142119666

3103113

282112

1211

111116553
159412

2

2311

2

3

2112

21

11

121

171462341

171462341
111

2

13

1511811
11

141181

11

11

11

131

131

818391297102513723932871971195
5548882716531848952367

231312
1716212479

1611

9322

5222

214

521

31
101

7

111

1511
1111

4

164141353

1016331

131

1256

212

31111

103716

103716

42

42

193412724

193412724
193312524

1

11

5114213
335223451311

284
122

162

4561

31121213

694712

14151

155121022

11

23831
916521944

474071
4740711

1

12

12

14182

13121

4261

4261

23548314

23548314

608787576527
113026291013

334412

721352

21

41222

14

3
31

1

432731

41

2

1

2

2

111
11

1

131

1

73

26321
21321

5

23

2883422

3

93402

14

2

2

10412221

5231

529211

4979246
551229586

24614

1

11

314

1

1842
6338612675

81637724

552261531

4410131

4410131

446514091502840
525151

451142

6317412

108016

215311

3769552

2223711

415532

31111712

120413312

23644141

1118311

47114175

713519216

61111

4334193

142427511

142427511

3351

3351

11511
151043451110

252

181912

21316

6110

3131

1

219

111

310

3616221232
92131

1

1475211

512

5

25611

112

6132

222111

222111

47153

47153

1
1715202410

651

351873

2

641137

211

3340261

21111

123925

121323774
2593763221736058

6

1

232232

13714553

831248341112

11

111

11

841031

113

2122

1

1

232451

8113525762019

928153933

2

611

11

1311141

61534731

61852

11

1

14321

1

1

3

662

41085649781047711733208151
7232611569767622160115803443

272118020100023

562960651100422

2141

446322273

126153265462

9012725716

6210018674

1472011191111

1421307351617

413931412634

65114248511

10716130999

4826453827028

9614837271

120153348781

4610635931

7099234981
73103235181

342

60510911276645

1652602675761

10211894327051054

849026148102

2061832113691

12113912665

8119966122

74888732616394

781404301316

7877283131

265319252

1982452226148

72907081

4103252750917

6811815824

4271014243986

911242259105

265112297

296511083

1491171512310

12

69697026328418

194585218318

3985982484243404

1852316817619

6792295261

2130433

4926124831

2173366622714

21834010515614

4455331318391

2074633863210

35852017521129

2451917168112

3074852329531

190250614854

102113983

4166403934

2634101465

10721369153

1771729105612

355611275

1409180833334862

2994133233228

146202318712

848121417

18722131456

106113945711

20137923234333

2164201455251

1924109315161

1047162527245947

45275242208281

22132219156136

47655423

4474195031

33438519329519

3190161372

43866716189929

6195710147

94107898143

26254913531263

885117846316633

381817821310364

1625287710961602

12195251047

67129233106

7441871178353826

8982543167

27238326298122

404530250271291

5714331266

1291515757
13517451298

623541

17427589018

73911894

3272511

13413587371

35398131

80696512376483

108202282117

6795142663

841126925813642

283406159309131

24948519583213

44862322483302

452974150432

664819105371

3638316105
3638317105

1

542941114553386
539940114550376

3131

27870030336363

15629743224123

3373102478

925289710511067112841

2

71116111357

821462013161

34

80181304168

47093226530414

7282112998

7412914659

17923420241133
18125520242133

2211

38264192197331

6423
149211351931815

10166431

5491141157
119176271671010

618214423

4313123

141522051

3351
21104523114

4424981

4622

43491

6632

22541

22541

469215593610

469215593610

88192745

142533

7815212

5320501786
1339331

19434
18433

11

13132

422531

391

25521

2242

14732443110672290285216
26337423624065833

242414440921

775218

443797

395781767

641412

3412

18171

8514111

1116783147
1114783046

211

31512351

28123186471011

31241

81436291

8437512

221916222

6119136

6112383511

759621

10812211
10912211

1

22209

2125103112

3281

36543

95212

3122231

3423924

22515

1341444695

442126

165321

2426112

113641

141421041

130167

151715

1593210

2655202512
2654202512

1

9335424

91261513

11173

1034911413

21621

31931

11131

2915821

16210

1411111
1411011

1

3271

11118114

81648411

29285

2314921

3021732023

7232231

7914921

1016151034

93335293

4839633

5625651

224131

468222

814491121

431

1112211

1530503633

134083121

1021231

41261216

91073

252039192
252038181

111

311245

69441021

579974

1131072

1310131212

17104842424

56202712

1422028

1

9311496

2

72554101

2732

5221010

1482031

762444

71333103

24421

29741

12124112

7132321

1328723

34070321

107761

917158322

8351731531

1

1666122

3910121

303929856035

23323

1646421923

954110

55989

213142

13323882

3223

41721

11

62231

56171512

6228311318

10152422232

41432

12222

1111

113

312412

5612131

62941

5114

74134

252871

11624412

2427342

15111

18271883492

31

31

10132342

31331

312811

412

2
6311264

2

6311224

5961

5961

789431

25231

22

3642

1

1

1
537932

41122

116712

41

41

38399535511
9151282

41306

313

5

331621

4310323

3381022

111

74311

2741

22342

1089733
34421

11

1

634523

4411101

4411101

43
10304627106

323651

16621

271411
312131151

151274

362183

34

34

24211165

24211165

12
1910971952

2
12

1

129

1457785

17

22112

2
25442

11132

1411

114181332
422

1

1642

3912

33

13121

71147103
893070602113

1141412

411

48919954

71321

23111

1531

111

1351

42

5111

21

131

2131

112

134

11

324

1

3233

3665

3665

16521041

16521041

5111152

5111152

2415623171

8313192

3813

141

91217821

3202

3610416504

3610416504

138281454
12

221

21

41

11126

222

1221

21512

3311
33511

5

3641432531

3641432531

1161571253
312032

11

1

66036933

1121
181610523

61171

4311

81231

21424

121

11214

135

135

53221

53221

19158545410
4125121

2561

10

23156

21012

13191

111

11

6521621

2301439701151421
735513061

2153313

3113111

1164

4614513134

9221321

5630171

2521

3632118

15127801724

41

14212

14212

333462

333462

123111

123111

41523
37399836912

1322

11

11711

12214

63511

21

2121

4103

111

21111

211

11

7331

202

123

391

434

2116411

21031
1510624

13323

552111
361413836

6212

8211

1

4262

4132

8312
8311

1

111

466744679813
351431

11356111

342

85402

2143

112531

1318220

3221

115

210

213

12

226211

4164

88108623

3411

46

561

1020631237
1232

7121

141

1121

21

222

311

162622

34621

1121
491818121214

278348

231

31

122442

16712422

8107141

8107141

111

111

882

882

1116542482
121

26

331

4326133

2221

2122

32123

131

11411

4406894115806113
189312127187213

58

6164225071

1

10882829071

15781

87153221

55051512

68164153189234
68164146189234

7

1611

1611

615107961

615107961

820461154
51

11

4782

11

7142

35121

331372

3
614171342

2410331

132311

3754

48886
12323752475

510521

4112

16262

63

1110191

1472

1

1111

2161

2114

1416211

221032

221032

213231
433177412051

1834143

227

11572

164

11131

731412

51041221

7455946

3535150421716
112

31711424

1134122

24722

1

3118141

3118141

8232424

611

1

221111

22511

411

211211

211211

67211714
1813883166

4244822

842363

7059621127325

1

1

1

312141

312141

81415
6558601116923

1

11351

34344038627

11112

214

1

11

11

16

44

12231

11

23

62

111

111

1231

3

66102

2227

21

11

11

51171964
83277729262252536544

2
112804251145645

18133113102
2111

752116

3381

42235

4122

4122

761712

761712

1797210810

1797210810

12
336451

335411

335411

2

2

5324134531417
116111

4813613

4813613

2213721

2213721

233174

233174

122621

122621

3313722

3313722

72491154

418522

3141632

1
92963

8173
7171

12

1233

3112512

3112512

12
2131180341915

41682

41682

148332

148332

31329771
1721

17923

12333

141

11
7492837

1171423

6320314

5945645

5945645

41732206

3211

41229143

2152

71167924522099468495
46501741402718

5845134933124
214102

326331

326331

1531

1531

418743
5040112742121

32

5

1

111

14512

2

2441

1

1

11351

62062

1323
1221

12

212

1221

11

621

13

28262638615
2

12

1

1142

2215

411611

1815516413

2

242

11

21

22736

22736

5331692
12194458219126103

1211754

122

21

1211422

616722
5612

111

111

4

21

141388424024
1058644417410494

2

1311

121

1121

11

11432

1

11

441

111

131

1

11

1

21

121

13212

1

1213

1

1

212

1

1

5121562
5221562

1

1

3

131

113

51

1213

11

8

52253

5

14

111

161322222

2

11
142832341217

1

12

121

11

1

423521

11121

1

1221

1

22

251

27131

39112

21

241

141

1

3152

311

1

1

251
25

1

21

1111

11

32532

3

313231

1

1

1

11

22
122

1

1

51131
31121

21

1412
11413

11

1

91

111

114441
121

1442

111

113

7

212

11

31

31312
2

3131

1

1

111

1137311

111

1

1

3

131

2471

11

71

132

1

11
1

1

23

52

1111
111

1

411111

82

1110

1

1

424551

12131

12
112

1

324

5957130968088
440141620

11444

11444

32

32

1324

1324

122032131

122032131

333634552932
845764

1211

1

312

1

8311

15

1

1

111
1111

1

1

113

361152

3411

11211

11

1111623515
7562139

46216

1

11

43832
23532

23

1111

22122

3

3

147231265
2

1111

412

342513

51213

2

1

214212

5861

261

56

122061

122061

122061

32264433238
22245

9104571
151891581

3

269

269

1
21

2

2221

17711
1462213107

21372

112923

123121

3

1111

1111

35921

35921

35921

42229821512

14
42229821512

3216691126

82311

2326534

1

1

621

621
121

5

33481121

24

11

13

13132
33241121

2141

1

1

8211

22

67316
229226727349118177

54561931053741
222217721347118167

1111

2

151

1211

43152

322

41

32

22

31

212311

751

111

22142

11

661

4

321651

1511
2521

11

1

912

2211

1535612

11

12242

4

263

2191
23136443159

11

1

1

214171

56211

321111

5

2131

1

12

213

1

331

311222

2122

2

231

1141

24

8

2

111

1

1

1

11

2243

136312

2

271

3

12

1

2404

111

3121

16111
21061111

11010

2111

1

10102714813
616950362845

21111

1

515722201930

1

22

11

1

2541
52641

321

9312

2

22962

123

1

111

21

321621

51

352225
15111

21124

93

1

2116121
211691

3

32124

41141
4141

1

1

2532218

221842

2

22052

1

11

224

224

111

111

52802669532515

12
56282

1523

314

1

3223

3223

332610272
47712629432015

812504411

23010

31151822

113762131

113762131

134495833

21491

145

62302224

13865412

21317782519
11

2182

2182

18297159515
111712

57

43362

22

211

1

11415

11112

2132

11

29111423

1

251

259411

11112

1

11

16

11

12641

12641

4438261942229

2025218821323
45332737

224

1

111521

14

11521

4202014

11

1575412
1

1575312

4

112211

141

131
21613

11312

21101

11512

620742

321

1

1
2413431296

31

1111
233211

1

123

1

21937774
21937874

1

1

111

1

11

582469424424

11132
582469424424

28363

28363

54194205

122

53174185

113
1013152225

36673

6731021
6781222

521

2412
29312856

315433

2423321

109223

109223

32526

32526

3222041110402185192

5747265733852
3222041110402185192

21112

3212231

1111

1

12212

1111

1

1

11311

1119

11

1431

112156

4521

11

1

221211

431

1

647214

1

10

372

21132313

2

323

251

718233531030

345611

118541

21

11513

2114231398

32

101222623

210712

1

1212

182

2

11

11

64561784

111

52221

1

521316

122

2131

1

115231012

93414

611421

64

1

2311

1112

147

21211

113211

82107621

6123

1954133

61112

1111

151028561

4141

211

5131

1

345212

124793

321972

4121

242362

9

1511

31

22

121822

31112

3163

44111

346764

7612311

324141

112212144

21

1

5191071627

5191071627

3765113725043

917914316

554914

31111111

1321

1121

2848104584727

2848104584727

2848104584727
11

14
132981

312

1631

11

118311

118311

203870372818
1221

131729232512
131

2163

2411434

314

26561

61

614333

62

821

621391235

41471
4234101

1303

2193225
2195225

2

1
55249107

3122222

21

211551
211521

3

1233

111
3947163924136

11453
3041136712626

3
10143821115

1215631

1215631

1215631

1252

1252

1252

911181064

123

32921

322431

11

12

1212

12121

11

697020512
192784451221

1

22231

1

12161

1

111

4411

2231

1

1

11

1

1121

11

11

3213

111

3311

3211

8626201510

22211

13432

43431

121211

11542

118333

25151618158
31

12

212

111

2641

4623

12

12

111

42

2

84311

13

111213

1

1

212

811150552531

522423

522423

522423

522423

522423
3212

2233

1013423

1013423

1013423

1013423

1013423

651044212025

651044212025

651044212025

1032391418
651044212025

11

1

11

211341

1

1

11

1

1

1

1

1

91

11

1

11311

1553113

1

11

112

1161

1161

444121

444121

1

22211

11

2111

724424
221

21111

21111

1312

1312

2111

2111

875198826450
11

311624262617
814793795247

1

1

11212

1

3142

1

1221

417812

1

1211

1345613

3123

23

24

112

13341

22

24112

1

51

111

38141

11112

26

11

21122

1

111

32

11512

12111

1322

12

111

111

2

51331

2321

111

11

334131

334131

334131

334131

3143

211

31282

31282

31282

31282

121

381

349131285

349131285

349131285

349131285

349131285

348131213

172

482649403931

8122

112

711

402648383731

402648383731
21

339123
11212

1211

1

611

372139373527
6251

3324

3324

4251145

1111

12632

31342

21272

11271

11

117531

117531

1324525

211211

1113314

22

22

12323
11

1312

11

321112

1111

2211

211
114351

111
112

1

12121

152122

152122

21213
21211

1

1

4111
34241

31
313

2

1

517121
304231389431305258

8154888614439
252337432719

11521

11

1131

1

112

11

12

211

111

53422

1

1

34

42335

11

612

22

1

92721

1027642

1231

1026762

22

11

211

113

212

1

22

111

11

112

1111

211

11

1187

218176294344159218

213171288323155206

213171288323155206

1662719114
242

61111

114

646242

211

13151

1

1

404950663181

404950663181

6151726126
242

2112963

2112963
2112663

3

3412
21

322

2411

2411

182732

15622

3211

18202691433
12

1716238529

142174

133811682038782
171830491216

1341

30123913126

3211

32437

3554102

112

7113121

6

126423

522

13242

1810151196

664144

3412

46634

31

343623

42232

423

13941

181941317

104837310

55621412

11522

17

1

21

3161523

573305013564416781333303465997
338443146443033929995419641028200504810878

957677
936477

212

303029244429693427675310002426705634399749
15182514455112896610758690200130696

4234114
743386796709501418

196913147
11111

1434774

1434774

434562

434562

151034231326

151034231326

4691454

114259822

773062672435
705368750669473371

86141455

86141455

171617191311

171617191311

5361413
35283643306

9291272
1216916103

314431

1781

97862
1071063

121

72621

3411301364

3411301364

125711031175872
3219284192

37

11125

11211120826

621346661717
651446661818

3111

24345

149723621

41185620297
11

25103211175

157249122

372143582032

331741271728

4423134

131016151213

131016151213

293122462833

121232787
293122462833

171919192026

9219461034135
25101937913

13542695

412112

43216361710

715355

232262182613
2

33532

411

4315312

9817588

11121

641

2223211

1311612294

1311612294
1311611294

1

222524

222524

1598222511915097
752878504235

929104516

363032161322
373032161322

1

21631

151

249183715

311317

84762761

5211

12722

11114

1063269372928

1063269372928

1063269372928
1

591729231716
511627221615

812111

471540141111
471540141211

1

34101692333

34101692333

34101692333

34101692333

1282
693506531675421369

15556717516176
613212

281729121819
1

872346

511123

326261

112

622122

4417426
6417427

1

11

5245

5245

344551204

303221191

413313

13
691924441641

36641310

331220401331

11
131310865

435743

810522

17412018187
532441450583249282

1053258652334
491347392489205259

1

11412

7111312

2111

336119

796658

121

3531

261
262

1

22112132

316223

1131073

13334726

224221512

123255917

495244

13738103

6111

61011622

32132

111

13311

131251
111151

21

742424

44321

1

23643

11137376
341211

896165

366772
356772

1

151

1416844

49422

112

2116191026

3297

434253

8115613

12

28116

221

161721101126

1131181012295682
1121161002275281

2

121221

2111

342

19120105

11592

527431

235226722416
1140411147

8422927

48203282

111242

1721

111

51

5989119

362384

13552

113

112

153972712301070547746
2371471010

21092127977476

772739312023
21092127977476

1966865

10918677

3014229920

967159

3091822102

561222

955281

452932

1227524

532221

651131
751358583637279394

3383061110

3383061110

14121310716

14121310716

2921191998

2921191998

11881574
313848682416

71

1520244436

51098146

14810281014

14810281014

169151126

169151126

16649688

16649688

332327352638
423199285358154238

631941531340

1072553403016

31161735134

652256691623

231686131

92597310635104
60143156919

324542502685

9191014812

2197229
1013246803151

44813291017

361526491925

682067502026
634332

2661635413

254269108

11721333

2259119614563104
424211

24211915810

24211915810

271426241315

271426241315

11785710

11785710

472932361319

211

11

91101559

7179732

1211

311191235

1742520411

1642119410
1742520411

1411

4463824617

4463824617

248264820
51844191121

12

112

11431

1511111

11211
915559584756

622583
32414151228

15187423

111432

533712
585144413427

34333

10117335
363235292723

22182491515

22111

12276

11922

14123232

181632241014

11312

45651

1211

418715

1

4210366

3423

3331

2211082191026492
412445131515

2110281465

1810271364

3111

6142
61243723821

40183011511

1566838

3592611611

633311

296238510

296238510

1

1

2517197127

2517197127

111120
382464341732

77221544

181112774

136191154

474245465367252256

1712834913

1712834913

1712834913

733415
457233457333243243

175499

175499

24232
1316389845343

651737392224

644648432817
644648432717

1

58131617128

58131617128

281725231722

281725231722

132641581035584
11

9930106642453

333452383130

33821291917
10066161989672

24109665

282451272130

131560204315

29201675

211
1227105111987321702749

3122
252024102931

246181

246181

121138
149139

1

271

3322418

3322418

372341

372341

121115

121115

12232

12121

12121

111

7924121318
1202102911737211673718

9713544
14510053433460

109164436

11165138199

1339744

21621937

392041422633

392041422633

574853283331
292532

392335161718

16161671311

247264272208134177
7988049345661527550

1

494266212235
504266212235

1

31385455

1211111

1112

21214

12104151

311

32

14724

1331031

532321

22174882325
13174772225

1

911

917321

311434

358231

721194

1

124

19349

61351

36294126612

1332

21131

1121

114323

4510184

6647131226

221116

235111

3019141224

440774

2

1

3221

14361668

13222

212

518226

13414411397154149
7679595511574

584653413974

1

18111

142

381848362716

11198418

62740867

5521625

523111

43381

12

15911434

976781
956481

23

768583

111

1246225
1564868304026

547208144

241961426

66183662211

225212190188536182

44244
225212190188536182

3210131

3210131

4214252154

4214252154

176192153162524177
3524332818454

22

1

2

137164113130310119
136162112129289116

1173

2

1212

311

3

111

21

1

31122

13

18111662613
22498759413292700146615218

62484

62484

37710131310

37710131310
16477106

1

1

2132633

1

1

1

12311
302241726840

201024373924

191022333924
201024373924

124

101115322815

101115322815
10810281315

35415

1031111107

1031111107

7140761343145
4321033

352544481322

321230761520

1533221
693123472125

14862299

16871469

24127946

4111522

4111522

311411
332726431719

131761478

6211736

11781864

9510374
322621222715

121044148

865951

1111

242511

972146441418
331133182302382129

8866781914867

222

8521211922820

2551322239
11

83513113

16289116

311

3143
343

1

11
141

13

2113

2

2

1

11141

11

11

11

81279137

1254811

4243

1221

149191862
211

3261

11452

11452

111

22381

1111

63421

422229536915
25311

321111

2

371524466613

1

1

13

21685718912753623039514865
714840333838

242951512

4612

24612

2214232157

534232

191617783

66915

255

2121

18311433

37344

812103105

311533

629434

31211

1

44112

4243

4221

113617

323312

42263281439632

1

8210431

131226

313121

2

1111

128651

5265210

115221

1

232222

31411

208135199

1

45741

14419882

2614829209

11312

22

112

20661153645536

41

1

1111

34522

16613843

946512

18613542

3111441

36211

226

24222

4122

3111

6105241

232635

1921139104

1371275

224512

1

19761746

237032324121

11

1211

3446263

21

624113

13221

52213

41111

422213

582435161617

2

686623

181225191420

1

32113613175

362353

112

27171771216

21632245

201617794

5472543

11232

1474763442532

34412

678456

2

1

11

14669106979863

1

21014142

742111

17543

24523191711

17355107

713421

12

20062605111006533828233974

1

144722642298296

1088121010

34

3113

211

1

981766

109317434298

11

45274716157

2882713310

1

51614

1083187483540

19223298

3119161147

3119161147

101

6

1

1

31

142
32132212313

76542

25613839

211511637

211511637

2224198244

2224198244
2223188224

112

366234133146266351742395
9251191916

365333883135265451552379
444477392336324403

824582605127
9917965

1415347206
1415349206

2

926125

25613964

910815124
910816144

12

16341633

576060554427
38122628189

172107

1

2

113

121

1

11

1

21

11212

1025264163

12

12253

1

1

1

111

121

538621528309308296
275225272221194344991750

1

11

41

16531417
16531415

2

1

2132

1

11

256268115
246268115

1

11

11

2958312

321

2922412

143116613

5113

232081155

11361

11

1

1

4610156212

116511112
114511112

2

21832

1613

565341

111

1311

19617693

948121
9511241

1312

12

111

1221

121

738535

1144111

23213

155623

1216122

1

1

13

11

421412610

724152
72422

13

48424

17731

31

1111

23614852

12

31293089

171013885

2

111819172813
1112

22326

631635
621635

1

14
1141

11

1

111
11

1

210106116

222

111

1

1

2

7323

21

111122

1331411

1681016163
232715242821

4

719581214

4196

1

1

2

25219111418
23219101418

21

3238444

153320121222

1861015125

22510113

543433

62453744418
795747105326

1746688
171210698

841

1

55325

11102

51101851
510321

111

1

132

1

22

511

66264132

3212

44211
34211

1

15

1372520172

22

44323

1023733

17362212

1145

1123

1

1

7110324

1

619131

10

122111

3316

11821
1110321

212

111

1317580774492

3693032803243134301
3492882733153126295

1351624

114142
1151524

1122

21

2131

3

1

1

222

111

2

26111

336427215114
837928601660458569

4921817166
112646

179813106

21

4

2312421
2412421

1

18713736

86122454

55231055

217353

317334183144166165
676641468514299454

1

1

1

849622207

351258277348111281

1

1

728151304319

31212122

8522
863293

1373

1131

63331

1222

1

221047

8273597

32151
53151

21

6665611

281107

22632

11

113

2117524

33492136

1

4121725

11

361025753
361329853

341

1

8231

2

1

3

41

3219374

85512

1071113156
1071013156

1

1171098771106143

1971414113

7291361

1255152

8661144
15121719920

111

333223

1

11

4264213

474549283113
24623330820519099

85105615

51

34341

4520551
46216142

11191

810246178

244112

5331184

1413

368218511
379229611

111

111

11

13141410172

35152

1713

3211

94141387

72623754
71422434

12132

1121

1

1

413382
72101085

31773

2111332

177347106
175267106

28

1222

1111

4423126583

5937
51039

12

15726833

424144

12

11212

12249

10564117
172131181939

29

1

1

22

1

41314652
41313652

1

129731

1339135

1339135

831356

81215
821245

113

1111

1061739342519

1061739342519
1

39416181111

39416181111

18915487

18915487

48481261

48481261

776839635669477730063839
905482823480

12211
1441091241074679

423730281223
213

101591187
91591187

1

30211817416

615171442239

603248381539
615171442239

1192367

402121331116

402121331116

442423493207293817462362
706249553742

310916962101216312871911
660330425428248273

27111718113

2111

7131482110100

60152234619
60152233619

1

262510471419

462591471117

281323241311

5751124

97810810

8481317
6381314

213

52221

871941221214

41262039197

4

431741257242

1969946

3121

612846514822
612846514522

3

311

1312131

3582373

141112934

113471

1319121284
19231416207

6424123

227311

277171375

101

4

4321

4321

91191827
854552601621441776

845541592603441749

75156183

1

288141713

72882

42612

241610101829

92622

177366

191113686

5126722

16128

1891410105

1

151115345

12381

66104440520

272112

2181410123

38171222715

361328162318

1331812

1716121562

13141

211

207101883

44132013611

3312612714

904349372419

1416181135

1291010115

2142171287

732112

62743

107631013

3012241154

15212985

3011222199

213

361926201621

27153110411

1771216114

1

1312891310

14711426

463463

2314973

2510122678

2516121763

20924311212

85142

52011541

2213

301012577
301011577

1

842635

141

2272411411

313415116

197191285

41771

762145252558

762145252558

250922011478356
732957521617

5113423387

832561311619

152010286

42202121157

161913221813
3821022

11136

342

732332

678561

8771076

8771076

66622

66622

2871271541757975
931745281116

44322027129

1043359512724

1616550620

1116129131

19131310105

453353501720

31

31

11

2

2

373150471319

373150471319

21122

21122

311121

311121

311121
211121

1

11

11

553372542230
9736103613040

311

7212

2112

12121312

1824354

2

435230456210154135

435230456210154135

2520191775

2520191775

182801341085365

83514310
12259106693147

3119377921

71274228914
72274528914

13

11101920102

602128392218

602128392218

15662134583444

1
472243271417

311

12

5341

14291062

28152813714

1094091312027

1094091312027

731545312113

731545312113
20616853

41644

115372

161

211

36213234

1234713

20169291409977654781
392027281322

835126484231
2212

37669147

171713221914

19541154

8212446

8232
815736401733

46102521118

27451116623

129131244
19178128736057

101422365

2381615125

1143057322631

321720111212

382145182316

371537141714

168462

402415222019

402415222019

489266372231122232
491370241121

1062558401320

6010124261691

3424251563
3421241463

311

28171022105

289191488

481354381242

1922327148

49213015119

682150302125

574845352635
41

251322888

18327141423

14316934

23815123311

422231
423241

11

822222

11413778

661831681925

661831681925

32121823316

32121823316

172017458
10412213

434234

313111

1333898482840
479160354198158144

86732

46131915713

343181021

1724333

414115

922424

2996131344

3614229411

1388713

17525645

255113133

582944

232524

65122172

1852297

542466

21231

702855341628

154151057
702563582034

325215

281741171112

1

131111

11112829

725253
1675080453439

4918191759

4412185911

4113221647

2

265145119

693076411021

58256833815

1158826

341217182014

341215182014
341217182014

2

27192891714
3

132

15610178

1110185104

14101661210

14101661210

269127276178151204
111

261326171512

261326171512

243114249161135191
491544171221

331136111315

199151394

136123073

725286

4109595

15818967

411226101513
411328101513

12

6240826456117

974648454339

974648454339
603234282323

1

19

34141316916

3

1

1

316192210253224172
1

23121216116

23121216116

271171191231200158
15378114886081

211931

1131

8111522772

14931177

322414

263

1

563625291812

1412

13

9179973

4226121
4216121

1

1822

13152

1111611
2141

9121

12

113231

271816

1

1311

311

21976138
111

1856554

331173

1

1

19815857
15389331369141611791031

684151635540

794101512

19122416116

2096341115

218173137

135

481514988

481514988

481514988

799395685650708437
362328292618

13623853

13623853

61214342

411711

217241

121101223
197802252146594

29102143813

7419222

1269715

6271187

10614854

24152552925

275171913

848245

19313313

131539121511
131542141611

321

3094041813

492432251123

492432251123
491432251115

108

451920141425

411115101114

4854311

1017533816

1017533816

862917149

862917149

5319241968

5319241968

281925243415

791011276

791011276

2110151379

8312984

8312984

81123381218

81123381218

4220224415020
5231383

31118211316

536246

145865
145875

1

341845311654

341845311654

7449243326916

7449243326916
7449223326816

21

1
504331746

11191134

1

37324612

1794131028

1794131028

432428131824

432428131824

78121388
311327331219

182131738

532313
632313

1

111

111

2681519814
471941332134

458414

5243510

12414776
12414766

1

9161111147

9161111147

9161111147

5658162652651

2821115241017
5658162652651

4321741

11

1

21542

131

1111
16231321222

15221320221

778668

11

21221

21221

44102125914
511383405572348463

713121399
21

5119765

223424

11
443541493318

191520201111

25192128227

8291985

8291985

251468494

251468494

971430139

971430139

174752322

174752322

13195112231139250
5441439749126

2

42323

112

1

523885
412845

1114

12

112

21

2122

222114

1

16371335
16371336

1

4

614192

11

11

112111775

12

2

56510105

10131415216
10131415206

1

2431

139541

1

1

24543

5312

411112

1

11

9

11

411360

11

1

11

21

1

1141

134545

134545

893726

893726

921749261316

921749261316

1155982

1155982
1155882

1

11792316726

11792316726
11792013726

33

2611281034
11

1958711

6619323

91617121940
1

3311

61317111740

91212668

91212668

475041552230
5511442

566636

25101230612

141732

686322

4174535

1111

261624361517

261624361517

261624361517

1
132616001235108413971349

638796479846
132616001235108413971348

638695

638695

5812662

4412631

1

4412621

1431

5513566120195273

5513566120195273
3359483

5213161111143266

1

1

22

2

1

3241410105
126242

1
2117863

2117763

111

19915442
39202720910

17851035

2331

3322

113

101524
611614221637

935

121

3575526

121123

113

1

11

11

1125

51712

708047189927
2

2221

3855319849
626036119013

2454264

2353264

11

1

1

2322

12113

3961310

614

127139432
72811

4226241
4356391

1315

1

1223

3610113

3610113

1

1

52321
333225251014

772

12

8108523

1

81313

621
52

11

216647

3915

1

234315
92826191116

85491057
864101168

1

11

1

1

31442
31542

1

51
52

1

1221
12121

1

6324

6324

1441

1241

2

65312

65312

11539156835
703903723656751749

1

4315863
125346

36243

53211

1

324

834312
212312

1

621

1

13255
13245

1

381121

11

11

127124

211

15323

640832636600632667
640829636600632665

32

201220151927

1

15610472

15610472

46641
159258135111130140

1

147227120107127128
3371131

1301311119698112

1

13932101615

3224211
3234211

1

4251

166675514342320858579423011444113
198672695213186326536952

1613404950546119784287780840168
10877397593856759280943580

1152549111715257
10395121

204918432914
235320443015

342111

1853145215
1752145215

11

84779297
84979297

2

3240917296

193818272019
193818282019

1

518149114

109762561379777
8704395457976730498

7323312422725

3716315371215

6933832766029

2122191

4324523613218

25233

8325437524042

137141177

7020345726333
7120546726533

1212

149648107221144102

102627471069265
101624461059265

1311

24127620123

4331834656431

2212314241215

2826420485435

3152424
3421239224372372306

1154483188100

6815333386418

447127323017

221011620726

2930132632534

2212115364224

9316711624437

2814223593725

2211417273321

6184525351912461939273464
249834773517134611921354

3417620381911
3418527411914

9733

422321216616

126344811017957

329412162

18945765247675150
269541669634111187

2529421357

121793

5452014662830
221731621

1483823109

74953

311713221617

11

13

4217858325019
4113258314418

146161

241476412316

362193811136
351533610115

1662121

9126537358640
134552476010958

74575
5162

22973

1117424
3624210181818

232235161814

1

211

93723261269

21231

21231

1084526168
141249282111

11

1

1

11

3384143

3384143

5

10981725
8901425

283

283

281832312830

52649132

70231169

219231

96314291423363

96314291423363

1685761110
1689761110

1
4

2

1

401578513627

4019328281732

1828522932

14037744704736
10419038442429

32166524206

4211231

4211231

2235818561215
12196113267

1016272468

1016272468

11152927863961

2533611107

23771753197118194
25781557216126203

41417
4

41413

413222

3371164

112

161

2

2811
62773

41962

2040713983623

12243009543931779848
26112115158

5221120312
9831531755739

197151365
313111

143711

16441643

7422215424822

11002582511841707801
9111840476702572700

2617
312110

3

133

3151413

3151413

311

12552618619759
11646218569354

848533

11612

1133
19

43

31925410

5415412662229
5013512622127

419412

2618829569220

6123417272020

9941723568438
9941523568438

2

833814145415
853934165715

21223

8641730443932
8643132463933

11121

31

14101143

5726122527125
4524117507123

1220522

63164145830530

254150185199216156
223139380182198142

211

4

1582261012
1783291112

23

11

11

1116252

1116252

2111

314

14

1

11

347547209190234171

314518209173219148
9920596769199

6643

11242

129427344515

31413

11

112304016218

39334

4659131112

133

20131

55942819378

3327161219
3329171523

2

134

14181111368

14181111368

1

1

8244257
632324071124338

311612752122526
301562640121425

13

7

7

1223

1171

1
11

1

2447917135

37441260983755282772423214067
1416303931651950642624135531431

65961312813

184252551121381

937751949732321246104610315

121032673214

11113453219

49684103113546

1045125307

29212801961048296

28324451823236111

3

3

63254539208933

207226338

122018

142846772336

391952497633

214

4839313402618

14651107234

59181214718460

2264

124229351073665

6525014811938

383391233911

3212846

2111

29

71027769952711397125085303

69387709152611249120265241
71027769952711397125085303

8443255

1152547

168220694

461132

2201346

6620914113020

1914

12661

18122

32310

6252

521376713720

52111

6

55

2226805322863124

5303720

111
221

11

3334616823539

161

84426622

527193865223088141

5151911791846

95501171055453

95501171055453

364112811216

1

221128

255913451945393

51

23513463361745289

111
4711427504529

20531517259

266112331919

851279681310011619586
198705304209455184

21

747634314623

476933262317

3122631

298723244114

11

7701023710

1

1

749921697918

205310248910

3317119383343
3317119383243

1

772385515911862

1

14210

14115112

40155355339019
41155355439119

111

209917522430
209916522430

1

5310825452920

2188211

467611532621
427110512620

45121

268844333421

52531

695711183

40414649412246

249119123014

285632242315
295932243716

13141

1

47830795822120746310680
3205117382058653981254302901

10824648710299165
420103716284916318301

3549612611117

3

681822626453455

13711182189914

10117721412935776
10117721713036076

212

11

351952987675140

8314617935

528613955257629

153301444137882240
2415

17671263323519

1

6011517644408184

761171375923437
761171365922837

16

771127220367114186361
3586019583251865176

142339131176

4334910945

185679141638

1257181

1

344

15202152511

8468292

1514228452

414

4332681710513
4332711711016

353

12182819984
13182919984

11

1

21411320

2253994520922

133261121754

46951914139413

5193021772
6193122772

111

1410331

294121

1233721814116

2224599668

1

1

144131336613

24192234589

59112

1113753

8951752511321

2951572312612

17651003

78

14361015227

14361015227

272431146813

272431146813

1075555999272510665041156
2501399246365917061268

18781263686911

28751193672613

105485825229489396

9260512502867047116

1821306223346221399296

131

19801382798920

12754110882575286102

24092015754957410218
23890815444767313212

2123119976

14661032381813

4632

45115966819227
11

216654499415
216654489115

13

244842199712

415493697527

415493697527

13713828012649858

13713828012649858

1849922814516

1849922814516

26741272223015

26741272223015

62142852225126
14428116710534092

121

474658164217

327

91931036

12137231411

11111

25

21

423745

41

626181119

317411

3502123823347525351206564926163
2702981659662447099285615616150227550

57812123
18416444825721987

2970134404921

1

2969134394921
2969134404921

1

110381631378337

110381631378337

4049143687526

4049143687526
3647135677326

42812

238221206120141235

1

1

1281171776910588

1281171776910588

109104295136147
6201713112

10459179123
9266123423141

82213171418

1118424

328998075611364758992473
263203311024877155002640612804

104133123879847
2032224287

709074524032

12132110297
14402711308

2276111

41067016985711231417
11594544144323122

311111899714184
311101849713481

1573

61872769521529
60552649320729

1321228

362211449816253
342211449516153

231

1361363287431980
141136462100336107

5134261727

262183375422
251447174617

17362085

722457229

611405209
722457229

111522

13728619213922277
1001841399717954

379551424322
379451424321

11

711

1

214365374250365195
1076141862542228632331452

20407274118

3498614045019

2

24

11111

11

1462112

183441364216

1

11511

13171943713

11

11511372

123639253916

111

11

11295

142913

399301317

39103

1232

134264444215

111

348276409042

11

1

321184

1226445

101823133148112842185971
224666364195502178

2

2122

1111

43111

1

384898167
496318134524

1112

12

1014752715

1

1

122

1

64126685514048
67130715714149

343211

1

922

113

121

1

1

21311

112

416

2313

211

2177

81

1

1451457923413370
1411186723211561

225

21557

265282
111

254182

121

11

4332171

101668217

713261

36263
37263

1

2

4351430521409817390
4641509555434850409

111
11021

31

61

341

15224

21
121

1

5

1

11273

215

42474

1

15531114198

1

1718

1

2113176

111432

1

32

25

2510766347730

1

1

122

169221212172216142

21

1

711

52

8985236612310832
8985237612310832

1

311

3744

32

121

212610141

227242333014

544557292129

617165359

212

10271423

361242

3512225

403368416218

555364

1

21

61716897

1

1

22865134

429869743954

429869743954
357763672651

72167133

8180794911220
350446883620248246132711

1

331744903386235443952625
305838533006210039762317

3

1

2

1

1

14142

1

915151513

361

21
1

2

14322

2

1

11

5

11

1

1

111

1

611967

11

11

7243

723

1

21

1

2612

1

2232

2

11

1

1

1

21

21

1

1115234

1

21

2

1

1

1

1

3

43

2

24224

1

1113141

1

2212

732217334

1

1

11

42

28134

1

1

1

632

3

6347323

1

2331198

2

1

1

1

1

2

3

1

142

6

2

1

1

1

1

612125161

115131

14

28411

3201441

4

1411

1

131

1

1

1

11

4332

1

1

1

1

1

161

5613

1

1

1

1

1

1

1

1

11

1

1

1309111910564125

3

1

1

1

458254

3109493
399493

1

1

7

2

1

11

1

1

11

1

2479212

1

21

1

2711362438624

3

1

3

1831

1

1

2

1

1

384665467

13145

1

1

1

1812

25112121

623551273832

5

5

11115

1

111

41

4

111845111318
162256131821

1

3310231

11122

1

204529203211
224930223311

12

241

1

21
276051251627

275748251527

11
121

2

511937962603965452
120802269417468

57931146112334
59941146113234

219

11

651731

31

1

1

5271511164

5271511164

2313

1

51

41488155

112

236948345523
133229161915

10371918368

1621

1112

4121611

1109121

345463293615
193424454321437248

8191816275

123

221713810

211

11

311

98151223160232141
92135223112231141

1

1

11

515461

209740432714
1192123199

988192085

243283

1614465

1130

191834233742

22221

11911

1717785

231

11121

43

50121871

1

213

1361126

26352913315
25322012265

13915

83231

5112253

141

13395114
13394114

1

587621

297517246742

217233

217233
217232

1

121619971612100814551111
305382334227

11731923149494513921064
574861905528761522

398734386926
5791047555413612542

8124103

1

612493

2

16
5

11

124

21

1

3

11211

311

1

31

623
227654

1

1

121

1

1

1

15

111
1

11

2

2

723

1

1

73

261361202193183239
261361202193183238

1

51

58

7

51

441321

11132

331

5

5

11

11

12

116542

1

1

121

1

3511

1

1

11

1

1

1

1

1

1

1

3110119202214
1452492

1

1

32121

2

11733

1

2

1

11

1

11

1

1

11611311810

121

121

701657917
701647817

1

1

51

51

71
7

1

42765

42765

35214
21113

1

41

1

1

111

111

163
142

1

1

1

12

1351817
3464818

211

9

13

111

111

3410143

349142

11

7020791597264

211

7020591597163

1

1

455442192912

112

112

435

435

131

1

15658326149130
7237926147127

1

1

840123

2

1

1

21

21

2111

2111

3412

1

3411

2142122
12112

1

1

1

8

1

1

2

1

2

1
122

121

2

2

18446
8436

11

11

3

3

3

62
61

1

1

3182
4142118

121114

21
2

1

111

921
141222

5121

2217

2217

102136291817
132136302120

1133

2

225147251115218110
613360265727

646040336733

10054151569450

398838499338611286
1282501239718481

13691311317
15722234338

1382121

12

1

311628212211

921155156
68123124

3133232

228349415837
184845354929

112

3353678

11432414698212106
791211026612777

1211

12

122

549462

3019731267625

563160193913
522656142711

4545122

1

1

141945164117
234056234824

32331

6198476

350778705335585241
177445152865164127731354

32206

100374350134260129
100372343133257128

2231

51

11

211

21

1475019

11

83124135647985
83124135647984

1

1

1

34321

4

1

4454

11

99257738712335
96257738512035

1

2

122

6654459949016

64934

2243191

1

1243191

5313882419248
4912375398739

274257

2832

211121

46611

851331956520783
851331956620883

1

1

16548171

900177310877661248695
84115599876751139629

186411
11

18541

1111

116231

112

27331

1

1

101

1

1

1

13

61

1

21

1176326

1

37

1

4101210517

1

3

21

32

11

7

1111

1

18341517417

3104969
179630444625

1

1

11

1614581

1267719246

13668

1131

12

111

2

1

1

11

2111

3

6112

143

11

11

5104234

222

2

1

1

21335

130436216138222156
41220356

1111

9633314996178139
9633314997178139

1

25316
121

116

311

113

1

45761384
11621719176

1

11

1

111

1

111
1

11

125231

1

1

4411

1

1

1

1

4102773
382462

12

11

11

1

587621
1213141051

512

1

31

733

1

1

866690816843

866690816843
834582735842

32188101

26435

26435

10801226930579890556

173238297160243227
6791029830475699472

7676798911940

2408952510115

1

174918131421

252641572522

81

172545306220

32413472

1433

1

15373

11

19149991114

511

1

11241161818

8193641710

5092121852957

384390324524

31421
40119710010419184

1723

1723

72092135

72092135

454

454

51352115

51352115

61111

61111

4312

4312

41

1

1

1

3

1

1

1171
279124739213461

408440603731

323126402126
408440603731

8521420164

11

451152

972112

2252730239125

2252730239125
732124143519

13545

151315516

71151252310

724131

724131

641384209
73422

9311

3862496

1722124

214372

11

21

7171

81
2042122

42111
12111

3

411

4111

3620050394334
181612337219230128

211

48341204027
48141204027

2

755694283021

213644214118

452341071097528
452351071107628

1

1

1

211248403147247114
294873253423

648182575740

5345114195215
5345114175014

221

1687414
171118517

131113

48631164110329

372622262411
11519022211320470

53871585312035

257742346024

126491586136845

124271465534741
126491586136845

211

220116204

4917224425724

4717222415624

2211

449578393245359165
165124335617

6410029194516

464918183012

381932141212
382032151214

1

11

1

213311

404453442320

102235192517

545453336026

16222358407924

171578212818

382279448193929134
3514108415910

308938264220

84821223911848
77621103410145

720125173

228881258067635

565573421

294291275259536278
12

394369845615
394770845716

4111

255243205173479262

336290385139413192
431

336286382139412192

81046386
278179386122189158

8789184516867

183801566811385

254347315084

254347315084

12920521510423972
3822118348329

4614268539429

26261511289

1

1915146335

212326133103167929311124
117120120558543

11114132513519387
5368451049608886370

336545545368520217
322492515354482202

4773182
41074182

31

32411435

29371
39371

1

41933107

7242116357
8282816357

147

141531412414

2

377059134019

11

11

203433286020
243745296622

3312162

1

6715674

7269125476536
297277416186217106

9869132433813

221952352616

345942304814

716165314027

4550116447133
312247338190789173

1014165102
1013165102

1

1

223432171910
19223013169

3122431

38245154467

11555121

267622

642313144

17410312882204108

4763115

600873853411715300
301220293196229112

264440233916
263036192915

3131

11437

915265195

1321

42351884213318
43351884213518

12

9111

488172364618
498574365219

14261

1012

5927035204046
5526334203244

47182

475895216334
403082174832

728134152

232

21966161

72161

47113886110946
4310483599546

41

491213

13131511205
261251327229239132

27

523

16714518312311185

81911179310542

31417555

31417555

31417555

55001949027112477108076951
1442937408666804359152252380151538

647142193451

647142193451

9198104607983
339308576328338246

3830148645130

10131011885
121710418128

24343

222813228611

98841181126363

694474473237

971551514

205322

205322

908831437315647849481218721122441
1011815853610247052345713683

2157351869651920955971810

7509216234219711004

95122677312194

232228171169264592

457135318332
74137544413161

22631694125

733474

575294458352

567820205042

215651234125252175

3611361934189

235764297340

3059176

232286323227237273

235132103121132233
22210299113122225

133048108

364163

21

437469565543

9022188439379
982351005013395

8141274016

1

4511650367062

151316147130141166

15033290503226880610

13223244380470111

731671150013999722876182740101512
741821165434043723093185085102745

1015154244021723451233

13832910863231160
1232687952168137

156129116323

2342

1

276380132163230289
241305106134207268

5143167

131

391

14881671

11

1

10152212

1

32710878

2

4472328143411
2

4452328143411
3952127142811

251

1

11

1

2

1

1

3

1

1

1

324

2

1

1

1

3

2

47040577238820568332244421836
35268513221371735451416115179

1183713888881014661082
1243784790883015231109

411

20232992913

2331231

283951081911

64862

316434342734

536956348555

161

161

1

357305597417375

351728

40193631614

12203633212147574513
10992476204130526462

12111578174851

265122284239

202922142014

2

181175166108275245

131620233125

19516620210215789

17111191155

6654583428

15952319

16911343

93207645574142
98259726181146

5528674

54531812214128

54123115506446

158229194132246522

33883954155445219121832

33883954155445219121832
32043629852943018411747

62768992117

341133431324

88134212103744

1261125

151615195515

12516661006643895

357424194546

5943114

1842947189

12634215276018

133859151912

13286131925

35100153684320

114

3710490418248

10020016347175116

312777916

274816141713

91996811

8426496507488

9134791434

94178875017

5314317145227

1128777899170

3163373124

1771579258134135

287456554421560

12526101222

51791092921

2621974343172111

1046774017274126

8314166568152

436558448326

1

1

796296947816888
762222817315069

176483916

17105293

17105293

29620027180126161

142210156117303182

915382110

685634572565

5189672004773895424
1334036922518166

351465591687682
1914581102356406227

51695

1122104172319

3158131353531

10923130212726795
10216527510424290

396881

4572115174

271936111710

916321079
271936111710

18341101

601852147811143
167450497181291121

2611246185218
2611846185218

6

1

14311

14311

4372140416332
47117165509034

436249232

12

81

11

9427131211
24932252211

15551210

9163791515

5921664078729848917159226488333857
17572186480102364414478548410645

8181610104

8181610104

6734181563433757290
834313292

3689555114628063

47891877668100

250231782198380125

261259438179320150
291956172811

1151112097612252

374634125944

80831397411143

859021210223263

82762099622663
859021210223263

314366

16341172498810742530550

16341172495410722530550
16181080490010562474547

16925416563

342

3657543062712819101721544
16711716246811005400632

8211722264
8191521244

2212

392747134015

6078954011624

51511689319829
56731969622229

52228324

12142647313437269
13147048114138673

104487144

189395356108229124
187388348103208123

2785211

12675817535

33911204110325
36931234611028

323573

85131

5129408614641001197
500929855454992196

1

1

77554

77554

111

111

111
23112

2

211

1

1

1

121

2

11

11

1

1

330861489257558134
166951517911433

11566917538

613371719418149
2816994569825

1

41

22

211

1

1

21

5217

1

14

1

1112847265618
1112847265718

1

2514811

22

11

1111

1

11

1

1

113

1

1

2

93712

69367836119336
66365826018936

1

12111

1

3

236156178

841851888722240
871941968923142

28612

112172

1081114113

213221545285773120
221262552285777122

825732

161

283184258105981105
280184257104966104

311151

12335821286

14693132485821415290731948441
27765359571162773041913930615518

47995597192514819237052469

212

73179227287183365343

113

36065119229415172214442502

4

1212246

1

214

10

186231

622

36426107120963632165081623
35035317116133435159791582

16884915688

12370243418246133

2755084971766821140

75514109406635495105260565077
1135365735303062321061190

27132

577488719531

20953941827540585
20350541026339883

5124441

1224831

932431

328912223913156

661001486211353

811861519412972
11822816111914179

11871022

6232664

3011941

6333

46271855

262113

1

1351233989415050

236

1

40304017745674851101193522243

113311

52941

1449354633586

306610658954838

1

11953

136032342216

1

18911028713713473
21023430917418184

2112422374711

1242

1

12123102

18615156
84121303610

7331515214

91981294583114099
12329413746771240120

92482

3219671709219

1

7

23421

1512515113

26403262599042

1128431802773

7351124446830

61913257571127855394
64914447841177889398

2232371

11721923202

29
1112171

15231

45

122

1531

5242

1

1

53

1721

318530452328

296364916284415127

31111

102111

3

1

140101128284530184
13395424272226378

25741

4272156193

3201436132

8242

771593602441125125
771623622520126130

327915

1023641616

1023641616

238226872387141726411313
1662211249224170

1021481477722248
294750274019

5778542610112
5779542711212

11

11

213463

346653

3325

3325

11

4141715135
371613105

17122

1

414116

129715312
18019516310219585

1

46385472

466050293316

76123985311155

764466484629
1782415187

124141242

1

262225161813

761435

1442122

1126580438534
506350480213394202

2021602198012786
1991602177412185

21421

124

124

1

582411192015

406386194921
396084184321

13216

903179389741
9438845211346

1111

1111

12382

3631072

3631072

434650473133
312562449325465228

531751007514630
283451336622

251414842808

1

1212232

1329339278
1122338268

2711

245643228819
265645229019

1

12

2

1610288125

12217994126114107
695529534022

5312465737485

184462182417
113953142415

75942

527261

1361815115
15191816126

213111

1983761084

1983761084

114433243644

114433243644

101011158885301032523
307295217133280127

44221

221192

16237148

19334141

10466711172

113226165

122368188

187290223104199162
16122216884141153

17254

2212

1644

369411

511

1658319354

11

211741483134

146141284318
47215197

105420232411

17917882

171620191810

356425

332631162015
404941183220

31451

471241

2932

1

17121371810

1004716146314

71122134

14221112183
15371612286

1155103

5412108202

101823156015

616206198

242241205615
275458246916

332174131

82119221010

1215249924

574439

2612681

424943391627

424942391527
1

17810526

17810526

1102

1102

151212

151212

232531321019
121

1917444
816343

11111

22151428414

11

11

239438821006662786129259723417123763978329
133300105119215388179359127578334793

212223211524
372521742530221221102111

2491411279
79691197912588

371101
27110

11

111334

1

1122

31

102743215440

311

4321115

11342

31212

158103

3121

141214449

1121

1087348
1087349

1

161

12112

1211

3

324211

908692848631444509
330918692146185217831829

35477

1

1

4612

26825657

11

121

31

212211

1

21

444954

1352

1

2

892

1

11

15251017

11

353674

13111

2743

1132

17681094

632613
732746

2

1

113

1

11

15

213

1

14962

6431

14218

311217

1

1321

264101235

21111

1536224

1638311

3121

825442

122

1

21

1186952
1175952

1

1

2

4412

153

131312

12311111

1

28112918115

23364

1

211336

312

854332

32

3
1805298383328

13

1805298343028

3112

3

32

11

1

221

11

11

121

331

2211

1

221211697

12321

11252

5217

1

482861076

222521

121

3

11

46531

213

111

2781422711
2781522711

1

218

24625885

1431

8082337814
8082338814

1

12

145171929

15242

1117975
1117965

1

13

3

33

26516
25416

1

1

27682858

1231411155

611112

11

368203267
368203257

1

1111

3410197146

81556421
81556416

5

3211

11

121

22

962111

143641
143541

1

811841

23

721

12434634

96732

1

1

2

32

514730396513
1411739635651938958

1

1

21

1524810301943

11

1

2

1

11

11

2

1

11

3

21

111

115

948393391352699504
1163597563540826757

1

1

1111

61

1

311

1

1

11

311

1

3380267529102

11

19121422

1

1

1

111

11

11121

1

2

1

15310112310384141

1

1

1

34

1

1

2820201077

21221

1

2

1

1

1

11198181115
11198181114

1

312

21131

1111

445213

28126

1326422

3692211394
14227

359189124

21133

15121217118

121
21

1

1511

11

2764

1122

71162612

210510813

43

27312

1

2

41651

112211

95

1

111511

1331232

9151716124

112

1

166136149135122107
765949533926

11105211

2

11

21

31

11111

9122

1

25832

1312

2

261451

1275324
1285324

1

1

1968421

1

2

1628147

121

31132

131

2123

1122112

12

111

41

16221

2111

13

11

33274334

15

21

10641

113

13

71

1211

1

213134
292522463216

144733128

13201212174

281412301019

281412301019

933959492328
4122

711935321517

1820231589

2711131651087479

2711131651087479

2711131651087479

317262461764573340156011349195
225577319927372567234241504215823263640985

21974521116278324

21974521116278324

1607617261198108

1607617261198108

1508795906245

1508795906245

222061419668222500191237858615211803590510
146776912830611807887160999012144412739790

534211301208107165

228496810601193550571

1282055467464518530944106346941
393001768219144175221207015015

659364427842278324
11286307891193455629

9283138955393

652726312232

483138331428

1016951632871

163561091296081
824053893765

811656402316

360189204143101158

1

439188201166320126
433170190158312104

618118822

24421628185313998931675
24451629185314088991676

226

1171

1111555511010781

434302386291152211
349192298186101121

85110881055190

1490871556482367544

459524332663303924332466
305512811635184316311238

20659125

10512930614177

535341803269

334238201251220273
10770381264468

554811365115154

17212050606151

304115163174113133
253951281459085

20716111118

311319181230

3923131313

21016413918497171

443126393846

7037111442750

812361552419

169234141193103222

111991109382150

565277295252144546
774397372364216696

342193026108

1759968824642

468349207158161223

2955130418071101721947

630522303370270910741887

798731243853361634303198
1112552492437415481

845328525953

1093063378332

613128102109284123

429116912385212119251603
2453965161013321092729

965152865145
883442814539

81710566

47618132713

35882144

953814445217

3551110186

283204254226135261

711728112948
1113649233762

37182112714

311

212247

95262592896

2093462705041

2228985749299
2518989769999

1

28427

335163143191176154
23897105779856

9766381147898

105153044255

1134918309321
1204918319721

714

755232285348211265
1778670783860664906

44599411

875239405256

3531010135

575304359398311532
554298358390300526

21618116

4791211176
5491211217

741

1352053172519
2286569445230

611174

874415102311

925947492444

593222313229011207431853319039

519632009826226182101505115760
593222313229011207431853319039

312

32439562560
26356552257

683133

893388134288388257
1155461182376509352

31

2520213

6322

26321128

22332126

57155294137

63237292318

92862

315

18133137

3091114612

2394229349724

111

1

2228060908468

25

19141181613
234913201514112011981772

1085176715056

1107767564559

3138395596669

401441221233196365
626645369369319572

383744302437

1

31721

109108656450101

121

514131

628151

1

222

111

311251

65167

11

1

24

2262

322114111231

1835

91285611

76213

11

363601516517136

1565651694041

19811017115296151

59204915516

9723144

1686732491232567

22013014816298198

18080114989588
1

17980114989588

5041331108316881
4851281067715880

19546101

4851111112

4472312146

26145353710225

4145337

1
3058397788485

3058397788484

411142

641711

28552101

3244595

2132

290155146139157157
371179153148186182

8124792925

111

1480514424353838504

7161311

176514410

1039320611868367

703046211758

34111230521

3472225832

201130561102

17124

999427

50711167539430

414270366195134209

11

1265299592927

2

792221

138963510475623981014
138963510465623981014

1

453149274493

114624

5

2115

497240331190165239

1

24031527932172394

379148199163100144

353

430190274447122166

121

16161328

21

281712261916

692947281117

1081344578377230263

1478383311249

79989819

11086241323143

964056341839

103212105

17452775812866

331

1

113

343957334625

276278244165130264

1

7933772535582346315

61716224415869112

32410218817493222

18112912510570124

1317215

641219359214120168

33361238219

797311457137191066337937947

484519211516

304231297391601486

1153738541324

7621

301331261531

104326

8841728813461

3

241544298113

3416162291411

1214788542345

111

1

3951021631187572

4

26

760273499296148346

19250124543738

11

19249124543638

429167323234119200
16392743312009883252178417

581226407245155143

581226407245155143

14828161050772345417

5371952551671501955

714303489309196305
677276451278175272

1511

71488912

22712888

761213313

11

19506331327706386492
19526381333712390492

25664

2651105218121062661657
2690106218361075677665

3292311148

71122

545731254714450227183457
524229324536431325553249

2

6651348

6111

531

1

11

11221

30930162222

63311

11

1

8

1

1

1

11

1

81

32

11

11

111

11

1

1

4

1

1

1

695570604174

659201111

636249

1

239

10364416
8221313

11

114213

21

111

31

1

412232

1

221332

3217

1

1

1

1

2

11

515

1

1274

51213

1

132

3

2

435331

21

4

151

22

210

12134

313313

6132937

11

1

21

13

11197272

465224330229124171
25162227513

31114822210870106
27813920310067100

1

26919735

71

1296086944952

57316030311448114

2227820610154180

3351131761056688

955326587267175230

1713992482322

1

162221

1

3

975428743485366542

1623121116434220

1

2223

21156161621

5116368615

158189324186370185

1495504905490299472
24389993414638988658068475

938311484361194318
933308478352190316

536942

4821873121848799

1546466959732661337075866
1489463209251615834615357

21

11621

14191013216

3141

14111

2

32132524824

5210176713

31

1

2

806589803992

4121

9321526919

3472610216

523940293146

603622421621

1029410910568127

120571198756129

41912

427153201226214247
425151200223213246

221311

1250448768396298319
1250450770397298321

2212

433316342234161510071152
433216322219161510071150

12152

4740607148177

2143

764273522258166171

23312017418711391

615161857

1713831432367

41111

1141921213

392737341020

1

1713158199

451524261120

5634576262560221837

54186427238

80954848326101703011920

992949385089

22511

722317181214

33611825716687139

359239456459256682

1

12212

333

1816014610660434878166111190

16902416945

32

2311181371075963

6

24011526825316995

624957502462

1321

12

811426

70016339130187117

4804174891133269584

127

1645955271399

242516741715

96374324255079606350230498

641745432318

1

82591539

152631009037184

11

721228477281155165
752237501299161176

3192418611

12512612

1

1

4332296512340

31

1

491636121125

1

2169420214493251

195110223107233147

26832108363338629536201

614652862165

49305027122157

49119133859

1

7641033

1503429807435300380

802291740629196497

1276449771870279727

1

34115

277710431629899509688

3422132319931091767906

21

1

340236577312145921

25631172686890510471

1446691645095

18131311236

147105150135104312

255480015657684421041

754511417325196507

41

15676781156905301657

319695154

193551221103641

1022258

55183734413

331202349473178261

58128016137

6036265593679

212

31

1103601710405244528

505236432220118198

1

668336461235139252

121

61637043621482101486868

6023563593992114539

101214

111

1

1

36826116

403249879279853415

1035347608384179240

45171554234151

1

118623

111

145136653141902248

57134691316

2111

18207211942157

1111

221314

793140171529

1503513165022

545725191132

12

19674631935953

813651391727

375132743

857573

242549261417

7383006231798158252

620186343202121152

1168542375174132133

20581439182082205154438578

980448640367219332

493212307190110160

21510426515345166

1242573421812

955369592323142248

260219208144313186

42

29390164854867

1

1199678101014367015090350542215364
1131082331220119152354821548

417227392878427913064017

1083471646549229789
1058465600546215750

2564631439

800407302544273294

272242261305355542

1057703321913555033356
1178809336715375873529

12110614818284173

220218366424160467

5494236890836216409528110122873
5937840001888776953031511130753

719111238129

1810418201

615554434194

192765312887

504586755099

12847326110227736

9771182101169118

12411216513384235

161645254143

302042522440

79057193810627671318

16641086133319175021590

10728371778158812532875

2624613511120

82320

204226212

1251028215592274

214321502756

96321822553

18681194360826606235534
20171279372827877105909

1498512012787375

31871189173516257161688

7704610150161370313489174

20241520173527008883028

35241972186135689803053
359720341909366610493178

3429465354104

38292241320

142121

256718662297337910442811
257418752313339410522831

791615820

43071633213329446892285

17761091137014007491338
1419752885971484746

276132552372

282253403328229484

482550461336

78406575810816255213015669
80926793849716648231916234

252218389393189565

1103422638461258562

806265136129497

254263440326203838

1157449953679424649
1414627140510656911114

240170416349255436

17836371229

467236567255183281

501436503499115684

425559611448261495

264277315261186313

6397866129416521322
6998126759847171394

602663436572

1069681111712813101582

61061568

61061568

79428317259147

533168281177102145

7783190895794

1

864252601626

435183332183108126

1016373764025

2

712259274246122207

196183121206131177

343531323224

171823175616

722252494270157184

12

720249657302187232

51951

172831145336850

71349711

7440371066852

5340962835

24313436024173257

461531

1376844332670

1131

11

145193177181467649714679

607610477231781431648067011

338178212208164299

326111

204991787866100

333135187164120158

161161144272249191

770331656364244441

265184199181159234

1346312

9389164196202423

698207354189117140

907273632372181210

1

1

1

111145015812

1574551497582455626743221

990567383377124885

1

336518399269762601441

35121717533466058441

328971901395671

568226367254125297

1

5102493147016852631202

4122144

3671092231087689

266319751995310387202501

745975383375

83324844823

211

1623268323

522

237199315419240774

1477875574750

14051246141117807201587

16682104644646

322123211325

1

17311523

321511304401256755716221

725267485346251260

49147316416

344144279168251207

662326469327241309

592184305255103140

184

12

111114

5231

175138408238155430

121

2644181532425

1

94034341579

12643121786434

124167895766196

716

80301828535130

377131353

34514614513352221

387332373242208588

462013392014

111

1111

20647664210660581531424417

37916220817074122

33111

1

927215612047211

1

1312617825580339589

13387341107903520945

411342332836532372
453361378858584392

421946225220

1

599646

1

503619974357201011001314

471162278262219235

284177362193190285

226122021

669419501336230291

285931771194366

271602811014148

1385506937652375438

367200264214325182

1418271211633

96131714450108

1204880645530

4142

18155143723848

552270486747

1

1617494464144

1005792503541

622329251619

1622774353024

11

879297485318175221

1548548768379419724303063

112

20968301355892509854

1

1

26351244171617286171320
26901261171917396221326

551731156

1735410711311771

1

1

1

13161

211158

683159261418

303438494344

7482316

2514

18571258107419815421248

1538421825664315

221

353718874927

1326715834711434491

4451472451439887

516199352207124135

20911008139431711164324490

2

1

46333244316

2

926234

497919213059182010541318

43813521214071111

1255743104313733601265

416266447306445283

53173040915

14455391007639366479

89144810413

1

115443815910030

1

1

10157541106698

5032410821

21911225316374208

3928125024221193720854

677811121350127

541134

5139801710861963831045587

1538918188115105

271239423824

22627291171744462606

14411067116223635351882

542243452641

6611

5971584021515086

5231932521551642536

21310715416572300

1635385442260

663276421261124193

120671711521311020

212163147974042

636292426398224380

3671982011020146191

973563514129

103133231510

3465696603851

212

1281237221636131

1

1330559858419226323

513378767563262527

34121939104

317175217187119151

248310421497983456840

124122114148134200

235841961616992

1

2869131616001017698890
2869131716001017698890

1

11258524917113

724174695261

542847211325

2308017631298109

131139784816674

266200173200129161

1

1

1

9884129285511209522

188162340132126643

904955372951

656126333906277317062304
625227409276157226

12131112367

18270103694035
20987118934550

27171524515

712441548487307645
658434534481307642

5471463

173557
450917442558176610931266

446917312548175810741255
434816852462171710321191

22

1112

2021531

253

3

874355293359

711

13332

1

231053194
16851194

722

49412126213968110

2031391918711398

23

1311191584421392

664233406236111159

12123

328210199124105158

12561882161318375531264

41

17288961192674493663

17

2183451342160

1182025136169

1

994581392140

53131122720

944455680561310644

512331

452957272312

1

11

1396920911259258

11134

1526148

346155180230116188

24051898212731137583253

1511654958770434546

2

298028723398566524193362

532136191921

1184043473069

62153728813

620216371205137130

3

6361221811

4810331075

29514411714270119

13233

55555104472561

2

246352696

2229111

263184295208352314

3

1

15715

3133

4079125723742434

41510721418576117

703837311524

24278141327979382518

1

7320112911776382

1

32

1383324425915

13175944335180

12

141

1637115827215496

330249191381376181

195122232150132219

110141126910

121

7586318746

12

1064567634659
792436312621

272131322038

132

12610428116177337

17790619232138

771275442276182227

2841322206

413369272932

1526231

768300496458210263

21

886268532248144159

254192314286111343

432210171516

7716696014435

1126522

186573213397674911082

1913192614

1795527930508337419

1771852181210

1

838439975555449958

1

1

341735231664

324413222218

455130356217104281

50394818616

703285606075

1

6381973

25559151534853

11

151

181

985954156378

4642914733143

28981086147517464951256

63805112703337285105345021

111

806344449464233269

16245

1927054916060680

1

133859262173

12571100633345

392813391083

186827435870135

1072507770525270759

5211

2961504651735739102

1

41124

28362136290850358754233

1199394781413239314

2023581572057

32

1728416313248102

2347189258473222114369666

3539131542543

52

7843261154

270227432313361539

392549111419

199111113947894

7675311175162197

34413722735888185

23768481737691810474

82717610653183

79161077

211

1436274423

15

12

973309517452212358

23894327

41231714828

141114235198118366

323615172021

24722

2952254912018

11

843543066

593432311119

11

1

22904186151764745885307629679

425195240135113120

1051151018964101

30112222311011890

82360381413133381281

2

7568984638128

793150421621

1675561352639

112

1

16393721910337847569709022
11450711795823

21327521238682776755

323717392406188116723017

567624923778345826562966
610926684075378028083316

1323453373423

21

1176812510355159
1095912410254152

891117

1232648213159

1232648213159

61467116131109

480120102547195316561911

2

32122714515

701420527

1919616820783392

44622

2688812411572103

612116932425

11737

1

1

8489546517256

3831122001516569

321

31282275147930258161927

17679251130210150

64284443

816331

1

15781115125322685901421

287176

514184252306183240

1535495693857

38410018320568306

12101111610

8769951167053

433986172934

16155495

163130344354310226

5635538178092761416

601439221113

3

256225514221184355

24621825201428919972252
247218272019289810072259

10257107

10257107

21141

1169811419855320

3422

329131239230110149

12

2148115310247664

248311096

1582

666693549370366941

17429713431293

351613173311

1161744610

134

358156350171150138

617723233766226416611453

1043241282626

31298348

21276125813830

6103656

838273501285189290

1188302420215365181

11210972682651

41381623

47355025832

1132391699369211290
1120361691343198269

12308261321

254247222559

172238631352

511846371620

321

40664111974538

623340223038

116171028720

1457407821553295376

12121

170108193123104104

2313961136741314

703043421422

11

1

893381541393210508

110

215811631075995

30813024718892180

33942

622978132225

1

1

186102192346138411

62832135088020607199714722

7

810230451284168156

51183336197444279732815

544167571630

11

626386289543218363

158124252

12

652251452117

2467103816541178583893

8858599077200

355132164

26747931294900460634

23860799311091

889346490406229350

612032500121133317814219470172600

351037193253

2

154043532289

4554447210751

11481085

119

20248731048587446616

221

3420345624587

669345990274297324

37612423913373109

9824431881513

9324

39926531

1

1

21306253128

576479510569168232

1808543831616414405

884383526367190435

13312

1591288521263168

442231191016

1

11

54374313484103

392080281621

8213103223641461985

267178237441108227

1

118841559324794

25313

56211334

2511051871375894

20127441110770560878
211885012108766571015

10610610010697137

10610610010697137

2111

1

11751340112915455281511

11

2

14621524

312410231847989622734

3541231981127090

3541231981127090

1
255093814841016625713

1

1

255093814831016624713
3341462101377295

1

620223412314142205

529150252162135103
565154258164138107

3646234

1

1030415603401272305

1319523812513288360

1319523812513288360
586762

1

5271987

19984139805475

886330519292165210

1184376353227

321822

382559671637

3121

82842

27

1112

1

1

13294101838560
392723523178218716661663

32163632158

15512752

122

97162044

746542

924476376123

2613171467
924476376123

31410534

561241

1

2415121

3317211

2491412438

52121

614463526325307382

614463526325307382
160115174117129138

42142

9319232

82343

1541113738

321

33

211

355211

1033542
21532

10121

46715121012
4571311912

1211

171465106

211

111

1

63522
281322431

221017211

1131324

12143

14

113

22211
2211

11

223

11

21

111

462221

2771822

111

234

6108321

1

1671314

2559732

1

2

19133

91032416

17
51492

41422

1

936621

1

301018797

3761719710

1

1

25

31011221

1292

11

31

4441

41322181

111624

15541

49162928610

2122

11

122

2413137128

221

1

11

11351

1822

22810435

3889344

81

33422

1
1286584874544

22
31223216114

17510961

121522753

964352713440
1285322

42711

146225523

4222132

161420471

1243225

347209146

590411556339286281
1

44201555

44201555

261951471286559
586407536323281276

1

11

212

81

3831

111

15

6111

22

11

1

1121

11

11

1

1011

42

11

132
232

1

5227883
5227873

1

193
93

1

1212
1242

1

2

118814713
1126969

62514

3142110

47105329163

21

3

12

1

31

1

24381

87

1211

131

29142108

11

2513415537

3

12

13

11

121

10581345

1

112

2

14212

331242

4

462112

1

20135101

1121

11

48234710

11

113

212

4812

13

532422

383593385190
6040114465691

2252175

1

1

2111

13

16

174

142

1

3

1

12310624

4314

1

41

711

61

1321

82111

322152233
216223

111532030

1204567815036

1204567815036
19314463

5121

112

81710455

11229610

33373

2322

41221

8612392

446532

1236558

31332

6281852

1
1671211291019772

10118457

10118457

333327262726
157110120979265

1311

435421

3451205

11

2111

2012221192
195181192

174

11

1

4667

122

414242

111

4021101517

24716621

17321061

121

225318
239319

141

4222

5121

319263

2531151921448685
627133

40172629126
33121

3311211

34112415114

2210131686
112111

63112

11

6331011

213213

623231

38113116148
18586146986370

4

1

296161125

114336

4132

316154

1

44262

2510169147
2511169147

1

5911717

121

572

233123

824

111471017

1287233

1411071

212321

17312535

7811615

17999781411958634672
14152218179

220821381126558
1045525727506323380

13215714

1923225

6233

49162822

14224

2715199820

31911866

418472
418462

1

11266

1111

389301155

2357825

241913933

1

391432121113

1132

19315722

742422

3515141564

15751521

2

23223

71

35112

42

1149885

614056633772

31787414

12

3466516

431531837

265191024

1

1285482

1214711176

667492

161

111

826132

2965155333

552311
652641

133

10726266

637311427

513671

34575610

5112643

341022634

124

1

1

3141

181014171210

72447

733664212818

304912885
16812733

1441152

34

7102262

7102252
7102262

1

353133161925
25151851013

132146

321

1

932513

6121

11

1211

2919251386

201113954

9812432

251157

251157

272327191915
111411

1615137105
161513675

13

10713889

4511126129

4511126129

3110125104
22911433

61111

316

422232251710
824311

102732

12321
1114542

112221

23

214242

19310722

213531

11
662856552219

601953511716
601853511715

11

693442

1
7151116483325

9112

5144263

17612658

136161444

1217481784

14628854

17132

392027231320
64232

54412510

281221888

841258432025

841258432025

6142721311

6142721311

293626381223
198132133926367

1

235311

11

312

45232

52

22122

24916596

112

1513

911

1821

22146844

122243

511731895

181314877

264103513

11422

428221

563497

563497

534488573338
6214321

383745

10841943

16148151712

1
11

1

22

221610

8311235

678232
678432

2

212154219205127287

1

53852

10355343

564944513154

1

1

1386215814188225

1

1

1

1

1

1

214431

2724105
986504711602461452

13911101116
482251300229212201

32619

32619

8178995
474526243322

16292

29115858

42111

31211

1713

162235

311

11

21
315176105

22

31513693

2801021371008184
23241114179

72121535

422330281810

4811341437

1455322

231612896

12

917201684

187131067

532311

849221

117321
182027271612

1121

111

22222

254442

7454

329731

141421

11141

86552611

11222

5332248
7533269

22121

511746182024
1121

458347813

15856

313121

127253

33132
593531331418

3511

221

14121

222332

136122

241431125

256131513
257131524

111

121
341313212121

111

1

11

241211192111
1628635

1121

1352

111

51

3

1

1

1312
4312

2

1

1

111

222
22

2

11131

11

819

819

382200337295191186
234

171550123434
454255

223052727

132221

1041231

12

9916148

9916148

142316281620

142316281620

523054395026
613122

1

8781022

610910146

1371413177

7112468

124819

178201164

7791143

1011121

123202148

123202148

221911311646574
4310271244

61143517811

264418413

1413181572
1413181672

1

871315134

432124441820

262210421120

38183019812

38183019812

1
483250442837

81096718

81096718

466933
402240382119

1841117139

14813446

12

1121

38511

2

1
1367347

231

231

1343247

1343247

1231051531399861
1011

21
8077112936632

22313121267
364547334018

11

3151

422123

111

35231

642112

121673

11

423165602614

42751
423165602614

1246322

1115

3314621

912621

12191423167
12201423177

11

6

125223

402527222524
23131

118126910

118126910
2113

322221

643244

26132

271712151313

41
271712151313

1122

921813

11213

31

44831

510456

118822

118822

118822

118822

226642

226642

226642

226642

3780524913831436634284909
214

78033011053092714783109
111

642032
74932801050690714563096

3153

3153

3153

197108256102927130
11

22

22

213

211

2

63723
801350201015

20421414

1

11

11

1

11

20617643

3139213

1713154
482045101950

233253

20622417

89163736

11

11

1

1

1113161042
64751596789765

11

1

1165

1

5252

1

1

22212
1221

12

511

1

1181

211

4111

11

2

1

11
222

121

112

341

12

20121031885131
1712991884931

1

21

1

1

1

2

1132

11

13

3

11

13

1
6195261114

5195261114

2

1

211

11

1

911

1

2711
2731

2

232113

232113

232113

5413165102277995222961
62212

4023112100996994772913
132616292217

1

11

412382

1

17594105573159187
212128988090221532

24315

31318818124928

637392

2
112

11

6121

33883812
33883412

4

11

21

2

2

1

1

1

1

1

1

1

1

1111

1141

323

241

32131969243243
15329111795481812344

28149639302731

10101457447

43431011
23365123122

2

117

1

172219204

372644

24232716481775

201075148

1272

434333

9612420159

511

1221

1111

124
24

1

11

112

361134968
872278601830

19

39310811

531

11

12

1111

7341347
733635

1712

142

41821210

42

121

122

122

329531
522446362616

23

112

113171345

23

142111121

211

2

11

3

3133

11212

11

62

41

311

3321

31

1223

431731

1
431731

11
111

1

221

221

111511

111511
11141

11

271823121811
1

1

1

2

2

3132
261723101811

111

51321

113

2112

51017

3

111

131

1141

1141

1141

1211314

532345472156
299819473301343519501800

17281886
109073411671307852641

339251436392263233
1141

471966524432

351453433824

351453433824

12513968

12513968

222172320263165162
11234

16

16

22134

22134

792486643230
111141

391142361312

361033231217
391243271517

321043

125141219186123119

125136218185122119
125141219186123119

311

21

1446769

1446769

321524301511

321524301511

321524301511

374426433927

374426433927

374426433927

363843523922

7131616105

7131616105

292527362917
1

191520262213

562663

1111

43341

1111

11

1

1

44265175
608375576739479337

13461599

13461599

13461599

1

1

664262754030

57142893
664262754030

1

1

1627

33181417610

1484966
1434663

533

91735

571616126

18212515517312596
1511

391

391

454625373031

1211

1211

444425372930

252243191913
136761161359365

11151822920

2424

2566205
1455184

1

111111

3571713104
3571313104

4

153875

462428652418
442428652318

11

1

1071717362
343200327470288197

3182020128

3182019128
3182018128

1

1

100781051509065
302330252415

563251823740
563150813440

1113

142324432910

181224551827

181224551827

1281914162
893492846846

1

8642251913

889101313
88991313

1

611221352018

381625462311
9561691446449

1281339411

453731593727
373330553421

841436

89681031056243

26192733288

26192733288

25192532247
24192532246

11

12141

634976723435

261711341116

261511341114
261711341116

22

373265382319

373265382319

7455124914541
13481

216541

216541

216541

322472401820
11

711161015
15211

541084

1141

11

12552

12552

2220774

2122

1

1

119752

228311899
62

20727366
20728366

1

113313
213913

16

213143
111

1

1

1131

1

1

1

372639371817

4111274

318
3182

2

11133

2121

17692648

17692648
926542

843216

211
31111221

2811

6

111

11

2

1

1

1

1378754
943

22321
23321

1

23

4143

68192269

68192269
1

123324

123324

11523

11523

36151422

36151422

287254
490314524512286350

121
14510911413275148

9331135

9331135

8612322

8612322

1774933

1774933

111
405046481654

313511

12651347

83343139

7181221

612214

37231372
35191062

2431

19111413156

5182

5182

1410145154
1310145154

1

5225253

5225153
5225253

1

221721292024
111474

2810458

936755

232311

1

8211016

11

2513121312

1513111211
1513111311

1

1011

134312
284183356341193190

2121
271013946

33652

1965121

31132

527873

527873

527873

1
832462

1

31131
3131

1

11

2321

211

4619657
254357

12

21141

735732

735732

767921

767921

644641425

644641425

5936701563445

84183152

5126481491442

5126481491442

64421

3

4
271831131912

36495

1

1527314

21

9815993
5811472

44521

2345151

2345151

1067948

1067948

51852

51852

16818131610

115121117

5362153

15101015611
11103

1394464

1614

15102230521

522115

1082029416

176161

176161

11411782

11411782

1
584374343329

1

84123

11

3122

3211

307181125

111

13

31343101811

19424

21

722122

253412

11211

11211

314721
58144635127

12

1

1212

203592

2133

1

811

1

61

3

1

4

8232

42221

921121

291

113712

321121
382323478425227221

11

11

315331191726

31312

31312

14116
285228191624

105813
125813

2

11121

2161

93263615

3101357

25261848228

25261848228
3137

64528

4721945

1377412

2532021

182834341718

389453

389453

314417510

214411410

161

23463

23463

66511

66511

4311261

311014

121121

305213393323169168
2410551

4932

3
4932

4632

222214
1

1311

9

1113

1171022

1171022

22441

22441

1310328

1310328

45922
7191624

31222
31212

1

25

146101479
112

724342

241613

1322

432
332

1

266168289279143143
181648272415

1113

10610773
1069743

1

3

748932

153834611

21211

2333

44632923

1241

173114

133522

3282

811543
811553

1

122672

123412

775662
764662

11

1211

1553

11321

9851038
17101719915

14513

818454

4423

3611

23123

418313310
418313410

1

121

817626

6481082

81631

25272

25272

31416113

311543

75132311
55122310

211

211

11164

1714

1418121
141812

1

113121

143221

63152

11214

72473

2381513
128612

1191

1732453

342621

1231

248123
148123

1

221422
322422

11

21

2211105

4
2211105

121145
11

112

1

42

1

12

432277560580299230

323271

323271

3353132

3353132

131137131

131137131

8448771136035
412269538567263225

5215

22231

1

11

3231

11222

1

21

211

3643

55293

1

1

212

32223

12

141031

121

171214723

2321

122

71282432

10941386

4125

2211

11

1

21122

18234

113

1311

1145

1959656

8431148

121

32343

2142

333

1

231

1

211

18082

11

271244

417221

22137114715

211435

14

111

13214234

111

8

7

1825

212321

111

13222

2

213411

2

62113

2

16892

111221

212

2542

11

1

11

133

1

311

11

12

14211

97472

3211

33341

1112

7565116

24122112912

142312

1121

1111

22212

1272

4411

11

1111

17361161

421

21171

12

722421

652

1121

421198511

12

581524

1

1361

1392

121

23321

56843

114

1

11

14172

1

3161

11

3

1323

123

1211

232316

11

81

112

23121

11211

12121

12121

11

11

449198345438186242
924527

451426471027
143942216

722222

5111

1111

1

15231

2211

246

21812

187731601697387
54511

12113

213

11

12
23103639237

228963

103

248844
247844

1

522124

142759

224221

224221

111
151561081224674

1224363
11161

111422

14125210

563057332727

2616922

23912752

81231115

62133

2951510312

51542

51542

208109155217101121
1311

181175810
1

54315

1374475

114121154
81921

2211

149132

231011561918
212432

1122

111

8423967

1235566

5

1122

21111231

21111231

163601231326688
203113397

111

1621

33742

175139923
176139923

1

48333

442522

11

11

634291

22817556
123

21815256

23531

43124118

5610723
56101024

31

27271159
27271059

1

11

916512

2251318310

21622028

9620452
1812309175

414

728473

1

11

1

1

21831

21831

21831

183981441658591

189334
183981441658591

833169953231
155761181386974

1012611

1

141169513

1

1

4

11

2

12

761346

365

111

1111

2131034

522

910248209

1311

3

271417241313

20881075
271417241313

445845

324623

2814718212

1

11

1

26121710

225

12

507220318298192163

111

1

11

681881012
506220317298191163

461126610

1133313

311112

862

13384

243652

61381425

1

14121721113

21011

6810773

8452121

491326351715

328103134956955

21131

8763313

133443

8111

392945503121

612313

232633

11251745
432731482221

4221022

5131

1

522473

310431

1

547516

432232

214231

123

2121

1

2504589948036189893712982112302051
1862768265658598141919

1727847166223497491021
462793511931

1459713425759106
654546732454

1

512

1

224423

16327522

333944

111

172121123

6741611

334106

3465

710123

1917

1

121

11

711110112

2111

23232

1341

331233

1

1

112413

514677

2

331

323151

1312
1075716729978107

11123426915

6101511713

52191522

22161586
112812

1

1146

1

112211

3

113421
301023731817

84426108
95426109

111

204164367

211035831936
13122

13821681021

721114713

96121085
11

565955

12

1

3411

331744921526

331744921526

466111315
567283537983261422

1193
391528842732

422311

156141643

1529271014

4633074

533020311627
219161184315120177

12131033428

18149784

1441125

356122

319221

12417454

206171001139

155523

3131

475372

4614976

691921

199192153

522

11381128

719713614

242668321

756878

6313681

122261053622291

1001661286874

221044761417

1837521421179107
40103562823

11451222

320513

1

2268452

176634723

1711

219913511

1220671

5113

41312161214

591271512716

5720657

467231409451155206
88141567

612564511136
4383427511

1251720612

43845
44845

1

2858

503246322422
106561001014045

15533533

7651245

432116

21410715

11101

834411

1221

1861

518644

317413

21231

2319251141

114311

201375

35433

22523
129381141044655

28111613131

1

58125333126
58125331125

21

30928481031

1141181014

1

361211291210
13584841634458

244552

1515812

32421

3531223

30353252918

1114

3131661

431212461217

1949017317610096

271934371619

271934371619

87202394

87202394

17122012139
159641191167573

64184118710

12515251925

2211

582331501727
592332511727

111

541110181

201621491327753

442131242014
201621491327753

37331412

815201134

811527182813
811529182813

2

11241

7271710

31142

223838

11

1

261

711862

66

10215655

142

231014842318260749281653
12671615

142334352142
74452692710914270772

7181714515
4118327

311

1

112

1

462
1562

1

1

17422

717720167

717720167

229761712904579

229761712904579

71410285

71410285

536101237

536101237

8514191510
11

29131

433555
433755

2

4742

1332

238104411
10424801894162

1261

5410491622141

26421151210

23

1111
8610326

36223

4533

444621

444621

1298423

1298423

1315161743
13

810141023

42272

185755
5171109723231

486090592224
435482541924

2252

213

3223

2314642

1

111

111

767311017784179
2742404404103930507

111

11

42522
152284104

11202102

3

12157
14158

21

7453147108357786
534412394356867

1

1

1

1

11

1

1

2092212817

21

31131319
31131320

1

418322450

312
314

1

1

1

1

1

1
3

2

11

11

1

2
12

1

1

1

2

663349794678
663348794176

1

132

1

845482
833312

1217

11

14221

22

22

11
1325

1314

1021
11021

1

1

2

1132

11

1

9731

1

318

41

13

1

2521

31112

42

2810471

1514
16114

11

11

1

123
122

1

13131719
232815143

11513424

14134

262039542727
155495213841500657876

552941681721

552941681721

731533712
1154173411720

301428281112

121212656

241231241310
21102923106

222113

121

37101925108

37101925108

5954661062642
12

181833521613

41353354829

489422449439255399

62844192712

62844192712

427414405420228387
16322215313174140

42122447710

22815191383

10251115

187631318

304069622730
304069632730

1

27481512740

629133

202513322415
202513302415

2

15333

52212

25112919278

2691971315

302441411617

3128

112129371111

112129371111

545150514437

545150514437

5712381878

5712381878

925465763231

925465763231

631460891129
208561331944066

2672323911

371514501114
361414501111

113

187313

8112292989

14835
107371281475460

421936681118
472041711629

5153511

461779733331

11134021297

11134021187
11134021297

11

633646491237

633646491237

1628971
184102273

3

222102

965057962965
991329215

43111

873741662649

323070322724

121923101212

201147221512

2420239516135285892310974363296107
4166200460701298918384312

9539841022928
378021004683971019192184

165452101837058
261216083492837813551562

2143464275151108124
271

37281751003832

17731825050447091

271432342217

271432342217
261432302017

142

142

121451341346736
572250667756291423

322124309441122301

262

851

1891825108

1311

234355

2711113952

2521

11

31

12342

1224106243131

91025211512

135719615

2281622178

24886

11212

714081563926
455289466395193226

262030532021

156137210
2372022213

817153

22251722112

591538581730

7135123863839

2334153477

14493122436153

16202021825

2733
331232383020

8412271111

256138169

82321301583154

82321301583154

1

1

18121021613
101611341925748

1

2812681001712
3923681071913

11131

1042

28101125

7681654

351138372513

111617201910
13385231232116130

572560312828

6532402142

161843432020

432179982830

8941991371555
320197510543154191

7059103914029

4932101713435

38311471024036

6132531402235

1327231

761767662331

110111
11011

1

741655652030
751757652230

1122

130882201947278
1

6526491002140

373283392913

273088552225

23
533261455829

1

1615118113

371550364722

1

231119248342104116
472851392529

8226591012237

702461522016

111036981514

15152641197

6161511313

202156492617

363025101

171526241616

8360871114334
100740610121083468530

1791833821

3

22

2421

12131

241131322117

251347483018

1416631

7523100571631

20625381321

11

281620153010

11

24334

301031282415

44253750914

84151532

2133

1123

1026522

921

71092846

67229217379121

2363829116

2221834913

201214241622

671871462127

123

4185363

333852121186667

1206

112

11

44675

291431752921

26103211

21947751131

1

6647951476764

732561097701892341595251
432638770992460537

242114292012
97565

1312614128
23313

11931398

223934

467815

467815

12171211108

12171211108

2019224821134
18417019624487249

6335611033372
6537711173575

21

22101222

639063521420
639163531420

11

362340261720

221101131737544
382311311313

18378120426131

1

115896
655863966244

262818363416

262233472721
282537512822

234411

254723372734289616441855
432506480524343348

1211

10713714215475118

4647624016667
1735192012220

122

21

111

4

1625554

7352

1

111

12453036

11

1

42

2

2311

3

11

11

1

76871191156361

273216258190136160

403158492729

546571745756

1211431121295894

810142

9664100714463

584436771421
604539901425

3

1

1

11

1123

454375642422

17157112844692

12172

4162935

10588991343955

465854685230

104123126853599

222354372526

203434341626
203434341625

1

274191264448120223
257183244433116222

1

381

1

1

1

23431

21

1

11281

11

521

147821341487580

2217138212

111682

516867696141

157901561316165

355741466538

383830792215
173129712210

11

1

61

2015

15

192848271714

192848271714

123790411701271520671
262719602319301511921744

22

1

3621

1

18

1

61211434

5521837

875727311825
875727301825

1

1731211313

22

8875952403084

1

12136

324654313023

12

20251460542

7131

8695881104499

2121

6118375

541718614

252

377142203470148329

10056641022748
10056641002748

2

16382648

1

1141329518212878
127135107193135104

251

5322

6348724

1

1651043

306121927

261

116544

1

91933322114

1

2030168223

423633292115

1

1

1

1

856961715134

2

5155628

585582582231

11

4145414

7469811093864

4221

17137424

1311

623229222022

211

1

412311

625142686032

625142686032

2523151389
2022111179

51421

3771173

3771173

846876
301030612214

64198137

141140

214521

23614421333495127

343782973324

20210713123762103

16616217919962123

16616217919962123

14820191616
167163434265140157

121421221719

4728175651847

413840683723

2327121393225

304857522027

71271373
143692022016284

5530891212346

813886673235

11361052
137641181385473

693381732344

572831552627

2218016724281117
177201873

351241623721

169611061623793

341163282593142287
3281273

1411

1411

212133156310104204

1
212133156310104204

212132156310104204

126271142703080
536884

214211

1

24213
3251542314

301134114

8717892181861

3574484827

528451331034

21

91246180120101333360235159
491267779866328270

137561512007672
503286748889300420

25819531333

1675950413

353274802130

662759614346
662760614346

1

2

301957362444

26201281

63141659

393441731542

242

4416591451052

1

11

251868452221

117272795

1

242650263110

412343631941

1

15982892035
9965232246100112

351847543424

49381031034653

282182415435169166
281742422424

80601431434668

8438931204448

90671371305526

91283152927

91283152927

13931431014
452244541324

2951192

382238

9131948216

9131948216

504391579704244324
824397702729

301629301412

673117411213

53101541

263440541923

1591368

623474772228

1021717314

121140182239108136
116134179238107136

34311

1

2

2

1

2377733

7122

2392935717

2

11

12

26395

30202640916
30202639916

1

15223654717

402885782523

402885782523
382584652521

23122

11

73411081014645

452297913531

281911101114

221616211
362203567549144266

131122

572593481222
592594521322

2141

1106216115961124

5412271312

136811751682560

39481945

151026591310
221026661415

7715

241032311015

874381913642
1251

522656622321
542657712422

21911

11

11

321424141219

286825804300389228531252
13791162193415981723461

7108883

10113416186

88302435

314

4829891133036

294728211115

1071612165

5447751294851

11111

6631150985430

448411498666322221
446407496661321219

24212

23

218164149

2529931223

23182724145

333389674022

21172317303

714239124

1035272655831

434456966645

61321801013935

6125114754925

91541252

1

71431621564443

35929301111

10731107943657

3233102783323

50403022106958

71121

1631127411620

1111

664783583612

29935189

441

127791612603281
7773707931319324607

472219459880240449

11

199201053

18391094

2459828

302231531529

217553

31191431814

78132261816

384195377386151147
9938108924429

74121759

201424281617

10117237

11107937

1119181573

384201115

213322

732356821721

12

112

442136401317

3730842

2

1622112089

211218331711

269222388

113

361722561816

361722561816

1
163526201516

1

6271510812

107101074

451634661720

451634661720

794562743359
363334322325

7644219

24310613

36112

93821512

441559261515

26112213813

184371372

101265912031676560675
360226404597184245

1791822

41724

22114105

15113

201932524716

1132063

382445223019
392446223019

11

1821

25

1

1116131795

1

29321

7102016128

41361

12158

4611

16163420927

252499943533

11141134

101822361819

761021610

10284242

60542154

3213733

4162135311

491749871138
321236531017

1751334121

221826

222344221410

192041261216

2124341

26

121

1551411119

442237147653

1611411

56151021

974572883745

741656671626
741656661625

11
1

1

125655

182435761720

861924117

2

11

11

8392161

1

2354

69151213

27211223026999165
763882993343

1327123

522259

311

2122

121

12211

11

10310631

71111393

1231891215

2286924

572563661828

1182420626

612143

1411

1161102

132

222

312

11

31

12

531

1251

31613

150801503877972
11048881204526

15221

1

115

11761

823

215

431252

161418

53454

73215468

1610224589
1610214176

411

12

2313223

231

79845311

79845311

353176475348176139
7349144936346

815165741832

84421421003441

2116532875

1

8275324197

12111828358

1
83581281456567

152141541732

683786904635
683787914735

111

74251845

74251845

772298532719
11

1654223117

601654301112
601656301612

25

21
1021916112

31

32556

1063

72211

1991102291789078
27152432918

371023834

269212

3336943
3337943

1

341553

2213371

29912131510

34111624

219881

28471723

182258221118

466519105

136446

1321109

119106842464869
319151694

10211825

511

10680672113760

1944765711129756284002146522266184
1388442863707165269311918818

291137336648107258
9234219322168342770

1415413430776104

121

492336841730

4710271091854

8441362243572
9547522473894

1161623322

95211591722067

14777712743783

3328471662365

252368160615

311737592831

311737592831
301732582631

4

1112

1123625054484131

1123625054484131

22237
19267248539132163

60271072054267

6823821594940

6217591533849

3541475361032330403
31816188

5116802195350

735412520096135

9232861933159

78171312175089

57271061878262

24292918117258624876982752
7109614422371513004519187450

23182964051252

1314365028

9477292885017658171718

55942816300976086640

33

93442202439971

12355

59811321344

3121623820451885291

83262262598397

33118338523266110325

1205938146230154

451480206481118965525

5745701925410892197690

11145031254087212621078

16213641627132266101252202229440
89391228455175334797758126336

19816128

2023953156857735210228655

34512353

216667483348324

317078120211292494193

12143082734640292127

8882341573268111261

64261352183463

44786412119428

421434

11342932804533211529

863226643497311415

282631381116

27470139924310

132515263219210

29813756867111250810342970

339761195816398

7311951733029311005

338874310294462

15431244577231

65315680239861033

335699179513515795173

16937227615275

17644325781253

7716331310223

10720254228173

53

144040678354211321853
138138473052261311794

592253195159

241922471138

579

14434403607149

109126469033041681023

1

14451487743125188

43512267157213593

20143520

29728330

7022274707104

2

1

22245733158727533265

28776611605240

6

4722456172

9512013053667171230

42587167164225565

462833451054204440

984988968743100

21047678763269

257010632434498010781713
99941310711591349542

39525412318024

2812353625

6516401011637

462045622919

11391527

502734971313

11034851483957

8337961203466

413136260101780395

1108315

16111448813
17111648813

12

11

103171583

177647724761100

9747591503643

602497627032

481417112236

552035812035
552042812041

76

6033761234560
6028761234554

56

412380863330

1435519569541149

17920481016

1

1

1

52518027902895207558

4231442633265496449

10236157240111109

1

11286443123464283041325
664031231632322364370826707

4

9763368480142153

3691185061139681225

113

2481488661286185402

4011103671195306441

663921417037960

226101773945989293

232110421900263239

3

98038217545331106

34422417061727269415

33781235965233388

20579462430186162

283542894118916797454
324553897119416827456

411135302

2

99382071296235107

1076432638532294

2341014421009384332

896120226974988

21222

304937461264105336

178953

213109879118981292

1701103345971986216

72

72

4

2491023338881668305

14171915

11114102

2121327277952404263

228112149424550375401376012730
344893067602880618302472

2198163924601031453322128
288150257328110154

38171128417

692658512325

5141176

11

1

1133
111

32

672792652221
10851119903038

21

40222722617

122

178233878

150412871859957950891750
148812821839956650871740

3133

11

124

12

101

4

1231

3

51

17212

212115331030

41

281927551028

115312

11

775455572252

714832

3011732415

3011732415

873844841524

358162746

482527391112

451186

2911032232187878
45727935

13247111974638

793745531622
793545521617

215

351240591313

181624191034

4163

141518161034

36315424927192139
747352605747233333

141329451815

272525612315

28222

1471117131

32243251714

22161945424

86121264

174183826

1104192733930

123

58162767823

342254341217

28715763

1910142096

31

2213

681422596742275341
19521119157622048091039

1011821

16272518465

434648751925

171927281121

9910364620

652136311023

151515291124

711858722018
872068782223

16210625

7321313

25143833719

8622221

4211171431

403522531636

1

18141236723
201734531237

232217514

732026531329

482764

1876795943445

1

6236103691862
6536103721862

33

1242760571447

1491611712

8331511

1238710815314462
1208310414513959

344853

582836521225

430591616

86231662

1987681219

301732067

15010714825249107
15010514825249107

1

1

31216437526376173
1836197823202543650957

282745272214

14112834126

29116725920

623060673228

9162430910

9201560867

22929291510

9139551192743
9147551242744

851

165942341312

364259982119

564180792133

3701200841888206232

632069401216

1512

512872481312

25233555724

203421272627

6758891271865

61111553

4442581941338

81163887623

392746422516

1104374752637

6683410

121117926

1093157412412

92511121336760
25630242717

11

2

21

433931

4123316

522561

121

1253

21

221

1

631024313

31451

4222

28311

2

112

1

1

156816211

216411

235

52141

148162765

131

782348346181876530423821
2465134915722157718982

322212

417230320410155166
224112821740226110651066

12285

617203293396159127

4331624

1673361

209561021443853
228651181884379

2

1891641326

11

11

31061591

1354133

2327143668

6782675

733464603635

641

221934172247

6778651491748

143041472131

2101223

1113126

1725

261811372

2671511325

23101815728

57283046964

832

11196

6149121925429
6252121965530

13111

3

1

1

16789791333670
15372681303264

21

1

5211

1

1387334

2

1

276233

5163345

121033636

15121810105

1129

643194892121
80391291183131

126261168

1

4261632

32

10611045

31

118515132017
78097187

21412

255218

17281339915

203123282306108211
209126283308108217

25

6211

1

89891601523537

2641862

9460115763229

81013578

966669602720

1

11

34232

31

536217

341547541914

41262012

8191

11102718144

674924

1003775542421

2317182676

12

7514764821053292466
31814012125447113

1

1

227813126
227813136

1

211

135578

22

170194211418152190
151159190388140167

111

2

1

11

1

1

1

2

1

1

1

3

11

1

111

1

11

1

1

1

1

113

22

2

11

31

121

1071112511

2

1

131

1

391

71

1

152

21

109386330015102
7432361581570

3562614232

1

1

12

41206151

624344402528
624946413028

14

1

61

1

15512757

1314211210

219

4

128111

1413

2106101

10152818198

206251255

10427982362674

1111

922960893332

3

211

282231321623

14515231

62323915

114

512542

1511712310

2

2

187523610

186113157302106106
186113158304106106

12

773156762628

694238495527
694239495527

1

423102

6110711

1257726541

422283772126

272731

2

1655145

25610

1221812

1

37416221

72261724926

53332

11144621301321

1751201641916779

3371561548

21287154

21423

1261634915

4211

656895842132

242031611527

11

36320244

11125

11

13232914522

1

8231

1391854

433366582017

2351

71701141291328

2761395

563140723327

13413

9272881332543

3461

25108564

54483852944

602245511217

15113925310

616474

4521242

221196

7919302391875

723343941641

421821492117

14

225111447

5112371

44135637821

44818730019689125

44818730019689125

61451081098047
2

311855443429
311855403428

41

113201159

16111230336

313212463

1

1

57516331310
8261242

3526652

14141546

375318212979336815121602
236152295250125135

366183281386105162

12768116783184

642357715032

682746944229

743452421516

1216482822144

182225522220

231732291117

152661302813

1452653427

1643712392550

483439672129
473439612029

161

14489253
14479252

11

907880793951

422233271014
42223325914

21

873168472033
873167472033

1

281532592621

1334173888834

15201840216

81536261315
81535261315

1

29161614611
1573165

14913136

823352362117
823252362117

1

121891301904751

106871041114470

12

643642793035

6044363220115

312711081004948

1214975463329

2253113

4555831092037

3014152394
267121042

4731352

32174243915

281450583316

592439731923

280951842135878

942566641622

5

132286412139

491327681529

12941

6912621017

314225481227

8521112493431

1162196771757
1162397771759

212

289587
69476

22111

123285431817
113041421615

1244122

30171929238

1273386

522954971742

282744652826

282744652826

201832182015
282744652826

7421735

61

111193

1

312

13923

124455911571466530610
2220938511

30173428289
15811474

831510132

213451

41

513922

21

1
309112318426141168

14102430107

14102430107
1410243097

1

1

1

3158501626

3158501626

119301171862552
2

10618841511043

13123335139

471843402622

471843402622

372261392616

20931201510

5415761

129151255

251230201613

251230201613
251222201613

8

351534612232

351534612232

796375700848309373
542454

9143661135244

652747713523

261619421721

272422442118

272422432118

1

794062713046
309157340355141143

231414685

472441471017

1562365

206830517

14101

14311

328144193
329166193

122

2212411

1771725189

251215421017

16152720169
16112720159

41

32242

1

183226033

12558

2411321022

36414727033290164
743364612935

83155514628

49305247725

151711201114

281444381612

392313341024

212

427152067

1925686

1541030213

1

3121013
8735961264749

311226252213

311226252213

321
532268912433

1971844816
1871844715

111

239302894

862017712

11521
108858710541267677555

332139243308142159
573049271723

27132331411

491233401711

9315179

261217401817

731018910

1071111511

971139671620

82015141017

201813421428

73224

1571627911

12211

12211

746438804939529388
110601501216659

262334

22121

674137483121

11351

67634

232

12311

6321928

85129122

4492832264

8142024555

20121118143

317442

1111

91113101911

312392

1021

311252

11814553

2610161944

426142

31204348199

19114364839

314

87101067

41

3121

13891487

11

76121186

154152237

8341366

12102013146

1910619819

324555

837896

561119113

310223

33212

2314121243

3557101

7653377

1

1

1132121

16328213613

1711111165

321

4491816

1

211

163241394

6191544

9310121

1

6463

1325553

19315637

58131165

62101444

117135615

11192014

954842

5414742

251636441122

9351783

12121516103

241

22145

16115153

11

31611
6741326

3212
3112

1

11121

122

11

122

11

11

211

11

11

11

1764714940723160

1

1764714840723160

16911010014479121

1
16911010014479121

112857211663100

112857211663100
2217821823

122518516

41231

1627811
1627711

1

62

1055887
10559910

1

13

335037453147

1574562

1411

5741289
332233

1

1341

141715

52182416811

52182416811

52182416811

113114281933193310191365
413964582361362758703039137650

772415910

772415910

46725

46725

3718875

3718875
3715475

34

139301668

139301668

139301668

139301668

650411612626383399
311

525397

525397

291621291835
11922

1

1

2686201432
11

7132127

4

46145

1211

1411423

176723

1

134513

3121

1

1318414

1318414

21633191017
579358533534300324

453111

3133

5621469

2534

8114665

12

11

129122365

441411

122321

2831375

321021161510

3118654

1

121326361725

133421

588824

7391011

311331

42113

23311

13

12113

11132

941898

1212

232323

502437373818

4211

149131126

110171277

11

1211

472027282321

431731

25107410

233344

2669953

23213

8132

14611

21

13388117

2212834

435167

32221

1

232

13120146

444534

313102

42212

131113725

1222

111

524659

15613982

276151163

2

815492

255211365

6831111

414102025

321531341125

11311818

1229

11

28681824

89655

363473

1

323551

472311

471463

11

3791712411

181916374514

454522

12126676
121266396

32

2262646

151218191015

151218191015
151218181015

1

232250293637
1405952194716589411014

656698745628
1

2773

19173826175

111

454545

13332425129

29102911155

131786417991555849949
13131715911

4533103997988
215187403293282303

21121279

2315366

1111

14762

7917141817

673243

4810459

1

138177155

22532

91225152013
91225152014

1

51317183428
41114153325

123313

1111

2

1468114

41520131212
41620141215

1

111

1

36204331814

2111

184864

2194104

992016510

212112

11

127212

1331

1151102

251533141243

1

124276106

242133361312
21712941731684114

231239311314

22193546815
22183546715

11

17111519612

3883414213

1014604494

26914935

392615163818

9422301316

9572395
95142495

71

292821

292821

231043

231043

313069623244
17

674220119

252326422128

187137208194114109
714555553235

362141422015
362141432015

1

22581559

1262220911

7194019713

21212

5878106

71220212210

2719141087

11

650356683657322364
191121249201122115

11

351952

251040161111

132928221310

513151722232

28122617109

256624

314

71631612

1463752472227

2215367

6116678

191325221912

1558653

615913746

1

103101668

179211556

10427621010

313731

1367724

41134

118

118

1417213

810720710

13

101

1

27114816811

19122431713

86788612682105761872
204471550323186423281391015229

21114614851912394
7703446152472038647344116

6761382242491884541683501
9755335372936788413

2146898594744312341185
12403781284908598480

10750691516061
10650681516061

11

1313

212

161311

11214

3161

11121

191712622810

62111132

15122728137
150157151338182318

74111746
52111223

11

12423

115
52387

42372

1041098724114495
86945320313155

12

111

1

543

151

2

18729241336

111

2223
22341

111

7831021
7931031

11

26763201
313994202

331

42

11

1141
113

11

3121
311

11

13412
114632

1122

243122
243132

1

13110098985774
13010097975774

111

13251

2111

2908951282123119

15101221175

21

145698

2681102211

22

5127472475839
4827442425639

3352

3111416

471435283228

323461292315

12

1

234

127

15132179

15132179

313663592920

1111

17152652127

13203761712

356323142985830320751846
9967398854169612545

24

21121

2

241321

1216112211312

2651147

11

75102183

124132

3626228625

41633

2012242393

1174939963349

2521382

43

151318262216

41010966

5611272477

17313825624196124
18014026024296124

7221
11

71

11

2
1

1

111411

10612622

271122131110
27102213119

11

1715102942010

13382

236

111523

522311

3383315910

42621292419

2681334815

97613213

2732575937103

315

6721264

48162535916

126934912

981932106

3210422

7172920159

3231

1122

137312

191121331018

761946391111

1521

8381458

12

10623172010

652040334713

555412

681716119

2511121

2771684

1381412311

1611111862

332138251219

461

6106959

1324

2151151

222912

352

34716713

115

411

10111534915

25958317029103

1189121212

3

371015341917

1111

421525421112
421525411112

1

39349

21641

1615263078

1

684777

22252

1034161797

19193

41

247133

5813

22111

1111201162

33426529103

1531

91314321420

225120552131

175133289

3912201913

6592

1046666

2113

171013139417

2

457721

6713621

2111013

21

2663031510

11

1

177241
214136117

176476

1112233059

431315971515

183621

741581311333
782190391935

131

1

359561

1

2315211851

361239262310

83552

13323

733864

91018784
91019884

11

11

24751394

113522

689843

2219113295

11421

138663614

12

27223

2653

4332527

3521019

3061851

221101

191513221911

1

1

681927

463537261926

152292581514

1718142101313

16327821

152

3

272316382018

5410211114

3141

28112816106

16234346721
1694144713

14228

3222192047

1

37819518968015624

371691074

14181131

453657833528

443356783427
453657833528

113
11311

11

22
212

1

467357590644310361
114931231476961

434653401926
16614119120592150

231751

13

1611141329
1612141429

11

65841

332412

313

3681014

5610203

1311

2139

447147

337518

41

1254411

1

41822

1

1

474524

462630593632
462630583632

1

649652

1

1123

1179669

201130442421
187123276292149150

313221

656382
656282

1

22211

213

33621105
33620105

1

38133221107

383656762363

363556752363
383656762363

211

22152112

9676116

941812114

342557532923
342658562923

1

2

11

411722

48344

24624

1

2911

122

11333

1

15317464

1
3310932

2151
2131

2

21221
21211

1

1

2411
1411

1

12

21710919228893130
241339261315

29213235815

652942231317

301928331627

6927501714356
6927511714356

1

1092034128
442458813728

261022251212

12

8411644

1

141394

495358812445

23123
495358812445

1712144686

415121553

612010615

2022118518

338724554027427720472430
244164362322164224

203664392332

203664392332

4048104933940
610171665

5102618107
5102620107

2

135963
145963

1

476615

533347

1841375

186432658

600371653675354411
192312802137231311861347

4271086

423

533394581984

1710211775

421051

322

1

141331

16818959

97512105

4441

121626371334

1132

891213412

122715

2514332459

2

4571121

51846

27111817

442

1281322

2416912411

141024421417

968512

269577

22

1620121275

12201332440

11111

112034222111

2321014

24761688

67132891111

20121114124

651

664932

7122537105

213422

312721

10132414168

7385120

492373774146

53266

1624334

22141723719

22913141120

16527145821

1216131468

23212

68111457
1181815627

571120

13342

456454

216724

31641

663273655553

125212

166733

1512

19310219219912887

127422

211315252714

3962244

521628241616

351616108

234549

3021321597

2416291779

113316

113316

582633412134

41014752

444774675524
474774675725

111

21

149142458

51611647

2036615816

1313119128

1411132493

61923123

8358272

451

3610911

72721141111

236755

7217154

23214728

99131455

124472

21111

21171614817

431114112

811622316

33247

20171012112

26528371313

1963638

92152187
642654412324

1463442

1410108910

27826825

312048252014

1131

301945242014

1922826166

1922825166
1922826166

1

539532686803304453
859014319567122

10222027321

22142720810

2971311117

243118371511

482620302025

502852462119

7131712620

21

2175

216224441215

681014711

861025116

46201766

81631221011

473929261120

11173129916

19191427818

93910514

191943401113

3692935816

832212511

8111815814

64425281110

493555612624

3431348

129109166241109121
314442552849

2411115675

629222

151526241912

181116341311

63820134
63820135

1

21621
216211

1

1111

2454

22

971619128

9101716514

6510777

1184478422128

1184478422128

191835331921
260174320332142141

1045

1651152202218787
1701232242229096

32

5533

1

111

112

1

531427611117

1893016177

250324244038562421262638
34131858

263663483031
272269408366220255

944685693747

122522222518

483977664353

21293643924

171938201818

212427312116

335160673748

1311182044

1311182044

221521403599522018972371
876946156924579091088

203052572026

104731611

272522601326

14444

5814769

531523

2231265

1131

262848802540

23111331417

211

16515745

382546

4112041

525683833552

6162124118

66810110

14581

5113252

456279673137
456380683138

1111

511162235

118621318

4391

12172239717

1310

292551663037
292551653037

1

81439291945

5101726710

254440663023

1941931

14641033

1

293032581825

3662

6241115

151101234

202835822331

534294996570

8101321156

26111767

122438323321

31101734

9049961514383

271525
5713

22812

556454

295201677

413422

6953901104153
7154941154356

214523

222921

751363

66471022314169

226312

273037701525

2211916

15341

31123019512

341414

14202125138

3332

392549544115

3293135

134111336

1234

1162516109

3316142026

12195635818

8101138917

736531

3846710

63128251710

11310319623483102

9440911103561

9440911103561
9337861093361

1

1231

12

1

114814

5131491

1617122598

124612

1612101555

375198844968
3952106865070

218212

136531

39835611319

92135592324

104161296
536363613558318292

172725321815

172725321815
162725291815

13

875585953445

875583953445
875585953445

2

108791131065379

108791131065379

282269491918

282269491918

173127281722
737393787951

2

2782514116

92724284618
92726294718

211

19715535

11

5614131510
553970573623

2217241656

28163228167

158641421297055
1541013211

1311

391736333713

1422

823882722326
823884722326

2

1

713422

1534222

37076512981071513784
185793903456

4612126918669165

4612126918669165

2122017910
88196317249143181

207089834067

3850114644645

286494854859

4673117885155

4673117885155

71834181623
111186266249132176

273362483342

4381911115269
4381901095269

12

12

345479723142

265071733250

265071733250

358216513652101

358216513652101
358116413652101

11

323043362829
15851197199820409791146

151645261711
21

88261287

78171394

271539252120

271539252120

17612241010
10751681014652

1

31314221012

17551735
17651835

11

191319191315

161471145

6911765

2214412328

2214412328

221
252938572032

251214210

181012301513

1

312141238

663572452826
173861651487073

332421381627

1

1

1151

412343302417

41441

27120313

398276411443193262
101285413441475700831

11

133842

111

325422

35381981274389

5431629

8610296

452112

165131047

211

111

1

1121434

6112

7519113

1241

2

5149

21081045

554621

11

432103

2154510

51618201013
610822

510812811

11

21

424241

1112

5614

3111

13277512116
12277512116

1

132151547

223

519783

1122

11

542851441023

1124

12361

11

151

918

483034251324

23111

20

12

122

22

127944

481912728

244621

3941272

13233

3311

131035181117

141225
141325

1

1

4461233

21416215

24111

21

1321

122

33474

162225161010

691063

11

141224212115
131221181915

1332

1121117

13

212534541517

214101116

111

2331

2

3144
12

3132

929334

145932

529426

111

10310927

2

1

1

9252830924

2522

344512

1

111

12

171835

376853

2173111

4571218

111121271122
57161988

4456313

221

61211

2382

11

1

1352141

4351022

332

111

21

23623121511

7112149

98976816612453

137

34211

19113417129

19113417129

566188784760
75121188

142119877

4241032

339675
439675

1

371297

51025612

126121466

536525

24121088

1
942485381017

75196416510

195212157

3681664

4

3681264
368762

52

1
11911615215682116

61266157

61266157

4291091

4291091

2311
524143401652

3223211667

20182021944
20162021944

2

7151681015

7151681015

412121775

412121775

201429291217

201429291217

8472144

8472144

18162925915

18162925915
18162725815

21

603967844747

603967844747
603461764141

33134

1

13432

3

9457851383763

9457851383763
1316151959

12

141

111

1

911

1

1

11

1

124114
1226255

22141

224511
1321

1

1231

321418651117

11211

121

2

133

1125

2

4531

1

1

12222

1111

1

1251

16191715

11

11

1211

716311

1111

23211

86566910581155533594
142823412014

259836

259836

585186784949
344233398482214237

11

131197985

19171627414

63341825

16124321122
13123181017

311415

2110151975

101015121712

91211265

5310756

14115

12291293

4114966

23828121012

8810446

381522141615

1431

45422

1915132293

11111635317

17511997

2513

4112

8511241

21

561181110

38161467

11111

8462956

2481019310

756097523034
594873392126

236522
366522

13

13618876
13618875

1

543185642030

543185642030

1155737

1155737

26184020239

26184020239

254202275288141164
726194873243

2417425214

9221314719

141212101711

334262055

499947

5314299109

1081530815

56635105

152534302016

12610674

1333

10175106

111

285466

23531

23531

78122654
344947842633

5121117310

810617810

1418182499

1
11

1

473153824045

463052804044

11121

4523221014
342020710

1113

3131

205695814051
443488710750337448

456494793850
293468562235

61071344

32131

258456

1

67232

256512

95173202265102136
154352522526

112823

632476

292551802135

19191927916

92524401723

73329361215

2152114810

792412

1121
471513167

16354

4588112

532439382718
1248713616477105

29221551324

5518264

10622162027

37181654

242324411628

242324411628

263127252425
1551011681486499

11193335612

743080362333

442128521129

368952

368952

368952

44421011376548
90470511392480675670

72558422827

72558422827

182115216899131145
5753796261844380344

293227481615

3142011

1344133

324663583030
324662582930

11

28111216159

252467583422

604644702932

221622451413

804566335932

22112

11

2431118457

6111229106

8123772343227

197161266306130148
131036281213

152432472420

251933332116

262640271318

453552
322040821224

28153777722

393140391721

442942442631

323655

2111
334947512838

253625261019
23362326918

2211

81122241718

11322033
4849411004465

237887

181120332013

173212391342

42412239
192181289337196369

71320331314
303442753332

313121098

313121098

11

1

12

12
102

9

1

16

17876
212

1

1

15674

15674

221

1

1

2

2114

2114

112

1
21

1

1

1

1

1

28

17175214963
1714469555

26437

1111

6837798561133
141128191236131265

5321956

11

8107654

571061

76743

719139159

862224568

32642

3382045
2381943

112

1956171310

21

12101114

411198711

2

1

71741977

22141261321

22141261321

74562411191071483576
22311

13551345

13551345

501475856760348416
14412524724895120

1

5225

222121

132036261823

10232728158

71114535

21

2111

121

124

785597534040

5913978

14142725610

1231222

152035512322

15132536314

213

1467510

253053752332

712181147

11631

112831191012

102236191524

1432

11

157181177

842011124

3

1222

51161

1572313125

111

45111188

571936261312

21

256342

6

51121

1132

202436361219

252439361625

252439361625

372227421215

372227421215

1111220710

1111220710

36191029
252357592136

231422

76111454

1411635

2114868

10311738

24753162

24753162

451944671826

451944671826

133042272416

133042272416

491629431625

491629431625

352441311825

352441311825

352441311825
5612445

61322

322721

13822515

872131112

4228684

4228684

4228684

4228684

283333134492740241
1112

24333

2211

2211

2223

2223

181756610557258
22

18321332
11

11

11

17211322
1111

1

21

161129

12359388410443

3214122397
12359388410443

1113

1

52131

812111199
8126979

522

22421

4967694

2321429712

2

11

22

1

1

1

1

11514
11614

1

1151

211
21221

1

11

1

11

11

211

211

211

31
3510242033110

35820183308

35820183308

21
121

1

11
111

1

8024147360146161

21

21

8024145360145161
11

32412
12

3221

1

361415

361415

97471204144
4123939354144154

127341012
88113644055

1

11

2
1

1

2211

1

11

11

124
11

23

2171101616
3181101616

1

1

221
211

1

211

492537

1

1

1

3125

21
2

1

512

2

1

21

12

222

422
42321

211

111
2997155

2896145

14334852629

11294852629
2221782428

1

11

1

973411

34

220253712
8426782221

13

151574

321

5821063

6512

3611

3611

3611

1
221216171716

113642

113642

211113111313

762171

1

226423

212

1

21416

212

211

2

21

4

123816261837

4541139

4541139

83312151528

322324
83312151528

73443

42046518

143243

12353217118

12353217118

52010546

52010546

715221272

4414741

3118531

7167188921964016738604711426
146213301252138143

264285402428263250
11921131417

843831
66701141187168

123482

425681

25518

941427107

245743

667559

4411555

257513

716131649

56646

2712716

84111094

513741

169453

102951441267983
29831211617

235223

11

16117114

221

27213

57324

105814210

111

107521

315122

11732

638241

22121

2211
2111

1

24423

613551

66101074

1

221

1214

827245

211

11

11

145644

2631362

14223

36151447
46151448

11

232

323745364432
36381

378869

791434

24107103

354846

179166139

25581
5169711285346

11151

322215

13133

10531233

42182

322111

13212

382312

121

5122729139

4141422

101311141113

152223

3151651

11241

257724

224624

331

562641720648302464
6663183131881215942557610966

92271547
5221845

2461

1

221

398093905140
440269308326164137

137753

2731053

19810723
19910723

1

12

1117342

769512

5108442

5108442

34131253

13522

11

312

111

1441

321232

101517113

371321116

6361013

16332

188441323

111225101

141291934

289532

232223

504131948

217926710

578793

1

161525111315

6531256

4211484

1131

284526

11332

1
7572736

3421423

3151312

1

1011531161358677

1011531161358677

6113632

5371675

90149961137670

976620867817448547
430139170148127132

103219441415

2432

12

15232

9515655

222122

10131316417
11202327827

263

1510547

2681984

111

1251565

432415910

145422

155410

174543

2136673

26872
28872

2

35426

632494

1514811213

481411149

121917151710

1

51026142310

22724266

1352622529
1352521529

11

121320301910

5613667

36830999

5148211812
5138171812

14

3102012617

26762

2556

5133824

7241912119

513157912

261734261315

1541222

273648391634

13311

13411

2425425

316188177
316187167

11

610151344

12

331658251515

412843301314

1

141
1241

2

245913

69111045

111

10102186

5331

36202628712
36202628813

11

302250191738
226512

8215612

58142115
57142114

11

1251131

35145128

2730371330107

1

1

2630371330107
2629341330107

11

11

2

2

118782558298519415474682
358515706154811277639418938

2352448220169279

20726520483454

102335392220

236065763248

4915861986845

710211056

151323271612

20102512311

8111723124

723472881418

231940311329
231942311330

21

131732117514127

2611968913647

126450472854

7323

345575

1

8361012

5018401758

321430202618

1211855

138164352331

8310624

3745025934

73639321613

163849551435

53565

2

161744331520
171844331620

111

116241

371461311465165

92443371521

1699112

4289584033499540

144427311625

92117261314

32122024149

54541

2534558319570254

61330191519

216264424740

213

233111

620302601020

103522451613

3122637

388986

1

11181627118

112

62317312210

7172314913

11

261827141912

314871414331

165143

2121593

52321382465

27632471394565

4021500331236246175

91038101566

304467943829

18122652621

163046472627

133026362330

2165113471377

5121

8577630629

1

32081349

364347443536

51204825616

4669921254953

435379774922

3211342

11

27114120593374

3346756621033313

3861966

72633392025

13681018
13981018

3

52721

112737321130

26111122
24881

23321

1

1

214043692835

291832311623

1215152696

22241850523

353457492626
353456492525

111

213027161116

14719122812

16471316

112212

7162313717

152129351628

116151586

383724

1615933

161432211212

112942251316

2

30129113966463

152221171412

1

722230231214

17251218107

1

14261727511

1623210

4131334210

38404424821

627141676

71515261316

343332322915

132423191868

881263

1

51023101014

4981344

2122

62525131310

62617191213

10141918227

63313261912

707889964068

61525221016

35522

242133431124

1673

6109673

111951651062092

959484593650

157616

2113119729
2113219730

11

322449263016

194877662744

34121358

114555782147

7721261816

2

2121514135

30418231013

161223362916

161223362916

293053582340
1272021323

351933

126565

27

113111

6210231

1611

1

38614

1

24141

4341

168122242178104101
88182311231072529589

11215

302457292137

241

241

812111447

5849661081434

95152676

151

211523585

241416261910

122

216231

946070764238

139113

3193814

6894

76125413

28141941618

367569

781725106

210147717

179513112

6312752

149141598
139141598

1

1

2

233419191213

933450533734
883450523734

51

13253382313

323

19312142512

343239

41213331611
41214341611

11

1103462

1120137610

3163621187

144245411

226423

14251223613

20313341

4

123634

11111718615

8121931910

325230331123

13233310108

73417

1511

126282259
126251949

331

265362
276462

111

4312646

6123978

1219211347

3101353

111

553525521617

9258119926546
10111417159

341

115955

1615872

42122

229213

1512

112

5

63411

3

17211892

111

2

95615128

23111

9266412

101411

43241648

11

1211

223624521

223624521

558351507879744139204074
701362061807992

77701121305563
1

223354583030

223354583030

553757722533

553757722533

33315
478552935761422462

11
614478494837

614368473729
614375484434

7175

12133

2614471638

2614471638

164239403028

164239403028

241752392621
372452768653340384

1

18510533

1

298644

112

41831371136068

2112

58911221145361

231234

545186663136

132548472226

12422151715

299432

213141

11

321943472762

1081332261918579

773082433729
228815332465238513721411

633683522723

633683522723

504772865237

494571855134
504772865237

121113

234449573926

234449573926
234448573925

11

12211
303044412624

110101175

844874

201430201114

12916948

12916948

1721159921

1721159921

111228181124
7102314722

32513
32413

1

1312

5472264

5472264

9173617812

9173617812

125410123

125410123

482150431824

482150431824

673748634039

673748634039

11261
482666452524

88121818

391752212316

519298897864

519298897864
710222192

448276686962

14122027138

14122027138

16101927128

16101927128

9757119846652

31423

444512

1175

15681135

25614969

131754201718

884211

6611456

2662222268

127101150168100100
159819179

42616810
343057453142

292650272329
302851292332

12123

161772295

152171183
175181683

2315

1710836
179836

1

66715710

6371554

1135873

24344

812219813

1157715

23455
232137342750

118149716
97107414

214232

101320211529
91320211429

11

8768133935441
576323559544337310

24222

312137283020

151521401221

15967107975165
189791371145779

30123017614

242143451629

791914

4211

75173

381417232425

333034653027

1405411810810857
1334811310210153

33632

43542

108161737

664041392624

664041392624

2993023109

2993023109

32321
2811249911

18717426

727254

393549322678

32173517871

7181415187

17835181021
3

428325

31012
31112

1

2268410

5410644

21051026

21051026

17711719421090103

17711719421090103
17711619120887102

13231

10918464
923264452829

31512748
31512788

4

195151674

32131918713

12940711184647
2081421732479396

32222

111212

91015534

7511

21122133912

845152114

65101344

1221

12

122

231422

12221

6567112

1112641

41

1614

356825

21231

231

21232

61

1110149166
21913217820097115

875476853261
905577983364

3111313

1166686854142
1186787934845

211873

131524171915

131524171915

2322
222746471514

89253197

1115211465
1215211465

1

1411

1

141

267028594160398119912045
156259314304118145

193155362116

193055352115
193155362116

111

132312

132312

3892101984639

389295984437
359293974437

321

32

32

32

1481671761809490
789563680643405447

161141

5522445

45531

21

16372618911

337123

11

237122

1131
22631

1133

111

1248213215661106

1248213215661106
4546781022739

793654543467

5931833
8869142866775

8360139686472

8364213
8464213

1

10129536

271026331015

4122

3410839

2598337814182

4813171178
51152215109

325431

664111

1814141535
1914141535

1

217127715

313111137

2233

131

348691

179166212307113122
5266781373357

131018231213
162433391815

311151351

3311

12512331
12813552

31111

11

3755332
3755312

2

11322

6512
186113

12111

21010834
21010714

12

310513138

441352

2513321747

552487

251220651815
251221651815

1

175347503228

175347503228
175147492928

213

4685135954851

4685135954851

61697211110560

57687111010559

41111

382229521821

382229521821

3737105565635
92130242024

449491
32237

127121

345016192

4

15771066

615222

104671731013240
18114436125586156

1261513852

311122146

1311

2121

1541153

5310413

7123817107

515231069

292173351619
432382752034

142940415

285545351627

285545351627

15414222417882102
181181312240108140

12232
121232

1

1142

1363

1363

1111233

111

11912543

2121

1322

211521

121034

125913

47241535
434823

420712

3335

423512

111

6213

13161217810

13161217810

492537816870471392
701111742358685

564292763735

11

3816171012

303942472927

614077662932

194249563922

53744

574555464226
576561484631

206245

40591051387852

91791161157759

644781613631
654781643632

131

143544682118

143544682118

1
2594214

2494214

43861811605158

41761781575058
43861811605158

210331

378423
484564231916

3615361046

923209137

120169265293156160
163556813737

384181532339

2951821075258

374246524426

261572

261572

76941381094129
1652002842549086

3111812911
3111612911

2

264343

2226171182

158122

11

6133114

7342425

2984465419

915291989
915312089

1

1

1

322
31812581

1

3159381

71523

394947431923
1

1

22

2311

332537251617

244324

1

41521111

412
332070512731

112

11

1

1

124
271965492630

110235
131035331514

1

1

1

12

21

1

11

1111

1

2

121

1

1

1171

21

11

1131

1111

11

324432
429554

15122

213115
13928121116

11

1

111

1

1

2121

11

1

63

212

1

1

2

1

33111

2

112

11

11

11

1

1

12

11

1

1

1

1

1313

1211

1211

672682475564
451328955685433932723692

3631558
384194320256169165

12923151712
414273

411944

368314
3610314

2

118151

864776392824

834373352722

343412

10818947

10818947

53102321

53102321

1536332

1536332

356764
151119221824

1035432

242

1221216

114103

641623241710
1115881754740

13

24713

1

22331

48551
486511

11

3103953

1611

215541

231443
111443

12

135355

341422

1

20415516

5111

112322

111

14411

5686139
1211

3354117

113211

2513179106

15914423

4115

632333

11

123325
973059402232

1166341

648524

351241

7317555

69102422516

401426145159395629853387
262187438271208247

2522

2412
2522

11

3410513

3410513
349512

11

111921181012

91311563

227927

43422

21624

21624

1111
101712167

41213

32331

123101

12311
112311

1

33632

33632

333316

333316

70353661813
167121024

7181

11

2122

1

125143

10351

1113

153413

1171

95222

291241

11

2611436

2611436

4242034

3221

1222013

13221
462958272431

2115

34623

118

22314618
22314617

1

6418322

1

28123

3122

861610312

6531075

6531075

55515110

55515110

51231

51231

1559520814994129
312126284151

259251

68371536

2

33623

134343

7755361025
765236925

131

5158899

77174929819

241617221212

6110161

6110161

915636

915636

231
24112057919

611043413

53312

1855843

57169412
2334

122311

335235

1612

513111
942470583250

2915352466

211

242127

343355

212232

12315645

101821113

69441112866679851080
19164277137193219

813201113212272

229328

16161413117

1672

121712

1

36265717

16764

29217467

28122417186
28122717187

31

9716164

3110636
3110736

1

6510782

27134122

1

9259810

1852281323

2

296557

334263

16171972012

13844

33601780137

282326652121118

27521616

26108574

2331

1

23612944

5292523510

9726289251

3622312269

579224

101223235241

341791945

871017712

21

1

1558761113

36312933617

18886188

412081315

145172057

1

112
382661281524

111138111011

27152216511

22112
442112912

1215734

15556

622342381613
17411017515889126

6351032

626225

1020142299

1122

15317323

231

23201512813
23191512813

1

3210147

939368

8723152110
67179168

1

26552

32

1021112

132

3331132

25766

251854

1138

697645

521

21131
62572135

1191

222111

12122

121521

25111416

2571016
25111416

44

2910231496

17513351

127211

31312

832622

533478643442
2111867

23

27131722

56464

22201632919

1123

12131

1343

36656

421541191936
987713310010980

9

1459115

1432

11133

311431

12

310614

81377193

22412

1

325732

22321

457455

33114

311914

545412

3521

121

1721232

16116341

11335

1384

568744

568744

71811261026947
5319985

15157585

346337

224631

2212
1112

11

1132

33522

11617834

15211

3962382
3662382

3

2534

43233

2211

1153481

439876

11

212411

98211375
57191143

412232

114212

11322

5723

5723

885683624849
12884

3222

11112

5561337

1038214
1268418

2324

14

43443
43343

1

181

1316411

11112

11213

4516435

301391095

1111

1

343241

221124

43332

72531

3112

12313

5176946
11

4142221

134624

451043131429
58245

181423

41

139228424

5813

119132143

119132143

2464103

3232

216271

432323

432323

16731057

231632

1442425

22442

22442

46521410

221

44320410

812944381717
431324838

35121032

22131

1532

312111954

4685622
1

3684511

1111

4226

4226

11212

11212

1433146

1433146

744350684726

334526

257252

32772
52872

21

3452

7761012

49151414177

68814134

231241

10135103984254
181324391324

121

66

57441

1332

21

3

30121639315

21

141

12421

1

2

12

11

1

27227476

1

42343

51101

21
241230331313

79151827

1531515106

198251194
871

141

711273

37644

79281019

79281019

13913151615

13913151615

149218510

11226

14820634

111
432356412737

329321443

86129155

1891742

221326

1
1313201275

358142

108121121
108121132

11

122154513

214119

1111344

471926473121
232

21112

1121041

2533677

6231212

243462

412214

43361

425212

32242

76632

76632

674415

674415

161611281618

161611281618

1094888854336
694871

269182367

1111

4427112

2115

1282010511
1282011511

1

155111242

18715513

2731617410

21361162

21361162

1621

1621

35121224104
836410711875105

1

13211

12241

243338

12

21282

12321

331026

12109510

454432

31111

1714444

412422

5231123

211
1311

11

6410256

21251

217111

33421

635565

647731

57262

13331

11241

31

31

1038443

1038443

1266771

1266771

151
291942551643

11272825

424533

178132

95116433

427342

2876327070

2876327070

2414622

125411

129211

1

1

41915658
1032261421024

22341

60134433115

10371766

4351433

62333

21182820616

26141425
26151546

1121

1912135210

444862663847
1238733

251939411525

3741189

2686105

2133125

31541

31541

121436201211

64161325

610207106

211

211
21

1

9442

9442

613297331426

161721

17549938

131926669
3149443

10517226

52441

335715

726542

21666

21666

101424434

101424434

331249

331249

365633

365633

112343

112343

1910657

1910657

616547

616547

142123

142123

87634

87634

12
273816109

22341258

53431

622541431925
5310252097

32515211

675255

66632

64321
10968108969360

4123162

1181

2673113

643137524232

352249321822

31124

153223

153223

414634

37423

17211

217219780

6174

21715966

2062046112
2061746112

3

435115

435115

114511

114511

148781801627594
215917123

33232

321321

111733

12632

18414833

4324238

211

11

1

241

412

11471

212

44472

25891

814348

1123

66121429

321

31

237223

121220361223

24518536

38925

45143

1433

833325

1956542

1

721111

111

544863593737
661119812

38161178

1

453436282217

1
142627251819

4177998

32131233

777467

16202827832
115149851168579

153621

1569333

1399715

131

1234

1666136123

11121

7311

111

363229

312110353520

1122

111

4111

134318113

33111163

33111163

221238710

221238710

1722201077

151820974

2413

93411
463029521316

13710587

22434

2218114444

213222
17210654

1517422

1

65171018

65171018

93824

93824

911153810
1

649133

376256
276256

1

1657932
843378582628

27320332

2712432

69315

17111

1681814117

1644425

1512024

1291

1291

1473417133

1473417133

105157114

105157114

352752462627

268211124
17613622

92852

51422291917
91931352423

459656

9132025158
11

11533

32461

24321

6251472

35111

311529272118

722652
321552

411

8414989

15512765

141522

211

211

4861122806275
7181921176

1

1212245

344234

314125

122

381

21142

222

41

51372

1123

373

344623

111512

11423

2417725

11311

2111

2921

119199129

403327542129

403327542129

202415261422

202415261422

209122877

6622333

14310544

224231562235

521131376
111

12541

12541

597856

346125

251731

3531758

11

11

2531757
111

1511547
1211547

3

11
111

1

141615261021
12422

1

68466

233322

233322

21322

16438

231511

1136853703746
310131

28925321221

28925321221

28925321221
1

1121210

9221234

175171565

113412

814926362023
3421112389

1

11

1

13

341611838

282134

71221

13211

11221

11221

331535232012

331535232012

331535232012

1
331535232012

57135146

57135146

641

641

7661134

111422

655712

121431

121431

2121

2121

1821

1821

456223

456223

456223

456223

113
456223

14

112

1223

202316423481290320971932
2011181082

45141132
98977316831578802793

818984
1457914012010563

1

518512

321421

11

2111

637214

251313726

115151

12122

2222

22231

12

7561263

1211

1

42

1122

21

222212

12333

112

11221

121

431917512

27711

4134

322320161014

2

111

1331511

131211

1

1336312

324461

111

1

362639542020

22161629813

21
22161629813

19161429711

9761532

745414

11243

1431

11331

1211

1211

14102325127
21111

42285

121

211141

21223

222

52341

21

322

111
80466314901393674708

3112292915020

3112292915020
11

921141386
2

4143135

1411

411125

315123

315123

1571118108
934844

112

1111

11

11

21131

135422

32253

32253

12111315207

1111111486

121121

142167601124
76164014471348503681

116881961275872
1653

682980462624

682980462624

314661452029
25252

12251921713

17213722814

171249311216

171249311216

4738871063546

4738871063546

32832

11321

353179812735

11

8651437

126211551583113220
229613

152156461131

152156461131

8415036940571151
31681631922460

3043941162748

2339112972043
1939108962042

4411

25381171263035

25381171263035

11313

1213

11

7741557
457282545469285316

412252392425
21

32112

2471910108

681113710

318421

235411

17725

1

1
6441102974854

272371632527

2128335

1

1

151623312021

7661333

7661333

431929411421

431929411421

43724
11429194053

342393749

4331

19912191010

18912181010
19912191010

11

151472058

151472058

2517121486

2517121486

171428271016

171428271016

171541191517
1

97197711

78221286
78211186

11

331740231815

7812101210

269281365

85815117

85815117

48165122

48165122

14278150985663
54203724919

18421221111

834752

44352

10612543
859322

213221

99166119

1

21113
20513845

1

18512722

18451

12

10511921

817286411

469569

469569

1742753

1742753

1742753

1742753

1741742

1741742

111

111

12
123103418202525411

53613415
1415

113

111

1

1

101

11124

1112

475846
117100412189519396

8371349151487357

8542142

8542142

21121

4121631

1313

11

7566345130483355
2242

231

231

7366343125479352

1161143116
7366343125479352

1831110
212284025

133

11

13

6

1

3

112

11

62

121

231

12248

1

354627993242260

11

111

11

1

1

11

1

1

2211
221

1

1911281816547

302258302833

191641181920
11213

611532
745645

212

1

1

1111
3111

2

211342

211342

2
8931966

42411
52411

1

5428543

1112

11244
1134

11

1

727543
11

421

421

316342

316342

32212

32212

32212

128658

128658

128658
114648

141

382573636750

3254124

3254124

3254124

3254124

325444
3254124

8

352368595546

352368595546
631023358

11313

433438

12432

312319

4131

1

2412966

512214

46953

31241

2122

7806901197939620641
464348382143

2613632

2613632

2613632

2613632
1322

121211
12211

1

321
11

31

1

20514331919211799
2243

119161474

119161474
1

5881174

11235

11235

475824

1

5183

1

522

11

5

1391012481186963

16217656

43

16213626

9813532
123992311126457

243254403016

9131418223

15194022813

1121

1121

45191149

45191149

3417231635
7945139501823

1261121

73342

85101666

18171021439

2111
695487

443183

23123

16818877
553153564129

645616

11

22532

21111

4311664

44231

214312

31352

1610624122

1311

184190277213250170

184190277213250170
314160506631

14822

3137767

213111

4551163

13108616

12132

5211

11331

927442

5731334

1233

211

211

431211

45111433

1421

112121

657634

251534101027

32422311625

11211

11

562643

3110149513

3246

1221

216

167481

223

152255

1091311223

111413111218

281191377333174210
125234

1211
254168339302148176

33552
13331

1

2111

1

14132929

141226

1293

12212
128801651498198

723883683448
1993214522

6310872

1742712

10101622716

751135

137121496

17916162115

1

16916162115

87122358

3331123

234532

1122

21451

241447331019

241447331019

61059106

61059106

161940321921

2981146
161940321921

4

4

33861

11102513914

575737

1
575737

233321

342415

342415

1691013911

1691013911

21112

14891289

1

723683663537

723683663537
254325

136181786

131332

562558332224
552457312223

11121

262133292330
235616

11312

15265

206145811

36119136

234
6211517412655116

11292616826
14713

43421

913116216

185546

101424331318

101424331318

4066117713364

4066117713364

4066117713364

14512

3662109703158
3962112713262

33114

144614

144614

2111

1

211

5635891046931

231

231

5635871016831
14122423157

23921

11

112

1

1

111

2622

11

1

11

11

51381

1

11111

1121

1

11

211

11311

1

1

212

11112

1

2113

1

51333

1

51

18

231

1

213

2232

11111

12

1

1711

1

122

1

116

10

24411

121

22

1

1

3122

1

3214933

1111

1111

207616252497293020861315
6215923

116068211881818794790
76464

39816816
633033281917

21191118168
61453

42

111

121

1

1

243

1

2211

21

11

216

3

11

3

1

4

511

11

32

1

1

2
336223

1

1

1

1

12512
136213

1

111

1

2132103

1

1132103

1
545222

1

112

21

11

1

41

2

1

412
1

1

4

1

26111

26111

1815923176

1815923176
124

211

141

1

1

21

31

754851

21

3211

1111

121322

12211

1115
1

113

1

1

23511232

23511232

333223
132

211

222

432033847
82

381029315

413131

121

11

6210493

1
6210493

12212

121

11

36251

11

1521

1

1421

112

1

12

23371
594340609635336421

403262445470253338
321740851923

122112

11

1

11

112

6421
2311

1

31

1

111
1212111

11

211

927339

112
927339

2

11

221

21

1

1

32112

1

1

112

1695726
31142

1

12

1

2

1

1

93413

31

1

1

1

242
17444

1

17

1

2

2412
351843

3

1

1

1

16

1

1

23

212
21332

12

1

2

32232
122

2

111

1

1

211
11

1

1

74221263
549441

134

12

1111

1

1

1

81

8132
32

1

2

1

22

1

271621191149
24714673

1131

1

1

1

31

1

11

1

1

1

1

12

11

2111

12

1

2

1

1

38

1

1

1

11

1

124
2425

1

1

3

1

11

1

1

229442
11131

1

14

11

2

111

1

1

1

21312
433431

1

1

1

1

1

1

1

11

212141
745642

1

121

1

2112

2

2

1

2111
23272

1

121

15

33164018189
21811683

13

121

1

1

3

111

11

3

11

551

4416513

1

1

11

1

11

111

5424
111

1

11

11

111

11

11

699957
113621181118

111

229

2

211

1

1

1

1111

2

73

11

11

1

6

12992637
5582413

222

1

1

1111

1

1

111

2

1

3251232
7481344

1

21

1

1

1

12

1

1

1

1055952
11222

23

1

1

4

1

2

1

1

11112

6

23113
23

2

111

11
111

1

21423

41

221

1

1

21112
211

1

1

1

214
1415

1

11

1

11311
131

11

12211
2211

1

1094494704963
7613655

1

1

54

12

1

1

3

1

4111

1

13

3

2

21

11

242

1

11

1

15

4121

1

111

221

1

1

1

5

1

11

1

1

2

1

1

1

1

1

3

731857242844

21

2

2

1

1

1

3181

132

121

1

521

1

22232
1221

1

1

1

1

1

7457878866112
1042542

21

1

1

1

31

1

1

111

11

11

21

11

1

321

1

1

1

11

1

11

1

112

545081765399

212
11

2

1

133113
1316163

1

15

11

541526148
187751561578281

14331
122

1311

1

374447
17121

3

12

1

121

1

1

1

1

1

1

1558776
1011444

11

11

1

1

1

11

111

1

11

1

1

1

6

34916446
34615335

31

1

1

1

41251
2112

1

1

1

11

1

1

1092012118
11623

1

1

1

11

1

74735

1

1

1

4

131

2

11

1

1

11

14

11
22321

113

1

1

1

1081714511
6510737

1

1

1

2

1

1

1

1

1

1

1

1

1

1

1

21

4

11

1

1

1241
11553

13

1

2

4

1

2

1

1

1492684
33720291217

1

19

271813

1

111

1

1

1

1

1

11

1

2

1

1744513
2044714

1

211

1

4312222

4312222

111

211

112

11

21

411222
111142055

1431

1

131

132

1

21

5113

222221212
20220711

11

1

1

11

1

1

1

1

21

12241
233722

1

1

1

1

11

1

1

1

2512

2512
11

111

131

2111

2111

463672473536
98561351049167

216644

1

11

1

1

112

3111

1222

21

1121

11421

2431

1212

3121

314831

206161135

14134

11

1

111

311132

2

1071

21

1

2221

11

12

1

11

121112

21

111

111

1
231

23

358421

358421

121

121

239652
122412

2

1

1

11

1

53

111131
103334411

1

2211

711828

111

11

25481185

25481185
3112

2

1151

1

1927245

11

11

35

35

1067734

1067233

51

422122
422137

1

5

1443524
195153737

1

31

4113113

7

1

113831
1121

97

131

9361726494

9361726494
14112

421333

2151

21371

1

11

18

12

1

251

111

21

111

2111

1

11232
4112333

311

110

1

1749612
20612846

11

322224

21131
953253

111

1

3

1

1

411112

178921
201010324

32113

32113

2

2

155141376

155141376

31

31

31

99186713
4310236

1

6

1

1

1

21

214

22

1

1

1

1

1

1

1

1

1

1

22341
23341

1

425851021
492374758129129

11324710

11324710

81131221619
2311344256349

2113634

11

1

11432

141

15

459251814

11132

21

2

1

1

1431121

1

1121

2

111266

284184

771030711

1244

22

113871

11

11838

5823

17241012

633

2244283

321432

321432

321432

536753
131

1

44231

2421

8326241
11426451

21

112

261311633
25710521

161112

963431

31223
963431

1

6421

1

139246

139246

1612

123234

11312

11312

135234
111121

1313

11

1

4131
386532

11141

11

1331

152119161817

474292

474292

11141514915
578734

422111

3112

224327

321

21112

1658936

1658936

57127117

57127117

131

131

131

131

36264

36264

8642114653447
885920127110811276505

6112523175

1

1

1149

212

17315555

211434

1

21221

41037

1781583

112

11

2

535375

423411

11

2311

215723

311

1

2

24

21314

42122

474551

111

121

131311862

2

555786

313

1416106113

35263

2

2322

1114

236524

11591093

7511

13

2

2213

412251

774499

1

336742

347672

1185

2

11114

11

74184225149

12511425

1132

331544

21072334

11

654986883534

3133

111

13464

111520543911

5546117

1222113

1

22

1

1

121

12

855483

3125

2

12

66221595

21

24612434

13

1

2621

11411

10779142

1

301116894

512311

1

412411

2212

211

343272

2122

123451

1

32

2

1

53413

124691371069954

18236078370549184

1

2

4811

231445

31132

1347756

1

112

3

1

223864

112

1

1

4781777

829674

1212

2211

27321

12

31422

23

1412

211

219313

12

3151

411151

236420344307406820462011
20620121612

606228522454517310
1111

134

134

134

578198474423478293
23061194139157131

23121

1372912242

11

12

1111

468115

234

61622

141

211

445135

73291

313322

31422

112321

13

43867

1047896

212281

1523

111

104331

2111

3161

18104103211

4211

11

253122

1

6151

54233

311431

111

23

816536

41254

633

32920171518

1

413223

21

313111

526134

1171

30910282329

1282116181

1

956795

23811162211

41531

1

1312

2

121

358243

24261154

13114

11

512411

1

231527253811

16

2382335186

1221

51255

201826182910

201826182910

201826182910

61119897

61119897

147117083554338113201532

147117083554338113201532
48611391103355

63104172
65601281239446

9187141

13

1121113236

385496994037

1131262502447399
135815873286314811931430

11
56521161034647

182554342618
192555342718

111

222251551719

154914210

28114230325

28114230325

4849109965155

4849109965155

232062453018

232062453018

322475863875268400
141633501417

48921761856072

51741611585364

9713222520257127
9613122320257125

1122

11015826527782119
11216126828084120

233321

99581461526164

99581461526164

267264596576258232
81016171010

292879833126

59691041405171

434688793532

56551831576447

433989744030

291737262716

338434957859304434
34861791925985

44691671526084

172651411725

243253558473168239
243253560474168240

211

64981451687076

64981451687076

11

11

1174083545796

211

1

1143882545796

1

132142385

132142385

1
132142385

6271542

6271542

77842

77842

131023361617

37102658
134

14103

3531255

1031310119

1031310119

1031310119

1031310119

124409110811239

112378910110433
1

882766697819

882766697819

882766697819
882766697719

1

241023312614

241023312614

241023312614

1232786

1232786

988613
289192425469302176

221139335387236121

72551091717254

72551091717254

7173443

7173443

64541011106851
312759753316

223211

1

21

12112

111

1

12

12

212
112

1

12

111

1

1261

11113
1113

1

1

211
111

1

1

1121
11121

1

1112

7544122
553432

7

1

1

1

1

1

1

1

211234

72756

22121

2147

22

551

131
3

11

11

114

1

21

31

1127

1127
126

11

651038654

651038654

651038654
2323361

13

2421

231

111

1119

214662

1

212

11

1

1

1

1

143792151789963

123

123

123

131701081619262
111

135656

1314

11232

2222

334417

1311

23416

2141
2161612104

13

4510333

1515321

11

113294

113294

314

1

34

3324111995

1

204151

23311

41553

517143

221222

11721
1431654

4531

242

692954975331
1512106105

14132

422545

3125

73953

733651

12323

1364

31

123

14
54

4

6222

3211732

4155

1

334

1

111

1111

2

21131
211131

1

5132

1

11

14

2

542

2
84365

121
83123

61

112

11

1222
11

1

1

21
1

2

44102821

44102821

20530131312
594582766552

6111

2215

2192414

2

1741

5113561

111

52151

11131

1

23213

454155

1111

11

12221

1171

1

32

112

1191

2311

21

313

555312

271854383928

261752363828

261752363828

201543323825
12

121
101026152817

4888203

52167714

951517108

951517108

62943

62943
2142

1

41522

11221
11

1

2

2

80819605220364440693592
190020286095782232439714705540329903

189412582217175811091310

187612492195174510991297
954383838495

11571786

11571786

11571786

94226

2151526

74312615

74312615

3326412

3326412

411623

411623

1426733
556408482553314292

25518421520

25518411520

18416201317

61212

1111911

1

1

1

41111
1941301791658875

27131339168

27131339168
187113566

9624102

107951341025648
527557

101681986

1812152122
1817152122

5

357822

535522

118563

3131024

52144114

41151133

111

16710525

8420916

312321

11820276

5623

26512536

23211532
26512536

3314

24151711118

24151711118

612814

612814

226315
193165161191129117

313524
13424

11

2

412825221121
177153138164116100

31224

1021

3

346

1

543112

1

22

1211

94225511

2

1111

11

25311

71733

2616

171110768

1

134272

33621

4212

233521171011
7411726

3251763

1

2112

133822

111

42221

2

3111

111

31

31561

1

48235

26575

1

3611

104113146

22142

1

41

1155

111411

173174

31

11

12

42342

121

77785

1212

1191317108
5211

333211

4214

2111251

11431
115321

111

12

12

8717131010

8717131010
356928

414431

11751

1
655670694941

766954
99151486

22431

1

131

231

611214103

611214103

969412
344025372828

32646

2232123

9351154

627414

434

11924

4131

16618424

16618424

1589916

1589916

1589916
1055816

5341

423522571920

1245735
423522571920

111

111

3412

1

26258491111
16533884

1

8191924

11

12

21112

1

1

2

111

421

1

603889583038
650413930550359447

21722

21722

21722

180115323193129135
3111477

44

44

44

84161669

2

2

636632

2181037

212112

212112

2293818226
726242

11

2

517111

111

1

44461

212112

412

1

3652

1

1

31

1

1

53

135871721487990
7720101214

1311386949
95321639

1

1

455511

1

1

1214
124

1

2112

52112118

7102
8102

1

81

51111

42221

233481
233482

1

1423924

3

1

11

22411

282191416

11253473

51133

14478211

214381

138281

131119

10

2

94326144

2

10134061421

411252

5115219
51113

151

6

1113221
112142810

11169

111521161174477
11

11628171012
103501071143969

422121

32321

18321213

28213

2242827
2242927

1

18131410521

3114435

27223

4215361

2371223

1519122
15191212

1

32111

1626636

728358

728358

52194223

52194223

52194223

925580364228
3229

61

61

11211

11211

332112

332112
33212

1

24131911104
33

241210534
1268413

1262121

1664

51124

1111

423

532723112614

2231
532723112614

51212082411

4223

326942

225932

111

34111045

34111045

34111045

925565474472
11583

629229844

629229844

629229844

294538303625
11

1

91479176
193913182712

102569106

237253

3

3

328643

1

21026

532392402837
112541

321228

321228

12215

12215

27249410

27249410
2520248

2472

31842
461242261713

47151

164

43121

2259355

12211333
1128232

3

11

1

1131

4541117383225

23271
4541117383225

15

5616766
8626776

3101

21862

13421284

1116191226

91123946
91123945

1

7821725

7821725

7821725

696055894651

211
696055894651

42326

11

131

12181

1

1

523211

13291311118

7671697

6141417

301524341519

42

71028211420

71028211420

71028211420

71028211420
11

451512810

3512969

523355

523355

523355

523355
423351

14

439260546357250347
101103102

107441259765130
53812

10120164

10120164
311

1141

1141

1

1

11

51434

1

51345

51345

51345

1111
171024122010

414372

414261
414372

111

35242

35242

111

24241

6217453

1113452
6217453

2

314

1

41154

133
41154

31121

401538601838

401538601838
761684

1111

32822511833
31821511529

22

1

1

1

1

1

11323
301435201771

3211433

3211433

4241044

21643

22341

16618110

16618110

6322851
1

32252

11121

2148

322215411257175215
1058537

254434172234

254434172234

116212

13192416

212132

16107879

32

517473

646442

646442

646442

1134897685250
113

51212121113

435696

197627

687324

687314
687324

1

437424
637528

1

1

14

211842

211842

738946
21121919148

14
324

22

211
31222

11112

11

37221

581321

46624755

46624755

84158102

84158102

18211348

10855186905374
21

81732

81732

231636282333
51333

221

759995
758995

1

8271144

1142

8355219

877844
13

11211

4131
3131

1

255133
255123

1

582273401226

2646447
2646437

1

53131831315

339554

6113313
1196312118

3628882
3629893

1

11

2221112

4613442

4613442

4613442

271124461512

428852

428852
427852

1

4113215

4113215

4113215

19833695

531332

831313

621233

294243232234

1381047
294243232234

432711

3414712

2204429

11

2

13

668975

3

3

2411
374458601319

77271633

23331
77271633

1

14422

431911

1811952

1811952

1811952

27281533410

101852519

115191

9341618

171010831

171010831

1123

1123

1123

18922131013

412412
23

12

1

11

11

1

21

5

12

149109911
143233

1

11

113

11

936534

1111

1

1

34111355

34111355

34111355

314187425454335250

314187425454335250
21

513481776154

513481776154
4111

2519853
231652272618

167362

38535

268364

1211

215542
21542

5

13212

331312

331212
331312

1

141421433130
211020109

22127

2

11121

3221053

222241

21

1

121

4122
4112

1

2111

511

1211

1123

1113323

1113323

263153344375274195

258147340358269194

363056785926
258147340358269194

1111

71

1229243

1042745819

4545816

10151085
6151085

4

810620714

239564

6591387

9111

3599188

5117963

20316889

8687410
768739

111

13146

51389147
3357105

2103242

12313

1181

3833
39431

111

2214

621010108

57853

422835

44127145

152561

34189155

43294

1111

2271386

1111811

3182615153

53161146

441022

216121310

5641751

5641751

5641751

182719113227253

141
46172733

21

1

11

152

1

11

1

11

11

17221

1

34131

11

23121

1

175657010426649

533238492720

1

1

533238492719

1

1

1111

11
1111

11

1

1

533037482618
21131

1

1

1

1

1

13

21

21

1

3

11

21

1

11

31

6116765

11

1

11

2296

3

11

123

2

6

1
2241024

2241014

2111

1

2

11

1

122

2

1

12121

1

1

121

6

1

1

2

1

11

2

1

1

1

1

1

1

1

141819812319

141819812319

579328

121

1

1

1

1

151

111

2516

21

91110512111
11112

1

1

1

7

7

1

1

22

1

2

1

11

1

1

88641109

88631108

1

1

1

10815134711610
11

1
5432022

1422
1

1

1

111

111

11
3320

22

1

18

1

95610231016

936761004

231712

853122

2162
853122

1

11

21

223
11

212

2

1

11

1

7558150299889831160988981269878
3681075139711079151305

683982463324

41544

41544

19153943
643967423320

6

71

1

1344

113

1

1

112

1

326211

11

1

3231

11

1221

12

11425523

431

1

2111211

52355

1

11242

11121

211

1

13131

2111

11

64471017617575
212

5033816117064
21

435235

43421

22321

211

134

134

12

11

1

67212268
1

3824

3824

52594
142

51192

1373

1373

1
1543

32

12

1111

3723553416151
1252

131852311

131852311

131124113418
3114118

113

122

111

25315

2

114331
114321

1

111

221162
11

22161

2

21231
21

2121

1

1

111212
1

1

1

1112

1

1

2111

2111

15

5

1

212424
11112

1

11
211

2

2

11

246296
167999011

11

2

7

2

31

3

11

1

1

1

11

3

11

3

1

2622

1

4212

11
1

1

2

2

14141814311

31562

31562

3111
411111

111

111

111

1123

1122

1

1
11

1

41211324

3119314

1121

31
362348393027

21

41

21

11

211

2

11

121

11

13

11

1

21

121

112

3121

1

12

1121

32

242

831111510

1421

212

12

1122

1

1

551514

117641

111

269237379295171177
1131

1721262281788993

9464173855449
5621321

52
218731

121

21431

1229

1229
1219

1

6612221

6612221

41

41

32115329718
2672516116

233

15

15

1

2116652

11

11

136

91217488

91217488

219233

219233
219232

1

207351297
361652262015

11

1337413

169371
168371

1

21633

11

11
1

1

5812

5812

4121
786255933544

13

13

1134
121647

1

23

1131

136823

111

14712

1211

21

21

2132
1198625

85522

32213

444631582113
137101132

142262

17

1271

11

1

1

1

11

1

11231010108

1

111

111

1

1

48122
312

452

2

1

142813215
1221

11121314

34

7292103966277

113523
7292103966277

2

2

831

81

3

111

111

11

11

218232

18232

18232

11

33914
496543512743

1

2

121

112

1441

4113

33111

33111

434823281837

11

51

131

1521

6820181117
151437242227

111

11

3

12

142

1

412

1

11

111
11

1

1

111

21234

1

1

314

314

11

1

1

11321
5371061

212

1251

113

11

11

14

221

25184718206

25184718206

111
11913922

28461

9152
9162

1

22

825162

825162

31321

31321

6416721
3223

52
11

41

11721

1

11711

21

11

11

147131387
215623833572136430516648458558

2143

2143

1
2143

131

22

855999937348
17111

22243

1
1212

11

12

1231

1231

22113525911
1

111

111

22

22

1

1

2133

2133

422
5591734

1

11

1

543133

21

7323112
12423526

31

1

2

1

2

11

1

11

1411

1411

11071

11071

11071

423857575734
135

3123

3123

1313112255

121

121

10671654
22133

2211

11

224322

419

25451

25451

11
3115

23

12

61333162515

61333162515

226352
347552

11

11

11

1213

1213

722211
732232

111

1

11181
11

1117
117

1

5371

5371

1786301

1786301

111

141

2

27

131211

1

1

2

11

17110924475109312471667
214363824771943428956635058471

212222

212222

212222

354266463831

131827231912

21
51491487

2541152
1541142

11

395213
395314

11

115334
84189115

211133

422131

1

112

101255123

224
101255123

8124373

1

1

831185

731154

731154

131

131

492310711
111

124

124

111

111

121

121

51

51

111

121
111

1

11

352
23

1

22

241

14

14

1

414

414

35111

35111

35111

18413035927225092
861516105

7799910
2113

2242

1

121

3222

24

1

1

111

211

11

111

2125137433320
3414323

11
11314

1133

1236

241122
341122

1

211112

7114

11

12412

261

412111

21
1

2

3211

31114

1511231

11
12

1

101

211

113214

11

11

1223

122

122

21

313324
313354

3

232321

232321

2428732

2231

19123

3141

112

16

16

11521

11521

421

421

2325

2325

122111

122111

352

352

1311

1311

771

771

11712

11712

1113

1113

1
1342423115

112411

11611

414533
417733

32

611253

322

31141

91

11212

22132

22132

1
19514516

2111

17413314

1

1

233

233

1

1

2223

2223

1

1

415976
13153127189

1

4

1

814221472
3314831

211664

2

3

1

341

3

3

11

511161011

1331

481671

121

121
17

14

1

1

11131

11131

524412
553032879321

11

24

141822

1110810114

1

11

132

1

11

3112285

11

111173095
515

111

131

21

21

211

3

414741

22

1131

1

111

121
12352

252

211154

3217561

12612

211441

5332

5331
5332

1

62721101386862
121

11
3642371

5164
5163

1

3

1113

1331

1211

111

11

351

351

311

311

51332151824

51332151824
11

41332141824

89213119
324853883934

2141

1
46225

46224

5211

1

123

1

5

11

31

122019381510
15

11

7

11151

11

1

121

14113

322

5183

1

2

612

11

1112

2121

1167

1

1

2

137513

3

115

711

342

22

111

23231

12

16316122

16316122

112

112

221228
837210413537054

473468577815
3216

21121

2

111

11

11

341

1

7555

13

1

4

21

431

1

311

61

1

12

11

2

1

1

11112

11

1

64291

211

113211

12111

12

1421

121331

1

424724

11

1313

111

11

332

1111

14122

11

138

112

1

422

1

412

11

11

21

21

421

421

1842

1842

11

11

13831
16710162568

11

111

2

1

41231

13

41

11

73522145

31

11612211410
222222

12332

12332

1

1

124

2211

21

1

31

1

11133
123853

12523

1121

53

53

7203557

51

21
7143554

7143353

2

1

112343

111222

1121

339281348

339281348
121

11

411

4132

28425835

811281111
70531187160360427394511

31022
511

211

13

11

672114102

211

34

3198

33

34231

132

112

2

128241320441

13212

12

108191120339

2131

2131

22112
43813

11

1

3

11

12

113

113

443325

443325

1128

1128

12123

12123

421315

421315

2152

2152

21211

21211

11711

2

121

131

131

54228

54228

1

1

21

21

132
13

2

121

121

3412
131342

93

1

2312

2312

4121

421
4121

1

13

13

122311

12221
12231

1

1

114

114

221

221

1

1

60930227037349424664411
6923104924335

4

11162

21

2121

24116

21431

11

2221841

31952

2

218

1

11

12

11741

1

132
122

1

15

1

612832

2

213

1111

2

11

28411

231912

152201303188273219

42

11

121
2131

21

21

6113

12

12

3223

211

456

21
2

1

1

2

112

52111

23111

51

111

1

2

71

72

7830241611
682617129

411

411

1

1621

1621

1

45251

2

2227

11

11

24131861716

11

311

1

111

68473387405947

952
752

2

1

123

351223

11121

2111

111

21331

1

23427

14

12

12441

111

11411

12

2411

2136

544

1921

1310315
1315315

5

4134

33231

47252
424

1

5112

34223

3942
31042

1

1

1

11

1121
2121

1

2

21

1

3111

11

5526232710285018683902

141711

61

245421
5133143806733

3132

1

1

2212

17925332510

11

2

162

2

1

11

112

41

1

4431

1

1

1221

481

2

1

1

211

1

11

1

2

11

161455

111

131

1

2

111

11

111

1

1

32

111

311

111

2121

2

1

115

11

11

261

111

11

15

1

12

11

2

131

212341

31

3231

1753

1

1

6

41113

1227

1710

111

1522661618

1111

1

1

21121
31121

1

11121

12411

111

19

1

1

1211

116

11521

82

11

11

1
281214637

192224

27312313

27312313

21212

21212

4571435

4571235
4571435

1

1

1041025232

84924112

84924112
17

84817112

21112

21112

195723103357086344445960347994
5311

4271

4271

2112
8222

41

21

1172

121

151

1212

1212

501511132027

501511132027

1
57141

111

3713
3712

1

5442475
12

221

2121

1114

71

1122

151

124

4556246
331314

121

11

111

219

1

11

11

2

1121

1121

12

12

5123132

1

2

1221

1111
111

10

31

2

11

121

1

21

1
11232

111

22

1

194123098257005343655951747939

194123098057003343655951447938
194063097256995343575950347934

1

1

5888112

1

2231

832445185

6244411
832445185

1

761174

1411

1411
1

1111

2

67123175
2

12212
67123155

121

2132

5622

111331

1

111

123

1

11

16171515715

6241

6241
4221

22

1721

1721

5695514

131

2121

4183413

422711

422711

35025042162272418403887
201626601813

4121241
5860771015435

1

1

2111

1

311

23222

422132

54221

11

1

24

1261

11

11

18121

1111

4131

1

1111

1

211

1

23132

2123

61513135

111011

23

2111

1

22

11

11

1

113414

232

1141

2111

1210211

142

114

11

226

31211

11

1

1

1

1
11

1

111

1

221

11

11

6

12

111
1112

2

344121
12

12310

1211

3324371
22312

12

12

1

1121

111

2

1
1111

111

1222112
1222012

1

32

32

4226

4226

2

2

117

11

16

69537748180106

69537748180106

213223

213223

21843

21843

231

231

56541
3043606313438

28212

1
8495415

111

1721

712392

2121

109542

113

2

2

1
7

6

135
259

124

11

6151291021
1

6151291020

215543

1

1

1

3121182
141

3111141

1

262

1160

1160

228423

228423

34197

34197

71327967

4322

122341

2103426
103426

2

144
2

14

2

12711

12711
12611

1

12231

1231

2

151020191611
115

112221

121

11

11

1

421

7510839
86141259

11442

71
7

1

111

21

1

1

31

31

14119
212

212

1

5

951622
1331627186

1

1

1

211614
1

11

3

11

1

1

1111

1

212

9214
9114

1

1

231

231

1
13211

21

1

111

4222211674217211963608
3251122

41251

141

2112
21121

1

32211

11

41

1

3432

21132

2152

12

54

21233

4342
4332

1

29131

1

2212
221

2

2

514321

12

114

1

211

62

12

121981598210811023590

1211

111

264
2442644

24224

211

15331

881

881

141116311017
32114

1312
4481047

3171027

1

4

422

11

1

2

111
211

1

331137

11

24

24

68241322
212245574912

311151

11111

2

1

1

1

11

11

1

1

71

2

51

1

114

1

1

2

1

1

12

111112

52121

1

2

19

12

1

2

11

11

11

11

108

11

232

1182
551653

1151

32222

111
1

11

21756324
42131

214

1

13

1117

23

1

2

1

213

114431

114431

21

11

1

56162053

31481

32312

21294

111

111
11

1

342813

342813

342813

617583

617583

319425

319425

319425

195132327328137128
13128104

611422

21322
611422

411

8756941318
112

5755941018

21

1638544

1638544

38324

38324
28324

1

1
1210472578

122

4214423

1

51

445813

2115101

1

11811

1111
2326

112

23

2315612
315183673215

1

151

212117

1211

22

1112

44282

33122

116521

423141

3121

41133

1

31

6102112204

1

563090573354
746211

922225

2

242214

7143
71431

1

462051424
11411

351641423

11

119322
19322

1

12111

21

21

31221

311

322

13111

112

234

21315
21415

1

211

3228

31922

6422

1412
142

1

12252

211

211

1311

1311

12225

12225

1081161

1081161

1211131

211111

12

164462
301314301414

21411055
21511065

11

2131

1

311
1411

11

5141215
5141225

1

31362

31362

1
261372402328

9654231214

11

11

2531

2531

6648201114
131

23145613

11

213

14

2652

31

124

21111
17718171113

127532

127532

143
4

1

3

122

122

311
1037415

5354

2231

11221

11221

223

223

237131155

237131155

237131155

237131155

237131155

36234219362259152
53157105141477771387219589673

94888728116073321071266567
2101834054792222207

492256211219

1513
271634999

33411

538112

121
1362112

124212

3122

2631

48122

194112

194112

12816

146

241

319224

212122

1712

1
119561871687042

377228225
112

13521071

231410102

5652

314543

312432

2111

106451021243930
196131683

217541

112

635961

14212

34651

3310811

214

2124

447171721

535634

23243

42211

473326

511286

124112

33845
31845

1

1

1

7291154
4321

62513

12332

5930601629432362925841969
3890391691926

23714163

13910

211443

1312

34088955908970345282
5771576024332310724381835

3339101
3139101

1
2

1

1215214

33

221116355
231116355

1

11101

93592

5610930536

1141411

3873953

1

4641413

104112812
117122912

1311

921

27146758106
26146758106

1

21462

15314

542010310

4581392013

112921

77101381
6691271

11111

20102217125
31

16242

1642012123
164208123

4

112

334523

6143117

2281611

27311

21282

5711

151

68172272

1142

2241

1751011

1

4341641

424316

33531

2942917

338105

5952641

12015

27481843

61

4211

1232

44623

12
9178115

9166115

21353

351413514

13

1133532

1

1171

1871

2105763

231581

641

21

41361

348

1

1

225191022

2431314

1313361

89636391112

8

11212251

2212

3371612

2912

128972

1916361

313633

3381961

1

3413151235

7915653

111031

55222235

11

45747

11

237432

1

1

21210

81241612

188121613

232251

321

2251

11714713

5411621

203141441

7472624

1112

81311162

362482

21761

21

239332994

1101323

149131071

14322

6111091384
6101091374

1

1

1242421

492421218

834

196221

2151

121223

261
2261

2

1211

9241

122135

71521311

14

2111

33171

47

12245514

1621

94111425

413923

312513

1115511

21161

323138
323148

1

1

1

1312

11

1

181595
3142

15175

9412116

4711522

4711522

13

2329113

189278887

62122122

45291922

1

3339823

32

193

651

4122

12

24352

66351442

21521

442

2371113

228275

3172261

61212

1476943

465762

111

112412

8826711

11145

13

29491443

253812

806762653843

318111

122412

569131

15264

11

121841

118231

81286

2111

94303412

919234
919334

1

211411642

114341

1229

12640551918871321983

6633012

1

2732703331

8154

125113521

421143
41142

21

4263022

121485

2015744410489

221141

4426351913

1

81361

17124917

92442

4213912

2

11

121581
221681

1

1

92158

17211

13117

511322

6152511

14361

223115

1

33116

1

1761423610

211821

8734210211

25145

314111122
166320542518

31041196

2143746

51511062

31011542

11102051915

43101415
11102051915

22716

121821
11821

11

213711

21322

32322

95102246
124

85431

155611

262
2426541001713

17131432

72543

33191

2141

32712

1614822

9794475

141192276
1

985955

5341311

139132171118
4545342834746

24482

812441

2161

1311

8785

11733

12151

479221

33

221131652

211123
211113

1

522541

8211426

1451

1113

22

39721

39721

40638281473363492

37637275466350485

16331130263190261
37637275466350485

1209109131113160
1209109129113158

22

2

10

11417

1

1

122

11

2112

4111

11

11

7111

311

5312

195736

21

1421047
1421257

21

1

177556

1

21

11

331

12

2209322324

224

21

21

21

3147136

1121
3147136

1253
1153

1

2

1

11

12

2152

630512406578916121530
2451

219152
411

123

11

111

21

246183
12

111

11

12

1

12351

12

12

22265
11225

2

11

11

311

311

223162
74242563

11331

121

11

221

1

111

1

111

1132

48211042170777696
616498401975915801516

55164

1

163334
3233

13131

11

1

122

2

111

12

1

111
11

1

121516172911
31484

9141613217

4187512
2186412

211

12

1

1

811492
3819968616035

11

11

3018864525932

1

1

11141

233

522
111

411

1

1

2

1211

11

1

5

313

2321652565353421432
4372332638448500544

1

1

19668718070107
19665698065106

1

24

24

1

1

1

1

231

11

1

1

11

32

52922

1

25523569

214

111
1111

1

1

1221
13222

21

1

15584546
116695251

1

1

1

110164

1

8243114037
8230113534

1

12

1

11

1

1

2

1

121

2

2

1

11

11

2

1164

21457151

1

1

51

211

11

14122

1114

21614

2765

111

23145
123155

1
11

1

2623125

2013207915

1852612

1852612

1358121
15515221

11

6

11

4312

11

3212

197210983236292019012094
10565761143550

123

123

111

111

454238

1131

21

11127

131

51101053
1111

131

22

41

1212

2125

421241

42231

11

113554
122

2

111

21

1221

125131969
11

222
1222

1

42923

222311

11

233
111

122

1

1312

2121

33121012

33121012
2152

127812

2741
43311641

31

2

1

11

115

2

1

222
212

1

191

13821
111

12

11

16

105111577
21

4193

12121

131

116151

4211

211
24121422

231

1311

23

62

1141

44172514
1

241

411

42112

19

3

333267
1

111

12141

111

11114

11

11

2155

221

3

14

12

12

11

11

52238

52238

1

1

111

111

7612277

116121

1111

64555

261716302515
3181672

1

121

1

1

1

221413

1

111

12

2

1

133152

381273

37261

37261

11

12

2

2

211113
111113

1

101422

101422

13

13

21334

11231

113

11

11

42121
3

11

2111

14898292690242415781749
190115455237138108

1121

1

1

12211

5411

141

2141

99181933

1

1

7141

3321133

43255

12214

51273932822
51273933822

1

1

26351

4627143872525
73731212605448801142

337141657201513596
336136650196509596

1

41

11

1

1

1114

31

1

5

11466184101117221

11

531617

7245175470

1064185333551
1976088503852

1

1

2

761

15181731

121

1112

46377

5141358

151212061119156

212412

1245

12211

1111

2

1

29

2311

621

121

22

131

2211

2812

121

242

3121

1127634

6511

2

1116333

14232

212

11

11111

541851411

26233

121022

23774319

1

31258241

2

1

12

11

17

111

2121015

111521

1046612

2

21

121

2312

1252

2

5312

13123

1234

835621

3173

1

111

11

12

12

41264

7210112

211422

1

18168528626

5

1

424455
425455

1

51411

33331

1132

64631

12

1

31

17114

2

11

412

123131

211

1

3215

1

3121

239122

1

161

121412

2

2

213

11

31

21113

14

11

431123

6333

52232

1

1

2

253

27123

11

62232
62332

1

1211

32112

35216

315262

4111

12532

11

1

2324

162

1

111

12111

111

1

53373

31

12

2

437411

319722

1423

111

811

1991069

15322

2222

1

1312

54

212232

3

1222

841143

29921

2

14521

111

314

111143

11132

2

112

12

214

8237823

1421

3311

22111

24103691

24103691
11

2493681

21211

622

3123

7430672
204318464118114

222763726

21331

21331

11911

1113
2113

1

1631

12181025
3113822912

273

111331
2116333

123

12

82241
82221

1

1

41640256271
41744266271

4

11

11

2

23241

1

1121

1

2122

1162

1

1

53414

211

11242

3353

211

2211

91941

337211

31312

223

241112

2161

7447

11

1111

215242

1

1122

441

223122

1

1

1

11

41

111

111

1301

1

1

411

11121

113

1

12132

12

23412

11

1

2

2122

657223

1

441152

121

12

1

1

35224

22

110

51111

4

1122

137711

233543

23714

6

21

2152

13322

211

111

1881

1312

715623

1

2

19765

1

522

5422

31

1232

121

341

21

485

2177912

1

2

18410462

2211

21421

1

1213

9413

22231

1211

4413181122

1112

1

222

1121

1132

121

11111

2421

21

1

11311

42251

3341

11211

1111

41

71815

2

3111

21253

1

2

32333

112243

112243

1111
31651

2441

1

2441685
402151452915

63111

75

131624

11511

1

134

34

2227221

12211

3

13

236

12742

3142

11

11

1
12881134

14231

5121

324513

21211

111111

111111

144131

1132

3111

1

19162315208
75311

26436

7243

31321

13111

141

312

1

3338

1821622526
1416212

2141

23

1

171612

121

1211

1211

23131094
492260473894

2

1231

315132

17111

121

1

2233

1171

142323

521

2641

92241

415322

826762

23
2

3

341

1312

12174

1115

11

114

116431
111

1

1

131

1

5

262556343732
7612761

1

373158

121

10913124

214164

111

111

11

612

11

121

13621

215

12

111

12114

3312

1211

34917725

34917725

249712
281525191010

3112

21

1

1255223

1111

4411

3321

11

113

1131

21

12

4124

322

1

111

9496145
1

4141

1121

1112

111

1121

11

13

2

118

12112

12112

724

724

44472
117801161166560

347255
131

1111

21113

13221

24211836917
3421416

11

322

1

1

1

3623

13582

2

21521

2

1

3231

103113

321

1

3111
266343

1

1

1

125

11132

221

221

737323
12

1222

513321

517331
35114114129

12

11

22311122

711121

2311

111

1431

123211

131

8512

8512

511722
121

1

11

4412
412

31

32871
21

2331

121

212

132
6311853

21132

112

111

21

15221

111
111210111211

21121

8725

3101465

33

1212

1212

262471

22341

111

311

11

45713

142

35311

519612
3217714020821893

23338131

131411
171411

4

42343
623412

29

1642631155
2937312819420490

1012332

625244

526
5126

1

21111

7113
7313

2

127107

37220821
37220831

1

411

512

42252

411

411

21
211

1

9371
1311

1

85

35121
3311

211

2

102152231
8152211

11

121

1494234668050
15849519011471

121

11

1

1

11261611

1

1

951179

276

11

6

251

8033158877046
52277103

111482

111482

521119211715

521119211715

1611

1611

31

31

3211

3211

53611

53611

528331

528331

12025

12025

171021
43251443

132

121

1611

21711

1

165469

165469

11

11

3122463
1

1183

213433

214151

214151

17152

17152

574232158419424
433071752295137814145732954

389584879338851159242
25415239751

282132596
51162

21

411

112

13211

3

182932

1

2

2

2234

2234

4161

4161

12

12

32221

321

22

25233858
7121122

121

1

1511214

1181

511

19

19
18

1

15424111

15424111

12166142
715221

2131

1

210

1

11

4

4

12315632
539211

1

6632

11

384593232335331145921
23433120346752

35203218310681053414
35447229313151060915

21011210601

343715

162

1991470141

44324701471
45124721491

822

724

2122

212

2

41

2

3161

341

13

214341

111

111

111

11

11

11

11

11

831405523646546446
4111

724267784429
805392505631531440

11

3

1552

6253

12

1

521

762

121
21

1

1

116

221

6
4

2

1

121

11111

11

233

11

11244

1

11

11

111

22

2233
336175538673

44476
34415

161

16

1

275773467964

12656

121

1342

2211

1

11

543

1

2

1111

11111

24

1
111

11

2

625824
2242

4184

23711

12

21

11

1121321

52935203742
53237234544

32242

4

1

19112

385945011691122
385904111166108

4952514

131

1131

211111

1

223

11

2

41225

22

1111
11

11

1

11

14
3

11

11

671222
14122

532

4112

113

1

121

111

1

121

221

616175

111

3231

11124

2

2122

12

1221

2

121

21

2113

1

21

151

12

11
1892

891

321381
311381

1

2164
1164

1

121

1

11

1

121
231

11

12

41311

1

1216

1

111

11

1122

111
11

1

111

11

1

2143

142

11

3

111

11
211

2

1241

1
2

1

1422

2

122122

1

21

2111

61141

11

11

95371237367
115376317368

4

2

11

2

24

2

2311

28311382012

3161

24211512

11

1111
111

1

221

27719108
27718108

1

1

2712

2

54
53

1

541
311

23

1
22

12

412693

114121

11

1

10221

362110217

211

3172

2

1

31

1122

21

2112

11

3

3

11

20131712146
1131

5131

11

31

1123

311

21

2311

11

756173

5311
756173

253162

2131

22

22

3936385310149
118

235
115132086

1

21

1221
21

12

11

128
127

1

1

112

11

23

113

4241

11

223638
162614118132

3111

225

1

1613

616164
9211639

35575

35575

21

21106

13

1

1

63109117

12516
13726

121

4101

1371

1
51154

13

41

121

2

30823313719

1232

1232

132

1

131

223

1141
414722

11

1

1

2111

1111

2441614309
1418852

11

33

11

33

1131

33111

211

2202

11431

11431

1211
13949311866161815372113

75249472011
73444

11

1214

21

6211

321522297
311421997

13
11

2

1

74117
94117

2

1
14

4

4521
21

311

4

13159061855156515152100
11762280161222250

1

1

1122

11

2

2

111

1

2

11211

2152

1

2545715
2445513

11

1

11

121

1

1

1

1165
11156

91

12

11

32

11211
22311

111

3

2

31

1

12
2

1

32652
3652

2

1

22332

1

1

155851

12

1215
1114

11

472101013
584101014

121

1

2155114
2155104

1

53

8232

61274199196
1

1914
2510351

1

24121

4764168595
4760167791

484

1020711122811169241522
1017711122811149231520

21

1

1

1

11

21131
21141

1

131

11

34117362113
411213452213

1

1

7158

1

3

2

2122
2123

1

11

11143

1

1

1

33244251

1

14

1

12

21

1

28

11

1

2

1

222763445720
8

12

148442
222762365520

326733

182148254815

182148254815

11

9

11

1

22

12
112

1

1

221

211

3216102621
4216102621

1

131

7671287
433435423429

24

746421

292420222421

12
1

2

111

1132

1432

11

7

1

32

11146910

11

11

1

1

2

2

2

122121

1

11141
1481300401997566260

2212
1353203

7110

24

443

2

152

152

311
71821

41

71

1

1621236329714259
1457292395941555257

311211

17

1

11

2

111

6121

2

22

1331

16222

7

12
10

2

3115

12121

83103114

2121751

2

152159

9211430

11131

211

422

1122

252

13195
3145

15

20819401686125
19218361465525

21

1

2

31

1

112

781

971

1

3111162

142

43

5321

11

3

4

14774155139158134
14776155142158134

1

3

1

946642

45

1

931552

4

551

1214

5111

2

1

1113

121

617

316

5292

24376102

11021424
11021212

1

112

2181

2182

1

3111
2111

1

20410677

1

11

115

123

213

68232319132

11

23521

454

321

1425915
62529

876

1

431

511232
511242

1

42

42

141

141

121112

121112

1

1

1

342250864134

342250864134

32181
342250864134

411321

121

111

311

211

211

62274
1

112

1

12

51252

1

16411
2

21
14411

12311

121
423101

31

39

1

1

2122

2122

1112

1112

11

11

3292016
2191715

121
131

1

1

121
24161

141

112

1411

1411

211

211

1

1

222531
1

221

11

2221

1

1

1111

1111

1312

1312

428534
1311

1

2712

1

1

1211

2111

2111

6321

21
221

11

412

111342

111342

111

1121

2

1

1

111104180151136117
133

51271886

51271886

51271886

51271886

51271886

121923242917

121923242917

121923242917

121923242917

111921242917
121923242917

12

93731471099991
161731332118

2

24542

79219710

43325

2514

11910411

1

1

2

21245

126

31299107

1

121141

12

2

1

1

13222

162248334037

13312

788465908774527546
241216212415

13717131011

13717131011

13717131011

7314847

7314847

643564

643564

1083654514655

212655

212655

212655

212655

6517952

6517952

6517952

1003035363648

1003035363648

1003035363648

1003035363648
21

9420131011

11

11

742012910
942013910

21

912615232436

85249211629
912615232436

626287

1111
463876762753

422862562141

422862562141

21813834
422862562141

141433291518
131433261517

131

11

113210

4331624

2

21

212

81

3101319512

3101319512

3101319512
327616

54722

32624

1
326206442356252237

231540161315

231540161315

191620221217
22431

1111

114322

111

32

1

1

322331

1111

31321

124

134211

12213

2222

111

141017211210
284175382317227205

3

2111

112

121

622770553324

1111

11791087

111

2121

5914929

2131

715464

121

1121

1111

1

10271214

111

23214265

1

633113

642

11

11

1722

133

347561

312312

3

111

1110111157

112

311

33213

23212

1132

111

31

7410854

4131

1

2221

839747

2114

21

21211

127221

325331

121

121

1641

39

21

2

121323

1

11

8410637

2121

31

11

111

121

835832

5342254

1

2213

2311

212

25155712

2

11

2

1

22284

126113

1111

313

154241

12

111

11

1

2151

11

111

121346291638

11

1

286644

132

22371

111122

1511

2

311412

1113

1

21121

1

1
140621511197175

10553110853956

10553110853956
5421

23544

23544

1152426168

12

212335

2232

41622

131561

121

11312

393342

393342

2132

2132

102765

102765

1242215410
1

32151114

213

925332

3212191236

3212191236

11335

11335

7106653

7106653

225116210

225116210

449233
34941343219

514462

11

74111

2644

111112

1

31

31688

11823

57274

5223

281615271222

233423

233423

11
233423

123313
233313

11

4341144
1

1

13

213314

43

111

2210812615
12

134412

3

1112

833237

5412

12141610810

12141610810

12141610810

12141610810

1214169810
12141610810

1

6133
1

122

1

411

685392744846

444071484233
1

101843333221
1

481811159
111

3411595

3411595

136564

136564

6925221712

6925221712
4211583

132333

18431

1

1

1

1252

121433

332228151012

332228151012

12111
332228151012

2317161047

1

1

9510453

24132126613

24132126613

15811612
24132126613

1

1

11

4351238

4351238

524713

172026272622

172026272622

172026272622

871214711

851214711
871214711

2

91314131911

91314131911

110339231747684197316690609275254152
232383409413606710460

61521481489861
2111

58481431409459
11

11564

11561

3

43351181146044
221

161945492622
212324

661526810

8112719168
8122820168

111

414834

414834
413834

1

2161031

2161031
261031

1

73151943

73151943

1413
2791275

31

1

11211

1241041

1252716158
2512

232

1112962
1112952

1

523251

22322

1113

131220262810

131220262810

131220262810
13912202710

3861

23731
144732

1

11

11

11

160130631148295139

154126623144289136

154126623144289136

43713
154126623144289136

125109558126270118
128112568127272121

3310123

1182711117
1182611117

1

11321655

648463

648463

648463

648463

216187462448408191
151330221019

26132

462615255

33122

1324

488625

15517301311

11131

128213712

1

133531

1

1

111

1

51441

11141810148

31

12112

121

1552066135

112

159546

6756482624

11

9971254

92512854

65191894

547523

14435

26282

562541912

321255

11

3462

43152

2222

171627262222

3

72203895

12

638411

3662319114

137133

2231

111

211

941116

1226

1

211
302497541714329268

11152129137
295481520699304255

104941511677666

104941511677666
5128645

384150513024

6141931104237

455198936637

455198936637

455098936637
455198936637

1

472521121498689

332861112
472521121498689

2012948703642

9142524149

157731492526

88691382616356
1131

262319271520

3723701872416

242348442419

5141413209

5141413209

5141413209

5141413209

225144

225144

109246230403678093313614601281252919
2562551160834173414532538683

1181627333981126693
4918438023548131464094314270

21
2051941278891807893

81353483131

81353483131

1931801192821760846
5856364213244266

411

1111
11111

1

21

41826239
41826269

3

1111422

19223

261321191129
261423191129

12

115235

22351679
22351779

1

11111255

47134166
48135196

113

8514663186212
8414162180201

151611

1

7334401114
7536411214

1

1211

14241054
424954

11

116129

3151145

462724910
462728910

4

152453293137

4221361510

1111142

3268153

26162538

34654

1121461
1119341

112

1

281611149

2169

9117112

231012122
241112122

11

10517678

115211

11131

243439127

127614

11

12434

1583416169
1583316158

111

1111487

5126
2123

33

15896

173

62315

4759

3429811612
3428791312

123

231814815
231814816

1

4131211616

4131211616

1216493626740
1469133551583607288023502

4735281111

4735281111

57749214031292724642
113113249223110117

12141141

261511108

13431

312131757

5114317135

761526203

921262

46561788

6522889

24161173

34653

12

2

11101825723
141219331825

3218112

213532

79183748

166341499

1310398108

271911830

12231

481716209

2121374

741528211

129932

16523231911

11132

1316122

2331276

1862625157

71318161123

14

443119811

2471

11

1281011710

3281352

2132092

15826

268795

731421162

263
81533112913

693382913

852223515

12612

71015121113

128743

1

113

122

2122

3610564

2571265

1156474

329543

31162

133653

7827211511

171021

2221131

58151388

861619630

314941

46813113

1937181010

1632

415111

728854

46214165

196151167

1161

141217301412

615241816
1719262117

114231

9191244

861116117

2211831

1131512115

756536

2281765

241141

18133589

356451468

61040171214

1

3295133

32372

1152522514

2152

111

152726159

1622126109

87682036712251278002809
4272901402806246422169

511

17917

1234142435512
1233140425511

1211

241

312371

82

622

321981

1123

1121429

121

2111

14512

91615

2111

1626

26

2136

432633

143121

11

372838224

3462

11672111

1013

2

11212

442536147

3232

13114111

45371

12232

119

132341

1312

216412

1267592

42
31

11

14534

5142125356

221424159

2265

3171085

112

331413163

282828159
282528159

3

111151

35882

213842

751013105

1119999
1118919

18

23321

141

1213723

162151523613
162151524113

5

1

185

3162168

149515

4431

83332244

1301213

65871499322174

217827

1622

15821042

33543

12

1214228324413
1215231324714

1231

1

21125

33

1111

8180232013
8181242113

1

1

1

382415136

1233374

372390206629
372390226729

21

11124

1721

31

1441143

811114491408106
81111446110983

1446

22

114013

1864

7

415628474629
201123372715

212

2145391714

3241

44624

2314682464431

134611

421920104

5916791035561

121

288171966

74547

2416653

74474694010

2241

515

439944
8421164

422

12

1

115472

124

1

2123305

3351274

135955

11

65222659
65222559

1

31271

11331

465

783137248
673133185

11463

2213

541533183

97171463

1

126

141

4093792844180612151069
6577233831

337332169712571101963
9389472363421289

854

127127917

1

124241

3622171178

1

18543

122263
26136154

1411491

13

181246

13222
12222

1

156321078

22271498

11171786
11161786

1

10109

1

7746193312
21510132

753192010

1714117

111

1

3

1

21151284

1311376

3

716642

32

1

129456
129256

2

1

321016159

11

11213

12

1

1102
182

2

41

32111056

1

111

21234

1

1691879

2112

113452

1

1

11

1181377

21431111

136834

14
214

1

1

1645163838
1849203839

231

1

4

2

1

245347
215347

3

5543

1911452

241641015
241641014

1

2311451
2310231

122

31

12

1

24544

1713124

13

112494

13

2

101613116

1224111583631

2

2141015

1

1521232

11

35313

1244
11245

11

122361226

72141765
72141764

1

3173810
3173811

1

1

1

111

11

112552

1

421
1422

11

1

14153888
14163888

1

243110

1323221716
1222221616

111

52314

1

121

4522

12

191336

1672411136

1410326
1410316

1

14666

211

2114976
2114986

1

123311

1

2

2231752

221

1

113211

1213977

1

55144

1

312
12

21

211335

211186

39765

1

11111876

37223

1

3113686

12623

39467

4513745

111

1

21

4347

121

1242

264131639

2

11134

3249

311

74174

210935

22211644

1841

32272657

5825111918
3822111818

231

111661

111

12

1

4141367

2311

15841
13821

22

1151342

61560383439
61459383339

111

9722201211
9622201011

2

1

114121

2

583810595036460

583810595036460

1
8411231215

84823915

2

1

11

1994679199444983984
1

1389676996944833975

1389676996944833975

652225149

652225149

111
249020445764487637423457

9104531824
242918512

557516

219816
219826

1

248120345718484437333433
55336512271142642675

351030251215
351031261215

1

1

27171365

521224

11615151514

11

5561815

112

21331

11

34183560847
33183560847

1

1

2233

212

1955493

1

2421104

1

42161194

4221

7138131012

154152599

4812854

123435532933

3

16912

14

121

2121

21311

20123130712

339514

516812

5141619113
7790196274181115

11

312
212

1

412421
6299122

2165101

1

34429613511973
466011518813581

1

714132997

2

6981775

1531222

1

1

215
1

115

1

51217
5221821

1

1

1

2

5

1

111

3

321421

815238

64232999

211

3314647

12523
12723

2

45216

21

1

2411

26191676

11

42241268

222

89111143

358629104112

111

1
1926114552227

1317104311618
1317102301618

1

2

2

13131

142103

22213

112332

2118

19325303531

1514886

12101010

3211964

11213

25743

21511

16331

111

761716137

1422

13212

229021511
252322857643780372

131417129
131632129

215

123105

114331

1112

243310718571738345

1069220926222091
243310718571738345

881691423

101172433254428201

223553427228
203353417028

2212

11

12

5231

1

121

13

1

1313451

21217

7154

7154

3962719528

345795

3471127

12441

1

732324107

511129106

2832

66561475

1341718118

1152

2123

1542

14222

1865245

244753

1111

111

14694

1417634

1115
342216158

2

242113108

221

37189123
37179113

11

1

323222

34101575

547844

1

121

1213

4558551411

223321

9181741

2111

10532461317

2231

34441

1151

14811

6105059314

17732

43158613

7168910

2227741
226731

1

11

1

1442

6734481316

7586911

1113

1

14

13213

1112

117221

737975

1071174

2

291

11211

1675

158393

15101832164

613611

2

111

224287

74111724

12

2

246161833

349342

973514915

1531

4815161414

22764

32923

993529174

12261

31023788

446844

327837

1

13753

64611

2818121517

23924291012

1

344211

113131

11

2632

554102

4471448

4231076

683258613959

1181215

11

34443

2882020155

13123

214612

2

2313494

9516987

9830411313

326783

1710923311

229105

4321388

226433

122

136311

13222

121

12

277321887

810281845

810281845

12

11191297

211

121251

9925202440

1

21121

5162444

11

23

121

16425253733

4

5

228512

3513

41

2131116125

15341362

214953

50571278910088
78681559710794

417312

223153

22818411

1154

1211

523153

1

1348102

12311
12321

1

818655
13555

5151

2

11

11

25511
25611

1

76151778
76151777

1

5114857

16712

7432271114

143733662625

122

5231

45331

61621

6617181514

11

111
1

11

1131052

6417554
6416454

11

21

73251387

111

4271223
492566625955

28251

114211

123

136223

4116

148151067

113221657

12
1221

21

12255

1133

11118

6232

84312

12183417

372353787452647795
83121127

1

236491
11

23549

347309726408599773
359342764433630783

2

1

1

1

413

413

1

162

1

2

1

1

1

111128

111128

1

21

1

3

22

11

11112711176

1

11

121422
21422

1

11231
221

111

12

12

1364

11

23

23

118

122611

211

413131

44916512

9182036912
8131928811

521

1161

2417453

26243

37731

211

441010525

1179

1105372
1117372

12

111

1

24222

224832

63969

21232

1332

663951833047
214141589

15132023717

302217451521

352

12131

132221

16431

532

1

222

641416127
641416167

3

1

1221

4

1591669

79792431787971

162083832320
79792431787971

13211

1011241246

66291359

3110171867

143411

551711410

312818114

72139172413

121427372211

121427372211
2241

591612134
591311133

311

5

5

7591856
7581446

141

503519912911452

503519912911452
6549513817

353721157

84115

124010109

2102629127

982811145

295113142

6744511230538538

3222321136395396
6744511230538538

15682

449134244
448124038

1126

25966

124061019

6930331613

412443134

1

34115

125585

4725171711

1333

79251893
859904252125481069931

43941711811216508455
151856672612

892021127

892021127

1191645
30561561437067

11102426135

111447283143

2182832129

11311

512354095

222576832829
134140433440189162

11111134511

91435321315

14837262925

1

2

172887493924

1021972

162

14926351511

351436910

451414112

18876471811

4143438813

811193059

199143586

199143586

53631181135276
14302314731

10520131612

149312261

67141524

91230492128

552151281513

552151281513

44261711464434
19745281213

4101631

35131113

1064744119

724038136

1616942

1442744118

1442744118

67711351798170
7514241612

83421251414
83422261414

11

20949641411

21283

211245413118

910316612

242226630648228226
528813

112052261412

112052261412

6929311023

6929311023

118101775
128131876

211

11

19941481410
208187528565196182

523222167

11625311012

552033148

10492787

188393978

46111576

122223161016

883233811

171525341817

81131321711

583031159

61456391522

1

521575391611

6825461111

75211585

63123048

917313673

6481562
171252685666324247

2131061
142132673514

2371654
2372054

4

36918134
361118134

2

7111119115

22541221386163
129194589518237195

4532

135432

617231537

141523515

7223230309

2

256762

271916167

329432

342222107

614714

338574

1

21

21141041

2123

104172691

611232996

2423221612

326811

2410732

2316952

472226105

331013510

34131854

57211767

52131331

3

1010731553

2215921

92715611

114322
110322

4

7591239

7591239

9173118179

9172815169
9173118179

331

61116362618

61116362618

166152716593355232
11442

11523
11312

11

1

1

112

112

112

12743
165150710583349229

4102627198

312412

12444361423

3736441512

7841151413

22675

7112741014422

17779210

114

11

5277755

21

12387910

10924151211

3135781044440

21441

111444892118

17571245

1782093

34118174

1361824912

41361392925

472240387

371830375
44

4324

3121

537

14432

12411

61

1
141012

382

11

11

170185217552407510360

170185217552407510360
539

1314541072219

1314541072219

1327691031720

1327691031720

91653852417
27351141864946

55274567

131434561922

117177115152002422275

117177015152001422275
116174914791963403274

1213638191

11

2111
254150466744476252

3571163
254150464743475251

11282
22102017

11272

451

1
211

111

1

124712

124712
24712

1

13
1017441916224

1017431915923
916411815922

1

1211

1

1831

1831

373

221

151
152

1

13611

13611

1516671848134
228124387655298209

1331

51

15211
1

2

111

11

11

7111

231319811

1195

413

24535
24635

1

276

26108

11411

3110

131

461994

125911

1112

3754

2

181513

181226117

1317

1483225453232
1232923342927

25321135

1313

3812

11

2

22

21

141716

25321
122

1311

11

226631

22952

1

12

11112
111

1

11

1412

312311

1141

13222

256

11

151041

2381
24812

111

332932511
23283138

1

11

1

1

1
122

22

113324

13121

2191

12

1916

312833
212723

1

11

11

234

483

44324

22551
229614

4113

371

315334

121

3532

476102
486102

1

1

35911

114193

13163

101544294419
12421

11

91340264318

121811
261921

1411

11

111
861031

243

736

169914734526438720921667

169914734526438720921667
26511591869366

55352021498359
21630411716

11351

57131346
56131346

1

5231

61301453
41291453

21

113

111762

7301395

23421

411512185
1111613185

711

329863

2101074

1218649
2219769

1

112

14151435

211121
404679775147

91247131019

1213927199

172022362018

39634012131040448465
84782341688769

11

12122617148

8332272010

14154442139

134521

204271163

121

43327

1331114
22113

111101

121757573315

135172779

6111039712

149523
149633

11

33124093

149291985

8549421818

14151924129

1886936468

6121926

1

23192173

421310224

84242166

1

146115
136105

11

4861464

434317918

52612105

21521

64262416

14

27443

1

852731206

5820211410

51255502324
51254492124

1

12

618126212

13824202313
13824192313

1

145131551

115523

218851

20124438107

2912281015

31291972

23143130717

25

25

14151117

113121347

17945
41301856

3123911

242080613121
17272099

1972123149

46321883

92632553615
2471151

671424224

1151120910

31120191411
274565693343

317111364
317111374

1

2

2

1882122722

39131536
39131436

1

12
181593924759

581118107

7222311137

6559432415

311
613381783029

452238441515

131143331414

161428271012

51

16142327912

141837562715

141837562715

90762831987667
42210136178

4122543615

711173289

331341331611

14

14

91050241911
91047231710

1

3111

43252257

28520856

68361761706957

68361761706957

1363
434718115012249

10112026385

101254321812

77192797

98272496

7948354519

61611731448261
61221132812

217301598

2219932

6184216311

9252494

8112143810

539141

2111315

441510178

2141
77331081487264

61142262211

52833541416

171333683236

695822121212684
151415104

1211

1211

422617187

362824105

201041472219

1521415124

19121476

11918351417

131566443222
121354333017

12121125

461317118

461317118

11224230258

11224230258

52
171634471116

2214335

15141542811

3138891015435
212

131427412918
111424372917

2341

182460592515

441992835714
135441701609942

6103634158

2111

519435

321111513

481116231510

42

161

19313632
22112840156

25121872

1331652

3853829391080441339
79812242659988

1

152036261722

523593772719

8718311010

21

1

163557641527

3251525

431946582518

24171

11

262348642516

3532821043421

871110115

2

222046421819

5103129197

11

6230441014

141341502210

16

25911145

122128301411

1832

531

13164153199
13174254209

1111

1

11

11837382516

1431

61232462011

1

36522

58346316202607689616
206581891620

13939391614

13939391614

13939391614

114173
47238813082067562505

154103308363136125
3463018701106375379

1

411976

5172216135

11351

322

531637211
318513

2283218

11

733636138

1237452

891819713

1

42108813

1

157241475

936453

1

111

131

1

251

3141

218372994

12341

4410612

1

102538582419

1

117191595

212

8122158

1

353839107

1

3

491336610

37331372874689

6

82353502423

121

48911126

1

121123151810

12586434944187123
121

1026236978116090

2324641612633

64481511988159

64481511988159
2491716

191973764320

4325691053733

1412641141418

1412641141418

1412641141418

418425328313484

418425328313484

23171523

23171523

9928482011

9928482011

36332875
307220822011270

3427362422

3102616133

91723251111

61553653114

71528135

2317842

410141498

512202882

512202882

510202882
512202882

2

40046314631682781497

30391271437947
40046314631682781497

4626771313234
1

2192839717

1682045119

992946148

176208641703340183
2232691024523

62181849

391530223

189831222820

2493818812

1111204467

39541294

792135126

893130138

2611191011

63133996

4723241311

183583538814

622126197

1710221686

483141148

371623119

82656421218
82456411216

212

6516991

358
537228332813579

2548741914

141463452510

111032692013

143197714132

91238613010

271337573727
95118335377195154

391625610

41030241416

271844421821

1293123315

4524241210

820311011

4734492116

522829208

52041271120

17111585
27121696

11111

210183064

88287204190514691233597468461160991
148734198676707285542190

51434165
1301648991245401217

8998634750214135
810581393119

131340862416

327941116

41120471611

35282393

221223292212

632931105

2425341015

6131346104

2614311110

423169156

51121621

34212386

319552810

753349246

14133435

416125146117177
716541061811

522446124

24182377

43124496

3722705211

562636147

17193296

561548236

332014117

5421231211

13201941

1562547990143384184151010389

31088907289710388983053
1562547990143384184151010389

15

15

8587192571030516424650189

1812363873810

622302733013

3633194849928170891589664
3556190349752167671555463

774501763223421

14511710226992449

57100120364615919

4870260396

6782125193012426

410161184136
476616321878161205509

30345719464795803388
5289252101914770

8121231961710

2929803323811

7141601503010

8144341263917

201464902116

791556125

1111421713018
1011421622918

191

3213102

1521443542821

1

1337822005734

71434172268

36415116

81639942413

88321181610

2151122

111451255337
111451256337

1

814371473914
814371413914

6

5631841017

1027532666516
1029552796517

22131

101337142184

1017671211818

1

1

471051621310
571051701310

18

473076175

1101763326

2314501132117

2453845

1325361923515
1022361913515

331

10193652197328147
265217812548729

6014772431267

71447326309

1339752

7131175362

2311532211310

2311532211310

36251463715233

25131111893121
25141151943222

14511

1111311772011

1111311772011

8172352513916

8172352513916

13252149

13252149

1412113045
951018246409471179

1617155202259371
107718898146

1

1

119234155

411341318

154746100149
15464497145

1234

11343

24743

2421103241
3433251148275336

191910181101143

131012957142152

51093748899

51093748899

171913994113148

171913994113148

5487437377
2218174111182180

1465

161066467768

1316212128

1152

1420812097529
1

1420812087529
32853

592556337

911531243719

11523727731479338668
1762222821340903248215530

62590475324

62590475324

39789161119144208
1

39687103112132204

12577124
1152162

15662

5181978921121

513120713215

577181796

23161858

23161858

544
1529138914844

9939472433

6184238249
62045402411

2322

101456474748
4045250143152174

11

2

1

111

1

12

12

1

262297616577
261992606073

3131

3223

117182

1111

2332

2581242542

1

111

1

3

243421480011956781180
843140415205436629723598

52918113

111253

343723910

12201311014632

164918534

31415713852

515135983527

32141151282

62233301122

14613

55231923

42214130460

1457282035

2121626

7143725516

21121163

32542

9644126012

53413

2243118

45

111022121613

1

2

11357281940

662320148

7336252124

3125947

6935512211

333214811

231

4153

9566401125

742819218

30412

2755191214

1041

1019112973840
13221241014240

331244

1

59101221311

3438392017

436214108

81258381017

9172584

743416239

621

1

112

342610914

670752427

11867601421

11643

7492

41558121324

1

1385823189

42181887

2238818765

1119108724330

231125235738314

16643221225

31011535

2237158

2810611169

2449521097

36125381244

1211274

491677277517

1226187723050

52125151522

2816

31634

11885171211

1

111171391

6211156

3844151628

91467542217

62219319

143714914

9

492415818

1569111512

33332746

117142493316

51910131

386031811

622918

99209711515

544618133

2935242616

41033503110

1

2627321213

2218137533115

253721234

9527133163

1110141

3133331111

4810629426

616128323451

3838161212

726128231283

235136148

6569442219

1141118167

21104533517

293029715

3749101917

584230317
61046301019

12472

2951192618

1

14161418

238014107

31810828967

111653

4104319617

1140939

29371045

19184351247136

31653661529

2

12941

59105472013

1

11

21

6317512568

2843944

1

5012162714528

3673401914

101812036867

5352

524439939985118
16255265

266751033616

6695481030

33567153

121568681231

4876561632
4880571633

411

19351061345742
214154

101446402526

72056792816

149122505469275216
38331601858265

4291377

3168107

213

14151272

83036811

14211143

16549

141012

1315953

12935

11132830318

1221

6596113

612235106

13132

13

521747381412

4948342753

171770224319
171768214018

2131

45393452020

45393452020
13329331213

32641287

193029197

193029197

22212154
18261632068543

32273054

4137571811

111529133

25181666

371930146

382623249

6347532613

6347532613

442417710

442417710

4932371111

4932361111

1

37602852507797
18181278

211582811934

6960501523

21147221613

71778852019

1721

1721

4338481616

4338481616

1529718973740
13311336

46136171312

1252

35142598

27218246

22143

124632

14134533

514249509188
262841096181151

1

1

1

1

1

1

131113

1

113

15

10678236025
1132107

9665215018

1

3

1

1

1

1

1

1

1

1

13101682

21

1

314341423
31202812

1

232510

1

11

2623
31024

131

1

5318101186
19526810002710598216

347921460513060
31265103

4537932511

102156122142
92056121132

1111

19461073197042
20501093258144

1426112

611651612414
223118533710830

8112162185

89991146611

1632813004618

1632813004618

968733497622164
21351073955913

561967443

19104

251831

193910142

26922

295191

281223

46341234457339
47371264607439

181

337

81779167345

81778156335
81778166335

10

111

111

1419892244133

1419892244133

286965072674410830172277751
67603148309458316156689397003148083

5861827553152124

5861827553152124

89831312423

89831312423
1

1
5422741311

2342733

31184798

3560571112
741

21232454

14302958

294352932111176

294352932111176

4365552314420113

4365552314420113

6
54821387560305173

153319914211839
173420814512341

219352

16194221816860

212975123411472

1121
11

5

61

124111511

124111511

8490453229
6777627357278183

7116534659
7116534639

2

547261517
546261415

112

16221721088052

6764472917

197111432932

61149381510

51029161317

111282642624

111282642624

243724827314078

1315951225133

1142

25261576

9171261228237

17826200612765573233106715546
259664135145033139144559024105

2091161883620690

6

14914721486315128

226212364149554199

12106092310

9758852312563

215636778239444285

9261219815758379601449

277155

311693956545583391

242332729207425264

10519171910

249234620182439249

753653115651914871154

898583219948

69598312624421128623

10542232319411922038633

103152395114220149

74491247110150

285281487183536291

201182553151446197

389286538171775357

482675277241

127135

187169274100467133

16312221353324151
13710518544294135

26172893016

8255762416

176151150119343126

185200676190375213

3443163359162

6345539472871075605

167152465163296137

6079513243149207

155187374138310193

283671274524

31211

16523428781642709383
193349936511461

32273141405436

1019177904922

8171641144933

18181531215333

13191911426136

6811248389

71797542917
917106593417

2955

17111491215819

13201851094337

12151911237533

8306372108147

27302261706788

27302261706788

67242102812
16318847892211433329

72862447960165151

72711881954187108

13242191957358

143162932481708711313
3412592567331001443691

3757162973633

4315238423511059

8912221244824

50380802486218143

1454209927335

12

8792271375225

117819211410733

11

26712241078826

5241491970073261096372
744212014712102371710624

59247261191316638

32363473219540

47175109679610536

15281341272116

14232011489713

16562191564139

101810489715

27463003618255

31422492568480

31422492568480

106125985635
13101561376047

2241629

3272323

12894645321036209103375221
18008085541619503186136978

1482661210713723412640

41131

82542817212650

801981926319989159

1111142

338084575238101

32

124438815111036

40204981163317155

36136732210270201

1019115747623

117239440537172614335

26694683711754

141
316031324513474

244619615010052

713113943422

226555746312824643299
45710111445924217549755

81212628554

1020137411026
1122142411067

12541

15028656057361816274
16531658467991998313

15302416318239
15302406317939

13

13352656515942

213133111017947

10211314028730

3191555612213

21504589351533491533419052862944
25723715741808671816722653878463

22366515362182750675

135131

20554813131201383463

1854291200941507396

129032697081842101875431

10834797867768686985407

123129247695870114473141

14113255

9513215991513350306
3715815801427815591063

415043836489108

21352221866969

152629415513142

1

1

111572

8143492945941

17163244236937

1016114971929
10201241032532

410663

1224150792723

10453149523639

912137824826

475563726014199
5264673276154114

5936161315

29462772437882

11

11

366559827616891
325541726214978

484181511
112

474061511

2140642

2140642

16322881744851

4460591412131132

4460591412131132

13611
54564931185230114

5838461214

1

8532801319

293127999216352

104122341418

2719262710

961197210089328127363883
20973805362239660575413182

11334

191421312613228

19161378411028

1122902811523

1318113626641

2

9233516878938

121883806526

1617103914528

135491476321

314

12

73067656711

15462

12232288013840

16345244

1

1517120798435

3472311510

6950223508

4559351099

16181693332020

211419611315739

2672934124189877

7779687415

11353289378335

692806542716

131610016

241522537186010

1

285325456

18211061179926

1012117759437

125122

3161703968429

33153152

10695506411

71030302420

12

1281482256113

92351182318

61382586113

1

14

11

61480437516

42757482721

13812711412420

310144188

513630

14312929011157

40112374447826

1

324118291813

5152303766329

54421

13156303

7671

5975773915

2325182215

21221

3352111074

56

7332847150222

114232

12161238514429

3844269718

8291406410433

8291406410433

1441535

7375123

32357
22247

111

30460191132

393297926639

161024115847120

251319213710460

24825

3

2333367147303044

1

11242181245146

19363203619

534119318

22171034

36283383070317

51076262617

12690173217

1

2218101866125

49192328046

1312

16284

21

1296443

6261319810566

28754

11

5221345712513

2453171086
12409864

12138222

4232874475529

58392512517

477929509

17231471036727

1513742

37140652273320105
36138649273316103

12342

251520011229837

2351126816318

13101035015523

742862116

19917512026220

4127274

152224210016536

2653168211

18252541637353

11

21123692

1244

555018429

36281636717649

41560242818
3948191910

1612598

81272415717

552617468

110242

1

42791331910346997156

36182256358230

632817245

242814168

2310911263

12872162609

23152232613

63427

9697939020

53221284

171291498863539

171291498863539

398938615527631161993
12336527691091373341

14291921194651

133218811

562110125

10940371311

621167426112

1122109731619

22272241915751

363315299

564122405

25232268

161025491012737

42121254

363138236

3317421

236954

281599725523

63920118

592031112343171220

462722208

25171053

32354351013

1330958

91463552213

31232101588945

18181961754059

13742601018

283745425414375

283745425414375

6286684550164157
21221534

414833334210393

9171891152636

1020140783224

307340286028286101353
5744824751670609111280620937

1480177612026938325816338

395761313678448700961513246

16182813039238
13814813121052850183

253147218

111099606219

10530473613

111487481045

33212161654013

22262188312723

446961349

294539178

5456544011

3755393113

1522756022612

11

4350461910

1431
252914810716534

1510694513114

91975593320

3247197314145156
11914810

6343813325

6343813325
6339753224

4611

1632471411

1632471411

42638
132864925973

81331462432
81329451927

2155

51131403233

53113497

53113497

6638462230

6638462230

721129591486317182
2

11
4695756961276139

61391572320

516593641506

1210961162422

3102331462213

5919501113

15362582274665

1135291129

1135291129

27644

16544
27644

111

2416612262530

2416612262530

173
243212839113733

9722835810

9722835810

91245115319

91245115319

612611864514

612611864514

314415344523421
4

252810633310815

252810533210815
232810526910815

263

11

11

616471081266

616471081266

132103683387300197

47271701546860
132103683387300197

421620135

248521

27354

12937261116

1947141

9442645

28562

2616101211

8665334620

1

1111143181412

1021617409
10216174210

21

12208132

128945

162616
132515

311

28820123

12522

129543
1210543

1

1

18937201616

8821

111

11621

136124482650370185
18612

2725

2725

131113412573355174
191937824740

3361382

6101721285

2311632

5722371521

162948108

15344

14831511715

111238402416

2525542014

121714662

21917108

4752232215

121478643210

4426561914

262331232

131

131

136485

18125242

18125242

33481282
15951

21

1111
11

11

11

2422

4585893287233522971873
1224294168111124

81126392220

81126392220

951551207468688757
253517298164186

3338232920

41544494832

1221844889111

111654424033

61312579188222
81316089212250

235102428

121145182128

58517332421

71452393953

81941292223

171762822026

171762822026

8067425365187178
36321931306378

122457

10834391121

358842

513633

418791

16740512424

5239431916

5566764733

20321221775986
8114

91240671624
91238651424

222

61350561938
71350621938

16

4721462220
4724472320

311

3729422122

3729422122

22391071036772
1431

172071655052

51935341419

131385977

131385977

5641347185120132
4641211412

181080543334
15978512834

31235

12660322322
12662322323

21

6853301221

12875232025

4336251817
4335251817

1

71017192316

71017192316

291458
1526991045061

92150433237

6340471316

7539291813
8883328386210180

5137543326
14848643929

97111063

442018106

321665933635
3327811225143

1111629158

5948521931
131461724242

8513202311

4737201012

131842614035
4725433127

911171898

2536271739
274012910876141

11191011

313024815

233857

152119181912

22811

3928141656

21526261219
73482814651

13101011

3934382022

142333

6134

3316

1212

1524110335472

1524110335472
4213

7780145121
7780144121

1

81726169321

47571371456352
2

252582934130

1411
252582934130

6112220115

1

1

181354722625
191356722825

122

223255522022

223255522022

222852491518

43354

111

111

2274308643396154892827325563
28392121584051

3412

3412

21251171296647
1695194910118846931672376

962024188

962024188

9294172
44521522056343

121468672312

202333521719

12622451610

22122615051011225300
106411986991530011201350

87161702543105107

39239925001963407484

36441222841783383459

1684585289

1684585289

271055643227

271055643227

11628662424

11628662424

90103381465305143
300364135515611348516

11

11

13131384

882245158

173554561413

1018110403017

1

3228421212

181150442211

632036245

5926371810

211213

1

3632262524

8850573018

21122053

1213477031213

161040372118

71160414015

5824291815

151555877822

5230431019

105724129

1

8634424525

10831343820

81025283012

114118

31417232211
21317212211

112

71125562614

11649452121

211

721123

1

21418361714

111

644731109

838261113

115165467518243194
122173923834

2313

7728463513

41338351617

661

13132313156

121141391017

31

41628611311

12635272018

134

131550503210

2945581222555

91524273210
91424273110

11

1513494461811

1513494461811

4715101
435618825211882

17759552318

7748403321

6725623416

133149801826

772332297
30462062198965

5963531311
5962531311

1

91948572223

91172772524

662951582629321372613008
55110983306368582506523134

343141229

343141229

414458
18261051206452

61118381510

62719196

12546592528
12546582528

1

31330331619

31330331619

272342571819

272342571819

1754513642

1754513642

92125770515170145
211

40603192445382

526544927011762
516143626311158

1413764

8281371154945
415324925198103

21

34151938

11652

321834611

3141249

71051541519

224262

1111

14123132

1213

1141

1381145
4722171418

13111

3112
13112

1

2384810

3831372416

3831372416

41402291387342

41402291387342

4995235280162122
41152573118

8920201418

3215361711

9633412119

4623281712

121631252014

811

122412

371816178

33130462318
53633532319

25371

2319631147167
115116794334648548

4186

33

11862218

157125364

4146

2941114

1

6179

5319471113
6421471214

11211

24758362916

2

1

1

260104846

12392410

768520513020185
758520513020085

11

92614

27154299

61066

1547

1

10247

211

249

118169

413

144

114288

342125514
322125514

2

8128114609201299718970
12561712294

1271204731
1165144327

313

1

15331

472145462
1414225116136

15464152

698

1242

93812

275111318
275111319

1

546474643621651465
541449540720561371

52512910994

722219389150375087225
3177381960230332842

13872626

633135818210641046

214198

648

8178

232

2131317

213520127131

235102029

22

1221

4

14975454

3843728

2011014
1

141711

623

264915

110168

3132719

38143933

32

12932014

1261610

2031522

13562423

12941312

416822114150

13151818

4153940

9152

472

20281535010462

1432019

111

173109

1

1192128

3583029

174711

11088

11931916

142129

3111928

23742130

138179

16174

134155

4245

6148

3131314

18293

1576

31

420955131721753

122
1041113

94911

433

24724136

1221211

414675129614582

11185

1

3572723

291126980

58177248

14187

33783630

2483136256

311

122452077

83155745

9278

13823720

157114944

111138

5187

3372731

13882539

1345

21350496441396

2

831215

135712

7536402321

7532402219
7536402321

412

148823171014214113

148823171014214113

23255
148823171014214113

1623801233528

592913964412849

7130942214435
7130952224636

1121

4683696

4683696

4683696

4683696
4673595

111

856533938625179

856533938625179

1111761252718
856533938625179

17631291215

1212123562517

1519531014713

9122322179

21533531237

303483903925

303483903925

393229710
303483903925

3312732

25147

1591784

236912

26512

413542

112111563

2433

141
547507139822501034822

52949312752108848782
7843702019

572537139
1291543

1

3

44162246

11

2101969117
351

231221

2361

123231

1681454

11
59137651412

1171452

28212945
28213457

512

1081723
21081733

21

4674299541630741693
4239150377149132

684111

11
1111

11

151

151

22

131544

7521
4511

31

11

223
1

213

14217933

113
1114

11

1

1371654
1251554

121

21522114

7

1

2322

114

123

13101446
24142159

1311

1442

231325

21821
21921

1

111

111

1615

1127

1

241428124

1

21121671

14146

29854

221

2924241214

1681716

261845

134181

621150202

431

122527101
1123955186

12

10132865

171566

42538155

36102673

4916133

11

10825181514
10825181414

1

11

131319152
231520153

1211

22

212104343

27645832340
421

27645792139

837624
316235273298210345

308232266292208341

5

25530383
25730413

23

412

272
16

12

1

11

6851582917

2236

1111
111

1

169812

16121

1

1181024

131
1132

11

13816165

161723

61851
6185

1

1

4538892711

136253
336253

2

1221

26111542118
3

228111964
228112264

2

1

4341754

2123686

1

2113686

281955710
1332214

15

2101354

361512
36142

11

24143244

24143244

11737721914
1531

112

74621

4632601212
2143566

25282566

181412213818639

181412213818639

6358231479

8436801914

110873

3718271313

1120991277329401415961

1120991277329401415961
3435941124230

24172746126

24172746126

52451041193848
318322959933425343

1

222080792131

121866704332
131866724432

121

202591603715

11

261772623827
261671623826

111

1593830911

1444534106

39381021064737

39183441728

1

12

303172773824

31048401510

2032109973837

173162794625

1

72334323312

1

13

154089683337

154089683337

161656714527

161553704327
161656714527

1312

1
353853792531

12102644196

23282734625

61151344

61151344

881828178

881828178

1265927328511383
50437211021082519332

26561

1

131

171416154

2810252467

63138116

11129

371

382236601116

1441

48191877
46161566

23311

21135

2418793

223631241617

4144320179

4781442

13

6413

2171133

317741

1982121106

3571093

64142372

11

15646

141

337465

31241

493292956

211152348

1521
115121

11

101014371917

26171741

237201076

26713118

25

451342

21917261311

26101242

49251493

44331968

55182275

52142893

76271485

4111995

3611761

32521

131

1

135132081

1

1

1

336723

4711263

2

1010182084

1042327108

12

261112923

113212

22101685

511218185

23101173

272021118

56264162

4212152

7731641

18132528247

18132428247
18132528247

1

8112725149
48531111307039

101515341913

252665643316

512641
514741

21

1820491012417

1820491012417

142245751741

142245751741

4621871153421
125253945

673338133

1142729179

175294

3113974
1613435912418

36172152

2

3261025

111211

73471096

111

111

11

11

11

11

1

1

50425933172061185991016706

50425933172061185991016706
85874462668682

46295578161591084187056352
121811653672280819221230

11

1222

231526

92323197619

2

2342

8516141

10414

510356

1

5133325103

14

8619966

21358291123

4

2295123

15
473235251728

463229251728

1

213132

1329834

123031

41

1122

39281661

271410126

1

11

87231263

1111341

2

38982

1

2257142

15

3241

22

115312

317624
36621

113

14132

1525721

26725451713

7519676

2111

13572

331

115733

251224

13111514105

43171

1162510

12426

11

4

3213743

72111177

1142822

11410844

211

231

145233

111313

772010710

11932471412
11934481412

21

261727191430

1

10172313174

1271363

111

55141257
55131257

1

6910862

9

562925214

25

246742

1

26111561

39201898

124427251537

341241462014
341241441814

22

1

6192035156

44

1

14426

18721

74141076

2

798212163

2411891

311422

241414164

121

11121

282116101425

1

310511512656116

1253844816

41

5111

1019102036

213632

23811103

13312

852238938

653646135

1111

12641

3

36111861

131

41191174
3981152

12122

124332

63651028

641416157

29252910148

1

11
305668674629

31533

31533

2611

51

1

5

410131568

41081468

5

1

4512367

4510366

21

1

1

1

1

1

711

71

1

72716362710

72716362710
72715362610

11

11

3515741

3515741

138313

91

57119111

12

15211

410412572

1

413262

124

2410142

2410224

128815

111

2101366

1052448617

12

377932

125164132

1362223136

225841

1710664

4331074

131031

5118631

3943118694315

333422

27279042277
3640115654113

341013112

69151024

1

381014810

311

1

12371

11171

2218392530840

2311
2211

1

134432

4421111

1112

16581035

134731

25821987

6751531

2412181287

17172396

16288137

81144124

1

51214351012

5

13313

2230691103937

345421

1241

23161161

3526321612

11

463020610

161125142814

391916102015

213131

3221926

393353

181741111614

517541

73191095

22171

3181732

131322

11251

32151655

53111

328321

1221142

21

1123

114122

2132

122

1

21

451011612

324201173

7551043

111

28541

127775

3413395

162430201212

12222

211

3322
3221

11

1214
5411441

421041

31

12232

211

273341

111

25122

74192476
86192886

1241

11

58747

1

1

144222

1

3115224
3115246

22

1

1

636234

6791477

37411

121914

2

2132010198

271629103

411

522738352332

24111329

1

1536

7268123

12

12

1

11

1

4111232

137813

5732361

331011812

11

111112

25152794

8249114

83189108

211

43678

1111

11416131810

12583

21119

1032236168

11

311

2121

13

12312

2418753
2315342

13411

1331

445974

12

1

84162043

11591

122312

21861

321

1310151

1

1829552576857

5241136628339

49411

231722

52141544

1

1110239

5211672

322764624938

116721

658782

338

8215842

7917261610

171816162914

1

613

285837

8121730104

161026144014169131664

2354151

361023

1232

582610915

5121361953

187191377
176181066

111311

44161752

1

119863

1

712182041

4105571

1

142636252716

215

1121

264547594423

264547594423

53211366

61141045

1121

111

61

1

12123

2512

1281312135

214317

711122482

722818

338944

118391

12222

3111518195
14111183

2747112

144179105

36131827610

21512

7131429136
712920123

15913

11

52131082

325745

311392

41

2221

2210241

2641211

1

211031171213
261138292014

51331
51431

1

1685

13101

26151178
26161578

14

4

23112

2613753

13

131121
141361

124

211314

1

72103414
7193314

111

23157141

2413853

216234

11051

1

181528211116

1258

2

1312

15111642
14111532

111

1

148161663

11

1410442

1688

16351464

11572

121

7435

2314

13

572

23233353272919

5173449

10271912

9492455

12

9633361114

123436

1222

67512417

1

31

1210711

272

411

203360343610
44874

162952273210

11124

3

73543

5111

61139115
61159115

2

2814121015

1034111

333834

641

1

217

2997814

26121033

21

171049211823

441712141

11512

1231142

351817811

52321964
52251613

7351

4578166

1

1744918710

798917

1311

3821914103

11

232233

15333

21
2

1

234542

8451782

312329

621672

621

1

21

1

23818682
25918692

211

233644

67111175

1

11312

36722

22210104

171111106
171315

10891

6311236

782125108

14181

63415237

127221165

11

1

4672453

241931222222

211731142022

212

1162

122112

44352146

1

1199853

6375124

21

111

2323515445431

925114219

11511
1411

11

2

21

1211192467

126259310

132122

81112092

1253

3315

617843

1

439842

213

2241

2475414

1415622

6141321

22142834129

22

42143095

21832

235514

41492

2967

1111

313664743534
313662733334

212

3461222

744045623

5739231010

54421

446572

11

33

1

11

121086452718

12

11121

247954

4145113

11

14112

1

38623

6412776

221523

34824

5162329128

3815292949

5111114165

11

37534

1

29332

365

1237

11

178584

1241

13661

4881055

6553038

1

172193485230

11313962

11

1137815

471612182

5781355

28181458

33131053

1

437654

1

3569184

833425

144831

115653

11

2213182083

2129742

653

514103279

710231411319

227552

14831

731020149

41111

33810142

12213

10232215513

10352946

1931316129
193101497

3232

361517168

212381

158926

158926

257874

736512

6818221011

1858211

2311

33101364

27131711435

35303072

126121252

33322

74041122

43631

33

1114613121

122

122

71327182519
5919171111

16186

14262

412763
413763

1

632483

2781011

2114641

1

271021

211414117

76727

138152

131

1111

33101369

97924

1

1

2

2

574232

77131985

44131642

25101595

223

6418122

2231

4

3101044

1

55134139

53923

371

1311

157142

2518943

121

7

13311123

42210225

1

1

2013221575

1

22

14121

1912

131332202020

441517411

7361485

1611

1416595

84620181820

2671164

1

2

1

6533261510

7349124

323422
223322

11

29557

2151134

361418125

923954

85532

3421

1

1

314

259334

3621

2261

1811986

1

1

11

79172784

1361023

338102

7212

1

21

1612201253

421619104

3561095

251015327

21121

325343

423611

881492

1410856

3

2191

92151096

231

23111535

2742617346

18145216

2571041

30122718117
40143024159

1023642

221818

113324

86143

6921242310
6921262311

1

2

12

11

657686

386516

1

1

1

1410121314

16

1

102710

6

624776

1312666

521015514
82953483522

3274333308

65131419177

11

1

33163579

111

11

144524

1

781881611

13

241652

1

1122

311

24312

1

1

8111081010

611152016

1214553

41621
1

472

81

8947371611

7439112

212221262123

11

1022

21512523

12

312

1

22

34422445

111

816241388
92438171912

18144114

1

151031231011

4

16721

351

301218975

111

2212101010

26

62227321514
61925281314

3242

1

24131131

34823

929616414

638622

132615123

281323281914
291326283514

1

15

31
2

11

112225192025

36410261409

1

2

95214347

113

35231984

2

3316854

11

312125342072

144591578

1111

1

1

1328815

11

1211

12311

17712597

6211277

2312334

4104721

11

2121

142131725

76101156

2243636
285420814

6111458

6111458

2941

232944

3210756

3610722

3610722

1121

15173255359
178148336426174162

252137542446

21122

59123125
59133338

1213

2210

7152547513

8724391111

721719119

236312

1492219131

47182369

1882214147

16612246

21

14531

891813159

138332788

241521511216
36153285

219619411

36331

10621744

6524421314
148114256318129110

321739521421

491052562510

9322311112

1121182079

132034471911

272213139

1826231210

191171688

612121876

26987

26987

593
878317521413996

302238343227

2211
302238343227

88144167
111

21

11

454143

11111

421

2311

2

1

11

21
3163102

3142102

616423
21111

11
1

1

1

25222

321621

321621

2132
546713

211

222

114

11

123
121442

12

1112

11235
1

122

5
15

1

72

72

11

11

1142

112

13

11212

11212

1121

11

12

12

111192
576113217110469

131

131

4233991028043
1133132

116232
1161

12

111

636372

1111

12

225351

2

2

2

223733
312584636736

1

5532
5512

2

211
221

1

1

2111

51

412411

511

23223

111

141953294622
311

141950284621

14452

32

32

2112

1112

1

11

1331

1331
133

1

1
142731472423

113222
2711555

231

11

122

1

21

1

111

111

31111

31111

664854
121719411717

1

1

141

1221

14

1

211542

12

1

234

1211

14232

1

2111

412
411

1

11

11

111311

351253441518

351253441518

351253441518

351253441518

351253441518

21122118105

21122118105
1

424141

424141
1

212

212

1
21241

2

11

1

22

1710161764

131

131

131
13

1

2591023

2
11341

12

12

12

1

1

1

1111
146613

12

1543
442

111

1

1457431
2

12
6221

6

11

1

1

1

221

221

2111

2111

11
22411

1

1

1

212

1036896

1036896

1036896

1036896

515736

515736

52116

52116

26630311216

26630311216

26630311216

26630311216
111

12
86424

5311

33212

1
121122077

12191837

323

111
428423

111213

362

223212
1

113211

11

96990621301824457481
1651832

1

1

1

1

1153

1

1

21

11

1

12

1

1

11125

111

11

1

2

11

1

113

1

223

11

1

7

115

1

11

12

11

1

11

11

1

1

728206

1

1

2

1

1

1

1

1

1

1

1

2

1

1

1

1

138752

22

131

1121

3

11

1

1

12

1

1

2

1

1

1

1

1

8214351882467

1

2

321

1

1

222491198

1

1

1

211

211

1

2

11

1

77883618501507357357

1

3

1

1

1

1

1

1

12

1

1

1

1

1

1

1

13

1

1

5162

1

1

1

111

1

5

1

413

1

2223

2422

14313233

1

2111

1

2

2

351

2

1

1

1

1

1

1

2

37

1

1

1

11

1

1

31

1

1

1

1

1

1

1

1

18341

1

147135166160115175

1212
147135166160115175

14231

14231

14231
2

21

1221

286858554054

286858554054
111

211

211

211

211

122
3213

1

1

1

1

1

226355543554
1879823

1

2

1

25643442749

1

1

1121

311

10050786451102

1
10050786451102

42272
1221

1

3131

1

1

1

11111
964878624499

954678614398

1

1
171526381918

59320138

213111
14314113

2

111

1

11

1

2
45625

14315

11

1

21

123

123

123

1062115610
26

72
210231

1111

122

235316
1049739

112

411

1

12

321

11

100561341058569

100561341058569
11221

3
99541321028267

1

1

7742109396451

12101

12101

97161065
7238108315148

1

1

1

364610
24136

12234

1

1

391868122719
36132392110

1

445369
1445369

1

1

1

1

13

1

114125

1112
112

1

21

1411

1784277

12
241632

1212

111

1121

1

2

2

1
181223631816

86198107
613432

211

2

3

1112

12

211

1

1

1
136111

135111

21

8
21951

11

12

13

1

11

11

221

221

7314627

7314627
7314527

1

21111

111

21

113
3275

2145

2145

2145

2145

11
2145

1144

214313138

214313138

214313138

214313138
11

811424
112

2

1121

41211

1111

1111

12792
251

311

664

1

11211

11211

11746455923911266686
692007307347607576518830265091

13214
1015574419262

371630182826

371630182826

371630182826

1

371629182826
111

371629172725

1
633642226036

633542226036

633542226036

633542226036

987187667979

987187667979
1

1

1

987187667878

21
987187667878

987187667677

506284290245283289
1143274

288169208175167185
1

11

11

1

1

1

1

131821341099588

131821341099588
22231

125801331068887

2

123801331068887

1
4114

2

2113

1568774667196

211115
1568774667196

1045843384259

1045843384259

2121

2121

1

1

482829252830

482829252830

482829252830

1

1

1

1

1

2

1

1

1

1

1

1

1

1

3132

12

12

12

12

1

1

1

1

311

31

31

31

1

1

1

202111796710598
373123

2111

1

1

1

1

1

1

111

1

1

1

11

1

1

1

1

1

1

943242

211

211

1

1

21

21

1112

1111
1

111

11

1

1

1

1

1
11

1

1

1

1

1

1

1

52212

1

1

1

52211
1

111

111

111

111

1

1

21

21

41111

41111

41111

11

11

3111

211

11

1839873629791
2111

1111
21121

1

1

1

11

11

11

1

1

1

1

1598264518779
12

1

1

1

1

1

1

1
1568263498677

1568263498676

1568263498676

21

21

21

1

1

1

21

21

21

21

31

31

31

31

1

1

1

1

11

11

11

21233

21233

1221

1221

1112

1

1

11

1

15105756
411

1

1

1

631211

11

11

42121

42121

1

1

1

423335

11

11

11

1121

111

1

1

1

1

1

1

1

1

11

1

1

1

1

1

1

1

1

1

1

114

114

112

2

1

11

11

2
13111

1

1

1

1

1

11

1

1

11

11

11

11

5522211918327
567146597740506515946624156803

513
9957076034579982612

9827025984569981609

513331244334
9827025984569981609

1

1

4482942561759563260

4342862441739558253
179142911

4172772301719549242

14812257

12

11

1

480374308254372310
12

11

111

1

11

1

478372307253369307

11

11

1

1

1
113312

1

112212

842113

842112

11

11

71212

71212

21

21

1

1

1

556646524839882511185607656164
959868568568696888

111

111

111
1

11

641142
384202215179329405

1
18794824912592

639444

639444

639444

639444

864744224847

864744224847

641138

641138

641138

641138

352366
1522751312

11
1

1

1552

57423

62111
64111

2

121

121

371085237

371085237

36812103413

36812103413

36812103413

12651132

12651132

12651132

4248
12651132

51112

3314

1164354

332112
1154354

5122

5122

32122

32122

32122

1

1

1

1
16892123125182305

111061079

321231
111061079

1
2142

2132

114

114

3121
1

3111

541423

1
98578494137256

351734169320

351734169320

27746114

27746114

3633467232232

3129447026229

3129447026229

542263

615537

615537

615537

615537

532328163533
1

462127143032
111

2491971321
1842142

65176919

10224111

10224111

1196359
11963510

1

621251

621251

621251

614338
740470465413511536

10177664462105

111
10177664462105

10077664361105

10077664361105

10077664361105

10077664361105

390259252226276275
1054698

2

2

2

2

15597827782100

11

11

11

15596827781100

251414121215
15596827781100

814538344452
373129232134

39136111715

51363

493730312533

11980717710076

11980717710076

11980717710076

11980717710076

11980717710076

1057594668591

1

1

1

1057594668491

1057594668390

1057594668390

11

1

1

11

11

11

11

243133143140170148

243133143140170148

243133143140170148
966557424650

1116

116

1

352227263423
11

1

352127243423

401222312535

2

401222312533

355754
713436414840

281815162916

401116181420

1

1

505156179436924484455137252345
405386266291311413

625946385367
380543992026255386393988031340

366663923025710379703833529353
889348636463

138610239027681071993

138610239027681071993

138610239027681071993
916148315465

683532485346
2221

1212
421918404036

211412212514
1121

135310116

77911127

1

2054181520
114291115

912945

2614126139

2614126139

2614126139

2614126139

2012941324
193169139114168170

22177141618

22177141618

22177141618

7118676
1

1

798676

798676

14861198

14861198

14861198

2282191310

2282191310

2282191310

3142
606952387249

78157128
424739305434

1

9764114
121

8554114

431242
434243

1

1

2

442252

442252

1

353425

121246188

121246188

385414

385414

1521981813

1521981813
1

1421981813

1

1

1

567588

1
567588

567578

794337

794337

794337

14121371016

14121371016

14121371016

14121371016

8956611

8955611

8955611

1

1387111113
421564

1
624234

624233
4

224233

342425

213224
1497861477968

1

1

373613182017
211144

1
19175797

18175797

1

16187976

22

11

11

11

14
522428123339

31161661012
391720101615

814463

126821324

1

1112144107
56151714248

41341

4126131
411471

125

1

423316271731

11

1

1

423216271730

423216271730

12
855534466566

462314183528

462314183528

11

11

382920273037

382920273037

1

1

10411817811514975

10411817811514975

10411817811514975

10411817811514975

22113
1651009186103115

31343
612034273736

21712838
29718121516

864128

1

291212112217

846648545569
16111014915

1
463927253737

373321152828

9651099

9651099

672123

1

1063652

1063652

6268212

11

11

181184107

161184107

161184107

2

483635182631

483635182631
91074106

1145267

1145267

41311

322

535313
531313

4

1616157814

15211182117

15211182117

15211182117

15211182117

493524333239

21

1

2

321
1

1

11

2

14181051316

14181051316

14181051316

321514261822

321514261822

321514261822

321514261822

281024102317

281024102317

281024102317

281024102317

242916192729
349272209185281253

272511141510
311

1
131768113

131668113

1175646

1175646

12984114
27271882716

11142

1

21

21

11

11

11

11

91571119
111673129

12

1

1

1

117621410

117621410
221141

9551109

2512791013
784041474946

125141

125141

223222

223222

1988367

23466

23466

1223

215434
2

215432

152121

8231851

8231851

1853798

111111549
151519101318

21
1

2

2

1

426588

192114142010
321

552142
552253

11

1

444472

444472

7108884

172016141212

172016141212

172016141212

221323

221323

261916132823

77651113

77651113

19121081710

19121081710

64435
191715191520

631495
631496

1

318733

318733

492536

522422131731
1221

308116412

308116412

411

7534712

7534712

1095266

254277

11

244276

30211073518

30211073518

30211073518

351923811424760371393720028297
1391357112210392

1661882242171187172

452242
1661882242171187172

566557367749

566557367749

29524
566557367749

545652367545

1061172183133104121

1061172183133104121
312915171919

343322162217

41552146996285

11

2

2

2

1

163192116136113138

163192116136113138

163191116136113138
304428313026

555940422048

555940422048
1

555940421948

313115241623

313115241623

313115241623

475733394741

1

475733384741
1

475633384741

1

1

1

347243759922331367103679727895
14611629977133110961297

319491218286232242

2
319491218286232242

14521
6712854566642

4811339374736

4811339374736
19234

2104332
1433913158

21111

1

2111

11

11

113331

113111

22

21

21

2211

22

11

12332
443443

111

211

112

12

12

1112

1112

173224

511

1

31

1

1

123213

123213

322

322

322

315823142815

13
315823142815

922

1212

304719122013
1

304719121913

11

2

1

132323

12

12

12323

12323

157815104
1

433661

121531

121531

121531

31213

31213

31213

1045943

141

141

141

945542

945542
832232

2

1121

11

342471

342471

342471

342471

636336

11

1

1

1

1

1

1

1

1

1

1

1

1

1

535226

1

1

1

1

1

1

425225
1

32412

32412

32412

32412

1212

1212

1212

1

1

1

1

1

246358158227163194

246358158227163194

246358158227163194

246358158227163194

246358158227162194

1

211
199161710710113109122003512961

41331022

41331022

4123922

4123922

1

4123822

11

11

11

410516257347326397
199101709410109109022003212959

11

1

1

1128041636953
129561005347305118119626990

13222410811892177
337267843471287527584887

1
455533297334382489

2
62147

21

22

21125

314392209223281341
455526295333378482

9490527359101

474434373840

131748411
6401072493582532859

1

1

337544265311295440

337544265311295440
1232

337543265309292438

290511223263233408

290511223263233408

290511223263233408

214549542573184117523362
325618326292270455

203319172145118200
134517131218

7212470534269

118150857964113

118150857964113

430750473345348514
243437282198164297

355857454456

10419810677104122
152255134102140161

4536

496157

6148468

1625891213

18468711

246390224225215269
629355465770

1

1

6711974696174

1171789411097124

1

1

62231367

62231367

1176526

1176526

159309146142113177

159309146142113177

159309146142113177

6210570585167

1

6210570575167

207384183175159288

12
207384183175159288

206384181175159288

49520509704414701379

49520489704414701379

2

1

1

1

111
907328921097193788041828

907228911097193588041826

907228911097193588041826
21249253787161426609

271216114180139143
2421

271214114176137142

2241458514312690

2241458514312690

1

321212112190245192

321212112190245192

27196101903092044374
613213934087056868792

34137832183964824418

111

111

1

11

399297121243331222

399297121243331222

11

11

398296121243331222

1

397296121243331222

21046149
1180143772097211001082

192205102168127168

192205102168127168

192205102168127168

541559246384284368

111

1

1

11

1

1

540558245384284368
9110039774856

14112049836990

14112049836990

14112049836990

22123394162118152
565134402735

11710526645665

487734583552

8710563624970

1

8710563614970

8710563614970

156217142136151183

153215141135145179

153215141135145179

153215141135145179

321164

321164
1

11

211153

1

288446226278524354

256415204254217333
288446226278524353

3231222430720

1

1

1

1

1

536350884402446566444489
8110864795083

128212104143111144

128212104143111144

128212104143111144

128212104143111144

128212104143111144

318382211274373258

555733473335
318382211274373258

1001409410180102

1001409410180102

1001409410180102

16318584126260121

16318584126260121

372285271251164286
483643864023396961104004

1

1

1

1

339434763151284654893145
269216177179129201

190153991753337137

190153991753337137

3

190153991753334137

1

1

190153991753333137
11

189153991753333136

189153991753333136

152511681302102610921387
203161193136135196

551356532375420649
1312

3

3

544353531373417648

544353531373417648

111

211

11799881057387
266239195198252184

439866

439866

1
121884106

121884105

8108826

312923253618
474134354732

973416

7586108

464320229526

464320229526

322528161921

322528161921

7144286

7144286

13911412410273125

1

13911312410273125

653553393730
264187178143143166

111
965755365047

955755355046

1039570685689
1

1039570685589
1

1039569685589

1

1

21222

1

212

2

1

939574685961

939574685961
2

939374685961

1
350214280255215255

350213279255215255

350213279255215255
2610411

348207269251214254

1

1

72014571064985564944
581161041015193

1

298482370349194294

298482370349194294

1113

1

113

363851580529311552

22

1

361850580529311549

1

78655
1

77655

340268229225152221
1

339268229225152221

339268229225152221

1

1

1

5132

5132

1

4132

1

1

1

3232
838438443685328410

1

1

1

413258249220196228

413258249220196228
1

413258248220196228

422177191463132181

111
422177191463132181

420175191461132177

2113

1

1

1

227184158184127163

227184158184127163

227184158184127163

155202113184138161
789012251703619053110599246

4358652140401464337364925
154215150257140161

586888873713513692

586888873713513692
183227132328

122

2
122

12

167314398235183233

167314398235183233
1111

22111

165313395233182231
287343

163305388230178228

336454603773

336454603773

1

326454603773

326454603773

5611368744754

5611368744754

5611368744754

8412287906178

8412287906178

8412287906178

364245613733
1

354245593733

354245593733

11

11

191201194178125191
14202314711

12913011612187138
355349583743

13117111713

11231316924

704347362358

1

12211
424652403041

1

404450393040

653311

653311

263615191720
1053135184710559571255

149200143144167208

149200143144167208
13141081411

14

14

205140333453

205140333453

205140333453

1

7287617483108
303218212630

11119111610
785496

12111

314663

533733

533733

264131353864

264131353864

1

434331293535
1

1

414331293535

414331293535

111

11

1

1419717188
301406259363332335

244330493336

244330493336

244330493336

547634467366

1
547634467366

547534467366
32125711

515432416655

345449
10216598175137138

1

1

193618362527

11
193618362527

193518352527

284523524530

284523524529

284523524529

1

528052836272
12

2112

244731383831

244731383831

263221342239
1

11
263121342239

253121332239

292014151320
10710390767187

534148423937
151315151615

11

229219117
2392114127

151

1

693437
141812131114

889777

121

254228191930

1

254128191930

254128191930

115136112102119134

115136112102119134
171914111020

522135
495445333353

21

445043322948
424640302444

111

111212

12231

233829364434

233829364434

233829364434

262524223227

262524223227

262524223227

1725551836
348397219272238419

10198171118

10198171118
3105745

562838

12112

111133

713410612

713410612
2211

471725
115

13

231225

341336

341336

422345

422345

422345

160176112114106180

160176112114106180
303215231927

2

646952445290

667345473563

1501628812393168

1501628812393168

1501628812393168
134126669874141

163622251927

548151704066

548151704066

548151704066

548151704066

548151704066

609548854473

609548854473

609548854473

609548854473

609548854473

1121

1121

1121

1121

1121

1

1

294233902641
2565406521691261621252817

181148110159131133
11

836147476965
86533

1

1

604328274838
182116141822

76393

2

3213971811

3213971811

313332

141214201824

141214201824

9887631126167
571110110

334026673225

334026673225

604026342832

604026342832

1

1

1922337916251190614882184
1411389029088114

6134999
269330229326290281

454127592544

454127592544

255522
981298011213199

283627392039

221919267318

342518141623

124411282017
3416101

94010221016

7105576
526564715359

141819271322

1

142216171416
1

132216171416

171524221914

334023423632

334023423632

354231333638
1

12131

11

1

11
333929293538

323929283538

160269184210145186
265321231725

293130313430
1

11

293029303430
1099111211

151712151811

448448

132424181919

132424181919

132424181919

222

222

1

342125
9016110913674110

182615341624
337444522339

15482918715

234535302428
203424232124

31111734

71012558

71012558

242816482030

202194164249143182
211

616154734257
187169143228127156

81312

1

20217381313

20217381313

241716321319

161318231618
1

161318231518

12

117718116
7351022

12

342874

1

1

513136332331

1

32313

183554

142118201524

142018201524

1

1412

1312

1

616647744552

303417342932
616647744552

21251930918
1614817512

321511

252613

48222

107111072

107111072

121214113513
7352001687104834551062

1

1

1

1

676347594042

676347594042

1

1

11181

1118

1

6551923625103014071006
103314105159968151

1

1

1

84116624828782

84116624828782
4566352655450

6752462

411224

673162
14

315

54272

321412

321412

108559109
3533375

4221411

1512

3711

53131

3341412

211921

211921

1

14163

14163

111263

111263

13122

13122

4841524

4841524

4

1
4858381292442

111
4755361212341

4754361202340

4754361202340

13281

31457814
2571173279731091555

13235107113

13235107113

2411136269712589538

4261522

4261522

4261522

4

7999522656179
192314761223

12

1614927810

2711425
41423557

2712132

1

511544810

1

201615441117
211815461318

12221

311
1318737149

1017737139

1

80161834907495
3248331391832

21012
14

1612

133671
8194571310

1

41413867

1

3192

12

2234211171115

74013821923

12

2

111611851013
71611791013

46

1

1

111

111

21141

21121

21121

2

2

647654686973
3212

364029412745

21112
364029412745

343928402743

1

1

1
112

1

1

1

233425263926

233425263926

288304169202252234
19111171114

102121708811194
253221243440

212510171211

212510171211

8442115

8442115

233111

233111

332342

332342

612812124

612812124

262916191622

262916191622

5766104

65385
664115

113

624323302339

624323302339
11

624323302238

2151

2151

444214273027

415
444214273027

404114272527

284826243729

284826243729

1

1

283223203129

283223203129

283223203129

32213
542541

11

112

11

1

1

433496401461480459
336336

370405318387415377

370405318387415377
951159488107104

212318261922

214415281519
547745604942

121717261414

2116136209

1

101012241119

101012241119

18118161420

232722231619

6831379

11162217811

171211172018

1

284774

284774

284220218937
221515176618

627542319

11
391824241419

391724241418

778151110

131

393027334340

393027334340

1
608877716276

2

2

608875716275
315628402652

122239161911

975976

975976

521573
7236106

22133

11

130253137223143178
337755282883422671854160

191229119195980156

191229119195980156
311512

599543836553

315523493934
599543836553

1120101188

1120101188

211
172010231811

15209231711

1

1

14141314712
12913375106914101

1

8783465888555

273616342234

315614275451311432
241341652138303355443121

108219132166112254

108219132166112254

108219132166112254

68512726768442065924
1321716514

11
1

1

151291145199141208
1733361762321596231

410101096

221

71611162911

11178714175

709346765972
499915492595464679

217404234236210279

11
212418212283195328

211418212283194328

1

1

841267012078114
444547328483393420

136189127147136112

1101561061328794
136189127147136112

52332

128104307

923811169

222231130216179194
102713221116

285763
9210357896471

909552825868

21

113
12010160105102106

11910159105102103

211
1

1

2

508838563159
324535268399271420

11

11

741598210977119

741598210977119

200287148233163242
445745

14217690150115145

2111
14217690150115145

14017590149115144

5410753764492

5379784596892392671
3123885

3154239

3154239

2023601632942050245

2023601632942050245
9817374115917114

100182861741119125

4535146

1112
111

2

328590288385331410
335935483640

163292151206157216
1

163291151206157216

132239102131138154

132239102131138154

1

1

25224
643879489775516704

476600343554360476
1

476600342554360476
259357217323196281

1372629

732634

12101061210

91261139

9139487

462432

271917181827

31511

291912482019

2

554655

1222

1326

203010251320

988876

6113558

1412

202412251522

31271054

25187151718
24156141615

131113

1026717178

4115768

165274146219154224

165274146219154224

165274146219154224

165274146219154224

211

21

11

11

1

1

1

14016310012480106

14016310012480106

14016310012480106

14016310012480106

14016310012480106

14016310012480106

679660645847
499859583887500442954043

28321

28321

28321

28321

28321

273230652074264326392384
617743583954

345222332553
590663455560971504

293810302028
1081228210751795

141915201720

141915201720

141915201720

13

13

352425231719
4938403746836

1

2

2

11112321

11112321

212

1

1222

1222

44431457

44431457

12311
12323

12

2442511

2442511

2134155
134143

1

2

2

111

111

15251719911

11

1
15241619911

15241618911

303218252722

303218252722

303218252722

303218252722

9597929212653

1

1

294119322414
675858594929

15423
331435262111

2773319185

11112

111

4111

534144
21123

1

312121

1

283933337724
17151611149

312713316
211712286

1111

2

812109329

812109329

267320208253237236
445332274829

11161216108

11161216108

11161216108

464034404027
278148166

532311

532311

571522
41412

2

11

411

92217241119

416145433756

416145433756

416145433756

473923453930

473923453930
221913271916

11

1615615199

62423

2312

294324323740

294324323740

294324323740

2122
496838502646

1317510614

1398101111
202217161314

7139623

1019922613
14281622716

49713

564033503945

564033503945

1

564033503845

564033503845

1041307810366108
208123251576202516291826

353322
280349188261193276

1

1

1614713818
217258144206138219

1

1

1013914818

781617

23
494432342747

474432312747

103717151620
366726393634

391674

23218181310

356026451938

635143553956

1

608641525255

11
608641525255

598641525254

159217351231155812981360
778853775175

9871054790930784841
308335261317252277

81718141110

694644
232212

462432

575820433943

575820433943

1112
11824153

7622122

31111

3

294032252325

294032252325

1

145811711

1

204129271635
11

204128271634

642525

642525

1

1

25266920812
373578261920

10996118

2

22239261725
1

22238261725

56113

27278172517

2111215138

1

122322
393917292531

383715262329

42121
423815252227

383615242026

1721917812

1721917812

953744

325331
8387164

5134133

11111

11111

542534

542534

14102081710

222313141016

282427191223
111

282326181223

3882320

8510866

2

274463
494773

2211

1

1

523646

412184

412184

364726482231

364726482231

13191213511

13191213511

253426241832

253426241832

455130283829
11

455030283729

474141474039

1711613119

1711613119

1618510168

1618510168

8910211213
91110231316

11

1

11

1111

1611814914
1

1610814914

12315093153139107
485542367510431414

212323171216

491363

182920231630
21

182920211629

1085863

187791418
14667610

125

311263

1

111

211611231413

311

1

3

1

16231415412

1012911910

213424211517

1

247332
12129778

51112

571344

571344

1
1691516109

169151699

211

819723221

551754

11

8811787

413412442647

91351689

342833313432

262618192118

92361248

96710106

232319292223

7101113

1012134146

12114
1

1

1

1113

1

1

424920382830

424920382830

424920382830

1

1

1
666930763952

2

1
2

1

666630743852

666630743852

666630743852

3

2

1

394249273330

394249273330

394249273330

224116251722
219727891750229515971612

14116014713586114

14116014713586114

14116014713586114

14116014713586114

557640635645
191724261475199014061392

333421235360221248
171921891330175812261240

630853497638449457
7410054634845

658258625135

2
6610357815351

6610357795351

7812981897162

103125821197091

103125821197091

616732573843

11213865906576

7110968775352

2

1077772
13320712216586105

6610162814046

6610162814046

579953773957

579953773957

17915810612912993
474838457429

454524

263921242017

364221332018
894824452431

536312413

31110846

31110846

1079256

2
173211165201156134

13115414016313197

415725362537

1

346828412630
180243151186131129

4251
12202122148

12161917138

282816232014

282816232014

434837363433

435433412428
364028371923

7145455

202516231316

202516231316

919654795474

919654795474

919654795474

1

1

1

1

1

142161105169123107

142161105169123107

142161105169123107

142161105169123107

1171621121458884

1171621121458884

1171621121458684

1171621121458684
311

1171591121448584

2

2

11

1

1

1

1

132563149963114921919
43678

21411997101146151

21411997101146151

21411997101146151

21411997101146151

715253574347

715253574347

1
715253574347

715252574347
161219161518

252521281310
1825182588

73352

9857811
1

9857810

2111
2177678

1967667

90535523838611841581

3078123247
90535523838611841581

2169960100264383

2169960100264383

2169960100264383

6592491702748881151

6592491702748881151
431313264977

11

411134

411134

6102341562468341068
22

1

6102341562468311066

2112

2112

313343304543

313343304543

313343304543

313343304543

313343304543

313343304543

1007265516789

1007265516789

1007265516789

241516111717
1007265516789

108113811

108113811

231916161619

111161259

111161259

11
32191692133

31181692133

12056214881040395151118120592
195287159161208387

1092777413793331
2425109471843215224248628

222319617237817781

222319617237817781

222319617237817781

222319617237817781
353665286790

32

1

37518750100138

33322

3101112

131115
45698539123129

44668438122124

114

558130459461274
123

558130357458274

2112

151

4765625653128

3
96389955969177645708

1

1

1

1

1
96289925969177645708

96289895969177645705
27632291752842012132

2801002133228264789

15124
2801002133228264789

279997132228262785

1

1

32555223731294

2

32555223531294

32555223531294

38545365642307

38545365642307

38545365642307

94805516951485
191258126102

35265182923178

33322181911160
40415252822205

24634525

54745620

45768345336424
791091577669650

73414730

73414730

27289221926196
19242201617159

84723937

11

11

40562404030326

40562404030326

40562404030326

27462344626279
12311978112676720

43415163726259
715412

36400163326247

1

241511020994

2916921231587

2
31

1

1

23

23

23

332312111237
113113565568617501808

111

111

111

111

1

1

1

1

486467212298314607

486467212298314607
2

486465212298314607
360385185259205539

12633107

3016661225

734414227128

111448168

23
392917151544

321
372917151541

282115111228

282115111228

11

11

26111

26111

1
4112210

31115

1214

1221

1221

1221
11

1

111

210316115208166471

210316115208166471

210316115208166471

11
210316115208166471

210316114208165471

362519199327241647

362519199327241647

362519199327241647

362519199327241647

103441320
1541129474180710401416

9081586267106

9081586267106

9081586267106

9081586267106

1
9081586267106

9080586267106

1008520305388578479

91549715
1008520305388578479

1441705811397135

1441705811397135

212

1421705811396133

1421705811396133

311
855332242266472326

1

1

423829263655
855329241266471325

1771571210

1771571210

1771571210

391722174624

343333111330

343333111330

11

743518194536
62817796150310163

55414278131265127

55414278131265127

945638555343

945638555343

945638555343

1

8

111

111

111

1

1

212

111
212

11

2

2

1

1

1

1

1

1

21112

111

111

111

111

11

1

1

1

1
11

1

1

1

245424191960
429690373352381809

72107635860161

72107635860161

223
72107635860161

3

70102635860158

1
113114757085138

113113757085138

113113757085138
333411241947

14

233523252735
16312

222923222633

574340213952

1

23

3

2

2

1

1

1

1

1

1

218411211203217450

10281010829
218411211203217450

283225252146
167282166162159343

193321242747
183221242647

111

1

174224242044

3714

1

5388474752116
4379424249108

1075518

22

477948423886

477948423886

4110035315077

4110035315077

11

1

1

12

12

12

12

7895896076606395750910161
10810310758108179

138150128153160181

138150128153160181

138150128153160181
111813161821

100978477110107
8111391316

523240396442

523240396442

405431293349

405431293349

273531603253

273531603253

327948184462340933785727
12715016682120159

127193190239373322

11134
127193190239373322

1

1

1
12

1

1

126192188238369316

126192188238369316

1

1

215336318208232395
271240843749281925364773

2213

1

122

11

1

2

11

11

365508440377305649

365508440377305649

365508440377305649

159524112286169414822697
230266282224206393

116141128103117198
437561549409434753

263553282666
183129172143

22535

62198518

7093937776123
386151463460

4276812
84126914

122

3312

137135816
15131671119

1

1

1

21

11

241121

2621158

2621158

581021410
791271722

113

14

11129

1

1

1

971561479599190
761311278682150

912871021

1213122719

128136127105116176

453433242236
128136127105116176

456163545895
415157484983

1

31

11

11

3

1

1

12

11

23112

1

11

1

1

2124

111

11

143223222337
384131273645

1825355

673283

9031556142810408191514
246451362269266491

84101946590123

84101946590123

247299288210221388
223281266194203349

111294923

1361312916

17350349334998223

17350349334998223

6381816363124
7094936972140

124436

26636

452234

83108987872149

83108987872149

252827212337

252827212337

252827212337

63625

1

1

63624

63624

1

1

1

111

1
111

1

1

1

1

1

22

1
22

21

314415381281262515

314415381281262515
1

716455513851

243350326230224463
2124

241350325228224459

1

213408316256254506

213408316256254506

213408316256254506

3311

3311

2111

211

1

12

12

2

2

313388357266348470

313388357266348470
14231532120

177185149136190232

177185149136190232

177185149136190232
126140114108143190

272124162127

242411122615

1
122180193127137218

122179193127136218

1

431438192910272038023979
215197190143198294

232021911503143621542155
464533376165

56418
415392352268378442

1

1

576647376089

576647376089

576647376089

576647376089

32112
290258256184256270

1

1

290255254183254268

1091141157382119
290255254183254268

171917231726

171917231726

655638365347

292331153325
443648255941

66114175

9766911

553036264335

3334

3334

3334

605845445775

605845445775

605845445775

605845445775

586540435165

976256
586540435165

262815282128

262815282128

262815282128

233019132531

233019132531
1

232919132531

841786520541713735
314328263858

112
636530555861

253815192929

253815192929

253815192929

382715352830

382715352830

382715352830

455233314252

455233314252

455233314252

11

1

1

1

1

34442
969362709282

584927294442

2111
19115734

1995623

393822224138

393822224138

354035374438

354035374438

354035374438

393322292233

393322292233

91296313
393322292233

17197171517

1326643

364354
80130614910180

463833156435

463833156435

463833156435

318624313241

318624293040

318624293040

221

2510751416
329238172170237217

524138201928

524138201928

524138201928

1

1

19014790103141125
362922223428

514217255125

23211462014

23211462014

211

633319292737

17201821820

17201821820

333383

1

333373

22111
593734395544

2

1214

2122
563730375239

543628355239

564342293055

564342293055

514042282749

53136

1

1

1

172712172028

1

1

172612171828

172612171828

2

2

9810758
856157657269

221417221932

221417221932

543930364829

543930364829

543930364829

731242
235228140153164254

768045365265

768045365265

768045365265

15214594115108187
711841013

454123243459

454123243459

414830602850

414830602850

414830602850

594533273665

594533273665

17716210891166136
15696118

969149468865

969149468865

969049468865

969049468865

1

646550396763

646550396763
1

636250386762

636250386762

1211

2

2

2

1324

1324

1313

1313

11

11

626836435082

626836435082

626836435081

626836435081

626836435081

1

1

505941404066

505941404066

374624282256
505941404066

9644124

9644124

4713866

4713866

145107695294110

145107695294110

161153810
145107695294110

995930225350

995930225350

1
303734273350

303734273349

431113
290276162168433200

704455499246

704455499246

704455499246

21111

21111

21111

1741887492297115

1741887492297115

1741887492297115

1741887492297115

1

1

1

1

1

1

394031264234

394031264234

394031264234

394031264234
11

394030254234

594018272926
168013741177108413901460

1095905773718893917
344956244650

122

122

21

11

111

11

11

1

1

414331243030

414331243030

414331243030

414331243030

785150365764

2133
785150365764

281926211931

281926211931

483223153530

483223153530

514032324142

1

1

511531113
503729313940

14312111411

14312111411

312312171416

312312171416

1
13322

12312

1

313124
9779594810187

2

2

11
524534265740

524534255640
272718164022

1797585

1797585

8894813
11

7884813

1

403322214243

403322214243

1047459756759

1555769
1047459756759

342318121217
1

342218121217

494233514130
554636564933

643583

207122120122148140
241

2221

2221

865064575255

865064575255

865064575255

282825172248
1216856639082

8361783

702816244324

15995177

1

1

1

1

131

131

131

323219192322

1

1

3

293219182321

293219182321

293219182321

1

393732303045

393732303045

14213
393732303045

383330293042

12
1

2

998152656864

998152656864

998152656864

211

211

211

656186695873
12613

282655382134

282655382134

282655382134

363325313636

363325313636

363325313636

1

1

1

273232253453

273232253453
1311

11

11
262932243351

262931233351

22
8369564710463

493835194732

493835194732

343121265531

1

343121265530

918055725077
8122510

504622342643

504622342643

504622342643

333331351824

333331351824

11

11

435034243443

435034243343

21
435034243343

434834233343

1

1

354270226228304314
9863711

694847396568

694847396568
22147

18181292318

18181292318

18181292318

492834303843
786225

14128121319

14128121319

28820162319

28820162319

233476
555626444844

323412302327

323412302327
696596

12101145

12101145

14156141016

211911101811

1
211911101811

211811101811

211811101811

148909069103104
56425

762431304231

762431304231

762431304231

762431304231

676059355968

793255
676059355968

292824183137

292824183137

312332152326

312332152326

736657738186

272924483743

272924483743

272924483743

272924483743

463733254443

463733254443

463733254443

463733254443
202612122218

10414577

167781518

21

21

1
21

1

1

1

11096967590118

11096967590118
1112

576967424971

576967424971

532628334045

532628334045

532628334045

16292393535

16292393535

16292393535

16292393535

16292393535

463441273950

463441273950

463441273950

463441273950

463441273950

995740576070

995639576070

995639576069

995639576069

995639576069

995639576069

1

1

1

11

11
1

1

423266

423266

423266

423266

526850535589

526850535589

526850535589

526850535589
8131116919

1

252318202331
1

242318202331

193121172336

193121172336

12

14385975395102
1

273134193351

273134193351

1155463346251

1
1155463346251

1155463346151

320338193266254322

406566513971

280273127214215251

1

167121158130473127

167121158130473127

10210336
167121158130473127

6256335238147

6256335238147

6256335238147

6256335238147

3
9563115758974

1
9262113758674

1121
9162113758674

9161112738574

312

312

14111081315
1961104193884219851081

3312133
10956376115441059665

685547354950

685547354950

685547354950

685547354950

10245795525081007612
763832185340

1
1649971866793

11

11

1639871856793
945441372648

502118292425

502118292425

192312191720

195123127121165147
235553

155797586126108
24158131519

764231559060

552236182129

384147303436

384147303436

593546223837

593546223837

593546223837

446246215232354246
824617283438

452727273426

1
452727273426

452727273425

452727273425

17490919713095
2291191219

893428377630

893428377630

1

1

634752504246
16151311419

382224312416

9101581411
111

759366

759366

246574

246574

14583808015687

14583808015687
6888106

653840405151
683842415153

111

311

31

713430309528

713430309528

8438612933049

8438612933049

11
8438612933049

8438612832949

642242187187682220

642242187187682220
11

1
21

11

11

11

641242186185681220
111

1

1

212121
640241185184681220

1

1

32111

1

31111

111

111

634237182182678217
11

634236182182677215
481191157137477180

41109181811

11235162718224

1

1

210151130103231181

210151130103231181

210151130103231181
381920263030

394424213848

394424213848

133888656163103
16108559

533635235351
432215

493333215246

1

6442422810543

6442422810543

311
467323320221360351

21
186160146113153157

574849244345

574849244345

112

11

1

1

1

1

1

1

564848244145

564848244145

544847244145
1

544847244144

21

1271129788110112

1271129788110112

1271129788110112

1271129788110112
11

11

1251119788110111

1251119788110111

278161174108207193
251

1318072487795

1318072487795
835285

593535182738

593535182738

634230284252

634230284252

634230284252

1

1

2

2

11
14581976013097

14581966012997
4113

1

1

1

1

2171021013
9951613511158

412631238627

371820101518

412934251835

412934251835

1

1

1

1

1

754116

754116

73242

1211

1211

1211

1

1

1

1

1

41121

41121

2

2

2

2

188810178
231012692259127782912609

16482566212673

16482566212673

16482566212673

16482566212673

1075559152666536091810
223222

7113373643221278339

202221173423

202221173423

202221173423

202221173423

202221173423

6913153433051244316

6913153433051244316

6913153433051244316
65244

19711714310157691
915131

1815145188

1815145188

1701011249555582
227131196

121011631112

1517177122

451929143523

211025303318

7924704

27131910109

2116813758

461172163174639191
502630202624

12

12

282020252923

282020252923

8019443051430

8019443051430
461125192819

118

11

111

20510412

21754588

1

2131

114

71

2678539774391

2678539774391

1463698

1463698

221626161615

221626161615

221626161615

272135262930

272135262930
141422162322

112

11
21121

2111

1

211124

211124

12221

1212

62651

362220115934123291469
21

1111
1066110248113100

522025133226

522025133226
1553383

33117

17511465

321132

13874149

13874149

1

544176348073

544176348073
882082715

4723710

1

13149497

71323

71223

1

5127103035

1

1

161013773

161013773

256159105729122161368

23

23

23

2

3

256159105528822161368

33920132323
256159105528822161368

12710489320816471185
11331

3119467
838179616410461055

2562133

786578115810271045

4143134
1

4143124

1
434152

424152

4133122

129

21336
21324

12

10117728540107
23118028547113

355

1

132

652168

3324512941651
93134

87143819

83238228
311131

13

52227184

122

1

2

11

711111838120
211

1

79101738119

5121
14392136

72252

21863

45188133113103
724351

113221

722434

1682471822
3013722410397

21042326

1

1

11

7172

211

621515

1

23

1

111

2

232674539

41134

1

1

1

784444354849

784444354849

784444354849

784444354849
1

213
774444354849

774442354746

2122
770446408377462532

211

211

211

998460486871

998460486871

998460486871

988460476771

988460476771

988460476771

111

111

11

1

3321
667360346329393459

280207163115169262
313339

765259394466

765259394466

765259394466
31115

12112

2

725156374357

663832241960

663832241960

643832241960

643832241960

643832241960

2

2

2

1

1

1

1

1

3624
1351166949103126

353216112234

353216112234

353216112234

1
977853368188

977853368187

977853368187
1229

1

1

121

133

610137
967552327873

906551297173

51211

51211

51211

51211

51211

379144181212222193

442123

442123

442123

34221

121

21

1

1

21

112
1

111

11
375140179211220190

374140179211220189

21

21

21

1

1

1

374140177211220187
1514852387162

844228328640

844228328640

4

4

804228328640

1

1

13849971416385

11

11

13748971416385

13748971416385

1

1

512

1

1

1

1

52

22

22

22

3

3

3

2041302171284029137
211

1931262101184016131

1931262101184016131

1931262101184016131
2

1921262091164016131
1931262091164016131

1

1

1

1

1

1

1

1

1

1

1

1111

111

111

111

1

1

1

311141

2113

111

111

112

1

12

1111

1

1

111

111

1

1

1

1

1

1

1

1

12

12

12

12

415474

1

1

1

415373

415373

1

1

1

25712410685132105
111

11
24811910281129100

2441181008012698
1

2431181008012697

111113
2431181008012697

242117997912594

1

1

112

112

1

12

12

313

313

311

2

954324

332112

332112

312112

2

622212

622212

622212

1097971436155

1097971415853

1097971415853

1097971415853

1087871405853

111

21

11

11

1

1

1

1

1

1

1

11

11

11

1

1

11

11

11

11

561323
939621685584703799

1

1

20713911991127106

22141

2112

2112

2112
1

2111

1

1

111

11

11

1

20513611885124106

1

1

1

20413611885124106

20413611885124106

20413611885124106
112

20413611884123104
1053154

19413111583118100

122

12

12
11

1

2

2

2241

12

11

11

11

1

1

1

1

1

1

21

21

1

1

1

11

11

11

12

12

12

12

894765594637

894664594536

894664594536
261124171012

11212
33151829118

71014541
3214162898

25422357

46132
1

36132

261421102314
51231

211319102013

1

1

1111

1111

1111

635427496431528652

74462

1

1

1

64462

64452
11

34331

311

1

1

1165228

1

1

1

1155117

1144117

1144117

1144117

1144117

11

11

11

11

111

111

1

1

11

1

1

624414487425520642
433220193122

351250328315338463
116136816

14478105
279191271283259399

594126434932

1

594126424932

594126424932

16215

16215

190144238232199357

123112188195143309

673250375648
212

653150375646

615344267148

615344267148

1

111

615243257147

6710254
22813213991150157

634337345061

11

11

624337345060

624337345060

121

121

1588292539492

1588292539492
2037685

674041214341

713943264346
422528162721

291415101625

1

21

21

222233

222233

11

12112

1112

11513

11

11

11

1112

1112

31

31

31

816464
280153177134306173

511

511

511

511

1225765647776

1225765647776

1225765647776

1225765647775

1

145941066522393

21

21

21

143941066522293
3

140941066522293

113
140941066522293

139941056222293

11
24513913292159127

1265259418254

1

1

1

1

1

1

1265258418254

1

1

1255258418254

1255258418254

1255258418254

113111

113111

113111

113111

1

1

1

1

1

1178670497572
2

1058263427168

312223

312223

31212

31212

2111

1111

21

21

2

1

1

1

1028161406965

1028161406965

1028161406965

1028161406965

31

31

21

21

1

1

1

1

1

3211
945333

111

111

3211

3211

21231

11

1

11121

1

1

211

1
211

111

111

111

3
241276416

33113

33113

31112

21

12233311

12233311

21

21

21

111

111

51

51

41112

41111

41111

1

1

562290300271496283

56243
530283297258489274

643035224029

623033224029

623033224029

21

21

1

1

1410671711

1410671711

1410671711

1367498
447237256227428231

23161393512

4364
23161393512

96104224

1073574

1341
492231266721

11
25711144113

25611134012

11

23151712227
1

143

22141312197

75432
1065799847070

723062554339
241123

571856473128
114183148

20725368

11
20725368

19724368

131710147

131710147
22

13178127

1

1

1266375
854344

312
211

11

1211

1

1

1

1

13539
12857107

221
12415

112

112

1032

1032

1032

272233262531
5211

5102061322

17101220128

252136106104247118
1686996

959596
401317143426

318892520
1142

2231

2231

286881817

286881817

7653353811643

243121222319
7653353811643

11141

1213842012

3985126911

297963014

297963014

297963014

311
463821212719

19151512147

1

2421681312

32131512288

12
32131512288

31131510288

1343432

1343432

1343432

31

31

11

11

30721378
2

1321412

72121

72121

512

42

11

11

11

11

1

1

1

1

11
1351956

11

11

1

1

1

1241854
11

21

21
2

1

531253
1

531233

1

2

2

33

33

11

11

11

11

11

1

1

211
1

21

11

1

332017261180169614521781

332017261180169614521781
20490558953100

71236

1

1

1

1

224

224

224

224

422

422

422

11

321

11

11

583012232020
1

1044343

1044343
322

1131

1131

4

4

21

21

1111

1111

121

1

21

3111

3111

3111

11

11

1

1

1

1111
20122753

1

1

1

17111532

17111532
1221421

1

1

511

11

1

13

1

212

212

212

521
1

411

411

1

311

1

1

1

1

1

12

12

12

23
22

1

1

111
2095998

2231

2231

2231

112

1
2

1

11

11

821234

821234
1113

4

411121

4121

4121

4121

3111

1
3111

111

2

31

31

31
3

1

21211

2

2

1

1

1111

1111

821315185284243363
1410658

126533
1

43121

43111

1

11

11

63312

5332

11

12

11

1

9241

9241

9241

6111

6111

6111

1135312

1
1135312

1111

1025210

11
759302172260230333

3910691014
754299170257228328

32711889114105146
111283

1355023344462

1355023344462

36710101213
1816764726181

813734383439

642320241529

642320241529

111
38817175134113168

38717075133113168
4218415916

1496530505665

1
1968741684887

1968741684787

532224

532224

532224

1

11

1

421223

922314

621214

621214

311

21

11

572418291819
1073630449744459700

12

12

244743
451326226393189349

1

1

15

15

949323

949323

948323
927323

21

1

11

11

419300200369168326

419300200369168326
1

2

419300199367166324

419300199367166324

2
22

2

123

123

111

111

11

12910889

12910889

231

231

21

21

2111

2111

2111

2111

1

1

11

11

253111563523

5222

5222

111

111

12245112718
12

12235112317

1

1

1

1

2

2

6234051

6234051

2211

2211

1112

1112

171214

171214

112
533239191255213298

25136141019
518233186248205297

3
744237322443

121

734235322142

3
2479892119109138

11

22

221346

1

211346

191908411597124

3073476

1

1

322

322

142313

13121

1

11111

8264

8264

1506648684980

1506548684980

1

881868

881868

21

21

33251

33251

33251

112353

102232

92121

92121

1111

1111

1121

1121

1121

632925

1111

1111

1

111

521825
311

21

21

12

12

31152

31152

4234
5234

1

1

323123

323123

323123

1231

1

21

21

11

1

223

223

1

1

1

11

11

18756811
1149658473550670585

940564402462570448
1167135474558

432352

1

1

432252

432252

446255189224177198

32

32

422
1

211

21

1

1

11

11

11

442250186221176196
22721

3232

1232

2

1

20413112110910185

11

1

12

1

2311166410369105

1

1

7554314410536

7554314410536

7554314410536

121

121

121

15157735
285178141137233148

11

11

12077526415865

12077526415865

1508682657277

1508682657277
23221

1488382637076

1424635

22113
1423635

13

821211

1

31

1

1

112

112

1

1

11

11

1

1

111

111

111

12333124

12

12

1

11

2113

1

12

111

1121

1121

112

112

712251

712251

712251

17783627979120
13

2221

2221

2211

1

16472587177114

16470586977114

16470586977114

2

2

2

2

211
52112

11

11

221

221

14

1

1

1

23

23

21

2

311

3
311

1

1

31

31

31

2

1

1

1

1

242428212317

242326212317
1

1
232326212317

1
111215697

2
111215687

111215487

111111151410

111111151410

111111151410
1010913810

1

1214

111

11

11

11

1

1

1

11

1

1

1

1

1

1032417

11

11

11

11

932313

932313
1

932312

13

13

13

12

1

2414149169
251615951106108987071193

1

1

1

1

1

1

1

1

1

1

1

1

114

1

1

14

13

3

1

1

1

1
4242

4142

4142

113

113
12

11

312

312

1086434434956

11

11

11

1086434434855

1086434434854

1111

1111

1076434424753
22

1

1

1006027394449
1076232414753

725234

1

1

1

1138256
3292101941887547192

121

121

121

121

1

1

11

11

1

1

2

2

2

2

2

122999286710583
1

1

1

1

1

1

119989185710583

1
119989185710583

11

11

1

1

1

118979185710582

73125
118979185710582

282022183120
121

282021182919

81746965707256

211

1

1

1

1

1

111

111

111

111

111

1931089498436103

1931089498436103
5522

11

11

11

11

7351463136448

7351463136448

7351463136447

7351463136447

7351463136447

1

1

1155247676953
611412

1

1

1

602425373418

602425373418

602425373418
22117877

381318292711

492721253433

1

1

1

492721253333

492721253333

10454106
815262577748

422529364122
213141

211217172015

211217172015

1912918176

292328172620

22

22

22

1
11

1

11

1

1

1219477667882

112
1

11

11

11

11

1

1

1

1

1209377667682
22114

1

1

1

1

2222

1
21

2

1

1

1

2

2

2

2

2

2

111

111

11

1

1

1

1

11

11

11

11

11
212

21

21

2

1

1

1

1

1

11

11

11

112
1128872627474

1108671627070

1108671627070

1108671627070

11

11

11

11

1

1

1

1

14

14

13

1

2
3213

313

313

1

1

213

1

23

994647425337

21

21

21

21

11
974647415337

964646395335

964646395335

1

1

1

111

111

17451111677678880762
603244314133

502618172724
1045660298358523414

692306231247407280
806755487564

1115

1

131

1

1812126217
321517143813

114

511364

112

1133

622122

1

112432
2110825127

20962195

444722223331

2

2106945588964
6311

2046645578864

30198827815299

1

1

303328489489110
2

26331543868297

26331443868297

1

39125769

39125769

39125769

11

1

1

1111

1111

393260208202215195

564834243129
393260208202215195

1439564457966
271796517

523011213116

644844184333

673344313128

673344313128

435235684138
12189281312

131916271613

131916271613

181510131213

181510131213

843231343334

843231343334

21

21

21

1

1

1

1

20011887667179

20011887667179

352521181215
20011887667179

1277244414750

1277244414750

38212271214

444040203040

444040203040

444040203040

444039203040

1

1

1

1

1

1

1

1

1
