# Supplementary material for: Characterisation of the Carpinus betulus L. Phyllomicrobiome in Urban and Forest Areas
Source: Front Microbiol. 2019 May 29;10:1110. doi: 10.3389/fmicb.2019.01110 (PMC6549492; doi:10.3389/fmicb.2019.01110)
Supplement: Supplementary file 3 [file Data_Sheet_2.ZIP › kaiju.out_all_Bo.html]

Javascript must be enabled to view this page.

magnitude
magnitudeUnassigned

kaiju.out.epBo13
kaiju.out.epBo14
kaiju.out.epBo23
kaiju.out.epBo24
kaiju.out.epBo32
kaiju.out.epBo33

392242545611523514164377901050136223692700

392242545611523514164377901050136223692700
1362192912278932054826

635110757817816355203948115
390485645583533511491377590150107463691007

33207515302022351218132691
235524774615

411812713
1

23

521

1

13743

1

1

12112

1228

21211
483132274611961203517

157149324315158102
391061212

131614185524

2219

2219

2219

131612165415

131612165415

331310
1111712336

223172

223172

4524124

4524124

21123
11123

1

2

1

2554219

2554219

61514242718
11

2
332746

11261

11

11134

235662
11

2212

1114

11122

1

21

21

2

2

1968139
2312

1111

1

11

3

132

211

2115

1

11

11

2

11

11

2131

2131

2131

2131

2131

131434
1611473317

1

1

1

1

2

1

1

132272510

21

2

21

1

1

1

1

1

1

1341032732073130
2

1

1

21
1

1

1

1341012712073128
5671218

121
12894256203289

1

12

11

1

1

1

2123
12593254201246

12591253201223

111

1

1

18311

11

16111

11

31511554008401026379

2234

1131
2234

11

1

1

1

211
31111533978371024375

2801089276768995332
1111

11
2801089275767994331

2232

1221

1

11

2801085272761993326
112

212

21231

2781082268751983323

4

2

1

25

11
1133

2

112

1716102281125

111
15139624820

411252
111

1

121

12

211

1127

1127

138254

1801

11

112

122

11

1122
111

1

1

1

7799311
232218

11

2

2

212

212

11

11

1

1

2

2

313321

236435

2
236435

1

11

12313
12312

1

111

1

31

22

22

123951
2

93921

141
93921

21
511

1

1

11

2111

1131

13

13
1

1

2

121

121

121

111

1

113514291314

1
42432145

191111

191111

1

1

31511934

32

31211932

155124

155124

11

111

321

11111

11

666775

111132
1

111

111

1121

11

11

11

111

111

131121

131121

12

1

2

111

111

422
412

1

2212
1

1

1111

1

2231239

1247

1247

121832

81217241525

11511
61215211222

2121

1221

1125

1

1

1213

31

211

21

212

12

32

11

1

11

1

121

111

1

1121

21

1

2

11

11

11222

11222

111

111

12131

12131

11
203828844334

12433

1
12433

112

111

1

11

11

111

111

111

21213

1
21213

121

121

11

11

11

11

515931136
172221

1

2

12

1131

122

111

12

1

21

111

1

1314

23

1

1

12

1

1

11

1

2623

2623
211

31

2

12

11

11

11

11

431
6552253

111

211

1

11

4

111

7

1

1

3

11

1

2

121

81110161916

111

111

111

2425
71110161815

121

11

52

11

111

484752

1215

2

1

1111

1
222428291926

644945

23232

23232

412643

412643

511
49136510

465219

11

13121

111

131

131

12101113710

275836

832411

24133

281219183
27536047216920933204742072

32173465

32161265

32161265

122

122

439151864913842175676
912516817

22
305138143111112003503

201630421924

11113

11113

1111

1111

1
321332

1122

2131

83111349
21512

11

41974

2111

2111

4161054
1111

1212

321

14

2232

121

121

146714

146714
136414

13

213

213

323232

323232

2211955

2211955

12161

18224

11

13

153026392740

115

115

24314

1312

232

22586
14415128

22

11

1

11

125

11
1

1

1

1

12
16719

214

13215

2

1422513

23

21112

11110
111111

11

10183221

10183221
10182221

1

242
117565

15145

1

268133136410191952434

268133136410191952434
233

1210
119

11

2111

3631

261132835710041943417

31131
13

311

431
411

2

11

556494986054

45127128
556494986054

772115912
11021

22

1231
11241

11

212

1131
1231

1

2112

1
11

1

315

11

423

22

46223

34123

121

2341023

11211

1

1124

1512

2112
133613

11

1121

112

176342
11

22

21

16321
16421

1

537555
121

111132

2111

22111

2

111

1231

1231

41643

1111
3321

221

11322

5852

5852

814141047
332211

111

11
1112

111

31

13
14

1

11

1

1

1111

47522

11
154851

121721

111

1112
112

1

38815116
33241

611

312

1111
11111

1

213

111
1

11

11
13132

1322

121221
211

111

111

11

7061119159104102
32421

411
253163965954

314134

314134

24711311
81317231218

1

1

11

1

11

21211

1143

11

121

1

31

1

1

21232
111

1231

12

12

1

22
11

11

253562

31321

2223

211

2162731
1

232511

1321

1
445565

11241

12223

111
211

1

1112

1
13584

12221

211
1353

112

22

121885

121885

1
3113956

23711

1110244

114314
2311978

1321

232

21

11223

1
423054594347

3113

3113

191931302723
742525

134

1

2

11

122

11
1121

111

2

1

1

2113

11

141

12
1

2

51

11

1121

221

1

2

111

123

1131

3122

1122
121

11

111113

1

121

17312

7312

1

2

211412
11

211

2111

12591285
11

1027542

134

11142

11

11411

11411

524415

524415

113

113

225435

25134

231

212
3431342258849

61510951
1122

1241

11

1

12

21541

229

122
222619717341

22

22

11

11

16124495717

1573435617
711221

862415416

15161
431

1

1112

1112

1

1

1

1

61312181423

31111
61312181423

111

11

3114

222341

31

11

2112

1122

131

1133

11

12

1

1

11

1

1126

1
62945105

4274084

2411

2411

121

121

2233952
37

111

222241

22421

112

11221

14224334121518905178661106
41

241145

11

11

11

1232
141144

11

13

532

21
11864246113818805178111038

11734222110318774177891013
1122

1011

1011

12611
10511948

211122

112

51121

1212

1221

1

272
3031026

1

31416

6

3101225

3101225

42311
4111

11

1

1

112113

112113

1114421610731874717779968
531313888321262

33112

15

1

1121

25211
15211

1

2132

2121
212

1

236324604016

711

662117218026656

94513325119

1614102

917418269

1111

7353143767864016443642

111

11

1

142

2035901388916623147

2

12235

5131716119

5131716119
1521

17314

1

1

53361

364

111

13411

13411

1
669999

657789
1414

11

546375

221

221

239617

125316

12435
1

1115

123

111

111

1

121
11421

2

12

8541062

8541062

8541062

11116

11116

73391

11

571741
2248169794461

1420191987

21212

11

2121

4185213

4185213

1
82131562

625121

1252

16911

21122

11
21122

1

211

1

41514

41514

41514

16511

16511

16511

294540262129
11

7242452
72111

14

31311

11

11

311

311

2426

2426

625367
2

32232
31222

11

321135

5122121
16182814711

1131

1172

111

1

11122

1

1231

1272

62528

568191018

568191018
11

1132

1122

3

23

3446415

211

21

21

161971441932

161971441932
211

141966411830

111

111

36512548
111733

431

11

1

12

2

1

211

11313

1

1

11

1

1

1
144426

13133

2112

111

1232
989101014

172141

13222

1

32235
32435

2

111

112

1

11
111211

111

11

2

2

2

322

321

1

3353

3353

11

11

1

333

34253

11
34253

32123

112

3121

3121

3121

3121
1

32

32

1

20181613020479
13642

1

1

1

11

1

14121012019272

1

1

1

1

1

1

2

2

1

1

1

1

132

1

11

1

1

42217967

42217967

411414

22

12

1

411212
1

1321

21

16

2

4186553

2
222213

121

1113

11
216434

1

1

11022
11222

2

211

121

79186610

79186610

13
78186610

21111
2

11

11

22

22

5416259

114211

211

1111

121127

1

1

6560851555963
2531911

252639823537
1141231

1223

21735

1322

4421

21

241324

1112

3121

3111

11

121531

1

1

231

2

1

311

2

1172

115

1

151363

1112

11

21

1

32111

142

121

1211211

21

639438

639438

2212

2211

219116

23221
121110264

1

1

1

1

1

1

1

1321

1

1

671

14

12642533

14
12642533

3

512

111211

21

1

111

211

1711

2
3361853

213

1

11

213132

11121

5614567

11

512

14322

14222

14222

1

311

32241

1

3122

1

11

1

111

7382625161535

22
7382625161535

7382425161335
243

111413
314813811

1111

1

1

113

11

12

11

11

3

1

1

1

111

1

1

1

111

111

1

32

11

7347123519
1

21

1

1

7261514
2

7261314

6371514

1

1112

111

11

151817202114

151817202114
1212

31

211

3

11

11

1

1

5

11

225121

1

1

121

13

14

1

1

12112

111

21

1111

1

121

12

2211

31

11

12

1

1

111

113264145407397788807410654537184
380001444122143194714346008748517663442673

56981161299190

261849442841
11

2752814
141528392036

11

225213

3351013

414714

1

1272

213812

1236

12321574

12321574

12321574

68111
12321574

1

41111

2211452

308067856349

2581414
122611

13461

222

287559716245
172345364028

3641496

2331154

11

11

54271087

4121422
2941375513581655675627

1

1

10520163021

1

63

2

1

1

1

1125

2

21

11

2

13

1

2

2442

335141

1

12

11

5311

11

1

1

1

1

370311201412

1

1

1

510

13

1

121

1

1

1

1

11

1

11

1

1

1111

1

1

3

1222

11

1

2

12

1

1

11

1

1

18

312

2

3

1

1

1

11

71

122

1

11

1

1

43

253065949561322568526

1

5556113125101

12

1

6122111

1

72

1

1

1

1

1

1

7421

1

3

1

1

1

21

1

12

1

232

131

1

1

1

132

1

1

11

1

35321

1

8962514325

285811111

1

111

442

1

2

4

111

1

1

111

11

211

1

21

2

1

1

1315

1

11

1

3

111

111

1

1

11

22

1

21

1

1

12212

1

1

1

1

1113

11

11

1

2

2

31

11

171111

23

1

2

2

51911

335323
285276428551387344

183037612958
707412918174136

11

2

56131838

212211

912636

1131

33221

21

24422

2111

322

2114

1118

121

11441

443219

11

3221

222581

1

21

1111

321944932

54918

2

2532

131

121

12

2112

111

1

2

1121

1141

1

1

211198294367311205

195191272348301186

195191272348301186
11

331251
18819241311

11

24415

51873

11

211

661341

93635414211
37732

5261919344

9933

5261010311

371034

371034
361034

1

21421

21421

12
2321

1121

111738482124
225

61327151213

54113176

2486169010

2486169010

133122174218135129
272037383014

1

11127

321217919

18343824

2141

3352

224655

3521061

13513101120

711

23131764

748143

123514

579142

69114

233552

5282658

6211918

136763

85814

151727102815

16722191019
11

1151114411

21311

222

5235

11211

11

1

1

333088237943434034476623566473607744
173037281648407168474240

712
98160166248177115

321721128

321721128

321721128

321721128

321721128

1281737149

1281737149

1281737149

1281737149

1281737149
1281734149

3

8315013218315096
181643551829

111

27353

258215618016

831020410

1

1

111

12

14

101211777

212221

128541

1

3291352

114241

119157

114

1

111

425103111

453361

11

137497115044392880383424422119555958
2936529219928635203

2111
313329427573541435

13101614249

13101614249

13101614249

13101614249

297312406551512421
221615183019

63497310912593
323

231316263517

121626284140

252031554733

121142141173199172

36410117
121142141173199172

576267617364

617470102115101

57531021498667

55118164
57531021498667

7718271221

303437703525

15736442317

3452751027270

3452751027270

1

1

3452751027269

354745

354745

141932483124
174318353543383382

14371

651017128

118643

1422

4321026

31563

5112536934

422

2

531364168

12

1

3596124

26544

2131

9169142218

271856

22627159

11302123294

122541

236251

121152

1271

45412102

111

332221

131740583332

16533

1356

1

1

2623

5411743

12434

12249241311

141617141327

13211

812182514107

11442

32

579647

1211

162219215326

3511

321645

24291

77320271313

212

1

311652
83109212226234243

151657507752

151657507752
11473

2221

2221

1131
7714172918

426563

12

112262

21

1131

1134

1

223
2155610

215347

21131

31

13211

13211

148532
1111

32

2432

111
1

11

2514
1

141

113

15251

15251

4121

4121

265114

2112

1

23412

212

212

24142

1311

1132

22568
112

121

1

11

1123

122

221
6592154170152189

413226
47561049587156

111

111

4111133
3851877469136

1341

112162

1243

112
1213

21

1

11222

11

7112
5

12

21

2142

1413364

2415

1

3121

2211

2122161

1320513934106
3511

1317513433105

26312

4

1

1

113

13
113

1

116854
14521

123

2

1111

41610107

1311

313694

312

11
183347726431

11212

11212

21692
316202910

21

2362

1131

1

1

1

1412

1121

11

117996
153139493419

2222

2

1112

1

2

13

3214

5273

1221
2221

1

113422

215431

261

111

31

222

21

11

12123

12

111

1312

1312

293450542447

293450542447

293450542447

293450542447

293450542447

136376113378390556380765419843549361
15415614710244513826931025721

8164154280115175

8164154280115175

8164154280115175
101431823854

11724661624

23517161330

14152324915

121417231016

11942692936

8364216487207161

8364216487207161

8364216487207161
15713

1

30956985249

3033701616345
2632681596344

41221

2321852219163

12
1724272531566388500

1138410
1724272531565388498

335232

335232

1123

1123

271135
441834615

1121
11

12

12611

17617

22911

12817613
51

126459

284

45935423338
111

44732362733
44834413238

11545

1

1

11

1
131822

13622

11

1263

1263
1262

1

9303594655072
1668248459448329415

4111

225537

1

25735

311310511

1212

437

1413

1110225

21452

12541

1111

11

1

1

576318211216

111
1

11

11

35412

33225

121721

3122

374550444751
476164506272

10161461520

1

1536
536

1

213111

123211

6441371

12654
11210114

146

1

1311

2111113
211913

2

313782

1

1122

23
12

11

1123

32244

31411118

112

221213

6985

171

1

23411

14414

211

11110517

2173762

611324
732424

11

1

11

36541110811
249888

11
3212

2

11

2

315011
11

11

3049

265745
112775555644

2

92170485039

28153

1286

1

2224

116448

3

18883

113331
21

1123

11

117

3110

21951
1221062

1

1

1111

31144

331133

1315518

3

132

6235

21

21

1638042143783340094691312789
156161044230

1543731231724277414509810807
5056145439915473259455561

2617821149067

2617821149067

7911381313

7911381313

5715412533

5715412533

9811125438310219174044067
401969821349

1165347124319454471246
88108663038337135153034

1761558123

71212222

211307

7316225

842345013

72183810

1
2318239

1

2311207

622

64195

4210315224252

121

410214

319121

4320186

1511324814

3871343512011912399

1158

3111

583

25973188521323291

411421

111

1113172

1505211493754165

161121459

32416911021

23133

29101

1

327183
112

226163

711185411

6054234713316170

71122253

10326396

24568227481187230

12232810

211

5519258

1021264815

841255113

21094

3342

123

136608915

591316312

1011434812

281113659664

91376413

291017613024

61214235

10524509

21873

321244512

21951

538274

38141

3121223

4611515220450

131564

2311205

616174

3213143

143

2215158

8123339

14242549

95232448

23448

9110162

41175114

171377112

1174

226

7111348

94126376

1341287711

9121261

113

146727817

112

4113246

1

22312176

48141314822048
24586010019

1193656618

1484

8214283

45184

2195337312
197529638

1122

11284

8903445315913366918
8463174814993172861

442759219457

311814493120

311814493120

6918717933464
235102257658915454

12131

41118209

1362

1

1782508017

8225418

11

2101

11

311

218559014

6530190157130240
6430183156130240

161
15

11

1

1156

11761

1124204

43

634171711

2311287

89162

1

1127

66920711
63817711

313

312133

1788

3111184

151633201534

123

1212

12719711

11

4117137
232457977371

521225199

81119412435
7919392435

122

61115241720

1
5454200321172189

2328721578473
2431841678988

131210515

302211615483101

1869594527

1869594527

29734125
721623

1113

2141062

71

161014542510

161014542510
151012522410

1221

2021351005652
5977117226142119

115625

71511

1163

381814142

2715

5391994

9914543220

61939225

2417107

5142

1113

18811602015

18811602015

111324773423
7674148298186156

11251

2121234128

162924242711

1914312520

7617322511
5415252110

222741

3913

57431129

6111301424

5741283146

2429391425359
92510852035615917721951

18855973468
178115301589226307

111

5129361372433

21

221

101228412234
101228392034

22

17617441012

16928442329

101027562523

18824572434

8834392044

141635523014

651620911

81133

140183209495249251
4105387411684844867

6713251754

481743

213014402527
213014402526

1

281920272364

1

14924502514

1

213113

42215382220

9113451616

91138613138

1

2

1123192

3639662231

112427492116

82715311118

886281112

8164985

91426543925

181043834825

2222072

2153

202526361417

112528382221

42214381820

142829574132

10822441733

1113

6416431919

211037894632

31014291713

62014492823

1851411

3211

6211531516

54421451818

54421451818

341453925
3726123225102161

6334592534

171234543852

11641593050
11641583050

1

1

3242135
51359526390101

12524552131

11821532533

12918481516

131128862616

1172195312870286217
1126665284014

27611369808157

49611483627480

307118110009166

91129502542

91129502542

281840673444

281840673444

151118341230

151118341230

7833232025

7833232025

2593187
4431641676280

11922471423

21924552539

1089341511

38511

38511

109811132563326816882222

2831771375784
109811132563326816882222

274354993781
7611241312

12171430835

51315261215

371419419

3412578861170645735
3043941437766

347691064355
337691044149

1226

10584632430

232071735742

2115112917482

22931724446

3

201125501827
201226511827

111

232361934352
232361944556

124

162038673040

21

301952885449

313255994644

192247373034

302284946195

11

32861894067

321671994645

321671994645

131420401619

131420391618
131420401619

11

102264513733

102264513733

14931512134
2161212

36410711

13933
23943

11

61010516

11

1271032

9899191238109193
49858510741133597932

11861897

11171018

327734361834

11

11

233824

33141514

311025512

46121665

661014555

4522112

7389102

6115201514

10617412116

101037126

3232467

221

271748

9413231310

161

61227

304915181321

262048192522

1141

4674

24710510

2151669

13223

11

2511

141

23212124

21

1255121730
1255425933

3423

615436

63116121412

29836191432

11922332425

6

21165291937

68917138

1231

392923251629

16555

53120181113

35864

1031127521

9623261414

538135

107

34201869

511243470

321216711

731920168

91819111423

2242

65110113

37433

24410156

11

126857

6781134

1316138252016

1

4157762

45104254

7471666

1211924118

10730403136

10730403136

1610501234437

1610501234437

235745773237

122423371624

113322401613

6138921247776
6118141012

362544673834

141026392726
141026382726

1

52224
523324

112

11

11

11

11

7817422229

7817422229

443181211

443181211

141249641332

141249641332

69761207810122137621153911811

69761207810122137621153911811
85137164249148365

10789226300132269
1232245720

5913161121

771117512

111122391522

20843532043

6716311332

23911113

6627402031

192532261560

191031221525

1111937

1111937

182256272394237260
121023593230

7491743157

282928351625
2171220911

7221615714

121317211923

431411611

471214108

10172343

2889611

121835

13665

213514

131107520

5726422215

167422463631

192110161118

22166812

2232119813
22322221016

1323

231426

1

20637372410

122515052424311217303585
6601115959449128101101910910

771689
713171011

6122

11132148

11

31137101

651995614326995

212426

2361

425724

2166114

64225

11

211

211

531421143
541625147

1244

1212

242681

1121

82412304211

84322585038126

84322585038126

7610162012

1

51491259

23224

1

165912710

31071588

31071588

1

739746

424611269
22454234

21735

21735

11513

12

1211

23148719

1

2377269474633
410191592

2336250323731

3781237

61

524962

42892

1133

3121

3231

222443

131

4210536

1521

811

215765

111372
124373

131

2

323

101152047

36331439

211

1

62108196

919521

225637

2261064

1

6681159

14

23216612

1436

22111

21

377048577043

23443

21

1

2691136

1

31453

2951628910

1212

41431331727

7113241

714188621

4481439

1

172132483823
151532413518

26735

11311120119

321617

1

74111615

5151446

11211

162064452723

3611

2114315

3

11456

1162

2211476

12610675

3101122

131

1631881

3271378

2221

116

131

43101649

3551536

1287101

310436

2216

44436

11

221

2540101545
2640111545

11

1132

59111699
5291576

72123

2325361217

121

6726421122

7316181025

1

11

12211
211

12

11323

62638341830
57529251425

519945

421013

251536

1

22122065

269829
28188210

291

297631538823

61671195

13

347872

499241320

11

11111

323521

9459445430

2

1111

317826
5181049

21223

1

111

963143146

12

331522341725
382025402125

55364

13791436

114

2341026

2117

11

4117564

1
165437445741

1336

165335415229

1125

5534

58232298

52

31161034
3116823

211

5203411

1

334432

14918122

21

1141232

556775

20461224234

42243

11331

1

1

323

1319

4617161625

111

397927747224

211035

1

115512127

139111153

2

4181674

1

3461322

315

13531

2111

604540523430
524538353218

711323

1129
1149

2

484646
463333

21313

1

103515161319
6341371212

412917

1

31993326

1111

23331

323

429562

51

11

339677

1

61730131432

171653535176

2262111

111

13323

15685

411133

121

8510893

44121962

223568

1262

71125361329
581834723

237266

1

3121315

3821015173

81525331413

134717

12585

617841

42

1

11

685747

131935393931
132136394131

212

373287

48111435

661215119

3518

32614117

531412118

2526111

241023

277935888037

448885

16421

741513163

512

421412

4241415

231774

319772

11840193776

416554

72011293215

41712104

118782

13134

88951314

213

1

11314

112

912621126

9524151118

12296233234

1124733

25161487

55234

63158610

1

313463

9712171720

341

1131

3314

3176932

1111

329213

142044391041

2998212

96172659

4175

37

91

91

7116261311

5721231314

5772155

218337

7512218107

15415

314319352235

41

18343

545216159116213317748

231

121

1

284217304815

102815101218
10281491117

1111

131

1

3551734

161032371829

15202219262815

1

11

3121563

41155910

3789511

6788951024669

5445215

8131117189

573411

1816231457

1816231457

111

8640241523

12101026

17536

4882359
81949523067

41141292558

29131

4210101212

1

611

13171464

21

3691175

1

1391278

41332471319

112

23414212212

2

6317171110

8125252217

163832435133

3568410
3569410

1

6719131330

112

557714

91114241115

6618101615

11

22716119

5218

859467
1

714244

144223

478126

2711822

29310129

111

2

294252

112

4139146

1141

32531

324421

52131242

456614

111624281515

113254

191333362333

431810419

11

11

111131436

335112

5417301016

122

1275210

21212

34627116

131047202014

83881720

212621

535533

44219710

1

111

76310515

125478

918784

323733

429657

3116693

132

83211313413

13461

161212161728
161211151626

1112

22211

7684134

19112

1

4228412
8771615168

455811156

71922

1

233116371121

517121

66282769

4149104

714157221

128103

31117828

29117141978

3211

188181376

11

1

24251669

2

22514

1091512926

514

1

1352111310

35107116

12

12

4822231121

1

2122

326452

1

152

33510886

1212

112

21314261630

51720310
31719310

21

16134

113095331

6

12

2

32441

3229

3223

3

185317484515

1

34313

6491154

1217322615

15214

1112

6519113

114612

235325

9621384

318231030

212154

12913229

33151178

51

132151048

111

191829512122
191628502022

2111

343322
333322

1

6920111015

9277226

25551

122121

931629416

1

141623

143323

2142035

6161020104

22129106

1171

81421291535

1

53991012

37154527

1141031

142536

1321101419

22813

65719412

41

841016612

64627522

741613817

122

51034411522

31120411210

34476

5198381

101215234837

22

1561746

716716242

231021122

111122323641
101020283338

1

112423
11

112313

4122918

4231357
4251469

2112

18623

135917116

2212202

31041343

61323301325

211132

54106416

1187123

43242

1271787
1261567

122

41920101818

339914

25143

1

1

1112

131

148410

53412111015

1621281

222212

1188811

41232

173613374312

3749624

1

327735

11

1521

5916382750

204515376414

1271353

21

410151957

9617221110

1

226426363328

251069

1132

2

143324452611

1231791

11

4331056

82739

1213

841513104

223112

1

2

11512

11127

16851

9835522113

12

62810412
114811614

52122

3491447

221612

6211
314334173866

124

254234133761

4451093

513219181424

2219856171146

27562

1371635

1316

7110838

184117153922

5441

371085

15827551325

141321152026

3548813

7311753

61061126

11122

1

73166713
83167714

111

244

2131043

271022

312626382911
312627403011

121

1213976

26521

517514

3

3498879

71411131116

4121851

2431457

181065333040

4131

12

15436

13721

6171513717

11316161513

6662124

2544

24512
1212

1241

2104314

421674

1312

42792866

221

1

351045

9111523921

14111074

7915858

141822141636

6411111116

261

21461218
416819513

222745

121551271032

1

814823

1

11

11

52814191329

138161110

11

4423241112

3618161216

323633

5626242011

41

59151054

11126

14

22

2211

21

5245223525

33555

3

224422

11132

526171012

4

111

21121

62915718

14121

278189105

11

231

1

61410311
72510511

1112

35810158

4214384896688
1

1

1

214743353332

214632353232
214743353332

10

111
1

11

115

2231024

2231014
2231024

1

1

8113271111
1

711124119

132

1

3124
1

1

3122

411116927

516

111

41055911

1
8111

80

11

310599

310599

113

1141

151

272742

121

331

5281367

1143

145826

111

71025171618

1111

411821

11

3114112311

211

22041765

17814111611

5210310

337667793390

314534

307563743086
103629471646

10131515619

10261912821

1

19301113728

1113

25456

434631

531318106

1131232

2181

505568804453
464668804453

49

228642

312392
21222

119

1223663

1

322958

1

83143237

441414727

673334836

2181

112010487

4211

961312524

81456322

1

21

61211211711

2

167110

42316161710

263920181824

3310611

161821271219

7326122

741

124542

124921

22571013

41411

1114

21

205820445824

1

131

45

184211129

35611

57232386

11

1221

47223

424732

21214

81247331513

112

2319
2210

19

191331141836

4791664

131913232815

1

42913126

45141755

1366945

54218533

11

34683

2161112

1242312

1

981316714

10913161114

1

426844

72639471920

13

241164

731229623

1

141226102812

11

374647

37975

1141121128

1

232452543624

13543112

6141227
21472

4525

4486216

712522

31734

8347212814

23323

1321

26489

17111159

2176814

11

192022161226

1111

1161827

11112

234783

1251

381611511

13112219147

4513564

122

12312

24622

106914167

15827432624
11716262216

2131124

28624

94746159

166121
61281257

5621136

19827472022

5481477
66922831

1218124

518611124

418977

181041

131333

12211

11

91720211321

11

333334

1123162

1

587311

121014351822
4255539561534708799

8518461915

8518461915

8518461915

651021042317781

651021042317781
232952

2456591083554
2456591063554

2

394642943725

8718551210

8718551210

8718551210

3435
3324298021167582671

1
26201371756386

1912711043548

7866712837

306409662988516580
114161319371183215

21151

1

1

11

1

11

2121401404557

182713162314

152212

1

1121

115

21

4627392113

21102

5

1125

161627816

222923371025

1

726945

111

5410201712

42910720
2162410

2138310

251532148

19726695217

1

661218810

132118291715

12

11213111011

252640563135

321

131020912

232102289

79111114

1321

2

132211

21

232876

459917

171117231325

1326948547384011439

4214
1326948547384011439

11
892235020442611284

1

1

892235020422601282
882134120202541261

11922621

11

1011212084137

1011212084137

118251583627
2620672587176

451351814

11729492735

7164721862738

7164721862738

46045310373839738945

2623884526378
46045310373839738945

115110276663244267
181028993528

7318551213

621557209

91831482224

231036772424

9718482221
9717452118

1313

92045812833

4327512252

7216311628

4611341613

10111025137

91821571415

3014562055650
162

9419831316

9314381710

11723782622

7791212549119209
111101

3335771134066

910321022545

11212170620

1214341642042

111047902736

2622663224260
2122154051970256341

351840711

214919139

5410571114

1413221081716

25222313

6733468

113552032222

541057610

9617461118

1921431042337
1920421042137

112

75124689

1072144620

121116492017

641392154

451536719

5141864816

361532146

1723505951732

44212176

44212176

1

1

34212176
34212175

1

962123176
100211571756274013621701

247321486804364490
2261047

102525271420

102525271420
102524241318

1312

10726321314

10726321314

611981010

611981010

1291
71127542423

1281467
1271467

1

441561
1441564

13

4315151211

1

1

5440931687687
212265393673299416

671914

41126462814

491818127

6481748

3110576

4212544

710138310

5717261510

6262387

771146926
7712461026

11

11

321012314

1

3651685

5722261612

85813123

5826424

211

16714222

1

353181539

1271810233

2218265

10314221513

2910511

640625911

111113111

2151251
13113

112121

141123131240

11958

32472

10411251714

296275399696346424
1231083

1062027312
73161627

12341

21175
215

125

405118513727

405118513727

234207345579283367
6611342012

4314191511

647111721

9622282919

254235362465

23528391717

202117361330

6174147

131522351826

4462020916

5214251114

7413331215

259301312

222129391522

1

1

8826462016

62313241311

8122326109

202335562344

1

1

11913291415

11913291415

4505558501217635781
181646601921

281112181412

281112181412

212243362645

212243362645

755110514588106
2413161010

10418242521

12

343229331118

7825401031

22320313224

35713331811

35713331811

6714421631
5266109182104139

11724553020

2249634

141833502867

3820201613
16821232115

131352

328662

11

1

1

51019211111
31115861284679

71182258

77624561423

6261720928

628979

32971141297093
71533382731

5662530915

10461555

5541322029

437946

142557

81018261310

81018261310

182110304459236265
251541814843

7511281618

10735441935

1

10626341510

422071572431

439101318

1

11

191325472139

8214311518

10423402320

1

81164103

161126461922

22121635138

4540137254130136

111
4540137254130136

10723381716

10723381716
10722361716

12

346141515
3330107205104117

51624814

1731478

4116571214

5419211230

932332205

16511134

5429321727

1361093

1361093

171416593080464223603039

171416593080464223603039
49471192189396

12823332017
7610649

3171354

21614114

4143688755113
2131

173237292292

24931573020

5052751236293
114611

101426342118

92017282028

301728552046

191121583037
4371367

94926811

645191619

3403667161260510710
7658152334133143

1

11

1

422

16824312323

4101

51436661942

1828451054358

510231147

11

23

212915

3154541103970

91028501939

151533542350

1

1

121

71132492413

1

21

9712321022

241446943343

4210181717

61430261515

12212

544112096

2221167

381951873660

21

62545

121042361713

142146611930

311026381031
311026401032

21

215
172136611433

7418175

101516391328

11

507416420495170
4185127431085558696

4471284

32541410

14926331020

611428362729

1311

6910211016
81216351120

2361414

73191175

41038910

4431245

12181313720

612111171

61771245

28102969

83216361619

1

3712613611

4415191016

631811612

677151016

15913

111441613336

41412171224
41411141222

132

3413292111

2211613

939271812

35936919

6479117

71619191315

293771

63642
731643

111

231

231

1121

372

221038472521

278521

121435493032
121437493032

2

131333282631

11919352625

7381224715

1

4319161313

7512181213

13

1731152

212912
4141512

226

54926119

11

79223271613

271578784877
223

3521151820

17845411831

7210201223

2822761007695
213

201451574546

8825413046
8824403046

11

10676209219108175
19738381520

31519361915

1014248226

82935812

15523381830

482343242152

2

2

3941382116
31053402320

11

12124

6433641014745
3212

9818151719

131614341111

10832521913

8721251012

8721251012

12612
43471001477988

51234381416

4212221115

161524352627

131015251014

5713211714

343052513633
342116128

829141013

232422211412

36921913

36921913

10321392836

10321392836

43618116182102
192737814451

422

6661797

121

4281355

11

6101631722

71214161414

34821333019

1

34821323019

13829421924
6639100168106119

1181617

126892
126792

1

11122

422

2216224

251

6131417910

9313121019
9313131019

1

2421

414111611
414101611

1

51345

441016187
44916187

1

20491776

4929231222
8454162152104148

4341625

652371613

24214231614

201636211645

19420271615

4101015519

121712117

2398108

1

1

6424451323
2822661114969

5411231114
5412231215

111

81323331019

917101412
916101311

111

527221211
163165143206134153

46929242229

222324391926

121

121

51520610

11826202620

201244492743
181135441638

2195115

941318126

4421151498

2711761047260

2711761047260

111536
364218453800315407

162
1722

1

11

21

21

1

1

1

174
363214452788309399

41411316

3635681154853

8108151919

25841

139281351817

1311791113635

8314352220

13433

493111253

4159721

3101218133

1

211

77611242023

1679912715

1

1111

1551746

13663662654

6947592830

11

5581468

15736732445

61333361420

131

351122911

351122911

351122911

351122911
351022811

11

465496112914329781479
110

41016492428
447462105013109251381

11233
13823243021

123215

11518202513
2566133

912141210

111

141237332156

35533

13643

51249843

61051367

11
8418241224

221143

641612820

81126391923
11

291481

4710161011
4771489

3222

227918
2379110

12

91812819
10127281332

1

11

1816413

43823526

43823526

3847137205127164
37540486710467811150

1141046
113944

112

3106964

10933622832

1451

222518

112

431

1922
2

192

6351055

11

51418710

212

113337

32213

2521

11115

9524251017

1

314512

14
1225

1121

2142147

3261
32162

11

11
111

1

14126827

1131
1151

2

121
1

21

1101496

2281153

31

202916231010

21101411121
21101310107

1114

22713135
21713135

1

17719343623
12716323621

5322

1

28333

62923614

1

11

11914133
11915133

1

4123

1111

57
77

2

12256

142112262123

12

1132
1122

1

11

4105229

311

311

11127811

152201339164294393
8312445

144198327160290387

1

22225
22012

213

3314401554
1272979

21711845

1261167

721726620
61323417

111

14223

11

2616778

15

361366

6513321825

1141

1

12

33661711144
222

3366169942

11726
1726

1

1131454

1816

2171436

51
31

2

4241865

4241665

2

7724261416
42141144

2452
24522

2

2121

1351069

1834791225288

1834791225288

61214382213

81036501846

21223251025

26924

22

22

22

22

22

22

393712015678127

142344773643

142344773643

142142742942

22371

251476794284

21432
251476794284

151155533068
151051522767

14131

832022914

910880099505198471924912904
2545126298922967463

2
1565479168388667552283

938431422

938431422

1556476160384367392261

1556476160384367392261

81012281827

1242
81012281827

3339711
2338611

111

56917714
5691677

17

21
242828943969574148814587

241928843957570348574570
4655509211348899945

111223

4320361617

131447

1133

221

461248

114

113953

3431

1211

4331113

155
1

55

1452554

211681

2113217

19530241212

1572

3261027

241

11

11

2371438

15819821

263944

224115

32

32127

83841

244

13335684

1

1

1

4291321715

4291321715

33525

11222

65149122168169104
88178137181185130

7134498

332836

131391412

13353

61246412424
61246402424

1

16810232710
16811232913

123

1

6223132012

322829242227

4211

75536

343537

421232719

2813

49841325413

133

14

124754

111

11

14371

1372351

44921139

32714107

2213121114

2244

46910721

3210912911
8811952836040

274821282414

296122432715

212

2

4522191211

221432

22331

321

96371096

85925128
85926138

1

1

1

1

16354

422193

1

121

22825

3161591
32716104

1

111

111

215338

1324

361729206

51

416854

1

1812515

1

33211174
84414195

11

512211

211012153015

224762

61959

2372035715

11431

13511107

3137171513
3092895386525611051

11823

8131226

615785

163954

259276504585510984

259276504585510984
69129187235125419

112

158124248281308443

21

1

33335

1

112

1

108920810
1081020810

1

1712523962105
1812524264105

2

112

1

1

3214111736
3214111735

1

132

44123

55148618

241676

1111

12

111

201199414379

7191096

22652

6141

7311491

29234

8261143

329936

342548376867

261478

11451

122015441614
111914431612

2

1111

8722473915

113837

114597

12431

773493

1

1

262352161449
232340141449

3122

12

212243

211

13214

81523362221

1412

11

41121235

2281438

4161337
4161647

31

1431023

1

26833

11122018217
11231

10121914186

2

3521

12321

2531

4415421421

551953

1112

22101997

4

4734

221211
221221

1

2114721

2241

11

11531

643123

71128311424

371212612
371213612

1

217445

171828352552
171828352452

1

14

8512201011

42420154

144949

101027621116

141

214851

5192346

1049222032
146246173267281186

10181011414

11
111

1

1321

45841

41

1

561324313815663
711869118119694

11111

1

221

11
104930251419

312494

1

11

7491719414

1253

341392011
1313511

1

1

128

11

111

11

1

1

124212
124312

1

1

3516512178
14125

34124796

12

112

1

121415252926

3

2141

12221

3244

662114523

451183

254541536051
1

1421

1111

15824

222512

184125295234
184125295238

1

3

3

314737

15112

82614673

1

225536

25661

262232

11

11111

217864

11455

11

61581043

3481032
34911410

1118

23102

3

3

222867745270
4151359

1562

452081323

512

112412

44535

32821

242467

1521

3
1

2

211

6816151414

223244

2

231

1

6201327348

21112

1211

1122

54719109

8817211512

133

320330391607930562
71713912

111

31132
42467

11335
11334

1

153171086
1312

2217886

522
422

1

275273300536877419
293324357576911536

1

1111

211

211

111

1

3

2

111

1

24011421

24011421

1

132

1

1

1

3111

2

1

77431728107

1

1

121

1

6251

1711

422

124242

11241

15441

12781011

10291993

1313619114

326223577019

2171366

14543

214619715

491844

125941

3341138

336886

41102486

1

225241

11

211375

62624

2061512719

52293

233147

11133

481517914

12324443812

3

1

4

11

4135

3112653

38215

24541

157823538015

11444

49515510

3

11

121215281211

21

1462
1332

13

23

351514117

113944

14861

1111

122211

211016151211

101226111129

2237102

111

51121045

415101664

1

11

11331

1

171331171020

3341

1

489412

8714184316

842310

111454312834

1132

2262078

7381067

610263

610263

1521

91012362317
34418138

47582

2611327

9133145211101140

9133145211101140
301242552734

413316

13882

11126521816

311220710

218202289

51101354

617131023

10924251526

5475031081226415561221
72013413325

445171810

11

44515169

1

11

489445823147914071005
136185195431514262

1

1

25112

1

312146

3244107

222211

114

258411210

1

1

12655

314141311
314141411

1

11

11

103101388

22482
22484

2

12

564223316
564233316

1

112681

1111072

65614124

37103220

323994

1221

461210104

24322

1057598

32109104

1

31

1

2131

11756
1715

141

11

4341182

1

3101068

4109212

21546

1122

225944

33572

1144

1

2112
112

2

41556

24410179

231324415241
221022405040

1

131121

191294
191194

1

112521

5

416107
4161071

1

1

12

2

13

1

124574

5131063

2254117

1121
1122

1

1111
11

11

111

1111
111

1

1261

4566

11

11214

435111328

218101010

11

1528241

12711119
12710119

1

321452

2241

211

1

1

1

7213191715

10147151620

122162

21712133

1

4232

241

22283
45899

23616

1117982

212824

1442

111

22511633

12

122

211051

12

221

11175

11

1444

222544

4137144
4127144

1

1

131033312935
251210107

11521211928

62711187

14311319

1

22

81114223

113561

12

71223121019

2

11

131314

1

1

1521

11

421

11

1

34612155

8114172211
8114182211

1

3285310

1156171

161223202045
141122182045

11

211

24188

151460553637

1

771021410

648191119
648191114

1

4

221

15141310

2235107

3

41211312

3311

233173

132641

34926106

227446

436111110

13426493319
13426493421

12

11617167

34131656

11

16

213637

473424072798181

473424072798181

174811228201047220

1245464598773147
174811228201047220

614365010

1

12413206

10412392711

1119103

27110192

1431535337

13416568124
14416589226

12112

111

234791

19115

1

11411

9939534335
135035723144428125313274

3113018331098655990
74328391979261615742133

691020108

1553

121

16311

1154935

12

681719205

21

161235653425
161237653726

22

1

1

22925

2151

11116

221436343738
221436333433

22

12

11

1114

1123

2

2

11

161

14144

11

43

3

1

341119148

2441

228676

251336710

111

1111

1

11

12520341516

416662

10823201713
10823191513

12

46

27101354

10619191116

4

9630543023
9632563025

12

12

132

12

111

12441

1

26765

12235

2322

251263

1252815

24177715133

8213321520
8113311519

111

44331

6202138108

256201210

1

21925

14875

2343

102031211028

10206634533241
10206640553341

621

1

31126191517

311015106

3123139925153

21353526

431

741435

1621172
37816115

2514

1143

21225

303698120136366

123

1281

8513392411
8516462614

3723

451519827

115

1114481052434

1229

31118141

32844

112

2412344

21

23751548

1111

31222

121211

6314214
6313214

1

31319

1122102

78833

2

1111

101336631640
111336641742

1112

1

121474

101832393119
102834453119

1026

1622
21652

113

1471228

1391164

17223

35734

4

422845683726
121129442925

211

301713234

131

1218

93812157

34568

11

24452

128

221

8521

21

3352

3111053

101117421615

101117421615

588713110915708981091
116174183265152192

18930291220

10931151222

891621911

4430172621
1112

4329162421

11101267

162026192042

3321151711

7810121111

14162040912

651121517

1493933

221643

3231553

531181113

5617281121
5617271120

11

6181911618

11322

5244

119794

10562297

514521

3111199

54211187

4221465

44820810
44821810

1

623467

6371132

6130271935

861121178

201813201711

58183137

11520262334

13111

112533

3216301511

2

21314161315

2113846

6161655

42101417

4618271122

681323723

3281635

3611211214

1

239754

627331115

131625

9538211216

3311

21311

4722201310

331281511

436974

231718403016

18235720

2521

2174720

34322

10714192321

11

21272

47126718

322636

1292188

711539920

5395137

1511

391023915

1781196

2241034

17839

551622128

53161738

27598562727
27595532116

33611

6715231431

11441

131

164212173

11112

421033

294815272123
26481425217

31216

154223221314

23291168

2239303

11711116

185158

3828651529074

3828651529074
8515462514

21761

7911271817

2528211

121121158

8211261411
8211261512

11

1051417911

36228350115421303615
12

34627545814421255565
9561119439388181

23

8814191718

42522
52522

1

3531

18753
29953

112

315

326018686931

60202536335960
59202535134458

112152

442131309
442331319

21

9273286

113711

818132

3465

911022388
9110243910

212

13323
13324

1

691836216
691937279

1163

231020911

121

14532321910
14432311810

111

311

4381755

781119149

7498514
7497214

13

651714822

711836167
711837177

11

4342147

11721

1131495

511422178
611523198

11

12

1822
72

112

249211520
349211520

1

34620920

551722168
671924178

11

1221

2361691

11341

3135812

12620611
12520511

11

1312141612
2313151812

1112

371

111577

18144

11

224841
224741

1

719598

1276344412

4112321118

4112321118

12731673730

12731673730

8082076667344174236869263284472899
1184120249695202384213654

148361184177

2316210
148361184177

151228925

11321743042

203225586554792644154870
163128437750403366

191428606828
654976228225110

3224297910038
302226749737

22421

2111

121

9714614428

1

1

1111

1

131

14

2

1

1

81010

121

33512

7742422732

7742422732
5432282426

23101436

22859866831
111

512336179
5124391910

1321

16734474821

302410920211879
109104325560397299

152438562618

11124320

24691

21142666

5284

64719115

8515312311

4671495

3215

11

55472020

4332233215
4332233315

1

5432471824

31185

7312

479484315

73922121

6913364439
71013384640

11221

3453133

1151023710

1151023710

110718704236464521912684
22447910081256545623

11123261518

2

22741

1

8332451415

321744107

204542282230

315879

431524181114

5191664

14846

161622181132

3422331615

321532

155112

21101428

13617

1

224210

32

23631473824

1814114662441

6151023

1

5321261316

42372031720

1

7551681633

351175854049

129334

3361965

17145701148047

151

101841492222

322430812

1

1619282111

241027810

112428331726

1294314

3171195

13416201815

7145391816

931746148

9624211919

9715271223

12233332011

10416332325
8415322222

21113

13917301915

1

371171211

412

4316181414

7413432112

511

61242622743

6338591934

29191131306689

4318231413

11122

12934371716

47749

2392237

111191210

10435451429

34129611

762117516

1

51024371922

171340504030

812848

1412861

31

3141097

2512776

11

1101785

41740521813

51482289

472318810

239244

1

1252924618

222191414

113444

53911276

231322362224

4436391721

732016815

84141285

2711

47111687

434521

8828601339

411222158

332515811

234534542736

4031769544134

116329

331515413

13443

1

2026791064080

1341129617

215317311112

1511661186895
1411661136344

15551

2705583

11

21011

15959743936

11

1032521106

317859

8430391314

12

71026522917

552626127

110129

12

10730832231

7323371616

12426

235440572724

7192697

141077612929

32331054

21833

11730283227

431315169

191782713420

61348282027

213912612

28493

1

11622113

5116361720

5116361720

205100395112300446

20598386100292441

291285
291173

112

531126412
2527801233970

54111726

112211

153

31516201218

71242689

2314211016

1

2625

415515120012373
3525311414

592577

3518401410

121534157

381617147

3819251510

232749284418

112
56391401587783

31114814

9628411113

11943542920
11943532820

11

25727362337

862818127

75202085

75202085

111343423855

111343423855

166052933277

64832681723

101220251554

10119201110
6128202349128185

16430871726

8518421715

13747653851

1411881354583

3420322014

3420312014

1

182689
3179616580144

21251843952

10437553383

9142238626

9142238626

17472

17472

772645823

772645823

7020106175214115
3782310613835

233111812
222582

1161010

1513

1

1

235
41557

2127

34147
21766313844

1

18762303337

32515811

1

1

11

1

1

11

1

1

111

1212

13627
15099385519348361

34211

34211

34674
31814119

114

1

1

11

122
1272

52

111233694841
14292361476326332

11122012113

12329582427

5620442420

7323392111

4811181713

712833

3515974

151062362659

117857

16725331832

2261994

113141186

3311402826
3311392726

11

8822172840

16629232815

21123

21123

8221301821

52921812

52921812

11173359534295286

38211031609899
11173359534295286

1073

13239401836

13

121763

11122

11252

121141562230

421324112

3181052

319744

2114

2

11

3492358

4351372

3

81012514117

16362

2167139

112485

2131977

2211412
2211512

1

7320551930

13362

772418618
772418518

1

127256431113

115
127256431113

914331234
914321234

1

3611311874

140216516776404655
2111221235

64137251284155188
10942771729

742131319

74126201626

172017710

63916512

34813612

3402029917

611120813

6527233119

482114920

111546241621

7478254470237432
15639863258

4711562259

11101977

91231553970

2213191329

141345

6412261421

731736614

4416211413

361715920

385191414

3322241821

7101671228

45723616

4622271543

1112241214

34612528817241461423
523663712

24183217014223

24183217014223

38102515215327
105266741835281

5410402213

34614664910

2641515112527
2641414312127

184

11624

12231

54264928126986
28102312610829

1621

11363

19112012011849

64526354

64187525821685

64187525821685

131662558
943562531445136

35153123419782

46192523519346

2814465791057688260024

2814465791057688260024
45727217112147930

1

1

427310419010513123
39711011899812278

32317845

520295618120

681261894

3252747301413

2955589829835

133168206301336195348

61117551259

12145478291101

111138
626211946321395

4510321410

4510321410

12928683822

12928683822

1122101
1820341869028

91111873711

9822693815
9822774316

851

162315592612

162315592612

114311053723

11429963221
114311053723

2952

1852285038494261393
461429661

567211822093271
33671313

33556011749167
34556011949178

1211

7424371652
7422371450

222

121028571528

20879863984

20879863984

5388161248161696
1741448245239

1315310

23258211178307
24268311481319

11124

1218

382117

1219312

681524972

22412

3274413

114152

221224924378

221224924378

24247512260129
8922502666

81338441746

8215281717

61832522474

22

22

61830522472
61830522171

31

1031591443987
1

91535873056

91534852955
91535862956

111

11

11

1162456931

1162456931

32177418474117
153

6646712838

6646712838

121015712231

121015712231

14112372445

14112372445

5113333929900166151562612009
7495770058326813210377247348185954

7152416503226268
12

4341042440

952634917

3529274241109161

141439544015

9442684435

2097311711792
15334

9636564864

11232586624

3231582061567259

3231582061567259

5833248266137179

5833248266137179

6822231341131161

6822231341131161

4226231522

4226231522

252411019310066
166117647758475439

4119261023

141334471928

1

121424734844

11220312915

19133351129

161463875043

258167848781

8627344128

8745502314

121566462439

5517211310

6722311919

1

9824734217423818531880
1871019841226554507

44221651648384

1617821535084
1619851585387

23533

2108517

44291652219397

1712851085234

26181441485643

1881051376348

1

1

4089282315133189

8021226362176154
8726251387189171

6
641121810

641115810

1114457
31

1111357

6932328546181162

28520103612552113

8052315293184173
8763333313198186

71118201413

1

1

2513991575757

34211452248385

9232356687180218

9232356687180218

4631294389128114

4631294389128114

22616010782816546479
24241441013

97654501456251188

36261071556663

91654971061219215

30318711191778723756
4933223373157115

3214922048472

20111151594771

2716751035937

2013881385157

151046632539
151248642741

22122

4313731285270

1512801166677

131037861927

2315781325357

172199773572

29171111987360

1081053541037224176

1081053541037224176

8635463231460813811511
12538844688625619451807

10440310335159027

422319323151359

188648728537

94635919931

3818220630144566

15555450427216749

698712317813

1817848526314

7523667445407529052380
142965922198953805277277336621

102103542173362627516113223086

333420141814613701873611155

23097176642769412669187759878
161091532814699523294774832

1

3419171012

148161199568695
131141190568489

1720926

4811972142193512151133

122

502171304228

53976396324

59087533261317216

10055505431310312

1642203495613455

36646349219238151

661105625417373204

258127392281250208

259353278315991

120435536186

36485644404448301

1771615310885109

25262251615

42232113246572836313

17420445139139103

15447273035

180290102415637

28046448274356189

1135458214413

49321

19942400458295165

327122

523781734488687346

37252034785169

24447337212260164

1451715811011199

15529350182190109

24846354162201150

8515109234032

2914333516017196

50984362864

197621584738

197621584738

2771069841684774574
7403632959432921421780

6715411219

152012015210255

421820823

16683634641371395

291251626652

30179618311990

388941284467

12544823422

156761135339

41822816

3624186284109102

3516201611

11251975

7233633319

12423113

91043412128

31412138

19101232028474

731521397

1

561832244

28231452429092

12133673317

2211241010

432127145

6379833634

12631219

8840381488265205

8840381488265205

1135
8

5

1122

121150894252
107109406526281267

13746482729

121136703534

31511141548793

7526362211
732433218

22313

151452543522
151451533522

11

8734531420

934822196

4950242265170187

2491569610760

18407212651108

5114421019

212

10681465804296372
2116271417

19181081276384

6938264508176200

1624771424371

379189852310101049681034
2661524817188192607718

2

41625255480135108

112039951011

2813821167146659

1414253893981

31171671819

26134911346310338

642184108019051911531
11813772170362033741192

1

19364354589499202

1

832618129525479

46168510813241

25151041417155

471710611614065

473283224120118

38129111712653

60118612511948

137991121316934280787808260820
221881566226596424199914594378

53435494104511079252927

1302134367191125718

3171186886212211964

7162

10323122192412440697338

94229438483781638

14253722

38032212250492381

47030256208611195

36725969012195156819283

176691007253

3173124

23135736251921351013

319811447047
36111051767564

311723113

217944

1716671545337

1716671545337
1

61018402011
1

1611664

541723147

2721
116491143325

4228632216

74194498

5521169279119126

5521169279119126

12764479733322461
5

381115321687149

5633195352124237

302012615110571
332013116011175

35964

14253
5345225349184201

3220157199134130

2124641484568

767198324413397174311333
2696452086619655642819710

2158441

18412

145842543792

56445910587

41351111

21141471

6837653038

1210481127253

6233198297156172
6133197296154169

11123

16888823958

121

4209101011

271256734067

22271041078150

3135126988177

9712291828

38623426

3

38101544656124

114874474772

2218311208637

8921401825

13212101115

1145

115371353618

1111

13177

1

2628721255356

581921710

9849757634

1

1158713

49189116123127223

29221461319066

203031594272

45514213

2912313723693202

31122102248

4027771095748

123

13960764646

161455492451

3319401011

2101134

17

4158122

4530281319
8647372429

411791110

1610

15131180

154545552440

3289311125

111945604536

1

181450674147

2269122

221544735934

411916169

9605338381428

47676

261817613

51133532633

8111026231

15241988

91530432827

2094495139163

1111747

4825262313

3413511014

1539
2539

1

3730514105

8226181614

103827435630

11

13330301321

1810641025464

269641004654

1131

1048414723151

84116821542

2320112944984

28885856080

34221141156196

195929604153

187364673396

3917961059795

720106666

104632773374

126

291773955770

102569773746

238265

234130424147

1522

12424

8576102

35221282308572

111134323413

7774522024

261812917387101

138581184643

1738185

507410141422810731186

10859773740

2246481093969

122433462234

121252592741

11731010

1241116414

14217271811

221

1262611

711153

101133382134

2481191235283

141661624151

141661624151

10281164317

252076996349

271548115
71515515167

581752

28221151776964

1011413117

1661623821337

172442643276

121695

6571311

8896603015710720271744
36232010754388687714

46192122188268

232854116922971

1920351102017
2220391122740

342723

205751214628

6544633524

3792309691905860712
3511948911752786635

2736761537374
2836781537477

1213

3133601316187

7976691213358624611384
52417025512153

8613382828

8613382828

548181115

548181115

8484171484218155
2427791678042

181017934234

117874

122030763322

111013582420

4141644

7716462115

5439611

2421113

6812996283179100
12171230283

101324542912
101324553012

11

657412318

102110462713
102111462713

1

255320504430
255622554436

3256

51720562718

52278020424183
2901543459681017326

201228796016

161631634736

19932343644

65393428834849
55352825730842

104631407

18121294316

211136524017

121332332322

141014582814

53163712815129

228392417

228392417

15519564036

15519564036

9188175458294298
385411635

17921915985
11511854932

641061053

111235703933
191641805149

846101216

751024229

2630441027658
191640786324

7144241334

6736765025
111240846142

55481117

881436920

46255120611178
15516483124

9614352122

651049144

824322018

443973

32222177

112111

32112124
27233915110670

6217694514

181921704952

2657331205243
8914312013

241537

3274

11911

1111

134510522419

1152

30226921610767
11242

625814434
7125864435

1151

131129643118
121028603018

11141

1

1

8913612813
9913623013

112

173
38247520411149

11529753018

2718461227831

15541904249

15541904249

12475523835341451
1

6415171252

6415171252

10251434716287334
1

8343553349

4415238287129146

10824852823

9757853544

239501424441

8921621831

151770933560

151770933560

134974

134974
134864

11

4192242741
383793115925118572955

38565114651137
1

72530813077
72428793076

1221

313121642160

313121642160

83132723670

83132723670

52162162377129316
26362592319546732018

324344

543355621116
543355622118

12

41013503140

242156552779

51638633555

7924402430

171346423869

71411481728

2023651383580

42113471344
42113491444

21

121669682959

51632502178
51732502178

1

3337621

1040371142681

51636481851

71627751329

133152722658

1692610101

10472114238117

8625401934

1210311131470
1211311191470

16

61121641288
6920621287

2121

1131054

112022511852

1114143118

11344611937189
10344511437175

11514

581425

42359617859607
1628558837116

42174616158

1126425

1737231172

4115151136

242325912076

242325912076

441028116

441028116

343969944860

343969944860

112315311316
343969944860

6289912

1352

4161622

33728

46381410

228223

1113712

3310955

41610231627

4166131010
131

2414

1332

1941

11121

11112

52

410617

1224

35411

32

16415023698136165

15413923293124161

15413923293124161

15413923293124161
11321

98925513

13812220655106133
13812520855107135

3212

6512131012

101145124

101145124

101145124

101145124

657412517395122
22

617112016993116
112

4659961337397
1113

11224
61723262120

167549

11

111221

83531

3181089

11

341711114

341711114

41818418

41816418
41818418

2

11
341436

32213

7

1

122

21

1

241116414

241116414

122234
312933472932

131114161212

171617291416

12923311716

12923311716

12823291514
12923311716

1222

321413

1

321313

433422
433424

1

1

1

190152115454346538229513406243879
26061909767107824561157

4313994283135109
13141

424298422611795

42667115

356592

1111

246482

121223

121223

3213

11

2212

851243115

21532
1322

221

221711

2151

1121

2457

2457

2
42285

11231

121

2113

2234
412244311410472

42
1152

1

1

1

2

2

11131

11131

1
574

2

571

375212423
242121311

121

1

11

21

2

1

1

1

33

2

111

11

22

11

1

11

11

1

1

261028654332
522201816

1

15

1123

1116121
111161221

111

121

1

54622112
1361

44316102

134

211342

1

1

121

111

3713514226

53514226
3713514226

366

2

2

311234

1111

113

2111

61010261413

3611

11

261

4441104

233123

21181

1112

1112

13121

13121

153
141

1

11

121114

212

1112

11121
234219379437247332

53101114121127131

132232
53101114121127131

2213

2213

1

1

22

22

21314

21314

6177666
13121

16111

21

141

1

1

31112

212322

151947535142
5637283934

1

1

2

27

1

2

113122
113132

1

78111

221

221

12

1

1243

322
323

1

1

1
2111

211

422022
246054496272

235345174763

1

12

5

231

1

1421

111

1

1

5

111

223

111

111

11531

111

143

143

172120531433

172120531433
3

57618810
12

411612

734

163234
164344

1

11

91183415
11

3

11

3441110

3441110

1221

131111
171113

42

1113

1113

2261628

2261628

16396244261106167
13

11340891025079
114412

233151663248
431213114

2

13

2211

13

171

2711
2712

1

12

21
23

2

12

1212

21

11818241234

2

42

42

37312

2

541

3

1

72412

72412
4

7212

63521
3121

111

13
12

1

21

211

211

34117315
3622

15

3331113

11252
44656

2231

1113

44141054
1112

312423

23

12211

49561521595688
365974

651822116
201231312025

23353

10783415

2221
2231

1

381425215

381425215

17

17

3372478

122215

2152263

43151046

43151046

782910514
14214640927

3521215

21832

255422

1611
1411

2

1253

1253

110932

110932

1212

1212

1211

1111
1211

1

111211

111211

112
273547753838

111

11

2221

2611

14325

311

512

1

21972

1113

231

12

711112

11

31

1411

11

2

14

111

213

111712

131

163

411

1

21

14219

41311

1

2

11

311

124

241

17111732420

17111732420

17111732420

17111732420

17111732420

136858106396253976776711756632406
799232119

2114

2114

1
2114

2111

2

43621191445992
24211

15714

15714

15714

112524

1133

1133

1
2221

121

2

122231

122231

713192158

2

2

3271031
11351

1

1

2

11411

1

21

2111

2111

211

211

2234
257825

1

12

1

2

113

2

21

1

1411

1411

2247
3241891084874

1121

1121

121717271016

815111979
211617

16331

11111

2

456951

1133

1133

325534

325534

354414
11

342111

133

111510210
18815

2

524

111

141

141

134633

134633

35452
1111

1222

1232

4832181726

4832181726

115411

115411

6342944
23434

346

1

117

1

1

5398310

5398310

11

1

1

2

315415

3

2422

1
142439603718

61719302410

3613

3613

1

1

2372
6171624227

1

1121

2221

4212

52111

59410153

114111
872030137

113443

113443

111

111

31111
11111

2

1231
3371021

21431

11141

31341

31341

111

111

40533610714125229711122
136789106298252196753111744532263

6225453511736430076872277
4131831551719

211

211

331904110713734

331904110713734

12471

12471

21
5113

11

22
212

1

1
10322411

9123

121111

11

11

43911011

1297

11414

2231

313956
31674779

14

13

1

1

42

42

31

271

1112
121122

121

112433

112433

2251151093
15533

1

211

41

184231

7

1

3152

1331

1331

836048601245348472291932
567314

2221

64212

11123

232

79323

1211

234211

1146

112411

4

4

2214

121

2322

11422

111

1

121

2411

112
770014243

770013231

1131
1111

2

114

122

4147

2431313

4312324

231411

113132

21

61647041182341671951888

1111

10705624201024
131

15

1070151610614
94325136610

1269344

21

1

11521

11

41

111

142

122

915111263

11521

5128632
6138632

11

21311

414257
94991817

1211

112

232345

381
1

38

21

361211

361211

124723

124723

4253529521014352
7562564

4063114

4063114

41

374721126152124
1

374721026142024

11

122652

1

12211

3571164

36752

211

311

211

1

2

34345

2211
9853938

12

9831825

2

11

11

661971
762472

31

12

3687176
41416252516

1

1

111

121

1

1531168
11

1

2121

11

222

1

121

11

3511

11

221
11

1

11

2

63

63

23
21

2
1

1

239
24151

11

3

12

3121

3121

52881562106966
71132277

121112

25346

11

2112

227312

1

1

3

1411

1322

511

29352

1211

385233

411

123

11111

1115

25

27

7311

2168

11

111

2

112

301

5

23462

1

1122

11

3232

1122124

10512

132

26112

21331

2

22

1

2

1192

1

21

22111

151

2212

121

1117

1111

321

11

11

5412

5412

12
12321

1

1

11

12

11332

11332

1144

11

134

224340793844
851322717

1

2112191

3

131433
131434

1

1

1

1242

2

1

23

119

321

1

1412

33

252

121

11

11

111

2221

221

2

1

531

1

1215

12

11

1

1

1111

12

123421

32333

1

1

111

111

11311
1382425915

111

111

1

2311
2412

11

218

112112

2441
61221621

41181211

211

12

12

1343

1343

321222

321222

61435

61435

22

22

421

421

421

7683109

7683109

7683109

23641

23641

5420236116238128115
213852

2911

2911

5864481315557
12292

121

1991094

14

131352

2112

131

1021132

1

1

11

1

2184

1

21

121

1311

121

1

21241

1

2

4

2310449

3121

3141

31

112

511

1

27253

1151

11

111123

2211

311

12

142154

11

31446

27

312

1220

21155

121

1111
53153714291910

1

2511

11

11
5311351021147

5310351021146

12221

11

5

5

1

1

1

1

1411
253812152120

121

11

1

211

1021211

3163

111

162112

1121

1

1066299

217

1661254

1661254

1531

1

1521

185730251916
3315742

1111

1

3132

1

112211

23482
5336897

3306415

1121

1

12

1

1221

2

23233

23233

1

1

4264

1154

311

243048536219708547519689925555
3111821

9422854

613
6113

1

1111

222

11322

111

312

121

11

11

114

114

12

11
12

1

1111

11

11

1121
122323

1121

111

252316125720
11

322

25231595418

49265

49265

1221

1221

111914

111914
11914

1

3832219
121

11122

111

18

412

1

112

1
11

1

3934106
1121

1122
1132

1

1231

1

3

3

1113

2121

9103543

331232
9103543

31

372211

42

41

1

242428529019668546249680925489

122

242428529019667546249680725487
242428529019663546219680725456

1

3231

1

314191

133

2117

91

9641113
29522110872101570690712508

101314284
1111

7121152

7121152

33121

8371827213

121

1212

7361427191

3135

3135

4271051

1821

1821

1521
1421

1

3122

16352

16352

32164

2132

11132

14331

14331

1133

1133

312442
11341

1

2111

391

391
39

1

1624

1624

312

212
312

1

16221

16221

151

151

2121

2121

3522

3522

11
11213

12

11

1

1
93158138

312

21214

322

41813
418131

1

32261

31571445

31571445
31571435

1

114611

114611

4204160984262
23284109691991549989642444

5682112

1

12

81211142423

1

124112
224112

1

221

212

131

111

11

1112035

22

221

910223
910222

1

5

1

1181

111

1

13121
13321

2

12112

1

1

1

2122

262

13134

322

2114

213

11

2

113

1233

1222

12334

13

2344175

331

172231017212015
172241118302217

1221

1221

1

17

17

1

119183

61845

3

41

1

18

115

662117

2281847

1

21161

23751

11211
11311

1

1435
11122255

18195

1

735397451711

1008100851358415480481914

1121

121

111

222

39522

1

1171

51441

111

1334

111112

21

112

1

11

1

2331

21141

431

1

313

33

31

111111

1122151

1131

111

1813812

2

12211

23

111

1242
1142

1

15162

115

53324

101201

21

4438014
41438014

1

1

1

121

1

110

122

2866474148458488178

2111

138792
85847861706653

2

12

52121

11

1231

19

232

192

2212

1

1

221

2

1

1

1

1

11

21

11121

22221

1

11

1

11

1

131

32

12

1182

1

4131

112

1

15

132

211

125

1

11301

1

2

112

21

1

11

13

36

111071

11

221

72

1

79022295

12

2521

3

1

11

1

11

1

221228301125

22

12131

12151

11

11

2

1113

331

351323110310434

1

111121

116
16

1

1

233341

11621

1

111111

11465

111

11411

312

151

211

1

1611

11014

83192011

32311

32311

141241

141241

111

111

2112333

2112333

1313

1

1312

191

191

12012

12012

141275

141275

1112

1112

616437931

616437931

114
426752

1

211211

32

211

2

1
294819351411

133231

133231

817782

213211

5214

11231
12431

12

15114

15114

28344445

63444
28344445

277141

37251747

37251747

37141747
37251747

11

211775

211775

211775

27618201119

27618201119

27618201119
31423

276156916

1848158811468547

6420731715
11

31

31

122
24532

1433

2112

119124

119124

311

311

21

21

611

1111

1111

32

11132

12

1412

1412

554756

21363

21363
21353

1

341153

341153

7010642494320

1862821208
536742

4111652

2311

628693

121

22111
5210014282312

429471036
429461036

1

2451795
84617205

6111

1767431517206

1767431517206
115333

11

11

176641814172

2529207288539210195
41015491410

432

432

1

1

11

11

1

1

651

651

159543571092940
27617

12431

122

11
1

1

127512

124

211

144

21221

155536

4242

51331

2321932

2113

454333

1512483
1412412

17

1

158254223
1

158253223

2111
4212

211

32411

32411

1

1

1211

1211

31321

31321

1121
751621716

13221

11211

11

132

11445

22

1113

111

112

211

13211

11

11

222122

21

21

1

121

121

11

11

113

113

522
13311651012

76134910

1

12

11311

21137

21137

7113466

7113466
7112466

1

12311

12311

8211525631216
7975101126

114

14

1

1

231013461010
184103675

1

563913

112

11

144311

144311

4449681617256
26534611

2141

11331

111
1

11

225

33153143

6517151420
6617151420

1

11

52333

9214
144135

5231

1

1311
162215421213

1

3

2

1

11

1

11321

13

1

1

11413

111842466

11

1211

13

2

1152232

241

241

125

125

2134

122

1112

13211820179

66122

761211

2531422

241821

1

261

116193
116163

3

332231

1211

1211

118657

421

114456

233881
12351

11

12

222

11

11061

11061

2
5512537

1

121112

324114

116111

2521

2521

1211

1211

121332

121332
22

1721

411

1211
622259102136108103

112
259683

144211

1

1

4132

1111

111

111

42325

42325

63

63

10326273535

10326273535
2112

8325273433

977102010
62043951966354

3711
618236735

61823324

11

211

212

12

111

11322

2232

11

11

1152
101720222418

451

1

22111

1681

322

1

223

2123

1

121

1

111

111

212

722

710

122

11

111612

1

22

122

1331
1231

1

11

123

1111

1

113

132

19121

19111

1

412

412

21341
1012202569

12462
811172068

11

527712

213311

1

31431

111

312
12

3

11

11

43342

1

1

62131218

62131218

243167239325217249
1315139116

7710227

7710227

4425163
1381528438

1

1

74711185
74710184

11

128
118

1

13111

443942

443942

22154

22154

24642
1

2332

2221

11223

11223

2
331412251425

11324

214

2356657

21153

211

351435

11

21351

11611
418312

3221

1

232

232

486061813772
64141444

51102

13213

1434

1321211

13321

332412

1131

1463

3121121
3121221

1

1113

161421

18131

211

11111

1133
11313

1

11141
1114

1

2

626453
726453

1

1121
2424111

231211

1322

216521

216521

8372415

3261283
414151995072

1121

20262233

1

7

8622212747

1231

11

12318

24224

25511

141

3822

1112

111

245

1333

73212
26532421826

1

19529391724

49274147

49274147
1264147

481

1446712

1446712
14512

14322

22753

22753

10203081121

10203081121
2111

919211519

1122

16421

1

491676739781012448135149707
10192239171215107

17372143376310192
2902045435207880692176253

314933
1912137135192417168

410248

211

48237

111

111

21734324
1905126128182410156

11121

2

11

1211

263642513
7542513

2561

1

1

3

3

122
1123

1

1

11

161

111

12

1

1

1386104616934878
1636113110131388105

243244325

52

12213

12213

121

213

2394

323212612

1

1

11421

13

2

6919711724

125033
40

222

12811

3361482

3361482

2564512
35117619

1

15217

8383155146104128
1

221221
6851074

223232
223231

1

241621

7161717117
4

248366

382641

24381

6356117
1

22454

432263

121015251317
635612811375110

164333

1713275525

227828

43121165

278893

524742
524642

1

1

1553

1423

1174110

459649

7112472

1111

344537

119959

1241

233

13512

327648498635874662

133693

2
133693

1

2

122

1

1

11272

422

422

422

326645495625863657

167355263377468367
326645495625863657

1

106190156172286230
106190158172288230

22

512133157

131

11

16333

111

43331

111

11142

23

1

212122

1

31568166

1

2

1

66131

163022233127

11515

1421

211

22

111

1

510210117

82121182

582013251967325928072306
665510711787115

163242
61326292318

13422

1134

1

1

1121

1211

11524

211

112

3

3412

2

2564

122222

51316151220
1111

122454

162531

3312

1

5

23

12121

225

113

211923

122

13

121

122

17254
51

221

12

32

121111

121111

21351

21351

1111

1111

1
1718844

31

1223

11231

1232

31

11
13552

1231

124

321691113

321691113
322443

145710

2311

2311

261850895964
7387107

324

123131

11

11122

1

1644

114

1

713311817

8614111115

3143

13662

1

1

1

1

11313

134

11

2141

2141

77917116
12

443

4154

1131

211141

13423

132

132

315332

315332

21662

3

21362

291061422351
326514

52

112

21621

3792

221

31463

9371619

11111

1132

632

321

3264112

41418

152823462218
1181046

1

1

1

1

2

1

82253276

1

2

11224
11234

1

1
8211962

8211952

1

2111

14232

1

11

1

111

1

11

3131

31622

31622

182225
132

112

1

11

3

211

1224
4335103

1131

11111

21221

11621

11621

243353
2115

1

2121

211

271
26111

244

201829531821
46534

222384

117613

212

11242

224211

54123

2372

315932

121222

121222

11131

11131

399521179
141

81112

211

11

421221

2321752

133

32242

12

1516227722

1516227722

123634
2

1232

2322

1121

1121

3
4546813

114

234

1131

1411

13113

64713117
2222

2321

21281

151

2333

176148208408179184
53178861356251623481796

11

92351419

14151

13111

11

11

1131
4131

3

442

311211

11

2

412

1211

112

534754

18

31241

1301

12

1

12

1

1241

116128

321

131

431103

4337206213915675
236533

1

1

11226

12

1312

121

8311548535
5310548535

1

1
3

2

12

973305722

431033131
43166374167

2

132
653

2

51

3343333
3343313

2

121414

68631

24

31

91583

1424

83

62445

21

287

24221

1

24624

232111

21

13214

213

11

23

1322

11

1732

11

1121

2431210

21334

1213222258

1

2533

1

244

1

23

131

23333

124

22

1212

2997

1

8127228

30

3414248

1

22821

1223

3111

22

214321

421

22733

314131

22

1

31

324236

311

1611

91

1841

2330133716

332

21

211151

4462185

113

6133
133

6

11332

5311

11114

221

11

2142

223738281651
223738281652

1

2431

2113

663163

11

11

423732

234

114

736171614

13161

11

1153
61153

6

623452

11

2221

112125

11

12211

12

4

6

131

211

1123

21

4572112

1

1

1

31

252

2412

11

1

26724

1

1312

1

1

211

6489310

268310

268310

6221

1221

1432

216542

1

1

1

1

111

322431

22132

13118

11

11

211

6

1142

11441

1182

22

1124

53335

3124

31121011

213536

2

1211

111571

331

37165924

3237

15132

1

2

3332

3434212

17

4

111

11211

51

1

1

1

43

2

1

1

111

6

1

1

11

228136

21

1

1423

11322

212466
112466

1

4123

212321

341

65163315

224371

1311

1

235431

1344

73345

1411

11

217171

1222

31102617

11141828

2

2239

448510

1

11

32

11163

112

641227

11

12211

128

13

21534

1

22

111214

41242

2152

71726304121
39531537010221586928

50555778194117

3332107
3332108

1

1773

362735517462
342735477360

1

1

21
1

1

1

1

21

1

5118233

13611839749

1193

2211612822668

14

1

253183212604932590
256186212605937594

1

1

1

1

1

1

111

221

1

1

12233
12232

1

2101355

31723

1

1121026

2212

12121

1

11828211

212

2617713

21828311

1142

1311

11

13

913411

22

1213

21

112021

435237

1

11111

114242

211

11

331

2152111

32

122

22231

14322

1132

17191

12121

1

134463
134452

11

121

2457

1

2231

2711331

321385

1223
323441

11

1

1

11111

15913161298
246842

149

1

2112

2811

1

1

5411

1132

1

1

1

12

12

251121

24111

111

84510164

31122

31121

11

1141

21351

453747

453747

165763

42361

12342

46313

46313

448213

448213

54151099

214

347558

841

11
122923

21

12812

5701618411
4111839

1

11511

333

21

6321

3312221

11

3292

32

61264694
208380813427

22112

11

3211

23119

12311

12221

67312

1

12251185
12251183

2

11

1431

13321

1211

12

3

536

123

24

1112

1112

312

312

11443935668179333261214
311633

1

1

12315
124110

4

1

1

211

211

132446112
12341

91341

12

22213

51618141311
13334

11222

1112

111322

11

111

2112

11124

212

1

41

5241233
31241

61

112

111

211

11414889637175232861195
29794608961404362

1

1

1

2

1

111

3

1311

1223

22216

11

4423
2211

2212

755494220
754463920

1

1

2

1

2

23

131252

84172184172325
661611286954

181117132720

11

1111
111

1

11

21

31231

111

1

1

21

1

25316186
24315175

1111

11311

7268126

12

2

11

112

112

1

1

11751

1

1

1

11

10820420262406686439
10929517428472855660

1

1

11

111

1069316463167220

1

211

21

1

10188426

8

112

2571094

162

121

91554

1215

15156112
15156111

1

1

116
1161

1

1

1492454822
17112536025

11

1

1

227112

451223812

11111

1

111

11121

281773
173713283015

152912212312

214111
17

21314

61213324

264442
79163167266141134

1
61063745

233111

475634

244224
613102077

36482

1243

2

12421

4310867
175330324222

143

2

1

111

15321

16

11422

271

11

242382

1

1

515

1111

11

12153

2

24151

81818102112
213

1

1

649372

1122

6212

2363107

111

111

122
492011611

122214

3412636
3312636

1

2431

1
255544

2222

1

1212

12

11211

3
398511111

21241

33121

414615

3111

11113

110753
15241

311

251

233039383142
866689

12

11141

244

11132

123

1541

21

112

12131511813

48427

11

22112

142913
5104435

1112

3614

1

314223

314223

333516

22232

111214

1
151953682940

2184
11735281318

224223

1147

5312

113712

4221
324354

32133

4171

321331

1

321321

251121213

251121213

2356118

23174

5544

910010601239191310771242
261012531014

1331

1331

9661244

13121

8351223

11
334634473233

224445

221612

14211161614

6561425

51181232

22331

422233

20711217119

141213

186691084

112522

151124362154
145752

13676

3234116

831017426

223244

20930341415

20930341415
36423

25243

1

1112
1122

1

2261563

117422

152552

1

2637301311370204303
8903774104615659301040

398127

13771

333

53715126

11

2114

1221

11

35843

13533

35533
1111

25422

17

21121

11

81211

1116222062239

32351

31612

791341019

212103

21

487939

212613

1

21065526031

11

25101

14

114535

11

11

332511

1221112

125432
25432

1

2

42321041

413

21221

311

1

5154516

2121

11431

3

12

105126898

2112

1613238514062

11111

111

691061217

52211

234743

2314

163

13418

4

677424

325764

5151

1223

98203268
98203368

1

18612

4231

7910210

12212

723516

1211

31244

1992554

10112251824

452

118242

14129926

56108415

336565

36629

145910410

91121

2111

2

121

31323

41

1221211
1121211

1

5

11123

331

3610126

1553

1541

1412

22432

353104

44324

737314616

248

31

33411

11

11552

21514

22824

91652
152

816

42572

301

11

632

2172110

1

1

11131

117112

4453310

521

16211

124

2361

1221

11

1

917567

7111

1

531813

21

117139523

419522

1441

3313552

221

172433

21313
21213

1

71

1331

2224

233711

2112

2122

111

1

1

629142

11

628596

121

111511

211312

3271072

410

210147413

21112

2123

221

321

113123

2161243
2161143

1

112351

11211

20112

6654310

233212

11

122532

5261022

114633

532922

122

4231

210831

507727121997

11
98617157

88616137

11

13

11

12464

441683

11

111142

113414

11

1118

11

689510

51224

16

2424

94823522

13567

23315

345623

111731

1217126217

3251446

21518

1

48822

21

1121

1121

22224

119613

61024

342422
322422

2

111

161

11

8128161112
65126601344757

312

11113

424411

114431

1524

6141930812

3

1324655

7267313

3521

32241

11

8336411

21

19531

331761

961212815
21

224352
224362

1

7487113

629216387115143
7478713

134333

134333

7823141834
3461710

5224

523

23122

7

2442515

1

4822

4822

3

3

1
27125126

2211

5115106

215982353361

215982353361

2211

2211

2321

2321

21461

21461

1116

1116

23126

23126

64851311

64851311

6385146

6385146

1

1

11

11

11

1912821742563347140823346
4078923252316

1220810311413174425381972
131

1218910171374171525161932
384466157285354300

2

111

15348

24342
242152

17

11

3313

221

331

2

132325
13423

1

191

132

2

2

1

113

2

2

2713288612353

218236911145
2210278011650

1241155

41553
531673

1

132

1

1

11

11

1111

18101

1

28632936613873

1

28632934603772
101621562

81130362969

81130362969

284522921

211
11

11

1

53

21

1311

1152

211

21653
21663

1

3

4111

4114223

1

7510128

1

53615144
53414144

1

1

1

121

1

2

11

1

122

1

5171322219
82713263010

1021

6

113

21

13

311

211

143475778375916201140
143575778476016211149

1

11

18

1

21211
1054315131

10541312

123

21

236
136

1

1

1

31

1

121695781462
397025326199228

274914717583157

511829

62545

21

11

3111

53216278
53216279

1

11

1

1

1

1

1

13

1

1

2

1

111

2

23

1

1

1

1

1113104
1113114

1

2241

582219131017
2857420161117

1

22752121

86315525

1

2

181238242237
27756

61352

42211
32211

1

11

1162681027
105255825

11
111

1

11311

31

21

1
1222

1211

1

49579313581123
2533

4211

4211

215625
14412

11

221

11

31

31

123

123

4222981
1351

1111

1

211261

141

141

11431
1

122

1111

311

311

111

111

74813104
183435424261

12

111

1

32

4

1228

323

102916222741

13

13

13

12928281734
1121953

87164626

11

212

111

45

3

2132

62107311
41102110

11

311

11

11

13113

13113

211

211

112

112

112

5834401476238
34

321028
211

11515

1

111

112

101514964617
1443981

1

4121853
44528124

331071

331071

113

311

241641

1

1122

1142

234157

512
42128261210

1

4152

21

11

38221

21122

111

2221

1111
11111

1

11

122

11

1

1

3271943
11

3

12111

271521

662032352335
8

115
14

11

122412
2212

12

11

2

2

31153

31153

1221

1221

2121
137253

1211

11111

11

1

2

66104815
591220151224

2

11

1

11

31211

515325

141

1211

1

1884741525916838649
11

1111
71313441021

253

13

311

11

121111

5262437

3171
5262437

1

523636

2212

111

1

131210

131210

1104679843681
1877726509871826617

1

1

111

1

211

22

31421

1151

22

161

622
621

1

2221713
186325013

1

18433

1

1111

11233

161

111

113
13

1

14
12

2

1

11

1

11

1

11111

121

1222

1111

8232362
11631

1531

811

12

12313

1

21

2

1

2

1

11411

1131

122

171

211

5676023547528
5716724558032

313

1

1

4351

1

1

18

1

111693116
111683116

1

978266

11

1

1

11514

11422
11322

1

32

37976
37676

3

2

1

1111
111

1

1
31

3

93

132

12335

1

1

131

11
1

1

1

11

1

12

111

1

1336

1313

28

12

441194811112475
12

441174410911873

2326
1326

1

1

3

3411

1121

1181

2114123521

24412

11

1

121

1111

318114479

25137
1

25136

1215

1123

111

13111

1221

71

212

33212

12

42

2

123
113

1

1131

31
23131

23

1

2311

2

1411
2421

11

3125136

13122
1332

9

122318

11

11

12

1343
1333

1

122

112

121

1

21764

1221

11

119
111319

1
2

1

11

11

1

13511

11223295110187149
11423296121204161

21111712

32117339014546
31117338814446

1

111

11

121

1
141

41

1

11

11122

121

11223

11

41

11

35411381317
51351315

349132

491018193

213

2224

121311

1211

1

14111

1

12
1

11

3

142

11

121

2531

1

115

11216

1

111

12121

1215

171

123

41

2112

1

1

784282437468515513
111

13
3132518

2241

11

111

21

11

12222

21

1112

1

21

21

11421

1421
11421

1

1221

1221

21151

21151

468712992111122
776277428434503500

31

1

211

1

111313

137109154182222242
137109154182221242

1

1

223

12311

435248

1146103

1234

11

121

221

2

5481880609884
291878589180

1

1

51912

2143

1

111

1242

1

161

32411

1111

1

1

1

1131

11

676571
574561

12

1

12253

2

11

22

11

11

11

1

1

111

3711

12144

32221

133321

1

11

23

221

11

21

221

221

335113

351571

31

161

1

1

11121

4

121

2831144

11

313679865159

313679865159
4111020712

313

22

1311

11

3522

3332

12

1

21123

116361

1

111

111

11

15126713

21311

1

51

1

1

235255

31214

3311

1

1

211231

11

112

1111

1

11

12

11

123613

4110735

7817063895151

7817063895151

7817063895151
54333

11521

11521

12

12

51
765

13

33

11

12112

12112

3941
321

13

5

11

1122234

1122234

131

102222

1

222
155754

121

111

1

1214

1121

112041

112041

1121

1121

4211

4211

2121

2121

1181
749716744

74656512

111

1

11221

21

11

11

123132

11

122122
122112

1

1

1

1121

1121

11

11

11

23312

23312

1333
12

11

1

121

13

13

102321013
1

2

2

10232713
211

10211613

2311

2311

1223

1223

2641012
1441012

2
12

1

1134

2

1

1122

23

23

31615103

31615103

3161593
31615103

1

173721732879374018462218

78101167178115114
172821622857370418322187

21711111
3404127811004429576

4123211
7411216623388151

14523
221464571948

831135622

43623517

451215

52421285
111131

23741

113332

11412

6552215

6552215

332313

332313

277872

277872

277872

3892277

3892277
231041

1321

1321

73614

41

12

132
156530582325

5114288

5114288

547101625

547101625

322214

24581411

114183

114183
12

113163

3693759

2443434
3693759

1

123225

11

281844563258

281844563258
56714210

81

231127352745
231229422948

1

1112

1

1

2

32

131859392218
264299608760340414

122235664745

4913121716
122235664745

1

23131011

4381854

43634

111

3461399

421

979620122395141
412522

8561448

8561448

1
334279603958

76519911

192634221438

783619169
794019169

14

257585
362465903327

12525
12625

1

1

1

22620291211
2062028107

2124

1442

962446103

154412

114311
10102332224

1

163136
63134

12

53105111
5295111

11

36115

61426221522
151155

6817101011
61217101012

1

4

1315
1415

1

3961021

3961021

3961021

331652622031

331652622031

23124

313

1981412813

13161425

71141864

1441722

3212
91111208186101147

233259512912

1326381914

10621321512

752919
810323822

198

3

15415
15545

13

36241222

36241222

241411912

241411912

251347552054
821417814

41

33423

131111525
131111524

1

2133

12514
85151539

75131025

321122

87151261
86151261

1

2101110137

1

1

210119137

202932101335

202932101335

7410108202

7410108202

7410108202

82337663329

3374526

3374526

3374526

4915121513

28378

151432

326553

1111591610

1111581610
1111591610

1

61352552120

61352552120

61352552120

61352552120
1211

382423814

352730125

5671711436167
6518488541177568748

81131666
484892766547

915146184

6491111
915146184

3115573

262853472830
87122012

1

23333

1

11

11131384

1

89183615

161243
151243

1

555453
135453

42

4312727

4312727

1111

1111

2319348

2319348

2319348

43131835
242362209370155229

4101311716

146132

344

2710410

213711

213711

419914
141534551724

1

1194

4612911

13135

2

11

17233

31

1242

31

1341113

13102320930
232

67161579

5343221

10918622218
205323123259118153

1111

11411

132

2

43616

1

47105236364

1152118

2131

37613
3741

212

11811

483819114

12

116739

1025974

751968

4436

111023261322
151127261522

214

11

1

1

1

33116

5681726
1181225

541

41

448461562

13

6342124

61

15731

2752426
26522

1226

51181313

51181313

51181313

33624

33624

33624

283160944089

283160944089
12521

1041823724

352542

1

77915316

5101471835

151719611

941481202159682
2281522

241
85117601308340

461493

732

6610424615315
25201725913

4184736442

85121759

76933108
7811331012

224

72952701140

72952701140

72952701140

11012318319171159
35721

18188238

18188238

10611516017667119
7122043326

191171038

56171159
56171259

1

346633

1319

2561932

24234

2612825

6227855

4559413

323785

81322191116

1231827115
831019115

488

2512359

1

1

1

43323

43323

43323

414743
595876647357

101

1

1

10

11222

11222

113

13
113

1

272640153221

272640153221
1271

1112

252432142921

21023

162

1421
1321

1

76424

75423
76424

11

6251310

425108
625118

2

1

22

1111
221220211513

201014121211
1139266

9751065

116822

121027441112

121027441112

121027441112
1

14112555

116161867

27253239819

27253239819

2220223167

2220213167
2220223167

1

55108212

55108211
55108212

1

38371101147555

38371101147455
21

2410261

21

22357

863023168

21

121336403224

2134

13

52191869

2631012

491

11371

111

1

1

1

5586917551011580592
478759

256312257327277196
2761352

230292208273236178
332039445526

33756

1251731

1112334

11

3

12

1431110

1

1

1212

233112

3111

28541311

124756

522527

10

13

121

1011185917

2322443

121434

31438

115312

1

1

1

112

2235410410

1

11

1

2

477719493518
3213836

1

8743349

34671525

2

1331

11

112

3111

23152

2323

2241342

1

11

10113

57151152

72816342

1

2453
2413

2

2

2632

13

3

34362

1122

1

2122

2

911

571112106

410102086

6222197

212

212
112

1

517522
31342

22

22

221

191324353214
242224

221013

1018843

2571535

335102

397727742930

397627742930
43731410

1

1

47223

3

1

1

21

1

412122278
256518382216

11

132

1

1142

1852316103

1

1

11

1

1

5311317621

425858
5311317621

25513

1273410

483711013559106
2

241859793048
44715413

461611512

238249

315621
215621

1

4212521

6231043

14122

41879

71623912

71623912
71523511

141

12157934

12157934

147161956
211020261112

34542

112

33212

133140284356175193
13613

51410710
8387202254110128

67111247

1871163

2773454

716162655

103261588

417102514

847845

6823201518

1342713

8425151114

782118918

811117310

107234179

126983

126983

171522582119

171522582119
111217441312

6351487

263336182527

263336182527

5315111013

5315111013
431510812

1121

656543802118

1711

1711

1711

656542732017
1

331713

1

1

556140631311

511363

8221315819

8221315819

62101475
8221315819

22031114

152162712138
18415

18168411

18168411

18168411

182

182

182

62132634

62132634
1115

18513
18913

4

4141221

8917311318

14711114

1234

135874

751020214

751020214

341711413

341711413

341711413

33154410
341711413

1273

91122361431

17829

1

33

33

1

117

2

11222

2345110
91015281222

11

11

131

12131

4761467

1

1111

122

64478411041383817830
271936633020

191636421738

13122
12102417827

11645

7510211

325555

1112

142

1
7281045

122

3211

22511

211

1

11112

1

441556

441556

2711
441556

21834
42845

2111

86118155155117134
11

292842455438
8414978

2

12233

334464

11

1111

411

266425

333515

1

521

2437104

3142178

21851

1

57901121096396

57901121096396
14211

1091328158

12131

212311

2421412

214311

111423

31135

12

1314193934

1314193934

1415298321

1415298321

263446

263446

3421221

3421221

17194

17194

721661010

721661010

113311

113311

17535

17535

18
5271929119

1414

3226525
3225525

1

249554

101022342618

101022342618

101022342618

3471422

3471422

7615202416

7615202416

304662844576
223

131112311415

131112311415

131112311415
95713911

33922

432932

173550512958

173550512940

21491237
173550512940

1

1211

12

1122

1211

1151

141834302028
141733292027

1111

21

18

18

18

133475

1

1

112373

211

14141226810

14141226810

14141226810

14141226810

14141226710
14141226810

1

191437291836

191437291836

191437291836
1

591212710

591212710

14525161126

14525161126

11
519758948362

13105

13105

13105

13105

428747707153

428747707153

428747707153

21
428747707153

314428455739

294225445337
314428455739

223142

114319251213

114319241213
114319251213

1

77111469

77111469

77111469

735713713011297

232131295830

232131295830

232131295830
3112271124

810513461

124915

124915

50361061015467
12

191234262448

191234262448

191234262448
213421

1282591342

1

1

5361394

22
302472752819

783731139
111

23201693

23201693

55161436

55161336
55161436

1

231633421510

231633421510
3571152

212121

447842
437842

1

122723

829611

525911

1

1121
314390546722354334

274321478652302286
111419441311

3211

16

51129713

22

2112

16

84125392513

67342068

2221

15

335

313311

232331

11221

1171

31121

1

511

2

2121

111

7

21412

31322

11

112

11122

426542

28268135

1411

22157

336765

3311121

1112

121116151815

210111595

1310745

4310411

1

1111

11

1114

11

22847

1

13

131

1

657511

21

11

1218148218

7552263

51

1121

451012717

1

94112597

1

11

2

66108127

11

31

32

5640881504550

1211

1

155416

321137

533413

42221

121

11

311

111

11151

112

10152553

1

5614

21121

2151012

113

456236

11

32462

11131

1

141

2241

233311

2111

34224

1311

11

1021321

2241

142

11

12

313616194

232512

322417

11

112

212

274234272719

274234272719

2410732
132633412528

12

113

111111

11312

44454

13

2233

4222

12111

11

211

112

11

111

11

2214

11212

1142

62545

11

3964475821176496394

3964475821175496394
1111

7513110016312690

7513110016312690
1129

124642

124642

244789

244789

2611111310
246351574648

8681433

63931129

442441

21911

1112362
1212372

11

11

34081361

1027182910
476139846831

1111

1

1231

13

3121451

233
234

1

11

418821
41882

1

131171

11

122

312

1

13125

121213

112541

184761183
18464973

11

1

12

533

3213154811011369304

98272085

98272085

98271984
98272085

11

312307454991361299

312307454991361299
5946842477454

6157101
614691

111

134651

1112

1112

21019991325

5423175

2171411324

31920177

10261219164

48637177

25113447

251121910

2643

741531128
721331115

2213

1112191361

111139441627

57161256

109121754

118233128

2133

213

25142172

162271226

561619147

2730132674

121361522

142

275310

542172310
542172510

2

11624461312
13627511312

235

231

6825321319

33632142
16328452011

132921369

211221

51092047

58113654

1

1

83415010627199107

212133

131

21112

9

13542
6512866

21

2121

121

1

1

1

11

1121

11

11

1

101
826144922509398

5213333632930

44119326
11141

1
441332

2

2

1

111

440

440

1
5153

4

3

123

16101589

1236
16101589

1

13

1

11

2

1

1

1

1

21

1144

1

1

131

131

12

792614162115

6918114

1025652111

27846401184459

5
27846401174459

112

1

1

2

1222

21

11

11

1112

1112

61691
27744361074455

1

1

211

16101

2

152

4112

111

11

2111

11

11

1

6551220
1

6551219

1

1

1111
2111

1

11

20

1

1

1

1

121111

11

41

11

11

249221
24911

1

11

1

1

11

3

1

4

1121
1111

1

123

1

1

11

1

1

1211

1111

112

1
3

2

13

1

1

222

1

1

1

11

2

21

2

41

2

2121

927784
937784

1

1111

111

1

1

17651969199

17651969199

57125944
11

12

17

3221

1111

32411

13121

14

1

11

12

12

1258710155
11

11
1

1

11

1

1

105638124
1

105538122

1

1

1

1

111

1

11

12

11

1

1

1

112190148176244160

362
112190148176244160

545270318276

545270318276
1

121
156135

3382

141

1

111

321725
545165256871

504864186665

11

1

192137412821

971510129
1

11131
554585

11

1

51111

1143

121

1241
4211534

31

11722

1

1

21

222

222

222

2221
101422291410

252214
111

251112

1

872025115
1361052

1111

1

1

11412

1

2

258

211

1

1

4151

111253

111253

111253
2

111221

12

35117408612958

35117408612958
2

8693316
32115356812252

111

21

24107236111835

221

13542
1131363

1821

11151

11151

111

111

312
3153114

1

212294

212294

212294

212294

212294
112223

171

116181788

116181788

116181788

116181788

62111365

62111365

547423

437423
547423

11

11163291828

11163291828

11163291828

11163291828

11163291828

7163171622

41226

1
8992180218102149

116311

116311

116311

116311

11311

321

211512

211512

211512

211512

2212

113

354085724573
8689173210100146

21111

1

37123

2

2214

24743

223

1

236

1

1

111

212

22412

3341

3222

21

2131

21313

612

121

111

11

1

1111

11733

6487211

597387

31

2311

21

31112

1

1

16223

113836

1181

11

2

11

11211

1344

5

11

1221

11114

21631101

446444

21

1872427022624478518862071
25161218414

4452078

4452078

34614212325

34614212325

15994148510561982649827
9111510515263108

225345

1

13312

1221467

2291063

12

5511614

211231

981319910

16291822217

262526

1111

1211

425521

20787618

7351363

2

1

1

448652

11

11

4784

101430101019

1

112

6563143

1513532637915

353358292056

2

115

441

554989

1121

726555

221

22211

1

1

1411

45245

3121112514

5131221714

136111214

12

1332

1112

44422

428856

4492137

1

797414916877165

4306942

1

11

1324

921222313

112

211

1

31311

652127118

325247

413613

1242

76751041226586

14112

513

23294

1

12

1

121

143131

1

74106223

6521109816

1521

247141412

91314291910

11

114112

12

13121

23522

12

7311534

1

11

13125212

1

122521

146652997291915

421

75551

215232

1

245412

21

12433

225225

11

12

76192193

22413

12

2109721

321

144

329133

11

356628

7413

1

31312

425

35724

23

64322

171541

453222

22411

12

11

3112

1121

202141733

121

12

224112

1

293111

2951351012

591145

1433

1

691126

4347

12925

12925

151

151

151

151

266511911535273012011191
47946410

1201
3133426

11

12

11

2161

2

2

1

1

15

512112

512112

13101739620
981226211

1

1

1121

1

11121

1

1

6

2

3

1

211

1

1

111

10107585

1

1

10107485
61221

1121

11

11

1

483223

11314

112

1

21

11

46810613

241

4688212

9412627
232189922833

46615611
3

11

11

11

11

3221146
4651468

2

4111

11

11

1

1

351617127
101171712015

1

1

1

1

111

1

1

111

1

1

1

12

11112

1221

1121

251

433

1

23212

112

1

1113

318724

11392

11392

192131562127

716736

716736

122025491821
6914959

121885

3692334

2112

383

21221
9243352

9222131

51033214
61447720

31345

31345

11111

11111

1219142796

11
1219142796

271

71310822

33432

126

1611

12163

12163

127262664
346734

116

1

2

112

8173

2153

16464
171075

11

1

1

1

1

1

1

1

26152617127
121

1533421

1

3311

101120885

44113435

44113435
3343014

117121

2

1

261031226
123212

2

1432513

42201

13143
241413

111

9113814820582115
32421071114281

12211

1

243631

1220221

12

1615

21

116122

4261321

121

13

111

2111

11

1132

62

411

1

21722

1143

11

2221

21

232

225552841

3123

21

141

1

1321

2

1511

12

1111

6102512715
581210513

11

1111

1

11112

222132
103256443

3112

102723111

42

42

1
761211616

11

1122

3215

1122

43

1122

431326

36101112
13611

25451

13

11154

11154

10233215816
920228216

12675

141

322061972518

2411943
322061842317

1

21122

13121

1111

111211

121

19441431

13312

1121

14

127193

111

22

2132

132

1321

1321

1321

1299142796

1299142796

2467
45782

211

11

1

54241823
11106327361394619610

284846883545
672401507970460464

1332
1211

1

111

4335457
101011611012

21

34

1

1

11

1

2

1

111

111

23

111

211

2

3324
1111

11

12

12

4291226
2412

1

111

113

1

1

2

2

1

1135

2283632
15512

11

2

125

1251

39162273136
15194652

1

651

1

12

311

1

19

5

2

4

2

33

11

1

21

12011

1

41323
515442

1

11

1

11

1

1

4321332
11211

4311

1

11

88100577310540
71116354

11

1

1

11

1

2111

2

11

221

18

1

1

1

111

1

1

1

1

1112

1113

11

638333335925

1

111

1

121

214913

2

172121

11

1

11

7263131
616272

111

2

1

1

1

23310

3

2115

1

1

11

11

1321
111

12

1

161487
122

11

232

412

11

11

1

1

312
5924

1

1

11

11

1

121

2

1

111
1121

11

4712271215
37277514710889

421

22

1

1

1

21

3

3

1

2

421

11

1

2

1

1

35

1

2221

1

17

11

2

521

1

4

1562765558

1

11

1

3

1

5

2

1

1

11

13

2

11

1

1

6

1

2

331

1

2131

1

1

212

11

1

1

2

1

12361

1

1

1

1

1

111

2

121

1

221

1

11

451072
32211

1

11

1183

1

2

111428201313
712171143

16166

11

2

1

13

1

1

111

11

1

1

1112

11

31799510
6192356914

1

1

21

11

321

1

34

11

1

1

1

1

8

11

3

1

11

12

1

12141712713
2561

21

1

21

12

1

1

1

431

111

211

568311

3951647
2321135

1

1

1

2

12

1

1

1

1

1

111

1

1

12422
32422

2

1

1

11
1

1

11412
131

1

1

11

1122
94144253

1

1

1

11411

31

151

131

89

2244153
132

21

1

221

14

1

12112
331538

11

12

11111

111

111

27111
33359320

2211

3

211

1

1

2

3419

8112020810
802246601871

11

22

114

1

1

1

11

121

131

11

112

1

552416

14416247

11

1

2911

11

21116325
41317535

11

121

11

411
1

1

1

1

2

931613717
225456

11

1

21

61025

1

1113

121

1

16171
1143915

1

111

4115

1

1

173744453038
5223522720

11

11

14131

1

111

1

11

11334

1

11

111

111

22

211

11

111

2

31

23

12

111

3122

1411

11

1

11

1

11

12

1

1

1223811
24684041

22

1

222

1

11

1

1

22

76174877

11

11

66164877

11

11

722

11

11421

111

11121

1

112

21

12

1

1

141

12311

1

21121

1

1

121

1

9323147
23

11

11

1

111

90

2212

121115
12103

2

1

1

1551353

183
1551353

1212

2122

1

1

1211

301219332313
432222200393152140

1131
246422

1

2

1

1

1

1

2

112

1

112642
112232

11

2

1

14131141028
14161461428

1

1

1

12

12

21

3141624
11812

11

1

1

15

1

1

1

1

12

29103177
2861722471413

1

1

243

12

3343

151

2

1

112

1

111

1

1111

1

277

13

1

311322
2111

1

1

2

11

1

8252524
523623

1

2121

11

11

14

41323

41323

112

213

1

2

1242182549
62211158

2

20

1

51

2

16621

1

5981553
1041134768

2

1

3

31

1

1

11

111

12

20

1

11

11

1

1

21

11

1

9131874209
141849952313

3

1

2

311

21

11

21

1

11

1

1

18

11

1

11

1

1

1

1

11

2

1

13

7111223125
688962

1

1

6

2

11

1

2

1

121

311

1

1

121

103112
118736

1

11

1

1

1

2212

1

1

21

3112284
163711422610

111

11

1

111

312

1125513

133315

123223
111613141318

132

1

1

1111

1

556114

2

1231

11

1

11

11

113

1

11

11

1

2

232

1

1
2

1

12316

12316

12316

212
2123

11

1

11210101712

11210101712

421218810

421218810

4271668

5222

1315111612
111456

121

1

2

11

13

11

1111

1

1

11223

1

12151
38131227

3

11

21

1

1

1

112

1

11

1123

21

21

211

426252311
43346530217273

112211

2511143

111143

21412111

21412111

1115122

1212193

112

11112

1253

13412

751437158
1812381467034

11222

1118123

1

31

1

2

4213152615

312473

121

22241

53162

65649118

4518111

231441

231441

1221
16435

423

1111

541211115

541211115

2441

33

2111

551310416
561413416

113

7523
64122954

111

41161

2

113173

252331643435

2431934
252231643435

1111

2113

2374

1031112812

21524

222243

5331

2321

131

11

33642

1

1

1

163775

163775

163775

163775

163775
111

11122

4431

11311

173419213241445422762545
91323999

142641252197561

142353891714
139641222197261

111

111

113

21

11

5

13

112

2

1341

1212

11

111

312523

11

1

25933

1201

121

11124

11

12231

8661543

1

1

1

3

4123

42643

1

21

21

1

21

11

1

1211

1

951

543

112

2

742443

1232

5153

321

1314

1

231

1411

72

11

1122

12221

1

13223

12

2312

1321

22

11

333

333

665924177622109341385
371017413

623369925758

351840483032

2
351840483032

43316

43316

311540432926
21422

611163

231

2121043

14814171012
1481417912

1

74171347

271529442726
235417

114

5231033

411

41222

212312

51015115

2232

663691

539762151419157461167
12

112633781532

112633781532
1

4816557
8212422817

212

2734

26166
25166

1

1412

12

11

12513

12513

111433

111433

112
2364639

133011

211126

1132

11231922816

1137571

10101217115

51251731039
517713146218147231117

2233871164767

2233871164767

3371048

162773993155

114353

212341

1113

124122

112

11

12

1

1

1

15935
85115450573142314

25241051103676

25241051103676

488229435482207
8241131522567

233790943771

1721911082069
1721891061967

2212

128461002126

128461002126
12846982026

21

313
8611020627285171

324072883970

324072883970

1951014
3540821453156

191745861431

151432491621

193049381542

193049381542

106251599
318441664779437524

12922646324
33192467

923345717

46
44

2

221326432125
12

15613201316

77121568
77122369

81

341001171134577

168137282031

121464771433

651681113

1052552522

1052552522

231541313

231541313

871521113

871521113

2281417

2281417

131735241415

131735241415

1
203138755138

92621243029

1151651219

14162931109

14162931109

31023171110

31023171110

232743361842

232743361842

81424111224

8142311923
81424111224

131

212339352863
106151165159131172

17814231310
194217261712

2343342

434524

171810141916
171812161916

22

11

712

3471365

131218121010
131117121010

11

91111121510

5976921
13421

1131

4931419

51071610

101242171528

1
313054633234

61146

41392363

238833

314422

12444

151028191720

25131799
61122183186127147

1

223849412431

1321

4

11111

12

22

4311612

111

1

315101279

278455

13222

12

2210371

231431

11

135711

11

8932

157356

3511

1331

613106617

22212

22474

122513

111

13

3893113

1

2

111

133

1

231125612

1

22

1112

117221

23433

4531861126358

4182978

4182978

4182978

4182978

3182978
4182978

1

413078835650

413078835650
9915181511

5744102

418915

37232

67101155

34823

423882

122

53181188

11

231049

1

22

1461

1461

55574610281289605706
101655453844

8691258232118176

10447381729
8691258232118176

22341

241133

1111

11

319281211

1262512941

22

7172228716

11

121

55211

3121

11

4

11121

121

21

14323

24111324

53121

27364

31333

146214

1621

111

2728131411

228432

88128814

332346

11527291011

435222

4471032

113524

21

21

499425

499425

499425

127212
499425

21

21

141113
4113

11

1

206223301520197228
327613

12154
178200246470174202

416212
21521

1

1111

135118589

11
135118589

10297848

10297848

232731

11

1

1

383543613341

383543603341
124

132122312320

2014564

4131722413

1

1

10311414721377119
2111

341214117

341214117

1
31242826410

31242826310

221132501726

221132501726

481520310

2281112

11136

145621

111

14201319617
4365591024166

45336

56119114

13101612825

6201142510

14517114

133123763815

133123763815
1281087

8231261255

1

463451

2

2

51414181612

51414181612

154432

4910141310

252148442223
2762

1313191548

11

76149119

1542

217932

14424
234387317420194211

3732821385246
71020302412

491633911

225311

21

434736

1

11

21102751

1

2381222

1141422411

54121

187332216260135157

181321201254132137
7513856

2837141686
1232

261241

28346413

131132

131132

314966643429

13734371214

184232272215

191233181428

191233181428

386520661032
95215741476866

7837105

11

361122735318

1

923112456

5712131215

611156320

610154319

121

923151854

1211
923151854

72071221

6203311

6203311

1491

237422
21

3

31

12111

1111

2
152086675642

111053433530

111053433530

111053433530

111051423430
111049403327

2213

211

21
1413777

23111

124355

431

341617144

341617144

221

221

241
302135196599583312

473765
288114185544573304

1326631103751

2
81144521329

2138

2138

31
362625109

2281031
228831

2

14225

13111253

2281329
25126

1

2323
1313

11

17613
139913

121

111

1

153733

153733

153733

15111537
111

2683

2683

135623

135623

355361812

355361812

355361812
34434139

11153

1

27181119427530248

26658107413525234
222143

111

111

26356105398522233

26356105398522233
4711172112

11

11

2202550269355172

2562
2572

1

11

11

22

111

1

32215165
1436446714

11

2

3

28121

1

414

111

175

1432

1

273

261

16

1

1431

111

202026597533

5231214514

5231214514
1

112

3194829

124233

11312

12171154108
312615

1

1

14221

41

13431

1133

917

341122

158310614

158310614

158310614

158310614

14835414

14835414

152

152

112320491730

112320491730

112320491730

112320491730

261

261

4792375
11

3662033

12332
112332

1

3722216

3722216

344464

344464

113113

113113
112113

1

22321

22321

203434882522

203434882522

1551646

1551646

1431344
12

121121

12

1

1211

1232

1232

21
192929722116

252821

252821

252821
2482

121

5141625109
11

312132148
11712

1

11

591

2116435
2116434

1

123451
11221

1121

1

111

121093896
12

43332
1121

121

411

6

6

3341714

3341714

111
24433

11

2122

2

2

39

39

3545821313863

210262
11

15111

11141

2

1

3345721293261

3345721293261

132853

13711
132853

193

212

3242721012758
11

842325833

842325833

31
243849751825

1552829118

9332143617

328336340127342624596267160941531152760711
437695022046976479755906043113

150123163238143131

150123163238143131
1

314716915
153685

27422

52

2316

1298912018210881

282323552014
1298912018210881

219529106

22141726722

1061221134
1061121134

1

512

1234815

353148345127

136843

172036402635

172036402635

172036402635

207415133612528453933029
12023340876167869234720322224156604

283353693034

283353693034

7101316126
283353693034

84910312

1092028123

24812

3877211

145281

145281

145281

145281

2718539412331
183018162307302221911873

121
64661011157392

171226281035

11126

16311936

174521303723

21

12

461352

9432341220

173917322153281319951750
14832431414

788865

788865

3596542

3596542

291276892748
270204361543243355

1392348373
13122548375

322

35122191253

4610481022437

6611411417

222030392736

253425302721

627967932276

101225201726

22733723636
22733713636

1

268259289350214249
8037949261099908767

283211231124

64413101

2191495

6131421713

5162

29938362839

65378411414872

131114351425
131114341425

1

291943452124

132933291411

1

342131321423

411

13121

71117167111

1576443

1

96147871159653
95147861159652

111

504028303314

56631517

9101121214

13122

274220352218

11

251552345830

11210231314

1

1

1

201133205526

301540472855

9336331021

182038552625

141629421418

44913127

279256286343192166
997788837759

20734353021

40732047178

11135245817

106121045

433848633434
193847603333

241311

17281119129

38112135712

13431

2

8718110418620297
345383496717602416

2013104521

8728453344

191734523117
191734513114

13

11

56758

191255351837

111

17717231224

10743472120

141143

201310132813

36111391

203416105

212711

273218535513
273215525313

312

4784

112533

4141973

1

1

822112177

1310262319

227993

536414116

29853241327

121711282010
162113392915

4421195

5744

121311

5616812

842022248

172245402323

172245402323

101125191414
21

249365

87141679

711202199

711202199

14163218738

14163218738

14163218738

14163218738

2581787304630252425
642

58886134304120102

58885132303119102
15111

1371223
42007759

64213
55113

91

3994433
2953433

3

11

1

15300135511

15300135511

3937011129010981
212231812113

11
15541265216

621

15481063215
323649164

31

12254115

431
8311217159

8191012158

822

58241923626
22525112

24141
2321

112

1

31122

178284

2121
41121

21

2

2

21

111
121

1

1111

1911

111

21

42514
42614

1

11

21

1

1

511

14181

14

31

4141

1

1112

11

5131

24

1

1

11341949
98114351627

3743

1221

1412

355109915

211

211

1

1

21581025

135713

125713
135713

1

223312

223312

251212

251212

251212

77241321616

13
66530251616

1111

112

11162

21111

123

1121

32315926

13510315

1211

17117

17117

3321
191806112270113300

1545798216874265

43175365312111
1545798216874265

12

1

1

1

9328181223
8316181219

1

1123

1

551128

1

5623421672272

21516

2132

1

5918111

135537148

491214

112

14313515
4312515

101

12

1

22

211

11

1

1

2

2

12222
6457934

111

111

11211
5424632

21

336111

3

11

11121

1

1

223

223

223

3116516423423
3372

11141

3016013193122

3016013193122

121

113
741713

12

7331

1

5211

5211

604161907950
656541486166854966035818

12421

12321

1

50521041599779
16914371415

12221

43

131512

14661

3422

6311661

21449

336312

2349169

8

1111

101326272016

134101

12181

11322

64718

126213

1232

11312

125934

1

275346488645566356
611537275547774661385275

12231824175
5679901198389

1164

121

11

11

223631

11

1111

1

1

12

7231730927

8143415927

1121

434204

12971248

136

2244
2243

1

743

12

1241

15

31

17444

17444

1381967

1381967

2161223
91035492629

2571497

3111899

231115610

371746
11

1371

24935

223651434348

6711162111

251537

6101891212

610181367
8142113718

243111

124543

124543

17621

17621

112910117

112910117

123
422543934035

311418681723

111023222312

5418302415
2111823

1153137

641

116424

1153

484272895045

151112684

613106210

821714106

9131723167
43131856

51045111

3267810

711111368

5461413

5461413

223202

223202

1011116154

8181071

23683

254813214

242610113

1223101

19201319813
145118170184138124

744325

4233346

13223

621018257

4591034

31215

137215

39345
39445

1

831

841914810

52173

111

41151

1121443

184268121

2463
2465

2

15442

436733

1011621411

5217

1261

2323657

31841026

5499714

213574

213574

33832

33832

141545303429
1134

51027141815

8415121614

8101611315
443910612848102

3261431

81918412

201966692468

57916146

102144891164266
5130518147

1

402663671845

118821301014

16419351016
31231

216727

8251255

21423

1591
159

1

20243313647
384663542367

1424

86192138

811715127

25311

15109121326

6774123

9328123
9328122

1

46611115

213324

253891

251838572934
99148196246157190

659969

124925

591325139

11101234

293765252874

4481848

162233391338

112

11491547578
12491651579

1141

261228231537

1151057

251123131030

432534353120
216340446189178245

33121958

8713314

3591176

281502225364108

155716317

3

122

6111

13

1117

1160114271950

22

13

1133

82732

22714513

1514

8818141311

231922

231922
23812

111

16918151511
4523

51316

26117

717328

4115

4115

1041416814

1041416814

252053382747
7374177186178156

1111251

223262

621911233

112

115864

124411

31452

48612712

342811

1312

471851119

17531

11

1454

533116

12422

21526

63712157

5626305130

3123
23

112

3641454

32441

7281061

7281061

1101163

1101163

310229
241234194932

54535

6998315

103114413

1

1

236167

236167

44641021

44641021

122018371927
1

6131025923

265731

413563

8713302020

8713302020

6613521117

3283668
32838710

212

3451447

26808814319490140
371336

262581831649

2321

26395232654369

66921124

1146114

527457

931927228

931927228

23525
941529713

32342

31413

381625

6268177
694410210287120

5381425
5381525

1

2378103

1068161413

782412832

11313

141220171225

1371317514

1121381918

41351644

41351644

321437751542
10292632

53

41

1110933920

112149220

152024313037

12191612628

14941

3527

2115181

12642332233

10630242232

21291

151643623437
111

231112911

37172395

9615261620

173315161523

162

17331515921

225424
7723163126

3495918
3495916

2

2197204

24824184

24824184

41521242523

41521242523

32543

32543

9726363713

451312144

521324239

904259495341
322945

11

12921151611
12922151611

1

56212

101017245

1112

64651510

8221

491321

1131

3267104

428464

428464

3121732

3121732

5522

5522

15521

15521

53460139

53460139
4336086

1153

991014813
111

452732

5376511

761326109

761326109

15515241714

15515241714

224323

224323

1
434713

3111

123612

6171116913

3145637

3361066

22421

1

11311

111

218312
372360414136

2272581213

438665

24414

658897

35712125

79101131210107179
6792847

1081781512

64426810
64423810

3

116417

131447

1

1311022

234113

1185

1311823
135622

621

32813

98131437

61939314

12412

410413114

154332

14

191819131137
201921181340

112523

113673

741622437

3112102

4231263

4231263

21321

21321

56111646
1

158136

41234

313211
444077586283

11513

1221

910751323
910751223

1

2321042

45221

14626143233

361113319

52392

256531

2613161113
100110214255216198

132411

31415269

11411

35525

1214

117181695

546473

1341

5151313523

7471478

21

121

511121924

1229

31

125962

1113736

141

74141196

64743

115841

759799

885998

1

715167731

511261814

212612

12

111719342112

12622

4582204

21331

6111022712

6111022712

313347643171
202228301949

335737

6371559

2571246

61135291823

281717721

431812112
331612102

121

12112
54527411610755

6183177

626993

54111666

161113

32516137
31316134

123

11114785

371186

203025145416

4281474

4281474

3561673

12442

2541231

38562214324427

38562214324427

633565734178818071003
144117120392418327

54774223547

76453136138358

55616176

12192234

135167917

35181413412023

13414152615

611216139
6113171411

1112

1187351716

5

61018162

6131620932

392130441717
381927401516

123421

13481920

32311103

1

132313314931

12420141718

11149718122

741022125

19521252127

517114223

6116352017

101320195863

48493

27421843

111

466816138

2221

6820127

64403815021741

11

9162754

42916123

2321

12281822232

143132173040

71319431415

1312

3218

19922875924

388191417

658111612

15926211238
34457211364105

16272

183640655067

2432320159

461196

23917963

84151625

84151625

11174

11174

3027891095638

1472719149
181436332719

479141310

10743522113
121353762919

26102486

74692111

74692111

228987
8915132420

151

6774112

2041010183

2041010183

4410761907577
612158610

1347

341456

13121412612

2532041

10111014615

5527111122

5108173011

92724231516

92724231516

5746419

5746419

31235
373668554582

528141213

487639

22511

2

43126318

181931181931

136445

648957

648957

251925552616

1294

339173

2018719310

531

1181522

163191208291227215
433338725652

24514146

73921522

425151154

1161

11212

325723

312

7612171211

211516104

1814714

23523

154

14151946

13

1

1

16219293017
14217262715

12232

11

17121739

4232

231720233032
231719232932

11

34210

187025343713

11

551113129

551113129
35813128

231

52923118

52923118

8281048

8281048

14943429641

14943429641

353751842166
8121915228

21241

17

4411239

1221

35611
3541

21

51381037

251512

93369

33

221331

23313

23313

329224

329224

851710169

851710169

2929801095043
112711

13121235

21010210

416474

2561265

41111751

143774

10131131146

21115

218852

3310899

3310899

68912108

235764

454544

4182485

4182365
4182485

12

541119633
331111229

2632

1212

2271153

2271153

5171726166

5171726166

6460117133105106
3225111110

1137672

145933

6293

261395

31

37122419
107177921

75552

36766

22

134

813332

2

4127

7661312

12133

3253

312543

741091017

9413181110

5451692

13643

293940562953
612

152123231231

596151010

131

9881269

8191215189
339328452550287413

343335601951
125128121196126110

1131

331

31923

4251413

2
1

1

327141110

14133211

471364

4452

321

6213109
22213109

16

11111410522

8258445

53431

16871

1371352

145491722

212

2

24617252021
1

2156999

15688

1253

2354

1423211519

1423211519

10716211011
222

457831

62711510

5213776
22925231619

266885

114

143711

1213

2392427519

2392427519

443934
8741441615

10921

32212367

117353

27813
493375871679

233731

65111124

85131547

24163036454

921110210

132920310

132920310

4385917756111
1

3569857354107

8166414

20522681819

20522681819

1751832713

1751731713
1751832713

11

3436116

3436116
3434116

2

31251151022757
13

111317313
111257621441

329612

329612

3221868

126312

5121331

2

115515

112
18851301214

1864018811

12113612

65271279

1101043

1101043

256712

111
254511

1312

1

12121

221

221

679797685111618125442269155109339
59012396812912086916

666917052109629122674265879107610
17283822872324257832739

24940504325

24940504325

276157405392463383
13015821955191725251808

17243233944

10141410109
10141510109

1

471461

14458374317

1

42766727252

161130472229

20546445049
291756647964

9129192514

1141

29232254218

1346415316514489
1346415316614489

1

154485210744
164515310744

131

2254

1214322

1

11

8977199

16211151217

10316161314

19348366152

11229165115

312134

1713124

6628316814

541317287

9141622
9141922

3

21132347356

19837233069

1189154

601011472229107

1581429725

653059473426

71871312

181110

8113191114

10831342817

141583210

2321645011357
2221625010456

1291

10721363027

271156949261

142514121017

16315374136

302353445358
322353515559

2721

181118152234

161313144315

7516211512

58341048212777

18235191033

14123305320

1

56218273114
56218273014

1

111152510

443333

11719262223
12720262324

1111

65441344439

379225

203531404431

183531374329
203531404431

1
111

1

1

1211

1211

152179206220303148
504476734824904501

2471

2049164922

5344

28231024157
2722924137

1112

159103

181428237618

1412114103

35915287

699211211

1541

754211526

2341

2017105

2232118185

18419193731

8317472414

421023172021

710124910

7515111613

21824282514

84561214

91315165014

91315165014

132575553024

7373959

729161510

1084224225

371449535043

3213161111

2839612

777641

724414

4727144

81924156

2983011
2738551605227

861431115

4916521910

6952554

751221107
751222167

16

181541332332
524810013413892

1

1

22413

1

72674212

144552

174885

4215222518

213376

8821993

56852

3

491524109

264121440492445386
18099582442255728552128

14355

358215

15192421156

1

18852737630

26515292211

425

1

8718263623

132

143042414730

59514161816

10571276

1441

11

38631433019
37631422919

111

8625302013

10013167112163144

642013628

27111

27111

302035374752

9616161821

112

261656488057

21382312

101025365630

1081415387

11569234172167169

10731222310

372275785751

12413141917

23838304132

241675

765660495754

311

882598149178140
882597146178140

13

1

1122333826
1021243218

11968

7422202312

753411512318680

4621

531552805749

2124246737743

361355647573

14518252814

10530335025
9528325025

121

312791211

21

29726703837

162225716710377

81312212623

431123133

4541056116174

111

22155365927
22155355825

112

231656473741

67701171069393

839155412

2138139

337741810605915933614905659162
610194427101122113424253270100074

6315101113208143

76521421198652

331042484935

511667699351
471563658047

4144134

20113309526355279

32832

234214293061152532

354953606934

721120259

1184

1781635163

11

23326505533

18332354300630290

31

368885611158

31448768042

817861109868

21526748652933217884365

14361174258280171

562160131181126

226142230241508287

307545511658

902998170153140

57169412517387

76616815915396

11519130210270158

11

241738426736

19547263292347308

32513407744545504

2328576356648313

8128144141156104
8228147142166106

131102

6319161175270111

751451914

461023956240

20820369397412316

64192666

78868989383

3323484192136

331246345461

413412

1

23216524935

34962576629

7316104117203113

14630296393405276

1174176146444251
1163169143427239

11731712

374724912548

3651156811471022466

4412851138157

21220717

64189214317380

255152067490749394

9123756419377031360

257325092676662412

632711913912387

15429289614422300

21522

111020223018

18317294133
24419374436

612833

344635611037
31358518433

3155264

23469457734

2211

512

462559012535

6372267811021131919

52107510012182

861610718013497

24223459952

2215400215749439

20913287953408251

9711172211

4178112

362

741412

117314144024

35578696564

1

363645448436

7251101379992

14413224626412168

32117829

1

16627273013

342780625143
302773593837

473136

2

242711

3331098087137

23416152641

98598183134128

1

487701248864

47955829852

1530254022

203424252123

56109712614547

70729161858731741836

282055484253

24140333132

532

16755471823

757141126202222

13420351816

38224

29357834750

76454165565829811392

17912141018

471255998372

7645572088164

36

52101

3957511312754

202317363912

680195151369703842

5077189162520111218544653

153340292622

803349619592030929

5013849717492

222138644433

27122268720

1

17917355338393253

312731562216
302731562216

1

29739657647

522450796964

403345425528

123328382126

31331485

30824343321

11246506322

11

235510863702027122156320

12828434325

111

29831493058136261013324

15718185232187241

523165878798

96341031299573

60281151268994

419565912540

28761728043

642808510062

18665541421765123522852

63279511712683

66181237813876

20223167393215275

2896232778537297

1

1

17586215231234147

17586215231234147

451618342423

11450177159191113

162020381911

111
19620381115

1221511610

2331852

5192

13543572022
9331401715

32121516

1221

5153428491170990674
383537763742

10371203300301175
3731136

1021821122

631819159

112811125

934367

12423173120

8728368614

23911914

499212023

451725410

419132414

551323103

7910382920

467954

14527423524

56338712211057
236846

4271575

63611134

17824184

241228223029

1963242389

21101799

151

1181298

11

202138575839
159108313336319202

8615141112
8715141112

1

361519127

326741

2113

782411

9831231217

82

228654

12345

238534

8281874

1242

3513614

11812159

41639

338621

112321

13823252211

2216849

1118156319

1322

8513143517

36111876

3516148

446734

1

32101

1425

6671495

23121023

361583

15794199319214189
33311019102

134642

13441108

101725262620

3110103

2031714137

4553

412167935598

143616110

33658

311614142

223842

31410158

611813138

1254181

162634217

11811282

11811282

172160161305192137
371128733621

621026712

1221

21

121

2101357184

21423

814348

774124

83812

294015322512

32

168617274

31628826

44713813

441391712

4686

1718181217

871316815

52634

224542
7155891125495

21222

12

1

1

2

2

6750841024789
5311456

142845541646
453118

111

14141029

22

251

1

514

2

11

221

11

1

11334

211

111

41422

112

1

16

1

161

2

111

1

1

111411

1

2

1231

21

1

21

21

1

111

1

2361
481928442637

11

2

2

132111

1

2351697
2351042

655

11

2

2212

122

28211

12211

11113

11

1112

351

11

143715

11

2111

11

1111

21312

21312

292743591923

292743591923

21172831912

21172831912

81015281011

3281326

5871585

23
203841383832

183239333432

183239333432
1473412

6871042

71814151512

42115116

3254

3254

235
5862881178967

116172614

1

1

1

1212781

24261032309

111

221467471941

1

54275210471339636732
14111

161331

161331

114121749

114121749

4787039331197567655
81635451817

310102452

397929

138

6581065

614872

11214

1762

10110162418

13525

313714

712536

11014

117815

233323

33311

156513

1151010916

3126671082841

562624117

12149152212

12

431083879168

8664101

23121568

33211

165675

1341212

71154577

192027231431

142217610

121037272732

36

11662845

19315678

11312

21411

11

118122

15719482630

211044

424543

31138

3518775

11211

23171142

4

275886815

13111275

35753

61322

531010413

33281

232752402340

452829812

18135513529

75415616

81014201310

260510191

8586669

729774

123

5221161111

15162426832

1

14865

26654

97141536

173635433323

322101

2262

41124625

441910915

42336147

452322412

13112913325

24291381

113442

191430373015

91910173

941315710

1981262

111126281119

111025281118
111126281119

111

211639472032
22533

11
55201677

316842

37323

421

212211

16917261022
3

846925

312

121

234537

626735

22351556792421224257637035
648918436680127

14306211347423196248
1641625804601600227303234

6715111515060114
133196308352128217

351420615

43207168

12

9724291420

6215143

145445

5491025

281593742138
101361541836

182322032

105124259

561462
243350682557

121018271313

12182627642

161851891515

161851891515

37471602835363

37471602835363
34461582774859

312654

77441532083777

77441532083777
77441522083677

11

281564

281564

13149137

51

1

1399117

458579854363
130163272278252193

347675

3121186

71224192420
151246474939

4112

818172319

541

1791811156

51115133012
51199145

53157

111

141730362212
141730362312

1

2141394
2131164

123

2182698
2182598

1

211141812

221832224125

18944362221
3419861084644

74163368
74122726

4642

52191076

213235

121

2332763

877517131310297
4144699221641496631

43331001685074

111

2638781242671
2658791282772

201411

70461492727775

314767964756
314455852254

31211252

5040961214364

122214451815

1615591551539

5211411021072105

2614661144531

1510182

33101

33101

1

1

112151202222107167
480616824756687729

8517372014
8516201914

1171

8348259

212122

12

16232513933
172827171141

152428

758513411912971
3243164

728313011611367

7133216719
6123215519

1

1

1

12

1121

2322
84763

82441

1032112322

6161978393106

463142683331
6161978393106

1

153055146075

62813824
62914841

1117

451418818

711691

4914111123
4914111023

1

23926312513

11

106194154104171136

242863

242863

1

36135818

13151

238942

81614978

111

13112

413816109

675712416634101

635612316333100

411311

323446663344

323446663344
323445653244

111

2424661492544
145136337557187202

4545901046151

302468893958

46431132156249

491317714

491317714

347587985857
11

337578935855
317376915554

222231

852

132947532530

132947532530
132847532529

11

232543473720

232543473720

192752402527

192752402527

278110269214175203
565712610286105

96149813

1614

335543

5312694

62191474

21523
1441132168

1

3

14318645

311011114

644665

292222222141
292222232141

1

1382114913

437243

148254385504195250
117121272342125190

722

14722

31

2421

18102
1872

3

12111425

581210512

2

419131

419131

222232

22961155

133

122

32792

63332

3101211

420151813
122330474017

8315293914

368841
3103829211591439591

1
2436691124839

2235681074131
2436691084434

211133

435

81417181420

81417181420

151252972019

151252972019

212448863329
2603147751356353512

133

42551282345274

3755881263942

71017322

596723138291130

91132883323

3312

6273

2346313

65213311

332171572551

1

34401203044697

1232

81524201915

1112211

1

566010317312187

222442843943

222442843943

343661898244

343661898244

1722

1722

4158901043971
550524553433410323912994

93321262319

93321262319

58711107
282353634858

11727262536
13728302638

21412

10818221213
765201012

3213221

3942671007178

3942671007178

400118315329352289
5519442015

74720417

234542254045

2152135
241732

1143

67513217

1492014721
2192118923

71422

5459155105192119
22522141511

523413391177108
473413184170103

52775

4251514610

81215241222

19102936912

2129554

231
263468853343

142041512030

121227311312
121227291312

2

461216918

461216918

101335432435
882325412

2347114

289189
2811199

21

781621416
4529941353553

1772627106

11932501223

105203798
105193688

111

6780114908264
128167221226183190

1262

4743

3

126174

5323

22118

462191937

3

136109514

2611

21

3312131

587779

112

214411

12214

2121

988161023

123

31183

14191015313

122

12113075
12113096

21

38182077

38182077

1
11811718619875116

2335313

341967652727

191634341522

1351434

4113695

5597215

202729281021

58162763

7251813216

251426643326

122125

5591262

11291666

966151913

6626371218

6626371218

851837811

851837811

13132941926

13132941926

4715367954563
1

2712532311626
272832291626

972

1

1

202834632837
202834642837

1

5479801245755

5479801225154
5479801245755

261

546559805867

546559805867

24131867
156127252294175172

5672122134101102
55711161249997

1161025

96501161396863
98511171426863

2113

131727372016

131727372016

262138471630
10171936

116119711

5142019613

929144
7217698682662

4213661331832

21113631826

114391371278379

114391371278379
111391351267575

32184

503972783167

503972783167

1416232179

1416232179

389127118141177105

11102026296
389127118141177105

3781179811514899

3311529754947467827
11113613621592138

112311

443047432424

9161828818

3116

12996211200143195
11986176159103138

101035414057

389758723131

718412918553282
738814520655301

51215

24119114

2826687115

456484

282212331

222135344933

222941562941

37951622334

261973552863

261973552863

5086691016938
1351

1061718812

5132431156

356625474619

156198236289135214

156198236289135214
155197230284135212

11652

297230471943

297230471943

41621281726

41621281726

183451732133040775873191827964
6838001739362415251318

115
101179238313153166

2

2

188146563819

188146563819

761517812

761517812

101831542630

101830522630
101831542630

12

2342259

2342259

81814231910

81814231910

851919611

851918611
851919611

1

752425129

752425129

404384903966
253

182350471337
182350491437

21

221829382529

432774863365

432774863365
2632

632512515

15102018619

7612391515

1381114714

721322915

721322915

721322915

1152062749

1152062749

353372782909480555
31018371312

1

1

51541231217495
11103639826

121627131527

124

21

1

131736543423
111635523423

2112

141123111615

43171439
788019520395127

111

4512236

12

15146214

12011

433473805268
433473856068

58

263376732628

40377512511491

40377512511491
4037751259491

20

282758672535

282758672535

57581611616682

57581611616682

83931011216193
111325381410

131428341543

596648493240

131350743220

131350743120
131349702720

144

1

1

142815512260344122072585
372149904068

91110881526219615981868
271451510723334468

1

61329325727

201144222322

131

122626

11111

12

732118113

13
22102

2172

37

43512

11

11

36303962367584

1111

111

121

91812911

101734

1

283246693751

1113

411694

221513

181029624316
181027614015

2131

64231615

442651

518525107

11

1212

233

13639
13839

2

41514221726
22717713

213751013

13716

13315

1472216915

221032

322933

3653213

3761484

2211

111481

112322

3

21262186

2

5101979

9291935
92921035

11

221261

838927

546156

13826201532

778210816614784

1212

3288131514

5162175

32631721148

1

892822

15713

11

11

151

111

322943

14

53667

38171157

2426

7161521

331111118

22

22

2271131

229121312

131

203242594038

112

11

24621

1

118

31810211

241113186

162910558

874836

894912

1091117419

14

14

11122

11282454

32

1124637

11227241520

514

11121

2431

1252

126653

3226292319

35181355

111

21320213

281

201620265914

112

44371161968799

1131

12

12531

112431

14937

34413333610
32521324

31281126

12

311048663833

311048663833

18322461110

82411

93153484
1032042109

15825

706213423111991
155244097

41210957

2691177

3310171810

222226352419

82728414
82728614

2

631231338

21152837

8821321412

13825391036

13825391036

75849117810289
161919351214

2231

2424814247

91322242114

3111117174
3111015134

124

13132654735

1023312114

144141146228115169
4139411224960

393233482038

11311

515954393455

781210212

2152

533541

1153
4761661023548

2243244788

171130392232

76121128

1
3360859549108

183159684294

14292627714

29539945331
121321

1411732512

15320494618

20829763734

20829763734

201148232301166469
556658478266845472129619

7111318713
11

2212

1152

1

54143

22515

4355
43551

1

8771197320271177
31128441825

252037512452

101345624232

21185411410543
21195511410543

11

28832498225

24818602830
83406112057100

42162133748

5111141212

13

111511101010

462850217111675860008104
5576536999275871053

4955719612162

4954699511959
4955719612162

111

11112
12

111

825052994374

142213

16202144941

652629533330

331031835609485145586044
10089352457186119041668

14521133014

271195

30916201212752032

4602232912

196039153046

222864371418

36526

1812

141022302111

134

1

5561395
71020201715

25147810

381623342029

134142

62799811

11

314514

21371243

316323

7141312714

53121344

1

1

2432101632634

1923433

312051785245

11171285

2354

141234452634

219138

334128381630

211921241228

912717613

233

622

14121555

11122

19932312721

171324433622

452834281914

37161812

225

4122

2112

5331162

4391

3310742

86513916

279379232303546221
240377223298543217

3814223
12

1

1

361

113

1

11

114311
1111

1

32

1

1

1

225

202327621723

2110

12

34763

161238192620

11

483764564979
413254414073

11

21

1

63101386

14927151911

21131317215

491419410

279644

1069516459159172

329384

513315

92721

8102518414
13282919918

5184154

243316131012

114312

132626341628
132626331628

1

1

2

341

114432

8371919734

22273741441

8

32109118

212027151641

81530311518

141442422232

991641715

61311121110

201627282342

1

1220196523

5782848155107

18122

910158213

1331

316428

682422

356940442866

412655474438

13

2182102

24916610

441417148

11

51

6242

24934

3441382

13388

3142124

22341324

1172715622

1

8422481810
8423491811

111

37426

1541218814

35411825311

9531233205

12

1131

15328324122

7525221322

176719202831

4214964411412

13212

32341

1

1092226479

11

91173216

422211610

211077349012

315819224132

3395922999

477942

526821277

273179563548

14489172013

203069411558

357673

591102124715
581101823714

1311

21491612013

75125219

91124341211

3744315621115

4

4591446

30314215

1141519720

142015191515

154235167

52131087

791529815

131453261722

22323

2181124

225014221619

1

6291036672773689866
91242163108139209

912624

155225281817
165194160244200198

1143524
1143513

11

11643
1121

1522

26581516
26581216

3

535
525

1

21
11

1

48615145
191216302513

1

1244673

36845

11322
1311

111

11934

11934

9011399143141143
8810786140116130

133

2

2

21

2361186

12

1413

123221
133241

1

2

1137610417

243141

112933

8176111114

2253

3212

311495

6779124111109117
6879124111109119

12

52343109

31339736

13524

247227231734

7412161613

2

113

121

22161513820

2184

1141

5111936652639
5111936672639

2

11917682114104145
11917681114104144

11

51169710

14321

11

14481225

14481225

97175168193115184
560556652937711756

524643729164
228154214352269204

131512511

1

1338223

451131

2161

41573

71219231520
71218231420

11

111

83132835

236

465226

587101110

29242341

15334

7232486

243647866739
243547856738

111

458926

1143

11

411

6532364

1311

421

14131812814

32321

273437697839
235227270392327368

257224

94239292772

7411161110
7413161110

2

525635

1

2322

12721

1

32

255182267

25763

314812271811
314812261811

1

372321110

251124939

3

304050686097

304050686097
254049655794

2311

1

222

1

2

812739717351
782539667150

1

11

311

2

1

11

2211049

411257

121

17382053734786566292
12101523110

106929638511443
14915510215214974

4111
403271573

1123

1123

398161233

398161233

1141
141

1

1
61

6

1

3

32

41
111611

1

1

1

1

1

111

1

11

81718241615

81718241615

1

1

2234
1

214

12

21

11311

227

11

4313
4323

1

182626274028
9191624823

3612223

6191102

2281222352846146180
50527499339585

11812335

1129

113

126

6716738722

3611313814

22127115
22159157

122

1

122

1222423436

4365424

2221

4913610

211

218131520811

1

453273618064

20827325313

264614347
264814377

23

37153345
2202227967791340558254623

7867952960608623391777
1937199862311266454644224

3256423312038

211246206

10620522516

128591113527

3158701247755
3158711247755

1

712361172035

12843

9223352913

2515297

6625461625

81272833048

2151171

1101458

3492351

152112

241435

1116312321

31011275

262113520965192

62929126

41320414

332143953926

111116191524

14319512613

413737

1193

6914323711918

37501263128199

201092583226

4631551410

1271576

1613791155750

311722714281051208

4257901555993
4357901616093

161

13162668

2411

211526199

291442265

4131734

203847662335

1231325514

5587109

137117276345121187

271536281637

756413922495107

756413922495107
666413621489101

12

13

635

9111

1

17373923225

5072151239144148

352076308

7518441527

13435251017

314421311

119182

10181926514

1152473

38291172088689

2934741234171

14713

221716169

11410113

2212181210

27144

19131031192991
19131031172989

22

4622431011

6639391126

636015323983115
57571412195791

6312202624

7132121511

81141731222

3757551023433

126402310

26281191715541
25281121615340

171021

161725395229

161643
21425118

18975

1

552731127

285252843627

117866

142015212

211321

61424431719

1322

912129135

1051145411

1051145411

252269522663353383
2131971104346

102434382723

52369312980100

746898967484

3237771255655

321732562222

71241332021

244450763132

151284817233354214621116
10839132351910376

2641624002834273223
3011453722725

810986105

3530633435530

4843665955050

3713502373525

2220394763241

2219375322536

1311766106
6216911933911

491574127295

71264
71123771274332

16824432012
16824431912

1

44943837109

112183577

5415705694732

5415705694732

2129
88111963707250

69103421385226
69103381334623

4563

177522232024

92739894826123924703
4561133426489314211

21811

105159414127939

47316330010874

21418404214

839712913921104102

4823406864425

5344663787874
5345663827875

141

19483

1474

5536879785651

1616364322668

32275115

125243656010

2711355641934

2

30047421632202670850
14321881866581

3472377338103140

3472377338103140

375229828066108

375229828066108
365229827966108

11

3112939615
72111573619151209

18281611864851

17321931924780

34401902025063

85136438466175205
91553621435

834601033939

25391741795962
25381731775962

112

112

43481511226369

35351301204142

35351301204142

23361591936965

23361591936965

161835516224
729679141317908661066

55121846

55121846

232382754170

232382754170

3232105964464
43421211205580

97613915
87612914

111

23101121

84451081636279
340292490737351416

112927371231

15521139

213432

2571011111

114826431617

18814122416

21

8928371912

12521211110

4461

51427411622

11113

6713243120

7112028711

38144735643

8817181433

23329251010
23329261010

1

31242155

4761486

8518121532

4178201

115133

521144108

165202373

21412201211

859846

681728410

13752

191351612324

191351612324

34421251565789
185192399468221316

322367584054

12216301715

812827511

11920201113

122

91243201938

19101834615

1030720132

34141983
34141993

1

241629271037

2

16411

12316141612
1131614157

5

11

5231718815

1

45132069

221183085
22124421210

5924

1321

303375914259

293275894259

111

1

109202364
5833931024140

1

1

1

201026391117

852415911

1

2092324138

44181366

44181366

431312815

431312815

255626964704573334053805
145145309382162283

7684175296122122
4375238511368717593

101413361019

1451612912

231532372832

14943531624

344267733435

5159121619

111342541914

141432481818

19221726812

392639561542

10924354715

79911823

474882743834

8320527325

152260634541

182834527924

9914201215

431520814

211525803323

112

3453

451730911

5715481610

51217451213

259129224613

141314682540280818212178
480444903875569800

17533291115

5213766

331014516

3212171111

1

18202224716

171726241725

8569104

9255655

4891386

31207101

62971186

12117

533635

5113161410

14564

2110

184151423358

25

41514

61311172313

214534

92641592

12111221

22645

21323302426

13495

15526313921

21

1031177254

2111

411120105

846147821

8734100786367
8735101796467

1

111

5882283

718101512

1

69918814

1639137

61226222825

272058582861

41514

851151117

345674884839

2421035

525616

1454676

91012294912

12725241021

203436231885

3321

29745

41251

214642

12214

2622545

2622545

1

11515211215

5171221137

521641

534117217

131319351422
141319352524

1112

147331

5638152018

221758

1034111111

402988904256

9619151210

21373

243

2810112

191541

32105811

211131616

2502118739

42121634

11731581326

10834312725

5614172011

387638

5361285

12483461166

71229161421

151822212510

1310143695
67113493

2

7332

19241912234

23752

318367

1141021

36371

115735

2768920

9930432117

82529351925

3358243

224872

127112190241130130

16511241610

77122277

27293233832

22141746
71721091536760

99716318

31111183

101730543114
101733583415

3431

3332151894

1141241
1241241

1

1392521513

12858773129

12858773129

10967205277219147
261454632824

642126105

1

10131025617

11111

361542363550

1273535

11

112813

13731
1373

1

114723

54920115
54920104

11

59192111116

1542326917

28451721920

28451721920
23451516920

1

524

503969526072
12417173013

26151714637

101427141718

268774

39631068038100

39631068038100

222168372455244274
361239592433

45261

108106234308172174
111107241319179182

2434

1224

2

1214

1

11

2

482463422138

232027291921

309868603749

299867552947

11582

56468412621915777985
827834

181548883432

181548883432

7514231919

7514231919

7248273616

7248273616
7247273516

11

22845671422

22845671422

252455551445

252455551445

505255623945

505255623945

100118252388124195
3524839321445568713

101025432533

5313411913

1432

12852

562022476

24111

927461023129

25361

11

113

212

331220125

1

51927743019

15726383223

31115410

111

152048811931

173235741926

19331612817

22241

121843382730

1

818421

252677893952

5

13181636512

111

2481455

1112

125112

51

101339581321

181026301012

2112

35

141

11111

117611

141130712

39679814161131

784356715

4481248

4481248

271742562442

271742562442

825141449
445048722239

37122049

8671548

1315644

5371066

78373

5850851146457

5850851146457
3348811026355

311611

221311

5

43200911676188

43200911676188
61223431511

2132

2121

1151
11262

1111

1111

21

3221

3912112

1311

132727
131522

12

5

2364224

8

1012114311010

1121

114

311

1111

33
23

1

82102117
629114

2123

1

22

22

1

1152

13103
1242

161

13513

13

1

1

1481243

1131

1

113
11132

12

1

1121213

21122

45142310551386576710
32291071312982

1325811233945
325013924211096

561022

5419251418

46915294

31217571423

23611124

11

135116225231138153
182049652454

254101833

11615321614

3419115823641

3419115823641

313329224134

1634712187

11787353482160184
22281091303755

13456782823

21313

36524281012

271252

51328732329

14742481715

202579963333

5371472

3163022816
4111

23213754

841314211

104135201278131179
91046682531

215046472854

526778964470

22831673424

1181
2328454116355815855793999

538108558013342529620
311712

3271445
47099453912822505589

2231
130158200306152216

2323

2323

42668
253837972179

12

131

87125229

51394817

321

32151

8111367222

1

2

221

221

616121733
343851471746

3811217

13

20112624335

1

1111

1

421251

7716351111
677510114911385

15

21212

14121

4313156
12

3313136

1131

12

421

31

11

111

13113
3

1113

11

1

237117

1

12

21

1111

203819476530
203715456328

312

1

1

1

1

1

11111

1231

12

1

111

131

2123

1511

31321

11222
11427

25

111

51

111

2221

21111

112

111

2

111652

1321

2

25615

2511

2314

25726

25726

25726

3348313259542346360
21351

131934361250
334178763797

3111

211

11121

84

119136437

1413
213

12

1231

1254

111

2

284116
321412126

12681

26

21391

138

211

121814401813
2717742016752268202

1132

1182

1212

23

6226457187180349
8534472258189466

177215587515

578131

11531

412173
41173

1

1

1511

11222

1224

102

116

221

1

1

31581

111211

4111
411

1

35811106

1323888628131494
186818445915

2

611212229
5213915536514

321123

2111

1

41125239411

111731

1020317264

715716227

164922493212

826617133

11117153

195913717735

112

31

111

1

311115

1

314
21

14

4266095
2615421953160

1111

11142

311

112

26

9

1332

511

422

11112

33132315

112313
1124413

1

40

15513

92716312

121

12114

11

2

222

132112

132112

132112

34773421

34773421
11

38221
21

1

372

21

21

306721

306721

311337392128
1

281128362026
222251

132

132

11

121

12

12131055

87519

111

11131

11111

3315

1

21122

32

32

326111

222111

14

2

2

57401111878459
2274543265774681630493379

21422
18859341702853429502

2243

2243

273236567668347403
52112717

2123346

2123346
231

2103036

11
5415424

3111

441233

8915274816
1161

527528

14517252

2121154
22124

113

1131
1121

1

1146193

1146193

5355672
213252

244
31254

311

2163

226755

226755

1271851022

1271851022

223205479490267341
1515561101837

4412572

12221

4167

15839252933
9522171813

2152

63157618

28153

315123

78161567
98171568

211

211

342

107151647
56151647

51

35222

1149942

19111513219
19111515219

2

543
36728

31325

2291175

5471512

21110957

561111211
46108111

1131

12532

12532

3915846

911306836

3281994

24211

4633

9126157

11143

6291352

22161729

98818223

111

12520875

1103816

12181

111

3612

143

778376

4962546

6545

66533

6471
2471

4

8584112
8574112

1

81477119

716189721

111124

9113836

158353256

185293648453622
1

185181122221810
1

21911

18516102213169

11061324
11

133123

6321

52954

52954

4105363

4105363
4104363

1

135265

135265

234838431925

234838431925
13141631

4579514

174233

15327933

216754

321946892547

211962
301742742039

61191443

54104416
549448

18

348112

91033743
121043743

31

214213

2241558

2241558

441013412
797214916290105

2372085

2372085

2372085

11
523961693535

6176669

1111

5176558
5176548

1

624677

624677

206102259
44721

2

1656137

1121

191441341710
21243

4271073
4271033

4

1311322077
1310322077

1

202461583949

1

1

44236
5912211512

12121

4

143811
143831

2

43674

1418915

112

411

1

1

112311

3221

5910141120

5910141120
5910131120

1

9221131212

17621

410322
410543

221

121

423247

322
1210244

1

11

11

1

11

51

11

627398624965364430
5553361

54666
499229398556216262

8111122138

14511

771117127

517767

517767

517767

11
241821291532

1411610612
724759

792313

1

7553216

1123

1

1

2291143

223113

223113

866919

866919

331371121183635

3182286962912

3182286962912

54422

2111

1192216520
1192217520

1

81151611

81151611

7138121013
3221

386237

132866

1111111144

1111111144

3715678
412

1

2645

13512
138221

311

13

81819105

81819105

67113764

67113764

7221338186
1143

1441142
1441242

1

2142851

3371463

395082954189

121

1111

1122

11182821733

141221

5773325

1211

11

2

12514

23331

15422

7528301014

7831963

323331

96202732

96202732

131341065

131341065

41022571214
1

41112265

9103569

111013361611
3382029

5631521
5621521

1

3121121

1141
100143176268114145

171226541318

13815131217
13816131217

1

44104111

44104111

4260591125133
122

6343274

17241952

81729302013
8151922612

2108141

98119109

58553

236262

76149410

76149410

14152112

14152112

215632

215522
215632

11

33322
93029512935

1383518

124576

21

1

12281434

6623124

111681554

111681554
111671554

1

10132015830

77159327

365653

1221

1221

11105127
2219431072822

121921

1

311

776842

11

311

311222

113

3

153101

11

224311

132544

324532

1

3

1

85345014121405528604
1121

12

12

431146666537166124
83843813781356518579

143744

25214

24244

11

1

133235

12224

231530621825

213

21

1131

111

11

1

2211

1291718137

5315

10394108

431031

213532

11166512

11

6810969

531

14612

7121310326

1

112

40694682012

26211

112

13

142323

1353

111138431523

1431

1

28722

1

1101

2

1

31115175

113221

1

1

6271011

114

27103

111022

222

11

21

1

2912

213211

3813444

13228

42252

11

411417112

24622

12131

111

1

11411859

14

37811

11553

1151314511

99101314

631481

1511634

545833

34860461116

4116

517517

214

85141385

215953

111

2183223

2334

2223

2

22830351410

11211

215536

11

225615

314

2211

2213

1

152

1

434552

127

11

1149834

1

11

21211

241323

2436935

51383

191914818

554625

11

1

12181331026

1331

13

226713

223115

22

5372492

3331331

3391641

1141812810

1212

233663

1

2347963

3481646

23111

1

3161215

3161215

871826615

871826615

132512

132512

25521

25521

77840473811

1
77840473811

5721527185

5721527185

1261574

1261574

14195122

14195122

186126052076237811501195
10113125127

800106010581194631682
17447381010

284259502122

284259502122

284259502122

67551181156875

67551181156875
222146312935

121129321514

612

14491547
12491435

2112

12112832614

1864143

21284
273347228326123223

232305167245102178
381854662431

425242602870
425142602870

1

12213
12212

1

173423302043
173422261743

143

2471342

1

1

11418726481024

1781325143
1881326165

1122

394249731745

394147721344

12141

12141

161541541012
415612606665409352

453442533227
1231591271729392

53113711044252
488065913239

53341399

214

251214151913

5176343578
811331331348837

82545172115

17642565011

5261226103
5261226102

1

344562433957
126197194188136145

142423442515

77126109986972
781281091017273

12331

362257583538

362257583538
362255563536

222

338654594728

338654594728

439437667784361340
9171231

302758672534

302758672534

302758672534

280341389473235223
15914

11

11

1062826712

1062826712

187267243311165136

187251243310165136
186249241304164135

1115

11111

161

161

415945

415945

11

11

647663
77671071165866

213238483026

503162622237
492957612037

12512

70311021165430

70311021165430

70311021165430

50371111164452

333274933646

333274933646

175372386

175372286

1

6968961686155

2311
5148711435344

75911198

16121135

31212

3837461182929

18202525811

18202525811

51151321

1

161

2551

211

461

538102820919483110
2

771341231325174
1

318841322129

318841322129
308837321827

1432

4646811003045

464681992945

11

46189484623236

46189484623236

46189465512834
46189364492633

11221

191142

380328603755342439
59181759

626656
211648251525

15251

24223

31513

2111

2

41641

34144314

21821

17232
222197288365145190

77740312
12629

12522

5313311

354029702221
231383

1

1721

4221

4752059

192981877

611219

431214816

431214816

13
366921221423

2757610412

9121412108

1407721221795116
2163137109

11

1512
12517395

11512275

12

742016167

63321459

1991124518

6571635
6591635

2

422624

31121116

612286

121221516

971514614
981514614

1

9715131314

194221655

13691562

531311

132106249348177215
213818

3251724

3251724

182471876944
1111

3241863

469855

4104841418

125941

635104916

2153

12

1151

2277316

2277316

12331
10577162224102140

33527143

57121196

161311

9269153

13722661023

1064935
613724

451211

811167522

553594894276

224532

224532

224532

56123194

56123194
2

4442352
2111

1411641

13

111

3

128822
114512

1

1421

15641281228
42186515

1

71121337

11

319824

12

11

1

1

1931
188185291612203203

4817301016

4817301016

4817301016

4817301016

54514168

54514168

54514168

54514168

5137711355458
4569112

81222271218

81222271218
11483

4441137

251339

1213532

392043994128

116
392043994128

91111

63822413

1132

910122465

415215

206122062

128136197424120120
22442

3544931526256
152046923530

232

44531

121

1

1

67101338

1

425752

54

1

2661056

5571599

121

11

91901002685664

481529146
91901002685664

5433301552728
5433301552828

1

9247955

11

371140212

24162049

151414934

365

1

385318591613434605

1

1

7827411417
385318591613434604

37111753

201742524879

2421218

751293

121181783

9258528

171537311541

121134205129143246

211833

38220335630

1

633971624346

3921161010

11441

224111132

121

44474285

25635533340

24438502628

310171357

348443

283693438242021949273172484337007442490035
1228001860019650562257167676129431

665131
24463840582930311828593049

12

12

237418304329397309
24463140522925310328583049

24412132442252202620732293
354336413405410443

1

449118

725533

1

321

113107

3

1

111511

318

671935615

2212

8

1401340198176252270
1461348206185261283

231

1

13

41

414155

414155
15

4145

21112

1

1

1121

2

1

1

101213

181

12242781440

87658

724639

134521537359
214827538259

8359
8369

1

12527
23738

1

1

11

2

1

411222

6211

26711382010
2661138209

11

133135

1

111112

6787711

342107

323412

3423

3114633

1121

1

1

1

3121

1

31222

151052

98182515

112141

43436

1214767518

211434

12611

123

1131

7575112

1

22171011159
221712121510

211

7522221020

111

111

1425396

198634

11

95318161

63123

35256

1

1

1461881217

11

381539
381429

11

1

2

1

442338
442328

1

111121

1492015629
1482015629

1

1

1161

12

98128323
98127322

11

127941213

1167154

113325
113211

114

126336

1113

31642149
31641149

1

3

1

124538373314
225243453828

10758514

11

1

123

351013510

6483430

4225

1

65168269145176189
65167269144174189

112

1261
8222516920

112

315134213

132224

11211

13

134121

1

19127

24311

21212

1

11

411313

1

21211

2131

243011718717521600575
5433163286

121103

331126272421

1252

813814610

14

1019134843
411114143

6827

792382

242690263338223222244
242893547605355506509

184270261104283264

1

1

1

113

1

1

1813622

3132065

1668136
166385

551

5410146

1115922

412

11

111

6584718

211

21731

4337511

1037524

913117920

727412135

2

126

111

23179121310

4321113

11416898849877

1

11623

1

1

4955813

11223

1857522

3424158
2423156

112

4441286

3362564956110
4101415810

71620121438

4536128

666214

81364714

4127101436
4127101236

2

412546394657
155188217478205206

13641

68324510
78325610

111

2421

1131

12

2111

11113

217232296

2481967
44102167

222

51412122014

2111136

3241326

12221871133

5661893
5661883

1

14341773

38417
14942218

1114181

652291

1191013759

103211

22512115

3355275

211121

61396106

77173813

241

71

512108108
512118119

111

937457

937457

7101473814
1

65111846

1435548

686567
235221402641

134110131230

2241653

14

11231

1

283323445316
385136786727

14336

111

15

41

1

2

111

12

1

12

11

22142

58

11

1

6111522

1

1

1

2134495

252213

82282

61014221237
3998414

23113

1

11871

32

11216

2026361173332

1111

1111

121
2026351163231

121021832116
11522

10615491811
10614491711

11

2341812
2451913

1111

4129738

4129738

133831

13383
133831

1

211855

211855

1171528913
7666349571789702943

471520610
1

12344

12344

35121566

35121566

29618641116
426344406856280453

1132013
141661643431

1

73371163

610911

1323410

12

35157

325964

11513

1614131231735
158213174420121266

36518538

34211314

292021912118
282021902118

11

751891013

639104

5119201411

61

1

327751962349

262428151055

31522421828

11611
1623391041433

323112

76132479

1333

111113

22411

471232311

3536

184159913835
54740114

52326321519

111

1

1

11

111

195322

118153

1

135411

1

25912

121

1581611272517
1

44220144

15312971113
15085689

344134

121463

121463

2
8741486

7631065

111221

242039682346
1131

42743103

213325

13132512831

434736

242227392691337384
2442

10514486
232130516244

13161

11241

514107318

611354419

261729332632

261729332632

171410241716

171410241716

193147914771
34589

7267616

15716517

3578715

49939119

461413105

13

61514371211

61514371211

7923371723

2415167

558361116

43820128

43820128

102026131538
140115227394144177

85311302417

181728222924

593063473129

2026351481639

131023201110

12719141820
12721141820

2

6328841585257

879151117

879151117

4210431310
5521751434140

1

46710413

1343

1

2

11

4683691

42746501013

202145361826
4232

1
1121

111

1

13221

13221

521310612
141642321423

11

135

12343
12332

1

1

1

1211

266321

142

122

11

23

2141

22

193

438521438517322367
11

181113441315

181113441315

181113441315

420509424473309352
10710296

8887127

8887127

1038513815994123
111

493896906671

534742682752

172625511726

172625511726

961591301337585
171940171822

324024481122

212044402326

19711110146

79111899

252821331816

252821331816

16119692888489
211

502843403238

11116648485250
11016648475149

1111

7507519281105659767
737762

11122
82229302220

391816510

391816510

421114158

421114158

7227148631003610726
585579765161

1091051281799398
15153548149

325323

1624

151951133

333346344845

13141725921

30241616713

152030234814

152030234814
672116249

91397245

3212161633230

486331163

283558321627

754740365448
382210413

82153210

233432

2510229
2511229

1

6194514
25359174314

19341338

42924372518

42924372518

492861664034
111

126453

4314

203313491

191516191222

13862

3613

2141

159146192226122236
917783905187

19212617915

1171111

67635186

24214

11390

3156

683316134

31112

112123

312525522318

152320312118

152320312118

76113811093051
297024231425

19252

18103646711

1028173099

3454

614731

614731

132315697

132315597
132315697

1

741012127

741012127

2
532171596177

261227121528

27942474649

13202123926

13202123926

131229652119

131229652119

54214778

888181411

1
1182107589799412061040

11

11

1182107489799412051039
25343451466053

526723

526723

2324

2324

21361

21261
21361

1

2481
79211101

22

5529

5529

212512
151414831217

36335

121

211111

1321

44183

2

2234266

111

2111

2111

3741842
58523133913

1225

48351531

211

722

1

43

1111

2

21731

111

11441
8314928788945

651295275835
751397307436

1

91023161
1

11

9913151

15191

2721

223

7562557

311124715

5433

1

1

3671415

3671415

333427

333427

352235
181240108118186142

331145
1672249711117698

130207847517091

3417102421

11

1113

6953512

23124

51

109921413

109921413

171116232443
212

9511151638
9511151538

1

643531
653531

1

1111

2132
231

11

1
22216

13

22112

211716557720
474407453369663541

3112

11311

5458218
4354187

1

1331

1

21
231

3

112412
132412

2

1

342360398266514488
429362399267515489

1

1

8721

1

7

11

12
23

11

11

7614111616

4443151

33553
1221

1

31

1331

131

122431
122441

1

191621463720
88725147

1

215275

12

231538

7361313
7361213

1

413181548
2212

11

411171226
310724

111752

1

46881254482108

91712765
46881254482108

3

37681133776103

681728919092136

681728919092136
13321

56138721687487
63918262832

11

4796501064454
439344994354

211

257

3

31

51

2

30

2111

10241451745

10241451745

171513

171513

13531315417
264316451771202914101597

10921684030

10921684030

10921684030
10921354029

331

597771923460

18

20814361215

21363119913

111771195

7151918427

4512924211131

4512924211131

4512924211131

102835191337
462459669843535521

10125936742

10125936742
10125836742

1

251
153522253714

112720242814

23119

324155633245

324155633245

51010261010

51010261010

4665681118056
176148193308188148

4222

1213

32

11

11

3341

1

1

812

1

521315

2211

1

6151

311341

72615121
1031117122

31521

1

112

541052

1

111221

21

122

1

2

1

3141792

1132

19830301114

2113

1

592135912

13

224123

61412

331412129

202262479

1

876377

31

1231

311

461512176

461512176

122728278

122728278

5481072

5481072

2771930819

2771930819

112
7441771074742

532545813019

211631251721

1117188187
1211

613123134
613123114

2

425543

52026181115

52026181115

7101181121

7101181121

239583
4036751127373

411518511

311410112

6412151415

421637

171223271725

4311311510

142521171912

132521171912
142521171912

1

21
151718161020

10121210413
10111110413

11

356657

52315

52315

4312893136102132

212545807156
4312893136102132

122

376688

11242821927
43283

7212613924

11

421

111

111

7671024836

141

2000761866836664792
314654352659

41219191812

41219191812

58873
100109153199170163

91513522722

541483

13714233715
13714233614

11

2112869

1392391623

610812319

92111181518

6258115

108192021

12101922139

101230152716

1

1

744539261942

744539261942
744539251942

1

398355652434

398355652434

26432882785

23392282383

34642

182735241837

182735241837

51114251124
273637754549

4242681

9209131716

11

11

939798
9391098

3

262454402824
1

131023201312

131431201511

1

131431191511

418557261551

408545251551
418557261551

1121

11
11401211159

556462

6356786

13931170676834

13931170676834

512158466940
64151036

231325135

22177296
22178296

1

20224232423

10122827194

10122827194

172633232244
1

162119161433

15147810

148201579

148201579

273434412933
1211181675

51010121917

1013613311

131782599

131772599
131782599

1

217752342834

217752342834

20151930820

20151930820

11241215199

11241215199

11241215199

877116391611218811064
11

12182834
869114590410318641044

58847711967113
6941758

61124111238

375629542647

9820372420

188282199284214207
593702683725663796

23221

23555

12612692213
8612681913

2

41
4

1

1

1

141

2

121

4811

1311

11212

5473918

51910628

151682

13123112

19889811

11

41256

1152

1362618522

443332171243

212

2242

84781015
85781015

1

2171

13221119712

62961713

2123328956
2123328958

2

2101012410

544151

73949324

2235

21261

341928272955

8236233

8101411

69144518

111131

21441

2123

33111

1231

2

119511412

6885773410748
9112810268166101

251

23422595852

11

3587511
3689511

12

3109913

61

473449523170
483449523170

1

11

3

355663
648542403544

2940718810

324030162131

4215134513825

4215134513825

10010566885862
812914816

141324281114

69627811

322710665

404417132516

122212
71812891720

42522

42522

1331133

12912

13221

13221

12115

12115

352234

352234

111936

111936

122731
222832

1

11

4063908065102

4063908065102

17
4063908065102

213641383056
213942393056

311

192447343546
192446343546

1

73107126173102140
252928603489383829703961

171166188273127244
14216210

11712213515194178
161723472933

31

441331

354277

161526231313
151526231313

1

337223

29623

12101718631

1481211213

4144214

4457224

6

21631013

431422

11

457345

121245

424823

1111

1

421

18313422

3212

2

372432511130
144102

4262123

32182220925

12331
161619552026

71143055

1358210

413665

511

45334

201533841936

3133822

11

651529523

10915161211

233017222032
115712071630149314431950

232380483843
151725322227

15561

112

24613

241

2236

113

431145

52511106

52511106
52410106

11

6266848587847551162
107152162155165241

1512252269

10181444

211727212023

7141691122

361341226

471618721

5210710109

433385

3210595

571815516

1312

1671432424

1712

6188261

452362505474

4541

411

1385119

2842573

331333403459

1761728610

131
121

1

8212372532

332752

13

471928102224

235853
234813

14

19828372220

2217

11125

7

213773
2131373

6

24625191821

11101643326

393472635496

12141631220

5

5

101411191221

2132124

8528112718

21274131045

1424

232227

4614171425

11122

21

11551

1512166634

8223644

494839

484527

21323

31124

610119616
5014211404219

441312936

123

2212

1225612
12251012

4

423560273452
131

7285711

353351192740

202230234431

8171571818

12515162613

373443224875

373443224875

78461519

78461519

761917924
14821211636

11

7214711

811774

811774

251928382143
65615410

6521046

21

3334
32

314

99123213

2134

25455

147146203201130172
572542742724

6631

7310238

11

3134

88167818

1461

15311

212031251521

441061

24225

132

246

1

1091521517

271531

111421301224

204333222943

11

1321

11116

31138327815

31138327815

355834
205142482331

751918614

104118221413

14193081651

14193081651

53381101065889
121

124

53191534

7418151022

171216121415

2223

17537381328

61216231315

172020162926
14124

4975136

12713101416
11713101416

1

5347607310163
131114

321

13885711
13885710

1

91914134

322
22

12

141820182423

21

67544711
67534511

12

5101516610

84549016410594

71618381221
84549016410594

5289143

2112

13207

58762113

332728154

871620516

23317

11776710

638993

111

1349145

836444
836544

1

349521520606509596

160119166184147210
343515504515485587

1111

1

66121424

38422

1

911

14113

9677510

111

1

122

14

51225

3223

24

231

4332

11

1

152030241729

1

154431

213

6

11131

15

1

817

2

551620616
771922816

22322

717202220

726

5212959697463
3912135606539

1

138249824

2

2

11

5127

1

1

11

42135421

1

866347411
1315261

735294810

1

15101

14835

165649

212

11

210111355
210101355

1

1

511

113413

11351

1510597

67776115

12

1211

231

1

11

11319

111

3110939

14

12133

19313

11

27

11

34
44

1

34

12122

2

2

4254111

6118419

1

111

76111323

661691249

661691249

378359715067

378359715067
204814262115

111

2172

13334

41564

76102065

7172961631

121415

101119177213107170
1111

9564311

9564311

91113170209104158
163431642446

9451

523773

8371067

1414289322

32

341

21

381918116

122

15101734916
1271431916

3333

174437

91619141327

78106710

1142

221311

1111

876756

1512
152

1

111

1111

1331

1
809480169109118

51181656
4948481578363

1741632

151313133830
151313123830

1

746897

35490167

74531

11899910

1221
314632112655

1336131916

1781981639

457494586592399546

457494586592399546
114138159223129203

1

678641

1

244

1

14144574

1112

56626206

883368

25688169

2

2

112

21

844635

6243583

332154

1

142087546

12231

828572

1131
8955134

7942124

1121

11

141229541834

1023242

1231311

55216418

13

1151

2112

11

14102

2

161

52111036

6247184
12161472522

61410718

13814323

193244

18272251423

243224

11121011112

12111610416

174911

31162

111

375571
495571

12

2233

20212722921

312311

1

34341710127
2833128104

615223

210

442342

221

121330202040
121331202041

11

20102710523

1

1424

13

115208749918681

1782023884

11

1

1

388042627

111

5512539

111

1

54333

1

1

1

1

4710933386762

24261319217

24261319217

24261319217

24261319217

4411525677148424463021452641
4383722565721420464549772235926448

7457545347634095235294291
92759764712493661700

158613398211990591690
538642332656260295

7

95417

611722

38221261213
38221159202

11

21

99737319

18771121

639150473239
639150483239

1

232130693445646
224129683335646

1

1

10

10

1

1

71

71

1

1

1034242922022
11546451002022

12438

38153249810
38153250810

1

2623313

627238531521
627138501421

131

261630692023
241530672023

212

139118641883637
145122661933637

6324

11

7958537782125
176133122323110148

3110372481
61204458105

1

1

1

4

1

1

3

3

1

11121

1

29952621
29952721

1

232419184154
232419183154

1

131053214

21111

1623818311

585542

585542

275526411127

275326381127
275526411127

23

173193196233106228
3213

324340741857

376822412423

1047913211761148

237323711894478215401777
657605540695404567

23143941

72

539741952938
509339912638

23221

11

121

323539762214

9615186

441216224

122711144

84621298774171

1

1

454829504421

876631421324

15610338

34139681215445
33133671125440

16194

1

302825313223

1382125910

71125
15

6175

11

11172423918

1

1

1101088714187151

116

11139

11

6323

2862743272437299303

1

765673614560
946479794660

1886181

14

1

8015850673536
122193651093538

423515422

1

454498276523254214
447490270514252212

211

1

211

32121
21

122

1

2232

1114

1311

1112

2

11

211

5440261572618
5130231272010

272

11032348

553837335127

56121274

56121274

118108755247266

118108755247266
114105735197266

4325

15819017319063182
101810131323

37243140215

84115845726103

273348802241

60724023111154186307
208558691420682474615

29

112113122247710899

23167399323

82556937327653

21

99392459261756128
99894461271458128

322762

221

1

26366241951225

315145703172
182916951385162311111705

231234327191145416
6067913841107

11219321213

472451341636

4655804742112

6767964034148

119196169120165198
204026244337

2612785

357757

962812

4111912444
4122018844

1164

13671922

6551993098101

15116

2411

11777911348187
750424330726296290

464249169285143144
491275179314148146

138161

21183
21203

2

1118712
12188321

111

11

131224161228

19191922199
19141922199

5

2761312281
246911881

344

13311041616

704314123

1

387296348385323478
181141177127111254

374055917335
364055867335

11

4

341625205342
361625235342

23

13151311817

21

118

553232

13

1

28117381235
28116371234

1

11

241816302837
251916312837

111

3111

132526251326
92326241226

4211

242123161828
492123161828

25

7966513
311494166131151251

814076555792

223445847089146

15113512819390133

10484731505080

10484731505080

61114966
475155434053

12213

1012485
403839333444

302635253439

87139111

87139111

87139111

232019311436
232215167375114103

141117881577146
395034502921

627447

37292650238
28252045208

54553

4

1

8921042
111371296

345254

325111
51

3211

452194033

2

2

1111112834

1111112834
10272634

1942

5767491592617
191631361

13611

5252262

3348381181313
2546331121213

82561

563723
9610097101142111

322531311214

322529281113
322531311214

2311

132525324729

132525324729

464438318165

464438318165

162979235490015833152306
36958420742410966234800849229169

154530321435

154530321435

5467777241108
238232248229153323

616171443084

463833263857

24177211015

5149611

392441411335

92415161513
81914111512

15151

36814209611177834597351700
34569119007710000932305424395966

3101571105172038
3631801295552540

9216

323

13641151

31

551121

158210

54

2

31022921860894107

3049851663208611828062412884181
3012541644788535427720312774136

3731184276434211145

16153444791015
23089625381015

69361859

925

3828527043
93667112656

5033175112

55251

1501371332676676

704631156153

2125115133

1

805552851114

110183493951532
116186564101633

6371511

62575291711

651742010462

24

32717714327061167

271730342815

8716486311357238365

7369622882152

2885865257510
262536423059

2651271

1

15729518623005183

595140371110

38516910522112567

292513

14349124313

732477954452880921492501
237739651550244910231067

514

3520251141112

3931394240337

171110452

3588594110512

50143091912

6016173719

2174011

1

1

2735173948

18421314619

4113161196243769
3762961146073567

352051722

7202333

2321105132

28101458394

17167661512552

32771967916

51

22342567239

484752564623

41262244913

10146171

1

10557795501271323270
11018315621319339274

2111

1293212

7

23

3042816162

3451491

11015114110975197

2311211163692439

9133931

976812

175668510

563243892520
6843431002531

112
1

111

1110911

32251011624

37103411602128

3825165611

5112234

112

547057943046

2750184218

218427

9273981284587

689523141520

762061813232

43461

18638214

182311

194271534

191291282

1011787369804101142

99775836379199139
1011787369804101142

8815

45212

2163721

17410514219160154

4203402

34111562398

173234251

172312341116

43172541

208117471

2842332442019
1435312371918

1472711

21

341513501118

44532

231412

231412

173722471436

173722471436
103422461435

1

53

1

1

1

323522782394356611311073
1388814527101401784195607427

320351333498289192
544848683954

12

1

1221

4323

1313162

24

3621

35662754197
35662652197

12

361652

12

1861322

3318164392
68101862

27106253

11

8131211

8543328212

1

114

121

1132161

1132161

2

8181922920
79116173195194118

128382
128362

2

13

1112

3

26

1

1

351423

11

321032

522156418

211

383312412214368
393312512214368

11

1

4221

14

1

33121

1

31

11

479607325857272341

11
9814692176124173

12211

12211

1

1

1

1

64242

64242

524411

524411

224

224

254211

254211

1521711

1521711

818123464

712112444
2521321

113

2141

276322

15222

1243

11

161102

161102

425102
7310962110112165

324815684335

263014632931
324815684335

53131

1152143

3053393157128

2547292852122
3053393157128

469356

11

44231

34431

230263923655458
381461233681148168

211724261038

8168727

12661441

11

102821423

1949423101

1041287

111

1519102477

1619199044

51

15713261413

242753633236

375251323482131170
7068951175252

26201521811
302317231111

43223

139771462491532
75481071811028

2

23

5113

1

5929266223

1

1420511829

1461019224
14481933

191

22

23143537
321961487

95395

171825181119
171825181121

2

4911923411
59201931414

12623

9746

14621679102

14621679102
8581372102

6332

15

7870162
1001342973142957

1618324312

86274

31504168621
42595280623

11911122

27431411681632

2029154710112347918617
36516115237010990

21

1

3122

3313

1

2425311839

3

1

2811144

21257

13132314

40

111723102520

20241821

1

1021

1

112259057

1

251252622

731520248

6121381

2

233

52140291611

1

51

564928531920

165411241419

1114132131

12
112

1

5413655

102925459

111

115

1

2

14

31

131210026181569648348
3411721192376855

1

5439174765
17132954

2

1

3725153611

1

111

131

1

626741713032
657542883736

115
2717

1612

2

11874

182120741853252

356537513236
355737513236

8

4725312976

3

42

21

2322

42302710687
3328269887

9218

1

1

11

2

121

1

161238

1

81432

231

1411

6151

459386234688420118
504431253785435135

19911

2

347

535434

1

23111

2

22
2

1

1

3435133311

21

1131

311

118

1

5111

1

11

12

1

17

1

5151713105

5661512

1

11182511

117
322425

1213

3211

31

332465291312

332465291312
302164231311

33161

17931858
20024310428569103

11362713

9591628

2212

81892414

20691760319

74492336209

153314531113

141510152028
131510151228

1

8

32401135411

561327714
69811241585995

321926382121

162716541321

111662351734

5127215
5137415

12

161629292240

161629292240

475044845443
191176159393137164

191886857

201818461718
201819473018

1113

681716264
12101817264

6211

8251850514
10251850515

21

8354501261677
8355521271777

1211

292431541536
10713213720453131

425353501022

3655531002873

25431

25431

311332
136114951713762

7862651072842

55412761918

51137

51136
51137

1

1371229115465102
2925183539

315740182938

1

1661114712

1015112587

511911621736

512627373716
302542852076424236621808

1

165411
1366711

123

1

253324281444

32

1

5511431
5611431

1

283542162018414636011736
263039961877385833591534

1

424

424

21

1

112

1

1

1910928

1

11

1

1

1

1

1

11521

1

1

1431

21

1

62131

1

1

1241498532

1

355651217784

11

41

1

1

1

1

1

1

1

1

1

1

1

1

1

4

1

1

2

5610141

1

13

13

1

2

11

1

1

1

1

1

10637
10537

1

1

1

11

1

3

1

5

1

1

29209332

12111

1

1311

43811

3

1

2

211

11

21

12

3113

141

2

1

3125

1

114

1

5314

2

2

151

417036586470

1

10418

720142856

720142856

467965595344
17498931007366

1281928362021
1281928412022

51

305441623565
483865

519433106
514317105

51161

212734211954

122130189166167215

122130189166167215

9578581664332
515263092

15403931

1

131061

293226332728

1
233182130135102112

2141631151096097

171915254215
181915264215

11

617766605454

607566605454
617766605454

12

8519156521014999689
122113241118

824892633977985670
415418273475410261

123
13

11

407472358499575409
403217403224

27171713919992
29192114320293

3

221431

21

1

111

1611
12611

2

215840275957

215840245956

3

1

11

11

11

182233162133206174
182232162132206174

11

241321191710

742243
221132

1

31111

1

1

1

27

27

1

1

111

1

11

11

211
21

1

323

1

32

2

1121144
2151854

31

1

1

4

1

1

411
31

1

1

1

1

1

1

1

43

11

11

11

11

131

1

121

41722979
34394237910

67142

19151817

415

11

1

1

293223312619

1

1

1

1

1

1423

512412

412412

1

134

134

2

2

8109331

1912

2122

56111

121

121

1

1

1

1

134

134

11

1
13

1

2

1

11

11

11

15261331
11161131

42

1

2
25341721226

32

22321719226

111521

111521

1783027816910052
15861234

1111

142282641369340

321622
15321134

1

11

11

4

1

51111

22

1

25151
252611

11

1

21
1

1

1

23321
33321

1

88217671375990
271923111426

332412363027

2817432901537

155019671147194310001057
316273350334112222

12423139620

574043844125
604144844125

1

1

21

25418

1

1

111

84714

1

84713

1141

766856411901614602
831905439992622607

11

141

2
1

1

95215

1

2268291

1

1

1

1122

322

111

21

1

1

1

11

2333133542
191

1

1

11

1

1

3

231892521

3

81

81

1

12

2

1

485252531555

173

1

1

2

385931643425
355730313424

1131

2230

1

109948819814361
109949320014361

42

1

2221

2

311

2

111

11

1

95484811542024

14

101213398491245735867
6472521374137

1311815157

1311815157

25556

25556

434655301418269363
1051606813259109

10512611

526421

5161

8751087
735887

142

271173

68932151322

3212

14242

111661468
201962668

9312

3181312

2710913116

2345132044
1443111243

2124

311

33

11

10451461

9359477

8718203

51612

78168926171114
58153774153112

1

107

1

4314

113

1

541312172

231284

3153132

28116142220

23151323109
469655

19941754

51126

221

1564757

731

668121818

201925422421
146188180257175188

541

384228501131
172224271125

21204236

133533
433733

32

232813392910
222813392910

1

72122351318
246444627534

174322276216

341521

139138914
1511149914

2211

44592
44372

22

92730125
15843332272

663631067

192218152212
789350454953

723443
101541063

313162

33243

33243

174724131433
174724141434

11

11

296115

12

274435312053
310114110

312352

247213

661011119

13235111219

12799601504244
31171

1055643732727

223913561417

21

113

121172158192119116
215429393314

13249412820
11229412219

3

1111

1111

112

112

1016131259

2225153193
2225153293

1

505073583668
555392684470

112

112

33181062

1

958218270215648288278370
8691781815404179485067647421128912

848658178769878424997597218266634
768651674974673618551092414

3221
254419449112161

9014724739123

16426918182837

13842422207335590128
65151089986215122410497

18332223386219
18031023386219

312

1643513684626

71269121

31785

429914511101135
428903511101135

111

23142429735243

1

67811067212928230

851582533342

1671516

111

24346944816545
24346944819345

28

19441620316724

2498310254315

5415563275

1361442731797

2685723466625

2574224710113029
2564224710012929

111

10691272608827653

251

1823612147608
2243612147618

421

2

211

17327835558824
12924335467322

44359152

323548509616130

7620642662117
7620642662317

2

7696186918825542
347222046

6353762612613528
6533963913915232

11

941

131

1

1

12131223

34

34

2614

1

1

8215028295710

361041636166

361041636166

55377812917118367
8125755

801623027238
8416432282412

422114

4036631198

611671927199
611671827199

1

15118330313814

9415318163813

115631931406
115631930406

1

1610721103
26573862212831931127

1893232215416915

261544238016910

285549221099723

1032117533911

307598267910814

871641637368

69532628609414

542688481499216

172352208911713

712199151855927584490946441023054
31377566535411343222282277571358

11118129138

1643941124

24303451635049416711352338661

197455084465759642

192544242036367523

19925015145

192628436664611710

212

5507743611624

2317603710724457333

344116041561232

8321679111111503

1164952530227938417724140730

1164952530227938417724140730
1154002501297898376823662727

3649463130962

1376

5101751

1374502982

94466

329965118145

78

4239834

11

22100146

5518215527

371219

751303241

19231

141331636232

2204583201010

117

3678885471275

2122414456315278441

310902399

7791698182032367

14

76011283871914

81

2252336110774118013

679148117258

612814373176221571623

5438415

1

65716721611813412

792417389278301669633

174911

31310201
313571

513

1

791771011271

477823332

31456504984325

1102273811954047

3

129828373130535318

129828373130535318

912

265458613501

111

394114

12863430422

1377253210528943637
104631394557021383114340

49311672928132620

49987148684368

12561831

10234738

3212551022431

1622011327744

89883942836855
88681942826355

122015

73013724423639637
72313514423539337

72113

458541321501138

6319473712128822

3376942218031919

2264161772928

3597201269866

3489230711737222585

46110033715317129

1

11

11

12761650207457597171

3495028817127569
9411397206404546171

699520391415

39111

9542

2393201587938

111614222

99215830297

17

2571413

1

312537

1133

11120852519747

9184411

33525315351
33425115351

12

370716936725089055190041855
157453048272133207649567

174934102

2852477131025
2872488131035

2111
11

1

1

1

15933819468333

160237171113325

561115212723738

10220813101314

50524134310866353

50524134310866353

321071218245
16921216185

161526

77152711188

7041363338515715

1

363514143016289

35440315478410

1924314325461

79116316726422657

16043305110384867102
17063608117434936110

113

143438162

143438162

53210235326
51195235295

13

1151

1

31

9132314

2

21

8112211

1

1

1

32

2245

687511215477
687601316657

91118

151911

151911

5127912558015762699575
198332449861

4334775254613322320558
281650864868491569530

1448941

6731299112755008
6151186102224458

5181413

53954942

101
4103561

4356

75912113516021116
79412773617422619

3566114153

132511211
121944

16187

1
207137

206137

150183711234
59510413019529316

62813
12820333048

667314

5610222941

3176552015422212

7443458282104

59312122023629814
59412132323629814

113

571119591647948
694161714019220253

88733101

88733101

115334461251134

1

259936059834554455
238033438330947348

739323

1557581
372

1250561

1026517181

6961714
1

6961614

566235

113

2033625

36057821207695

2946048134954

12340558274910
832405214315

33145612115

33145612115

7196

11

1

24542120466422

12493441

11014343154

16682030127843160621
13631314106687134418

258623181222201
12541315

39781425241

202479288173

512168

2

1

4593334421

41135126343

426721346915351
407680336212251

19411731

54064536598110
54169842668310

153561

111

3941276285612322
64818083819626525

2660111221
112015

1540110171

14294912
22847291291202

321

214

20944051191042

19156926543833
1735051050316

1864164727

881881345682

1

4365129503
4262129483

132

132

13233364923214
9122941815214

4110423180

4110423180

52458616545515
55360018547217

29142172

607841107205202107
21230546927856

32111

1

475929215634

94130527154

354651

446985136

19541

23422

1

12183881

28553471

11

142

8915034216

10331859578431972118061801
209297620081633559112

26433781838826

15130035364617
15030034354416

11121

113374647429

286348116499078

286348116499078

10321586601371818378
27438415192153107

21

9328925213133

1663361164610933

352219273712

11116860415853

49155661023

138144816911072
138144816911073

1

1

1

166228926721664

383574185113200147
14

22135864369762

7312380495477
7312279495477

11

89794128498

7373801004100132957
3154168739083995876384

53418054937

3

3181943793947461

91526167872177053

923132

48236235518

3933527436191272
4033547576195472

1021442

1

62753778517

2812034157544940

2941274603261524

346225915

10511538654727

10511538654727

3131029364016

3131029364016

669836253012

669836253012

701792628409

701792628409

28022326797604991607
818978352217347265

6215330333617

20451414235
20121414235

33

37692256

491237

104775

6123181

11385234

847037111938

656827462423

1840810102

619248207457

126551319453

1724136204

34423

5759611412

22652212789

1

13369721

1332513

502222143034

1171111826245

6114664235049

6320152

10311514166917

33731115711
49781215711

1651

11

181063172

212

8266854293627
8266854313827

22

1411

46591836275

46591836275

11319271533523165567967224427365212279423
5003121480248161156618848431789

15216616950227239

15216616950227239

15216616950227239

1

1

60458890031214541344

60458890031214541344
71412

251

76996

877615255233182

4014035861831062927

153781619

11

1231

4944893676149

312843186158

62721

10787791508216162668265751925427202242536
14017146601624867723613520973

261765347874223

261765347874223

12851367241964618922561
4

2

1

1

162171240107298344
12851367241964618862561

1

5516027802537821057

3073131125177485651

249271260107317488
264281274109321509

1510142421

126144189109217303

126144189109217303

796910692195135

796910692195135

68038393486895783042180217264511406105
10629581491817160711964909225041992218434

62871583231411231472

4941130186364

111

10111472718

1

1001091195893201

5636488782317471240

6413101

52

708011032122137

132347113149

145

14

11

114955573840111

393430506198643954

12

264336407122507624

746475209376

292929918647

21218525977267489

92412211593182977592304

35722

211

1

586711641122110

2

4544316781891025868

311

474593386252116243705123

2362063521022991414

1

29453934347

7056700881769380871082

191520104517

17421181031

31053606076086

10832533471885081430

27810712312

373419557136497823

9074865451652660847

313730134735

143162311234

397130174554

7361523

3

2121

33514831011

374949294869

394939166570

757910642145151

1172

1551513

7019371943836

1

1781988828

15983436

53521501952123

5239183157945

714050185895

21101284622

65066710643029271411
10710124441805253022020423990

214231327117320566

18911852269184234394243
18961854269684434434243

52524

11

394468548165663777
394467546164655776

12181

15029132983341311
14729132980334306

3375

4756958212469331099
4766958222469361099

113

692982381226535451357415583
666978971186834011280315107

181626123734

32531210

1221

2718153

44212

4183815

326345

10181471317

7123083321

6166843515298

1

3121311

2739301014819

83113102317

345249139659

191217105240

56679227171124

111

5565852681109

12515321291209222

712263

3

1

12

324523133470

6891731710998

293025192942

14201142618

16

1

92989851127123

1

1

79162988124

910740124322

28109932

14312620561329207

453403506206694775

565465196963

12113017180176275

27

447342193873

887546395427

4735721613953

55322218

325739939102095

5350621814135

444657427174

4967441441101

2941289943795197731036659

281624510432

113

1127

685560379967

392126361584

1417103141

2

810732622

6712443151291251472

70795366326310121095

1

221

275322394115590533

23723715441144130

23513

33428231086872391

1112

1148267

14737931138

22530131473155476

20314084243

521147

491

12310517954173210

322045164875

15218722675299306

837015121180133

22

322

258303368122402649

16117223157311292

281835168018

9731040142738815202203

15976075912788796795

13321414153648029782634
13531438156348729952642

2124277178

44104514

1

5722106027268114850666842

982331328

321

6597519532718431492

3223351910285

378372505160935859

435385572162542753
331437944437145448226755

738512040176196
909813942190205

1713192149

4710219
210625752996101331354722

20662387290498830064270
209924592975101131284699

1219

121237

254556196782

2131112

1214318

4

12

51271220

310911254
310910253

11

17931125272202447

5171092525

437406451153712567
453418468156728603

16121731636

14251530183655219672986

65021173622

1

1191135216991358323

9212713571804316535

303232299104502544

25151766143

32526943267214548

364336463171590680

2721298409

4475985881676241073
4395685631626071036

8302551737

1217353961

207175269157309468

135961811

43437

5860861042136

535064216362

9435623022871

121

16112732475578142

15014516047307283

19293343852

9733643161833794509

113

313829227448

768412831155122

1221

42

359355476145545828

54354

1358413652179169

1129162276

359417542205574765

42618745784181508

292143134779

1628619129952157

1118

665911335167189

175221199103218342

247725503276101034655181

841012245

1

19321724678305247

23314114839174263

2

64586225217129

15814312742268142

3211

9168711752756115

534111218120246

2935271110940

1

10115

227258327108363435

17163952826

30193064864

11210012335105187

79629430138148

1

1

1

18625628293311489

1

18232061439

337419479155505679

201141

69678695126710381390

10101211339

111

321

163320582446639915253536

358415457142524695

317368470142548685
334382481151580704

17141193219

14519518047205188

3381

3339642389120

989034358348206

1

4160572211364

29413492441

1

2257

11

4445357304851

11

5486357781755991093

3088697130

68397244950930661216313953
82501352720290

942986131635011802097

19431929278884839744131

22882654302998637684559
243027793190104840134737

3750643394103
294750318399

83142114

61

28255562321
1

28255562221

383221116525

331821126229

14421500208079327942898

4157

71032

36145157297

1111

2311

1

13317016653194132

8910142710

15477347502329220841215510343

7181022

1133203

137745414

212

58161141112820

25

10761092156040517312152

16744471115934

73073293926516981584

10109620

321

851484224

11

5

242

83969346134130

29268149718

54631111968117

1

545185188375

333405485135571822

14

1

7833719

379026124741

562562872112144031423

910621410

5952824341012029470

16121

2291

5133922121242

2031795126

450441517126664734

19116522873291308

431387496175718759

13292

129

333529715358

731168343109127

252353214830

284252321123525503

72521002465100

3931251417415

1

11

15214921158241270

270301400145516478

2

414400563163699841

621293028

303341176470

406156200862162246

725879106092

222119232081

3

5465837892107881179

19562836

514265305193

5123241823821

938811136174181

717848121137711611890

394255214782

445185298892

4634146862117661096

21357885936

4969772613388

10613611736258242

307372485113326679

19224057965504466

1

1627171657510

11235615

71649025135119

39523598727

17171972246

412

172239162846

2719521322114

6405677222848351163

10061230126733811431981

14216422981230398

2344112133021

11

111416915617

111015

1

529124

2

10412914739101182

171987134196

17

6855700098582521957614801

5

531697703165796895

1710156258

114

4572334

1122

202428103342

14914716751237282

173044214912

149159257144392399

54577129122141

922790118436413381661

9192984162138

9102072285

19

11751457146447217382380

18115812751185219

36

37204401957288

374147116377

11311811639175168

1

1

385477

866889431304228731026419727

1

5911265256797

375366459199622824
380431459199623829

56515

14011815764435261

834158620612396961043

1

322699222411243

821

989114

10712111438494188

1

221213173014

344913120152322

12

48721205091144

394843952664

337445480135580748

152316121035

931934137542416302042

465447362171251

213830166743

12711513838227148

1

333233144732

946488217118728205

13815817871213151

41184

241335263616

1345

12411514760284273

14317320363289311

1

3

8516013948103186

1241

11310015576255243

34808462527

242228274127334453

18212482742

8

1101021

6438115859456146659818712

1

11413125550223222

768114144166158

22

312243378135592521

11410513552141175

311

304374466156573760

14353572314

19211862426

296293300119741514

282

1

711158223106195

2256876

9010915770148187

211340300155364337

3524232310724

13

473845125363

818273217

11220616136289242

1

248125128561198122

16520533174232371

384464682306428795

11710416445168219

464795229388

1

111

323161157914

861149645168153

15715719291354311

20918176240

163232122657

32

51

14213315245305150

33352342935

342929182398

842441210

312713

16282191734

832

10358653044647

1

255269388125354573

715164512

15034855902465899984

1115

614422435

1

743954107130210211582

5

30302795535

2

32414843276

13123786717

3355224

1

17141183910

4904515991597541032

1

3514327519047

1132

354383500121577758

151629173041

76856189756224505292316057747419

18445132899332628

7602924211913276385

1

1179711432183195

11

13

12

4233411

1113

420454580186776936

56891013896145
56891013896144

1

7263754081394090466

79631008093103

121

49313192848
794958205763

301827112915

17242

242237113746

2314114546

1

879813432112173

168138240177287278

10313215542116229

81571103295187

13

81571093292187

1

322364433132431646

729916121331011221434
682840114229110121371

4776711911063

4776711911063

132450112554

110

4124826311825651007

4111

123739513302239

605029941063128769367

246289332152411488

416

2

22223091370

1

14563592530

26526631281253350
32329132791287396

582515103446

632280290882441470

1684635817825

2

345145176574

583963308789

11

28612751344

903127330110187

211010511041

392741404957

63731582828298321052074093

6541471221291

346365504176532744

791188843290119

11

447562187884

13426111217

1171251

7056681523102081541873

1

6867913150101

414545294047

1

14810010638503135

4848653559142

951278035164153

534451115550

343347500149559826

13916524269240360

15717319256204276

6216191613113

1646010934431141

5216356

72989029142128

359213521814798176262829
4186024721300971052115838442979

90189289530824771444

6233303221442077463
6983353301492213497

7558513634

24810410034492195

263941454929914388675

292156161781275243

36753548161452286

8622683111632537752

1955013053434119

103152567225133971144
102852567225033871144

3110

14517822366348564

121674495029044661198

316234302138701350
340256318141757369

24221635619

38311977053755862365

1167518832370130

326442537201653815

20776123781612152927669321285
19099115471519949197122719852

27412174537

29263148249

91631920

11152

20342

1011351

6504634911441849835

74262

370878445151386

393422520

1

12212251316790

773037914039

236628083112490

6372147

3848473012233

9215413

3021422110631

15114716

1820123674

953910732315113

13897691930878

13501176103121828111468

56058157421213451455
44448248017910301350

515123113

111848230304102

4863384261601800535
5423864801711881633

564854118198

1270751108338442731417
1243734106437040881394

425

14912136213

134511235

38232728118107

354411620140502514
354411620137501514

31

78978895235117171288
62563079929413071115

1418204414

12711410446335138

23262973431

251556066236515564988
259756866937515950996

8287103868

112020113016

21279482058384

262940103459

57691081862127

19321823673291376

48925137310418011121

71760687030214111300

11411

11

10421147140731312822163

1

123

223848268235

15714623063229387

263838113260

776511296125126

8847691844369

3609655801433582351

303638204565

252115354067568

267340398107466689

111

8243951931554319265

1009612233160168

2

56678527115116

121101

225215328131462530

135

713

4131251417959

111

23128027194284435

1

611076523143148

11182832811634459384

283746145250

11

11791119190847319242535

2910111412315

1371230

411

330381373114480667

18035724166674

141320163227

3215283014444

351526155338

748833101040510071492
748833101040610071493

11

152433122452

775815104328410211645

1

35492667

14513526868336324

92692124

16318833993311485

1161549644253208

15191992224

269298394115326548

2172267922

1132

291159174541179243

22122563563

99658082734831941078
99858086235031991078

2
23525

2355

2158210

1

23214916639145171

6121761927

121685175

49

11274

5574672813384

3256314014791864106651676

9653622851157

21443

3828661368116

12132561215

482167174343

11

1

141914124037

13192372427

10218663235380

14581540200673827363527

2

223141633

16417518567236234

17219421974375302

8480119831229377701600616588
218219328208360693

205401280118329594

267260337144386452

267260337144386452

743787149635222432079

24326833599234473

14204042246
222247332110323512

81728241110157

11313919960173283
12715521065191309

1110851412

363414

440436590164668857

840862128639013451646
841865128939013551652

133106

384403468200856675
370370450194815656

21023151

10131321915

28317

23

163133202111240348

320461734518513464554988
334063664678523767135249

12

922113186

121

1

7741911
5441910

2

31

2

182

1

2

326157

1

1

1

11

122

35116

41117212336

111

251

6

411

1

343352

3115

2

211

121511

1

1

1

15

1

111

1

1

1

1

11

111669178

1310

2

1

79

116

2

4374

1111

221105

2

1

5255

11

1

1111

2441

287457266963

2324

14143

1

1

2

12

1

11

6592024

1

212

2

212110

41

14

44711115

1

9511116159164191

13191487179757821352813
12931469176357021062756

25182872555

16142

1

1

324273228371

345367447105554668

52671052291143

21142

361654135846

3850722266102

10111916052157215

117211313

11424

131220825

6793716599022227804315075

31351361841730

8928231650333

1

272647152860

242842121474

11211611149381203

1

24022626328891231784728

166025122539

283635164661

23112

355444146564

33

324349480130614668

14017824993212448

13449564640967

1

723104174983

353320138358

1

249256433142484696

5657598512177491083

14251443191657919462964

3232354

3232354

10241135145040415492532

12837792336889

61010455

271243395109393559

251

3041240103424

7181922966120

24025035596396404

431292391

5836647942449321287

113

13318016951235307

1

435142828

365116146257

168221237140465612

7021251312827

1

1125383

16217525895314376

2

128182221013433

282229429116440507

142182133245

464451195736

284132111631601121

1

889711035190140

1

319443552139517719

26035225468282385

131

8841291526572

14861786170645917932819

446066165262

4252611864102

106832156

636875474874

155365628699110

4459672346155

14811416968347193

584370179360

5152774652152446815

52751995621177628254476637393336
1593531803345671917123961115480

138016671922286915052659

6428889322637011359

3710848271462890495420029
5019347623447832104

44123765465334

1088253653352393643

16342758275087017264970
2288489641841524270310503

48340249160

10325122174144537
151405322108219793

481541013475256

572581307683680
522481237480659

62112

545139

970492444184

19113726249368

543321913134

30761135457139
31881235972171

1121051532

28145734133312
974381517153

1967352516153

416

1687363517296

10623619660221364

4232013343

1373482132400

18534714873881312

241561343394298
261671453994316

21111618

381591849149302

7311545157554211153143
3706579452524981228

30103642247215

18412669106

26126472822233
47229885672483

18102392541235

3123915

244408393168463825
239371371153448711

537221515114

367232210161

1138361610124
1940361616125

8261

265477557132392704
3336767492105561210

1660462941178

5213914649123328

306339337131368401
291326309126344376

15132852425

12331557186970218002558
12321547186570217972551

1237

192

5611711093122277

2301144821595405205632641232156

2301144821595405205632641232156
2098435943536474730128793199244

411138140

453126360

122

3103212

1427171245

836341718142
1051361921159

21522317

623501911801882342
724612502352193178

10111595531836

2113

227

60322149142733849

1131

18318133692235633041

62227168126791144

62227168126791144

87

487881145

153221141156

24318164176493077

16341182

3373541

1105988139801562184
1055677499071502022

53164736162

12

3551654117811914907272
23113639049393465914

3401001038197

1073262713407

223515122352

2766194747162

512887109

2858

2293632472

2594140

2251

6218

5123531033250

763481581451511769

763481581451511769

3266343154147

18354574255676

97724242427139718635815
7308101757

4482783871242

27111774243203

38411472597981675

12317217779181381

6615711987194457

40185510043257161541
272503742181528898

31

112

348316

284535181274

1117

32116815

4111010525

1

127881016

64717121974

31

12

39150924687316

114623

2

315431

12165

163211

244139244057

163220

21223

1

213

112

52810131454

791528656144293

1666471044149

8617016848193301

8519018192148462

3685928624917711949
5621076130977212392974

2288614631222
451541227984393

2366613353171

5996966281233

488912030176143

2258548755134

2087552372122

265456413183283815

445988147285

17230931196234860
17230931196235860

1

20328730498210443
272412399126294588

69125952884145

193637644660195335596600
125020843208121622133499

80215178103187618
95280206120216738

534126846

103116112174

72241382189214208

185850267863

41191872281

8918513472251370

101360262121225675
331621354763260

461881216569376

2210699339

204021202396

42152984557232

2147262339119

21130693546185

2153902625151

1451159653150183

1

289431363410101543465285

11114119450245308

153

243127584016591

63618913198103

5383672140107

1

5383662140107

213017404123
236130733111279736363515

15835303529
93188513

23711164

4210111412

112233
424478367199

282335144969

132041201927

10298169262144117
281330493230

41411141115

11

1172521

5
195

19

11

382555

22211

124133

112

52511

204310706337

731314117
731114117

2

24741

414422
415424

12

4103136

111

11

221913

235521

1211

7066771628977
147125200329211190

11

2912
21012

1

11

101624

1

1

12101196

41185

1

34522

32373

1

14621

1241235

27105517

212

11

1112

1131

224

2821

711434818

198542

1

7331

2223

968359

31

15

24141137

1

212

1

323

1

1118

614

1141

12

121

111

44

6214113

63510488927739911129
199327402600206231083037

3

231326

164227

215291

154311

41

235033102338
245134102338

111

471427132921

113811

1011307185

112

111

22

1021

12

231336321719

12

31122

99151320

13129142513

2

1

2264154

2584113

12

111

2

21356

1014196641

2111321

7112292323

1

1

21

11

111

3344101

318

32311145

14172

23314122
2131492

13

1

774398

428931
428952

1

2

21422241117

21

4455

11

6

13

1

63221

1241

11443

143332

8059938116701260922
526230525236

12112

111

1

11

12

1

11

1

12814121020

9

536629441476886581
6678647025491086786

2

5513486122916

537814139151149

623

1

1

32

1

111

11

1

1

1

11

1

25

2

1

13

815131630

21

1

1

1

1

1311

1

1

61461

1

1

1

1

24117

11

11

1111

2131

503337227252

11

1

21

91118142113

1431837511
1431938511

11

122414

53712109

11

11613

1242

142

31

62536

82726113458
13

8272583458

22121

36231

12

4911

121

11

33215

21211

1

1

2121

3101424413

3235312

21144

1153

2

253431

1

7742711

2

1

111

1541

2487117
2476117

1

1

88101314

2211

1442

21

16252181127

1482762715
1482762714

1

12

21

123224

111

5152

2112

1

4432524
3332524

1

1

111

1122

1
726512234163197

1

716512134163197

413249513

1

132022123780

1142

121411

457993

8211811138

4221

1

1

1

4111041212

263

10143021419
10153021519

11

241413

59122418

11

11212

1121104

1231

2211

6

563476

12301011186

1271351711

33

1461

3144145

1

3545515

1

134

1

113

1

3102216

124

121132

1

12

10

311

4

17265254
17265234

2

116

43553
44663

111

43541

1359410

192181244
192181234

1

121225

54103

325124

412812382620

412812382620

33587948421087510548
11344

10211218220456141
38112510

12203612619
31

815928

420182411

16
332762971566

11111538741

22164743825

363237612630
1

161721016

93192579

1

424243

11113

1171578

584833

11102524313

68222027

523416

71511813

71511813

4912336564510
3243671632840439392

2
231010104

2221

16331

22372
12372

1

87157326391
9315934527020

5224222719

1744

37591011555882
3099386552722314358

4213563

3112612
3112611

1

751121019

8419475

7131135211

234679

161

475621071312416
2793631081352616

2318142

1

27141142
32495575

5354433

1322

132124

10121071314

291374

7241844

1532

98143510

189742

7565510

811810711

31131752

38252

22

313813

1181443

31375

24513

913411621

1

332212

2113

312672
5212872

222

781738610

3

218244

34344
314364

1

11

292025

1

33513

712164313

25264

717184914

21121

8612

17122

7411386

12371

1341

541021144365

6741133

121025391115

3610338

2241156

2242

5292111

3784741082644

3784741082644

212034661023

212034661023

1073132813

1073132813

65791088

65791088

481955776986511064478309
50648762103107

68151128184178128

4464207
551389013913883

2511145552631
2511145553331

7

201824423724

6515384821

131338454045

131338454045

293469726145

8923392216
293469726145

122

410113919

55121184

6315531

2513111

426964

133186199237290298
2

131622433036

131622433036

120170177194258262
263243326242

14919108328

449221613

122833501740

235528343550

781511920

101388816

121078138

121115191545

96112137233121140
111

1832501644943

1632491574843
1832501644943

2171

282522312851

282522312851

505564374346
2112

29361425167

191850122637

5658957310099

404860445562
5658957310099

1

1

2111

1

1

1

11

1

1

1

14930254134

111

1

352385473408287345

181822252116

181822252116

326353426344257320
229251162189105177

1041426187
1041424187

2

1

125322

71321

911

107102811
44

63102811

142

7145141813

3112

41111221

32621

848101311

335446335047
335446324847

12

211

84771513

36635

1

173363

12263

231

4112311

1762

814253999
3222

52112225

312111552

262729453432
120613201684149714222131

302838572144
297379355329281417

132214423240

352273794845

232137222311
252237222311

21

563925103623

182520171239

2612834363761

524968243087

221134171256

203312253011

54691363852224
131

49651212737211

5312101513

1313118613
1077638

343134

2111

5553751337778
232221

251518151421

141421923032

142134243124

221134452116

323223

7918321412

12131151

363145323443

363145323443

11111392020

11111392020

191934482844

191934482844

431121212
5565878391116

3811111420

181331293536

112015121327

192129191721

129117162116164277
8111514107

13917121718

8202431924

62656949107145

381237102183

698911188105139
257267354250348450

627101169

46123

1373148

16810102218

991681325

15111

7551635

23635

2731582310

31113

684694

14182131118

321839264659

3311864

26163392363

162025132933

111427141434

7141391710
7131391710

1

5451921006888
25123

192129232319

9713161913

151237531737

91187716

274466462845

274466462845
274466452845

1

213227313759

213227313759

526847282393
625337

285121161450

1815219636

11
141430303031

771623229

211

77115821

322537643827

6184887

262429163020

322450624258
751327621

1812

161524212628

211222

7241167

275131803990226737844821
4460555910070

18297311914
313845554647

1

3232711

2113

5121388

452281211

313337484352

313337484352

6811273255090
176197216110167282

272568402252

181212103030

544548232693

9315123917

44107710

44107710

4548510

4548510

142208239144244275
385449266287

14729291116

202860285639

122725183823

16414131017

428862306793

337311621176406813

337311621176406813

7078675412884
412236348959

31252135

172626145

413172412

213872

22411

1211

151715151624

151715151624

7685130273257

7181124252754

7181124252754

211

211

1113

1113

43413

43413

43413

381522606354563629
182121442551156425903097

3885139

156
371527572211761758

370527567211761752

242655223287

131019323121

251

791391115

3351334
3351335

1

11

2312

3

2261

111

346669345294

333213

9111731823

205526171733

27420132031

32212

172720213150

944

251340395339

27331581617

11

312041192127

3112

36531881215

12114181638

141939525311

12623214

1010771218

53

7623171518

98811107

211732

306068222686

254940152238

161716142039

22121

98718339

12141992424

5595129

111619152924

12253114

127951010

371019233120

1517103228

1254282

191037193113

421710118

182296

971741811

1

5147347

16624131440

1511

764653294380

447620153940

394544

71325521745

331532293636

628663

171221112036

447631

1912122632

11

191312

6618101220

3141136

22183272343
22183272443

1

61319221231

15141482025

2123

913241726

3516101410

11813231125

417232

835375

14719161433

31101382

36134

1734219525

4384112

303328283727
213120242826

928491

13541

10122082732

814193697

18712121120

251221321033

151228142050

778712477101194

312443404454
1

201029303533

112

22473

111

81292814

4663813757140

4663813757140

11

11

11263119391498658901237422280
81624361419

81281
3329751223523

14102534185

14102534185
9101729154

58531

191149601618

191046561615
191149601618

1343

2011028542148
13281026105

11

2791016431

4251059

112123

8515783725784
3712885

417535312238

417536332741

5698761114749
10666108881421249551177121609

6161012

106155131177108161

4671657

421147811

133121

15179531219

213137

5434385

11

12

37441

2112

11

4264854

11232

327324

435279186498

222151

13475

101816554721

1344215

12

235616

52528236115136

719736

137483385

33231

413131

4

61815166

141117111612

114121367

5111315919

35616

341

13822

132531

51114239

32133

1

151331141320

12

671628312

241

217427

241118251015

232518172619

243210251617

141244

39615113

7481813265116

111

7111552010

125101484

71133133

6134423

1

222229103855

19842

11211

1291133

102225192222

917519

41152

111753

74138

15157462014

31

112

304433122959

1251

711286215

1192235

2112162

313176410

235247

131524

13141880548

41114884

5422814

5511139

4243

81226112214

2751124

5539

422337

541010128

7614543577519

81474

1

88014335317464203

125323

10108171122

755145

253931466

431014

27206929

2128

9142491424

33121

315414134733

142123

12132

61779844121166

5842611

451720442915

12112

11141

514038

1441159

86509365127162922991519871

1121293

12211

42510877

1123

1671522814

116

211

272447

156251899

156251899

71858146

71858146

22521
141828191823

584485

982210817

191513142023

191513142023

396078642240
631314

6502814517

122919611

1572028108

442552
223154663231

11

161911

1112

1

172745402425

81483310
434847532867

11159321122

1261713719

1213135716

111317271322
12

6511542

6511542

115317

112

1133

362434

8214

12112

1

1

111

111

33618196

33618196

338447493681
242484240304258221

514315

244332565527
872413

4262327910

12107254514

1231042

11611

1

1

41333

11

11

11816110213210832

21121
22121

1

318423511

313

3111320122350

321

1

121

121

16402012159

112225341827

112225341827
71114111322

1

49112344

1

2

19111510119

19111510119

5810161311

5810161311

109011061579139013131494
161618181619

12113
83561091196676

583464925550
3151375

123821

202645613439
45111367

314581

11

2173

1251466

101724211125

3451110125

6139676
242044271023

91152027

96201110

124159213167182180

124159213167182180
362845144977

4715576

28394724916

8149153315

478732

66183569

11152663730

426107186

1041881210

71171049

6210364

166152214196174202
135274

495528333618

495528333618

81329402219
283043534343

74441215

21131

9128538

21151

201128191539

201128191539

101022211524

101022211523
101022211524

1

302647354028

131522

6811171610

17312661

21

6102271615

731022611

731022611

211431111235

211431111235
181224101024

3271211

151726303022

12

325748

31121

111113

26185

3421314

356461

32418

253628193645
154175210165200283

155
225546203447

75771019

58277716

1041126127

494563453887
3172

343835172067

12621281818

91016162019

91016162019

351629473952

1569514

201020473438

201020473438

141328183332

24319

121324153223

1

1

532531789695645712
3293774

1114129915

1114129915

162321612328

162321612328

1141415514

1141415514

172019111216

161918111216
172019111216

111

422536172327

422536172327

5060124554675
1315162511

152841163328

22176737836

15814342315

15814342215

1

638147575052
273611151612

233211151217
233210121217

13

11

131325262222

258256429358403382
5137926510143

432951246761

181230552019

5280999870125

114356813

1512130189

475752457762
242430393436

23332264326

312649354250

416665
463864414484

281424131235

81715121423

6619101221

4208874
296753575531

32121

235

1

6111

83226158

11

163132

164253

175538

1

3667101

111

5722182

176152229261129141

6131324
176152229261129141

159140207212111127
53741251283343

2163579

4233276

111

414

1

212131

1

11

1

11

118612146

1

6261

725127814

4332616

1212

1

1

481238441323

111119361610
1

106815610
111118361610

155123

597

110141064
6672118947584

232932221718

232932221718

1131
232932221718

238241

3444

1254442

917159514

43311

43311

41037322518
422969595161

1

22

11

3

13132241432

3

2466638

11073

8529131122
5518111119

2723

4

1

219185843416752746549
258131910874526476540196445365738

191720232764319418991970
7221210106

125113411778167511431318
5311252702730

132522461535

132522461535

142151212242124152
425380505468

324872293054
294872283054

31

152276610

2624141735

272444703115

451646472113

451646472113
431635472113

211

2152815513
2863623922

7411

14748

201433364237
181431343930

22237

433449994437

433449994437

384394485050

171029222321

213365262729

424520543400296389

234226252331

234226252331

20824921014762128
401478517375273358

8423432321
7423432320

11

111419261932

447046293149

3133

352341283419

2482

46279179

212637182638

415169117

271940301828

11391394

422

16472761713

7115228

596769805253

596769805253

829011218310179
464652

303840455740
313840495740

14

4746681283937

1514207541
333082522779

972818157

993427731

392188408271

392188408271

422118251338

422118251338

485880585153

485880585153

361754552218

361754552218

4
443895819689

192948395047

25943424642

627893907173
183827511812

5125

394054345361

20720751816
6596609741509746646

2

2

12411925112
16422032214

2

1

2

1

3

132

11251

1111
15161778

111443

311233
321237

14

3535233478

3535233478

11
18191345628

121464136

6564222

961711676

961711676

10
1371551179

13221

3191295

2123

85258

44712167

44712167

112142120117133139

112142120117133139

136138412
75321661902935

11541

11

501011913397

111533441115

69168214

69168214

842517922410341
300236474732422306

311

211

112

2
11422

11222

2

711318

2336
2346

1

21

345217
5745619

2742

2

614861
514751

111

111

345336

255

133

241676

11
1111

11

23455
231655

12

1112
1111

1

1

513864

2123442
2123431

11

11

1

11

117363
15233

1213

1213

372565467170
352565427068

1

1312

1

2

132

133

133

11

141

27742

1

1

21

1

1

2232
66806459424

66786259392

11

22

2453225

5256751126855
57699812183104

1

1

1

111

5122361448

2

1
2

1

111

311161610
451433822

14272212

2

1

1

17551

17551

3511372736165
385646

124881316

51

2010159544343
188049483940

17312

1193311

22

114
1294111319182375936964

325236449464243260
453778532070

15113930269
15113729269

21

76112476

131812134213

751225812

40215827422

891264891718

9420282719

11192829818

1937252192

332638433433

374064824138

413222

413222

44136955
316111

1212

1112

1329

2211

172

21

21

1111

111

1

148150239291119109
96086914521834685696

1613

7349110885649

1324

971336195

23

34393153

151183065

1191423135

851651

123331

611268662025

12

1

12101148

1131011

4473224126

1422162253

312848801215

181533343413

628714

2561452

242011193

151236431113

11325611012

31710101

3

4

74914419

66121184

2294115

21211

661315615

4065968912

171

302533303040

1331216

431366

544101223

13

11141617415

10141353

1

121

1124

2122

76212658

6624844

18431027911

10112271

1221111066

1412531

1

65793641522

4171672

41

71302622

2072915227

134034773322

96151869

19334038275

11410413

5881639

12

4318881

11671055

1211

712311221320

1532142

1541236626

215322231413

2127952

10511251616

18338471714

27182921527

125223066

22192449128
1389140016661942850885

105471891659181
551321

6826130905448
23

331135232715

2594737720

10646272013
10645271813

12

322154623532

322154623532

86796210191240536564
337151

6844821233837
341246315432135208

18153878723

534968362568

4132531

211233591123

6101517113

1111

1

1

882023151122

576921402224

2625222776

6322561092221

6322561092221

357606490518316230
92621171559747

8339181418

6555113

25630191118

516557476234

132511

1121

2438447421118
2439147431118

71

311251

21852323123
20850313020

12113

232120261516

131115291519

252325281517

721811

93211

21564941015

11
1038515116662105

504775963129

533875693176

289189377419189213

124351011223237
475

1082867592123

12727581114

132232383026

132232383026

191638441112

1

1

181638441112
1716364269

12253

1
4536101944134

17192911516

1442430237

141248531311

445668484086

445668484086

891119138

891119138

361526542210

361526542210
36152651227

33

1014187314
10618357692219

2020304273

671496491

931631

112282082413911

111282072393610

11231

249530842534389156361075883460127
54162110100971683620861674

37527714654460457255
91618056205195024888436742

231241
45421372737773

917501262431

3425841354940
3324831334838

111212

1

1

1273932228743811101811
4043788051589370291

2217

132

141

251

13421043

762757119

194023462820

5217911618528
2717381027326

255359122

26215511

1

131

1

281

1111

27161111942816

59351042634833

14635232

265212619

102286284

962496141

293759883038
281858873038

11911

342993214

2322431043415

1582561

2519691391426

10132741113

74171546

3

3

24112144

622050904117

64521062715980

4448641153660
4147641103660

315
5

31

1345

234

2

2452071

121

13

32621

2513972

623820618

2251829337

2115175

42

541674151

7428621412

2924741353619

32643

823113

12261

1410936

1552126187

80462203688037

661643118

15591311562183145
171550782629

2553575

111333441026

104172489

57162772

6122810

18940792118

14151922

241319292523

32103834

161524621213

74412843

733053411

1591844

211016103268

211016103268

2423931544144

2423931544144

81042013081732475556
925021642884193

331663634140

261532873025

121187775

7828441010

282543703456

12203273157

1328964420

587257866817250200

1

18

77110921943097
11

5298481301064
5298531321164

521

251239611932

62724512702579562423
133413736429886

21

331942993712

3811

38181131643843

14152438109

23840120314

3771

1212

1424794215

46181061835425

7724751823728

51942

2

181241119

318801853113

41318521610

6319992585856

101431393095676
91431373095676

102

2817831755643

6519771213742
2184987237

354181992

971015533

772
1245428458114499

23201142005038

93341673678857
94341703749261

13744

103622002708977
4423547881081342341

232933321422

174

18393240179

15644382812

603679922125

1533573186

412091011

512151193

311054731519

11449951013

7061871294875

23316

178151

315853922211

181821111536

151348871118

113

701292095616249

142814311721

561011955304528

43348367
3922028911663445371

28737863011

632546614

9275235348147124

12544811518

1146630666714296

6142664

89361311915568
89371311925568

11

413641343823
416651343829

316

4333771614047

3827761574047
4333771614047

4614

1

3738641242435
82621111985953

11391044

2910212037

281125155

33619132

6088352860109761084668
2007199066492323926741846

483333103113

40241012814757

41240321119

71341123464072

127531672013

55341915667776

9101843193

17241444707837

27114414

32322

25

36831737812

1241

1411501464829

58101255105511762

1

23232869122

125339569

8123

111

111

8132274167

351642922823

711301142066422

3562697752374366278
3532697692363365278

541

315

2

6743307107812844

84494776

114461362383062

54312277676941

1076115548980105

45361695017862

3360457212314119

12122667918

21

139742202837

38221395154449

20375334214
2321

1127141012

893514151

11273

6205727511384501322
49401012095348

1662361152711

26510015628615874
2659615328415672

221

1

21121

2815751103614

1112

13

251853967

12813241010

141636481912

1

14291

376766952218

281554805725

831231161755857

72143386

1

17201430147

2556631253031
2556631253033

2

14775469132621284
34181414045015

773923465310647

3618942695622

32225510562062320301
337882642736

105882884939086

98914317869066

8669249519113113

101626712811

101626712811

4538911512931
12

263251722222

196397779

79689119610560
324190187397184100

16647311232112
16630301202012

11

1711

1

26141510244

241

3178195

1

12991

11219952

2

1

181225

215610

2151

134451012

119

17164

104811563149563
371333131

11651021

23

100581362688061

49661083027768
346253469860363167

1231

5381542

1

29174

34311

134162276

1942251

1042013122621330

2

21311

62111

5431449

1119

11

2386368815

512148315

27323064810

33131

10262931

121

1

3696352195

82

511

16921

1

172801101813
187

42575093

121551810
121552910

11

1

1541641154

1341430154
1541641154

2211

409621295726

409621295726

47815039561216207280
153633634236257

73261051193154

108591421523034

826513424155100

1

151289751042228

47113717677

4128821103876

4128821103876

88491692344477
4757344576291486730843219

3026239152291163621612229
18315622435074125

22
59261081325339

11

371583803536

22112251163

7157841469269
412445633955287311

93921852842957

2614731062928

2639721305

28551415411

1221501221978281

12123052618

4394234
54135734

11415

2922105771238

3667761181625

3667751171625
3667761181625

11

11

8951851353664
336233528935209276

183432752545

63311352463535

5

674213

90851432384467

70251292366752

34920746757812099
391674962311

65371051654824

1301121631603143

5555481129

60377176612

4654871203151

778
37781

31

4347801123150

31
1307223148180123

3227581212646

4617802133541

5228931441935

89341711774447
415304727715139197

362259842137

53517489159

1201851381393453

14353453322

103231511732229

5132358961028334383
1236527425875103

11411

21332243320

35104412

2

6815301122

11122

24388383262140164

57204

181243542613

8102524185

32

271

3262

2173

414

19223631612

523101

531655781122

1962391704028238

1962391704028238

6242532713
1061581392929778

612327166
612326166

1

312134662631

551639146

5478321253212
58107411283422

4592110

2411

12111722
2119511233727

14819672222

1

692039133

22

22

1671537365226551423
118127

1211164494349483382

45362798656141

57211561795134
111

42171381431731

1441735343

104531172477069

153995221142750809844
932151982714862

1

1

433822

2

13648751754138

35197225

272459104269

15

325150265185200345

152051931928

211544762035

1572

13161311

152792

25129105

8314311

100139879515425

1

1112

23941711919

592171177

38467046924119

37475117144

2858687266

191023501210

13

11136

166

6

7972481121542

19323

11

384744643418

1148322

2712501391620

248567

111

6462744

111

743356933140

121046371617

283159921448

333186366426202139
92121542

32114

32114

21109

21109

1762084155
179191872734220

12

1611115616196

1328479374

2931096621

2

1

34

1151485
121022147

15862

12

140162156115156117
1

4

4

136161156115156117
1

136161156115156116

6526569210374772048116262
4772541172754266285

121266121964

121266121964

7698313
1028916015697162

171045553429

78731069360120

31923116

31923116

14422826823190160
13295646317

303946301828

50128901005246
4012676934641

10214765

513276551769
513075551469

213

291159951012

291159951012

64746441025
2317839

32661

5922330715
212823

5721122512

265107364315217160
15240311614

293484849

23966666516

485779574257

312362354625

1191369784439

934773

934773

240522552960204017592240
803106813808237441116

2014216510
181496510

212

383457403450
393457403450

1

21

11741814713

1521254767

12

221

233976286859

232046181331

287742261426

561932

215123344185205268

1211

7127

3

22527818

212

22

1

213

1522

2

75106113668894

31

114

112

251127102227

6

2649154

13

11

11

5

4393

864287

9011895745978
8911894725778

1112

1

668120190140117105
675138207147127106

1231

2110

418153

1

1

132

51176810

312273

624817256

1

1121321117588102

2

1364777

3

3864886178132

929875575041

1

1

638573

1141

282257301731

111912243

12411

231

11262

124

208320633672269416272061
381308756422202301

94133158914383

312494503056

220274284196108217

243227350346214144
250244361357229147

11

11

1

12

121231

12

2

242

383281

13211

247125

1155114320611199

1

1

326921

816416712178117

63711391126571

7732382127

302061582337

61671521027650

978119910386136

191134252325
15624221424

3561

2131

121

12

3

6122

991413134

1271351025535103

32994522332
32995522332

1

5355106944752

551019

3012101682844

726418199101128

7256121613859

64331121236483

415155412950

4166

1917590611440
722001231186994

31

3

1113

2

11488617

13

1

1111

1

321

16126

6

11

101

1

214

3

21142

65104411

11

1

1

1

512313211

11511

425457527539
425557528140

121

4

7250869
351370291860

610893

1252329

2184918
2184818

1

1121

6722402
13670198210122159

853194778082

453282934077

9911723312787141

9911723312787141

11
183112344218150202

3928102852957

14484241132121145

11418151118
11423191121

121

422

9231737310
14189325460114149

782417618743101

54421322366838

592994504165

592994504165

157818
90881471518148

566962814424
526949724222

413922

331478623616

1791401608574239
262166291677

12

4

11

152118945058161

20189733972361336505260108417311
13147364924562460031031464

6148931471710

4114931451610
6148931471710

203411

1

106461817361932477705
239613083752426411691546

107221833

6432138906689
6431137906689

11

76461071264077

2121232183449565

114451982146037

22181071

24526411514
24527421514

11

6144921091943

6531961172732

601863592126

90421521784568

4422951144235

17411430737878149

3763115663043

342330471416

311960733424

293111978

121411

303484688

534294925678

161483821

935761442122
945761482223

1411

4631237929458686
617301

204393174053814

130402122731643

123442462373129

1

1

1

3217110362
126422520222307411186

186593113612541

153352643542030

359304865255439

1752129236321537

359795986019137

166858322829697727709342292106
3686666167520635281136532

3

8814311531

75858379134860121900487247
75651379134596121708484247

2072641923

88919217712561122110

107711451927217364231114

9941141338187111068

8819254163081421215482

3575108585957599251

5090798389815129675

483122113013136848

1319292

4439985313296523

58429116412976927

30368454925079248130

87840137914627938

9405463

226776305028433414

7236

1180672096237118882

48513886212719232

174312421

823851718141875101

158233277425714836

426577

173724

7732163129771246513093

1048110227622459544

10119

19221132955339421789

11731372200235618566

725521783149041473371256995
281511197533576605458417

5537157304102

631280810511913414093

1144118630248231130120

25814804611001510240

217584594553335

141521701

33044063231826962

40040790031026156

52132103335269

1219

2992025503118017142

244834266542040

3210136713

12

764191817861326912576

2709104349028059701497
84462669146412122019971377

877521506175812036

158203245

911711

148

97

1721384524

112

4321287961304173108
4361288331334173109

437301

4181832932

682084411415

102336042

459441

5222791

13383311

11435442732117

42123341

423

3101766931155115188

501728282

9194312

67252096161

2532621113324

8

5629711521470116101

56381977102

267544619772414

129502583433131

147398032

2781495598798255

1853611152665225

315101

278121112712810

101312

1

103192092518122

20234111661

278723106147

1062820523961

3892628091171136146

161833174367725

1251622812

331361372312724

5423846145

1951613

841411013498

14787343771286

1

1

1

7813241592297911794

36427787421986033

417477187815761

300119503709140134
96052015032278488398

4416691341610

779917020413624

151

2

8340751492935

47219616976

2714741192023

1912512301419

269793093656687

1

851161441705657
941201551945858

94112421

266954986864048

266954986864048

214403079228594407502896734780
2766226737353507505216503

462016236809422131128601809
239438307383155213

122224273812

3262133218

3262133218

1926716158

4571
4599

28

4213952036

406515509737221345124951189
407315512737721372125041196

121104

12

1131

1

31

1175

1

1

111

11

51

1311

33162327422

4812885

2163

404040431755

69201942

85351421052055

848121111

3651404239149
5893656853175

1

224225251426

13

13

597549908897408664
163815722254213611651694

17522121514712387
15713121313811784

18902963

23829515567

513413118

221238

23424217226

3831627

1709215715373122

33334156

371665453857

3813866522683

202129251320

111572516

11

3848117604352
4052117634552

2432

496259231349

5830221197

501328313219
621847553319

12519241

15113

9243131430

212443362536

274254942751
274154942751

1

2021728225

242

111820113

4752947811

273251452244
285055483245

11843101

11250134167102126
11250143174102126

4

93

9241517720

354170493166

272228221430

326541

232021161029
242022171029

111

222
1073815127111379

62134463

24949861217

371961978051
371960968051

11

3882642158

435276363219250391

435276363219250391

6496102713666

113511

151332141129

488267522436

251191339201266469
126314551924150217623834

15171697

386044232868

232157263279

124371

231541273775

271947329848

9539471919

181831432236

1011131119

212828495464

5744674741104

2143093632645601906

221544513031

4535726784109
4535736786112

123

413157544873

132101846552126

342252343780

156029282334

23833413476

262838376551

9812551622

37116671138

357378507454

64841066962129

596314412349101

141702928822

131935181544
215194346287108220

3464143962816

522650373564
552652383564

321

113851161353096

170184290210194245
229620423378287424942967

271245611431

482839271070

966159543153
916159503153

54

136301158
1993820619

6389111

424451495864

3830192818512

425356662638

1072320535

9419243510

37738454237

312123382220

1379213
35824

1219

73561281046597

18638165859

1351541628455172

7574111424078

1

253429243724

211465644818
211572654919

17111

109157159143136158

931011176
83109164

1212

352779963331

3664

181622323533

191630205039

6271886459111

9860166129112129

4650105765879

195148398356218367

352264402232
342264402131

111

614664527448

353623525016

5399107617591

513460315776

2312812414

54481031084430

395439401851

6847828610958

444442346430

181321242128

7552172823

323151622949

375857474652

522481247

171624191633

10059818774127

322459333168

494267432366

18913131015
251126252715

72131217

709741730606393630
283270309247158284

51611698

5910748272018

1243134

2548922618

346045352027

30631313

9

142261576

81

387510171213

721925258

4456701727102

495210

8335102785794

421629491212

321721303527

1112142544
384960571024

21172022515

620261015

711557377143676846346480
136110741704145412291477

41525

831001038347109

226352912

1016224418

355856

494456362659

1662415310

463338

1711061651274189
6101300687706330344

1211

2921

821

2189131

711214

201338197260138108
234387230299172122

1

13

411

1

122

2

31

2340913122

3

1

11

1

155

1

1

1

11

1

1

3

111

141

1

2

1

1

1

1

1

3122

1

3

211

1

12

6

11

1

1

2

12

131262528

51

5433471013752
533141722950

42921

1

1161

1

1

2767248301411
2867249301411

1

1

2

151

29341611923

741011155

111

5334116822225
5327116632220

7194

1

304561403378

14

57171446196

4114623312154

2132118

2249575594

241757323249

11

13

1

76421077474150

1

4063103865385

1221319617

12787276174141180

737795491751

4130655614140

191738351812

1

13474

5128102027

1113111258

1

35612208

314101

1162412715

181525244124

44211

52216142

4593239711

31252

2831

12

121034

25521

111

3531775075121

521010411

96183126174132117
96183126175132117

1

83152241872418

1211

14

7225

773857351531

1

148255935

10714121016

185378101

502450243348

141025413065

10253141

1041612811

22

962124171516

424762243359

92201410

22

322217322046232312791647
1846237402414259292

111

212321

23121

14514319517166351
15314919618280359

3

5

711567

181

1

2

1

114212248163149156
112212248162149156

1

2

319471387674195234

8221251

6551661014696
90551201434797

233534211

211

282

8143

122

377925413414

11388

9911529563623

216924

583168

12722

628311207
194014212511

431721

9910333

6828891071131

124166

28223763587

3941

966378685586
9972879170100

34617

153

35512117

33102637175

23141

7441391

61220231723

931829233231

12172861730

921610522

2

392443

499178674953
417375594641

1386

81736

755782816380

121125

3161483

36533162137

104581061104979

10181555

20101437412

2

20333371029

5251

10350956482324

83112568

11111217529

2092029155

112017191224

11241

12161

383283235068

34996

1064111

4316141314

651811

302547366033

223148242939

1122

403354676448
413457706451

11323

1

541013614

111221

118311

1112

136221

51191479417

251637282525

371759571815
146672832997653

14111

1114

71171146

110251

31

19302221

12

166212457

132

11

2

823218159

112

11123283

1141121

4121

21115

13316113

5471212

1031421106

512121

12623

3164

511161731

165013972792335311211188
5728931094336

163117235329107103
11111

121
49943572925

224203456

27523222218

14881585
344654

1

32511

11

11

131

1

177253712512

265163113

1573740249

4
9620251515

6210151311
6212161311

21

22424

12312

12

161233551730
1331

73132299
73132296

3

891730820

479771051316

479771051316

6567108975633
175147257270140116

2

3584

122242133

8281784

14335

1674

33152

11132

222

2111

1

8967211

1

194231338

9111922

2

141

1231211263

7992119

1

11

2

5147164

2218221511

42102110

4

5

11

61112553

4363317861019300277
312023214

102101992252212

21

42485961911

60611212831

23542671816
1231832052859271

3211633512011

1061620156

811215316

5491

12352949109

5671132

6153

21412

234752

158312321

83242962

372435682617
185114317433141162

513153827

4122514931

715762

22151252

1473527910

263304977

57723

157101231
157101331

1

41

14123137162

852234113

3191

8491693

13173073

202231899

441

761328232

1

58142243

3263466

43252
232345532428

36315108

962212118

72151151

631061

5
169134370394144146

151942392112

151942392112

271544591212

271544591212
271544581211

11

127100284291111122
14940100816

314260483830
314261483830

1

371111

193448212551

2526555207

351363561917

5304338521004302396
32232843135

337553431227
1631132803427090

1259121110

1511

9113181

10371383

23372

65201431761724

2634863198
2634864198

1

633416

25272222118

43131513

21249715

80811321538676
463674823234

9101113511

14

451323148

113

8320202014

6237316

13331

3391

1

2232

71391671902783
33193361852

1041422623
1031421623

11

84141831

201210689107

522101
1015120721275105

263387432161
263387482161

5

15520303111

2756676310

28632482022

589916423019

519410312615

1

1211

654934

1

1

1

12020719822885114

12020719822885114
437748553235

1232

23121

748541

23

24111927

11

222

375062962632
365062952632

11

3

113

433716

211187619

911711

9122713811

51510921

481543552844

481543552844

31736302435
481543552844

1

1

1011

451322

5276

21232

10162655

1

1

42

511

1

816211

514131014
1293298539666356399

1227237415524296311

1164136235253101189

1324171015

1324171015

11
21492584

2952043
432

1

1

1

1116
2116

1

13

1

31

111

1

44441
1

1

1

42

1

1

33

33

2
116111720221183168

1381279
32712

5544

1123

8142319517
12623

36352

1112

461310112

11

36763

36763

257522

24342

1412

1107491001013169
14101821219

1

11323

21

11

1121

111
1

11

11

1

121

111

1

622

1112

111311

122112

5422

106241
14

10611

10611

33

72151033

11021

3651242

11191034

361

235134

22104211
22101211

2

1

111

144115

111111

121

1342

912528138
1

517867

4181971

183396510
11

11333
12333

1

12325

1631421

131222331450
1

213749

3

349528

6391164

1313125

11413

22

22

22

1111

1111

1111

1111

5676156219179102

1
5676156219179102

942012218

941912198
942012218

12

436812920614490
2836671153438

126

41431

1221

514488
414457

1

1

1

1

1

11

113861

12

231

21

1221

14
3

11

11

1

1131

115743

1

1

1

134

31

31322

1

24312

1

4351032

23

221
1221

1

1

43132

54114

11

313113

1

1

1

11

1

211

248573
123553

125

125

2

1111
111

1

3471144

3461143
3471144

11

72423511619

72423511619

72423511619
14393

21
11

1

11

22313
22312

1

21215369

111

115

1

2
21

1

1

1
11

1

13541

1241

123

19274832820
61601201295074

1

1213

616989

4122

111

1

26241

154425

210312

33323

2211

4764

21111

1

213133

1

111

22312

453111

1

219624

34

23553

2361322

442739

213131

235931632541

235931632541

235931632541

1121
235931632541

6138211219

57712416

161983

32
2136835

15122

155631

10992169
1

65448

4941711

13233
42381138

266615

11422

101521451615

101521451615

101521451615

101521451615
12

3591094
23

21522

4141

152131

331124

331124

228511

228511

12113
2522746

111

11

11

1311

21121

6515270245183134

1122
6515270245183134

111
6215069239181131

92117504111
111

41710
381234328

1

11

6

18243

18243

12

211422

223

121112

1

1

291911

281911
291911

1

1

1

322361
1

11

111121

1

111

111

11311

11311

1
5312951189139119

141664

141664

113552

113552

1223
3345195

1212561

1113

123

1

87732717
3512338127121112

179713196964
189914217265

11131
121131

11

1

1

1

1112154
11572

782

1111

1

1

21

111421

12

211
11

2

1942135

7

1

1

401

558416

21161

11

2151

437390994770

407087964668

407087964668

327315
346583894665

2
321

2

11

53212
211

131
231

1

111

131411

131411

1161325717

851219513

311624

113521

11232

121

227407122

432

1435421

123181

5141913

5141913

5428

5428

34256

34256

11661
311661

21
1

1

1

322722

322722

113477
22125

121

9151

65473
3224

211

11

1123

1

2
333312

1132

231

169627834832532060911726227631
432327615

66122585

2261823
66122585

1

11

445661

11
2522451051536

17113189932
1111

1146

77138214

452239

412454

115

115234

711141663

161

27

52341

31

1323

13161

166877426827152053031699527221
9561130324

90621521778056

55451221507250

55451221507250

55451221507250
16334

22371557
1211

162921

2222

531313

1429
222887735026

22132162

1112731
1112931

2

1111231

192670503524
124192

1211

8131152

1822112
187112

15

27766

21

1421052

25111136

538953

5211153

5211153

111
51211441410

12725
12735

1

2141

122571

2952733

3517302786

34171375

287761

2356

2312

1

165287280823302047691683027069
24518013413931348499

11694

11

11683

1952375971257187250

1952375971257187250
38361352713358

11

4421

54283

1

12

143

111

1

671713313

2261011

21371

12

111722

2411854

1212395

134614

16134564

2287

11282

152511

1

111

121

62151031

1

1621011

1481032

512

1211721

210862

111

13

282

12

2221

12

131

213412

2281

41

4101714

17944

1131313

371723

23

35102435

1171913

11

1

131

181319701022

11512

23914

11

1312

1

111

2221081

21951

1334351601016

16844

148244

12412

2130

343568

112247

23111

521

514924

120441

61028511

22

841222

40442044

11

22102616

35164493

11

1211

111103

3562053

11

12

1121

214

1

1512

1121011

18

1

22

3

6101

35352030691361576971092120433

482813332889213368
35352030691361576971092120433

412185915
1271993571326181186

19166534
381731

1420

310

124183
124173

1

42766410

42765310
42766410

11

562643

562643

427422
123128192913

10102113
1081713

24

3546222

435171

1823623

31424

24166593

24166593

122945792217
33

8293031199

4124538

3933134415
60681807049197

35812

231622

232832

1352275

9161225926

1172722

181121

111082

236232

134244

16163

32833162

189518162

2112

122632

1110203

2312703
2312733

3

5142313

2252514

1361982

17171

55922313

2491237

2491237
22997

233

283128303224

283128303224
273027283023

111221

2442833

2442833

187116278734103115
1441613

2451192
91218592015

1142586

1151

4561425

12242

46236866

46236866
33216664

13222

8264672517
32111324

216206

7232

110513

126132

3113182

1178
39937602510

11102134

364818182

13121344

14418591312

14418591312

45199954

45199954

65195756
11

61522

437173

216253

18264423

18264423
18254422

11

36145434
17112

1562212

21212

2541541461835

1

2239491071132

23324

22733

1229932922232
78283026596989613961

525821112

1121

425701111

821761821721

821761811721
821761821721

1

216133926

123901721120
28271844892675

910451301329

111122716

1216538

5121107112

1613
70371613573681

616721471341

6420831972340

1641422843576120

1641422843576120

1123170711
1141313

7153025

31122743

219401122010

219401122010

74266695

74266695

1053814097

1053814097

746128877471120
1313741721636

15747131711

42246436

362649616

29125025

45203936

8315321411

21114723

375545
375335

21

36182854

55174810

124175997

712691701452

712691701452

22432046733067
1503009953036185306

1838661702023

61660194812

6221163361925

621771871019

143110045

817632201023

817632201023

116691562125

98341241218

715391151011

242065412

24297019

2346732901623

29561143362034

1522
2214691272548

32274996

191241731440

12171529712

12171529712

102643891123

102643891123

310102329

310102329

1410511351636
53142920

222293

31102665

442551711

1141523104

114152394
1141523104

1

99441372618

18373

7825652311

2113534

52113648

52113648

2314882242245
1112321

12102973525

533362511

52566108

105641222027

105641222027

105641222027

54124599

5333356

5333356

191243

191243

4363338

4363338

4363338

132319523517

8372279
14

434633

231145
331246

111

223492
5201215286

212192
224793

1261

11654101

152

152

4141433

4141433

4141433

981326706425408853
230769763775144147960418488

143652291625

143652291625

6140456103080171
8141102551945

11211118

204225

281

12356949

637416399

562037510

6240109109

20743821725

5549126723

73067312

21216135

291234075356119
216290616

4162122811

43461371125

7153123816

3341117517

93761641834

343119104032357615193011
204260058731132233885016878

17343592986174

152612

551113793563214445

2517585141782201
2517591145987206

64255

40246761538112196

156434178967115

2628644143083179
2526620137479168

122456411

1796511448109216

15518411496525641074

239542105071152

4725571128182153

831664342743

640104220114728134576196

2724792198899230
2724755186295220

37126410

621116203436241484

1

17137081743108

53208481699122248
52208251645118241

1235447

1063935058513561963

1

391210022325162333

1231725826002343764

31169482128176299
31189712183180311

22355412

2527537142384161

11

211436772247109

711518414148273520

372010842137132316

193466111667151

21132671618

632915133477194442
1

632915103475194441

221

10940551116

74252158

74252158

35153468

35153468
35153368

1

351045731262118219

105802172629
4251161

52681861524

1172054

415236426

415236426

61441831436

61441831436

157042989876148

157042989876148

3111629816

3111629816
27818414

138932

121

3441641611
144020743216771319862049

143520193179761419732027
71625881813

1532421

1532421

1532421

11243

11243

11243

41629521413

1331244
291340116

7

3213

4

1

11

1121

1321

73

14

121

27161237

14324

2612913

53110195491147115
135918732935704218421885

9161678149
81

211931

4441912

1652472

2561824

73719448
151

114

315
15

30

3281

12362

1742

1251

31

434

212
472531727

1181

111

96319

141326

121

96

11

133

1213205258
623922

591

26

11523

14

1

45452
45442

1

1481

1226

2830341114341
7710541211

2

2

2111

11

11125

211

12
11

1

12

1

11

334

11144

111

1

1242

6963316

23251
22241

11

331612
331512

1

1

91
1

9

613841

12

4191

4191

6881822

112

23

46251

112

112612

222322

222322

234611

234611

36312

36312

514234991315
24516642

32421

143544

117

122882

19

114

1124

573

151

23211

133124

565812

565812

32323
111916451315

412

1921

111

19452

2421444

225
215

1

32512

121312

141

141

3110818

314313

655

5942

5942

42113612

4162912

157

682421421
782641921

1

21

15

31124910

21

23432

1214

223

41515

42172369
317522

1241

1212

2231

21

2113

11262

11262

3213701261614
6731532

53211

15282423

2314124

114132
115132

1

7101
791

1

1143442

2161623

1121522
21566845

11011211
1101911

3

222911
223011

1

21111

14
6673031

13111

563153

21
2863135078

141412

25613722

1311

812

315142

2

916252491012
44107768

3731132
3731122

1

1361623

13131

18

113

219

1111
1499172367

22411

121111

45111235
1466131735

15

14211

65292765
21

55821

44822

1120912

1421
68582

53381

1

2258681163434
63143065

1134

2551342

2224

313

113644

21144

3411

4108932

42314

2153

29101542

131314

3272

1254

11693

53920211
121533971724

1171122

2211

32

14351

21394

123

1122551

1221026

252

151

712212325

712212325

111

111

2231
4418501910

21112134

16252

27711

2343

16736

16733
16736

3

123521

123521

292013

292013

129189212376286198
3426367051245759643

2323

143

427723

14181

26419112

151

161927

3351298

3421

114213

1141

62819284

223872

232217

35102044

22223

512511

8789127

35741

1241

3331163

1511104
1511103

1

297334335867

22

41

3122321

224

1274

2321

472522

51912183428

181233
181333

1

11516

112614

3142124

281222103

131

115653

2321

121

1

21731

1111

1252

59846

462111618

12

411231

5754110

1234

52112152120

9109152114

2391326

4233

5591348

3241523

1121

2291023

142

1

11

1111

11

71213201618

11413

1411

2530113

22411

21531

2122

228751

122718212934

1

2

417464

11

3611

2311734

22811

821

411692

12223

111614

221976

32

12435

12101

241132
2141132

1

48212139

143

14916125

1171343

144

532

113

153631

1473272

1243

2624

12

6155621

128513

127854

2678535

21

131744

2391344

4132

2222

1241

1

1322

151

1112

49742

11

11

1351

1351

13103082

13103082

1231834
11

11113

12721

4171212

4171212

12104

12104

214151061110
52192

24

1311

115

21231

151134

11021

1152226

7122

7122

772

772

2
6161423

132

1

4331

2131

131

113

113

141

141

2562816941784274377
45581613487589

4162

1361

45

13

11472

3116607

13161

16101

14913

5121

1

3181345

15311

125922

2

122

43565

6101

214761

451948614

11812

7121324817

11822

91514

7123

3813

1156

7121529611

1115931

4121014

191

1121

1743

1254414

113931

1310

12432

25512

1

2231

2321

1115

11012

15812

1421

325

1753472

1162

47125

141231

1352847

894

2

4622

45322

481

1393

6271

3231232

2161022

351
352

1

1171327

115

1313

1122

1412

3242822

125

11522

1111

295

741

411438211
411437110

111

11

27246

1

383

3111

131211

1

727121

21351135

41019311

521975

241291

112234

421315

141

32154584

28193

172433

112

3410413

161

662538

11102

114101

73316994

451021

45292

124717

4313

31134524

5122

181

3113

1151

192

11224

211414

331

2132527

2111

1

20

7542173

3311

1191

1122611

181832

2231

211

7422

17

314294

1142

4413165

251534502821
23111

13212831

1121

12141

831015164

26710715

1131014

1131014

1313236

1313236

263641691030
619183

1262

9

3714

2112101

691612322

41711

141

112661

514193010

514193010

693

141

552

261311
71746903228

51

24161
1082107

84146

11411

321

191

2152

12322

31

1422

15821

11

11

14714

1332

11393

21311

721

113

1373013

1373013

7172013

7172013

14122

14122

171

171

2323421712513
1111712

1

111213

131

384

18

7127442

246

111041

24391

112832

114

1841

31

1

11251

731

34

36113173

145821

2131542

1381

1248

1248

1141

1141

2561041

2561041

46138
21261133613

112

212231544

141

5476
4132231418

331

7822

224

12511

161

31213

21221

36776

2141

135

3333

31952

31952

173450922428
122714

21211

5417111013

4310962

3141133

41

4

1

2123

2562933

1152

1152

1171

1171

277203867
214102

12833

15311

2

32
2532

25

25

1221

31

11511

3044771223157
36111754

2513

1221246632

1921
1911

1

122

13814

3721

311932

61

22103

114

1

471

31212

2111

336

2891

11394

261431

111

2431

2431

4103

4103

611945
14112

55331

252

2352
2962267

31

231047

1

11

214

1
36144

22103

144

2331236

2331236

371221472588171
1693395

7251

1

4221

22323

56172744

2

120713

31

2

4639242

121

411

4

497173213

221

131

11

12

1

2511

1

2261513

1111

42

11

11

4

1

12812

67

221

211

1112

593

41612187

51343

659797

1145110

4462252
181645672112

119292174

1111

11

1151212

1

13963

111

1

2
1282067415

23314

23732

33242

232

13122

121811

4344

1232

38711
1710123433

12671

1121611

11441

1
2136711775547

12123334
515371023217

12141839

3111151264

45134243
1

2422

291521

25222

393974

393974

12410410

12410410

851413813

5332

42133

111

1147

242128

1261244

1261244
1138

1

1

1

1

21
2132

32

112

28721102153861

2121

2121

331522

331522

2552053
27671061983658

32

2121

112

11211

1348631041635

1

112

3122

231

1323

32821

117

26511

2

1141

12

3

1

113252

4234

11

1361

2101

1

12

1

22411

1

1

1

2112158711

11811

14411

1121

21111

13111224

1211

111

5111

841918579
2825147449712737018382045

22932124
2623113

3

11111

1

124

42710923
2815146849432715318192032

2751141747612655617331908
1514118013295856

83211211113

83211211113
83211191113

2

1983809249513137849889
6001524183954158179

932412368

17624108146

107321361321

28920204313

9131194129

111011425

1912210825

228241161016

843184641913

318381791614

51826294410

31828202188

4312283601317

51103313

292172071010

61731132797078

6183812

831615452

1

4552526549

2381811259

146279233558257244

38427303119
38427308119

5

6910534442024

539293101310

841414137

2631812113

141735210616

655731282

2252147422

8612355381813

1

64922398216

631342318412

911379

333172161012

5

55144742

311910845

32624189107

28218155132531

142217961

399683241518

3317261771819

1411371381022

1411371381022

4917225191263103
30519314299526378509

162733572129

161159333926

2310633263636

93442781015

137996932738

73512333115

77924141435

1724663471520

511870122

661411

1611813741831

124673481630

941491312

31622081416

145491941011

3386817441418

7171124882527

25101227732825
25101247763126

2331

7131254313

1641235543

121712

8727113149

8727113149

3221712
511411953018

222185226

7189379
71893710

1

1612391881019

49216057

3353837

9139025

2124822843430
384022

53235474

6122155713

59186584

21270107

2092973321008320315
120183123267164154

1832

111242

61492

21116328
22136429

1

121

831711057

112029492628

121

12211

218252

2331321

71711191924

4221092618

4221092618

1163461

6576246

51166154

1

1311

511552128

55131966

2172256

3342015
3342016

1

133

34928310

21630123

6792598

31123654

3162021

319785171928
732412773

10224204614

14429169611
14430186611

117

21

2

1

604917348884120

604917348884120
1252169511

124

11

61211

5415471523

1

2172513

129371021422

191732

14925512022

792164714

221571

3782258

2394545

1

1101516

40748513803738464685

40748513803738464685

40748513803738464685
1711271001611

109471421719

109471421719

171532

171532

1614441611626

7726851216

971876410
971873310

31

2749641682738
3181121

1538123788

2322621010
6322631010

41

131434613

2482216

1

30281242693474
11

1152233

611944516

111546941025

42124244

352430418

2153033

332755

1514722481629
1571785631700178258

393492916

4441156825

14382976412

712456357

13145134
24155477

111343

3152390710

342273916

714355088

1163664916

68391091212

881369316

2

2

16281310468

191033781117

7243111815

9337114818

2310541541826

6817751035

6817751035

521713

521713

351001573603856
510217673

1173315888

613659813

24177969
241883610

141

11125184922

1151123

1151123

2111922
2812

158

1431

13585

13585

4
8103298628

23174426

671550422

2324652103928
1141

441038810

48256156
48256146

1

592171225

10283646

141554881828
11342

81043661522

243821

3351

2512

4316861441932

4316861441932

138511103421

138511103421

181943416

181943416

394592963378

2
394592963378

43147320

43147320

1032113717
121

27411

829563
8212966

343

212353
81521401029

310102955

349821

4810946

4810946

41012511

41012511

96142085

412412

55121673

655916836080101

211
655916836080101

121241971915

7102977913

7102976913
7102977913

1

521220102

521220102

1081840107

6692132

6692132

4291975

4291975

42381072184777
21

3734971923872
112912

371428411

43102988

64724517

19163351923

2162797

23152424

54102485

54102485

11432

11432

777669514232598973925
4132053

111426135

1264

3963

191132

770657012302161844782
2939671403266

81632441329
223914

1

1

128963
24139910

425

12112

212

1

1

33121
4101626314

22

12

1131

45957

12233

1729531222425

1

1

1123

1123

351021

351021

1725461101924
1542

112

272

3

1241

11431

11121

1210721

1131

121

53

122412

2121

12511

1631626

131

5714713

111

232131

36915

36915

36915

83408121316483
116173373

41105
3

3

1175

524

524

142633

142633

1

1

59112588
1

121

1

11132

12122

26821

141

231325

111

1

1

842336918

842336918

11

11

2

2

5417431026946
6410151317

2112

83

1

12726

1113

261131

121813
111813

1

113
1113

1

1

1

12

1

11471

211

51

47213222
3111111

4412111

5

3116113

1221

72164511

1

111844
3843931796356

4331

4331

8173

8173

3433861435150
471331112

1131

38251

111

1

1

22223

21243

211

33

12

4121

529838

122

222

2

21
111332352019

3631

110165914

31

1

95611

1211522

111

21

111

135

41228
411

227

121

321513

122

321821

321821

1181343

1181343
11331

1

8912

42712

42712

42712

1594573839

27
1594573839

753014428

331014

21

14124137

12471
7338203435701250257

232514

3

232511

221

221

3272401072514046
7335198427687248252

322

110

2111912

1

1

212

36292

216

3663143

12

1112

33213
3323

1

71

521155

15423
15433

1

171

111

4171

42896211

182222
1222

181

1

3
147314

147311

2131

22

23

111712
222122923538

2211

12

1234

1

1122

1

32219

21142

1

111

1

22383

11

25

1

2

22115

2

1

111

3241016

111122

212913

452

16

2

1

2

31

3

1

1

1131
1141

1

22693

1

1511
151

1

44411

23

20219249478474
4958221614

1

183611

1

70

3

5

588740256758

1

32222

311151

14321
132

41

11911

12102

11

2

111

1

111

32111

41

111

111

131311

2

154335

1

162
13771272

1377111

41161

1331
123311

21

1

2

11

11455

1122

3

281931

7595436

111

2438681604061
45

121619691728
11

352633

12121

6911171121

33622
63733

11

2

11

6

2

4

122245862333
471430813

122221

1

122211

2

2333

26

1

1671

1313

1

1211

121531

1221

2

121222

111

121312
12211

111

322

1725

122
2232

1

12

426662

426642
1

22

111

121
12311

1111

4121

2

2

2212741162430

2571

2571

61522
32

1

391

42

221063942128
5142113

5167

1

241213118

241213118

18634

2481726

22

251013

114131

36531

127131260378214124
2371246

24111943

1481312
11171

37611

1211

1242

2316381024930
95118233334204109

1114
2214

11

11

111

1313

11131

1

1

14172

1311

123

111
11

1

42

11

1

12131

1

11

311762

21

3121

221

111

11131
1131

1

12121

31

123

4

131

1

13

1

1

124422
224422

1

1231

721691

211

11

122212
12212

2

121373

1521

2

1

83

122

211

1

13721

212

13421

283

61

1

1

1

62

2123623
173313

1531

1111

1121

111

1

1

2

22

4

1

145427

11

11

141

214334436516
1112

1133

11221

112

41221

13

1122

221

1

11

1

14315911

2

21

2251

13312

11210223

122

4

2458

121

11

1

132

27931

1

1

1

1

1

62

34

12221

11

212

124

111

21

1

121

7423

3441

11

17412
7412

1

11

1

20111

2

1221

1122

3622

1

1

3521

285672
282451

1

3

3

22

12

12

65123176391111135
1132

2281578
1

5346

5346

2221232

2221232

1118521382645
1112

141136

141136

16101559

16101559

66112748
11232

2342

3121421

224623

121343613

121343613

1
32134187

217965

1153222

2674801175748
1521

43101143

43101143

155153

155153

2511983

2511983

5818272612

47106222

11821410

54112373
3

125872

426121
225101

212

54584418

54584418

1281652

1281642
1281652

1

2241024

2241024

132125691314

132125691314

1281049818
1211

222912

222912

51225510

51225510

5461315
2

1121112

43223

2
57751521957968

91631281912

21152

257222

3161071

134663

19333

154211

48591211676054
459836

151429572117
1

322933

322933

322933

11
10924391810

159621

2351

14962

121612

11253

31433

1371

22394

22394

22394

4

4

294083983631
161840372018

1

4741122

1212

22732

1

1341

2

2

1

1

116522

14681

15

2521341

3

4442

12312

111

34203234

34203234

361412110

361410110

22111

1212

12634

1563

2

2

2413
191839301329

111

2111

216

21111

225223

213417

1

42522

11

43643

11

142

51

3222

1221

3622

122425622820
4692847

2212133

12324

22112

51461164

21

451

2

4948681353364
11

3545481212142

3545481212142

3545481212142

14223186915
3545481212142

1112

21131

642417

23329

23

31

1612

1

1

13221

3

122

51

4111451

313235

313235

313235

313235

311135
313235

21

4511

4511

112126816

112126816

112126816

112126816

112126816

152277302347153219
14931

1211

11

11

1

75961221355293

75961221355293

21
75961211355293

426270803737
4212

124753

412821

4231215107

197161157

26725

617151999

3512

6581321

293444461537

293444461537

45819

45819

1

1

213038452633
75175171208100125

1211

1761410

2310613

33111

3131

12631

1

121

6111215169

1

1

321

24

1

318113

1121

11461

1

4539824

141

511246

41

31111

133

147213

82523242219

11

345644

51078710

9884822

3

11

312411

312411

11512
23841911

232822

232822

13532

13532

11335

11335

139815344013430019592598
101419221520

304364722122

122032301211

122032301211

122032301211

122032301211

18233242911
11762

155182337
175182437

21

1771244

1212
363545651653401532

96154205188145169
325507569577372494

42212

211

11

72332142119

1

221213182919

11123

111512

463933

333

11222

213353

22612

11211

38436

115313

2112

11111

34215411

123428

1191

13591025

135

3114449

11702433929

72101624

113123

23

39201646

112

12

123832

44664

3631015

436247

1162

11

1212

353

2221

1211

233511

12

619308

20293321949

6313627814

16141

1250121

22523

33114

11

224512

52192067

55524

111

111

11331

24

21

15413

336337

12425

21311

96112247

182339132423

114101

114101

114101

322768492128

322768492128

322768492128

48915710

48915710

6975961145870
2

357435

357435

1
6470891105565

201628201729

201628201729

201628201729

445461893836

445461893836

445359893836
445461893836

12

8588033099337413391878

8588033099337413391878
1519991103565

4262
39131711093158

51161

247163922955

41431

24121

11

11

614723222191129
8047712829315412721755

1
57581061227566

231039522420
22939512420

111

191141574833

15372612313

413112316652115

413112316652115

182131321217

182131321217

9236441024
224214846856335542

5971306200118142
5971308200119143

211

675622627391171
665622426989170

12421

32281171884994

575715915166110

33331801347776

33331801347776

241255922434

241255922434

184194509544280348
121525313

353275724845

251770934660

183132432627

2743125954779

423880744547

36311121426577

1371111723548
116109649898274364

7874365511119192
7774364509119190

1122

19181361675989

61037486135

465298895264

465298895264

4638482910563

12112

1

4336462810461

1

1

1

221636362013

221636362013

221636362013
11

168192698

168192698

671610115

671610115

95171127867305579277108116773237528
88815762164231015822394

92422211027
189627603373464647624575

213
232299276275297368

11

11

11

11

111

111

111

111

11

11

11

11

422548

411223

41122

41122

111

11

1

11325

225
1

224

111

111

1

1

1

1

1

12113

12113

12113

12113

220282266262276344

220282261262276344

220282261262276344

2112

1

1

212

212

219280260261274343
220280260261274344

11

5

5

5

1
132331

1

1

1

121321

12321

12321

1

1

1

1

1

3824128

112

112

11

11

2812128

2812128

115211163183172171
1

31

21

21

115206162183172169

115206162183172169

115206162183172169

22

22

2101751
83712791199267930212243

384711660801728807
1

274942544938

274942544938

274942544938

274942544938

274942544938

357661618747679769

357661618747679769

663484
357661618747679769

285028443654
189297334385326375

95134160130164155

95134160130164155

1

1

275544544056

275544544056
112025261531

1

1231

111

111

111

1

2

3

33311

4125427

112

9119131910

33

2111

3611232416

3611232416

152441612343

1
152441612343

152440612343

202850733951

202850733951

69511911
125297223283291312

106231200247253272
193622374443

8181211118

84633282819

82115322529

131720273434

305244547374

112426342443

91728241422

135718252929

1

135718252829

376158755478

376158755478
253937504156

311
1

31

1

21323
2133

2

121211

851210310

224446

224446

172622

1142
451558538187122881435

1
366363326149620541143

17

17

17

366363325148920541142

366363324148920541142
2647551114691

28121717111911750830
33343

47146610

24315512410781571726
42010301413

23813011410431549711

15582

35151111

35151111

264497

2322126912155
2423138213461

12

1118104

2

212

1
162415

152415

164553
185563

211

345484

214759625778
163584

11175201513
111

11

11154191512

115

12

13

4911151512

51439221944
42512

13131

5934161541

324031108183127
108466

81012247072
25222099164110

43242610

2131

1

11

11

101414820

127383

11

11

2552

11

1

331235

4523106

143333

1

1

584141513
754

252475

332334

1

1

22357
85195211374230290

57126164235165206

57126164235165206
14129283928

121415191815

13391051302390

7272415826

112920122241
21

112720122240

513455

513455

1

1

2667471366077

91510162111
2667471366077

3626676

52225171324

1

351545

6199321431

6199321431

1

1

6068241625135311501635
35912611

494664144111639711405
526864

311245

311245

311245

311245

311245

1

1

1

1

1

244365612554528649
3323181312

61130178174166178

61130178174166178

5257610
61130178174166178

1111

15135

55127167165156163

133172314287261344
8151882026

404062837097

404062837097

404062837097

85117234196171221
11

1

1

1

85117234195169221

46187117
85117234195169221

1314

1

21

1222

22

121

121

80110211178154207
7619141911

73104192164135196

4760977588115

1
4760977388115

1

1

1

4659977386115

4659977386115

4659977386113

2

2

2

2

2

1

1

1

1

411
242296815596431747

11

11

11

11

240291809595416733
1

240291805595416731

421

421

421

81111379305190339
240286805595414728

11096258199133203

1113
11095258199132203

11094257198129203

11

49791689190186

49791689190186
1

48761689188183

1

322

322

1

1

12

12

12

2

2

2

2

12

1

1

1

1

1

1

1

1

1

21511412

21511412

21511412
1

231
11

121

2131111
1

143

11

1

1124

122

1

1

1

332

1

1

1

1

232

32

32

32

2

2

1

1

1

1

1

1

2

2

2

1

1

1

1

1

1

1

428657

428657

428657

105153167172168212

1

1

1

1

1

105153167171168212

1
105153167171168212

151

151

15

1

104147167171167212

104147167171167212

104147167171167212

9512384131112130

9512384131112130

9512384131112130

41113
9512384131112130

9112283130112127
131

9112282130109126

222

222

1

122

95142138235118189

32

12

12

2

2

422

422

215826

215826

215826

215726

1

93137131222113179

122

122

122

92135131222113177

92135131222113177
111

92133131221113176

1

1112

11

11

12

12

88161103146144179
411293

1

1

1

1

1

1

1

1

418437514170

418437514170

418437514170
1

23322
408436514167

408233483965

112

4375649394106

1

1

1

4275649394106

4275649394106

4275649394106

13944

13944

13944

13944

197928843637303731093895

197928843637303731093895
101148190140171203

7212
18223

11

11

11

11

195458394949
58611721570109612021734

56101079

1

1

112211

112211

111

122

458858
1112

116534

116534

2121

2121

1411

1411

28568110345846701145
3131671420

51

51

11111

11111

11

11

52111994

52111994

62311

62311

7215102611

7215102611

2131

2131

2516449545266081095

2
2516449545266081095

2113

12

1

21

11
2496449525246061091

2496449515246061090

1

11

771915510

771915510

771915510
65151139

124421

2

2

116421

116421

225131

225131

361161319

311

331161218

102428372417
1

8181120149
211

1

1

1

1

1

718917119

11151

11

151

12

12

14

14

11

1

1

753

753

237553

11

237443

2113

2113

262400429419439492
11

255397419408433483
141416152628

14312

1121

312

212214

212214

132523
129201189170172227

11

44114816

44114816

3112

111176163151152186

131898920

3361710

3361710

357160767478
1

356859727378

2141

68102128131141124

11

68101127131141124

111

111

43138118

43138118

21

21

1
327455

327154

113

113

316124

316124

21

21

21

42714

42714

42714

31132
283354323647

4246
1141922022

1321

1321

1321

731421415

41821114
731421415

1

1

11

1

1

1

31

1

11

1

3411

326323

11111
326323

111

111

1121

1121

1131

1131

11

11

1
1153

1152

1152

11

142

10151411912

115213

115213

115213

1111

1111

1111

211

11

11

1

1

1

1

113

113
1

112

32

22

22

1

1

12231

12231

112
12231

1211

765256

241212
765256

32444

2

1

1

1

11

11

1

1

1

1

3451213

3451213

3451113

1

971161515
403514601602683646

11442

11242

2

217519

217519

217519

591210423
11

324138

324138

4

4

2769110

2769110

1

1

6610236

6610236

6610236

23446

2235

2235

1411

1

411

12111531415

12111531415
11321

11793118

3316

222339120
370476529572638570

11

11

131821252116
364470492526635547

165219217226272268
2553143

152130283536
106126141143146176

484348564757

484348564757

436263596483

5788718011289

5788718011289

186233254275342263
111

222528322634
184233254274341263

11

8395125126139108

78113100116176121
111

78112100116175120

1

1

3412723

3412723

3412723
11

1

1

3412422

1

1

1

1

3

3

3

1
381911214

341710112

341710112

341710112

452

3128112

2211

2211

2211

2211

21

21

671223

671223

671223

571222

24

21

1

1

3111

3111

11

11

11

172110141516
85110021194114910111245

11113

1

1

1

112

112

112

11

11

11

11

242333
90117172190151190

11132

11132

11132

2344
1

11

1

1

22

22

1221

1221

1112

1112

112

1

84106149170142174

1217

1217

1

1

83104148170142166

83104148170142166

1

1

1

11

11

11

71

4

4

21

21

1

1

310

310
5

34

1

1112
1

1

11

11
1

1

1

1

24331
311

211

211

112

112

7348509899378351025
68997010492102

4651098

455998
11122

323668

21

11

11

1

1

6811141821
149278306295272311

57133124127130129

57133124127130129

1

1

1

1

85137170154124161

2815753
85137169154124161

83129154147119158

1

2431112

1

1

11

11

24392

24392

11

11

231363509445403524
322

3221031
228360500434401521

111

1

1

2113410

11

113310

1

11

2

125199278223235296

1

97156217197157212

34311
2

21

12211

21

21

21

2

1

1
211

111

1

1

1

189413528

189413528

189413528

919381665569

919381665569

919381665569

3111

3111

3111

22

1

1

21

21

71121788

5715474

5715474

5715474

121

121

23411

1311

12

11

11

1

1

12

12

1

1

1

121

121

1

1

21

21

120211145249194213

1

1

1

120210145249193212

120210145247193212

120210145247193212
521732

120205143230190210

2

2

11

11

11

224326300578312364
511

130165193396180200
3

23244
10717221020

181238

181238

211221

211221

11

11

535717

1365

522112

2

2

2

2

2

416931

12

12

415731

1

1

1

1

45721

45721

116157170362167177

114145166340161172

114145166340161172

1

1

13221

13221

113142164337161171

113142164337161171

21242265

21241915

11
1331

112

112

11

11

411513

2
411513

21

2151

1

2711

171

11

11

35

35

35

1

34

131

1

1

111

111

111

111

1

1

1

1

93157106167124157

11

11

1

1

1

1

1

1

93157105166123157

93157105166123157

93157105166123157

93157105166123157

1

1

1

1

2

2

2

131755

131755

131755

131755

193277250364363380
7281239

8213589148160173

1

1

1

8213589148159173

8213589148159173
1

8113589146159173

2

11132

11132

11132

11132

1

1

1

12
103140151203197196

103139145195195191

103138145195195191

103138145195195191
41314

99137145192194187

1

1

16723

14613

14613

211

211

11

11

11

11

147231194246268304
51

141226183237261293
1

135221177228254285

1

1

1

1

134221177227253285

134221177227253285
323762

131219174220247283

1

1

124432

13422

3422

1

2

2

11

11

532545

532545

11
331524

331513

2121

6569710
1

23651

23651

2

23451

122

1
122

112

352127

352127

352125

2

73112364

2131311

2131311

2131311

251

251

251

426422

426422

1121

1121

1121

415236474055

415236474053

415236474053

283626351841

283626351841

1

283626341841
232318261430

213

3106619

21122

131610122212

131610122212
212

131610102110

2

232336
11

311

311

33

33

33

1

1

1211

1211

111

11

9112334517
1112

134

134

134

134

561624316

561624316

561623316

1

21

21

222421

222421

222421

111

12142

12221
163257523056

3104123534

3104123534

2721

3103916333

4711416

451314

451314

2112

2112

16142

16142

11

11

6116101612

6116101612

21132

21132

21132

347650798190
85914113310289872258934100579218587

131918044799429217593876
8490411131928905325789899023216794

213313322387310418

213313322387310418

213313322387310418
122216261818

1

1

1

124187175199178244
221

22414

22414

122187171193177239

122187171193177239
24312

120187167190176237

77104131162114155

77104131162114155

77104131162114155

77104131162114155

54188095011258561144
62966

256406442526405538
223

74138142162131171

74138142162131171

74138140162131168

74138140162131168

74138140161131167
223

72136140161131164

1

1

1

23

3

3

2

2

180266300361274367

180266300361274367

180266300361274367

2
180266300361274367

1

1

180265299361272367

180265299361272367

279472508590445598
231021

11

11

1

1

1

1

11
125236249284225287

367128
125236249283224287

43711071007175

43711071007175

1

1

1

1

79159135181151203
132120252235

304454715165

1

3693618578103

152232259296218309

11814181212
152232259296218309

1

1

11

11

6211411312475109
1

6211411312375109

79110131153131187

1
79110131153131187

79110130153131187

2

2

2

2

2

2910567
307458393413510540

157227267265222280
11

75124177193108188

75124177193108188

1

1

91613111910

91613111910

693429

693429

693429

693429

1722

1722

153636214130

153636214130

153636214130

481722293226
164812188

11113

21731375

1111
11

11

949379
847348

1

1221

3181371914

3181371914

3181371914

3181371914

126196101125251217
12431

255431445045

71139159

71139159

71139159

184328353536
11111

11134121413
11134151415

32

1512459
31916101313

2144684

3117977

3117977

81894352176141
1

2959182492109

2959182492109

21171181711

21171181711

311314206720
1

22838599

22838599

951112711

9965611

9965611

9965611

9965611

104217211919

295375
104217211919

3125423

3125423

253533
1

253532

12221

12221

2144757

1016971122

1016971122

1016971122

1016971122
71257212

3452

448

12106112014

612436
12104101913

352275

352275
1

252275

34492

34492

211
111

1

1

1

1

1

7796510311027672224502091281204388
7431037330929759682402

205194437452195441
486915299614372212093351984124200

1

1

1

140141415371163357
376674454312406110003442184102471

155023414021413823124112

155023414021413823124112

80133298261147269
155023414021413823124112

4

4

2

1

1

273068534579

273068534579

273068534579

273068534579

559310210661112

559310210661112
192626211132

1

1

121420211025

121420211025

161233362427

48111288
42912211111

211933

267529

254236

2

556513112876141
1

1111

1111

11

536212912067138
1

536212812067138

121731

21711

11

1

14
1

13

2610435
60103167212120174

366713116281131
14635

82953533937
62343382623

2610151314

324
2837741033989

132024441635

151747572350

223026463638

223026463638

223026463638

223026463638

398579887960555870
496293966994

164662483565
13444

82027281735

82027281735

2
82532161426

82530161426

183765653256

183765653256
2

183765633256

172337481940
9111717019599173

6581067

164638

164638

481415519

481415519

171627311526

6818191115

6818191115

1181517716

1181517716

171830291818

71391027

5128101317

5128101317

152718412435

152718392435

11

152718392334

1

1

174465685570
92031343722

14912116

59209718
61221111422

1

1

1

11

111

1

1

11

2

1

1

2

2211

2211

1
153727

153726

1
1121

1111

1

1
353849594268

202125312045

202125312045

151723282223

151723282223

1314869

1314869

81823222324

81823222324
131441

71522181923

1

1

101329211120
403985963852

1092127611
1092128611

1

2371875

2371875

141224221415
181428291416

42471

323848803059
141514431033

182131351724

1121
111

11

13111

254068594148
112

131742232326

31415251112

31415251112

88911710
7791179

1

11

34522
4352104974894

171632441723

171632441723

5817

13926221231

11

131933261630

131933261630

81723261323

1

71723261323

444593974884

444593974784

444593974784

444593974784

1

1

116198276339153271
64101969

439512912768131
235842

203361684071
122448523258

891316813

215963512258

2

81632321024
162451571837

154931

7391235
731516512

6216

211

281212511
5175861356093

253938683339

1

242836542243
12

242736542241

11

11

1

1

1

21
679220621497174

1

203973714170

1

203973704170

203973704170

11747

1

1

1

212

2

3

1

1

11

11

1

1

1

43501231365395

43501231365395

43501231365395

43501231365395

1
221222

112

2211

163746654478
243428686686425678

112229381738

112229381738

112229381738

132443352633

1
132441342632

132440342632

211

211841442841

1111
211841442841

201740432841

4

4

222940402946

222940402946
1

222939402946

131425212427

131425212427

131425212427

1

1

1

162636512648

162636512648

162636512648

162636512648

81020201122
23431069156104

111863501858
144328334

211113
101420221524

81319211421

41523212724

74150200196108177
3210444

194034361936

194034361936
1

194034361935

5210815615685137
172237392633

210121628

210121628

1

1

11

11171415128
11

11171315127

11

2111
6162926822

6142825821

1

641291511

2212

32735371737
1112

32634371635

781011314
781213414

1

1

1

11

1

253583593858

253583593858

253583593858

92637462927

92637462927

92637462927

161284520503277473
343844

691256
5810016114093149

285468524857

1

243792864085
162032311825

81760552260

82158310298158272
141427322139

8211411263382

11

5111012212197132
311

193038453754

193038453754

328084725977

1

353315

661514613

661514613

1

211

1
182246572248

1

172146572248
1

172146562248

124139328293169534

2
124139328293169534

124139327291167534
2111

122138326291167533

1

11

120152259282139253
26132

647012813963131

647012813963131

112

1

111

1

1

548212314171120

548112314171120
548112314171119

1

1

3597742061119625955253970998002
147182518401157478

217302629613291591

217302629613291591
6101717106

1

1

1

121

121

12

1

120156378331173343

376712511636126
120156378331173343

3350103944497

1

503915012193119

1

1

1

1

1
91134233262105242

2

2

1

1

90132233261105242

661514620
90132233261105242

8412621824799222

111
1

1

1

1

1

12

12

12

154916275528430818294973
3537641258117776938893891996283

154092037434844269781266627192
311

41045610486673901007
154062036834840269711266227173

421528759832874636546754
786918816044162

3063531003803329998

3063531003803329998
3249908147131

116134430335128424

116134430335128424

116134430335128424

158170483387154443

158170483387154443

131151388292150343

131151388292150343

131151388292150343

131151388292150343

131151388292150343

370023028253749131315251
266175666781220369

36732713171783367790
113423

2051518391408221425

2051518391408221425

2051518391408221425

161175475371144362

1

1

1

161175474370143362
1122

160174474370141360

1

133150432346141374

133150432346141374

133150432346141374

1

1

1

1

2241145

2241145

211

222644

4

293216485833457023993713
170112382325140270

12085318257138194
9657202338186910721448

11

11

1312
310243723505413376

1

2111

2111

310241716503410372

310241716503410372

1111

1

7112669

7112669

220160504458240351
8770255214101173

372267652659
472982823280

641011510

4356111

362570603235

362570603235

8599107

742126813

742126813

1

1022023612
1

1021923612

252447445131

252447445131

12113

2

113

1

695116213675133

1
695116213675133

695116213575133

1
1016924119077132

1016924019077132

137109377315123249
3835108862361

4229110994281
2

4229110994081

11
574515913058107

1
564515912958107

564515812958107

12391377274101209

12391377274101209

1

12391377274100209
1

33331
12391376274100209

12088373271100208

12088373271100208

2571481132969346481
1

2571481130969346480

2571481130966346480
119315

2561471121963345475

3

3

11

1

1

252138536465154289

252138536465154289

252138536465154289

116543910686685861016
104421237549105

13223

13223

1

1

1

2214
1

212

1

2

372217418293216399

372217418293216399

3

3

11
688177523293320503

2

1

687177523292320500

9467156152094215337747716325
7813015612966174

48825385180738434572912453
166192522234174376

1123

1123

1123

1

1

1

3378360613696629743028835
46052817548625561287

311
117412024789215017483139

21

117212014786214917483137

1

3793571300689389923
828531614990219

171172596339191452

126100388200108252

1

2633501022488321724

1522
2633501022488321724

2633491017486319724

500576275910136191271
2702891634547340743

535229313077115

177235832336202413
141155669266178323

13627133937

43176212

993312225

4417538

62251488

9881311152120254

9881311152120254

102532171122

92531171122

1

1

260235972514276551

260235972514276551

260235972514276551

6102081416

1

6102081316

228242737404248648
222978323055

747625613579241

132137403237139352
11

132137402237138352

151241231634
8931100236411828442181

2

2

1

1

2

2

45454712225594391069

3211347
45454712225594391069

45154512115564351062

42454011015993871074

42454011015993871074

42454011015993871074

12

1

11

44448614907194091058
111

44348414817144051052
3233311117519285740

37581377546105

839522712074207

1
128445

1

1

1212

3111

21321

409597602198608414743048
1

409597582198608314743048

409597582198608314743048
1102223175712725151123

21022221617096267

21022221617096267

102829473281894262454
228267196964194574950

125437723682300312496

263356252219144350
1

263355252219144350

3215
238230277228145358

235228277228144353

2

2

2

412340515690208650

412340515690208650

1
412340515690208650

1

412340513690208649

1

9723121127
131214223018222111413085

1

1

1

61254211369494171151

61254111359474171150
1008920215777215

137119272246102256

2
137119272246102256

137119272244102256

1

1

1048424521290243

1048424521290243

1048424521290243

11

11

271249415331148435
4332926536112

1096116114465183

1

11915616112247140

1
1121

1111

11

11

179181439370188539

179181439370188539

179181439370188539

153152435357172469

1733

1733

1

1623

1

152152428354172466

152152428354172466

152152428354172466

1

1

1

1

1

1

1

1

359539985532353898

359539985532353898
325496942500338857

344343321541

22

1

12

3337319

11
3337319

2337215

2337215

2337215

4

4

4

207279725634279714

11

11

11

11

1

1

12321
207279724634279713

165221557510228554

165221557510228554

165221557510228554

165221557510228554

165221557510228554

1488514

31

1

1

1

21

21

21

1

1

1

1

1357513

11

1

1

1

1

1257512
1

12225

1

1

1

12125

12125

11125

1

23527

23527

23527

1

1

1

1

143235
405215611446144

11113

11113

11113
1

1113

12

12

111

111

114322

114322

114322

114322

91752371651

562614421

1

1

1

342311219

342311219

342311219

222322

222322

222322

41126231230

245

245

245

4926191225

4926191225
2591199

24525

21636

1715

11

11

11

1

1

1

1

273096702483

2

2

2

2

413415
273096702481

131963451444

131963451444
2

1527

11
131656381437

131656371337

25

131121

131121

131121

742615728
42132415

3
381

1

1

1

141

32

32

1

1

1324

1

1323

1323

1323

21114

11114

1

1

1

1111

1111

1111

1

1

1

12241

1

1

12231

12231

1

1

1

11

11

1

1

1

1

1

1

527323932295324
524459861712419683772119264

1985211371741060636169663
27198015240128

4243201345109286
167417656330955531478444

121
21

1

129127531811235641

129127531811235641

129127531811235641

129127531811235641

150215955595839428037514
255292104018105381617

188207613830304770
374513522458198

24299712030127
334312516145172

91428411545

5121761164897

5121761164897

151457663362
1716

151456593256

162660773369

162660773369

365816018687172

71023521334
124144463670209657

4457212307102296

4457212307102296

737722831194327

737722831194327

211143685996325779
212

1

352914517673179

176114534818252598

31

534713519251187
141133442526208529

8819181420

212010813146135
353112214170148

141114102413

333411413953127

662014614

662014614

6732221433

5082238414147336

1

5082237414147336

5082237414147336

5335942114314810722826
5771242403137341

90109314441163388

11

1

565717623098254

4271210379117300
122

4271209377117298

6582248374129332

11

4843233296109296

8170312490150424

9490377534169490

9490377534169490

1122

11

11

1

1

111
1

11

11

1

1

1

1

1

1

118154400529193482

118154400529193482
1

11

1

1

118152400529192482

118152400529192482
31131

115151399526192481

166175364369236609

166175364369236609

166175363369236609

166175363369236609

1

1

1

1

377933

377933

377933

377933

377933

796420020886196
320437939704874640069274

242228227549674530946995
101136393361151368

585113916049156

585113916049156

585113916049156

3633921052829379937
237726

286302807664295717
182552472049

628519714562123
253391632962

303992732446

713149915

21

1

12925121226

2221
604114511847149

603914511645148

1

2

2

11

546015111755143

1

121347372142

686818918478181

1331

1331

758623515582213

121
758623515582213

748623315581213

120103282240114295
185321825791522524325325

545117816483143

545117816483143

545117816483143

112

167187489399175427
562651172914726651578

12112

1

1

22
284286833586

284284833584

142741502036

1

101138191336

91131231125

34461199444110

71324271421

1

1

1

6101217616
101225241932

41137613

41137613

173

1

1

12

3

1

11211
131155431443

131054411342

9101710513

132030281235

10754291331

312880842882

102187803557

1123

371213619

383583822985

1

141039401125
171040421130

2122

13

43391068151111

792317920

152655402953

81131281232

131438281435

262372643576

1

1

232785783074

398458116111065431140
111613773601334715703309

972943519
562435313

1

415825

1
661315714

661314714

121136402343
1

121135402343

163266583271
1211

163164583170

1

232564473270
344615012753142

15

112185802167

1

4838113874595
22324

4636110854591

2339581164264

2339581164264

151449411843

151449411843

5991088
122

464343

134543

32

32

82137492532

9233030317

2

191841451043
18163535838

11262

112

22

1131

1091913627

161643461243

467024321058183

467024321058183

6810102816

4171524925

191942272051

272781714183

272781714183

7789

1

111
395412412552116

395412412451115

8892329260108253

8892329260108253

1

362415516

362415516

1

1

1

458015014284141

3835104694691

3835104694691

1

364816313950128

364816313950128

1

181739673555

3

1

2
121248281640

121248281440

232452533356

232452533356

1091417138
819853

285985

558588
2111

556477

1

1788810
51514121415

486465

2

1

11

1
101434251532

101434251531

1111

5310658
28411211014585

2338111954077

112062683144

1

151550381846

476117417083209

1

1

466117316881208

1

466117216881208

466117216881208

11121

121

11

424514714965121
703907195517938262083

393911910841115
134159395354174374

322582874278
546512713868122

65111058

65111058

387835

387835

358747
4812899

1141

11

11

1121

313854
111722

1121

1211

13213

1211

1211

331012413

331012413

231

231

21

21

151864472749

151864472749

151864472749

253384603887

1
253384603887

243384603887

1211

1211

2838919853103

121

121

2838909653102

2838909653102

2838909653102

131
101246252265106410

31339211241100
565813515062239

1415262916123
4310769

215927

45

79106697

121325

5511747
1110179516

22412

1312

3215

1333

1333

4518611111143167
1716956531963

652321360

652321360

221232372144
211227351841

121

1423

11

11

586813413580164

586813413580164

586813413580164

586813413580164

340351936792348911
555814313365159

43491069943131

43491069943131

43491069943131

201356531557

201356531557

201356531557

644818414162169

644818414162169

644818414162169

47631131086188
202247371524

469647

469647

351017711
325937

11

1443

2143

203047483546

345912810151112

222874662978
345912810151112

2261

243323

527547

32542211623

776120615751195
43853

9125036751
211980551480

1273019729

132660452055
162872582368

321213313

361146391444

119132382321157388

119132382321157388

119132381321157388

119132381321157388

119132381321157388

119132381321157388

1

1

1

128471286059173419651626643752
25124113739533691065

334347214714604841467
6507684540886274691006328792

8949444450296612803666
562316823

302312157410814811260

302312157410814811260

302312157410814811260

302312157410814811260

587626285218677912383

384382180211724821501
587626285218677912383

313235

69101956

151561453552

141463381667

81242351340

21211027234104

4622141319

5627231122

5144724936

4717

41214241018

3417111315

595332929

4837201033

182683522475
272793612485

9110910

11137228940

162290623284

4519911

343

19281457529101

14659471769

151267431636

12
2

1

2265391153938361986

2265391153938361986
41137341439

27313

21

25212

1

7795472375147400

4654296245102261
7795472375147400

221989622671
1213

211788622668

22
92287681968

92287661768

143433637526199544
23241131013088

78329308233120278

427921619148176

1112

7497375327345712023766
505350153313522105793822673

11

1

1

1

11

11

11

1

1

1

642638349222878952546
139141820536198570

1882331007669280740
21

1

153188856575225630
1882331004668279740

232984572660

652410919

61140261931

1

39251528939132
315264165910724151234

144115741512174545
25211357

142110720499169538

1

1

132122766471202557
623

132122760469202554

1

1

11

11

1

1

362
21

33

11

3

2

1

11

11

108811686921439415984680
8447321732

2211
11

1

21

842617619

842617619

384446249715515391711

1681961203731246789
384446249715515391711

5122712819

2112381267808285903

1

1

1

1

7588533355156389
6877124349279110352917

1
310315210313094681367

310315210213094681367

302309171111274111161

302309171111274111161

2

2

2

2

1

1

1562157510663735926467253
243119511237116

429408288220217371825
478474327322948342068

1610107811752

1813123752877

154315911051109

2715

2

1

1

1

1

2

2

133134943633195659
106010707191495217755069

412472299921137462090

111
515464324922068342320

514464324722058332319

1

11

11

11

11

113112845544180515
7566194930342511823189

1

1

1

1

2051671290870305836

2051671290870305836

438340279320116971837
8554451346

276217178312974501191

131
276217178312974501191

276216178312974471190

154118956669234600

1

1

256277180011834141235

256277180011834141235

256277180011834141235

1

1

1

1

250240755538223633
608957741691413543583413895

211

211

211

211

211

694815613369134
338732439112764232367361

2122
194224649514231515

6621222223
94100309219111235

3536137834090

11

3536136834089

535815111449122
283685842869

2619349

1

28221

171536201342

171536201342

413421

1

1

1

100121339293120278
71342321230

1

1

425512212055108

425512212055108

515317414153140

515317414153140

1

1

1

1

431618820
649660206416337091683

524573170413215841397

178166529405165439
524573170413215841397

283183564580
122121

272981554379

8511427018786178
4872130934782

122477542450

2

251861401546

131479581146

465413514868120
1

465413514867120

3631112953587

12166428838

151566482854

212268532479

11111

1672222418

532827531

532827531

8923171023

8923171023

1

203096764190

111

41601269953112
2927103743974

123323251438

2

2

2

12184344292117266
11

795124217772205
12083344291117266

1621713102
1722421128

17826

182051662240

23

61025241113

61025241113

1

1

247523116243536122275029
206126380301130349

11472279218100217

1

1

11471279218100217
59351549452105

352578833574
251959652963

5334

2410633

326934

1

201047411338

201047411338

357384950809335817
171734361641

211

211

52281078837115

52281078837115

52281078837115

3334
3556

1

1

1

11

1

5540123974599

5540123974599
112

5540122974497

626918115347150

626918115347150

405911611747105

111

1

1117
395811611747103

385711511047103

1

1

130163357304142289
44491169739100

273264552945
1131

263161542945

233177492750

233177492750

581113812
71015151216

224244

445776

445776

7599215

7599215

101336321428
1

101335321428

210111418

210111418

6920251021
6924261121

311

1

13258112

13258112

111
100146276214103212

624517814039121

1

132214
624517714039121

614217513838117

3810097716389

3810097716389

32

2

12

11372
268268762647304672

4410755

3310745

111

231232660543265587
10986283231106259

22
212258472651

212256452651

333487784086

1

252687603279

1

132233291933
122018241728

123522

123

1

2584

1

3122219514

5102211816

1

221762602744
1

221762592744

323189903478

323189903478

20103726711
749530184417395931311

1008624420760197

1008624420760197

2

2

1

1

12

12

1

1

1

1

3

3

1

1

1

1

1

1

1

1

1208231429788258
628433155915035191098

1
1146120618584153

12

11311

1136020318383150

1136020318383150

1136020318383150

1

1

151143

21

131142

131142

2122

11

1

1

141
774933542172104

73463214066798

33101456

33101456

1036922920192201

1036922920192201
503112610855116

461510412

16109911

217335

73101738
221

4181427

12211

437717

437717

3271157

3271157

2363

2363

219214

438522

438522

81252

81252

11311

11311

1165526
15122415823

2294416

1251

125611

231749462239
975718415575140

651614817

11

6114627919
51

6114126919

391228341426
391228341427

1

12522151223

11720191014
95131259

227755

1

1

1

1

1

114113283229103236
49331031023898

161445342447

153527281228
153426281227

111

1
122121

122111

641510811
14124233922

1

662523111

11

1

2

191764311840

1

11

11

1

1

12

12

11241

11241

11241

4164491068889395901
161133271537

1111
62801559759113

21

13211
56771529155110

56761498954109

333522

685714714050117

685714714050117

393711311243115

393711311243115

1028921819996200
201737351625

2

271955442350

301958663261

253466542564
1

243466542564

129175402314132319
491725414

274079532575

1

498315014252127

71223
291469522042

221367501742

192987423160

1

386512710050106
264335682540267549

1
2121

1

211

162200376298141299
3313934

272777703166
353994803584

8121710418

282878823770

211577452461
8911916010953118

6810483642957

32
71131181323

71128181123

222885593875

222885583875
1

222884583875

1

132

132

1
394194803666

11

1211

384091793664
181739352033

17163729726

37151595

764650228017437251909

764649228017437251909
322474712160

205146507342170463

22
205146507342170463

2111

449637
203146504341169461

199142495335166454

935931424689205

935931424689205

935931424689205

1

1

1

1278229625587276

1278229625587276

1278229625587276

436

31
436

126

178193637482207546
151149332039

133
444116413745158

11

444116413541155

117137413308140345
385115612546137

485818212858146

211843261729

101032291933

2411424

2411424

1

1

1

617425621592198

617425621592198

617425621592198

617425621592198

686719612957155

11

1

1

676719612857155

676719612857155

1

1

168816414765362016493991
393512611024103

416490132210974991160
172558542071

398516010143142

1

1

398515910143142

398515910143142

596319317176155

596319317176155
11

3101520811
596319217076155

565317715068144

189179567467227503
71127241335

514812611055124

514812611055124

514812611055124

1
211

2

1

1

31
8480299230112246

394714411640123

394714411640123

443315111372123
111

1111
443314310571122

443214210570121

78

11

1

1

31

31

47381111014797
21

45381101014797

45381101014797

112138344304133289
212987732879

192262582168
517715313464128

213663493042
85225819

133138371922

3731

111927261317
111928271318

111

4032104974182

1

3932104974182

3932104974182

58346913868334421095
322796522972

211031131327

4597610
211031131327

1026325

2236

5314326

5104126535

5104126535

5104126535

299224741430226514

299224741430226514
57391027177110

32321

1198629715862189

1209733919987212

1

1

200179442292163425

200179442292163425

200179442292163425
168151340221135342

3228102712883

26193520622

47621
26193520622

44228415

44228415

952522
18871026

2231

63323

1

969430828897294

969430828897294

2
969430828897294

969430728597294

969430728597294

11

218209573484201468

272178693072
218209573484201468

797423620569185
249869

12221

746621018460164
776922519561175

2311616

243

1212

836014711853117

1

1

1
835914711852117

835914711852116

1

1

2854112924994

2854112924994

2854112924994

805825318779207

805825318779207

805825318779207

805825318779207

805825318779207

256286797621307664

252860471546
256286797621307664

114138358300147331
404911610740119

10932213220

10932213220

9141618319
11174530631

11512

122412210

536316514269161

536316514269161

475217415167135
1

475117314966131

475117314966131

11

2

13

14134

14134

1
696119511870146

696019411870146

696019411870146

1

310452

29441

1111

237319702621342648

237319702621342648

557915814654160
237318702621342648

40511101085598

40511101085498

40511101085498

1

7497221198148199

7497221198148199
111

7396221197148199

689121316985191

689121316985191
11

689121316885190

1

1

1

1081982591922420447960521287
394591873595

998672511676117898849818990

272220511569256583
998672511676117898849818990

243516994386457621004814

243516994386457621004814

243516994386457621004814

727953321186412753614213593

358366398656379754
727953321186412753614213593

1

1581615217
689449251144312050574112789

955518

687049171142212030573412754

1124620726

1124620726

131717241521

131717241521

21

122

98108210224107216

98108210224107216

98108210224107216

98108210224107216

98108210224107216

98108210224107216

1

1

1

1

1

170205390455250411

170205390455250411

170205390455250411

3648841096384
170205390455250411

1
252048442148

252048442048

262955553452

262955553452

1223
485811013572112

485711013370109

35509311260115

392488148714515381239

392488148714515381239

392488148714515381239
1

391488148714515381239

134162285332177335

134162285332177335

134162285332177335

134162285332177335
596011214674145

2833
142542442438

142540362135

363878735091
445510010465113

81722311522

172231371439
142

162231371037

1

28531490771296911211123832977786
531928296426287781775

2980275711637927234457398
146106385406112259

1121

1121

1121

1121

1

1

11

1

312061642038
12141118274431779391934

21

11

1
11

1

1

1

1

4074949961093419783

4074949961093419783
1

4074949961093419782
342388863954325680

82226271627

425369625852

7121419910

81924311113

1

1

1

1

269244624721215445

269244624721215445

269244624721215445

269244624721215445

269244624721215445

48434910271268269631

48434910271268269631

48434910271268269631

48434910271268269631

1

1

1

1

1
17929211433

16929211433
221

421

421

312111

1111

221

11821181330

1

11820181330

425924

11

11

11

425823

44422
425823

1

121

1

11

3823809311422941806

3823809311422941806

3823809311422941806

3823809311422941806
485315722079145

21

123522
8786169266167169

8684166261165167

7865168251148125

1113

2442
8580157390396178

8380157386392176

262915516

7383203235122153

33420184

45111

233219211

1

451

123711537576426514524399

2
123611537576426514514399

1

1

1

393283257613504581514
123611537572426414514399

3243191099903243669

13311
3243191099903243669

3243181096900242668

90103706349144380
1313127532151

415333417855190
313425313935138

374218724

71239211328

363724511868139

158182955479220608
575037016778203

1616120542866

99228514
629134918785243

538232717980229

2324116712895

111

1

1

21

585350625182241

585350625182241

585350625182241

384437021167227

2

384437020967227

384437020967227

635543920587261

635543920587261

635543920587261

112114918516150498
545252723687277

525936826157216
395033122953196

1393732420

63231965

63231965

11

11

11

11

1

1

1

1

1

1

1935841054674
196739128009888635435652

78142304373167218
96022784363563520953203

1
186314832837410542

186313832837408542

70110310330163213
186313832837408542

13148418
4482233223102145

437921921598127

69111280276133174

310981010

11

11

11

35944

35944

35944

35944

21234

1
11

1

2112

1

1

1

1

1

1

1

11

11

112

112

176305768920439479

176305768920439479

542118613
176305768920439479

53171077

166298729892426459

1

51815132452349310711960

268214917156108
51815132452349310711960

1411

11

131

141

1

111

2

387918182624557781356
58122278371114193

121270662809232458
12

121270661807232458

1181025618

4710921730983163

92266427598214340
89246385534196311

25453

31837601326

11

65122215327122173
65131223337128179

981066

2

328413

1

105511473863236494

1

105511472863236494

11

11

11

11

11

213319

1
213319

12

2

2

1

1

1

1

1

11121

1111

1

1

1

1

1
11

1

17

17

1

16

79313692918246811191916

79313692918246811181916
295017715151130

132230664558210371

411
132230664558210371

13

132229657557209371

132229657557209371

276114

12

12

275112

275112

6291081205917508441399
14214

12

1

11

6281081205417488431393
62106269317101189

121

1

1

46741221434598

122

1

3846921321987521884
398313114074103

1
3456091190847447781

3446091190847447781

89128183158104126

89128183158104126

89128183158104126

222976652641

222976652641

222976652641

255179764254

255179764254

121
12

1

1

1

1

1

24102211

1310210
24102211

1

112

1

1

1

1

1

1

188227633668275443

188227633668275443

188227633668275443

188227633668275443

188227633668275443
21

186227633667275443

12322
528676

211
513221

312121

312121

312121

13
2

11

11
1

1

213

11

11

1

1

111
1

1

1

1

1

415681242422555861415
23053414801070811003263056262961

51656481835

51656481835

51656481835

51656481835

136228557549169365

136228557549169365

136228557549169365

136228557549169365
40491531473895

526920923464129

526920923464129

11

4411019216667140

4411019216667140

211

157372832168724644611853536975
578951221219706561147

125172121443204387171357922777
110518623963347711852077

146344

111
146344

1

2211

1111

2321

156225225422464916973001

156225225422464916973001
116552

131221584301397213292338
32272275

130921564274395013222333

2493631115670363661

2

142595442448

142595442448

142595442448

51021837
23

1212
236415

1

1113

11

1212

37312
2512412

13

1

1

121

11

11

6840115952344621293747312321
107418523890352611912031

197833316827625422173640
54691719191764600963

13

173274608515207307
3065211142977394593

29491131133162
233586871941

4101113413

24161388

213292924044

213292924044

43711451255395
608719614967113

211

368412

47372

11

1

319517

103261146

23791331084967
1952100813553

156436

245

116171348

12414

12243

112184377332129188
144236487438160255

253588782650

7172228517

32111

1
49581815211503515890

122247429397143256
49581715211503515890

180299595631189354
210336665703214390

21

1

1

32

12212

343

2471336

432

211

1

1132

114211

1

91124211011

24513

349214

511411

23112

12231

23422

1221

1

106186332318118184
163234427403158244

192254412432

382641441628

39768514771325451788
48383717571571547936

43701531224974

43821271244774

120520014207371412732136
36856212123881118339576484

92416533227292410001675
100218073535315110971802

4686154983962

32681541295865

38557211711094387680

38557211711094387680

36664111731102385672

36664111731102385672

35058211811057418596
3095311071997384534

182445251524

11183316919

12932191019

37760911201065397598

37760911201065397598

1

103200341330108166

103200341330108166

103200341330108166

2111

211

211

1

1

114321824179365813422172

114321824179365813422172

114321824179365813422172

184530136071558218513146

184330136071558218513146

184330136071558218513146

2

2

1

1

1

11

11

11

4

2
4

2

2121453
1

2111053

11
111

1

113
1184

1

11

6

122

112

1

4

2
1

1

2

1

1

129638531836119230277610425

31

31

31

416105851758
129638531835819229277610425

35142168

35142168

128238141820119022274810318

128238141820119022274810318

21122

21122

273212
1131582121

11074617

1132

1

131
1

12

1

21

21

111
232314217

222113216

11

134423024945453015152623

2

2

2

6711323325279125
134423024945453015152621

22

1

2

1

12655
6561080236621496801203

6551077235821426791196

11

11111

1

1

1

6191109234321297561293

11
6191109234221297561293

492896189217395951052
6191109234121287561293

78124286242108147

49891631475394

1

28453215771637455954

28453215771637455954

379026528874159
28453215771637455954

88159556514130292

88159556514130292

394817016052104
159283756835251503

56120309405110200

56120309405110200

6411527727089199

6411527727089199

64761170233743313761079923217
460914320729468322045

11
110187588555171362

1124

12

12

12

1112
11

1

1

11

11

14111

1

1

1311

1

1

131

131

1

1

1

108185583552170357

111131

11111
111121

1

1

1

1

107184582551167356

107184582551167356

107184582551167356

107184582551167356

25814349126211140439668154
78126412410129253

57118357353100230

57118357353100230

57118357353100230

57118357353100230
1

5711835735399230

179293844753246596

38141458
179293844753246596

6912029029599227

6912029029599227

6912029029599227

1

1

107165539444142361

107165539444142361

511803242321907951644
2529105813868

141223

141223
1

2
141112

411
14412

11

1

1

11

5

5

1

1

1

1

1

1

105168581498183429

105168581498183429
472729725

62116427335132312
131180772346

1
306526016281175

306525916281175

194087962891

194087962891

39451271344492

39451271344492

39451271344492

149223541537225343
682222916

469419720971124

469419720971124

469419720971124

469419720971124

97121322306145203

97121322306145203
101443432427

47551501465780

47551501465780

40521291176496

40521291176496

181455591334
93136417403131294

475818920764163

475818920764163
142675982664

151251431839

182063662060

182063662060

28641731375497

28641731375497
1

28641731375496

28641731375496

131
138243767669218507

699528326183189

699528326183189

699528326183189

699528326183189

69148483405135317

69148483405135317

69148483405135317
2

327118016664127
69148483405133317

245120917441141

132694652849

164128108042715525225064
107164615565142383

76155554489131374

2531524
76155554489131374

14

1

22

74149549473129364
12311

74148547470128363

1
5382378346100212

121
1

1

2

1
527937634599210

527837634599210
2

527837634399210

111
1

1

1

1

1

1

1

11143

11143

3

1114

157737
105203582518198313

5510428425087133

1

5510428424987133

4994291261108173

4994291261108173

122
11

11

11

1

1

171193699611219412
11111338

323

11

1

122

6985399297113222

6985399297113221
1

6985399297113220

1

101107286299102179

101107286299102179

101107286299102179

1

1

11

11

11

69112343321110214

67110341316109212

1

67110341315109212
227824

65108334307107208

11

11

112512

11

11

11411

101863491729
114253685556220432

12

12

4612832521598207

4612832521598207

58107296290105196

58107296290105196

58107296290105196

8724221115
144240676567218459

458227419797178
11

1

1

458227219796177

91151378348110266
11

11

12

91151375344110266
589420818671145

161938331527

161938331527

17381291252494
22

17361271252494

21

1

1
2

1

4141314
237384982847316631

116188522446171336
517531325885200

11

356498865673

163176522338

351613

1

14153032621

121189427373139277

121189427373139277

121189427373139277

31915514

31915514

97168388369121209

97168388369121209

97168388369121209
64251958

91164363350116201

84100326291114203

8196311286110198
2129971082150

2722106794759

2722106794759

3345108994289

3345108994289

122

112

1

2413525
11

13744

31

1

11

1

11

5554

5554

5554

1

1

1

1

110177495435150345

1

1

45118312
110176495435150345

629929326083212
1

629929325983212

447219116764121

447219116764121

99235483422187344

99235483422187344
142563522761

69168339296119232
63159309266107203

6930301229

112
164281744151

164180724151

111237519482186318

111237518482186318

1

111237517482186318

1

1

1

1

1

8917425

3

3

8917422

8916422

1

6391302305101183

6391302305101183

6391302305101183

6391302305101183

115199543543174367

115199543543174367

115199543543174367

115199543543174367

115199543543174367

332562521732716471583012656
97211685583172408

23236611271087315731

23236611261087315731

23236611261087315731
252590722158

1
125215612617195409

125215611617195409

8212642439899264
1423

8212542039699261

1

1

1

119623435678574920844454
76146361360131288

129290659653217518
583842923

1

1

21
64157378368112295

143212
11159141210

1011612118

53142367354100284

53142367354100284

6012424324396200

6012424324396200

6012424324396200

9926910441058222554

9926910441058222554

9926910441058222554

1

1

1

325410710244104
423879153614727461584

78206321269132280
15105314

215236

74193296258122251
327136

71191289257119245

1710439

1

12

1

2

7717151519

1

7716141115

1122

21

1

1

1

236482886869426878
69123284245103267

376915316264168

376915316264168

4810114611992153

49118166185105153

1311195

11

326712615753131

326712615753131

70129204217126302

70129204217126302

70129204217126302

1

1

446418515460121

11

11

1

1

436418415460120

436418415460120

111

1
111

1

1

628319220671161

628319220671161
1

628319220670161
202255632245

36491221273995

6121516921

94170381484175280
11152735825

4694193181101125

4591185178101112

4591185178101112

138313

376116126866130

376116126866130

376116126866130

124186634684201466
10145979944

82115399429145294

2
82115399429145294

82115397429145294

325717617647128

325717617647128

1122
91132376406135266

406819119260132

406819119260132

406819119260132

516318421275132

111
516318421275132

506318321175132

51121304270121216

51121304270121216

581813109

1

46113286256111206

1

32414

32414

31414

1

1

1

1

121216592536156404

162275731234
121216592536156404

569827124185204

569827124185204
2

569827124183204

499624622259166

499624622259166

499624622259166

5104031330
318596177415537831471

21

21

11

1

54138364351113249

1

1

54138364351113248

54138364351113248

578109
54138364351113248

54133357343103239

13266

13266

13266

75138435430139283

75138435430139283

75138435430139283

183307931735528902

183307931735528902

183307931735528902
131

182307928735528901

96214614607178397

96214614607178397

77153467459139308
96214614607178397

81332451719

81332451719

11481151032270

11481151032270

64911713694335010072385
111441381127

1081215144

1081215144
1

1

1071115144

121210614632158427

121210614632158427

121210614632158427

121210614632158427

2

2

232

232

232

377683191417405801245
12734

1

1

11

1

1

376681190617355801241

376681190617355801241
160297999900276644

32102137974181

32102137974181

538125225187159
30531641745587

131237371732

101651401540

8310328024498201

489823824378156

1302561111920244680

1302561111920244680

11

1302551111920243680

1302551111920243680

1543881044268
25244610921019419835

116219438415180357

21
116219438415180357

113219433414173354

113219433414173354

3373

121184566500197410

121184566500197410

21

121184566498196410

121184566498196410

881829819
261496143913715061171

74149421381162378

74149421381162378

74149421381162378

1793381000961336774
123580982174

5210129826696228

5210129826696228

5210129826696228

60107351299107255

1

60107351299106255

111

111

1

1

5595269297112216

5595269297112216

1

1

1

571816715

571816715

111

111

471816614

471816614

98186614600203385

98186606594201379

98186601588198378

98186601588198378
1

98186600588198378

5631

5631

8626

8526

8526

1

1

316628313577383926023688
212740423847

1288605727646636751

1288605727646636751
11411

1
1287600723638635748

1287600723638634748
8141421814

335548
1277581701614622730

270438523508508558
1272577692606617722

3397104556362

96942654346102

11

21321

212

111

11

256242

256242

14342

13342
2

3

3

1322
111

11

2

1

1

1

1

160517482193246115242270
106610710

151515971997223613882037
68146169186120142

976660819923558817
62811089875100

21

21

21

155226282356190270

155226282356190270
193333373646

1
69921081396999

69921081396998

1111

639613417678120
6610013717680120

1

1211

1221

14344

5710310512068118

5710310512068118

5710310512068118

5710310512068118

103856632750
700250324349224329

5312411212795120

5312411212795120

5182

4172
5182

11

143452542440
6328815615894157

1

5992667435068

1

4281046
192837602048

3131519614

3131519614

121314311028
1

111314311028

83117147174131195

83117147174131195

83117147174131195
27

83115147167131195

212338459510290434
101018171314

263143481952
141236302344186298

567212012762117

59133139169105129

619213814990122

619213814990122

1

1

1

1

1

1

1

111
122231268292186302

2

2

12211

12211

121229265290186298
6310313413196139

245053643459

3476789556100

3476789556100

53105135151103147

53105135151103147

53105135151103147

80145190215128221

80145190215128221

80145190215128221

80145190215128221

12

12

12

1

11

252451617690404620

252451617690404620

3575841276695
252451617690404620

5510414116181150

5510414116181150

11

11

233449403838
162272392401256375

67131162169126177

67131162169126177

5939151115
7110618119092160

669714217581145

1

111

82913621401177312131662
71211251914

111714341421
4887397941025680978

164227232324224294

21

21

21

164225231324224294

164225231324224294

15624
164225231324224294

163225226318222290

1

1

1

1

211

211

211

211

12132

12132

12132

12132

183267341385221322

180261296370213298

180261296370213298
204242675360

88130144163102134
6168741006987

7761056

205564532841

728911014058104

21

21

21

364513724

364513724

364513724

127226206278218340

127225206278218340

127225206276218339

127225206276218339

1

1

2

2

1

1

1

137207209263199224

2
137207209263199224

1121

1121

1121

44

44
4

2

2

411

2

2

211

211

136207204256198217

136207204256198217

136207204256198217

136207204256198217

197404387460315446

197404387460315446

197404387460315446
81171149220138197

6410121113
7011112411981125

335972513251

314842563861

3112

3112

4112
307065605557

306664605455

164949604065

164949604065

1
425329647760255762

665125618062176

359278390579193585

1

1

473629829

473629829

1
133225203257227279

5263798782109

5263798782109

81161124170145170

81161124170145170
111

80160124170145169

23328

23328

23328
1

23327

976191576995714751703
2162

972191176495614621693

972191176495614621693
589443599394

131

131

331

331

400798338428634725
1

10241192023

111815182614
390774327419613702

379756312401587688

3
5917

112

4812

5041002382464732863
2

11

3221

11212

2

11

501997379460729856

11

11332

1212

112

1

1

11

11

1
424178

423157

323146

323146

11

11

1

1

111

111

111

78311910
86712981206149213541582

12

12

6278759294129

11321

11321

11321

6178748992128

6178748992128
223419353145

3263210

3263210

311347
173338384147

143237353740

19911131826
232322

1

211121
176991624

155881423

216335263340266341

214334260326262337

214334260326261337

213333258323259332

213333258323259332
28652

312

1

1411

71251085
208332244316252329

201320239306244324

112325

112325

112325

1

1

1

22

22

1

12

111

1

1

11

11

121314

1222

2
1221

21

1

1

1

1

1

1111

1111

1111

1111

132712118

13261218

11

112752
1

11

11

11

11

3
1

1

1

1

1

1

1

1

1

111351

111351
11

11

11

11

1
1151

1141

1141

21
58087383610309781080

91021161627

1

1

1

1

81021141425

1

1

1

1

1
81020141425

81019111423

81019111423

81019111423

122

122

122

2

2

2

2

22

22

22

171424616037105

171424516037104

171424516037104
11814713

412914715

12131981322376

11

11

11

376935696983
554849569854923947

62012191919
189326243379312410

128214155244169267

142618271928
128214155244169267

511096612470130
357436703684

163530543446

1

6379719280109

114

114

559275115124120

549275115124120

549275115124120

1

1

2131

2131

102418233426
328452291405539453

8512680117105141

8512680117105141
1

13251

8412280115100140

151615182722
233302193265400286

138140103139286148

6566517110

351311
7313497134115138

7012996131114137

11321216

11321216

691147410686110

1242

681147210284110

681147210284110

5358410

5358410
13

213213

1311

212323

110137148
456760615686636852

1
1132

121

121

121

121

11

11

11

11

111

111

111

111

111

141236319250254333
4103328729

2

2

2

2
1

1

327875

327875

327875

327875

1111

216865

115412
126209253188228286

12425
61226221520

111

11

11

1

1

2514479
1

22123

111

111

1
2212

11

11

1

1

1

1

11

11

2121

21

21

21

1

2

11

11

11

121

121

121

4212

1

1

11

311

11

11

11

122

122

1

112

111811

1

1

21

21

1

1

161

161

247944
1

1

1

224543

224543

1

1

2221
2

21

2

1137

1137
122

1

1

15

15

133

133

1

1

132

132

24447

1
24447

211

1

1

1

1

1

3435

3435

1

1

25525
11

15524

2

2

2223
15324

1

11

2

1

121

121

121

121

211

211

11
1

1

11

11

1

1

1

1

3212
42146123

112

112

112

1183119

183117

183117

12

12

31

2

2

1

1

1

1

1

1

1

1
113187192138201226

3131
31

1

1

11

11

1

1

21

1

1

11

11

11

1

1

1

11

11

11

1

1

1

1

1

1

141467

141467

1112

31347

1

1

1

109182173126199218
131

109180173124195217

109180173124195217

211

211

1
7121821811

414432

413422
1

121

121

1

1

312121

312121

11

1

1

1

1

1

1

1

1

16121419

1210819
16121419

1

1

1

1

1

44
45

1

24122

24122

24122

3121

211

12

1

2

2

2

31

31

31

31

135512

11

11

11

11

11

11

334

324

324

11

1

213

213

1

1

111

111

111

111

1

11

13
313513279428367509

115

1
115

1

1

11

11

12

1

11

201301158241161279

201301158241161279
15133

13

13

1
2451143

11

11321

35622

12

12

197292152226152273

197292151226152273

1

111211115187206227

4231
111211115187206227

741296894129141

741296894129141

357543827683

357543827683

357543827683

3251

3251

232

232

122724512337
175829342887333726783179

5092839879108
112119201887200416911919

115178171173156202

115178171173156202

304123343451
115178171173156202

648810510379112

648810510379112

214943364339

329510435566476490

471007910483107
329510435566476490

535445925658

535445925658

137203175204183171
314428413229

619593939380

456454705762

1

497484847482
282724302824

112137242740

112137242740

102623301918

102623301918

437952828072

437952828072

60889810788118

60889810688118

11058

11058

11058

60878810188110
131

11

1

567965898197

12

4

351711313

1

1

1

287171785651
566104810971056890999

11

1

1

80189261253143245
399783877818613816

1

2816747
222439402426

201623332019

123171215176162179
146942

122167209167158177

1

1

1

187344341939
116331201422

1

13724

134716

222415

32311

1

107236217198201232

11

2

412

4881951115987

123224

1

1

21

139194149160220131

2111

2111

1

1

11221

1111

1

1

1

1

115166122143209116

22

115164122143209114

212623151014

212623151014

212623151014

12221

12221
1

1

2

1

111

11

11

11

312

312

2

2

31

31

222332

222332

222332

222111

222111

1

1

121

11
121

2

821119814997116

821119814996115
111

1

1

1

122

122

821099514995112

821099514995112

11

11

11

11

94132137170143180
17168101216

482963
48748710181123

244043604672

244043604672

202642322948

294242595041

103164212270191267

454755
103163205266190264

243549
1

1

1

1

1127

1
25

13

1

1

112

112

31342

1

1

21
41

2

313

313

1

1

1

1

1

1

1

1

1

1

1

2

2

2

2

1121
123221

1

1

1

1

1

1

1

1

1

12

12

11

1

21

21

21

1

11

221

221

221

6
90145186239172245

1611

4

4

1

1

11

11

1

1

1

1

1131

1131

1131

4

1

1

3

3

1

1

1

88145185225170238

88145185225170238
11

2

88144185225169236

1

1

1

1

444663

444663

1423
212

121

1

1

1

1

1

1

1

132

132

1214

1

1

24

123
1

1

1

1

13

2

2

11

11

12
17413

311

311

311

311

2

1

1

1

1

1

1

1

1

1

1

131

1

1

1

121

121

121

2761

11

11

11

11

2651

1

1

2551

1

1

2451
22

111

21

1

1

116735

1

1

1

1

116534

116534

11222

11

1122

4332

4332

2

1

1

269446393522418517
512511715

1

1

11
121192177233169220

11

11

11

11

11

21

21

21

21

11

1

11

11

11

11

11

121190176230169217

121190176230169217
41

1

1

1

121189176226169216

121189176226169216
57611513

261

11
275932585467

275931585367

87117138156110136

142242210269241280

142242210269241280
3738108

91148131180147169
614964

1

1

1

11

11

11

111

111

1

11

396953776872

14

14

396952736872

396952736872

457773947192

457773947192

212825383019
457773947192

244948564173

1

1

14

14

1

1

13

1

3

4786768080103

4786768080103
1

1

1

4785768080102

4785768080102

4785768080102

1

1

1

1

11711

11711

11711

11711

1

1

1

1

111

111

6

6

1

1

1

1

1

1

1

1531512

1
1321511

2141

2141

311

311

11

11

2

2

2

11

11

11

11

121

12

12

12

1

1

131

131

131

1

1

31

31

69125116135107133
12

69124110135106131
5

69124109115106131
26

68122108103105131
23

1

11

1

1

641149390102124
68122106101104128

48131124

1161

1161

115

115

115

412

412

412

466891558671582683
1

21
8181472320

311

311

311

311

3532911

1

1

1

221145

221145

122156

1212

1153

1

1

1

1

1

2112
51085128

1

1

1

142
51054116

1
481374

12

1

47353

11

11

3

3

11

11

457873539663557660
1044757

448887755864

11

11

111

111

448787735763

448787735763

151914262022

151914262022

1

151813252021

111

388762434555474567
52616201518

153227482147

242656
153227482147

81012271017

5181315624

6111544706365
112

414123322841

21

413923322741

1
207321373522

197120363521

1

121

123119211528
215441211246260275

13141211814

13141211814
1

13131211814

52155578410087
1

297237496447
52155578310087

1127412716

1143333

114213192621

131
264944584537

132626232520

11

122118321917

72815171524
226230272441

16122
15341510917

265111
1

165111

122297616

122297616

294225232937

294225232937

294225232937

618824223931

1
618824223931

618824223930

91145128168111158
1851198

12

1

11

4117113
598970916795

11

11

63217191417
418614512

1114431

1
1114431

1113431

13112
137164

1122

233

36311107
495552654274

882819845

882819845
1

872819845

10181017910

1111
10181017910

1017916810

7999119
282311181512

1852722
1882822

31

36121

341118
314753643555

111723391419

172629242028

21

21

136244

136244

1
15113

14111

11

141

11

11

1620787014462216822867
2814971191120

1195583210

2

1

1

1

1

1

1

651173
11

551172

2

2

46

46

5517

5517

7132

11

1

1

1

1

711

1

71

11

11

1

1

1

1

1

1

213

1

1

1

1

212

1

1

1

1

1

11

1

1

1

1

1

1

1

2192

15
2191

11

1

1

21

1

1

1

1

1

11
14123

142

1

11

4

1

1

1

1

332929483023

4931724

22411

1

13

1
13

1

1

1

1

1

1

1

1

1

1

1

211

211

11

1

1

1

1

1

42

1

1

1

3

12111

2111

1
2111

1

1

1

1

1

1

1

1

1

1

1

31121

31

1

1

11

1

1

1

1111

1

1

1

111

1

1

10

292026312819

261213

1

1

21113

1

1

1

1112

1

1111

1

1

1

1

1

5

5

5

5

1

1

1

1

111

1

1

1

1

1

1

1

1

1

1

52212

1

1

1

1

1

1

1

1

1

1

5211

11

1

1

1

1

52

2

1

1

5

5

179135189

13334143
11

8

23453
1332453

11
112

2

2

449126

7

442126
442124

2

2

1

1

1

1

22

21

21

1

1

1

1

1

111

111

111

111

1

1

1

1

1

1

1

111

1

1

1

11

1

1

3

3

3

1

2

332731

1

1

1

213

12

12

111

1

1

1

13143

13143

1

1

111

11

1

1

1

1

12

2
12

1

1112

11

11

111

1

1

1

14511

1

1

1451

1451
2

1

1

1211

11

121

121

1

1

1

1

1

2

1

1

1

1

1

1

1

1

1

2

2

340186233261125128
23411412

1

1

271

2

201

1

124

1

1

5

14572161

30

1

8

1

11251

1

49

1

13

72

11

9915570287652
2

21

9915369257552

11

398

1

61062649

1

4

11

1

1

71

1

2

1

76652714

1

15

5

1

1

1

157

21

1

1

1

1

1

1

2

11

1

5112

21

18761
1

11

11

1

132

1

21

1

1

2

2

1

82211

82211

1

1

1

5

11

2

1

1

3
1543768107

121

1

1

1

1

163152

1

24252912

832

3414

5184573

1

1

1

2

2

2

2

1

1

1

1

1

1

1

1

3213

1

1

323

22

13

4132242

1

1

4132232

4132132

1

413232

413232

1

1

1

77165131147176153

35342

11

11

34242

34242

18486

4482

4482

2

2

124

1

24

221346
5410166120126114

469259868092

264449
164216213440

1511

1

1

22

524782014
17112

23234
1213

2221

214451512

8658714

12122

7457512

434435

311327

2524

1

1

1524

1524

1

1

133642
11156111421

812341012
142

2211

1

1

1111

1111

61021510
1

11

11

42127
4113

214

1
27123

1

2713

2713

2

2

2

13
113

1

1

1

1

14
1

13

314652
1114

1211

21231
21131

1

163331432525
1

101510796

1

1

211

81410785

81410785

222

222

61619291617
4

4141719128
41417201210

12

12

12

131
111

2

2

2

2

1

1

122224
122244

2

2

2

51

11

4

563283916

2

2

321
313283915

282

282

152

312135

1112115
21

654

11451

1
21

2

1

11333

11333

2

11331

2511
205854233831

12

1

2

2111

2111

111

111

1412

1412

4112

11

311

21

11

1

1133

1132

1

164146213125
152222

1

12

1

1

11

31
133441182621

3153

1

11

11

111

167135

11

2

111

21213555

1

11

1

1

1

7914975

1

11

11

157074198991502430521
211412

141
1

1
131

1

1

1

1

112

111

111

111

1

1

1

12
12262428815

3125916
411

121

1

121

2

11

12311

21
12253

1

1

1

1

22

11

4231

2

1111

1

3

1

1

1

21

21

1131

111

1

11

3111834

21062

11

11

1

1112

23253
112

11121
13151

2

3

1
33111

1

1

1

21111

1

21

11

111

111

111

445128361574

34442432861

1

1
34442432761

82856
1

82756

26151926761

10744713
12

32111

211

1

3

6433411

12

73494104

1

1

1

11

1

1

1

1

1

3301133
11

8

8

222

222

2

2

19

19

111

111

1

1

446271

4

1

1

1

1

422

422

1

1

1

1

2

2

31

31

11

11

3111

3111

3111

4255532
155211335041082189215

492234

11

1

1

1212

1

1

47

1

7411182427
22040104186130114

1

21
211221

1

1

1

1

1

1

1

1

1

1

1

1

11

1

1

1

1

17821841399663
2431415

12

1

21

1

1

1

2

2

1

1

1

2

31

1

1

214

25

1

1

2

48

2101

11

1

621

1

2717

1

1

11

1

1

1

1

1

1

1

1

2

1

2

1

1

7111151

2

1

11

1

1

1

1

7

1

2117

1

813

24

1

1

12

2

1

1

4

11

21

2

7

72521

11

35

15

91

2

2

1371

1

34

1

1

1

3

1

1

11

3

1

1

3

1

3

1

1

11

11

1

11

13

2

11

11

1

11

1

1

1

1

1

1

1

1
28

27

21
2211

2

2

1

429

29

8

1

2

4

1

1

18

18

18

1

1

1

1

1

1

1

1

1

1

1

2

2

1

1

1

11

1

1

1

1

1

1

1

1

1

12
1

2

1

1

1
11

1

1

161

161

161

161

3

3

111

1

1

1

1

1

9

9

9

9

394983912047
1281013

1

22

11

11

11

11

1815613
501

1

89

453

151

111
11

1

1

1

1

1

2
6

4

3

1

513
25150741325

34

2122

1

238

1

3

3

435

112

1

1

2

4

1

11

116

3

22

5

1

1

1

121

2

18

1

6

6

6

61142311
4

612310

2
612310

1

611

2

3

1

13
14

1

135

15

15

119
2

1

1

115

1

1

1

1

1

6342

6342

15209852474773252
117144

130

130

3
32114721

12

2

2

1

32113211

32113211
31

2

1

1

19

91

1

1

1

1

1

1

1

1

1

1

1

1

1

1

139

11

38

1
2

1

1

1120

1018

12

1514239852732444
5423211

1

1

81211

3

9

21

21

3

4

29

1115

121

1

139

11

715

32

4

1

1

1

1

301

1

1731

1

169197428

1

2

1

1

1

1212

14885872747

1

1

1601

23

23

23

242

11

11

11

1
31371

3137

3

11

12

24

14

11

11

11

19231

19231

2

2

17231

43

13201

718143161545

2254
16954314

226223

1712

1712

1137115

5241121230
111

243614

10512

4264513

4

1

1571

1571

571

1

1121

1121

1121

5335109

111

11

11

1

1

1

1

1

332131

31

31

1

1

1

1

1

113

113

11

11

115
1

13

1

1

1

1

1

16

1
11

1

5

3

2

111

1

1

1

1

1

1

43103811
3132951801723

2312632
202276851417

23261
1

5

2321

131271481013

34

34

4292712

4292712

56591389

32355

2456839

143412

143412

34241
11

1323

1

2

11

6664325

6663825
6664325

5

11314

11314

11314

11

11

11

11

2
813914810

234322

224322
1

21
23121

2111

2111

1

1

212

1

1

221
23343

3123

1

475625

16253

2
16253

11

5233

3111

2121

211
2111

1

1

457397

14335

14334

1

3211

3211

1
14151

31

3

11

1

1

172514211822
1

1
152212191722

12173

51

51

112

1

1

1

1

1

111
1

1

1

1

1

2575513

1

1

324

323

23

3

3

1

2521

1

1

1

121

1

12

13
22128

1

111

11

1

1

113

113

11

1

1

1

121551256

111

111

4121511

11

1

1

131

1

101

111

31

31

31

533634
221

1

1

1

2

111

1

1

1

1

11312
112

1

21

23211

112

1

1

12

1211

11

111

12011

1

1

1

20

242
3640248711175

1
3239208410360

1411

121

11

1

1

112131
121766127

443272

381323
24123

1412

321
21

111

1

2

224
11

13

11

1

1

13

13

111
211111

11

1

1

1686708845

1465708844

21

111

1111

1111

9
91

1

111

111

11

1

1111412

1

11148

4

231
331

1

1

1

1
7261191012

191234

112

11

6111

1

5

1

1

1

2

11

1

11

11

61510676
1121

1

1

1

11

1

22

1

211

11

1123

1

3

1

1
144112

1

1

2

111

11

1

1

1

1

1

1

11

1

1

262385

2311

2311

2311

32275

31275
3275

1

1

1
