# Supplementary material for: Characterisation of the Carpinus betulus L. Phyllomicrobiome in Urban and Forest Areas
Source: Front Microbiol. 2019 May 29;10:1110. doi: 10.3389/fmicb.2019.01110 (PMC6549492; doi:10.3389/fmicb.2019.01110)
Supplement: Supplementary file 3 [file Data_Sheet_2.ZIP › kaiju.out_all_Wa.html]

Javascript must be enabled to view this page.

magnitude
magnitudeUnassigned

kaiju.out.epWa11
kaiju.out.epWa14
kaiju.out.epWa22
kaiju.out.epWa24
kaiju.out.epWa33
kaiju.out.epWa34

421249430626822982130424022447191303925786

299617762142145214891567
421249430626822982130424022447191303925786

420787530592082978101423817547171013923475
155901393612823110511048110172

55716429267569738470151054130435
1003136031271708519570

22521

22521

22521

22521

241410
1161203021542062

2

2

2

2

73851881001146

7384188991146

7384188991146

7384188991146

11

1

1

1

1

41319844916

41319844916

40319744916

40319744916
115121

39309243715

1

1

1

1

10162713110
20129554131591120

121
801072071363236

1

1

1

791042071363235

791042071363235

73972011363235

676

12
1091703061645873

221

1

1

121

121

1091673021635873

1091673021635873

2454
1091673021635873

1071672981585869

22121

22121
2

2121

1

1

121

1
678433

52123

52123

52123

15722

15722

15722

15721

1

38231272
75794324651541329322

51464317701153230203

1
55402623

55362512

1

1

1

1

55352512

55352512

55352512

55352512

411

411

411

3232146711110
49863517291127210192

2152627213627081
620441743

57732191222432

1

1

2

2

57722191202432

57722191202432

211

211

1501684572234246

1019523557
1501684572234246

6165188911418
264410964511

3520782797

11

7984217972321

1142

1142

1017462326
250340858692129101

18323655851310264
815692851

921

921

1031522883777445
11

353559243489

671172291342536
22411

651152251332535

72691921062317

72691921062317

72691921062317

57872541562531

57872541562531

56852521432331

122132

1131188

1131188

23
1131188

711146

2212

1631805142987377

1612

49

49

49

49

1

1

1

23

23

1621805082866876

131

131

131

311

311

311

1581765062866876

2212

2212

2212

1561745052866676

1561745052866676
632

249532
1531714992836476

1511674902786174

33

51

51

51
3

1

11

134212

1212

1212

1212

1212

12211

12211

1111

1111

1111

111

111

111

51

51

1

1

1
76104152761838

1111

1111

1111

75102151751838

13286230415
75102151751838

375314
1

275314

48547235718
4318311

44515432617

111312761
35663

2421
886131

64611

741242761893569

1

1

1

1

1

1

731232741893469

731232741893469

1
731232741893469

731222741893469

11

11

11

1

1

1

1
6148291229585267242

11
131617131318

244389

142125

142125

1

1

1

11264

121

11143

1

1

1

1

122
1111121049

81112747
123

12

12
2

1

2

2

1
5810632

32

449612
459612

1

1

11

11

11

11

11

1

1

1

1

1

1

343
12

131

121

1

2

1

1

4411434
5988131207569254224

5459139651624

12

12

2

2

5458135651624

5358135651624

1

5357371003471232193
1624351454

111

111

31748057124615898
2122492339

39366035766

39366035766
1171

38355334766

88134176701426

36496435715
88134176701426

3662691737

1321421833

32111

325567281113

325567281113

325567281113

6711292412421
2524

375549171410

285241201011

61105110371919
1393421106

466154
48967316813

3678531426

3678531426

81214113

81214113

31

9161712114

9161712114

9161712114

952

952

222660341415

2416811
222660341415

611241252

14102014812

1

5531
7174102591420

2623482679
1

1811

2622392578

40465132711

1

3946513279

2

1091332251134053
4515202

29346132925
41622

1213311366

13202417119

7494148613025
7518731

3

3

21

21

912181859
212537291010

439931

2872

411211

8101022

8101022

8101022

5720811
446192251714

1288144
2219297610

33912
341012

11

771156
651135

1221

5181147

5181047

5181047

1

121732633

121732633
1

121632633

2111

11

11

1

513542933

513542933

513542933

3944161128647208293
12414456

186209566307107139
431145

2112
16919355030389126

21113
141935121113

36211

2

1

1

32

3111

123
4918475

2

2

2

2112

1

1

212

21

1

1

1

1222

1221

1221

1

1

131

131

131

11

11

11

332

332

332

11

1

1

1

1

212

1

1

12

12

1

1

3314524

2212

21

211

1114412

1114412

1

1

1
4322

3322

212
3322

11

1

1

1

1

1

1

1

2411141

2411141

1211141

1

1111141

12

1

1

1

1242

1242

1142

1142

1

1

14416349727770104
11

165

165

165

1

1

1

211

2

2

11

11

1

1

1

5

5

5

2
14116148526967100

1

1

14116148426965100

14116148426965100

1123

1123

1123

11

11

11

11

113
1

11

11

11

1

1

1

1

1

1

1

1

1

1

121

121

11

1

1

11

11

163

163

163

163

112

51

1123

1121

1121

1121

21

21

11

1

1

1

1

1

1

1

1

111215261

655

2
655

11

11

2

2

22

22

131

131

1

1

341

341

341

341

84

82
1

51

51

1

1

11

11

2

2

2

131221

131221

131221

122

1211

1

1

1

1

11

11

11

11

11

31
19320054633695146

111891951316494
1

11521
111891951316394

37722
1

32321

54

11

11

107881831226090

107881831226090

821113462043152

821113462043152
1341

11

11

42512191411725

42512191411725

21124

21124

3658124581222

3658124581222
1

3558124581222

2

2

2

2

2

2

1

1

1

1

11212

1111

1111

1111

1111

21

21

2

2

1

1

2

1

1

1

1

1

1

1

1

158433874

12581

12581

12581

5211

5211

5211

2136

2136

1124

112

83292042

83292042

22

22

4111

4111

2659803488

12
2559783488

11
2459763488

1628492285

1628492285

1628492285
1420381654

5541

11413

1221

73127113

73126113

73126113

1

1

11

11

11

1

1

1

23

23

23

23

17361328291015577951207

9040159856282
17361328291015577951207

40171382939
1

187551418
3112

72328

1111
72328

1

12

5117

222

1
222

212
21

2

122

11

11

1
111

11

21122

21122

2122

1

211173

211173
1

2

1

21143

11

11

11

2

2

2

2

2

2

1

1

1

1

1112

213

1
213

1

1

12

12

1

1

111

111

111

112

112

112

112

122
16712119

1171286

8443
1171286

21

11

1

2

11

12

12

411

411

411

254
1

243

243

231

12

1

1

1

1211

1211

1211

1211

1

11

1

1

1

41225

474396758365153299
181817

411372751353118229

1

1

1

1

405370749353112220
145259210

1942424571845191
41722

229632748
881032641222947

3349100491523

3345101461016

3345101461016

102138186602242

102138186602242

19712326716059119

22202211107
19712326716059119

9747105692448

9747105692448

7856140802564

62169

62169

62169

52168

1

1

2217
1

2216

2216

8422210

8422210

8422210

2148

25
21

1

3

2123

2123

15513613
1112

21

21

81234

81234

5237

5237

1810361312

1810361312
41111

12734108

22123

2113

23

11

21228

112

112

21116

21116

21116

111

215

6435121248709328410
9254513

1

1

1

1

5340103631219
5304351054599259313

594757312422

594757312422

594757312422

243339

243339

243339

2
252210626359109134

241203622358108131
13211

1

108914102416875

1

1321041951053754

514102

1

21

412101

1

1

1
11

1

111

111

1

1

9721

2111

761

416323

1122
316323

11431

1

1

1

1

1213

213

213

1

1

159133257140108123
7514733

714992465546

714992465546
22156

694990455040

8179151875074

8179151875074

121

121

121

101241711

11

11

1

1

1

1

8111167

8111167

8111167

12

12

112

11

1

1

4653
92631831055572

112
122

1

1

8656174994751

1

1

111

111

8556174994550

8556174994550

114
3

1

1

11

11

215
13

22

22

1112

1112

1112

1113

1113

1

113

1

1

1

42

42

31

11

11

1

1

1

1

11

11

1

1

1

1

31453110714
484358731386218363

1149

1138
123

2

2

1

1

13

13

11

11

1

1

249192429261112185
121

62210

23

23

23

627

525

525

12

12

241190425261111173
12152414413

228

228

13

11

2

6645125842735

6645125842735

1112
111

2

493295601930

493195601930

1

11

11

7335

7335

124
105921801005378

8685171873953

731349

731349

10481079

1

112

111

111

12

12

12

179982830
1

1

1

3112

3112

2116

2116

2

2

12

12

96872719
121

1

86872418

1

1

18411125910565121
231

13

13

34

34

121

121

1112

1112

1

1

161952541035791

161952541035791

161952521035791

161952521035791

1

1

1

3

3

692114

692114

582113
692114

111

81116

81116

1

1

2110

2110

11113

11112

1

1311

1311

211
27632254428911097

1051082351496157

2

2

2

11

11

11

11

11

4411936
1

113111

113111

1
1111

111

11

11

216715
3

2135

1241

12

12

12

12

1

1

1

1

1001022221375851

94972171365850

94972171365850

94972171365849

94972171365849

1

1

65511

65511

65511

65511

31

3551

2621

2621

2621

2621

1672103001394739

1652102981374739

1652102981374739

1652102981374739

1652102981374739

1
222

1

1

1

1

22

22

22

22

23

2

2

2

2

21

21

21

21

1

1

1

30213309812627459081395
17204324110

1
20517448427283130

2

2

2

2

18915544625180118

18915544625180118

18915544625180118

1

1

1

18915544324980117
18915544625180117

32

15251119

13

13

13

2

2

1

1

2231119

2231119

2141

2141

2141

2141

634221

52211

51211

51211

1

1

11212

211

211

111

111

61042

1

1

1

61041

61041

61041

5

5

5

2

3

1

1

1

1

83103207922544

83103207922544

83103207922544

83103207922544
1

82103207922544
22

80103205922544

1041523011503444

1041512981473442

1041512981473442

1041512981473442

11

331

331

1

1

44131112
1937199352731241462736

147815094097582231359
221011

1273
1271544341684542

94115297774030

94115297774030
2929771665

381221

1

371221

1

142536838

6897265

2932961435

1

1313663016

1313663016

323713088512

68251423
323713088512

812432024

812432024

1

118333814

1

4415101

34145

134913533653413185317

134913533649412185317
1

2

333912844145
134913503649412185317

5147811
3255411443

122018421

152116211

71717419
1141373771241735

15111013

84993341171326
141217710775

11

4

4351

84317

1

321

1

1

22

1

59123

2

36369446

15205022

213

11

810162

10989832983203125244
3582

12197322

11
139121101

129111101

122011221
896121

41151

2
351012

35812

1127151013

946804269318089217
1620996

927783267617185209

31842

959121941718
84861954815

2211

5

1251

831731

33822

3451

69132115272529
41420622

203547737
581231

142735435

11

174331356

16351171212
17122

22

13261071010

21
1256432

23331

823121

41

41

41

41

11
4554801163648230375

484671462430

484671462430

11

11

11

484571462429

1
484571462429

484571462428

4064341091602206345

4064341091602206345

4064341091602206345
531482

209212400217114187
285611357128

457181331930

457181331930
215837231011

1

112

11729317

2

12210

2

1112

1

312

6131

284

9245105544084

9245105544084

49161375

49161375

221548451849

221548451849

171537141711

171537141711
2

171535141711

1111
1

111

16616553829884138
6530835

363767201218

1

363766201218

12412344127069115
20158356129

2917149992147

1717331166

112435281128

1717522538

1414412797

1212361647

4712833

265413979818

265413979818
153210859614

13
23

1

451

11

24107

6781111

6781111

11
211

11

13422

3151143
6718601813962302429

161

161

161

3412
5797111446767263383

292379691354171242

1

1

1

1

291379672342171242

291379672342171242

2352

2352

2352

1191562821387852
289376667342171240

10613221112571134

3

3

1

1

10513220812571134

10513220812571134

6388174792254

6388174792254

6388171772254

32

32

1

1

1912

1912

1912

65

65

137

4

43

43

11

8711242
27632274740492138

901032281192065

901032281192065

901032281192065
511

1

1

851022271131764

152

6253131752220

6151131752220

6151131752219

6151131752219

6151131752219

1

1

1

12

12

12

12

7411412
1161593772084651

761082411163435

761082411163435

11
761082411163435

1

751062401153434
4613734

711002271083130

111

3347125881114

3347125881114

3347125881114

1

1

1

1

1

1

1

1

1

1

1

86771

86771

86771

86771

86771

921453461833543

921453451833543

921453451833543

12

12

12

1

1

1

921443421833543

921443421833543

921443421833543

1

1

1

1

1

475411

475411

1

47541

3214923

3214923
1

11071

224212

1
36662

651

651

651

34

34

1

1

2

2

2

87122177882521
44918310694993125771146610124255

682641171199614621214
43888295414630723945146338123844

1927713489
280731104357246412341030

154113891191615335323

12
154113891191615335323

154013861188614331322

111172
154013861188614331322

1124
153913841187613323319

153913831186611318319
510821924405191214

874309971507629

155253165565176

1

11

11

1

1

31141

31141

1141
1

11

1

111

1

1

11

11

1030144225011488754571
69821

52531851044133

2
12

1

1

51531831044133

51531831044133

51531831044133

53

53

53

53

65991911033425
972138023031379712538

456344
15023244423211496

3063132772516

3063132772516

6114021107
1161643061528576

2847104683224

82106162634345

520794989643413259
435386362328

14306221813
237396450391281122

12223823132723141
19136117910

394965182411
317432

173031552

173031552

1918279167

1918279167

6417610529219820

101128156424268

101128156424268

1

1

1

1

5577113522317

13
5577113522317

5576110522317

5576110522317

1852683401648692

712352393
1852683401648692

92112179795647
91107176785445

532

112

1

86143126622142

86143126622142

4537156993027

4537156993027

4537156993027

851092921716049

851082921716049

131
851082921716049

841052921716048

1

1

2
1071092311316182

1071062301316082
4543119812943

15154815916

474863352223
1

474862352223

111

1

11

217252594327137127

217252594327137127

217252594327137127
3661149761714

1

1

3947101682821

3947101682821

1421443441839291
51449221311

11114
4745138774529

4744137773425

1

8985157843451

8985157843451

761
3905561542907219235

17721861532610897

1181393862107072

1181393862107072

1181393862107072

1
1181393862107072

1

1181383832107072

1181383832107072

11

59792291163825

59792291163825

211

211

11

2

59772281153825

59772281153825

11
57742281153825

56742281143825

23

213336920575110136
141

2
1142154272636477

1142144252636477
1212422

1

1

1854112801823

1854112801823

1

1

941583001794452
817583759

4781150833035

3960925998

1

1

1

981194933084559

981194933084559
1312261529

1

1

1

1

49553262052317

49553262052317

49553262052317

3650141872033

3650141872033

1

1

11

11

1

1

1

1

22

22

22

12

12

1

1

129131055
4786501164598152158

2647468108

2647468108

2647468108

2647468108
101427666

8167231

8171211

1
2232994712524866

61326912

61326912

61326912

61326912

1291692661462423

1291692661462423

20444930719

20444930719

109332313
2524523268

1

6813712

343222

53321

5515521

5515521

5515521

5515521

38436330813

38436330813

38436330813

2032745903128775
153

1381804192364848

495429777

495429777

414436532522

9192444223

9192444223

3225129319

48823541761619

48823541761619

3716621

3716621

3716621
1

3715621

5279107543422

224
467086472921

91916327

91916327

25282612410
263540281811

171416141

1114281293
1112281293

2

6921751

6921751

6921751

98431334

98431334
51611

1410311

1410311

22311

22311

17411

17411

11741

1421441624

2102
1421431424

5813613

5813613

71320611

71320611

71320611

12

110768236189215

85586116163176

251821202639

75492921441204446337
109191632

981293342097042

981293342097042
1

2

2

2

981293342096742

981293342096742

981293342096742

981293342096742

250265564318111114

250265564318111114

250265564318111114
831082551383724

413770381214

413770381214

1
7346102711820

1

7346102701720

781084
5273137714454

212769411818

243858222236

112

112

1521331486
3965261227661262179

2

2

2

2

951134942559653

951134942559653

1

1

951134932559653

951134932559653

4

2

2

2

2

2

2

1612343582058883

1612343582058883

1834775156
1612343582058883

5662112752921

3348117622014
87138169795456

47711

548645103341

1

1

1

1

1251583351867037

1251583351867037

1251583351867037

1251583351867037
12

1251583351857035

1

1

1

1

1
1382015502856564

991583511805139

991583511805139
131

991583501775138

39421991051425

39421991051425

1381

1381

11
1381

127

1

31108

31108

349291532273853547
38365232043423217135142417120502

1327615299613191
8380777391404629229387855

127106179
18342696218074719811006

133323271388317942631

133323271388317942631
1332820326

2254

2254

2254

120821501202238710565

413279296132
120821501202238710565

10221967958102512418
659661204141

957187189782471377
1

956187189782471377

11

231727173125

231727173125

264352302527

877971525155

877971525155

877971525155

811158298
1

811157298
2

811157278

1

2

2

1101721585919356

1101721585919356

1

1

1

1101701585919356

1101701585919356

380248501266837297
181339214317

1

1

1

1

4

4

4

4

88681075016570

88681075016570

321949196819
88681075016570

213
191618103716

171518103416

1

333140195733

42132

6942563916162

6942563916162

6942563916162
112

6541553915361

3161

204125299156464148

11475246
204125299156464148

341644128926

341644128826

1

181540293921
159105248139351116

17940205112
18942205312

122

12

583977379232
543469358226

3582105

11

231191

211332185223

4225563410427

4225563410427

10711228015818267

10711228015818267

10711228015818267

10711228015818267

10711228015818267

10711228015818267

2112

1
12

11

11

11

21

2

2

1

1

21

21

21

21
11

1

1

1

1

1

4648391453773124172905823
52426626325104

23146112

23146112

23146112

23146112

54381105412847

54381105412847

54381105412847

54381105412847
1273011388

291942244820

291942244820

131238194219

131238194219

12010419710330396

12010419710330396

12010419710330396
1262993913

70661177516746
1082310389

223164476315

223164476315

382730186622

382730186622

383251199737

383251199737

39292316496132
14139221027621107082897

111268575245886102338
80354830779227

1278694391116275

1278694391116275

1278694391116275

14

14
1

2

11

2

1

1

1

1

1369785441099322

1369785441099322
212

2713121329593

109827231802229
1153

109817231787226

88588230808215
66040544330147531321

611913689

611913689

611913689

2011081531091462413
46274018400122

50424735357111

1871479424
50424735357111

81113711241
44858329

34222111

13381

2122141813142
2424202115146

11

1

222

1

2

1

2

1

1

11

21

1122

11

21

3

1

1

2868413536
4414221925273

64772614
4444156

21366

2

1

131

1

41354114
41343613

1

131

1

1133245
1343316

2131

4

5193

5193

4819282233779
4113241727261

3425347

4223111

841078521
136161511628

2246

544257

3652281991492415684
109556648805231

4748291223967

4748291223967

3729131324886

3729131324886

4522192119964
5426242822568

31235

3312101

322113

72445232682161
65424831616147

32267

4221407

4626151621671

4626151621671

107618144820184

107618144820184

107618144820184

11368

11368

11368

111

111
1

1

1

3204

1

1

1

3194

12
1

1

1

122
2174

3

1121

1

946411254443107
1

926411254443107
11

926410552442107

926410552442107

1

1

52

52

1

1

1

165144140931139316

165144140931139316
76986117

11

11

97766949581150
1

97766948581150

97766948581150
6035513443395

711363223

303015811632

61606236496149

60606236496148

1

1

1

1

300728053963231458152677
184159223127496143

127914381822104021571145
7410082336019

371523135912

371523135912

371523135912

371523135912

371523135912

64781137510876

211132
64781137510876

182238264326

182238264326

182238264326

445574486248

445574486248

293382548521

293382548521

293382548521

293382548521

293382548521

81286310725921369792
493744278830

1122
16820018059187115

504954187526

504954187526

504954187526

1

1

12

12

1171511244010887
181621156024

1991511417

77546

7311983202846

1
14302884718

11
1

1

14302784518

13302784518

13302784518

1

3

3

3

444041335743

444041335743

1
444041335743

444041335643

391250217934

391250217934
42

1
351250217931

351250217831

1

212751384730

212751384730

212751384730

212751384730

74961034311959
226143

232152333917

232152333917

48724497639

48724497639

48724497639

111

11

1

332
513376478665

242335224725

242335224725

242335224725

1

1

261038253638

261038253638

261038253638

546684728765

546684728765

546684728765

603850359680

1138516
603850359680

11
1681261931

1681261830

332730246149
281926195940

584529

7169954413781
144

41
433851258944

312332125416
433851258543

8131051021

8131051021

11
4298216

3298206

273140154837

273139154837

1

13

13

5459916110038

8511
5459916110038

181730173810

181730173810

364253396127

364253396127

568860379563
14

325842234326

325842234326

233018144837

233018144837

344054367833

12

12

89184144
323853347628

71020162311

71020162311

171915143913

171915143913

1

1

211213
1

1

1111

11

11

222665306035

222665306035

3144
222665306035

222662295631

123

123

123

263349450273476225
1071710193

23168121
366975308522

204027104811

204027104811
6932114

627164172

8484205

8484205

142632122510

142632122510

142632122510

142632122510

53511237615651
1

262867429125

262867429125

262867429125

262867429125
12111310429

101763512

4164726144

272356346426

272356346426

272356346426

272356346426

124223
1191331569011895

639783225139

639783225139

639783225139

639783225139

553469666553

33264
553469666553

341733372523

341733372523

181733273426

181733273426

133

133

133

133
123

1

448976649854

232
448976649854

297461406935
2212574

41815222716

41815222716

235434133515

235434133515

151315242617

151315242617

151315242617

626376419849

616376419447

1

1

1

616376419347

616376419347

616376419347

616376419347

142

131

131

131

11

11

2616632214731
148211451842110630641340

4017533112841

4017533112841

4017533112841

4017533112841

4017533112841

81601206418881

81601206418881

112116157
81601206418881

3725562610536

3725562610536

1
433443226838

433443226738

121063
1238217111025793

393173478824

393173478824

393173478824

3

3

3

432244219538

432244219538

432244219538

432244219538

402744366831

402744366831

402744366831

2141
402744366831

382744356430

4021613111643

21123133
4021613111643

191420136018

21
191420136018

191420135817

19629154322

19629154322

19629154322

243

243

123

123

12

12

111872
129120197137298126

13134264
88781478022293

212935225324

212935225324

212935225324

191635226723

191635226723

473064327642

473064327642

404149496931

404149496931

404149496931

3131504310037

3131504310037

3131504310037
222038318125

35495

35495

61178107

61178107

311233332186627289
1482135

5125

1
5125

4125

219154199116357196
11

218154199116355195

8558884218470
218154199116355195

191330164631

454028226335

12517151317

12517151317

573836214942
17111893120

20762109

20201210813

2

1

1

3936664212033

3936664212033

3936664212033

3936664212033

1
4738592613550

4738592613549

4738592613449

4738592613449

1

9974186
130981437417469

715178427836

715178427836

22

695178427636

695178427636

503858287827

503858287827

503858287827

503858287827

202239138929
5694676524081025527

171127154116

171127154116

426462
171127154116

3167

10815112814

2243114
435076409647

253052243322

11

252951243322

252951243322

161820135221

161820135221

161820135221

322382
6639837711144

421645424019

1131
5391174

4391043

371336313315

371336313315

212136326323

212136326323

212136326323

161227133117

1

1

161227132917

161227132917

1

1

3827393213338

3827393213338

3827393213338

112

112

12

1

71114
573860388938

142633153016

142633153016

361127234818

361127234818
1

361127234718

121115102416
237186213120296219

13191613

1313513

611

212

211

1

19152783718
15312612270172126

1351443726

1351443726

2

534732233237

384834254035

281115102610

281115102610

241623184212

241623184212

241623184212

111
353242205052

13
353242194951

343242194651

363443326138

363443326138

303143325034

63114

384845287739
2

182632173717

182632173717

182632173717

202213114020

202213114020

202213114020

1766108714316593054835
7422531917132

6053405632471296338
152115316

278222292124462186

278222292124462186

19012519896385113
278222292124462186

1227236910

20177398

6494174

504955154251

1

1

1

1

256174908

2

2

2

256174888
124

216112748

216112748

2610
11

123

136

1

1

164561146038677

164561146038677

164561146038677

164561146038677

1

1

1

1

122541295432760

122541295432760

122541295432660

122541295432660

122541295432660

1

1

111

111

111

111

1

11

465381319113375139

465381319113375139

465381319113375139

484842136817
465381319113375139

21

712335

2112
876671307227

856671306125

776433207627

563161237214

14

1

132

2111
188160109277446

188158108277345

6213434962801211326

6213434962801211326

167811506440284
6213434962801211326

30161613579

30161613579

30161613579

322111115923
563226259637

21101193213
189872213

313210

314551

314551

241923165010
8950595214771

171510152735

144871512

1

3

301218135414
212142

281217114012

11

171314114818

171314114818

171314114818

31

31

735697648325
10114132

1591010242
332724183410

6262

121614846

302872423613

46303595921

46303595921

46303595921

14365964231960

14365964231960

14365964231960

29636151402456012233118626112100
46268244145107

29104146442363511695114598110081
564010257138177

2784813299206219964113281109086
72315828212207

85735955428426052633
2759613124202779749112822108700

1377111269232190

1377111269232190
14

42143

42143

42143
2

21111

21111

21111

21111

213

213

213

2107
451715107763

26141184046

26141184046
231

125
6149

121

121

1
43

22

1

1

1

1

4211415

1

1

4211315

4211315

88351010

1
88351010

111

882499

882499

5253810

1

1

5253710

5253710

11211

11211

1111

2

1514248
1

9112

631

631

631

381

381

381

613136
11

31

31

31

1
61295

3184
61195

111

1

11

2122

2122

2122

2122

211

211

211

1

1

1

21

21

21

87529659151120

87529659151120

87529659151120

87529659151120

87529659151120

543552358158

543552358158

543552358158

543552358158

543552358158

543552358158

335
1850574321295951329873795384

1850174321295151309872095359
262104146618781018

4923303611809561153
75348

1918412876347352

1918412876347352
26152596246

582034239385

582034239385

582034239385

331722156564

331722156564

331722156564

2613743239
74324729126157

20719124554

281221134964

1

1

1

1

1

1

106637532153160

106637532153160

106637532153160

61786628102109

58756628101109

58756628101109

58756628101109

331

331

331

1261008944350523

107696435263372
1261008944350523

193125987151

1

1

27131746121483863975371
36271996157

553350187585

553350187585

553350187585

553350187585

553350187585

155915834319293
24321609102976658514765

3113510

3113510

2113510

1

76515821111114

76515821111114

76515821111114

22
19711310958359349

83445940177159

83445940177159

83445940177159

112695018182188

112695018182188

112695018182188

91735133244205
2001135380365050573999

124844757321245
108575542748431442216

451620105972

451620105972
1

441620105972

11

11

212984651309244
431915158057

124
11843171211682

11743171211478

51361424113105
1

50361424113105

50361424113105

111

111

54343923143143
15510611956298274

1
611144811

611134811

36171895246

36171895246

1572062624
30162885141

634192

96411615

181112102316

181112102316

71371810

71371810

4411137

76582551160181

76582551160181

46639216325919911195
2262

11

46238716325719841193

46238716325719841193

3

3

111

61653

61653

143994530170132

143994530170132

143994530170132

119656334222238

119656334222238

119656334222238
1124

118646334220234

4828211111992
4863071834811941123

2157

1

2156

1

1

2361747024392402

2361747024389398

34

2

1

1

2001059013677620

2001059013677620

1

775434218384

775434218384

775434218384

775434218384
1

1
775334218384

765334218384

1

1

1

1907711645410464

1907711645410464
219932438

90225424164198

90225424164198

90225424164198

79465318222228

79465318222228

12

12

89344927418426
1503452521122940499048987817

52184121199316
133367273144942485957

151537339449622
78340043825722522945

269171571105

269171571105

5251433

5251433

1

1

75435221268391

1
75435221268391

75435221268390

102422538
79395833253280

39182917145152

39182917145152

301925148390

147877646437521
62253118199254

53361818107135
74532821179224

4175

134334864

22567

29265

158

1191775943

107655231291377

107655231291377

66423424163217

66423424163217

66423424163217

351419774104
125587542295391

58293513147193

321521227493

1

2211117

2211117

21

21

21

1
2131221278510461544

1428167637481146
2111211278510371527

47183011193268

2222301196113

21916
114

113

1

124

1

44

11

1

1

1

1

3231021
285131123857511151

1

1

127534729321424

127534729321424

127534729321424

155767653420705

155767653420705
1114

154757653419701

1346744861037035448559181196
111

1

1

1

1
1346544861037035448559081195

281487918466811614515282
1346544861037035448558981195

282891414513471329

282891414513471329

45551543375112813037528854
99083358820027516663063131

53531815444914703625534277

314
257928530759760

254928530758756

203689837708692

203689837708692

11

145607929232238

145607929232238

145607929232238

145607829232238

1

4521420

1
4521420

4321320

4321320

4321320

2

2

2

7452392512899
511934623660270468726768

57312717108123
19261391107653729702842

123083857032416531283
10364513013396

19812011759328249
212410

621

621

13483179
115677344202145

622435167571

622435167571

4039302511065

4039302511065

7350411512093

7350411512093
322220106454

1113

40282145536

1

1

22
21

1

745228137869
506420184103444394

21

21

8332116
110927939162132

1

595852279168
1

585852279168

2

433024105858
42312112

39282193746

3202767751204192

3202767751204192
2492235944156140

2119741416

3928913034

1162242

511159
26615914285456349

68613216124107
544221610477

817119821

11167

5152

1

1

1

1461142612
1939710968326233

7750423014386
339

7450423014077

102415634157135

1

1

1

4725251010463

4725251010463

4725251010463

167652412
110485137188132

2516864227

2516864227

11
6925372612293

3381183743

361725188450

1111
361725188450

351725178349

1

1

14
298199287124456416

1

1

239159235100361337

239159235100361337
1347810353218186

559254

222

122142

1161121813

2754127

79244

22141

1782421023

211

961351432
7472918

2263514

218131

561731516

417274

415852

8333116

752289

546626

11

778365

434174

224248

111

594052239178

1

1

593952239178

593952239178

593952239178

1

1

1

1

341323191727531020

341323191727531020
213277

1085930137776

40241372917
1085930137776

91512
47181343545

38171343033

2017421314
32

1715421314

1

23126215857668937
12922144449

21

15218810923415535

67652720207352

11

1

1

106577879154127
311920192545214237743827

381913192814
10665856279951001874

976711158117104

82121215
976711158117104

321740122520

1

321740122420

321740122420

161936131724

161936131724

161936131724

412934316345
22631620

212144

342125244219
372626274321

11

1321

21111

1

1

1

1841041013
229156237155301295

332024134851

332024114851

15241413
332024114851

18182473438

2

2

29151162914

29151162914

29151162914

502972548373
993013189

21814192321

21814192321

21814192321

7497911
201228224243

8711683
8711693

1

14342
51892429

4452025

112

998812078131144
610536

1

523980486896

201441263560

2229

181439263351
11

181339263350

322539223336

322539223336

2517963715

2417963715

2417963715

1

162225242327

162225242327

162225242327

398527215634

398527215634

398527215634

398527215634

398527215634

393735306242

393735306242

393735306242

393735306241

393735306241

1

487155108633285196
4112949187

17364464639671

17364464639671

17364464639671
14348373356653

301691283018

92111719

92111719

92111719

407681012

712
407681012

1364365

1364365

2011527
711115

31

1312

328271822

328271822
941576

5121

311

15311815

192624410512665

391710212516
192624410512665

64167415219

852727434828

11

3112

136669679152189

735576
136669679152189

17101092721
633440347868

147574
57781112

21

43327

427192

427192

371516163133

371516163133

361224224022

361224224021

361224224021

1

1

1

1
271727182486

11

11

261726172485

261726172485

1

237

237

16331102156589722242248
171720172920

1331
321180238132444420

532956227588

532956227588
301237145259

1

323155

323155

1415831216

56266
68368

1212

1

1

1

267151179110365331

585036248696
267151179110365331

2

1

1

158610207

128761713

63458

1255485

1255485

1

15101545716
19192366323

498267

1531542017

1

19714101521

9274728

9633911

241327133326

241327133326

1167589

1167589

441016154542
256551926

10487167

933109

1011962824

1011962824

684046298782
1174831118568816281689

432529187696

422528187391
18911103625

7931518
1610942340

1111

944

54317

1

86841326

86841326

1135

1135

22234
1296918090220235

333

333

16736172120

16736172120

1086214271193208
401631237359

2

13121051515

13121051515

16101441531

34755

6333814
2137

5156

13111

10234192023

10234192023

95158

101538154550

1153

121321109
207150181110265281

83788638116126
242321102917

416124
516126

12

4161465

4161465

1
1691253238

1691253237

1351062530

1351062530

513151173
613151175

12

442147

442147

1
5544916

5544816

62222

62222

211834214332

211834214332

211

211

24816181511

24816181511

24816181511

1

1

301417144866

1

16

301317144850
311813

271216144037

153136
142135

1

1

11

211623172931

211623172931

211623172931

393561347063
21

202227223443

202227223443
112

202226213243

2

2

171334123419

171334123419

374263313193467524
3263

1111

1

111

1

1

1

1

11

11

321224258166410470
535534346881

1

38192073982
1

37191973880

37191973880
1

37181973880

112

211
573026184147

41222

41222

532922183844

296

294

294

1

1

1

1

31914153460
69305837108152

3114
65921118

11346

1

245158

1

1332182420

2

1

1010421512

9310102439

62931111
332357324937

88115610

3453107

3462
618813

314211

1
7543173

7543163

23201043

1

696763389563

262134286021
696763389563

22211

41211

41211

23155

23155

65275

65275

311123

4111

22

22

312112

312112

1311
14162

131

21

3952210
12314

14126

112

2

1520113711

21

21

523454244950

523454244950

857369
200185226147268268

463854317238
132183

1141191811

11

16181671510

16181671510

171325143113

638168549599
2162

132510113241

132418111630
34546

102013111224

1291182011

1

252327232015

302133195151

22131
302031185146

281831174845

1215

272331172239

272331172239

261733232232

261733232232

121

121

121

141030142018
1136414967173139

6136692510377
21152

151023101615
201333101920

531035

345273
11

344263

65432012
1281254635

63822623

24101882617

281024143340

281024143340
1139566

133431

1641252433

1082614174

1082614164

1082614164

1

2

2

2

2

1217412258123119

1

1

1

573673306562
411523

372835184441
191717102110

5412373

5412373

1

75421012

6212616

1

1673771918

1673771918

477712
643849285757

34132272623

34132272623

261820143032

261820143032

2421

2421

2421

2421

2421

312271273171394578

312271273171394578
1022061114

281714134055

281714134055

271714134055

271714134055

1

1256

1256
1

1156

69344332115228

69344332115228
1

22

12121122
67344332115225

55334132104203

251014212921

251014212920

251014212920

1

1

242029255882

242029255882

242029255882

1241541295510499
312178

82499

82499

112728152732
88126103416966

669658183021

527278

6110655

25252491916
11

2

25242491715

313224193273

313224193273

313224193273

1

1

1

1

1

311831104849
292417652939152542953667

1211520

1211520

1211520

1211520

1211520

116571511
112450374537113971024

573182269358

573182269358

573182269358

573182269358

1

1

1

1

563040308555

563040308555

1

1

563040298555

563040298555

563040298555

36162154321
9994366183081204900

1

1

1

8629482510168

8629482510168

8629482510168

8629482510168

8773915492781059809
1707110350178112

833954468679
2714894232

533436

15716171713
251018192019

1032236

635233

16718101516

422233

422233

34142894735

34142894735

34142894735

31816521787390312
372125222935

42182374825

42182374825

432635155046

37122994746

25161463435

432821135641

36192765428

55254397256

55254397256

8636512614484

219862614

6527432011870

653023178872
2244

33131134525

33131134525

301710143943

301710143943

121367343126115
2321483124

13682812

13682812

132132413

132132413

1761151516
2191472223

433277

11572714
31

8572713

401229213429

1

1

1

1

1

1

176812412163114328352574
412038196660

407339470273540471
231325113324

8052503810986

1

1

533124228153
13710122020

4542715
11453

311

211

311

41143
36191085418

143

2713674510

332112

22

272126162832
1513181089

643139

643139

53551411
64551714

11

1122

167121215139234189
17723184232

374239243734
6510875

202618121516

766189

766189

455374
1153

11

1221

14311

282438204528

282438204528
15615111415

643263

1131

713196209

12

33835333413

33835333413

33835333413

9285812

9285812

9285812

131525152421

131525152421

131525152421

21322
302347244449

4388128
111119101621

78112413

571481716
791581920

22124

10210676

10213122820

10213112820

10213112820

10213112820

1

1

366549263443

366549263443

346449263443
1

346449253443

21

918611847102109
161224112731

101426111619

101426111619

101426111619

1

1

52485

52485

1461271312

1461271312

1461271312

34432382122
465252183742

12446

12446

54611

54611

1

1

5513
16523

1

1

1

2131

1322

1322

222357

222357

521
52

1

413395448874
1320882165585122292043

381536202728

21

21

361536202628

361536202628

361536202628

271638283236

271638283236

271638283236

11
1569114478302264

1287511061239213
103921111

1

11

321820147653
1291481420

313459

178325724

1

361357

1

191528144649

11
311021145354

301021145254

1

181124123432

141161145

271634176251

11
271634176251

261634176151

1058727134268117801641
46184417110112

101538337193188
343188353171588530

13141041712

1

231194
4831157

1

25253

25253

16101183529

1571792020

19111372223

1341862220

13616131512

13616131512

1

21
12843138

12643128

736633

631143

10112122

1165

326164

12533183720

1441642135

11189119
7188106

4113

1

927575

81113103

201533125936

231531

1072081511

214148

101074117

1

151013866

171120455286295293
6444959254791052968

1

564399

549283
1

449283

18131573830
111123

17121463627

99621111

99621111

16182273446
11215

15182263231

11

871552414

871552414

3371388979

3371388979

857497

1141111714

1141111714

23141682717

23141682717

18161073226

18161073226

191622144142
71018121617

126422525

1
34445

34444

4572184

4572184

138126157

138126157

34442342336

34442342336

11581814
5347

655147

1011571516
59571214

211

1

21

1111

11

11

1

1

31462

1
14132032111

14122032111

3423473111890

3423473111890

5332612
5232511

11

1

15126

112395

112395

332239175330

1086920

1086920

128118110

127761216

1

12123715

12
13218573

2131

13198322

1

2332511

2

29172894333

29172894333

115
41015628

31015613

20201472222

3224

1

2

14910495

171320142930

171320142930

171320142930

81311

1

81211

1029817212015789

1029817212015789
4433

22
362959377029

362959376827

362959376827
2333

342959346524

6269109808460

6269109808460
992872221

121830121617

414251614622

7846114679090

7846114679090

7846114679090
1461916156

1081911159

1081911159

1081911159

301532192225

301532192225

301532192225

241744213850

241744213850

241744213850

12001305291216741179818

12001305291216741179818

42562361388388
12001305291216741179818

17216563126

17216563126

17216563126

17216563126

4863110483431

9102914115
4863110483431

131413487

3812954

3812954

19194016814

1913311
1712311

21

33321

163185544349136117
71015911199

912351579

912351579

912351579

912351579

616362649

1
616362649

616362549

7565113794836
29311

564890584428
1483520138

3311231

3311231

11

11149432

11149432

85952

1
641320109

64132099

69115105
6810584

2

11

1

1

642232

642232

1

1915141837

1915141837

1915141837

18234017712
1

18234017612
111

18233916512

4112920810

4102920810

4102920810

1

58282253

58282253

58282253

89301485

89301485

89301485

71121132011
2110373

3975124

151122
3975124

2464102

214514

191743261311

191743261311

191743261311

5310662

5310662

5310662

1

1

281379811453267212
3334104563212

1
16202914610

16202913610

16202913610

253341151712
1216308121

12

12159549

122
1222

2

117382884

3112
117382884

86372882

222879301813

1484516147

1484516147

820341446

820341446

3732

3732

4516935
161557412319

3461182

23362

23362

93321844
943218610

126

74151288

74151288

162249262717

162249262717

162249262717

202865321913
311

111117995

111117995

29242
212242

3

614362165

325767272118
1630458118

133442

2411

2411

464523
1113151345

1

1

2

61

111

113

211

11

1

211

11

1

1

1

2

1113

1113

2622

2622

8142418710
3557122855045

335481

335481

116114

116114

337632

337632

26422

26511

516402477

13612

13612

10414141422

7923

7923

31
314175481824

91113838

811281663

811281663

1219302269

1219302269

21134

173063361817
13

614182157

614182157

101642151310

101642151310

161661247224567

161661247224567

161661247224567

161661247224567

13717222110110071
11763

3853
263556393834

81427182625

81427182625

1617211599

211

738759221818
953010612

129342

638020984
587720843

53141

34111

3311

311

3

11

1514
354985334018

152325201811
192843242016

4518425

1

1521378152

6068141665862

1

1

172963252921

172963252921

423977412940

423776412940
423977412940

21

11

1

1

1

1

1

3728116584116

996533177

996533177

996533177

27195025239

27195025239

27195025239

27195025239

11

1

1

1

12010424616210883
13112

413878514737
36861

271428232727

111742221910
310331679

8796121

1

1411272828

1411272828

1411272828

6452140825738
11143823123

3245105

1

1672315

1672315

322994502923

12142817105

12142817105

201566331918

2252

252473381812

252471371612

242471371612

1

2

2

21

2220172
92111167876044

434390534326
41

232040232516
17143718145

66351111

202346301710

10182618117

105201263

476657171716

476657171716

476657171716

476657171716

16285839179

16285839179

16285839179

16285839179

258112012
48647084349438821912

13516016687363237

13516016687363237

13516016687363237

13516016687363237

5664171767359

5664171767359

5664171767359

15245835348
5664171767359

1112341168
213

911341165

161536152228
161852212334

316616

12
1410279109

141026989

776322414110259

776322414110259

776322414110259

776322414110259
182261432214

161742222511

8724161210

8724161210

132221597

132221597

221575453417
41

221575413416

294778574229

294778574229

294778574229

294778574229
1

294778564229

294778564229

18713119612232821516

2111212533
18713119612232821516

60467650755369

60467650755369

60467650755369

125841097024021114

10431212133
125841097024021114

265

265

63
11580106672177975

4

11580106672167972

1

1

51

51

1

1

1

16017452131214480

16017452131214480

95212183
16017452131214480

111
801042871587636

2133

2133

24
781032841577235

1113
781012801577235

771002801577132

71652131336041

71652131336041

71652131336041

71652131336041

943140634471738247390
2105

940138834211727247388

940138834211727247388
48102191901127

27311

2631

11

21

21

221
4315821666874116206

21

1131

2123

111

132

2

4275761657862115203

2

1

1

31
43851

42211

133

11

11

4557011546754118152
2

912352333

4466891509731115149
1414452884

4326751464703107145

1
1519112

1
5

4

4

1414112

341

341

11

11

14107

14107

22
32

1

218822304039236610421656
1929582757

1

1

1

1

1

4522
11

1

1

1

4312

1
4312

1

1

2311

2311

1

1

1

1

1

1

22

1
22

21

21

3112

111

111

211

1

1

1

1

11

11

3111

1

1

1

1

311

311

1

1

31

21

1

8788163972436

1

1

1

1

11
8788163962436

8788162962332

8788162962332

13

13

18122

18122

18122

1212

12
1212

12

62

62

15741489224213777781327
5649147953023

304361701428125125

5352138952013
304361701428125125

1822462886
1021242851745266

5354143922641

314896541819

423776411018

423776411018

608298603010
18272418104

1725302582

1725302582

25304417124

25304417124

4766104581318

4766104581318

5349143781718

5349143781718

5349143781718

5347142781717

1

1

2

2

9988819886085521059
433463502838

220105201181136548

41

41

14141910913

14141910913

14141910913

20290182170127535
11

20190182169127535

1

1

735742724377388473
143130167739585

11

111

2

112231

253

515557316247

1

242820121411
455644251820

13

992145

721391

414433

122

233573
311943261911

29164021128

1
1601311579397131

1601311579297131

29733824412791176

4561

16314926316854102

16314926316854102

28238864411
16314926316854102

11090103573482

11090103573482

25367247169

78241416
254320662397111158

951312111415465

941312091415464

941312091415464

931302071415464
12

86191211
921282071415464

72
24299063921

24298361921

609297664440

112

112

112

1

11

1

1

1

1

1

1

1

111

111

111

111

111

11

11

1

1

1

1

1

1

1

1

1511804272425687

211091
1511804272425687

1

1

1

1

1

6194184962139

6194184962139

6190183962036

6190183962036

6190183962036

2113

2113

2

2

2

88842321373547
168312

3729116641721

3729116641721

3729116641721
8517587

29249959914

1

1

1

5049107701724

67522

1

67521

67521

4442102681722

4442102681722

1011173801786056

1001153791775953
2110441

12

1

1

1

2

2

2

12

12

12

361013

1

1

1

321

321

321

12

2

2

1

1

2101

2

2

101

931083561715249
186

911053481655248

911053481655248

911043481655248

1

1

221

221

221

11

11

1

1

1

1

1

1

1

1

2
121113

12111

12111

12111

12111

1

1

1

1

1

24341853
72912921713935

222089471312

48671691062120
23352

264696651116

264696651116

2018703684

1
68862351142130

68852291102128

21

21

21

66852291102028

3

3

41
66852291102025

59752171091824
66812281102025

7611121

1641

1641

1641

1011093071993446

111

111

111

11

11

11

1001083051993445

981083051993345

981083051993345
1

981083051983345

2

2

1

1

15511

11

11

11

1

1

1

1

1

144

144

2613866113066477

1
2543845972966477

233112

233112

2

1
23311

2331

2503785912936374

11

11

2503785902926374

2503785902926374
199741

2493695812855973

23221

23221

23211

1

721410

132

132

132

62118

62118

52

6266

687850272345
11246108417435397840339171

111
175218311522629780793

12

12

710543

78443

21

21

154816601340540585390

1
154616561340539583387

2

2

2

2

13
151016351304499555352

151016331299499554352
1

150716281285499553350
1212

121

111

150516241280497550346

1

21

1

11

3101

26

11

31

32311

1

1111
11211

11

111

121

121

24142426821

368426

11
368426

1

1116

2134

11

23

2181622615

51468

51468

2121

1149734
11311

421

421

232

2122

2122

4211

1

1

1

111
312313

1

1

1

1112

11

1141
1

1

1

4

43222

11212

11212

11
1111

1

1

111

321

321

32

1

831013910
1

2121

2121
1

1

1

1

2

5271146

211

21

21

1

1

1

1

31141

3921

22

1
11

1

213

3

21

12

2

2

1

1
112133

112132
1

22

1211

19

19

11
1

1

23113

1
23113

11

21111

1

133025121018

1130158718
1112310

2

22

1

2

22

111

113

1

11

311

141

151

1

311

11

2111

2

221

22

22

11

11

1

1

174

62

112

877158
18413014972185381

1213184728
2

1

1

1
2126

225

1

212

212

1
11

1

3513
11

131

2

11

1

1241112
412103416

11

41

141221

1

1

11

1

1

2

2

1
119777044144281

115322
321

1

2

1

211

1
118756541142279

117736541141278
1121

4

1

114706236140277
1

114706236140276

11

1

12

12

121

1

21

211

211

211

211

211

2517335511
11

1

1

1

2

1

3

12

1

1

1

1

181331317

5

201420182353

2311

2311

2311
13

1

11

201418152252

1
201418152252

9121391622

9121391622

11255630
239

15
115

1

1
7243212

7243211

2114

2114

1
1824226

21

4

1322112

1

13

5371143
933188885750323731918293

998627

998627

582411

2
341411

1

1

2111

1211

241

131

11

416216

1
31425

1

1

14

142

1211

11

111

21151912913

21151912913
11

121
20141912913

1
346432

1

21

1

11

125221
132

11221

1

12

1

11

158136610
72212

2

3

1

13

13

1

11

1

1

1

111

1

121

1

3

1

11

21

121

11

28041914144210311156174
93321494

11111
263418161257943107259

258517781221922103321

414
258517781221922103321

151312
171312

2

11124

21

11

11
111

1

25811770121491810213

21

2413

2413

1312

11

1

22151252218

314

314

22

22

5

5

7

7

3112
9532411

11

321110

11

21
2

1

111

1
2123

111

1111

111

1
111

11

2

2

26

26

9123

1

211

11

71

2
241922151319

2313
1

12

211

1

5
911241

1

11
111

1

414

31423
1

1

332

12

3321
1

111

211
221

1

3678413
1351

1

1

2119

1

113

11
111

1

1

21

111

45722

45722
3562

112

804891504767
1

422747232541
11

311

211

1

13
847232

11

12311

6
61

1

2111

51121

51121

1
34242

2

1141

1

11
22

11

4271021
11

21672

12

111

10141724

11

10141623

3

3

412123
11814467

111

21

11

1

111

11

11
1

1

4

4

12

2

1422

111
1

11

36633

25613
433

21

211

112

382144272225
1

2572

2572

2421

2321
2421

1

6121

11

611

1211
23451

123

121

21

21

21

21

332233
221037141418

511

1

221
22

1

1126

11

13
3

1

321

1

231
23

1

11

11

1

112

11

11

411

21

1

129231
923

121

1

11

1

1

3

1

21

121
1211

1

21

32

32

814762242844

814762242844
144345

31111

211

111

3311
111

11

11

21
11

1

1321
14118237

2
1

1

26

31
6413

613

523
1

513

211

211

11
211

11

821517

821517

411

411

749131
31

11

321

11

16
6

1

111

1

21
858243

11

1

1

1

38111

11

2

22

1

223211

1

111

2311

33521

232

11

122

21
6644813

147
1157

11

1
2

1

11
21

1

111

1

13

111

111

12221
122

21

105711
1

111
321

21

425
415

1

6

1028222

21

211

61311

21211

129

129

129

745072393838
221

168103312

158103311
51

21111

11

15

1

4

611

8

42

11

1

11

7113119
231

35

5

12

4

11

1

1

1

1

541

1
541

111

1

32

56651

111

543

125

39264722158
4213

1

11

1

311

11

14513

1

7221

14411

6

221

31

1

9241

1122

2111

11

41

21

12

11

2211

1531

12

2111

11

3

16

1

12

235598

1315

1315

1315

111

31

11

5

111

111

122

136105248131216
5231

1

1

1

37

1

12590241111112

11

1

1

1

1

1

2

1

1

2

1

1

837211

837211

637211

3

3

1
227211

21

1411

1

21

11

11

2

2

1

1

1

1

1

1

443134261416

443134261416
1221

1113

1113

12

12

12

423030241113

4147
1915211835

374

43621

31

612

1
11

1

22

21

11

311

2

111

1

11
411

3

1

11

11

81
84111

31

1

1

1

11107477
93113

1

13422

1

11

211

1

11

15

6065129141515
1

914422

321

311

1

612412

17411

6211

112

231

21

21

32

32

2
4449120121014

312697492

2533
32743

111

111

11

1

28249062
111

1

27248942

12

12

12

3

3

3

121618719

223
121618719

11

211

122

21

1121

12

61

221

1

111

1231

21

111

11

2

1

351

241
351

1

1

62531
21

211

113

1

2

1

1

153112612

132111312

132111312

2113

2113

53832

1
53832

1232

31512

1
614966873763205319377999

22
608466143722202919057948

279255

2111
279255

14

14

12211
122111

1

1

1

1

322

21

607265923708201818857941
122

568713
4342

122

111

1

21

11

111

12

12

21

21

5221
7421

1

1

2

11513
1

13

1

113

1111

1111

605665793691200918787933
2241781119279103

350376735112116114

121

222715655

112

71111
511

211

1

21

383826171721

1

11211

13

11

11

114411364843952572518

15716481183040

408546262202134913625110

1

1211
11211

1

3251
335112

112

1

21242414314

81222101
2

412117
61222101

231

1

1

231551

2312

1

1

2212
1

22

2

14

1
211

21

585939202546
23521

36

36

36

221517111240

13211

13211

12423434
9112

1

1

1125

21

1

12

321

4

526512

4211

16411
1641

1

2411

2411

211
222162

2111

51

51

51

71523

71523

71521

2

2

12

1
12

1

1

415323

415323

21

4111

2

11

11

111

1

2325282

6341
4

13

3

111

151121

141111

11

22112

22112

261262

261262

261262

2

2

13114

1312

581113

55113
122

211

2211

31

31

31

2211191687

2211191687
2122

111

1

5211

11

1

11

312

1

1

1

11

321

111

1

1

21

4111

11

111

11

1

2321

3

1111

11

1

242564531917

579213
11

21

1

5

1

15

1

3

2313

23

4615889

3414578
29244

131

2

11

131

11

2

2

121311

111311

1131

11

1

79221344
1211

11

11

12511

334

1

1211

66

21

11

11

41

2322

2322

1

11

12

12

211052

211052
111

11

32

31

1

122

32232

32232

212

212

111

111

12

12

1

1

1312

1
1312

131

131

1

1

31626141010

5214535

35

132

216533

1431

1431

2438232

11121

11121

2327211

2327211

2
11433

11

1131

1

1

412532329915052882146418467645515333773697
79969637771207295970296082103269

313829182318

313829182318

313829182318

313829182318

1
16782121

135141

37317

516681

516681

1112

1112

362114

362114

3635

3635

385632

385632

8721155
3414109619368369651207

1

1

2

7121236

312123

13

2

1

51

1

3

141

131

12

11

11

1

30758931671681755875

1

1

1

1

1

1

1

1

8121112

1

2

1

1

2

11

11

112

111

1

1

7

41195

67

9

44

111

1

1

111

1

1

952812

111

1

1

111

26

1

13

11

4

1

2

2

123

1

1

13

1

141

1

11

1

221

1

1

1

1

1

1

3

11

1

1

2

1

1

1

1

6240403875170

1

134

1

11

139111

1

12

1

1

214213

2

21

1

111

2

11

11

1111

121

1

11

1

1

144

3222

1

1

1

1

1

2

1

34363

99

1

513332

41

1

1

1

11

1

1

2

11

1

7121937

1232

6136212

1

1

3

1

1

943

3

1213

1

1

1932813

3

8

45312172411

1

1

303330365134

303030314834

11
303030314834

11116
4112119

2513

12

21

1

12211
261828302933

1111
111

1

217433

113113

1421

11

312437

312437

233

233

22321

22321

2114

1
11

1

1113

1311

1311

311113

311113

11122
12124

1

1
2

1

433424

112

32344

34421
111

2

21321

514458
11

214437

22

353

1

343

2082253331595957

2082253331595957

1952253321595855
1

40512716127
12

542

51

41

31

31

523511
113910961

521
421

1

1424

221

1

1

1

111

1311
13

11

1112

1

1

8421

8421

1039423
112

51

521

12

811

315211

315211

111

111

1541733041434647
1

2086521
22111

61
81

2

8544

2111

1322716

1322716

28136

28136

251912266
1181572951363424

112

121

121

1111

9313

71

231

561132451251911
551072441211610

12142
1131

211

1

41

2212
1212

1

2162

111

1

112
12

1

2

11923241
1513121

1041012

131

13112

111

1211

755372903712329655171075
6560287878073591348570121188205216116

9064739754641254742

9064739754641254742
122

863746296087

863746296087
331

85738
32171473026

51117

11211

1352152

241231

1

212254

11122

12103

12103

513221

513221

491925151856
1633826

41211

11

132111

4421417

11

82

1

32113

22

11

21

7222412

321212

121

2

131

8194369294351192653
11

8024218934131081606

21390245102283179
8024218934131081606

14131373211

22201063512463

1251051910

12499127
12499117

1

19569123674540

122121

20123216227

97151107

23567

644399
18101362011

12693112

823434

43651541

1077122

49403719254

872382614

42317

22122620125

23113

271522205239
261021194831

151148

6103054022

145343

751058819

236164104
23415394

2111

343141

13115677

19925134239

16663115

12141

116147167

15926113119

11684148

1034151

61123126813

931531612
941531612

1

1714362211047

1714362211047

1714362211047
1714362210947

1

8485114

8485114

8485114

39349320603912888579298461913141778
383892211316046536565

1053129567512283

36651612

36651612

36651612

36651612

1050123507010671

1050123507010671

813
1050123507010671

98911737497357
97511637477157

14122

314991011
314991010

1

22149233

14696162112247181
11112

14191158107242168
352

271128183423

271128183423

271128183423
251028173423

211

465274

465274

1107412287196139
14231

525584

525484
525584

1

3153133
141820143719

111

25335

127267

434161

441437

213

1825355

1614355
1825355

211

65447358122100
627598

332236295946
332236296147

21

262030245245

5332122

5332122

3412398
12

1

11

6211

133154

1212

4434310
4444411

11

1

295355355247680702
124

513237

513237

280342341236663678
201624143326

553759329088

553759329088
4313126

1971672724

18142283334

141320141824

48855845101125
1231

132412212637

192929195150

15301752137

505661319271

505661319271

505659319071
505660319171

11

11

11

107148139114347368

59842728
107148139114347368

43425736120124

59977474200216

9121191213

9121191213

9121191213

9121191213

41217
183889883125117

612726182215

612726182215
3514

82233
11

11

11

2

6222

11

11

21432

1221

11211

1
26971185

1
1234564

1233564

13421

1

11

21

311

31

12

101

11

2
333111

13211

11

2312

2312
212

21

111

111

10652

2

112

61

11

233

111

111

331

2

311

11

11

11

11860706410395
1210

414111
734441396368

1164553

121

1131

131452

8

710

710

1

1

61213120
553532334853

3

2111

2211

1

11

211

111

4321

11

1

1

32159114021
122

3115794021

15222

12

7101

2132
2133

1

1222
112

21

32

32

111
1

1

1

451528222823
1

312

312

131433
18686129

11

1214

4251

11

11

71

1

24918141614
624322

1

1131

1

2

2111

12

13316

111
121

1

212

1211

1

1

311
3111

1

11

31213

22

321

1124

1124

17171291715

17171291715

17171291715

17171291715

17171291715

3549559633171324409110507061
38766320425812684778033453822139820

563329405256552477
155102195

201431112412

201431112412

201431112412

171176138

171176138

171176138

443277302207434413
121

733632166189

472718153118

2691413071

370241270190371323
1319410288153152

87221218

271621131624

1

635285

111
222151

112141

211

1

1

1148553

1291141517

2212822318

11

1279101015

11

324

31

112

676366

6155198

5211

153261

111

6667134

323251

11111

8913181010

1132

11

112

2112

11

622223144117

131

14

9512277

782187

11

2111

517454

682255306239

682255306239
533442

331129133119
331128133119

1

30823132718

21311

21311

21311

21311

21311

21311

443194239163342254

11131
443194239163342254

2849311283168137
11

2849311182168136
2789310878163134

63452

1

1

342550266942

342550266942

252729173318
884844367047

29107111314

3411882415

362732183227

362732183227

7783104

24
1

1

1

12

578364
1

11

13221

1321

211

1131

112

125970495654314461135

593543167937
125970495654314461135

77104109
353748206837

12121532611

131821122617

3216

11658855510781
514277393218536443

831031214
5293713

31151

17496129

3242314

991551619

513173

1125

13445

1313

3222

42024107

53111477

147101615

132153

9471115

937615

11

3192244

647578

10234421

56137510

245112013

111

12425

79113513

9651108

1

13752234

112

834155

242

31142

27352

12131

75641512

4211233

1

8832918

1248478

612472

98582010

32111

11226417

75132157

531376

41121

1

575575

84452

645275

310155223

333

445136

1517208122

114358

448442

11

75115103

21113

11

927266

826384

223241

121332

2211421

386210231148465336
442829156735

1

391220133737

1215251613

36121071414

1

71

161115124027
161316124627

216

421618153758

1

221616163719

1

292023153328

1011116209
1011105197

1112

27131482418

201424131318

39181484526

16142764923

247792611

292427212730
8331194

1015105115

742142

15

11313

3192131

24923122826

1148101515

1351521311

5117676

5117676

21122071621

21122071621

1512147228

1512147228

341425173728

341425173728
341424173728

1

23112493221

23112493221

3061882446

3061882446

238185379

228165379
238185379

12

8513101212

8513101212

81355821
534342395675

261828232830

1333
1233

1

1812981721

1038861846523443775444651

129591105913369
1038861846523443775444651

1591848126168415251074
996859396224424371314360

1

633364

2791501411151352

42113

2121

16956295

3111

1

13341515

21757112
2175792

2

19102162214
18101861913

3

1

1

3

7710537241828

1

129861420

11131

123

111

636847

424121

321024153814

13823117
13843127

21

213

843221

848934

21112

11311

922171

417

21

3374116

14

55523

75232

61422

8321111

2213

1

212

224443
225443

1

553486

1

114114912

221

217314

623142

963153

725297

3711

12142

13221
518241

4152

412153

1122630

3121212

11111

211

27727132026

212121

1

21461

31

223113

11

322253

13

11104296

357115171211

355123

11

92103362
914139

162323

1

61121

712393

511111

211171

222

38162
36162

2

1

1044911

63326

3214

43467

21064109

21121

1

2122

114

211

2312

21121

1112

32

225241

336999

331

181412111223

2226

411517184723
623668

351314124115

7519917

12112
12322

21

23442

43444

109441014

811

2

171515938

19813
4

1977

12

16287

42235

34

3512542

1111

665934203036

514

665233153035

211

436541

95388

21143

112

377772

1141

12943254

3122

15844105

712521

436214

12278538

21

141

215114

12112

82618

1211

171281492

354211

31664

1521011213

2411

11111

447353

23148

3427133

2111

11

4211
42111

1

11

2463144
6473144

41

657378

311113

1111518

736131

111

20812162312

115112

15191991321

1

1

11

41212

111

2222

756232

12312

1

11

1254135

661111

112

555545

555545

72261

43361

1

191244

3123

1436232

211

1421

85111

11

372423

6221247

11

7184718

31122

22345

13612681

3223

113411

41143

74111032

111312

111

41

258679

21541

4654115

51381511

13

113

1

73163

19

42751512124

11

11

1811

264265

11111

111

81263104

11

4461133

44431110

13101031211

9113535

23

68847

741312

559535

11

5105672

131

1922

2710764120

1

123

122

1019574

6113222

8512255
539255

323

411112

874215

104576

333

358362

21110

57221

57221

3

231

543

2414148114

1

59144

5

13131

1

3121

821141

242112

2112

111

24113111

1123265

85124107
1162091816

24157

114432

1421

19211272329

2111

3122

312144

545561

11

11

3232

13361155

11

1

453342

1317352

141241

11

1

936347

33111

1

9581163

52231
1598362

1096131

111

1

1013121

722121

4810483

21331

511243

1

104731414
84531213

2221

111

115332

1

32346

31332

3113630

564342

3113
1

3112

2212

614272

539259

256

6289214

1412177536

1

31321

594118284121

913

751271812

1

141

11123649

785

637373

21112

2722411

324253

2125

28197789

11524735

1744315

535142

216121

21

11

11

4255610

111

1

822425

513232

2

1

31047

1

116991022

512122

11

51186

2915

1155251

634535

21122

25

213324

2110126911

11
11120

1119

5393

1122

21

44224

241

2223

122

1111

1033493

815574

1112114

553026236819

522227

2218

112

4393

33

3116

847289
115741010

31221

525333

124911510

2

1

756453

746287

31421

7617476
7717497

121

10381032

112

763432

21

531291524
431171424

1121

22
321113

121111

21214

4251

315552

2

4282

111

12312

95462

215231

3447

11242

531243

603435233730

12553
1553

11

53459

525153

121

851133

111

14
117

13

3116148888

111310283

1

2

111421

1522

3331

2

102474

12532

111

351652

472311

712143

2174

795193

512233213515

1

492130193511
31142011167

8366131

1044263

2324

411

1

1711741514

1

2

3121
1791072012

148872011

53171

5

1

2133

423263

21

316451

3

4212

211

51733

222171

1

181827213324
182027213324

2

114123

2415783

49313922186

11

11212

15284125

311

1

21169151417
222110171517

15121

1

5862112

1

5245513

1222

71123

11

11495103

3

114

154139

1

1

18126709

3145222

211

374443

137510117

2164151

515236

11

851914141417

11211284364951

114411

3121

31311

1322

281410131216
221478612

63564

31

621355

7111

5

314263

34274

6103684

13922
15922

2

2

11

161248157

1

2

2778532

111

4362

2

2221

476693

925445

367273

322121

314363

1

23114

311

339621

31411

646438282418

11

595142

158144117

21

6231913

11

102118107

482530256632

482530256632

342272

12

423238

16532117

111

642048273936

434

615558

2

3

415528

1

1

1215223

1115223

1

326612

326612

21

1

11

1
161211696

1

14129695

21

2341771412

2341771412
2241771412

1

1

25

25

1

42248

322

57531013

1331

106194

311

111

212334

11

223542

13

1413773116
231112

1210672014

433728142829

121

342229234767

11

61

121

25491

413585

12

843332

186333

12453

94421

179181075

4423

872176

147113

53312556

112322

724351

41112

13113

681192417

2

6212544
7213654

1111

221172

553435

617454

337541

1529884

2112

1

315223

1
2

1

1

234

1211

5413

211365

432213

3638197815341288169317

1

2

1121

33131

613413

8812776

1571617139

1

131232

61133514

32631

2176566

3312813

18192613128

1849322

1111

211

1

8132103

1111

814121

2710128118

14112

2

1

11321

71111

14322

1213613

12

314411

744950468131

652111

583022292318

411227

31424

303115531120

656658

1210461119

11123

824379

231117883

1

111

74442

11

1

2115

111353

1

223431

47233

42461

115114269

637357

12133

1272

923587

11

135135

1326233

54351

12121

1073385

1

362
152

21

325617

821136

5116136

41323

2411487

127

533277

614117

1113

354

21211

118144

62511

14143

222210

83103611

333173

631585

94197122
5211592

42823

111

32

583343

311819161112

1

61

11221

638313

5122

981631615

1

1

1654247

13134

342522152047

1227577

112

314194

1

1

315344

4121

5461710

215522

211321
314341

132

1433

1231

143103
1144103

11

11555211

22

1111

2

2421

11252

412

1

8386176
57612

331164

1

767443316025

1024247

442118

52123

312172

2111161

16863149

3644611

1112631124

1

1

1654537

62223

3516153

422144

1

27541

1

1

1

13523

317151

7712464

1566122

536431

212521

31312

626841181420

17534

311

11

11

11

114355

639144

523271
513251

12

21112

2211

1266213

544487

2691

1251061012

21211

3417

12

42154

321

343020122617

254421111423

211

6484611

111

322

17111610690159161
14131113214

4141

1541271113

21

1

5953627

86631218

11241

1

162

33111

18344515

1

911641217

31221

528535

1111453

402323153816

1

4482218

8211

81758147

1769101711
635664

11344117

118115103
11769814110859

811881313

124126145

26583127

414322

1211123149

731211

1851152310

155125177

433242

3124132

3124132

28418817990290165
211

28218817890289165
532134

12124

12124

2318592113
12

221658219
2217582111

12

1

1

11
523151

2111

32113

2111
564362

2411

1

12232

11

504526204622
23715116275235138

3

1

1421

1

11

2

233

1421

311

4
41

1

81

44713

211
212

1

2544

23101

1

1

131

1212

212

111

22112

52521

12
1

11

727433
725432

1

2

2

5512814
421614

13112

271
251

2

1

162182

21

31227

131

13242
3323610

113

113

215
12115

11

311
411

1

3221

236173

11111

1111

11

231

2215

141

2

11271
3081172714

11

198871913

124121

1131

221

2141
4141

2

2

221
22

1

122

13

584313
423313

161

55214

4

2111

44132432247
56163592453

11311626

1

2

1

1

311

132

132

32131

32131

2142

1131

111

188310073212166930671259

188310073212166930671259
251119495237436179

1119023912619083
5764011069592995402

927721611621579

5933107436922

271348323616

502755365525

31941224220

282045234823

5843101628140

301857376830

321562318121

221954263919

363744387124
333641377021

313113

5762927983481017387
84401165814458

392365336618
412365346921

2133

171226152215

7913632014333

251123192614

321129114222

1592614217

3073011268

7323112814

189257258

12142572412

10417685

92391425125785

181237173313

17162512269

361044223619

213223122711

1162791811

112132124222

395147699420492228
1442718127

148391306611736

6222703510337

964532223315379

5225102458152

231248232617

85481517212763
153

191835274319

461673264416

191443144025

865170469063

865170469063

63239

63239

617535
623354367050

313383

10678106

119136910

16761143

7394119

64781312

322122

18151471113

11

11

17151471112
17151361112

11

109183146

109183146

109183146

109183106
109183146

4

348339298172463301

348339298172463301

348339298172463301
5852155

17722214199249194
17621614199241192

1682

483644337029

118731083812973

822552463459841464
1

803237379823

803237379823

1064777

4092019597

413245

2616109284

741520426422743441
1811115114

7484132

1

7384132

23812
451047

12124

1

111
11

1

139961415
6341712

11

665563

710731414
159

276272
277272

1

43123
4323

1

1

15516515

15516515

622428349376621369
12887572910641

111311

1

33123

55121

859746

412221

71197128

121

35

35

11169

1710102124

99165412
1110186413

21211

11

21

2

1

3311

11

213263

7331

1

13171

11
11111

111

112

63222

22
12

1

461111

62751

1717108118

11

1223

1111

83222

633251

111
11

1

232222
333222

11

354133

51169176376

51169176376

241079306
523282

17846224

21

111

2

2

1373443
1143422

1

232

1

166124112214251124
111111

164123110213250123

11

451622
34622

111

282142

32111

412

412

555224

331353
111223

2213

1

1

267135148
297135168

32

2411

1374373

142212
122211

21

1

11

22112

716331

1

6562113

1124

41

18104643

545121

1231

1224
1221

3

1

23

11
161257128

713111
12115798

5102687

312

18131510163
11

66537
766572

1122

333231
433251

12

73624

21231113011
1

1

57211

12148295

324

11

565709296125324210
144761909411341512992585995

1
854838401202388317

2415176439

2415176439

821811371186338301
220197105437582

1312
1412

1

574121
574122

1

2411

31414291

878292
9882102

111

3524

26

857522
857523

1

864986
8641186

2

916424

1461051312
1361051311

1

1

3411

97552

631114

2329624

13131210735
12121210535

112

52112

34111
12111

22

1122

1011216

11

13117183
131181114

131

992365

3232
434121

1111

1

19219246162035
19919747182036

75121

189134244
189124234

11

11

31211

1665232

1662265
1762265

1

11913179
1110131810

111

21
31

1

151163107

69732412
610732512

11

832

2111

1

1

11

664358
674358

1

1065715

442121

555141

10016653413523

314111

15105144
1595144

1

911131077

911131077

8175108548268

8175108548268
173147173624

995399

157161088

314353

866221

644634

7552102

9810338

7411869

362328304655217936472490

4125143
14211491015

627411

4724510
4724511

1

685557497351921727
360928094641217036372475

36612714107

3

1311

1483342

111

52119

211

1531

894246

213522

2

2

294632453411

1113

11111

142121

16586124
11

15576113

1

1

21

51864421

613

1

1

1

7854153
422930194526

314124

41131

51112

121

5232

1111

10711372

12311

21121

11
111

1

623153

2113

211131

62414

35531012

1

124113186837169
129119193937671

1121

12134

435511

21193614

142112

92813122968

12231

9185578

1

201817122424

3214

3721
37311

11

1233

346134

1

11121

11

445246

61353

114

8632613

4413

104113710

1

3533

111

33111

44282
34282

1

22432

22

34371

211

21

64212

11

14156543

1122

11111

3531813

122

583438262913

23119115615115899
1074473

2111

5111
511

1

21
2

1

14

16815186957667
19116311010810582

2

1

11
3112

2

2

1

1
14919112514

1

2243141

1171581013

1

13

3111

11

21

1

111

1122

591417114
25252

11

42

2481552

22115

11

1

16881145
15881145

1

45112

34

1252

847025061020

743143

41244

111

5433

364162

131122

22259

6352119

1

744225

13

3232

3651

211

1111

772479
281624232924

1

1141615109

10564116

11

5352103

115963014

467176

111922112225
111922112224

1

313311

21

2214

211

1

1

58

362115

886294

12123

231111

53124

13

121

4875410

510225615

31212

51117

1131441831

435142

121111

161126

21

1

2213

1

1234

32

61126

2161

12223
324123

2

21

121252115

10109156

10739108

10739108

2111

14112

6121
6111

1

211

21

1

5764137
5762117

2

2

8687110

5112167

1111

11106544

4112

11

3337103

11137

12

32754

413223

769891

22132

142

1121

1

741031612

410421015

542177

2

3142310

1

1114363

221713111410

11

11

32292

43637

1111

14

11

321

829768

1213

7524413

121

1

352314

5322

323113

221127232314

73182

55454

1

2111

12417111

1

1

1133

119124510

52141

1

1

433534

131

16422211

541223

131224

26132181819

323223

112131

12145

31913141423

933323

374222

141

11449108
10339106

1112

551146

63124

313

6636102

23134

344231

613327

10191913129

677593

1

229105201

1

1111

2456

310691126

143

169104187
16962187

42

456238

44241

2

1029557
562330433230640253

527313408217622227

527313408217622227
154911564827693

1

111

1475781

11

111

641218166533
621017146533

2212

1

2

1

29320222414427098

11

22

121

433131

755134
765134

1

1142524

31510

54522

456315
457316

11

31554

638463888599468206
1078625

21

21

583428854575405175
619449875589454196

1

1112

1912432911

111

111

2

1

1

1

1

9485133

9485133

2

1

11

4

12

1

12

1131

1

11

2

21
211

1

22161
12

1

1

115
114

1

223
32421

1121

23132
2321

1

11

541555

2111

835398

11

262521162127

331221

935969

144

11524

31313

11
211

2

1

11

79601206272624

1162
1262

1

1

1

11

617587

12104443

2

12

11

1
4525217122120

3382

12221

22

4131

513

3219166620914
3219156620914

1

33111

4232

1411942215

12

827414
1027444

13

1

242122

21

1121

224

222

101711674

567688

462564

1

242143

111
1161

15

11

3294321

433310161216
12851095683408663433

1397174

21

1

2

21

875144
976154

111

388384169136156126
11621041646381533360

211

181235122
181435122

2

1

1

1982532

2441

723463

1

21

23191112294
13946142

101076152

20321

2112

10166134

2212

1

25522

14

74442

104

1111

562545

7951112

38211

1

66121

392820102545
392921102645

111

7142232

955413
755413

2

103231

522

12

9114264

11

79311
65311

14

3212
3112

1

7184242

321

12

1

1

1

411

913131

4124132

9311

45453

3315

111

392235

1

12

24131

21

11123711

6111

23935113
2273593

122

65332
65311

21

171619121413

112

445262

1

49411388198
47401358174

2324

1

151
351

2

1

69595

4211

1242
11242

1

9657101

19144791
19144691

1

41

9143272

21

1

434122
435122

1

51171

1072497

5

413

2331

1510533

67443

324

4431

21

66412

1

41555

11

721

1111462
910362

211

33111

12

1

752232

189132

1

311

29328239
27328139

21

42111

1

367321

44132

11

1112

247311

12131

494141

15722

33221

3435

124321

1

511439
511329

11

385271

11

2

962482

273411

71421

211

1

1131

32121

12136231
12135231

1

828152
1028252

21

1

31

812592

1

1

11
1211

11

1

12

164473
64473

1

1785974
43113

1354844

61

1

1

1

21212

206732

22242

13106486
12106485

11

11

1

9125

6712201011153

6712201011153

1
386984932054391180481

385284552047388162473

385284552047388162473

173773188

173773188

554514393176170185

403368292131107131
554514393176170185

1825136107

14315586

11104145

1

9613653

65114

222811218

3611

24152314174

1

864271

36401781015
33401781013

32

14319285317054

2940891421
14319285317054

1499665

7111952486

789175
788175

1

963393

107372514

33111

14116125

14116125
3211

64332
51312

132

3525
55382

2132

394632155019
348843372660156337221752

671419468338732551
12580925411881

67105192

133443

11175921

33132

551662

107107711

12

82432

187147105

12

615561

5315753

12212

6

11465125

1368549

161611986
16161110106

12

544642

41113

1111

7288958

225342

364541

123533

12231

184851319

102314102236

71122

5583625

186771

41231

52531618
52521618

1

2231

945258

873296

444563

724233

459212

484483

12243143

745113

2213

451119

733223

112

115

631010145

651113

432553

4131089

1

41088133

61110674

17111431013

557365

62227

26342

2059788

2

723354

525633

441431

334655

1025493

85455

23232

874264

1232164
103254

211

2612

114

133761010
1871082914

5432194

113
14674127

1457397

12222

632359

82225

179442617

4332

6111

16411

111

55232

12565101

432662

92103912

445242

2

14569136

1410159167

1410159167

1618306515397991863653
276438622145120129241175

361116

6725101

143

25141272612

3121

1

131

2131

212

544023165523

1121

13211

524511

131

7126211

11

1619338249

422444

2

11131

211211
1121

21

2318159367

62665

327352

213243

625451

2636

410322

1111

915122

11812763

31152

723101

31212

13473157

11461

1114

54211011

12211

124364113

2

27

11

23122

11411

1

28191818408

2111

1111

14847104

223

223

21

514231

41124

1

1181681810

1

502966477833
492965477631

1122

1176959
12107989

1313

4222

1

121

26246203

203134812

23221

21

2812116883
2712106883

11

17121971810
17121961710

11

1

91118393

532

27232

1

1

1

710851913

114

211

512532

2

11121

1

7847151
8969174

12221

12

94437

1211

1211

1668438

24

1225534

322122

474253
485263

11

1

41431

11

232192

11185302520054

2212129125
2212139126

1

1

31612

2811

718292

2332

38141

1

18910486
181111696

2121

11

21312

11

1021610218
3213373358131

111

1

2211202336121

1

111

1113

6571

111

351414493310
401715093512

111311

41231

71

21

35211

132

3211142

2112

141

2561316278

544313

211

512457304325
1

231226122913

231226122913
121222

644485

166216196

281230181412

281230181412

63111313

229195139

6664163

6664163

6664162
6664163

1

292861713336900454946407462109605
6611212816461067121692124

856181
3149715182307132

2215158384
16153805518385

13274413

15145174

17241174

3157

93236

12244910

241342

53343

64101193

823663

8361125

934499

16272116

9554185

724947

246146279
14539652611646

12253154

22575512

5131101

856284

1033183

4312

244111

6274163

48108292

415157

313156169138226138
91221

827195

827195

553420222921
288145155133210130

1057276

166106141

255791720

554374

2199965

198871510

2115118116
2015117116

11

15479182

899766

1362911

203125155

579813

1871371914

2116861611

11316122114

533471

764322
231

11

22
341

121

2112

11

12113

12113

18955344
617291248147770229

34141973412

34141973412

4414189407

4414189407
4413189397

11

571538348631
19413143011

26815113812

123109188

1

1

4419228624

4419228624

10148613216265
5181142

27101071918

15101733012

522725218732
542726219933

21121

3181728552352106
874522169430

15979392518340
16982402518942

4

63162

432513

12211111

11144372

4721109

416

345324

241493285

121
803449399238

3161217318

3161217318

27826174717

27826174717

22191131313

22191131313

409224392922158731752377
304189244156236272

35310830242939
1422531333201283260

119282311149

1896226133024

7615791628

126273292726

956092647343

91311911218

97291581819

1871104316739
20312950161046

1619737

734239364518

10369564089178
363020193597

6122

5211216

362420173251

655166

73564

743264

11486109
271157395171289181

484396537639

212110291112203133

271420162628

271420162628

348332225153308387
138157695388202

84731514

453326112825

6743430

9914374

385322264042

373514162220

10104164

572465379846

372431177235
323242190104367228

323326144032
644034197840

32785388

28132193327

34302062624
433828133326

988772

202221103224

565527174237
796537187445

2310101328

524018184531
443516173528

8521103

323242344015
1411171438313865

778671

363339153624

2319107167

8614273

251726163011

1034324

1511842031

1511842031

625680557539
633467737498614348

243178349218256139
231171341214244137

12784122

8359996010158
88611066010858

5277

652955545232
612751535032

42412

1119197766748

645248325630
645250355632

232

42223
143969444112161

502729143964
502728143963

11

493327182736
513529182737

2221

38323394457
37323394356

111

13

13

12545372648143

12545372648143

4015185249

4015185249

423328223351

423328223351

149274334
1551213946458835

36232510259

105893425053022

471224217013

471224217013
21361

143810306

318168346

1
592364298842

281126136636

281124135535
281126136536

2101

1

1

31123716226

31123716226

12574156488
11987912415809352781961

9814237344031541553
2077719697337138

33271672813

4216321510520
4216321510721

21

241511115326

22575259

511726248525

4819401410116

3492093313
3592093413

11

2951994416

682343215416

264933910

208101132

1614177157

11356184

411722118811
411924118911

221

2142284613

20101244214

1682093111

211515125732

1151391212

411624114740

315124

3322811376
2922811326

45

3191862624
3191862411

213

541191

21111552215

6256917513552
70561027915253

8114171

2

1314861

31171212639

31171212639

81546917597183471169
107138548752478975343

2842194031617

567456501710922

32432324912619

138463341266253116

11
7842492511433

563129117317
543125107016

24131

221119144115

221119144115

1155

1155

582017113510

582017113510

62871090234055431489215
79625151571893833277146203784

381713105518

381713105518

501503816
539927028891550158016

48462172224476150011

503526151036645
47349595983565

30320538

165413102

165413102

179994084297133262504630
5824412237114572023587432515

23151431

59071431983571574131

9621151

24962592

33717191722

21691054

58133323309

175446372597

74111525176

562193915

872212498191

231225193121

1851314184

3183018185

2811116242

1

3371614156

53162525334

331971133

1

17534673

692645364314

195125153

48182423269

39421220159

113341

43131320265

224411011538529
21641961488529

855

2

62411027171

32272233115

833711945539244

307101965

3

411

203131445

8322121

381718191810

419901228211447

3611440301

1823772

2731017291

36102716164

276975739182

21

11

20412846151925

2281320136

3371617182

34141615155

24109101088

17619151712

2810238

10429112

7982381181

4391031488713550

2891515113

361941388313622

321213195310

5222

192114183

1

36102420156

311112

411

12

30779103

18722611542

6931579241

27123326117

237141281

57922281513
52820271513

5121

30102512104

11

134621124616115556

9427

6681261389216430

1155131352711

963338353416

7511214162

391610305216

29173125397

27121618172

4311221288012719

391924352211

962651142

311417102

83171

82314

3055975

78202636519

11

11552811103

55173031418

30121918195

41331

1

29181825148

16692173

18294038271337

92067810968274

93427230619137699

93204536417

21

198811164

24228364718

355141696

1

593202018

42512128

1

18661451

541534512

246918157

1181813104

25635151

201371341285410

7497415215773927

1283046424812

414513102

121

207610104

107102944122

164714162

1136912

1151840453712

8598316595632410930

304122461

11

1

53212729269

1

953811

4844374

4505138320976322335713

1

9231561

1847642
1685283857220

43112120162

3091916206

32111815155

45171828175

1434094544920
21212423

486309103

1354895

12610772

244201283

778341

1810101193

201061096311541062147
2808892136619301579267

321012171713

1223775

10327136

1

116265666724

56823333914

221082164

806268360197

411671

673232485211

33121613287

17911111813
15910111813

21

1594965911077

1092963496811

10541063

3313

53172827415

1441010149

72594020614
1358657384937

10234

2

121221
35135112717

1

3410492516

3241
664232

33132

1

1

1

7

1

1

4

1

11

1

3213
5213

2

423

39154161186

39154161186

17897132

17897132

813946395013
3412421

41231417226

37122018266

45311

45311

17447679013531

72212189223

102264682438

20831383

20831383

510443157328012640
6036253822320

37497342380286

14792084233
153102187243

61131

46373271

1

4211292

415295193

424734276177

46364316

37112333315

12

36112331315

372956359153444
1

15791652
11671432

41222

356885443148442

285712155

285712155
1445764

1412591

191091031
2237512914410547

31102920142

43183532296

64122531231

3069211429

31191929228
36192230228

531

259117816227970
893825131224

10344222524434
934220252032

1112192

9115

1

3611152

1

112

51251920157

111

1

31

1

113

1

711

29618608

29618608

660812237514616340921249128054

660812237514616340921249128054
1211641422711648409605419

17276763241754

1

1

3021392562744953

29121493339

39181052310222

174100872951667

259585766212699351471
2770925697133104461547

1756835751176

504431695210659249815952520841

361111104212

310130137260167122

310130137260167122
11234401316634

28109311612

411412
414

112

4

113

191053511

11129449

3414

1535714
1645714

11

115314

1

1454891

20674116

886434

410493

724962

1057943
957941

12

1

1

42111
4211

1

31323

13714310

111161

834621

42124

52245

412

5431522

1122

572655355024

615456
572655355024

352135203213

1641511135

79264524097222249
251612415

71662511613
17016565263241

1111

2021136115

441

19118347

55621761016
54621751016

11

714430172119

714430172119

13713425166344
5765891725

46231

4222

5651124713

161031345

1197433104659

1197433104659

27021275245981
30303257

1239639132134

117863393340

4058658209450875413470
12149518465279041369815983471147

447838354136566325716803626
27406519791603425495321456

1861472013

2

792130145714

42814115915

71

3691592511

7828373418123

2231111

341314610810

1211

772123157528

12846483420547

15838713414921

15838713414921

852339158024

679881726655632

612133

33101364613

732945567127

15117622314236

1

2096785
271177187

721102

1

192106204

18339886327476

35512182

45162410418

3156071332823

219104177

531117144411

2

161255192

26949575034143

11521261

716211

67922225916

702438205454

1119

15684426

6133111

11

521023284010

511120162314

21601733212283045131

1384317

12432484911518

10573752221974880209

2

1

258853185115017

1122

17512264

15358176389287

841156

5314236289

1272247

16641213

11683266

741532246023

53412177

5411

144385814214027

4313106339

38477689

51244

19046815833933

311427527

23232815142520

1222952276243

30967898140630

25543282

13111

121614619518382940

10174242

902035175829

43142286213

1653242

4

11556292019532

149511624097219

1323821497

1392778257836

15966223

1152043329836

14147281417536

3681071106275096

236371

12348252

321610133612

541819104712

425

601618227819

321289289

23121282634

3731246270374121747

912

127601075620280

2411179319
12673206

125106113

21

16223143

1981841235222

9721385

16271183

47202584114

5743

32111684312

15443168

11

24554945734234

11042634111141

615138126272477

45111

1552743193424

181092164

3913743111131

2

4

15441183

236422811

1371330198626

12

60341783316

15614102

3183139

11031532911742
10831532711642

221

1324152

1343354
1332344

111

44884298

14361203

19046594239752

2511140192210

44131783315

44131783315

5671915114215
212634223071651039303

74169563918791

19955334236115810197

458993115

458993115

511142194129473180
932738228632

3661175310

37112052217

471466319
491567319

211

381415154216

3841194110

251211112115

491023264332

32111153612

411415133910

3391974513

409142144

768249323212580242
285811267619372

376811428

125115113

171255184

7795159

973836197037

914129

1111222

35131416289

812141

91211

65221

10482112

2112521

7142122

1051343

51193

34241882911

281418123217

1

1344562

113332

1441

216145222

13599161

21251

923225

49915183213

391522203315
341222182312

33121

2182

972335208831

16563376

21

751827153921

422114

532

532

8223261411042

8223261411042

732731243961473545
10991161937242511758

2125631579

50691894720

899131631212

125634913

86811765218

717131621911

224781915

1625142545

1813105235

1
1813105235

2
6753202

5221132

1537

1264233

3512

914221

16801256145822326391255
10298839901381600730

2281253012

4019394160104

653846411038

552554514968

252222511959

25167761711839

1

9413413728247143

9960821610361

31

821842268343

821842268343

541257
176397545482166

56112514241104

391211132013

7616351719642

44771434110772624031325
54321695134190727939436

67303119131014

26775603661540

26289473361726

5461781154

121

265537673112718

4083121098

3

14860837118350
1

10411394

676565

11040535514728

17696176

524247

8316381269414991426875
147495518476301634640127

88556144

4110646260

118208279509

11516

21111113219

50233547011907953

152575630327614

1

455262251435

5129208138265

13

3514177117339

229152626839802995

12336146

12336146

360619647293100
13143784421871348392

812025115925
771924115223

21161
1

2161

211

211

1992517812263

315121

441111204827

661923167930
631422156923

3511107

5791284917

1

1

30141471315

24886167

25016529164110

682023122724

3791251145436

23167613
2611171314

3571

2110671715

47537101158

2901792138725980
602643204315

141133262

1857643484

1

925745

2113157175

1

2771310144

15744

31141962520

2711128243

9332143

1449272

131067104

31211411239

681425164828

681425164828

6338618121726361
222

5225814719720430

47202492415
52202692516

5211

578893215

197291673218513231131211768
359403417375826912156018141

887524639248113

22342710

49221152715785

7031743824

4232039169071

967422233

3761763239

1041138613567
93934412563

11242104

3471511228549

62297768456661538411

13

657293566177107

523542334

154725223324

3

5114

21

449523036

17531181695297

319242634220180

993507047918443

10921088166429174

65432116

13520121681204

2622124167069

3511021188989

2912817239594

20424122216181

796374345229122

9135143

3281724137943

747326523547873107

32316138

5926239103274215

1122

1

1451211114841

494841884

1
471728338830

32915216422

1581212248

71910422412522011368
37344113491347829

183522819

3951078456

184332710
184332810

1

296875344

190245341499290
217407857566333

271625166643
271625166743

1

7112511

18167066

633222
10728302510641

511213145717

239332918

274116184

1183245275617

1183245275617

652546383417
26084205182300106

32132428208

4119221514937

1642015138

2833527208

185712135
195712135

1

196199219
1961910229

11

40932372914

11
492011172112

251079106

231048106

39129669169659

39129669169659

953238355827

953238355827

748145
199149139698471

513834162714

463630162413
513834162714

52431

452231
646734322619

27281416105
25181315105

21011

333418141313

412137111114

412137111114

179146912

179146912

191012377

191012377

1828212256316100

1
10555894919873

112131075

101388338

13485297

30172383113

251028117511

159972329

4317

237
4317

21

2861026812

2861026812

45182254315

45182254315

433552293123

433552293123

433552293123
191712895

747561

1122

31941

4624

1421

434844

368215

23143

10166915114882

10166915114882

211120143926
10166915114882

211519133314

787267

341929144824

56105175

1376356

19161199164896921201660

745461439965
19161199164896921201660

21218161423

21218161423

704449335640
87117137

714233

52223

10156281

41564

466225

105117101

12256811

1063245

10241100145652

10241100145652

22112063210
334173

146105163

52694

783667558348
407254333214445351

43413

423421

26112212

1

112254

321710112049

321

665365
965365

3

166136188

952282

1134384
412172

722212

5233

5353106

3123147

43174

74861023

221323121526

13788287

11897165

6515262

323298

745323

1

1

1412108287

144186158

211

148451

132

835363

3633

222241

7335411

8688136

41224

11885923
11996923

1

11

12242

561141310

1775186

66191567

12473

776564
776574

1

1211631415

11104184

6128464

211133
211132

1

955472347856
2551441815

21243

613243

1211

2

2

11511

111

432126
432136

1

1054247
1165247

111

53342

41231

535777

312233

10512692
1059592

31

11915365
172417386

615221

13161311248
22222

4736132

798394

1012173911

1012173911

181616172112
972564

565684

439674

1417761027
1

91765108

11119

3

191021142411
9150854511971

32319

12101531310

561061214

12811101917

1011161711

3012145258
3013145258

1

333347345659
1123310

161718152521

161527162828

481332448247526420
14259118629691

1

6741109

315515

21172

222

221117152032

81387149

54345

28101292214

11

11

17918122316

242832164620

18141371313

1

301129153125

192329133421

11

1

12

13

12122361513

201020102524

94911311

8181191815

213

7311410

27171982220
27151972019

2121

324150214628

12311

371423174937

1

92467960130112
629655

1

1

1581142551
15101352751

2212

10611131312

611911425

262101915

5235116

15520111112

9838126

15777132

15777132

11

11

12
3723311813616

857791

102111104

1915131011511

563260286639
26162412379

442311

117175136

1

8410765

615818

211

483634385246
64121

21912172923

1

111458128

1091613913

1731381918

1731381918

10565728
311115192360

9247825

644461
54446

11

61326

664151345248
22113

1

17798166

1971281121

618255

171094157

51511246

1
613240184869

13141671921
13141671821

1

471824112948

331
423231153645

1882081718

11655818

131532109

1111
331211262149356269

3121
3241

1

1

1

2116

2116

1

1

214

1

326209259143350267
141312

13274248

132

411648145724

26464205

81010111417

291822144714

76513818

52132

311232152229

1

923871284957

934379

5394227

1

84105615

1044112

1

338587

101145202

431165

5223112

211

5571357

1271172511

112

2

11

10906968205461098809
88651917

714424
269182220173274213

302119222412

302119222412

166164811
192148168127217164

6965910

1810962310

8516231622

1

12211771

7256815

73991914

211013131817

10524127

9963107

91297124

7119455

991681111

246119156

1

9987411

71056155

5266382

74103176

11

1

1

3191491217

3191491217

9314111816
5113111210

1

1

2136
32166

1113

513334392227509389
271223143022

491010258

491010258

502130183135
14797118

415116

2213

234318

2346749

74441

433135
433646304241

102102613

10776126

131817796

12

66913129

11577116
463435275956

745627

2091591927

26421615

61043111

31212494622
20415016084203152

1

1613189146

1121

31

364919326

13915598

189971313

1351161214

10824102916

159971713

146361313

1052215

76126111

19714133334

251591415

251591415

198512413

198512413

121410381

121410381

833564398346
22121241814

35371

1992315227

17346

22612435
22512435

1

2

2

163763314

300172202141296190
10435118

12684164

12584154
12684164

11

4413332

4413332

243131155111226157
632342265352

454361

1

5311

41611

12746711

622295

514152

12810967

5131

32143

79109118

323233

1151010167

477251

56595

1

12355101

1131
11131

1

211

1136495

1132

434254

1042713

451143

1914173117

6255107

7411102

53317

1242323

61342

212

9610386

9610386

222113153213
23126

412246

581618
582618

1

1198547

251984239614059392975805506
966274208834

204234051510642234221533257
103832085151399514691525

2914168197

2914168197

312227111210

312227111210

12297874312186
474738194427

312525112816

22

323218

11

1875677

11

4514

118

412231

12612397

211211

480169316195446238
21111

411143256159355199
391139254152346191

2042798

672559369038

201919143521

201919143521

471723226311

461723216311
471723226311

11

1132316210316
49767524876225126

11

91144

11113164

52551

871251111

386216334

61051

16672693317653
16673693317753

1

1

1

1

141318554

1

15

6411

34133

1

348219316

4522

1

18352253

141213697
131013695

122

21261213

1

1

581

51511

111

61021

5831

11

3103

381219152015

381219152015

834058386254

834058386254

623137294119
554247

2813209238
2812199238

11

1775762

12681182

473437132411

443336122211
473437132411

31112

111413538
485235

65522

11311

83256741417
82691836943897644101017

389518596
318617344

34152

351

11

497636716
93159609214

6921

1911

686

315715216

234448321185194133351
5927118092911515320859

51311

101343

23467215

3923

7813144112026

12271031

1318411

1417411

162051

342

1626212

223213213

17541313

365316342

2540731

5721

38912128

5741

718512

1123

2

1213412

111

1020541

161771

316712216

357619628

938413

4113
21

2103

25421226

1220321

6185411

1431614

254510111

1936831

51621

2

23461123

22

582

8145

71421

211

7183

4121

816121

9731

11
112163

11

81443

152

1628522

231

466900223382454

26489913

1838921

5110116939

396618614

31032

5861237309482472

18451123

235313334

315795

33667615025947

516411

24421126

90416764286938135

92173517417

11

97207566114

711511

16351233

92463

29399413

9175311

2029713

1419715

1411

41223

5092297309

275213523

241

8143554

20165707124920367121
21206036132321673127

104329741366

884473575117
304151214158203109

241020161410

287218206

443032231516

30101914269

251115121211

652191

29181982431
322121112831

33234

232141

2510915247

4132

4132

95571158210665
467518183343156753362213

281721145112

281721145112

215435310802271901548
419481612231371

7268025338532132

376153407120563193

6337225947493152

2313772619

2313772619

764549189035
14893198

2214842110

30172092812

106122225

227149275

227149275

25101992919

25101992919

9763704112076
1681351811

242923164120

411518102319

1

1

161116103825
151115103725

111

422741186719
245156267155381149

119138214

112

221418141910
211414131910

141

11122

247171673

7112

33182992213

13768137

15818103519

12

2723814112421

121219122314

383022144437

449274437251655359
1452882125173620221058

552129235518
542028235418

1111

103601025915676

965375

221216102710

11

341915132112

221328173214

351834235631

14312

353227174632

341920143512

302624154622

522256278239

10256803511449

6849523315071

4837432610851

6142113

231221112012

1

45442

543542185436

181218103218

1

221311121614

385563

21122219299

41132875527

1

897995

11311

31

1

2122121

1

7848543710661

341140203125

1016722521

151614141614

1

1

39143053111

39143053111

419201420264552216
442155336016

703168337831

754256398843

1839320413728999

471437223727

298174224135364295
16171662126

1

3

2111

9510676

2273410

32213

26273618318

1314

132532

1

713482131

111

423126

32542

111

414212

62181611954

433181

131

1

12121682638

53371010

1422

11271310

3111

3124

113

2

815168173

1971031118

123

2

111

725467

232259

1371082137

2612911

949463

54342

52397

633463

12633

31165

3112

111

11251

19411518

11741
28519219683244186

71414655

71414655

71414655

71414655

71414655

23516615569214164
452320102853

333236

7

11232

147631214

1152

1

2940275811

1417563

2

814113

31

112

1

1

725222

121

1

3311

1214511

1

9280582913353

11

1

7310532

42112042417

42112042417

42112042417

42112042417

42112042417

33765131617111084
5

3216082991519976
22

1
210406211553749

9405356

21113
9405356

2

1

11

12

1

1

11

21

1

21

2

2

11

2911

1312
1

1212

21

12

74311
1

1
211

1

1

1

4421

41

1

3

11

201359202483142

544129211

196355161192931

7714548483121
341

1

1

7714243473121

11

1

1

11

11

11

1221
7413642463018

111

1

11

111

1

2721

211

211

211

3

2382161

1

21

3

13

1

31

1

1

51411

41

2

21

1

2101178

2101178

1

1

1

465

111

1

482

1
111

11

11

211

1

11

154
21

133

1

411

122013415

22211

813821

4411

12

2

211112
1

1

1

1

2101

433252

1

1

1

1

22

11
1

1

1

1

1

1

1

1

16

24113

41

1

23

34553848316

34553848316

1
19133312

331

131

11

1

632

1

1

1

1

721

1954327
15423545304

1

1

11
32

21

3111

3111

3

1

2

33
111626223

111323223

1

1

1

1

3151935
31

1115

21224

121

1323161183
24431

1111

3141

1

211

27

11

1

111

1

3411

1

331

25007057110122246977173671394967942
60025898383230575042433

771698417619828

771698417619828

277114
771698417619828

1321

1321

1

1

39322

39322

121

121

12114

12114

111
313546

4211
4431

22

31

2613

2

2

12482154803
2311

10421853762

21131

211

11

11

1

1

25

25

2110

2110

361242
222

112

322

1

216545323

216545323
1

116545323

264783
3104984

1421

166152
111

111

2

21

312
3112

1

1

11

9231041253

9231041253

132

11111

320639211

411

11

11

2111

2111

122283

1

1

121283

121283

3416111

15

1261

2215

251
46121

11

1

2

1

12311

111

1121
1

112

331

331

222

222

922

922

11

11

452673

452673

452673

452673

452673

7879431123224

2

2

723431
7879411123224

24

1

25

2

11

1

41131

1230

1

251

2142

1

321

11

12

1133

32111

4111

255321

11

7244

232311

52

103111

602

9112155

112

53241

2951

231

121

17
363622222411

21

1

2121

2

11

21

1

111

1

211

242

1

1

1121

1

351

4112

2

111

3112

11

421

31

1

21212

111

1

1

1

21622

3

12

1

11

14121

31351

15923386187159168
12112

171771055

4621

4621

713712

113611

611

14

1

13

42

42

2133
1

1

133

3121
321

1

1
14121478176154161

610321

4432

261

4103122

231

231

273121

14212

1311

510138
12415759111108130

111

111

1575516

1575516
1454416

11

12

74321

3431

411

11
11111

11

1

314421
2221

3
1

2

221

161
151

1

1

5451

5451

577228858576
1428329205

3312119
96141523

632144

1

11

21

221

1231

11864329

23

212

854113

4121

1

12411

117441441
103231341

14211

1

1

496545
31441316177

34

1

4

1

1

1

61

8910
1

1

6910

3

1

1

41

11

21

874111

321

1

2

63713624329

2512

2311

21

12121

12121

22159

1

211

239

11115
3289593619

2279583514

2
325387224464335141

3216122699

3216122699

22241

22241

85332

85332

1
14321416

13151

9162
862

1

1

433

88574
513

1312

2

1

1

1

14322

1811331091189074

895637373735
17313

62233
1443545

62122

1

212

2265683
523820202015

5

22810861

411

111

111

1

1

211

11

1

1

1

313

11

12

1

1

1413
11

1312

211

211

5823
5113

11

6

12

12

741115

741115

5337

3

4

2333

423324
111

11

214

1221

423322
927772815339

4310536

31233

128233

2111

2111

77231
212199104

3212
3112

1

516661

61131
41011

212

11

11

15133

15133

141141

141141

1

1

191174

191164
191174

1

2341981

2341981
21

34198

423442251920
3793312

1137

11
12162011

2152011

2812221

2812221

444

511

1

11023810332023658

3312
11023810332023658

111

111

321

321

1

1

11

11

11

63111
1

511

13

13

6

6

511807629716825
331466226013016

1

121

1

1

4331

4331

1

6212215

4

4

11

1

151
191

4

12

11
11433215

1

11233214

411

111

764223
112

2311

121

111

211

222
2221

1

11

25221
394521215326

11

1

2

223416154315

2

11

11

1

4

1

1

813

1214

13322

19798519017796253637206979729169
143287148

19778118997296135636096968029082
71656811320623102614187

42114227195
1675216454781949705497383

3211

1

2211

111

111

1

1

29417211
61

2232211

1

15

38751910427
412

319

81311

11111

1

71

11

11

2272157316
216812525

1432111

33141
21

1

1

1241

311

3

11

2432

2432

544036132113
111614442

1

211

1

8211

11

221

211

21

2

1

1

121

211

111

112

112331

11

11

1

1

111

2111

11

321

1

24322
34322

1

115

1

15

11211

1111

1

111447917
1114

2

112
1112

1

11

121

112

21

4611613
22114

24159

111

1

211
266732

112

1

46511

362313

121113

2412

922

922

23332
9552911

2

1

12

2118

11

1

1

5

1

1375413
971313

42

21

322111

32211
322111

1

122

122

11331151

53211

4911

2113

321

321

2

1

1

266352182999481

266352182999481

1112

1112

1297959473755
71047311

211

22111

14112
15112

1

211

91122

11

4253317

6322

521

1131

1

1111

9312

534

111

11

1

12

3

3

10611

3111

11

241

33311

6151
6121

3

11221

31

413

1

162212

11111

14711

311

41211

131

111

111

437

11

11

13

141

3

1

112

1

32

224

272

422116
24191010618

21

12
1

2

821

1

211

211
101337510

2

1

222216

12

2

111

52

2111

321

1

234
10131012413

4821
49211

11

1

1

232123

232123

10
11110

111

25

1

1

126311

74311

52
5

2

311

311

43243

43243

20116639
6738354413367

3224

1

46413

11111

1

12747322
31

9747321

521

2217

1219343
1722

212123

18334129919
11212

11

17332117817

11
111

1

2387112

1

245102

121

122

1

1558732
159791538173234595521420

31651

1

11111

3221

21

1

321

21

11

1

4761

21

611

24

1111

2211

131

1211
1111

1

412

572121
211

3722

1311

28432
18332

11

7512

15884153207275455551893

1

1

107332

53122

2121

1132

1113

4342

135235443
542

113

2451
451

2

5239

1

14311

14311

21

21

12

12

4114

4114

2499414

17312

62721

141211

1321

1321

2221

2221

13211

13211

899762414248

391027152128

247133106
21112

112

1411

678262

122

15314121122
31312

17464
37466

22

9365414
6355414

31

256713111113

256713111113
1441

2564861012

2111

211
1716151075

45222

45222

135

212

2

2

52

11

11

131

214

214

141

141

21

1

31

31

847532

23433

23433

61322

61322

11
919831213

919731113
32121

611

1

2

1

361255

1

113

251125

1111

32

323

11

8336

8336

2311831301079853
8223

131

131

3212

11

2211

353332241711
107132

631

277

21

31

894101

7

2111

6316716

1

12

124

11

1321

1

132

81112

81112

1

1

1

12

12

102

102

426553
5681819117

3111

11

21222
1121

121

11

2

1

392552

392552

41

2412

12

11

3312

3312

3111
697354503920

11

1

12

311

122

34321

312

12

121

121

623121

31414

111

410111

211

322

11

52

22

1121

231

541132

142

710122

1

1

12111

112

228322

11

3

21

22482

444

452181

512511

611

2

1

3431
36561713219

1

4221

43123

3
2547149176

2547119176

214

214
4

21

15594714889476245498935494627432
101811851

42

42

21

2
21

1

3131

3131

212

212

259341187203825

1579862
259341187203825

244334178123223

2413144313

841

169134313

2422

2411

11

4111283
5123

2355

1111

1

2

14

312172064
11

145612
146622

11

321221

311

11121

3111

1142

113

111711341
311

115

2111

1612

311

123

211

122

631416

62314
621314

1

112

38123
27121

1

111

12211

12211

22565127

22565127

12564927

12

101521
171

81

1

81

15561314841475977497715484927367

15560014839975970497475480927356
15561214841075976497685484827362

129521396

1

2

141315

9147282221286

9145282221276

9145282221276
33412

9142252181264

21

21

6141246251
29233224326324077

32

32

3111

3111

816154304120
94163122

1

1

11

72112

1

31271311108
11

13

13143315

411

1131

1

23331

1

542

111

1

311

2111

1

111

10106533

776112

541

41

22261
221

224

321

533221

11114

11

11

321311

321311

3

3

3221

1

2221

12165281
21311216219

11

211

1

21

111

1

3511033
71241295

1

1

34252

211

11

111

12

1

24155785
221

312

2121

2

613

1311

5112

4

11

1

1111

9

121111

121111

102612
11

1

41

12

211

11

211

512372

14

111

111

1221

11121
29121

18

632614910

14512

1122

3121

3

21

37

1221

18846

7415521

1

741552

83113

83113

111

111

631558410410117
9471

211

23
21

2

2212

5
4934287691

4934282691

7511
7531

2

2413

3422

10159122

33311

1121
1

121

1541

1211

410371

7

15115

523422

132221
22221

11

413522

413522

531

531

11
673534

1

111

1233

14211

22

411211

411211

211

211

111

111

2313

231

12

1321212
911212

1

1

11

1

12

12

6111

6111

1

1

41271

41271

111

111

131

131

4141

4141

6133554
1695716654816655685815667

292619112810

4121

1

252417102810

63131

63131

41122

41122

11

11

43164

43164

636221

636221

15111
131

11

1

1

132212

132212

11242

11242

2

2

222227

222227

20132994

20132994

29521

29521

1141

1141

311101032
1541

321

103621

441311

131211

311

311

36131

36131

1137

1137

5011012

5011012

342

342

63311

3211

11

11

212

2312

2312

2
7112

7

1

1

1821

1821

1678216445806554705722612
9312357313143

91112101

22213

12

232491335

1143311

1

2052

342

123

11

1

91

8512131

35211

122

71432235

24411

11

1113

2

594112

2421

111

1

1

11

333111

1

8105411

572521

131

12

31111

3381

39411

8312743491634

4521138

213

2

21112

1

1

222

124343

21111

21
51121

3121

22761028

34121

131

32111

53144391567210
541935123213514

1481175541

1481175541

193

1

1

1

752313

12141

17347

1

211

31

1118113

1413

621311

342211

4927

2111

101861081

213615
23615

1

23

27342
27242

1

21251

13

32112

3411

1

4398737

1

11

1212

311

23421

37623

13212

131

3

1726331

520

793970518363249204

11

15

3621

31

211

2521

13611

41211

12

1

121

1253

1

11110663586045
521233

1

12

352926213522

111

1

111

121

111

11

2111

1121

1

1

21

1

1111

11

2

221

1

15

3132

11

1

11

11

1

1

1

1

1511

112

1

112

11

332

21

1

2

11

611

26

6311

1

211

311

112

21

136275

142

1

361

1111

311

1

1

1111

32131

2

1211

13

46123

1

22132

2522
252121

1

1

11

1111

133

41

149341427270014421493930

112

313123

34112

33916799556250

11

111

31

3613

2442

652

17425

1

1

115141

21

3635719
3634719

1

612

1

42

531

421

121

21

2

4111

421

5131

911222

83410

144111

11

2712

432
212

2

2

2

2

21341

111

1124

85422

85422

191215485

71121

1322

5211

2322

596211
5106211

1

12

12

71357131
3213

124

142

14110

122

11

71

71
61

1

5112

5112

372311

1
372311

121

412

111

11

77421

77421

77421

6195767
183202146157204133

425831275625
711734

81532

31

13217

311

211

16532
16522

1

11

11

21213

514

1

2711

711132414

11

121

241713

1114

711

711

116

116

3611
3971

26

1

237581

12437
237581

113211

824541
434435553630

21

354

21

2233
1233

1

11

5

321531

1

212

212

22361
12351

11

221

1

2211

7511

111

12

1212
4621865

451763

122611

1312

442

5461221

13231

1311
311

1

143

143

11

11

1332

1332

11

11

111269

111269
15269

6

14171213185
1

11

35314

44642

121231

1411

4127

23121

12324415
382924175429

1

191

251722124914

822234

42123

41211

16131114827
945421

11

111

142

14118

542728

34152

34152

34152

722111

722111

722111

91223

91223
122

22

71

13431013129

10187964

63531

49511
411621

211

41223
41222

1

1

1331

1331

224212

1211
224212

122111

1114

1114

133
9510355455453

13

13

6
19424103

8214

1112

131

312

212
211

1

18151
647240383044

12112

11

45325
45925

6

212

55511

3

112

5
15

1

31

11

112

4121

221

21

11

412
162318111223

1

38312

14

1211

1

4

12341

1212

1

213

22

2213

241

1217

11

2

11

21
15191248

13191148

34

321

31

14

14

21

2
21

1

2

2

917102142

917102142
1122

9169122

2105647

2105647

2105647

3
11711365688248

706343465942
82321

111
985313

381311

531

11

11

46310

46310

1213942215

123

123

23111

23111

109641814
1

78511011

111
11

1

1132

1151

421

421

71013154

71013154
7913154

1

1

4442

4442

1115521
161361097

1

1

1

1311566

1

1422
1

21

4
41

1

3331101
56619101

11

12218

1

37515

23415
2

3414

1

141

141

5212

5212

5212

12

12

36421518166

1

1

1

1

510433
22111

1

28211

11

1

9454

9454

76127
1828611114

2

1171

3131
391

4

111

3

1

11
211

2

24

1

11

141

312

312

555535302023

1221
211410468

1311
31313

1

31

12311

12311

1422

1422

1
941112

211

91111

52

52

1111

1111

344125261415
1

4104523
314020231413

31111

13161

15199766

43211

1

131

21211

1321
2321

1

1

11311

11311

1221

1221

73546

73546

112

31434

1

1

31

1125212

1125212

1
1125212

9111

114211

260118145017342852345
4540437411712192370399263835537965

1171155664877543818446033118
11010077951865283

112
546961504833

221

221

32121151

125251
128251

3

249

18282419159
54413

1251

1112

21

55514

3152

111

2

11

11

11

14352

1

4

1211

11212

111

112
11

2

303521151918
35361

1242

412

2222

111

77823

1
11

1

1219107412
568634

1
7132118

7122118

2211
221592

1

12

1

27

1

115139172
2203941391508221127

24228

24228

11225

11225

15111
638156614

52884312

412

1611

25296932916
13120074134213481

24232

18112

76115487710048
757943745443

13653465

3711

4182

93

221

1564

1032452
1032422

3

381219
381218

1

3422

311312

3821

111

162973

1341341

912111

912111

151114

151114

2221

2221

1
2154712

21126

41112

15421

15421

35322
25

1

121

1
11

1

1

24731986463
181761651

32221

1264

39860362

1

1

2232

815311

815311

11311
23178853

1112

132

2

6211

2323

1324

1211

1421

1421

6

6

6811

6811

426225173115
31

313112
20176467

2612

11

11

522

1121

134111

9291510165
393543

413

112

3112

3224
33126

112

4211

2121

31

14

151131

151131

1
3111

311

413
44113

3

11
1

1

241

241

3111

3111

1741214
11301690100412951102460

2318158843
33141

112

1

722131

722131
2121

5221

31

131

413

213
21

3

11351

21

90132777211331
1087166498412751080417

1

1212

491111

111241
1632001041327345

524
523

1

1

1571891001286845

1111

35

611

121

263814134817

132

2

11

2

1

33
331

1

121
12

1

6142

61

111

1

122

1

18132

123221

111118
111117

1

14312

11

7411

12

44126132
2441102

253

26

423
43512

1212

133

1

112

1114

24

41

2

1

1

12
2

1

31

291131
99918142821

7901714179

2

1121

41

17

11121

1

1881112008876147
1801001518876143

811494

22216

22111

21

121

1123
1121

2

1

1

1521

1

41

411

1

1

1521

1

211

171

21

221
121

1

54121

1

1111

12193634
12193632

2

22

12

31

1311

1

221

693972
673252

272

1222

921

2

121

9797100464831
969554324031

1

1

1244148

1153

2

3131
311

21

21311

121

211

11
1

1

223414

111
112

1

213
2713

7

2
21

1

121

93111

135

11

11

1

212

4322
43223

3

26537515312312214
28141817317314515

11

1640205022

21

2

11

12455234
2761813289

152128055

1

1

1211

111231
122242

111

111

1

2125

11

121
321

2

1

23221

633

5391092436

211

1

53221

41111

151

211

111
121

1

2

1

1

31

1212

221

2

1

193129
2951210

121

11

1

1

1

416241
416141

1

1

1053882

12

11

11
1

1

1

212
312

1

312

2

3

1

121

3222

21
2

1

4

13

31

111

1

2552

11

111

11

161

12953
1295

3

11531

211

4311

111
11

1

341
331

1

25586164
2865142

472121

1

2101292361747

2

41

1

1211
21

11

12322

1

11

1911

1911

21

21

21

25231968187
841413891221101817954831562

241221

241221

784

784

409727648218615896827426
83741348119799817851331360

212111312

1149
11410

1

1128

251235

11

3617

202665

21825215
22

1825015

71113

1311164

111

33152

1212
121

2

12176613

9615078180812371
75814140341276

1

1

1

4

71

283

3229589311

422482371

11

21

2

1

1

1

2

1

18129571

1

1

1

1

1

2

21307

11115161

3522

1141521

325181412237

11

1151

119425

563134

322111710

3112

1123

13191
13181

1

1428151

218026

5141

31

535

141161

2221635

353626

1

11

11

2231156619

232122

41124

1591969

13274

1

21062278
21062268

1

41664

121

421408

3041052121901714
2601052104541439

809

710917

11

4917

321339218

514814

153163818
156375439124

321543536

21411162

551124

4

6924900

1123

1111

125810

257336495286871184
2773545293161282250

1

1

62

1

1

1

1

52

1

51

111

3

1

1

11

16929211

1

1

1

1

1

2

151131350

1296

21

1

3

1

4

1

2

2

2

1

2

1

23

1

2

2

219311413

311248
311278

3

321131

11

1852734

4263

1

14813121

232132
232111

21

2342816
2322216

26

21

152119

96

31

911463056
1011466563

1357

2117814

2701164

1111158

111

1111

3322

41464382
142

722111
72241

4

3

331211

216

7136995
314221

11

221521

1

15242

51

11475

11475

172

172

17411860234914551645638
182118

26429816025623787
16961796230714051355629

354

1011112
910112

1

1

81

211

1

11

1

1

6234

23

1

21

21

16

2

32
31

1

2

1

72662119829

64571861526
69622093827

53233
5523231

221

34262
24262

1

1

1

15

1

2

1412
1437

25

525461

2133972412
2122872410

112

1

1

111

232736591
242736591

1

26

11
1

1

1

111

32

73221730
4221179946668

176165923828

1

1812111110

1

311

2

1

1

12

111

1

1133

13

11

122

35133

1

2114
3993531

1961111

212

1

23212

11

1

457129116331
567934176933

111

24132

1

84325

511

1

13

2

1

18121
28223

1

12

1

1

41

1

1383311

1

1057141

11

163672
485424166218

851
3

241

31

1

4751112
48302195015

43161553112

43161553112

171381

4

132111

13111153
1310143

1

1

1

1

111

1

111

21138
21118

2

2

334311
63431183

3173

2

1

3

9939831443915772313
9899381431851715308

1

1

31174

1

1

3273831

11

1

113258

119

342896146

1112

11

437116
547117

1

1

1

1

11

2

473

11121
21126

2

13

14
13

1

111

311

11
6514

453

2

385140482689
1315132

792161

1311

11

1

14

222013132256
243116352366

23

103208

21
2

1

312112
312111

1

2

236

1222

1222

1222

334323673681165966968184510414502
929693318889024627292

1441187118

4213323
7226684

122

11

13

1131

57823

57823

31
2124111

272

21111

1

1

1

937667628167

674440364957
15665910

1111

233

12241

32128

11321

122

311

234611
234411

2

111
11

1

331221

243141

3611

64162

1173231
1174231

1

11

1

11232

147109524

18201513152
22

94726

475352

57364

1
595965

33231

462734

126261
3374113

1112

2132

226836031718172112821529
481124

8261016211
31151

5317

4618

410438

2131

17212

17212

433
4631

11

2

220327
548155503

211

11

1215

12482272

22

7113353
284897128

31

3111

612212

11

711

2

6112

2331

21

552

555119235730316687
221834541678168911861511

2311

2311

1

101573

1

27314

3

132

11

1

1

2522

773353

12348448
13358448

1
11

1

161

311

3860504158

311

204535346
194233345

21

1111

11

79423

311

3111
13121

11

23
22

1

2132
2232

1

1

1211

2

11

1

10336516879773
762924348612

1

1

277276431161

23452

2311
2111

2

1322

55

16

69542
68542

1

1

2

224

11

581

1

27331

1

10511118

21211

41652324174
36592120144

21

1

1

1

1

11

32

1

1

2

11

1

52211
52111

1

1

21313815

10591557940876605319
123517199981142813554

111

168743680108229
16256315695227

1

1

11

2

1332412

4

1

2111

2111

1

1

1

1

1

1

4832018289

1

11314

2271

17137311
486038171515

314731141414

211

2131

541313

1

244621556
266122657

212111

1

1

1

1

31

1

2212214

5

5

1

7553743

1411

1

711

2111

1

63

63

3026110724135812740231371
11790112745630133

2161841

81721

2812

14311
513984

1

11

11413

1

3615

213221

213221

2941

2941

2128626254
671541

21

33111

311

129415102

449

292471
5244891

41

3111

3511

32

223
414101

111

2161

692451

692451

1825115131926
1611

362

44813826

813103

4104356

23422

15222

15222

5412068695620
83331751

11

5310

210222

1044418

1

261

345213

23733

21

312212

1124

221111

6319

111

1142311

862142

2214

24

4841091

16311

823316141
7112

171

4113

6214

1214

12111

6924264823013734
1977710352821

11312

431

71481010

106131

234263

113395

8

1

1392

9

6921

501341

3125321
3125221

1

95312
94612

7

116

20133

9

17414166

71601951

923915113323

4895196

163

3583721

246253

933634122
42

741112

921

1913

175287

8122146

8122146

41131

41131

551521560349
830142

51141161

4841

212562

214374

5211681

2417

1153112

11125

286

233457493503573727211069
26413213041390679153

3323571

1

514917

263111

165412632

929366182

1

516124

356223

1325228122
132111332

41159

1815634175

77111

2143121

3803231

125418284

9231

1

1

1711

1972

447

4211231

1

1

6193321

1

21313

2163303

1102952

2

5211

185325

121

114122

9514614

112214

45121

12242

1

241

311133

12518223714110675
12241092

302183236

217
1

116

1

174329

19116
716

121

4471474324
282723

1

132754261
128749251

451

174134
73124

111

11

132159

111

6775129524145
6369127372445

1621515
1

1621514

32

32

1

4534727

1

1

74433

52221

3

262

112

1

2141113

2122531

621351

1202321

222

451631

314610

313441

7521

5234

1152

53323

1513

1111

672323

1

872641

816712141

1

415610131
417

4116961

31121

2

959112922571562604371
5312456607949

110123

1

2431

87681559311238

13395326211619

6381839283

33444231

5555861470597181150
5455591468580175149

1

2

121

2

1

2

2

5

1

1

22

1201

15

3

2211

1

511111101319541
4971106907541

7

132

1

2
21

1

11

3321313

4231

152

710131352

5947117243756

1312

4154811

31671698

1

581523

5291382

510111
5101111

1

644135

68274

626344

1311551

2144761

8111

3711153463

11

1274

1274

31525

11

13

13587

2721

102591777

91

1131115

272581

7211

3

1

8154165

5326315

21

2441

4

11

271

71622

3921

2451

41041091

830164107

3151391

5212

82

34

111

23915924

49151

5412

712

1195921

2211

12

1018183

2

26113
413

221

24520

1

1311

1

52122

55143

215115121

13725

11

141112

2174

3

31371461

1202434

515227614

1421

3133231

3265743

3162631

541

4111

1218

1

17344

111121
11121

1

6262

160151
160251

1

561

21331971

293411

12

482751

122

1

118143

1

2291

4413512

121

2

1

111

12

2

1

7921

15246

4164

31

111

412

8745512

13363118

115

315321

225123

111

211254

718164

62

611

14232

23121

2

21121

7232

91

811

172121

1

2332

2

111063414

2

171121

716846107

6322

1112

1014

5373

3101242
2101242

1

17292

51151
51161

1

1

3114

32

1331

2915

14410

3461059

6513

214754

91432

134321

11

1231

118

2

35435

1153311

112

244621442515
244619442515

2

1

1

23173581

821

111

1382431

442221

3822

233

37221

1224

42

516

132104

306028530716
306028530736

2

11351664

16651

1

342213

11134

49251

42964420711628

51128

153442

1933

1

642

28121

101365

217516139

11

15221

62352

22112

1

14762365

1

112231

11

111

5

971951
1381951

41

110452

1

1

192

3113251

11132

42131

5367

97416

22222

13

1

643

1

412133

1815262

241138851

214371

110112

532192

15215

173824

3

32212

15341

32132

1

992

411

51481611

32

452422

231

41232

4491

892

461231

46173

1634416149

31132

178122

110142

4123

1114631

112422

12131152

9172151

1

2133

281221

323133

2

1211

64121341

21634834

1422108

1321

11195842
10193732

1211

12114927

172

2221
436781

221

212561

312046151
1742511

521

711

97212

417924156
461736220410421

111

2652014966

2

1116

194817524

223

1

1

1

21

2

3

1

6122

1

22

1

311

1

14186297
2

4

4
1413457

149457

14

1

1

1

11

18122

51211

51211

13111943

13111943

262632

262632

1752

1752

466541
11

22432

24221

226828127942
1613341

563319

11

161

1

22

6235
62199

32

132

2

122

111

363311

121

28106358

51782413
1

1103203

114

351410

31664517

1

811

2

12

3751316

1431
197013562711

35341

31425

4612

24163
21143

32

5411

4964183

12111

1211223

4124733

133131

1

3962

4142851
281002440309

519131

77132

521622

3116212

823

14116

12

211

520111

1

113

441

24141

1412

1153

1153

118332

118332

38152

38152

218715352211
571942

3141511

11

413431

271332
21021052

3172

314213

210212

1256

1171223

24241

24241

17114

17114

7
33467131

31646101

11213

18107316104

191441

1312311

23263

283322

2111

2111

65612141
1

13211

4

2111

2232

2276

8716

8716

2116921683531
2197645

32

31

293

11

210344

5281

45

2431221

17232

2511

219142

1431

21013310

1811

12131

17151

61515

61515

181264284
62743

652

1

655722

224631

224631

4206562
1

315513

141432

22111
135283904012

191136
191336

2

610113

3103382

13238121

181125

107325132816

2523
107325132816

86825130786

1610633166833
5221

119221

4511

210311

1671915877

211243

245331854188912004746661605
2453213969342329

3831362
589035876417

173121

1

1

5645

111

911619112

57569
57568

1

2

1121

11

4455

562191

10841811

37112

11887103

9851
9831

2

15111

16810838

33414

416313

971111

23967158518620473046022495
261030615667137939299160

84151

7231441

531241

5234

8293457

2271210291

10421586

111139244

514

2567381791081

27177211313
29177231323

1

1210

97486

4121

1212191

1211

42878

22911121

1171

17

212648101

641142215

28221874

861759644525810

1

69116101

92283

33

13473641

11242971

11242971

7251853

9316111

271273

1376

714102631

10541252

1511293461

42231

32271

10115

9313

1915118

102221

1541866

2231135595
2331235596

111

5221122

81073816

2211

21

106583

156101012

1673

5351021

1113

323

25931032

101275

175140

1011534

197331127

71

1115116

991314

8671142

41414

7278227402020

122281

1303312

141

12221212

29

2236

18831512

3

42110719

2273

4111

721216

591931

1229

54110
114411717

64717

1

91124

38917181

914973

454232

331553

515546

12

3431433

1210

4103326

21122

8542042
10542162

212

231

4863138

42251

12

154431512035

1021564

31118376

3688825

312182

9181

3182

7102342031131
414

7102302021091

921391

5451412

571361
571371

1

171126

61121541

256713121885

2

2

1

6282

83493

1111

2421114
2421116

2

46887

418323

4952893

111

12

11

566103

101141

51

19624251

20631741

61363

11471129

1010123722

1324

421251

7411163588

41931

6421514

11

6213

41131

521946

141261552

635131

2867255

3

24

13741375

2

7234241

51224

311105

654845

33231220024

232

77214

31193

2

8442352

1

2432

4174

5926141
4826141

1
11

1

141

1819

11181

12412511

3651221

179421257

85417101
105418101

21

1613323564

23654

71214

2

7131

845116

17111

3182

41

761461

215310132

52133

12

137401051
12736105

12

1

2

1164

511971101511

13162244121

311

4521683

164

155210143

4615

2110121

21433

193113188
193115208

22

1121419

2

53201322175
251

47181115145
47181116145

1

42212

41382

5342850623156

1211

3

1031304235

1

63432

5216131

415

1052730718

168621111

52221
52211

1

106274

1393

2458

188073213883864

2

62563733

524

21

3242

319636

71535
41131

344

322332

1

303413274011

303413274011
198733

3744

224524

1377141

56317

8213

21105361674
1064541143544019

1410830018

2281341781411
2281341791411

1

371212205452

1253150462

542152

542152

3216
1611326136

11411452

2511224

121221
773525764815

7436102

14241982

352419203

382823

5961351

5555

73413

131033165

10421442

3611723

140931916889183655753390862502
72

23

23

23

139731915289178655742390856498

139731915289178655742390856498
77217100850498333519247953284

2

1685127114532511

21826261530

1658

1891876324

41

45172922200

1231088812962625655685367168
1241093472973325742085747169

14661078643801

10669811011171328585

729202620

160011910562666

2032585330

1266581405111

33791146721104

107

1162

54164

3491143031705

133210129399

103719628687922468

122019324471358

13121

21162292562233593107714

110925013542635

3598252182761
3608252185761

13

2684339

1

111446116

2112101026

71847161

378110170921

114585248721

1661

31072087

29276639

2729091698

19491

7665616571314846677
7701017261391749487

35469769281

533314320

10162444

10162444
111

22

11

16132

7711

11

105128519281204
73923645316931107773

9212383

9212383

5753368
121296411231

43681110

622
522

1

3156221

8

51

1316721294
153421

124212

11812

10721541
10722541

1

16123928243
4291

2161332

22361412

5271751

21732

111111

17202171
150102236111120

91531413

113371457534
113371456534

1

53242

6272252

32021651

32021651

4174536112
108

2589232

210481392

29227413163
2021

5111

8511111

556541

105025

645324

41699744176615
132111323146

51

81

63

227533

132

221332213

162573
166573

4

22

4604

1

18852

29261582

11

471

31

11

81

14681830732

141

1511211

19

11511

121

1

21

72716742
171

2122241

51324322

104169361041961
32852013530157712

5125

2037526193

1218138

87421450281

4

13431523221

932214
1132214

2

94525347

57383

91

1

41223

2066724224

1117710521

1843159180
1843159186

6

4818111

111511
12167762019201

71326913413

772112

27684135

35

265321

128

487334

487334

21302771
63061021192

98266

14312

162323
3633341

21111

114

325136494121

241922351115
14162

642331

4592131

414312

1321

62112

2311
3

211

1753171

163341

1213

278222

278222

51819113

26167

312343

1411614122
77142511057888

161161

161161

4521311

4521311

11253045

11253045

22213
91468811

334

11

211232

31014

122

21621
72651024

253

519413

161751

161751

25121

25121

272716192861

272716192861

283241

283241

11314

11314

512

512

293

293

11

11

312783292992653146
102441

9
26522

9312
10312

1

721
421

3

84721104401
311747285988647143

35141

2
1

1

21041
1931

111

12
112

1

413741

433

121

280557241786527138
2342489327217290

411811

111241112

121

2

11

2166

2272

242877650333641

351

1

1

23161

14

211

111
11

1

1111
62111

511

5

1

1

11

11

152749522
112241

40747481

312

10236

121
4723

2

46

34
12

22

16

1

1

271938232329
830644717400709751

7121581110

7121581110

7121581110

7121581110

7121581110

673052316659

21111192115

21111192115

21111192115
178531

931393

11121911

11121911

461941224544
11

32132693130
1

2341261721

1027397
2341261721

1

111

1

51210

51222

22141

99143148

6252114

6252114

379134

379132
379134

2

13614131414

13614131414

13614131414
43252

318954

624448

124166904810095
222

9513463316354

79623
9513463316354

23201551921
1

1311

1

105611014

111121

364223

61332

1411

9994

9994

33254

33254

63292

63292

6771

6771

335541

335541

11
2875841011

1017165

1774323

122

132236

132236

4232

4232

565774

565774

762488
273027173541

114121

25441

127312

1

113

1

32112

1

1

123

427286

131

45156

1

23416

112

15222581313

15222581313

15222581313

101318686

101318686

597257

597257

36182572316

36182572316

36182572316

2210137187

2210137187

1481259

1481158
1481259

11

1
396302362196392389

132021141527
23213

4

1

1112

41

11

2213

3

12

31

1122

11236

11

2

1

1

244323

4312

1622

361275327173348347
171016152013

211

711111

23122

1

11

741078

11

1471752018

3

21

12

1132

121

49961722

133622446

1

1

413412

21

11

434222

143

133

2

811113

2

21

11

31

1121

255

1111

211

233242

312128

112

1122

113

1111

111

4

1479123

16

2111

1

1

121

1112

121

31

111

11122

132

55143

11

1141

423

211

12

411

311

121

424153

646455

211

21313

113441

344132

59173168

1

1

2125

316511

11

2151

134

54322

22112

22611472

15151081622

311

621051610

1213

1

113824

75831012

11

17211

1

3222

23122

12

5112455

1182

1

1025177447071

1631

1294

67114127

2171492915

2171492915

712446
11

32221

1

314

1

2

291017111214

653325
1

1112

111

311

1

12122

1

333132

333132

333132
212

2211
33112

1

12

11122
20211777

12321

115111

11

5141

1122

744342333761
1

633934303357

633934303357

633934303357

111
633934303357

382622242534

372522242334
382622242534

112

2412116823

1912116823

5

5

3513

3513

3513

353
3513

1

842241

842241

842241

1241
482149312859

321740191613

321740191613

115423
321740191613

21211

222

21321

111

128197107
138197117

11

111

231

22

154781245

154781245

71444
154781245

733425

1431416

254614131856155944983554

1358011176342223
250313791840154444843543

12

12

93948052635110391015
112284831165122

167103673013890
241207

11
795647239938

3345163

5474114
2474114

3

1433133

121

11101152112
564633145626

45362293514

86471661945

86471661945

86471661945

9274139

9274139

9274139

14441337

14441337

14441337

128140100386663
423141

1113143

111
1111

1

13132

14

14

9913271151730

11110
9913271151730

1

9713270141720

21273

21273
21263

1

102424

102424

216121

11

115121

10217121621
121

811391114
664109

217515

214236

94462316
653644378959

1141

1141

61343115

61343115
61343114

1

361121142827
734325

1113

31

18351104

133432

2122

13165

1226
93136

1

8

1

1

1

13814142310

1081111178
13814142310

32362

1

11225118

11225118

11225118

14246835813498
713

828653

828653

12744745212695
20414143219

213282
213272

1

1823669

534165
534155

1

72431411
424294

3157

151111113

8712612

2513294
2524294

11

12482117

4210384

71145

274373

185256

1395

441722206160

24127
441722206160

66231114

4421420

739443

10211912

152581411
152581611

2

318342

318332

318332

1

1

14453711
257101141121345467

1016549
24112393722

82

1

121372

25

122

21

11

3

1

11

74152

132

1233

151

531674

1231

41231
41232

1

12211

63241

63241

191719788266405
28917186289

21336

14222914

51551045

6121726

111116
111126

1

335392

142

1153

2

751146

112

21272

141
151

1

11313293623

31

21122

5331125

64611221656
294671251

31

42125

2723131

11

21216

1015573

193372828
173352523

1

2234

1

72221480

2343

11
171212132424

5784146

115391018

28143528274163

28143528274163
1

24113326252156

24113326252156
24103326251153

113

4322217

4322217

6669830
52629148433817401164

515334
13994182117145213

11778159101118183
23722212457

11

111142

131

111

2311

1

311

112

315

111

1312

11

11

1

1

1

112111

1115811

1

346

1221139

113

39711

51

11

212122

1252

2112

41

2

1

112

1323107

1

114

27312423418
44102612

1

2141

1416

1

212112175

1

1231411

74215

13

1

354144

1111

9

221

1

92583

21

5421151219

31123

111

8914101616

627469

14244

32233

1223
14123

21

8633610
212254

4514

1

1

212

112

112

20111251212

20111251212
1757237

241133
241153

2

11

124141

1031372312

1031372312

1031362111
1031372312

121

403741192917

403741192917
1154274

1

312

22

1

1

1

11

1121

1

1

1

1

131419152
23292951710

1

1

1014103106

12

1

156

1

694548176054

9312399

6312399
9312399

3

31282592723
73152

856145

723121

25233

221

443245

234443

144153

9411118
127831519

38221
37221

1

31

1

1773293

1773293

382179611224622

211

211

211

381978601224622

1

1

271977591219620

9142

21

222211
20474103103239204

162557365158161
526292

7382619

7656

19162158

424564

882824

1082455
1082355

1

51127103031
51127103231

2

122421814

511286

4295512

2373189

153125828

1325152113

522542

422532
522542

11

16558158

16558158

111015204330

727123920
111015204330

4888410

863182

863182

574564429273

22
574564429273

211210

31

133333

4586214

111

1

141321

11

8484148

282328206134

48611

1121

11

8332129

8332129

8332129

6321119
8332129

2111

131610182020

131610182020

131610182020

131610182020
11122

656375

6104141113

12232
736427585669906830

736360978548
583357506592726703

342
191114166210235269

1344
361934304675

3

121

1

15613131310
15612131310

1

24511

512

12516142458
12516132257

121

11262
111086185

43132

1

43327

232123
232112

11

12543612

12543612

23181892615
453466

45341

158125168
157125148

1

11

10662102158137162
1

54199119

1531051526

865573144111126
865372134109123

211023

210112158197253224
534343

224752214
341018204035

312139

225213
225415

22

12

614992
614994

1

1

122
1233

1111

311313232526

311313232526
311312222426

111

12
1586785

1466785
1455785

11

11374196

11374196

7666714
435571

1

1211
121

1

2112

5812111717
5712101712

3

112

32171179

32171179

995975112126109

8549541047662
794752997558

622514

14102185047

501935203265

501935203265
12864616

12

1267511

659132

224211

312

223215

162441318

61542112

61542112

61542112

292740554949

1

1

292640554949

461071521

68725613

126311126

111

4487148

3211411

32431211

32431211

32431211

21202863925

108831113

651211

325812

11212

86143229

86143229
75143219

111

36663

36663

36663

54731211
153697775177125

19372206

19372206

19372206

15171016128

15171016128
1241

11168543

11821

11821

1

1

31113

1261682016

43175

43175
43164

11

426446

426446
15326

112

111

11

236293

1
236293

22541

1252

21112

11
21112

2111

572212226142
42399

1157

157
1157

1

23213185

23213185

2242102111

6321044

161177

12132
71666810

383121

35425

12112

451725245242

451725245242
3471064

112

401215134322
411218144626

111

2

1

1

11

1

1

1

1

1

13236107

13236107

13236107

211331

1112376

472119144937
32332

2111

2111

2111

963246

963246

963246

12855216

3411
12855216

1

3241163

521442

215762122

51351217

51351217

1644195

12132

1523163

433416151411
1

4681
871913

1

1

1

1

1

3111

96411
3526156138

17121

2

33121

22111

1879394

1

1642932231537

1642932231537

1642932231537

1642932231537
1114

9866113
31212

217

464514

12
35147467

15145225

192222

83271039

473232

362487

26511318
211

915312

15455

4856382
31121

4545172

4545172

4545172

4545172

1

454522
4545162

14

977495

977495

977495

977495

977495

765463

21232

1028072436989

352329214238

92323119

92323119

92323119

1

1

92223119
611714

1

1

11

21

211213
211233

2

262126191119
216243

5

311

2

514623

196548

597213

3331

1

675743222751

20619101523
595241182450

2938157213

73546

352138

852431
54221

31221

213514799

213514799

213514799

213514799
111

1
516331

2121

114111

21

32

32

24122

24122

720311
13267326

111

1

1311

431212

11

273220259212207364
146613

1

1

422327212117
835875565251

2

1

121

22

34222

15121

11

1

1

4211

11

1

32

134933

1112

1

14131

71211

3221

111

1

21

11

3153

131

5731

2221

1211

1232

111

41951

1

4425

2111

111

11

189158178149154310

180140164143152297

180140164143152297

111
81685164

81551114

1234

613131
1471971518

142121

142121
14111

111

62114314

22412

429212

12
1131

1

1

1

1241

1241

292238123843
12389956397185

6127

95512526

445130

311516

1

2112411

412

4413211

22121

412

1045325

954244

141612

694472

544368

651132

221312

329233

73343

8353310

391156

13233252108

13233252108

22510161482
13352

102152

711244

3114823

1

1241

11

33
918146213

1111

11

3

34629

6134

763175448051

642973428051

642973428051

1731122

1731122
3142

13115

113

124221
632270416849

1751616167

1751616167

451550235041
4471116

19414122124

22729101811

22
12222

1

1011

1

7675746
87249155796187371892311680911172

121
383751131425

321

3812

1

12111

51

1

1

2312

33

2

1

111

3286912

155

232517111

562230201722
1

471421131117

4

1874435

834113

4413

26311

1543155

989755

11

683511

1

31

1122

3111

87186055431186884889591643710757
263662161712

2898090615168
1

316633
2177072504352

1154836223035
68161310812

631171

6

21113

5161

22

1

36642

32

11

1

53211

114216

31

1

1

93114

1

3443

541111

711630281014
21

92332

92332

92332

42
41121317511

5211

157

231

2272423

31

121425

3111

19214832

19214832

19214832

72101810816

3413123

28311

272

41122

152449

152111

1055270459449

2692313138

2413666

1725431

2111

53433

794347328141

794347328141

243
794347328141

111
621928213326

1634481

41417
101437

62

62111

451624172424
7131

3443
2443

1

934152
633152

31

2242

112

54431012

3122

53321

143414

88751410
141

191

12249

1133

5112

546242

546242

4124426
1

62

35216

1410

73517836

73517836

1798587561391400485354
87113055288186645888291627210622

4086162767138810897323281128
1279121316596

1317209124
105314773

12211

1123

11

1

132

3

6187523642
4072062748138659889923071118

404941260813832586582125953
1844883340761412065

10722185651215

10722185651215

9696534964
2884151661591912

9814515274
9354474773

46451

9345625864

284220834
196832183842919

2994461662

41512382175

46110381762

5094412276

7006663578

7006663578

14152350833661345316
2713631481286052

12042986573743
11852884573743

1912

5801831202214

1362181754

5602053282218

739124631910

323361

545183524178

5991836392118

103684331135

5781534291822

401152025713

10724561411814

2012

40382620320

611123240138

1993111042

421822181411

167712541

10241747401915

6471134342022

4651835181714

1449031237571264531325398
484642531613216139074

34851797146315

603236436143

18897110100226

351414112145276

219699695164

53874318104

917186430227

189846961156

41411782702691488

111

11131063513141

84210374077

7607311794

5494123206189247

8631703784334714
8592693774334714

3911

898195944138

353819129150308

11167485292

1041124741145

11037504979

349419132161296
346119132156296

335

1184146743122

13998665994

1718269293133

7849524082

52

2456201041271911

399318129160295

2154159682207

6956251943173612

323640144155186

573337273226389

3504281872

686253236297407

3730622091916344

2993132121

713112224148

361211102112219

25052111123

14261447701313

1333843682112

134643

1026124946104

769103042138

764103042138
769103042138

5

823156440158
891421

3168271583

4186322374
4186332374

1

137839507462124

472102529716

13615141096

77014468752

21784480012727245921980
143933237816171168317

419121701051

5256501532

7921661341229

735153391252

270626714

176872312094124341

179682590529101

179682590529101

193007994201213

72742322051094

537593214

1953481012

1080387093

16

25140

49021161753

2653751221

59812881691

580101432442

8

1

7

27423189

389615621

10427393113

1171264241815
1138263521715

1621

1770

300681306

3161121132

54213427

1963124231

61211721022
60111611022

1111

3132691421

41

41

1643133282204178163

2991125321511
1643133282204178163

7764323

1982133244042

1913221

413

1

2882548312932

2021227122017

2827514

3432

9492317249

17696116

1712146542023

1531

1901548151415

108787921571980977790

108787921571980977790

3693947413534
108787921571980977790

4627516210510897
111112

2073269406137
2153470406137

821

2464091644658

4327151
3862948312737

4021312

21212220

6136532

2212033201612

3383322

3383322

78181111798375
715264

1971936252525

1511919887

2192126261920
2092022251920

10141

2072125182519

112121981111

112121981111

57847104727445

57847104727445

3061048281611

3061048281611

87737131414734
673166126

3715

151123010156
151123010136

2

1617179711

2571129765

204334976

1593438304635

1593438304635

2883029111316
3112

981114349

187813897

1034431
6047985

1712222

331332

8289014910010273
12111

40412352

20610181282

1014811137

1373845352931

2582051253722

45108764

4026734

65989139839197
781731131818

1512419242520

1961424202122

1271024161118

1

1072439101519
1072440101619

11

68312542

68312542

4444172424279194178
6801952452925

33741927206

2851523989

2329287105

3511026301632

29612271775

24681910187

3131029261324

2055231692

119417564
117417563

21

193202312118

17115258108

26972222212

1988207910

1337201196

277113213912

1398191483

2123443

2123443

204232

204232

42730934910933

42730934910933

31210126281911
39866346787295957355036642404

39810946635295307351036372388
18221621031492116

50315299617774156223199
39470846294292307327435392262

12135
2462116122546

5665158

5254115

5564822

7143986

5265331

184321

3422121

216874708764
42121

53129311

378732

9642739921
121764588043

18134155422

723417

49715333
172233

45

42

1121

22

171

4241

223122

223122

82714

82714

292522

292522

1502163061011

1502163061011

335823

335823

41216408712
4034122

38121

51411

165215314

656121
655121

1

392111

467611

142111

241611

241611

14515361

14515361

11
13816131266

131171

4565211

10

2831

4194343

82115

82115

90354112
450252335722

21121

3

20232

2221

811122
1251122

44

60112

112

18311

473222

921

13113

8

31111

1511

3512

2

2

9

252

151611

1221

4236539

4236539

10857536

3911313

1922

5044221

201626131630
19146311

395132

2411222

131422
131322

1

221111

511639

21211

14111

17142

1084213

1084213

1547108416
591317

6421333

231425

83231

30918191732
29131

9861331

1821251121

161895914
31

5633379

271311

471122

283211

53046102632636
9914282053

1023

3955511

6

90341

35285317

87213392

64519359

35

751

908162634

5955543

5955543

2
52535

24212

26323

2931

2931

4326951

4326951

1754913911

1754913911

1153861071306795628383
24653849343148169120

808816

3312

871017522

5635142

2725213

14269

96513332

72181

8543453
8544453

1

483132

89123658

311213

1238153117

260686114

2917311

1025411

352193210106

791412

356521

106153731

4058231

10315

7076422

343721

1932511

121

1

5043244
5043243

1

5215116

8241334

23643217613

10420663

4292731

63233310

1125916141226

45313

7944613

89925922

441311231

332412

12446453

264685127

5644432

1869188114

6656223

133318242

1505141244

691544

37551

2994132

931414421

21921

64616231

6310616

337752

1551

322416

5493341

6227112

2521131

8265523

555414

503131

512264133

6712521

15638911

28421

4811312

456242
466242

1

8186434

31231

381322
391322

1

40115732

4714672

77514431

2

1

3417210103

272933

572552

35163111

9351911

10111061
1011961

1

21

95871159

609923

509122

24331

5411271

11487443

561010011

57172292

8154122
7554122

6

171212

461

13912323

57512811

20542212

735231

18216252693

40721

3722163

2129

39412

5852522

364125131

119919915

4744431

3579921

42108352

282213

2864224

761621

6825111

932

657121

451194611

1661114986

164733453

254518668

5532132

1521

6761023

2089321

264233
184233

8

471116453

481161

3383

12546312

2211
20911171834

42111

282211

4831

631

12311

7211

16132

2723121

11

156113558
5302054342435

1821113

251116

421311

2553427

11

3351

1

1221

4713531

2412

211213

1

246322

391422

1515

20352

2111

4

102232

22221

294617869
1721

311

2611
4311

17

78122

1523223
152223

3

4611

7

471111

271512

141111

1788043181324
40111

31343115

25131

427330185

24231

16121233

51512362

2438311

272451

2211129131279
50163526

294125

423525

142112

415105437
415105438

1

36313

91

3734466182534
3473275

36331

13411

3322

21110132

4927212

1462212

3111312

15212

25312

341012136

201431

42223

9314

134512

1425212

169512522

169512522

5311831191323
12326656

12

253132

29122

192414

552411

2341225

483342

1211

501213

146351

25321432671461917
1248481126663

1

6981

1622631223

416506634710
412496634710

1

4

12681362

38215

4729351232
4709351232

2

1

182111

182111

19
263922131019

111431

27121

501563

383159

86341

327336

88571

88571

221
9818563

31221

4315331

2211

23
79714133

3821113

39233

21713301179
5627312

591922
6011022

11

26223

61

421

131311

12

3153

11

2623

39247
3451843171149

401345
431345

3

16783223

7248221

102113

12019721

2217128

14221
23321

91

4223191

4223191

931114677
47311551522235

201113

191317

4871220

2052229

82211112

41104211

4127

2843444

14117

5211316

521112

631231

631231

102
22110105312

5833331

15357211

33122112
24381314742

1911

221432

81315311

15114

4715112

71

1911219

2011311

2011311

20414264427
4496775211636

42221

2

32291

1942
742

12

71

22121
19121

3

3833512

10

1

1

2
3

1

32

32

1012
1013

1

31

172

2

8394

6732

4522715

72722

5

12

21

1532820523
6310331

2152

11

88215212

4826114

3

4526114

1212

1212

13291210612
21

814113

201422

18112

22213

6441642

83478173601075238981902233
2866833596724347673352111536

5302411110723

6286536100101

48

4661701081009

417651287653

314247316121

1881138311

57816112

2571513211154

155251

37541781544

942613632

2211327151

20946315043

3991458217843

695948219811

4583336233

13317769811731

2457810

557538157251

441414994
440214994

12

348114121261

173124

219706110961

5639132532

386313204931

67112672167179

1047333151136

14048323121

1635159222
1655160242

212

85172033

71486108191113
71086108191113

4

24934224313

49062119

1

82141151

213812302

326883918943

2503335573

30724583961

80328211952

111

13959316113

20127709212

6891926

1321126231

4392111

1501121432

301272833251817

1328811838

148612252

67965114112
68985320112

10226

42594

5310913

172553878

422119245710009182

93321726

103101822

8518136043748

23343287271

87102356112

29940554711

26555315263

15

1121422201

935246251

1089295473

9676111314133

114614212

207110202361

22953559111

15716194551

68990492994

520917101171
536417111171

1551

20617413222156

30601241043

43224501310

2112124222

5071886511851366

436816161244

39224779111

332389465

582219133129695

17713282111

12020283731

362932331709044

775763018722

166110109187172

44681563321125

19724163041

1293637131

15911312113

3593167392

67141636724

16124242351

33452422491850

27847438121

366211141062

2172825472

7145335543

3994824221401

4223155331

27637571111

213154959211

3901172934014424168

502118805591

429378748891

29925183531

13324414

8210235211

98548407838

180631038133

30326344722

56581637751

5174112065424

66918016045831910

2531115

13152161214
111381254

2014471

510711019151

18221

4275618

2064517

221111

11
112275731

3913142

271125

4533124

11742824106

11742824106

6728424

6728424

5259251

5259251

353101713144
1821

342352

5911

281111

531126

3522313

351521

38212

5311

2151327171218
7213

413

18155
1715

15

9122

791312

194131

3934344

2012732

2021

20511210

20511210

6025222

6025222

3384244191814
5226111

27372

31113

35111

17211

24315

1031

101311

4591

2131

24

42321

981522

6411

511

2753431

115331

2111

67222
17811152710

4314121

111113

1310321

2823

163

8729934
2121

1011

1

4424722

1011

13

2824232

2824232

72217

72217

47132311

47132311

44214213
2211824747

362411

23311

25321

201

12313

271

1221

222212
102212

12

199529364

199529364

6924713

6924713

12112

12112

15781732

15781732

8123

8123

26183132182946087
546816435

511

641222

44712

91

9251944

81

81

104103241421612

2914112

1

1041

211

3411

373114

13211

1447426159117

5717815

575132134

724

21

1

87102415114

521

6111

712

311

41

712

15965214

7

231

4137

1

7621

811

31213

12213

19

11282111516
102211

353711

2011139

17732

233413
3044413

714

1101413224
135321

321

682

29664

81

81

111211413

111211413

573617314

24134
573617314

8221

3721

211

1

131311
4111

921

1
111

11

1

41261

322311

322311

322311

1702234866

991816544
39116311

52

26111

15311

51

21

11

1122

51

1

2

21

71418322

66415321

531

9016883483668

31

31

1211821

1110711
1211821

111

1

19563228
8865775453667

91

92111

18112

6211

4841947231420

12211

22112

11

311

149221

1

11132

101

5531

11

7211

2

4

1

6694152

1

4111

321

1853364

1

121

1

2332

4105062243130
4422

241253711138
5524353

67520532
77520532

10

1091813353

2
4210733

11

92421

81111

1571

71

1525473

1525473

2932452

2932452

79107315
3

45725

321

2833210

911121

911121

911121

2

2

24251391265

166131

28

1992

101

42411

9331482

153713131

1

191

50244119646161

22
50244119646161

1191134211417

263111154

263111154

9382310913

9382310913
9382210913

1

92916797

3645244

3635244
3645244

1

56511553

56511553

1
2762161363536

17322

17322

553
2581861363334

11472114127

2735432

34410643

3933135

1410764

25171513

133631

133631

734049183941

734049183941
317212

173631310

21

17363119

117312
164125613

4113

1125248

324131

324131

10611256

10611256

1256466
111

1024245
1024234

11

23121

1238353

1238353

59310321

5416211

52411

2409526598116282779564937

478441875818394
2409526598116282779564937

158324212621

158324212621

158324212621

4213443

4213443

4213443

1
2601102711381440

61511

61511

11
864115

13111

63313

802110247810
245892661371235

8221763410
802174349

221

78467848213
83478756415

519822

1

443512519
2473141265192172379

31
19181814712

107211718

8157764

6448347

5948347
6448347

5

6345310
3101934292285

67454214

5535736
4634736

91

336887

47233810

45492638

66413652

66413652
66413651

1

11531291219
315247

14122

3133555

394115

168152081648
1398761429284188

481332

37244

331346221

364413

12924247

8235558

34582230

8868836

5222232

2

534422

11991710138

4845656

688634

193345

74411547

161124

321721

8125224

5024232

5821375

7116579

59574712

59574712
58574712

1

867115410

867115410

123121217824

123121217824
120111016822

31212

1731141

173111
1731141

13

1
9010229914

53116679

53116679

3795325

3574325
3684325

11

111

413168416

29277313

29277313

1219113

1219113

10246755

10246755
4522

3633545

21111

2531350355833
9435759149684813831305

3274012851131114
1715209564250576396

1343074446975

1

1

31681912

64210351

701228112510

1923156346444

6813187296

107924133924

2411876184921

46413121617

821829112616

100929123910

100929123910

58716153019

1941055184527

5342558

5342558

22313241297

22313241297

104821141512

104821141512

2722331371421
5726412

1291115241013

5369723
5389723

2

3321213

1471222101512

1471222101512

1811
3642751172240

1421026644

2041724111835

163834153114

159834153114
163834153114

4

2442432232640

2442432232640

8063566

8063566

977119105

7629785
7628785

1

215222

300825192714
8072066555924

1775231483

25531317185

3621241

3924321

2401532212116

7178384

32515858

137391084

30222
139816101413

7646531

33285910

2

7772570375739
3237101254168171160

143626849

1816131352

1287135324

121515867

198517151719

164511675

4191930242432

228514992

2922211493

2791214102313

3074101975

34210410676207405

34210410676207405

1436282
3572659263021

138321684
137321684

1

10213166810

1037161265

819339111411

819339111411

15258641

15258641

2151652276427
184311

981026154220

996229216
996229206

1

58589311

9212117922

9212117922

2413114
1301105151117100152

3101225252331

3021019192128

53511

323112

115412
11911101085

83221

281233

5513

67231
66221

11

122310496

122310496

8551731019

8451731019
8551731019

1

15241415108
24

7734551

3415745

391312

253262
141281528718

4887113

39114325

29621848

594105711

594105711
5949569

112

475186610

475186610

1201822111229
3842858

152516

30161

235322

5731

9191

3243412
1221479711

4381446

4723123

2235753

2235753

2235753

943841488816395

2444236313651

2444236313651

5433332
4942237404114

897105165

12

350122432207

71145667419

71145667419

134619111211

134619111211

875013925522133058882810
3085523349215127

3051734273322

3051734273322

12905913210312355
9981214236

122142211205

36482927216

273226202815

269820171612

1631923141511

19987319015316589
3911529364216

16661112133

8438812

1114

3261035192420

641141045

160911872

662613

6051212128

358933251912

15181511217

161515111910

8512759252251166431
46421166478897152182415

43530184510

1574423316214142

7819105198247

99311083113188

1128722111433
1008682110832

12461

4975274215

128331053113640
125321013012940

31417

31645136228

1501789218841

2296019262258139

1333110427164118

951764228514

2223122823243139

11239682213033

117211093016158
120221143116370

3151212

2825518744391116

74115313519

493269197717

180231503215679

912231125987827563

121113133

119632218722198
1256622790228109

6363711

1013979366820

9916601410231

14123852313053
13922832312951

21212

552373214927

3113

2072214527134102

2072214527134102

3225408533296230272

5636983694844
3225408533296230272

993222

309116

1825

62431

140121912138

128313

1724442

121

1

9621

24541

2744212

82695910

5825331

991022121031

1

6729311

1

3157572

1

511812658

65113613

1

6

6111322

176102122163

311

14541

11

311

141113

3435137

3314112

23442

711

1633411

9311

14

21311

363311

67181717423

1643217

1912221

21

211

121

23211261495

473611

1

311

11855566

73221

1

7

52321

34512612

1

29

18244

41

1

56311511

81

2012131

1835122

7

33361

82222

31

1

1543263

15242

3732

5111

30421625

10613

13112

51

171

1934424

81511

7232

9655334

34714742

6

832724

4582493510

225263

2

1539813

83111

2

51

1

5

120611142

111212

1456111

70349131703718530634
94881233

81
1153108159866690

189122212811

189122212811

411318622
311

211012312

211012312

17362

17362

7162
3693441341730

217741

217741

8389272

8389272

4442427

4442427

107471024
81

1214

3533222

521332

107101012517

52332416

55771011

4003865283037
8121

1622426

1622426

7531123

7531123

11
6812128515

30844214
30945214

11

3828231

37210342

37210342

2
967131118

1819122

766496

411742

411742
411741

1

266722

266722

3353322

3353322

13
14611136810

67462210
3

3233212

321318

325523

325523

342223

342223

78415933
1

2132311

632

5011062

57097931521611458538
46645108512820

23310221

6
213812

212

631

76

76

1

2211

211

11

1
2943055151914

421721

421721

13121

13121

2502737141512
35131

3825312

1132

7323

1121

71

11

1212

725

1

5523

714132

3324221

4112

21

41

79524

74111

1

1

621496615
48892192472740

611

611

1111
1168221943

11

4822

21412

2921

3646121

521

52

4111

252398122

252398122

1463844151014
461020417

4221

1

11
1

1

3

833

21

339322
268222

1

71

21711

63122
6212

12

1521

22

121

2311

2

2

7211
6211

1

11

11

711
411

3

353

251732

171731

81

6738251

6738251

22

22

2

2

22

22

17322

17322

5425353

5425353

5425353

140122513128

140122513128
111

4858917

251741

566947

2504478383843
121111

621

621

741020435
2093075313740

4113

12

1

311

1111

1

2118

133

13111
4882014168

915192

1

31941

11

812555

111

241131

21111

2716124

71213

1

411

2131

1516621

54111
431

148

21

1

123111

1

102141

102141

13121

13121

17822

17822
1472

312

1634262479170171167
10716

1603260469164167159
58279152404061

1119

1

19121

94321

1

3

38512

322111
30313111

27111

718731

2135
2535

4

1612642251915
1611

31

1

124

812

61121

1011

131

1231

4

71

11

10721293

40410211

621

1

2

5

813212

11

1211

23411

1

1

1

2

11

222

1

1423

3

12111

10

30810453

7

412

22141
35141

13

113

112111

316

102

1

712532

371211

20110521

3

11

7111

21

1

131

11

3812

411
412

1

271321

2

1

111

21

6424274312
1077359264644

1

1

11

404731204328

1

11

1

112

7511

38513214

21

63111
53111

1

1

9

1021

1

5111

2011

44312611

1

5122
51212

1

212

211

1

1511

91
911

1

41

16641
15421

122

1957252
194723

122

4211234

11

3164
3163

1

3

515

1
11

1

21126
21132781

12181

131

142317
14131

17

52

12211

1

103111

831

7131
11141

41

1812522

1

1812512

31111

31111

224212715820
33112111

3683433
86119843

1

1611

322541

13

1

31224

1

105816535
5416131

63

9411

152111

11211

101321

111

111

105019130011096129
2066113

1055212

13
12

1

5522
3522

2

421

95617328410586124
2424446191624

31

732

3424

61

3813118

1

1

43110
42110

1

26432

1

5111

111
1

11

174411

4211
21

1

4

6121

2

12111

1111
12211

111

2101

51712

121

3231

121

11

1

6

12311

212

234

15261
47293

3232

136

8412

12611

23

1111

2211

1311
1211

1

322

128711
1310711

12

2

1

1

1215

5

41

19421

1

2

151612
15112

6

44419685

67211
67111

1

33117

216

62912

411

914

102

11711
12711

1

12

1
1583851351817

31873

11

711

9

21

811111

132

1181

10332

62314

1683363

1

12394

16141

9611

11

425321

7461

10123

2112

262211

1831

1675

11

545
645111

1111

721

1111

18512
122

1

65

3

612

23

21

1

11

1

2

1

114

71

1

1

1

1

1

2

2

9

21

222

12

933
12311

38

711

121

61

11
111

1

1

1

7

7

375418
9118

2012

732
32

43

1

1

5
20211

41

82

31

51539125932355
1

46233119911754
1043181928

343131812

6211

842445

656111334

656111334

8812581731

2541512

77111211

47131230
48131230

1

71

41
2325151

15121

22

41121

294111

294111

338142

338142

338142

1
52144107512536

4632597472220
1365331227

4325622
115112

3215

1

1

1

6232

77620553

191281

6112

3

113

16131

6711

221

321
22

11

8111

1121

1452

13

2

3619231

1

87

2

28231

238211

5719104316
612

111

6311

211

61

11

161211
1831211

2

1

1

1

151242214

756133

756133
231

1

11

533122

332632131512

161

161

161

161

161

1121

1121

5222

5222

5222

5222

5222

26252411129

26252411129

26252411129

26252411129
161014459

321

11

21

21

1

1

1

1

1

1

11

1

21

121

1

33

23

222

1

93111

93111

2912824

2912824

111

153

131113

1761

14313387
8232

2121

2121

611122

611122

62312

62312

10367135
372224322203295323

17711416284149133

17711416284149133

17711416184149133
3311

437681
976596467577

1267364

434251

4144613

5136415

134356

411525131613

36644

271223112430

744665387141

744665387141

3214

3214

1

1

331637141921
182104154112131185

22121

211

5335617

111

337366

1

1

371532221734

11

416121

96471311

211

221

321354

362230191723

2

21

11

11515

1

2

111

22

364457

5433610

11

162234

1

1221

1369101119

33

1317

6104113

332
2

312

24291531412
811522

71113

2

2

11

5126231

212

1

212

1

22151

497338212312

497338212312
1

1
42693018189

414221

414221

313221
414221

11

131
1335231086

11

11

11
15113

3

11112

41192
242

2

295

4167252

4167252

322521

12

211

211

31

1
25205872

74333
21

1

233

421
321

1

1

17162542
915243

1

1

11

11

61
71

1

1

748253

748253
4

623

623

34223
22

1111

21

11

1

12914103234
12841092121097217252117

1
6483675190157

11
6274594984149

3740423566118

3740423566118

11

11

3740413565118
3639413565118

11

253416141830

253416141830

253416141830

198268

198268

496319255262458694

7634815
496319255262458694

171820171633
459300231220429609

161815142734

161815142734

237513816

237513816

28139101024

28139101024

318
353230315583

275779
2758710

11

202023204147

1052378

1045433387397
11111

2320962231
2320762231

2

19128171421

138781216

4913862228
4913862428

2

881182630

881182630

115726054126179
21435

168961420

18167121319

1310852236

20219113335

25712121825

2191182339

1115621122

1115621122

1026342337791
201044911

1022961817
1021961817

1

673269

662426214354
662426214454

1

281119281770
3523

2252218

817

222110

1397241312

222104

222104

1
443346703021

11

1

392943642614

1

311434

1

1

12

12

12421
6025807665401047964

11695611

11695611

108669862177134

108669862177134

108669862177134

146162186140268259
482506655472861816

111

21453

65108410

21101

13121

1313565

710641727

14523

72871507214490

372530173228

22272

411141

352946429172

161612123124

2

132113

11118111318

224049449147

121221

21

34

311224

2

344

22

102129264728

122129

34425

2311

62125110

3519431

637569

121

112

3321

211

7257

12121

3106984

74433

1112

214254

92123

22232

3313

1

1132

521

51118

1

643123

11614

22

287566

9351517

15

11111

211334

22

11

311114

11

2572

11449224

112

112

112

17151784157

17151784157

17151784157
1

64643153

64643153

111110414

111110414

495345316490

31
241110101819

43254

178891315

254235214671

254235214671

254235214671

254235214671

296426821912118114521411
81073410

1146667324152
14981151692321642471

2

1

514325

12

1

642123

31

1084775

6548712186

1

221

131

14312

136

2113

12152

176493201133

1

4

11

1

142481

1711

2121

12

5674

442166

111

463262

4

9215754

2

434168

23

381416123218

452

1

271742

112561515

11111

41

12213749

3

1

133459

61721

1

38

1211

3142

223

32121

1

332211

111

1111

8

53

3310284

34

126

1

64217

1112

61311

32242

635789

864466

331476

635412

421234

23191023417

111

2131211

11

11551

12113

121

1

3135

521127

3710342443

251519123129

1113

2

235220214

11221

111621413

512238

91111

11

2

11

10210564

3

1115

8131031

152914511

42611

11

515344

11131

3134

11223

3322

2212

712335

11

191161421122270

651

1

1823443

1

11

112

22212

422814

1

5279636

1484115

9885810

327

4117

234

11221

3631

24141033

41521

10113614

8118443
141015041199838791918

2621110
2732112

112

1

11

11

142

142

11322
123322

112

14119454
241611476

3421

621

1

1

732769618483357373
81712

23319118411099134
141413112015

626116
638326

1

1

1

1

1

1

1061010423
11222

11

12

1

9121

231

531

11

1

12112
22112

1

74381113
12381012

2

4

1

21

102214
121

61

2

1

23

30161812911
432929131513

1

1

1

1

2

1

11

1

1

2

1

1

1

1

1

1

121

2

1

511

1

11

1

11

1

1

1111

1

7142935
110252

1

1

1

1

1

3

3

5111

1

44211
3221

11

2

554222
3111

11

11

11

1

1

12

1

1

1

47335

47335

22

22

1211

11

1231

8146846
243111

12

111

1111

1211

11

1

11

11

311

321

1

19113236
29207599

1

1

4

1

121

1

21

22

21

1

1

1

1

1

1

1

1

221

83523
10147578

13

12

63342

11

1

11

10552413
11

1

1222

1

1

1

1

1

3

1

1

21113

2

3615381568
191214822

141

311

11

712

21

3116

1

41

4

2

11

1

11

12

1

1

11

3033431456
1511621

1

11

2

111

16

9141

1

211

1

322

1

1

111

11

7

2

44

217121

1

122

344249562319
484576426371250236

3524121077
15159257

1

2

1

61

23

1

211

1

531

2

1

1

1

2

121

1023136
316

131

11

71

25211
141

1

1

111

234111
9194151

121

74

2

1

768838
33

3

422527

221

11

2

111
411221

1

3102

1

1221
132211

1

1

1

1

261834
15722

14

12

2

1

41

10911545

10911545
1

111

1

5123

1

12

1

1231

14112

1

21

11

1

1116
1318221

1

2

1

22

1

356111
388224

2

11

1

1

1

12

1410912
20312536203

3

1

211

1

31

11

1

1

1221

31

12

1483116

1
13171

3

16

1

1

610761
36511

11

11

4

1

1

1

1

1

1

136168411
8314214

1

11

21

3

1

11

1

1

1113

1

1

1

1

1

1

111

111

2
111321

1

1

111

11

5117453
333221

1

132211

14

11

1

1

1

12312
2

1

12

12

63313
851334

1

1

1

2

11

1

1088856
7455

3

11

2

122

2

111

232

2124
9415611

1

1

2211

1

1

7

1

99128213
555311

1

431

3121110

2

1

111

113
123

1

61312311
26621

1

1

24

11

1

1

1

3

1

11

9286

1

1

131

811

1

2

32

8126354
1717114910

1

2

1

1

21

1

1

1

11

11

1

1

12

1

11

1

11

11

1

1

13
73541

1

111

432

1

2

11322
1111

11

1

1

1

15414934
2710181197

111

1

1

111

1

1

11

1

1

2

15

1

31

2

1

2

1

211
21111

1

1

633411
532

1

11

1

4

27412471114
9157349

11

111

2

1

11

1

712

8

1

21

11

1

21

111

11

11

1

1

11

1

111

2

11

151

12

711

697434
2162

1

1

112

2

4

21

211

12

710645
516847705144

2

1

11

1

1

11

1

1

1

1

2

1

31

21

1

1

1

1

211

35111

111

1

11

3

363933654431

8986116
4315

1

4

186

26

12

31

2721
6722

1

1

21

19146582
887944745143

131

11

1111

10

171

2

5192

1

1

1

5

2

11

1

2

1111

1

1

411

1

1

11

1

2

2

11

2

11

1

1232

1

21

121

1

16212

1

1

341315322613

1

1

1

12

1

112

1111

1

1

2

1

1

11

151

1

1

1

1

2

1

1

610222

2

11

3

1

1

91533
927313

1

12

711181

1
711181

1

2

11

4117

31111512
11182091111

1

11

11

11

1

11

113

22

122

1

122

1

211

51

2

1

2

1

311

311

522331

105326
1264269

11

21233

21122

111

11111

431342
12

2

121142

1

131811162327

142412
131811162326

1243

1

22111

624476

1231

21

1411

1

1351

19

21122

1

1

6771035
13

1

113

445114

12321

153104233

153104233

11

71112

417228

112

3112

121

121

13332
18208185

1111

1

1512331

14211

246524
12

12533

12533

12111

12111

9697102

9697102

32231

647492

8712137
1

11

1112

221

1411

34

1

1111

312

554244211420
314215

1221
748654

626644
515543

1

11

1

1

45373213811
192412544

2

121

1112

1

2

7321

11

211

2

231

32

2

1

21

1231

2321

112

2121

1

111261

111261

52141
17107944

1

6523

1

911

211

14

32311
1966455

1532322

11123

579321

579321

579321

1510121033
363112

32

1

232

221

2

2

111

11

1

1

17

131310831
77731

1

2

111

11

1

121

11

4

211

16821

16821

1023211
1893311

871

1
211

21

59371
7107385

1

11

984

642219158

642219158

451843333363

1

1

1

451842333363
12222

11

11

1319162559

1

66111

1

11311

1

121

61

422

324

1

21322

41

452

135

25332

25332
221

4

1111

11

113572

631137145108158
2873462543101

1

152121

2143

222135

33

1

14

32144

111

1415

222151

1211

112

13121016

1

91

2

2231810

11

21

1

11

91

131

3541

1121

2

32121

1111

21

2

208511
67411

1

1

1

13

1646317
21

211

21215

6

231

1

11

11

1

12

438423

438423

624141

624141

51

4

11

17201711610
120167159828688

1297762

152025111510

387264323543
132329161213

4108535

1

12437

11

21

61212135

583245

21

253214

11

2421

1

1

544143

48613

24431

387122

81111612

81111612

18

511

13131

8974105

753421

5212

5211

11

1

31

11

11
616141

61631

231816121515
1

14131071010
834146

332323

133111

22

22121

856555

856555

118445

10211

16434

492417477

587211
492417477

11

410811

10

11

511131

7

64124

1

102451

102451

2573578

2573578

135101023

135101023

311

311

311

311

1408297576857
121

513

513

513

513

311

22

2461

2461

2461

2461

2431

3

1327587576856
603131242615

1

64811

11

8467510

21

11

1

11

3

671121

11

4321

23

511

1

12311

1

21113

1141

41

1

4212

124336

434111

242121

1112

14

31

2111

1

44123

1

111

1

11

21213

11211

112

1531

1142

10111321013
262119072589117217502150

574411869132179161
221

565394855128173156
422353

735560212441
11

182872513

372312

372312

15215221

15215221

13522

13522

13522

7314313

7314313

539113
7314313

252

11
472134141722

3322
3212

1

1

39721718
469315

1

11

1

341121
11

33112

12

71043711

141111

121143
321143

2

342127

116262

116262

488337793104144112
1

47832578395131109
5331

3232

3232

47032078095127107

47032078095127107
19193641023

1

1

1

3123723712
1272923

133

115213

1

11

231211

22

1

111

1

23311

1

12

431

526

21

123

21

1

356250590809550

1

2

61258281321

1011109133

1011109133

3124

351123

11

4474

1

71513465
13231

1

3331

1

2313

431

1332

11

11511497485077

37301991720
11411497474977

211

11242

5111

421

412631

2717

322

11

111

1

11

1

2

6511

1

653219

1

15313

2161

111

1

1

14213

23621

12

632

1121

115711

21

1

21

1211

11

1

1

21

11

2711

661225

2311

1

31113

21

1

11

1

1123

1

1

21211

11

1

8251

11

22

11

11

2

211

6111

111

111

222316

222316

222316

222316

21314

21314

212

212

9988019975589111054
346376

914847
13692105719099

24224

2

1324

5

243221

3314246

52562

10751125

1

211

411

1

542

1372552

1

151321191519

121

1

13212

23341

10944

31

1

132

1781123

912234

12

1

16324

7

51211

21131

1

3115

1311

8977616

456474

572552253257

14211
431121151543

9333315

6142

911134

8131

11

5434115

115

51326

141431101714

213
141431101714

1221
7132481212

211

3522

11

449452
447442

21

256326

516222

516222

113213
802680834459782892

13111351514
741638771433733834

1311

1

1

111

1

10810613180171191
31243

4641119
485868338473

182222153336

263042174028

222626153345

222626153345

352236305070

352236305070

19151791742
441369462235381442

1167188585388
28273413816

517712

1710741018
1815751019

1511

534432
433222

1121

9868517

822335

21675712

10210495

1031444
931443

11

48525

2211

129931511

129931511

263244254334

814561516

181839192818
181739192617

121

16189132015

16189132015

125154134

125154134

221919132338
12

89156525

1310471811

25241991818

25241991818

139541119

139541119

524961375758

281524132819

7965127

172531191732

14108434

14108434

1
344247273643

1319191097

255374

24123

1121

75106811

88108918

43317341712
745193

362366257

4232
42132

1

201221102016

201221102016

101015102230

101015102230

7131251310

712125139
7131251310

11

14411413168140167
41

7675923392118
342929103832

221926142245
222026142245

1

20263793241

133129107

133129107
132129107

1

553623263841

553623263841

353734422519

353734422519

151221

152316181612

4221

13115876

2163

45164173240

45164173240

17182
10830238

11

2315

12

67121

621514

621514

934241

934241

1
203632017

10233416

41131

5213

152519171615

81511171515

71081

724967444856

11912482

11912482

11912482

11912482

11912482

614055404054

8818161221
614055404054

1

73151

1034152

1

2331

1035231

7541321

75622

43444

475414

342124

115

211

211

848518544385551782
251715101617

18012010461103177

464027142638
18012010461103177

3555

21232

222243

34112

62131

910107169

144113917

2111

1522210

371

13142

322532

12443132

11974521

12115

11

21

336213

31

11

11

1

1222354

811121

884164

111

11

1112

1

1629237

221

71232

11

11

2221

22

1

11

1213524

1213524

1213524

1213524
1112

711
7111

1

1

42311

554455415055

21
455542

421111

11

2233

433536293950

433536293950

433536293950

316343

403430263547
403127223445

33412

11

11

7314773

7314773

439681
327216199148224277

285168168131190229
11211

31
4111

11

1

1698475

1698475
1

753361

753361

945113

505133214054

2425
505133214054

383014122638
342514112437

45121

61211687

456364

19101691523

19101691523

1910821422

671

21

272620182926

272620182926
644546

18201682215

32535

12211
12468866598119

2451291015

2451291015

101916112521
523833326164

1075279

134151114

4121

6277912

952797

24922161419

24922161419

161156814

44246

94414

211

311234

7312156

7312156

444311

251

194310

165211
384522112647

121573613

57763

143

1913361030

249118168118154251
12466

155591046887147

37611

21

35511

18363210
15259976286146

611832224034
1454153

111

71231

24913162320

1516356

12833

14819263
1

8718131

611131

512331272663

1912712929
1912812929

1

321123151634

1

1

7498835

7498835

1341

1341

188158159
624951395593

1121

108691521

491115

111

274328

62345

8672514

107811825

113

3441

1111

31891165

31891165
11

2668553

2321

2321

2436543

511512

21

5111

13

24123917924211947

24123917924211947
1

313534604210

313534604210

234131
273433594010

22312957368

11

1

3

1
41112

111
11

1

41

1
293434211630

1216159827
212

323518

22338

21

1

713104719
2332114

1

21

1

2

2131

1

11

1

1

81142

33511

33511

33511

1415131173
1

1662
229822

1

21

1

1

112

4311
12133351

2

1

131231

77

175165108161605

175165108161605
1

4971
385

1111

1

1121

1121

42331

42331

16615298161552
12791

4

1551498970522

61

1

3

1

65212

65212

65212

51

14212

15318884

15318884

15318884

1
15318884

10213253

10213253

514631

514631

576271382249213215
254364

467217287194168153

17687121867148

1
17687121867148

16775117796038
793040291820

62151724
621144

1432
1232

2

3311

513611

612132

11

13

121

2

21

11

1111

31338

14

32253

21441

1

121621
1111

161

1

11

81

11131

15411

825221

636132
414132

2

2

12

12

1

181

1111

42467
42477

1

111

1

2

151

7141

7141
6141

1

2104779

2104779

2013831715

2013831715

5412
2013831715

21

11

23

2243149
2243139

1

1133

13

112

32

1

311

1

2711171581048089

9342323

9342323

172104107967583
21

1511814

111131

4513

34121

34121

11
2594253

74321

16215

122

998274
21421

42111

142231

22111

756253363643
958666

1

1562

2213510

1

11

1

2

9783515

3123
3121

2

2

986
996

1

2611

211

1131

2175

11

541

1112

5

222

441

1

4

672

1

77275

3111

541

121

271026261523
1

2512

21061014
21061116

12

1324103

62383

64421

644143
3123

23

1412

10510675
1

643112

317563

1
6137322

1116111
421

11
22

11

1211

32

52121
211

3

3

1

12

2211

2211

2211

11

11

11

11

2221281273
1074991523958

13238315

112

629221

1127

15331

21

122

101832

7612

1

423224

5143

3311

11

211218

14

2113

2213

1311

757514

1

111

5412

7211

14423

250456220759662205692317344332435823435249
311192485928509373514692843920

1489255
567153

92212

91639592105179

91639592105179

223730212
61041341115

211

54382

231

87691443

87691443

87691443

7746484980121

7746484980121
20649854

564794
563694

11

332321224429

8331123

122153

444511

41711

1

213218

3211
438137233140225929248928

122
1600169910465675765883

13104224

13104224

128312
1111

1

11721

1111

111
1111

1

11111

11111

35653
1552166310205515645837

34214
206230152127137105

5122

5111

11

21161817104
632564

97932

13

1154

121

111

2211

11

36824192031
29763671128

3221

1

3211

1

1

1

1138

911858
135126889710555

2

1

32

3

1

73514503323
11

63414503323

211

1111

531
421

11

11

1

14

11

112

11

16214

5

12
112

1

232

44112

544646275012
544646275112

1

3111

1

1

5

2111

2

12

21

23

4223

1

1

2

12

1

232

1114

2111

11

11

11

51329

51329

212222

212222

212222

125217
134014258584174215729

1113

1

113

897635301322
12341032

21

21116438

1

1

1

23

73431

512

7141

192

82633

3

413274
313274

1

111

13

331

33631

611

211

1

39573111137
119113397493523925677

331311

4121

476223

3111

111

11

1

43

11

1
11

1

11

231

1

3511

111

466485259140124242
594604331174162298

10810157263343

432221

1615136312

1

2112

12

31111

951437024292556
5046313561462055352

1343

415421151716

384026173016

8411854295134

122419742

414634386

513244102931
1541925

211

34282592725

2522117112

1161497334332679

3

2311

3

131210239

2

1111
111

1

1

6112

58769331420
312562968

1

51

1111

3112

11

24
81116

61112

1121

112

3142

314

1

2

11

12211

12211

12211

12
342422141040

1324
302021111040

321

6113

1

3111

3111

231

132

113310

2

104104422

312

231

2

2

241

11

131

277820242094169023473044
423332313226

88558782759110001319
51211153

23

1

22

404560395794

211
333841253537

11

233030182324

347226

534586

7719142257

7719142257

483273415305612682
738597

512440305362

512440305362
2614121113

614432

9753717

27510487

65552014
75652114

111

1239

9810763

9810763

9810763

213
10810812071148181

172926133617

172925133616
172925123514

112

11

11

242720192832
91779457112161

27112892023

51525

10213131813
10212131813

1

112115818
313116818

11

11

5132108

17221462367

308130237192396429
16923121753

30828101435

30828101435
29827101335

111

261519139441

261519139441

12537655980151
14716141722

702434385080
682134364978

23212

4161571349

21812132120
642651486872

154752219

221223151823

62915710

1141573727
4735515012377

4177113

323029367547
311320145944

117922163

736594637185

212538252137

212538252137

142233251835
142032251835

21

73532

11
524056385048

15111218915

15111218915
13111216715

222

372943204132

372943204131

1

284192247183253452
44221

433631213257

161566830

161566830

272125152427

272125152427

252138222769

252138222769

252138222769

171821182636

171821182636

171821182636

195113155122166289
21110

1

1

891011813

891011813

162799780111209

1

1

162799680111209
157799580111209

5

1

251845304354
125522

1372191320

11919162832
11917162531

231

4243

4243

741331
7213202598

1

1055543

26211

21

27311412

1

1

1

11

1042132
457588320390454488

267506218319297365
534632

1121

1121

4968241

458139

9112

5142216

5142216

10263107

10263107

21295
871031821

221

12623

341615

553659232762
11

1

16312

33

8112811

5442

2121

12

173

311

8452125

1321

1411

12

22122610916

62211

533364418310579
1

441122

122311

42321381799674

42321381799674

69352

835185

835185

74142

74142

515125

515125

1271491019

7353415

549664

1210151043

1210151043

774474

774474

774474

121412
31259520

2844418

21

1463164
52142

122112

821

21133
6137146

1042

32

11

1111

1
443224505955

12886319

132

11

2635241
312312435236

5207414835

11

9335529

9335529

128414
382619151515

6411

122112

1

112431

21112

433112

1

14

1

222

1212

8111

1

24121

12

129528052138106

402228106636
2131

54311

1341344

87631512
1481172124

6154612

1213

923124

8364

1041256

82193

82193

22163

1111
501627211830

10153522

1

1284163

61111

11112722

955841

106121

106121

16314

16314

731610206

72105185

16521

11242412

11242412

1221

1221

13131

13131

10815111519

10815111519

4433102

4433102

3263415

3263415

326512

326512

13112

13112

13112

321115736

321115736

589212
14121

1

34811

2736524
113411

102

311

1

13131

422262
8271615553119

441927172625

631
102108610

214

32842
31442

14

1212

211

125463
11

24132

1011

1

12

11

11

1398866

1328866
1398866

7

835176

132114
533134

412

11

2122

11

136412

136412

136412

43111
31

1

1

3

1

294426311989
1

324619

324619

761144

651124

112

1261
13261620975

812512646

41257329

695451
231

11

35434

111

6106216
347171190162203360

14773906985110
211112

11111
1154752465064

2141113

52541

161251

14411

1024457

602828232739

17421

2142141

4111118

4111118

431453

331452

11

211929161220
111

510108313

476342

31111

42111

4110323

127163

1

126163

31
159767782110200

2211
3461136

4103

12133

424314

424314

12042544678161
1991411821

12210

176621021

621667

74428

72433

1621721

12166

1313

1251181

97549
97569

2

1310971326

5444

1211

6122123

31344
15179161219

2

1776

1421

1133

5412512

212

1411461510

10424102

472258

8512418
3512179744

181223

11237

212

6211

1221

111

21

1

438333378239345370

6821

6821

231

231

965881466180
427313356228333363

1

21311

1375246

125216

2

23513

111

111

2215

12115323

4105345

1

11

61131412

6231

3111

942344

110422

96112

5113

1

1121

231

1

785348

3122

2

21

1111

3367530

141155

424353

221

13231

11

1

46211

14

11

142

12

4123

613227

1121

372322

11

1111

11221

52262

1

231113

2241

23

211

3

12

1

11

111

11

32533

12

14233

12

5313

1

2

6110631

21111

12221

81121

1361

111

3213

8

22224015127

22

4211

1112

1210

511

23

433311

21125

23121

11

1

1211

412342

2311

2

12

1213

31121

211

121

138446

211

5421

186531129

373257

33662

3312

11

582410

11

1111

1

21312

2012783516

21

345111

131331712

1211

111

271818111210

243331

68221

1

11

4210622

11

2521

12231

12231

54621

54621

466575

466575

11212
412206235178233327

3677151517

261
3677151517

21213

2941748

22222

313243

2161791424

10181

10181

11
115981424

211

1211

7121119

112412
11241

2

22131

42113

42113

21369101415

5295277

3

5265277

12411

12411

21

21

342224

342224

121242

81122

412

11262321
329157199141188266

2323

2323

882233
1

813
823

1

2

73

242118165117158227
362929212322

1532

33321

5126

822231

33

93311011

125415

4298310
4398312

11

1

18

31629

111

816225

224
214

1

831510
83159

1

156671211
197671518

425

112

526234

423321

24745

74118

5122

4521

12

9643212

223

312645

21221
22221

1

5815346

1

2131

852112

1125

11

4433

4433

1745221

22

452212

232425

42446

4455

812

64132

522253

41

31723
328251

1121

521421

14426830

1

9511

9511

12
11265134

31112

4111

131
1312

2

31192

3121
662441

11

36113

38211752

11
38211752

38210742

4124214

4124214

228141211113163203
111

116111
523144294633

10920161122

11
10920161122

5415922

5311815

24514

412118123410

412118123410
78111

101121

214211

615482

71211

7654194

2412

16113673

16113673

16113673

16113673

962315740

962315740

962315740

962315740

15010313162102127
2111

582333181526
875751262953

936142

27212

211

593126

4

1

111

21

283127

1

1112

351345

614580357274

61212678
614580357274

4216549

135781519

2

11

83439

231

1

271335113131
271335103131

1

182264

136667969125311

136667969125311
14447

18189111125

1094639
18189111125

4442210

451366

117447054110279
7226373153178

11

21761311

1

5141219

2

189992248

325353

1

233

14

1211

41227

1

1131

1

31

22121

2

61

4211626
553036304243

41111

41111

1413331615
331817112827

4263

514474

13

11

2421

62214

149713119

149713119

21
149713119

3271

213341

432158

3322

1381740109160812531105
350682032927680165664021338951

638491313022
141

69241

1

5723

1

1

2

41

41

11111
537485302620

1412
20303819168

1

1

3121

1

1

111

11

2

1

1765

2522

1212

3

11

1

11

1

1

25

3

3

1

186

3422

2

1

1231

1411

1

2321
33434610911

211

1

11

21112

111

1

3621

11

2221

242

2211

2

2

11

11

1

11

10158251
141721351

42131

133

11

3

3211

22105819

22105819

22105819

22105819

148906765922256431942616830
678285391245940695

3932472713437
7895617164641380800

16215413597169204
354349274144

10542136

11

19221572618

1163467

1

36232

331511232514

25511111

612912814

633262

131393107

122

561131231

13455618
13455636

18

51263512

271731232722
322438313124

251211
241211

1

326631

383952476966

12

383952466964

735336

735336

1125245326136
57310153

8891581418

1633209208

3355127

2

746127

746127

15141131416

15141131416

1591141024

1591141024

96599160300112
355225352199878357

1121

6582345

95204239

18614102511

4261141

1

2161155611

85538410

19844135

311

311111

182020114620

94155187

21

25142872234

18815113723

38448

121211142314

4332228

91664415

4235114

1251451811

971271514

14466102

7352612189
7352612179

1

762463

21

126199187

4112

1386418
53914822

41724

12

225611

225611

9985509164921012917
352542163218

1362171615

1162071614

211

676376614350696558
246121188127226204

57131

5133

71422

10422

251152
13626137

1111485

1

216231

2134
1

1134

61

5111613

1

1

1782591621

115102

1

111

22112

2113

614135

11

11711

318167123

211

511

1

1315152

2122

824

13

2

361420142218

11

432144

131

21113

476419

2135

1

132

114231

221

33231

2

343156

211

111341

181

321432233940

123

534

141

124182

8421710

1221

228429

1

72113

2815268298

1

1

22163

147

14121

2111

35261221

1

24152793213

844101012

11

11

1872081827
1892381827

23

211

311161

453593

3

13

2

121

3226

111

127185128

8312895

146610822

61311393

2155

21

85

2

11

961630334

311

1212

1525
1421

14

3

112112

95761010
316279

641431

121162

112

1

1211

221

36174

22483

1

20101731833
31

754294

135101829

1
3152551838

74154915

241101823

9463520
492633335495

3211

124444
124446

2

914818

223274

8127121515

5247511

4253

8261417

301624102924
2

1233137

728775

1110113912

1

4211475
504656205028

454566
464566

1

193019273

1210

6410677

34272

1346165

4410854

4410854

931921516

931921516

351313193740
682742306061

107174139

1221

1

18674810

11311

31

1361381927

1361381927

476389448302501494
31241

22526141321

22526141321

2388112853

2388112853

233436175046

233436175046

272218269185320291
654649286761

3822

1

2221

113111

121

10621711

142253

382230263821

131224153129

52231

564865

1

424134

4483910

1121

41

41212

182521131931

1

2

11

4713594

71

3

111

11

69751315

291011136

115951416

536278

1271624167

14216373

11

32159

11

15125121316

343281

2112126818

1211791412

1211791412

121316101512

121316101512

47865522124

47865522124
47855522124

1

63198
511019203249

264246

66451010

43331

331111

6428513

842493

842493

39842

39842

2800876183491522812393
626254

371121156667
178114176123400359

151819153136

101214112726

251321236654

9811142815

60406022111120

17921145026

53992115

2607757164378818642011
142737487639110321191

83153109

2681241114

133251

251524193717
251424193517

12

23131

33111

63242

4343

251536243734

251536243734
211334233433

1111

1

2

111

111

6347134

63221

2113

2681291736

42174083023

276451337

13413

41

5532411

4131127

461614173327

2213

8312453

2323616

1684182158

9562623

123263

987218

3354104

312221

52353

31612111942
31612111943

1

31

2231861425

326

814132

371833133427

2122

1311

53214094126

21311

961118

123214

1122050143317

22

11511

1311

266882124

241

52444

247311104

321121

251030143022

13523

9474106

112221

321

622132173845

721542183225
651438183022

71423

1

1

111

22641
1211

1243

142471214

2441152015

102314914

10512473

1422

12482187

33644

463113

2282

3111

2413177149

5311

715318197

157174258

42412

31101491712
32101591712

11

161123

33823131912

281021223251
271021223151

11

93921219

93921219

361815162417

1223
361815162417

1331223

9124286

103384

335734

301212152336

301212152336
271012152333

2

23

1

232816248339191
582518092458149595356672

7984331827458
416382210156986370

1651957973180179
31121492921

1

11

112

138642218

32421034

1252

151087288

211

1164631

111152

172424

14

442719235640
392619225437

1112

4

3

1

53123

613593

5241

3

433138

112

21129151628

21129151628
20119151528

11

1

1721039865532133
4231331635828

62332

1

1

24143

111

11

123132

2542115

522268

421633

1113115

471613234525
471613234225

3

13

13241

1

1143

11

36394

189212
189222

1

121191

101692113

611418

315132

945194

1

43144

1

157433126
321520126257

11484

814363

861221724

2

2

672461253266
1566128

114196318

628425

12820885

234861730

506612942095125281065983
8791703091601522855

521159424152

321

32318113642

1783931410

163187159472382
66927048229930201925

91272529

8102123

21113

1674121910
2189225412020751162

3311010
33910

11

622174
8374116

1211

1

2324

112

112

11142
131

1111

1312
1311

1

11
1223

11

121

51216
512

214

1113
112

11

181742279520241114
172682197520121106

14

11

11

7571966

1

11

1

117644

2445261

1331

11

11

59273429114102
60273529119104

1152

916271

16618931

125

46620248923

81163

1

194

301123113538
291122113538

11

2117

131843373

1

392018144630
402018144630

1

299973412

1

818552423

332252

4921116

4921116

147925943527414271318
3420812120673234613046

3117

12673

116851118
115751118

11

11

123

414

6358

5235

21716

1151119

9151497

717105912

1

9245238

32572

552041610
552041710

1

117116248
9675167

1

212171

1

1

1

1411

184

1233268

2

72521119

21433

21522485123226

233

3616

1

111

2741172

11

21132

11582146

91081144

2212

1710961310

1311321

311

32445

325443

1057375

5111

242613

110124693198

15231

913

1

234862342

114112

139465

8111251358

1327221

8399117

4321112443

724997

12341135

151653210

22151

1

31157

9631913

84452514

1

411

112

94865235165116
92865234164111

2114
212

11

1

1

1

15637252953166

148722914

213513

1

4161

6421

11

438682

9112

1

1957674

732187

439597
4310597

1

11

1968113548

11216

1

15372155

313

209741314

111

41361

212

1211102110

1029412252

279571713034

23354

23

931141912

33144

9576917

84781433

113

12311373024

162

122

111

136443810

9182711

15581297

1

12474157

2193

251

93925632

4432119

113742312

173121193

231

123251

112

111313

31

1445186

21293

6436104

2131

11212

6181048935

111

236244
3563146

121102

3623

204533

1

1417

7352

2462372314

41476

1

1121

135284
236285

111

338443

102434914

2531061720

65152

192019137841

352122

17332215

101175456

40111186512

310151

645124850

422237186189

422136175881
422237186189

1128

11

34111
1013102105

1614

1121

364

11

22112

1392252

1392252

1392252

159109145102165155
3310844

8253512
113153721

3923

1

16

117789078128106
463023203819

84731216

1327
135

22

2564472
2564473

1

121

105981523

610235

9177712

11

2423710

2913125

1

1

4558

11

2191192

3241421

215541311
282530132624

31
1

1

1

1

1

1

5544118

5543118

1

6

1

2

1

1

1

27

1

2
32

1

1

1

1

2

1

1134314
11

21

21

93422

93422

1

13415310558181228
17221397139797221572712

302222272166346464
9049652784125

635779

1216107

21

102561117

66771131

8510911

788697

12898138

1617652110

158104720

13102192014

65761115

1061192242

101516111335

5881156

131371377

85123104

23312311

2111

131817132250

5318578

1135567

518147185

211117

367496

3542712

334269
3542712

113

1

110719165131209
111212192849

13998435

122572

11655810

7442632884113
7441612883111

1

11

1

11

102722122326
437296

155514

353262

3187334

21241392028

1421

20201192027

186119189

186119189

71276142
466340187654

51224
5224

1

741021424

1344

1210622411

568474

142342119
13224299

112

141310102031
83866783111148

75661112

12171823239

2741017

252426

31222
31232

1

111

1358121433

1131

141464918

44541210

1013771118

1111

331253214143390486
97071374452712001486

5447205

21472

8393720

222333

261313

936441

511162

2374113

1732

462864

3211121411

31562444

43

121163

322182

111

6870504888165

235224

622285

21611

12111

1

722535

1451241225

7155712

961412917

932321

25111

1

241

21201592234

12

1

93128

1212

111211

14742

710316116
811516116

112

611154

1

373529264549
363429264348

1

111

1

211142

113

12453134

148651316

2212

43645

10810677

247356

5832818

347751018

3332

575824

1

2561192723

1

111

65117414

342

12116

12116

43141563655

6111

14778365

1025237

2361110

135231

296111118

712661317

22144

71464810

325527

53111089

2213

234248

513146

313712

442461

62731111

13223

933164
9331165

1

1

621284

111131

312123

17321

12243

31141

351195

475

2

3534292650124

1861472417

135

262264

842135

2

466452

7349297

131231

214233

121125

3622717

41534

252728234448

252728234448

18911915296187265
13433

3911852021

3911852021

291622163131

291622163131

20101791724
4342310

41254

126117910

81719122627

81719122627

293122242442
121497716

119491019
11949919

1

387134

13

31443

51437
411849215580

12131

1

1

12112

1112

11122

19101382834
17101382834

2

1463151731

22131191137

22121191114
22131191137

123

384668314321

1014129125
384668314321

11

11

1

1

3821

3821

211
281

7

1

11

2621

1

111

7238156

111

111

1

1

222

112

353411
453421

1

1

131

141

111
13211

311

12

12421
11421

1

1

1181

11

1

111
1111

1

659301341211672965
56182274163

1
503439123126

129142913

372525102112
382525102113

11

110386229158225
311122168105360521

225962417

27816192553

11294127

1561021122

3631165296

23511

122

451815162438

642181

738464

22113

1

232817132844

423398

160797174169314
224761228

14119121222

93331525

8855818

41613181753

1221371516

113852435

4323231866117
1034377

3320191559110

6012652523

6012652523

22363584618
262344

515293
516293

1

824233212

754129

1314831810
213138150145258352

108731110
16131641817

21243

1

1

112

1111

33412

693943275496
106127135

211

62461413

311

14422526

13885211

132

710141122

17523636

7138162232
493434486392

8875810

163971219

181010202131

10118151211
1111

585262

4331258

143541135
5627414893126

44167106

1012710

4232512

19119345556

19119345556

566157

445482
1667410282151183

402332182726
31212

1511166910

11465108

1178776

227331835

227331835

175871227

175871227

151414122430

141314122329
151414122430

1111

512125
571929354844

18252127
18252126

1

1

15410201324

19121212218

1121131419

1121131419

104613124

104613124

8968694580147
1

726373

726373

368525

368525

10376119

10376119

49116119

49116119

1712951341

1712951341

192215111926
1211

135771522

5176334

24579642

24579642
24579641

1

5951112

5951112

342131201817

342131201817

23915994

141431
14331

11

22514563

11121611913

11121611913

231615162631
1

1

1

1

312342

118711012

965111117

8151281517

8151281517

323324
8151281517

124323

131111

2263

362146

39538221431

3543321916

181212
3543321916

1716333

914516

925145

4151515

4151515

1639918134556311851720
763480164551

492849284553
1

1

11

9891117

761451115

1045234

1

108126710

1229131215

15152291648
1514856121651910951616

29818111948
204468175137224

2151081971

55513121318

7944148

63141

2822437

6885119
687599

12

151391232

1759143218

203117911
2031171012

11

1042137

1042137

123865458109186
371271246192386657

16914112033

57771722

11425

4211

165381929

53641530

414127173238

62114

1

4121

22

1

112471269

9457916

142820121424

1

324213

311236213644
321236213644

1

1044384

1317782124

6672734

1012221115

212315

2132

12953523
12953623

1

511153

52541015

17121151814

34111135457100146
743377713166393463

9322

382236197838
382036197837

21

1315233

3462104

23115128
451351310

22212

141

10322516

12461611

19621151638

1083341513

1442382319

1232

1777143

193523

35152861413

23402961741

42312421

102153158

16616112131

22111

321

21

211860121520
211857111519

1

111

1

22222

26973725

403111

671255

66104910

8366

8366

7251891917

39116105

41673912

15611813167135194
584231253655

231029103443
22102993442

111

10132181710

1019109929

7833126

53211

1012125811

1111

47168

29101651130

151620201555
1

4279550
1

1133

1133

3265250

2234147

13113

11511893
221

22132

31

34112

21141

21141

1322

92311

2

2

72311
212

1

1

41

111

375334033450209332163875
395355425143

1

1

344031283190189329123564
248261284138234225

201011121814
62452

111

1

113

5213125

112

1113

421

12111
11111

1

111

111

772183011

3391134

44127177
44114177

13

6663116

6663116

2522
222

23

22222

22222

243865

243865

1761081630
22362

324325

94176

3221117

521245

521245

4954115
79929358100108

131

3104135

111123

1251

236483

524133

11322

32

212

1013691919

557355

91721

6241124

11112

2

11312

311254

69335

355222

1331

111

7137224

343

32111

121012

115

331

213242

3221

15421

21142

7274915

524318

23187

41081174
575161477672

111

123

321

314813

1

131

1

3121

6312

2163721

45157

9422117
9524138

1221

612133

143

135

63342

364417

226

3311

94164108
176174108

821

143221
263326193443

656653

415

3447310

42335

312113

3831715

294286

1247196

52523
52323

2

722173

42128713

42128713

33156

33156

16242715

16242715

1544

1544

393443212549
1

2420145316

4254515

13436

23145

11841

334333
334313

2

212141

116422

2111

2111

293265

22145

9112

11111221617

11111221617

664494

12322

562172

261317182852

1112

211231

8212610

11355312

1287613

3512914
3412914

1

8344114

8344114

1625821

1625821

1061581011

633477

4312434

116
151421182712

337452

111

477775

736685

1131

1131

8328

8328

502338445656
211574

11

62212
52111

111

1312

222

411053

2111210

1035633
1036633

1

213312

1

314271

3238

1233311
1212311

21

25222

113425

1

1

3141

1

212622

414

2111

454223

231211

22312

328239
3461571427

27275918

422

12511

12511

521562

521562

5972918

373131

2241617

312187
27111362115

1112

1042132

12413

1234176

3522351632107
315426

535266

558126

323112

2235711

10412444
10432444

2

2113

633138

15341

54221

54221

154219208
1

101104105

111162

429441

143715721271567672984
438480354188203234

1

71511417
71511416

1

12710775

11411377

32121

17

12512739

2419204823

6945525

1

1

20216101014

111

417444273633

81063156

422

275255

11

191424111668

292823121033

132

99104814

213

1

951725

14848912

391445

11884712

111

373048261814

364745291121

9764912

3635289922

1216127208

1018278559551

1

80122342227189

788321111519

15174514

857528

111

19142110610

15916473232438

12965821

21

232336

14118190384025

536643

3101141

19142081213

19142081213

234334

234334

8229745687127
648399

111111

1731282021

6111

2723

2211

11421

2256710

11332

101542
91512

13

112

23153

111111

112112

12233

324233

111311

141

815191

1313

3724

4224221

7332413

121125

2221

221211

221211

45554

45554

42112

42112

6525530

6525530

494352438289
8816132726

9510182

253419

21228

181102

26211

1121

2221

313

31131

112

4631
4641

1

1

111

14

1

5232

135

52639

1114

11226
12226

1

21262

1123

4
1111277

1124

2112

41141

351217201458
11

1671110553

19551085

13231

13231

4103231

4103231

2134

2134

111193617

111193617

534444706358
812815127

201012181419
201012181421

2

1

31232

163343

361749

43534

13

7342172

71256610

968254
1

244111

3111

61

11131

1826594
382133302427

21

31512

111

22

5221318

11

2

1

24221

111

111

1

11222

4252

1

1211

121

331252

32

1

1522112

1522112

424523
352527222633

1481091426

1715138104

129761115

31135

9875810

7673173

7563143

113

3445421

3445421

1037182
113111

711

21371

1
667127155387

667127155287

15412

15412

271747263664
1112

69342

233

589129

10384622

23151

21321

21320

113331

1

1

410532

313152

313152

21121

21121

1051192014
4548

33595

2172

1122

122

221485

221485

147266

147266

1131
1

1

121

37112

37112

1311255

105253

362

111131
8264108

12

515364

111

18141122311
221

222213

3172

77363

22422

2225

161520191917

891281310

623432

245735

1051681427

452379

12132

3712

254314

15111061328

114

2382

10883421

4211

771714126
1211

3512981

42234

221

18112116824
1

3111

1031211318

12131

23652

23111

85756874121187
57581614

11

11

12111

1

3

523131

311053

1

1

22

611211

41112102141

2211

161594121

3512

19132

2025253768103

1

452251

452151
452251

1

353252

1321

221152

1252
254625272821

622342
522342

1

1

1

32212

232

411328

461372

131

7442

1141872

122311119

122311119

1211
635310881140115

4438846011582
3931735610374

57114128

191422202433
10717141931

975652

1252

1222
1252

3

1
5121

4

111

1

353113

353113

11

11

20621183524
2221

27

921437

11

211

41911252

22

4121

1235

1

523126
42115

21

111

57625

23413

34212

6243
715740478779

10101272320

2214

533917334636

11

4533510

111259

133124

133124

32515

32515

12266

12266

1112
52153122

23241

34111

13

143

11712381

12725

155131

1058242

743132

31511

65144155

65144155

335133

32113

143

14214
25261783339

411172

33225

116921928

912234

352411

252411
352411

1

1379113
11111

21271
2271

1

5632

121232

121232

72332

72332
72322

1

236233

236233

712113

712113

629311

629311

63222

63222

1411151311
31516121822

1231

52513

1231217

34157123

1027

345553

28819141121
98708954108113

1

1033102

111

439314

64455

532359

1

1

194291817
2155102119

22131

11

1

2

115

33411313

1512

43534

6211451211

2382711
2392711

1

596377

435265

21

83121
221219191427

121

121223

32413

21112

46216

2266711

2341

75851814

42231410

326244
336244

1

211166162119180209
51215817

261010101715
122235

625589

43121

45324
44324

1

7516999
224342

311645

22812

1

2

241414101859
432113

23123

123

1054268

514728

4422214

104541211

104541211

274733182228

413229

234630162019

35310

35310

311234

311234

11
149710106

76124

6261082

8455136

11232

323283

3112

211

846056387254
2217206206

312

4137

1434

121

653482
654482

1

11216

1211

51252

21651

1

11

24221

32131

852155

1242

223432

61353118

152273
172273

2

635543397359
17412131111

565295

125

1122

411

33113

2111

512171

1211

313

51615

241

1010116811

516124

27143

133

221

111

2411

242

42211

86015132808048001055510414
204185314171323263

959013491139202
3739433554110

551241311

1036472

439599

112141

1

432322

33313

8571410

214

11129

73362

222317

31016131110

41012873

111

61351011

31131

122

589437680509744583
373348352933

332140415321
208124233153240127

2

711111126

332314

11

1581911159

713

1111

645266

161435132512

2

145122

13513865

1021141

443143

373872

115104117

221

1

1249964

6211357

91516769

45113137

2216277

1131

91

1444285

31111
31121

1

1112212

222482

25523

11345123

11131211137
190152234177260203

1112511165

855577

1561481123

223139324522

942355

839876

7310264

1481791525

1391411168

232129263621

9567919

211535204122

9121671710

10521171619

4487145

1151

347794

9712111318
11581124106135165

3610122438

812222

511

213113

322621

635216

144

77361019

825321

444634

332263

868676

211423102427

262243383018

231245

354333316650
337345

2138588

131153

4367818

12148102010

13735216

384283420201451479
771144186951402093369364

1128119298165186
602362777442727916

364126

1491510149

111

18111061930

129891615

11

7131313157

3912162

211111

527256

101010111521
111110111521

11

1

2

198204166

11

401595193394

41102133

121381132

174186611

11551

281018173364

581242323

281246222764

1

132759

337455

515213

25912161832

1078798

434152

1022591
52249

511

1

71012449

11121

1391831216

13243

322124

11

1113

141

1155467

6564910

6114

5319

3515213

12792105

201024112528

231940354027

31245

31245

1

1

1547122152

955883

16928141515

10715222348

51117171910

2059688
659688

14

13611699

9785775210879
477324401258586491

6410149

1

81631012

184871622
184871422

2

43315

59185

122513

2212

277493118

2364233
2364223

1

17371552515

222632

11131791918

43641115

19339

4102162

1053283

811131

5192187

10563153

516138

529584

328255

913467

922766

1

5718121311

51211

113

95114

14473

1148484

61311586

421

23421

262618112929

101023163225

13

3

1

112147

2431

11911586

267667
131717181113

111181255

21

281711103446

31111

35329

332351

65288

1262
2262

1

33415184254

1144

1

10141581824

10111581824
10141581824

2

1

1

1044589
1

1117

11312

921252

123129
141022121361

2115214

249224

525448

414236

151119123019

151119123019

393445446332
471219149

1126

2122

1

568142

3413

721575

754383

67119137

462263

13

1872515308
97189167

67513

3111

582031044865277669616906
245611921840100028243008

552126143462

853659224362

385357

6519272373100

1417972218
1215952218

222

691011077416076

1155764
855764

3

1431010194

341725163522

2410645

235146

311015103624

2111

521717173934

3674117

472655206679
472655206779

1

341827133439

36

1

1

46451410

5213364

1274138

5233552711051

14112782834

8111482133

193589344

31

52859

4542851

211523121321

1

564710429139210

121

11123

712132146049

23131161625

16132792919

4143414

1349361

152732245462
152932245664

222

191213548

34214

472127272020

113122136

893360186460

34361915611

20987926

28151293036

1

221121102716

51313

18101321822

1582010178

1391862723

1541071626

181615102912

2

11

33201753444

1315269

2

1652062720

111

99109141106143149
99109141106143147

2

166142518

4222

821251818

6427613510586

552253

101284109

133118166

357382

62837

25121891218

165169280242286136

96553

225

64842313

83323
62223

211

5463512

331329204225

291655144638

4825582613364

26232573924

81413111711

1944244

351729182731

241112122524

44371

231331235724

24121483239

11452118

191817192276

727232

211118101315

531

11

243017183316

117562117

8431676115192

32102692442

7111061722

196213129

473742195869

2114211978179233

21615112914

2818152017

433348205535

8434610

40923103146

97435544108124

334952417

331819154844

2959627

1111

107691831

16131692419

1256373

10122091214

146145117

12813495

533262

13202252724

623056316845

481439234665

5392512

492457256862

561432182327

18721122326

741021814

5124612781110

301529165227

1011362212

115931512

12598138

261823113164

221217152445

662047216251

1111

6850877210491
271240306208380353

881661615

5496916

1058792
1048792

1

5564147

2221

521115153

1291710187

72

2110171

7368417

1131341417

5433109

234452

219114126

2122

2112

312253
313253

1

1

11

7110343

152361

7155518

981991012

53982827

7333524

713433

4510101242

955362

43183198

3561102

3561102

1965267

543596

1

4255715

424442

512552396966

512552396966

3148117

11112317115

371325144754

2219132

2219132

1
35951953213174168

290412418535085

5211
10212074231727

111

11

9711772221526

9711772221526

223825136
211

203824133

121

111

17322233

131

2

2

159250315252552

159250315252552
341577416

11

3211

1314

1

1

296

12519694111518

222024428
221922328

1

111

1

1271

1

1

1222

531
16631

16

1

12

223
2

22

1

74222

74222

74222

517490632075

22

12

1

497290632075

3856710
486183591971

5
71017155

1

438123

26421

111

27711

27711
113

16411

7151435
11

7151334

618451
29214051351

10

1

54621

1

1
2

1

2

2711
281511

15

1

5231222

11

1

1

31

11

3

124

2

2

241
16542

14132
122

1

11

111

3212

3212

111

111

111

1731201447

251

251

1526201446

111

41

1211

241

11711

11

2411

1

599422

173

1

221

221

221

345536

345536

345536

345536

25820153128

25820153128
1

8397122

8397122

1751081926

1651071826
1751081926

111

2112
458394401284462520

711244

711244

1619971019
21231

763110

1

22119

11

342

9115478

112

23133

532312

242121

43451116

43451116

16512132027

62461316

324447
324448

1

714333

1017448

1017448

405363367253412444
17421391611

11

659819

19118132249

111111

123123

2291110

342232

113485

374687

1532

3271

2025444

1234

2222

34781211

1214

410412

613111

51116

11351

11131

2111331

111

3151810

812139149

2567575

215545

313

466495

89176820

711951711

823663

557486

941072414

108221

16884199

2

343127142028

31211

2223

2211194

1131

1

511

113

14153

2111

22

18511

275326

322122

1010831010

222

2341

322816213949

41426

3763

12651112

264324

324225

1

824571414

1

101396813

6221214

46981614

451132

11121

154143

11

206423

22416

8181049

215153

312269

14712143710

14712143710

823331

52213

3121

65911349

65911349

713250246549
376122803305228838454083

41

31

1

391629134445

117117146

117117146

2891863039

2891863039

329246252176270328
532115

241518202220

241518202220

1311
284223182529

223320172527
22281682526

5491

5631

9636814

9636814

28122692421
263180206131214260

271934254150

221820122232

148129125

12

11

4322

204523173841

222

612832182439

713642273965

12

1035141

11

468652

1111

195710931721118720041898
116541057110396

33211

33211

891110108

891110108

622835223955
914255325677

2

321

11

21

1131

42115

22

11

11

1111

131

16512269
2247

16310222

1

1

21

21

3131

21232

21232

442312

442312

411419102822

411419102822

602257429881
14414173115

12111

12520

12432

7294113

191951412
1416558

5364

3

525113

33162

12321
12341

1

1

45210
43210

2

42235
531235

111

751

594362

594362

8341574290106
28171018248

4362114

214462

415252

261119144958
115752522

1561292436

121

153123

115224

335114

28131661130
1058234

6326

41418

11

7441811
6441710

111

361928102821

36192672821
361928102821

23

281735194217

281735194217

1666656

1666656

263112
12251491215

3225811
2225811

1

7179332

5895133

5895133
5894113

12

364180247270278395
492347494954

421824454619

144121089
421824454619

281412353810

11511

11

9

7834364652119

14

12232

6112110

11

141

62212

211

2

721

8448821

33322

111

22222

1115
3

1112

643633
512532

13111

53106218
5343216

632

1538622
1638622

1

2321
11056887390104

11054887088103

5213

912410923

812822
61312

21151

27225010116132

25204910116131

2211

44111

44111

327150272199326226
734676629239

672142395453

1305911663134103

572438354631

17191882216

3

15171482115
17191582216

22111

25279211917
659937363652

1116

132159

144

9110125
64

914121

111

21

52241

10

86284311
86294311

1

21

4312

615335675391718588
117469457104108

2414117122

362758295040
352658294640

114

55322

301621224429
322224295132

263773

17181274159230144

723452318187

11

282823143224

292621134260

754087458578

25162642911

1365893124988814621762
251220232648

5614108

5614108

371324173833

1

1

26910111324

114146249
114146199

5

19895117

19895117

81219182514

81219182514

261209412199308446
462028123857

66166159

3623322

583111

34211

3322

33914174481

32665

732265255878
752767266485

14153

11214

181218142430

61

185672224

1511972139

1

26502389058102
355724610170110

97811128

322216192739

312115182539
322216192739

11112

12910111411
13313

32365
33366

11

562545
864755

3221

342322203433

342322203433
342319173131

3332

61155

61155

121924151411
11

349686

566511

12241
23341

111

25643

642761

642761

11
3671964035

918910

204851516

7221168

16588930

16588930

4321514
147781025

622336

423425

312029181730

312029181730

221413223353

221413223353

221
341418101536

164126930
163126930

1

1686456

3020181936121
323455

334101334

1265227
954215

31112

129631675

79981318

79981318

1
804151464679

16231599

12332

89105817

2532

255232

583116

3454

336332

201316111437

605074465242

605074465242
15411458

454663424734

8450333267115

8450333267115
8249333264109

2136

279118811

279118811

91321131616

91321131616

11247165
8254876711170

63381213

112368
11257

11111

71625628

1361
301827244511

301724243910
29172423389

1111

9336810

63217

1121

578871

324343

31694
31584

11

9234411

9234411

1710610178
235164181147347216

157131135107286151
167137143113296158

10686107

511732243450

16343115
16564319

121
1121

1

213

164691021

164691021

1066135

1066135

122
281521151838

1261210920

15975916

261936152944
6247685886115

5391
5291

1

11112

1241

322

817563

12114

228168

41213

2

1

132212

11

1

755131133

211131

211414

11101

11232

1212

1

7771864

362111

111

311

21

214134

243212281063126232256517
260790153977105429127233355329699524

151898291676615493699
5024242443141

654636203696
152948145143310

423

512821415

1

312118

1

92631319

41

17233832

1

12

181331844

151115

111

732244

2616

63621210

4152510

31126

14234

1

11

13

315422

1142

4713820

231

110

1951151463
4173682361854211245

28352332556

514724

121228

1134183164

6237735
5014182637213

32791215114

41417

22

22

84631435

15125210579169417
264128113650

6518528

63911417

5333434076183

3

1766423

12

154111

358141122101

8235814

6724332965184
4514181742112

311

12

11314

13220

35298

242129

9122819

611122

95431321
9435433799281

4112231444138

3

1

1

132551264
162551264

3

89831613

2063121145

83361231

5113525

322376

149529765166345
211

14319171835
1312

81671314

62127419

11219
4467172977

334282162

1114776

1
104115810

21111

5323

524246
525246

1

121

511842970167

511842970167

212343
525357

1

11

11

114

1

251911133648

1459102444

111423124

5073593093075081007
712571415

352622154285
223224

158124831

7331817

711231618

4225815

88754357112283

3218162445113

5457273366170

21

197130129150198381
231219273486

6106141318

1471162328

9151081538

25206302325

61654143

62155619

251

2635524

15826736

1521512815

112

31216

1311316

679547

22116132327

22211

715455

14677521

86315

402923202692
18011611078142243

13141062019

23328718

45172292746

1543712

52677

24213

151024101818

1113

632910

13778612

1625531016

159539682157380
3

1757122036

1757122036

16615182762
96316858115283

82811615

4311

2881682279

24914183460

16615101355
16615101656

31

461721122258

441721122258
461721122258

2

84327358111271

24727203574
84327358111271

1421358

2543413

1231

7174523

1

23123

132

2211

72

21411

2

62141239

2310211934102
2310212034103

11

1

112

212229272649

212229272649

212229272649
101720171628

84412

111258

111

33310

1

141

86765652125278

84755652124278

211

5459223339902680719313869
253273149921101118123384346580682509

2602501841489351067
294220522333177669327675

61811813
1239141015

11

62222

43753045
93768779287356

111117155441

151719123258

251214205778

221711114379

161619167155

1911862453
69647955136209

181421143747
181421153847

11

559123224

203236204073
273441224285

7252212

16510134556

16510134556

251820154351

251820154351

296122

296122

61163616
27211251833

1

5164

1

16942613

32112
212055162569

121210

42678
171653152159

131447151451

4211568
4321222212091

3819111611483
3719111611483

1

1

11

39311327161197

39311327161197

204751724
6332514486180

17520152361

262324244695

101669068655825772686
1071058476552532

24158126344

4312211693115

38162016147118

193130129116248409
208136136128261437

1

4

512

2

1

1

1131

11

736129

1

1

1

2

6215

252223197366

182

533523239891

21148745128

461827166992

623

34259

9098134329280
26745163414

1

1

221347224

212

111

3369308716

222720

4

1165

1

137

11

11

11

1

112110

12

14211

1

113

1

2532

165762245

4022162490111

77233336
89343336

1

1

2

1

58295243265123

1110945142

196875466

34212027122158

92469

12129125851

43262929109103

22118114026
221212155726

17

42

2

1

24221334221

262112149597

25147195974

311135

366

366

54462829108145
22116

371416174672

12299115153
173010116167

5111014

1741431329
148698561166285

6823303370128

6342412583128

55442528

55442528

279
5430412370102

37112592134

171914144259

320242311177845799
93759188761419301919

11

1

11110

1

932316938162109
982817145172112

4

1111

68

4123

2231137

941993624

53238

3

521827136473

21

1262021

1

8515114735

1523399

22

173112
173213

11

14224

321

12

1452681

1141145

11731

29329122324

33413111

12

631161

3514421365134

14119132753

11133282828
11134282928

11

81224

221

1

432

11

8131222726

28692146

331111330

114

132

2212192556142

1

45183094017
452031104619

5

21112

1

11

1411864571

32610511

1

16

212

1

101138

101

22

132112103181241236

7211

55715448182178
1

39563736126127

161517125650

345014711950192142687735
8425575082128

125586161104186
225910121274135529844967

144522433
82535152128178

201610104049
19139103045

12

13192

49513911

261517162767

18914112818

4021283456101
261151197175426680

4616263078182

33211687231

59618650116139

113

82314150104227

712953316770
1

1

28132291725

431530225045

151689385406221

77475845304107

74213540102114

272122282870

272122272870
272122282870

1

1

98538445108259
339203241293474775

54661411
54651411

1

311

1152

174651441

17198123549

2117

43691413

11

1727

12122

1211

173541335

17316162928

5218320

11

2

150105103165237299

11

98203141118237
2579793128353690

18811167999

151015192160

16982124

431592354124

6744182160146

131465483178253

131465483178253

313329
361614143260

149641434

196571617

275126149159296541
402128303775

276883448

2315951217

8925444795159

2620102142132

201210161013

175982026

271721173457

128454
651071214

5323710

123567394138284

5917294657114

4023323058119

241612182351

1

1

211922142867

191821132466
191821122466

1

211141

11
13617313967176

11213252950138

2336101738

2245212099259715
327201837130

294171960106

301418823103

5014402766186

8313252773190

10334524188242

100440056747511142398
95315934127196

291521244596

11

192417113279

2

31138

7243811

141011102631

521734324187

1

4221072071803741109

2

43322396190

2081273573

1

5812231747114

262613164481

1

1121

8264217

251628283362

101012103227

22

1515893841

1

29171415

106361425

32342

711

1

1

42557

72416

1111

1951051020

47839213971

171067208

159881111

164

2739163191

1

421162

121557459665815222501
125648509842966191188216730

3821222331103

301412142563

453331

4276339

19281172821

1728931616
18281031618

112

114123

88465147140264
285112189119324583

2211

712231

8378185

12113710

2124

73951615

425114526

15123061730

7687619

388211230103

1661013229

37267

58146912

11421813

2238142249

478310270309540927
5434364173133

151212171524

631191011

44713

21510101831

12223715

1126112434

386

374312173743

157851731

4436522

1517461214

13710181231

982373327

311836

21218292758

1463235

238961859

2415982616
241512122617

341

183013132321

5611511

159993418

873141928

13666813

1361031428

8735475775192

155451419

112

112

6627232265146

6627232265146

85838355747014381664
351121168195426768

281429175844
1682611347

126362437

341329193828
311129143827

3251

6466563110397
67846333116108

318721311

20214103542

444104112

70373342136157
70373542137171

215

9

24319165152

281037517126

53213812

781672838
781152638

522

105671617

75983531

331421

321101

20105233

112757

101386117

11681623

36231721107112

1

1

335

1

11

1226356

84511143

2521471618

44311010

91207716

236853979

3333043024

7131028
103235454127217

21722111651

611326296686

1426113552
1426112852

7

1291807768122259
41

302118131952

9117111339

5313027304370
5313028314372

112

331824134696

148645961165242
505145353958221128802594

1451182726

3148
3138

1

7241879

11126

31144

332223205095
271813113288

64109187

11126

72261615

467844003801202623551984
466743973797202323471980

11132

11

6

1

131

1

3221

1

1

22284

6510311320

1

2111

509302652132

2297232233

2297232233

8118275210

2418341530138
8112361376

1461041441

2115321

292917431965191033774980
6892884155368161143

533636

5214

1

2971

461825285467
461828285467

3

3421562

52516

36315194065

1491672615

2357

2

6534105

3210172841116

18914121714

22121372130

176

1

111731220

81431

112

1

1122

39423183868

31

3156

22165

415132

202841118

84311537

17634815

11354

121030

234572013

31116194432

2311246

12

32211311

22

304412164583

1457122829

913126

131

21172

11

535

7352395490208

367

865163

421212

142291325

1

1

22818131765
18818131749

31

15

1

1112

8236624

2147132

261317726123

21

219

11

125731515

422101017

22211975049

343221272282485648
3728376385108

43311

213106125128264289
2009810296248265

121

44416612

2

2

11

1

1

1

11

11

2

11

13

1

1

11

93

1

1

17

1

1

1

1

1

1

11

1

141

1

12

31352240

11

16719181721
161836183523

2

2

1114132

11

1

1

1112

315353

19522122156

122856

12

3

4

3944293649112
3731273249107

1

2

11345

253

111

13677247

13111071019

5117412579101

1

22

1

222

5122

1456

168731720

1817946

2981781933

11212

27518221757

9467187135759791401
19611285154222289

42224

13856513

941272720

5151

301138154235
2672782624

11167

338693

1

211

4241

241317244834
4425354894114

181117184475

211214

411

18451101

71561211

112

19111

3

241

1

799341377364
889743397867

12

82123

11

1

1

11

38312041637

163131

2039278914
2339278914

3

225152225117224272
222152225117224272

3

312

749356415877
749355405877

1

1

2432

273226288161
203121267154

612117

131

1624

4462156

61

11715141612

401623173151

112

15

1

1120

4539153748134

14211

1241

321144

2212

123314

22133282035
231335102335

1322

1

36464

3

1561034545

76226

827988

111

1571661520

223

9264712

24451816

1

63349

811752

24610152372

1073546

443179

4

5231

171

2861483171
131505457138297

13375
113375

1

4131

74561232

211416

11151711

112

12

413414

115

5211511

103123

101531637

12241425

8

320315

116661530

1

1631

10422228

1

422

222111

89295746109170
123850561869512602298

6440344556128

66142

8167
4166

41

43271228

1296171816

551913231938

1715222626
1714222626

1

323310360
1247415370

9241510

124741122

27313101920
27313101815

15

261322114294

2046112856

22925131132

513374

352319326341

926131819

10168833

12211544025
12201343425

126

131291236

113791323

58730152359

34143631

115595061104185

29324122455

31128114021

2046133323

582728334482

291722183474

42923236298

42712171331

94411938

1112661531

7388725

243027224244

143151117

5075183013
5075173013

1

81122104

6424245

6466529
5234317

1232212

103691611

23151694893

1434752
1434769

17

161310323739

165102389

1112681619

12246218

682811132125

1211113430

7165510
208159117157243355

122157

122157

1339877108151140
1

1

1

1339877108150139

1339877108150139

6657324381198
5226720

5648283070170

263417133377

1

1

291411173792

311

51437
572437

71

111

111

145807516539973041866742531
21672713136478591105757304436620610

8892833554179002462
29178146124285808

4812121953156

5211161631107

48219196292

13036433694478

9031409690196
8026368384179

105413617

211

152655073168357

1313141472106

6514251944162

5431431983086051248
1441061331

152435955156339

1673955156218368

210577491218510

173647383385819544220
7491873434018412003

136591645

3052183769

37412

25713143779

146406787179354

5010292644102

29718153889

161408457148309

34920184275
34920143868

447

104394134101150

2213101820

1256102043

209671620
219671823

123

161310213750

1512952548

5010122143135

3316151975167

421750214764
421750214536

228

233176

95264940103206

429232630180

826871

7162083093507872024
1

6

4021101631694251193

31497146181356831

402197270145161654551484118758
180307327396451574543262256518979

214

7531102752958561992

382166113221558419110941

141432931

206237471977620075154

33

5061071632116881323

413131754

121321123

8306148829363416904524498

126425345852712793135

373942973699921705589

507761812095141469

1

122122145400847951396834008

129222041247314113653

132

132

32485681217140632738790

6212532

198640069388720735592

22388921

5311172102646301475

102426933147011292994

11212443864499702268

549151852130

7511412913488052180

7761963074189712230

90791690334339401152826466

47229116038

120326246648812643308

7415212655149

80113146332946233672145728236922
80370146732952333764146136237444

257406192408522

530299219582221558815228

132235359355614063313
5120332466142

193548988223405

32992156144315706

200649481221605

201477583208585

34876146136373870

7565168729694248832323293
29966451159172032389874

32392119203391965

408851412113871920

2314293121244518

4325172610363

6371422093606831829

135361488

4371001452264951192

6312233595178

217487687247635

3481791081

307881432664401096

113423949358512872638

7221563303936892216

1

1

6923303044146

6823292844145
6923303044146

1121

28059107138381900

28059107138381900

2835511661294878334138164
871587870327817004911321491

355671121252631286

13711916358555

8323724

117506431319

42218181624

34596401635

6911812

1

2010914492238

1169311613

12569271632

314103939

139305265178282

654183612262263

43226716226845131384

4111320

71304181123

8331772773575861698

4021361813044911217
3961191742964771199

617781418

2812012533364

2631733499

12943249291148321

12322

413616

3641023824

1064113112464

246718392550

2317113141

29620715418604461147

581291511782135

20128352158103219

279210292762

81

371322

179445449122243

21716152176149129

55103246868197

31

22

13458282034

211136171621

101218833342610142524

436566126428627874

8416010231431

1712329353638

31

2688104639

147972910271291

41

1893178264290149325

51052

51052

51052

1

1

1

659248293252887868
119234133570844741162615535

6836595027984

5932503927179

9491185

3917361615958
153588966428232

311013217949

83314029190125

572751226898
553161305226503701

19381681824

24421173944

13341226

5871212

12

243

841141223

31718124625

1076110466116212
1065710365116206

16

411

411

27711102733

3169620

19812133831

361620275373

621223

22522154382

366650961247211131052
518212221525117429122783

493231225385

462931386059

3

762688

163731814

1

393236125365

107165218

473247348491

151645

2217137112

221812203038

1211233

1141

1141

152909882201133

15173711

3345303496149

52115228

19101853415

2178714219

14

8234553910789

277120184137316377
278120188144322378

11

1351

7

29231294630

22610113035

1

543646236265

20148202536

163646972168201

1

473438417673

2

25115071612

424284

5845454510379

284122255853
1

132612132425
11237122222

235123

151510123427

14010259676248169
61473983510379

25312116

21

1051061217

425626

1014

2429108178233
212695178233

3313

245

10

113

9143111

2102217

21531

1592

2

1356376
543737236775

372928195953

122115

31111

3612163061109
251212183466

104102612

12131

4823223593946951151
5236423685119

35271014

1

162781834

10761310

108972137

153461422

77841536

367244270313507851

1171071227

1

1

5744464182162
332168212159337572

211515102559

11

1272

47964138

9144

40815122759

24131

9956207

121311161924

271016253025

2321

36104883375

1458512

401423112265

23171653449

31279

11013811

364180190200663812
5428222110260

115808090246380

109354642158215

86374247157157

1910742426

1910742426

361944275777

361944275777

21615123653

21615123653

381016115935

381016115935

250547681181599
5261812092215441441

411011143677

4610211867169

4015323262160

61734038100186

8819293898250

23131
6933532368288

111

111

3210161138118
3512181138118

322

3418321228138

25386146128335410
5926462074107

1114

3

17313201

1961073324

6172718

62

101112102728

52331319

2

1

11

154136

11151

19131

61461531

1014924

532231287766

54731329

52241411

245

11831

13

67351433

9821

94687572144375
41211121622

555283

63461118

7942815

23251325

7825211

202751533

53991817

52371418

206991932

31517

45952128
45652128

3

2343216

46621120

337117153165422783
125155825

7435404584164

204873324

8036473496175

4415102037131

521415

7713193894169
8113203894169

41

193882959

26373721

6317323380363
291013204334

41354

15210813294

94621117
104621117

1

53814

291317193746

291317193745
291317193746

1

18867108104213435
6573203543826951292

5521192347106

301424373135

1056121824

401314161324

333

4724202267111

221212

24101553232

667101042

332216223867

743441244895

21

214510164450

649161423

4529313658122

31

211

7124303256122
7124293255122

11

372228154764

372228154764

211
12912131717

333125

565211

2

266310

1

1

89431521

89431521
69331414

2117

405114189162538740
138444961753716342212

5421120

1

21143

1

20151692441

191017112647

13

43

15616112013

3128

54211195477

12

7

40222892319

4713181576102

22121617

331012186810

11224108

512257

2

605132685103

98258187

2929141770

1911941424

86681020

1

14610122765

31514134254
31514134354

1

1

1567

3238524

755449

1

441118128

211317102023
451831195499

2451493476

1612511236

122

21

15138122

3151019

101113511

2181

1742668

21134

4533124

9334810

42311014

15131361747

451629247453

21

203484146129227

581413134059

8592414105106

2153

339241839106

2041481723

13510102283

11438
21814818797194248

17012213766138167
17112213866141168

1131

462648275072

5315613
382012194169

2013831936

1343111620
1343111520

1

452394
1563793106273514152172

28621223765
8843354353597971325

231236
5622422051120

37924112975

1491561534
17101771939

312145

162514102222

162514102222

8125504263183
20312162269

116771323

26374642

85533

161319101946

2820262631170

2820262631170

17211181830

17211181830

161045733
211256947

1213

5211

5715379

5715379

3212992250
2012881547

61

121112

1
38862117113238272

1762218183

1762218183

10222313181128
370569595220269

111351911

174571114

1261259

115215

423351

12641093515

71105819

16117

6213

81

2351053

632135

9137711

43714133118
43711133118

3

27152283037
311631719

2341451210

1218

28811132142

28811132142

321114152228

321114152228

19814144459

19814144459

36112893839
4682113

16103510

1651042226

3
353417154684

14261092633

218762048

536333527

536333527

1
301916136341

2010694416

991041925

675453625373609843
421813123832

1115206144
21

314151

22722
23722

1

41153
41151

2

2263

1266885
13711111315

3210

1132

1

2

9127584684215
337443

2411

1862011511

6718273075200

1

241124292845

241124292845

121324322

121324322

12

12

5618348

5618348

12371
562757453725

2163123

113

312243123020
412344133122

81112

11

1

8582804474184
391315400186350427

31353

152

1

1

54112
24

5272

2

38117

504540435241
494029415039

15111

1

1

111

216

333473322
122039111

211434211

12241

2
511

311

1

3174

2

1

1

73

2

237

11

111

1210
582929155687

562829135677

2

12211
211

12

122

122

14441
25841

114

114

31311

11

1
11

1

2

13

22212
643131

42111

1

1

1

735822
745822

1

1

2

1

413233

1

21131

2

2

1

1

2324
32326

1

22

109761025610070
9870100498462

7627144

1

1

12

112

1

32881028
11

214325

14863

12
542372

42116
32116

1

12111

332

332

1864141919

1864141919

2541

2521
2541

2

12
107560169255512342188

28324

28324

42469
1212671416

23113

543233

11

1413
143

1

1

221110182035
219105114104271454

854369

54641516

1451582745

1451052230

23119144477

11235103518

31913163557

233821021

1131371344

37221351228
37221351328

1

2048123174

8404735664419471712
1108410268149302

25135

127144

375278

324213

2311225

122561626

14212

262741245

9513318

233125

1053342

53361114

169154834

1

9393419

62076614

6551915

121

9171772825

442229

33463

1313692532

192022182633

131018101939

214

51

1

5111715

1336111210

173621121

1351551530

6115918

6286910

1

1031671334

11810131625

63751117

1

1231

241915101645

44169

131

5610678

41139

331911134489

237251619

2953142663

2761111635

17311181528

16468919

1

147103922

2561141232

26142

361517183837

125514

6029283045128

21714125147

233206

622121

391819144086

2

151614121821

21215

141053824

1311101

8155718

31111

1051010929

761051322

131338

112

75531215

21

21

1
21112

111

111

82235449345211752112
166683459

1224
17610412396204386

312129
6224343248117

2510791534

341325223174

1322761424

1322761424

322812132440

322812132440

3911291850117

14314715

123541215

13523103187

1031251729

1031251729

8319142731

8119141929
8319142731

282

1213962224

1213962224
1213862224

1

23311316
5082002962848061403

2317231425153

1

2317231425152

6532344056334
13

3816152327104

2616191729227

411016152876
154478291303361

1

205573234

7361213

33510111893

1388122146

7271819

52471627

11916123018

52712128

1245812627

7916353181203
264101154128419539

29611614326

523334

1071061537

251318234668

742

219311

551757

411267

142015102628

28313114

2449132553
2549132754

121

119343

19121172231
19121162231

1

5122917

6101151343

55731017
2620201843114

1

147741760

36291624

324211

2

9624484688150
21117

411023282846

411023282846

531424176087
22

13431821

2241192126

188951940

151100122150193229

2175103
151100122149193229

1111

745212

82218931

954323

516413

12611

756513

38811443855

1879102713

3923514

71085115

121623141723

186455

3314525

95510259

135

1561591910

211221

1221

311

1

1

332951152966
95161423

1158349

146

452226

21

651141012

11

45211

12

12117

2

1211

1211

217248918726342040324298954527975202642886
8282559831566418071612860058395

185121042154102611911891
23211161615

111

111

111

122317566
213529123321

73842110

152253

1111

13111

3122352157105
122138

23152582554

23152582554
21141672454

21911

868111943

868111943
457101735

411128

2111211
321519181970

181177415

2153725

10267619

161387614

161387614
10354211

5103342

1

1

6665158

6665158

5103172

5103172

17478232

17478232

193821143632
361231

117861118

5251262213

1219128716707947471130
636245203742

3192512

21

8134

111

234653

124

41

3136348

422

21333131

11268478

421118

213

6814131332

1

54712414

262156209315172348

15115181011

5113

741451113

529567

123

111242

224131

1

665225

5714511

1111012919

215723

113103815

915353

2231

354143

732

2321

1220611024

412943353542

72235

341211

1

7921

58912

12810262

11561211

1010113137

1118

41111

8347558311

89722

11

1510531

48441

11221

31

171481012137

1585151712

339543

211

21561

151

3111

15171761448

636234

2211

24107108

728885443686

11112

31

674176

335

42111

12711

11113

394215

61189523

43225

25228

7115223

8101091918

44331

270208697215462

114211

337

92116114

141278216

41331

2133622

111

33522

5312

11312

1

25222

247212

313451

9111217

12411

747132

2213

22237

48232

11

17484123

101453

412112

11

2414103713

318523

42232

33133825

63083

97272319

1

627255

411323

122131

122111

514511

24161881954
2

6971108

5562415

13255529

102215131012
2212

612712

28811810

6817353101527

6817353101527

27298358
1311

21111

51321

51321

2112

115213

461122

21

191183825

191183825

2912236342

19571921

94814121

117311

117311

467347102056
513231

41136

31384071549

31031

21011

1

1

1

17577517

17577517

22120215283166261
82295912

12259115

1

1

431313

22211

25114616

1733

1733

114211

42126
323

1123

54322

107826550100127

37342241319

12161692558
1611

681151041

52441417

4321

1

1

1

1

3321

241720152851

241720152851

41
241720152851

10555159
11567159

112

1381481341
128981341

15

217834912378184618392859
71011181224

217034772367182118232835
228186231294265311

164921327

4844

124117323
124116323

1

713717814
311212

1

11631

11

1138212

11111

41944512
334728372638

644127

121591196

142146
256248

11412

5251963
5251863

1

422

1972265115

1972265115

239149249228167487
165929481803132213192165

1

1141
11

1

13

1

21183091415
20183071415

12

21

1261611

12

7123687

1

62225

12

433419

112

42512
42612

1

191
192

1

11352

1

1411

121

3

121

1

1523545

2

1657957

2833118

1

212233
215233

3

14251

4112

11221

5135106

134

112

11

1311
16811589

144143
134143

1

211

211

33

2145

512

12

41110

1027451
1026451

1

411152

211

1

1321111

30

1492002671123740
1451972611012638

43611112
43510112

1

1

1

1

3

1487

11

1

111

5193311

12

1

92792246398221
91792246398217

1

4

1

6551639

72612725

1

12

14221
14121

1

11

12111

352923

1

47151

93542

133453

846104

21117
121117

10

218

111

11

22

491111

1

75

4271121

1

1

1132

1211

132512

1

1396

139

11

211

11

7

8050383875118

31

66151161

1

813213

5

71

49355
411357

22

916511

2

442296

1

3123

2310313

141

11

24111

5211

174269284246199178
182276305254208204

1

212

11

3

1

1

21220

235422

11

2241
224422

2212

112

1

1111

221

1

1

5853124

12

227113

4211

1

3211

11

1

11

3111

32312

43252575

5201943417395488550
24122593948

2611767209147128248
4261895321316358365

114131023

1631289715621994

1

1

1

3221

1

12811
4211812

3291

151524254321
141524254321

1

5124

371111192087
6774713

4

2744151374

9536

113638

632266

33192

111

322

1

2

121165127
21157191619

94114412

135

2118

5223210

2211

43

11132

112

15211

131318131520
363215

17761012

67522

21

1111

4318110485455
2691082714

141

1

123132

324

1

11

1

311218

21

11

783611

411

25

49

11

21

1

2

34113

1

222318151831
14512512075120202

142

52

114

3218339

141022

1219

5205549
5193539

2

11

11

130

3725559

4134384

254649
254334

12

33

11312

8765511
8645411

11

11

11

112

315422

8

654922

121

12122

4323

63761615

8455

151633132

613

123114

173253

28241
36125710

344339

1
7637318

641523

1222114

1474

1474

251066142275

1
251066142275

115107914

115107914

65731123

65731123

8494237

8494237

2835241578

2432241578
1

51313

51313

91014845
331

32121
2211

111

6810334

7275

7275

37123

37123

43

43

573314323286519801
37355

536275278267479736
472314133597

91564644

91564644

321522215637
126574879139113

552414

2312

24512174326
24412174326

1

174661114

188391413

4541

4211

2

17751394

4312

11

16416333421

16416333421
737181818

91915163

393033152340
1

9738625

1718352

111

1116512

31

1017183

2227

13245528

13245528

28798239
903030236878

29116122023

269821031

52111214

21631

71175613

71175613

1
269972867

167762364

102253

62642117

62642117

25256

25256

2945632
7437424453111

15104141924

271672

51113

132524

1125

21917131479
211117131479

2

21162610943

1212124

915249739

237863018

237863018

372727142240
6811345

101137

13

22
2214

14

18181381118
12445

1716941113

12232471929
1

51616489

51616489

77831020

77831020

22161491631

22161491631

5352623

171397108

621173
575556482258354613

221203185112155222
1431

7111414611

7111414611

618171177

618171177

9325922

9325922

894734
44435710614

232843212

11117

11661

131

6763194

1111

5752193

11516
385228182440

131610279

11

356253

8164727

744223

5104432

2838311
9556534677119

652567
652667

1

2376625

48251583333

1331610822

15533107

97751114

1595565

1595565

1
16187357

694227

694227

109312

109312

15191820641
245247232119157305

312131

312131

1042121
31343116910

7249515

2331

31

51221

232431

11011

512

1214512
13

2

234

91153

18191261423
77108654263139

7351105

51045412

1

1218122157
1218122156

1

1

3811313

65232

432323

588525

5431427

12291515744

146111
181827102331

12211

3222

11

131

2721

531

112531326

111
13242616

51111

11115

411

322

2729295817

2425204616
1925194615

511

349121

493552233438
13326118

141423121118

212

4

1
11

1

651242

275161

233

422

21

91

8221

1

423627142550

23123
393423122442

1

378153

533227

23181081427

2

33212

324218

324218

67414
4550309526

452

152

3

1321

1321

4116111
3435248420

254121

111111
241111

2

11

111

21

1

512611

101315

1

31

13

1

1

211

5463126
6624174843876471098

23281861619

11112

4139153

213

111126

62125

911323

1

411

387203223192397723
3984122

9341052

9341052

13710925

13710925

98861517

98861517

544134

544134

27379635

27379635

115655

115655

24121633360
13

13761417

1151031840

654276
371218142333

824868

2026267

3342412

391725134672
242130126110237501

273119334168
107852060

17241128218

1899141215

59617182868

5418292079197

285511

2691971137

1732351533

1336737
301220163939

1

4672111

119821420
129821521

111

815382405396

815382405396
10101311712

101221

26321

4511348

7153411

6211319

8101251434

41255

1417248

613354

101517653

105133715
714874496189

11

11

778111118

778111118

11
171514111418

10256413

7138595

1831511109

173141179

173141179

113

151122111421
1

836257

4210257

356747

461258

461236

22

5755475778137
3321

554346

554346

744862

444861
744862

31

89972223
131512132429

254

2

214

1322

86461132

86461132

5242610

5242610

8743842

8643742
8743842

11

111612191715

6125958

33215

14872

11124

382634403028

3111
231415302618

161010251212
222221

122

21113

33516

847186

4123105

32131

15121910410
361334

5416215

72251

1131511826

1131511826

1131511826

1131511826

9335396374878801505
5347106

403748232748

1055632

136124713

713145524

10711579

6635

15112592316

15112592316

15112592316

222

222

1520124938

1520124938

1520124938

418237260216446805
991181222

16510112340

16510112340

3661723
242328111748

251115

88105820
88125820

2

114941

12894412

12894412

168611515

168611515

613864
311516203660

124941036

13111313

63767
1273777

1211

14710163480

14710163480

19101472423

19101472423

30827202839

1321191511

17616111328

17616111328

2134112055
13113918

522733

321644

11
79148215

11

222142

36127172

24526129

34435

2112294

622437

622437

12952818

116369

132229

9064603886169
871225

623242

6887106

95311320

24221061016

1321671321
13135921

11324

61122210

2131165

5236

4213210

511510

21451658

31920163263

31920163263

17621321

17621321

10955615

10955615

1485142642

1485142641
1485142642

1

152010142662

152010142662

391215262191298521
24118131739

123218618

123218618

15263831

15263831

17643913

17643913

121310295
11021

83453
83443

1

34232

14442213

13242213
14442213

12

1121
201814131625

118

191714121316

778497

778497
768496

11

2
382934281758

496829

342026201549

2871021617

2871021617

492426243643
2125112

2113231

57336

21068213

712251

242145

10743185

3411385
2

17241

314144

137558

137558

2222413

2222413

94251615

94251615
84151515

111

212142

212142

118799373116209
422739274947

316694

11

1

13131

2

33310

1106

1312126

21

1

31

1

7

22129

5113720
5315820

221

266267

35

154

12

311

11

11

11

311

10325815

21

12

1

1

4523111

31

13381734

5846413

12

1

2112

1

1

11

21

391217325649

2399112430
391217325649

11

1333

823964

14

2

5122610
61232210

1116

211

10473922

10473922

10473922

8053961740721451883591432759166
11658501167984125882423823231757132502896

161523282116

161523282116

161523282116

1082555110401476199920126051601909491766
655676247840949537173143159049

14930412618083109

14930412617983109

1

34210398123304492

34210398123304492

2
224153116273300212

111

111

222152116272299212
291220143029

314417695952
304417685852

111

503936959863

11257439411268

14121030239

14121030239

75890068180150776216376981093865342610
1016252104095672069319582621569741431873

84195

24305010600227

1

11114419863710257

3

27191215343

1

121211

38

6627338

12

522928755941

371826824126

17132114637

1

21269

10171818814

1

151

16726485959761515167471937
5061022540044152233212993712802

7111845105

405431267220
374921216515

3510575

1397233263712634222293
6569092275432771137

951154812010020

337411497

31701713131

32241332452

19306343

391333916369

231211

30471933215

125684212490328

8311139715817

13142111022212741
4615536435316

4072371644117

451943715338

100251545612419
121299648113423

6821161

154081443

127545211721249

291286119354585241

8016847478117
8016947478117

1

417146333482319288

9090627712940
524134437425

384928345515

863016012021621
14259910313930928

331672814775

23131155162

471546275693732160
471551278694734162

1221

4111

11724554347527

23766516717921446
28326111147915301868573

331382711133

64129199265

10191573232797320

236194547482156
466960322436394237

15128206202
362406021283

22

19112381581

24992522305

415102121

128395105

14527915031022867
14024613630922166

53314171

9501424

471775285285590120
101326467128161148262

1052217715818667

3336619165

107122627

32125238112

187014456

25822925214
21782924154

4416

33135384182

9237369561018
9538473571028

244

1711

61041

271182856325
281263060325

1824

89262611229229
5819247924211

317014305018

16115566
301882011141

136513581

182

17891654441

2172143154

248331654423098512959136989005

248331654423098512959136989005
215291454702681511352123128628

6373115

43

24195815613115

112466

83391052
93850571122882636189

101124541226333

311072011164

22667421729623762
12126113018413438

11111

1111

21534

5131

12891323174

34

92018

424

1

521

18220

11

121

730886

105815171

5716340555314

550664

40962934315

92223737610222

111153721833221

1953306586345422

4210717426318

189122185

6993175024

213

125113

9742051393913

9742051393913

211

6804829124110319032
47736279504017524

273

261221251

1418321

11

101915833

9386164922

7466

55

276571821

21554354

21515

186604118936110

186604118936110

246490389025287
221456282125267

25341692

2699921681468519
1896951341227717

80297342482

291743514224

623511

22

1693215185
1575155184

11861

20572516720918755

305746160365614

9151

1

151442

5173461

319215254316

3712530664713

382790163337829104
1992695816255945

22571523326

5310539395216

288314197212

5421032667316
301502632648

246063498

2666528419

59722024224259

6441652184447

7518

91421246314

171424903815

1153271

792653

66411083229319

11

86302415727425

123937272408

4555355844

221212397898214147

11722491

19164

8563201

41583

1144

125125328170714955

554025516579
403023513364

151023215

261

1

22223411

513043845556

111

211111

21301936532

189315460951

27101321810

1468754

194122067013

2

521111

253321535155

145140365116110273

1

13510121138811764

50523135596928

1

501923488023

12

381123513352

328634502524

2339

1281523142

1

711785

17544015721113375

1

5322

40345414710312

1178453114

7617310850137980

133449580015593
133449580015092

51

1291444236

2446543311

225121

362416535574

774630422325

41257121

5161275394618

2742091438240115350

3

1

1

393816828

1411313182

456016128911548

313131179278397267

537217710301076

1

892131061315130

2630377218423

45372450886

11

1591351013

1537122322

124711187044415718

402332512

9269873064587

15951457

9737242
391715241644

301012171402

49478817118536

111919711

1164731310

2129470186391293
2129469186391293

1

1

451119522819
461332766575521950301662

5533446010157
7047528413064

1

232813

842885

564882

26439419143423

271624293420

355628335933430633661099
365929136000442035611153

41243

2

1

2

1

2

1

2

531237

4

1227

1116

11

31

5243

524

162

7

1

534566

2

1

1

11

1113
415341

21211

1211

117

5

15

1

71

3

12

9112395

1

1

1171417306

11

1

2

21

1

11

1

111

2

122

242813366913

1

1

1

117

1

5

2

1

2

13134

22132

2112

211

1

1

1

1

25216

1277214118917475
1146413918712674

13822481

16716204313

473248557638

554056534333

554056534333

241513133221

59712399

18549120158343165
18849123162353166

33161

34

862635398328
11115122

422225235412
361420225012

51514

17

3339111714

32301680234

634854

2231

211

1254921930510950

112

16141774

120231040663

37299187533

1

7161

20977112204221485

125722547910171736717

121135

43115

473428306734

8027311083911

15516628998315542

1

12

162151112401

1

28

2582431282127

1386811348315896

101537

6629139327479

449012211513312

462339394429

19113977213

51212811

1

1526782472713458

911817811413

20814101511

52217081

2547919944154956

891119213112

461289221303156576

222

13534304

1323107

35138820819586382

111

11

11911

51151448633

161152193

2031141003134574551

1351019204

5226741332419

1

161225393224

6232008645818525207

394746583913

1121

46242231323528

9591693

77807181313434153

17

11

3

342

375448313

254

4249458913147

261144

63681543613224

235105241288461318

132

1151

416214291510

2

363191

7050762455111

341031271254697

21

99863928489454

98461031507553

2152

884153482

80721543295266

556114656046110

3

1

1

83532141

27

624212166

18144526

1879114421

1436528929038433

3626135261201274853

554549751519

762332450853

2

183103131

2331816717202814960

7627146641

244060743684

511114195

1

153653119596

10445517929212536

11171158

58391163474113

352728477434

30828825662430399

254

25122381134

160232151110252

54118

4646141025

9388914

112384243251

19918202433

1375563

5022033105151969731

11757801453867222273936

511

1

231722115564

11

572121758323

884532

1041013296728055

2444281067599239871

90149240120014219

32410459126830131

51

111

542620595523

301531138283

7957814

12

122239609458518442

6550461757489
7250461757489

7

74551181566251

595511830511327

1695123322

88511134384645

180393312749473147

6388646

874367512676911

84471192404437

1754143

1

182415182

1

3797231998236

8159452

1331459

53371181463025

14342171

15425218720513583

115121

3711540911222

76122843822739

42697294584831

311

6131275

161510254913

6555232

21944

2107981181215116

528057199188247137

1

4131

9256822169621

1715113803
285525876614441223581083

533498673849709164

803143310941764671533
75213709991586596491

51214351212

1

323953855225
293951825024

32321

6451344

6451344

88234471

1531201334599578

134953546631327803305

1

1

231

2

21

148891832071899498274

5911010134696115

9322074506450

45237155104746113

31222

1613368

6812811

1041021194

14544623951

10278147

39231777144

54781

1

221927195010

61361

77631253314551

171517282729

5213184

25132121520

11

1

1

25810242340

9612117938710493

20164292

13321045

15611429690412098

142864117644

2

8214930301707

121284047673936356049

5488451593257

1155714

94758535

16124013117187123

1

32221568164210

3615867

17081326

12131648302717903861393

1091111853776030

114

14481110

1841032325517

3

78153352

15229167

277936410

11

121139125476

6198211

1

203886331335163101

353648234529175403

2431

3311

36618487346323853140

3328131364819

40521201848219

121

252190183537

5511336788812525

124229522610

1111

23178162212

6257764003327

3651812113712

18972689553868298921335

262422454921

62542513

2379232825

28127183233

72213332425124868

281340474414

111

399060203147185674

401729142194

7936621474060

1

1

11

22888842425634

213632782

63135

492123523577

36624534513

115128105

45

2261011713

1452132

105411243816827

3

1

915120176

77610274

22

542174

5913277022141

1

65312

101871262355363

11

1

1361581531138616722

169751639756041102724574014399

79521341476960

3125161

1801485933206615052

72328314

139119581638673

9910141415

291920443744
372226504556

8366812

3785628328841423

822165310

1327

219739434511042633693

208731169

111

2162115

346241269775251218
287191231633172169

5950381427949

5950381427949

133113273916

331432707874

2514180172152

11879276410415

741737161

18441635

1

22120

1

412114

1

1453145427343442015

5848336168

511381

11631

51371401015322

644715

486891

611110

736152245118

7999962626857

77124136393516760

1757317270125420

12555922614339

87664614025476

101

32914702924

271386459413863

211

1

2

3552225611

3411133924120

43413

1122327652122

4641

220196232125514562

102351004305644

23671174

63111

519372661316121546

4910351759333

27913131494

261217362427

2519519333544

46975536434005359474625136334
10482388443041953399215

281360370157421891
5485386142214408241

84938182

259174235602172148

404349377985527271
238232231608358176

1521541253

14171326811

1379811831013681

2631210201123293156

1541851352138537870

1292171345221326345

26515355580724672

41451381875410

178841082257896

1

17115757193330540
15914355286028035

111

13232

11101670243

2541187236910551673834
224113703489251589533

30502211308431

302825563362

8930728038358610

2445394153409

122163146092929355
123166147194429555

1311152

531142264887169663
493136242828168262

3862259141

4930188286494

106868430013255
112868730413458

63423

1371557553700233114

112105111763712633

229159679104035648
18612558895024744

433491901094

40580325522099929561322654328
41551333162290734715334614599

2315432403

11726

19552976

1213362

1118329111561434

19

2617351032111

26187

131066125314

251249505165441932

85504410313410

9364710121817224

132728259307

66559

13631727

70101456493

564140452740

1771760125

1

17017711317785115

1712821446319740160
1452531374295435849

2629722434311

158119169113010925

62361172187814

9044135262936

51231612403624

23551

321

1013624521

60437220610181

2

161945321214

18484138236228189

273248644104222762

3982518332226172384879
3367435309424631854432

6158322815453447

421318

983525681495

111031

1

634223121949

4821852

58483427

3950991547919

2773911

131

276811

14451335061495799128982076
1938589191544133

12180197951135188
12181208961138189

111131

3526165210514
3425165210514

11

281614317189

20813493271420146
20813493271427146

7

114243687204044
112233684203943

21311

1339928585589480567201370
1375229845693507185731461

11533244960732

511241

11221022

198101414912

1

631222

28435497

2333152

132213

51127

1

4

412

5125196117012

11514

4529265231716

3310

120

9111099

10762478

5

254926959

18826832629

91911492547850

232318403334

6619504271

1889514327720493

521343262

3528103

6912501912812

1

47142912975119

50620435619

1111

214921

41136294118

12710911952

2323471152810

1

1

53496815264721

4261745826523539

1468916438917332

3611

106651343747689
106651343747690

1

1

1111

204462245277178116

3131004

41113

15610121426

12

1558121426

1724281281623

74991

801561601494074

3

613314

1314980247

1093236116810349

535327304839

2241

5512452472314

1281232586948962

1336811915517391

922744633

1915341283017

213892

2

1851731055124737547

1

62352

333046455160

2017829237

4333922636426

31131127554

54312401

178614855541764

189190143275223153
185190142972922953

43232
3

13232

19526628521761196

3445112

1

59432383337114

727452786657521

1111

2

257959699135

8659466726649139924

2171

6681551

431013213757632

1699282026

1392014711

2078317724613694

2

1

79339034197

134063682413

441713443937

1

8557872617158

303399985023

106513912897102

282137563344

197242

49410242

311024313246

1

1

4858116171648

8713376935971

532323444349

104419114211480

726931

1712615135

12

11

4

131356242395

12

11

4722706362194567219

11

25432

45132740115110

173029642922

274291

48182376145

211515253112

60076968230293152

1561042321

92211

618215631311634

15319825089312235

99671134927438

64741523678640

432264361831549119

1212

84155735

221

511224

15121

14

222

49194157

4122

1

70601068510434

6812651411

11541

23353070179

1

991556

71141444

334362742087

1881211619

7

397123250403103254

1

282225722725

1

106669439544071206554
3638204504937

16141422147
201614332410

4211103

306741440

22729815557459287
22829815557459287

1

95219361

45413
7433311973327491379

202161311
19516811

75

5373251913310489375
4813071833259451364

11

111

36

151

1

632

4

17116372910

17

114

3

1

7182231

62

140761212865040

251489113644

1

512

583223414826

21054974919570

341667316458814

991662858558383

89818119312954

21314398243

25742816614

44083519148164405

46811

1

1

502023413541

1

2433136

86128

637513224910857

1

242226303839

1

4311

34

4941641117346

1341241337080018346

1

55322296364541987105

1115321212

21111301016163

1

3971258381222

9121318

111

252034152069

11

93721022594428

211353

4158165352353

21132758159

75282751104932

150511152808493

3551

18760331204614

1346533141891478

20716368

211

312

200160296110011288

1115251177

49247594110848903147

21954182613

2

2471115273

7

672135313620

116506627212436

131332172

2979121

112

11

103811809

1

301714412611

974911341709797

33313

354612812837610

57831128810179

21452226

2537382644556

2

135272049

239117522013

1

1

1381

21

159124651684812

2

11423611107

601471202248161

7125229

1

1830132037

3844173

555

9211995

173225156

1418

11

20642011551295229298

12528275301

1

33181613373026

16811252924

32276410

787202722

787202722

1

1

646022116789222

646022116789222
11535

181

11

428638

1

1

237

939161311

231

14231

11172

3738324340170

1112654830

19182913104110117
26762155424436181164118381725

6512
483036213018

176229133

251913101715

39212771389135
19762302614

10595463110

109120623411

21551822420708178009114571392
4754403152911408408945346

11083

1297

23872211

195511

90312

2267966

164212

23

1441

317825645

1052023

147321

1482124

3247341

11658223

218637

110424

329461

11138583

21526351

12061074124304964691565
231624528454560

2711504432

15

2344253

1

331611882673121
331611392473120

6

3819

2

1

211

1

1694

1

1

9

1

1426

21534

523

3

2403

4451731781121
4451811791221

31

31

1

1

1

4

11

1

4

1

137711

1

141

351

2012

11

4

432

4

91

9312

3

5815711241318
5815001141318

2

10

152

2

428

121

111

34

24165

1069102458572856569438
98393743712291359383

1

2

2

5

1

312

1145191

16

3141181

5521467125

1

6

62

621

2

1

6

232

5

3

142

1

21

1

110

4

276151

1

2

82

39121

139

1

4

9

1

2

153

721

136

57561661428414

123

1

1

1

2

44921714

1

151

3

321

1

111

111844841

1

21

1

2

3945

2

2

8

1221

2613

1

26

1

1

3

1

1

3

21

12

2

1

3521

2

221

192

411

1

7

11

1

79225

4

2

1

1

6

1

102

224

42361113

10139143679

1

811

1114

1

1331

1

1

1

2

241

31

792691

22

1

7211

12

4012

23077

33111

2442

462147106393
26199482383

111

536

3

286171

15215

1493211

141

1289715

16197827034
16190825434

204

5012

116561111

672407172
672266352

1482

1356
1203

153

13248518

1

598196992

53215051

20101

51616601169114

131045171

3818

17456

784337932

137141

2767616

1

45193217
43886217

61

76

52117827731

2

2

70122

54145545655

42021527155916

3131617412
3144638613

132121

76301
5318119613830

531836303730

71

5118

148212

285752

1153121

71260892514
6214884432

1302

1271756

21111

52

13311

27

3883

60323

1413811

275

18076

97293

77473

21211

7514143816

127857873417

21991782

24110

41221467331113
461984674713

137

63922

94381

1361

36

13128

2152122166

2797378031

1

213935945

1212518

15627191

218130441

302168112
302238322

5

111

11

13823431

4128586726

43188

623067125
6125875126

12881

4

219058

441425288111
441406287111

191

1993332319
311031362201

61281

123271

54

10211

2273311

45175621313

1912153

8115

161

22489210

2621815

54235263713
54222260713

133

123682229

123813691

10464

1132495171

15422230

1114733

1

148121

3128187

22

25237811112
25238831112

11

1

22

11109459
83058723125

72947818616

26

867190

61223652

1748

452641

21214256711
21253281711

3925

4122030

12123628

431195245

436642

115913391

6139413116
6137612013

41

3

21

1272

2115

953433
963433

1

911032

976051

862581921615
862741921915

61

1

92

10621

641515

283544

517876111

12582189

461647413

411046766

2207315223

16881352

532281

7616914177
7620115297

25101

711

2391

2

1029723528

12219283

1035314661

8

345591203

42171

13

135942

28141

21105110191

119

1214236751

2235217141

153298

11656401

5026

119

4

601010

1

155371

32273726851
82328032961

3410

2441

9

5256501

10414

3144252226

282

213834359
213975269

1491

536

211113

812062

327639127

335290

119826162

23355466412
23375483412

2017

2115211

315111034

414264104
410139104

4125

191

31937244

25

641382753

134830603

132

421547572

124153710

1997959

1997959

1291461721436080
323035451314

1313

1311

24322

21

446042201827

6211
8221

21

4452

121713541

611512

3212132

3112

43

34221636

952

1421

411441

444422

111

5111

413422

2

119411357604571
267109323180394174

91982813

51

161865863

172

1186

42846

23822

1215

115

1

1151

1

11711

13533

312152

31421

114

312331

422

12

24

12312

23

613511

323187921

1

2118222

1141

1511
1513

2

116

12

77461

1

6

321151

1892

3

112

118

62217321

251

651

4141512
414151

2

239335

6111

1

4

6
36

3

12371

10182
759726443266409582

563448313205332465
483830173431

29111

141

31

121

545124222842
438342252174262403

4314112

52776106

52322

26112

111113

23325

722

21134

62136310

5283310

22213

131

411589

9931495

65111

43615

1341

121

238124

85534

5423
121

4222

1

213

112

11

335159
4361510

111

33142

50433152223

12232
121232

1

43693

11

11

19231

114112

223452

14261232
14222

28

243

32111

13133

5121

13525

9945619

52514

236512

14221

8487854596145
8383774594142

13223

16

121

12112
1212

1

12112

755930143530
584020132921

711545

1085124

2122111
168231112445983

5657271597

2184112

3115233

5731

1093912

1317421

45812

651235011015

601154911014

5811

72112262

214714

575122

291721253256

201513172651

928865

9112

9112

182916171834

6831117

598221

12192

610451316

5289618258503068023871743349422
2849201209122906904215404432561723117

245620529

1

1

245619529

245619529

46961224276259051158434152
19258031343770152527742860901252948

282741373180
95111144115179245

92230113340

1757

101131124041

131524321732

23189
43441618

24389

302614143534

373
164850481441382120150

8121

8121

163750291437382120150
151746491311369110135

1

1

4321

14

14

3

1

828121

21211

11

1811

2437

1

1

1

22

33

1121

151

186

613611

231

391

292

411

16111

261

1

111

295

28

12

1154

172

6419065433

2321

121

23

61

222822921136

222822921136

17965420440374732378871015671200030
1599121697231569212302820551037570

16772428152

1

45127324713

1

1

41284358115841

1601310104110430

20412163081753206711017
1528156246135818138713

51360623952542304

262737326596

54163421154312

24826729647

26601231757115

128801519242116827717484794

119811428225315475673178919
128801519242116827717484794

2553250303942341

19018365131621171

45341825361872318

145

237258165422723558

59279541433473
703110349453564

53551788

53551788

613333

50555641198167

21142

223207756196

67812221

48396354116231

11

41310101055

2217442079151

4540454475237

2243145129516387

20194378983018187913145
18214088572774176512035

12861467

7310128831440

109181812867582

1171

3135121

7965293035914

431932108913068
6126441281093167

18712201899

851311582

32420511143126

10320311136142234

386386531783298

24223462901042158

34499434184772

13

13

58921631622192

923523593

43781854101

4251256124

223163821273

21451123392

361531945719080

4524554575510

1545638445

20717612011977

496319876195219

45176020115362

33825492411041480

21436901865893043

2634452567703

467172353107

14197727161105

3926144171

18242127295

21

756244521055922

1

20213617580

2118201711489

661641

27107515

13919415632003574

5618177999

5618177999

14566874356353189992374
6409431135096846717261018226

6293225714901293

2111405148563
3317546380624

31374

9511122557

1256159735

9

146863999482

114639

39151758968975391468

2244223149168

6915933387849226

1

13

4

2811521411749162
2896551512070166

853103214

71057227501119

6372170303075293
7980178403325371

4431316

13311

1

131111

61225

1236

1

31337

3

2

171029185284

1

234815970269171

7132567825

377532166

1565511469145164

7153482827

8355178149582
685216496166

1531453416

15104112112160

9211049219314361494251650
9051044217174291478921592

1652147153358

2525103353461162
2927104363591183

42111321

406422355218149

44103961201281611149

11

16681575659703153662652112797
3459756476142936375067585888451

523611
7016149563

16511412

1

27461434

2241151
162761

6149

7605821144157

7605821144157
6563712129153

14205154

14

74540432647

126

74438432047

42137112103165279
1

36827685131231

65536173448
65532173444

44

1655292976522471725

1655292976522471725
1544283373832380707

111962699118

273918902366160248257408
9626238528225012502

127

2

212

1112

4

72713823

1

72613823

71433246

20

274453106117375

54547686218341
54537186190194

1

1421

6

1147

11

21

1

76646252466

1221

55519677284307
55519669267305

114

3

7

1

1

25

1

125

1131

2

16

2111

4144755258124
383847354453

23158640

13139831

8

34

1

4

22991116118677111442530
23561152127085113102842

2128

15572333103

1

4

113

1

13

1112

7111

41132
22

1112

2

2

1

3

22141

214

63134

11

1

1111

4

222116

5

9241

2224

1

11

1

1

12

1

52

21133

567

122133
2220393776122

1

1

6

1

151

321125

11113

3112

1

211

1

1510262548114

1

11

3383

1

1

1

1

1

1344342

46

1384

8520032493195596
8620333493198597

2931

1

11

11

25885010204567351667
24176949271191

179401482189348918
106647173739

1191

1121

11

2102382

14

851

113449451471
16111434

111838311167

22

13112

21492

211

935

1

14143

9944

1431

13015126976180113
9814325369176112

11

1

91

11

328571

3233222

8379184550

17493121

2

361331013114
361331313114

3

11

288410

5881266755128
5881266756128

1

4751022

2

19792

362

221191510

221191510

312226

3317

1

42

1111

2736

4472319

11642193

73346103019
154585266837

81239163818

1

16114109
16114119

1

116345215130
116348215230

31

3128216

414231

2922145424

1

1510

44

2511

1
4176111310

4166111310
1166598

3642

152246324021
1

152246323820

11
1

1

91236815326

91236815326

22228111444
4010416996135205

3193641711

51042352035
51042352135

1

6172672616

12

233434395599
233635395799

11

11

1

24512832783101138101
4298854242859

16480431814658223

62031017

2

420959162

3122612412
381362451362712548

114225241633

381101234

10516189183685

1431262937214
1328212829207

135187

2937614486167
91430192351

141417173839

691482577

12253
19312823013804682373410635

28356459135117107966
642672821331854913

48642513

131615151773

111

76118754

5351343491602

106736261811

61

38280480572

2

421232329235

101023191147

26283018701671

8851674947256

1122

11

121117

142314245
1901776641320833120242666

17188125

17188125

8422655138

8422655138

2032106815

2032106815

1861265821289530119782587
1555512822229

1

1758552681198224318272418

1758552681198224318272418
1720651591172523517692346

73235451632

3068620334240

15286114

847119377153123122

847119377153123122
443451387377671

8519429

396737365124542

93882514

1745923

1745923

11

1

1

2042115210

2042115210

364
1

22

143

165315222

165315222

214636186132330

3313034238

3313034238

181506152112022
276517233

256219142

922828151012
1

6016951268

3211230344

33

391

257822334

1

979

121456157332678
622911872211885803

921192449727
922242449740

1513

4711414168
475414164

64

4412551321231
4412751361244

2413

1015271820274
1015261818264

1210

2361366635679
2361376736699

11120

136169266152213483
3695119

118138228125156326

1

112

1

111

3233109
35431110

11

11

2

2

216247
34951214

1112

2121

1

26

1

31

5117914103
914151422121

10

1

1

11

1
121

21

11111

1

1

1

1

231

1

12324

1

318223

318223
318213

1

103213677
15110997137310

21913913125
219142013170

11145

359724517133

3131512827
26728510489209

1239536246143

112017303539

2182014963595781239
4655108112146145

81724134733
172641227456

491772415

5238

2643182444
131657233452

91012487

22121

1168151527
1561054

101251023

715161544

9854206138254916
413514046150640

31

21

3541

5014548488246

21224

5441123

9722171910
772011139

22661

151331132712
172238173229

341

2634416

1

1
2444419266128

2443419265128

1

7841126632933160
4321254

531559281342885

21236534157221

8379891724108415584018
65566305676

8279241639103714803862
3574857344178412709

152393285763
4504318926185961075

126953343633118
11486318151635

2

1
11

1

1

12916211481

3213
531661314

1

11

1

1

12216

63

112

1

11

11

11

2

1

2

11

1

11

1

1212

1212

1

128

114

4

1

1
1111

11

1

123

123

1

1

136189216368229263
136189216367229263

1

1

221

221

1

2
7124535

12

4941

3221

2

1

1

1

42551018

42451018

1

1

11
1

1

1

1

11151
12151

1

12

12

11

61

61

1

1

1

1

131

243243
243223

2

1
13

12

1

1

111

111

1

441031
441021

1

1

42

1

41

459
4691

11

1

13311

1

1

1

12

22

3

5

17312418
17312419

1

114

114

1

1

111

111

24225

15

15

11
12

1

42724
541461910

1

1

241

1

1

1

542

1

1

1

11

12

1

1

51

618

14

514

1141
114

1

124
13

11

1

2244

2244

423244454483

413243444478

1115

446131887
3415354659249

1

1

22925292990

1

31

11

4

1

1

122315

321

13

211

247

1

1

115
159

14

3

281753324255
281853324356

111

1
21213

21211

1

11

11

111
1121

2

1

1

219

219

14445
1

3221

1

11114

92415847
937151048

1211

11

11332

11332

1

1

21

21

21

1

1

1

653
22158

1

1

145

2

2

14313
2081121914

11165
11265

1

27

27

548102

5

3
5

2

41019172280
4919171779

2

1

1

31

466177203116822601247
234771165911918953179

23139736249353568
23140437149359968

7964

4

2

2143

121

121

67634173531
34315160142174133

88947443526

117443485846

26425192620
26025192319

431

51098182
71211142010

222628

103131232944

102930202643
103131232944

21331

782

782
772

1

937657773023613307
13954149168962994563327674

1111

3224

231921
111011

1291

1

2133

1111

5116635

274613929951

14112

4419191739

5541036

29171512262

73028273023

1162132

13342

2323134

82275100

141215369

142769932257

3

633

2

119

1

409571071055245

236

2442

66

2228117149

1

1514202

1

12116417285

11632

13319161915

15111

2

12752919

11497

814057483318451011
7552276117162212474322105

43

673

238812761268888
3210917963298907

17272182
11

7272181

2371

81222516

975979977865

114

1010436264

2216

1

1

3831

1

9

11992

21

11

221

1141612

1384310

1

379974154155326652252
360868126951524812108

2

3

4221165

21121

25

2137

776123

6

23111918
151

381

131317

21

21

321

12812043013880

3311

281528102
25152892

1

3

114

41

2

70801423710512103
70831533710512103

311

1218

1

13

1

7335

11

3146666

2143

11

7185208631962275
4151136591702272

334724263

716201827109
820502279122

11

1

221

14262371

1312

21221

1

21

392

623

111

13571

1

2132

4936886409763012199

264110

11

1

1173

11

44

22111

11

11

421

2

1221

22152

12

1

1111

249183314642

6631377285248

12

1

1

1111

1017445110266

3371330

14555

22

56152205154402694
429202917107

7272230153171

14141561810

16681258
1538820

111

1

2238

212342010686

42224131210

146216470

1224

425352431116

5926184566

76186546714

76186546714
76186546713

1

7295108817104551546637195
1848513555181

12

21

311

1

1

1

172113

63610212488542260

121

41911241635
215519635

4457

223

111414111767
111416131968

121

21

1

1

21

7022105445405263236335970
7254107545995295440256643

1

1311

1

1

1

4

2

1

3723121116
3623121114

1

1

1

1

1

1

131251114

11

4

11

123

1

2

11

1

1

1

186

3

1

1

2

2411311

17

1

1221155

1

1

111

1

1

4111

15

333031735920

1

2335

2

1

1

9

1

1

11

16

4

21

2

2

2

1

1

13

1

1

174112

1

1

21115

1

1

2

21

1

2

11

11

2

21

1

1

1

1

11

6

33111

2121

11

1

5122

11214

9

1

11

1

1

1116

2115610

1

50471523511

4143151124

1

1

1

11118

2

3

11

62416291210

1

2

1

1

1

1

1

1111

1

13

1

1

1

1

1

4

11

1

1

3

15

2

6171217

1

12

11

513315

1

11

6

1

1

2

1

121

1

12311

1

2

11

1

26143615123

5

1

4

61119448

1

1

21

2

114

215

151

2519

1

1

2

6545117437282

232164

3

1

1

116

1

1

1

1

1

81712941

1

124

815219

1

1

1

1

33211

1

1

14

1

1

1

1

1

11

2

1

1

2

1076900100876614355888
41277056146232

7591011134

7591011134

6971514321
3733344055910

364510169

12

213131525126
214131626126

11

1

11184

1113

1113

53412195
713833195

210411

1

555217299

555217299

143100124863971431
22172218115216

3216361684338
3517381688363

42

1

1

3223

123

31

66524034102288
66544135103289

1

11

111

1811181463513
1811181261511

222

21532850

72104953
6045814895682

4340463467434

102241018195

111

2992782923184641328
83778410092197

13349

87812114

112749

143254

376123925

52112110

347112121

232537

3432325270230
5849487489311

22

212324

13

2115915851

1

117324

1

913563388

351310172793
31128132184

412469

9867923

344

291374

112353

4133

2114365

3323767
132313111794

10201181027

7129325

31132917

45233

21334522

38222221
69342230

31129

2261128

213711912

2852081874
35822112397

11

13

3

123119

1124

311

153207216174181740
271213202285

12

2530437752230
97222220188

162321553242

1015115426
715104410

31116

297226448

614211310289
2548753442335

193454213246

6610346
106115516

412110

171525132819
161420122119

15

117

3438814

7478167
7478147

2

451711109
3312001773269132

754366133533

171458325

33732413

1615371112062

2511183599114
69381099382111623397

1711851705189209
141217202333

12221382542

85210

415567

3156428

33773841430

80396951127
92447861235

1259118

2491341
2491441

1

354133

6346685176136
2413083974184411357

1211145
1210145

1

647191114160

144178205284270795
145187213306296845

12513

21444

6415213
6215143

27

112

113

85221126136

171556321820
171455311316

1154

1

1685652

3512814959164194
382261

1

1611111938114102

16928184491

55306260138127
161020153247

1261342918

691115144

616113735

15412152623

1661481971982311396
4565618281669

11354442

41

1

1

61012161548
71013161753

1125

1110

227255

132113152876
122113142172

1174

75883522
8710113929

112347

1

4921

428112919208
4210133224216

22358

2421402519250
2421402519247

3

21

88148129
981481214

15

1

497712038128359

2339872494304

2339872494304

113016458
263833143455

235415
1571591446

1341051341

121151

1381502122673051007
1012155016112

7381726

7381726
7281626

11

264831193176
919799108187558

13449

5118547139
715101756145

2421296

51121930111
1325291634137

3

12227

18

7126448

427254661176
447255062188

24112

11123
1

11122

30389010895311
15246629

2021366955198
2623447763224

6137825

1511

112

341

3618242358
2618242258

11

2838127291797949083643121459
48323405911164044691015

71733373260

61732353045
71733373260

112215

36771260

36771260

341019047510995

34998946110895
341019047510995

21141

444486810109324543346
1242003034347041218

4313427151

443249120162118
443252122166123

3245

2152219

19133138510303
19133038510303

1

38929192542
37927172542

122

8917352779
6254108114220287

132162

729422512764
730432612765

1

1

11

23428

9719232487
2312425142141

11

12

1

1141523949
1141522949

1

1

131

21

11

111

1

1

1

2

1

1

1

1

2212

1

12

4141043109

14436266123110
14436568124110

321

242433254919
232132224717

131322

1921363954105
2522374961134

61110729

705784167437695
706384171441713

1

1

215

215

224

1

124

123

81224212631
81225212837

2

124

123

2839656

412344

163258
163256

2

139161331731421916867
2763109918626323503700

11304627611155

1

59253527923

42403242324480166

40421290369583275
43432329382984287

31037125111

1291

2

36538534341126722

11111

2

187911
32439596156218

1213303162120

17517234819

32440353768

14941002573995023278
1160638915354477258015754

724414246

31117231647

2812192853595662844
2742152763425572696

21

19

3221214
311111

111

13

133

2

152

42515429

1

220796293

1036161210

16121074

1

212533453581

11

318132111

213317193734

3

594363832901636
624468862941655

312318

14111

3

745261734661

1

1374373

1411

1

1

14843527137102
20914652170185

1

6611253383

12619293115120605
170241129132153664

2051645

23138

24442011216

6

204529255370

11

1468847

1

1

2639505563187
1423393051114

121611251273

228360110931978
2493641151002089

4241

33325

2211105

1

7715221918

28303215322095

13319125028

1161

1515233142936240308

1

4333419
6559736

2226317

33165221

13

12

2

11

1

37519272478584
41552272579876

43311292

12110

32431791641

32431791641

22410712
367195295162100170

413267562716

1298171569

292150210825783

2184325
291131014799157

165231146477

165231146477

71237121132

61137101132
71237121132

112

43129182243

43129182243

21
1002944109310628861147

999943109210618861146
96121663837106

627362

627362

161382216

161382216

381350461595441517
39281452450

111

34

28122

432

1

113

32724
32524

2

11

1837201772

2

1

1

682

2

623

1

7233313
6231310

123

1

1

4

298249406565392445
297248402564392443

1

21

1

1

111

58562
183128131413

185242

27712

1

3103

937337
937437

1

2612751324
141

1

126

671122
119651318

52541116

42559

42559

4813592
48323

9261
11

826

111

442

442

483112

27212

27212

2111

225311

224111

12

44533512930
52428

111

9115323

11

81231

85334

92017332

41439
414311

2

210519541

210519541

11

1

1

21

21

6121
159101236159145158

12

12

111

15199227155141155
231

1259118213210760

1

24542233395
24544233395

2

115121

889010113415889

2415852025
889010113415889

61759212913863

1

11

2

967457233351
111

11812
1112

8

622

574332172628
704438192632

131624

1122

1322

111

385133

81131

6103121

1

1

2553
1935377180

11

624283167
624293169

12

106237

11

143455
2927148115

3

26611

1

7311

1111

1

11

1

412121

12

1111

1111

157208205118205374
1

141199189107198354
110124

92896831

92896831

343440163761
2212

1191571538

21232592021
21232592121

1

171716113528

171716113528

72011121442
4171733678138

6411759

81272182

10252772764

10101781421

17142315

17142315

2
384227353577

202914131730

161313221847

1691511720

1691511720

1691511720

229017511886195225672458
363440574142

572526243524

311213181913
572526243524

11

1

11

41

1891261310

111

1

12

945910912392106

114146510

114146510

81514494
8355951178796

412513

8774922

221529862723

1238596

4333721

12516473

10493115

327379

353639203453

353639203453
122117101021

483477

648

2423

116122

618449

415153

1011891411
506357424401542571

423448162026
7126243

8212417

13115121

4434102

105225313

251316101429
533416

233

139124710

5143

117
177841221

4251511

1253363

1357231230

4233721

11

9341958
9341858

1

1251153418

1251153418

108991139110499
305334293251

35821

212

11221

12112756

74994
274994

2

342273

121183

215343

3331

12

131114

151

1238

8811433
8811423

1

844636

32723

325114

911141067

111
104894119

1223612

8165357

2312
4753185

222261

23122

13710101010

757889

623221

231412101439

231412101439

6122051212

6122051212

252125213936
2

863242

3251110

9121831520

1

5221174

113726813191114
1466111311

224611910

2620192856

975396

735131019
535131018

21

21067710

435101312

12105331725

634817

1167778

431661016

431661016

511147

511147

4131
322826284124

313451

515571712

14106663

621111107

4222
40620144247

13336910

1431

2211353035

1
4825108

21

241214

221383

644442274184

2021961631

2021961631

264132
442333212553

1323106

107155220

19811151025
1979141025

121

171018222645

7328614

7328614

10716142031
1

21363

65771324

24171

122

145128159101182167

211
91178146

3457134

46212

677489367875
12011313985154148

1241

1396191719

12

22

21

43414513

52127

61

1111

322242

22

1

21

1

32212215
31212215

1

111

254555

101110231

112

112

2211

314265

1

1

1641381413

1641381413

111

1

11

463128444562
13041031986114815061345

9475123
632421276979

10245538

1023612

1

311455

1611661510

156132611

114321614
40921274953

211413

16378188

10551114

1158314

48923111922

82541

82541
511

21

11

12

1811

1811

21

131178519

131178519

184012321

184012321

184012321
174012321

1

916812

916812

9743117

9743117

313364

313364

151314101322
852328

456238

22214

14

134232

221242

221242

70236683617845

70236683617845

21089120160216238
98859576697011001032

1311

413814

671841016

7442114

195761030

9

921213

15353

7436619

1027812

21

6558117

17144145

42223

174821117

2313154

232110

25

3121

112

8643525

105362517

61531518

1

422479
684479

262

1

11

453510

5442273

1

21755

3051215917

5246118

424

15173

33132

21

211
4111

21

2117

91421

832

212

211

3141

14151123216

11

37441815

1159111810

3113

733833

21421155

308265379503413139
11

308265379502412139

1132

112

1051

1142

1113381221

2

376415

922872716

3572619

15210101115

1

739722

4294612

5122418

5381230

11315724

1411833

3

1

4321

112

96232

1

311171

181

544464

11

611111

12

12229

325931

621720

7112257

1676867
1045435

631432

319655

8256619

31620

113

12105358

51262

51262

152128131615
131219101212

933

1

123

151

1

312643296721
31

129165348

129115317
129165348

531

911106197

911106197

113
761718136

4451283

218653
219653

1

1
5016024273807371045

4695193893687221024
1027133317

14548409825

14548409825

303018101615
1113

181445710

111514582

329321299263457883
977511676152246

333

222115

512321

314446

223122

115

1

1

31

12112

9538358

27331

17812237162
17813267169

17

3

125106910

3212

1

11327

1

11

281

325620

1411

1111

24

2

46305459148131
29162227109121

1

17143131379

1111

2415341629164

1179221751

1

3141212

5135

4252

22165

211

84723

262

5612

13173

2511

3012

1542

984815

21

12

32

327
324

3

122421

1

1131

4101256

535424189138
3141

134286738

17576515

206561314

431132
333327342956

14644542

114531

1110132359

31351132

13122
328337121521

1138142

1138142

363236

363236

113211211

8207111

31241

31241

7133221
3113221

22

2

545134

545134

4126225

4126225

11412412618212253
12

91651

1

1

1

11

11

2944441394425

111

322133132416

21641

414038214910

62525251030042165595532456629341265
505945473742102139172735

605841355883
12715716485144152

429168253

3

202

917

273218172822

5995714

13111

71523

1217359

1

2211

1244510187

41721356023092381883123220855
61849550419841671393741451229337080

419292163
718620586339668512

685093195948

775359414736

9169894212270

85169935516474

785947637875

593159274039

373232162416

10188393243112

1185046427539

33296330317323037339278283204116990
55668745223637156278721402766308286

115127150169632

5951816692494252192124512160239

58968712729935030263

1

313293307120247165

313293307120247165

4252361016910

7085158211635731017875

1428139918

556257365190206109

3494201135812236

66863623119373310001429

16013419810610828

19514424277127135

742527163712

261816167

1

84185328547217

15133642345

16022798579040

32126

11

5

43459047428332180

85626525336466361971112

45642582822753673358

482483937391304147487

1094732016010322

4841218659

1061671348212495

4471

87241543

128191

2384852497423044

2

436112
747155

31143

52141510

541657483113

40203515269

34233013189

54532

1

2112109

1362281065467141898427092425403

1362281065467141898427092425403
1354691060307091396937041325177

1361281103011073

1426272

722135105

3551611677515055

55764

171175111

291531124

29543034312

1164445236834

27243954416

5312244

16911718

320792

4122103144

8010410620

235288301

972161482026698

2813816232

1

1

25871405151579313181175
439278256177300342

1

8243157428947
8042156428847

2111

17611368408545
17111368408437

518

663829304549

464745225226
534745225226

7

582144223145
582649233447

55132

21

413051143226

12824442

2911331

872950303753
10236108324759

157582106

757151354044

6373105487853

1813

582312131420

3881101495683118

1

1

986760246890

4662051526012777

411

3

1418063357351

1065953356629
1064651346629

1321

11141319

462725112612

84265

11

594132843432

21126116074171154
109112410

2615926111014

16142081715

312044111914
322044111914

1

361012105027

283023183128
283025183128

2

631922144046
601921143945

3111

1711761537212563
415118124

42183318269

21

21

123143109468650
114140100407845

12

1

1

1

133

13

73235

11

11

2111
1589073377237

1104746214920

484126162216

352332062179110726541054
455262309215276200

20920021910919460

1311316232

8671422420119977145

56112633276

1956950445362

434571

127122824810949

193117987415166

325154290142209104
323151290141208104

2311

896569436216
896574506316

571

1187994426935

55182421205

2751391127410880

3023292228427655

12220313945112168

407631363217409190

524241375110
513935344810

13523

11

1543331286119168
355589322180358180

11

812521

62

656558334134

1

37983913507

535446314253

121112

1111

111

221337232511

42111

92538

12297994991844185117814732
511538383940152359851807

2310910230

131142126489152
11213191437231

19113551921

36671361814239

25112

25112

543775406187341205
617839440202392242

234624113022
264724123122

2111

1

192469

11

1

2

162368

512

5

21

14932114

14932114

21

3512

122203115377327

486950353311

5628274034913
5629564034914

1291

758860255714
869465266516

1165182

4

15329916110123056

34241911108

712449455016

708347317922

8249613513026
8649713713230

410224

627412183423
587312173321

41112

1362427

22920516875188123
23020516976189126

11113

2

210158114

884929344816

685955153331
18381637160083817161192

10228869246091038623
1512134413267701468924

11546

11546

6
17

11

9244310

2

32627228899245201
383308311110273220

2211

553423112718

1581710
15713

18

132

82118654513251
95126754614154

13810193

25823421953215237
362068233112

1821791314015795

403520112530
6244

822

26352072124

72408520366
51377515355

21310511
21111

21

271

4

671388300151360185
4782531998822455

13844116
1821038653124122

102225

3399142642

1268473338569

3

1

1132137128

1

587060173834
23453691717

35252482117

35252482117

211201271112153117
13987176578130

51

63104455

63104455

6111184116782

284727111627

712041204714
491834123610

31

22247114

22247114

16231525161
18291525351

2619

2619

601810272112

414292400115241111
2301812997113342

151
1311175195

1310125185

396194138
17110084398964

31

11

1319465327555

1029213073

1503166367447

15913595726531

15913595726531

9915095515549

85166834014257

412333287121
292028246319

1235482

121317131141

599330430229324156
688376477243363178

3233232146

4215
1046210

64415

154

7532

53226

209103108
12

209102106

1

235105936310761

15221483322

15221483322

15711398103668513391298
1087760516356

322432132852

322432132852

1
7054504610654

482926266644

22252420409
18252418399

421

277258255100235402
796437176479

1

141822111576

1116561207

432120163410

161221

101016181843

615651174794

576752183592

555510313244450411
21914495100167116

212531131642

13192113934
13192113534

4

9811

1

1613327122

10255414

1161216

15345

2336

91513581

2110

379111166

5331024

9171291114
11181391114

211

113652211

1

1747476

1214407159

1

3131

93552

8

30162191313

48664613

88351124

182111

21213843

322373

514741010

145541210

1423871030

126311322

1461571118

1461571118

20920171921

20920171921

339241176147201136
437131314030

5166105

21229253014

910211

522428242112

3511432

403417203521

775853162832
775853163232

4

352619192018

1

214291

3611861438

3611861438

1
7112561268863

35542073516

212712133638

1444296179

172223188117

172223188117

326123104330

326123104330

185815761609106614292491
767062456574

8242710831
817514662417649930

5764119

5764119

6313
172919141719

321
1321

1

474164

3132

442722

733167

1632

552042274862

1313

1

711181616

7353115

1455247

14553103

12612101718

21721111013
12

9316778

1234235
1235235

1

26717122157
242314

214117832

33231011

1514113

1514113

4952114

474184
4952114

2113

5633394273110
340229271211303440

435328

6221

41110

185332

3122

547429
447424

15

3415

6512

728359

8175437

1414162

34461

11

124581225

1321

31234

274413

221

12711115

437

18181

1

41321

2921156

7138634

53215

231

12121

124251

2132

6541102

61264

121523

11

1

2129
35283572229

35263562020

62844

1

14

11
111

1

251628433310

913157
91317

5

78163

45236

13253422

42

72796167

4

5

642116

6541212

211

59844

9975817

32357
55577

12

132

77581111

77581111

31151393159
10101042139

112

2143

113
3

11

15314313

111

1

11

1991001415375110
622468182442

20162228

322425

211

219413

1

51331

117531

12

11

11273
11243

3

3171

13

102122

13112

2578564

7142

1

2539911219

1

112119

11

141159720

9234514

592526

973183

973183

5365115

5365115

11
18143571118

64313612

1194456

382441254859
1510433

111

42433222

413218
414218

1

1121

89112910
8962910

5

12231
12131

1

18488115

11

266280235182248712

266280235182248712
9393614686129

1112

41

171116517331

2311

22135313

23115

1

111

5521
55822

81

8

421351

125431

214243

111723
211723

1

111

3

3110174
151

215164

26

4

1351

15532913

6185232

666212

131

68151438

169911820

2131324
231324

1

211115

1

211

182433

1

1

15

1

1

11

453131
454131

1

821358

543413

82316367

1125

13459

131

16121

2

1116315
6237527

5121212

3

3

1

11

8321

375147

1

12221

1

341

59421715

122

511

1

1116266

3

21

878753373847

878753373847
153717779

3113445

3151

41127

323

2736535

1687641

413921

66534

5

3231

128

634426

343236142419

343236142419
14161331912

1111

5121

321

11815114

4141

422221

8487544873123
1111

7984544573119
16161671219

2

3112221

289258

111

2125

83612

4112410

221225

3132413
32321014

161

452129

1643

10149
101159

11

3213211

81011047

441533

2333

211

2327

21251

131

4223

4223

1411701197096116
3115212

18633172013
7410383567070

522

131

211131

4931

3229

353

112164

31832

37532

11

633

25212

213158

1

10874411
10874311

1

7176210

351

3

312

3341

111

722

27432651333
31122

1074132

3211

52710134

451233

2381121

61323
3713571211

8103378

233113

265268328208176312
11

31131112

31131112

262257325206175299
819885714991

31

11

215

515813

1

121112

8

11

1

112

11

1

4831

6

1

12

1

1569

112

111

112

52111

11

6

12

864412
862412

2

332245

221124

22
22145933

22145713

1

781512917
10818141118

33221

3

22102

11

324651173745
355453244346

1

381761

81123

1

111

16

12

61

3

39

1

1123
123

1

4

411

52

21

11

41222

12

111

6

681314

2151

32

1112

21

14

3

11

12694318

22421

1

1382

5

1

21

122

3212

11

47252063

2

11

1

1330

1
271616132038

215552

15543833

996473

111

6152443240120

1481613517

743238

74131129

4744281935103
91033147

264323

1411541

13118449

222219

101536

10119458

1011621526353136

1011621526353136
33923

23493491116
891411325644124

218

1

21

3741

1

11

567990442692
6283914530100

211

213

617

9910755

9910755

9124

9124

7112125

7112125

7112125

7112125

22
7112125

151121

3311

131

1093657131721
162316981887597540744

14217599

2232

1

1

1

2222

1

2

21

1

1

36814
31

6714

1

1

1

1

373

373

291

291

71
2

5

5

1

413412

3112

211

211

1

1

1

1

1

1

1

1

1

1124

1
1124

1103

1103

1

1

1

11

1

1

1

1

1

1

1
1313

2

2

2

2

2

111

1

1

1

1

11

1

1

1

1

1

1

1

1

1

1719845

1719845

1

1

1

362

2

11

1

334

1

1

1

111

1

2

1

1

1

1

11

31

1

11

2

18820932913460102

66875221319

1

1

66875221219

66875221219

434
1

234

234

1

1

43821
981152681212651

3111

3111

11

31

69741751182240
121

161753327
16411

134
155

1

1

1

141026205
241

9919104

21

21

56223

56223

56223

431471
31921

1255
1245

1

1

11

1
11

1

2139

2139

1
11861

31

31

11451

212520191420
11

111

111

442625

11
442625

432525

12

11

11

1

15201813913
11

101211969
111212969

11

1

1

1

111

111

274132
274433

3

3

1

1

1
11

1

1917906466
36292211

11

1

151
95312632

11
1211

111

84242431

45271312

411652

411152

5

41181

22311

4726

1

4626

4626

2531
761121

51

5131

512

1

1

253584139

1

1

242682117
2361

56273

33101

12131

12131

1312263
1

55182

8681

2

1821

112
2049111732

12

1

11

1

1

112
143981526

1

12

3

133761420
1

511

1

165

113
114

1

1

1

1

714415

1

111

1211

2

1

1

11

11

111

111

21

1

1

1

1

1

528225

1

1

11521

1152

52

11

1

1

1

1

1

1

1

1

4133

1

1

1

1

4132

2

1

1

1

413

413

413

19899240142167144
27302951121

1

1

6482235

718505622

1

10121517

11

1

13

1

1

3112

141821215

34211

15

1124

11

2

7

33911

1

1

1

1

5513927613679

2

1

5513917613677

1

1

1

42

112

1

1

314542171413

233638151010

1

1

1

1

1

1

2221

21

21

1

2

11

11

1

1

1

1

1

11

1

1

1

1

1

1

1

1

371121

251121

11

1

1

1

11

11

11

111

111

1

11

3

3

11

11

11

2

2

2

1

1

1

1

1

21

21

21

795141

1
2411

21

21

21

11

1

1

11

1

1

1

1

2423

2423

131

11

3

3

1113

1113
3

111

31211

2

2

1211

1211

1

1

13

1

1

1

12

12

12

61026113

11

1

1

1

1

13841

3841
13841

1

1

471672

471672
261261

21411

21411

1

1

1

1

1

1

1

1

1

1

1121

1121

1121

1121

11112

1

1

1

1

1

1111

1111

1

1

11

2

2

894243

12

12

12

3621

2111

11

1

1

11

11

51

3

2

1

1

1

11

1

1

1

1

1

4111

21

21
1

1

1

1

1

11

1

1

1

1

1

1

1

1

1

1

1

1

1

4112

2112

2112

2112

2112

2

2

2

102812401118280260426
13415

12112
429281357101130220

14186455
6914156244656

17

1

1

1

7

11

1

1

1

1

1

271
11

2

2

11

11

1

1

1

1

1

1

1

1

112
11

11

11

1

11

11

40

40

7710828
504644183239

6

61112

1

1

1

11

11

3

1

2

1

1

36

1

11

1

22

11

1

1

11

112

13

1

3

9

1

2

1

2

1

1

1

2

13

1

16

1

11

1

1

1

1

1

2

11

1

1

21

6

1

1

1

2

1

1

1

1

1

1

23

1

1

13

61

1

1

1

1

2

14

12

2

1

1

1

1

1

1

1

1
115

13

2

11

431

32

1

22

111

20

20

20

111

11

1

1

142222
2932107323551

2311

1

1

1

2211

2211

2211

2

1

1

1

1

15

5

5

1

1

31411
21

1

1

321

321

1

1

211

3

3

3

11

11

11

11

1

1

1

182194282928
227322

10

1

291

1

22

1

11

31

1

1

31021

1

1

1

8

1

221

1

1

3

1

1

4

4

1

76735

1

12

12

1

4

9

11

1

21

112

1

1

3

1

1

1

2

2

2

2

1

1

1

1

1

1

1

3301061934246103
311330279

5

5

5

22131

21121

111
21121

1

1

1

1

11

1

1

1

1

1

1736294191755

1736294191755

1242962161828
2

31

2

11

1

1

12

1

2

11

1

1

1

4

61

2

1

62183411519

2

1

53817236

1

11

2110

2

110

2

238
7

1

1

2

2

516231233

1
822

2

2

21

1

21

1255

1255

4618711

1

461871

22511

22511

22411

1

1

8813731

15821

331

331

111

22

1252

22

132
32

1

4232

1

1

4222

4222
1

1

11

11

111

111

21

1
31251

21251

19203720811

1816341969
21

24362

11221

112

12

1

1
11

1

1

1

111

111

14

14

2

2

636455

1

1

1

1

12243

1

11
1112

12

141

41325
1

1

3224

1

1

10923812

2261

21

251

1

1

151

51722
241

111

1

11
1

1

211

21
3695

3

2362

2362

2

11

11

11

1

1

1

133122

11312

1

13

12

22

22

1

152719945

1

1

1

1

1
6117322

2412

1

12

11

11

1

1

631

1

1

1

4

21

11

1

1

1

81412523
12341

11

1

2

1

1

1

282121

1

2511

1

21

1

1

11

11

23

8514892271237
34521

6358319

6358319

22811
761418217917

1

1211

721337316815

61122579810

61122515810

64

11641

11641

610913

610913

4121

4121

41

2

21

451352

135

3

105

452

452

452

337544289577880

77493111
193

1

1182

31221

111

222

2430241

330537240267779

726711

321509233267677

279465213256965

1
4144201712

4143201712

1

11

111

11

11

11
1

1

411
21

1

1

2

1

1

111
566919121614

338

218
1

1

15

15

1

1

2

2

12

12

23
526617819412

3953141127

3853141127

1

23112
99271112

135
11

124

6613412

1
31111

1

1

1

1

1

1

1

1

122

112

1

12

12

12

222

121

1

1

11

11

14121

121

221

31

31

31

31

2851299535

1
2

1

8142133
511111

1

2112

1
1112

1

1

1

1

1

1

2

2

41
15281555

322
15281045

1325813

23

1

272
1

62
52

1

11

11

3551127

2125

132

212

11

1

25313

3

3

1

1

1

21313
1

2

2

11

11

1

1

1

1

111

111

254330949

2211

363
132

13

11

111
203525848

714623

510712

71111632

2

2

2

221
101382

13

3

3

1

1

6931

11

593

111

111

11

1

11

1

1

1

1

111

11

1

11

1

1

1

1

1

1

91111211

21

11

1

11

1

1

3

41

2

2

1

4

1

1
23168858

13322

323213

1

1

1981413

212
