# Supplementary material for: Characterisation of the Carpinus betulus L. Phyllomicrobiome in Urban and Forest Areas
Source: Front Microbiol. 2019 May 29;10:1110. doi: 10.3389/fmicb.2019.01110 (PMC6549492; doi:10.3389/fmicb.2019.01110)
Supplement: Supplementary file 4 [file Data_Sheet_3.ZIP › rpoB_all_kronas.html]

Javascript must be enabled to view this page.

magnitude
magnitudeUnassigned

epBi32\_0
epBi41\_1
epBi42\_2
epBi43\_3
epBi51\_4
epBi53\_5
epBo13\_6
epBo14\_7
epBo23\_8
epBo24\_9
epBo32\_10
epBo33\_11
epWa11\_12
epWa14\_13
epWa22\_14
epWa24\_15
epWa33\_16
epWa34\_17
all\_datasets

706495068263649491259007673390686310726495186370835964486398829394947715141429
1121117

706395068262649491259007673390666310726495186370835864486397829394937715141422

705594998255648991219007672390466270723194956340835564396368828094837703141159
112122110

13127

13127

13127

13127

22

22

11125

11125

12518499365323364

1113

1113

1113

1113

1113

1113

11

112

112

112

112

112

112

112

22126826311236

11

11

11

11

11

22125826311235

11

11

11

11

21125826311234

1113

11

11

112

112

21124131116

21123131115

21123131115

11

11

1331321115

11

11

1121319

1214

1135

11

11

123

123

11

11

11

11

11

11

11

11

316132212122

2114

2114

2114

2114

112

22

1124

1113

1113

1113

1113

11

11

2131111111

2131111111

11

11

11

212117

12115

12115

22

22

112

112

112

11

11

11

213

213

213

213

213

31217

31217

3126

3115

33

33

112

112

11

11

11

11

11

11

11

511321312471445661577159145

421123341125107711266

212222112

212222112

22123121123105711154

22123121123105711154

1111112913834444672

1111112913834444672

1111112913834444672

45832122330

45832122330

3511131112120

2111117

11

11121118

11

33

11

11

1111116

1111116

11316

11

11

1124

1211117

11

121116

11

11

11

11

1113

1113

1113

1113

1113

11114

11114

11114

1113

1113

11

11

460495658139917939383902901124116312081399102715336442061278855919929

25944359213111737749339246986109611001262872527259189108635813411

111111421114

1111116

1111116

112

112

11

11

112

112

11

11

1113118

112

11

11

11

11

11136

1113

11

11

11

33
22

11

24223122821235645

24223122821235645

21211221315

21211221315

111115

111122210

12112119

12112119

1212118

11

2111115115221

2111115115221

2111115115221

121239

121239

11

11

11

121228

112

112

11

11

11125

11125

25443959013071731747338243985109310871256861524254184107734913319
11

13212211

13212211

13212211

1113

321118

112

11

11

11

11

11

11

11114

11114

11

11

1113

1113

11112118

11112118

112

112116

2241221221221

2241221221221

231222113

11

231122112

112

112

1113

1113

1113

1113

2236211219

2236211219

2136211218

2136211218

11

11

11111117

11111117

11111117

11111117

33151122119

3315112218

1115112214
1113

415

11

11125

224

224

11

11

11

244231121623141

244231121623141

12134213

22

11

11114210

243219123128

243219123128

71039679137161241637460567319103428737

151125522112111122743149

11

11

151125422112111122743148

151125422112111122743148

1591134358716131356100

11

11

149113435871613135699
134

11

121228

11

11

131141516

11

11

1326

11

112

112

121122110

1111123161119

11

31125113

11

121228

213

42718139139161522172383154189

11

11

42718139139161522172283154188

11

272133221124

425975126101015101362102119

425975126101015101362102119

1113

11

112

112

11

11

1113

11

11125

132121123511326

11

11

22

28929392567272724201110531415281

114512441532437
12211122416

11

112

11

11

112

121311110

1124

2782434255725232019115211311242

112

2122221113

11

156

111115

2762032235722161818113211210215

112

112

11

11

11

231118

231118

213

21115

13149

11

11

12148

134

11

112

11

1372623111431

131319

21216

21216

11

11

112

112

114312315

1421311

11

11

11

112138

112

112

112

112

11111117

1113

1113

112

11

11

11

11

11

11

22452574829116260

22452574829116260
1111318

11114

11

112

11

11

11

12115

11

2114

11242535154134

11

112

11114

11

11

1113

11

11

11

1113

1113

11226

112

11

11

112

11

11

11

121127

112

2125

1010183448361411354425543171011419448

1010183448361411354425543171011419448

11

11

1010183447361411354425543171011419447
111115

22116

112

112

11

11

11

111317

112

11

2136

11

11

27373211215124252

8101423382111103027204625457337339

22

11

11

22

11

11

2112112111

11

11

1112143127259615918617625390110232661738211699

27411132021835727665109

11

11

61211222118

6121121216

112

548831123122141
112

213

11

11

11

11

112

11

11

53481121126

112

111115

111115

112

112

2235941341311140
1213119

1214

11

11

213452121111125

112

112

95139116246415916516825085103230591132161590
23796513482852811403248121264510793

31424223122

31424223122

23229

23229

1312119

31217

11

11

1113

1113

11

11

11

4218152814941233027242791173256

112

4218152813941233027232781173253

11

2311333116134

2311333116134

1353361122272567523257723411458

112

1333357122272557421237221411439

23112110

111137

11114

11114

111143213

111143213

1112218

112217

11

12115

11

1214

2710241956613167713343157167

2710241956613167713343157167

112

112

11136
11

1135

132115131119

122153115

1113

11

111126

11

11

11

11

112

112

112

112

112

157211745511964732106123

157211744511964732106122

11

12227

12227

12216

12216

12216

12216

22139737410471287581240196880762714101963918814113392628010025

22417431630

21417421628

21417421527

11

112

112

7182146624617111842369315960426910116864
1416

1111127

1124

11

112

121246112397322551

112

12112311113

12225195212436

1111321616

11

11

1113

22

1225

1113

11

11

11

42511

42511

21111211111
11

1113

11111117

1581311245216623161252167
11215

1117111

46411212125101271279

33

11

182112

11

4116

11

11

11

11

11

10212

112

1141121112

11121118

11

11

112

11

11114

11

1111116

1111116

22

22

6131234423714912322983662717328914568

6131234423714912322983662717328914568

102618

2147

81211

112

112

112

27626222366619188

27626222366619188

11114

11114

11114

1141144218

123

11

22

1211128

123

12115

1135

1135

112

112

1591311268130187

414413

414413

15213244125167
41117415143

1122711024

21115

1113

112

112
11

11

7913282529252332164437251066212385

212111210

121127

1113

213

213

121116

1113

22

11

112

112

1126516325

3216

11237

314

321118

12111112111

12111112111

211213110223

212119218

11

11

11

112

12112310

11

112

21227

6952216182421271144269745311295

1113

6952215182421271144259645311292

14207552552274165164502610312416600

1316746224126314414034151029614485
11

141185310134

112

331281116228

112

13157332129223114111241181709374

57124119241

22

11

14931111212416111282115

14931111212416111282115

112

112

112

355392717521293109

355392717521293109

355392717521293109

17531033084311513792161718346536494883084158513942077258
2241110

2238832910462113165

1214

22387329846213161

16921753842

1691753840

22

1111419

1111419

1113

1113

1212225122121

23261565152232112379

23261565152232112379

376521311130

3643211121

111115

1113

11

121563152213436

121563152213436

11

11

116212361241158

116212361241158

116212361241158

11521236124156

112

28613470111423729110758045464243433845
173221496928211783626337272212224577

22

1113

11

511119

2316

215231645211235

244743142212238

112116

2312111112

62061424710614611415283156

11

11

1234131116

112

1214

11

1122129

436783188256884633184108145131535141599411599

436783188256884633184108145131535141599411599

231526311125

44

112

112

112

3212311114

11

13112102316141311573

4275119

131882399131554

223531109138102941314378
19312794118102841313330

34415201148

613271355512241508568321911152555

2513833126

613251350501941428265311911152529

221821117

221821117

11616245516862623196124295248
56152694219157341253126

11

111115

753152222521147

1322722543121136

111418

111115

22622122120

497983341340894164162165139129149163020198412135
1518102128141347

11114

22

613148176221212293127261534103613430

325

5113313

8109734132979421635145775365

112217

22

11

4672732961212141245231156

11

42151681314855274

11

112

11

3312312

211610

1113

24424912016144202494848565444655119941

11

11

112931213371943

11215

22

112

11

11

112

12222751117425229141106152124182222074993
1111318

441511421225

3461241121445538373021214275

918213571293224866611190142211757685

235313219
22

11

235112216

11

11

11

15219

15219

11

22

33

112

11

111842943125451811114214479

111842943125451811114214479

11112

1013367291004317912614404

156232421211663

121512113

1113

1113

44

44

121116

121116

1381011943118876

13691833116565

11

13661833116562

22

211112311

111238

11

112

212114111124

212114111124

212114111124

212114111124

112113111122

112

433648241396107324912109

433648241396107324912109

112

112

112

112

11

11

11

11

1113

1113

1113

1113

112

11

11

11

11

11

12141110
11

112

11

11

11

11

11

11

11

112

112

112

1113

1113

1113

11

11

11

32343522975822124872

11

11

11

11

11

11

123

123

123

2111321312111323

211321212111321

112

12112111313

11

11

2114

11

11

11

11

1121211110

1121211110
1121117

213

12231115221223

1131115221220

22

312121212

1113

112

11

22

22

11

11

122131111

2111117

2111117

1124

11

1113

111123112114120

1111121210

11

11

11

11114

11114

111115

11

11

11114

11114

1111212110

112

112

112

11111218

11111218

11111218

21216

1113

1113

1113

1113

1113

11

11

11

11

11

112

112

41443211112327

112

112

112

112

112

121127

121127

11

11

11

11

121116

12115

12115

12115

11

11

11

1113

1113

1113

1113

1113

2114

213

213

11

11

11

11

3121119

3121119

3121119

112

112

11

11

22

22

11114

11114

112

11

11

11

11

11

11

11

11

11

19748607546173473811751941221451000372186616821836316

2215

2215

2215

10510711188414181428203219413611306

1231111212

11

11

11

213

213

11

11

11

311128

11

11

1113

11

11

11

314

314

1111116

112

11

11

11

11

11114

11114

112

112

2215

2215

2215

2215

1058711168414151326162818403410283
23112714223

321231172343241443
112

11

11

11

11

112

11

11

121231723332333

121231723332333

134

123

11

2718321

2718321

2718321

2223323111111124

11

11

21213111113

21213111113

212117

212117

11

11

112

112

112

112

112

2121152216

1111116

1111116

11

11

123118

11

23117

11

11

22241222137105109264

11

11

11

11

1113

1113

11523113

11523113

114121244268238

11

112

1539

11

11

22

11

123

22

21115

123

11

23117

11

11

1112117

1112117

11111117

112

112

11114

11114

11

11

412312212461910158

12126189140

11

11

22

26189136

112

112

112
11

11

11

11

112

112

1113

1113

11

11

112

112

213

213

11

11

11

11

11

11

11

11114

11

11

11

11

112

11

11

14142111116

11

11

112

112

112

11

11

11

11

1214

1214

31116
11

3115

2114

11

11

11

213

11

22

22

18138445621146363395227088113948345181716351695857

105223537714163115101649981861

21121142152932366

21121142152931365
11

11

111211114114

11

11

21113822240

11

11

33

112

11

11

11

117110

1719

1719

11

11

8631681123609261

11

11

802866112478233

106141133671

34111964

1961944

1629

3249

11

11392833

112

11

11

112

112

11

11

139114

11

11

3148

3148

211117628854361

11

11

213

213

1117626450330
1267979

265875

11

2125634

321529

11346859

11

22729

17623

1121326

13215

118111

11

11

1211216221239814145

5111162213621095
2911

10111

11

11

2115321

11

11

11

11

11

411316227

516

1214

11

11

112

11

11

112

11

11

71136449

11

71135448

221418

221418

221418

76364221186936329121648510294333518011136884996
112

51223516131734268818597948238145974684

11

11

51223516131734268818597948238145874683
11

22

11

51213416111732268815597947238135774671

11

2215

112

3112273810100

134
11

22

11

22

22

11

11

1313
1111

22

31237723

31217

11

36615

11

11

51152

11

3030

2121

224

224

1215170341951014414

1113

1113

1115169341951013411

1115169341951013411

2142113578202124372322762

21113578202124371712749

21113578202124371712749
1111013

11

44

12216

123121066373801438

527

1112658460232511280

11

11

11

3116112

3116112

617

3115

7104321123524427263293

11

11

167

167

7104311123424396659278
31217146429

213

11

11

11

1742115

33

2163113

311104291361

11

1710138252488

11

111112723

11

213

11

112

11

1113

32510

11

151714

112

347

33

44

13349112248175482641757716
11

22

22

11

11

3231142767343163

3231142767343163

33

1717

55

1212

11

11

1013612228110391961374521

12135271664

11

11

11

2127214

18110

25126028125

61421651158568239

61421651158568239

11

22

11

22

11

11

9516939

11

1214

11

11

11

216312

112

112

11

11

112

112

11

11

11

11

11114

1113

11

11

11

11

11

1113

112

112

11

11

112

112

11

11

213512216

213512216

213512216

11

11

11

11

11

3447942177373386179

24457321743511251

111115
11

11

11

11

11

112

112

2445622173341246

2445622173341246

11

11

2435612173341143

11

11

11

11

11

1211323274127

1211323274127

123

11

22

1137416

137415

11

3115
33

112

1113

112

11

1113421137551144255

1112421127551144152

1124212754424140

1124212754424140

1124212754424140

1117212

11

11

17210
22

1528

11

11

1113

1113

1113

121161113

121161113

121161113

11

11

11

11

11

11

11

11

11

11

11

1618

1618

1222111411321224

1222111411321224

11

11

11

11

1221111311111219

1221111311111219

112

11

11

121111311111217

12115

11114

11

11

11

1113

11

11

1214

1214

11

11

11

123

11

22

1211123123278135

1211123123278135

1112128

1112128

11

11

1111127

111126

11

111113121176127

11

11

11

111113121175126

111113121175126

11

112

11

112

11

1112121110

1124

33

11

11

11

11

11

2121212112

2121212112

2121212112

1225

1225
11

112

112

2111117

112

11

11

112

112

213

213

11551114

1113

1113

1113

1113

1113

451111

451111

11114

11114

11114

11114

11114

11114

11114

111115

111115

111115

111115

11

11

11

11

1113

1113

111126

111126

111126

111126

1113

11

11

11

11

11

112

11

11

11

11

1112121121114

1112121121114

1112121121114

1113

11

11

112

11

11

111115

111115

111115

1111116

11

11

111115

11114

11

640987427572505464257611629987354920558482344863521847685319601966047097115473
213

11125

11125

11114

11114

112

112

11

11

11

648141423248384117911101141427283

2124472211444541241174

1113

1113

1113

1113

2124472210334441241069

11242243121124

114223212119

11

21216

11

11

112

11

11111117

111115

11114

11

2234726111313945

32128

112

123

123

22476111211937

212761121730

11

11

1113

11

11

112

112

112

4361010162262737135661021016209

23311471123

331119

112

11

11

213

213

2114

112

11

11

2461114

11

11

2451113

11

112

2215

11

224

11111531111118

11131119

11114

11114

11215

11215

1122118

22

22

112116

112116

11

11

11

112

112

112

1112124212219

11

11

11

11

11

11

2124111214

11

11

112

112

213111211

22

11

11

213

11

11

11

11

1113

1113

1113

133313121316522151089

113321112

1113

1113

1113

1123119

1123119

1123119

223129792223962

223129692223961

123124661123950

213

123122561123947

11

11

153110

153110

11

11

11

113341215

112

112

112

3231110

11

11

223119

223119

1113

1113

1113

11114

11114

11114

11114

121412332323330

122121110

11

11

1214

22

112

1214

1214

11

11

1213113222220

11

11

11

11

21126

21126

11111117

11111117

11

11

11

11

1113

1113

2112151121118

112

112

11

11

211116

211116

211116

11232110

11232110

13116

1214

11

11

11

11

21115

22

22

11

11

112

112

6212996791221812311383275637232939991111621221083537

142620183339313342615920241814191716504

10251916323631293750551921131081216439
3115

913147213127233236511519875914341

11114

912147192827223136511519875913332

11

123

123

11

11

11

1113
112

11

1113

1113

11

11

22311151117

22311151117

11114

11114

112
11

11

12121112211116

11

11

1113

1112121110

11

11114

11114

111216
112

11

112

11

123

123

11

11

3231343120

3231343120

11

112

112

11211219

11

1211218

1111223318225

2215

2215

1112318118

1112318118

112

112

31111233941233340

11

11

11

11

112

11

11

11

11

112

112

111132111113

1112117

11

2114

11

11

11

11

11

112

112

21115

21115

11

11

11

11

11

11

11

11

1112117

1112117

11136

11136

22

22

11114

11114

11

11

11

11

74322132723140111742345062002820412239541604

3513410212244317213185144

2226226224

11

22412112

11

211419

11

11136

11

11

11114

34134819193715211123114
33134819193315111122107

11

11

145

7401712241913091622104631832619382131491460

111121431217
111221210

11

12126

112

112

112

11

11

11

11

11

212647113525469114451091671934456

11226

11

22

431391024511162

112

211212110

1844393045578635971071832365

11111128

21163732328

21163732328

111141161451111342
11

11

11

11

1416135111336

112

225861778741001323241301281912126911
6246648355681737373935471

111115

4281391138

1113

1113

12112117

22

12194118

21689197355

161141023

11

736517185717130

4261542134

11

14218

112

325

123

112

11125

112

11114

1211117

11

11

421119

123

31421112

1112218

132133345621135

123

11

11

11

11

11

11

11112118

11

11

11

11

1113

11

11

112

112

11

11

112

112

2849273943107515583226111503329321038201031

112231111

112231111

22

12216

11

11

11

1125111122

12411119

224

1227113

22

1113
11

112

232425247321340

1122121241118
112111124115

11

112

121131132217

11

112

12113121214

33

33

112

112

1161442160111184

6111523

44

6111119

111332144111160
1010

314

11

11

11

1115118

11221121121

213

11

11

11

162321096824235253232116

11

11

22

22

11215

11

11

123

16221796822234243132106

11

131319

224

11

11

11

1621147661722424112289

112

112

21211595121125139

11

11

1214

11

112

11

112551116

112551116

111121113113
111317

11

11

11114

112

112

213

213

11114

11

11

11

11

112

112

121331112311121

11

11

213

213

111115

111115

1312119

1312119

112

112

11

11

1920821135627363366632517162132111476

11114

11114
11

1113

1131219

22

11215

112

410536126521321716346223157

112

410536116521321715346223155

1510218640203012344691210141198306
13116

71173181211327575282
411631321132623259

11316

11

11

1214

11

33410

11

11

79111318183010234375571136217

791631418309234375561115203

2215

11

3115

1113

1113

12122534412222235

11114

11

1113

12122523412212131

12122522412212130

11

2116322121613211154

215322111612211150
236121116

21322157124

314

11

11114

11

11114

11

1113

28459315183462474

11215121115

11

11

165127121127

165127121127

22311132217

11

112

12213110

112

112

11313111214
11

11

11

1213119

112

34696185455

122351317

12216

22131211

212218

212218

11

11

22343103128

1214

2132393124

11

11

112331335543613347

112331335543613347

11

11

11114
11

1113

22111119

11

112

211116

1214

11

123

2331313421311129
121116

11

121116

11

11

1121231112

112

3372632261211212654566121

123

123

123

211112531133422151

211112531133422151

2111152123322127

11

11

1113

1113

2115123322123

1124

11

2422212

11114

112

3115

112

213

171119

171119

11

11

11

14326311212430

14326311212430

14326311212430

1121221718323236

115131113

115131113

1113

11

11

12115

123

1112112110

1112112110

11

11121219

11241110

112217

213

213

123

11

112

111111211111

1113

1113

1113

112

112

11

11

112116

112116

112116

13116

13116

13116

13116

11

11

11

11

1113

1113

1113

1113

7516498591221377611103149193

11312222313131329

11

11

112

112

1113

1113

11311121111317

11

13111111212

1113

11

11

11

112

11

11

1113

1113

1113

1113

641348657101933631071118161

11

11

11

11

112

112

11

11

123
11

11

11

1113

1113

3115

3115

123

123

1113

1113

123

123

11

112

112

11

11

11

11

11

11

11

11

112

11

11

423251510236142270

423251510236142270

1233151723413137

1113

112

1113151513412130

112

44

44

11

11

314

314

11

11

11

11

11114

11

11

112

11114

11114

11

11

584184476888475655665606552484063642349173734511446642354910561455945272100142

761897510773788412449627258627481721141961646

761897510773788412449627258627481721141961646

112

112

442121265611512101181166185

442121265611512101181166185

2125

112

123

11226

112

224

11125

11125

11

11

11

11

11

11

721847395707558684847595847597070981891440
224

721847393707458684747595545577069971881426

211111119

11

429258337610176105366783723006433344643673698843792270913979

413993318414411963311780525183467611881509

333753215212310560251571419183054541701340
20186116771482166311155153229101665

11

811220742940381993713112182255498

17131215

1411122719

4221615

963331126

11

352220811298

11

11

112

112

5102210813135384992
112

11215

31176421438

1921313115244946

192313114244944

112

11

81542231413943

81542231413943

354742530

112

112

1110520282421221714247524961414347

117920251819221513247523761414304

83012177967513742226414155

34988111016881111510149

11

11

2636221242

2636221242

6324101041991213124221810561260

112

112

63041010416881322121310559230

11

112

516

63041010416881316121210556220

11

112
11

11

3126

2114

11

11

121217

11

121116

19111
9110

11

22

22

29518132757154073684752572094401925460370533672604211610053
224

118771732531182497

118771732531182497

235

235

6153321232

6153321232

11

11

1113

1113

2223113115

123

22231111

11

122611325

11257319

11

1157317

11

145

11

4213242342251461930121

41712414251461926105

11

42131415

311193172257

216184233

1113133224

11

11

3117155555411198526147

2315521244521073

8173542715331674

12418

12418

967111002851491741851087917246151681642462237743695

967111002851491741851087917246151681642462237743695

11

11

11112641320

11112641320

1215316116856756761287521721419

1215315104856755741287021719405

311262318

311262318

121221166325

121221166325

111115

111115

10101137443464136104461510269

10101137443464136104461510269

112

111589126

9101136342361267730139204

691125342261206025138168

311116175136

112

1115197135

11

11

1135

1135

1111123491817682

1111318

13461617671

13461617671

11

112

112

112125214

11

112

11212411

11111117

111115

112

112

112

146835139321184155209105922088101779419634126310484531

1113

14683513832118315520910592208891779419634126310484528

11

11

271721974392258263041222611235460175861

11

11

11

75233447825239758468668131259102871083301796

1111210117

1111210117

271073462151779414078511118166274687

1315

11136

269832621415794040774911295951628

181122511839

11

112

11226

12110595513843368126229233554
1101211133766

4121421

11

111159

11

343111812201087186

7202954136221124151129142

2941212353104

2941212353104

11

1121117

3111814

112

35154651572563842407191723538

35154651572563842407191723538

11114

11

11

1113

1113

111121119

111121119
123

1111116

3661441421324555258

4512311212114230

1124

1113

11

411122111317

11

112

11

11

22

312111110

21115

11

2114

11114

11114

32121311355128

21211119

21211119

11

11

112

112

112

112

334414

334414

42448678261119322492022197

42448678261119322492022197

4234867826112222081622169

4234867826112222081622169

14104120

14104120

13138

13138

111211272119

111211272119

1111116

1111116

1127213

11

127212

332332411712423243

231129

231129

231129

33233111612411234

112

112

112

11

11

1311311112

11114

11

1211117

11114

11114

2114

2114

112

112

1214
213

11

134

134

152758485010743029331252392317418

122548374710522629129242272115387

122548374710422629129242272115386

11

122538164710212627129242152114373

11

11

112

1113

112

11

112

11

11

321113224221122231

32111114121112224

2111111210

111114211114

121116

1113

1113

11

11

3243132017116012773560106210134173644729
11

1113118

1113118

22

1113

112

11

322211112116

1111116
112

1113

11

1113

11

112

1211117

1211117

411155447413321461580

1113

1113

41155437313321461577
410532242121461460

11

11

112

213

112

11

11

1225

11

221111122321221

221111122321221
2111311111

11

1214

1113

11

11

1122111211

12115

121116

111111321122118

11

11

11111132122117

112

11

33

1214

1113

11114

11131116187242

11131116187242

1113116186240

112

11111627211749

11111627211749

11

11

111162721747

222779655211412949243492052617483

11

11

22111512125

11

11

11

11

111512121

18261423481122444135321632414407
1626141348112224333201532213382

11

11

11

11

2211116

11

11

11

11

11

1113

112

11

11

11

1111217

111216

11

1211117

11114

112

11

11

11

11114

11114

11111111210
11

111111129

123118

2215

112

11

31111119

2111117

112

44012461656051032162139434554118318115614421160994309111072718105

17649341361877220174293384530932281116

1141701852299230
11431120

112

44

15745215598

777103234106

112

112

1113

1113

11114

11114

33931132153

33931132153

164671171111752176418038432993228806
33

1113

11

41738212222411130

164264028631944417637432983228669

1621111

1621111

55

55

112

112

4231182156264671982062412537114117185614041115964300107869916989

1113118

22

11

11

112

112

4815232035117141958295149

4815232035117141958295149

14951939109656945191655217834605528426974722967476
46152104412234664515428621620515238164932574

94063266134465941233943420526

11

111216

11

57675232

11

1721124922118561116

111115

902942161530503106342894283336277217542501764101

112

11

11

11

21821520379741113102

11

211116

122328314514556810253104362433300272872922094506
254843228

223429

62614

1113

2033449102176459014443862952666

2312513320

12126

5597221305210

10023427351344638432603092315249232602271393530

10023427351344638432603092315249232602271393530

11

11

147320821035191514111331280471493342702511112891794686

12517

147315821035190500110831279471493272692491062811774635

11111117

474823

11114

1214

1214

421112554181111561

42111554171111558

1113

11111218

112

1113

11

112

1151102679212525490
11

112116

1416

11

312118

11

1113

19197810213262

112

5585736803985515803615283742676904853033053035084522978213

5155406353695125423324943302416384402852662844754162877601

11

11

1111217

11

111126

5155406353675115423314943302416384402842662844734152877593

111115

11136

5145406343665105403304943292416354402822662834734122877576

1113

1113

3327381727311918261538341217420262404

3327381727311918261538341217420262404
1112117

112

3225381625311918261437341216420262395

11417

1113

1113

134

134

221216723118211242

221216723118211242

112

112

12121672311711238

41223212525317434253

11215

112121114321321127

112

11

213

11

11

11

11114

11

1124

111115

11

112

11

31111171117

213

213

11

11

111171113

111171113

111171113

11114

11114

32255113916527443366

211116

211116

1113

11

112

1111211210

11114

1124

112

115111111

11

213

1214

112

11

2114

2114

11

11

1113

1113

11215

1214

11

1121111131114
11

12115

112

11114

112

11114

11114

111115

11

112

112

1122331421232542139

1122331421232542139
1312119

11

11114

122211115319

112

11114

11

11

11

42153670550325234124413620662696280312164588380118301747263137792991119555514

42023657549225114117412620522681279812084572379018051731143534042937118053698

161491237210233164

161491237210233164

1113

1113

1113

41853656548825114108412520502678279112084570378018031728143434002936118053631
181616191937202241119027381211245817496

33

1113

11

11

133281861118152910187

415

1221118

22112121221421

453279436946178132351713121660334544
213

112

2234323527357351

433078386541145823414591160241482

111115

11

11

11

32313154241038

211073127121618210252145

1124

821556351541

11

9235137363031505811366713138491

84629632218515071288232461322

134

33

11

112

11

83534436952311121795
3233121241234121760

512322454735

512322454735

3111127522126

3382354301733132761652042099432927713612981218217553879

378931851022141626211163

3002264211702952701541821959332325013412880218217543710

1111116

235

1292563621047721994

11

11154113

12115

1407915282153126643970171197481463311857691519

11

11

1113

112

11162112

196892158917313180931073115012560324917973261898

1115329123

111112119

112

59333793533173436847601118612223480

11215

14451232205890414551607674934997381164414495926534041130108227318914
11

13891188197187014031570646891945348157613805756364021096104626818200

14111122112118
112

1211112110

21216

112

5126683148302332423163631517233223600

5126683148302332423163631517233223600

2149442882321068

32632221111125

1298816870118106517862351468847446811182191510
112

11

1312742121

1278816867117102517860331398244436811181191478

112

112

112

1113

11

1548166620699791561156984410831057570176713677426737081397114567921424

111115

1113

2215

11

9866198100132114382832131276551613214067141376

121311971014154816112516119637554141810

19542711472162171376113

75427113711611666496

1113

1315

1111318

11

51121632541167891126

521141131230

11

51141131228

11

11152256477196

115223647792

1214

1111318

111115

123

11

11

63102123442485211083543861562
11181120

112

1111

14216

1157

33

134

66

44

22

8917

11

1115210

11172331109

113813356

11

224

11

11

11

11112145914616628

11

172120

111510128

5172233221422323125126502

123

112

1117551129

81110

31422112132261435

1618

191231

131122105610284

11

66

13116

13116

13116

123251221312126

123141211312123

21121119

21121119

112

112

12137

11

12126

12115

11

1214

1113

1113

1113

11

11

11

11

11125

11125

11125

11125

112

112

112

11

11

11113151881456626101713658164

11113151881456626101713658164

21361215

2361113

112

211111111313

111115

11

11

11

112

11

11

11

11111751119

112

11

11175116

112

112

11111117
1113

1113

11

782141461354625446341108

11

113117

112

1113

11

11

1113331311213134

1113

111322212125

1113

11

112

1113

11

442152831311338

11

1111111119

122117

10151022833554516294243252261714376

1412111431120

2111117

11114

1113

11114

11

11

112

213

11

112

112

112

11114

11

1113

911219231532132720144917812289

911219231532132720144917812289

112

112

910119231521132620144817812283

112

2211112114218

1113

1113

123

123

21115

21115

11

11

111126

1113

11

11

11

1111211012323

1112218

1112218

11101215

11

11

11101114

122422112323126

11111128

11125

11

112

13116

13116

11114

11114

12328

12328

12328

86213729610274796513107

7419325251523432260

11

11

112

112

11

11

1113

1113

1161111122118

1131111110

3111118

533111322331230
111115

22

11

11

2133211

11

112

111115

112

11

11

112

112

11

11

11

11

1433341224523138

11

11

1113

1113

11114

112

11

11

11221142115

11221142115

112

112

11114

11114

112

112

11

11

11

11

1113

11

11

11

112

112

2114

2114

1113

11

112

112

112

1113

1113

1113

324238142916141923162926183530241829452

273635122812111621122421111119191325353

273534122812111521122421111119191325350

273534112811111521112421111019191324345

11

1113

11

1113

1113

33221113156713342

11114

11114

111151111

111151111

2111117

2111117

1211325217

1211325217

1113

11

112

2313232424218442157

112

112

11

11

111211211112

1113

11

1113

112

11

112

112

112

112

1113

1113

1211222183335

1112122120

11518

112

12115

484253221587611834588

11

11

112116

112116

11248

1247

1247

11

11

11

11

213

22

11

112

11

11

11231163231125

11114

112

1124

12115

1113

112

112

213

1121121211

1121121211

11131411114

11131411114

211111119

11

11

112

112

112

112

112

112

112

121116

11

111115

5001615792057231801520182913148911950641429287339874168911501

2124114211133715292097

211411210133615281988

11121219

112

1121117

21411291244261875

22

21411291242261873

1214

11

213

12211119

11211118

112

121116

11

41210947417461715454331198151340703354731321627777115029767

5911113032153285012320883648338

11252112

11

1252111

1113

1113

112

112

123

123

11

11

1536188521332184411321145

112

1113

143515852113184411120133

112

12115

112

112

111115

11

11

12115

12115

1144111

1144111

11

11

11

11

327410208131686262120146
11211222223423

22648197101464241816122

11

1522145217171121321021115

12226515151123192094

11

11

224

224

11

11

12116212117

12115212116

11

1113

1113

1113

112

11

12115

12115

11

11

1313610261859

1313610261859

38222111121

38222111121

9543118521353571147116928242251369059631883562395
11

11

11

67368541106253946713923039241146051551562811898

25144116418761616610426165335332798160964

11

25144116418761616510426165335332798160963

112116

4211762103134232758

26153213401032123970613697132674533

1231110155510424536321159123040333

12115

123119155310424525321159123040328

11

11

112

5272514287252152390

11

5262514287252152389

1211633421

11

112

211523418

11

11

317272412148
31727141246

112

1111312120

11312118

112

112

112

21115

21115

18419620621641336211611642446308

751161131

511235115143228953

631341641154123011127421637224

22111113111419

211113111416
11

111115

112111310

112

112

11

11

1758819328145171164251413123947484
11

112

112

11

11

112

112

1113

1113

21141112316

213112313

11

112

11

11

11

11

514681283451471141

11

21134325412147

2337461111469

1157

1225

11114

11114

134

22

22

1151210

115119

11

114913112932

1493112930

112

112

112

11

11

71529154134582106441420169
22

51413821036618443814118

11

1116722912214544

11

1113

112

112

411162113193732291387

41162113193732291386

11

213

213

1124

112

112

112

112

11

11

11

2925233010043011092914155581710335118813219249710266416

16334122314351742133

11

15334122314351742132

37117024762004711911753252312127751581111
5131361102063231428124

112

3711701963187411080154324628192461130985

24630345386128424323721564854902394681902
11221411

1121411122642233117

32148825131873

15122433617510184867273

11131119

1181871331293497

23516421591115111172212948110247865

141161113812411023104

2371141

81851519252354612876103361310

22

11

11

21115

21115

112

112

121312717

21312716

11

2173522128197740691913126041899238451042232513
1482042

123

2123928531453546138672151340

42713

11

331035241119337241121623211315

1432515017127537521189190374663035791611789

22419

11

11789879427741223914162562128732

132520

22978216366351431159

13116

117553703455780331413204897547

564481307817957495187371351493650651131126

564481307817957495187371351493650651131126

1214

1214

2211118

1113

21115

111115

112

1113

29112116

11

11

112

91111

11

112

112

564278286917855474985361251453449631131090

564278286917855474985361251453449631131090

11

11

111211357112127

1111127

112

112

22

11

237111116
1371113

112

11

1113

1113

11

11

11

11

11

11

11

11

11

11

1315

1315

1315

213

112

206151145617822275318331862533327

1321011249112172531136552224221

2412110

2412110

2182112112223

2182112112223

11

11

11

1113

1113

22

22

3126

3126

224

11

11

1129112366161852231013137

1129112366161852231013137

11

11

2556127836

2556127836

11

11

7452766522252713139106

11114
11

1113

11

11

31196121

31196121

112

112

3132442312631237

3132442312631237

32123212121241440
12238

2111112111113

2113117

11

11

11

11

916864361211102673210115

916864361211102673210115

511111116236148

213

511621640

111115

45763261243132967

1112311111

1113

256422610422853

114176141035

114176141035

1476141033

147641032

11

112

112

112

11

11

11

11

11

11

111674356321343

111674356321343

1156333331130

1156333331130

1156333331130

115632331126

11

33

1111116

11

11

11

111115

112

11

11

112

11

11

11

11

122117

122117

21115

11

11114

112

112

2114

2114

2114

2114

11

11

11

11

112

112

112116

112116

112

11114

1112117

1112117

1112117

1112117

112

11

11

11

11

11114

11

11

112

177242152586943127101894573963208710639218861295407

11

11

11

11

11

11

112

112

112

112

112

112

1762421525868430279188457396320849238816937295338

11

11

11

11

11

11

11114

11114

1762421525868430278187456396220839238816836285330

231415417432

231415417432

231415417432

3112310

3112310

112

112

1113118

1113118

11125

112

123

22

22

2114

2114

11

11

21115

21115

1113

1113

11

11

11

11

478471727496241813324321621935

478471727496241813324321621935

478471527496141809324221621927

37536932448502157226221621465
3432561123281286118136635

3118527

44

262212

12126

21362112177168

81110

12113

151292350

21130412510612121276

1719

14377705232

181111123

1010

1010

22

22

516

516

314

314

141292266

141292266

31811101161244

711121123

213

112

11

11117415

1111174152330
201636

11

394382

11423781

121830

1413173

22

101525

21418

21418

21418

4574104110254574524432254

4574104110254574524432254

11

11

11

11

11

11

1572411225015111147

157241122501511146

156241122501511145

11

11

11

23461341211242146

314

314

11

11

21115

11

11114

2342124116122132

112

11

235

11

11

23411612222

112

11

11

112

112

21121136111214146

21121136111214146

11

21121126111013142

213

213

11

11

11

11

11

11

11141210

11

11

1114119

11

11

11

11

11

33

11

1582223212338024296411147181130232235

1582223212238024296411147181130232234

1562203192137814196410776780130232181

11

11

112

112

11

11

11

11

11

11

112

112

21216

2125

11

11

11

143212319363249686468123131973
91613341

11

372214

22325

88

123

22

123

232411

16262101571380

213

311721

506729155142592630551659

1322117576210119

361122222351

1717

11821184

617

15116

295198

99

446431362361023361553

11

55

11

22

31132

2226

11

1212

11

99

22

224

224

86201335221031118

86201335221031118

11123329

11123329

11

11

11

11

11

11

112

112

8210

8210

112

11

11

11

11

77

77

1124

1124

11

11

2221211374153

22

22

11

11

221211364150

221211364150

11

11

8844710162162358255174837121815855

8844710162162358255174837121815855

136263422249

51129

51129

13620332139

13620332139

11

11

11125

1124

1124

11

11

123121212

1312119

1312119

1113

1113

2122215109258223323118107505

2122215103252223323118107493

13112118

314

2110303735151643117

415

1113

31212110

2245697211102

10212

1517

116281313246

11113289713381157

123

369

6612

369

11

11

11

221296132165

11

11

123

123

415

11

44

11

11

215276121155

215276121155

54134612241438310441151

54134612241438310441151

54134612241438310441151

1172111171822154

11

11

7154712128

7154712128

112

11

11

11417

11417

11

11

4419

4419

22

22

22

22

11

11

11

11

1226213

1226213

1126212

11

11

11

11

52311214

52311214

224

224

224

22

11

11

11

11

51118

51118

51118

1113

1113

1113

1113

11

11

112

112

112

11112134192365

11112134192365

11112134192365

11112134192365

11112134192365

11112134192365

11

11

11

11

11

11

11

11

11

11

11

11

877541020403323303929131012263

3522269302211193712437147

12234343121127

123

11

11

112

112

2232343121124

2232343121124

2232343121124

2232343121124

2232343121124

34224524176143610335111

34224524176143610335111

1211375252622443

1211375252622443

112411211115

12115

122121110

111134132512328

1111121412116

11

1131111211

12671112122

1225

1225

1225

11451112117

112

112

415

44

11

11412110

11412110

22122115384211145

1113

11

11

11

11

11

11

12122115374111142

1212210425111133

1212210425111133

11

11

112318

112318

11

11

11

11

1221219

1221219

1221219

5253241110111211217975116

32412241651168349

22113133166130

1124

1124

1124

221366121

11

11

121366120

121366120

3115

314

314

11

11

11215

11215

11215

11

1124

221111112214

11

11

11

1111217

1111217

1111217

211116

111115

1113

112

11

11

1125110

1125110

1125110

1125110

11

11518

11

11

11

11

11

121321111

121321111

121321111

11

11

123219

123219

11

11

2114

2114

2114

112

11

11

112

112

11125

11

11

1113

112

112

112

11

11

11

11

11

11

11

121211311

111137

1135

33

33

112

11

11

112

11

11

11

11

11

11

213

213

213

213

1111144145225

1111144145225

1112117

1112117

1112117

11132144118

21126

21126

1111124112

1124

1113

11215
