# Supplementary material for: Characterisation of the Carpinus betulus L. Phyllomicrobiome in Urban and Forest Areas
Source: Front Microbiol. 2019 May 29;10:1110. doi: 10.3389/fmicb.2019.01110 (PMC6549492; doi:10.3389/fmicb.2019.01110)
Supplement: Supplementary file 7 [file Data_Sheet_6.ZIP › bin_report.html]

xml version="1.0" encoding="utf-8" ?


ATLAS - Bin Summary


# ATLAS - Bin Summary

Contents

- Summary
  - Recovered Bins
  - Best Bins
- Downloads

# Summary

## Recovered Bins

In some cases, percentages can be above 100% (See: CheckM Issue 107).

## Best Bins

Genomes with >90% completeness and <5% contamination:

| Bin ID | Completeness | Contamination | Taxonomy (contained) | Taxonomy (sister lineage) | GC | Genome size (Mbp) | Gene count |
| --- | --- | --- | --- | --- | --- | --- | --- |
| epBi51-1.003 | 95.23 | 4.87 | f\_\_Sphingobacteriaceae; g\_\_Pedobacter | unresolved | 39.860 | 5.711 | 6600 |
| epBi42-2.001 | 98.63 | 4.34 | f\_\_Pseudomonadaceae; g\_\_Pseudomonas | s\_\_ | 59.960 | 6.743 | 6504 |
| epBo14-2.001 | 99.57 | 1.78 | f\_\_Xanthomonadaceae; g\_\_Stenotrophomonas | s\_\_Stenotrophomonas\_maltophila | 66.509 | 4.581 | 4152 |
| epBi42-1.001 | 98.86 | 2.80 | f\_\_Pseudomonadaceae; g\_\_Pseudomonas | s\_\_ | 59.950 | 6.735 | 6503 |
| epBo14-1.001 | 99.28 | 1.86 | f\_\_Xanthomonadaceae; g\_\_Stenotrophomonas | s\_\_Stenotrophomonas\_maltophila | 66.488 | 4.604 | 4192 |

See full list at Table\_1.

# Downloads

> Table\_1:
> :   genomic\_bins.tsv

2018-07-25
